# 30-day postoperative mortality and the effects of hospital preparedness during the COVID-19 pandemic: a pooled analysis of prospective international cohort studies

**DOI:** 10.1016/j.lanepe.2025.101566

**Published:** 2026-01-29

**Authors:** NIHR Global Health Research Unit on Global Surgery, Dmitri Nepogodiev, Sivesh K Kamarajah, Aneel Bhangu, Radhika Aacharya, Waheed-Ul-Rahman Ahmed, Ehab ElAmeer, Ruth Blanco-Colino, Muhammed Elhadi, Dhruva Ghosh, James C Glasbey, Arda Isik, Kate Jolly, Haytham Kaafarani, Bryar Kadir, Hans Lederhuber, Sezai Leventoğlu, Omar M Omar, Francesco Pata, Maria Picciochi, Peter Pockney, Marie Dione Sacdalan, Joana FF Simoes, Georgios Tsoulfas, Daoud Chaudhry, Ruth Blanco Colino, Irani Duran, James Glasbey, Rohan Gujjuri, Sivesh Kamarajah, Santhosh Karri, Kayani Kayani, Stephen Knight, Samuel Lawday, Elizabeth Li, Harvi Mann, Fatima Mansour, Kenneth McLean, Omar Omar, Maria Piccochi, Irene Santos, Joana Simoes, Chris Varghese, Maryam A, Sultan A Al Amri, Juan A Mejia, Gregor A Stavrou, N Aagaard, Junaid Aamir, Jesús Aarón Martínez Alonso, F Aarts, Y Aawsaj, Norhafiza Ab Rahman, Islah Munjih Ab Rashid, Teresa Aba Mensah, Muna Aba Zaid, Muath Abaalkhail, Adnan Ababneh, H Ababneh, Hazim Ababneh, Laila Ababneh, M Ababneh, Roba Ababneh, Rafael Abad Alonso, Alfredo Abad Gurumeta, A Abad-Gurumeta, A Abad-Motos, Ane Abad-Motos, Mussab Abaker, John Abanga Alatiiga, S Abas, Adam Abass, J Abassy, E Abate, Emmanuele Abate, C Abatini, Sheraz Abayazeed Ahmed, Olukayode Abayomi, Alaa Abazeed, J Abba, Abdurrahman Abba Sheshe, Bader Abbad, F Abbadessa, Francesco Abbadessa, Osaid Abbadi, Malaz Abbakar, A Abbas, Ahmed M Abbas, AM Abbas, AS Abbas, Asad Abbas, Aya M Abbas, F Abbas, FM A Abbas, FMA Abbas, Jihad Abbas, Manzar Abbas, Omer Abbas, S Abbas, M Abbasi, A Abbasov, Aykhan Abbasov, Olivier Abbo, Daniel Abbott, S Abbott, T Abbott, Tom Abbott, Tom EF Abbott, Waleed Abd, Tayma Abd Alghafour, Wael Abd El-Ghani, HA S Abd Elazeem, HAS Abd Elazeem Mohammed, Reda Abd ElGhany, Mustafa Abd Elsayed, Ahmed Abd Elwahab, Sami Abd Elwahab, NN Abd Kahar, EH Abd Wahab, MM Abd-Elkarem, AY Abd-Elkariem, S Abd-elsalam, Sherief Abd-Elsalam, Joel Abdala Junior, Ahmad Abdalah, H Abdalaziz, Lana Abdalgadir Ahmed Mohamed, A Abdalhadi, Alya Abdalhadi, Ahmed Abdalla, M Abdalla, Samir Abdalla, Shimaa Abdalla, Siddig Abdalla, Hdaya Abdalla S Benabdalla, EA Abdallah, Emne Abdallah, Ghaida Abdallah, Lubna Abdallah, M Abdallah, Munir Abdallah, Rasha Abdallah, Hani Abdalnour, Bashar Abdeen, S Abdeewi, Saedah Abdeewi, L Abdeh, Louai Abdeh, S Abdel Al, Shrouk Abdel Fattah, R Abdel Jalil, Ali H Abdel Sater, Mahmoud Abdel-Aleem, M Abdel-bari, Wafaa Abdel-Elsalam, Areej Abdel-Fattah, Nour Abdel-Fattah, K Abdel-Galil, Ibrahim Abdel-Hafez, M Abdel-Maboud, Abdelrahman Abdelaal, Khaled Abdelazeem, S Abdelaziem Mustafa, Areej A Abdelaziz, Mohammed Abdelaziz, A Abdelbagi, Mohamad Abdelbagi, Abouelnour Abdelbaset, Hesham Abdeldayem, Mahmoud Abdelfattah, Alwaleed Abdelgadir, Khaled Abdelgalel, Moslem Abdelghafar, Mohamed Abdelghafor Hassanin, Mohammed Abdelhafez, Abdelkarim Abdeljalil, M Abdelkabir, Mohammed Abdelkabir, Ibrahim Abdelkader Salama, M Abdelkareem, Mohamed Abdelkareem, Mohamed M Abdelkarem, M Abdelkarim, Mostafa Abdelkarim, M Abdelkhalek, Mohamed Abdelkhalek, Fatima Abdellah, A Abdelmajeed, Ahmed Abdelmajeed, Abubaker Abdelmalik, Ahmed Abdelmawla, ElTahir Abdelrahim, N Abdelrahim, A Abdelrahman, Abdelrahman Abdelrahman, Haneen Abdelrahman, Ali Abdelraouf, Karim Abdelraouf Moawad, S Abdelrhman, K Abdelsaid, A Abdelsamed, Ahmed Abdelsamed, K Abdelwahab, W Abdelwahab, Hafni Abderrazaq, Desalegn Abdissa, H Abdou, Hossam Abdou, Mostafa Abdou, Diallo Abdoul Azize, D Abdoun, M Abdoun, Meriem Abdoun, Mohammad Abdow, Ahmed Abdrabou, Mahmmoud Abdualqader, Aya Abdul Al, DA Abdul Aziz, Jumana Abdul Hameed, Najat Abdul Hameed, N Abdul Maei, Norazila Abdul Rahim, Noorneza Abdul Rahman, UH Abdul Rauf, Omar Abdul Salam, H Abdul-Jabar, Hani B Abdul-Jabar, Alhassan Abdul-Mumin, Safaa Abdulaal, Omar Abdulateef, Abdulmajeed Abdulaziz Saeedi, F Abdulfattah, Muhammad AbdulHakeem, Sakhr Abdulhakeem Al-maswari, E Abdulkader, Adnan Abdulkadir Mohammed, Amal Abdulkareem, Miriam Abdulkarim Polo, Mohammed Abdull, N Abdulla, Abakar Abdullaev, Abdullah Abdullah, Bahiyah Abdullah, Nabila Abdullah, Saleha Abdullah, Shahbaz Abdullah, Nayrouz abdullah Abulshuwashi, Zakarya Abdullah Al-Zaazaai, Ibrahim Abdullah Hakami, Alsnosy abdullah Khalefa mohammed, Muhammad Abdullah Khalid, Ahmed Abdullah Shaalah, Lamess Abdullaha, Alhassan Abdullahi, Habiba Abdullahi, IH Abdullahi, Lawal Abdullahi, M Abdullahi, Sani Abdullahi, Sani Abdullahi Yunusa, Abdulmalek Abdulmalek, Abdulmuez Abdulmalik, Ahmed Abdulmohsen, Seemal AbdulQadir, Nabil Abdulqawi, Abdullah Abdulrahem, F Abdulrahman, Mamuda Abdulrahman, Mohamed Abdulrahman, Taha Abdulrahman, Muna AbdulRazzaq Tahlak, Albaraa Abdulsalam, Fareed Abdulsalam, Khalifa Abdulsalam, Moruf Abdulsalam, Taiceer Abdulwahab, E Abdulwahed, Eman Abdulwahed, Murad Abdunabi, L Abdur-Rahman, Lukman Abdur-Rahman, Ebrahim Abdurab, Oumer Abdurehman, Abdussemee Abdurrazzaaq, R Abdus-salam, Rukiyat Abdus-salam, Mehmet Abdussamet Bozkurt, Anthonia Abe, Nobutsugu Abe, Tatsuro Abe, Engida Abebe, Kirubel Abebe, M Abebe, Metasebia Abebe, Nebyou Abebe, Mersha Abebe Woldemariam, JT Abebrese, Francisco Abed, Haneen Abed, Lina Abedalqader, Y Abedin, Yasmin Abedin, Marian Abedua Harrison, Livingston Abel, MK Abel, A Abelevich, Alexander Abelevich, M Abellán, Miriam Abellan Lucas, JMI Abellera, J Abeloos, K Abhilashi, Wijden Abichou, Rubaba Abid, Adekunle Abiodun, Olajide Abiola, Paul Abiola, Henry Abiyere, OH Abiyere, Ahmad Abo Arar, Mustafa Abo Mohsen, Ahmed Abo Shanab, Ghaleb Aboalsamh, Hajir Aboazamazem, Aya Abodeeb, A Aboelkassem Ibrahim, Roger Aboelkhel, Z Aboharp Hasan, Ziad Aboharp Hasan, Farah Abojeila, Ibrahim Abolaji Alabi, Orlando Abonia Gonzalez Abonia Gonzalez, A Abood, NE Abosamak, Yahya Abosnaina, Ahmed Abostate, Amna Abou Bakr, MK Abou Chaar, Mohamad K Abou Chaar, Hussein Abou-Abbass, M Abou-Abdallah, AK Abou-Foul, J Abou-Khalil, Jad Abou-Khalil, A Abouassi, Layth Abouassi, Majd Abouassi, Samar Aboubakr, Mohammed Aboubeirah, Mohamed Abouelazayem, Galal Abouelnagah, Yossof Abouelnagah, H Aboulkassem, Omar AbouMadawy, Shereen Aboutaleb, Ayham Aboutrab, Amr Abouzid, A Abozid, H Abozied, Hesham Abozied, Bejoy Abraham, Camara Abraham Faya, Jenevive Abrahams, S Abramowicz, Shelly Abramowicz, M Abrar, A Abrate, Alberto Abrate, Tassew Abreha, A Abreu da Silva, Alberto Abreu da Silva, Teklebirhan Abrha, Arsan Abu abed, Laith Abu Abed, Najib Abu Draz, S Abu Freih, MFK Abu hallalah, M Abu Hamraa, M Abu Hilal, Mohammed Abu Hilal, Dima Y Abu Ismail, Mustafa Abu Jayyab, M Abu Mohsen Daraghmeh, Mustafa Abu Mohsen Daraghmeh, Dima Abu muhfouz, Hamza Abu Obead, W Abu Rashed, R Abu Salah, A Abu salhiyeh, Alaa Abu Salhiyeh, Md Abu Sayed, Ammar Abu Tarieh, FJ Abu Zanouneh, Hamdoon Abu-Arish, Basil Abu-Eisheh, Nizar Abu-Ishkerih, Luai Abu-Ismail, M Abu-Jeyyab, Tareq Abu-libdeh, Marah Abu-Mehsen, I Abu-Nayla, Ahmed Abu-Zaid, Z Abual-Rub, M Abualjadayel, Faisal Abualteen, Carla Abuawad, Abdullah Abubakar, Ahmed Abubakar, M Abubakar, Abubakr Abubakr, Asmaa Abubakr, Hossam Abubeih, Burçin Abud, Hadeel Abudari, A Abudher, Abdulhafid Abudher, Ehab Abuhamour, Maaly Abuhlaiga, Khatab Abuissa, Mahmoud Abukhadra, S Abukhalaf, SA Abukhalaf, Sadi Abukhalaf, Sadi A Abukhalaf, Muhammad Abukhater, Daniel Abulafia, Amro Abuleil, Saleh Abumahara, Huthifa Abunawas, A Abuown, Mohammad Aburahmah, Fatima Aburayyan, Sarah Aburima, Abdelrahman Abusabeib, Samer Abusadah, Marwa Abusalem, Malek Abusannoga, A Abutaka, Ahmad Abutaka, Malak Abutaleb, Khalil Abuzaina, IA Abuzeid, Y Abye Negatu, Eduard Acatrinei, Giulio Accarino, Guilherme Accorsi, Alfeu Accorsi Neto, F Acebes García, Fernando Acebes García, M Achalandabaso Boira, Mar Achalandabaso Boira, Hriday Acharya, M Acharya, Metesh Acharya, Shivanie Acharya, AR Achek, Michele Achille Crespi, P Achimas-Cadariu, Patriciu Achimas-Cadariu, AS Acikgoz, J Ackah, Travis Ackermann, Jesus Acosta, Maria Acosta, S Acosta, Úrsula Acosta, Yancy Acosta, Lina M Acosta Buitrago, MA Acosta Mérida, J ACourt, Jane Acquaye, R Acra-Tolari, Ricardo Acra-Tolari, Gastón Acuña, Luke Adagrah Aniakwo, AA Adam, John Adam, MAA Adam, Eman Adam Abdalla, Laszló Ádám Bihari, ME Adam Essa, Soujanya Adamala, A Adamec, M Adamina, Michel Adamina, L Adamoli, Laura Adamoli, C Adams, J Adams, Katie Adams, S Adamski, Nicholas Adamson Barnes, A Adamu, Akshath Adapa, E Addae-Boateng, Bassam Addas, A Addissie, Andi Ade Ramlan, Miguel Adeba García, Emmanuel Adebajo, Ademola Adebanjo, Idowu Adebara, IO Adebara, Olaolu Adebayo, Sikiru A Adebayo, Ganiyu Adebisi Rahman, Oluwaseun Adeboyejo, Muhammad Adeel Akhtar, S Adegbola, Samuel Adegbola, Samuel Adegboyega Olatoke, Dina Adel, I Adel, Ahmed Adel abdelaty, E Adel Hamdoun Aziz, Mohamed Adel Nassef, Aderinsola Adelaja, N Adeleke, Amos Adeleye, Elena Adelina Toma, Ephrem Adem, Samuel Ademola, A Ademuyiwa, Adesoji Ademuyiwa, AO Ademuyiwa, Abimbola Adeniran, Abiodun Adeniran, AS Adeniran, AA Adeniyi, Adebayo Adeniyi, Mehjabeen Adenwalla, Adewale Aderounmu, Opeoluwa Adesanya, Oluwaseyi Adesina, Muideen Adesola, AbdulHafiz Adesunkanmi, Musliu Adetola Tolani, Masud Adewusi, Olabisi Adeyemo, OT Adeyemo, A Adeyeye, Ademola Adeyeye, Ibrahim Adham, Mohamed Adhnan Thaha, S Adhya, H Adi, Hussam Adi, A Adiamah, Alfred Adiamah, Valentine Adikaibe, Ahmed Adil, Md Tanveer Adil, Ali Adil Ali karar, E Adinolfi, Andika adiputra Thehumury, Adewale Adisa, AO Adisa, Dita Aditianingsih, Theophilus Adjeso, TJK Adjeso, Alsafe Adlan, Tina Adler, Ainal Adlin Naffi, A Admasu, Azarias Admasu, HM Adnan, Nadir Adnan Hacım, Yvonne Adofo-Asamoah, Gustavo Adolfo Angel, Carlos Adolfo Marroquín Paiz, Mohammed Adrees, Zulay Adriana Calderon Barajas, Ludwigvan Adriano Bustamante Silva, Marcos Adrianzén, María Adrien Lara, A Adroher, Maame Aduse-Poku, R Advani, Rajeev Advani, Mohammad Adya Firmansha Dilmy, Joel Adze, G Aeby, Majed Aeed, S Afaghi, L Affronti, Amgad Afifi, Eman Afifi, Nermeen Afifi, Abdelrahman Afify, Abdulrahman O Afolabi, Akinwale Afolabi, BB Afolabi, Bosede Afolabi, M Afonso-Garcia, S Afroze, Salim Afshar, N Aftab, R Aftab, Raiyyan Aftab, Oludolapo Afuwape, Ameer Afzal, Maimoona Afzal, Mohamed Afzal, Sadia Afzal, Christos Agalianos, M Agapov, Mikhail Agapov, Anjoo Agarwal, Arnav Agarwal, Gaurav Agarwal, K Agarwal, Ketan Agarwal, Sunny Agarwal, Varun Agarwal, Ervis Agastra, Mouhamed Agbadebo, Ademola Agbaje, Kwabena Agbedinu, DYD Agbley, Nelson Agboadoh, P Agbonrofo, Peter Agbonrofo, Mohammed Ageeli, A Aggarwal, G Aggarwal, Gaurav Aggarwal, Manisha Aggarwal, Sonali Aggarwal, C Aggeli, K Aghababyan, Kristina Aghababyan, Ifeanyi Aghadi, Ilgar Aghalarov, Ughur Aghamaliyev, SMK Aghamir, ZS Aghamir, Afag Aghayeva, Kayvan Aghazadeh, ET Agida, Eyaofun Agida, J Agilinko, I Agledahl, A Agnes, Annamaria Agnes, Salvatore Agnes, H Agnus Moorthiraj, O Agodirin, Hermann Agossou, C Agostini, P Agoston, Amit Agrawal, Mansi Agrawal, Rachit Agrawal, S Agrawal, Tulika Agrawal, LA Agredo Luna, F Agredo Villaquiran, Héctor J Aguado, HJ Aguado, H Aguado Lopez, H Aguado López, Omar Aguayo, JL Aguayo-Albasini, SV Agudelo Mendoza, M Aguennouz, Renato Aguera Oliver, Mauricio Agüero Mariño, S Aguiar Jr, S Aguiar Júnior, Samuel Aguiar Júnior, A Aguilar, Ana Aguilar, Ruben Aguilar, CE Aguilar Alvarado, Jorge L Aguilar Frasco, J Aguilar-Jimenez, M Aguilera Lorena, Maria Aguilera Lorena, M Aguilera-Arevalo, Maria-Lorena Aguilera-Arevalo, ML Aguilera-Arévalo, B Aguinagalde, Borja Aguinagalde, Asier Aguirre, L Aguzzoli, Lorenzo Aguzzoli, Fareeda Agyei, S Agyeiwaa Owusu, Akosua Agyemang-Prempeh, Thomas Agyen, K Agyen Mensah, Kwasi Agyen Mensah, S Ahad, Waseem Ahamed, Roghayyeh Ahangari, K Aher, Thomas Aherne, TM Aherne, Hamza Ahlaou, Saravpreet Ahluwalia, Afaf Ahmad, Aline Ahmad, Amer Ahmad, Ashfaq Ahmad, Aya Ahmad, B Ahmad, Basel Ahmad, Fateen Ahmad, H Ahmad, Maleeha Ahmad, Manzoor Ahmad, Misbahu Ahmad, Mohamad Ahmad, Niarah Ahmad, Quazi Ahmad, Reyaz Ahmad, Rofida Ahmad, S Ahmad, Shabir Ahmad, Shahrukh Ahmad, Sheraz Ahmad, Siddique Ahmad, SJ Ahmad, Tareq Ahmad, Y Ahmad, Zeeshan Ahmad, Muzaffar ahmad Ahmad, Ayah Ahmad Al_shraideh, Haneen Ahmad Alhami, Shafiq Ahmad Chughtai, Zainab Ahmad Haq, Faraz ahmad Khan, Mumtaz Ahmad Khan Khan, Fuad Ahmad Khan Niazi, Zamir AHMAD Shah, Imdad Ahmad Zahid, N Ahmadi, Navid Ahmadi, S Ahmadi, Sayedali Ahmadi, SMS Ahmadi Rashti, Ali Ahmadvand, A Ahmed, Abdelkareem Ahmed, Abdullah Ahmed, Abedelrahman Ahmed, Ahmed Ahmed, Aishah Ahmed, AMAM Ahmed, Arooj Ahmed, Ayman Ahmed, Azaz Ahmed, Ehsan Ahmed, F Ahmed, Faazil Ahmed, Faryal Ahmed, Ghazia Ahmed, Haseeb Ahmed, Hassaan Ahmed, I Ahmed, Idrees Ahmed, Iffat Ahmed, Irshad Ahmed, Islam Ahmed, Jawad Ahmed, K Ahmed, Kaleem Ahmed, Khalid Ahmed, M Ahmed, Maira Ahmed, Manhal Ahmed, Mariam Ahmed, Mnewer Y Ahmed, Mohammed Ahmed, Muhammed Ahmed, MY Ahmed, Nasir Ahmed, Nauman Ahmed, O Ahmed, Omar Ahmed, S Ahmed, Safia Ahmed, Sara Ahmed, Shahnoor Ahmed, Shakil Ahmed, SM Ahmed, W Ahmed, Waqas Ahmed, Zubair Ahmed, Abdelrahman Ahmed Abdelrahman Ali, Omar Ahmed Abdelwahab, Manal Ahmed Altoumi, Chamakhi Ahmed Amine, Mohammed ahmed Babikir, Mahmoud Ahmed Ebada, M Ahmed Elamin Elnour, Kemel Ahmed Ghotme Ghotme, Abdilaahi Ahmed Hayir, H Ahmed kareem, Abdallah Ahmed Mezel Al-Azzam, ME Ahmed Mohamed, Elmi Ahmed Mohamed Jimaale, Muhammad Ahmed Naseer, Talha Ahmed Qureshi, Tilal Ahmed Raza, Shujat Ahmed Riaz, Sarah Ahmed Saad, Khursheed Ahmed Samo, Imtiaz Ahmed Shakir, Mostafa Ahmed Shehata, Ebrahim Ahmed Yousof, Mahlet Ahmedin, A Ahmeidat, Ismail Ahmet Bilgin, Muhammad Ahsan Iqbal Siddiqui, Muhammad ahsan Khan, Naeimah Ahseen, Abdurahman Ahtash, N Ahuja, K AI Nwijy, Khaled AI Nwijy, Turki AI Zahrani, Martina Aida Angeles, AL S Aidar, Askar Aidarov, E Aigbivbalu, F Aigner, Felix Aigner, Taylor Aiken, Ramez Ailabouni, A Aime, Adeline Aimé, Rakotonarivo Aina Andrianina Vatosoa, A Aiolfi, Alberto Aiolfi, A Airey, Angelo Airoldi, Oseremen Aisuodionoe-Shadrach, Rita Ait benhamou, E Aitken, Jake Aitken, Javier Aitor Zabala Lopez-Maturana, Giada Aizza, Shereen Ajab, Okeoghene Ajagha, Olalekan Ajai, Hatem Ajaj, Dany Ajami, Akinlabi Ajao, Adekunle Ajayi, Peter Ajayi, G Ajcip, Gaby Ajcip, Samuel Ajekwu, Temitope Ajekwu, Narjiss Aji, Hafees Ajibola, Olalekan Ajiboye, Abdulrazag Ajlan, Nadia Ajomah, G Akaba, Godwin Akaba, OG Akaba, Ali Akadh, M Akalin, Murat Akalin, Chris Akani, Bolaji Akanni, Ömer Akay Ömer, E Akaydin, A Akbar, Ali Akbar, Bilal Akbar, J Akbar, S Akbar, Hazrat Akbar Akbar, A Akbas, Ahmet Akbas, Oktay Akça, Mertcan Akçay, Yesim Akdeniz, Nouf Akeel, Adeleke Akeem Aderogba, Taiwo Akeem Lawal, Utku Akgor, N Akhavan Fomani, Hamed Akhavizadegan, Melika Akhbari, Amina Akhtar, F Akhtar, Fahad Akhtar, MA Akhtar, Munazza Akhtar, Naseem Akhtar, Tasleem Akhtar, Ramsha Akhund, Jehad Akiely, YB Akililu, E Akin, Emrah Akin, Opeyemi Akinajo, Success Akindoyin, Olufemi Akinloa, A Akinmade, Akinola Akinmade, Sanusi Akinsola, Tosin Akinyemi, Khalid Akkour, Michel Akl, A Akmercan, Ahmet Akmercan, Elif Akova Deniz, AG Akpede, Marcellin Akpla, Oghenevwegba Akpoghor, Shahzad Akram, Farah Akthar, YE Aktimur, Anton Akulaev, C Akyol, Cihangir Akyol, Aggeliki Al, Murad Al Abdallah, Qurrat Al Ain Atif, Ghadeer Al Ajmi, Khalil Al Ajmi, Nazeeh Al Aktaa, Marwan Al Aliwy, Ahmed Al Ameer, Abdulrahman Al Amri, Ayman Al Amri, A Al Ansari, Tala Al Asadi, Samer Al Athath, S Al Awwad, A Al Ayed, Rawan Al Azhar, Abd Al Aziz Lanagrán Torres, Ghalib Al Badaai, Nawf Al Balushi, Z Al Balushi, Zainab Al Balushi, Wameedh Al Bassam, Zakaria Al Bdour, Seif Al Dahabrh, Mahmood AL Dhaheri, Sajedah Al DHOUN, Mohammed Al Dosouky, Mohammed Al Duhileb, Jubran Al Faifi, A Al Farai, M Al Farsi, Maather Al Farsi, Wadha Al Ghafri, Alaa Al Ghafry, Rawda Al Gohary, Hessa Al Habes, Hiba Al Hage Diab, Abdulaziz Al Harthi, I Al Hasan, M Al Hinai, AbdulAziz Al Hindi, Mohammed Al HOSNI, H Al Houri, Lama Al Humaid, Rayet al islam Ben jouira, Mohammed Al Jamahir, Ziad Al jarad, Hussain Al Jawad, Maha Al kalbani, Moza Al Kalbani, Lamya Al Kharusi, Safiya Al Kharusi, Ahmad Al Khassawneh, Salim Al Lahham, F Al maadany, Faraj Al maadany, Leyla Al Mahdawi, Hossam Al Mahdy, Reem Al Makari, A Al Malkawi, AR Al manasra, H Al Miskry, Rehab Al Moagal, Tareg Al Momani, Fadhl Al muhtadi, Rahaf Al Mulke, Mohammed Al Mutani, M Al Naamani, Aminah Al Nafesa, Hamza Al Naggar, H Al Naggar., Syed Al Nahian, Majed Al Najjar, Tasneem Al najjar, Awaji Al nami, Saleh Al Nassar, Shehanah Al Omair, Faisal Al Otaibi, Hani Al Qadhi, Rashed Al Qudhaya, R Al Raddadi, Amani Al Raisi, Asmaa Al Rashed, Nihal Al Riyami, S Al Riyami, Salim Al Riyami, Hilal Al Sabti, Ghiath Al Saied, Ghadeer Al sanany, Mohamed AL Sayed, Maha Al shaibi, A Al Sharie, Ahmed Al Sharie, S Al Sharie, Asmaa Al shukri, Hana Al Shurman, Omar Al Smadi, Saud Al Subaie, Kais Al Suyyagh, M Al Tarakji, Hajr Al Wadei, Zakaria Al Yahya, Attiya Al zahrani, Zeina Al Zein, Y Al Zu’bi, D Al Zubi, Anwaar Al_Dhafif, Buthina Al_jarmozi, Siham Al_maqtari, B Al_sharash, Salma Al- Houssami, Rashid Al-Abri, Emad Al-Absi, Sofian Al-Adwan, S Al-Ameri, Saba Al-ameri, Hussam Al-atiyah, M Al-Azzawi, Marwa Al-Azzawi, Nimer Al-azzeh, Ismail A Al-Badawi, H Al-Balas, Hasan Al-Balas, A Al-Bourah, T Al-Dabaa, Tawfik Al-Dabaa, Ali Al-Darabah, A Al-Darobi, Awsan AL-Dhaheri, M Al-Dhaheri, Wedad Al-dolat, S Al-Embideen, Somya Al-Embideen, Fatima Al-Eryani, H Al-Fahel, S Al-Falahat, A Al-Fraihat, Maha Al-Gilani, Amro Al-Habib, Y Al-Harazi, A Al-Harbawee, A Al-Harbawi, Fahad Al-Hasani, Amer Al-hebbah, M Al-howthi, Mohammed Al-howthi, Ali Al-Isawi, Mohammad Al-Jadaan, Amar Al-Jarrah, Hothaifa Al-Jarrah, Salsabeel Al-Jarrah, MA Al-Juaifari, SA Al-Kailani, Adil Al-Karim Manji, F Al-kasaji, Farah Al-kasaji, Ziad Al-Khaddar, E Al-Kharashi, W Al-Khyatt, Motasem Al-latayfeh, Ahmed Al-madhrahi, Sanabel Al-Maghrabi, Shehab Al-Mahdi, Husain Al-Mahmeed, Hazem Al-Mandeel, S Al-Maqtari, Sahar Al-maqtari, Hayder Al-Masari, Mohammed Al-Masood, M Al-Masri, Mahmoud Al-Masri, Abdulrahman Al-Mohammad, Ahmad Al-Mouakeh, Abdullah Al-Mujaini, Y Al-Mukhaizeem, Youssef Al-Mukhaizeem, A Al-mukhtar, H Al-Naggar, Hamza Al-Naggar, Dania Al-Najjar, H Al-Najjar, Hani Al-Najjar, Y Al-Najjar, Bilal Al-Nawas, H Al-Omishy, Ayman Al-oqabi, Saeid Al-oribi, Malk Al-Osta, Hadeel Al-Othman, Mohammad Al-Qannas, Ali Al-Qannass, M Al-qattan, Mohammad Al-Qattan, Qusay Al-Qurashi, Ali Al-Radhi, Osman Al-Radi, Ibrahim Al-Raimi, Wisam Al-Ramli, Aya Al-Rashdi, Nina Al-Saadi, Rafat Al-saban, Yusra Al-Sabbagh, A Al-Samaraee, Tariq Al-Shaiji, Ghadeer Al-Shaikh, Sonds Al-Shammakh, Ahmad Al-Shaye, M Al-Shehari, Mohammed Al-Shehari, Z Al-sheikh ali, Zaid Al-sheikh ali, Abdallah Al-Shibi, Abdel-Ellah Al-Shudifat, Mutlaq Al-Sihan, Ibrahim Al-Slaibi, A Al-Sukaini, Ahmad Al-Sukaini, S Al-Tahayneh, Y Al-Tamimi, Yahia Al-Tamimi, Mohannad Al-Tarakji, Abdullatif Al-Terki, Mohammad Al-thaher, A Al-Touny, SA Al-Touny, Mohammed Al-Urfan, Muntadhir Al-uzri, Omer Al-Yahri, M Al-Yaseen, Mustafa Al-Yaseen, Karim Al-Zazay, Reham Al-Zyadat, S Alaa, Sherif Alaa, O AlAamer, Ohood AlAamer, J Alabbad, Jasim Alabbad, Amira Alabbasi, Yousof Alabdulkarim, Reem AlAbdulwahed, R Alabo, Mohamad Alabras, A Aladaileh, Ammar Aladaileh, Omar Aladawi, Samuel Alade, Timothy Aladelusi, A Aladeojebi, Ali Alafif, Abeer Alaglan, Adel Alahaidib, Hani Alahdal, Feras Alahmad, Ibrahim Alahmadi, Ahmed Alahmari, S Alahmed, Salman Alahmed, N Alajaji, Nouf Alajaji, A Alajalen, Amer Alajalen, Suha Alajmi, Turki Alajmi, Felix Alakaloko, Deem Alakeel, Amira Alakhdury, Mohamed Alaktaa, DY Alalawi, Y Alalawi, Yousef Alalawi, Ahmad M AlAli, Mohammed Alali, Azhar Alam, Junaid Alam, Mahmood Alam, Muhammad Alam, R Alam, Ruhina Alam, Walid Alame, H Alameen, Hind Alameen, S Alameen, E AlAmeer, Ehab Alameer, A Alamin, Samer Alammari, Ahmed Alamri, O Alamri, Ossama Alamri, Gary Alan Bass, Zehra Alan köylü, F Alanazi, A Alanbuki, Omar Alannaz, M Alansary, Khalid Alaqeely, Sara Alaqel, A Alarabi, Alarabi Alarabi, R Alarabi, Rehab Alarabi, Zuhair Alaradi, Isaias Alarcón, VD Alarcón Vela, S Alarood, Salameh Alarood, Raquel Alarza, Ala Alasadi, Sabreen ALashmali, Datonye Alasia, A Alasmari, Mohammed Alasmari, Mohammad Alassaf, Ali Alassiri, Blanca Alastrue Giner, Abdullah Alatar, Mohammed Alateeq, Zainab Alattas, Abdulbari Alawadhi, Khalid Alawadi, Huda Alawami, Mohammed Alawami, Ahmed Alawi, K Alawi, Khalil Alawi, BOF Alawneh, F Alawneh, Fade Alawneh, Y Alawneh, Yazan Alawneh, Mohammed Alayan, Barnabas Alayande, A Alayed, Ahmad Alayed, Nada Alayed, Hala Alayyoubi, Emad Alazab, Basma Alazabi, M Alazabi, D Alazawi, O Alazki, Ghaleb Alazzeh, Jr Alba, Basil Albaba, Ismail Albadawi, I Albader, Ibtisam Albader, MA S Albader, Zamzam Albadi, Obey Albaini, Adel Albaiti, Ibrahim Albakry, Erminia Albanese, F Albanesi, A Albani Forneris, Agustin Albani Forneris, MEH Albanna, Konstantinos Albanopoulos, F Albaqami, HM Albar, MN Albaraesi, Ali Albargawi, AA Albaroudi, Antonio Albarracín Marín Blázquez, Abdullah Albarrak, Majed Albarrak, Mohammad Albasheer, Aysha Albastaki, Mariam Albatoul Nageh, Nof Albawardy, A Albdah, Abdullah Albdah, M Albendary, Mohamed Albendary, Igor Alberdi San Roman, Laura Alberici, Abdelrahman Alberkamy, Kim Albers, Mara Albert Fort, Julius Albert Sugianto, D Alberti, José Alberto Atristain Pesquera, Jairo Alberto Dussan-Sarria, Julio Alberto Gobernado Tejedor, Antonio Alberto Martinez, Carlo Alberto Pacilio, Ramon Alberto Ramos, Luis Alberto Reyes Figueroa, Jose Alberto Rojo López, Guillermo Alberto Sarmiento Ramirez, Cesar Alberto Vergel Cabrera, M Albertsmeier, Markus Albertsmeier, Markus Albertsmeiers, B Albi Martin, V Albino, Nouran Albishty, Majid Alborzi, A Alburakan, Ahmed Alburakan, Bader Alburayh, Hanadi AlBusaidi, Asem Albzzaz, F Alcaide Matas, Fernando Alcaide Matas, Ignacio Alcalá Rueda, Marta Alcaraz Fuentes, Luis Alcides García Barrionuevo, F Alconchel, Felipe Alconchel, Khalid Aldaghiri, Homoud AlDahash, M Aldaher, Mohamed Aldahma, M Aldakheel, Ahmad Aldakhil, Lateefa Aldakhyel, Abdulrahman Aldakkan, Shuaib Aldalal, Joel Aldana, M Aldawbali, H Aldawoody, Wassim Aldebeyan, C Aldecoa, Saif Aldeen Al Dwairi, Ala aldeen Hasan, Hossam Aldein S, A Alderazi, Amer Alderazi, M Alderuccio, Naif Aldhaam, Amirah Aldhurais, Ehab Aldlyami, Jose Aldo Guzman Barba, J Aldoori, W Aldressi, Wafa Aldressi, K Aldridge, Kerrie Aldridge, Manar Aldubaai, Fozan Aldulaijan, Hamdan Aldumaini, Omolabake Ale, Anna Alecci, Muhammad Aleem, NM Alegria Navarrete, Maryam ALeissa, Gabriela Alejandra Buerba, María Alejandra Caicedo Giraldo, María Alejandra De León Lima, Maria Alejandra Giraldo, Diana Alejandra Pantoja Pachajoa, Claudia Alejandra Rivas Torres, María Alejandra Torrado Varón, María Alejandra Wagner Useche, Daniel Alejandro Donoso Pizarro, Cristians Alejandro Gonzalez, Jose Alejandro Mata, David Alejandro Mejia, Oscar Alejandro Sánchez García, Camilo Alejandro Velandia Sánchez, Sergio Alejandro Villeda, Evgeniya Aleksandrova, L Aleksić, Lidija Aleksić, Aisha Alelwany, María Alemán, G Alemanno, Giovanni Alemanno, M Alemrajabi, Berhanu Alemu, Megersa Alemu, Verónica Alen Villamayor, Jurij Aleš Košir, Abdulkarim Alesmail, Angelo Alessandro Marra, Cosimo Alex Leo, Nicholas Alexakis, Dinesh Alexander, Golomidov Alexander, ME Alexander, Philip Alexander, Tamara Alexander, Thomas Alexander, Edison Alexander Benavides Hernández, Jhon Alexander Hoyos Castro, Israel Alexander Ostrovsky, Dominik Alexander Ratiu, H Alexander-Leon, María Alexandra Heras Garceau, Josephine Alexandra Lim, María Alexandra Pesántez Peralta, Lisbeth Alexandra Urueña Pinzon, Maria Alexandra Velicu, Andreas Alexandrou, Vlad Alexandru Gata, Vlad Alexe, M Alexeev, Jan Alexeis Lacuata, V Alexoudi, BahaUldin Alezabi, J Alfaifi, A AlFakhri, Abdullah AlFakhri, Mohammad Alfarah, O Alfarhan, Hilda Alfaro, A Alfaro-Goldaracena, Alejandro Alfaro-Goldaracena, Dina Alfarra, Mohamed Alfatih Hamza, Fatema Alfayez, Ahmed Alfeqeeh, Abdallah Alferdaus, Alex Alfieri, S Alfieri, Sergio Alfieri, R Alfkey, JP Alfonso, Danilo Alfonso Arévalo Sandoval, Carolina Alfonso Carrillo, Diego Alfonso Paiva Vera, Stephanie Alford, Doaa Alfraidy, Luis Alfredo Betances, José Alfredo Calderón Arancibia, Diego Alfredo Palta Uribe, José Alfredo Pérez Meave, Raymundo Alfredo Pérez Uribe, Helmut Alfredo Segovia Lohse, M Alfuqaha, A Älgå, Andreas Älgå, Noran Algadi, Barbara Algar-Yañez, M Algarni, Mohammed Algarni, Saad Algarni, A Alghamdi, Abdulaziz Alghamdi, Abdullah A Alghamdi, Ahmad Alghamdi, Ibtihal Alghamdi, R Alghamdi, Rami Alghamdi, Saleh ALghamdi, Susan Alghamdi, Thabet Alghazal, Abdallah Alghazo, A Alghuliga, Lolowah Alghuson, Azuolas Algimantas Kaminskas, Sultan Alhabdan, Marwah AlHADAD, Amani Alhaddad, W Alhaddad, Wafa Alhaddad, Ahmed Alhadeethi, Abdulmueti Alhadi, Albrra Alhag, Ammar Alhaidari, F Alhajami, Awatif Alhaje, Baba Alhaji Bin Alhassan, Zahrah Alhajji, Naser AlHajri, Nuraddin Alhakami, Hani Alhalal, A Alhamed, Ahmad Alhamid, Aos Alhamid, Mohammad Alhamid, Othman Alhammad, Tariq Alhammali, Abdelrahman AlHarazi, A Alharbi, Bandar Alharthi, M Alharthi, Mohammed Alharthi, Nawaf Alharthi, R Alharthi, Sultan Alharthi, Hasan Alhasan, Aya ALhassan, Basmah Alhassan, Turki Alhassoun, Naif Alhathal, Majd Alhattab, M Alhawatmeh, Mohammad Alhawatmeh, Alaa Alhazmi, Barrag Alhazmi, Norah Alhazzaa, A Alhefdhi, Amal Alhefdhi, Boshra Alhelal, Fahad Alhelal, Nawal Alhemyari, Ameen Alherabi, Rahaf Alhindi, Mofarej Alhogbani, A Alhojaili, R Alhossaini, Rana Alhossaini, Tarek Alhouni, A Alhouri, Ahmad Alhouri, S Alhudhairy, Adnan ALhumaida, Omar Alhunaidi, Dhyia Alhuq Al-surimi, Ali Alhussaini, Ahmad Alhussein, Meshari Alhuthayl, A Ali, Ahmed Ali, Ahsan Ali, AK Ali, Almigdad Ali, Ammar Ali, Amna Ali, Aneeqa Ali, Aoun Ali, Danish Ali, Douaa Ali, EE Ali, F Ali, Faizah Ali, Ghazanfar Ali, H Ali, HB Ali, Ibrahim Ali, Imran Ali, Irfan Ali, J Ali, K Ali, L Ali, M Ali, Marah Ali, Maria Ali, Mehboob Ali, Mohamed Ali, Mostafa Ali, Muhammad Ali, N Ali, Noman Ali, Roshneen Ali, S Ali, Sadaf Ali, Salem Ali, Samar Ali, Sana Ali, SM Ali, Wagdi Ali, Yakubu Ali, Sani Ali Aji, Amir Ali Akbari, Imran ali Ali, Majdi Ali Alqudah, Luqman Ali Bajwa, Christian Ali Buesaquillo, A Ali deeb, Muhammad Ali Ghufran, Hossam Ali Hadiya, AA Ali Karar, Asad Ali Kerawala, Mehmet Ali Koç, Farman Ali Laghari, Mohamed Ali Mohamed, Abubakr Ali Mohammed Alhassan Humidan, Mohamed Ali Ossman, Saad Ali Saad Salama, Ismail Ali Saleh, Faaiz Ali Shah, Shafqat Ali Shaikh, M Aliaga-Ramos, Alfonso Aliaga-Sanchez, Alfonso Alias, A Aliev, Mehmet Alim Turgut, A Alinaghi Langari, Oleg Aliosin, Badra Aliou Kone, Mir Alireza Hoda, German Alirio Tovar, Halil Alis, A Alissa, M Aliwa, Mohamed Aliwa, Gunay Aliyeva, Z Aliyeva, Zumrud Aliyeva, Ibrahim Aliyu Mukhtar, Narges Alizadeh, A Aljaafreh, Alaa Aljabali, F Aljaber, Maniee Aljabri, Noor Aljabri, Abdulmalek Aljafari, Abdulmoiz Aljafari, Anas Aljaiuossi, Mohammed Aljanabi, F Aljanadi, Firas Aljanadi, Asma Aljanfi, Ramez Aljasem, Fawzi Aljassir, Abbas Aljebur, Ali Aljewaied, A Aljiffri, Murad Aljiffry, Alia Aljifri, R Aljohani, Adil Aljohari, Lylas Aljohmani, Ayat A Aljuba, Roaa Aljunaidi, Abdullah Aljunaydil, Abdulaziz Aljurayyan, Omar Aljuroushi, Ali Aljuzair, N Alkaabi, A Alkabli, G Alkadeeki, Ghadah Alkadeeki, Maher Alkahal, Mohammed Alkahlan, AbdulAziz AlKanhal, Samer Alkarak, Safya Alkarky, A Alkaseek, Akram Alkaseek, Soliman Alkassem, Ola Alkasser, Abdullah Alkassim, Hani Alkattan, Muhannad Alkazrooni, M Alkchr, Hana Alkeelani, Ahmad Alkhaledi, Wael Alkhaleel, Ahmed Alkhalifah, Nawar Alkhamesi, Ahmed ALKhamis, Jumaa Alkhamis, Waleed H Alkhamis, Omar Alkhanbashi, Nour Alkhanji, Nadiya Alkharousi, Al-Salt Alkharusi, Suhad Alkhateb, Abdulrahman Alkhatib, Ahmad Alkhatib, M Alkhatieb, Maram Alkhatieb, Wafa Alkhayal, Saud Alkhayrat, Ali Alkhdor, A Alkhuzaie, Khadija ALKiyumi, N Alkreedees, MM Alkurdieh, Rauoof Alkuwafi, Hasbi Allah Amin, Abdallah R Allam, Mohamed Allam, AY Allan, Jennifer Allan, A Allana, Carola Allemand, Calisha Allen, Laura Allen, M Allen, S Allen, Jc Allen Ingabire, Jakob Allerstorfer, J Allison, M Allison, Caterina Allmer, A Alloush, Mohammad Allouzi, Marta Allue, Abdulaziz Almaawi, Ydyrys Almabayev, H Almabrouk, Hadeel Almadani, Mahmoud Almaghrabi, Asem Almaghrebi, Noof AlMaharbi, M Almahroush, Ali Almalaq, Osama Almalik, Ahmad Almalki, Saeed Almalki, Nora Almana, Raed Almannie, Maha Almansour, Khuloud Almaqrahi, Hassan Almarashi, Fayez Almari, Felwa AlMarshad, Amal Almasri, M Almasri, Murad Almasri, Ashraf Almatar, Bikheet Almatar, Mohammed Almatrafi, Sulaiman Almazeedi, W Almdallal, Adriana Almeciga, A Almeida, AC Almeida, Alexandre Almeida, JI Almeida, Mafalda Almeida, João Almeida Pinto, Juliana Almeida Rego, J Almeida-Pinto, R Almeida-Reis, Rui Almeida-Reis, Meshal Almeshal, Razan Almesned, Heyam Almezghwi, N Almgla, Naser Almgla, A Almhmadi, Sari Almiani, Bushray Almiqlash, Teresa Almiron, Abdulrahman Almjersah, A Almofarreh, Jorge Almoguera, Khalid Almohaimeed, Zuhoor Almohanady, Hadi Almohsen, M Almond, C Almondo, Abd Almonem Shaikh Ahmad, Noora Almoosa, Jaime Jr Almora, Amira Almosa, Eman Almotairi, H Almoumani, Abdullah Almousa, Aya Almoustafa, Abdulrahman Almuawi, Saif Almudares, Abdullah Almufarrih, AAY Almugaddami, Ayman Almugaddami, Abddulrahman Almulhim, AS Almulhim, Ahmad Almulla, Abdullah Almunifi, Majed Almuraee, Fatema Almushawah, A Almutairi, Hanan Almutairi, Abdulrahman Almutawa, Hisham Almutawa, S Almutrafi, Omar Alnachoukati, Ahmed Alnaeem, Hareth Alnahr, Manal Alnaimi, Zahra Alnajem, Louy Alnajjar, Tareq Alnajjar, AQ Alnami, Mohammed Alnamshan, Mohamed alnaser Alnehum, Mahmoud Alnasser, Mohammed Alnasser, AK Alnemare, M Alnemary, Omar Alneser, Mohammad Alnoaiji, K Alnwijy, Suliman Alobaid, S Alobaysi, Saad Alobaysi, Marwa ALodine, Alanoud Alomair, Abdulaziz Alomar, Amar Alomar, O Alomar, Osama Alomar, Soha Alomar, Marta Alomar Bofill, Faris Alomran, Hadeel AlOmran, Nivaldo Alonso, Santiago Alonso Bartolomé, N Alonso de la Fuente, Marta Alonso Fernández, Erick Alonso González-García de Rojas, Jairo Alonso Hernandez, Miguel Alonso Juarranz, V Alonso Mendoza, Veronica Alonso Mendoza, P Alonso Ortuño, A Alonso Poza, Isabel Alonso Sebastian, L Alonso-Lamberti, Hadel Alosta, Ahmed AlOtaibi, Haifa Alotaibi, M Alotaibi, Naif H Alotaibi, M Aloulou, Mohammad Aloulou, J Alowais, Jalal Alowais, Ous Alozairi, Nuri Alper Sahbaz, N Alpert, Poppy Alport, A Alqaarh, A Alqabasani, Abdulmajeed AlQahtani, Awadh Alqahtani, Bandar Alqahtani, Fahad Alqahtani, Loai Alqahtani, Moraya Alqahtani, S Alqahtani, Saad M Alqahtani, Lina AlQalisi, A Alqallaf, M Alqannas, Mashhour Alqannas, Abdulellah Alqarni, Saad Alqarni, S Alqasem, Saad Alqasem, Nooraldin Alqasemi, Hussain Alqaser, Mohammed Alqassab, Abdullah Alqattan, Shatha Alqawasmi, M Alqedrh, Mohannad Alqedrh, Maitha Alqemzi, Shahad Alqreen, Sadeel Alqudah, Basma Alqudaimi, D Alqunaibit, Dalia Alqunaibit, Aya Alqurpaa, Mera Alrabadi, Murtagi Alraboui, Qutaiba Alradawneh, M Alrahawy, Reem AlRakaf, M Alramadhan, M Alrashed, Muath Alrashed, B Alrayes, Bourhan Alrayes, Mohammed AlRayih, F Alresaini, Fay Alresaini, Mohammed Alreshidan, Mohammed Alrezami, Omar Alrifai, Lolwah Alriyees, Meaad Alromaihi, Sarah Alrubaish, Noorsabah Alrubays, Sara Alsaad, H Alsaadi, Hayder Alsaadi, Sabaa Alsaadi, Shatha AlSAAFIN, Salman AlSabah, T Alsabahi, Tareq Alsabahi, Mohammed Alsabri, Norah Alsabty, Ahmed ALsadek, Mohammed Alsadiq, Wael Alsado, Sara Alsaeiti, Maryam Alsafi, Sameh S Alsafty, Abdulrahman Alsaggaf, Yasir Alsagoor, Jawaher Alsahabi, N Alsahan, Abdulhakim Alsaiad, Bayan Alsaid, Abdulaziz Alsaif, Laith AlSaket, Musab Alsakka, M Alsakkaf, Mazen Alsakkaf, Radfan Alsalal, Ree M Alsalamah, Khaled Alsaleh, Mohammad Alsaleh, Nuha Alsaleh, AlHanouf Alsaloom, Alanoud Alsamari, Hisham Alsanawi, O Alsaraireh, M Alsayadi, Musaed Alsayadi, R Alsayadi, Ramzi Alsayadi, AB Alsayed, Yazeed Alsebayel, Nahar Alselaim, Muhannad Alsemari, O Alser, Osaid Alser, M Alshaar, Muhammad Alshaar, A Alshahrani, M Alshahrani, Mubarak Alshahrani, Mushabab Alshahrani, S Alshahrani, Jaffar Alshahri, B Alshaikh, Khaled Alshaikh, Reem Alshaipani, Omar AlShakhshir, M Alshalhoub, Artefaa Alshamari, Abdulaziz Alshammari, Nouf Alshammari, Sulaiman Alshammari, Turki Alshammari, M Alshamsi, S Alshanafey, Saud Alshanafey, Qutaiba Alshannaq, Rana Alshara, Ebrahim Alsharabi, Mohamed Alsharedi, E Alshareea, Entisar Alshareea, Khayriyah Alshareef, Haneen Alshargabi, Mokhtar Alshargabi, F Alsharif, M Alsharif, Nasser Alsharif, Marwa Alsharji, Fadi Alshawared, Ibrahim Alshaygy, M Alshehari, Fatma AlShehhi, A Alshehri, Abdulmajeed AlShehri, Ameen Alshehri, Khalid Alshehri, M Alshehri, Mohammed Alshehri, Yasir AlShehri, A Alsheikh, Sara Alshekh, A Alshitwi, Mahmoud Alshourman, S Alshryda, Sattar Alshryda, Sareyah Alsibai, Mohanad Alsidig, Saif Alsobhi, Zeyad AlSolami, Ali Alsoudani, A Alsoufi, K Alsowaina, Fahd AlSubaie, K Alsubaie, Norah Alsubaie, A Alsuhaibani, Youssuf AlSuhaibani, Rima Alsulaiman, Samir Alsulaimani, Thuwaiba Alsulaimani, Yazeed Alsuliman, Afnan Alsultan, Thuraya AlSumai, Zaid Alsunna, A Alsuradi, Munir Alsuwaimel, Saleh Alsuwaydani, Suhir Alsuwiyah, Abdulmalik Altaf, Kiran Altaf, Zahra Altaf, T Altahan, Talal Altahan, Assma Altaher, Bassam Altalhi, Jaime Altamirano-Villarroel, Cristian Altana, O Altarhoni, A Althobaiti, Awwadh Althobaiti, W Althobaiti, Waleed Althobaiti, Ibrahim Althubaiti, A Althumairi, Azah Althumairi, Gaia Altieri, Y Altinel, Yuksel Altinel, F Altintoprak, Fatih Altintoprak, A Altobal, Ahmed Altobal, Abdulmajeed Altoijry, Roula Altom, DF Altomare, D Altun, Abdulrahman Alturki, Mohammad Altuwaijri, Talal Altuwaijri, Basmah Altuwayjiri, Ikhlass Altwejri, Antonio Alvarado, Rosalinda Alvarado, Vítor Alvarenga, OV Alvarenga Pereira, Andres Alvarez, E Alvarez, Estibaliz Alvarez, FA Alvarez, MR Alvarez, Sofia Álvarez, Javier Alvarez Gama, Alfonso Alvarez Manilla Orendain, Yicel Alvarez Martinez, JA Alvarez Nufio, Iago Alvarez Saez, Eva Alvarez Torres, FE Alvarez-Bautista, Mario Alvarez-Gallego, Fernanda Alves, Paulo Alves, R Alves, Ricardo Alves, Rubem Alves Da Silva Neto, Rubem Alves Silva Junior, A Alvi, Kashif Alvi, Abdullah Alwabari, Muath Alwabel, DAH Alwadani, A Alwadiya, Athari Alwael, Saba Alwahedy, Mustafa Alward, MM Alwarfalli, M Alwash, Mohammed Alwashahi, Ian Alwayn, Nasser Alwehaibi, Mohamed Aly, Hussain Alyafii, Reem Alyahya, A Alyami, Ali Alyami, Alwaleed Alyami, Hamad Alyami, M Alyami, Mohammad Alyami, M Alyazidi, Mohammed Alyousef, Ana Alyra Carvalho, Mohammad AlZaatreh, Adil Alzadjali, Maen Alzaeem, Ali Alzahir, Fatma Alzahraa Gamal, A Alzahrani, Abdullah Alzahrani, Abdulrahman Alzahrani, Ahmed Alzahrani, Meshari Alzahrani, Mohammed A Alzahrani, Mosa Alzahrani, Saud Alzahrani, Mamdouh Alzaibak, M Alzamanan, Mahdi Alzamanan, Mohammed Alzamanan, Khaled AlZamel, Motasem Alzaqh, N Alzarooni, Ahmad Alzedam, Hussam I A Alzeerelhouseini, N Alzerwi, Nasser Alzerwi, A Alzetani, Yasmeen Alzghoul, Gmaan Alzhrani, B Alzomaili, Mai Alzoubi, Malak Alzoubi, A Alzouhir, Sayel H Alzraikat, Mutaz Alzubi, R Alzubi, Yazan Alzubi, Tareq Alzughayyar, Ahmed Alzughoul, Abduljabbar Alzuhair, M Alzwei, Faiyazudin Amado Ibrahim, Munira Amadu, Mohammad Amaireh, Emma Amal Nahal, Nur Amalina Che Din, Mudesir Aman, Yuki Amano, MJ Amaral, C Amarante Dias, Yenuksha Amarasena, Abrham Amare Tesfa, Justin Z Amarin, Ghandi Amayreh, A Amazouzi, Maricely Ambar Perez Fernandez, GK Ambler, Marijus Ambrazevicius, Gabriela Ambriz Gonzalez, A Ambrosi, Pantelis Amditis, Abdur-rafee Ameen, P Ameerally, Ibrahim Amer, Mostafa Amer, Francesco Amico, D Amin, Dina Amin, H Amin, Mohamed Amin, Sana Amin, Shehzadi Amin, Shivang Amin, Verda Amin, Vishal Amin, Mhd Amin Alzabibi, Mohamed Amin Bakr, N Amin Sahid, Mohammed Amir, Doha Amir AtaAlmanan, Malaz Amir Ataalmanan, Tebyan Amir AtaAlmanan, Seyed Amir Javadi, Ahmad Amir Kayali, Mohamed Amir Mrad, G Amira, Gamal Amira, Nicolas Amisi, Zarafshan Amjid, Ahmed Ammar, AS Ammar, Khaled Ammar, Imane Ammouze, Mohamed Amnaina, H Amo, Kwabena Amo-Antwi, George Amoah, Michael Amoah, Joachim Amoako, Yaw A Amoako, S Amoako Asirifi, Mabel Amoako-Boateng, Ricci Amoils, E Amorim, Edgar Amorim, JE Amorim, Jorge Amorim, Robson Amorim, L Amorim Braz, Tatiane Amorim Coelho, Happy Amos, Hoora Amouzegar, Regina Amparo Ugarte Oscco, L Ampollini, Mathew Amprayil, MC Ampuan, Sultan Amrayev, A Amro, Adham Amro, Sarah Amro, E Amzallag, Vinna An, Khoirul Anam, Akshay Anand, P Anand, Premkumar Anandan, G Anania, Sankar Ananth, R Anantha, Dulce Añasco, M Anastasakis, A Anastasi, Alessandro Anastasi, Jose Anatolio Resendiz, Ernesto Anaya, DM Añazco Mareco, F Anazor, FC Anazor, Fitzgerald Anazor, T Andabaka, Carmela Andal, Veronica Andaya, Jon Ander Lizarbe, Kajsa Anderin, Megan Anders, C Anderson, Ikenna Anderson Aneke, H Andersson, Henrik Andersson, Chihiro Ando, Kohei Ando, Tadao Ando, Enrico Andolfi, FA Andrabi, A Andrade, Arnulfo Andrade, Marlene Andrade, R Andrade, Francisco Andrade González, RP Andrade Salinas, TO Andraschofsky, Benoît André, M Andrea, Vito Andrea Capozzi, Viviana Andrea Hernández Angel, Jhoana Andrea Murillo Castellanos, Natalia Andrea Rivera Rincón, Paola Andrea Tabares Romero, L Andreani, Lorenzo Andreani, SM Andreani, Stefano Andreani, D Andreas, V Andreasi, Erika Andreatta, Predrag Andrejevic, P Andreoni, Isis Andreotti, Fernando Andres Alvarez, Paulo Andrés Cabrera Rivera, Jose Andres Calvache, Carlos Andrés Carvajal Fierro, José Andrés Cifuentes Rodenas, Carlos Andres Colunga Tinajero, Oscar Andres Escobar Vidarte, Patricio Andrés Freile Pazmiño, Ainhoa Andres Imaz, Carlos Andres Marulanda Toro, Ricardo Andres Niño Corredor, Camilo Andrés Polanía Sandoval, Henry Andrés Rodríguez, Conrado Andrés Ros, Eva Andreu Riobello, J Andreuccetti, Thomas Andrew Maccabe, Emmet Andrews, Kiah Andrews, Liantsoa Andriamanana, Alexandros Andrianakis, Herimampionona E Andriantsoa, C Andro, Christophe Andro, A Andronic, Farah Androus, Iván Andújar Lara, IA Aneke, Alexandre Anesi, Katerina Anesti, E Anestiadou, E Ang, K Ang, Keng-Leong Ang, W Ang, Wei-Wen Ang, Z Ang, N Angamuthu, Carol Angel, J Ángel, Miguel Angel Alonso Prieto, Miguel Angel Calderon-Llamas, Jose Ángel Diez Ares, Miguel Angel Freiria Eiras, Miguel Angel García García, Miguel Ángel García Ureña, Miguel Ángel Gordo Vega Gordo-Vega, Miguel Angel Hernandez Bartolome, Miguel Angel Jimenez Botello, Miguel Angel Martin-Ferrero, Miguel Ángel Mercado, Roberto Ángel Núñez-González, Luis Angel Suarez Gonzalez, Miguel angel Zavala gonzalez, Maria Angela Dealino, Elisa Angela Diego-Alonso, Estefany Angela Flores Anaya, Stefania Angela Piccioni, María Ángeles Gascón Domínguez, Maria Angelica Arada, María Angelica Cendales, R Angelico, Roberta Angelico, Sophia Angelides, Fragkiskos Angelis, Carlo Angelo Cajucom, Erino Angelo Rendina, Luigi Angelo Vaira, Mario Angelo Zamora, D Angelou, Dimitrios Angelou, K Angelou, Kyveli Angelou, Eva Angenete, Rafael Angerer, Reinhard Angermann, F Angles Crespo, Francesc Angles Crespo, Raquel Angulo Artal, Joshua Anicetti, Hilmi Anil Dincer, Olalekan Anipole, C Anis, Dayang Anita Abdul Aziz, Dusabimana Anitha, Laura Aniukstyte, N Anjarwalla, Naffis Anjarwalla, M Anjomrooz, Momina Anjum, Waleed Anjum, Mariam anjum Ifthikar, sushil Ankadavar, Jacob Ankeny, Frank Ankobea-Kokroe, Jo Ann Chiu, Mary Ann Johnson, Leigh Ann O'Banion, Ju Ann Tan, Patricia Ann Uy, S Annamalai, Nathaniel Annan, Angeli Anne Ang, Mary Anne Carol Cueto, Daryl Anne del Mundo, Sheela Anne George Varayannoor, Shireen Anne Nah, Jeryl Anne Silvia Reyes, V Annessi, J Annett, A Annicchiarico, Alfredo Annicchiarico, Paolo Annicchiarico, Rebecca Anning, Filippo Annino, Dominic Annor Mintah, Willmar Anoso, Philippe Anract, Mehwish Ansar, MM Ansari, M Ansarin, Mohssen Ansarin, Muhammad Ansary, N Anscomb, George Ansong, Zsuzsanna Antal, M Antar, Srdan Ante Anzic, R Anteby, Alaa Anter, J Anthoney, Dennis Anthony Isah, Robert Anthony Keenan, C Anthoulakis, Christos Anthoulakis, M Anthuber, Matthias Anthuber, A Antic, Tina Anto Menachery, Claudia Anton, BT Antón-Eguía, Filippo Antonacci, Pantelis Antonakis, A Antonelli, B Antonelli, K Antoniadis, Morena Antonilli, Marcelo Antonini, Giuseppe Antonino Pellicano, Codina Antonio, Darienzo Antonio, Luca Antonio Aldrighetti, Jose Antonio Carbonell Lopez, Francesco Antonio Ciarleglio, Juan Antonio Corralez Alvarez, Marco Antonio Correa Guimaraes-Filho, Luis Antonio Cuellar Martin, Caio Antonio de Campos Prado, Marco Antonio de la Rosa Abaroa, Jesús Antonio Echavarría Uceta, José Antonio Fernández-Dívar Sánchez, Jose Antonio Gazo Martínez Gazo, Juan Antonio González León, Carlos Antonio Llanos Lucero, Gustavo Antonio Martinez Estrada, José Antonio Ortega-Jiménez, Pedro Antonio Parra Baños, Luis Antonio Pascua-Gómez, Vito Antonio Piserchia, José Antonio Posada, Jose Antonio Salud, José Antonio Sánchez Martínez, Marco Antonio Zappa, Ingrid Antonios, A Antoniou, Afroditi Antoniou, George A Antoniou, Stavros A Antoniou, Theofani Antoniou, P Antonogloudis, MI Antonopoulou, Carmine Antropoli, J Antunes, Antonio Antunes Rodrigues Junior, Christina Antzaka, R Anula, K Anuszkiewicz, M Anvari, Mehran Anvari, S Anwar, Sibtain Anwar, SL Anwar, Mariyah Anwer, Rabia Anwer, Lofty-John Anyanwu, N Anyaugo, Ngozi Anyaugo, Solomon Anyimba, David Anyitey-Kokor, A Anzak, Anam Anzak, Alejandra Anzures Mendoza, Nesrine Aouabed, Hulrich Aouagbe Behanzin, Salah Aoun, SG Aoun, AA Apampa, Daniel Aparicio Sánchez, Eduardo Apellaniz, Olus Api, Jeyakumar R Apollos, JR Apollos, Aya Aposaeeda, C Apostolou, Christos Apostolou, K Apostolou, Konstantinos Apostolou, Enoch Appiah, Adu Appiah-kubi, Peter Appiah-Thompson, J Appleyard, Akhila Appukuttan, Tedy Apriawan, A Aprile, Alessandra Aprile, Vittorio Aprile, IA Apse, SS Apte, Ahmed Aqeelah, Muhammad Aqib, Sabra Aqil, F Aquila, Laura Aquino, Fatima Arab, Khalid Arab, N Arab, Ebrahim Arafa, EJ Aragon Achig, J Aragon-Chamizo, Juan Aragón-Chamizo, Paula Aragón-Ramos, R Aram, G Arampatzis, Iñigo Arana, Francisca Aranda Lozano, Roy Arangoytia, Coro Aranzabal Urrutia, Z Aras, DH S Araujo, Marcelo Araujo, MS Araujo, Asuvathan Aravinthan, Hana Arbab, Gill Arbane, Jeric Arbizo, J Arboleda, Ricardo Arceo-Olaiz, James Archer, JE Archer, Leigh Archer, Daniel Arco, Javier Ardebol, Javier Ardila-Montealegre, Antonella Ardito, F Ardito, Rohan Ardley, Francisco Ardura, L Areias, LL Areias, Alexander Arekhandia, I Aremu, II Aremu, Isiaka Aremu, M Aremu, A Arena, Alessandro Arena, Octavio Arencibia, G Aresu, Mapuor Areu, A Arévalo Barreto, Alejandro Arévalo Barreto, D Argandykov, Giulio Argenio, M Argentou, M Argo, Leah Argus, Claudia Arias, Fernando Arias-Amézquita, Sarra Aribi, Tomather Aribi, C Arican, Vittorio Arici, Celso Ariel Fernandez, P Aries, Iqtaza Arif, Numera Arif, Salman Arif, Wirsma Arif Harahap, M Arigoni, Hassan Arishi, Abimbola Ariyibi, Z Arizavi, Parisa Arjmand, Nikolaos Arkadopoulos, S Arkani, Y Arkha, Yasser Arkha, Lorenzo Arlia, James Arlidge, Edward Arlu Dinoy, Ralph Armah, Nii Armah Adu-Aryee, Anuar Armando Idrobo Escobar, T Armao, Maria Armas, FJ Armas Zarate, Kunwar Armash Ahsan, G Armatura, Daniele Armellin, Jan Armelynn Santos, Mikel Armendariz, S Armentano, A Arminio, Armando Arminio, J Armitage, Alison Armstrong, Lara Armstrong, Laurent Arnalsteen, AY Arnaout, I Arnaout, Khaled Arnaout, E Arnaoutoglou, Eleni Arnaoutoglou, A Arnaud, Alexis Arnaud, Alexis P Arnaud, AP Arnaud, Ingus Arnolds Apse, Abayomi Arogundade, Soliudeen Arojuraye, Rajnish Arora, Lorena Arrabal, R Arrangoiz, Rodrigo Arrangoiz, Eduardo Arrea Salto, Catalina Arredondo Soto, MD Arribas Del Amo, M Arrieta, Mirentxu Arrieta, Giulia Arrigoni, Rodrigo Arrivabeno, Roberto Arroyave, A Arroyo, Diego Arroyo, Analia Arrua, E Arrue, Emmy Arrue Del Cid, Mani Arsalan, Daneyal Arshad, Hajra Arshad, M Arshad, Muhammad Arshad, Owais Arshad, Kalbim Arslan, Kemal Arslan, Muhammad Arslan, Nuhi Arslani, E Arteaga Cedeño, Miriam Artés Artés, Mariano Artés Caselles, Joshua Arthur, B Arthurs, Darwin Artidoro Quispe-Cruz, Enrique Artigues, Dmitri Artioukh, F Artukoglu, Evgeniy L Artyushkov, Sandra Aruachan Vesga, A Arulanantham, Arulprashanth Arulanantham, S Arumugam, Sathyaseelan Arumugam, M Arumugasamy, Abhinav Arun Sonkar, Kseniya Arutyunyan, Shobhit Arya, Teguh Aryandono, Fachreza Aryo Damara, Vasileios Arzoglou, Y As, Malke Asaad, P Asaad, Mohammed Asaad Salem, M Asadi, Andi Asadul Islam, Muhammad Asadullah Khawaja, Doaa Asal, Nita Asamoa-Manu Gyimah, Moses Asante- Bremang, Alvin Asante-Asamani, Elvam Asaph, Christopher Asare, Offei Asare, Ashishkumar Asari, Fernando Ascanio Gosling, F Ascari, Francesca Ascari, J Ascensão, Frehun Asele, N Asemota, E Asensio Díaz, Enrique Asensio Díaz, Luis Asensio Gomez, Fitsum Asfaw, Mohammed Asfour, Ali Asgar Hatim Ali, S Asghar, Syed Asghar Naqi, T Asgill, Ruba Asha, James Ashbridge, J Ashcroft, James Ashcroft, S Asher, Qamar Ashfaq Ahmad, Susannah Ashfield, Robert U Ashford, Mohamed Ashiq Mohamed Salim, Keyoumars Ashkan, C Ashmore, R Ashour, Subhi Ashour, F Ashoush, Fouad Ashoush, Adeela Ashraf, Anam Ashraf, F Ashraf, M Ashraf, Mohamed Ashraf, Olfat Ashraf, Sumaira Ashraf, Abrar Ashraf Ali, AU Ashraf Butt, Mohammed Ashrafi, John Ashutosh Santoshi, Daniel Ashworth, Yvonne Asiedu, Muhammad Asif, Suleman asif Asif, Daniel Asiimwe, Lois Asiimwe, Ibrahim Asiri, Mohammad Asiri, Muhammad Asjad, A Askari, Alan Askari, R Askari, Aatif Aslam, Imran Aslam, Madiha Aslam, Muhammad Aslam, Sher Aslam, Dawit Asmamaw, Mona Asnani, O Asodisen, Sanjay Asopa, R Aspide, Raffaele Aspide, A Asqalan, R Assadi, Reza Assadi, M Assaf, H Assalaarachchi, Muhammad assam Sarwar, Melatework Assefa, Solomon Assefa, Yared Assefa, Marco Assenza, Adel Assiri, E Asti, ELG Asti, I Astreidis, Ioannis Astreidis, Aalap Asurlekar, Manisha Aswani, Sertaç Ata Güler, Gustavo Ataide, Aditya Atal, Khalid Atallah, B Atanasov, Boyko Atanasov, Hana Atarabulsi, Henry Atawurah, Abdurhman Atea, Mohamed Atef, A Atefi, E Athanasakis, Apostolos Athanasiadis, A Athanasiou, Antonios Athanasiou, R Athayde Nemésio, Zeenia Ather, Muhammad Ather Siddiqi, Yoann Athiel, ADTS Athukorala, Ruvinder Athwal, Nora Atiah, Clara Atieno Odhiambo, J Atienza Herrero, H Atif, Terkaa Atim, M Atiq, Gokhan Atis, Vincent Ativor, A Atiya, Bence Atkari, Joseph Atley, B Atnafu, Bahru Atnafu, Kazeem Atobatele, KM Atobatele, O Atoyebi, Oluwole Atoyebi, Amit Atrey, Mulu Atsbaha Weldu, R Atta, Rewan Atta, Joseph Attard, Ahmad Attia, Hajer Attia, MG Attoum, R Attoum, Majed Attoun Attoun, L Attwell, J Attwood, Safa Atyah, Anne-Marie Aubin, Angelica Aubrey Morla, E Aubry, Estelle Aubry, Ziad Audat, François Audenet, A Auerkari, Aino Auerkari, G Augustin, Goran Augustin, Iñigo Augusto, Jorge Augusto Centurion, Gabriel Augusto Cuevas Almando, Marcelo Augusto Faria Freitas, Fernando Augusto Lima Marson, Nestor Augusto Muñoz Botero, A Aujayeb, Avinash Aujayeb, Pritpal Aujla, Randeep Aujla, Y Auqui Medina, Nicoleta Aurelia Sanda, Fahad Aurif, Akawu Auta, Andrea Avanzolini, EK Avci, M Avelino, Melissa Avelino, P Avella, R Avellana, AI Avellaneda Camarena, S Averbach, Sarah Averbach, Alessia Aversano, Konstantinos Avgerinos, DS Avila, Micaela Avila, Pedro Avila, N Avni, Naor Avni, Samuel Avoine, Yonatan Avraham Demma, Emmanouil Avramidis, Andrej Avsenak, Ahmed K Awad, AK Awad, Hadeel Awad, R Awad, Rabih Awad, S Awad, Selmy Awad, Yasir Awad, Alaa Awad Hussein Ameri, M Awadallah, M Awadelkarim, S Awadi, Mudi Awaisu, Ahmed Awaji, Kholoud Awaji, Mirna Awbakh, Lawrence Awere-Kyere, Abimbola Awopeju, Ahmed Awrayit, Ziad Awwad, K Ayad, Kusay Ayad, A Ayala Ochoa, Tewabe Ayalew, Omobolaji Ayandipo, F Ayasra, Faris Ayasra, Y Ayasra, Yazeed Ayasra, A Ayav, Engi̇n Aybar, Eli̇f Aybeni̇z Yildirim, Fatma Ayca Gultekin, Fitsum Ayde, L Aydemir, C Aydin, Cengiz Aydin, Y Aydin, Yener Aydin, E Aydın, Hüsnü Aydın, Karim Ayed, Brook Ayele, Adewale Ayeni, F Ayeni, Funbi Ayeni, J Ayers, Iyehunwa Ayinmode, Y Aykanat, Andrew Aylett, A Ayman, Ammar Ayman, Segun Ayodeji Ogunkeyede, Olabamidele Ayodele, Lateef Ayodele Baiyewu, Malachy Ayogu Emeka, J Ayorinde, Abdu Ayoub, Islam Ayoub, Telce Aysen Gurbuz, E Aytac, Erman Aytac, B Ayub, Bakhtawar Ayub, Bushra Ayub, Khurram Ayub, A Ayubi, Azaz Ayubi, E Ayuso Herrera, M Ayyaz, Mahmood Ayyaz, M Ayyub Anjum, Muhammad Ayyub Anjum, MA Azab, Mohammed A Azab, Junaid Azad, S Azadnajafabad, German Azahares Leal, Ayesha Azam, DS Azam, Mohammad Azam, Riordan Azam, S Azam, Tayyab Azam, Tariq Azam Siddiqi, Elias Azar, Faris Azar, Gholamreza Azarnia Azar Nia, Chisaki Aze, Imran Azeem, Mohd Azem Fathi Mohammad Azmi, Felipe Azenha Lamonica, Constança Azevedo, José Azevedo, P Azevedo, Pedro Azevedo, L Azevedo De Camargo, A Azhar, Amirah Azhar, Faryal Azhar, Saad Azher, Nor Azimah Abd Aziz, Z Azimbeik, A Aziz, Aliya Aziz, Amr Aziz, Humaira Aziz, MU Aziz, Tehmina Aziz, Zaheda Aziz, Gowhar Aziz Bhat, Oula Azizeh, MG Azizeldine, Marah Azkoul, Nor Azlia Abdul Wahab, Dawit Azmach, S Azmanova Mladenovska, Angélica Azucena Soto Carvajal, Ahmed Y Azzam, AY Azzam, Belal Azzam, Abdelrahman Azzam Omran, Rabindranath B, Srinath B S, J Baaij, Mahdi Baba, Abdulaziz Babaier, OF Babalola, Olakunle Babalola, C Baban, Maryam Babar, MS Babar, Mustapha Babatunde, O Babawale, Zeneb Babay, Auwal Babayo Kwankiyel, A Babazadeh baghan, Maryam Babba Danagundi, B Babic, T Babic, U Babic, C Babin, George Babis, BH B Babu, M Babu, Narendra Babu Siddaiah, Miruna Babut, B Baca, Bilgi Baca, N Bacalbasa, Nicolae Bacalbasa, J Bacarese-Hamilton, T Bacarese-Hamilton, M Baccar, Domenico Baccellieri, Matilde Bacchion, Roudi Bachar, Sutej Bachawat, Christopher Bache, Shivani Bachhav, A Bachiri, S Bachiri, T Bächler, Thomas Bächler, Ivan Bacic, A Bacon, Andrew Bacon, F Badahdah, Vanessa Badas, C Baddegama, M Badedi, R Badenes, Rafael Badenes, Thomas Badenoch, Giorgio Badessi, Vivek Badhe, Sanjiv Badhwar, S Badiani, Sarit Badiani, M Badiel, H Badr, Salma Badr, Roberto Badra, A Badran, Nour Badran, Saif Badran, Ssekitooleko Badru, Albert Baduell, Saleh Baeesa, Santiago Baena, Pedro Baez, N Baeza Pintado, M Baeza-Murcia, Mohammed Bafaquh, Anthony Baffour Appiah, Darshan Bafna, Giulia Bagaglini, Jose Bagan, Dinesh Bagaria, Hamed Bagheri, M Bagheri, Nima Bagheri, E Bagouri, Mohamed Bahaaeldin, Mojdeh Bahadorzadeh, Ece Bahçeci, Mohammad Bahhour, Hans Bahlmann, Kiarash Bahrehmand, A Bahreyni, Nazli Bahtigur, Xueli Bai, M Baia, C Baía, Catarina Baía, Amal Baicha, Mariam Baidoun, AM Baietti, M Baig, Mariam Baig, MM A S Baig, Ashley Bailey, Craig Bailey, James Bailey, K Bailey, E Baili, Efstratia Baili, M Bailón, Martín Bailón, Aditya Baindur, L Bains, Lovenish Bains, G Baiocchi, Glauco Baiocchi, A Baite, Ankur Bajaj, H Bajjah, Hadeel Bajjah, O Bajomo, Minu Bajpai, S Bajramovic, Khalid Bajunaid, Abu Bakar Hafeez Bhatti, A Bakare, Adewumi Bakare, Jennifer Baker, Joseph Baker, M Baker, Markus Baker, O Baker, OJ Baker, Olivia Baker, Thomas Baker, I Bakheit, Imad Bakheit, H Bakhit, Ahmed Bakhsh, B Bakhshayesh Eghbali, Akkasha Bakhtiar, F Bakhtiary, Khalid Bakier Mohammed, Batoul Bakkar, WJ Bakker, B Bakmaz, Bernarda Bakmaz, G Bakolas, Lubna Bakr, Abdelrahman Bakry, Ganesh Bakshi, R Bakx, Roel Bakx, Pavin Bal, Miklosh Bala, Abubakar Bala Muhammad, Vladimir Balaban, Mohamed Balabel, Abhinav Balachandar Subbiah Ramasamy, B Balagobi, Balasingam Balagobi, C Balague Ponz, A Balaguer Román, Andrés Balaguer Román, M Balaguer-Castro, Mariano Balaguer-Castro, Edward Balai, A Balakrishnan, Anita Balakrishnan, D Balalis, Dimitrios Balalis, Cosmin Balan, Julián Balanta-Melo, Reyes Balanzá, A Balaphas, D Balasubramaniam, Dinesh Balasubramaniam, Srikant Balasubramaniam, SP Balasubramanian, Supriya Balasubramanya, P Balau, Egine Balayan, H Balbaloglu, Mohammed Balbola, L Baldari, Ludovica Baldari, T Baldasso, G Baldazzi, C Baldi, Caterina Baldi, Manish Baldia, E Baldini, Edoardo Baldini, A Baldwin, AJ Baldwin, Melissa Baldwin, R Baldwin-Smith, Paolo Balercia, I Balescu, Riccardo Balestri, Ameera Balhareth, Krittika Bali, Oussama Bali, Lipika Baliarsing, MB Balictar, E Balik, Emre Balik, H Balkhi, Alasdair Ball, Alice Ball, L Ball, A Balla, Mohammed balla Yousif balla, M Ballabio, Mohammed Ballal, K Ballantyne, Eulalia Ballester, E Ballester Vazquez, Roberto Ballestero, Marta Ballesteros-Pomar, Monica Ballon, Q Ballouhey, Quentin Ballouhey, M Balluerca, Maria Balluerca, Brenda Balmaceda, R Balmaceda, Zsolt J Balogh, JA Balogun, Mosimabale Balogun, Simon Balogun, M Balouli, Maram Balouli, I Baloyiannis, Ioannis Baloyiannis, G Baltazar, Carlos Baltazar Branco, Andrea Balthazar, Saba Balvardi, F Bàmbina, VS Ban, Tuba Banaz, F Banchini, GB K D Bandara, A Bandiera, S Bandyopadhyay, Severine Banek, Ayan Banerjea, A Banerjee, Abhirup Banerjee, Shubhabrata Banerjee, Sumit Banerjee, Anant Bangar, Mohammed Bangash, P Bangeas, Qaed Bani Amer, Abdulsalam Bani Hamad, Morad Bani-hani, Eman Baninasr, N Baniyas, Oluseyi Banjo, OO Banjo, W Bank, Charles Banka, B Bankhead, B Bankhead-Kendall, Brittany Bankhead-Kendall, Thomas Banks, B Banky, E Bannone, Kuldeep Bansal, S Bansal, Sujesh Bansal, S Banting, Simon Banting, Anas Bany Issa, Hammam Bany yasin, Carina Banziger, Abubakar Bappah Jaafar, John Baptist Ssenyondwa, JC C Baptista-Silva, J Bapty, M Baquedano, Mai Baquedano, David Baquero, Ahmed Barakat, J Barakat Awada, Jamile Barakat Awada, Oussama Baraket, Perel Baral, E Baran, Elif Baran, A Baranov, Maxime Barat, Baratte Baratte, Vidmantas Barauskas, Frezza Barbara, Andrew Barbas, Giuseppe Barbato, A Barbazza, Y Barbé, Rafael Barberá, Cristina Barberio, A Barberis, Andrea Barberis, L Barbier, Luis Barbier, O Barbier, FJ Barbosa Camacho, Genival Barbosa Carvalho, Joao Barbosa-Breda, F Barbour, Ida Barca, Indalecio Barcelata Rodriguez, JC Barcelon, Elizabeth Bárcena, A Barcin, A Barclay, Laura Bardelli, Metaxia Bareka, Vanona barijaona Razafindraibe, Kathia Barillas, Sabrina Barillas, Goran Barisic, Tatjana Barišić, John Barker, Jonathan Barker, Sergey Barkhatov, C Barkolias, E Barkolias, AM Barlas, Adam Barlow, C Barlow, R Barmasse, B Barmayehvar, C Barmpagianni, L Barnard, Stephen Barnett, Isaac Barnor, Paolo Baroffio, R Baron, Ryan Baron, M Barone, Mirko Barone, R Barone, F Baroni Alves Makdissi, G Baronio, Gianluca Baronio, Salma Baroudi, F Barra, Fabio Barra, A Barrabe, AG Barranquero, A Barraquio, B Barrat, Benjamin Barrat, Mauricio Barreda, Diogo Barreiro, C Barrena lópez, Cristina Barrena López, E Barret, Alexandra Barreto, Analy Barreto Galeano, A Barreto Grimaldos, ZM Barrett-Brown, Diana Barretto, Belinda Barrientos Nuñez, JM Barrio, M Barrionuevo Ramos, Maria Barrionuevo Ramos, Maria Barrios Carvajal, A Barrios Duarte, Amalia Barrios Duarte, AV Barros, N Barros Jr, Hannah Barrow, J Barrow, C Barry, Jessica Barry, Mary Barry, Peter Barry, Stevie Barry, Ashish Bartakke, Andrea Bartalini Cinughi de Pazzi, B Bartalucci, JL Bartha Rasero, I Bartolini, Ilenia Bartolini, K Bartosiak, A Bartsch, Raquel Bartz, Dimitrios Bartziotas, Anupama Barua, G Barugola, Giuliano Barugola, Elisee Baruwa, Enes Baş, Vikas Basa, VS Basappanavar, Silvia Basato, Vladimir Bascarevic, M Basendowah, Mohammed Basendowah, A Basgaran, A Basha, Beibit Bashabayev, AK Basher, Aladdin Bashir, Alia Bashir, M Bashir, Osman Bashir, Rasha Bashir, Y Bashir, Ali Bashiri, V Bashkirova, Tayseer Basi, Francis Basimbe, Muhammad Basir, D Baskaran, Dinnish Baskaran, Oshan Basnayake, PS Basnyat, GA Bass, Ali Bassi, C Bassi, F Bassily, Muhammad Bassiouni, Shahin Bastaninejad, F Bastard, François Bastard, Mostafa Bastawesy, Alberto Basterra Rincon, Joaquin Bastet, Mae-Lynn Bastion, S Basu, Somprakas Basu, Utkarsha Basu, Mediatrice Batangana, A Bateman, Antony Bateman, S Baterl, MF Bath, Michael Bath, A Bathgate, RE Baticulon, Ronnie Baticulon, Oguzkagan Batikan, S Batista, Sylvia Batista, P Batistotti, HH Batjer, Brian Batko, Olivia Batog, Fizza Batool, Sehrish Batool, Nitin Batra, Martin Batstone, G Battello, Melanie Battershell, MJ Battista, Giovanni Battista Fonsi, Enrico Battistella, Marlies Bauer, R Baumber, Rachel Baumber, L Baumgart, S Baumgarten, Sabine Baumgarten, JB Baun, Mireia Bauzá, D Bavishi, Dauda Bawa, Kyriaki Baxevanidou, Z Baxter, Zachary Baxter, Muhannad Bayazid, J Bayer, Jörg Bayer, Z Bayhan, Zulfu Bayhan, Firew Bayissa, Morgan Bayley, J Bayne, A Bayomy, AbdulHakeem Bayomy, Gabriel Bayona-Alvarado, Ç Bayram, E Bayramov, A Bazaev, Andrey Bazaev, Borja Bazán Inostroza, Alberto Bazan Soto, Muhammad Bazil Musharraf, H Bazzi, M Bazzi, N Bazzi, Brian Bbosa, Manjunath Bd, Saja Bdour, David Beahm, P Beak, AJ Beamish, Carlos Beas Ruiz-Velasco, Ana Beatriz Calderon Alvarado, O Beaumont, V Bebia, R Becerra, FC Becerra García, Luis Becerra Mendez, N Bechar, H Bechri, Hajar Bechri, J Beck, Jürgen Beck, Manisha Beck, Renata Beck, Johannes Becker, Karin Becker, Andrew Beckett, Dereje Bedane, D Bedane Hunde, María Bedate Núnez, D Beddy, Antoinette Bediako Bowan, Alvaro Bedoya-Ronga, A Bedzhanyan, Arkady Bedzhanyan, Charlotte Bee, Helen Beech, N Beech, B Beelders, A Beer, Phillipa Beesley, Paul Beganton, E Begoña, Alvarez-ramos Begoña Aranzazu, María Begoña Gregorio Crespo, María Begoña Pastor Nieto, A Beguiristain, Adolfo Beguiristain, Vuqar Behbudov, P Behera, Prateek Behera, Kevin Behm, B Behmanesh, Michael Behr, Mona Behravesh, Abdollah Behzadi, Khalid Beidas, Klara Beitl, M Bejarano Serrano, Miguel Bejarano Serrano, Ephrem Bekele, K Bekele, Kebebe Bekele, Philimon Bekele, Mahteme Bekele Muleta, M Bekheit, E Bekhor, Fanny Belais, Laurence Belanger, Mathieu Belanger, P Belani, Othman Belarabi, B Belarbi, Armin Belarmino, E Belcher, Elizabeth Belcher, Raluca Belchita, Rocío Belda, Uros Bele, María Belén Alonso Bartolomé, Ana Belén Casas Marcos, Sofia Belen Diaz Pineda, Ana Belén Gallardo, María Belén Ramírez Senent, N Belev, Nikolay Belev, A Belgaumkar, Ajay Belgaumkar, AP Belgaumkar, Orkia Belhadri, Francesco Belia, Orimisan Belie, Diego Belisle, ZH Belkhadir, Susana Bella Romera, A Bellacci, L Bellanti, Luca Bellanti, V Bellato, Vittoria Bellato, D Bellemare, Jack Bellerby, A Belli, Andrea Belli, Gabriele Bellio, C Bellis, Sergi Bellmunt-Montoya, Funmi Bello, J Bello, Jibril Bello, L Bello, Kabiru Bello Abubakar, Shahir Bello Umar, Nafisatu Bello-Muhammad, F Bellolio, Paolo Bellora, T Bellos, O Bellou, E Beltrami, GA Beltramini, Larissa Beltran, Miguel Beltran, J Beltrán de Heredia, Juan Beltrán de Heredia, Pablo Beltran Miranda, P Beltrán-Miranda, A Belvedere, Angela Belvedere, Orlin Belyaev, Etienne Belzile, Anass Ben Amer, Esraa Ben esmael, Omar Ben Forge Risk, Mahmoud Ben ghrema, H Ben Hasan, Hayat Ben Hasan, N Ben Hasan, RAI Ben jouira, Emadeddin T M Ben Khalifa, M Ben Othmen, A Ben-Sassi, D Benali ammar, Y Benallal, Nassim Benallel, S Benamar, A Benamwor, Adeka Benard, EA Benavides Hernández, M Bence, Meryem Benchekroun Belabbes, L Bendjemar, Lynda Bendjemar, Semir Benecha, E Benedetti, F Benedetto, Magdalena Benegas, O Benet Muñoz, Giacomo Benettini, Maria Benevolo, Savitha Bengeri, Erika Bengtson, P Benharash, Peyman Benharash, Rema Benhariz, Kristina Benirschke, Oscar Benitez, Nicole Benitez Benitez, Ana Benítez Riesco, I Benítez-Linero, Miles Benjamin, MW Benjamin, Santosh Benjamin, A Benkabbou, Amine Benkabbou, M Benmamar, Fatma Benmasoud, S Bennett, M Benoit, M Bensghir, Mustapha Bensghir, Guy Benshetrit, EA B Bensi, Zaineb Benslimane, K Bensoltane, Charlotte Benson, Ruth Benson, Malissa Bentham, R Bento, Domenico Benvenuto Giuliani, R Berbash, German Berbel, Norberto Berber, Martin Berden, Gutiérrez Bérénice, Luigi Beretta, E Berg, C Bergamini, Carlo Bergamini, Andrej Bergauer, D Bergeat, Damien Bergeat, Eyerusalem Bergene, Julian Berger, A Bergeron, Nicole Bergmann, M Bergonzani, Michela Bergonzani, Alazar Berhe, Ataklitie Berhea, L Berikashvili, Müserref Beril Dincer, J Beristain-Hernandez, Jose-Luis Beristain-Hernandez, Loreto Berjon De La Vega, Muhammet Berkay Sakaoglu, Eva Berkeveld, MT Berlanga Rojas, Hugo Bermejo, Lorena Bermell Marco, AA Bernabé Esteban, M Bernabei, Massimiliano Bernabei, Silvana Bernadetta Puglisi, Fabio Bernagozzi, Aldo Bernal Hernandez, JC Bernal-Sprekelsen, P Bernante, Paolo Bernante, D Bernardi, Daniele Bernardi, Laura Bernardi, Martin H Bernardi, M Bernasconi, AE Berndtson, Allison Berndtson, Emily Berner, C Berney, Christophe Berney, M Bernon, V Bernotaite, Rania Berrami, Roberto Berretta, Stefano Berrettini, Sara Berrocal, Juan Berrocal Cuadrado, Pedro Berrones Moreno Berrones Moreno, F Berrospi, Francisco Berrospi, Brendan Berry, G Berry, Janet Berry, Richard Berry, Bruno Berselli, M Berselli, Mattia Berselli, G Bertelli, Nicolas Bertheuil, Pierre Berthoumieu, L Bertoglio, Luca Bertoglio, P Bertoglio, Pietro Bertoglio, L Bertolaccini, Luca Bertolaccini, Elisa Bertolani, G Bertoli, Francesca Bertolina, Giacomo Bertolini, Marta Bertrand, Coro Bescós, T Bese, Tugan Bese, Hasan Besim, WF Besira, Marc Besselink, N Besser, Nikolaos Bessias, A Besson, Alex Besson, Lauren Best, D Beswick, Daniel Beswick, J Betalleluz Pallardel, Jenner Betalleluz Pallardel, Alva Bethurum, Nagat Bettamer, Ricardo Bettencourt Morais, J Bettoni, Jérémie Bettoni, CS Betz, Alexandra Beuca, K Bevan, E Bevilacqua, J Bewarder, Julian Bewarder, Gaym Beyene, K Beyer, Katharina Beyer, YS Bezabih, C Bezede, Cosmin Bezede, TS Bezerra, Andriy Beznosenko, Ashwin Bhadresha, S Bhagat, Bhuvanshyam Bhaktavatsalam, Bhalchandra Bhalerao, A Bhalla, Ash Bhalla, Rohan Bhalla, Anuradha Bhama, AR Bhama, Shivam Bhanderi, A Bhangu, Balamurali Bharathan, Rohit Bhardwaj, Aman Bhargava, Manoj Bharucha, S Bhasin, Dhananjaya Bhat, S Bhat, K Bhatia, Kailash Bhatia, M Bhatia, Mohit Bhatia, Anuj Bhatnagar, DRK Bhatta, G Bhatta, Gakul Bhatta, P Bhattacharya, S Bhattacharya, AB H Bhatti, Arun Bhatti, Hamza Bhatti, Khalid Bhatti, Samiullah Bhatti, Waqar Bhatti, A Bhavaraju, Avi Bhavaraju, Vishal Bhende, D Bhojwani, Deepika Bhojwani, DP Bhor, Pramod Bhor, S Bhudia, S Bhusal, Subarna Bhusal, N Bhutiani, Neal Bhutiani, Shameen Bhutto, T Bhuvanakrishna, M Biala, Marwa Biala, Alessia Biancafarina, E Biancardi, A Bianchera, Lorenzo Bianchi, Valentina Bianchi, M Bianchini, Agustín Bianco, F Bianco, Francesco Bianco, Giuseppe Bianco, M Biasini, D Biasoni, David Biau, L Bibby, S Biber, B Biccard, Bruce Biccard, David Bichell, Jordan Bickerdyke, J Bicki, Heena Bidd, Lauren Bidois, M Biebl, Mumbere Bienfait, Johannes Bier, W Bierman, Christopher Bierton, W Biffl, Walter Biffl, D Bigam, David Bigam, Katherine Bigay, Benjamin Bigelow, A Biggs, Michael Biggs, Sarah Biggs, P Bigot, Okker Bijlstra, Yemurai Bikwa, Saad Bilal Ahmad, Mustafa Bilal Hamarat, Javeria Bilal Qamar, Ali Bilal Ulas, H Bileid Bakeer, Roman Bilenko, M Bilfaqirah, I Biliatis, Ioannis Biliatis, A Billè, Mestan Bilmez, Jerko Biloš, Kim Bin, Manerh Bin Mosa, A Bin Nasser, Ahmad Bin Nasser, Sari Bin nour, Fayez Bin Omran, Khalid Bin Saad, Osama Bin Sohail, Sinan Binboga, Elif Binboğa, Barbara Binda, A Binder, AD Binder, Alf-Dorian Binder, J Binder, Johannes Binder, Ahmed Binjaloud, M Binnawara, Faiqa Binte Aamir, Bojan Biočina, Alberto Biondi, Massimo Biondi, Vedrana Biosic, Garance Biosse-Duplan, Hari Bipin Radhakrishnan Kattana, David Bird, Sophie Bird, E Birgin, Nuha Birido, Arianna Birindelli, Elisa Birnbaum, Erdal Birol Bostanci, SL Birolo, G Birqeeq, P Bisagni, Theodosios Bisdas, Tayfun Bisgin, AK Bisoi, Daniele Bissacco, Guido Bissolotti, Maria Bisulli, Samer Bitar, S Bitsianis, MN Bittar, Reinhard Bittner, Kristina Bitunjac, Achille Bizimana, Yemisirach Bizuneh Akililu, Karin Björnström Karlsson, Peter Black, James Blackwell, Michael Blackwell, J Blair, James Blair, Anne-Sophie Blais, I Blake, Nikita Blake, T Blanc, Claire Blanchard, David Blanco, J Blanco, Lara Blanco Terés, R Blanco-Colino, Nyinawabagesera Blandine, JL Blas Laina, J Blasco-Moreu, L Blasco-Torres, Alison Blatt, Alejandro Blaubach, Ben Blay Ofosu-Barko, Aida Blaya, Krešimir Blažević, A Blazquez Martin, Cait Bleakley, S Bleda, Silvia Bleda, S Bleibleh, Sabri Bleibleh, N Blencowe, Natalie Blencowe, NS Blencowe, C Blier, Jeremy Bliss, Frank Bloemers, Nina Blomme, O Bloom, C Blundell, Benedict Boakye, M Boal, T Board, Timothy Board, Rachael Boardley, Abigail Boateng, P Bobak, Peter Bobak, Bailea Bobich, Marcin Bobiński, Dino Bobovec, G Bocca, A Boccabella, L Boccalatte, LA Boccalatte, Luis Boccalatte, G Bocchialini, Antonio Bocchino, Melissa Bochner, J Bock, Jacob Bock, Wolfgang Böcker, Gabriela Bocsa, Guillaume Boddaert, A Boddy, Alex Boddy, Chris Bode, CO Bode, AS Bodla, Zsolt Bodnar, M Boeck, Marissa Boeck, A Boeckxstaens, C Boeker, Clara Boeker, Lars Boenicke, Andreas Boening, Catherine Boereboom, D Boerma, E Boerma, EG Boerma, Evert-Jan Boerma, MA Boermeester, G Bogani, Giorgio Bogani, Amalia Bogarin, M Bogdan, Monica Bogdan, Andrei Bogdan Văcărașu, Aleksandar Bogdanovic, Ivan Bogdanovic, Manobhiram Boggavarapu, Selene Bogoni, Matthieu Boisson, S Bojic, Jovana Bojičić, M Bokenkamp, Mary Bokenkamp, Areej Bokhari, Covalic Bokossa, M Boland, F Bolanos-Morales, Francina Bolanos-Morales, Raikhan Bolatbekova, A Bolbarán, Christian Bolenz, Emmanuel Boleslawski, Jarlath Bolger, S Boligo, Rafik Bolis, Dinimo Bolivar Saenz, Enton Bollano, M Bolli, S Bolognesi, Silvia Bolognesi, J Bolota, Joana Bolota, A Bolouriyan, M Bolster-van Eenennaam, John Bolt, Basak Bolukbasi, D Bona, Davide Bona, E Bonaiuto, Marta Bonaldi, G Bonavina, Giulia Bonavina, L Bonavina, Luigi Bonavina, E Bonci, Eduard-Alexandru Bonci, G Bond-Smith, Peter Bonde, A Bondurri, Andrea Bondurri, Mireya Bonet, Barbara Bonfanti, Christopher Bonfield, D Bonfili, C Bong, L Boni, Luigi Boni, A Bonilla, Ana Bonilla, Carlos Bonilla, F Bonilla, Fernando Bonilla Cal, PV Bonilla Sanchez, FJ Bonilla-Escobar, A Bonnard, Soline Bonneau, Stéphane Bonnet, Jorge Bonnin, Stefano Bonomi, J Bontinck, Julie Bontinck, Kian Boon Wong, K Booth, M Boras, Miran Boras, Sorour Borayek, G Borda-Luque, Giuliano Borda-Luque, M Bordenave, P Bordoni, Pierpaolo Bordoni, A Borello, Elaine Borg, Jeremy Borg Myatt, F Borges, Mafalda Borges, N Borges, F Borghi, Felice Borghi, AB J Borgstein, Alexander Borgstein, Morteza Borhani, Kurosch Borhanian, F Boriani, U Bork, Ulrich Bork, H Borla, Hernan Borla, N Börner, Nikolaus Börner, Emanuel Borovic, David W Borowski, B Borraccino, R Borreca, VM Borrego Estella, G Borroni, Giacomo Borroni, D Borselle, M Borselli, Kim Borsky, Biplob Borthakur, L Bortolasi, G Bortolin, Carlo Bortolotti, Marina Bortul, Maksym Boruta, A Borzacchelli, Ana Bosak Versic, D Bosanquet, David Bosanquet, KD Bosch, Marina Bosch, D Bosch Garcia, David Bosch Garcia, M Bosch-Ramírez, J Boschet, Paolo Boscolo Rizzo, Lorenzo Bosio, Raul Bosio, EB Bostanci, Lais Botacin, Pedro Botelho, Vipul Bothara, Jyoti Bothra, Iva Botica, Carlos Boto, A Bottari, Andrea Bottari, A Böttcher, Arne Böttcher, L Boualila, Lina Boualila, R Bouanane, Othmane Bouanani, S Bouaoud, Souad Bouaoud, K Bouchagier, Konstantinos Bouchagier, M Bouchard, PA Bouche, Pierre-Alban Bouche, Kamel Bouchenak, S Boucher, Sophie Boucher, Sofia Boucher-Kovalik, A Bouchetara, N Bouchiba, R Boudou, Rocio Boudou, Judy Boughey, A Bouhuwaish, Ahmad Bouhuwaish, Alassan Boukari, AZ Boukli Hacene, Cindy Boulanger-Gobeil, K Bouliaris, Konstantinos Bouliaris, A Boulton, AJ Boulton, Natacha Boumas, A Bourial, G Bourke, Grainne Bourke, E Bourmpouteli, Anna Bouronikou, S Boussedra, Safia Boussedra, Saber Boutayeb, N Boutimzine, M Boutros, RM Bouttelgier, LA Bouziane, Raffaele Bova, S Boveda gonzalez, Claudio Bovolenta Murta, Conor Bowe, D Bowen, J Bowen, Joel Bowen, Christopher Bowler, S Bowman, T Bowman, Louis Boyce, H Boyd-Carson, Hannah Boyd-Carson, Joshua Boyes, C Boyle, Connor Boyle, E Boyle, Ellen Boyle, T Boyle, Rebecca Boyles, K Bozada Gutierrez, Katya Bozada-Gutiérrez, A Bozakok, E Bozdağ, Bahadır Bozkırlı, E Bozkurt, Emre Bozkurt, MA Bozkurt, Nulvin Bozo, Adel Bozorgzadeh, Antonio Bozzani, Andries Braat, Umberto Bracale, G Brachini, Gioia Brachini, Muriel Brackstone, Molly Bradbury, Thomas Bradley, Catherine Bradshaw, CJ Bradshaw, L Bradshaw, Luke Bradshaw, S Bradulskis, A Braga, Maria Bragado González, H Braham, D Brahmbhatt, K Brahmbhatt, Yasmine Braimah, Konstantinos Bramis, Irene Brana, G Branagan, Graham Branagan, C Branco, Mariana Branco Lopes, Marcelo Brandao, R Brandariz, Rodrigo Brandariz, Jury Brandolini, A Branquinho, R Branquinho, Rita Branquinho, D Branzan, Daniela Branzan, A Brar, Amanpreet Brar, T Brasileiro Silva Pacheco, Benjamin Braslow, C Brasset, G Brat, C Brathwaite, CE M Brathwaite, Nikolina Bratošević Vučičić, D Bratt, D Bratus, Dejan Bratus, T Bratuš, C Braumann, Chris Braumann, Mauro Bravo, SL Bravo, SL R Bravo, Layze Braz de Oliveira, Esther Brea Gómez, S Breakeit, Sarah Breakeit, D Breda, H Breda Pessetti, Mikhail Bredikhin, K Breen, Kerry Breen, R Breheret, Renaud Breheret, O Breik, Omar Breik, Zdrinko Brekalo, Mark Bremholm Ellebaek, Signe Bremholm Ellebæk, C Brennan, Caitlin Brennan, P Brennan, Paul Brennan, F Brennfleck, Tiago Bresciani, F Bretagnol, C Bretherton, Christopher Bretherton, C Brett-Miller, RG Breuer, B Brew, H Brewer, Hilary Brewer, E Brian, D Briatico, SK Bridges, Elsie Bridgman, Carolina Brienze, Tim Bright, Rhiannon Brignall, L Brignone, Pradeep Brijkishor Sharma, Svetlana Brincat, Lukas Briner, Rebecca Brinkler, Petra Brinskelle, Edulfo Britez Barrios, Kattiucy Brito, Francisca Brito da Silva, Analia Britos, Tim Brits, E Britton, John Britton, L Britton - Zier, Linda Britton-Zier, Ariberto Brivio, G Brixton, Genevieve Brixton, Lucija Brkic, Samuel Broadbent, Phoebe Brobbey, A Broch, J Brockwell, C Broe, Claire Broe, Tobias Broecheler, Alessandro Broglia, N Brogly, A Brolese, Alberto Brolese, M Brolese, E Brolo, Estuardo Brolo, S Bromage, Stephen Bromage, H Bronger, Mark Brooke-Smith, P Brouk, Peiman Brouk, P Brouki Milan, Peiman Brouki Milan, T Brow, A Brown, Allison Brown, Andrew Brown, B Brown, BC Brown, Benjamin Brown, C Brown, Christopher Brown, D Brown, IG Brown, J Brown, James Brown, L Brown, O Brown, S Brown, Sarah Brown, SR Brown, V Brown, Victoria Brown, Wendy Brown, L Brown Fumeau, R Bruballa, Nolan Bruce, Jan Bruder, Elizabeth Bruenderman, Nicolás Bruera, Carlo Brugiotti, Marcos Bruna Esteban, Laurent Brunaud, A Brunelli, Alessandro Brunelli, Aina Brunet-Garcia, Federica Brunetti, Eberhard Brunner, SM Brunner, Stefan M Brunner, U Brunner, Chiara Bruno, E Brunocilla, Christiane Bruns, A Brunt, Luca Bruschini, A Bruscino, Alessandro Bruscino, P Bruzzaniti, Placido Bruzzaniti, F Brzeszczyński, Filip Brzeszczyński, Domagoj Brzic, R Bschorer, I Buarque, Igor Buarque, IL Buarque, Amina Buba, Olha Bubliieva, Pamela Buchwald, Benjamin Buckland, Abbigayle Buckton-Perkins, Georges Bucyibaruta, Alina-Maria Budacan, Karel Buddingh, O Budha Magar, Veronika Budyakova, R Buenaño González, Javier Buendia Pérez, AD Bueno Cañones, Luz Bueno Rey, GA Buerba, T Bueser, Teofila Bueser, Marco Bueter, K Buffenoir, Daniele Bugada, N Bugdayci, Mumtaz Bughio, D Buğra, Dursun Buğra, Lily Builth-Snoad, JJ P Buitendag, Acosta Buitrago, Miguel Buitrago, Cristina Bujoreanu, Mohammed Bukari, SI Bukhari, Walid Bukhari, Ruth Bulder, M Buljubasich, Martin Buljubasich, D Bulthé, Danny Bulthé, M Bulugma, ND Bulut Yüksel, U Bumbasirevic, Uros Bumbasirevic, Boris Bumber, Francesca Bunino, J Bunni, Gisele Bunogerane Juru, Andres Bur, A Burahee, Ahmet Burak Ciftci, Muhammet Burak Kamburoğlu, Cemil Burak Kulle, Arturo Burchakchi, Emine Burcu Cigsar, Lukáš Burda, Eleanor Burden, Gemma Burdge, L Burdine, Lyle Burdine, Zoe Burdon, S Burg, Simon Burg, Dania Burgan, Julio Burgos, Barbara Burgos-Blasco, Mohammad Burhan Khan, M Burhan Ul Haq, Cathy Burke, E Burke, JR Burke, C Burks, Ciersten Burks, C Burlew, Clay Burlew, Nikita Burlov, Alexandr Burmistrov, S Burnard, N Burnside, Nathan Burnside, J Burtscher, Johannes Burtscher, FE Buruiana, Musa Busarira, Opeyemi Busayo Borokinni, Chia-Jung Busch, CJ Busch, Hassan Bushaala, Raisa Bushra, Ayşe Büşra Önder, N Busse, Edoardo Bussolin, Lara Bußmann, Francesco Bussu, Juan Bustamante-Munguira, Nasir Bustangi, Laura Busto, Sara Busto Suarez, Ronald W Busuttil, Marko Buta, T Bute, C Butler, Charles Butler, John Butler, Roshan Butt, U Butt, Usman Butt, W Butt, Martin Buttaro, W Butterworth, Giovanni Butturini, A Butyrskii, Aleksandr Butyrskii, Alexis Buunaaim, Süleyman Büyükaşık, Çağrı Büyükkasap, Dmitrii Buzanakov, M Buzejic, Kefas Bwala, KJ Bwala, Matthew Bye, F Byiringiro, Fidele Byiringiro, J Byrne, Matthew Byrne, MH V Byrne, Edward P Bywater, Savitha C, JM Cabada Lee, N Caballero Otálora, Alejandra Caballero salas, VD Caballero Sarabia, J Caballero-Alvarado, Alberto Cabañero Sánchez, Pedro Cabeça Santos, A Cabeleira, Carmen Cabeza Oliver, K Cabillas, Ana Cabral, Daniel Cabreja, Marino Cabrera, PA Cabrera, Wilton Cabrera Cruz, PA Cabrera Rivera, E Cabrini, Elisa Cabrini, R Cabula, M Caccetta, Crescenzo Cacciapuoti, NA Cáceres Cárdenas, LE Cadena Castro, P Cadenelli, Pierfrancesco Cadenelli, A Cadersa, Luciana Cadore Stefani, Sarah Cadwell-Sneath, S Cafarotti, M Caffo, Maria Caffo, EP Cagigal Ortega, Deniz Caglar, Mehmet Çağlar Çakıcı, Elena Cagnazzi, Hüseyin Cahit Yalçın, R Cahyono, Tommaso Cai, A Caiado, André Caiado, Camilo Caicedo, Lina Caicedo, M Caicedo Toro, Bartomeu Caimari, François Caire, Alison Cairns, Scott Cairns, P Caja Vivancos, Patricia Caja Vivancos, Ensar Çakır, G Cakmak, Guner Cakmak, Gül Çakmak, F Calabrese, F Calabretto, M Calabrò, Marcello Calabrò, E Calcerrada Alises, Marta Calderón, Aranzazu Calero-Lillo, B Calik, Bulent Calik, F Calikoglu, Fikret Calikoglu, Ana M Calinescu, G Calini, Ş Çalık, AS Çalış, MP Callahan, R Callan, C Callari, Cosimo Callari, R Callcut, Rachael Callcut, Paola Calleja Hermosa, Laura Calles-Sastre, Alexandra Calmels, V Calu, Valentin Calu, J Calvache, JA Calvache, Jose Calvache, Jorge Calvera, P Calvo Espino, Pablo Calvo Espino, Marta Calvo Fernández, Raul Calvo Gonzalez, A Calvo Rey, Jitoko Cama, C Camacho, Aldo Camacho Gomez, F Camacho Zacarías, P Camacho-Carrasco, Diego Camacho-Nieto, Jaume Cámara Cabrera, Marina Cámara Vallejo, E Camarero, Enrique Camarero Rodríguez, Daniela Camargo Gómez, K Camargo-Parra, Marta Camats Terré, William Cambridge, Amisha Cameron, Iain Cameron, RB Cameron, Robert B Cameron, Joan Camí, María Camila Carvajal, María Camila Leyva Martínez, April Camilla Roslani, A Camillo, Juan Camilo Salcedo Moreno, NG Caminsky, E Cammarata, Emanuele Cammarata, F Cammarata, L Camp, Lauren Camp, Khaled Campa, Luca Campagnaro, T Campagnaro, Tommaso Campagnaro, N Campain, Sofia Campanella, M Campanelli, Michela Campanelli, Abigail Campbell, Cassidy Campbell, Katie Campbell, R Campbell, W Campbell, William Campbell, Paula Campelos Fernández, P Campennì, Paola Campennì, Flaminia Campo, Ana Campos, J Campos, Jose Campos, Elena Campos Carot, Juan Campos Garcia, FE Campos Montoya, N Campuzano, Nicolás Campuzano, Borja Campuzano Bitterling, B Campuzano-Bitterling, M Camuera, Maite Camuera, U Can, Ugur Can Dulger, Ahmet Can Sarı, Ozan Can Tatar, MªPilar Canals Sin, A Canas-Martinez, B Canbay Torun, M Candan, Mert Candan, Susana Candeias Rodrigues, M Candiani, Massimo Candiani, Giorgio Candotti, E Canelles Corell, Ruben Canelo Professor, C Canhoto, Dalibor Cankoski, J Cann, A Cannavera, Alessandro Cannavera Putzu, M Cannoletta, O Cano, V Cano Busnelli, Virginia Cano Busnelli, J Caño Velasco, Jorge Caño Velasco, E Cano-Trigueros, Emiliano Cano-Trigueros, A Canonico, G Canonico, Giuseppe Canonico, Rita Canotilho, Samir Canovic, Elif Cansu Gundogdu, M Cantalejo-Diaz, Daniel Cantero, Miriam Cantos, Stephen Canty, Ulrich Canzler, Daniela Canzonieri, H Cao, R Capanna, B Capdevila Vilaro, B Capdevila Vilaró, Blanca Capdevila Vilaró, Nathalia Capellan, P Capelli, L Capezzuoli, Daniel Capitaine, L Capitan-Morales, Luis-Cristobal Capitan-Morales, P Capitani, H Capitelli-McMahon, V Capizzi, Giampiero Capobianco, R Capoglu, Recayi Çapoğlu, GT Capolupo, Serge Cappeliez, Alessandro Cappellani, Antonio Cappiello, Giovanni Capretti, M Caputo, Maria Caputo, Massimo Caputo, M Capuzzo Gonçalves, Mateus Capuzzo Gonçalves, A Carabias, Petru Caraja, F Carannante, Filippo Carannante, C Carapinha, Andrea Caravati, Inez Carballo, MI Carballo, F Carbone, L Carbone, E Carbonneau, G Carcano, P Carcoforo, Stefano Cardelli, Daniel Cardenas, T Cardenas, Jimmy Cárdenas Coaquira, F Cárdenas Escalante, Fernando Cárdenas Escalante, Laura Cárdenas Puiggrós, D Cárdenas Ruiz de Castilla, J Cardenas-Gomez, Kristin Cardiel Nunez, Etienne Cardinal, Luca Cardinali, Uriel Cardona, Monica Cardona Marin, Cláudio Cardoso, J Cardoso, N Cardoso, Nicole Cardoso, P Cardoso, Paulo Cardoso, Andre Cardoso Almeida, Roberto Cardoso Cardoso dos Santos, Fabiana Cardoso Pereira Valera, Cintia Cardoso Pinheiro, M Caretto, Iva Carevic, C Carey, Charles Carey, M Caricato, Marco Caricato, Jochem Caris, F Carissimi, Giuseppe Caristo, Janaína Carla da Silva, Renan Carlo Colombari, Giorgio Carlo Ginesu, Luca Carlo Nespoli, A Carlos, W Carlos, William Carlos, Jose Carlos Barcelon, Juan Carlos Bernal-Sprekelsen, Juan Carlos Catalá Bauset, João Carlos Costa de Oliveira, Juan Carlos Dueñas-Ramirez, Juan Carlos Enciso, Juan Carlos Ibarrola Peña, Juan Carlos Martín del Olmo, Juan Carlos Navarro, Juan Carlos Rodríguez-Sanjuán, Juan Carlos Sabogal Olarte, P Carlos Santos, Laura Carlson, M Carlucci, Ofra Carmel, Maria Carmela Giuffrida, Marie Carmela Lapitan, Ana Carmen Carbajal, María Carmen Cervera, Maria Carmen Suescun López, H Carmichael, Heather Carmichael, Alyssa Carmina Almelor, Tomas Carminatti, Lizette Carmona, A Carnevali, Adriano Carnevali, E Carnicer Escusol, Esmeralda Carnicer Escusol, A Caro, Cristina Caroça, Maria Carolina Castillo Florez, Andrea Carolina Perea Serna, Ana Carolina Scintini Herbst, Ana Carolina Tagliatti Zani, Amanda Caroline Dawson, Diana Carpaneto, Osvaldo Carpineto Samorani, Antonio Carpino, YT Carpio Colmenares, S Carrabetta, R Carramiñana Nuño, FM Carrano, J Carranza Sarmina, Barbara Carrara, Yolanda Carrascal, M Carrasco Prats, Milagros Carrasco Prats, M Carrasco-Prats, Carla Carratalá Pérez, R Carreira Garcia, Carolina Carreiro, Guillermo Carreño, Jacqueline Carrera, A Carreras-Castañer, Anna Carreras-Castañer, Alessandro Carretta, MM Carrick, FM Carrier, M Carrillo-Rivas, Mariana Carrillo-Rivas, E Carrington, Diego Carrion, Conor Carroll, Jesse Carroll, PA Carroll, Paul Carroll, Daniel Carson, Samuel Carson, WS Cartagena, J Carter, Clifford Caruana, Edward Caruana, Edward J Caruana, EJ Caruana, Ed Caruna, Ambra Caruso, Gerardo Caruso, J Caruso, James Caruso, Carolina Carvajal Calderón, AA Carvalho, GB Carvalho, Joanna Carvalho, L Carvalho, M Carvalho, MF Carvalho, VC Carvalho, Vladimir C Carvalho, MM Carvello, Karen Carver, Shea Carver, M Carvill, Riccardo Casadei, Alfonso Casado, Biagio Casagranda, Mauro Casagrande, Núria Casanova Torrequebrada, Andrea Casaril, J Casarin, Claudia Casarini, Estefania Casas, Felipe Casas J, Marcos Casas Sánchez, M Casati, Massimiliano Casati, Ottavia Caserini, L Casetti, R Casey, Rowan Casey, Arianna Casiraghi, T Casiraghi, Florence Caslake Holding, Gianmaria Casoni Pattacini, C Cassinello, D Cassini, E Cassinotti, Elisa Cassinotti, Francesco Castagnini, Antonio Castaldi, Néstor Castán Villanueva, W Castañeda, Ivan Castañeda Giacometto, António Castanheira, S Castanheira Rodrigues, Sara Castanheira Rodrigues, Mario Castaño, AM Castaño-Leon, Ana M Castaño-Leon, Fabiola Castedo, Rute Castelhano, Christoph Castellani, J Castellanos, A Castells, K Castillo, Maria Castillo, Laura Castillo Pardo, Cameron Castle, FB Casto, C Castoro, Carlo Castoro, EJ Castro, Emma Castro, Marinelle Castro, S Castro, Filipe Castro Borges, Beatriz Castro Catalan, R Castro de la Mata, C Castro Ruiz, M Castro Suárez, Marta Castro Suárez, F Castronovo, JC Catalá Bauset, V Catalán, Sandra Cataldi, Sergiu Catalin Baraian, Ioan Catalin Vlad, J Catarino, L Catarzi, Lisa Catarzi, Mariano Catello Di Donna, F Catena, Fausto Catena, Andrew Caterson, N Cathala, Nathalie Cathala, Russell Cathcart, X Cathelineau, Valerie Catherine Linz, LD Cato, Bruno Catoia fonseca, S Cattaneo, J Catto, James Catto, A Catton, MM Caubet, M Cauteruccio, Michele Cauteruccio, A Cavalea, Alexander Cavalea, S Cavaleiro, Davide Cavaliere, P Cavallé Busquets, Matteo Cavallo, Francesca Cavenago, L Cayetano Paniagua, Ladislao Cayetano Paniagua, Valentín Cayuela, M Cazador Labat, Antoine Cazelles, A Caziuc, R Cazzaniga, S Cebi, Sukru Cebi, Laura Cebolla Rojas, L Cecchini, I Cecconello, E Cehic, Albertas Cekauskas, F Celebi, Fehmi Çelebi, AP Celi-De La Torre, B Celik, P Cellerino, Paola Cellerino, Claudia Celotti, Ahmet Cem Dural, S Cenciarelli, Sabine Cenciarelli, Rosario Cennamo, Omer Cennet, Ana Centeno Álvarez, Jorge Centeno Lozada, Alvaro Centeno Velasco, Jessica Centurión, Carmen Cepeda-Franco, G Cepele, Miljan Ceranic, Marco Cereda, Julian Cereghini, Ülkü Ceren Köksoy, Marco Ceresoli, Petra Čerina, C Cernei, Ondrej Cerny, Jan Černý, A Cerovac, Anis Cerovac, Cristina Cerri, C Cerro Zaballos, M Cervellera, I Cervera, Iria Cervera, S Cervera, Sergio Cervera Bonilla, Andrea Cerveró, Laura Cervini, Giovanni Cesana, Andrei Cesar Abella, Pablo Cesar Arteaga Asensio, Francisco César Becerra García, Matteo Cescon, Z Çetinkaya, Mehmet Ceyhan, Hilal Chaaban, Abou Chaar, Mohammad Chaar, Reem Chabaan, Carolyn Chabuz, S Chackan, Hannah Chacon, R Chadha, Radhika Chadha, S Chadha, Sami Chadi, T Chaki, Tomohiro Chaki, Sohini Chakrabortee, Koyel Chakraborty, Marc Chalhoub, B Challacombe, D Chalo, Alexandre Chamouni, Pierre-Olivier Champagne, Albert Chan, Annie Chan, Carlos Chan, CD Chan, CH Chan, Corey Chan, Elliot Chan, Eunice Chan, J Chan, L Chan, M Chan, Matthew Chan, S Chan, Bruno Chan Chin, Aye Chan Thu, Prem Chana, Gyan Chand, M Chand, Kshitija Chandanwale, K Chandarana, Karishma Chandarana, Danny Chandla, Amarbaj Chandock, Susilo Chandra, Karthik Chandra Vallam, C Chandrakumar, Bhargavi Chandrasekar, Pramodh Chandrasinghe, N Chandratreya, Nitya Chandratreya, G Chang, Grace Chang, Steven Chang, Abera Chanie, K M M vishvak Chanthar, H Chanty, Lidya Chanyalew, Ghita Chaoui, Reema Chapatwala, BK Chaplin, Brandon Chapman, Tracy Chapman, Preetam Chappity, A Charalabopoulos, Alexandros Charalabopoulos, Vasileios Charalampakis, H Charbonneau, Helene Charbonneau, Eduard Charchyan, L Chardalias, Leonidas Chardalias, A Chari, S Charles, Shane Charles, Gabriella Charlton, L Charre, T Chartab Mohammadi, Emmanuel Chartier-Kastler, T Chase, Muhammad Chatni, Debarshi Chatterjee, S Chatterji, Somashree Chatterji, Somnath Chattopadhyay, A Chaturvedi, Arun Chaturvedi, Arvind Chaturvedi, P Chatzikomnitsa, M Chatzikonstantinou, Sohin Chaudhari, Vikram Chaudhari, Ramkaran Chaudhary, S Chaudhary, Umer Chaudhry, Madhu Chaudhury, H Chaudry, G Chauhan, Sandeep Chauhan, Akhilanand Chaurasia, Rahul Chavan, N Chavarrias, Nuria Chavarrias, Vijay Chavda, G Chavez, J Chávez Pacheco, C Chavez Rivaldi, Cristhian Chavez Rivaldi, Sergio Chávez Valladares, Petr Chavkin, Priyank Chawathe, Shahidah Che Alhadi, NA Che Bakri, James Chean Khun Ng, A Chebaro, Alexandre Chebaro, Michel Chebel, C Checcucci, Carlotta Checcucci, SY Chee, Nabeel Cheema, R Chelva, Ruth Chelva, A Chen, D Chen, F Chen, Ji Chen, John Chen, Lee-lynn Chen, Lee-may Chen, Paul Chen, Pengchi Chen, Si Chen, Tony Chen, Yuan Chen, Zehua Chen, Arthur Chen Wun Tan, Jade Chen Zhao, D Cheng, Davy Cheng, Antony Chengahomwe, Martin Chenu, YJ Cheong, Wai Cheong Soon, Nathalie Chereau, Lisa Cherian, O Cherkaoui, Zineb Cherkaoui, Huey-Lan Chern, Viktor Cherniienko, Roman Chernikov, Maxim Chernykh, Davies Cheruiyot, Jayant Cherukat, E Cherullo, A Chessa, Antonella Chessa, G Chetty, J Cheuk, J Cheung, Kin Cheung Ng, M Chevallay, Dylan Chew, Kenneth Chew, M Chew, Michelle Chew, Natalie Cheyne, Ernest Cheyuo, HS Chhabra, R Chhabra, Swati Chhatrapati, David Chi Hau Tan, Leslie Chi Yan Cheung, Z Chia, Zoe Chia, C Chiang, Chu-Hao Chiang, Ryan Chiang, Vito Chiantera, Valentina Chiappa, C Chiappe, MF Chiappetta, A Chiappini, Marco Chiarelli, R Chiarpenello, M Chiarugi, Massimo Chiarugi, Yoshihiko Chiba, Ihediwa Chibuike George, S Chidambaram, N Chidumije, Nnaemeka Chidumije, Christopher Chien Liang Liao, Roberto Chiesa, I Chik, Maxwell Chimhina, Theresa Chin, Hiong Chin Lim, John Chinda, Rosanne Ching, Tsz Ching Chang, Simbarashe Chinyowa, Mieko Chinzei, Franco Chioffi, A Chiow, Adrian Chiow, M Chiozza, I Chipurovski, Francesca Chircop, P Chiriapanda uthappa, C Chirico, Carlos Chirico, Radu Chirvasuta, Emiliano Chisci, U Chishti, Uzma Chishti, Meer Chisthi, T Chituku, Tsitsi Chituku, A Chitul, Andrei Chitul, JA Chiu, Shelton Chivanga, C Choi, D Choi, David Choi, Sarah Choi, H Cholewa, Hanna Cholewa, Alyssa Chong, C Chong, L Chong, Lynn Chong, Yew-Lam Chong, C Choo, Candy Choo, J Choo Jun Hao, E Choolani Bhojwani, Ekta Choolani Bhojwani, P Choong, Peter Choong, Sophie Chopinet, Rupali Chopra, S Chopra, R Choron, S Chotai, Silky Chotai, Ravi Chotalia, Richard Chou, A Chouakria, Narendra Choudhary, A Choudhry, Asad Choudhry, Yousuf Choudhury, RS Chouhan, E Chouillard, Elie Chouillard, MF Chowdhry, A Chowdhury, Abeed Chowdhury, M Chowdhury, Mahbub Chowdhury, S Chowdhury, Sharfuddin Chowdhury, Shihab Chowdhury, Nisar Chowdri, D Chrastek, MA Christensen, P Christensen, Peter Christensen, Bordianu Christian, Nicole Christian, Franz Christian Horstmeier, Johannes Christian Lauscher, Jean Christian Urimubabo, A Christiano, P Christidis, Panagiotis Christidis, Adam Christie, Julia Christina Kaiser, Ekaterini Christina Tampaki, Elaine Christine Dantas Moises, Ruth Christine Schäfer, Gregory Christodoulidis, M Christodoulou, D Christoforidis, Dimitrios Christoforidis, Alexander Christopher Rokohl, C Christou, CD Christou, Chrysanthos Christou, N Christou, Niki Christou, Angeli Christy Yu, Megan Chrysikopoulou, E Chrysos, Emmanuel Chrysos, A Chrysovergis, Aristeidis Chrysovergis, G Chrysovitsiotis, Georgios Chrysovitsiotis, F Chu, Francesco Chu, H Chu, K Chu, Andre Chu Qiao Lo, A Chua, HW Chua, Richelle Chua, CT Chuah, Alwin Chuan, Rachel Chubsey, KM Chue, Kwok Chuen Wong, Ankita Chugh, Jason Chui, K Chui, I Chukwu, Isaac Chukwu, Sunil Chumber, Felix Chun, Mohsin Chundrigar, Tariq Chundrigar, C Chung, E Chung, H Chung, J Chung, Raymond Chung Siang Lim, Yuliya Churina, C Chwat, Carina Chwat, Fabio Cianchi, Antonio Cianci, P Cianci, Pasquale Cianci, A Cianfarani, D Cianflocca, Desiree Cianflocca, Marco Ciappara Paniagua, FA Ciarleglio, C Ciatti, Corrado Ciatti, Sandro Ciccarello, Pietro Ciccarino, Flavia Ciccarone, R Cicco, Candan Cicek, PM Cicerchia, M Ciciliot, Marta Cicuendez López Ocaña, Luciana Cidade Costa, MP Cidón Palacio, AB Ciftci, Luca Cigagna, N Cillara, Nicola Cillara, Ángel Cilleruelo Ramos, Bonifacio Cimadevilla Calvo, S Cimbanassi, B Cimenoglu, Berk Cimenoglu, Marilaeta Cindryani Lolobali, Charles Cini, Matteo Cinquepalmi, Juan Cintas Catena, A Cioci, Alessia Cioci, SP B Cioffi, SPB Cioffi, E Ciofic, Tommaso Cipolat Mis, Alessandro Cipolli, Federica Cipriani, R Cipriani, Riccardo Cipriani, V Ciriello, B Cirillo, Bruno Cirillo, N Cirocchi, B Cismasiu, Brigitta Cismasiu, G Cisternino, B Citgez, D Citterio, Davide Citterio, C Ciubotaru, Cezar Ciubotaru, Agne Cizauskaite, H Claireaux, Cillian Clancy, H Clancy, Maria Clara Mendoza Arango, Ana Clara Valerio, Olga Claramonte Bellmunt, Guillem Claret, G Clarizia, Guglielmo Clarizia, J Clark, Julie Clark, Mhairi Clark, Sara Clark, Simon Clark, Eleanore Clark-Mackay, Eleanor Clarke, EM Clarke, Gareth Clarke, Theo Clarke, M Claro, Mariana Claro, Sharnel Clatworthy, Marie Claude Renaud, Ana Cláudia Deus, Muhawenimana Claudine, Oliver Claydon, Sean Cleary, Joe-Nat Clegg-Lamptey, G Clemen, Quentin Clemens, M Clementi, Laura Clementoni, P Clermidi, Pauline Clermidi, R Clifford, Zoe Clifford, Paballo Clinton Khaeane, Octavian Clonda, E Clough, Ethan Clough, Jonathan Cloutier, AL Clynch, Daniel Coakley, Amy Coates, C Cobelschi, L Cobianchi, Lorenzo Cobianchi, M Coburn, Mark Coburn, L Cocchi, F Coccolini, Federico Coccolini, Angela Cochrane, Elliott Cochrane, AJ Cockbain, E Cocozza, Eugenio Cocozza, Clara Codony, Peter Coe, JC Coffey, Daniel Cohen, Oliver Cohen, Olivia Cohen, David Cohn, T Cohnert, Tina Cohnert, TU Cohnert, FJ F Coimbra, Saverio Coiro, JM Cojulun, Jose Cojulun, JM Cojulun Barrera, A Cokan, Andrej Cokan, Sara Cokarić, N Cokleska Shuntov, Natalija Cokleska Shuntov, Ashley Colaco, E Colak, Eli̇f Çolak, MK Colakoglu, Simone Colangeli, L Colao García, Laura Colao García, Pierre-Antoine Colas, E Colás-Ruiz, Enrique Colás-Ruiz, Marco Colasanti, Roberto Colasanti, J Coleman, Julia Coleman, NL Coleman, Laura Colet Oliver, Katerine Colina, J Colina Casas, M Colino, JA Collantes Cubas, B Collard, M Colledan, P Collera, Pablo Collera, G Colletti, Gaia Colletti, Tom Collicott, Amber Collier, T Collier, KP Colling, Kristin Colling, CG Collins, Chris Collins, Emma Collins, Michelle L Collins, ML Collins, Rachael Collins, J Collis, F Colombo, Francesco Colombo, Giovanni Colombo, Anthony Colon, E Colonna, Emily Colonna, ET Colonna, A Colquhoun, Pablo Colsa, N Colucci, CA Colunga Tinajero, Gary Colville, HV Colvin, T Combellack, Tom Combellack, LV Comini, Amanda Compadre, B Compagnoni, Bruno Compagnoni, MDP Concejo Cutoli, María concepcion Alonso González, V Concepción Martín, S Conci, Simone Conci, Odilia Conde, Danny Conde Monroy, M Condon, Melissa Condon, Sarah Condron, Ana Conesa, Anna Conesa, M Confalonieri, K Conlon, Tara Connelly, TM Connelly, E Connolly, H Connolly, Michael Connolly, Patricia Conroy, Soraya Conroy, Shane Considine, C Conso, Christel Conso, E Consorti, G Consorti, Giuseppe Consorti, J Constable, Houlzé-Laroye Constance, Raquel Constantino-duarte, Monica Contador, Alfredo Conti, L Conti, Lorenzo Conti, Luigi Conti, Ioannis Contis, Elisa Contreras Saiz, Luca Contu, I Conversano, P Cook, Fiachra Cooke, Paul Cool, E Coomber, A Coonar, Aman Coonar, M Cooper, S Cooper, Z Cooper, Zara Cooper, D Cope, Daron Cope, Jennifer Cope, Chiara Copelli, D Copur, Francesco Coratti, C Corbellini, Carlo Corbellini, Harriet Corbett, HJ Corbett, Lisa Corbiere, Sasha Corbin, Francesco Corcione, Marta Córcoles, F Cordera, Fernando Cordera, Saray Cordero Spencer, Timothy Cordingley, A Cordonnier, A Cordova, Adriana Cordova, Diego Córdova García, E Cordova-Calle, Fernando Corella, Guido Coretti, Joel Corkill, Valeria Cormane Alfaro, Tommaso Cornali, DK Cornelio, Philip Cornford, J Cornish, C Cornwell, D Corona, Jose Corona-Cruz, M Coronas Soucheiron, Maria Coronas Soucheiron, Alma-Andreea Corpodean, Paula Corr, Eva Corral Rubio, A Correa Bonito, Alba Correa Bonito, AM Correia, Bernardo Correia, J Correia, R Correia, Rebeca Correia, RM Correia, S Correia, Igor Correia de Farias, Tiago Correia de Sá, T Correia-de-Sá, M Corrigan, Mark Corrigan, Patricia Corriols Noval, Ramon Corripio-Sanchez, Simon Corriveau-Durand, Julian Corso, Henry Cortes, Ludivina Cortes, Ruben Cortes, Susana Cortes, D Cortés Guiral, Natalia Cortes Murgueitio, D Cortés-Guiral, Delia Cortés-Guiral, C Cortes-Mora, Edgar-Joaquin Cortes-Torres, Sara Cortinovis, Umberto Cortinovis, Jordan Cory, M Cosimelli, Maurizio Cosimelli, R Cosker, M Coşkun, Codrut Cosmin Nistor-Ciurba, Francesco Costa, Francisca Costa, Laura Costa, M Costa, MJ M A Costa, Tainá Costa, Brena Costa dos Santos, Fernanda Costa Pereira, D Costa Santos, Daniel Costa Santos, Rui Costa Soares, Andreea Costache, Victor Costache, S Costantini, Andrea Costantino, Caterina Costanza Zingaretti, A Costanzi, Andrea Costanzi, F Costanzo, A Costas-Chavarri, Ainhoa Costas-Chavarri, Simbad Costas-Ochoa, P Coste Mazeau, R Costea, Radu Costea, B Costeira, Beatriz Costeira, Cristina Costeira, Rad Costel Claudiu, O Costerousse, Renato Costi, Mathieu Cote, Maxime Cote, G Côté, Mathilde Côté, A Cotoia, Antonella Cotoia, C Cotsoglou, Estefanía Cotta, E Cotte, Tatiana Cottin, O Cottle, Pietrina Cottu, J Couch, P Coughlin, Patrick Coughlin, R Coulson, Valérie Courval, Ianis Cousin, Mariana Couto Bártolo, E Couture, Brendon Coventry, Sarah Cowan, Aram Cox, Daniel Cox, DR A Cox, G Cox, India Cox, Shanice Cox, Joanne Cozens, V Cozza, Valerio Cozza, Federico Cozzani, Emily Crane, M Crank, A Craus-Miguel, Andrea Craus-Miguel, Joanna Craven, J Crawford, Emily Crawley, Simon Craxford, M Creanga, B Creavin, Ben Creavin, Diana Crego Vita, D Crego-Vita, Camilla Cremonini, V Crenn, Vincent Crenn, Giacomo Crescentini, R Cresner, MS Crespi Amor, Ant`Onia Crespí Mir, A Crespo, Aldo Crespo, J Crespo, Jesús Crespo-Sanjuán, C Crétolle, Célia Crétolle, Benjamin Cribb, Mark Cribb, A Crichton, R Crichton, Peter Cripps, Mambote Crispin Olivier Ntoto, Alessandra Cristaudi, D Cristian, Joana Cristina Domingues, Claudia Cristina Lopes Moreira, María Cristina Martínez Canto, Aida Cristina Rahy-Martín, J Cristini, Ignacio Cristobal, Lodovica Cristofani Mencacci, MG Cristofaro, Rebecca Critchley, Sergiu Crivat, Bojana Crnobrnja, B Crnokrak, RS Croattini, Daniele Crocetti, SM Croghan, Stefanie M Croghan, Gheorghe Croitor, Cristina Croitoru, A Cromi, Rachael Crompton, P Cromwell, Lauren Crone, R Croner, Roland Croner, B Cros, Beatriz Cros, GW V Cross, Katie Cross, Rebecca Crothers, Clare Crowley, R Crowley, AS Crugnale, EM Cruz, Joao Cruz, L Cruz, A Cruz Cidoncha, Maria Cruz Iglesias Moreno, Octavio Cruz-Pineda, A Cuadrado-Garcia, Angel Cuadrado-García, María Cuaresma, Aldrin Cuasay, Daniela Cubek, Eugenio Cucinotta, Ciprian Cucoreanu, Osmar Cuenca, Miguel Cuende Diez, M Cuesta Argos, Mario Cuesta Argos, FJ Cuesta-González, Francisco J Cuesta-González, MA C Cueto, AE Cueto Valadez, TA Cueto Valadez, Esteban Cueva-Martinez, Ainhoa Cuevas, Gabriella Cuevas Lantigua, I Cujiño, Indira Cujiño, V Cuk, Vladica Cuk, M Cukier, Moises Cukier, Serdar Culcu, Ivana Čuljak Blagojević, Ivor Cullen, James Cullen, C Cullinane, Carolyn Cullinane, P Cullis, Paul Cullis, O Cullivan, SL Cumine, T Cuming, A Cumpstey, Wang Cunchuan, C Cunha, MF Cunha, Miguel Cunha, Miguel F Cunha, A Cunha Viana Júnior, RM Cunningham, Y Cunningham, T Curl-Roper, G Currò, C Currow, Chelise Currow, T Curry, Terry Curry, C Cursiefen, Claus Cursiefen, Alexa Curtis, M Curtis, Nathan Curtis, Carolina Curtis Martínez, C Curtis-Martínez, JM Curvas, J Cuschieri, R Cuthbert, Rory Cuthbert, C Cutolo, V Cvetanovska Naunova, A Cvetkovic, Ana Cvetkovic, W Cymes, L Czako, Ladislav Czako, Louie Czelline De Leon, W Czerniak, Viktoria Czok, Amanda Czyz, F D’acapito, Alonço da Cunha Viana Júnior, D Da Luz, AM R da Silva, C Da Silva, Cassia da Silva, T Da Silva, R Da Silva Freitas, L Da Silveira Botacin, A Daadipour, Dennis Daary, Ezeddin Dabbagh, Payman Dabirmoghaddam, John Dabis, Latif Daboo Salifu, F Dacapito, Fabrizio Dacapito, Niccolo Daddi, JL DAddino, Shailja Dadhich, Dora Dadoe, Nikos Dafnios, Hattan Dagestani, Yaquob Daghriri, T Dagklis, Themistoklis Dagklis, M DAgruma, Lara Daham, Hiba Dahhan, Abdifatah Dahir Ali, Nick Dai, E Dainius, Edvinas Dainius, K Dajani, Khaled Dajani, I Dajti, Irida Dajti, Elorm Daketsey, S Dakpé, Stéphanie Dakpé, Anuj Dalal, R Dalal, Mohammed Dalaleh, Tyson Dale, Valentina Dalessandro, Rossella DAlessio, B Daley, Brian Daley, S Dalgleish, Stephen Dalgleish, Lucrezia DAlimonte, P Daliya, Iacopo Dallan, Matias Dallaserra, Khadija Dalmar, E Dalmasso, Giordana DAloisio, Francesca Dalprà, A Daly, Catriona Daly, Michael Damah, FA Damara, PD Damasceno, R Damaseviciute, Manuel Damasio Cotovio, Z Dambrauskas, Zilvinas Dambrauskas, Giancarlo Dambrosio, Nurullah Damburacı, R Damian, Maria Damico, Ana Damjanović, B Danaei, Nyirasebura Dancilla, G DAndrea, Giancarlo DAndrea, Marcello DAndrea, Vito Dandrea, C Dandurand, Charlotte Dandurand, Piergiorgio Danelli, G DAngelo, M Danguy des Déserts, Marc Danguy des Déserts, Aghyad Danial, Syed Danial Syed Ahmad, Nikolaos Danias, A Danic Hadzibegovic, Ana Danic Hadzibegovic, D Daniel, James Daniel, Mario Daniel, Toman Daniel, Carlos Daniel Beyrne, Esteban Daniel Mendoza Galván, Ron Daniel Rivera, karla Daniela Pérez, A Daniele, Alberto Daniele, IR Daniels, Emeka Danielson Odai, Daniel Danielsson, Muhammad Daniyal Daniyal, Muhammad Daniyan, Simone DAnnunzio, Mark Danton, Israel Daodu, Muhammad Daood Daood, Mohamed Daoub, Hasan Daoud, Hassan Daoud, Maher Daoud, Reda Daoud, A Daponte, Alexandros Daponte, Jih Dar Yau, F DAragon, J Daramola, Rhea Darbari Kaul, Sabatino DArchi, D Dardanov, Dragomir Dardanov, Vincenzo Dario Mandato, George Darko Brown, Regina Darko- Asante, Lynn Darragh, H Darraj, J Daruwalla, Jurstine Daruwalla, Amr Darwesh, Abdelrhman KZ Darwish, Sara Darwish, Deepu Daryanani, Andre Das, Devishmita Das, Gurudip Das, Mautushi Das, Nivedita Das, R Das, Rishi Das, Shefali Das, Sunit Das, M Dasa, L Dasanayake, Lanka Dasanayake, Mustafa Dashti, Matheus Dasqueve, D Dass, Debashis Dass, Silvio Däster, Avinash Date, Philip Datler, Debajyoti Datta, Rupen Dattani, Mashal Daud, E Dauer, A Dauksa, Albertas Dauksa, Z Dauksa, Giuliana DAulerio, Marta DAuria, Raphaël Dautry, D Davenport, MG Davey, Andras David, Avril David, Bryony David, Martínez David, PZ David, Rotimi David, Shakina David, Alejandro David Bueno Cañones, Godwin David C Mathew, Mamun David Dornseifer, Cesar David Galindo Regino, Parvez David Haque, Jose David Jimenez Parra, Juan David Lalinde, Edward David Lumley, Victor David Olave Montaño, Brayan David Pedroso Alvarenga, José David Pérez Cajti, Juan David Rivera Garcia, Juan David Saavedra Henao, Ever David Sosa Ferreira, Jorge David Vera Florentin, José Davide, M Davidescu, Mihnea Davidescu, S Davidesko, Lazar Davidovic, B Davidson, Brian Davidson, GH Davidson, A Davies, Angharad Davies, Anna Davies, B Davies, Camilla Davies, E Davies, Elinor Davies, GS Davies, James Davies, M Davies, Mark Davies, Peter Davies, RJ Davies, G Davies-Jones, Gareth Davies-Jones, Amelia Davis, NF Davis, Niall Davis, Sean Davis, Timothy Davis, S Davison, Stephen Davison, Anthony Davor, Kaveh Davoudi, Abdulla Dawaishan, Baheya Dawaishan, N Dawe, Nicholas Dawe, Rsheeda Dawelbait, Lujain Dawod, Omar Dawod, Oseyi Dawodu, Christopher Dawoud, AC Dawson, Hannah Dawson, Jonathon Dawson, Joseph Dawson, Pamela Dawson, S Dawson-Bowling, B Dawud, Bashar Dawud, A Day, Arthur Day, Davut Dayan, Julian Daza, Rachel Dbeis, S Dbouk, Samer Dbouk, S DCruz, U De Andres Olabarria, B De Andrés-Asenjo, Beatriz De Andrés-Asenjo, Itziar De Ariño, Alvaro de Arriba, Mario De Arriba Alonso, Gustavo De Bacco Marangon, J De Barros, R De Berardinis, Rita De Berardinis, Henry de Berker, Karina De Bleecker, Yasmine De Bruyne, J De Ceulaer, JN De Chavez, FL De Cicco, Michael de Cillia, J De Coster, E De Crescenzo, Eugenia De Crescenzo, Carlotta De Cristofaro, Francesco de Falco, Francesca De Felice, M De Francesco, A De Gea Rico, L De Geer, Lina De Geer, C De Gheldere, Nine de Graaf, MR De Graaff, Pauleen de Grano, J De Haro, Joaquin De Haro, I De Haro Jorge, Irene de Haro Jorge, T De Hoop, Nicolas De Hous, P De Iaco, Pierandrea De Iaco, Luis De jesus, Milton de Jesus, José de Jesús Cárdenas Barón, José De Jesus Casco Samudio, Gregorio de Jesus Labrador Hernandez, M De Kock, Marcel de Kock, Michèle de Kok, S De la Cruz Ahufinger, MF I De La Cruz Monroy, Paloma de la Dehesa Cueto-Felgueroso, Angela de la Hoz, A De la Hoz Rodriguez, Anabel De la Llave Serralvo, R De la Oliva, Jairo De la Peña, MA De la Rosa Abaroa, M De la Rosa-Estadella, Marta de la Rosa-Estadella, Javier de la Torre, Olga De la Varga-Martínez, FB de Lacy, Francis De Leon, Murilo de Lima Brazan, Marcello De Luca, Francesca De Lucia, Nicolò de Manzini, A De Manzoni Garberini, Andrea de Manzoni Garberini, J De Marchi, Paolo De Martini, Jorge De Medeiros, Matheus de Melo Lôbo, Federico De Michele, MD C De Miguel-Ardevines, P De Nardi, Paola De Nardi, C De Nunzio, Cosimo De Nunzio, AL De Oliveira Lopez, Alba de Pablo García-Cuenca, Alessandra De Palma, GD De Palma, Jojiemar De Pano, Gilda De Paola, M De Pastena, C De Ponthaud, H De Praetere, Marco De Prizio, PR De Reuver, Giacomo De Riu, S De Robles, Katherine de Rome, Raffaele De Rosa, Silvia De Santi, Chathuranka De Silva, Kanishka De silva, R De Silva, T De Silva, B De Simone, Belinda De Simone, V De Simone, Veronica De Simone, G De Smul, Anabela de Sousa Salgueiro Oliveira, A De Souza, G De Toma, Giorgio De Toma, S De Vergie, Stéphane de Vergie, R De Vincenti, Rosita De Vincenti, A De Virgilio, Armando De Virgilio, Carla De Vita, Jean-Paul PM De Vries, JP P M De Vries, TS de Vries Reilingh, Stefan De Wachter, Marco De Zuanni, Christopher Deacon, Laura Deacon, B Dean, Benjamin Dean, H Dean, Mainak Deb, G Debele, Ashe DeBiasio, Samuel Debrah, Laila Debri, C Decker, Cassie Decker, Georges Decker, B Decruze, Kristof Dede, Georgia Dedemadi, Florence Dedey, Natalija Dediulia, Haya Deeb, S Defaee, C Defert, Coralie Defert, D Degarege, Eyueal Degefa, Hailegebriel Degefu, S Degener, Stephan Degener, I Deglurkar, Yoshihiko Deguchi, A Dehal, D Dehart, Dustin Dehart, Enrica Deiana, Krisztian Deierl, A Deirino, D Deisher, DO Dejana, T Dejanovic, Tatjana Dejanovic, Inga Dekeryte, NA M Dekker, Nicole Dekker, Mathilde Del, Celeste Del Basso, María Del Campo Lavilla, Marco Del Chiaro, Massimo Del Gaudio, R Del Giudice, Roberto Del Giudice, Maria del Mar Martí-Ejarque, Maria del Mar Soriano, Maria Del Pilar Concejo Cutoli, Arazzelly Del Pilar Paucar Urbina, Eneko Del Pozo Andres, Paolo Del Rio, MD del Toro Lopez, T Del Toro Simoni, Czarlo Dela Victoria, Christopher Delaney, KS Delank, S Delazar, A Delegido García, Ana Delegido García, Daniel Delfau Lafuente, Roque Delfino Licona-Meníndez, E Delgado Blanco, Elena Delgado Blanco, L Delgado Búrdalo, J Delgado Fernandez, Juan Delgado Fernandez, Malik Delhen, Samir Delibegovic, D Deligiannidis, C Delimpalta, Demre Delipinar, Olga Delisau-Puig, A Dell, Angela Dell, JS Della Fontana, AN Della Gatta, Carlo Della Rocca, F DellAglio, D Dellaportas, Dionysios Dellaportas, I DellAtti, J Dellonder Frigolé, Cristina DellOro, Mathilde Delorme, Marion Delpont, Paolo Delrio, M Delsoz, D Deluca kobelanski, Suzanne Demers, Z Demetrashvili, Zaza Demetrashvili, Andreas Demetriades, Andreas K Demetriades, C Demetriou, Charis Demetriou, Jevgenijs Demicevs, H Demir, Hakan Demir, İ Demir, Anil Demi̇r, Bengi Demirayak, Gokhan Demirayak, Sibel Demirel, Serdar Demirgan, R Demirhan, Recep Demirhan, F Demirkiran, Fuat Demirkıran, S Demirli Atici, Semra Demirli Atıcı, JA Demma, Daniel Dempsey, Khaled Demyati, S Demyttenaere, FC den Boer, Frank den Boer, Vincenzo Denaro, Christine Denet, Stuart Denham, Franck Denimal, W Denis, Waast Denis, A Denning, M Denning, B Dennis, Grace Dennis, Jonathan Dennis, Barry Dent, H Dent, Rahul Deo Sharma, N Depalma, Norma Depalma, M Deplano, Yadani Deressa, Tilahun Deresse, Habtamu Derilo, HT Derilo, Aleksandr Derinov, Tyche Derksen, Aleksandar Đermanović, Cedric Dery, Malcolm Dery, A Desai, Anant Desai, C Desai, Nimai Desai, NR Desai, Dawit Desalegn, M Desalegn, Christine Desbiens, S Desbruslais, L Desender, Amit Deshmukh, Anuja Deshmukh, Ajinkya Deshpande, Aniket Deshpande, Anvay Deshpande, Charulata Deshpande, Mandar Deshpande, S Deshpande, M Desio, Matteo Desio, Eirini Deskou, Ashwin Desoouza, J Desroches, Francesco Dessole, S Dessole, Salvatore Dessole, S Dester, Salvatora Dettori, AC Deus, Preetham Dev, Eliya Devan, Sreekar Devarakonda, B Devauchelle, Bernard Devauchelle, V Devezas, Vítor Devezas, Grigol Devidze, Michael Dewan, Nikhil Dewan, Tanushree Dewan, V Dewan, Varun Dewan, Hubert Dewanon, Jayan Dewantha Jayasinghe, Ffion Dewi, Madlen Dewi, Swarnendu Dey, Jamie Deyell, Daniel Deziel, K Dhaduk, Ahmed Dhaif, Fatema Dhaif, Bharat Dhanani, D Dhanani, A Dhannoon, Amenah Dhannoon, DS Dhar, Sanjay Dhar, Tapasya Dhar, Satish Dharap, Ashni Dharia, Dharminder Dhillon, Govind Dhillon, Haradeen Dhillon, Mohit Dhingra, B Dhinsa, Rishi Dhir, Harshadkumar Dhirajlal Rajgor, B Dhondt, Bert Dhondt, Slsabela Dhoon, Zainab Dhorat, Hailu Dhufera, Parag Dhumane, Ritika Dhurwe, Matteo Di Bari, Mattia Di Bartolomeo, A Di Bella, Annamaria Di Bella, F Di Candido, F Di Chiara, Nadine Di Donato, FM Di Flamminio, G Di Franco, Gregorio Di Franco, D Di Giorgio, Lorena Di Girolami, M Di Giuseppe, Marta Di Grezia, P Di Lascio, F Di Lella, Sofia Di Lorenzo, Pasquale Di Maio, S Di Maria Grimaldi, C Di Martino, M Di Martino, Marcello Di Martino, Francesco Di Marzo, D Di Miceli, Dario Di Miceli, M Di Muro, S Di Saverio, Salomone Di Saverio, Alberto Di Somma, G Di Taranto, Nora Di Tomasso, Ettorino Di Tommaso, Marcela Di Vincenzo, M Diab, Yasser Diab, B Diaconescu, Bogdan Diaconescu, A Diamantis, Alexandros Diamantis, A Dias, Andre Dias, André Dias, Beatriz Dias, David Dias, Nuno Dias, Richard Dias, Vanessa Dias, A Dias Samarawickrama Yapa, Consuelo Diaz, J Diaz, Jose Diaz, K Diaz, Raquel Diaz, S Diaz, Sandra Diaz, C Díaz, Gabriela Díaz, Pedro Díaz, R Diaz Del Gobbo, G Diaz Duarte, A Díaz García, Alberto Díaz García, Eneida Diaz Martinez, Alba Diaz Padillo, P Díaz Peña, Patricia Díaz Peña, D Díaz Pérez, David Díaz Pérez, R Diaz Serrano, Gonzalo Díaz Tapia, Tamara Díaz Vico, Fernando Diaz-Couselo, PJ Diaz-Delgado, Berta Díaz-Feijoo, R Diaz-Ruiz, Aubrey Dickason, Edward Dickson, K Dickson, Kathryn Dickson, M Diczbalis, Marcel Didier Ndayishyigikiye, H Didriksson, Helen Didriksson, Victor Diego Caballero Sarabia, Lucia Diego García, B Diéguez, Beatriz Diéguez, Maria Dieguez López, T Diehl, Thomas Diehl, Joachim Diessner, MD Diestro, E Díez, M Diez Alonso, Manuel Diez Alonso, Fernando Diez Burón, A Diez-Fraile, ES Dif, Holly Digne-Malcolm, Emilio Dijan, E Dikicier, Enis Dikicier, Merve dilara Öney, Seda Dilek Yetut, Tina Dilevska, Michael DiMaio, A Dimas, DM Dimatatac, Julian Dimech, Papalouka Dimitra, James Dimitri Kane, D Dimitrijevic, Ivan Dimitrijevic, Christos Dimitrios Terzoudis, Nikolaos Dimitrokallis, Dimitrios Dimitroulis, D Dimitrov, Dobromir Dimitrov, M Dimofte, Mihail-Gabriel Dimofte, Danielle Dimsoy, HA Dincer, D Ding, W Ding, Maggie DiNome, Joseph DiNorcia, Ettore Dinoto, Marie Dione Parreno-Sacdalan, Marie Dione Sacdalan, Sandra Dios-Barbeito, Matthew Dipper, Tesfaye Diress, R Dirks, Tariq Diryaq, S Discepola, Karla Disla, C Distefano, M Distler, Marius Distler, Antonino Ditto, Emre Divarci, H Divecha, Pawan Dixit, J Dixon, Lauren Dixon, Toby Dixson, Bahraoui Djahida, Z Djama, Kaveh Djamali, V Djan, Vladimir Djan, K Djebabria, G Djedovic, Gabriel Djedovic, A Djelloul, A Djouani, V Djukic, Vladimir Djukic, M Djuric, Igor Djurisic, Christopher Dobbins, A Dobrescu, S Dodd, Sophie Dodd, M Doe, J Doerner, Johannes Doerner, NU Dogan, Bayram Doğan, Keziban Doğan, L Doğan, Lütfi Doğan, S Doğan, Selen Doğan, Selim Doğan, M Doğangün, Francesco Doglietto, Anthony Dohan, Sasho Dohcev, Ciaran Doherty, Daniel Doherty, Laura Doherty, Charalampos Doitsidis, K Doklestic, Kingsley Doku, Rachael Dolan, Giampiero Dolci, Brett Doleman, María Dolores Arribas Del Amo, Maria Dolores Burgueño, Maria Dolores Mateo Arzo, R Domagalski, Maurizio Domanin, J Domenech, Julio Domenech, J Domenech Fernández, M Domenichini, Marco Domenichini, L Domenici, Giovanni Domenico De Palma, Efren Domingo, EJ Domingo, María Domingo, Carlos Domingo Del Pozo, JC Domingues, Marisa Domingues Santos, CM Dominguez, V Domínguez-Prieto, Ismael Dominguez-Rosado, C Domröse, Christian Domröse, Jimena Dona, Hans Donald de Boer, Danilo Donati, G Dondi, Giulia Dondi, Niren Dongre, Urszula Donigiewicz, MA Doniquian, Marcelo Doniquian, Francisco Donis, Henrique Donizetti Bianchi Florindo, N Donlon, Noel Donlon, Lauren Donnelly, GF DOnofrio, Christopher Donoghue, Emma Donohoe, C Donohue, C Donoudis, Richard Donovan, Maebh Doohan, A Doorgakant, A Dorafshar, Amir Dorafshar, Amy Doran, C Doria, Carlo Doria, Ander Dorken Gallastegi, A Dorken-Gallastegi, Isabella Dornauer, Vitaly Dorofeev, Panagiotis Dorovinis, Emmet Dorrian, Isabela dos Anjos, L Dos Santos Carregal, Jorge dos Santos Silva, Anirudha Doshi, Neel Doshi, Alexios Dosis, Michal Dosoudil, Markus Doss, Francis Dossou, A Dostbil, Ivan Dot Pascuet, C Dott, Cameron Dott, Sarah K Dotters-Katz, Zain Douba, C Doudakmanis, B Doughty, T Doulias, A Doussot, Alexandre Doussot, G Dovell, Andraz Dovnik, Joseph Dowdall, Elizabeth Doxford-Hook, Alex Doyle, Joseph Doyle, V Dragisic, Vedran Dragisic, Antonella Dragotto, A Drahman, P Drakakis, Anna Drake, Frederick Drake, FT Drake, T Drake, Thomas Drake, Thomas D Drake, A Drane, Andrew Drane, R Drasovean, Radu Drasovean, Madhulika Dravid, Walter Dreak Erabu, F Dreier, William Drew, Davide Drigo, S Driouich, L Driul, Matteo Droghetti, E Drozdov, Evgeniy Drozdov, R Dru, Vincent Drubay, Isabella Drummond, Katharine Drummond, JP Druta, Joe Drybrough, Carl DSouza, D DSouza, J DSouza, H Du Preez, Therese Du Preez, Mohammed Duah Issahalq, Mireia Duart, Dayhana Duarte, Geraldo Duarte, Handsome Dube, M Dube, Manas Dube, Ngqabutho Dube, SK Dube, Vivek Dubey, DD Dubinski, E Duchalais, Emilie Duchalais, N Duchateau, S Ducic, Sinisa Ducic, Stefan Ducic, E Duck, Abigail Duckett, Thomas Dudding, J Dudek, Nagendra Dudi-Venkata, NN Dudi-Venkata, S Duff, Caoimhe Duffy, Ana Dugandžić Šimić, Anchal Duggal, L Duggleby, Domenico DUgo, Massimo Dugo, S DUgo, Stefano DUgo, Marc Duinslaeger, Paula Dujovne Lindenbaum, Prabuth dulanjan Weeraddana, Onur Dülgeroğlu, Sébastien Dulisse, David Duller, A Dulskas, Audrius Dulskas, Mihai Dumbrava, S Dumitra, Cătălin Dumitraș, O Dumlu, Christian W Dumpies, G Dunbar, Mariya Dunbobbin, R Duncan, Sharon Duniya, Cheryl Dunkerton, J Dunn, Julie Dunn, D Dunne, Declan Dunne, Henry Dunne, N Dunne, J Dunning, M Dunstan, Leanne Dupley, A Dupont, JP Duprat, M Duque, V Duque Mallén, Victoria Duque Mallén, V Duque-Mallen, Igor Duquesne, AC Dural, I Duran, Oscar Durán Anguiano, M Durán Ballesteros, I Duran Sanchez, II Durán Sánchez, VM Durán-Muñoz-Cruzado, Francesc Duran-Valles, Giulia Duranti, Norah Durayb, Andrew Durden, Natalie Duric, M Đurić, Katarina Duric Vukovic, H Durio Yates, Vladimir Durleshter, E Durmuş, A Duro, Agustin Duro, A Durrani, Amer Durrani, Antonio Durso, AZED Durst, Ayberk Dursun, I Dushin, Vincent Dusingizimana, Lambert Dusingizimana Rutayisire, K Dusu, Debnarayan Dutta, Rohini Dutta, B Dvoranova, LS Dvorkin, Y Dwa, Yam Dwa, SE Dwaga, Mays Dweik, Simon Dwerryhouse, O Dwidar, Oliver Dwidar, Adam Dyas, Claudia Dyball, M Dyer, R Dyke, Daniel Dykman, Z Dzamic, Zoran Dzamic, A Dzhanaeva, Khasan Dzhumabaev, Berik Dzhumabekov, Timur Dzhumatov, J Dziakova, Jana Dziakova, Daniel Dzinotyiwei, Mawutor Dzogbefia, Andee Dzulkarnaen Zakaria, Gul e Raana Raana, Yegeremu Eado, N Eardley, Nicola Eardley, Sophie Earl, H Earley, Helen Earley, Hannah Earnshaw, Asha Eastmond, Samuel Ebbs, John Ebenezer, H Eberbach, Helge Eberbach, Georgina Eberle, Mohamed S Ebiad, Fabrice Eboma, A Ebrahim, Abdulla Ebrahim, S Ebrahim, Keramatollah Ebrahimi, JA Echavarría Uceta, Alejandra Echeverri Moreno, G Echeverría-Dávila, T Echim, Timea Echim, S Eckhouse, Panagiota Economopoulou, Salah Eddine Oussama Kacimi, B Eddy, Ekaniyere Edetanlen, Fabian Edinger, Stephen Edino, Ulbar Edinson, Dileepa Ediriweera, Hans Edison Yap, Florian Edlinger, Jennifer Edmondson, R Edmondson, Edwaldo Edner Joviliano, Kemebradikumo Edonkumoh, Maria Eduarda Bellotti Leão, Byron Eduardo Lopez De Mesa Lopez, Ruben Eduardo Morán Galaviz, Carlos Eduardo Otiniano Alvarado, Luis Eduardo Pérez-Sánchez, Raúl Eduardo Pinilla Morales, Blas Eduardo Quintero Sada, Carlos Eduardo Rey Chaves, Alvaro Eduardo Sánchez Hernández, Jorge Eduardo Sisa Acosta, J Edwards, John Edwards, John G Edwards, Tomos Edwards, Amy Edwards Murphy, Andrew Edwards-Bailey, Isaac Edyedu, Arthur Ee, Cho Ee Ng, S Efetov, Sergey Efetov, SK Efetov, Chizoba Efobi, Erik Efrain Sosa Duran, S Efremov, Edgard Efren Lozada Hernandez, Evangelos Efthimiou, Matheos Efthimiou, N Efthymiou, B Egan, Richard Egan, RJ Egan, E Egbor, Esezobor Egbor, IK Egbuchulem, John Egbuji, A Egdeer, Eva Egger, A Egglestone, Peter Egharevba, N Egoroff, Natasha Egoroff, Tamás Egyed, N Eibinger, Klaus Eichhorn, A Eid, Abdulrahman Eid, Ahmad Eid, J Eid, Raja Eid, Taher Eid, C Eiriz Fernandez, Gustavo Eisenberg, Vitalijus Eismontas, Abdullah Eissa, Eiji Eiwa, T Ejajo, Ikechukwu Ejiofor, Aruna Ekanayaka, NG Eke, Onyeanunam Ekeke, Sebastian Ekenze, Murat Ekin, Perihan Ekmekçi, S Ekpemo, Ekemini Ekpo, E Ekrami, Elyad Ekrami, Bosom Ekwere, H Ekwunife, B El Ahmadi, Brahim El Ahmadi, Zein el Amir, M El Amrani, Mehdi El amrani, N El Arbi, Hadi El Assaad, A El Azhari, Abdessamad El Azhari, Hind El Azzazi, Yassine El Bouazizi, S El Drubi Vega, Sara El Falaha, L El Fiky, Lobna El Fiky, Khaled El Gazzar, MA El Ghali, A El Ghoneimi, Nancy H El Goweini, M El Hadi, Mohamed EL Hag, Joe El Hage, M El Hechi, Amin El Helw, Abd El Jawad Al Gasi, AES El kady, J El Kafsi, Jihène El Kafsi, M El Kassas, Mohamed El Kassas, Ahmed El Kelany, Rasha El kharashy, Saja El Masaoudi, M El Moheb, Mohamad El Moheb, Sara El Mustapha, Badih El Nakadi, A El Ouahabi, Abdessamad El Ouahabi, Mehdi El Ouazzani, Mustafa El Sheikh, Esraa El Shemy, Mohammed El Sherpiny, Amr El Yamany, H El Youzouri, Mohamed G El-adawy, Magdy S El-Bahnasawy, K El-Boghdadly, Kariem El-Boghdadly, Marwa El-Deeb, Abd El-Fattah Mouhandes, Mohammed El-Hag-Aly, S El-Hasani, A El-Hussuna, Alaa El-Hussuna, M El-Kassas, Hanna El-Khoury, O El-Koubani, Mustafa El-lami, Y El-Masry, Khaled El-Qawaqzeh, Jazal El-Qudah, Abd El-Rahman Hamed, M El-Rashid, Mohammad El-Sharkawi, Ahmed M El-Sharkawy, Yasin El-Wajeh, Ahmed Elaffandi, Faisal Elagili, Minahil Elahi, Z Elahi, Zain Elahi, Mohamed Elakkad, Faisl Elamin, Mutaz Elamin, H Elamin Ahmed, Ziad Elassar, A Elawad, Amar Elawad, M Elayyan, Abd Elazeem, MA Elbadawy, Seifeldin Elbadawy, M Elbahnasawy, Mohamed Elbahnasawy, W Elbakbak, Osama Elbargathe, Sofian Elbarouni, Ibrahim Elbashir, P Elbe, Peter Elbe, Gehad Elbehairy, A Eldaly, Abdullah Eldaly, Tarik Eldarat, M ElDeeb, Hossam Eldeen Soliman, D Elder, Omnia Eldesouky, Nour Eldin Abosamak, Nagm Eldin Abu Elnga Ahmed, Moaiad Eldin Ahmed, Nour Eldin Nader, Alaa Eldine Elmaghraby, Sami Eldirdiri, Samwal Eldirdiri, OA Elebute, Olumide Elebute, Mahmoud Eleisawy, Peter Elemile, Carmen Elena Badillo Bercebal, Felicia Elena Buruiana, Laura Elena Fernandez Rios, María Elena Muñoz Fernández, Anna Eleonora Gut, Mustafa Elfadli, A Elfallal, Ahmed Elfallal, H Elfeki, Hossam Elfeki, H Elfeky, M ElFiky, Mahmoud ElFiky, Jamael Elfitori, Jane Elford, Alaa Elgaili, Omar ELgamal, F Elgammal, Sarah Elgarf, Ibrahim ElGarhy, A Elgazar, Abdelrahman Elgendy, A Elgenidy, H Elghadban, Omar Elghany, M Elghazal, Shrouk M elghazaly, SM Elghazaly, S Elghiati, Mahmoud Elghoury, A Elhadi, Ahmed Elhadi, M Elhadi, F Elhajdawe, FAD Elhajdawe, Fras Elhajdawe, I Elhalaby, Ismael Elhalaby, AS Elhalawany, A Elhamshary, Rewan Elhawary, Abhay Elhence, Yassein Elhussein, M Elhusseini, Ahmed Elhussiny Salah Mahmoud, Liolis Elias, Bottazzoli Elisa, Maria Elisa Lozano Miralles, Irmgard Elisabeth Kronberger, Sara Elisabetta Dester, Carlo Elises, Nebiyu Eliyas, Sandra Elizabeth Centurion Rolon, Joby Elizabeth Ninan, Hisham Eljack, Mohamed Eljack, H Elkadi, R Elkady, D Elkebir, C Elkettani, Abdulmohimen A Elkhadar, Fatimah Elkhafeefi, H Elkhayat, Hussein Elkhayat, Dina Elkhity, Esraa Elkouba, A Elkoundi, Abdelghafour Elkoundi, Hamed Ellakwa, H Ellauzi, Yosef Ellenbogen, Tressa Ellett, Brodie Elliott, JA Elliott, L Elliott, Michael Elliott, Clayton Ellis, S Ellis, Susan Ellis, Ibrahim Ellojli, Amna Elmabrouk, Khaled Elmaghraby, Walid Elmahdy, Asma Elmahgoub, N Elmaleh, Nabil Elmaleh, Omar Elmandouh, S Elmarimi, Amr Elmeanawy, Ahmed O Elmehrath, Walid Elmoghazy, Randa Elmokhtar, U Elmore, Rami Elmorsi, T Elmoslemany, Tarek Elmoslemany, Nabiha Elmsherghi, Mohammed Elmujtaba, Mohammed Elmujtba Adam Essa Adam, R Elmusa, Reem Elmusa, Hassan Elmusharaf, AlaELDEIN Elnaema, Hatem Elnageh, Ahmad Elnassasra, Sarah Elnems, M Elniel, Mohammed Elniel, S Elnikety, Salma Elnoamany, Mohamed Elobaid, Enilde Eloena Guerra, AM Elosta, Abdulrahman Elrahmany, Mohamedyasin Elrashid, Nasre Elrefai, Alaa Elsabagh, Seif ElSaban, M Elsabbagh, Menan Elsadek, Marwa Elsadig, Mohamed Elsaeed, Said Elsagheer, Ahmed ElSaghir, Reem Elsahti, Mahmoud Elsaid, Alromisaa Elsaka, Mohamed Elsalhy, K ElSanhoury, Kareem ElSanhoury, Abdelrahman Elsawey, Ahmed Elsayed, Hend Elsayed, Hisham M Elsayed, Manal Elsayed, Mostafa Elsayed Elsayed Hewalla, ME Elsayed Hewalla, Shady Elsdfy, Ahmed Elshabrawy, Enas Elshabrawy, Mohamed Elshafey, Ghazi Elshafie, H Elshafie, Mohamed Elshaibi, Seliman ELShakhs, Mohamed Elsharkawy, Ahmed Elshawadfy Sherif, H Elsheikh, Hizabr Elsheikh, Randa Elsheikh, Motaz Elsherbeeny, Ahmed Elsherbini, Hashim Elshibly, Khaled ElSisy, Natasha Elson, Naira Elsoudy, Chase Elswick, Mohammed Eltahier Abdalla Omer, Aymad Eltawab, Almoutaz Eltayeb, M Eltayeb, Momin Eltayeb, Sherif Eltregy, Alejandro Elúa, Sherif Elwatidy, B Elyafawi, Bilal Elyafawi, Mohammed Elzain, M Elzoghby, AE Elzoubi, A Emad Mashhour, Ahmed Emad Sayed Hassan, Eurico Emanuel do Vale Gonçalves de Castro Alves, Carmen Emanuela Scandura, MM Emara, Sherif Emara, H Embarek, Ysabelle Embury-Young, Stanley Emeka Nwabuoku, H Emerson, Hannah Emerson, S Emile, Sameh Emile, Sameh H Emile, Tano Emile, Herica Emilia Félix de Carvalho, María Emilia Muriel, Mahmut emin Çiçek, C Emir Alavi, Cem Emir Guldogan, Luis Emiro Vanegas, M Emiroglu, Mustafa Emiroglu, K Emmanuel, Klaus Emmanuel, Muhawenimana Emmanuel, Oscar Emmanuel Posadas-Trujillo, Benjamin Emmerson, O Emmerson, Basonga Emmy, M Emous, Ayyah Emran, Yunus Emre Aktimur, Murat Emre Reis, K Emslie, Nosakhare Enaruna, Alejandro Encinas Bascones, Alberto Encinas Vicente, O Enciu, Octavian Enciu, C Endara, F Endara, Jana Enderes, Shunji Endo, F Endorf, Frederick Endorf, FW Endorf, Cristian Ene Roata, I Enemosah, Akif Enes Arikan, J Engel, C English, Sefiu Eniola, DT Eniu, D Enjuto, Diego Enjuto, R Ennab, Raed Ennab, Brendan Ennis, Loreno E Enny, E Enoch, Elizabeth Enoch, Frank Enoch Gyamfi, Yutaka Enomoto, Laura Enrica Benedetti, Adolfo Enrique Gómez Ortiz, Carlos Enrique Melo Moreno, Guillermo Enrique Reyes Gamonal, Christian Enrique Soulé Martínez, Joaquim Enseñat Nora, David Ensor, Annika Enste, Enti Enti, Kenneth Enwerem, Sarah Epton, Aritz Equisoain Azcona, Huseyin Eraslan, Aydın Eray Tufan, G Ercan, Gulcin Ercan, C Ercetin, Candas Ercetin, G Ercolani, Giorgio Ercolani, S Erdene, Sarnai Erdene, T Erdil, Hüseyin Erdi̇nç, B Erdle, Emre Erdoğan, S Erel, Reza Erfanian, M Ergenç, E Erginöz, Ergin Erginöz, S Ergun, Sefa Ergün, F Eriberto, Jan Erik Detran, Thomas Erik Wurmb, E Eriksson, Clarissa Ern Hui Fang, A Eroglu, T Erol, S Erridge, O Ersen, Cevper Ersoz, Ş Ersöz, Mar Escales, J Escartin, Jorge Escartin, Alejandro Escobar, D Escobar, Daniel Escobar, Pedro Escobar, Elkin Escorcia, Remberto Escoto, B Escudero, Berta Escudero, Mario I Escudero, MI Escudero, L Escudero-Roque, Anita Eseenam Agbeko, Akaninyene Eseme Ubom, Marlow Esguerra, Fares Eshac, Youssof Eshac, Mahder Eshete, Mabroka Eshnaf, Mohsen Eshraghi, F Eskandari, Ammar Eskander, Antoine Eskander, P Eskander, S Esmaeili Fathabadi, F Esmaeili Tarki, Muhib Esmael, E Esmail, Mohamed Esmat Mohamed, Nereida Esparza Arias, Isaac Esparza Estrada, E Espin-Basany, Eloy Espin-Basany, M Espino Segura-Illa, Carla Espinola, CA Espinosa, J Espinosa, Alfonso Espinosa Ruiz, M Espinosa-Bravo, Martin Espinosa-Bravo, LP Espinoza Padrón, R Espinoza-Llerena, F Espitalier, Hernando Espitia, A Esposito, G Esposito, Giuseppe Esposito, Carlos Esquivel, Mehmet Eşref Ulutaş, M Essa, Murtaza Essajee, Leila Essakalli Hossyni, ME Essalhi, Esraa Essam, Amr Essameldin, Sumayya Essayah Dwaga, Abdalrahem Essied Alzubi Alzoubi, M Estaire Gómez, Mercedes Estaire Gómez, Mohamad Estanbouli, Agustin Esteban, E Esteban Agustí, Enrique Esteban Agustí, Gustavo Esteban Lugo Zamudio, Ricardo Esteban Mentz, O Esteban Sinovas, Nuria Estellés Vidagany, R Esteves Pires, Robinson Esteves Pires, José Estevez Tesouro, Maria Esther Ferreira Aguilera, Mª Esther Valsero Herguedas, Lujan Estigarribia, EE Estrada, B Estraviz, Begoña Estraviz, E Estrella, Emmanuel Estrella, E Etchill, Eric Etchill, Geley Ete, M Etezadpour, Mohammad Etezadpour, Masatoshi Eto, J Etra, Josune Etxabe Gurrutxaga, M Eugene, Muneza Eugene, Ngoga Eugene, Mª Eugenia Marín Martínez, Fabiola Eugenia Michel Campos, Maria eugenia Torguet muñoz, Felice Eugenio Agro, Corinne Eulalie Solo, Narimantas Evaldas Samalavicius, R Evangelista Zamora, Betsy Evans, Daisy Evans, H Evans, J Evans, Jonathan Evans, Jonathan P Evans, R Evans, Rhodri Evans, V Evans, Victoria Evans, Annija Evelīna Berga, Nissi Evelyn, Nissi Evelyn R, C Eveno, Clarisse Eveno, Anokhin Evgeny, Moataz Ewedah, Christina Ewington, Emma Ewins, R Exley, K Eyuboglu, Kayahan Eyuboglu, AC Ezanno, Anne-Cecile Ezanno, M Ezeanochie, Michael Ezeanochie, Manal Ezeddin kamel Sheta, Sean Ezekiel Seow, Constantine Ezeme, Ekene Ezenwa, Francis Ezenwankwo, Grace Ezeoke, U Ezomike, Uchechukwu Ezomike, Yara Ezz, Esraa Ezzat, Mohammed Ezzat Mostafa, Horacio F Mayer, Erich Fabbri, G Fabbri, N Fabbri, Nicolò Fabbri, Cristoforo Fabbris, Robin Faber, Robert Fabian, Carlos Fabián Cárdenas Melgarejo, German Fabian Godoy Perez, Nestor Fabian Pedraza Alonso, Elio Fabio Sánchez Cortés, OM Faboya, Omolara Faboya, N Fabregas, Neus Fabregas, Berta Fabregó Capdevila, Mariana Faccini Teixeira, Dedy Fachrian, H Facundo, Helena Facundo, Ayrton Facundo Valdovinos, Luciana Facure, T Fadalla, M Fadavipour, MG Fadel, AAM Fadhel, H Fadhel, HA Fadlalmola, Saba Fadli, Begoña Fadrique, Alice Fae Ferreira, J Fagan, Paula Fagan, Mohammed Fagihi, K Fagnon, S Fahad, Shoaib Fahad Hussain, Muhammad Fahadullah, Mahmoud Fahd, BA Fahey, Mir Fahiem-ul-Hassan, Ahmed Fahim, M Fahim, Muhammad Fahim Ahsan Ahsan, T Fahlbusch, Tim Fahlbusch, T Fahmawee, MW Fahmi, Mohd Fahmi Abd Aziz, Mohamed Fahmy, Waleed Fahmy, Aini Fahriza Ibrahim, Ahmed Faidh Ramzee, Ahmad Faidzal Othman, Mehmet Faik OZcelık, Sara Faily, Martina Faimali, K Fairhurst, Mohd Fairudz Mohd Miswan, Abdulrahman Faisal Al-Garadi, Muhammad faisal Khan, Muntasir Faisel, Mohamed Faizal Bin Sikkandar, Fouzia Faizi, Ildar Fakhradiyev, Ildar R Fakhradiyev, Seyed fakhreddin Hejazi, M Fakhrolmobasheri, Mohammed Fakhrul-Aldeen, Ghinwa Fakih, R Falah, Areti Falara, Federica Falaschi, M Falcioni, G Falco, Giuseppe Falco, Mónica Falcón Coronado, GM Falcon Pacheco, BV Falconí Noriega, Fabio Falconieri, Sian Falder, Eva Falkensammer, G Fallabrino, Jean-Michel Fallah, Simon Fallis, Deema Fallouh, M Fambrini, Massimiliano Fambrini, P Familiari, Pietro Familiari, Kathleen FM Fan, Sui Fan Tang, Maheriandrianina Fanambinana Voahary Rajaonarivony, A Fancellu, Alessandro Fancellu, E Fandridis, CEH Fang, F Fang, M Fanjul, Antonios Fantakis, Francesca Fappiano, A Faqih, Frederick Far, Ahmed Farag, Mohamed Farag, Ahmed Farag ElKased, Syeda Farah Nazir, Ahmad Faraz, Hala Fares, Imadeddine Farfour, Anthony Farfus, Abeer Farhan, Siti Farhan Moh Pauzi, Waad Farhat, C S Faria, Giles Faria, Mohammed Farid, Ratna Farida Soenarto, Shehla Faridoon, A Faried, S Farik, Shebani Farik, Sofia Farina, Ignacio Fariña, Anna Faris, Amicur Farkas, Andras Farkas, Tallat Farkhanda, R Farnan, F Farnesi, Francesca Farnesi, Francesco Farnia, M Farooq, MS Farooq, Muhammad Farooq, Umer Farooq, Usman Farooq, Muhammad Farooq Afzal, Kamran Farooque, Bakhtawar Farooqui, AM Farouk, Ayman Farouk, O Farouk, Osama Farouk, Barnaby Farquharson, Alex Farr, Faisal Farrash, R Farre Font, R Farrell, T Farrell, Paul Farrelly, Ramon Farres Coll, Cristina Farrés Pla, Anna Farrés Rabanal, Joseph Farrimond, A Farro, Isabelle Farrow, Melanie Farrugia, A Farsi, Ali Farsi, Deema Farsi, Nada Farsi, S Farsi, Sara Farsi, Omer Faruk Ozkan, Anas Fatani, H Fatemi manesh, Hassan Fatemi manesh, MS A T Fathelbab, M Fathi Al Gharyani, Muad fathi khalleefah Abu hallalah, Muhammed Fatih Simsekoglu, Aleeza Fatima, Mishal Fatima, Zareen Fatima, Sara Fatima Faqar-Uz-Zaman, Martha Fatima Irene De La Cruz Monroy, Dahiana Fatima Velazquez, Belabbes Fatima zohra, F Fatouh, Fathallah Fatouh, Adedeji Fatuga, Alastair Faulkner, G Faulkner, Gemma Faulkner, Jordan Faulkner, Ntirenganya Faustin, K Favilla, H Fawi, Ahmad Fawzi, Ahmed Fawzy, M Fawzy, Mohamed Fawzy, A Fayad, EA Fayad, Elsayed A Fayad, MT Fayed, Yahya Fayed, Julie Fayon, S Fayose, Samuel Fayose, Ana Fazenda, M Fazlur Rahman, Marta Fazzin, N Fearon, IS Febriana, S Federer, P Federico, M Fediuk, Melanie Fediuk, Florian Fegg, M Fehervari, D Feingold, E Fekaj, Liane Feldman, Luis Felipe Ávila-Ramírez, Luis Felipe Cabrera Vargas, Alvaro Felipe Guerrero Vergel, Diego Felipe Tellez Beltran, A Fell, Adam Fell, Lucy Fell, Emanuele Felli, S Felmban, S Fendius, Sarah Fendius, Anne-Sophie Fenger, Nathalie Fennell, Joseph Fennelly, Humberto Fenner Lyra Junior, Jibril Fentaw, Mark Fenton, Carlo V Feo, CF Feo, Claudio F Feo, CV Feo, Ksenia Feoktistova, Sina Ferahman, Alessandro Ferdinando Ruffolo, Michael Feretis, Maria Fergadi, D Ferguson, David Ferguson, Douglas Ferguson, H Ferguson, Liam Ferguson, S Ferla, M Fernadez, Luis Fernand Betances, Maria Fernanda Acuna Saravia, María Fernanda Cedeño Bruzual, Maria Fernanda Gonzalez Mosos, Maria Fernanda Mijares Olivo, Maria Fernanda Pedrero Escalas, Djanira Fernandes, PHDS Fernandes, Sara Fernandes, Sarita Fernandes, U Fernandes, V Fernandes, Vânia Fernandes, R Fernandes Rezende, Ricardo Fernandes Rezende, Damaris Fernandez, Jesus Fernandez, Leticia Fernandez, ML Fernandez, Aida Fernández, Esteban Fernández, P Fernández Bernabé, Patricia Fernández Bernabé, Marlin Fernandez Camilo, À Fernández Camuñas, Alba Fernández Candela, Elisabeth Fernandez Castro, L Fernández Gómez Cruzado, Laura Fernández Gómez Cruzado, Maria Fernandez LLorente, J Fernández Manzano, MT Fernández Martín, D Fernández Martínez, Daniel Fernández Martínez, M Fernández Mendez, FJ Fernández Pablos, Laura Fernández Vega, A Fernández-Candela, A Fernandez-Colorado, Anna Fernandez-Colorado, PV Fernández-Fernández, Maria Fernandez-Hevia, AJ Fernández-López, Antonio-José Fernández-López, MR Fernández-Marín, Elena Fernandez-Martin, A Fernandez-Monge, Arantza Fernandez-Monge, María-Carmen Fernández-Moreno, Diego Fernández-Samos Fernández, Paula Fernandez-Valdes-Bango, L Fernandez-Vega, Chamal Fernando, D Fernando, Daniel I Fernando, Diego Fernando Castillo-Cobaleda, Fabio Fernando Eloi Pinto, Carlos Fernando Roman Ortega, Diego Fernando Ruiz Chiriboga, Jorge fernando Tone, John Ferns, Federica Ferracci, A Ferraiolo, Antonella Ferrara, F Ferrara, Francesco Ferrara, Mariantonia Ferrara, Paula Ferrara, F Ferrari, Federico Ferrari, G Ferrari, Giovanni Ferrari, Maurizio Ferrari, L Ferrario, Luca Ferrario, I Ferraz, Inês Ferraz, C Ferreira, Cassandra Ferreira, Cátia Ferreira, Filipa Ferreira, Gonçalo Ferreira, Joaquim Ferreira, Rocio Ferreira, Carlos Ferreira dos Santos, AP Ferreira Pinto, F Ferreli, Fabio Ferreli, M Ferrer Banús, A Ferrer Fuertes, Ada Ferrer Fuertes, Carolina Ferrer Gomez, E Ferrer-Inaebnit, Carlos Ferreras García, J Ferreres Serafini, P Ferrero, S Ferrero, Simone Ferrero, E Ferrero Herrero, Abel Ferrés, CC Ferro, C Ferron, Jenny Ferry, İbrahim Fethi Azamat, Giacomo Fiacchini, V Ficarra, Andreas Fichter, Valeria Fico, Lovely Fidelis, Mark Field, Michael Field, Xavier Field, Drew Fielder, Roberto Fierro-Rizo, Marcelo Figari, Helen Figgins, Blas Figueredo, Jatnna Figueroa, Juan Figueroa, Luis Figueroa, Rodrigo Figueroa, Rafael Figueroa - Casanova, Carlos Figueroa Avendaño, A Figus, Papa Fiifi - Yankson, RF Filarca, RL Filarca, Matteo Filardo, Marco Filauro, Venko Filipce, MD Filipe, D Filipescu, Daniela Filipescu, E Filipov, Emil Filipov, M Filipponi, Eva Filo, JG Finch, Oliver Findl, Alasdair Findlay, L Findlay, Austin Findley, N Fine, Andrea Fink, Marcus Fink, JB Finkelstein, L Finnegan, Michael Finsterwald, M Fiore, Marco Fiore, S Fiorelli, Silvia Fiorelli, Guido Fiorentini, E Fiori, Enrico Fiori, A Fiorini, Alessandro Fiorini, N Firat, Necattin Firat, Mohd Firdauss Osman, Kanwal Firdos, Fatin Firman, Mohammadreza Firouzifar, Anne Fischer, I Fischer, Ines Fischer, Isabel Fischer, B Fish, Brian Fish, A Fisher, Y Fishman, Yuri Fishman, T Fisseha, Tigist Fisseha, Andreea Fisus, Robert Fitridge, Aidyl Fitrisyah, Sophia Fitt, C Fitzgerald, Laura Fitzmaurice, Mohammed Fiyaz Chowdhry, Aleksander Fjeld Haugstvedt, Silvia Flachs Nóbrega, Etienne Flamant, N Flamey, Nicolas Flamey, Michael Flanagan, O Flannery, M Flatman, M Flavin, Julio Flavio Fiore Jr, Jose Flavio Videira, Daniel Fleitas, C Fleming, Christina Fleming, J Fleming, F Fleres, Francesco Fleres, Angelica Fletcher, Alana Flexman, I Flindall, M Flint, Neil Flint, Vojko Flis, Souha Fliss, M Flitcroft, C Flood, J Flor, Delia Florean, Krisniel Florence Solis, Agustina Florencia Castro Lalín, D Flores, Mario-Andrés Flores, Natalia Flores Amador, Paola Flores Becerril, R Flores Clotet, AM Floreskou, Ioan-Alexandru Florian, Ignat Florin, F Floris, G Fluegen, Georg Fluegen, CD Q Flumignan, R Flumignan, RL G Flumignan, Ronald Flumignan, Roland Flurschütz, William Flynn, Philipp Foessleitner, Amy Fogarty, Alessandro Fogliati, Samo Fokter, Eugene Foley, Katarina Foley, Niamh Foley, M Folic, Miljan Folic, S Folli, A Folorunso, Filipa Fonseca, GB Fonsi, M Font, M Fontana, T Fontana, M Fontanella, Marco Fontanella, Jonathan Foo, Chui Foong Ong, Clara Forbes, D Ford, S Ford, Samuel Ford, Jennifer Foreman, Jorge Forero, Alexander Forero-Torres, S Forlani, Martin Formánek, A Fornasari, Anna Fornasari, Alice Fort-Schaale, Beniamino Forte, L Fortuna, Laura Fortuna, Maruel Fortunato, J Foster, L Foster, Paul Foster, P Fotheringham, C Fotopoulou, Christina Fotopoulou, Mohammed Fouad, Mohamed Fouad Elganainy, Daniel Fountain, DM Fountain, Olivier Fouquet, L Fourcade, Laurent Fourcade, M Fourtounas, A Fowler, Alexander Fowler, Amy Fowler, Carolyn Fowler, GE Fowler, Adrian Fox, Wiliam Foy, Sara Fra Fernández, Danniel Frade Said, E Fradelos, Evangelos Fradelos, Gema Fraga, Georgios Fragkoulidis, Henri Fragnaud, Socrates Fragoulis, Uriel Fraidenraij, A Franceschi, Marzia Franceschilli, Gianluca Franceschini, Antonino Francesco Germano, M Franchi, G Francis, Okedi Francis Xaviour, Elaine Francisca De Araújo, Salvador Francisco Campos Campos, José Francisco Farah, Murilo Francisco Fernandes, Antonio Francisco Guisado Calderón, Álvaro Francisco Lopes de Sousa, Luis Francisco Martín Anoro, Juan Francisco Pintado Mejia, Labissi Francois Amossou, E Francone, Elisa Francone, K Frank, Konstantin Frank, A Frankel, Adam Frankel, J Franken, Josephine Franken, K Frankowska, Pietro Fransvea, Helmut Franz Georg Novotny, Albert Franz Guerrero-Becerra, M Franza, Mara Franza, Alina Franzen, M Franzinelli, M Franzini, Marco Franzini, Denis Frasca, M Frascio, S Fraser, Sheila Fraser, S Frassini, M Frasson, Matteo Frasson, A Frati, Alessandro Frati, C Frattaruolo, Colomba Frattaruolo, Antonio Fratto, Wolfgang Fraz, Fabien Fredon, Ebru Freed, J Freedman, Hawys Freeman, P Fregatti, Piero Fregatti, Ali Freihat, M Freijeiro, T Freiman, Thomas Freiman, A Frena, Stefano Fresilli, Claudia Fretes, Martin Fretes, K Fretwell, Matthew Freudmann, MR Freund, C Frew, A Freyrie, Antonio Freyrie, Christian Freyschlag, K Freystaetter, L Frias, Roland Fricker, Danielle Friedman, Sabine Friedrich, Pamela Frigerio, H Frima, F Frio, Filippo Friso, Tommaso Frisoni, A Fritz, Frank Frizelle, S Froghi, Caterina Froiio, A Frolova, Y Frolova, Yulia Frolova, F Frongia, Federica Frongia, A Frontali, Alice Frontali, K Frosch, J Frost, Rhiannon Frostick, Maximos Frountzas, Brytt Frunt, Tiziana Frusca, R Fruscio, Robert Fruscio, Diego Frutos, Fares Ftaieh, Panagiotis Ftikos, H Fu, Ali Fuat Kaan Gok, Hans Fuchs, Thomas Fuchs-Buder, DP Fudulu, L Fuenmayor-González, JR Fuentes, Lucía Fuentes, T Fuentes, Tyare Fuentes, C Fuentes Orozco, Clotilde Fuentes Orozco, Lorena Fuentes Rivera Lau, Alvaro Fuentes-Martín, Reinhold Függer, Mette Fugleberg Nielsen, Ayataka Fujimoto, Y Fujimoto, Yuki Fujimoto, Tomoyuki Fujita, Yoshinori Fujiwara, Yasuyuki Fukami, D Fuks, M Fukuda, Satsuki Fukushima, Christian Fulghum, S Fulginiti, Alamea Fulivai, Mairi Fullarton, Lucy Fuller, U Fumagalli Romario, Uberto Fumagalli Romario, Kentaro Fumoto, N Fundano, Tania Funes, C Fung, Christian Fung, Stephen Fung, Man Fung Ho, Kiu Fung Wong, N Furbetta, Niccolò Furbetta, Hasim Furkan Gullu, Ibrahim Furkan Küçük, Chris Furkert, M Furlan, Micaela Furlan, Yukari Furuhata, Taku Furukawa, Yuri Furukawa, G Fusai, Giuseppe Fusai, Daniele Fusario, Stefano Fusetti, F Fusini, Federico Fusini, A Fuson, Elisa Fustec, K Futaba, Kaori Futaba, Konrad Futyma, O Fuwa, Sotonye Fyneface-Ogan, Franz G Bader, Jorge G Boretto, Nasly G Patino-Jaramillo, Anitha G S, K Gaballa, Khaled Gaballa, Linda Gabellini, M Gaber, Ana Gabersek, A Gabr, Abdullah Gabr, Ayman Gabr, Hesham Gabr, A Gabre-Kidan, Juan Gabriel Castro Ríos, Juan Gabriel De Leon, Leyla Gabriel Fernández, Esteban Gabriel Jauregui, Justin Gabriel Schlager, Mircea Gabriel Stoleriu, José Gabriel Yaryura Montero, Cristina Gabriela Alzate Arsuaga, Claudia Gabriela Mitrofan, Laura Gabriela Peña Balboa, Angelo Gabriele Epifani, Maria Gabriella Dona, Sofia Gabrilovich, H Gacaferi, Hamez Gacaferi, Mahir Gachabayov, Dalia Gad, Corina Gaddi, A Gadducci, Maya Gade, R Gadea Mateo, Ricardo Gadea Mateo, Anup Gadekar, Rabea Gadelkareem, Inas Gadelkarim, Ziyad Gadelrab, P Gadelsyed, Alice Gadotti Yasuda, Taha Gadouali, B Gafsi, A Gagliano, F Gagliardi, Filippo Gagliardi, N Gagné, Joel Gagnier, Gunilla Gagnö, Lara Gahan, Nitesh Gahlot, Yarub Gahtan, S Gahunia, Sukhpreet Gahunia, Milad Gahwagi, F Gaino, Francesca Gaino, A Gainza, Stylianos Gaitanakis, A Gaitanidis, Apostolos Gaitanidis, K Gajjar, Ketankumar Gajjar, Shreyash Gajjar, Urska Gajsek, Delali Gakpetor, T Gala, Tanzeela Gala, K Galaal, Jacopo Galafassi, S Galal Eldin, Ilias Galanis, C Galata, B Galbreath, Zinaida Galchikova, Alessandro Galdini, Mark Galea, Ana Galevska-Dimitrovska, Elisa Galfrascoli, M Galhoum, Mohamed Galhoum, Luis Galindo, Lorena Galindo Iñiguez, P Galindo Jara, Pablo Galindo Jara, Antonio Galindo Nava, M Galipienso Eri, B Gális, Branislav Gális, B Gallagher, K Gallagher, Nicola Gallagher, P Gallagher, T Gallagher, L Gallardo zamora, R Galleano, Raffaele Galleano, Lander Gallego, Diego Gallegos, R Galli, E Galliamov, Eduard Galliamov, A Gallinat, G Gallo, Gaetano Gallo, O Gallo, Oreste Gallo, Daniel Galun, A Galvan, Armando Galvan, Yaiza Galvañ Félix, A Galvan Pérez, Heloisa Galvão do Amaral Campos, M Galvarini, T Gamage, Abrar Gamal, Dina Gamal, Ibrahim Gamal, Mennatullah Gamal, S Gamal Badr, Nehal gamal Omar, Mohamed Gamal Taher, Erick Gamaliel Amba, C Gambacciani, Denise Gambardella, Ahmed Gameel, Hammaad Gamieldien, E Gammeri, Abdulmohimen Gammoudi, SW Gan, Grace Gana, S Gananadha, Thanga Ganapathy, M Ganau, Mario Ganau, Lundeg Ganbold, Sumir Gandhi, Suraj Gandhi, Pablo Gandia Gonzalez, Kugarajh Ganeshapillai, Gagana Ganga, Srinivasan Gangadharan, J Gani, Jonathan Gani, Babak Ganjeifar, I Ganly, Ian Ganly, S Gans, Abel Ganso, A Ganta, Antonia Gantschnigg, Jiali Gao, George Garas, Daniel Garay Lechuga, Stephen Garba, Saudat Garba Habib, E Garca Rico, R Garcés García, Raúl Garcés García, Sofía Garcés Palacios, M Garcés-Albir, A Garcia, Alba Garcia, Andrea Garcia, Cesar Garcia, D Garcia, David Garcia, Felipe Garcia, Francisco Garcia, Iset Garcia, Luis Garcia, Luz Garcia, Manuel Garcia, Sean Garcia, A García, Antonio García, Federico García, J García, M García, Lucia Garcia Alcalde, M Garcia Alonso, Cristina García Amador, C Garcia astrada, LA García Barrionuevo, S Garcia Botella, Juan García Cardo, Carlos Garcia Cardona, Miguel García Castillo, Vázquez García César, Elena Garcia De Castro, E García de Castro Rubio, U Garcia de cortazar, Unai Garcia De Cortazar, Amaia Garcia Dominguez, Antonio García Domínguez, Melody García Domínguez, J Garcia Egea, D García Escudero, Damián García Escudero, LJ García Flórez, JL Garcia galocha, DS García García, Elena García García, JJ Garcia Gutierrez, Saura García Laura, D Garcia López de Goicoechea, V García Milán, JD Garcia Montesino, Mauricio Garcia Mora, Tito García Moreno, G García Operé, Victoria Garcia Peces, D Garcia Perez, Cristina García Pérez, JM García Pérez, Pablo Garcia Pimentel, EP Garcia Santos, Miguel García Sanz, Javier Garcia Septiem, J García Septiem, Vanesa Garcia Soria, S García Valenzuela, O García Villar, JE García Villayzán, Mariana García Virosta, Jorge Garcia-Adamez, David Garcia-Aguilera, L Garcia-Aparicio, Luis García-Aparicio, J Garcia-Borda, Hector Garcia-Chavez, M García-Conde, U Garcia-Dubus Rodriguez, D García-Escudero, Luis Garcia-Florez, Francisco Garcia-Huidobro, E Garcia-Loarte Gomez, Daniel García-López, Belen Garcia-Montesinos Perea, Oscar-Julián García-Montoya, Francisca Garcia-Moreno Nisa, M García-Nebreda, Guillermo García-Operé, Herney Garcia-Perdomo, V Garcia-Pineda, V García-Porcel, J García-Quijada, L Garcia-Sancho Tellez, Luis Garcia-Sancho Tellez, V García-Soria, MA García-Ureña, V Garcia-Virto, Virginia Garcia-Virto, V García-Virto, Jeronimo Garcialopez De Llano, Laura Garden, Padraig Gardiner, A Gardner, E Gardner, Elena Garelli, R Garfinkle, K Garg, Mayank Garg, PK Garg, Rajnish Garg, Surabhi Garg, Vipul Garg, G Garganese, S Garibaldi, M Garino, Mauro Garino, T Garmanova, Tatyana Garmanova, Gregorio Garmendia, M Garner, Madeleine Garner, A Garnero, Z Garoufalia, Zoe Garoufalia, R Garrido, S Garrido, S Garrido-Ondono, Gonzalo Garrigos, Richard Gartrell, Kendyll Gartrelle, G Garulli, S Garusinghe, George Gasana, I Gascon Ferrer, Isabel Gascon Ferrer, I Gascon-Ferrer, Peter Gaskell, Cameron Gaskill, KAD Gasmalla, Anis Gasmi, Giulio Gasparini, Hrvoje Gasparovic, ML Gasparri, J Gass, M Gass, Markus Gass, A Gasset-Teixidor, L Gasteiger, Matt Gaston, A Gasulla-Rodriguez, Anna Gasulla-Rodriguez, VA Gata, Anna Gateley, Lucija Gatin, Juan Gatón, Inbar Gatot, Francesca Gatta, A Gatti, Arthur Gatti, A Gattolin, Andrea Gattolin, S Gattoni, Serena Gattoni, Domenico Gattulli, S Gaujoux, Sebastien Gaujoux, John Gaul, A Gaunt, A Gaurav, Neha Gauri, Claudia Gauto, Julio Gauto, Guilherme Gava, MT Gavaldà Pellicé, Valerie Gaveh, G Gavilanes Loor, Haim Gavriel, TS Gavriliu, Mothana Gawad, Mohan Gawande, Wladyslaw B Gawel, Larsa Gawria, I Gawron, Oswald Gbehade, Olalere Gbolahan, Tjokorda Gde Agung Senapathi, D Gearon, E Geary, M Geary, Michael Geary, A Gebran, Anthony Gebran, Hiwot Gebre, F Gebreegziabher Gebrehiwot, Fitsum Gebreegziabher Gebrehiwot, Gebreagziabher Gebrekirstos, Zersenay Gebremeskel, M Gebreyohanes Mengesha, Mengistu Gebreyohanes Mengesha, Gabriele Gecchele, I Gecim, A Geddes, Yetsedaw Gedefaw, R Gefen, A Gegundez Simon, Alberto Gegúndez Simón, AA Geisler, E Gelarda, Enrico Gelarda, Chiara Gelati, Ryan Geleit, Jaume Gelonch, Scott Gelzinnis, Lidya Gemechu, NA Gemelli, G Gemes, C Gemmell, E Gemmill, Elizabeth Gemmill, J Gempt, Jens Gempt, Eben-ezer Genda, Y Genda, George Gendrikson, Sileshi Genetu, Abraham Genetu Tiruneh, S Gennari, M Gennaro, Massimiliano Gennaro, Ludivine Genre, L Genser, Laurent Genser, Fred Gentili, S Gentilli, Sergio Gentilli, Charles Geoffrey Dermot Stewart, J Geoghegan, Clemens Georg Wiesinger, B George, Gejoe George, K George, R George, Smitha George, Suku George, John George Massoud, P George Pandeth, SA George Varayannoor, Tom Georgi, F Georgiades, Fanourios Georgiades, Gregory Georgiadis, D Georgiadou, D Georgiev, G Georgieva, Gordana Georgieva, Irina Georgieva, Sotirios Georgios Popeskou, Nikolaos Georgopoulos, N Georgopoulou, Kleoniki Georgousi, J Geraghty, David Gerardo Miranda Gómez, C Gerber Gerber, Anna Gergen, F Gerges, A Germain, Alicia German Dihmes, Paola Germani, Christoph-Thomas Germer, Giuliana Germinario, Daniel Gero, I Gerogiannis, SL Gerritsen, A Gerundo, S Gerus, E Gessa, M Gessesse, F Gessler, Florian Gessler, Michael Gessner, Eneyew Getachew Siyoum, Hanna getachew Woldeselassie, Aderaw Getie, Anne Getz, Khaled Ghabban, Raghad Ghadri, S Ghaem-Maghami, M Ghaemi, F Ghaffarizadeh, H Ghaith, Mohamed Ghali, Asma Ghallab, Basmah Ghallab, Amol Ghalme, G Ghaly, Galal Ghaly, Mira Ghaly, Reem Ghamgh, Naji Ghamri, A Ghanbari, K Ghandour, Hossam Ghanem, R Ghanem, Anum Ghani, A Ghannam, Abdelilah Ghannam, R Ghannam, Rana Ghannam, I Gharbi, Khaled Gharbia, Mulham Gharib, Ruwaid Gharib, ABDUlRAOUF Ghariba, H Ghattaura, Sakina Ghauth, Anas Ghawi, Ibrahim Ghayada, Sumayyah Ghayth Bahroun, A Ghazal, Ahmad Ghazal, Muhammad Ghazali Hasheem, Ramlah Ghazanfor Ghazanfor, Mojahid Ghazi, O Ghazouani, Salman Ghazwani, Rahel Ghebre, S Ghedan, Marwane Ghemame, F Ghezzi, SS Ghiasi, F Ghini, Bennis Ghita, Peter Ghiya, M Ghobrial, Marios Ghobrial, R Ghodke, Rahul Ghodke, Mohammad Ghomeisi, Alaa Ghonaim, Mohamed Ghonaim, A Ghoneim, Ahmed Ghoneim, M Ghoneim, Ali Ghorbani Abdehgah, Paloma Ghosal, D Ghosh, Dhruv Ghosh, Indranil Ghosh, K Ghosh, Rukmini Ghosh, A Ghouri, Amna Ghouri, Youssef Ghoussoub, S Ghozy, Sherief Ghozy, K Ghufoor, Bhavisha Ghugare, Muhammad Ghulam Qadir Qadir, Zohal Ghulam-Jelani, Saqib Ghumman, M Ghunaim, Mohammed Ghunaim, M Giacometti, Marco Giacometti, Anna Giacomina Carta, E Gialamas, Eleftherios Gialamas, D Gianardi, Accarino Giancarlo, Maria Giangreco, Alessandro Giani, G Giannaccare, Giuseppe Giannaccare, A Gianni, A Giannini, L Giannini, Lorenzo Giannini, F Giannoulis, T Giannoulopoulos, Uyen Giao Vo, Alessia Giaquinta, M Giardini, Matteo Giardini, Alessandro Giardino, Ludovica Gibelli, A Giblin, Anna-Victoria Giblin, C Gibson, Joanna Gibson, Manuel Gielis, Cecilia Gigena, J Gigliotti, Giacomo Gigliucci, CG Gil, Marta Gil, Lucia Gil Cidoncha, Catarina Gil Gil, J Gil Martínez, Inés Gil Prados, Ismael Gil Romea, PJ Gil Vázquez, O Gil-Albarova, Oscar Gil-Albarova, A Gil-Catalan, Oscar Gil-de-Sagredo, A Gil-Moreno, J Gil-Rodriguez, Daniel Gil-Sala, J Gilabert Estellés, Juan Gilabert-Estellés, C Gilbert, Gasengayire Gilbert, S Gili, Simona Gili, Mireia Gili-Bueno, C Gill, DF Gill, H Gill, Ian Gill, Parmilan Gill, S Gillani, SFM Gillani, Andrew Gillard, Lars Gillberg, C Gillezeau, J Gilliland, Jack Gilliland, AE Gillis, Amy Gillis, Katie Gilmore, G Gilna, Gareth Gilna, Clara Giménez Francés, T Gimenez Maurel, Teresa Gimenez Maurel, C Giménez-Francés, Antonio Gimenez-Gaibar, Mar Gimeno Gimeno Vicente, Jesus Gimeno Hernandez, Cesar Ginesta, Christian Gingert, O Ginghina, Octav Ginghina, Audrey Giocanti-Auregan, R Gioco, Rossella Gioco, V Giordano, Andrea Giorga, E Giorgakis, Emmanouil Giorgakis, Federico A Giorgini, E Giotakis, Evangelos Giotakis, Konstantinos Gioutsos, Jesús Giovanni Inzunza Miranda, Luca Giovanni Locatello, M Giovenzana, D Giovinazzo, F Giovinazzo, Jordi Giralt López de Sagredo, Ricardo Girao, E Girard, Edouard Girard, Ève-Marie Girard, Karine Girard, Noémie Girard, Camila Girardi Fachin, G Giraudo, Giorgio Giraudo, B Giray, Burak Giray, Nathalie Girgis, Adissu Girma, F Giron Luque, Fernando Giron Luque, E Girsowicz, SS Gisbertz, Suzanne Gisbertz, Laura Gisela Alvarez Calzaretta, Laura Giselle Contreras Baquero, Shanthamoorthy Gishanthan, Martin Gisinger, Francis Githae Muriithi, Adam Gittins, Marco Giudice, R Giudici, M Giuffrida, Mario Giuffrida, MC Giuffrida, Bonfanti Giulia, Vitiello Giulia, Maria Giulia Cristofaro, A Giuliani, B Giuliani, Giuliana Giuliani, Tommaso Giuliani, Cesar Giuliano Sisa Segovia, P Giulianotti, Pierluigi Giumelli, Marco Giuseppe Iannuccelli, Elia Giuseppe Lunghi, Marcello Giuseppe Spampinato, A Gjata, Arben Gjata, Ulpjana Gjondedaj, Kostandin Gjyli, Ioanna Gkalonaki, Ioannis Gkekas, Georgios Gkiokas, Antonios Gklavas, Nick Gkolias, E Gkrinia, Eleni Gkrinia, Laurence Glancz, N Glass, Nina Glass, O Glehen, Ana Gleisner, P Glen, Alexa Glencer, I Glisovic Jovanovic, Claudio Glowalla, Tim R Glowka, TR Glowka, Zhanna Glushchenko, Tamara Glyn, Orna Glynn, C Gnanachandran, Subhadra Goala, S Gobishangar, Huseyin Gobut, I Gockel, Ines Gockel, C Godbole, Jerry Godfrey Makama, N Godin, M Godinho, Gaëlle Godiris Petit, André Godoy, Rosana Godoy, J Goedeke, Jan Goedeke, N Goel, Neha Goel, T Goel, MA Goh, N Goh, Farhan Gohar, M Gohar, Muhammad Gohar, Amish Gohil, K Gohil, Raluca Goicea, Amy Gojnich, E Gokcen, N Gokhare Viswanath, B Göksoy, Krishnan Gokul, Mohammadreza Golbakhsh, H Golcher, Ikponmwosa Gold, Ruthie Gold- Deutch, A Goldenberg-Sandau, Anna Goldenberg-Sandau, Carma Goldstein, D Golijanin, Danica Golijanin, S Göller, Can Gollmann-Tepeköylü, Grigoriy Gololobov, A Golomidov, Maher Gomaha, Fabio Gomes, GM A Gomes, GMA Gomes, H Gomes, Alden Gomez, Alejandro Gomez, Dhanwant Gomez, Hugo Gomez, Humberto Gomez, J Gomez, Laura Gomez, P Gomez, Viviana Gomez, Nadia Gómez, Manuel Gomez Cervantes, CJ Gómez Díaz, H Gomez Fernandez, L Gomez Fernandez, Laura Gomez Fernandez, L Gomez Lopez, JR Gómez López, SC Gómez López, B Gómez Pérez, Beatriz Gómez Pérez, J Gomez Rivas, Juan Gómez Rivas, Carlos Gomez Roig, Maria Gómez Romero, Nuria Gomez Romeu, Tania Gómez Sanz, J Gómez Suárez, Paula Gómez Valles, Leticia Gómez Viana, S Gomez-Abril, Segundo Gomez-Abril, Francisco Gómez-Bosch, H Gomez-Fernandez, Hugo Gomez-Fernandez, A Gómez-Pedraza, Antonio Gómez-Pedraza, J Gomez-Rosado, Juan-Carlos Gomez-Rosado, T Gómez-Sanz, Álvaro Gonçalves, BT Gonçalves, JP Gonçalves, N Gonçalves, Rodrigo Gonçalves, Rita Gonçalves Pereira, KM Gondal, Lior Gonen, Bernhard Gonschor, E Gonullu, Emre Gönüllü, AD Gonzales, DS Gonzalez, Elena Gonzalez, Felipe Gonzalez, J Gonzalez, Javier Gonzalez, Judit Gonzalez, Marcos Gonzalez, Nelson Gonzalez, Paloma Gonzalez, R Gonzalez, E González, Enrique González, Gloria González, Natalia Gonzalez Alcolea, FX Gonzalez Argente, Miren Gonzalez Benito, Rebeca Gonzalez Celdran, M Gonzalez De Miguel, Melania González de Miguel, Carlos Gonzalez De Pedro, Daniel Gonzalez Garcia-Cano, JA Gonzalez Lopez, E González Marín, Adelina Gonzalez Martinez, A Gonzalez Ojeda, Alejandro Gonzalez Ojeda, Marta González Pérez, Elena González Revilla, Esteban Gonzalez Salazar, SM González Soares, S Gonzalez Suarez, DS Gonzalez Vazquez, Rocio Gonzalez-Aguado, Carolina Gonzalez-Gomez, E Gonzalez-Gonzalez, Rogelio González-López, MT Gonzalez-Nicolas-Trebol, Alejandro González-Orozco, Susana González-Suárez, FM González-Valverde, Azucena Gonzalo, Paula Gonzálvez Guardiola, S Goodrum, Peter Goodwin, Ferhana Gool, Janindu Goonawardena, Gomathy Gopal, S Gopalswamy, R Gopi Reddy, Praveen Gopinath, Rajesh Gopireddy, Merima Goran, R Goran, Jeremias Goransky, Raghunandan Gorantlu Chowdappa, Sergey Gordeyev, L Gordini, Luca Gordini, Alasdair Gordon, Mangesh Gore, Stefanos Gorgoraptis, A Gori, M Goricar, Matej Goricar, David Gorin, Hugo Gornes, S Gortazar, Sara Gortázar de las Casas, Christina Gory, A Gosain, Ankush Gosain, M Gosau, Martin Gosau, Matthew Goss, Karo Gosselin, M Gosselink, Martijn Gosselink, D Gossot, Yvonne Goßlau, Lucy Gossling, Mark Gotecha, Iltimass Gouazar, A Goubran, Alex Goubran, Omar Gouda, Swati Goudar, Ben Goudsmit, N Gougoulias, C Goumard, Claire Goumard, Lysander Gourbault, Ralph Gourlay, Konstantinos Gousias, Nikolaos Gouvas, Henry Govekar, Arantza Govela Hinojosa, A Govil, Akhil Govil, Mithila Govind, M Gowda, Ravikanth Gowder, Benjamin Gowers, A Goyal, Amit Goyal, Y Gozal, Yaacov Gozal, K Gözal, D Gp, Michael Graber, P Grabowski, Rosalind Grace Beckett, Heather Grace Dulnuan, Harelimana Grace James, Linda Grace Puerto Tamayo, Eileen Grace Tancinco, Charmaine Grace Valeros, Cesar Gracia, I Gracia, Isabel Gracia, M Gracia, C Gracia-Roche, Carlos Gracia-Roche, G Gradinariu, George Gradinariu, Christian Graeb, C Graham, C Grainger, T Grainger, F Grama, Florin Grama, Marco Gramellini, Madelyn Gramlick, V Granata, Michele Grande, Alessandro Grandi, Samuele Grandi, Steven Grandjean, Carmen Grañén, Lucas Granero, S Granieri, C Granja, Cristina Granja, James Grantham, Bianca Grassano, E Grasset, C Grassi, T Grassi, Tommaso Grassi, A Grasso, M Grasso, João Graveto, A Gray, H Gray, Matthew Gray, Maria Grazia Matarazzo, Jacopo Graziosi, M Grechenig, Michael Grechenig, A Grechi, Alessandro Grechi, G Grecinos, Gustavo Grecinos, L Green, S Green, Sofia Green, Jennifer Greenberg, M Greenhalgh, Michael Greenhalgh, MS Greenhalgh, H Greenlee, D Greenman, Dmitry Greenman, Rebecca Greenop, A Gregg, Emilie Gregoire, P Gregoric, Pavle Gregoric, Minja Gregorič, Paulo Gregorio, Gordon Gregory, Kate Gregory, Thomas Gregory, Veronika Greif, Jens Greve, Dilraj Grewal, Rebecca Grey, Maja Grgec Dragicevic, Petar Gribnev, Benjamin Gricks, C Grieco, Christian Grieco, Ryan Griffin, XL Griffin, E Griffiths, Ewen Griffiths, T Griffiths, A Grigonytė, M Grigoroiu, Florian Grill, Richard Grills, JV Grilo, Gabriella Grima, G Grimbizis, C Grimes, E Grimley, C Grimm, Christoph Grimm, A Grimonprez, M Grishenko, A Grivas, T Grivas, A Groen, LC Groen, A Grogan, R Grolman, A Gronchi, Alessandro Gronchi, G Groot, Gary Groot, J Grosek, Jan Grosek, Jefferson Gross, JL Gross, U Grossi, Ugo Grossi, Travis Grotz, Ellen Groundwater, T Grove, Kresimir Grsic, Željko Grubač, Michaela Gruber, R Gruber, Ricarda Gruber, N Grubor, Nikola Grubor, Danica Grujičić, Adrian Grullon, Karlo Grulović, Martin Grünbart, Catherine Grundy, N Grundy, L Grüßer, Linda Grüßer, R Grützmann, Robert Grützmann, I Grypiotis, Ioannis Grypiotis, S Guadagni, M Guaglio, Marcello Guaglio, E Guaitoli, Javier Gualis, M Guardia, N Guàrdia, Luciano Guarienti, CA Guariglia, David Guarin, E Guasch, M Gubbiotti, Marilena Gubbiotti, Ali Guboug, Rita Gudaityte, A Gudal, Vasubabu Gudala, Senyo Gudugbe, HJ Guedes Neto, Noemi Guemes-Villahoz, C Guerci, Claudio Guerci, Sonia Guérin, Bayron Guerra, EE Guerra, Glen R Guerra, Jeffy Guerra, FJ Guerra Brandt, Odoniel Guerra Garcia, E Guerra-Farfan, Ernesto Guerra-Farfan, P Guerreiro, Francesco Guerrera, Fatherin Guerrero, Claudia Guerrero Martinez, A Gueutier, Alexandre Gueutier, J Guevara, O Guevara, Oscar Guevara, R Guevara, Ika Gugić Radojković, L Guglielmetti, Laura Guglielmetti, A Guglielmi, Alfredo Guglielmi, Mario Guglielmo, Nicola Guglielmo, S Guha, S Guicciardi, Gustavo Guida, B Guidi, Gonçalo Guidi, Afrika Guido, Marco Guido Confalonieri, Christopher A Guidry, C Guijarro Moreno, Jeremy Guilford, José Guilherme Gonçalves Nobre, Jose Guilherme Vartanian, Laura Guillamon Vivancos, P Guillamot Ruano, Paloma Guillamot Ruano, Hander Guillermo Acosta Diaz, Andre Guimaraes, Lilian Guimaraes, A Guimarães, André Guimarães, Felipe Guimarães Pugliesi, MA C Guimaraes-Filho, Patrice Guiraudet, John Guirguis, María Guisasola Rabés, Rohan R Gujjuri, Ambrin Gul, Ayaz Gul, Hina Gul, Sana Gul, Brian Gulack, Emre Gülçek, CE Guldogan, Mert Güler, OC Güler, SA Güler, A Gulla, Aiste Gulla, Murat Gultekin, A Gumarao, S Gumede, Yohesuwary Gunarasa, Filip Gunaric, Nalaka Gunawansa, I Gunawardena, Indu Gunawardena, J Gundara, E Gundogdu, EC Gundogdu, Emre Gundogdu, A Guner, Ali Guner, A Gunjotikar, Matías Günther Wood, A Gupta, Amit Gupta, Anand Gupta, Ashish Gupta, Himani Gupta, Ishita Gupta, L Gupta, Michael Gupta, Rahul Gupta, Sameer Gupta, Shivangi Gupta, Shubhra Gupta, Stuti Gupta, Sujoy Gupta, Addisalem Gurara, Ahmet Guray Durmaz, B Gurbuz, Alican Güreşin, E Güresir, Erdem Güresir, Radu Gurghiș, Muhammed Gürlük, Tegenu Gurmu, E Gurrea-Almela, Elena Gurrea-Almela, Thomas Gürtler, P Gurung, N Gusani, Aleksandr Gusev, C Gustavino, Claudio Gustavino, G Gutiérrez Carrillo, Gonzalo Gutiérrez Carrillo, D Gutiérrez Medina, Valentina Gutiérrez Perdomo, Alitza Gutiérrez Ruiz, M Gutierrez Samaniego, Maria Gutierrez Samaniego, Bernardo Gutiérrez Sougarret, JA Gutiérrez Vásquez, Jose Gutierrez-Banos, CA Gutschow, Fatih Guven, Lillian Guzman, Marlin Guzman, Natalia Guzman, T Guzman, L Guzmán, Marco Guzzo, Sadiya Gwadabe, Usman Gwaram, GP Gwini, Grace Gwini, Solomon Gyabaah, Daniel Gyawu Aning, Adam Gyedu, Derrick Gyimah, Elitsa Gyokova, A Gyori, Joseph Gyuro, G Gyurok, Anton H Schwabegger, Jorman H Tejada, Jeong Ha, Patrick Ha, Mohamed Habad, Charlène Habarugira Inyange, A Habeeb, MK Habeeb, O Habeeb, A Habeebullah, Alaa Habeebullah, Awais Habeebullah, E Haberal, Peter Habertheuer, N Habib, Zakaria Habib, Sosthene Habumuremyi, NA Hacim, A Hackl, Danilo Hackner, J Hadaya, Joseph Hadaya, E Haddad, Elie Haddad, S Haddad, Alyazeed Haddadin, Basel Haddadin, Monique Haddleton, James Haddow, James Hadfield, JN Hadfield, S Hadi, Seyed Hadi Kalantar, Amatallah Hadi Shamsan, Danny Hadidi, Thirza Hadipranata, Alexis Hadjiathanasiou, Andreas V Hadjinicolaou, M Hadjipavlou, Theodoros Hadjizacharias, Claudia Hadlow, L Haenen, Rehana Hafeez, Abdelrahman Hafez, Ahmed Hafez, Mahmoud Hafez, Nour Hafez, Youssef Hafez, Verity Haffenden, Surgeon Hafiz Riaz Hussain Awan, Jonathan Hagan, L Hagander, I Hagbevor, Stephanie Hage, R Hagger, Lea Haiby, Arwa Haidar, Hanan Haidar, Mohammad Haidari, E Haiden, Fayza Haider, Sajjad L Haider, Ali Haider Bangash, Humaira Haider Mahin, Nisar Haider Zaidi, D Haidopoulos, Dimitrios Haidopoulos, Assia Haif, Manal Haij, H Haile, Mhreteab Haile, Nebiyou Hailu, Niguse Hailu, S Hailu, Samuel Hailu, Alexander Haim, Nadav Haim, Maira Haimona, A Hainsworth, Mohd Hairul Nizam Harun, Bashar Haj Hassan, Luma Haj Kassem, Huzifa Haj-Ibrahim, M Hajalamin, S Hajdarević, Mohammed Hajhamad, Farnaz Haji, J Hajiioannou, Jiannis Hajiioannou, Samira Hajisadeghi, R Hajjouz, Tereza Hajkova, M Hajlan, Mana Hajlan, Awsam Hakami, Hadi Hakami, I Hakami, Ibrahim Hakami, Riyadh Hakami, H Hakim, H Hakmi, S Halaseh, Sattam Halaseh, Maxime Halden, Yusuf Halidu Bako, UA Halim, Usman Halim, C Halkias, B Hall, Benjamin Hall, Claire Hall, J Hall, Nicola Hall, J Hallet, E Halliday, Richard Halliwell, Safa Halman, M Halpin, yasser Halwani, B Hama, Ahmed Hamad, Hisham Hamad, O Hamad, MK Hamada, Mohamed Hamada, Mohammed Hamada Takrouney, Shahed Hamadieh, Z Hamady, Hesham Hamaly, Khaled M Hamam, Omar Hamam, Mohanad Hamandi, H Hamayel, M Hambraeus, Ahmed Hamdan, Alaa Hamdan, Haya Hamdan, Sara Hamdoni, Emad Hamdy, Mahmoud Hamdy, Mohamed Hamdy, Omar Hamdy, R Hamdy, Rana Hamdy, Mohammed Hamdy Al-Shazly, Emad Hamdy Gad, Sarah Hamdy Soliman, H Hamed, Mona Hamed, Abdelkader Hamed Abdin, ALi Hamed ALSharqi, Takwa Hamed Ellakwa, Mohamed Hamed Khalid, BZ Hameed, Hiba Hameed Chagla, S Hamei, Peter Hamer, Adwaa Hamid, HK S Hamid, HKS Hamid, Khalid ZMY Hamid, Mohammed Hamid, Muhammad Hamid Chaudhary, Amani hamid Lamari, A Hamidi, Barbara CS Hamilton, E Hamilton, Agnes Hamilton-Baillie, Ali Hammad, Essam Hammad, Farah Hammad, M Hammad, Mirza Hammad Rauf, A Hammed, Ali Hammed, Salah Hammed, Jonathan Hammerschlag, Jaap Hamming, Bernard Hammond, Eric Hammond, J Hammond, John Hammond, JS Hammond, Rob Hammond, Dalia Hammouche, S Hammouche, Salah Hammouche, Mohammad Hammouri, Jacob Hampton, M Hampton, Matthew Hampton, S Hamrang-Yousefi, Alexandra Hamshere, Ahmed Hamss, Ammar Hamza, Amr Hamza, HM Hamza, Muhammad Hamza Sadiq, Basem Hamzah, Ismail Hamzaoglu, Sook Han Yee, K Hanazaki, Kazuhiro Hanazaki, Angela Hancock, S Handa, Siddhartha Handa, Chin Hang Sophia Sin, Chi Hang Yee, Christine Hangaard Hansen, Umma Hani Jaafaru, F Hanif, Mohammad Hanino, VM Hanjoora, John Hanke, Ciara Hanley, Margaret Hanley, A Hanly, Ann Hanly, H Hanna, Joseph Hanna, M Hanna, N Hanna, S Hanna, Sam Hanna, Wael Hanna, A Hannah, Ahmad Hannan Amrullah, Jonathan Hannay, M Hannington, Rodina Hanno, Abdullah Hanoun, Tasvinder Hans, L Hansen, A Hanson, M Hanson, Melissa Hanson, Islam Hany Metwally, Zi Hao Reuel Heng, S Happ, Bushra Haq, I Haq, Rehan Haq, Maha Haqqani, MH Haqqani, Izhar-Ul Haque, Saeef Haque, Cheyaanthan Haran, Carina Harasser, Timothy Hardcastle, H Hardgrave, C Hardie, Claire Hardie, J Hardie, John Hardie, Max Hardie Boys, T Harding, Thomas Harding, Ruth Hardstaff, J Hardt, Sarah Hardwick, A Hardy, Alistair Hardy, NP Hardy, Rawan Harfoush, Dipti Haridas, Sandeep Harigond, Solonirina Harinarindra Ranaivoson, Muhammad Haris Chishti, Muhammad Haris Janjua, Nagenthiram Harivallavan, A Harky, Amer Harky, Niels Harlaar, NJ Harlaar, Natasha Harley, AT Harmantepe, Laura Harmon, Christopher Harmston, Camila Haro, L Haro Supa, Aaya Haron, M Haroon, Waqqas Haroon, Felix Harpain, L Harper, Luke Harper, Aenone Harper Machin, Gemma Harrell, R Harries, Rhiannon Harries, G Harris, Johnathon Harris, Margaret Harris, Muhammad Harris Siddique, Annabelle Harrison, Ben Harrison, E Harrison, EM Harrison, Ewen M Harrison, Haidee Harrison, Siew-Ling Harrison, K Harrison-Phipps, C Hart, V Hart, Bettina Härter, Chris Hartley, Daniela Hartmann, Jacinda Harty, Jessica Harvey, R Harvitkar, R Harwood, Rachel Harwood, AK Harzif, Ahmad Hasan, Asif Hasan, Batool Hasan, E Hasan, MT Hasan, R Hasan, Raashad Hasan, Rama Hasan, Hajar Hasan Kheslat, Damir Hasandić, Ariola Hasani, R Hasanov, DM Hasanuzzaman, Hirotoshi Hasegawa, A Hasenburg, Annette Hasenburg, Fuad Hashem, Lina Hashem, M Hashem, Mohamed Hashem, Yaldasadat Hashemipour, Adil Hashim, AT Hashim, HT Hashim, D Hashimoto, Daisuke Hashimoto, Ali Hashmi, Shiraz Hashmi, Ismail Hasirci, David Haslhofer, A Hasnat, A Hassan, Abdulhakim Hassan, Ahmed Hassan, F Hassan, Gehad Hassan, Ismail Hassan, Karim Hassan, M Hassan, Mekki Hassan, Muha Hassan, Muhammad Hassan, Murtuza Hassan, N Hassan, Nabeel Hassan, R Hassan, Ramy A Hassan, Roa Hassan, Sadik Hassan, Sadiq Hassan, Shamira Hassan, Sulaiman Hassan, Usman Hassan, Yosef Hassan, Arowa hassan abdulrahman Alansari, Seyyed Hassan Adeli, Abdelmonem hassan eid Abdelmonem, Mohamed Hassan Fathy Hassan Abdallah, Mohsin Hassan Khan Roshan, Hiba Hassan Rehmtallah Ahmed, A Hassanin, Ahmed Hassanin, Aliaa Hassanin, Mohamed Hassanin, Karen Hassell, Chiraz Hassoun, Maher Hassounah, Anas Hassouneh, Esraa Hassouneh, Yoko Hasumi, Andi Hasyim, Jaber Hatam, Mohamed hatem elmetwalli eldwini Eldwini, Isobel Hatrick, R Hatz, Rudolf Hatz, C Hatzantonis, Victor Hau, AF Haugstvedt, J Hauptman, H Hauser, F Hauswirth, T Havenhand, Tom Havenhand, N Havers, L Havranek, Hanadi Hawa, Nouran Hawa, I Hawal, Islam Hawal, M Hawari, Mohammad Hawari, A Hawila, Ahmed Hawila, Lydia Hawker, P Hawkin, Alexander Hawkins, Robyn Hawkins, J Hawkyard, S Hayajneh, M Hayashi, Megumi Hayashi, K Hayat, Khizar Hayat, Zara Hayat, Wasim Hayat Khan, F Hayati, Firdaus Hayati, Hussein Hayati, C Hayden, Dana Hayden, Max Hayden, Andrew Hayes, Ross Hayhurst, Tony Haykal, Dickon Hayne, AB Haynes, Alex Haynes, Amelia Haynes, Abi Hayward, Marko Hazabent, I Hazan, J Hazelton, Mohamed Hazem Okail, Khairul Hazim Hamdan, Luis Hdez Miguelena, Zhexi He, E Headon, Tristan Heath, Chelsea L Heaven, Daniel Hechtl, Matthias Heck, R Heckburn, Andreas Hecker, M Hecker, Matthias Hecker, Mohamed Hedi Ghalloussi, Alex Hedley, Megan Hedlund, So Hee Kim, Chan Hee Koh, Anna Heeney, B Heer, Munish Heer, Abbie Heffernan, Elsayed Hegazy, Ibrahim Hegazy, Osama Hegazy, S Hegde, Siddhi Hegde, E Heidari, Farrokh Heidari, Hamid Heidari, Julia Heider, F Heike, Juuso Heikkinen, LM Heindl, Elmar Heinrich, E Heinz, Karl Heinz Stadlbauer, J Heisterkamp, Hala Helal, Dulce Helena Ferreira de Carvalho Carvalho, Sabrina Helena Rossi, Pablo Helguera, Dubravka Heli Litvic, Michael Helley, Philipp Helmer, Hadeel Helmi, O Helminen, Olli Helminen, Youssef Helmy, Ahmad Helmy Zayan, Tessely Heloise, Hanan M Hemead, Abdelrahman Hemida, N Hemmati, M Hemmila, Mark Hemmila, Nigel Henderson, ER Hendriks, H Heneghan, Helen Heneghan, Christin Henein, Kerollos Henes, Marilyn Heng, EA Hennessy, Elizabeth A Hennessy, Sarah Henning, Nicolas Henric, Jose Henrique Albuquerque Messias, Paulo Henrique de Sousa Fernandes, Tiago Henrique de Souza, Thiago Henrique Sigoli Pereira, J Henriques, P Henriques, Pedro Henriques, S Henriques, Susana Henriques, A Henry, Alastair Henry, Jaymie Henry, David Henshall, DE Henshall, Patrick Hensley, Mariel Henzenn, Hwan Heo, D Herappe, Dorihela Herappe, J Heras Aznar, Matheesha Herath, Beate Herbig, Marit Herbolzheimer, György Herczeg, Fernando Heredia, Carles Heredia Llinàs, DM Herghea, Tojomamy Herinjaka Ralaizafindraibe, A Heriot, Alexander Heriot, T Herklots, Koushik Herle, H Herman, Alba Hernáez Arzoz, Jesus Hernan Tovar, E Hernandez, María Hernandez, P Hernandez, Roberto Hernandez, Araceli Hernández, I Hernández, Inés Hernández, R Hernández, MA Hernandez Bartolome, Guillermo Hernandez Gauna, A Hernandez Gutierrez, J Hernandez Gutierrez, Alicia Hernández Gutierrez, L Hernández Miguelena, Araceli Hernández Ramos, Estefania Hernández-García, M Hernández-García, Miguel Hernández-García, S Hernandez-Kakauridze, J Mindy Hernández-Nava, JM Hernández-Nava, Pedro Hernando Calderon Quiroz, C Heron, Charlotte Heron, J Héroux, Ana Herranz Arriero, Danilo Herrera, H Herrera, Héctor Herrera, Miguel Herrera, Enrique Herrera Castañeda, Norberto Herrera Merino, DR Herrera Mora, G Herrera-Almario, Gabriel Herrera-Almario, J Herrera-Esquivel, A Herrera-Gomez, N Herrera-Merino, Julio Herrera-Zamora, I Herrero, Imanol Herrero, Sofía Herrero Gámiz, M Herrero-Lopez, Jose Herreros, Rubén Herreros Ruiz-Valdepeñas, Barbara Herritsch, F Herrle, Johannes Herrmann, P Herrod, Caroline Herron, Jonathan Herron, Yehuda Hershkovitz, Viktoria Herterich, L Herve, Luc Hervé Samison, E Hervieux, Erik Hervieux, J Herzberg, Jonas Herzberg, Torsten Herzog, Helal F Hetta, HF Hetta, Thusitha Hettiarachchi, R Hettige, A Heuer, Annika Heuer, Mateo Hevia, Pelayo Hevia Rodríguez, Nicole Hew, Matt Hewitt, B Heyd, Z Heydari, Marie Heyne-Pietschmann, Emily Heywood, ML Hibbard, M Hichem, L Hidalgo Lariz, C Hidalgo Salinas, Camila Hidalgo Salinas, J Hidayat, Lydia Hiddema, Hishikawa Hidehiko, L Hidi, László Hidi, Masaharu Higashida, George Higginbotham, Andrew Higgins, M Higgins, Mark Higgins, S Higgs, D Highton, Felipe Higuera, Eva Higuera Miguélez, Amanda Hii, Raid Hijazeen, Zaid Hijazi, N Hijazin, Takeshi Hijikawa, Ahmed Hijjawi, N Hilal, Fabián Hilario Mendoza Pedraza, A Hill, Arnold Hill, C Hill, CE Hill, Charles Hill, Ciaran Hill, G Hill, MJ Hill, Rhodri Hill, S Hill, A Hilley, Roxane Hillier, Ahmed Hilmi, Thomas Hilton, Alexander Himstead, Marisol Hinaoui, J Hind, S Hind, T Hine, C Hing, Caroline Hing, JX Hing, Janmejay Hingu, Adam Hingum, Haruaki Hino, CA Hinojosa, Carlos Hinojosa, A Hinton, Naoki Hirai, Teruyuki Hiraki, Chetan Hirani, Kouichi Hirano, Naoyuki Hirata, C Hirche, Christoph Hirche, S Hirji, SA Hirji, Sameer Hirji, Reina Hirooka, Jinso Hirota, Kazuyoshi Hirota, F Hirri, H Hirsch, Jakob Hirsch, Scott Hirsch, Markus Hirschburger, Mohammed Hirsi, Omar Hirsi, D Hiršl, Yoji Hisamatsu, I Hisham, Intisar Hisham Said Hamdun Korea, L Hitchman, Daniel HL Lemmers, Ahmad Hmaideh, Majedah Hmeidan, Wut Hmone, B Ho, M Ho, MF Ho, Michael Ho, Cheuk Ho Lam, Yick Ho Lam, Yuk Ho Liu, A Hoang, Katie Hoban, Maria Hobrok, Andres Hodali, Katherine Hodge, Victoria Hodgetts Morton, H Hodgson, Harry Hodgson, R Hodgson, Russell Hodgson, Ghazal Hodhody, Min Hoe Chew, Daniel Hofer, Markus Hofer, Mary Hoffman, Sebastian Hoffmann, Thomas K Hoffmann, Gwen Hofman, Aisling Hogan, AM Hogan, D Hogan, J Hogan, John Hogan, Kathryn Hogan, A Hogea, M Hogea, Mircea Hogea, R Hogenbirk, Rianne Hogenbirk, RND Hogenbirk, Anders Hogh, SP Hogston, P Höhn, Philipp Höhn, Hannes Hoi, Chi Hoi Lee, RM Højsgaard, Christie Hok Yung Shum, Cole Holan, S Holawe, Simone Holawe, C Holbrook, Charlotte Holbrook, F Holc, Fernando Holc, Daniel Holena, Johannes Holfeld, Abin Holla, E Holler, Paul Hollington, Alexander Hollis, M Hollyman, Marianne Hollyman, T Holme, Thomas Holme, A Holmes, Angela Holmes, Merran Holmes, Samuel Holmes, F Holmner, D Holroyd, David Holroyd, M Holscher, Christopher Holt, Phillip Holt, Katharina Hölz, F Hölzle, Frank Hölzle, Clemens Holzmeister, Seyedeh Homa Hemmasi, R Hompes, Sophie Hon, H Honarpisheh, Human Honarpisheh, Bridget Hone, D Hong, Dennis Hong, T Hong, Jen Hong Ong, Bee Hong Soon, Iain Hood, Kheng Hooi Chan, Mee Hoong See, N Hope, Brent Hopkins, J Hopkins, M Hoque, Raymund E Horch, Vladyslav Hordoskyi, N Horesh, A Horiguchi, Karoline Horisberger, AP Hormis, Julian Horn, Cynthia Horner, Jonathan Horsnell, Rahim Horuz, S Horvath, Maher Hosain, Tatsuki Hoshino, S Hosny, F Hossain, Fahad Hossain, Kamral Hossain, N Hossain, Ruhella Hossain, T Hossain, Ahmed Hossam, Ahmed Hossam Eldin Fouad Rida, Mahmoud Hossameldin Saad Abdelhamid, Mohammad Hossein Khosravi, Mohammad Hossein Nabian, Seyyed Hossein Shafiei, Elahe Hosseini, MR Hosseini Siyanaki, Seyedmohamad Hosseini Zavareh Hosseini Zavareh, Masoumeh Hosseinpoor, M Hosseinzadeh Maleki, Zuzana Hotová, Lachlan Hou, Zhen Hou, Yara Houdifa, C Houlden, A Houmada, Corey Hounschell, H Houshyar, Zakaria Houssaïn Belkhadir, A Houssem, Ammar Houssem, Helen Houston, R Houston, CHC Houtsma, T Houwen, E How Hong, T Howard, D Howden, Michael Howells, Sean Howells, Emma Howie, L Howse, Dileep Hoysal, Tarteel Hrerat, Evguenia Hristova, Kalina Hristova, Marvin Hsiao, Michelle Hsiao, V Hsiao, Yu Hsuen Yang, A Ht rao, B Hu, Jiankun Hu, Sophie Hu, Yu-Ning Hu, L Hua-Feng, Lien Hua-Feng, Eduardo Huaman, E Huamán, E Huamán Egoávil, Huilun Huan, Abel Huang, Lana Huang, Linna Huang, Wai Huang Teng, Thomas Hubbard, J Huber, Verena Huber, Kristin Huber-Strößner, Lauren Huckaby, F Huda, Farhanul Huda, Shahab Huda, Igor Hudic, VE Hudson, Victoria Hudson, Mónica Huecas, M Huecas-Martinez, MA Huertas Fernandez, Benjamin Huggon, Thomas Hugh Lynch, Andrew Hughes, Anne Hughes, Dominique Hughes, F Hughes, I Hughes, Isabel Hughes, JL Hughes, Víctor Hugo Alcalá Torres, H Huhta, Heikki Huhta, Sze Hui Wong, LF Huilca Logroño, L Huisman, R Hultgren, Rebecka Hultgren, Waseem Humayoun, Luis Humberto Govea-Camacho, D Humes, David Humes, Bailey Humphreys, L Humphreys, S Humphries, Ellie Humphry, Majd Hunaiti, WeiPin Hung, Adam Hunt, Benjamin Hunt, I Hunt, J Hunt, Janette Hunt, Louise Hunt, B Huntly, Lucy Huppler, F Hurasha, Heather Hurdle, Conor Hurson, Najam Husain, S Husain, Shatha Husain, Amy Huseyin, Mohammed Husien Yosif Elhafiz, Azar Hussain, Fathi Hussain, Mohammed Hussain, Musarrat Hussain, Zahra Hussain, Zainab Hussain, Shabbar Hussain Changazi, Zahid Hussain Khan, Musheer Hussain Mohamed, Riaz Hussain Siddiqui, Shahzad Hussain Waqar, Dr Hussaini, Ahmed Hussein, Bili Hussein, H Hussein, Hamzeh Hussein, Hasan Hussein, KM A Hussein, L Hussein, R Hussein, Rand Hussein, Haithem Hussein Ali, Hamza Hussein Aly Salama Aly, Maab Hussein Yousif Elhafiz, Husnia Hussen, Romeo Hussey, P Hutchinson, Peter Hutchinson, PJ Hutchinson, R Hutchison, Jörg Hutter, Mohammed Huwaysh, K Huynh, Victoria Huynh, Louise Hviid, Suk Hwan Lee, ES Hwang, Joe Hwong Pang, Ayah Hyasat, Alexander Hyhlik-Duerr, GY Hyman, Boel Hynning, Arianne I Lupián-Angulo, Alessandro Iacomino, Despoina Iakovou, I Iannone, Christopher IAnson, F Iazzetta, Yutaka Iba, Marta Ibáñez Nieto, FJ Ibáñez-Aguirre, T Ibekwe, I Ibi, Treasure Ibingira, Betul Ibis, Iftekhar Ibne Mannan, Gbadebo Ibraheem, M Ibraheem, Maher Ibraheem, Omar Ibrahem, A Ibrahim, Abdelrahman Ibrahim, Adem Ibrahim, Ahmed Ibrahim, Firas Ibrahim, Hamza Ibrahim, Isakwa Ibrahim, Islam H Ibrahim, M Ibrahim, Mohamed Ibrahim, Mohammad Ibrahim, Mohsen Ibrahim, Mostafa Ibrahim, Nourhan Ibrahim, S Ibrahim, Sadiq Ibrahim, Saidu Ibrahim, Shaimaa Ibrahim, Sufyan Ibrahim, Tarek Ibrahim, Z Ibrahim, Zainab Ibrahim, Omar Ibrahim Elsayed, Mohamed Ibrahim Gbreel, Mohammed Ibrahim Mohammed Ali, Samah Ibrahim Omer Mohamed Osman Mohamed Osman, Mustapha Ibrahim Usman, A Ibrahimli, GC Icaza de Marín, M Ida, Mitsuru Ida, Denisse Idalia Campos Mejía, Damaris Idara Anabel Zezular, Yabasin Iddrisu Baba, Ehanga Idi Marcel, Louis Idier, UO Idiz, Jeuel Idowu, Olufemi Idowu, Muhammad Idrees Anwar, M Idrissi, Michele Iester, G Ietto, Giuseppe Ietto, N Iflazoglu, Fizza Iftikhar, Muhammad Iftikhar, Zainab Iftikhar, Ahmar Iftikhar Talib, Kueni Igbagiri, Oluwasuyi Ige, Eva Iglesias Garcia, Jose Ignacio Blanes, Carlos Ignacio Ferrero, José Ignacio Gerchunoff, Matias Ignacio Gonzalez, Jose Ignacio González Martín, Ricardo Ignacio Olmedo Bareiro, José Ignacio Sánchez Méndez, Juan Ignacio Stenner, Mihaela Ignat, Batog Igor, Daniel Igor, P Ihnát, Grace Ihsiu Todd, Katsuyuki Iida, Koji Iida, Patricio III Dumlao, Cesar III Jacinto, Yusuke Iizuka, Munirdeen Ijaiya, Adebimpe Ijarotimi, Attiya Ijaz, Ferdinand Ijekeye, TR Ijichi, Shingo Ikeda, Tatsuhiko Ikeda, Hilary Ikele, Adeel Ikram, H Ikram, S Ikram, Syed Ikramullah Ikramullah, Ijezie Ikwuezunma, Haifaa Il hadad, N Ilahi, R Ilic, Rosanda Ilic, Drochioi Ilie Cristian, M Iliescu, Madalina Iliescu, Ivelina Ilieva, Abdullah Ilktac, Matthias Ilmer, D Ilukpitiya, Hiroshi Imai, SM B Imam, Alphonsine Imanishimwe, Abbassi Imed, H Impellizzeri, Harmony Impellizzeri, O Impey, F Imran, Farrah-Hani Imran, Jonathan Imran, Rizwana Imran, Muhammad Imran Anwar, Syed Imran Bukhari, M Imran Khan, Muhammad Imran Khokhar, Joe Imumoren, Hina Inam, M Inama, Marco Inama, Ilker Ince, P Incollingo, Paola Incollingo, Joseph Incorvia, Mayang Indah Lestari, F Indrarti, Ivana Ines Pedraza Salazar, Vera Inês Ribeiro, César Infante, A Ingabire, Carlo Ingaldi, Nikhil Ingle, Laura Inglis, Zorka Inic, César Íñiguez Martínez, Maria Inmaculada Ruiz Montesinos, María Inmaculada Valldeperas Hernández, Yasushi Innami, Junichi Inokuchi, Hiroyuki Inoue, Yui Inoue, Osvaldo Insfran, Sylvie Inyange, M Inzunza, Maria Ioanna Antonopoulou, Evangelia Ioanna Tsiourva, A Ioannidis, Argyrios Ioannidis, O Ioannidis, Orestis Ioannidis, Oreste Iocca, Cojocaru Ion, Serban Ion Bubenek Turconi, NS Ionescu, Sebastian Ionescu, Florin Iordache, Eirini Iordanidou, V Iori, Valentina Iori, RV Iosifescu, Olga Ioulia Semkoglou, Claudio Iovino, D Iovino, Domenico Iovino, Christopher Ip, J Ip, M Ip, Ayberk İplikçi, S Ippoliti, Simona Ippoliti, P Ipponi, Atif Iqbal, Ayesha Iqbal, Faizan Iqbal, Javaid Iqbal, Mohammad Iqbal, Ramiz Iqbal, Zafar Iqbal, Omer Iqbal Cheema, Magnifique Irakoze, Shirin Irani, Iran Irani Durán Sánchez, Maria Iraola, MJ Irarrázaval, Patrick Ireland, Beyza Irem Yabaci, Rebecca Ireson, Oseihie Iribhogbe, Tomoya Irie, Daisuke Irimada, A Irimie, Alexandru Irimie, Gunko Irina, Paola Irina Eusebio Jimenez, Tomoko Irisawa, Omorodion Irowa, Abeer Irshad, E Irune, Ekpemi Irune, E Irvine, V Irvine, Isabirye Isa, Otolia Isaac, Olusegun Isaac Alatise, John Isaac Merin, Anna Isaacs, R Isaacs Beron, Joana Isabel Almeida, Ana Isabel Avellaneda Camarena, Maria Isabel Manso, Maria Isabel Prieto-Nieto, Inês Isabel Sampaio da Nóvoa Gomes Miguel, Frigerio Isabella, Tetsuro Isada, Aliyu Isah, Andres Isaza-Restrepo, Gloria Isela Mendoza Frías, R Isernia, Muhammad Isfandyar Khan Malik, P Ishak, Amna Ishaq, Nazia Ishaque, Katsuhiko Ishibashi, S Ishida, Sachi Ishida, Yusuke Ishida, H Ishii, Haruka Ishikawa, M Ishikawa, Masashi Ishikawa, Makoto Ishitobi, Taku Ishizaki, Daniyal Ishtiaq, Christian Isichei, Mercy Isichei, Filipe Isidro, A Isik, Ozgen Isik, Monica Iskander, Othman Iskander, D Isla-Ortiz, David Isla-Ortiz, AA Islam, N Islam, Rahela Islam, S Islam, Shahnoor Islam, SM Nazmul Islam, Sumayya Islam, C Ismael, Salam Ismael, Fahad Ismail, Hlma Ismail, L Ismail, Lamiese Ismail, M Ismail, N Ismail, Nasiru Ismail, O Ismail, Samir Ismail, Zainab Ismail, Jameel Ismail Ahmad, Mohammad Ismail Attar, Hafsa Ismail Ibrahim Naiya, Taha ismail Sefrioui, G Ismaili, Ghiath Ismayl, SM Isolani, Fadi Issa, Michael Issa, Mohannned Issa, N Issa, Adamu Issaka, R Itani, Rania Itani, Shingo Ito, Oda Ituze, Stoian Iudin, Muresan Iulia Andrada, Kevin Ivan P Chan, Giorgio Ivan Russo, N Ivancevic, T Ivanov, Tsvetomir Ivanov, Anna Ivanova, Nenad Ivanović, Igors Ivanovs, Andjela Ivezić, Ebikela Ivie Baidoo, Paolo Ivo Cavoretto, Hideki Iwahashi, M Iwasaki, Masae Iwasaki, Shintaro Iwata, Ifeanyi Iwuagwu, K Iyengar, Karthikeyan Iyengar, Priyanka Iyer, Vikram Iyer, David Izadi, A Izaguirre, Aldo Izaguirre, J Izbicki, Jakob Izbicki, Ana Izquierdo, O Izquierdo, S Izwan, F Izzo, Francesco Izzo, E Jabagat, Abd Jabar Nazimi, Nicolette Jabbour, Ahmad Jaber, Kefah Jaber, Abdulla Jabr, Massa Jabra, R Jach, J Jackman, Jamaall Jackman, A Jackowski, Claire Jackson, H Jackson, K Jackson, Karl Jackson, Richard Jackson, A Jacob, S Jacob, Arun Jacob Philip George, Adeline Jacobs, Daniel Jacobs-Tulleneers-Thevissen, F Jácome, Filipa Jácome, Jean Jacques Tuech, Piyush Jadhao, J Jaekers, Jay Jaemin Park, Ahmad Jafar, Mehraneh Jafari, Alisha Jaffer, N Jagadeesh, Tarkan Jäger, Tomaz Jagric, Asif Jah, Abdussalam Jahan, Alhadi Jahan, Shahrokh Jahan Bini, M Jahnen, A Jain, Amit Jain, Anshini Jain, Anuj Jain, Deepak Jain, Divakar Jain, Kavitha Jain, Manoj Jain, P Jain, Prateek Jain, R Jain, Ritu Jain, Sunjay Jain, Vaibhav Jain, Anthony Jaipersad, Somil Jaiswal, Molly Jakeman, Patricia Jako, James Jakub, Abdul Jalil, S Jalili, S Jallad, Samer Jallad, Heba Jaloun, A Jamal, Abid Jamal, Aiman Jamal, M Jamal, Mohammad Jamal, Faris Jamal Abu Zanouneh, Mohamed Jamal Elshref, Sarfraz Jamali, Saja Jamaliah, Suniza Jamaris, PS Jambulingam, W Jamel, Rorisang Jamela, A James, D James, Deeptiman James, G James, Sophie James, Tobias James, Tracy James, Ayotunde James Fasunla, Matthew James McGuinness, Ryan James Ocsan, Ifeanyi James Orji, Salem Jamhour, NB Jamieson, Nigel Jamieson, Chloe Jamieson-Grigg, Manahil Jamil, T Jamil, Tahir Jamil, AA B Jamjoom, G Jamjoum, Mohamed Jammal, Kashif Jan, Yousaf Jan, Gregor Jan Kocher, Josif Janchulev, Aeris Jane D Nacion, Jingya Jane Pu, Seyoung Jang, Akash Jangan, A Jangjoo, DP Jani, A Janjua, Atif A Janjua, Azwa Janjua, MH Janjua, J Jankau, Wolfgang Janni, Shirley Jansen, Yanina Jansen, J Janson, M Janssen, Y Janssen, Ward Janssens, Gediminas Januška, P Januszyk, K Japheth, Alejandra Jara Maquilón, Enas Jaradat, Gustavo Jardim Volpe, Ruari Jardine, Peter Jarin, UM Jariod-Ferrer, Natalie Jarkas, Abdulaziz Jarman, Stefanie Jarmusch, C Jarry, M Jarvis, Kristijonas Jasaitis, D Jasarovic, Elmer Jason Cruz, Miren Jasone Diez Zapirain, Y Jauhari, SM Jaume Böttcher, S Jaunoo, SM Javad Mortazavi, M Javadpour, Mohsen Javadpour, Haroon Javaid Majid, Hannah Javanmard-Emamghissi, Anum Javed, Ayesha Javed, Aymen Javed, Dania Javed, Hina Javed, S Javed, Saad Javed, Sundas Javed, Umer Javed Chughtai, Muhammad Javed Iqbal, Manal Javid, P Javid, Domingo Javier Aguilera Maidana, Francisco Javier Bonilla-Escobar, Euler Javier Burbano Luna, Francisco Javier Fernández Pablos, Víctor Javier García Porcel, Carlos Javier Gómez Díaz, Antonio Javier Gomez Poveda, Ernesto Javier Guerrero Casillas, Francisco Javier Ibáñez-Aguirre, Francisco Javier León Frutos, Francisco Javier Llamas- Macias, Francisco Javier Ortiz de Solórzano Aurusa, Francisco Javier Redondo Calvo, Veronika Javurkova, M Jawad, Monir Jawad, Muhammad Jawad, Z Jawad, Muhammad Jawad Zafar, Yashpal Jaware, Haya Jawish, Natalia Jaworska, J Jaya, Abhilash Jayakumar, U Jayarajah, Umesh Jayarajah, Harish Jayaram, RB Jayaram, B Jayasankar, Balaji Jayasankar, S Jayasekara, JD Jayasinghe, Ravindri Jayasinghe, Sumudu Jayasinghe, DMCS Jayasundara, A Jayawardane, Asanka Jayawardane, Tanmay Jaysingani, Basel Jazieh, Haragirimana Jean de Dieu, Clement Jeandel, J Jeater, William Jebril, Julia Jedanowski, Nicole Jedrzejko, Ana Jeelani, D Jeevan, David Jeevan, Niall Jefferson, Nikola Jeftic, R Jeganathan, Reubendra Jeganathan, Shah Jehan, Muhammad Jehangir Malik, Emran Jeitan, Clara Jeketera, Ivan Jelčić, Jelenko Jelenkovic, C Jelley, D Jelovac, Drago Jelovac, D Jenkins, Victoria Jenkins, M Jenkinson, MD Jenkinson, Michael Jenkinson, Michael D Jenkinson, E Jenner, Seamus Jennings, H Jenny, Eric Jensen, C Jenvey, SMH Jeoffrey, H Jeong, K Jeremic Stefanovic, S Jeri-McFarlane, Webster Jerry Noronha, Ora Jesner, Hans Jesper Del Mundo, Jose Jesus Herrera, Antonia Jesús López López, Rey Jesus Romero, Joel Jesús Sánchez Estupiñan, Marko Jevric, Anupama Jeyakumar, Nivedan Jeyamanoharan, Rathan Jeyapalan, D Jeyaretna, Deva Jeyaretna, C Jezieniecki, Carlos Jezieniecki, Deepak Jha, Mark Jheric Tesil, Eu Jhin Loh, Fangzhi Jia, Wei Jia, Shawn Jia Hwang Tan, N Jiagge, Nuna Jiagge, E Jianu, L Jiao, M Jibreel, T Jichi, Tarik Jichi, Wen Jie Chin, Cristian Jimenez, G Jimenez, Raul Jimenez, V Jimenez, Virginia Jimenez, Laura Jiménez, V Jiménez Carneros, E Jimenez Higuera, M Jiménez Jiménez, J Jimenez Miramón, Javier Jimenez Miramón, LJ Jimenez Ramirez, Carmen Jimenez Sanchez, M Jimenez Toscano, Marta Jimenez Toscano, X Jimenez Villanueva, Carlos Jiménez Viñas, Marta Jiménez-Jiménez, L Jimenez-Roldan, Luis Jimenez-Roldan, J Jimeno Fraile, Jaime Jimeno Fraile, Nam Jin Kim, Hiang Jin Tan, Jang Jin-Young, Daniel Jira, Moa Jira, Ghassan Jisry, Jin Jiun Mah, Haithem Jlassi, Manal Jmaileh, Luis Joaquín García Flórez, Fahmi Jobran, D Jochems, Carolin Jödicke, Derlis Joel Ojeda Villasboa, HK M Joeng, Shivangi Jog, S Johan, Syamim Johan, Anika Johanna Agoncillo, Marco Johannes Battista, Celestine John, J John, Gareth John Bowen, Christopher John Macapugay, C Johnson, D Johnson, David Johnson, O Johnson, Brian Johnston, Sean Johnston, Craig Johnstone, J Johnstone, Vladimir Jokic, Miloš Joković, Joshua Jolissaint, JS Jolissaint, Danielle Jolly, Sami Jomaa, Danny Jon Nian Wong, E Jonas, Boakye-Yiadom Jonathan, A Jones, C Jones, D Jones, Elizabeth Jones, G Jones, Gbenga Jones, GP Jones, J Jones, L Jones, M Jones, Mark Jones, R Jones, Robin Jones, Rosalind Jones, RP Jones, Terence Jones, TR Jones, Larne Jones-Whiting, FH W Jonker, Frederik Jonker, Pascal Jonker, PK C Jonker, PKC Jonker, C Jonsson, Carina Jonsson, ML Jönsson, R Jorba, Misericòrdia Jordà Solé, Caitlin Jordan, S Jordan, Shannon Jordan, Stevan Jordan, Henrique Jorge Guedes Neto, Eduardo Jorge Premoli, Lars N Jorgensen, T Jorgensen, Thomas W Jorgensen, TW Jorgensen, M Jornet-Gibert, Montsant Jornet-Gibert, Noriega José, Diego José Almada Casañas, Antonio Jose Alonso Villalba, Francisco José Barbosa Camacho, Rafael Jose Beltran, Diego Jose Caycedo Garcia, João José Corrêa Bergamasco, Rodolfo Jose Favaretto Filho, F José Fernandez Coimbra, Felipe José Fernandez Coimbra, Pedro José Gil Vázquez, Maria Jose Gomez-Jurado, Maria Jose González-Gimeno, Juan Jose Jaramillo Roncancio, Christoph José Klein Zampaña, Marcelo José Maia Azevedo Costa, María José Martínez, Maria Jose Martinez Velázquez, Antonio José Montoya Casella, Albaro José Nieto Calvache, Carlos Jose Perez Rivera, Maria José Reche Padilla, Enrique Jose Ruiz Velasquez, María José Sangüesa, Juan José Segura-Sampedro, Carlos Jose Zuloaga Fernandez del Valle, María Josefa Cuevas López, Johanna Josefine Strotmann, Lallu Joseph, Lule Joseph, Reece Joseph, Sinu Joseph, Treasa Joseph, Yorke Joseph, A Joshi, Anuja Joshi, Ashoo Joshi, D Joshi, Mohit Joshi, P Joshi, Prabesh Joshi, S Joshi, Vaishali Joshi, Vinay Joshi, Y Joshi, Zaman Joshua, Shosaburo Jotaki, Harihara Jothi, A Jotic, Ana Jotic, Lionel Jouffret, Patrick Jovan Gagno, T Jovanoski, Tomislav Jovanoski, K Jovanovska, Katerina Jovanovska, L Jovcheski, JM Jover, E Jovine, Elio Jovine, DP Joyce, Yan Joyce Ming, Karen Joyce Velasco, Kathir Joyson, Jesus Jr Dabalos, Eunmaro Ju, Jose Juan Gonzalez Sanchez, Rodrigo Juaneda, Moises Juarez, M Juarez-Pomes, Yolanda Jubete Castañeda, N Judkins, Nicholas Judkins, Mary Jue Xu, R Jugdey, C Juillard, Catherine Juillard, Matthew Jukes, M Jukić, David Julià Bergkvist, Maria Julia Corbetta Machado, E Julià-Verdaguer, Maria Juliana Sanchez, Andrea Juliana Vega Calvera, Benji Julien, Yuki Julius Ng We Yong, Aaron Julius Punnen, J Juloski, Jovan Juloski, Isam Juma, Irfan Jumabhoy, S Junca-Marti, Josephine Jung, M Jung, Stefanie Junker, Evelina Juodiene, Domantas Juodis, M Jurado Ruiz, Maria Jurado Ruiz, Jonas Jurgaitis, M Juricic, Melodie Juricic, Steffanie Jury, Richard Justin Davies, B Juthani, Gs Jutley, Zeljka Jutric, Nina Jyne Minette Dela Cruz, Gayathri Jyothish, Tejeswini K K, DKK K M, Samuel Ka Kin Ling, H Kaafarani, Claudia Kabanyana, Rakan Kabariti, Taha Kabbaj, Saadullah Kabbany, Obaida Kabel, Tousif Kabir, Mohammed Kabir Abdullahi, Mohammed Kabir Abubakar, Apoorva Kabra, Navid Kabuli, Wilberforce M Kabweru, Stephen Kache, Ahmad Kachoie, SEO Kacimi, Akram Kadamani Abiyomaa, K Kadantseva, Saidu Kadas, Nardeen Kader, Mustafa R Kadhim, Mohamad Kadi, M Kadija, S Kadija, Innih Kadiri, Lama Kadoura, D Kaemmerer, Alper Kafkasli, Abdullah Kağan Zengin, Kota Kagawa, S Kahane, Hiba Kahi, J Kahiu, Josephine Kahiu, Ehab Kahka, A Kahn, Alexis Kahn, J Kahn, Judith Kahn, Li Kai, Dilyara Kaidarova, Yu Kaiho, S Kailasam sivamurthy, Suresh Kailasam Sivamurthy, M Kajic, Martin Kajic, B Kajmaković, Boris Kajmaković, Michael Kakas, Andrew Kakeeto, V Kakotkin, Victor Kakotkin, Navneet Kala, Prakash Kala, Dana Kalagi, I Kalaitsidou, Theodosis Kalamatianos, N Kalavrezos, Nicholas Kalavrezos, Ayrat Kaldarov, A Kale, Ahmet Kale, S Kale, Sachin Kale, SS Kale, R Kalenderov, Anna Kaleva, Fotios Kalfas, JC Kalff, Jörg C Kalff, Christos Kalfountzos, I Kaliamoorthy, VK Kalidindi, Muhammad Kalim, M Kalın, Murat Kalın, Senad Kalkan, M Kalkat, K Kalkwarf, Kyle Kalkwarf, Mostafa Kallaf, Mamdouh Kallas, Vasileios Kalles, SK Kallikere lakshmana, Michael Kallmayer, Katinka Kallos, Attila Kalman, I Kalogiannidis, Ioannis Kalogiannidis, Nikos Kalogritsas, K Kalopita, Neeraj Kalra, NS Kalson, F Kalt, Athula Kaluarachchi, Neha Kalwadia, A Kalyanasundaram, Asanish Kalyanasundaram, N Kalyva, Aristotelis Kalyvas, A Kam da Silva Andrade, M Kamal, Wajahat Kamal, Awad Kamal Awad Osman, Bhavani Kamalakannan, A Kamali, Nelson Kamali, A Kamalov, L Kaman, Lileswar Kaman, MFA Kamarizan, N Kamath, Tatsuya Kambara, B Kamburoglu, F Kamel, Mahmoud Kamel, P Kamenova, Paolina Kamenova, M Kamenskikh, BS Kamera, Aleksejs Kaminskis, Akio Kamiya, Mizue Kamiyama, Hatem Kamkoum, Christian kammerer Kammerer, Christian Kammerlander, Elhusain Kamoka, C Kamphues, Carsten Kamphues, Liisa Kams, Rahul Kanade, D Kanagal, Trisha Kanani, Anastasios Kanatas, S Kanavathy, Prodromos Kanavidis, Akihiro Kanaya, Pankaj Kandwal, S Kaneko, Satoi Kaneko, Vasiliki Kanellopoulou, Eiki Kanemaru, Sayaka Kanematsu, Pepa Kaneva, Sara Kanfar, C Kang, J Kang, Niel Kang, Harsh Kanhere, R Kanitkar, Hadyn K N Kankam, Burak Kankaya, Lavanya Kannaiyan, Ravi Kannan, Sreejith Kannummal Veetil, Kari Kansal, R Kansay, Rajeev Kansay, Baturay Kansu Kazbek, Ravi Kant, Uma Kant Dutt, S Kanthasamy, E Kantor, Astha Kantroo, E Kaouras, Nandkishore Kapadia, G Kapetanios, Georgios Kapetanios, S Kapiris, Stylianos Kapiris, Mark L Kaplan, Mehmet Kaplan, Nathan Kaplan, Tuğba Kaplan, M Kaple, C Kapoen, K Kapoor, S Kapoor, Swapnil Kapote, K Kapriniotis, P Kapsampelis, Lakith Kapuge, K Kapur, Anna Kapustina, AR Kar, Madhabananda Kar, Halil Kara, Y Kara, Yasi̇n Kara, O Karaaslan, Kerim Karabulut, Mehmet Karabulut, Bugrahan Karaca, H Karaca, G Karadeniz Cakmak, Dimitra Karageorgou, G Karagiannidis, Georgios Karagiannidis, Omer Karahan, Tayfun Karahasanoğlu, Sema Karakaş, HK Karakullukcu, Basil Karam, BS Karam, E Karam, James Karam, Jose Karam, Mohammad Karam Chaaban, E Karaman, M Karamanliev, Martin Karamanliev, A Karamarkovic, Aleksandar Karamarkovic, T Karami, S Karandikar, Sharad Karandikar, P Karanicolas, AKA Karantenachy, Shoura Karar, Irem Karatas, Ioannis Karavokyros, A Karbalaie, M Karbowiak, A Kareem Hama Ghareeb, CT Karia, Maahir Kariem, Nazmie Kariem, Costa Karihaloo, A Karim, S Karim, Sabbir Karim, Ebrahim Karimi, Ali Karimi Karimi, Liz Karina, Rajeev Kariyattil, Shirin Karkada, B Karki, Robert Karlo, Rahi Karmarkar, Santosh Karmarkar, Sunaina Karna, Gomathi Karnan, Ruchi Karnatak, Paraskevi Karona, Eleni Karoni, Mehdi Karoui, L Karout, Lina Karout, S Karout, Samar Karout, Mariia Karpenko, Ilya Karpov, M Karthigeyan, Madhivanan Karthigeyan, Intan Kartika Kamarudin, Eranda Karunadasa, L Karydakis, Lysandros Karydakis, Ashenafi Kasaye, C Kaselas, Christos Kaselas, M Kashif, Muhammad Kashif, Eiji Kashiwagi, F Kashora, V Kasivisvanathan, Ł Kaska, George Kasotakis, D Kassa, Dawit Kassa, Haftamu Kassa, MB Kassab, Mohamad B Kassab, Berhanu Kassahun, Miklos Kassai, Al-Faraaz Kassam, K Kassam, Nausheen Kassam, Bersabeh Kassaye, R Kassir, Radwan Kassir, Terhemen Kasso, Pagona Kastanaki, Zeljko Kastelan, Dimithi Kasthurirathne, A Kataria, Lena Katharina Mueller, Sheyla Katherine Diaz Mora, Tharangani Kathiravan, Anne Kathleen Ganal-Antonio, Mary Kathryn Abel, H Kato, Manabu Kato, T Kato, Airi Katoh, I Katsaros, L Katsiaras, Emmanuel Katsogridakis, A Kattakayam, Arjun Kattakayam, Abdullah Kattan, Jevgeni Katunin, Matthew Katz, Philipp Kauffmann, Micayla Kaufman, A Kaufmann, Angelika Kaufmann, Claudia Kaufmann, P Kaul, J Kauppila, JH Kauppila, Joonas Kauppila, Joonas H Kauppila, Krista Kaups, Amanjot Kaur, Apjit Kaur, Gurleen Kaur, Gurvinder Kaur, Harmanpreet Kaur, J Kaur, Jasprit Kaur, P Kaur, R Kaur, Navdeep Kaur Ghuman, Jaspreet Kaur Seehra, M Kaushal, Manish Kaushal, R Kaushik, Robin Kaushik, Tirathram Kaushik, Vivek Kaushik, Alfie Kavalakat, A Kavaliauskaitė, Dara Kavanagh, DO Kavanagh, N Kavčič, Niko Kavčič, S Kavic, Thumuluru Kavitha Madhuri, Aya Kawachi, I Kawagoe, Izumi Kawagoe, M Kawaguchi, Masahiko Kawaguchi, Akira Kawai, Natsuko Kawamata, Kenji Kawamukai, K Kawamura, Kenji Kawamura, Abdulmonem Kawas, Yosuke Kawasaki, Mahmoud Kawu Magashi, Hannah Kay, Rozan Kaya, T Kaya, Tayfun Kaya, Akshat Kayal, AA Kayali, B Kayan, B Kayani, K Kayani, Stephen Kaye, B Kaymak, Olushola Kayode Fasiku, Victor Kayode-Nissi, E Kayombo, Emile Kayombo, Silvia Kayser Mata, MY Kayyal, Ammar Kayyali, E Kazachenko, Ekaterina Kazachenko, Ozgur Kazan, Mohammad Kazem Moslemi, K Kazemi Esfe, Amr Kazim, Muhammad Kazim Rahim Najjad, Abbas Kazmi, S Kazuma, Satoshi Kazuma, S Kazzaz, Sarmad Kazzaz, Joshua Kealey, D Kearney, David Kearney, James M Keatley, E Kebapçı, Andrey Kebkalo, Aristotelis Kechagias, Nahla Kechiche, B Keeler, Daniel Keese, Samer Kefo, Farid Kehdy, Wang Kei Chiu, W Kelder, Dionysia Kelgiorgi, Sorcha Kellett, Brian Kelley, Andrew Kelly, Ben Kelly, John Kelly, Kathrin Kelly, Kevin Kelly, M Kelly, Mairéad Kelly, Michael Kelly, Orlaith Kelly, Ronan Kelly, Sarah Kelly, CJ Kelty, Ifeanyichukwu Kelvin Egbuchulem, Uchenna Kelvin Omeje, Aya Kelzia, Ifeanyi Kem Onubogu, Ben-Lawrence Kemah, Achmad Kemal Harzif, G Kembuan, Gabriele Kembuan, Vanessa Kemmetinger, P Kempter, Sebastian Ken-Amoah, Brittany Kendall, Farzaneh Keneshlou, Mihaly Kenez, M Kenic, Marko Kenic, C Kennedy, L Kennedy, N Kennedy, R Kennedy, Andrew Kennedy-Dalby, R Kennelly, Rory Kennelly, Maria Kenner, Yvann Kenneth Benosa, Adi Kenoshi, A Kent, E Kent, I Kent, C Keogh, John Keogh, S Keogh-bootland, Markéta Kepičová, Alexander M Keppler, AM Keppler, Lena Keppler, MR Keramati, MD Keramida, AA Kerawala, Cyrus Kerawala, Thomas Kerforne, M Kerin, MJ Kerin, A Kerman, J Kerman, H Kerndl, Jola Kerpaci, David Kerr, Megan Kerr, NA Kerr, Megan Kershaw, Venkatesh Kesarla, M Keskin, Metin Keskin, S Kesseli, F Kethy, MN Ketkar, S Ketting, MH F Keulen, B Kewlani, Vishal Kewlani, Amana Kezze, Nikhil Khadabadi, Mamoona Khadam, Salma Khadem Alsrouji, S Khader, Sereen Khader, Kouidri Khadidja, F Khadwardi, Ali Khafaja, AKM Khairul Basher, F Khajavi-Mayvan, M Khajeh Alizadeh Attar, A Khajuria, Abdullah Khalaf, M Khalaf, A Khaled, Ahmed Khaled, Mohamed Khaled, Mhd Khaled Alkasser, Nuran Khaled Aly, Omar Khaled Mohamed Eid, A Khaleel, Tahir Khaleeq, M Khalefa, Ismaeel Khalid, K Khalid, Muhammad Khalid, Shahril Khalid, Tabinda Khalid, Ziaullah Khalid, Ahmed Khalid alhadheeri, Shayan Khalid Ghaloo, Raja Khalid Shabbir, A Khalifa, Aya Khalifa, Eiman Khalifa, Houda Khalifa, Islam Khalifa, M Khalifa, A Khalil, Kareem S Khalil, Mariam Khalil, Mohammed Khalil, Rasha khalil Alsayyad, F Khaliq, Sameera Khaliq, T Khaliq, Tanwir Khaliq, Mohamed Khallaf, A Khamees, Almuatasim Khamees, A Khan, Aimal Khan, Aneesah Khan, Anwar Khan, Asher Khan, Farheen Khan, Fatima Khan, Fatma Khan, H Khan, Hamad Khan, Hamza Khan, Hassan Khan, HH Khan, J Khan, Jamal Khan, Jim Khan, K Khan, Karishma Khan, Khizar Khan, Maaz Khan, Maham Khan, Majid Khan, Maryam Khan, MK Khan, MS Khan, MT Khan, MTJ Khan, Najeed Khan, R Khan, Rohma Khan, Romaisa Khan, S Khan, Sabina Khan, Sadia Khan, Salman Khan, Sami Khan, Shane Khan, Shifa Khan, Tabassum Khan, U Khan, W Khan, Wasim Khan, WH Khan, Tanishq Khandelwal, Aseel Khanfer, Sukhwant Khanijaun, H Khansaheb, Hamda Khansaheb, Zubair Khanzada, Siddhant Khare, H Kharkar, Dimple Kharkongor, Barihan Khasawneh, Saad Khashogji, Hazem Khatab, Roa Khatatbeh, Chetan Khatri, Mazin Khattabi, F Khatun, Rong Khaw, SC Khaw, I Khawaja, UA Khawaja, S Khayat, Maymona Khayata, Tareq Kheirbek, Samer Khel, Zine-Eddine Khene, Osama Kherallah, Safeena Kherani, Talal Khewater, Priyatma Khincha, MJ N Kho, A Khodarahmi, Ahmad Khoja, Christopher Khoory, Victoria E Khoronenko, Amir Khoshbin, M Khosravi, Mohammad Khosravi, Gleb Khrykov, KJ O Khu, Wafa Khudier, Susanta Khuntia, Helene Khuong, Taran Khurana, Muhammad Khurram Jameel, Zain Khurshid, K Khutsishvili, K Khwaja, E Khya, Kevin Ki Wai Ho, Yong Kiat Goh, Sey Kiat Terence Lim, Muthoni Kibunyi, H Kiconco, Tatsuya Kida, B Kidane, M Kidane, Meklit Kidane, John Kiely, P Kienle, K Kieran, Aoife Kiernan, C Kies, David Kieser, SY Kiessling, Omar Kifayeh, Motohiro Kikukawa, Nura Kilic, G Kilinc, Gizem Kilinc, G Kilinc Tuncer, S Killeen, Shane Killeen, Rhona Kilpatrick, B Kim, E Kim, G Kim, Gleb Kim, NJ Kim, M Kimbrough, Mary Kimbrough, Motonobu Kimizuka, Angharad King, J King, Jasmin King, M King, Martin King, N King, SD King, Sebastian King, Stratton King, B King-Koi, Dale Kingsley Sy, G Kinnaman, Fumio Kinoshita, H Kinoshita, Hidefumi Kinoshita, J Kinross, J Kinsella, Racheal Kirabo, P Kirchweger, Patrick Kirchweger, Nikolaos Kiriakopoulos, Lydia Kirillova, A Kirk, EF Kirkan, B Kirmani, BH Kirmani, Bilal Kirmani, S Kirmani, Y Kirmizi, Yasemin Kirmizi, Mikhail Kirov, A Kirschniak, Andreas Kirschniak, Joel Kiryabwire, Ravi Kishore Barla, A Kisiel, U Kisser, Ulrich Kisser, Castro Kisuule, Masato Kita, Hiroaki Kitade, Hiroyuki Kitagawa, M Kitchen, Yury Kitsenko, Madhav Kittur, Ronald Kiweewa, Tevfik Kıvılcım Uprak, Abdulqader Klaho, D Klaristenfeld, Jaroslav Klat, T Klatte, TO Klatte, Tobias Klatte, Friederike Klauke, J Kleeff, Jorg Kleeff, Z Kleiman, A Kler, Fredrik Klevebro, Karel Klíma, S Klimopoulos, A Klimov, JH G Klinkenbijl, W Kloc, C Kloppers, Christo Kloppers, J Klose, Johannes Klose, Luis Kluth, G Klutts, Garrett Klutts, S Kmezić, Stefan Kmezić, Elizabeth Kmiotek, U Kneser, Ulrich Kneser, Jure Knez, D Knezevic, Djordje Knezevic, Darko Knežević, Dominic Knight, J Knipschild, Julia Knipschild, M Knitschke, Michael Knitschke, WT Knoefel, Christian Knorr, H Knotzer, Hans Knotzer, Brett Knowles, C Knowles, J Knowles, M Ko, Satoshi Kobayashi, Takayuki Kobayashi, Toshinori Kobayashi, Yasuma Kobayashi, Yoichi Kobayashi, Isaac Kobe, Nina Kobilica, T Koc, MA Koç, A Kocatas, Ali Kocataş, B Kocer, Belma Kocer, C Koch, Christian Koch, I Koch, Oliver Koch, GJ Kocher, Hemant Kocher, HM Kocher, Stanislav Kocherov, V Kochetkov, Viktor Kochetkov, VS Kochetkov, T Kochiyama, Tsukasa Kochiyama, Koshy Kochummen, Milan Kocic, Bogdan Koczy, S Kodange, Almat Kodasbaev, D Koenig, F Koeninger, Evans Kofi Agbeno, Yuko Koga, P Koggoh, Patience Koggoh, Eleni Kogia, P Köglberger, Paul Köglberger, A Koh, Amanda Koh, Cherry Koh, Frederick Koh, F Koh Hong Xiang, Frederick Koh Hong Xiang, Z Kohistani, Zaki Kohistani, D Koike, Minako Koizumi, Edem Kojo Dzantor, S Koju, SY Kok, H Köken, Georgios Kokkinos, A Kokobelyan, P Kokoropoulos, Panagiotis Kokoropoulos, George Kokosis, ÜC Köksoy, V Kolaityte, Valdone Kolaityte, Juraj Kolak, Arif Kolethekkat, M Koleva Radica, A Kolias, Angelos Kolias, Angeliki Kolinioti, Georgios Koliopoulos, Florestan Koll, C Kolla, V Kollias, O Kollmar, Otto Kollmar, Maria Kolokotroni, Kemalettin Koltka, A Kolusab, Snigdha Komatineni, Melanie Komaz, YS Kömek, N Komen, Niels Komen, S Kommu, A Konarski, Ibrahima Konate, Can Konca, Igor Koncar, S Konda, Panagiotis Kondilis, Anish Koneru, S Konev, JCH Kong, Daniela König, TT König, A Königsrainer, Alfred Königsrainer, I Königsrainer, Ingmar Königsrainer, Suzana Konjevoda, Anna Konney, Yoshiharu Kono, C Konrads, Christian Konrads, M Konstadoulakis, Manousos Konstadoulakis, K Konstantinidi, Michael Konstantinidis, MK Konstantinidis, Sofia Konstantinidou, C Konstantinou, J Konsten, Joop Konsten, E Kontis, C Kontopoulou, Christina Kontopoulou, Konstantina Kontopoulou, C Kontovounisios, Kenneth Koo, Mohsen Koosha, Slava Kopetskyi, T Kopjar, Tomislav Kopjar, Leon-Gordian Köpke, C Korais, Christos Korais, S Korasidis, Stylianos Korasidis, C Koratzanis, Moniba Korch, N Korchazhkina, DS Korkmaz, D Korkolis, Dimitrios Korkolis, S Korn, Sandra Korn, M Kornaszewska, Lucy Kornblith, LZ Kornblith, Grigory Korolev, Porfyrios Korompelis, N Korres, A Korthaus, Inye Korubo, E Kose, Selçuk Köse, Andrey Koshel, Z Koshnow, Rakesh Koshy, RM Koshy, J Kosir, JA Košir, T Košir Božič, Lisa Koslowski, M Kost, Ioannis Kostakis, M Kostic, Yevhenii Kostiuchenko, Albert Kota, Tuncay Kötan, Jan Kotarski, Mostafa Kotb, A Kothari, Tommi Kotkavaara, Denis Kotov, L Kottam, R Kottayasamy Seenivasagam, Rajkumar Kottayasamy Seenivasagam, W Kotyczka, Ali Kouhi, G Koukoulis, Georgios Koukoulis, O Koukoura, A Koulouktsis, Marinos Koulouroudias, Mohammed Koumu, Mandeep Koundu, Alaa Kour, K Kour, Mohammad Kour, Robindera Kour, A Kourdouli, Marinos S Kouris, V Kouritas, Vasileios Kouritas, S Koussayer, Samer Koussayer, M Koutentakis, A Kouyoumdjian, Basel Kouz, Bojan Kovacevic, M Kovačević, Petra Kovačević, Ivan Kovačić, Rok Kovačič, Shama Kovale, M Kowal, Karl-Friedrich Kowalewski, KF Kowalewski, LP Kowalski, Ana Kowark, Pascal Kowark, LP Kowaski, Youssef Kozah, R Kozan, Ramazan Kozan, Sarunas Kozenevskis, Akvilė Koženiauskaitė, Zoran Kozomara, Richard Kpangkpari, Cyrille Kpangon, Martina Kralinger, M Kranawetter, Marlene Kranawetter, Peter Kranke, Dietmar Krappinger, Peter-Martin Krarup, Virgilijus Krasauskas, C Kratochwila, Chiara Kratochwila, Johannes R Kratz, T Kratzer, A Krause, H Krause, Hardy Krause, Joanna Krawczyk, W Krawczyk, I Krdzic, Igor Krdzic, S Krejovic Trivic, Sanja Krejovic Trivic, V Kremo, Josip Kresic, Tanja Krešić, M Kresoja, Giorgos Krestinidis, D Krief, Maximilian Kriegmair, Eslam Kriem, Andrey Kriger, D Krinock, Sunil Krishna, Ashvin Krishna Nair, Bal Krishna Ojha, Mahesh Krishna Pillai, Pradeep Krishna RV, murali Krishna Voonna, Vijay Krishnamoorthy, Gargeshwari Krishnamurthy Guru Raghavendra, Bala Krishnan, E Krishnan, Emily Krishnan, Ratha Krishnan Sriram, Sivakumar Krishnasamy, Cristina Kristel Tonos Sardiñas, HØ Kristensen, Jens Kristian Bælum, S Kristinsson, Abirami Krithiga, Neoklis Kritikos, Zoran Krivokapic, Marie Kröger, Irmgard Kronberger, Joshua Kronenfeld, JP Kronenfeld, Hidde M Kroon, HM Kroon, E Kropf, I Kruger, S Kruijff, Schelto Kruijff, SH Kruijff, Jakob Kruschwitz, SureshKannan Ks, DS Kshirsagar, Antigoni Ktisti, Beatrice Kuang, E Kubiliute, Nikita Kubin, Hubert Kübler, Hisako Kubota, V Kubyshkin, Valery Kubyshkin, A Kuc, F Kucuk, GO Kucuk, S Kudchadkar, Shantata Kudchadkar, A Kudpaje, Y Kudryavcev, Søren Kudsk-Iversen, B Kuehlmann, Britta Kuehlmann, Marlene Kuen, D Kufeji, N Kugler, Magda Kujawa, Yerlan Kukubassov, K Kułak, MA V Kulcsar, Marco Kulcsar, Judit Kulcsicka-Gut, O Kuleshov, Justas Kuliavas, Mukhtar Kulimbet, Tomislav Kulis, Amol Kulkarni, Amruta Kulkarni, Avadhut Kulkarni, G Kulkarni, Gauri Kulkarni, Nikhil Kulkarni, R Kulkarni, Rugved Kulkarni, Yogesh Kulkarni, A Kumar, Abhaya Kumar, Abhinav Kumar, Ambrish Kumar, Anil Kumar, Aruna Kumar, Arvind Kumar, Ashwani Kumar, J Kumar, Kranthi Kumar, L Kumar, Manoj Kumar, Mohit Kumar, Naren Kumar, Navin Kumar, Navneet Kumar, Neha Kumar, Pankaj Kumar, Ravi Kumar, Rohit Kumar, S Kumar, Shashank Kumar, Subodh Kumar, Upander Kumar, V Kumar, Vijay Kumar, Vishal Kumar, Sanjit Kumar Agrawal, Akshay Kumar Bisoi, Navneet Kumar Chaudhry, Pawan Kumar Dhruva Rao, Surya Kumar Dube, Pankaj Kumar Garg, Sunil Kumar Gupta, Anoop kumar Jaiswal, Saubhagya Kumar Jena, Vijay Kumar Kumar, Barani kumar P B Pb, Piravin Kumar Ramakrishnan, Santhosh kumar Sampengere Annayappa, Amit kumar Shrivastava, Piyush Kumar Sinha, Santhosh Kumar Thangaraj, Virendra Kumar Tiwari, S Kumar Venkatappa, Sunil Kumar Venkatappa, Sunil kumar Vishwakarma, Sumudu Kumarage, NK Kumaran, Kanesh Kumaran Seevalingam, M Kumari, Pushplata Kumari, Sameeta Kumari, Sujatha Kumari, Philemon Kumassah, Kashmira Kumawat, Sean Kumer, Sho Kumita, Felix Kumolalo, JD Kün-Darbois, Jean-Daniel Kün-Darbois, Rastislav Kunda, R Kundra, Rakesh Kundra, Nikhil Kundu, Kristian Kunjko, Joseph Kunju Mathew, Yunus Kuntawi Aji, Priyanka Kunte, Isabella Kuo, Louise Kuo, Stephen Kuo, Bruna Kupper, Mohammed Kura, DA B Kuramoto, N Kuratani, Norifumi Kuratani, Musbahu Kurawa, W Kurdi, H Kurihara, A Kuriyama, Akira Kuriyama, Andrii Kurmanskyi, Juni Kurniawaty, Kento Kuroda, N Kuroda, Naoto Kuroda, Cameron Kuronen-Stewart, M Kurtenkov, Mikhail Kurtenkov, Michael Kurtz, A Kushairi, Tetsuya Kushikata, Kwasi Kusi, Sidharta Kusuma Manggala, A Kut, Mariya Kuteva, J Kutkevicius, E Kutlu Yalcin, Kudzayi Kutywayo, Ekins Kuuzie, Vsevolod Kuzkov, C Kuzmanovic, A Kuzovlev, Shigeki Kuzuhara, Aswathi Kv, Nana Kwaku Agyeman-Duah, A Kwan, R Kwan-Feinberg, Daniel Kwesi Acquah, A Kwiatkowski, Patrick Kwizera, AM F Kwok, Kelvin Kwok, M Kwok, Stephanie Kwok, George Kwok Chu Wong, Kelvin Kwok-Chai Ng, David Kwon, Audrey Kwong, Jin Kwun, Ishmael Kyei, S Kykalos, Stylianos Kykalos, Matthew Kynes, Harry Kyriacou, Ioannis Kyritsis, E Kyrodimos, Efthymios Kyrodimos, Eleandros Kyros, Ioanna Kyrou, V Kyvelos, Vasilis Kyvelos, M Kyzer, Badareesh L, Sharathkumarkl L, D L. Cruz Condori, A L. Minussi, E La Corte, Emanuele La Corte, Antonio La Greca, Roberta La Mendola, Carlotta La Raja, D La Regina, Stefania La Rocca, F La Torre, Filippo La Torre, M La Torre, M Labalde Martinez, Simon Laban, Fernando Labarga Rodríguez, Ivan Labetov, A Labib, PL Labib, Nicole Labine, Nathalie Labrecque, Domenico Lacavalla, Vasileios Lachanas, Tunc Lacin, AM Lacy, Kaylan Lad, P Lad, Parag Lad, Vidyadhar Lad, MR Ladd, Karim Ladha, Oluwaseun Ladipo-Ajayi, Roland Ladurner, T Laeke, Tsegazeab Laeke, L Laface, Letizia Laface, George Lafford, Yasser Lafi, Anaïs Laforest, R Laforgia, AS Laganà, A Lagares, Daniella Laguado, Gregorio Laguna, Emilio Lagunas Lostao, K Lah, Raghad Lahlooh, W Lahlou, M Lahmar, Zoe Lahood, A Lahoud-Velaochaga, Jack Lahy, S Lai, Alexandros Laios, A Laird, Alexander Laird, A Lakehal, Aarti Lakhiani, K Lakhoo, Z Lakkis, Zaher Lakkis, S Lakpriya, Sathya Lakpriya, Prashant Lakshman, Antoine Laktine, Nikhil Lal, Ashish Lal Shrestha, AK Lala, M Lallemand, Lara Lallitsch, Ismahene Lalmi, A Lalos, J Laloze, Jerome Laloze, Andrew Lam, Dominic Lam, K Lam, Susanna Lam, YH Lam, B Lamb, Anton Lambers, Leo Lambers, J Lambert, Virginia Lambert, Antoine Lamblin, M Lami, Mariam Lami, R Lamm, S Lammy, Simon Lammy, P Lamoral, C Lampert, Valerie Lan-Pak-Kee, L Lancerotto, Luca Lancerotto, Antonio Lanci Lanci, A Landaluce-Olavarria, Aitor Landaluce-Olavarria, SA Landeo Agüero, FJ Landete Molina, Giovanni Landoni, L Landoni, Giulia I Lane, J Lane, Oliver Lane, Rondall Lane, Allison T Lanfear, D Lanfranco, Elena Lang, K Langeveld, Ream Langhe, Eve-Lyne Langlais, T Langlais, Tristan Langlais, F Langlands, Fiona Langlands, A Langone, Maximilian Lanner, R Łanowy, Brent Lanting, G Lantone, L Lanuza, A Lanzone, Maria Lapeña Rodríguez, MC Lapitan, P Lapolla, Pierfrancesco Lapolla, G Laporte, Gustavo Laporte, A Lara, Antonio Lara, Arkaitz Lara, Sabino Lara, S Laraqui Hossini, I Larbah, Antonella Larcinese, A Laredj, Andreas Larentzakis, S Lario, Sandra Lario Pérez, Meghan Lark, J Larkin, JO Larkin, John Larkin, Brett Larner, Christopher J LaRocca, Audrey Larouche, Juan Larrañaga, B Larsen, David Larson, Kelsey E Larson, J Laryea, Inmaculada Lasa, Maria Laseca, Mehran Lashari, Valentina Lasić, Kingsley Lasing, Konstantinos Lasithiotakis, Parbin Laskar, D Łaski, S Lasrado, Luis Lassaletta, Bibiana Lasses Martínez, Manju Lata Verma, I Lataifeh, Edward Latif, Ejaz Latif, Haider Latif, Sehrish Latif, Usman Latif, Stojan Latinčić, Raquel Latorre Fragua, M Latorre Gómez, Raúl Latorre Tomey, Abigail Lau, Godfrey Lau, Jo Lau, Joshua Lau, K Lau, Maisie Lau, Rainbow W H Lau, RW Lau, Annamart Laubscher, Maritz Laubscher, Veronica Laudani, Johanna Laukkarinen, Jheff Laura, S Laura, Sharon Laura, Maria Laura Cossu, Maria Laura Pelegrina-Lopez, Vladimir Laureano Velasquez Huarcaya, E Laurent, R Laurente, A Lauretta, MP Lauretta, Oliver Lauridsen Siaw, B Lauritz, Brianne Lauritz, JC Lauscher, N Lavagen, Nolwenn Lavagen, Laura Lavalle, Andréane Lavallée, Alcimar lavareda dos Santos Junior Alcimar, Vincent Lavoue, R Lavy, Ron Lavy, J Law, Adedayo Lawal, B Lawal, Ishak Lawal, Jamila Lawal, T Lawal, TA Lawal, Ismail Lawani, Ismaïl Lawani, Souliath Lawani, S Lawday, R Lax Perez, Raquel Lax Perez, R Lax-Pérez, H Layard Horsfall, Hugo Layard Horsfall, GR Layton, Engels Lazala, Gabriel Lazar, Kirill Lazarev, Alexander Lazarides, A Lázaro, A Lazic, Aleksandar Lazic, E Lazova, M Lazovic, Mikan Lazovic, Stefano Lazzari, Lan-Hoa Le, P Le, T Le, Louise Le Blevec, Adrien Le Fouler, Hélène Le Gall, S Le Grange, B Le Roy, Khrissa Lea Elisa Violago, C Leal, Clara Leal, Rebeca Leal, I Leal Silva, Inês Leal Silva, Sofia Leandro, Y Leang, Jessica Leary, M Lebe, A Lechiancole, M Lechner, Michael Lechner, WK G Leclercq, Wouter Leclercq, V Lecluyse, K Lecolle, Katia Lecolle, D Lecumberri, David Lecumberri, H Lederhuber, FS Ledesma, Adelaide Lee, Alexandra Lee, Dominic Lee, G Lee, J Lee, Jonathan Lee, Ken Lee, KJ Lee, Lawrence Lee, LD Lee, Lucas D Lee, M Lee, Minna Lee, Rex H Lee, S Lee, Sharon Lee, Shawn Lee, SM Lee, Susan Lee, Vivian Lee, YM Lee, Kurt Lee Chircop, Jeremy Lee Jun Shern, Mary Leech, E Leede, Renee Leen Laudato, Christopher Leeson, S Leeson, R Lefroy, Christian Legal, Adrian Legaspi, Gerardo Legaspi, P Legeza, C Lehmann, Carlos Lehmann, Christian Leiber, James Leigh, AL S Leite, Fernanda Leite, AM Leite-Moreira, Andreas Leithner, A Leitner, Aran Leitner, JM Lemée, F Lemma, Francesco Lemma, Chris Lemos, Madeleine Lemyre, Anna Lena Huber, Catherine Leng, S Leng, M Lengauer, Rebecca Lenihan, P Lennon, Hannah Lennox-Warburton, Cumhur B Lent Urman, Neil Lenus, E Lenzi, Elisa Lenzi, Riccardo Lenzi, CA Leo, B Leoce, Brian Leoce, R Leon, Sergio Leon, ZS Leon Cabrera, Elizabeth Leon Cuevas, Ricardo León Fernández, LF Leon Giron, Eduardo Leon Llanos, Muyenzi Leon Ngeruka, JL León Palacios, MA Leon Valarezo, Gustavo León Vizcaya, Alejandro Leon-Andrino, Carlos León-Espinoza, Vicente J León-Muñoz, Julia Leonard, L Leonard, Laura Leonard, Camillo Leonardo Bertoglio, Lorenzo Leonelli, CH Leong, D Leong, F Leong, M Leongito, Elena Leonor Delgado-Nieto, L Leotta, Jeffrey J Leow, P Lepiane, Dominic Lepiorz, Milan Lerch, M Lerchenberger, Ricardo Lerma, P Lerut, A Letaief, Noelia Lete Aguirre, Ludvig Letica, I Leto, Ahmed Letrache, Andraay Leung, Catherine Leung, Elaine Leung, EY L Leung, Philemon Leung, S Leung, Sebastian Leuschner, Andrea Leva, S Leventoğlu, JH Levin, Meta Levstek, Yael Levy-Zauberman, Chen Lew, PS Lew, Francesca Lewis, Owen Lewis, SE Lewis, C Lewis-Lloyd, Christopher Lewis-Lloyd, R Lewit, Ruth A Lewit, A Leyte, Antonio Leyte Golpe, Francisco Leyva Rodríguez, M Leziak, Sara Lhassani, Demin Li, Lucy Li, R Li, Ryle Li, S Li, Z Li, Zoe Li, Chin Li Tee, MR Li Valencia, T Liakakos, Hsin-Ping Liang, Ina Liang, Kaifeng Liang, Tingbo Liang, Y Liang, J Liaño, Julian Liaño, CC L Liao, SC Liapis, S Liau, Siong-Seng Liau, M Liberati, AS Liberman, Tatiana Liborio-Kimura, L Licari, Leo Licari, Anthony Lichaa, RD Licona-Meníndez, O Liczbik, S Lidder, Surjit Lidder, A Liddle, Samuel Lie, W Lie, Warren Lie, Michael Liebensteiner, T Liebs, Amanda Liesegang, B Lieske, I Liew, Ignatius Liew, JC Lifante, Nicholas Lightfoot, Anne-Louise Lihn, V Likhvantsev, Abu Lil, K Lilaj, Nzabamwita Liliane, J Lilienstein, C Lillo, Cristina Lillo García, Alicia Lim, Chetana Lim, D Lim, Daniel Lim, Eric Lim, IM Lim, Ivy Lim, JA Lim, Jeffery ZK Lim, K Lim, Matthew Lim, Seantee Lim, Y Lim, ALM Lima, Leonardo Lima, I Lima Buarque, Igor Lima Buarque, Leticia Lima da Cruz, Catarina Lima da Silva, Susana Lima Oliveira, C Lima-da-Silva, ZM Limalia, Haruna Liman, RK Liman, A Lin, DJ Lin, JF Lin, Peter Lin, Rosalina Lin, C Linari, Eddy Lincango, EP Lincango, E Lincango-Naranjo, Eddy Lincango-Naranjo, Karin Lind, Andrew Lindberg, J Lindenmann, Joerg Lindenmann, Cortland Linder, G Linder, Gustav Linder, J Lindert, Judith Lindert, Ebba K Lindqvist, EK Lindqvist, Elizabeth Lindsay, Wee Ling Koh, Yii Ling Lau, Eu Ling Neo, Erika Linmey Tay Lasso, Rui Lino, Kenneth Linton, VC Linz, Paris Liokatis, C Lionel, Charre Lionel, Ruggero Lionetti, C Lipede, Christina Lipede, L Lippa, A Lira dos Santos Leite, Amanda Lira dos Santos Leite, Anna Lisa Pesce, Joana Lisboa, Robert Lischke, C Lisencu, Cosmin Lisencu, G Lisi, Giorgio Lisi, A Litchinko, F Litta, Francesco Litta, Max Little, Joe Littlechild, A Litvin, Andrey Litvin, Yauheniya Litvina, Biquan Liu, David Liu, H Liu, L Liu, Shirley Liu, T Liu, A Liveris, Anna Liveris, Marie Livin, Charles Livingston, Lorenzo Livraghi, João Lixa, AS D Liyanage, Pituwala Liyanage Adithya Sirisena, Juan Liyo, María Liz Sánchez, Aintzane Lizarazu, JA Lizarbe, Diana Lizet Cruz Condori, G Lizzetti, Grecia Lizzetti, G Lizzetti-Mendoza, V Lizzi, Vincenzo Lizzi, F Llahi, Florencia Llahi, Eduardo Llamazares Cobo, Alice Llambias-Maw, Tamara Llamero, Oscar Llanes, L Llano, Lionel Llano, H Llaquet Bayo, Heura Llaquet Bayo, CA Llerena Ojeda, Juan Lliteras Jorge, Alyssa Llorando, Almudena Llorente, IC Lloyd, W Lloyd, William Lloyd, T Lo, Terence Lo, Arturo Lo Giudice, Yuri Loaiza, A Loayza, Niklas Löbig, Pablo Lobos, Dmitrijs Lobovs, Andrea Locatelli, LG Locatello, Florian N Loch, FN Loch, Johan Lock, Jessica Lockhart, S Lodhi, S Lodhia, Andrew Loehrer, Markus Loffler, Markus W Löffler, MW Löffler, Chiara Loffredo, N Löfgren, Niklas Löfgren, Anitha Loganathan, K Logishetty, C Loh, Christopher Loh, R Lohia, Rajan Lohia, V Lohsiriwat, Varut Lohsiriwat, Wolfgang Loidl, Evania Lok, Siddharth Lokanathan, Lokesh Lokesh, U Lokman, CP Lombardi, Gaetano Lombardi, Raffaele Lombardi, Cristina Lombardia Gonzalez de Lera, D Lomiento, Daniele Lomiento, Z Loncar, Zlatibor Loncar, German Londono, Eduardo Londono-Schimmer, Rubina Lone, C Long, Emma Long, Samuel Long, M Longhi, A Longhini, Alessandro Longhini, L Longstaff, MW Löoffler, C Lopes, L Lopes, LM Lopes, Luciana Lopes, Ariela Lopez, F Lopez, Fernando Lopez, M Lopez, Manuel Lopez, MP Lopez, MP J Lopez, Raquel Lopez, Angie López, I López, Iker López, María López, Emilio Lopez Alcina, L Lopez Antoñanzas, Leyre López Antoñanzas, Aldo Lopez Blanco, A López Campillo, Adrián López Campillo, A López De Fernández, Alina López De Fernández, CA López de la Manzanara Cano, Clara López de Lerma Martínez de Carneros, Sonia Lopez Flores, Isabel López García, Patricia Lopez Gomez, Ruth Lopez Gonzalez, Pablo López Martínez, I Lopez Muralles, Ismar Lopez Muralles, Maria Lopez pais, Francisco López Rodríguez -Arias, I López Sánchez, Isabel López Sánchez, C López Viloria, M López-Baamonde, Santiago Lopez-Ben, Javier López-Martin, G Lopez-Pena, Gabriel Lopez-Pena, Enrique López-Ruiz, Jaime López-Sánchez, Pilar López-Toribio López, P Lora-Cumplido, J Lord, Julia Lord, Dalila Loredana Lo Bue, Charmagne Loren Ramos, Z Lorenc, M Lorencin, Mia Lorencin, Kerstin Lorenz, Cara Lorenzi, A Lorinc, Fabrizio Lorusso, M Losada, Manuel Losada, E Lostis, E Lostoridis, Eftychios Lostoridis, B Lotfi, N Lott, Natalie Lott, Lawrence Lottenberg, Marco Lotti, Christopher Lotz, Wenhui Lou, Amina Louari, M Loubani, Mahmoud Loubani, VR Louçao Prada, Mauricio Loucel Bellino, S Louette, Stefan Louette, P Loufopoulos, Antony Louis Rex Michael, Sina- Louisa Patrizia Jentschura, Hannah Louise Morley, Petros Loukas Chalkias, SM Louraoui, María Lourdes Ramos, I Lourenço, S Lourenço, Juliana Lourenço da Silva, Carolina Lourenço Gomes dos Santos, L Loutzidou, Lydia Loutzidou, A Louvrier, A Lovece, Andrea Lovece, Amy Lovett, Agbenya Lovi, Maria Lovisa Jönsson, Dana Low, Y Low, Yee Low, AJ Lowery, Aoife Lowery, Megan Lowey, MJ Lowey, A Lowy, Christian Lozano, Santiago Lozano Calderon, P Lozano Lominchar, Pablo Lozano Lominchar, H Lu, Htoo Lu, A Luberto, Gian Luca Baiocchi, Roberto Luca Meniconi, Marie Lucas, Marco Lucchi, SM Lucchini, Stefano Lucchini, Carmen Lucero León Gámez, Ana Lucia Lemus, Ana Lucia Munhoz Lima, Ana Lucía Portilla, Ana Lúcia Preto Barreira, A Lucianetti, C Luciani, Antonio Luciano Sarni, J Luck, Tara Luck, R Luckwell, Rhys Luckwell, J Lucocq, Isabella Ludbrook, Stephanie Lueckel, Laurene Lugans, Ferdinand Luger, Matthias Luger, Gaetano Luglio, Carolina Lugo Duarte, GE Lugo Zamudio, Ignacio Lugones, Sumant Luhana, Stefan Luhne, Pier Luigi Filosso, Emanuele Luigi Giuseppe Asti, M Luis, Angel Luis Agüero Delgado, Juan Luis Blas Laina, Jorge Luis Bustamante Polo, José Luis Castillo, José Luis DAddino, Rene Luis Filarca, Victor luis Gomez corujo, Jorge Luis Gomez-Mayorga, Jose Luis Lucena De La Poza, Jose Luis Muñoz de Nova, Jose Luis Rabago, Jose Luis Ramos Rodriguez, Maria Luís Sacras, Ana Luis Siles, Jose Luis Uquillas, Jorge Luis Velez Bernal, Maris Luisa García-Pérez, Maria Luisa Gasparri, Maria Luisa Reyes Diaz, Matteo Luisetto, Florentina Luiza Popescu, Maria Luiza Rocha, AC O Luk, M Lukaszewski, Claudine Lukban, L Luke, L Luketic, Lea Luketic, Alexandr Lukianov, Dejan Lukic, B Lukić, Lara Lukman, Sumadi Lukman Anwar, L Lukoko, Ausra Lukosiute-Urboniene, I Luksic, Ivica Luksic, Pwaluke Luku, H Lule, Herman Lule, Joann Lum, David Lumenta, DB Lumenta, Olivia Lumiap Serevina, EWY Lun, Joaquin Luna, S Lunca, Sorinel Lunca, R Lunevicius, Raimundas Lunevicius, C Luney, Catriona Luney, EG Lunghi, A Luo, Haili Luo, L Luo, Weisang Luo, X Luo, V Luoma, AI Lupián-Angulo, L Luques, Victor Luraschi, O Lusawana, WG Lustre, Anil Luther, Charlotte Luths, Nicholas Lutton, Martha Luz Torres, A Luzzi, Emilia Luzzi, Jasen Ly, M Ly, Victor Ly, Marie-Louise Lydrup, G Lye, Julie Lykke Harbjerg, P Lykoudis, Holly Lyle, Michelle Lynch, Anders Lyng Ebbehøj, Grace Lynn Estanislao, Louie Lynn Sajorda, Maria Lyons, O Lyons, Oliver Lyons, D Lytras, Alexei Lyzikov, Turki M Alzaidi, Jose M Barrio, Ahmed M Chaoui, Rosa M Jimenez-Rodriguez, Jose M Jover, Mahmoud M Mohammed, S M Prasad, Mashitha M S, Josep M Sole-Sedeno, Damien M Wu, Jolande Ma, Justin Ma, Tao Ma, Vanessa Ma, Sara Maa Albared, R Maala, Ghina Maarawi, A Maashi, Marah Maayah, María Mabel Collado Expósito, RM Mabeza, Waleed Mabood, Findlay MacAskill, T Maccabe, Giuseppe Maccagnano, A Macchi, Alberto Macchi, Roberto Macchiavello, Andrew MacCormick, Hamish Macdonald, L MacDonald, Luisa MacDonald, Taigh Macdonald, Nicole Macečková, Alexandre Macedo, DA Macedo Falcon, P Macek, Petr Macek, A Machado, Daniela Machado, Francisco Machado, Luís Machado, N Machado, Nuno Machado, Vinicius Machado, N Machairas, Nikolaos Machairas, Camila Machareth, V Machatsch, Solomon Machemedze, Marvellous Machiri, J Maciel, Alasdair MacInnes, Emma MacInnes, Dillon MacIntyre, Nicola Mackay, Sean Mackay, TG Mackay, K MacKenzie, S MacKenzie, Shawn MacKenzie, T MacKinnon, Anthony MacLean, Catherine Macleod-Hall, Brooke Macnab, S Danielle MacNeil, Mahmoud Macshut, I Madabhavi, Irappa Madabhavi, S Madan, Omar Madani, R Madani, Saeed Madani, A Madden, Vijay Madduri, I Made Gede Widnyana, Galia Maderi, Devdas Madhavan, TK Madhuri, K Madhvani, Kiran Madhvani, Suyog Madje, Azza Madkhali, T Madkhali, Tariq Madkhali, Abdul Madni, Massimo Madonia, M Madonini, Constanza Madrid, Andrea Madrigrano, Helen Madsen, Doris Mae Dimatatac, Liana Mae Lobo, Junichi Maeda, Y Maeda, Yasuko Maeda, Mohammad Maen Ghannam, Federica Maffeis, A Maffert, Alexis Maffert, A Maffioli, Anna Maffioli, A Maffuz-Aziz, Antonio Maffuz-Aziz, C Magadán Álvarez, Ana Magalhães, M Magalhães Maia, Mariana Magalhães Maia, Catalin Magan, Nasir Magboul, María Magdalena Vásquez Sánchez, Ammar Magdy, C Magee, Astrid Magele, Moses Magezi, G Maggiore, SM Maggiore, Tamara Maghathe, Ashraf Maghrabi, Knut Magne Augestad, Stefano Magnone, V Mago, Abdula Magomedaliev, Dimitrios Magouliotis, D Magowan, Nikko Magsanoc, Barry Maguire, D Maguire, PJ Maguire, R Maguire, N Mahabbat, Nehal Mahabbat, Amit Mahajan, Anupam Mahajan, Dhruv Mahajan, C Mahakalkar, Chandrashekhar Mahakalkar, Praveena Mahalingam, Talanayar Mahalingam, Jehangir Mahaluxmivala, Ishtiak Mahamud, Subramanyam Mahankali, AK Mahar, H Mahdi, Maryam Mahdi, Shareef Mahdi, Ameerah Mahdi Abraheem Hasan, B Mahendran, V Mahendran, M Maher, Mark Maher, Natalie Maher, M Maher Al arje, Sarah Maheux-Lacroix, Moufid Mahfoud, Abobakr Mahfouz, Arwa Mahfouz, Mehmet Mahir Ozmen, Ansar Mahmood, Arif Mahmood, Ashraf Mahmood, DN Mahmood, Farrukh Mahmood, Namrah Mahmood, S Mahmood, U Mahmood, Usama Mahmood, Z Mahmood, A Mahmoud, Ahmed Mahmoud, Fedy Mahmoud, Nada Mahmoud, Osman Mahmoud, Safa Mahmoud, Y Mahmoud, Alaa Mahmoud Abo shabana, Balqees Mahmoud Al-Manaseer, F Mahmoud Ali, Ayesha Mahmud, Zayne Mahmud-Ahmad, Taher Mahnashi, Ebrahim Mahomed, Wasim Mahomed, J Mahon, Freyia Mahon-Daly, R Mahoney, Wesam Mahran, Doha Mahrous, Mert Mahsuni Sevinc, K Mahuli, DA Mahvi, David Mahvi, P Maida, Pietro Maida, F Maiello, A Maiga, JM Maillet, Betty Maillot, Claire Mailu, Mayaba Maimbo, Miriam Maimbo, B Main, Grace Maina, J Maines, F Maione, Pasquale Maiorano, Charikleia Maiou, M Mair, Montserrat Mairal Fraile, Siobhan Mairead Rooney, Patrick Maison, E Maisonneuve, Emeline Maisonneuve, A Maity, Vincenzo Maiuri, M Maiza, Omar Majadla, AM Majbar, MA Majbar, Faizan Majeed, Zubair Majeed, Suvendu Maji, I Majid, Ibrar Majid, Sabeen Majid, A Majkowska, Agata Majkowska, L Majkowski, Hardil Majmudar, P Major, Piotr Major, S Majrashi, Brian Mak, JK C Mak, Josephine Mak, Baje Makama, Ronald Makanda, Ayomide Makanjuola, Fabiana Makdissi, FB Makdissi, C Makepeace, R Makin-Taylor, Jun Makino, Raghad Makki, Abdelrahman M Makram, Amany Makroum, Bogdan Maksymenko, Florian Maksymiw, K Maktabi, CC Makwe, Christian Makwe, A Malan, Asha Malan, H Malapati, Harsha Malapati, O Malard, M Malavolta, FL Malcolm, FH R Maldonado, A Maldonado Del Arenal, Meredid Maldonado Santiago, Yeray Maldonado Sotoca, Eloy Maldonado-Marcos, Y Maldonado-Sotoca, Almantas Maleckas, Ans Malek, M Malerba, Michele Malerba, Albert Malet Contreras, Leeany Maletta Francisco, Igor Maleyko, Maulen Malgazhdarov, B Malgras, Brice Malgras, Rajesh Malhotra, N Malibary, Nadim Malibary, Ziad Malibary, Aamer Malik, AR Malik, Boulaadas Malik, Kapil Malik, Kiren Malik, Komail Malik, Mariam Malik, MR Malik, MS Malik, Shahbaz S Malik, Sobia Malik, Tahir Malik, Lucy Maling, Konstantinos Malizos, J Mall, G Mallabiabarrena Ormaechea, G Malleo, Shweta Mallick, Evangelos Mallidis, C Mallmann, Christoph Mallmann, Michael R Mallmann, MR Mallmann, J Malmstedt, A Malomo, F Malone, Fergal Malone, Gianni Malossini, A Malpaga, Julius Malte Vahl, G Maltese, G Maltinti, Gherardo Maltinti, Luca Malvezzi, S Mambrilla, Sara Mambrilla, Nora Mamdouh, Zaman Mamedli, M Mamic, Matija Mamic, Rakotonaivo Mamisoa Judicaël, Mickyas Mamo, A Mamopoulos, Apostolos Mamopoulos, Sidi Mamoun Louraoui, Chi Man Tom Chow, Enrique Manalang, Mallikarjuna Manangi, J Manara, Dimitrios K Manatakis, DK Manatakis, G Manca, Gilles Manceau, G Mancebo, Gemma Mancebo, MM Manchego De La Cruz, Marta Mancheño, R Mancini, S Mancini, Roberto Mancino, Valentina Manciocco, VD Mandato, V Mandic Markovic, Facundo Mandojana, K Mandrelle, Kavita Mandrelle, RP Manecksha, Rustom Manecksha, Steffen Manekeller, S Manfredelli, Alfonso Manfuso, Ahmed Mangahy, MS Mangano, Aneish Mangarai, V Mangaroliya, Moses Mangena, Ali Mangi, S Manglik, E Mangos, J Mangwani, Jitendra Mangwani, J Manickavasagam, Jaiganesh Manickavasagam, M Manigrasso, P Manikis, N Manimaran, EA Manioti, P Maniscalco, Pietro Maniscalco, Saptak Mankad, R Manley, H Mann, Harvinder Mann, A Mannan, S Mannan, Syed Mannan, Aidan Manning, D Manning, Debra Manning, R Mannion, David Männle, I Mannoh, Ivy Mannoh, T Manogaran, L Manoj Joshua, Lokavarapu Manoj joshua, Veronica Manolache, I Manolitsis, F Manresa-Manresa, Francisco Manresa-Manresa, E Manrique, Susana Manrique, GC Manrique Sila, P Mansilla Doria, Percy Mansilla Doria, MI Manso, D Manson, J Manson, Joanna Manson, Maheen Mansoor, Aisha Mansoor Ali, A Mansour, Ahmed Mansour, Atef Mansour, F Mansour, Omar Mansour, Nayef Mansour Alshammari, J Mansouri, M Mansouri, A Mansuri, Ahmed Mansy, A Mantevas, Antonis Mantevas, Henrique Mantoan, B Mantoglu, Baris Mantoglu, J Mantri, N Manu, Nichola Manu, Meshach Manu Agyapong, Juan Manuel Baez Melgarejo, Vicente Manuel Borrego Estella, Jiomar Manuel Figueroa Germosen, Jose Manuel Luna Vazquez, Florencio Manuel Marin Martinez, Juan Manuel Martos Martinez, José Manuel Morales-Puebla, José Manuel Muñoz Camarena, Joaquin Manuel Muñoz Rodríguez, Jose Manuel Rabanal, H Many, Lisa Manzenreiter, Elena Manzo, S Manzoor, Shahneela Manzoor, Sobia Manzoor, Partson Maphosa, Natalie Maple, Sabreen Maqbol, F Maqboul, A Maqsood, Fauzia Maqsood, Rocio Maqueda, R Maqueda González, A Maqus, C Marafante, Chiara Marafante, A Marano, Alessandra Marano, L Marano, Luigi Marano, Salvatore Marano, Dimitris Maras, André Marçal, Brenda Marcela Coll Tello, Josie Marcelle Lira Albuquerque, Juan Marcelo Delgado Godoy, Juan Marcelo Portillo, A Marchbank, Benedikt Marche, G Marchegiani, Paolo Marchesi, Claudio Marchetti, V Marchionini, Valentina Marchionini, Arnold Marchis, Alfonso Marco Garrido, Gian Marco Prucher, A Marco-Garrido, S Marcos Contreras, Sergio Marcos Contreras, P Marcos-Santos, Pablo Marcos-Santos, H Marcus, Hani Marcus, HJ Marcus, M Mardare, Mara Mardare, Hiba Mardini, MMA Marei, A Marello, P Maremonti, Pietro Maremonti, Gianluca Maresca, A Margalit, Adam Margalit, Nevo Margalit, Concetta Marganella, Ana Margarida Cinza, Ana Margarida Correia, Lavinia Margarit, Chrysoula Margioula-Siarkou, Joseph Margolick, G Mari, Syeda Maria Ahmad Zaidi, Marta María Arroyo Domingo, Anna Maria Baietti, Jose Maria Barreto Angulo, Monica Maria Bejasa, Francesco Maria Carrano, Luz Maria Cespedes Ramirex, Pierfranco Maria Cicerchia, Davide Maria Donati, Virginia María Durán Muñoz-Cruzado, Sandra Maria Gadin-Lopez, José María Gallego Sánchez, José María García Pérez, Agustín Maria García-Mansilla, Angela Maria Giraldo Velasquez, Monica Maria Gomes-da-Silva, Ana María Grande-Gil, Ursula María Jariod Ferrer, Pietro Maria Lombardi, Jose Maria Lopesino, Carmen maria Lopez Lopez, Margarita Maria Maldonado, Tommaso Maria Manzia, Ana Maria Marin Gonzalez, Jose Maria Martinez-de-la-Casa, José María Martínez-Gómiz, Stefano Maria Massimiliano Basso, José María Matilla, Vincenzo Maria Mazzaferro, Luis María Merino Peñacoba, Ana Maria Minaya Bravo, Luz María Moratalla Charcos, Jose Maria Muguerza, Josep Maria Muñoz Vives, Carlo Maria Neri, Jose Maria Nieto Rodriguez, Anna Maria Ntziovara, Alma Maria Ojeda Rojas, Ana Maria Parada Rodriguez, Marco Maria Pascale, Giacomo Maria Pirola, Silvana Maria Quintana, Francesco maria Romano, Thilo Maria Schulte, Jose Maria Silva Barandiaran, Ana Maria Simono Charadan, Lina María Trujillo, Iliana Maria Valdes-Duque, Ana María Vargas Patiño, Lina Maria Vergara Galliadi, Lina Maria Villegas, J Marialva, Joana Marialva, Laura Mariangela Castellano, Alessandro Mariani, NM Mariani, P Mariani-Kurdjian, Kalyani Mariapan, Leo Maric, M Maric, Marjan Maric, Maximiliano Maricic, Hatem Marie, Joan Marie Flor, Alphonse Marie Sibomana, Lysa Marie-Macron, Anna Mariel Torio, Melissa Marien, Gabriel Marin, H Marin, Patrick Marin, C Marín, H Marín, Héctor Marín, Severiano Marin Bertolin, Luis Marin de Amesti, Glenda Marina Falcon Pacheco, Hajamihamina Marina Parfaite Randriantsoa, Sotirios Marinakis, Srdjan Marinković, F Marino, Fabio Marino, MV Marino, Ivan Mariño, L Marino Cosentino, Luigi Marino Cosentino, Daniel Mario Chircop, Antonio Mario Scanu, Herjean Marion, Giovanni Mariscalco, Elva Marita Sarte, Puscas Marius-Emil, Marcel Marjanović Kavanagh, Fares Marji, Vasanth Mark Samuel, Ormond Mark Taylor, D Markaryan, Daniil Markaryan, Naveed Markhand, G Markose, Pavel Markov, Duska Markov-Glavas, Ivan Markovic, Velimir Markovic, Frane Markulić, Nici Markus Dreger, Cynthia Marlene Rodríguez Sosa, Derek Marlor, Neale Marlow, W Marlow, Marilena Marmiere, Elizabeth Marmol, Urska Marolt, G Marom, Gad Marom, Georgia Maroske, Elena Marotta, Ioannis Maroulis, M Marques, N Marques, Narimã Marques, Prescillia Marques, Rita Marques, TM D M Marques, Tomas Marques, M Marqueta De Salas, Maria Marqueta De Salas, L Marquez, Lucila Marquez, Ruth Marquina González, AA Marra, E Marra, Ester Marra, E Marrano, A Marreiro, M Marro, Matteo Marro, Miguel-Angel Marroquin-Alpirez, G Marruzzo, Giovanni Marruzzo, Imke Marsch, C Marsh, Calista Marshall, Rachel Marshall-Roberts, Matthew Marshall-Webb, Abadeer Marsis, Ben Marson, Ella J Marson, FAL Marson, Maria Marta Modolo, G Marte, Gianpaolo Marte, Andre Martel, Sophie Martellotto, Jacopo Martellucci, M Martens, T Martens, C Marti, Rosa Martí Fernández, A Martin, Alexander Martin, B Martin, Benjamin Martin, E Martin, Emmeline Martin, G Martin, J Martin, Janet Martin, L Martin, Louis Martin, M Martin, Mercedes Martin, Niels Martin, RC G Martin, Rhona Martin, S Martin, Sean T Martin, Sergio Martin, Silvia Martin, Fernández Martín, EJ Martin Antona, Belén Martin Arnau, JC Martín del Olmo, Pablo Martín García, Elio Martín Gutiérrez, Sergio Martin Lucchini, Javier Martín Monterrubio, P Martin Playa, Pilar Martín Rodrigo, Luis Martín Rodríguez Ortegón, MB Martín Salamanca, M Martín Sánchez, O Martin Sole, Oriol Martin Sole, L Martin-Albo Caballero, Cristobalina Martin-Garcia, R Martín-Láez, Rubén Martín-Láez, E Martin-Perez, Elena Martin-Perez, O Martin-Sole, L Martinek, Lubomir Martinek, F Martinelli, Fabio Martinelli, G Martines, Gennaro Martines, Alejandra Martinez, L Martinez, Laura Martinez, MJ Martinez, A Martínez, Francisco Martínez, MJ Martínez, Walter Martínez, JA Martínez Alonso, Sara Martínez Castro, A Martínez de Aragón, Charo Martínez García, Jairo Martinez Garrido, Yaiza Martinez Lahoz, MJ Martinez Lara, Fernando Martinez lascano, Teresa Martínez Marivela, CM Martinez Moreno, Gonzalo Martinez Municio, Victoria Martínez Muñoz, P Martinez Pascual, Paula Martinez Pascual, C Martínez Pérez, L Martinez Perez Maldonado, MJ Martinez Velázquez, J J Martínez Zarate, JA Martínez-Alonso, AM Martinez-Blanco, Lucía Martínez-Costa, A Martinez-German, JM Martínez-Gómiz, E Martínez-Hurtado, Sara Martínez-Núñez, Irene Martínez-Padilla, Carolina Martinez-Perez, C Martínez-Pinedo, Carlos Martínez-Pinedo, Hector Martinez-Said, Emmanuel Martinod, Vlatka Martinovic, Bardi Martins, D Martins, Daniela Martins, Paulo Martins, PN Martins, R Martins, Rita Martins, RS Martins, Ruben Martins, G Martins dos Santos, Gildasio Martins dos Santos, Daniel Martins Jordão, Mafalda Martins Sousa, A Marton, P Martorell, Edgar Martos, Nahom Maru, Diana Marujo, D Marujo Henriques, Jeremy Marume, H Marwan, Hisham Marwan, W Marx, William Marx, G Maryan, F Marzi, Maini Marzia Isabella, Natalie Marzouqa, C Mas, R Mas Melendez, Robinson Mas Melendez, Mohammad Masaarane, Shah Masabat Saleem, Nawar Masarani, Mona masaud Amro Alazabi, A Masciandaro, Antonio Masciandaro, Fabrizio Masciello, S Masdoos, H Mase, Michele Masetti, Javeria Mashal, Sarah Mashaly, A Mashat, H Mashbari, Hassan Mashbari, D Masheka, Yehia Mashhadany, L Mashhadi, Mohamed Mashhour, Sonia Masih, Namrata Maskara, Sushil Maslekar, MS Masood, Rumaisa Masood, Khalid Masood Gondal, M Maspero, Joseph Masri, Noor Masri, R Masri, Ruqaya Masri, M Masrur, Anas Massad, C Massaguer, Clara Massaguer, Marco Massani, Dimitrios Massaras, Olindo Massarelli, Simonetta Massaron, M Masse, P Masseria, Benjamin Massey, L Massey, Lisa Massey, E Massie, A Massobrio, Andrea Massobrio, J Massoud, Erfan Massri, Domenico Massullo, L Masterson, Liam Masterson, Aikaterini Mastoraki, S Mastoridis, Sotiris Mastoridis, M Mastrangelo, Francesco Mastriale, F Mastrilli, V Mastrofilippo, Valentina Mastrofilippo, Gustavo Mastroianni, R Mastroianni, Riccardo Mastroianni, Manuela Mastronardi, A Mastrosimone, Alexandra Mata, J Mata, Javier Mata, Juan Mata Gutierrez, Anil Matai, Luka Matak, Oksana Matanova, Maisoon Matareed, Paloma Maté Mate, Sergiu Matei, Mirian Mateo De La Cruz, O Mateo-Sierra, Olga Mateo-Sierra, Elvira Mateos Alvarez, Blanca Mateos-Serrano, Nikhil Math, Erwin Mathew, John Mathew, Ryan Mathew, S Mathew, Stanley Mathew, Shefin Mathews, Neil Mathias, D Mathieu, G Mathieu, P Mathieu, Mariana Matias, B Matías-García, Belen Matías-García, Slavko Matić, Christie Mato, P Mato, David Mato Mañas, Maxime Maton, Leandro Matos, LL Matos, P Matos Costa, Paulo Matos Costa, Faisel Matoug, Petr Matousek, Paul Matovu, Derek Matsika, Fedra Matsouka, Shuko Matsuda, Ryota Matsuki, Yuka Matsuki, Hironori Matsumoto, Takashi Matsumoto, Kiichiro Matsumura, Susumu Matsushime, Daniele Matta, Kavitha Mattam, Ahmed Mattar, Rafif Mattar, Ilaria Mattavelli, L Mattei, Gianluca Matteo Sampietro, Jacopo Matteucci, Catherine Mattevi, KT Matthew Seah, W Matthews, S Matthiess, Gioacchino Mattisi, Jordan Mattson, M Matuszczak, M Matute-Najarro, Julie Mauger, Rebecca Maunsell, Amelie Maurel, LR Maurer, Andrew Maurice, Sandra Maurício, Edgar Mauricio Barrios Vidales, William Mauricio Riveros Castillo, Javier Mauricio Salgado Tovar, David Mauricio Solano Varela, José Mauro dos Santos, Eleni Mavrodimitraki, A Maw, Proud Mawere, M Mawlichanów, Ludwig Maximilian Heindl, Alexander Maximiliano Martinez-Blanco, Fraser Maxwell, R May, Amanda May Ong Vaño, H Maye, Erik Mayer, Franz Mayer, Robert Mayer, J Mayes, Julio Mayol, M Mayombo Idiata, Michael Mayombo Idiata, Thomas Mayr, K Mayson, Kelly Mayson, Tagleb Mazahreh, D Mazingi, Dennis Mazingi, Muhammad Mazketly, Eshan Mazumdar, CA Mazuret Sepulveda, V Mazzaferro, Diego Mazzatenta, Michael Mazzeffi, Carmelo Mazzeo, F Mazzola, Francesco Mazzola, Michele Mazzola, Erica Mazzotta, F Mazzotti, Ndubuisi Mbajiekwe, Ronald Mbiine, B Mbwele, Andrea Mc Carthy, Santiago Mc Loughlin, Stephen McAleer, Ian McAllister, Peter McAnena, B McAree, G McCabe, Olivia McCabe-Robinson, C McCaffer, A McCanny, Damian McCartan, C McCarthy, Claire McCarthy, L McCarthy, Conor McCartney, Katie McCaughey, J McCaul, Craig D McClain, Adam McClean, Anissa McClelland, S McCluney, P Mccormick, W McCormick, Wendy McCormick, A McCranie, S McCrindle, Sarah McCrindle, Peter McCullough, Rebekah McCullough, E McDermott, Enda W McDermott, F McDermott, FD McDermott, Frank McDermott, C McDonald, Sophie McDonald, Brendan McDonnell, Jack McDonogh, K McElhinney, Kathryn McElhinney, Kevin McElvanna, K McEvoy, R McEwen, Amanda McFarlan, Jacob McGee, R McGee, Richard McGee, K McGivern, Connor McGladdery, John McGrath, JS McGrath, N McGrath, Michael McGreevy, R McGregor, Richard McGregor, Cieran McGrory, L Mcguigan, Merrill McHoney, Elysse Mcilwain, Thomas McIntire, Nick McIntosh, Stuart McIntosh, J McIntyre, Joshua McIntyre, RC McIntyre Jr, Robert McIntyre Jr, G McKay, J McKay, Joanne McKay, Joseph McKay, Siobhan McKay, Mark McKeever, James McKelvie, K McKenzie, KL McKevitt, R McKinney, Rachel McKinney, Chris McKinnon, C Mclaren, N McLarty, Jared Mclauchlan, Nicole McLaughlin, Kenneth A Mclean, Morag McLellan, Thomas McLelland, S McLennan, Lucy McLeod, M McLeod, Robert Mcleod, J McMahon, Sam McNally, Anna McNamara, John McNamara, Áine McNamee, C McNaught, John McNelis, P McNelis, Catherine McNestry, F McNicol, Rebecca McNicol, R Mcnulty, D McPartland, I McPherson, Michael McTague, Christina McVeay, J McVeigh, N Md Din, Norshamsiah Md Din, Azmi Md Nor, A Meagher, JG Meara, John G Meara, Eneyew Mebratu, Mostafa Medhat Fahmy Fahmy, Mark Medhat Mikhail, Pourya Medhati, Esther Medina, Monica Medina, L Medina Mora, Manuel Medina Pedrique, Heriberto Medina-Franco, E Medina-Manuel, S Mediratta, Yuliya Medkova, M Medone, Paloma Medrano, R Medrano Caviedes, A Meelad, Ayman Meelad, Rainer Meffert, Diane Mege, Abebe Megersa, S Meghji, S Megna, Stefano Megna, Robert Meguid, M Mehdi, Mohammad Mehdi, Mohammad Mehdizadeh, BJ Mehigan, A Mehmood, DM Mehmood, Manzer Mehmood, Maria Mehmood, G Mehra, Hassan Mehrad-Majd, Asif Mehraj, Seema Mehrotra, A Mehta, D Mehta, Sachin Mehta, A Meidany, EM Meima - van Praag, EM Meima-van Praag, Nikolaus Meindl, Cornelia Meisel, P Meister, Vivek Meiyappan, Arnaud Mejean, Dolores Mejía, Ana Mejía Casado, D Mejia De la Cruz, Dolores Mejia De la Cruz, D Mekango, Margaux Mekann Bouv-Hez, Alem Mekete, M Mekhael, Mira Mekhael, Haile Mekuria, Polina Melashenko, I Melchor Corcóstegui, J Melchor-Ruan, Javier Melchor-Ruan, S Mele, Simone Mele, H Meleiro, Hugo Meleiro, M Melendez, Antonio Melero Abellán, L Melero-Cortés, Lemi Melese, P Melgar Muñoz, Sara Melgarejo, Sara Melhem, G Melina, Shannon Melissa Chan, Ana Melissa Hilvano-Cabungcal, Karla Melissa Marchan Palma, Nensi Melissa Ruzgar, Solomon Melkamu, Andrea Melloni, C Mellor, K Mellor, Christopher Melnic, Ana Melo, Isabelle Melo da Camara, Silvio Melo torres, Gianfranco Meloni, Charlotte Melot, R Memba Ikuga, E Memişoğlu, AS Memon, K Memon, N Memos, Nikolaos Memos, Javier Mena, Jimmy Mena, CA Mena García, MD Mena Ramirez, C Menakaya, Thomas Mendel, Cláudia Mendes, Filipa Mendes, JM Mendes, Margarida Mendes, Manuel Mendez, G Mendinhos, G Mendiola, GC Mendiola, Gian Mendiola, G Mendiola Barrios, Nishantha Mendis, R Menditto, G Mendonça Ataíde Gomes, Gustavo Mendonça Ataíde Gomes, Efrain Mendoza, J Mendoza Quevedo, F Mendoza-Moreno, Fernando Mendoza-Moreno, Fabrice Menegaux, Adam Meneghetti, S Meneghini, Simona Meneghini, Francesco Menegon Tasselli, AR Menendez Mite, Alejandro Menéndez Moreno, A Meneses-Garcia, Hao Meng Yip, M Mengesha, Mengistu G Mengesha, MG Mengesha, Workineh Mengesha, Lucas Mengíbar, Alemneh Mengist, Netsanet Mengiste, Abeje Menjeta, Abel Menkir, C Menna, Cecilia Menna, A Menon, G Menon, PR Menon, Tomas Menovsky, Philip Mensah, Samuel Mensah, Antonio Meola, E Merashka, Eunice Mercado, Jhomayri Mercado, MA Mercado, Pedro Mercado, G Mercante, Giuseppe Mercante, P Mercantini, Maria Mercedes Caubet, Abel Merchan, Irfan Merchant, Richard Merchant, A Merdrignac, Aude Merdrignac, Leila Mereles Noguera, Liliana Mereu, R Merh, S Meric, S Meriç, Serhat Meriç, J Merkle, Janica Merkle, Katheryne Merlos Garcia, S Merola, M Merrakos, N Merrett, Tamas Mersich, Tamás Mersich, O Merzlikin, Mohammed Meselhy, JA Mesias Logroño, SN Mesli, Nouredin Messaoudi, Gianfranco Messina, Alex Messner, F Messner, Franka Messner, YY Metaferia, Symeon Metallidis, Panagiotis Metaxas, Gabriel Metcalf-Cuenca, M Metro, M Metwalli, Maram Metwalli, IH Metwally, Khaled Metwally, J Metzger, Jürg Metzger, Marcus Meusel, Gwenaël Mevel, Patrick Meybohm, B Meyer, Bernhard Meyer, F Meyer, Frank Meyer, Inna Meyer, Lucy Meyer, C Meyhoff, Christian Meyhoff, MA Meza Fonseca, M Meziane, Rameshwar Mhamane, Sang mi Lee, A Mian, Iftikhar Mian, G Micha, Georgia Micha, Afieharo Michael, ALR Michael, S Michael, Vishal Michael, John Michael DiBianco, John Michael Ranson, D Michailidou, N Michalopoulos, Nikolaos Michalopoulos, CW Michalski, Eden Micheal Ssettabi, Christodoulou Michel, M Michel, Maria Michela Di NUZZO, Stefano Michelagnoli, Alessandro Michele Bonomi, B Michelitsch, Birgit Michelitsch, Andrea Michelle Lowey Medina, Taku Michiura, S Michling, D Micic, Dusan Micic, Andrew Middleton, SB Middleton, R Midha, Rajesh Midha, Oleg Midlenko, P Midrio, Tsutomu Mieda, J Mielke, Sven Mieog, M Mietła, Christopher Mifsud, Mohammed Miftah, M Migliore, Marco Migliore, Federico Migliorelli, E Migliorino, G Mignot, Toledano Miguel, Francisco Miguel González Valverde, José Miguel Izquierdo, Gustavo Miguel Machain V, Luis Miguel Martinez Parra, Santiago Miguel Mata-Suarez, Juan Miguel Roberto Delgado, Jose Miguel Villacampa Auba, Jose Miguel Zaragozá García, L Miguelena, Radu Mihail Mirica, M Mihalik, J Mihanovic, Jakov Mihanovic, I Mihaylov, M Mihmanli, Borz Mihnea Bogdan, Pablo Mijahil Avilés Jiménez, Saulius Mikalauskas, Sarah Mikdad, Elie Mikhael, Pola Mikhail, A Mikhailova, T Mikhaylova, Hirokazu Miki, Z Mikovic, Filagot Mikru, Iva Mikulic, Vytenis Mikutaitis, Duha milad Abdullah, Marko Miladinov, Rita Milan Moussa, Flavio Milana, M Milanovic, Miljan Milanovic, Theodoros Milas, Ruben Milciades Varela Cano, Claudia Milena Orozco-Chamorro, M Milenkovic, BA Miles, Stephanie Miles, Fabio Milia, Mihailo Milićević, Borna Milicic, Filip Milisavljević, Gabriela-Mariana Militaru, P Milito, Aleksandar Miljković, T Millane, Kate Millar, A Miller, Clemens Miller, D Miller, Douglas Miller, Sarah Miller, Matthias Millesi, Keith Millikan, Emily Mills, L Mills, CP Millward, K Milne, Stephanie Milne, Sean Milner, B Milojevic, Bogomir Milojevic, M Milone, V Milosavljevic, B Milosevic, Genevieve Milot, Ioan Milotoiu, J Milovanovic, Jovica Milovanovic, Amelia Milton, Vladan Milutinović, M Mimbela, Ximena Mimica, Chew Min Hoe, Marina Minafra, Fabio Minamoto, Maria Minasidou, A Minaya Bravo, AM Minaya Bravo, Ana Minaya Bravo, AM Minaya-Bravo, Ana Minaya-Bravo, F Mineo Bianchi, Matthew Ming Kei Kwok, A Mingoli, Andrea Mingoli, Joan Minguell-Monyart, G Mínguez Ruiz, German Mínguez Ruiz, Andreas Minh Luu, Alina Minich, A Minicozzi, Annamaria Minicozzi, Stephen Minlah Allah, Francesco Minni, G Minto, T Minto, Pawel Miotla, Anum Mir, SA Mir, A Mirabella, Domenico Mirabella, Sepideh Miraj, Ester Miralpeix, Marcos Mirambeaux, BH Miranda, I Miranda, Ignacio Miranda, P Miranda, Norberto Miranda Espinsa, Enric Miret Alomar, RM Mirica, R Mirnezami, Reza Mirnezami, A Miron, Adrian Miron, Scott Miron, Aurel Mironescu, Taha Mirsal, M Mirsalehi, N Mirtorabi, M Mirza, MB Mirza, Monireh Mirzaie, M Miserez, Krisna Mishel Morales Chew, A Mishra, Abhijeet Mishra, Anand Mishra, Anjali Mishra, Anurag Mishra, Brijesh Mishra, N Mishra, Nitu Mishra, Swastik Mishra, Tushar Mishra, J Miskovic, Josip Miskovic, Biplap Misra, Gourab Misra, N Misra, S Misra, Sanjeev Misra, Vincent Misrai, Rajesh Mistry, Riyam Mistry, S Mistry, Courtney Mitchell, D Mitchell, S Mitchell, R Mithany, S Mitrasinovic, Magdalini Mitroudi, N Mitrovic, Nebojsa Mitrovic, Abhishek Mittal, Radheyshyam Mittal, Rohin Mittal, S Mittal, Samarth Mittal, Yash Mittal, M Mitteregger, Martin Mitteregger, C Mittermair, Christof Mittermair, Tajnin Mitu, V Miu, Morikazu Miyamoto, N Miyashita, Noriko Miyazawa, Toshiyuki Mizota, Fatimah MJ Hassan Almukhariq, Catherine Mkandawire, Aleš Mladěnka, Jan Mlakar, Busisiwe Mlambo, MM H Moahmmed, N Moawad, Nader Moawad, Ali Moazami Pour Moazami Pour, Maher Moazin, Shahd Mobarak, S Mochet, N Möckelmann, Paulina Moctezuma Velázquez, Tobias Moczko, A Modabber, Ali Modabber, Nawaf Modahi, Gautam Modak, N Modi, MM Modolo, Nikolaus Moeckelmann, Aiman Moeen, Bolaji Mofikoya, Chiedozie Mogbo, E Moggia, Elisabetta Moggia, Mahmoud Moghazy, C Mogoanta, Carmen-Aurelia Mogoanta, S Mogoanta, Stelian Mogoanta, Roberto Moguel, SMR Mohajeri, Balqees Mohamad, Raied Mohamad, A Mohamed, Ahmed Mohamed, Ali Mohamed, Awadelkarim Mohamed, Ayman Mohamed, Elsagad Mohamed, Eyas Mohamed, Guleed Mohamed, H Mohamed, Haider Mohamed, Hozifa Mohamed, I Mohamed, Ishak Mohamed, Liena Mohamed, M Mohamed, Maria Mohamed, Marwa Mohamed, Mazin Mohamed, Mervat Mohamed, Moustafa Mohamed, Muyed Mohamed, Omer Mohamed, S Mohamed, Sami Mohamed, Sugad Mohamed, Y Mohamed, Z Mohamed, A Mohamed Ads, Mostafa Mohamed Ahmed, A Mohamed alabany, Ferial Mohamed Ali Abbas, Omar Mohamed Alsamahy, Ahmed Mohamed Altukhy, Dina Mohamed elsaid, Ahmed Mohamed Farouk, Aliae Mohamed Hussein, Mokhtar Mohamed Ibrahim Abushanab, Omar Mohamed Makram, Mahmoud Mohamed Mohamed Shalaby, Abdelrahman Mohamed saad, Noha Mohamed Salah Ibrahim Moussa Hamouda, Amr Mohamed Sayed, Ali Yasen Y Mohamedahmed, AYY Mohamedahmed, A Mohammad, Abdulkader Mohammad, Adam Mohammad, Ahmad N Mohammad, Alaa Mohammad, Ammar Mohammad, Mazen Mohammad, Aqsa Mohammad eqbal Patel, Aminu Mohammad Mohammad, Syed Mohammad Umar Kabir, E Mohammadbeigi, I Mohammadbeigy, S Mohammadi, H Mohammadi sardoo, A Mohammed, AA Mohammed, Abdurezak Mohammed, Altayeb Mohammed, Ayman Mohammed, Burooj Mohammed, D Mohammed, Diary Mohammed, Garba Mohammed, K Mohammed, Khalid Mohammed, M Mohammed, Marwa Mohammed, MM Mohammed, N Mohammed, R Mohammed, Rawabi Mohammed, Rayhaan Mohammed, Tajudeen Mohammed, Naseer Mohammed Abdul, A Mohammed Abodina, A Mohammed alameen, Usman Mohammed Bello, Ghena Mohammed fawaz Ashour, Ansam Mohammed Ghaleb Alrobaiee, Anass Mohammed Majbar, Abdul-Jalilu Mohammed Muntaka, Mubder Mohammed Saeed, Seid Mohammed Yasin, Ricardo Mohammed-Ali, Amina Mohammed-Durosinlorun, Shamshuddin Mohammedali, H Mohan, Helen Mohan, Helen M Mohan, HM Mohan, M Mohan, Vijay Mohan Hanjoora, K Mohankumar, Khaled Mohd Ahmed Hasanein, AFN Mohd Ghazi, Nik Mohd Hazleigh, Ayesha Mohd Zain, S Mohindra, Sandeep Mohindra, Obaid Mohmand, Tamara Mohorko, Shahin Mohseni, M Mohsin, Muhammad Mohsin, R Mohsine, Raouf Mohsine, Omar Mohyieldin, Inshrah Moin, J Moir, John Moir, Peter Moisiuk, G Moitzi, Hossein Mokarami Mokarami, Ahmed Mokhtar, A Mokhtari, Mayad Moktash, Bogdan Moldovan, RH Moldovan, A Molero, Ángela Molero, Sarah Molfino, Andrej Moličnik, Marta Molina, Olimpia Molina, M Molina Bravo, GA Molina Proaño, V Molina Santos, M Molina-Corbacho, Matilde Molina-Corbacho, Joel Molinas, C Moliner Sanchez, Yegor Molitvin, Million Molla, Cea-Cea Moller, J Mollinedo-Hun, G Molteni, Samuel Molyneux, A Mombet, Annick Mombet, Ahmed Momen, Moses Momoh, Luisa Mona Kraus, E Monaco, Fabrizio Monaco, A Mondal, Jose Mondino, M Mondragon, Hamada Mondy, M Monfort Mira, Montserrat Monfort Mira, Lorenzo Mongardi, F Mongelli, Francesco Mongelli, M Moni, FA Monib, Fatma A Monib, Rocco Monica, Elsayed Monier, Mohammad Monir Abbas, Ana Monís, Keisuke Monji, Hervé Monka Lekuya, Berta Monleón López, Chloé Monnier, Sonia Monreal Clua, Guillermo Monsalve, JR T Monson, S Montal, Silvina Montal, G Montalvo Dominguez, C Montan, Carl Montan, Blanca Montcusí, JM Monteiro, Cathy Monteith, KM Montejo, M Montelatici, M Monteleone, Michela Monteleone, MT Montella, Jon-Alexis Montemayor, Andrea Montenegro, Emileth Montenegro, Lourdes Montero Cruces, R Montero Macías, Rosa Montero Macías, M Montero Vega, Lourdes Montes-Jovellar, Mario Montes-Manrique, Nuria Montferrer Estruch, Spencer Montgomery, Eleonora Monti, Marco Monti, Guillermo Montiel, S Montolío-Doñate, Margarita Montrimaite, J Montufar, M Montuori, Mauro Montuori, J Moon, RD C Moon, Alex Moore, Emily Moore, Hamish Moore, R Moore, Rachel Moore, T Moores, Thomas Moores, N Moorjani, Sergio Mora, Isabel Mora Oliver, I Mora-Guzmán, Isabela Moraes, H Morais, Henrique Morais, Mariana Morais, A Moral Duarte, I Moraleda Gudayol, Ines Moraleda Gudayol, E Morales, J Morales, Steffanía Morales, X Morales, Xavier Morales, Víctor Morales Ariza, Clara Morales Comas, N Morales Palacios, Nelson Morales Palacios, Eduardo Morales valencia, Carmelo Morales-Angulo, JE Morales-Castelan, D Morales-Garcia, Dieter Morales-Garcia, Olaya Moramay Romero-Limón, M Moran, Andrea Morandi, S Morarasu, J Moreau, Joshua Moreau, Cátia Moreira, João Moreira, F Moreira Borim, Andreu Morell, L Morelli, Luca Morelli, Alessia Morello, A Moreno, Amabelle Moreno, DH Moreno, Emmanuel Moreno, Jaime Moreno, Nicolas Moreno Mata, JA Moreno Muñoz, A Moreno Pérez, Mucio Moreno Portillo, Jesús Moreno Sierra, Marcelo Moreno Suarez, Teresa Moreno y Suárez, L Moreno-Gomez, Angela Moreno-Gutierrez, E Moreno-Palacios, M Moreno-Portillo, FJ Morera Ocón, Charlie Moret, G Moretto, Gianluigi Moretto, Silvia Moretto, Daniele Morezzi, Catrin Morgan, E Morgan, EA Morgan, Ellie Morgan, Katrina Morgan, R Morgan, Rebecca Morgan, Steffan Morgan, M Morgom, Jemiludeen Morhason-Bello, Krinal Mori, C Moriarty, Catherine Moriarty, Trpimir Morić, T Morichau-Beauchant, Tristan Morichau-Beauchant, Manuel Moriche Carretero, D Moris, Demetrios Moris, Tomonori Morita, Mai Moriyama, HL Morley, MT Morna, D Moro-Valdezate, David Moro-Valdezate, Radoslav Morochovič, Melanie Morote, Domagoj Morović, Brian Morris, C Morris, Daniel Morris, R Morris, Rachel Morris, Richard Morris, Jo Morrison, V Morrison-Jones, Victoria Morrison-Jones, A Morsi, Amr Morsy, MS Morsy, Hatan Mortada, Xavier Mortiers, Brienna Mortimer, P Mortini, Pietro Mortini, A Morton, Alastair Morton, Mostafa Mosabha, A Mosca, A Moscalu, F Mosley, Frances Mosley, Roman Mosneaga, Matti-Aleksi Mosorin, Monica Mosos, Jana-Lee Moss, Mafdi Mossaad, Victoria Mosshammer, Ahmed MHAM Mostafa, B Mostafa, Badr Mostafa, Mohamed Mostafa, El mostafa El Yaqine Er Raoudi, Ahmed Mostafa Saleh, Alejandra Mosteiro-Cadaval, D Moszkowicz, Clara Mota, Reza Motallebzadeh, N Motas, Natalia Motas, Masoud Motasaddi zarandy, Akira Motoyasu, H Mottaghi Moghaddam Shahri, D Motter, Dema Motter, GIL Mottola, Kanchan Motwani, Mohammed Mouaz Shebani, Susan Moug, Mohamed Mougahed, Hajar Moujtahid, Didier Moukoko, F Moura, Mário Moura, Mohamed Mourad, Mohamed Mourad Gargouri, Frederic Mouriaux, P Mourmouris, Panagiotis Mourmouris, MA Mous, Mohammad Mousa, V Mousafeiris, SA Mousavi, SH Mousavi, Ahmed Moussa, M Moussa, Esraa Moustafa, L Moutinho, Lana Moutinho, Tyler Mouw, O Mouzakis, H Moxon, Joaquin Moya-Angeler, Vimbai Moyana Muguto, Pablo Moyano, Assel Moyo, Gilbert Moyo, Nkosikhona Moyo, M Mozafari, Masoud Mozafari, M Mozel, Michelle Mozel, Lynette Mpagi Katassi, Christophe Mpirimbanyi, Bushr Mrad, M Mraiyan, Mohamed Mraiyan, A Msaddi, P Mshelbwala, Philip Mshelbwala, A Msherghi, Ahmed Msherghi, Wanga Mtimkulu, L Muallem-Kalmovich, Limor Muallem-Kalmovich, A Muashi, Mulaya Mubambe, Kamilia Mubarek, Areeba Mubarik, Teeba Mubaydeen, Isaac Mubezi, F Mucilli, Felice Mucilli, L Mude, Dillip Muduly, I Muehlbacher, Iris Muehlbacher, Hosam Muftah, Mohammed Mufth, Dionizi Muganga, Aamer Mughal, Samiullah Mughal, Didace Mugisa, W Mugla, Walid Mugla, JM Muguerza, Liviu Mugurel Bosinceanu, Arnold Muguwu, Rafat Muhammad, Saminu Muhammad, Shamsuddeen Muhammad, Shoaib Muhammad, Nasser Muhammad Amjad, Hafiz Muhammad Arif Arif, Sulaiman Muhammad Daneji, Usama Muhammad Kathia, Muheilan Muheilan, Roger Muhemi, Daniel MUHIRE Runanira, Khadija Muhmmed, Haidar Muhssein, Ali̇ Muhtaroğlu, Mahmoud Muhtaseb, P Muiesan, William Muirhead, Carolyne Muiru, Mohd Mujahed Alkurdieh, Sonia Mukase, Rene Mukezamfura, Partho Mukherjee, Poulome Mukherjee, S Mukherjee, Samrat Mukherjee, Soumitra Mukherjee, K Mukhtar, Venkat mukund reddy Galiveeti, Claudia Mulas Fernández, Manoj Mulchandani, W Mulder, Lemesa Muleta, F Mulita, Francesk Mulita, Husain Mulla, Omar Mulla, B Muller, Bruno Muller, M Muller, A Müller, Amelie Müller, Katharina Müller, P Müller, Sophie Müller, Thomas Müller, Tobias Müller, TU Müller, Andrea Mulliri, Esubalew Mulugeta, G Mulugeta, Gersam Mulugeta, Khizra Mumtaz, Kudzayi Munanzvi, M Munarriz, Marina Munarriz, Fasiha Munawwar, N Mundkur, G Mundy, Severien Muneza, Simbarashe Mungazi, S Mungo, Maathichsudhaar Muniandy, JA Municio Martín, Martino Munini, S Munot, Herisardy Munoz, R Muñoz, AS Munoz Abraham, CE Muñoz Aguirre, Iciar Muñoz Lindez, E Muñoz Sornosa, Ernesto Muñoz Sornosa, JM Muñoz Vives, V Muñoz-Atienza, L Munoz-Bellvis, L Muñoz-Bellvis, Luis Muñoz-Bellvis, William Munro, Fatema Munshi, E Muntaneza, George-Ovidiu Muntean, Maximilian Muntean, Alexandra Munteanu, Hal Munton, Emmanuel Munyaneza, Hussain Munyif, Akutu Munyika, Gianni Mura, A Murad, Musab Murad, A Muraglia, Angelo Muraglia, T Murakawa, Tomohiro Murakawa, Sreedutt Murali, V Muralidharan, Vijayaragavan Muralidharan, Musa Murat Caliskan, A Muratore, Andrea Muratore, Alfred Mureko, A Murgese, Alessandra Murgese, Uxue Murgoitio, ME Muriel, Judith Murillo, Bhaven Murji, B Murphy, Ben Murphy, C Murphy, James Murphy, Matthew Murphy, Niamh Murphy, S Murphy, Seamus Murphy, Suzanne Murphy, Daniel Murray, Emma Murray, Isabella Murray, James Murray, Ghulam Murtaza, J Murtha, Anandan Murugesan, Kesavan Murugesan, V Murzi, Darya Musa, Eltahir Musa, Reem Musa, Salam Musa, Kabir Musa Adamu, A Musa Kirfi, Abdullahi Musa Kirfi, Muhammad Musaab, Muhammad Musaab Munir, Norah Musallam, Othiniel Musana, Nizar Musawa, Esther Muscat, Luca Muscatello, Serena Musetti, Trust Mushawarima, Willard Mushiwokufa, Asma Mushtaq, Hassan Mushtaq, S Mushtaq, A Musina, Ana-Maria Musina, L Musini, Muhammad Muslim, Gurbankhan Muslumov, Fawzie Musrati, A Mustafa, Abdulla Mustafa, Fatima Mustafa, H Mustafa, Ismail Mustafa, Norasyikin Mustafa, Q Mustafa, Shiar Mustafa, Ahmad Mustafa Ahmad, Muntaqa Mustapha, Mushawiahti Mustapha, A Mustea, Alexander Mustea, Muhammad Mustehsan Bashir, Shameel Musthafa, Emmanuel Mutabazi, H Mutair, Nora Mutalima, Precious G T Mutambanengwe, Nastassja Mutarello, Guhan Muthkumaran, S Muthu, Sathish Muthu, Immaculee Mutimamwiza, D Mutlu, Nyasha Mutsonziwa, D Mutter, Didier Mutter, Fredderick Mutyaba, Twaha Muwanga, Peter Muwanguzi, Nasir Muzaffar, Rahil Muzaffar, Syed Muzamil Ishaq Andrabi, Michael Mwachiro, D Mwagiru, Garikai Mwale, Victor Mwangi, Claude Mwaria, Edwin Mwintiereh Ta-ang Yenli, Ha My Ngoc Nguyen, Khine Myat Win, D Myatt, R Myatt, Richard Myatt, J Myers, Jonathan Myers, Yulanda Myint, Aye Myintmo, P Myrelid, Anna Myriam Perrone, E Myriokefalitaki, Stephanie Myszkowski, Ranganath N, Isabella Naa Morkor Opandoh, N Naabo, Nuhu Naabo, L Naar, Leon Naar, Farhat Naaz Amir, Assumpta Nabawanuka, Bibi Nabeeha Peerally, Syed Nabeel Zafar, MH Nabian, A Nabil, Ahmed Nabil, Sara Nabil, Syed Nabil, EA Nachelleh, Emmanuel Nachelleh, L Nacif, A Nada, Ahmed Nada, Danilo Nadal Rodrigues, Sylvie Nadeau, Areeba Nadeem, Syed Nadeem Mujtaba, Anuja Nadeeshan Kumarasinghe, Narita Nadia Maria, R Nadina, I Nadj, Mandar Nadkarni, A Nadler, Marah Nadreen, Muhammad Naeem, Shiza Naeem, F Naegele, Felix Naegele, Abdullah Nael, Antoine Naem, Joelle Naem, Mohamed nafea Shaar, R Nafees Ahmed, Geerthan Nagachandra, Shoichi Nagamoto, Sriveena Naganathar, Hidekazu Naganuma, Mahesh Nagappa, M Nagar, Manoj Nagar, Pirashanthan Nagarasa, A Nagaratnam, Fumiaki Nagashima, Katharina Nagassima, Ahmed Nageeb, MA Nageh, Mohammed Nageh, Ali Naghibi, Marina Nagiub, Eleni-Aikaterini Nagorni, Pooja Nagpal, S Nagra, Sonal Nagra, SA Nah, MA K Nahid, C Nahm, Christopher Nahm, O Nahtomi Shick, Orit Nahtomi Shick, J Nahum, S Naidoo, Mohmed Naieem, Prashant Naik, Dina Nail, Gabriel Naimy, Asha Nair, Deepa Nair, Dilip Nair, Gavin Nair, Manojkumar S Nair, R Nair, Sreedevi Nair, N Najafian motahaver, Nima Najafian motahaver, M Najdy, Ramiro Najera, Norhan Naji, Osama Naji, Suely Nakagawa, Yuta Nakamura, Rinako Nakanishi, L Nakano, LC U Nakano, Luis Nakano, Cephas Nakanwagi, A Nakas, Apostolos Nakas, Yoshinori Nakata, Harumasa Nakazawa, Teddy Nakirijja, U Nakshbandi, M Nalbant, D Nally, Syeda Namayah Fatima Hussain, G Nambi, T Nambirajan, T Namikawa, Tsutomu Namikawa, Dr Namrata, Christine Namugenyi, Esther Namutosi, Gael R Nana, Asanga Nanayakkara, S Nandhra, P Nanjaiah, D Nanjiani, P Nankivell, Paul Nankivell, Naya Naoum, L Napolitano, Lena Napolitano, F Nappi, Francesco Nappi, Gennaro Nappo, SA Naqi, Z Naqui, Zaf Naqui, Wajih Naqvi, Akarshan Naraen, Sara Naranjo, Venkateshwaran Narasiman, G Narasimhan, Gomathy Narasimhan, P Narayan, Surya Narayan, Viswanathan Narayanan, Kuppurajan Narayanasamy, C Nardi, Caroline Nardi, B Nardo, P Naredla, Pradyumna Naredla, Dhanyata Narendra, B Narice, Harry Narroway, H Naseem, Haris Naseem, J Naseem, Mai Naseer, Ramisha Naseer Nagra, Ezzeldin Nashed, A Nashidengo, PR Nashidengo, O Nasim, A Nasir, Abdulrasheed Nasir, Ahmad Nasir, Areeba Nasir, Manal Nasir, A Nasirpour, MH Nasirpour, Upasana Naskar, A Nasr, Mohammed Nasreddin, H Nassa, Goretti Nassali, Rosemary Nassanga, Ahmed Nassar, Ahmed Nasser, M Nasser, Nourhan Nasser, S Nasser, Shaimaa Nasser, Y Nasser, Mohammed Nassif, Yasar Nassif, Moses Nassimu, C Nastos, Fernando Natal Álvarez, J Natale, Leidy Natalia Idarraga Ramírez, Marisa Natalia Martinez, R Nataraja, Ramesh Nataraja, RM Nataraja, R Nath, A Nathan, Meena Nathan, Senthil K Nathan, Rajesh Nathani, Paulino Nathaniel III Zamesa, A Nathens, Hilli Nativ, D Naumann, DN Naumann, Fatima Naumeri, M Naunheim, Matthew Naunheim, Shazia Naureen, Sara Nausheen, Devaraj M Navaratnam, L Navaratne, Lalin Navaratne, A Navarrete-Peón, Alberto Navarrete-Peón, A Navarro, Alex Navarro, Daniel Navarro, Rosalia Navarro Casado, Sergio Navarro Martínez, Jorge Navarro-Alean, Alvaro Navarro-Barrios, A Navarro-Sánchez, Antonio Navarro-Sánchez, Ł Nawacki, Hossam Nawara, Anfal Nawawi, Hassan Nawaz Yaqoob, Prakash Nayak, Jvalant Nayan Parekh, Alejandra Nayen, A Nayen Sainz de la Fuente, Syed Nayyar Afaque, Fatima Nayyer, Falak Naz, Humera Naz Altaf, Mariles Nazal, Zuhail Nazar, S Nazarian, Nawshin Nazia, Shahani Nazir, Umer Nazir, Mohd Nazli Kamarulzaman, Ahmad nazran Fadzli, Ahmad nbrass Kabawi, Isaie Ncogoza, Gamuchirai Ndabvonga, Fillipus Ndatewapo, Chinedu Ndegbu, Olivier Ndizeye, Sibusiso Ndlovu, Abdourahmane Ndong, Alexandru Neagu, Annelise Neal, N Neal, Naomi Neal, Gregory Neal-Smith, P Neary, Paul Neary, PC Neary, Peter Neary, PM Neary, Ahmet Necati Sanli, Yen Nee Jenny Bo, Wan Nee Shue, D Neely, Samuel Negash, I Negoi, Ionut Negoi, V Negoiță, Lucian Negreanu, JR Negrete Ocampo, G Negri, A Negussie, Abraham Negussie, Tihitena Negussie Mammo, Archana Nehe, D Nehra, N Neidert, Nicolas Neidert, J Neil-Dwyer, H Nejad Biglari, Valery Nekoval, Corne Nel, D Nel, Daniel Nel, Henco Nel, R Nel, Prathibha Nelihela, Ellen Nelissen, D Nellensteijn, T Nelli, Tommaso Nelli, Caleb Nelson, T Nelson, N Nemat, Marcus Nemeth, Abhay Nene, J Neny, Y Nerabani, F Neri, I Neri, J Neri, R Nerlikar, Shuichiro Neshige, LC Nespoli, Ashrafun Nessa, Carolyn Nessim, Carl Neuerburg, Filipe Neves, Miguel Neves, Arnaldo Neves Santos Silva, Andrew Newcomb, Laura Newitt, Jeremy Newman, Matthew Newman, S Newman, Samuel Newman, T Newman, C Newton, K Newton, Lydia Newton, V Neykov, Vasil Neykov, Yves Nezerwa, Giulia Nezi, A Ng, Benjamin Ng, Calvin S Ng, CE Ng, Chi-Fai Ng, Dennis Ng, J Ng, JC K Ng, Jia Y Ng, Jimmy Ng, KC Ng, M Ng, Michael Ng, Sherwin Ng, Simon Ng, YJ Ng We Yong, Joshua Ng-Kamstra, Ming Ngan Aloysius Tan, S Ngaserin, Sabrina Ngaserin, CW Ngo, G Ngock, AW T Ngu, James Ngu, James Ngu Chi Yong, D Nguen, Allan Ngulube, Peter Ngungi Njuki, Alain Nguyen, Anh Nguyen, Huan Nguyen, Sebastien Nguyen, TA Nguyen, Ncamsile Nhlabathi, Orna Ni Bhroin, Arastoo Nia, Mourad Niazi, C Nic Gabhann, A Nic Giolla Bháin, V Nicastro, Eu Nice Neo, Jaya Nichani, Margit Nichita, Ella Nicholas, A Nicholson, K Nicholson, Kristina Nicholson, S Nicholson, Mutekanga Nicholus, Sergio Nicola Forti Parri, Antonio Nicola Giordano, Sina Nicolaiciuc, Beaud Nicolas, Humberto Nicolas Galleano Ruiz, Juan Nicolas Rodriguez Niño, T Nicolás-López, Tatiana Nicolás-López, Cristina Niculae, S Nida, Melkamu Nidaw, Andreas Niemeier, AJ Nieto Calvache, E Nieto Ortega, Ana Nieto-Moreno, Elena Nieto-Moreno, Ninad Nigalye, J Nigh, H Nikaj, Herald Nikaj, Nikolaos Nikiteas, Kiselev Nikolai, Karine Nikolaieva, S Nikolaou, B Nikolic, Srdjan Nikolic, Zoi Nikoloudaki, Taxiarchis Nikolouzakis, B Nikolovska, Bisera Nikolovska, Hamed Nikoupour, Alexandrina Nikova, Boateng Nimako, Jilac Nimako-Mensah, Suad Nimale, Bahaa Nimer, Oti Nimi Aria, Avegail Niña Uy, Aleksandar Ninic, M Ninkovic, Marijana Ninkovic, M Niquen-Jimenez, Milagros Niquen-Jimenez, Anand Nirgude, Sudhara Niriella, Kanwal Nisa, P Nisar, Sebastian P Nischwitz, SP Nischwitz, Noriaki Nishihara, Kouhei Nishikawa, Karolina Niska, GE Nita, C Nitschke, Christine Nitschke, Amy Nixon, S Niyas, Irénée Niyongombwa, Maria Nizami, Rafal Niziol, Narindra Njarasoa Mihaja Razafimanjato, Nwabundo Njeze, Tsi Njim, Vinod Nk, E Nkenke, Theresa Nkole, John Nkrumah, J M Solange Nkubito, Elysé Nkunzimana, Ikenna Nnabugwu, Henry Nnajiuba, Kareem Noah, S Nobile, DM Noboa, G Nobre, A Nobre Pinto, Antonio Nocchi Kalil, Gianluca Nocera, Michio Noda, Chiara Noe, C Noel, Colin Noel, J Noel, Audrey Noël, Sandra Nofal, SJM Nofal, Hikari Noguchi, O Nogueira, J Nogueiro, Jorge Nogueiro, Lidia Noguera Roman, A Nogués, Ana Nogués, M Noguez Castillo, Monica Noguez Castillo, Barbara Noiret, B Nolan, Deirdre Nolan, Florencia Noll, Peter Nolte, Milou Noltes, Hamza Noman, Takeshi Nomura, Anthony Noone, Anthony Noor, Fazal Noor, M Noor, Mohammad noor Sultan, Shahryar Noordin, Ehsan Noori, Farshad Noori, R Norawat, Rahul Norawat, C Norcini, M Nordberg, Martin Nordberg, L Nordin, Zahida Noreen, Mohd Norhisham Azmi Abdul Rahman, David C Noriega, D Noriego Muñoz, J Norman, Lisa Norman, Shusaku Noro, M Norouzi, Samieh Norouzi, Jonathan Norris, Alan Norrish, Michael Nortey, A Northey, Joel Norton, Sam Norton, William Norton, Jaysonnel Notario, TM Noton, Toby Noton, F Notte, Quirin Notz, T Nouh, Thamer Nouh, Mohammad Nour Kitaz, Mohammad Nour Shashaa, Sadaf Noureen, Yasser Noureldin, Z Novak, Zoltan Novak, Maria Novella Ringressi, S Novello, Simone Novello, Daniela Novembre, Alessandra Novi, David Novikov, A Novikova, Anastasia Novikova, Beatriz Novoa, R Novysedlak, René Novysedlak, René Novysedlák, K Nowak, Kai Nowak, Mostafa Nowar, Yvonne Nowosielski, Josephine Nsaful, Steven Nshuti, Edmond Ntaganda, Maria Ntalouka, MP Ntalouka, Arnold Ntege, Joseph Ntege, Japhet Ntezamizero, F Ntirenganya, Faustin Ntirenganya, Sarwar Nubair, N Nudell, BD Nuertey, T Nugent, Timothy Nugent, W Nugent III, AAA Nugud Abd Alwahab Aljafary, Ashwani Nugur, Afnan Nuh, Yasir Nuhu Jibril, KS Nunes, QM Nunes, Rafael Nunes, RL Nunes, Sara Nunes, Margarida Nunes Coelho, M Nunes-Coelho, J Nunez, Jade Nunez, HM Nuñez, J Nuñez, Jorge Nuñez, Ruth Nuñez, Analia Núñez, Bernardo Núñez, J Núñez, Henar Nuñez Del Barrio, Jorge Núñez Lucic, Sara Núñez OSullivan, H Núñez-Del Barrio, B Nunez-Garcia, Brenda Nunez-Garcia, R Nunn, Duarte Nuno Amaro, Cesar Nuño-Escobar, JW Nunoo-Mensah, Anna Nunzia Della Gatta, Syeda Nureena Syed Jafer Hussain Zaidi, Talgat Nurgozhin, Amjad Nuseir, SE Nwabuoku, Callistus Nwachukwu, Ijeoma Nwachukwu, Ikechukwu Nwafor, Chimaobi Nwagboso, OG Nwaorgu, Onyekwere Nwaorgu, Nnamdi Nwashilli, C Nwegbu, CG Nwegbu, Chukwuemeka Nwegbu, Hope Nwinee, SO Nwose, David Nwosu, Beauty Nyadu, Krystel Nyangoh Timoh, Yaa Nyarko Agyeman, M Nycz, Miriam Nyeko-Lacek, Rachel M Nygaard, RM Nygaard, Domitille Nyirahabakurama, Jeannette Nyirahabimana, Alexandre Nyirimodoka, C Nzekwue, IC Nzenwa, C O connor, Clare O Connor, J O Connor, Helle Ø Kristensen, Augustine O Takure, S O’Brien, A O’driscoll-collins, Kristin O'Mara-Gardner, C O’Neil, C O’Neill, Izegaegbe Obadan, Osarobo Obahiagbon, Kadhim Obaid, Munzir Obaid, LA OBanion, Shinju Obara, C Obasi, John Obateru, Ambe Obbeng, K Obeidat, Riyad Obeidat, M Oberlechner, Hamoud Obied, D Obrand, S OBrien, Stephen OBrien, Christian Obrist, Ozoemene Obuekwe, Ralph Obure, Lorena Ocampo, Lorena Ocampos, Montserrat Ocampos Hernandez, J Ocaña, Savino Occhionorelli, Francisco J Ochoa Carrillo, Katherine Ochoa Gaete, E Ochoa Maldonado, Begoña Ochoa Villalabeitia, C OConnell, Eimear OConnell, Rachel Oconnell, Brendan OConnor, C OConnor, DB OConnor, Donal B OConnor, J OConnor, Jennifer OConnor, Z OConnor, Zachary OConnor, Narcis Octavian Zarnescu, Sandhya Od, ED Odai, Amar Odedra, A Odeh, Abdulrahman Odeh, Funlayo Odejinmi, Diego Odetto, Olubunmi Odeyemi, F Odicino, Franco Odicino, Guillaume Odilon Tsiambanizafy, Eddie Odonnell, Richard ODonnell, Cristina ODonoghue, Gerrard ODonoghue, A Odriscoll-collins, Stella Oduah, Tunde Odunafolabi, Oladayo Oduola, O Odutola, Oluwatomi Odutola, Eoin OFarrell, Richard Offiong, D Öfner, Bernard Ofori, EO Ofori, B Ofori Appiah, Obed Ofori Nyarko, Munehiro Ogawa, E Ogden, Emma Ogden, Roy Ogenya, Tomomi Ogihara, Richard Ogirma Baidoo, Michael OGrady, Rosemary Ogu, H Öğücü, Olukayode Ogunade, Ibukunolu Ogundele, O Ogundoyin, Olakayode Ogundoyin, Omowonuola Ogundoyin, Olumuyiwa Ogunlaja, Tolulope Ogunrewo, Oluseyi Ogunsua, AA Ogunyemi, Benson Oguttu, Cebrail Oğuz, Ufuk Oguz Idiz, JS Oh, Patrick OHagan, Shinnosuke Ohama, Rozana Ohara, Ephraim Ohazurike, K Oikonomou, Kyriakos Oikonomou, S Oishi, Cristina Ojeda Thies, C Ojeda-Thies, Olugbenga Ojo, Owolabi Ojo, H Okada, Hidetaka Okada, Hiroshi Okada, Reina Okada, Shinichiro Okada, Toshimasa Okada, Barbara Okafor, BU Okafor, Kaio Okamura, Yukiyasu Okamura, R OKane, Y Okazawa, V Okechukwu, Aloy Okechukwu Ugwu, John OKelly, Oghenekevwe Okere, Chukwuma Okereke, Amina Okhakhu, L Okiror, W Okoba, Louis Okolie, Ijeoma Okonkwo, Kelechukwu Okoro, Eloka Okoye, Onyedika Okoye, Anthony Okpani, Charles Okpani, Thomas Okpoti Konney, V Oktseloglou, Stanley Okugbo, Kehinde Okunade, Abiodun Okunlola, AI Okunlola, T Okuno, AA Okunowo, Adeyemi Okunowo, Blasius Okwara, Afusat Olabinjo, Titilola Oladejo, Ajibola Oladiran, Thomas Olagboyega Olajide, Naomi Olagunju, Hadijat Olaide Raji, Adewale Olajide, Peter Olalekan Odeyemi, Julius Olaogun, Oluwole Olaomi, Rasaq Olaosebikan, Ifedolapo Olaoye, O Olasehinde, Olalekan Olasehinde, David Olatayo Olayiwola, VD Olave Montaño, Olayinka Olawoye, Aminat Olayinka Ahmed, Tolulope Olayinka Sayomi, Dina Olaywah, Noor Olaywah, O Olazábal, Massimo Oldani, Megan Oldbury, Gaia Oldrà, Claudia Olea Vielba, E Olearo, Elena Olearo, G Olgac, Annette Olieman, P Oliva, R Oliva, Ramon Oliva, F Oliva Mompean, Rachel Olive, A Oliveira, Antonio Oliveira, CM Oliveira, J Oliveira, JM Oliveira, Joana Oliveira, João Oliveira, P Oliveira, Priscila R Oliveira, Thomas Oliver, Tibor Oliver Andraschofsky, Darwin Oliver Desposorio Armestar, Luis Oliver García, JR Oliver Guillen, M Olivera Villanueva, CA Oliveros Ruiz, Ana Olivia Cortes-Flores, J Olivier, M Olivos, Maricarmen Olivos, Didier Ollat, I Oller, Inmaculada Oller, Sue Olliff, Benjamin Ollivere, Stefano Olmi, C Olona, S Olori, Samson Olori, Tope Olowogbayi, KA Olson, Kristofor A Olson, SA Olson, Adebayo Olugbami, Denis Oluka, D Olulana, Dare Olulana, Adeola Olusanya, Anne Olute, Fatudimu Oluwafemi, Adeoye Oluwakanyinsola Debo-Aina, Oscar-Everardo Olvera-Flores, I Omar, Kadra Omar, M Omar, Mabruka Omar, Mohamed W Omar, MS M Omar, Samaa Omar, Sana Omar, W Omar, Wael Omar, Hayat Omar Abunaaja, Ahmed omar Abushahma, David Omar Arriaga Zavala, Bashar Omar Falah Alawneh, Mohamed Omar Herdan, José Omar Zorrilla Lara, Usra Omara, Rand Y Omari, Saranda Ombashi, L OMeara, Ahmed Omer Kenawy, S Ömeroğlu, Sinan Ömeroğlu, A Omigbodun, Akinyinka Omigbodun, Paul Omiragi, OA Omisanjo, Olufunmilade Omisanjo, I Omiste, E Omling, A Omnia, Justina Omoikhefe Alegbeleye, Philip Omotosho, Janna Omran, M Ömür, Paul Onakoya, MA Onan, Julian Onate, Mikel Oñate, M Oñate Aguirre, C ONeil, A ONeill, Aine ONeill, C ONeill, Christine ONeill, JR ONeill, Robert ONeill, CS Ong, CT Ong, Daniel Ong, Lester Ong, Oluwafemi Oni, Samson Oni, T Oni, S Onida, Stephen Onjefu, Motoaki Ono, Elliot Onochie, Ilaria Onorati, L Onos, Güralp Onur Ceyhan, Mustafa Onur Oztan, Ijeoma Onwuagha, Ngozi Onyeagwara, John Onyeji, Ndubuisi Onyemaechi, A Oo, Aung Oo, Chun Ooi, Rucira Ooi, SZY Ooi, W Oosterlinck, Wouter Oosterlinck, S Oosterling, Steven Oosterling, Benjamin Oosthuizen, Rivero Opano, Hameedat Opeyemi Abdussalam, Janis Opincans, Wisdom Opoku Amankwaa, Ijeoma Oppah, Victory Oputa, Alex Orădan, Emmanuel Oranu, Ngozi Orazulike, Jorge Ordemar, Sebastian Ordoñez, E Ore, E Oré, Nicole Organ, Ahmed Organjee, Sergelen Orgoi, Nazlı Orhan, Mukadder Orhan Sungur, M ORiordain, J Oriordan, JM ORiordan, Mathias Orji, Till Orla Klatte, Martin Ormeño, S Ornaghi, Sara Ornaghi, Mariano Oropeza, J Orozco Mera, J Orozco Perez, CM Orozco-Chamorro, J Orozco-Perez, Jaime Orozco-Perez, Soyombo Orsoo, Ma Dolores Orta Díaz, Natalia Ortega, Shannat Ortega, Manuel Ortega Oria de Rueda, J Ortega Serrano, I Ortega Vazquez, Irene Ortega Vázquez, Liz Ortiz, N Ortiz, Fabio Ortiz De La Cruz, FJ Ortiz de Solorzano-Aurusa, David Ortiz López, Camilo Ortiz Silva, Almudena Ortiz Simón, MR Ortiz-Argomedo, Mustafa Oruç, L Oryadi zanjani, Leila Oryadi zanjani, Anita Osabutey, L Osagie, Olabisi Osagie, Shuhei Osaki, Muhammad Osama Khan, Syed Osama Zohaib Ullah, T Osborn, Tamara Osborn, Laura Osborne, A Oscarsson, Anna Oscarsson, Lydia Osea, Eustace Oseghale, Peter Osei-Bonsu, Dorcas Osei-Poku, Ethel Osei-Tutu, A Oseira-Reigosa, Osarenkhoe Osemwegie, Margaret OShea, Philip Osho, OA Oshodi, YA Oshodi, Babatunde Osinaike, Antonia Osl, Alkhansa Osman, Amna Osman, Elaf Osman, Halim Osman, I Osman, Imoro Osman, Khalid Osman, Leyla Osman, Mohamed Osman, Rafael Osmar Adorno Garayo, Filipe Osni Coelho, A Osorio, Derlis Osorio, walter A Osorio, C Osório, M Osso, ME Ossola, Charles Osterberg, Georg Osterhoff, Erica OSullivan, H OSullivan, M OSullivan, Laura Osuna, Jaqueline Osuna-Rubio, Roland Osuoji, Edgar Oswaldo Hernandez Burgos, Wessal Otaif, E Otañez, Sameer Otayfah, Robin Otchwemah, Ahmed Oteem, V Oter, Volkan Oter, Henry Othieno misanga, Sudheer Othiyil vayoth, A Othman, Abdullmujeeb Othman, Ahmad Othman, Eyas Othman, H Othman, Mohammed Othman, Salasiah Othman, James Otieno, M Otify, Mohamed Otify, CE Otiniano Alvarado, Job Otokwala, Riinu Ots, Yuji Otsuka, Johannes Ott, Helena Otte, Stephanie Ottl, J Ottolina, A Ottone, C Ouanezar, Takashi Ouchi, MY Oudrhiri, Abdallah Ouf, M Oukan, Anas Ould Si Amar, J Ourieff, Jared Ourieff, Susana Ourô, Samia Ousouss, K Oussama, O Outani, Oumaima Outani, A Ovaitt, E Ovejero Merino, Rawan Owaimer, Qais Owais, Muhammad Owais Abdul Ghani, R Owen, David Owens, P Owens, Patrick Owens, Cornelia Ower, M Owiedat, Mustafa Owiedat, Jefferson Owusu Adae, Emmaunel Owusu Ofori, A Owusu-Addo, Tasuku Oyama, T Oyebanji, Funmilayo Oyediji, OA Oyelakin, Oyeleye Oyelakin, Oluwole Oyeleye, Nasir Oyelowo, O Oyende, Olamide Oyende, Oluniyi Oyetunde Olubayo, B Oyewole, Olugboyega Oyewole, Esther Oyewusi, Olumide Oyinloye, Ahmad Ozair, Muhammad Ozair Awan, Farrukh Ozair Shah, Gultekin Ozan Kucuk, Ali Özant, İH Özata, V Ozben, Volkan Ozben, A Özcan, Adem Özcan, Necdet Özçay, MF Ozcelik, Egemen Ozdemir, Kayhan Ozdemir, Kamil Özdoğan, Elif Özeller, D Ozgediz, Doruk Ozgediz, U Özgen, Utku Özgen, I Ozgur, Ilker Ozgur, OF Ozkan, Güneş özlem Yıldız, BB Ozmen, MM Ozmen, Christine Ozone, H Ozsahin, Joshua Ozua, Hareesh P B, Thirumanikandan P L, R P Shenoy, Peter Paal, Anishka Pabari, Carmignani Pablo, Juan Pablo Alzate, Juan Pablo Campana, Pedro Pablo Díaz Vásquez, Juan Pablo Idrovo, Juan pablo Villate leon, D Pacheco Sánchez, David Pacheco Sánchez, M Pachl, Max Pachl, CA Pacilio, M Pacilli, Maurizio Pacilli, F Padilla-Lichtenberger, D Padilla-Valverde, David Padilla-Valverde, Greg Padmore, Walter Páez, Gianluca Pagano, Luca Pagano, Richard Page, S Page, Naila Pagès, D Paglione, U Pahalawatta, Upuli Pahalawatta, M Pai, Lucia Paiano, S Paiella, Salvatore Paiella, William Paine, F Pais, José Pais, S Paitici, Stefan Paitici, Joana Paiva, Filipe Paiva-Santos, H Pajan, Hendra Pajan, Srbislav Pajić, M Pakiž, M Pal, RR Pala Bhaskar, Carlos Palacios, Juan Palacios, RM Palacios Huatuco, S Palagi, R Palaia, attibele Palaksha Manjunatha, Naresh Palapalle, Kandasami Palayan, P Palazon Bellver, Pedro Palazon Bellver, F Palazzo, M Palechor, A Palepa, Alejandro Palines, GM Palini, Effrosyni Palla, Henrik Palm, Matteo Palma, P Palma, Nikolas Palma Caucig, M Palmeri, Matteo Palmeri, Emanuela Palmerini, Gerardo Palmieri, Silvia Palmisano, Sara Palomares Casasús, Liliam Palomino, Fiorella Palomino Escalante, Nicholas Paltoglou, Alessio Palumbo, Mara Palumbo, EC Pama, Jessica Pamela Portillo Sosa, Jorge Pamias, Sousana Panagiotidou, Peter Panagiotou, P Panahi, B Panamarenko, Yiannis Panayiotou, Guergana Panayotova, Suresh Panchakshariah, Abinash Panda, Ritesh Panda, S Pandanaboyana, Amita Pandey, Himanshu Pandey, Sanjay Pandey, V Panduro-Correa, Lucía Pañeda, Divyansh Panesar, H Panesar, M Paniagua Garcia Senorans, Sibasish Panigrahi, Carolina Panis, Taufiq Panjwani, S Pankaj, S Pankhania, Mario Pannullo, Igor Panov, Adrieli Pansani, Rajeev Pant, Christina Panteli, Maria Pantelidou, DA Pantoja Pachajoa, JC Pantoja Rodriguez, Pavlos Pantos, Vishwakar Panuganti, VK Panwar, A Panyko, Arpád Panyko, Luisa Paola Garzon, Maria Paola Giusti, Maria Paola Menna, Anna paola Pezzuto, Francesca Paola Tropeano, Ximena Paola Vasquez Ojeda, Benjamin Paolini, Pier Paolo Panciani, Adrian Papa, Ioannis Papaconstantinou, A Papadia, Andrea Papadia, M Papadoliopoulou, A Papadopoulos, V Papadopoulos, Vasileios Papadopoulos, Triada Papadopoulou, V Papagni, Vincenzo Papagni, Alexandros Papalampros, Stylianos Papalexandris, R Papalia, Rocco Papalia, Matteo Papandrea, V Papanikolaou, Vasileios Papanikolaou, Theofanis Papas, Panayiotis Papatheodorou, K Papavasiliou, Kyriakos Papavasiliou, I Papazacharias, Nancy Papendick, Ketevan Papidze, A Papinutti, Sam Pappas, B Paquette, Mrunal Parab, Sandesh Parab, J Parakh, Thammawat Parakonthun, Arjun Paramasivan, Javier Páramo Zunzunegui, C Paranjape, Charu Paranjape, M Paranyak, A Paraskeva, KI Paraskevas, Kosmas I Paraskevas, K Paraskevopoulos, Konstantinos Paraskevopoulos, Rivka Pardes, Ana Pardilho, Dinshaw Pardiwala, JM Pardo Garcia, I Paredes, Igor Paredes, Susana Paredes, EJ Paredes Alvarez, Angie Paredes Caturiny, Dilber Pareed, P Pareek, Puneet Pareek, R Pareja, Felipe Pareja Ciuro, F Pareja-Ciuró, A Parello, Angelo Parello, A Parente, Alessandro Parente, Charlotte Parfitt, Pinki Pargal, R Pargaru, C Parianos, Sucheta Parija, Mélinda Paris, P Parise, Andy Park, C Park, Chang Park, Felicity Park, Jennifer Park, Melissa Park, P Park, Paul Park, Dominic Parker, Katie-Louise Parker, Robert Parker, Benjamin Parkin, Cameron Parkin, Edward Parkin, K Parkins, D Parlanti, Daniele Parlanti, C Parmar, Chetan Parmar, Matias Parodi, F Parolini, Katherine Parra Abaunza, N Parra Paredes, Fazl Parray, Pedro Parreira, B Parrella, MD Parreno-Sacdalan, P Parri, Paco Parri, C Parrilla, Andrew Parrish, J Parry, James Parry, Laura Parry, William Parry-Smith, A Parseliunas, Rajinder Parshad, Simon Parsons, Thomas Parsons, Ramaa Parulekar, S Parveen, Sajitha Parveen, Hammad Parwaiz, A Pasca, A Pascale, G Pascale, G Pascarella, Giuseppe Pascarella, JA Pasch, J Pascoe, LA Pascua Gómez, À Pascual, Ángela Pascual, FR Pascual, T Pascual, Victoria Pascual Escudero, Isabel Pascual Miguelañez, Montserrat Pascual Pascual Arellano, M Pascual Samaniego, Miguel Pascual Samaniego, Patricia Pascual-Cambero, M Pashaei, F Pasini, Miram Pasini, J Paskas, A Paspala, D Paspaliari, MJ Paspuel Villacís, S Pasquali, Ernesto Pasquini, R Pasricha, Martin Passadore, G Passot, I Pastau, Oscar Pastor, Tania Pastor, Ana Pastor Zapata, F Pata, M Patabendige, Malitha Patabendige, Robert Patachia, S Patauner, K Pateas, A Patel, Akshay Patel, Arjun Patel, Bhavin Patel, CHK Patel, D Patel, Jamie Patel, K Patel, Kapila Patel, Krishnakumure Patel, Lopa Patel, M Patel, Minil Patel, Mohammed Patel, Nikhita Patel, P Patel, Panna Patel, Preemal Patel, Priyank Patel, Rakesh Patel, Reece Patel, Riana Patel, S Patel, Shriyam Patel, Sujan Patel, Nikolaos Patelis, Muna Patell, J Patena Forte, Joana Patena Forte, Amanda Paterson, H Paterson, Hugh Paterson, Manish Pathak, Prachi Pathak, Samir Pathak, Sohilkhan Pathan, AM Pathanki, C Pathirannehalage Don, O Pathmanaban, Omar Pathmanaban, Keren Pathmanathan, A Patience, C Patient, Manish Patil, Ninad Patil, R Patil, Rakesh Patil, S Patil, Romeo Patini, NG Patino-Jaramillo, Amrita Patkar, Pradnya Patkar, S Patkar, Shraddha Patkar, P Patki, D Patkowski, Ioannis Patoulias, Saroj Patra, Daniela Patricia Escalante Ureche, Ana Patricia Legido Morán, Martha Patricia Pérez de León Vázquez, B Patrício, Andrea Patrizi, R Patrone, Renato Patrone, P Pattyn, Kathrin Patzer, M Pau, A Paul, Claudia Paul, Emila Paul, Michaela Paul, Rajesh Paul, Sharmila Paul, Marc Paul Lopez, Christopher Paul Millward, Jean Paul Rugambwa, Jean Paul Shumbusho, Ana Paula Ferreira Pinto, Ana Paula Riverola Aso, Henry Paulino, Jeffrey Paulino, Martin Paulo, Luiz Paulo Kowalski, João Paulo Medici, Nipseey Pauloe Candelario, Laerke Paulsen, P Paunero Vazquez, Patricia Paunero Vazquez, Aleksandra Paunovic, I Paunovic, Ivan Paunovic, Petra Pavic Palac, Aswathy Pavithran, W Pavlis, Maja Pavlov, Pavel Pavlov, Ivana Pavlovic, M Pavlovic, O Pavlovic, G Pavone, V Pavone, Jānis Pāvulāns, Abhijit Pawar, Neha Pawar, P Pawar, Pranay Pawar, Shweta Pawar, Carmen Payá-Llorente, A Payandeh, E Payet, Eduardo Payet, CJ Payne, Maria Paz Bohórquez-Tarazona, A Paz-Aparicio, V Pazin, T Pazionis, Janneth Pazmino-Canizares, Andrejs Pcolkins, A Pearce, Adrian Pearce, J Pearce, L Pearce, J Pearl, A Pearson, Natasha Pearson, F Pecchia, Francesca Pecchini, G Peck, A Peckham-Cooper, Adam Peckham-Cooper, Alessandra Pecoraro, Felice Pecoraro, Abhinav Pednekar, NF Pedraza Alonso, C Pedrazzani, Corrado Pedrazzani, Joao Pedreira Duprat Neto, R Pedrini Cruz, Ricardo Pedrini Cruz, José Pedro, José Pedro Fernandes dos Santos, João Pedro Melo Neves, João Pedro Reis, Leire Pedrosa, R Pedroso de Lima, Rita Pedroso de Lima, Michał Pędziwiatr, Shanell Peeriyah, S Peeroo, I Peev, Igor Peev, R Peevor, Hrvoje Pehar, A Peig-Font, Christian Peiper, C Peirce, Colin Peirce, Bryony Peiris, S Pejkova, Sofija Pejkova, I Pejovic, Ilija Pejovic, MR Pekcici, CÁ Peláez Sánchez, PM Pelaéz Torres, D Pelaggi, Stéphane Pelet, Aylin Pelin Cil, G Pelino, Arrigo Pellacani, Alice Pellegrini, L Pellegrino, Gabriela Pelletier, J Pelletier, Massimiliano Pelli, R Pellini, Raul Pellini, G Pellino, Gianluca Pellino, M Pelloni, Maria Pelloni, Roberto Peltrini, C Peluso, P Pemmasani, Christina Pempe, LG Peña Balboa, Emmanuel Peña Gómez Portugal, E Pena Gomez-Portugal, Emilio Peña Ros, GV Peña Saltos, DS Peñaherrera Toapaxi, Julio Peñarrocha, Luka Penezić, E Peng, Ed Peng, Pasi Pengermä, Rachel Pennington, Edgardo Penserga, F Pepe, Gilda Pepe, Philip Peprah Oppong, M Peralta Ferreira, Marisa Peralta Ferreira, Rajeev Peravali, Digby Percy, Eduardo Perea del Pozo, J Pereca, Jelizaveta Pereca, Adela Pereda, Mariana Pereda, MR Pereda, A Pereira, André Pereira, C Pereira, Cristiana Pereira, Mário Pereira, R Pereira, Rute Pereira, FA Pereira Júnior, A Pereira Rodrigues, A Pereira-Neves, António Pereira-Neves, Diego PereiraNuñez, Oleg Perepelitsa, Eranga Perera, R Perera, Remei Perera Sarri, Michal Perets, Marine Peretti, Irene Perez, S Perez, Y Perez, Yolanda Perez, Clara Pérez, Marisol Perez Cerdeira, DF Perez Correa, Cristina Pérez Costoya, MD Perez Diaz, Marta Pérez Febles, A Perez Ferrer, Antonio Perez Ferrer, M Perez Gonzalez, Marta Perez Gonzalez, Marina Pérez González, Carolina Perez Granados, Francisco Pérez López, A Pérez Núñez, Sara Pérez Palao, C Perez Rivera, Carlos J Perez Rivera, CJ Perez Rivera, Natalia Pérez Romero, Álvaro Pérez Rubio, L Pérez Santiago, Leticia Pérez Santiago, V Pérez Simón, Rafael Perez Vidal, J Perez Villena, Joan Perez Villena, S Perez-Bertolez, Sonia Pérez-Bertólez, H Perez-Chrzanowska, Hanna Perez-Chrzanowska, B Perez-Lozana, B Pérez-Saborido, Baltasar Pérez-Saborido, LE Pérez-Sánchez, A Perfecto, Ilaria Pergolini, V Peri, N Periard, Ivan Perić, S Pericleous, Marcos Perini, R Perinotti, Roberto Perinotti, Nuria Peris, Gordan Perišić, D Peristeri, K Perivoliotis, Konstantinos Perivoliotis, C Perkins, R Perkins, Romeeka Perkins, G Peros, Georgios Peros, LO Perotto, M Perovic, T Perra, Teresa Perra, Konstantinos Perreas, Alexandra Perricos, D Perrina, AM Perrone, Fabrizio Perrone, G Perrone, Gennaro Perrone, Ophelie Perrot, M Perry, A Persad, Amit Persad, Roberto Persiani, M Pertea, D Pertile, Davide Pertile, Abirami Perumal Kanniappan, A Perutelli, Arsalan Pervaiz, A Peryt, F Pesant, MA Pesántez Peralta, A Pesce, Vito Pesce, T Peschel, Giovanni Pesenti, Vishal Peshattiwar, Laura Pesquera, P Pessaux, Patrick Pessaux, Maximilian Pesta, Amit Peswani, L Petagna, Lorenzo Petagna, M Peteja, Matúš Peteja, Chidiebere Peter Echieh, Osborne Peter Vaz, Luke Peters, Paul Peters, SM Peters, ML Petersen, Josefin Petersson, Stamatios Petousis, F Petraglia, Nikki Petrakis, Donatas Petrauskas, Konstantin Petrenko, A Petrillo, Marco Petrillo, P Petrone, Konstantinos Petropoulos, R Petrov, Biljana Petrovic, Milan Petrovic, Alexandru Petrusan, Bogdan Petruț, G Petruzzi, Gerardo Petruzzi, Andrea Petzold, M Peycelon, Inês Peyroteo, Mahdi Pezeshki Modarres, I Pezzoli, F Pezzolla, AP Pezzuto, AC Pfaff, Carlos Pfingst Rojas, K Pfister, Karin Pfister, J Pfuner, A Phadnis, Ashish Phadnis, H Pham, Hong Pham, Terence Pham, Du Phan, YC Phan, Isaac Phang, Ajay Philip, Ken Philip, Melissa Philip, T Philip, Mark Philip Hehir, Anupam Phillip, A Phillips, AW Phillips, Drew Phillips, Edward Phillips, Emil Phillips, J Phillips, Jonathan Phillips, JR A Phillips, Rachel Phillips, G Philouze, M Philp, J Phull, Daniel Phung, Athanasios Piachas, R Piagnerelli, C Piazza, M Piazza, AL Picardo, Antonio L Picardo, Miriana Picariello, S Picazo, Eduardo Picazo Pineda, Fernando Picazo Pineda, Andrea Picchetto, A Picciariello, Arcangelo Picciariello, M Piccino, M Piccirillo, Micaela Piccoli, C Piceni, Merycarla Pichardo, Barbara Pichi, C Pichler de Oliveira, Cora Pichler de Oliveira, H Pickard, L Pickering, L Pickett, Héctor Picon molina, R Picón Rodríguez, Rafael Picón Rodríguez, Emanuele Picone, V Picotti, Veronica Picotti, Samuel Pie, Emil Pieniowski, Alberto Pieretti, F Pieri, Stefano Piero Bernardo Cioffi, Trocard Pierre, G Piessen, Guillaume Piessen, L Pieteris, Linas Pieteris, A Pietramala, G Pietrobon, Giacomo Pietrobon, N Pigadas, Manon Pigeolet, G Pignata, L Pignataro, Lorenzo Pignataro, M Pignatti, Marco Pignatti, Lucinda Pigott, N Pijanovic, Nemanja Pijanovic, C Pijoan-Lara, E Pikoulis, Elima Pilar Cagigal Ortega, María Pilar Camacho Carrasco, Claudia Pilar Clemente Tomas, Adam Pilarski, I Pilic, I Pilkington, Silja Pillai, Srikumar Pillai, Nivashen Pillay, Robert Pillerstorff, J Pilling, M Piloni, Martina Piloni, G Pilu, Gianluigi Pilu, AS Pimienta Ibarra, T Pina-Vaz, Teresa Pina-Vaz, Ugo Pinar, Camila Pincheira, S Pincott, Anna Pineau, Esther Pinfold, Hock Ping Cheah, L Pingarrón-Martin, Gabriela Pinheiro, J Pinheiro Santos, S Pinho, Sílvia Pinho, RE Pinilla, RE Pinilla Morales, Muni Pinjala, Veronica Pino Diaz, E Pinotti, Enrico Pinotti, Agustin Pinsak, Amanda Pinto, Fabio Pinto, J Pinto, José Pinto, Pilar Pinto, V Pinto, Valentina Pinto, Victor Pinto Angulo, VM Pinto-Angulo, José Pintor-Tortolero, Celestino Pio Lombardi, Clarence Pio Rey Yacapin, A Pipara, Amrit Pipara, RJ Piper, NS Pipitone Federico, Mirzemagomed Pirakhmedov, Antonio Piras, Kanapathipillai Piratheep, Franco Piredda, Erick Pires Ferreira, Ivonizete Pires Ribeiro, Alessandro Pirina, Setareh Pirmorad, Francesca Pirola, GM Pirola, Laszlo Piros, Nello Pirozzi, G Pirozzolo, Giovanni Pirozzolo, Roberto Pirrello, A Pirzada, Darryl Pisani, Michele Pisano, A Pisanu, Adolfo Pisanu, F Pisanu, Francesco Pisanu, Givi Pisarevi, Lorenz Pisecky, E Pişkin, Erol Pişkin, T Pissanou, Meron Pitcher, Joel Pitkänen, L Pitoni, Bradley Pittam, Idoko Pius Ogolekwu, MJ Pizarro, P Pizzini, Kerasia-Maria Plachouri, Puneet Plaha, Jordi Planelles Gómez, Carole Plante, Marie Plante, Philipp Plarre, Maria Plata, Julio Plata-Bello, Rebecca Platoff, Guillermo Plaza, Cristian Plaza Valiente, Stefan Plontke, J Ploski, Jennifer Ploski, Katherine Plua, Artem Pobelenko, José Poblete Carrizo, Marc Pocard, Adriana Poch, P Pockney, Madalina-Claudia Pocol, M Podda, Mauro Podda, P Poddar, Guillaume Podevin, D Podolsky, R Poelstra, C Poggi, Catalina Poggi, J Poggio, G Poggioli, Gilberto Poggioli, Elia Poiasina, Bernhard Poidinger, Nikolaus Poier, Harshwardhan Pokharkar, Martha Poku, L Pol-Fachin, Laercio Pol-Fachin, G Pola Bandres, Guillermo Pola Bandres, Verónica Polaino, Lucía Polanco Pujol, CA Polania Sandoval, S Polat, Süleyman Polat, Natasa Poldan Grabar, E Poletto, T Poli, A Police, Andrea Police, Antonella Polimeni, A Politi, Esteban Politi Vidal, C Politis, D Politis, Dimitrios Politis, S Politis, S Pollesel, Tommaso Pollini, JM Pollok, E Poluyi, Andreas Polydorou, Leo Pölzl, C Pompili, Cecilia Pompili, Luca Ponchietti, Sabarirajan Ponnusamy, Aleix Pons Bartroli, L Pons Pellicé, Alex Ponson, Jose Pontes Junior, N Ponugoti, Nikhil Ponugoti, Rita Poon, I Pop, I Popescu, Irinel Popescu, SG Popeskou, A Popoola, Ademola Popoola, D Popova, Maria Popp, Francesco Porcelli, A Porcu, Alberto Porcu, Bálint Pordány, Andrea Porta, L Porteiro Mariño, Lucia Porteiro Mariño, Carmen Portenkirchner, Anna Porter, AL Portilla, M Portinari, Vlad Porumb, JA Posada, OE Posadas-Trujillo, T Poskus, Tomas Poskus, L Posma-Bouman, Lisanne Posma-Bouman, N Post, Spyridon Potamianos, Ojas Potdar, Amit Pothare, Stojan Potrč, R Potter, Ryan Potter, A Potts, S Pou Macayo, Frederic Pouliot, T Poulton, R Pourahmad, M Pourfridoni, S Pourhedayat, Arvid Pourlotfi, D Pournaras, Dimitri Pournaras, Andrés Pouy, Andrej Povalij, S Powell, Simon Powell, E Powell-Smith, Nicholas Power, Dilroop Poyyil, Carolina Pozo, P Pozo Quispe, Ioanna Pozotou, Mirko Pozzoni, Jorge Prada, GM Prada Hervella, IS Pradeep, C Praetorius, Christian Praetorius, Mark Praetorius, Prabhat Prakash Narayan, Sankhya Prakash Vel, C S Pramesh, CS Pramesh, B Pramodana, L Prantl, Lukas Prantl, Aveechal Prasad, Navakoti Prasad, Shalvin Prasad, Sathyamoorthy Prasanna, Nil Prat, J Prat Ortells, Jordi Prat-Ortells, Dedy Pratama, D Prce, C Predoi, R Preece, Ryan Preece, S Pregnolato, Y Premakumar, AB Prempeh, Agya Prempeh, J Presl, Jaroslav Presl, Michael Preston, AL Preto Barreira, J Pretorius, Drago Prgomet, Martin Přibyl, Benjamin Price, C Price, Cheri Price, T Price, Thea Price, Veronique Price, Ruth S Prichard, M Priemel, Matthias Priemel, M Prieto, Mikel Prieto, M Prieto Calvo, MI Prieto Nieto, Rodrigo Prieto-Aldape, N Prijović, Nebojša Prijović, Florian Primavesi, Heather Pringle, S Pringle, Shirley Pringle, R Pritchard, Anna Privratsky, Noopur Priya, Vijayam Priya Nair, Pratyusha Priyadarshini, Priyadarshini Priyadarshini, Affan Priyambodo, Sujeewa Priyantha Bandara Thalgaspitiya, E Proaño, JA Proaño-Zamudio, Chris Probst, P Probst, A Prodromidou, Anastasia Prodromidou, A Pronk, Alejandro Prosperi, P Prosperi, Paolo Prosperi, Doriane Prost, M Protic, PL Proto, D Proud, David Proud, Arina Provozina, Ratko Prstacic, Andreja Prtorić, GM Prucher, V Pruiti Ciarello, Vincenzo Pruiti Ciarello, Lydia Prusty, FR Pruvot, C Pryce, Michail Psarologos, Dimitris Psychogios, Ning Pu, Marco Puccini, Caterina Puccioni, P Puchwein, Paul Puchwein, Emma Puertas Ruiz, Angela Puerto, A Pueyo Ferrer, Alfredo Pueyo Ferrer, Alberto Pueyo Rabanal, Jonathan Pugh, Pierfrancesco Pugliese, M Puglisi, JJ Puig Galy, Marcel Pujadas, Antonio Pujante, Natalia Pujol Cano, R Pujol Muncunill, Rosa Pujol Pina, N Pujol-Cano, R Pujol-Muncunill, H Pülat, Eliana Pulido, C Puma Pagliarello, Calogero Puma Pagliarello, Rodriguez Pumarol Próspero Enrique, Oliver Pumphrey, Diana Puozaa, A Puppo, Andrea Puppo, Ajay Puri, A Purohit, Irina-Maria Puscas, Silke Pusch von, J Pushpa-rajah, Kameshwarachari Pushpalatha, S Putnis, Soni Putnis, Vadim Pykhteev, Amjad Qabbani, Kawthar Qader, S Qaderi, MA Qadri, Shahin Qadri, ST Qadri, Qutaiba Qafisha, Mubasher A Qamar, H Qandeel, Haitham Qandeel, Mahmoud Qandeel, Layth Qaraqe, A Qasem, Abdulrahman Qasem, Ahmad Qasim, Dalia Qasrawi, Faisal Qassem, Mohamed Qassem, MS Qatora, Abid Qazi, Mehdi Qiabi, Cheng Qian, J Qiao, Kirby Qin, KR Qin, Joo Qing Cheng, Alex Qinyang Liu, Yaman Qoudra Danial, Helen Quah, Kofi Quansah, M Quante, Markus Quante, Elizabeth Quartson, Mohamad Qudah, B Quddus, G Querini, J Querolt Coll, Jordi Querolt Coll, R Quevedo, Julien Quilichini, Ralph Quillin, Andrew Quin, Eduardo Quiñónez Lorenzana, E Quint, M Quintana, Natalia Quintana, B Quintana-Villamandos, Begoña Quintana-Villamandos, J Quintens, Ma Andrea Quintero-Ortíz, Claudia Quintero-Pérez, Saray Quinto, Valeria Quintodei, Ned Quirke, Sinead Quirke, Raquel Quiroga, Manuel Quiroz, Kewin Quispe de la Roca, JF Quispe Mateo, Zaki Qulaghassi, Yazen Qumsiyeh, Meysoon Qurashi, Homeira Qureischie, A Qureishi, A Qureshi, Ahmad Qureshi, AU Qureshi, Maryum Qureshi, Sajid Qureshi, I R Fakhradiyev, P R Oliveira, A Rababah, Asmaa Rababah, Lorenzo Rabadan, Mahtab Rabbani Anari, Mohamed Rabea, Mohamed Rabie, Salma Rabie, Igor Rabin, Will Raby-Smith, Louie Racelis, J Rachadell, Juan Rachadell, Nur Rachmat Lubis, M Racine, D Radenkovic, Dejan Radenkovic, A Radhakrishnan, Ajay Radhakrishnan, Mohamed Radhi, M Radojevic, Zeljko Radojkovic, Rudolf Radojković, M Radosavljevic, Milena Radosavljevic, Ishan Radotra, Dragana Radovanovic, Zoran Radovanovic, Dana Radu, Pisica Radu-Mihai, O Radulova-Mauersberger, Asmaa Radwan, Emily Rady, M Rady, Fraser Rae, L Rae, Hazim Raed, Marianna Raevskaya, Henry Rafael Acosta Castro, Mario Rafael Medina Hernández, Tiago Rafael Onzi, Tanvir Rafe, A Raffaele, A Raffone, Yaseen Rafi, A Rafique, Atif Rafique, MN Rafique, Abdelrahman Ragab, Mohamed Ragab, Marawan Ragal, Mahmoud Raggad, Vidya Raghavan, Franco Ragni, K Ragupathy, Kalpana Ragupathy, Casimir FP Rahantasoa Finaritra, N Rahbari, Nuh Rahbari, F Rahim, Ferdous Rahim, Rehana Rahim, Sybghat Rahim, M Rahimi, Mana Rahimzadeh, Atiqur Rahman, Ayesha Rahman, GA Rahman, Khalid Rahman, M Rahman, Mohammad Rahman, R Rahman, S Rahman, Abdel Rahman Ashraf, A Rahman Mitul, Ashrarur Rahman Mitul, Abdel Rahman Mohannad Ahmad Alwardat, Faizan Rahmani, Yazan Rahmeh, AC Rahy-Martín, A Rai, B Rai, DAS Rai, L Rai, Lajpat Rai, S Rai, D Raimondo, Diego Raimondo, I Raimondo, Christopher Raine, Ryan rainiel Abary, Amit Raithatha, N Raj, Sumit Raj, Mohana Raj Thanapal, H Raja, Nancy Raja, Roopak Raja, K Raja Shabbir, Parisa Rajaei, Niranjana Rajagopal, P Rajagopal, A Rajagopalan, Saravanan Rajakumar, Firoz Rajan, S Rajan, Shiv Rajan, A Rajanbabu, Anupama Rajanbabu, Tsirimalala Rajaobelison, K Rajaratnam, N Rajaretnam, Niroshini Rajaretnam, JS Rajasekar, Mohsen Rajati, Kishan Rajdev, Srujan Rajesh, AD Rajgor, Amarkumar D Rajgor, HO Raji, A Rajpura, Asim Rajpura, Deepak Rajput, K Rajput, Sunil Rajput, D Raju, Mohamad Rakka, K Rakoczy, Gergely Rakos, Haritiana Rakotoarisoa, I Rakvin, A Raluca-Cristina, Apostu Raluca-Cristina, Ruchi Ram, Gautam Ram Choudhary, Dharma Ram Poonia, Jeewan Ram Vishnoi, Nuno Rama, Abdelrahman Ramadan, Dina Ramadan, Rana Ramadan, Sara Ramadan, MA Ramadhan, Pooja Ramakant, Aravindh Ramalingam, I Ramallo-Solís, Irene Ramallo-Solís, R Ramamoorthy, Jaishankar Raman, Archana Ramaswamy, Kamarajan Ramayah, Makhmud Ramazanov, Sean Ramcharan, R Ramely, A Ramesh, Ashwanth Ramesh, P Ramesh Menon, S Ramezani, Maharo Ramifehiarivo, L Ramírez, Rossana Ramírez, Erika M Ramírez Amaya, P Ramirez Nieto, Pablo Ramirez Romero, LJ Ramirez-Nuñez, Nalla Ramji Narendra, S Ramjit, ARH Ramli, R Ramli, Roszalina Ramli, A Rammohan, Ashwin Rammohan, Arvind Rammohun, Catalina Ramon Barcelo, Juan Ramón Gómez López, Felix Ramon Montes, Jose Ramon Oliver Guillen, Juan Ramón Sanz, Carina Ramos, CL Ramos, D Ramos, German Ramos, Kirsten Ramos, MFKP Ramos, Nuno Ramos, P Ramos, Patricia Ramos, Pedro Ramos, R A Ramos, Rodrigo Ramos, A Ramos Bonilla, Antonio Ramos Bonilla, A Ramos De La Medina, Antonio Ramos De La Medina, Raul Ramos mange, Andrea Ramos Mantilla, JL Ramos rodriguez, A Ramos-De la Medina, Antonio Ramos-De la Medina, Adolfo Ramos-Luengo, P Ramos-Martin, Ritika Rampal, Ganeshan Ramsamy, Akash Ramsaroop, L Ramsay, W Ramsey, Shivanand Ramsubhagh, J Ramzi, Ahmad Ramzi Yusoff, Eyal Ran Nachum, Reesha Ranat, Probhodana Ranaweera, Alin Rancea, Jonathan Randall, T Randau, Thomas Randau, A Rangan, Amar Rangan, Hardlife Ranganai, J Rani, Pallavi Rani, M Ranisavljević, Jyothi KR Ranjan, Mark Ranjan Jesudason, Smruti Ranjan Mohanty, Gyan Ranjan Singh, Kul Ranjan Singh, S Ranjit, Srinath Ranjit, Anuradha Rao, Dominic Rao, Milind Rao, Rohith Rao, Suresh Rao, Nayzak Raoof, Sarah Rapaport, F Rapetto, Filippo Rapetto, J Rapp, DA Raptis, C Rarras, Mamisoa B Rasamoelina, Hanta Rasataharifetra, Abdallah Rashad Temerik, Yasir Rasheed, I Rashid, Isbah Rashid, Madeeha Rashid, MM Rashid, Pueya Rashid Nashidengo, M Rashidbeygi, Mohammed Rashwan, Slobodan Rašić, Hasan Raslan, Fanjandrainy Rasoaherinomenjanahary, Iqbal rasool Wani, Karolina Rasoul-Pelińska, F Raspagliesi, Francesco Raspagliesi, Melroy Rasquinha, J Rassam, R Rasschaert, Ricky Rasschaert, M Rassweiler-Seyfried, Marie-Claire Rassweiler-Seyfried, Pejana Rastović, Sebastian Rath, S Rathinam, Sridhar Rathinam, Kirtikumar J Rathod, Tõnu Rätsep, R Rattan, Rishi Rattan, Pornjittra Rattanasirivilai, Francesca Ratti, Deviney Rattigan, S Rattizzato, Simone Rattizzato, C Ratto, Carlo Ratto, Varkha Rattu, K Raubenheimer, Kyle Raubenheimer, Teresa Rauchegger, F Rauf, Yaseen Rauf, Abdul Rauf bin Ahmad, Diego Raul Abente Arriola, Jordi Raurich-Leandro, E Rausa, C Raut, Chandrajit Raut, CP Raut, M Raut, Monish Raut, M Ravaioli, Matteo Ravaioli, S Raveendran, G Ravenni, Selina Ravenscroft, Akshaya Ravi, N Ravi, S Ravindrakumar, S Ravindran, R Ravindranath Nambiar, David Rawaf, Faisal Rawagah, Arab Rawashdeh, Shireen Rawashdeh, J Ray, Jaydip Ray, S Rayamajhi, T Raymond, A Rayner, Anthony Rayner, Tom Rayner, Fadi Rayya, M Rayzah, Musaed Rayzah, Ahsan Raza, Ali Raza, M Raza, N Raza, Syed Raza, Waqas Raza, Ali Raza Malik, Jeannie BA Razafindrahita, Mohd Razali Ibrahim, A Razik, Naila Raziq, Syed Raziuddin Biyabani, Ahmad Razouk, Radu Razvan Scurtu, Hassan Razvi, Alessandra Razzaboni, A Razzore, Fatima Razzouk, F Ré, Matthew Read, Pedro Recabal, Daniel Rech, Ewa Rechberger, G Recinos, Gustavo Recinos, Salvador Recinos, Adrián Recio Ayesa, F Recker, Florian Recker, A Recordare, Souheil Reda, Mohamed Reda Loaloa, Madhuri Reddy, Saiesh Reddy Voppuru, Roberta Redfern, Rajeev Redkar, NOT Rees, J Reeves, Motasem Refaat, Mohammed Refaat Ibrahiem Amin El Ghalid, Basel Refky, Daniela Rega, Trina Regalado, Guillermo Regalo, Carmen Regan, N Regenet, Nicolas Regenet, V Reghuram, Carlos Régil, Maria Regina Alvarez, Lia Regina de Sampaio, J Reguera-Rosal, Abdul Rehman, Hina Rehman, K Rehman, Riaz RehmAn, S Rehman, Abdur Rehman Malik, Martin Reichert, Daniel Reichhold, J Reid, Jennifer Reid, Jeremy Reid, Matthew Reid, Rebecca Reid, S Reid, J Reilly, John-Joe Reilly, D Reim, Daniel Reim, Daniel Reimer, Dietmar Reinaldo, T Reinhard, Tobias Reinhard, C Reinke, Caroline Reinke, F Reinkemeier, FJ Reinoso, I Reis, Igor Reis, ME Reis, Janani Reisenauer, Anne Reiss Axelsen, C Reissfelder, Christoph Reissfelder, A Reiter, Christian Reiterer, M Rela, Mohamed Rela, M Reljic, Milorad Reljic, Panagiota Rellia, J Relwani, Jai Relwani, Xabier Remirez Arriaga, Dong-Lin Ren, Gabriel Renan Soares Rodrigues, M Renau-Cerrillo, Marina Renau-Cerrillo, Elizabeth Renaud, Annaëlle Renault, Luke Render, Vladimir Rendevski, Juliana Rendón Hernández, Walter Rene Fretes Gonzalez, Mauricio Rene Hernandez, Agustina Rene Oliva, Herald Rene Segovia Lohse, Teresa Renedo Villar, A Renne, Sarah Rennie, M Rennis, P Renovell Ferrer, Pablo Renovell Ferrer, E Renza-Stingone, Adolfo Renzi, Islamic Rep, Luca Resca, E Restini, Enrico Restini, Joel Reuben Abel, T Revez, Tatiana Revez, Remedios Revilla Amores, Julia Revuelta Ramírez, Rocio Revuelta Zorrilla, MC Rey, S Rey, C Rey Valcarcel, Cristina Rey Valcarcel, J Rey-Biel, C Rey-Valcarcel, Faten Reyad Bani Hamad, Carmen Reyero Fernández, Emilio Reyes, GP Reyes, JA S Reyes, JAS Reyes, JT Reyes, Rudeily Reyes, Albert Reyes Claret, Adriana Reyes Echeverría, MDC Reyes Puig, E Reyes Rodriguez, Carlos Reyes Utrera, IS Reynolds, J Reynolds, JV Reynolds, C Reynoldson, Charmian Reynoldson, M Rezacova, M Rezaei Tavirani, D Rezaie, Daniel Rezaie, Esmaeil Rezghi Maleki, Mohamed Rezk, M Rghioui, Mounir Rghioui, Jae Rhee, HL Rhodes, M Riad, Mahmud Riad, Xiana Rial, Romualdas Riauka, Kazim Riaz, Sidra Riaz, Luisana Riba Combatti, M Riba Martinez, Mireia Riba Martínez, A Ribeiro, Ana Ribeiro, Barbara Ribeiro, J Ribeiro, Margarida Ribeiro, R Ribeiro, Ricardo Ribeiro, Rui Ribeiro, T Ribeiro, VI Ribeiro, U Ribeiro Jr, U Ribeiro Junior, R Ribeiro Meduna, Rafael Ribeiro Meduna, M Ribolla, D Ribuffo, Diego Ribuffo, Guijarro-Jorge Ricardo, David Ricardo Herrera Mora, José Ricardo Negrete Ocampo, Luis Ricardo Ramirez Gonzalez, Andre Ricardo Stüker, Vincenzo Ricchiuti, Claudio Ricci, S Ricci, Silvia Ricci, Pietro Ricciardi, Sara Ricciardi, G Riccioli, Alexandra Rice, D Rice, HE Rice, Henry E Rice, SE Rice-Townsend, Harvey Rich, Dylan Richard Barnett, Stephen Richard Knight, J Richards, S Richards, T Richards, Toby Richards, P Richebé, J Riches, MC Richir, Lysia Richmond, M Richmond, E Richtig, Francesco Ricotta, Mohammed Rida, Sophie Riddell, GE Riddiough, Georgina Riddiough, A Ridgway, Paul Ridgway, PF Ridgway, Johannes Riecke, P Riedl, M Riehan, Caroline Rieser, John Rietveld, Ebaa Rifai, O Riffi, J Rigaud, Jerome Rigaud, E Righini, Caitlin Rigler, L Rigueros Springford, Karolin Riips, Sushil Rijal, C Riley, Christopher Riley, Lara Rimmer, R Rimonda, Roberto Rimonda, Mauro Rinaldi, MN Ringressi, J Rio, Javier Rio, L Rio Rodrigues, July Ríos, J Rios Chiuyari, Jose Rios Chiuyari, AJ Rios-Diaz, Gabriel Ríos-Samper, Victor Ripardo Siqueira, Brianda Ripoll, Francisco Ripoll Vidal, J Ripollés-Melchor, Javier Ripollés-Melchor, F Ris, M Risaliti, Mohamed Rishard, OB F Risk, Razan Rislan, Stefan Riss, A Rissmann, Anke Rissmann, T Risteski, Anna Rita Tanca, Ana Rita Teles, Otávio Ritter Silveira Martins, Tiago Riuji Ijichi, FD Rivadeneira Proano, D Rivas, F Rivas, J Rivas, Julio Rivas, R Rivas, Ruben Rivas, Barbara Rivera, Pablo Rivera, RD Rivera, Teresa Rivera Schmitz, D Rivera-Alonso, Joaquín Rivero Déniz, Anas Riyahi, H Riyat, M Rizk, S Rizvi, Andrea Rizzi, Davide Rizzo, Roberta Rizzo, V Rizzo, Angelica Rizzoli, Ahmad Rmman, Justin Roake, Jun-Neng Roan, CE Roata, D Robayo, Lydia Robb, C Robba, Matthew Robert Marples, P Roberto andrea, Miguel Roberto Li Valencia, Luis Roberto Nadal, Juan Roberto Torres Cisneros, Carwyn Roberts, D Roberts, Jayson Roberts, Keith Roberts, L Roberts, Laura Roberts, M Roberts, Matthew Roberts, Tobias Roberts, Phoebe Robertson, R Robertson, S Robertson, Vaila Robertson, B Robertson-Smith, Fabien Robin, Nicole Robin, A Robin Valle de Lersundi, Alvaro Robin Valle de Lersundi, B Robinson, RJ Robitsek, Carlos Robles Vidal, A Robson, ML Robuschi, Nicole Robyn Bangayan, Andrea Roca, A Rocca, M Rocha Melo, Miguel Rocha Melo, João Rocha-Neves, EC Roche, Emny Rochell Bobadilla Romero, M Rochon, Thelma Rocío Jiménez Mosquea, Peter Rock, Alejandra Rodas, Lesly Rodas, S Rodimov, Sergey Rodimov, K Roditis, Konstantinos Roditis, K Rodkey, A Rodolakis, Alexandros Rodolakis, Luis Rodolfo Bonilla, Josep Rodoreda, Laura Rodrigáñez, D Rodrigo, VS D Rodrigo, Walter Rodrigo Martínez Torres, José Rodrigo Oliva, Catarina S Rodrigues, Josy Rodrigues, M Rodrigues, Rodrigo Rodrigues, SC Rodrigues, Sónia Rodrigues, ML Rodrigues Barbosa da Silva, Juliano Rodrigues da Cunha, C Rodriguez, Camilo Rodriguez, E Rodriguez, Eduardo Rodriguez, J Rodriguez, JL Rodriguez, Juan Rodriguez, Juliana Rodriguez, Kenny Rodriguez, Natalia Rodriguez, Ada Rodríguez, Carballo Rodríguez, Araceli Rodrìguez, RDLC Rodríguez Ciria, Jaime Rodríguez de Alarcón, MI Rodriguez Fernandez, A Rodriguez Fraga, L Rodríguez Gómez, Lorena Rodríguez Gómez, Agustin Rodriguez Gonzalez, A Rodríguez Gonzalez, Carmen Rodríguez Haro, A Rodriguez Infante, Antonio Rodriguez Infante, Cedillo Rodrìguez Jonathan Rubén, JN Rodriguez Niño, Lizeth Rodriguez Sanchez, Ana Rodríguez Sánchez, Leticia Rodriguez Vaquero, J Rodriguez-Abreu, Julia Rodriguez-Abreu, Mario Rodriguez-Lopez, Ana Rodríguez-Tesouro, Raquel Rodríguez-Uria, H Rodriguez-Zentner, Homero Rodriguez-Zentner, J Rodriquez, Jennifer Rodriquez, Anne-Jasmin Roelofs, Judith Roesch, Nicolo Roffi, A Rogers, L Rogers, LJ Rogers, Luke Rogers, M Rogers, S Rogers, Pat Rohan, Himanshu Rohela, S Rohleder, Andrej Roj, A Rojas Aguilar, Antonio Rojas Aguilar, Fabian Rojas Portilla, Yesenia Rojas-Khalil, DA Rojas-Tejada, J Rojas-Ticona, Javier Rojas-Ticona, Gheorghe Rojnoveanu, Alicia Rojo, JA Rojo Lopez, AC Rokohl, Christina L Roland, CL Roland, Pedro Roldan Ramos, Marta Roldón Golet, Dirk Rolf Bulian, J Rolinger, Jens Rolinger, P Rolland, Udo Rolle, RA Rollett, Rebecca Rollett, L Rolli, Luigi Rolli, G Rollo, A Rolls, Catarina Rolo Santos, Guilherme Roloff Cardoso, Aristide Romain Raherison, E Romairone, David Roman, Laura Román García de León, Angela Romano, M Romano, Maurizio Romano, Vito Romano, John Romany, A Romanzi, Sèmèvo Romaric Tobome, Adrian Rombach, Esmeralda Romero Bañuelos, Alejandro Romero de Diego, CS Romero Garcia, Fernanda Romero Lechuga, CA Romero Manqui, Guillermo Romero Reyna, E Romero-Bañuelos, A Romero-De Diego, Ivan Romić, O Rominiyi, Ola Rominiyi, Saleh Romman, F Ron, Michael Ron Freund, Arturo Roncone, U Ronellenfitsch, Ulrich Ronellenfitsch, DA Ronquillo Andrade, L Rony, J Rooney, Joanna Rooney, S Rooney, Ryan Roopnarinesingh, Oscar Roque, F Rosa, Fausto Rosa, Isabel Rosa Fernández Burgos, Maria Rosa Ortiz, Lilia Rosa Reyes Guilamo, Rember Rosales Arriola, M Rosario, Patricio Rosas, C Rosas bermudez, R Rosati, Riccardo Rosati, F Rosato, Fernando Rosatti, C Rösch, J Rose, Matthew Rose, Nantambi Rose, Emma Rose Michelle Woolcock, A Roselló Añón, Alejandro Roselló Añón, Lauren Rosenblum, M Rosengart, Kari Rosenkranz, SM Roser, Steven Roser, Vittoria Rosetti, dolors Rosines Cubells, M Rosines Cubells, Aida Rosita Tantri, A Roslani, AC Roslani, April Roslani, Carlo Ross, E Ross, Fiona Ross, H Ross, L Ross, Lauren Ross, S Ross, Samuel Ross, Rolf Rossaint, Ayane Rossano, C Rossborough, Domenico Rossi, G Rossi, Giulia Rossi, Gustavo Rossi, L Rossi, Luciano Rossi, S Rossi, Serena Rossi, Settimio Rossi, V Rossi, Vanessa Rossi, Kayla Rossini, Matteo Rossini, Tobias Rossmann, Andres Rosso, Klara Rosta, T Rostkowski, J Roszpopa, A Roth, Andreas Roth, Nicole Rotter, M Rottoli, Matteo Rottoli, Nicholas Roubos, Amy Round, Morgan Roupret, O Rousan, Bikram Rout, S Rout, T Routledge, Alexia Roux, Frederic Roux, Marco Rovagnati, F Roviello, Franco Roviello, S Roward, Alistair Rowcroft, Alberto Roxas, V Roxo, Vanessa Roxo, Ashutosh Roy, C Roy, H Roy, Jennifer Roy, Nathalie Roy, S Roy Mahapatra, Sunanda Roy Mahapatra, T Royle, AR Royson, A Różańska-Walędziak, F Rozet, Balazs Rózsa, Sophie Rozwadowski, Ana Ruano, Adriana Ruano Campos, Michele Rubbini, Sonia Rubbo, Wilson Rubiano, Nina Rubicz, K Rubin, E Rubio, Enrique Rubio, Mercedes Rubio Manzanares Dorado, J Rubio-Palau, Josep Rubio-Palau, I Rubio-Perez, Ines Rubio-Perez, Irina Rudenko, M Rudic, Milan Rudic, Gareth Rudock, Victoria Rudolph-Stringer, Agris Rudzāts, Mario Rueda, M Ruel, SR Rufai, Enrico Ruffini, G Ruffo, Giacomo Ruffo, Anya Rugendyke, F Ruggiero, Silvia Ruggiero, M Ruhosha, Manuel Ruiss, A Ruivo, G Ruiz, I Ruiz, Fernanda Ruiz de Andrade, FR Ruiz Echeverría, Alicia Ruiz Escobar, M Ruiz Esquide, F Ruiz Grande, Catalina Ruiz Lopez, I Ruiz Martin, Rebeca Ruiz Roman, María Ruiz Soriano, A Ruiz-Escobar, M Ruiz-Marín, Miguel Ruiz-Marín, Francisco Ruiz-Navarro, M Ruiz-Soriano, L Ruiz-Villa, Laura Ruiz-Villa, Jordi Rumià Arboix, Cristian Ruminot, M Rumyantseva, Stuart Rundle, Mohd Rusdi Draman, Loren Rushton, J Russ, E Russe, Elisabeth Russe, Christine Russell, Crispin Russell, N Russell, Neil Russell, Victoria Russell, Maria Russi, Davide Russo, Elena Russo, IS Russo, AO RUSU, M Rutegård, Martin Rutegård, D Rutherford, Daylen Rutledge, Michael Ruyssers, NM Ruzgar, A Ruzzenente, Andrea Ruzzenente, Oscar Rwego, Christopher Ryalino, É Ryan, Éanna Ryan, ÉJ Ryan, James Ryan, Jessica Ryan, Neil Ryan, O Ryan, William Ryan, Justyna Rymarowicz, O Ryska, Suganya S, Inês Sá, Claudia Saab, A Saad, Ebtesam Saad, Haisam Saad, Mahmoud M Saad, MM Saad, Moustafa Saad, Nader Saad, S Saad, Safaa Saad, Sanad Saad, Ahmed Saad Elsaeidy, Khaled Saad Elsaeidy, R Saadeh, A Saadi, Jose Saadi, A Saadya, Sarra Saaf, T Saafan, R Saaid, Rahmah Saaid, J Saavedra, N Sabanovic Bajramovic, Marta Sabater-Martos, Luciana Sabatini, Abdulrahman J Sabbagh, Danielle Sabella, A Saber, R Saberi, Rebecca Saberi, Z Saberi, Predrag Sabljak, Angelin Sablon Herinirina, Arfa Saboor, E Sabouri, Yasser Sabr, Joseph Sabra, R Sabri, Nur Sabrina Binti Babe Azaman, A Sabry, Ahmed Sabry, Aya Sabry, Hesham Sabry, MD P Sacdalan, Rekha Sachan, B Sachdev, Bobby Sachdev, A Sachdeva, Sanket sadanand Shetty, F Sadat Rahimi, Anwar Sadat Seidu, Mohammed Saddik, M Sadek, Saravanan Sadhasivam, Haleema Sadia, Uzma Sadia, H Sadian, P Sadigh, A Sadioğlu, Amr Saeed, B Saeed, Bashayer Saeed, Kareem Saeed, Komal Saeed, M Saeed, Marwah Saeed, Rafeh Saeed, Ridwan Saeed, S Saeed, Summaya Saeed, U Saeed, Waqar Saeed, Y Saeed, Yousif Saeed, Umer Saeed Haroon, Faisal Saeed Hassan, Sawsan Saeid, Somcharoen Saeteng, Manuel Saez barba, P Saez Carlin, Patricia Saez Carlin, Maialen Saez de Vicuña Salinas, EM Sáez-Cerezal, Elena Sáez-Ruiz, Mustafa Safa Uyanik, Bassem Safadi, H Safari, M Safari, Seyer Safi, Najib Safieddine, Fanonjomahasoa Safiry Andofenohasina, Sergej Safonov, Mohammad Safri, György Saftics, Hadeer Safwat, J Sagar, Jayesh Sagar, Sushma Sagar, R Saghir, A Sagnotta, Andrea Sagnotta, Robert Sagoe, Sunita Saha, Arun Sahai, Egbal Sahal Abdelmajed, Zeynep Şahan Çeti̇nkaya, Inci Sahin, Can Şahin, R Şahin, Abat Sahlu, K Sahnan, Kapil Sahnan, A Sahni, Anjana Sahu, Arnav Sahu, Banchhita Sahu, Rabi Sahu, AM Saibene, M Said, Yasmeen Said, A Said Bayazeed, Skender Saidi, Tim Saier, Sara Saif, S Saifuddin, Altanchimeg Sainbayar, Fani Saini, T Saini, Thomas Saini, A Sainz Lete, M Sait, Salma Sait, Junichi Saito, Ryoichi Saito, Takuya Saito, Tomohito Saito, Miguel Saiz Sánchez-Buitrago, Hannan Sajid, Zaina Sajid, Y Sakaray, R Saket, Rawand Saket, Orazbek Sakhov, Rajendra Sakhrekar, Mehmet Sakinci, A Sakr, Ahmed Sakr, T Sakurai, Tomoe Sakurai, Laura Sala, Michael Sala, A Salah, Omar Salah, Alzhraa Salah Abbas, Amna Salam Al-Wandi, H Salama, Paul Salama, A Salamah, Abdulrauf Salamah, Sara Salamah, F Salameh, Michel Salameh, Mohammed Salameh, Babatunde Salami, Giuseppe Salamone, R Salas, Claudio Salas Garrido, E Salau, Eniola Salau, Adedayo Salawu, Hafeez Salawu, Nasiru Salawu, A Salazar, Adolfo Salazar, A Salazar-Tantaleán, DF Salcedo Miranda, RA Salcedo-Hernández, Rosa Salcedo-Hernández, DV Saldivar Ozan, Cesareo Saldivar Patiño, Danjuma Sale, A Saleem, Abdulaziz Saleem, Bushra Saleem, Humaira Saleem, Irfan Saleem, MA Saleem, Maleeha Saleem, Mohammad Saleem, O Saleem, Tayyaba Saleem, Samina Saleem Dojki, Mohamed Saleem Noor Mohamed, Aasim Saleemi, Ahmed Saleh, C Saleh, IA Saleh, Ibrahiem Saleh, M Saleh, Mahmoud Saleh, Mamoun Saleh, Mohannad Saleh, Abddulrahman Saleh Almulhim, Alhosen Saleh M Aldelensi Alzubi, Mona Saleh Mesbah Mohamed Elkaffas, M Salehi Shadkami, Mohammed Salele Aliyu, Alaa Salem, H Salem, Hani Salem, Marwa Salem, MC Salem, Moacyr Salem, Nourhan Salem, Osama Salem, Rima Salem, Amin Salem Ahmed Egdeer, Roaa Salem Jwaid Alneimat, G Salerno, R Sales, Inês Salgado, Wilson Salgado Jr, N Salgado-Nesme, Noel Salgado-Nesme, Shemsedin Salia, Timur Saliev, Mohammed Salihu, A Salim, Azra Salim, Hashem Salim, Radhwan Salim, Ruqyyah Salim, Shaharyar Salim, Armando Salim Munoz Abraham, Amrollah Salimi, A Salimi asl, S Salimoğlu, Semra Salimoğlu, JR Salinas Peña, S Salindera, D Salinovic, Thomas Salisbury, Kabiru Salisu, Stefano Salizzoni, A Sallam, Ahmed Sallam, Ali Sallam, Asser Sallam, I Sallam, Ibrahim Sallam, M Sallam, Mahmoud Sallam, Moataz Sallam, H Salle, Henri Salle, Mat Salleh Sarif, Salloum Salloum, Reem Salman, S Salman, Samar Salman, Muhammad Salman Farsi, Mohammad Salman Siddiqi, Enrique Salmerón-González, Bethan Salmon, M Salö, Ana Salomé Cavaleiro Leitão de Carvalho, Emily Salt, JA Salud, P Salunke, Pravin Salunke, Dhanshree Salunkhe, Ariadna Salvadó, Renato Salvador, Abegail Salvana, Maurizio Salvati, Maisa Salvetti, R Salvia, Roberto Salvia, R Salvioni, Roberto Salvioni, Garrett Salzman, Musore Sam, Samy Samaan, Zeljka Samac, Nilofar Samadi, Mustafa Samadony, E Samadov, Elgun Samadov, Rehab Samaka, Diana Samantha Gonzalez, A Samara, Athina Samara, Evangelia Samara, Mohammed Samara, Dharmabandhu N Samarasekera, DN Samarasekera, D Samaraweera, Dulan Samaraweera, E Samarut, S Sambhwani, Sharan Sambhwani, Daniele Sambucci, E Sambugaro, E Samed, Mohammad Sameer, Mostafa Sameh, Sarika Samel, Ashraf Samer, Mhamed Samer Alkhatib, Omar Sami, Ahmed Samih, Ahmed Samir, A Samir Abdelaal, Ahmed Samir Abdelaal, Ahmed Samir Farahat, Fuad Samir Lopez Fernández, L Samison, Ali Samkari, Suzette Samlalsingh, Sari Samman, G Sammarco, Giuseppe Sammarco, T Sammour, Tarik Sammour, KA Samo, II Sampaio da Nóvoa Gomes Miguel, M Sampaio-Alves, Mafalda Sampaio-Alves, GM Sampietro, Jack Sample, Inderpaul Samra, Mujuni Samson, Chathurika Samudani Dhanasekara, Diego Samudio, Abhishek Samuel, Gilbert Samuel, Habie Samuel, Odongo Samuel, Claudio Samuel Dóleo García, Thomas Samuel William Greensmith, M San Andrés, Joana San Anton, Carlos San Miguel, C San Miguel Méndez, D Sanabria, Daniel Sanabria, Aly Sanad, Aaron Sanchez, R Sánchez, Rosa Sánchez, G Sánchez Aniceto, Alejandro Sanchez Arteaga, Alvaro Sanchez Barrueco, L Sánchez Blasco, Laura Sánchez Blasco, F Sanchez Cabezudo Noguera, Fatima Sanchez Cabezudo Noguera, S Sanchez Cabús, Ernesto Sánchez Castillo, Ailén Sánchez Cruz, Isabel Sanchez Cuadrado, C Sánchez del Pueblo, Cristina Sánchez del Pueblo, Fátima Sánchez Fernández, A Sánchez Gollarte, Ana Sánchez Gollarte, J Sanchez Gonzalez, Javier Sanchez Gonzalez, R Sanchez Jimenez, A Sanchez Lopez, Anna Sánchez López, A Sánchez Mozo, Ana Sánchez Mozo, Alejandro Sanchez Pellejero, Barriga Sánchez Raquel, Marina Sánchez Robles, Carlos Sanchez Rodriguez, R Sanchez Salas, Miguel Sánchez Suárez, Cristina Sánchez Torralvo, Vanessa Sanchez Torrents, A Sánchez-Arteaga, Santiago Sánchez-Cabús, AB Sánchez-Casado, N Sanchez-Fuentes, S Sánchez-García, A Sánchez-Gómez, Andrés Sánchez-Gómez, TA Sánchez-Gómez, L Sánchez-Guillén, Luis Sánchez-Guillén, JI Sanchez-Mendez, D Sanchez-Pelaez, Daniel Sanchez-Pelaez, C Sanchez-Perez, M Sánchez-Robles, C Sánchez-Rodríguez, M Sanchez-Rubio, M Sánchez-Rubio, María Sánchez-Rubio, R Sanchez-Santos, Guillermo Sanchez-Villaseñor, P Sanchis, Antonio Sanchís López, J Sancho-Muriel, Jorge Sancho-Muriel, L Sanchon, Erdene Sandag, G Sandblom, Johannes Sander, M Sander, Michael Sander, J Sanders, Julie Sanders, L Sanderson, Gagandeep Sandhu, A Sandhya, Anu Sandhya, R Sandkamp, Richard Sandkamp, Laura Sandland-Taylor, Gursev Sandlas, Yael Sandler, Saleh Sandoughdaran, Camilo Sandoval, Hernan Sandoval, John Sandoval, M Sandoval, Mauricio Sandoval Tobar, Marco Sandoval vaez, Cristian Sandu, S Sane, Miguel Sanfeliu Giner, Vinita Sangai, C Sangani, M Sange, Andrea Sangheli, MJ Sangüesa, Burmaa Sanjaa, Shaikh Sanjid Seraj, Ghaidaa Sanjuq, Mohamad Sankari, Pushp Sankhwar, Satyanarayan Sankhwar, S Sankpal, AN Sanli, Anand Sanmugam, Angelino Sanna, Tsuyoshi Sano, K Sanserino, Carlos Santacruz, G Šantak, Goran Šantak, Roberto Santambrogio, Diana Santana, Eleazar Santana, R Santana Ortiz, Roman Santana Santana, M Santarelli, M Santas, MS Santero-Ramirez, Oscar Santes Jasso, G Santhirakumaran, Gowthanan Santhirakumaran, Alvaro Santiago LeMarie Guerra, Silvia Santiago Maniega, Manuel Santiago Mosquera Paz, JA Santibanez-Salgado, P Santillan-doherty, Patricio Santillan-Doherty, Mario Santinami, A Santini, AJ A Santini, Alasdair Santini, Matteo Santoliquido, OS Santonocito, A Santoro, Antonio Santoro, Giulio Santoro, BC Santos, Blanca Santos, E Santos, Ema Santos, Irène Santos, Jorge Santos, Jos´é Santos, L Santos, Marco Santos, P Santos, Patrícia Santos, PM D D Santos, R Santos, Rui Santos, SS Santos, Victor Santos, Tainá Santos Bezerra, Pilar Santos Cidon, Marta Santos Espí, Paulo Santos-Costa, H Santos-Sousa, Hugo Santos-Sousa, JA Santoshi, Nada Santrac, V Santric, Masamitsu Sanui, Mohammad Sanwar, Sudip Sanyal, C Sanz, Cristina Sanz, Edgar Sanz, Mercedes Sanz, Sandra Sanz, A Sanz Larrainzar, Amaia Sanz Larrainzar, Andrea Sanz Llorente, R Sanz Lopez, G Sanz Ortega, J Sanz Romera, Jorge Sanz Romera, Rosa Sanz-Gonzalez, Ricardo São Pedro, Codin Saon, Rita Sapage, P Sapienza, Paolo Sapienza, Gianmarco Saponaro, D Sapre, Dimple Sapre, Teddy Saputra, Zain Saqfalhait, Madiha Saqib, MW Saqib, Hafiz Saqib Sikandar, F Saraceno, Giorgio Saraceno, A Saracoglu, Ayten Saracoglu, K Saracoglu, Kemal Saracoglu, KT Saracoglu, A Sarafi, P Saraiva, Pedro Saraiva, Wislene Sarajane Moreira Alves, Mumtaz Sarang, I Sarantitis, Ioannis Sarantitis, Sarbpreet Sarao, A Saratziotis, Athanasios Saratziotis, Athanasios Saratzis, R Saravanan, Husam Sarayrah, Imraan Sardiwalla, Kiran Sarfraz, Muhammad Sarfraz Khan, Matthew Sargent, AbdulRahman Sari, D Sari, Djayanti Sari, Can Sarica, Monira Sarih, Ankit Sarin, Divya Sarin, R Sarı, Ramazan Sarı, S Sarıdemir, E Sarjanoja, Elise Sarjanoja, Abhishek Sarkar, Hrishikesh Sarkar, Saurav Sarkar, Riad Sarkis, Mathilde Sarlabous, DR Sarma, Muhammad Sarmad Tamimy, Aisulu Sarmenova, A Sarmiento, Abigail Sarmiento, JA Sarmiento-Bobadilla, S Sarnacki, AL Sarni, V Sarodaya, Varun Sarodaya, G Sarpietro, Giuseppe Sarpietro, Khaled Sarraf, KM Sarraf, Claudia Sarrais Polo, C Sarre, Catherine Sarre, Sera Sarsam, S Sarsik, Sameh Sarsik, L Sartarelli, Enrico Sartori, J Sarveswaran, Janahan Sarveswaran, A Sarwar, MZ Sarwar, Safdar Sarwar, Jahangir Sarwar Khan Khan, Takeshi Sasaki, L Sasatti, Lokesh Sasatti, D Sasia, Diego Sasia, S Saso, Srdjan Saso, Amit Sastry, Alima Satanova, Prassannah Satasivam, V Satchithanantham, Balasupramaniam Sathesan, Niranjan Sathianathen, S Sathyaprasad, Hilda Satie Suto, Kozo Sato, S Satoi, Sohei Satoi, Okazaki Satoshi, S Satoshi, Sato Satoshi, Afroz Satpathy, Bhaskar Satsangi, abida K Sattar, Muhammad Saud Khalid, S Saudi-Moro, Charlie Saunders, Rachel Saunders, L Saura Garcia, M Sauvain, Marc-Olivier Sauvain, F Sauvat, Frederique Sauvat, Farokh Savaddar, Alistair Savage, Stephanie Savage, Vinno Savelli, P Savic, Predrag Savic, Tatjana Savic Jovanovic, Nevena Savković, Ajay Savlania, Vanessa Savopoulos, R Savoy, Rachel Savoy, Atsushi Sawada, Ikumi Sawada, T Sawadi, Taher Sawadi, Mohamed Sawalem, L Sawalha, S Sawalha, Shigehito Sawamura, MN Sawas, D Saxena, Rahul Saxena, S Saxena, Reem Sayad, Shaima Sayad, Aİ Sayar, A Sayasneh, Ahmad Sayasneh, AK Sayed, Esraa Sayed, D Sayed Ahmad, Thabet Sayed Bakir, Bushra Sayeh Mohamed Elhabashi, Anna Sayers, Adnan Sayid Abdo, S Sayır, TO Sayomi, R Sayyed, Raza Sayyed, RH Sayyed, M Sbaih, Mohammed Sbaih, S Scabini, Stefano Scabini, A Scalabre, Aurélien Scalabre, C Scally, G Scambia, Adriana Scamporlino, AM Scanu, Lorenzo Scardina, S Scaringi, Elisa Scarnecchia, O Scatton, Olivier Scatton, Vuk Šćepanović, Miski Scerif, A Schache, Andrew Schache, Tobias Schaetz, B Schäfer, Benedikt Schäfer, K Schaffer, Kathryn Schaffer, Christoph Schäffer, Débora Schalge Campioto, D Schaps, Diego Schaps, HM Schardey, C Schauer, Christian Schauer, JC G Scheijmans, A Scheiwiller, Andreas Scheiwiller, M Schellenberg, Nicholas Schembri, Rebecca Schembri, E Schemitsch, Emil Schemitsch, Peter Schemmer, Pablo Scher, Georg Scheriau, Claudia Scherl, Jordán Scherñuk, R Schiavina, Riccardo Schiavina, M Schiavo, Marcello Schiavo, L Schiffmann, Scott Schimpke, E Schindler, C Schineis, Christian Schineis, E Schipper, PFG Schippers, J Schirnhofer, Jan Schirnhofer, Moritz Schirren, G Schismenou, V Schitcu, D Schizas, Dimitrios Schizas, Daniel Schlager, Nicolas Schlegel, Nis Schlesinger, Tobias Schlesinger, HJ Schlitt, Andrea Schmedding, Benedikt Schmid, S Schmid, Stefan Schmidbauer, Birte Schmidt, Götz Schmidt, J Schmidt, Nele Schmidt, Isabelle Schmit, F Schmitt, Françoise Schmitt, Kamilla Schmitz Nunes, Christoph Schmolmüller, E Schneck, Emmanuel Schneck, MA Schneider, Rolf Schneider, Dorien Schneidmueller, O Schnell, Oliver Schnell, AA Schnitzbauer, Andreas Schnitzbauer, Fabienne Schochter, T Schok, G Schols, Martijn Schoneveld, Daniel Schöni, T Schreckenbach, Philipp Schredl, T Schreiber, Valentin Schreiter, MC Schrempf, L Schröder, Lars Schröder, E Schröder-Langfeld, T Schroeppel, Thomas Schroeppel, TJ Schroeppel, P Schuh, Richard Schulick, Antonia Schulte, Jerette Schultz, Sissy-Amelie Schulz, Patrick Schuss, Karl Schwaiger, Alexandra Schwartz, Gary Schwartz, Patrick Schwartz, Lilian Schwarz, D Schweitzer, Donald Schweitzer, G Scialandrone, Guido Sciaudone, Fabrizio Scognamillo, Federica Scolari, V Scorcia, Vincenzo Scorcia, Benjamin Scott, Christopher Scott, D Scott, Erin Scott, L Scott, Lucy Scott, R Scott, Rupert Scott, G Scotton, Umberto Scovazzi, Lorna Scullion, RJ Scurrah, RR Scurtu, KM Seah, Mark Seamon, Ian Sean Reynolds, Jasem Seba, Samuel Sebastian, Roberto Sebastián Croattini, Juan Sebastian Figueroa, Esteban sebastian Gallino, José Sebastian García, Juan sebastian Guillén, Guillermo Sebastian Martínez Fernández, Juan Sebastian Ramirez, Elisa Sebastiani, E Sebestyen, Tomislav Sečan, Raffaele Sechi, Ismael Sedano-Portillo, G Sedda, Giulia Sedda, SEH Seddik, A Sedighinejad, Ahmed S Sedik, Marián Sedlák, A See, K Seebah, S Seegert, Sara Seegert, B Seeliger, Barbara Seeliger, YLM Seet, Sivendran Seevanayagam, Sheena Seewoonarain, Sena Sefera, Fatma Sefi, Edoardo Segalini, Emma-Tina Segall, Nicholas Segaren, Silvia Segattini, Josefin Segelman, HA Segovia Lohse, HR Segovia Lohse, A Seguin-Givelet, Agathe Seguin-Givelet, Segun Segun-Busari, Josep M Segur, Concepcion Segura, Raquel Segura Roselló, Bárbara Segura-Méndez, JJ Segura-Sampedro, Herman Sehmbi, J Sehouli, R Seiberth, Rose Seiberth, S Seibes, Atsushi Seichi, M Seid, Marta Seid, G Seidel, Adriano Seikiti Stychnicki, Tin Sein, Thomas Seisen, G Seitinger, Gerald Seitinger, I Seiwerth, Ingmar Seiwerth, Arunkumar Sekar, Raghuram Sekhar, Nagaraja Sekhar Ayyalasomayajula, Saumya Sekhar Jenasamant, GK Sekhon, Hiroyuki Seki, M Sekimoto, Yuri Sekiya, A Sekulic, Aleksandar Sekulić, A Seleim, Ahmed Seleim, Salma Selim, Eyyüb Selim Ünlü, Hannah Sellars, Jenardan Sellathurai, Jonida Selmani, Francesco Selvaggi, Lucio Selvaggi, J Selvakumar, TY Selvamani, Ezhir Selvan Chidambarasamy, Daniel Selvaraj, David Selwyn, Miguel Semião, E Semina, Ekaterina Semina, Abigail Semple, Cherith Semple, Nedal Semreen, Qusai Semrin, C Sen, Murat Şen, YK Şen, Benjamin Sena Fenu, Fátima Sena-Ruiz, KJ Senanayake, A Sendad, A Senent-Boza, Ana Senent-Boza, S Seneviratne, Sanjeewa Seneviratne, Christopher Seng Hong Lim, Lip Seng Lee, Shomik Sengupta, Milena Senica Verbic, E Şenödeyici, Gregory Senofsky, Bruno Sensi, R Sepulveda, Rina Sepúlveda, VJ Sepúlveda Zambrano, Liliana Sequeira, P Sequeiros, Renato Seracchioli, Ahmad Seraj Alam, A Serban, Andreea-Madalina Serban, Ashleigh Sercombe, M Serenari, Matteo Serenari, OL Serevina, Sikachov Sergei, Erin Sergey, Dragan Serghei, Fatima Serhan, Deniz Serim Korkmaz, Haseeb Seriwala, Mehmet Serkan Ozkent, A Sermon, Mohamed Serour, Aina Serra, M Serra, Margherita Serra, Maria Serra guivernau, P Serralheiro, Pedro Serralheiro, D Serralta de Colsa, Daniel Serralta de Colsa, Berta Serrano, Dennis Serrano, Oscar Serrano, J Serrano González, Javier Serrano González, P Serrano Méndez, Patricia Serrano Méndez, E Serrano Yébenes, M Serrano-Martin, Monica Serrano-Navidad, İ Sert, Ismail Sert, M Sertkaya, E Seruyange, V Servín, Irena Sesar, Makafui Seth Caleb-Joshua Kwasi Dayie, Russell Seth Martins, Aman Sethi, Neha Sethi, A Sethuraman Venkatesan, Violeta Šetka- Čuljak, Noullet Séverine, Hüsnü Şevi̇k, Fernando Sevilla, Barış Sevinç, J Sewards, K Sexton, S Sexton, Hanife Şeyda Ülgür, P Seyed-Safi, Parisah Seyed-Safi, F Seyedi, N Seyfried, S Seyfried, Steffen Seyfried, J Seyi-olajide, JO Seyi-Olajide, Justina Seyi-Olajide, Z Seymour, Zoe Seymour, Z Seytnebieva, Server Sezgin Uludağ, Joseph Sferra, Dimitrios Sfoungaris, G Sganga, A Sgrò, Abdulkader Shaar, Salem Shaat, Mahmoud Shaban, Nawras Shaban, Walid Shaban, Najat Shaban Ben Hasan, Rabab Shaban Ben Hasan, A Shabana, Ahmed Shabana, M Shackcloth, Rachel Shadbolt, Mohamad Shadi Alkarrash, A Shadrina, M Shadrul Alam, Mohammed Shadrul Alam, Daniel Shaerf, Ali Shafiee, Zahid Shafiq, Aymen Shafqat, Tanveer Shafqat, Sana Shagour, Aaqil Shah, Ami Shah, Amjad Shah, Anneka Shah, D Shah, Fagun Shah, HB Shah, Ipsit Shah, J Shah, Jigar Shah, K Shah, Karishma Shah, Ketan Shah, Mamta Shah, Muhammad Shah, Munjal Shah, P Shah, PA Shah, Raahil Shah, Rakesh Shah, Romil Shah, S Shah, Sajid Shah, SB Shah, Suliman Shah, Ugam Shah, Ushma Shah, V Shah, Vinay Shah, Qutaiba Shah Mardan, Sayed Shah Nur Hussein Shah, N Shahabinejad, Tal Shahar, E Shahbazi, Ahmed Shaheen, Aneela Shaheen, Azka Shaheen, Farah Shaheen, Muhammad Shaheer Akhtar, M Shahi, Madiha Shahid, Shayan Shahidi, M Shahine, Mohammed Shahine, Nur Shahirah Binti Muhammad Shahimi, S Shahrestani, Nazanin Shahrokhi, J Shahu, Aniqa Shahzad, Farakh Shahzad, K Shahzad, I Shaikh, MTA Shaikh, PDA Shaikh, S Shaikh, Shafaque Shaikh, Taariq Shaikh, Sana Shaikh Torab, K Shaikhrezai, Arjun Shajpal, Tony Shaju, Muhammad Shakeel, Malik Shakeel Ahmed, B Shakiba, T Shakir, Z Shakoor, Sudip Shakya, Rasheed Shalabi, Amr Shalaby, Ghada Shalaby, M Shalaby, Mohamed Shalaby, Mostafa Shalaby, Samar Shalaby, J Shalhoub, Joseph Shalhoub, Mishary Shalhoub, Anwar Shamandi, Aliaa Shamardal, Ali Shami, Sarah Shamim, Shahzad Shamim, S Shamoon, Saydash Shamsutdinov, Bee Shan Ong, S Shanbhag, Smruta Shanbhag, Priya Shankar, S Shankar, Shivakumar Shankar, Uma Shanker Pal, K Shanthakunalan, H Shanthanna, Harsha Shanthanna, Henry Shapiro, Manaf Sharabaji, Alaa Sharabi, S Sharabiany, Shashank Sharad Kale, Yassmeen Sharafeldin Mohammed, M Sharara, Premalatha Sharavanan, Said Sharawi, Mohamad Shareeda, Mogahid Sharfeldein, Yaron Shargall, Atif Sharif, Muhammad Sharif, Amal Sharif Eljali, A Sharkey, A Sharma, Abhishek Sharma, Ashish Sharma, DK Sharma, I Sharma, Irvita Sharma, K Sharma, N Sharma, Naveen Sharma, Neil Sharma, P Sharma, S Sharma, Shilpa Sharma, Srujan Sharma, Tanisha Sharma, Tanishq Sharma, V Sharma, Vijay Sharma, Marika Sharmayne Milani, Kaitlyn Sharp, Alexandra Sharpe, C Sharpin, Claire Sharpin, HM Sharples, Mohamed Sharshar, Ayesha Shaukat, Hassan Shaukat, AV Shaw, Catherine Shaw, K Shaw, Kalai Shaw, Katie Shaw, M Shaw, Michael Shaw, Richard Shaw, S Shaw, Balqis Shawer, Momen Shawk, Sherief Shawki, Mohammed Shawqy, E Shaykhinurov, Azriny Shaziela Khalid, Lisa Shea, Lesley Sheach, Harriet Shearman, Wei Shearn Poh, Shahrima Sheefat, S Sheehan, AR G Sheel, Jonathon Sheen, Nikolay Shefer, Reham Shehada, Daleen Shehadeh, Abdelrahman Shehata, S Shehata, Sameh Shehata, Mostafa Shehata Qatora, E Shehi, Ali Raza Shehrazi, A Shehta, Ahmed Shehta, S Sheik, Shahila Sheik, A Sheikh, NA Sheikh, Nomaan Sheikh, Majd Sheikh Alganameh, Duaa Sheikh Kadro, Shashank Shekhar, F Shekleton, Yashwant Shelke, William Shelker, Marie Shella De Robles, Mohamed Shemeis, Maria Shemetova, Richard J Shemin, Amanda Shen, Jeannie Shen, R Shen, Vin Shen Ban, Jian Shen Kiam, Deana Shenaq, A Shenfine, Ziyan Sheng, Kai Sheng Saw, Karim Shenit, Catriona Shenton, J Shepherd, Sally Shepherd, Fateh Sher, Muhammad Sher e Murtaza, Balakh Sher Zaman, K Sheridan, M Sherief, Mohamed Sherief, A Sherif, G Sherif, Hoda Sherif, M Sherif, Mohamed Sherif Morsy, M Sheriff, Mohammed Sheriff, S Sheriff, Maryam Sherwani, S Sherzad, S Shet, Mennatallah Sheta, H Sheth, Hemant Sheth, M Shetiwy, Mohamed Shetiwy, Mosab Shetiwy, Athish Shetty, Prakash Shetty, R Shetty, Rohan Shetty, S Shetty, Vijay Shetty, Shweta Shetye, Bonnie Sheu, M Sheybani-Arani, Margaret Shi, E Shiban, Ehab Shiban, Mosa Shibani, Y Shida, Irena Shiderova, Joud Shiekhoni, H Shields, Sarah Shiels, L Shien Loong, Toshiya Shiga, Kenji Shigemi, Emily Shih, Justina Shikongo, Marwah Shilfeet, Alexey Shilyaev, Dagim Shimelash, SJ Shin, Dimiter Shinkov, M Shinkwin, Michael Shinkwin, Patricia Shinondo, Masaki Shiota, Carlos Shiraishi Zapata, Ahmed Shirazi, Abrham Shitaw, Santhosh Shivashankar Chikkanayakanahalli, Dmitry Shkarupa, Alexandra Shlomina, Darya Shlyk, D Shmatov, Dmitry Shmatov, Muhammad shoaib Nabi, Moses Shodipo, Jessica Shoemaker, Tinashe Shoko, Ahmad Shokry, P Shokuhi, TT Sholadoye, Tunde T Sholadoye, Ashraf Shoma, Susannah Shore, Hany Shoreem, Marwan Shorman, Brooke Short, Matthew Short, Kirill Shostka, Mohamed Shoukry, Fareed Showqi, Ifeoluwa Shoyombo, S Shream, Sarah Shream, AK Shrestha, D Shrestha, K Shrestha, R Shrestha, S Shrestha, Sushruta Shrivastava, Sebastian Shu, Sebastian Shu Yip, SB Shu-Yip, Salonee Shubhen Phanse, A Shugaba, Ahmad Shuib Yahaya, Noura Shujaa, A Shukla, Ayushi Shukla, Anshumala Shukla kulkarni, P Shukla Misra, Amal Shukri, Mohamed shukri Najjar, Andrei Shulgin, Chabwera Shumba, Eric Shumba, K Shumbash, Kibruyisfaw Shumbash, Getachew Shumye, Blagoj Shuntov, Joudy Shurbatji, Mohammed Shwin, Ian Shyaka, Ts Shylasree, Samantha Siahetiong, Maphios Siamuchembu, Filip Sianos, Ioannis Siasios, Babak Siavashi, Kwabena Siaw-Acheampong, M Sibilla, MG Sibilla, Isaie Sibomana, S Siboni, G Sica, Giuseppe Sica, J Siccha, W Siccha Dionicio, N Siddaiah, Manjunath Siddaiah-Subramanya, Amin Siddig, Nidhal Siddig, Haleema Siddique, Hijab Siddique, MH Siddique, Usman Siddique, Ahmed Siddique Ammar, A Siddiqui, Arshad Siddiqui, MS Siddiqui, MT Siddiqui, Nashat Siddiqui, Safia Siddiqui, T Siddiqui, A Sidhu, Astad Sidhwa, T Sidiropoulos, Theodoros Sidiropoulos, L Sidorova, Ludmila Sidorova, Carmen Siebenhofer, Julia Siebert, J Siegrist Ridruejo, R Sieira-Gil, Ramon Sieira-Gil, Leonie Siemen, Nuria Sierra, Anushka Sieunarine, Denise Sievers, Mousa Sifat, Kohila Sigamoney, KV Sigamoney, MA Siguantay, JM L Sijmons, Julie Sijmons, Franck K Sikakulya, K Sikka, Vlad Silaghi, M Silaschi, P Sileri, Pierpaolo Sileri, Abdul Sillah, AH D Silva, ANS Silva, JDS Silva, Jeancarllo Silva, JO Silva, M Silva, Melissa Silva, N Silva, Nelson Silva, Rita Silva, RL Silva, Sílvia Silva, TP Silva, Carlos Silva Faria, B Silva Mendes, Brasil Silva Neto, D Silva Segovia, E Silva-Alvarenga, Liliana Silva-Igua, H Silveira, Helena Silveira, DW A Silvestre, Tedesco Silvia, Isabel Simal Badiola, Dainius Simcikas, Ian Simel, S Simeonidis, Ondrej Simetka, Andrea Simic, K Šimko, Kristián Šimko, Malin Simlund, Mark Simmons, Vicente Simo, A Simoes, J Simões, M Simon, N Simon, Clarisa Simon Perez, Okello Simon Peter, Dima Simona, A Simonato, T Simoncini, G Simone, Giuseppe Simone, Francesca Simonelli, Luigi Simonelli, Marcelo Simonsen, Jessica Simpkins, Cameron Simpson, D Simpson, Renwick Simpson, T Simsek, Doris Sin Wen Ng, LO H Sinan, M Sinclair, Yeong Sing Lee, Umang Singal, Srihari Singaravel, G Singer, Georg Singer, A Singh, AA Singh, Abhijit Singh, Abhinav Singh, Aminder Singh, Anjana Singh, B Singh, Baljit Singh, Harsh Singh, Harvinder Singh, Inderjot Singh, J Singh, Jaswinder Singh, K Singh, Kavindra Singh, Mahendra Singh, Mohit Singh, Nanaki Singh, Narinder Singh, Nirbhaibir Singh, Noel Singh, P Singh, Pooja Singh, Pratibha Singh, R Singh, Rajdeep Singh, Renu Singh, S Singh, Sahiba Singh, Simran Singh, Simrandeep Singh, Sudhir Singh, Sushil Singh, Sweta Singh, Uma Singh, Urmila Singh, V Singh, Vidit Singh, Vivek Singh, Mandeep Singh Bindra, Govind Singh Chauhan, Tapan singh Chauhan, Rajendra Singh Chouhan, Sarvpreet Singh Grewal, Mahaveer Singh Rodha, Moorat Singh Yadav, Damayanti Singha, M Nongalei Singha, S Thoibisana Singha, Camila Singhai, R Singhal, Rishi Singhal, T Singhal, Anusha Singhania, K Singisetti, Kiran Singisetti, W Singleton, D Sinha, Deepti Sinha, Ishani Sinha, S Sinha, Saurabh Sinha, Giovanni Sinibaldi, Jaakko Sinikumpu, Saija Sinimäki, SE Sinisterra Díaz, Robert Sinnerton, Robert Sinyard, M Sion, Melanie Sion, Nicole Siparsky, Zsófia Sipos, Nathalia Siqueira Julio, Vincent Siquian, L Siragusa, Leandro Siragusa, Amjad Siraj Memon, Eyerusalem Siraw, Venesa Siribaddana, Mateja Sirše, Silamlak Sisay, Christian Siso Raber, Daniel Sitaranjan, Magdalena Sitter, Bess Siu Yan Tsui, Soorya Siva, S Sivaganesh, Sivasuriya Sivaganesh, Thanusan Sivapalan, R Sivaprakasam, Rajesh Sivaprakasam, Gausihi Sivarajah, S Sivayoganathan, Armands Sivins, E Sivrikoz, Emre Sivrikoz, Sophon Siwachat, A Skaria, BL Skelly, Christoph Skias, J Skillman, Helen Skinner, Richard JE Skipworth, RJ Skipworth, RJ E Skipworth, Neven Skitarelić, I Skitioui, A Skolarikos, Andreas Skolarikos, Antonia Skotsimara, CE Skoulakis, Charalampos Skoulakis, Dora Škrljak Šoša, M Skrovina, V Skvortsov, George Slade, Isabel Slark, M Slavchev, Mihail Slavchev, Vygintas Šlenfuktas, Algirdas Slepavicius, Z Slevin, Zack Slevin, N Slijepcevic, Nikola Slijepcevic, Karem Slim, Juraj Slipac, Ali Slitin, Vladimir Šljukić, Illya Slobodkin, D Sloothaak, Pablo Slullitel, Fda Sma, Aseel Smadi, Oliver Small, Sarah Small, SR Small, Chloe Smart, Christopher Smart, CJ Smart, Neil Smart, YW Smart, Frank Smedley, Ludi Smeele, R Smeets, J Smelt, Jeremy Smelt, C Smets, Henriette Smid-Nanninga, R Smillie, Anna Smirli, Marieke Smit, A Smith, B Smith, C Smith, Cecilia Smith, Chris Smith, Christopher Smith, DE Smith, H Smith, Henry Smith, J Smith, L Smith, LA Smith, M Smith, Myles Smith, Nicholas Smith, Richard Smith, S Smith, Scott Smith, Thomas Smith, Sebastian Smolarek, D Smolic, Wenko Smolka, Freyja-Maria Smolle-Juettner, Alannah Smrke, F Sneddon, D Snee, Mostafa Snosi, Gordon Snowden, Ana Soares, AP Soares, Tilaê Soares, Agna Soares Da Silva Menezes, Silvana Soares Dos Santos, Carolina Soares-Aquino, Helena Sobrero, A Sobti, D Sochorova, Dana Sochorova, Peter Sodde, Torre Soderlund, V Sodhai, Vivek Sodhai, IM Soeda, Isami Soeda, R Sofat, Asmaa Soffar, S Soffer, Ana Sofia Gonzalez Rubio, O Sogair, F Soggiu, Fiammetta Soggiu, JY Soh, AH Sohail, M Sohail, Mohammad Sohail, Qasim Sohail, Rubina Sohail, Asohanpal Sohanpal, C Sohrabi, Catrin Sohrabi, Saeed Sohrabpour, Selman Sökmen, M Sokolov, Manol Sokolov, Filipp Sokolovski, María Sol Crespi Amor, Maria Sol Fernandez, María Sol Siliato Robles, L Solaini, Leonardo Solaini, Faisal Solanki, Vandana Solanki, Sara Solar, L Solar-Garcia, Lorena Solar-Garcia, Felipe Solares, F Solari, C Soldevila-Verdeguer, Carolina Soledad Romero Garcia, Celeste Soledad Zarratea, Iñigo Soler, Maria Soler Pedrola, Á Soler-Silva, Álvaro Soler-Silva, H Soleymani majd, Hooman Soleymani majd, A Soleymanitabar, A Soliman, Antonios Soliman, Elsayed Soliman, Emad Soliman, F Soliman, Mostafa Soliman, Wael Soliman, F Solimene, Folco Solimene, AP Solis Pazmino, MA Solís-Parra, M Soljic, Martina Soljic, P Solli, Piergiorgio Solli, Giacomo Sollini, Jeremy Solly, Oleksandra Solodarenko, Solofoarimanana Solofoarimanana, Efthymios Solomi, Yoseph Solomon Bezabih, H Soltan, Hatem Soltan, Mina Soltani, Damiano Soma, Vikram Somashekhar Basappanavar, Shanmugam S Somasundaram, Alaa Sommaq, B Sommer, Björn Sommer, F Sommer, Florian Sommer, E Somuncu, Kyongsuk Son, Rajendra Sonawane, N Songthawornpong, Anushri Soni, Gira Soni, Rajesh Soni, J Sonksen, Julian Sonksen, WC Soon, Peng Soon Koh, Charitha Sooriyabandara, F Sorbi, Flavia Sorbi, Gioia Sorbi, Kjetil Soreide, P Sorelli, E Sorial, D Soriero, Domenico Soriero, José Soro-García, L Sorrentino, Luca Sorrentino, Dianne Sosa, MV Sosa, EE Sosa Duran, EE Sosa-Duran, Denorson Sotalbo, MF Sotalin, Lidia Sotillo Valenzuela, M Sotiropoulou, Maria Sotiropoulou, Joel Soto, Carolina Soto Diez, Fidel Soto Hernández, C Soto Montesinos, Cristina Soto Montesinos, Iván Soto-Darias, P Sotonyi, Peter Sotonyi, M Sotudeh, A Sou, A Souadka, Amine Souadka, Zineddine Soualili, Mikael Soucisse, R Soudi, CE Soulé Martínez, Bassem Souleiman, Rachel Soulsby, RE Soulsby, Fouad Souri, AF Sousa, Carla Sousa, Diogo Sousa, Inês Sousa, Liliana B Sousa, Patrícia Sousa, Rita Sousa, S Sousa, Sandra Sousa, M Sousa Fernandes, Mafalda Sousa Fernandes, Donzília Sousa Silva, R Soussan, Andressa Souza, Henri Sova, Savas Soysal, Mustafa Soytas, A Sozzi, Andrea Sozzi, Giulio Sozzi, Tsiona Spaeth, L Spaggiari, Lorenzo Spaggiari, G Spagni, D Spalding, Jessica Spalding, M Spalluto, MG Spampinato, Peter Spangenberg, Martin Spångfors, Andrada Spanu, M Sparavigna, Marco Sparavigna, Ernesto Sparrelid, E Spartalis, M Spartalis, R Spasic, Kolyo Spassov, Kayleigh Spellar, Fritz Spelsberg, Gavin Spence, N Spencer, John Spencer Daniels, B Sperotto, U Speth, J Spicer, C Spiers, Harry Spiers, EJ Spillenaar Bilgen, Alfio Spina, Giacomo Spinato, A Spinelli, Neville Spiteri, D Spoletini, A Spolini, Alessandro Spolini, G Spriano, Giuseppe Spriano, Rebecca Spring, Y Sprunger, Igor Spurnic, E Spurring, I Spyridakis, Ioannis Spyridakis, S Spyridonos, Alexander Spyridoulias, John Squiers, Yara Sras, S Sravanam, Sanskrithi Sravanam, L Srbinovic, B Srbov, Blagoja Srbov, Karuna Sree Pendyala, L Sreedharan, Calum Sreenan, Sachith Sreenivasan, S Sreeram, Shreya Sreeram, P Srekl-Filzmaier, Rachith Sridhar, Pooja Srikanth, Vithranage Srimantha Dewsiri Rodrigo, S Srinathan, Manikandar Srinivas Cheruvu, Rajesha Srinivasaiah, Madhu Srinivasan, S Srishankar, Selvaratnam Srishankar, Nicha Srisuworanan, Aseem Srivastava, Chhitij Srivastava, Suzana Srsen Medancic, Gowtham Srungavarapu, Peter Ssekweyama, A Ssentongo, P Ssentongo, Kagga Ssenyonjo, Peter Ssenyonjo, Henry St Aubyn Bilton, Leslie St Jacques, Pascal St-germain, Raphaël St-germain, G Stables, Fabio Staderini, Josef Stadler, Rupert Stadlhofer, A Stafford, Rose Stahl, E Stamatakis, K Stamatis, Konstantinos Stamatis, SA Stamenkovic, Paraskevas Stamopoulos, Nikolaos Stamos, Konstantinos Stamoulis, Stephen Stanek, M Stangenberg, Martin Stangenberg, S Stanger, Elena-Bianca Stănică, Paul Stanier, Aleksandar Stanimirovic, Oliver Stankov, B Stankovic, Claire Stark, Stefan Stättner, Erica Statuti, S Staubli, M Stavrakas, Sotir Stavridis, Olga Stavrinidou, A Stavroglou, F Stavrou, GA Stavrou, Gregor A Stavrou, D Stavroulias, A Stavrov, Tobias Stedman, B Steel, C Steele, Kathryn Steele, Christopher Steen, Susanne Steer, M Stefan, Mihai Stefan, S Stefan, Samuel Stefan, Tudor Ștefan Dumitrescu, Ioan Stefan Florian, Dragos Stefan Morariu, T Stefaniak, Harald Stefanits, P Stefano, A Stefanovic, T Steffen, C Steidle, Christoph Steidle, John Stein, Dana Steinel, Florian Steiner, Martin Steiner, D Steinhart, Diana Steinhart, Andreas Steinisch, P Steinkamp, Pieter Steinkamp, PJ Steinkamp, J Steinke, O Steinmetz, L Stella, Nimanya Stella Alice, Belise Stella Uwurukundo, John Stengle, J Stephan, Christian Stephan Betz, Ann Stephany Sanchez Marmolejos, Edwin Stephen, A Stephens, Alastair Stephens, Ian Stephens, Matthew Stephens, Samuel Stephens, M Sterrenburg, D Stevanovic, Dejan Stevanovic, P Stevanovic, CT Stevens, Heidi Stevens, K Stevens, Samuel Stevens, Sean Stevens, Andrew Stevenson, James Stevenson, Lianne Stevenson, Dobrica Stevic, Lauren Steward, Camille Stewart, GD Stewart, Grant Stewart, Grant D Stewart, KE Stewart, Peter Stewart, Cornel Steyn, E Steyn, Gert Steyn, Duncan Stickle, M Stiegler, Melissa Stieler, Roxane Stienstra, Jasper Stijns, Z Stillman, Zachery Stillman, T Stockdale, SJ Stoeckli, PT Stogowski, B Stoica, Bogdan Stoica, D Stojakov, Dejan Stojakov, B Stojanovic, Dejan Stojiljkovic, S Stokes, MG Stoleriu, M Stoliarov, Peter Stosberg, Radoslava Stoyanova, J Straehle, Jakob Strähle, Vladislav Straltsov, George Stranjalis, Conrad Stranz, Nadine Straßberger-Nerschbach, T Strate, Tim Strate, E Stratilatovas, T Straube, Dirk Strauss, J Street, John Street, E Streeter, J Strickland, M Strickland, S Strieth, Sebastian Strieth, Anna Strimmer, TD Stringfellow, L Stroman, CU Strømmen, JJ Strotmann, K Stroumpoulis, Alessandro Strumia, K Strupas, Kestutis Strupas, Bynlee Stuart Go, BM Stubbs, W Stupalkowska, Carmelo Sturiale, N Stylianides, G Stylianidis, Malcolm Su, Yu-xiong Su, L Suarez, Raul Suarez, O Suárez Batista, LA Suarez Gonzalez, Paula Suárez Mansilla Suárez-Mansilla, A Suárez Sánchez, Aida Suárez Sánchez, C Suazo, C Suazo Carmelo, Claudia Suazo Carmelo, D Subasinghe, Armande Subayi Nkembi, K Subba, H Subbiah Ponniah, Anbukkani Subbian, V Subbotin, A Subocius, Yamini Subramani, Sentilnathan Subramaniam, Robert Sucher, Cristina Suciu, A Sud, Ajay Sud, Vikas Sud, A Sudarsanam, J Suderman, Sanjibani Sudha, Paul Sudhakar John B, Omar Sudig Abboud, Peter Sudworth, Irina Suero Almanzar, Oscar Suescun, H Sufrin, Hilkiah Suga, Emin Sugaipov, K Sugand, Kapil Sugand, Motohiko Sugi, Adhrie Sugiarto, Teiichi Sugiura, Michael Sugrue, Ramanen Sugunesegran, Amina Suhail, Supisara Suk-Udom, Alaa Sulaiman, Bilal Sulaiman, A Sulaiman Khaled, Rezart Sulce, M Sulciner, Samuel Sule, SO Sule, Ibrahim Suleiman, Salisu Suleiman, N Sulen, M Süleyman, Serkan süleyman Özpak, Taha Suliaman, Ibnouf Sulieman, Mohamed Suliman, Siba Suliman, T Sullivan, Laurent Sulpice, A Sultan, Abdullah Sultan, Dana Sultan, Rizwan Sultan, A Sultana, Asma Sultana, E Sultana, Mahesh Sultania, Adnan Sumadi, Genichiro Sumi, Natnael Sumoro, Alexander Sumpner, Hibba Sumra, Sanela Sumrak, Eiji Sunami, M Sund, Malin Sund, S Sundar, Sudha Sundar, G Sundaram Venkatesan, Gowtham Sundaram Venkatesan, J Sundaresan, M Sundhu, H Sungurtekin, U Sungurtekin, Ugur Sungurtekin, Tanvi Sunil, Melka Supha, S Supparamaniam, Shreyas Supparamaniam, Haya H Suradi, Khushroo Suraliwala, Veena Surendrakumar, A Surendran, Arthika Surendran, Sumi Surendran, Suraj Surendran, Hrishikesh Suresh, Sreelakshmi Suresh, A Suresh kumar, Arjun Suresh kumar, N Sureshkumar Shah, A Suri, Avni Suri, E Surmei, Atul Suroy, IU Surya, Bhaskar Suryanarayanan, Parijat Suryawanshi, Gaby Susana Yamamoto Seto, Ade Susanti, Markus Süss, N Suszták, R Suthakaran, A Sutherland, J Sutton, Jeffrey M Sutton, Robert Sutton, Pavel Suvorin, Aloka Suwanna Danwaththa Liyanage, Naoki Suzuki, Saulius Svagzdys, Clara Svenberg Lind, Nicoletta Sveva Pipitone federico, Sean SW Park, Saleem Swaes, R Swan, Reinier Swart, C Sweeney, Alaa Sweiti, Nepomnyaschaya Swetlana, Hala Swied, Paul Swift, Shreya Syamala, Erwin Syarifuddin, A Syed, Arooj Syed, Danish Syed, Faisal Syed, Bilal Syed muhammad, SAH Syed Nong Chek, Hafiz Syed Zaigham Ali Shah, Rosemary Sykes, A Syllaios, Athanasios Syllaios, D Symeonidis, Vilius Syminas, T Syryło, G Szabo, T Szakmany, Tamas Szakmany, Lilla Szatai, P Szatmary, Peter Szedlak, György Székely, D Szkudlapski, Malgorzata Szpytma, Tamás Sztipits, K Szyluk, Karol Szyluk, Hariharan T D, D T Sathyapalan, Raha Tabasinejad, Meher Tabassum, R Taberham, B Tabeti, S Tabiri, Stephen Tabiri, John Tabiri Abebrese, Ketema Tabore, Sofia Tachella, Keikoku Tachibana, S Tachibana, Shunsuke Tachibana, Enoch Tackie, N Tactuk, Nassin Tactuk, A Taddei, Antonio Taddei, Anteneh Tadesse, B Tadic, Boris Tadic, Naoki Tadokoro, D Tadross, Daniel Tadross, M Taffurelli, Mario Taffurelli, H Taflin, Noha Tageldin, Gashaye Tagele, M Taggarsi, F Taghavi, Mohammad Taghi Imani Khosroshahi, Amir Taghinia, Fulvio Tagliabue, M Tagliabue, Marta Tagliabue, L Taglietti, Lucio Taglietti, Adam Tagmouti, Hassan Taha, Youssef Taha, Areej Taha Al_anaib, mohammad Taha Badawy, Stephanie Taha-Mehlitz, Jamel TaharAissa, Ahmad Tahboub, AS Taher, G Tahhan, Ghis Tahhan, Sheraa Tahhan, I Tahir, Imran Tahir, W Tahir, Warda Tahir, Zaheer Tahir, MA Tahlak, MN Tahmasebi, MI Tahmid, Kay Tai Choy, A Taib, A Taibi, Abdelkader Taibi, A Tailor, Bhavesh Tailor, Rajen Tailor, Muhammad Taimoor Shah, Matthew Taingson, E Taioli, J Tait-Bailey, Ardavan Tajdini, Atsushi Tajima, Bakare Tajudeen Ishola Babatunde, M Takada, Misa Takada, Shintaro Takahashi, Emi Takano, Masashi Takano, Kobayashi Takashi, Saori Takatsuki, Chikashi Takeda, F Takeda, FR Takeda, Misuzu Takeda, Tomohide Takei, Natsuki Takemura, Ario Takeuchi, Mhamdd Talal, I Talal El-Abur, Issa Talal El-Abur, N Talat, Nabila Talat, N Talathoti, T Talbot, Luis-Fernando Talé-Rosales, Abu Talha Siddiqui, Luis Tallon-Aguilar, L Tallón-Aguilar, G Talwar, R Talwar, Rishi Talwar, Irene Tamanini, Madelin Tamar Rosario Villa, Edwin Tamashiro, Sara Tamburello, A Tambyraja, Andrew Tambyraja, Oliver Tame, S Tamer, MS Tamimy, N Tamini, Nicolò Tamini, Hankore Tamirat Derilo, H Tamiru, Hailu Tamiru, P Tammaro, K Tamoos, Khalil Tamoos, Albinas Tamosiunas, EC Tampaki, A Tampakis, Athanasios Tampakis, A Tan, Alex Tan, Alvin Tan, C Tan, Carol Tan, CY Tan, Elsie Tan, EY Tan, Hannah Tan, Jonathan Tan, JR Tan, KL Tan, Lorwai Tan, MNA Tan, MT Tan, Ryan Tan, Silvian Tan, Su-Ming Tan, Teresa Tan, XS Tan, YC Tan, C Tanabalan, Shynar Tanabayeva, Hironori Tanaka, Saori Tanaka, Yukio Tanaka, Kenta Tanakura, M Tanal, Mert Tanal, Yousef Tanas, Andrei Tanase, Alvin Tanaya, EG Tancinco, A Tang, Howard Tang, Lydia Tang, Martin Tangnaa Morna, Tamara Tango, Shinji Tanigaki, James Tankel, R Tanna, Christine Tanos, R Tansey, Gilbert Tanti, Apichat Tantraworasin, AR Tantri, Asher Tanweer Tanveer, J Tanyi, John Tanyi, M Tanzanu, Marta Tanzanu, Jin Tao, Leopoldo Tapia Moral, C Tapking, Christian Tapking, Ritesh Tapkire, Roidah Taqiyya Zahra Wathoni, Kentaroh Tarao, L Tarawneh, Mohammad Abdel Elah A Tarawneh, Mariam Tareen, Ahmed Tarek, Ahmed Tarek Said-elnaby, Ali Tarfiee, J Targarona, EM Targarona Soler, Josefina Tarigo, Ahmet Tarik Harmantepe, Aiman Tariq, Javeria Tariq, M Tariq, Muhammad Tariq, Nida Tariq, S Tariq, Muhammad Tariq Abdullah, Ammar Tariq Alvi, R Tarkowski, M Tarle, X Tarrado, Xavier Tarrado, Bakri Tarras Jarkas, Achille Tarsitano, D Tartaglia, Dario Tartaglia, N Tartaglia, Nicola Tartaglia, C Tartari, I Tasdoven, Marta Tasende, Hanaa Tashkandi, W Tashkandi, N Tasis, Nikolaos Tasis, C Taskiran, Cagatay Taskiran, Androniki Tasouli, Mikko Tastula, Katherine Tataje poma, C Tatar, Cihad Tatar, OC Tatar, Saverio Tateo, Yahaira Tatiana Carpio Colmenares, Karoll Tatiana Meza Garcia, Yeniffer Tatiana Moreno Salazar, D Tatsis, Dimitris Tatsis, K Tausanovic, Rachel Taute, F Tavares, Francisca Tavares, Marlus Tavares Gerber, Noura Tawakl, Alaa Tawalbeh, Voraboot Taweerutchana, Tawfeeq Tawfeeq, ibrahim Tawfiq Daghash, A Tawheed, Ahmed Tawheed, T Tay, Tricia Tay, Seifu Taye, Tewodros Taye, S Tayeh, A Taylor, Amanda Taylor, Danielle Taylor, Elliott H Taylor, Emily Taylor, FG Taylor, Lillian Taylor, OM Taylor, Robert Taylor, Raed Tayyem, L Tchabashvili, Adrian Tchen, S Tchoba, Simplice Tchoba, Rebecca Teague, Warwick Teague, Ella Teasdale, GD Tebala, A Tébar Zamora, Sita Techaboonanake, Vui Teck Lu, Alessandro Tedde, Matteo Tedde, Manfredo Tedesco, Y Tedla, Yonatan Tedla, CM Tedoy, Kiat Tee Benita Tan, PH E Teeuwen, Teshome Tefera, Wajiha Tehniyat, Shahrzad Tehrani, Bernardo Teixeira, J Teixeira, João Teixeira, Maria Teixeira, MF Teixeira, MJ Teixeira, Nuno Teixeira, Pedro Teixeira, PG Teixeira, Rodrigo Teixeira, Jordi Teixidor Serra, Isbaah Tejani, FJ Tejero-Pintor, Francisco J Tejero-Pintor, Guillermo Tejón, Aklilu Teka, K Teker, Oğuzhan Teki̇n, Daniel Teklu, AR Teles, T Teles, Tobias Teles, Rodney Tellez, Manuela Téllez, Clara Téllez Marquès, Isabel Tello Galindo, Cristina Tello-Díaz, Hinerangi Temara, Penias Tembo, S Temkar, Andrew Temperton, Oleg Ten, V Ten, Jacques Tendeng, Cindy Teng, A Tennakoon, Athula Tennakoon, A Tenovici, Nádia Tenreiro, NZ Teo, Leonardo Teodonio, Gonçalo Teodoro Fernandes de Freitas, Anthony Teoh, K Tepetes, FM ter Brugge, Katsuyuki Terajima, J Teran Jurado, David Teren, Maria Teresa, Sintayehu Teresa, Maria Teresa Albiol, Gabriella Teresa Capolupo, Maria Teresa Fernández Martín, M Teresa Gavaldà Pellicé, María Teresa Yepes García, Gilce Teresita Villalba, Salah Termos, RM Terra, Alexis Terras, Kano Teruaki, M Tesei, Marco Tesei, Abdi Tesemma, Getasew Tesfaw, Mequannet Tesfaw, Abraham Teshome, Henok Teshome, A Tessitore, B Tesso, Birhanu Tesso, V Testa, Valentina Testa, S Testelin, Sylvie Testelin, Caterina Testoni, M Teunissen, Sarah Tevis, N Tewari, S Tewari, Shruti Tewari, Vivek Tewarson, S Tezas, Kanat Tezekbaev, Yi Th Ng Seow, MA Thaha, Sumit Thakar, Purvi Thakkar, A Thakker, V Thakker, A Thakrar, Chiraag Thakrar Karia, AW Thakur, B Thakur, Bhaskar Thakur, Dwarakesh Thalamati, Martin Thaler, SPB Thalgaspitiya, Andreas Thalheimer, D Tham, Robin Thambudorai, K Thammasiraphop, A Thanasa, Nova Thani, Apostolos Thanopoulos, Kavya Tharanath, Navamayooran Thavanesan, Meera Thayalan, Rajendram Thayaparren, Mohammad Theab, Dinesh Thekkinkattil, Nikoletta Theochari, Emmanuel Theodorakis, Theodoros Theodoridis, Charalampos Theodoropoulos, Katina Theodoropoulou, Theodosios Theodosopoulos, Barangwa Theophile, E Theophilidou, Elena Theophilidou, D Thereska, Dariel Thereska, MS Thet, Raghavendraswami Thete, S Thiagarajan, Shivakumar Thiagarajan, Sasha Thiel, Clément Thierry Cazemajou, Daniel Thieu, G Thiruchandran, Selven Thirumalai, VG Thiruvasagam, P Thoenissen, S Thole, Panagiotis Thomaidis, N Thomakos, Nikolaos Thomakos, A Thomas, Ashley Thomas, AZ Thomas, E Thomas, Efeson Thomas, Ellen Thomas, H Thomas, Jija Thomas, John K Thomas, Joseph Thomas, Josy Thomas, Kathryn Thomas, M Thomas, N Thomas, Osmond Thomas, Paul Thomas, Rachel Thomas, Varghese Thomas, Vinotha Thomas, Rohan Thomas Mathew, K Thomas-Fernandez, A Thomin, Anne Thomin, A Thompson, Amari Thompson, Carlie Thompson, Daniel Thompson, Gabrielle Thompson, K Thompson, Kyle Thompson, L Thompson, Richard Thompson, Fraser Thomson, Christoph Thomssen, Shu Thong, Nithin Thoppuram, Vaibhav Thorat, Anders Thorell, M Thornhill, S Thornton, Thouement Thouement, Camille Thouny, S Thrumurthy, Srivishnu Thulasiraman, Ruben Thumbadoo, F Thunnissen, Kabilan Thurairajah, Benjamin Thurston, Zoe Thursz, Yuvaraja Thyavihally, Sarah Tian, Kimberly Tiang, A Tibelt, Admasu Tibelt, Silviu Tiberiu Makkai-Popa, N Tibi, A Tidjane, S Tierney, Victor E Tihmanovich, Kehinde Tijani, I Tilaveridis, B Till, Holger Till, EJ Tilling, Areti Tillou, Lola Tillson-Hawke, Thomas Tilston, Michael Tim Yun Ong, S Timbrell, I Timchenko, C Timon, Charlie Timon, Caleb Ting, Hon Ting Lok, Sin Ting Natalie Cheng, May Ting Tan, S Tingle, Samuel Tingle, Jose Tinoco-Gonzalez, PY Tint, Berta Tió, Sebastian Tirapegui, B Tirapelli Gonçalves, Bruna Tirapelli Gonçalves, Leake Tirfe, Dejan Tiric, L Tirloni, Luca Tirloni, F Tirotta, Fabio Tirotta, A Tirsit, Abenezer Tirsit Aklilu, G Tisone, Giuseppe Tisone, Abraham Titigah, Stefan Titu, Christi Titus, A Tiwari, Abhinav Tiwari, Saurabh Tiwari, A Tizmaghz, R Tjipetekera, Mahadewa Tjokorda, Siempui Tling, Christopher To, Kendrick To, Johannes Tobias Thiel, Gabriele Todaro, L Todorovic, Lazar Todorovic, Marko Todorovic, Ali Toffaha, M Tofighi, R Tofighi, SK C Toh, SKC Toh, Calin Tohatan, Teri Toi, F Toia, Francesca Toia, A Tojal, André Tojal, T Tokic, Evripidis Tokidis, M Tokocin, Merve Tokocin, Catherine Toksoy, MA Tolani, A Tolat, Engy Tolba, M Toledano, AI Toledo Castejón, E Toledo Martínez, Enrique Toledo Martínez, T Tollens, Lukas Tolzman, EA Toma, M Tomaiuolo, Hitham Toman, Nefeli Tomara, Jose Tomas Castell Gomez, José Tomás Reyes, Lucy Tomasetti, A Tomazic, Ales Tomazic, Mislav Tomic, Josipa Tomić, Vajdana Tomić, James Tomlinson, Kyoichi Tomoto, V Tondolo, Evgeny Toneev, Alberto Tonelli, Cristiano Tonello, Kaloyan Tonev, A Tong, L Tong, Erik Tongol, V Tonini, G Toogood, Giles Toogood, Yew Toong Liew, A Tooulias, Andreas Tooulias, Thomaz E Topczewski, Jorge Torales, Marco Torchiaro, Francesco Torella, Lucas Torelly Filippi, Gaik Torgomyan, Carlos Toribio Vazquez, C Toribio-Vázquez, J Torkington, Ahmed Torky, S Tormey, M Pilar Tormos Pérez, Ramon Torné, P Torner, Pere Torner, S Tornese, Paul Tornetta, Alejandra Tornini, Fanni Tornyi, A Torquati, Alfonso Torquati, V Torrano, Marta Torrent Lluch, A Torres, David Torres, Janina Torres, M Torres, Patricia Torres, Paula Torres Gomez, L Torres Íñiguez, MT Torres Sánchez, S Torretta, Sara Torretta, G Torzilli, Luigino Tosatto, Euan Toshney, B Toskovic, C Toso, Dezso Toth, JMA Toti, Leandro Totti Cavazzola, J Totty, Joshua Totty, S Toumi, Saam Tourani, P Tourountzi, Paraskevi Tourountzi, M Tousidonis, Bayan Toutounji, Rahaf Toutounji, Tayf Toutounji, S Touzani, S Towell, D Townend, David Townend, P Townend, Philip Townend, Matt Towner, D Townshend, Adam Townson, Satoshi Toyama, S Tozdogan, N Trabulsi, Nora Trabulsi, Lauren Tracy, Luke Traeger, Francesco Traina, Charlotte Trainer, Andrijana Trajkova, V Trajkovic, Velicko Trajkovic, Kelvin Tran, Steven Tran, Ton Tran, S Tranca, F Traunero, Grazia Travaglini, E Travaglio, Elisabetta Travaglio, Carlo Traverso, Rogelio Traverso, D Traxler-Weidenauer, J Trebol, Jacobo Trebol, A Trecci, Kevin Tree, Hendrik Treede, Ravi Trehan, M Trejo-Avila, Mario Trejo-Avila, H Tremblay, V Trenchev, Viktor Trenchev, Tresallet Trésallet, Agnes Treutlein, G Triana, Guillermo Triana, I Triantafyllidis, Alexandra Triantafyllou, Tania Triantafyllou, J Tricard, Jeremy Tricard, Andrei Trif, Aleksandar Trifunovski, Danielle Trigg, Daniel Triguero Cánovas, D Triguero-Cánovas, Vivek Trikha, B Trilling, Bertrand Trilling, Marcial Trillos, Natasha Trilokekar, Santi Trimarchi, M Trindade, Isabela Trindade Martins, M Tripathi, Manjul Tripathi, R Tripathi, Shiva S Tripathi, SS Tripathi, Sujit Tripathy, Jovana Tripkovic, M Tripoli, Massimiliano Tripoli, Anand Trivedi, A Trivic, Aleksandar Trivic, F Trivik-Barrientos, Felipe Trivik-Barrientos, MA Triviño Cortes, A Trofimov, Alexey Trofimow, Rebekka Troller, A Trompeter, P Troncoso Pereira, Paula Troncoso Pereira, Giuseppe Tropeano, Isobel M Trout, S Truant, Stephanie Truant, Wolfram Trudo Knoefel, LM Trujillo, Y Trujillo, J Trujillo Díaz, Jeancarlos Trujillo Díaz, SA Trujillo Ponce, LM Trujillo Sanchez, J Trujillo-Díaz, Ramon Trullenque Juan, Alexander Trulson, S Trungu, Sokol Trungu, Adam Truss, Marc Trust, MD Trust, Ida Tryggedsson, Ioannis Tsagkos, L Tsai, Meng-Ta Tsai, I Tsakiridis, Ioannis Tsakiridis, Peter Tsambarlis, Tsanko Tsankov, N Tsantikos, P Tsarkov, Petr Tsarkov, Edda Tschernko, Sahle Tsegabrhan, Bereket Tsegaye, Milkias Tsehaye, Oltah Tshuma, A Tsiaka, P Tsiantoula, Paraskevi Tsiantoula, A Tsiaousidou, J Tsiaousis, Zac Tsigaras, P Tsinaslanidis, E Tsiridis, Eleftherios Tsiridis, Evangelia Tsironi, Dimitrios Tsironis, Ioannis Tsitiridis, A Tsolakidis, D Tsolakidis, M Tsopozidi, Maria Tsopozidi, Ioannis Tsouknidas, G Tsoulfas, Gerasimos Tsourouflis, Daniela Tsuchiya, Petr Tsugulya, G Tsui, Grace Tsui, Munechika Tsuji, Junji Tsukagoshi, H Tsunobuchi, Fatima Tu zahara, Andrea Tubaro, Ronald Tubasiime, Mayra Tuboi Lamonica, Francesc Tuca, O Tucker, ON Tucker, SC Tucker, Gireesha Tudawe, Kiara Tudela, Maria Tudela, Gabriele Tuderti, Dan Tudor Eniu, AE Tufan, A Tufo, Andrea Tufo, Elif Tugce Kaplan, C Tugmen, Cem Tugmen, M Tulbah, Maha Tulbah, Isha Tuli, G Tulic, I Tulina, Inna Tulina, L Tulinsky, Servio Tulio Torres Rodríguez, Haitham Tumeh, F Tuminello, R Tummon, R Tumolo, K Tuncer, King Tung Cheung, Hei Tung Natalie Chiu, PK Tungala, A Turan, Alparslan Turan, C Turco, Célia Turco, AF Turgeon, EI Turhan, Stefano Turi, S Turial, Salmai Turial, M Turina, Gaston Turinawe, Hazel Turingan, K Turk, Furkan Türkoğlu, A Turna, Akif Turna, A Turnbull, Gareth Turnbull, Paul Turner, R Turner, Scott Turner, V Turrado-Rodriguez, Victor Turrado-Rodriguez, G Turri, Giulia Turri, Laila Turshani, Vaidotas Turskis, Linda Turyabahika, S Tusheva, H Tustin, R Tutino, Roberta Tutino, Gianina Tutoveanu, Zachary Tuttle, M Tuveri, Fatima Tuz Zahra Shakir, A Tvaladze, Josh Twigg, Mayank Tyagi, Volodimir Tyselskiy, Nathan Tyson, Evgenya Tyurina, S Tzamtzidou, S Tzedakis, Stylianos Tzedakis, L Tzelves, Lazaros Tzelves, H Tzerbinis, Angelos Tzouganakis, G Tzovaras, George Tzovaras, M Ubarale, A Uberai, Lisa Überrück, HK Ubhi, Paolo Ubiali, Raul Ubinas, Matteo Uccelli, Kenechukwu Uche-Okonkwo, C Uchendu, Tokujiro Uchida, Z Ud din, Mauin Uddin, Zeeshan Uddin Mughal, Ubong Udoh, Anna Udre, Mahanada Udukala, Tomio Ueno, Katshuhiko Uesaka, Chiharu Ueshima, C Uff, C Uffelmann, Constanza Uffelmann, R Ugarte Oscco, B Ugarte-Sierra, Bakarne Ugarte-Sierra, Henry AA Ugboma, Andrew Ugburo, G Ugolini, MU Ugurlu, Umit Ugurlu, Veronica Ugwi, Ebere Ugwu, Eberhard Uhl, W Uhl, Waldemar Uhl, M Uittenbogaart, M Ujiki, Jörg Ukkat, RD Ukrani, N Ul ain, MA Ul Haq, R Ul HAQ, Noor Ul Huda Maria, AB Ulas, HS Ulgur, A Ulkucu, Tahsin Ullah, Zaker Ullah, Nasr Ullah Kabbani, Sami ullah Niazi, Camila Ulloa, Sarah Ullrich, SJ Ullrich, Hilmican Ulman, Mehmet Uludag, SS Uludag, SS Uludağ, M Umair, Usama Umair, Adam Umair Ashraf Butt, AM Umar, Aminu Umar, M Umar, Muhammad Umar, Muhammad Umar Fawad, Ibrahim Umar Garzali, Lavanya Umashankar, Julian Umlauft, P Umman, Tumay Umuroglu, Ismail Umut Onur, Sandrine Umutesi, N Unal Odabas, Jose Undabeitia, Kirk Underwood, Cristeta Unira, A Unnithan, Richard Unsworth, DR Uppal, SR Uppal, TK Uprak, A Upton, Ishtiaq Ur Rehman, Shams Ur Rehman, S Uranitsch, Stefan Uranitsch, Cihan Uras, S Urban, Shane Urban, A Urbani, Gustavo Urbano, A Urbieta, Aitor Urbieta, K Urbonas, Kestutis Urbonas, Edgar Uribe, Alissa Urich, Porfiria Urquhart, E Urrechaga, Eva Urrechaga, Germaine Ursabia, Ricardo Urzola, Minhaj us siraj Siraj, Muhammad Usaama Bahadoor, Kirill Usachev, A Usai, Sofia Usai, Muhammad Usama, Muhammad Usama Aziz, Usang Usang, G Uslu, Natasha Usman, O Usman, Yasmeen Usman, Muhammad Usman Ali, Muhammad Usman Malik, Idris Usman Takai, Hasan Usmani, Tomas Uson, J Usseglio, Julie Usseglio, Ilham Utama Surya, NZ Utkan, Nasuh Utku Dogan, AF Utria, Alejandra Utrilla Fornals, Marta Utrilla Pérez, Charles Uwagboe, Nicolás Uxmal Soruco Heredia, MS Uyanik, İbrahim Uyar, Ahmad Uzair Qureshi, Kohji Uzawa, M Uzun, Faik G Uzunoglu, FG Uzunoglu, AB Văcărașu, G Vaccarella, Carlos Vaccaro, K Vachtsevanos, Jamshid Vafaeimanesh, Aldo Vagge, Kalpesh Vaghela, Ilias Vagios, Roberto Vagni, Gabriele Vago, SM Vahabi, Mostafa Vahedian, Alexander Vahrmeijer, Narendra Vaidya, M Vailas, Michail Vailas, Dhaivat Vaishnav, Carolynne Vaizey, D Valbuena, S Valderrabano Gonzalez, Santiago Valderrabano Gonzalez, Pamela Valderrama Rocha, Álvaro Valdés de Anca, J Valdes-Hernandez, Javier Valdes-Hernandez, P Valdez, Pedro Valdez, S Valdivieso, L Valeanu, Liana Valeanu, P Valente, Pedro Valente, Vítor Valente, A Valente da Costa, Dominik Valentin Flury, Lara Valentina Comini, Valentino Valentini, F Valenza, JI Valenzuela, Marco Valenzuela, Adriana Valenzuela López, Carlos Valenzuela Salazar, C Valenzuela-Salazar, Irma Valeria Brancaccio Perez, Laine Valerie Kongsun Ching, Razvan Valeriu Iosifescu, R Valero, Ricard Valero, Isabel Valero Lázaro, M Valero Soriano, María Valero Soriano, M Valero-Soriano, Alexandra Valetopoulou, A Valgarðsson, KM Valiouli, I Valioulis, Ioannis Valioulis, Nimisha Vallabh, MI Valldeperas Hernández, Antonio Valle, A Valle Rubio, Ainhoa Valle Rubio, Kyriaki Vallianou, C Vallicelli, M Vallve-Bernal, Marc Vallve-Bernal, Cristina Valor Garcia, L Valsecchi, V Valtzoglou, R Valverde-Vázquez, P Van Amstel, Paul van Amstel, Cornelius J van Beekum, K Van Belle, Koen Van Belle, Mark van Berge Henegouwen, MI van Berge Henegouwen, Elke Van Daele, K Van Dam, Fenna EM van de Leemkolk, D Van de putte, Lucinda Van de Ven, A Van den Berg, Anne van den Berg, WT Van den den Broek, J Van den Eynde, Jef Van den Eynde, R Van den Eynde, Raf Van den Eynde, L Van den Hil, Sjirk van der Burg, SW Van der Burg, L Van der Laan, Jelle van der List, W van der Meij, MJ W van der Oest, Willemijn van der Plas, WY van der Plas, Tim van der Voort, Joost van der Vorst, P Van Duijvendijk, D Van Eck, R Van Eekeren, Ellen Van Eetvelde, Max van Essen, Martijn van Geldorp, AA W Van Geloven, Tessa M van Ginhoven, TM Van Ginhoven, Harry Van Goor, Anne-Sophie Van Haver, M Van Heinsbergen, Maarten van Heinsbergen, C Van Helsdingen, Claire van Helsdingen, Arnaud Van Linden, Justin Y van Oostendorp, AS van Petersen, G Van Ramshorst, Gabrielle van Ramshorst, GH Van Ramshorst, Stephanie van Ruyven, M Van Sambeek, Jan van Schaik, Stephanie Van Straten, Helen van Vliet, Tomas Vanagas, Lucille Vance, Peter-Jan Vancoillie, PJ Vancoillie, Antoine Vancon, Mathieu Vandeputte, Evelyn Vanea, Marco Vanegas, Dayana Vanessa Baron Fuentes, Srikrishna Vangipuram Suresh, Marian Vanhoeij, R Vanker, A Vannelli, G Vanni, H Vanommeslaeghe, T Vanounou, G VanRamshorst, Gabrielle VanRamshorst, C Vantini Capasso Palamim, Camila Vantini Capasso Palamim, A Vaquiro Valencia, A Varazzani, Andrea Varazzani, M Varcada, Massimo Varcada, Raquel Varea Malo, Rodolfo Varela, L Varela Rodríguez, Y Varenne, Yoann Varenne, M Varga, Martin Varga, J Vargas, L Vargas, Lara Vargas, RP Vargas Cordova, C Vargas Reverón, Felipe Vargas-Barato, Abin Varghese, Ashish Varghese, Benjamin Varghese, Paulson Varghese, I Varley, Anil Varma, Kalyan Varma, Madhulika Varma, JG Vartanian, T Vartanoglu, Talar Vartanoglu, Vladimir Varvarin, Manvydas Varzgalis, Aurelia Vas, Edward Vasarhelyi, Vladimir Vasconcelos, E Vásconez, Ravleen Vasdev, Carolyn Vasey, R Vashisht, Bernardas Vasiliauskas, Marcu Vasilica, Anja Vasilj, M Vasiljevic, A Vasireddy, Jovica Vasljević, Rafaela Vasques, X Vasquez, Ximena Vasquez, Victor Vasquez Morales, Jose Vasquez Valverde, XP Vasquez-Ojeda, Panteleimon Vassiliu, N Vassos, Vanitha Vasudevan, D Vatansever, Dogan Vatansever, J Vatish, M Vatish, Hartmut Vatter, P Vaughan, Rachel Vaughan, Peter Vaughan-Shaw, Peter G Vaughan-Shaw, PG Vaughan-Shaw, Sergey Vavilov, Petr Vavra, M Vavro, C Vay, Christian Vay, Dennis Vaysburg, Charlotte Vaysse, Carlos Vaz, J Vaz, James Vaz, OP Vaz, S Vaz, Susan Vaz, João Vaz Grilo, Ricardo Vaz Pereira, Charles Vazeux, S Vazifekhah, Andrea Vazquez Fernandez, A Vazquez Melero, Alba Vazquez Melero, Alejandro Vàzquez Perez, A Vázquez-Fernández, Beatriz Vazquez-Rivero, O Vazquez-Romero, Marília Veccechi Bijos Zaccaro, CM Vega, V Vega, MC Veintimilla Gonzalez, K Vejsbjerg, Karen Vejsbjerg, A Velandia, Felipe Velasco, Eduardo Velasco García, Rosalía Velasco López, Marta Velasco Martinez, Camilo Velasquez, Miguel Velasquez, Susana Velasquez, BG Velasquez Cuasquen, M Velasquez Galvis, Mauricio Velasquez Galvis, V Velchuru, Vamsi Velchuru, Sandra Veleda Belanche, N Velenciuc, Natalia Velenciuc, C Velez, Cristina Velez, JL Velez Bernal, Marcela Velez Botero, E Vélez Segura, Dejan Veličković, M Velidedeoglu, Mehmet Velidedeoglu, G Velmahos, Catherine Velopulos, CG Velopulos, M Velten, Markus Velten, S Vemuru, Linas Venclauskas, Cristiano Vendrame, Vincent Venida, Sudhir Venkataramaiah, M Venn, Mary L Venn, D Venskutonis, Donatas Venskutonis, D Ventura, Sara Venturini, Anuradha Venugopal, Beatriz Vera, C Vera Mansilla, Cristina Vera Mansilla, AF Vera Portilla, GF Veraldi, C Verberne, Arnau Verdaguer Figuerola, Cristina Verdejo Gimeno, O Verdonck, R Vergari, C Vergel Cabrera, M Vergel Gómez, Kélig Vergriete, Romain Verhaeghe, Merel Verhagen, N Verhagen, T Verhagen, K Verhoeff, Gopalkrishna G Verma, R Verma, Rajat Verma, V Verma, Virendra Verma, M Vermaas, S Vermersch, Sophie Vermersch, BA M Vermeulen, Dorien Vermeulen, María Verona Stang, Silvia Verónica Domínguez Ovejas, M Veroux, Massimiliano Veroux, Pierfrancesco Veroux, G Verras, L Verre, Matea Veršić, Laurence Verstraeten, S Vertaldi, D Vervoort, Dominique Vervoort, G Vescio, M Veselinović, B Veys, Antonios Vezakis, J Veziant, Raj Vhatkar, Matteo Vianini, F Vicario, Francisco Vicario, Pedro Vicente Fernández-Fernández, Irune Vicente Rodriguez, E Vico, Vimla Victor, Jose Victor Pérez-Navarro, Jesús Víctor Pérez-Tierra Ruiz, Jose Victoria, Karena Victoria Garcia Tirado, Alexia Victoria Paluso Montero, María Victoria Simón Sanz, María Victoria Taboada, Lucian Vida, Francisco Vidal Candia, Daniel Vidallet, Jerneja Vidmar, R Vidya, Raghavan Vidya, Barbara Viegas Moura, B Vieira, Bruno Vieira, I Vieira, P Vieira, Paula Vieira, A Vieira Barros, Aldo Vieira Barros, AC Vieira Paiva Lopes, I Vieira Toledo, Isabela Vieira Toledo, G Viel, Giovanni Viel, Belén Vielva del Campo, Charlotte Viëtor, CL Viëtor, Stella Vig, A Vignali, Andrea Vignali, Prem Vignesh Mohan, Chiara Vignotto, F Vigouroux, V Vijay, M Vijayakumar Vijayakumar, Paari Vijayaragavan, M Vijayakumar Vijaykumar, Balint Viktor Lovasz, Alexandra Viktoria Behr, R Vilallonga, Ramon Vilallonga, D Vilar-Compte, Diana Vilar-Compte, Elena Vilardell Ortiz, Rodrigo Vilares Morgado, YE Villacorta Acosta, Ainara Villafruela, I Villalabeitia Ateca, Ibabe Villalabeitia Ateca, B Villalonga-Ramirez, I Villamor, Idoia Villamor, I Villamor Garcia, Julio Villanueva, Paulo Villanueva, V Villefranque, Vincent Villefranque, Aaron Villegas, D Villegas Montalvo, Ricardo Villela Prado, B Villota Tamayo, Kelly Viloria Campo, Tim O Vilz, TO Vilz, D Vimalachandran, Dale Vimalachandran, Thanusan Vimalakanthan, Dale Vimalchandran, Koculen Vimaleswaran, Raman Vinayagam, A Vincent, David Vincent Antonio, Prof Vincent Odigie, Cara Vincenti, Matteo Vincenzi, Marco Vinicio Ramírez Sánchez, Acharya Vinoba Bhave, Franziska Vinz, S Viola, A Viola D Souza, KL E Violago, KLE Violago, T Violante, Tommaso Violante, P Violas, Philippe Violas, Mukanyange Violette, P Violo Gonzalez, F Viotti, Fabio Viotti, Amrita Virdi, Edoardo Virgilio, Ashwini Virgincar, Ville P Virta, German Viscido, Jerocin Vishani Loyala, JR Vishnoi, Vartika Vishwani, S Visiedo Sanchez, Ravi Vissapragada, Claire Viste, Y Viswanath, Yks Viswanath, A Vitali, L Vitone, Julius Vitowanu, A Vivas Lopez, Octavio Viveiros, David Viveros-Carreño, I Vives, Inmaculada Vives, Irene Vives Roselló, M Vives-Barquiel, Marian Vives-Barquiel, Josephine Vivian-Taylor, Michel Vix, G Vizzielli, IC Vlad, Schitcu Vlad, Novgorodskii Vladimir, Nils Vleminckx, Jon Vogel, K Vogel, Keianna Vogel, Rachel Vogel, Holger Vogelsang, Thomas Vogler, Kelly Vogt, R Vohra, Ravinder Vohra, KR Voigt, Ismail Vokshi, Olga Volkova, T Vollkommer, A Volpe, A Volpicelli, E Volpin, Enrico Volpin, Francesco Volpin, Jacob Vömel, M von Ahnen, T von Ahnen, Viktor H von Ehrlich-Treuenstätt, M von Fluee, U Von Oppell, Janusz von Renesse, F Von Rundstedt, Friedrich-Carl von Rundstedt, P Von Theobald, K Voon, Sameer Vora, Peter Voros, C Vosinakis, F Vosoughi, Fardis Vosoughi, Kristen Vossler, Manon Vounckx, Srinivas Vourganti, F Vovola, Fernanda Vovola, Marin Vozian, V Vrangalas, Enxhi Vrapi, Marija Vrdoljak, A Vricheva, MR Vriens, PW H E Vriens, Nikola Vucic, Marijana Vučković, I Vukovic, Anuhya Vusirikala, Rini Vyas, Mukta W, Julian W Mall, George W V Cross, D Waast, J Wach, Marta Wachtl, Eilidh Waddell, Oliver Waddell, R Wade, RG Wade, Ryckie Wade, B Wadham, Bianca Wadham, Rekha Wadhwani, Abdullah Wael Mostafa Khalil Bahi, M wafa Hamoud Alhussein, F Wafai, Imad Wafaie, Dalia Wafi, Omar Wafi, A Wagner, Arthur Wagner, D Wagner, Abdelrahman Wahba, Mahmoud Wahbah, S Wahed, Kalsoom Waheed, S Waheed, Fatema Waheed Akbar Mohamad Akbar Nawab Deen, Isaac Wahnon, Artanto Wahyono, Katherine Wai, Ho Wai Ip, Santosh Waigankar, Vaishali Waindeskar, Steven Waisbren, Sofia Waissbluth, Fadi Wakill, Ibrahim Wakim, David Walawah, G Waldolato, Rufus Wale Ojewola, M Waledziak, M Walędziak, Jeremy Wales, A Wali, Sabaeena Wali, A Walid Mohamed, C Walker, Catriona Walker, D Walker, Megan Walker, R Walker, Sophie Walker, T Walker, Thomas Walker, Keisha Walkes, J Wall, Michael Wall, R Wall, Lindsay Wallace, S Wallace-King, Sabina Wallace-King, P Wallaert, E Waller, Christopher Wallis, J Wallqvist, Julia Wallqvist, K Wallwork, Kate Wallwork, Matthew Walmsley, C Walsh, SR Walsh, Stewart Walsh, A Walter, A Walters, C Walters, Camila Walters, Holt Walters, L Walther, Thomas Walther, T Walton, Justine Walusimbi, Mustafa Wameedh Ibrahim, Randa Wanees, Alice Wang, J Wang, Joanna Wang, L Wang, N Wang, Shawn Wang, W Wang, Yi-Chen Wang, IR Wani, Rauf Wani, D Wanigasekera, Samuel Wanjara, SH Waqar, U Waqar, Usama Waqar, H Warburton, S Warburton, A Ward, AE Ward, Alex Ward, Ben Ward, Chloe Ward, Fadi Ward, Salena Ward, Thomas Ward, Y Wardeh, Jonathan Wareing, Sam Wareing, M Waris Farooka, Muhammad Waris Farooka, Michiel Warle, TD Warm, Elizabeth Warnack, M Waroński, Shahrukh Warraich, Leigh Warren, O Warren, J Warusavitarne, Janindra Warusavitarne, S Waseem, Shafqat Wasim, T Wasim, Tayyiba Wasim, Shruti Wasnik, S Wason, Shaun Wason, Amr Wassef, K Wassim, Hidenobu Watanabe, T Watcyn-Jones, J Watfah, Thomas Watkinson, Sven Watmore, Patrick Watmough, Antonia Watson, D Watson, David Watson, Eleanor Watson, Jessica Watson, LJ Watson, Matthew Watson, Nicholas Watson, Sophie Watson, David Watt, Imogen Watt, D Watters, David Watters, Marianne Watters, Leo Watton, Arun Watts, D Watts, Daniel Watts, D Waugh, PA Wawer Matos, Teagan Way, Utkarsha Wayal, H Weaver, Helen Weaver, JI Webb, L Webber, Beatrix Weber, L Weber Graeff, Leonardo Weber Graeff, G Wechselberger, Gottfried Wechselberger, M Węclewicz, Stephanie Weedon, Lotte Weenink, C Weerasinghe, Chamindri Weerasinghe, George Weeratunga, JA Wegdam, Helmut Wegmann, Sheng wei Chiam, Siang Wei Gan, Michael Wei Hii, Tjun Wei Leow, Chi Wei Mok, Frank Weidner, Emily Weisfeld, Helmut Weiss, Gabriella Weisz, J Weitz, Juergen Weitz, M Weitzendorfer, Michael Weitzendorfer, Ken Weixing Ho, C Wells, Cameron Wells, F Wells, T Welsch, S Welter, Stefan Welter, J Wen, Hui Wen Chua, Elizabeth Weng Yan Lun, Samuel Wenham, F Wensley, Rene Wenzl, J Werner, Jens Werner, Joshua Wesley, Wikus Wessel Mulder, Christopher West, Douglas West, R West, Silvia M West, L Westh, E Westwood, Daniel Wettstein, Dániel Wettstein, SD Wexner, Michael Weyant, Felix Wezel, Olivia Wharf, Jennifer Wheatley, S Wheatstone, James Wheeler, Andrew Wheelton, M Whelan, C Whelton, Harriet Whewell, Lisa Whisker, A White, Annabelle White, L White, Louise White, T White, Tim White, E Whitehall, Andrew Whitfield, Robert Whitfield, H Whitmore, Jessica Whitney, Amy Whitworth, A Whyte, Elvina Wiadji, Lucy Wibmer, Matthias Wichmann, L Wickramarachchi, Lilanthi Wickramarachchi, R Wickramarachchi, Dickson Wickramarathna, D Wickramasinghe, Dakshitha Wickramasinghe, D Wickramasooriya, Dj Wickramasooriya, Robert Wickstead, AB H Widatalla, Jeannette Widmer, P Widschwendter, Peter Widschwendter, R Widyaningsih, Rizky Widyaningsih, Yunita Widyastuti, Zuzanna Wieczorek, Dominik Wiedemann, Armin Wiegering, A Wiener, KR Wienholts, CG Wiesinger, Dennis Wigle, Anjelli Wignakumar, W Wijenayake, Manuk Wijeyaratne, Duminda Wijeysundera, Barbara Wildhaber, P Wilhelm, Peter Wilhelm, Karl Wilhelm Henkel, Anna Wilkes, R Wilkin, Richard JW Wilkin, A Wilkins, Alex Wilkins, James Wilkins, Francis William Quayson, A Williams, Christopher YK Williams, Emmanuel Williams, G Williams, Gbolahan Williams, J Williams, K Williams, L Williams, MA Williams, Marli Williams, Matthew Williams, O Williams, Olatoyosi Williams, OM Williams, Omolara Williams, S Williams, Simon Williams, T Williams, Timothy Williams, Vivian Williams, Zoe Williams, Marian Williams-Brown, K Williamson, Megan Williamson, Ellisha Willoughby, K Wilmott, E Wilson, M Wilson, Michael Wilson, MS J Wilson, Pauline Wimberger, Angela Wimmer, M Wimmer, Simon Wimsey, Amadeus Windischbauer, Tony Wing Chung Mak, Yat Wing Smart, P Winnand, Philipp Winnand, D Winter, Des Winter, SC Winter, Stuart C Winter, Thore Winter, B Winter-Roach, Claire Winton, J Winyard, Markus Wirth, Dieter Wirtz, Amy Wise, AX Wiseman, Henry Witcomb Cahill, A Witek, Lukas Wittig, M Wittmann, Maria Wittmann, Tor Wo Chiu, Fredrik Wogensen, Peter Wohlrab, A Wolde, Amanuel Wolde, Dereje Woldemariam, Axel Wolf, Brigid Wolf, Katerina Wolf, S Wolf, Sebastian Wolf, Susanne Wolfer, Kathleen Wolff, Melatework Wolle, A Womersley, S Won, Sae-Yeon Won, Mulualem Wondafrash Mengesha, D Wong, Daniel Wong, EG Wong, Enoch Wong, GKC Wong, J Wong, James Wong, Jane J Wong, JJ Wong, K Wong, Ken Wong, KY Wong, MP Wong, Randolph Wong, S Wong, Valerie Wong, Chang Woo Kim, Daniel Wood, Melissa Wood, ML Wood, Sarah Wood, Esther Woodfield, Thomas Woodhouse, R Woods, Z Woodward, M Workneh, Wubshet Workneh, Bereket Worku, D Worku, Dawit Worku, M Worku, Derje Worku Degefe, Benjamin Wormald, Jamie Wormald, Deborah Wright, FL Wright, Franklin Wright, Gavin Wright, James Wright, Jenny Wright, Matthew Wright, Michelle Wright, Naomi Wright, Estifanos Wubishet, Zinaye Wude, Janet Wui Cheung Kung, Funmilola Wuraola, Obafemi Wuraola, Rosanna Wustrack, Thomas Wyatt, H Wynn Jones, Athanasios Xanthis, Ruben Xavier, S Xenaki, Sofia Xenaki, Andreas Xenakis, Jun Xian Hing, M Xicola, Marina Xicola, M Xicola Martínez, Yi Xin Joanna Fu, Cindy Xin Yi Ou, W Xu, William Xu, S Xyda, E Xynos, Evangelos Xynos, Harish Y S, A Yabas, Kenneth Yabut, Awdhesh Yadav, Mona Yadav, I Yadev, M Yadgarov, S Yadu, Gabriella Yael Hyman, Odai Yaghi, Jamal Yaghmour, Shintaro Yagi, B Yağmur Aras, G Yagoub, Ghozlan Yagoub, Mundashiru Yahaya, Mohammad Yahmad, Reuven Yahud, Lachlan Yaksich, Alfa Yakubu, Musah Yakubu, Husseini Yakubu Mohammed, S Yalamanchili, Ali Yalcinkaya, O Yalkın, Akira Yamada, Takayuki Yamada, Ai Yamaguchi, Michiaki Yamakage, So Yamaki, Maki Yamamoto, Mari Yamamoto, Masae Yamamoto, Naoya Yamamoto, Tomohisa Yamamoto, GS Yamamoto Seto, Esra Yamansavci Şirzai, Ahmad Yamen Arnaout, Evy Yamil Huaman Huallanca, A Yammahi, Qi Yan, Henry Yan Chi Cheung, Jacky Yan Kit Ho, Wayne Yan Lau, Chung Yan Vernon Lee, Hidesuke Yanagida, Hakan Yanar, C Yanez, Carlos Yánez, Rosa-Maria Yañez-Lpez, H Yang, Homer Yang, Kun Yang, W Yang, Wah Yang, A Yanishev, Alexey Yanishev, Özge Yanık, Fady Yanni, Simon Yanni, G Yanowsky Reyes, G Yanowsky-Reyes, Guillermo Yanowsky-Reyes, Lu Yao, M Yao, Mark Yao, E Yaqoob, Eesha Yaqoob, Karla Yareli Hernandez Skewes, JG Yaryura Montero, Levent Yaşar, Mohannad Yaser Alkhazaleh, Fahad Yasin, Tayyub Yasin, Rihab Yasir Abdelmagid, Muhammad Yasir Naseem, Irfana Yasmeen, Farhana yasmin Laskar, Kanastana Yasotharan, Mohammed Yassaad Oudrhiri, Taha Yassein, Ammar Yasser Abdulfattah, Mohd yasser Kayyal, Ahmed Yassien Abd-Elkariem, Abubaker Yassin, M Yassin, N Yassin, Nuha Yassin, C Yates, Jonathan Yates, Tin Yau Ngan, Mesut Yavaş, Aydın Yavuz, D Yavuz, İ Yavuz, Yessica Yax, Guilherme Yazbek, Nasrin Yazdani, Zahra Yazdi, Natalia Ybarra, James Yea, Evie Yeap, A Yebes, Alvaro Yebes, Kwasi Yeboah, Rammohan Yedave, MS Yee, Ka Yee Liew, Mei Yee Ng, M Yeh Ahumada, Nermin Yehia, Shlomo Yellinek, Fasika Yemer, K Yemez, Kürşat Yemez, Jien Yen Soh, A Yener, EMT Yenli, Li Yenn Yong, Sharon Yeo, Clarence Yeoh, T Yeoh, Tien Yeoh, Austin Yeon Suk Lee, Rosie Yeoward, AF Yepes Cano, FE Yepez Yerovi, D Yershov, L Yesantharao, Lekha Yesantharao, P Yesantharao, D Yesilyurt, Gürkan Yetkin, SG Yetkin, Elizabeth Yeung, J Yeung, Justin Yeung, T Yeung, Kar Yeung Kenneth Ko, SN Yew, Anneza Yiallourou, Abiboye Yifieyeh, Makafui Yigah, B Yigit, D Yigit, Merve Yigit, Ulviye Yigit, H Yigitbas, Hakan Yigitbas, Chu Yik Tang, A Yildirim, Asif Yildirim, M Yildirim, A Yildiz, Alp Yildiz, Aybala Yildiz, Burak Yilmaz, Hakan Yilmaz, S Yilmaz, Guang Yim, Beminet Yimenu, Hanlin Yin, Yijie Yin, Y Ying, Kit Ying Au-Yeung, Mei ying Chin, Ho Ying Flora Wong, Zhi Ying Lim, Chung Ying Mok, Li Ying Teoh, V Yip, Vincent Yip, Ampere Yiu, Metin Yığman, İshak Yıldız, Bedrettin Yıldızeli, Tuncay Yılmazlar, Prashanthan Yogarajah, Dagnachew Yohannes, Toshiya Yokota, Hugues Yome, Lydya Yonael, H Yonekura, Hiroshi Yonekura, Kar Yong Wong, Aya Yoshida, Y Yoshikawa, Kazuhiko Yoshimatsu, Yuki Yoshioka, T Yotsov, Tsanko Yotsov, Shahzad Younas, Eman Younes, A Young, Alastair Young, R Young, Thomas Young, Balkees Younis, F Younis, MU Younis, Yasir Younis Abdullahi, Hafsa Younus, Temoore Younus, B Yousaf, Rowaida Yousef, Sally Yousef, Y Yousef, Mohammad Yousef Karasneh, Naser Yousefzadeh Kandevani, Sherine Yousery Askalany, Abdallah Yousif, Omnia MM Yousif, EA Yousof, A Youssef, Ahmed Youssef, Amal Youssef, Daniel Youssef, Fahed Youssef, Hesham Youssef, M Youssef, Mina Youssef, Mohamed Youssef, Omar Youssif Omar Fouad, Ammar Youzkatli Alkhatib, Ihor Yovenko, Dirk Ysebaert, Victor Yu, Yin yu Eva Siu, Tsun Yu Kwan, Ern Yu Tan, Hui Yu Wong, Mustafa Yücel Boz, David Yuen Chung Chan, Kai Yuen Wong, Jeremy Yuen-Chun Teoh, Jane Yuet Ching Hui, Geoffrey Yuet Mun Wong, C Yüksel, Cemil Yüksel, E Yüksel, Akhmad Yun Jufan, Melissa Yun Wee, Asmaa Yunes, Saleha yurf Asghar, Sakoh Yuri, Nazir Yurkuliev, C Yurttas, Can Yurttas, H Yusefi, Hossein Yusefi, Mohd Yusof Sainal, Mohd Yusoff bin Yahaya Yahaya, Safia Yussif, Nuhu Yusuf, Sofiat Yusuf, Mohammed Yusuf Parker, Shamil Yuzbekov, AN Yuzgec, Daniel Zaake, Igor Zabaldin, J Zabaleta, Jon Zabaleta, Ana Zabalza, Carlotta Zaborra, V Zacesta, B Zacharia, Modise Zacharia Koto, D Zacharoulis, Dimitris Zacharoulis, F Zadegan, Frederic Zadegan, Gelareh Zadeh, GM Zafar, M Zafar, Muneeb Zafar, N Zafar, Shahbaz Zafar, SN Zafar, Zain Zafar, Evangelos Zafeiris, Feyme Zafer, Nihat Zafer Utkan, Vladimir Zagainov, Evgeny Zagaynov, Y Zager, Louay Y Zaghlol, Mohamed Zagho, Mauro Zago, Fadzlien Zahari, Mohamed Zahed, Z Zaheen, Fahad Zahid, H Zahid, Z Zahid, M Zahir Aldalati, Farnaz Zahoor, Sanja Zahorjanski, Raihanita Zahra, Mohamed Zahran, Mohammed A Zahran, Hanaa Zahrawi, Abdelrahman Zaid, Robert Zaid Diaz, Ouijdane Zaim, Syed Zain Bukhari, Rizal Zainal, Wan zainira Wan zain, Ahmed Zaitoun, K Żak, Sabahat Zaka, D Zakai, AD Zakaria, Benjamin Zakaria, Hazem Zakaria, Jasiah Zakaria, Mahmoud Zakaria, R Zakaria, Z Zakaria, Zaidi Zakaria, Eman Zakaria Abdelbary, Muhamad Zakaria Brimo Alsaman, Randimbinirina Zakarimanana Lucas, A Zakharenko, Aleksandr Zakharenko, Ahmed Zaki Zoghary, Imad Zakieh, Mustafa Zakkar, GE Zakynthinos, Jihad Zalloum, Anas Zamame, Junaid Zaman, M Zaman, Salma Zaman Zaman, MF Zambelli, M Zambon, Martina Zambon, E Zambrano, B Zamburlini, M Zamfir-Chiru-Anton, Mehroz Zamir, Irving Zamora, Lindsey Zamora, Álvaro Zamora Horcajada, A Zamparas, N Zampitis, B Zampogna, Biagio Zampogna, V Zamvar, ÖP Zanbak Mutlu, Giuseppa Zancana, Aline Zanella, Antonino Zanghì, Alberto Zangrillo, L Zanin, Luca Zanin, N Zanini, Caio Zanon, Gerardo Zanoni, S Zanotti, Simone Zanotti, G Zanus, Giacomo Zanus, I Zapardiel, Ignacio Zapardiel, James Zapata-Copete, Enrique Zapater, Bernhard Zapletal, AG Zarabini, Islam Zarad, A Zarand, SA Zárate León, A Zárate Pinedo, David Zarate Saenz, E-C Zarnescu, NO Zarnescu, Eugenia-Claudia Zarnescu, A Zarour, Ahmad Zarour, P Zarubenko, Victor Zarza Fernandez de Alegria, Alban Zarzavadjian Le Bian, ME Zarzosa Martin, J Žatecký, Jan Žatecký, A Zati Zehni, C Záu Serpa de Araujo, A Zavala Segovia, Mateusz Zawadka, H Zayat, Fatima Zayed Gjam, Sabri Zaza, Aya Zazo, Rama Zazo, I Zbida, Gulmeena Zeb, Laila Zeb, Misbah Zeb, N Zecevic, Henrike Zech, Pradeep Zechariah, Jurica Zedelj, Saqib Zeeshan, BM Zeeshan Hameed, Muhammad Zeeshan Sarwar, Milan Zegarac, H Zegarra, SA Zegarra, Sergio Zegarra, Claus Zehetner, Sadaf Zehra, Shahd Zeid, Doaa Zeidan, Alain G Zeimet, Omar Zein Elabedeen, Zainab Zeino, M Zeiton, Moez Zeiton, Meryem Zekhnini, Toni Zekulić, Ewunetu Zeleke, H Zeleke, Karol Zelenik, N Zeller, Nina Zeller, Simon Zeller, SA Zelmat, Arunas Zelvys, Biniam Zemedu Assefa, S Zenger, AK Zengin, SC Zequi, SDC Zequi, Stenio C Zequi, Alessandro Zerbi, P Zerbib, Philippe Zerbib, Gulilat Zerihun, G Zeringa, R Zerna Encalada, Sebastian Zerwes, O Zerzif El Miliani, LA Zeta, JC Zevallos-Quiroz, BW Zewdie, Betelhem Zewdneh, Natiq Zeynalov, Ümmühan Zeynep Bilgili, Bo Zhang, Charry Zhang, Helen Zhang, Michael Zhang, Tyson Zhang, Y Zhang, Yuexin Zhang, Yuhao Zhang, Bichen Zhao, Chris Zhao, Jie Zhao, F Zheng, Setthasorn Zhi Yang Ooi, A Zhou, Allen Zhou, Jessie Zhou, Xuanyu Zhou, Bonnie Zhu, MZ Zhu, Zhaojun Zhu, Sofiia Zhurba, Victor Zhurkovskiy, Baurzhan Zhussupov, Kashif Zia, H Ziad-Aboharp, William Ziaziaris, Ghina Zidan, Mazen Zidan, Mhd Zied Nasri, P Ziegler, Patrick Ziegler, S Ziemann, Sebastian Ziemann, L Zier, L Zijaj, Lorena Zijaj, Justas Zilinskas, Zoran Zimak, Melissa Zimel, Natalie Zimmelman, Alexander Zimmermann, Benno Zimmermann, M Zimmermann, Matthias Zimmermann, Petra Zimmermann, Daniel Zimpfer, Louise Zinck Mogensen, D Zineddine, Denis Zinkevich, Paola Zinser Peniche, Anna Ziogkou, A Ziółkiewicz, Hubert Zirngibl, Matthias Zitt, V Zivaljevic, Marko Zivanovic, Rastko Živić, D Zivkovic, Dragana Zivkovic, M Zivkovic, Marko Zivkovic, M Zizzo, O Zmora, Oded Zmora, Osnat Zmora, Jacopo Zocchi, C Zografos, Constantinos Zografos, Gavriella Zoi Vrakopoulou, Matteo Zoli, Anna Zolotoukho, Gema Zomeño Bravo, S Zonta, Sandro Zonta, W Zore, D Zouzoulas, A Zowgar, Muhammad Zubair, Albina Zubayraeva, Vanja Žufić, Mohammed Zuhdy, Akmal Zulayla Mohd Zahid, Zulkifli Zulkifli, Alessandra Zullo, T Zulović, Mauricio Zuluaga Zuluaga, Nan Zun Teo, Emma Zuppi, A Zurgan, Amany Zurgan, Robert Zürner, CE Zurstrassen, Matea Zuzul, Y Zwain, Paul Zwittag, C Zymperdikas

**Keywords:** Pandemic preparedness, Health system preparedness, COVID-19, SARS-CoV-2, Surgery, Postoperative mortality

## Abstract

**Background:**

Surgical services were poorly prepared for the COVID-19 pandemic, leading to widescale disruption to elective activity. This study aimed to identify actionable priorities to strengthen pandemic preparedness of surgical and hospital systems.

**Methods:**

This study pooled data from three international, prospective cohort studies including patients who had a positive SARS-CoV-2 test result in the seven days before or within 30 days after surgery. Patients were included across four pandemic time periods: Period 1 (January–May 2020), Period 2 (June–July 2020), Period 3 (October 2020), and Period 4 (December–March 2022). The primary outcome measure was 30-day postoperative mortality. Hierarchical logistic regression models were developed to explore association between pandemic periods (primary analysis) and hospital-level preparedness (secondary analysis) on 30-day postoperative mortality. Hospital preparedness was classified in to poorly-, moderately-, and highly-prepared tertiles based on Surgical Preparedness Index (SPI) score.

**Findings:**

A total of 31,751 patients were included from 1589 hospitals and 102 countries. From Period 1 through to Period 4 there was a decrease in the proportion of patients aged ≥70 years and with ASA grades 3–5.30-day postoperative mortality fell from Period 1 (18.4% [1378/7502]), Period 2 (9.9% [219/2234], adjusted odds ratio (aOR) 0.65, 95% confidence interval (CI) 0.53–0.78), Period 3 (10.5% [246/2427], aOR 0.60, 95% CI 0.50–0.71), through to Period 4 (5.8% [1132/19,588], aOR 0.33, 95% CI 0.30–0.37). During Period 4, SARS-CoV-2 vaccinated patients had lower mortality compared to unvaccinated patients (4.9% [603/12,361] versus 7.4% [529/7178], aOR 0.49, 95% CI 0.42–0.57). Compared to poorly-prepared hospitals (11.2% [1019/9071]), moderately-prepared (9.4% [857/9071], aOR 0.84, 95% CI 0.75–0.94) and highly-prepared hospitals (5.8% [530/9071], aOR 0.70, 95% CI 0.62–0.80) had lower mortality.

**Interpretation:**

Postoperative mortality decreased over the course of the COVID-19 pandemic and was lower in better prepared hospitals. Hospitals are critical national infrastructure and strengthening their preparedness by developing formal pandemic plans, establishing patient and procedure prioritisation protocols, and ring-fencing surgical beds would ensure safer surgical care during future pandemics.

**Funding:**

10.13039/501100000272National Institute for Health and Care Research, United Kingdom.

**Research in context**

**Evidence before this study**

Surgical services were poorly prepared for the COVID-19 pandemic, leading to widescale disruption and cancellation of elective activity. Perioperative SARS-CoV-2 infection during the first pandemic wave was associated with significantly increased mortality, but it is unknown how mortality changed over the course of the pandemic and what factors contributed to this. We performed a structured review on MEDLINE for studies published up to December 1, 2024, using the terms “preparedness” OR “resilience” AND “hospital” OR “country” AND “outcomes”. We found several international studies (CovidSurg-1 and 2) national cohort studies (England, France, USA) describing outcomes in patients undergoing surgery at specific pandemic periods. We found one study assessing the association of country-level Global Health Security and Joint External Evaluation indices and country-level SARS-CoV-2 infection rates. We did not find any studies reporting changes in perioperative outcomes over the course of the pandemic or the impact of hospital-level preparedness on clinical outcomes. We searched for policy documents related pandemic preparedness, including the Lancet Commission on COVID-19, and found that these focussed exclusively on the regional and national planning with minimal attention to hospital-level preparedness.

**Added value of this study**

This study aimed to provide longitudinal evidence to identify actionable targets to strengthen hospital pandemic preparedness. It included prospective data on patients with perioperative SARS-CoV-2 infection from 1598 hospitals in 102 countries. It found that 30-day postoperative mortality reduced over the course of the pandemic. Using the Surgical Preparedness Index to measure hospital-level preparedness, we have demonstrated for the first time that better prepared hospital systems have better patient-level outcomes during a pandemic. Key domains that were strongly associated with lower postoperative mortality were developing formal plans, patient- and procedure prioritisation, and ring-fenced beds.

**Implications of all the available evidence**

During pandemic surges hospitals should implement measures to keep patients safe including patient prioritisation protocols, vaccination, and infection control measures such as transmission-free pathways. When infection rates fall, hospitals should rapidly de-escalate these measures to reopen to minimise disruption to patient pathways and reduce treatment delays. Preparedness is critical to ensuring hospitals are agile in ensuring patient safety during periods of external pressures. The Surgical Preparedness Index should be used to measure hospital-level preparedness and drive continuous improvement and this requires dedicated funding. These findings are generalisable to a broad range of health security risks, including extreme weather events and natural disasters, conflicts, and terrorism.

## Introduction

The 2022 Lancet Commission described how the COVID-19 pandemic led to widescale societal disruption and economic losses, including over 6.9 million deaths worldwide.^1^ The high-risk of a new pandemic,^1^^,^^2^ means that pandemic preparedness is a priority for the World Health Organisation (WHO),^3^ the Coalition for Epidemic Preparedness Innovations (CEPI), the G7 and G20 health initiatives, national pandemic task forces,^4^^,^^5^ and non-governmental organisations, including the Gates Foundation.^6^ The WHO's Health Systems Resilience Framework^7^ emphasises the importance of building adaptive and integrated health systems that can withstand shocks, ensure continuity of essential services, and promote rapid recovery during health emergencies.^8^^,^^9^

Health services were severely impacted by the COVID-19 pandemic, because of both the need to divert hospital beds, staff, and resources to support COVID-19 care, and the need to maintain infection prevention measures. This led to disruption across all care pathways,^10^ but elective surgery was particularly impacted with millions of operations cancelled worldwide,^11^ in part because of the finding that perioperative SARS-CoV-2 infection was associated with significantly increased mortality.^10^^,^^12^ Whilst urgent research led to evidence-based strategies to reopen surgery,^13^, ^14^, ^15^ globally the recovery of surgical systems has been slow, exacerbating waiting lists^16^ and severely setting back progress towards achieving the Lancet Commission on Global Surgery^17^^,^^18^ targets.

The aim of this study was to produce actionable evidence-based recommendations to strengthen preparedness of surgical and hospital systems and inform future pandemic response and recovery. To achieve this, we have pooled data from the two published CovidSurg cohort studies^10^^,^^19^ with fresh data from the unpublished CovidSurg-3 cohort study to explore longitudinal trends in surgical care over the course of the COVID-19 pandemic. In addition, we have used the validated Surgical Preparedness Index^20^ (SPI) to the influence of hospital-level preparedness on patient outcomes.

## Methods

### Objectives and primary outcome

The objectives of this study were firstly, to determine whether mortality changed over the course of the COVID-19 pandemic and secondly, to determine the influence of hospital-level preparedness on patient outcomes. The primary outcome measure was the 30-day postoperative mortality, defined as death within 30-days from surgery, with the day of surgery defined as day zero.

### Study design and setting

This study includes data from the three international, prospective, observational cohort studies delivered through the CovidSurg Collaborative. These studies had harmonised protocols and consistent patient inclusion criteria. TheCovidSurg-1 and CovidSurg-2 (SurgWeek) cohort studies have previously been published,^10^^,^^19^ whereas the CovidSurg-3 data has not previously been published. Patient data from these studies were split in to four distinct pandemic periods: Period 1 (January–May 2020, peak of the first pandemic wave), Period 2 (June–July 2020), Period 3 (October 2020, emergence of the Alpha (B.1.1.7) and Delta (B.1.617.2) variants), and Period 4 (December–February 2022, emergence of the Omicron (B.1.1.529) variant). A detailed description of the different pandemic periods is in the Appendix (p11).

### Inclusion criteria

Participating hospitals collected data on consecutive eligible patients who had a positive SARS-CoV-2 PCR or rapid antigen test result in the seven days before or 30 days after surgery. Patients were identified based on SARS-CoV-2 tests performed as part of routine clinical care as no additional tests were performed as part of this study. Any surgery performed by a surgeon in an operating theatre for any indication was included, with the exception of minor procedures as previously defined.^19^ Patients were only enrolled in any one study once; if they underwent multiple operations within a study window, the procedure closest to the time of their positive SARS-CoV-2 test result was taken as the index procedure.

### Data collection and integrity

Patient, disease, and operation factors were collected. Patient factors were age, sex, American Society of Anaesthesiologists (ASA) physical status classification grade, and Revised Cardiac Risk Index (RCRI) score (categorised as 0, 1, 2, ≥3). Disease factors were indication for surgery (trauma, cancer, benign), urgency of surgery (elective, emergency), and timing of SARS-CoV-2 diagnosis (preoperative versus postoperative based on the timing of the first positive test sample). Elective surgery was defined as surgery on a planned admission to hospital, whereas emergency surgery was defined as surgery on an unplanned admission to hospital. Operative data that were collected were grade of surgery (minor, major), and general anaesthesia use. Grade of surgery was recorded based on the BUPA Schedule of procedures.^19^^,^^21^ In addition, patients were recorded as vaccinated if they had received a first COVID-19 dose, at least two weeks before surgery. Countries were classified as high- (HIC), upper middle (UMIC) or lower middle or low income (LMLIC) based on the 2022 World Bank country income classification.^10^ All data captured were uploaded anonymised.

Data were collected through a secure online Research Electronic Data Capture (REDCap) database.^22^ Integrity of data quality was maintained through previously described strategies (Appendix p12).^10^ Follow-up was based on data captured from written patient notes and computer records relating to inpatient stay and at routine follow-up visits or telephone calls.

### Hospital-level assessment

SPI was developed through an international Delphi consensus process in 2021 to assess hospital readiness to sustain elective surgical services during pandemics.^9^ It is comprised of 23 domains relating to facilities and consumables (n = 11), staffing (n = 2), prioritisation (n = 2), and hospital systems (n = 8). Hospitals are scored from 23 (least prepared) to 115 points (most prepared) (Appendix p13). SPI has previously been demonstrated to be associated with hospital-level performance measured using the surgical volume rate (ratio of the observed surgical volume against expected surgical volume) as a marker of pandemic-related disruption to elective surgical care.^9^ However, it has not previously been validated against surgical outcome measures.

We used SPI to understand the influence of hospital preparedness on patient outcomes during the pandemic, categorising participating hospitals by SPI score as poorly-prepared, moderately-prepared or highly-prepared.^5^ Hospital-level assessments of preparedness were evaluated at two timepoints in participating hospitals. The first assessment made between June 6, 2021 and August 5, 2021 was linked to patient-level data from Pandemic Periods 1–3 and the second assessment made between December 13, 2021 and February 28, 2022 was linked to patient-level data from Pandemic Period 4. SPI measurements were assigned to each patient based on the hospital and pandemic period in which they were operated; all patients within the same hospital within a given pandemic period were assigned the same value. Patient data from hospitals for which a SPI rating was not available were excluded from the secondary analyses requiring SPI data. The data used in this study has not previously been used to validate SPI.

### Statistical analysis

This study was conducted according to the STROBE (Strengthening the Reporting of Observational Studies in Epidemiology)^23^ statement. The χ2 test was used for categorical data. Non-parametric data summarised with medians and interquartile ranges and differences between the groups were tested using the Mann–Whitney U test. Parametric data were summarised with mean and standard deviation. Differences between groups were explored using a two-tailed Student's t-test (two comparator groups) or one-way Analysis of Variance (ANOVA, three or more comparator groups). Further description of the statistical methodology is presented in the Appendix (p14). Statistical significance was defined as a p-value of <0.05. Data analysis was conducted using R (version 3.2.2), with performed using R Foundation Statistical software (R 3.2.2) with TableOne, finalfit, dplyr, ggplot2, Hmisc, and mgcv packages (R Foundation for Statistical Computing, Vienna, Austria).

### Primary analysis

The primary analysis of this study was to explore the association between the four pandemic periods on 30-day postoperative mortality. Multilevel logistic regression models were constructed to account for clinically plausible patient, disease, and operation factors that were selected a priori, informed by the findings of previous analyses of the CovidSurg-1 and CovidSurg-2 datasets (Appendix p14). For all these analyses, both hospital and country were included as random effects. Multicollinearity among covariates in the logistic regression models was assessed by calculating variance inflation factors; all variance inflation factors were below the commonly accepted threshold of 5, indicating that collinearity did not bias the model estimates.

### Secondary analyses

We performed three secondary analyses to understand the association between hospital preparedness, measured using SPI, and 30-day postoperative mortality. Firstly, hospital-level SPI categories were developed as tertiles (−∞, [33] ], (*x* [33], *x* [66] ] and (*x* [66], +∞) where *x* [33], and *x* [66] are, respectively, the 33rd, and 66th percentiles of SPI. To explore the relationship between hospital-level SPI and 30-day mortality, multilevel logistic regression was fitted with country as random effects.

Secondly, to explore association of each SPI domain to adjusted 30-day mortality, we developed an adjusted logistic regression model for 30-day mortality using patient and operative factors, with country as random effects. Predicted probabilities of 30-day mortality for each patient were summed by hospital and observed-to-expected ratios of outcomes derived, which were converted to adjusted hospital 30-day postoperative mortality by multiplication with the population outcome proportion. Quintiles of hospital risk were formed with intervals defined by groups containing close to equal numbers of patients (rather than hospitals) after ranking by hospital-level risk. The relationship of each SPI domain with an aggregated rating of good at hospital level (i.e., rating four or five) was calculated for both the top and bottom mortality patient quintiles. This determines how many well-performing centres were present in top/bottom quintiles for each SPI domain, allowing a measure of discrimination to understand which hospital factors might be responsible for the variation seen. A difference of 10% or more between the best- and worst-performing hospitals was considered clinically meaningful, and SPI domains meeting this threshold were highlighted. This criterion ensured that the domains with the greatest potential for impact were identified, rather than relying solely on statistical significance, since most differences were statistically significant due to a large sample size.

Thirdly, to isolate the role of preparedness, independent of temporal changes such as in SARS-CoV-2 virulence, we conducted multilevel modelling within Pandemic Period 4 alone. In this analysis we explored risk factors for 30-day postoperative mortality, including vaccination, and hospital-level features, stratified by country income group.

### Sensitivity analysis

A restricted cubic spline transformation was used to the continuous representation of SPI to account for potential non-linearity. This was replaced in a final multilevel generalised additive model for Pandemic Periods 1–4.

### Ethics approval

These studies were observational, with no changes made to patient pathways. Only routine, anonymised data were collected. In the UK, the study was registered at each site as either a clinical audit or service evaluation. At the lead UK centre (University Hospital Birmingham) it was approved as clinical audit. In other countries, local principal investigators were responsible for contacting competent research ethics committees to obtain local or national approvals in line with applicable regulations, as well as seeking approvals from data protection officers. Informed patient consent was taken unless the need for this was waived by the local research ethics committee or institutional review body.

### Role of funding source

The funders of the study had no role in study design, data collection, data analysis, interpretation, or writing of the report.

## Results

### Overall study characteristics

**Fig. 1:**
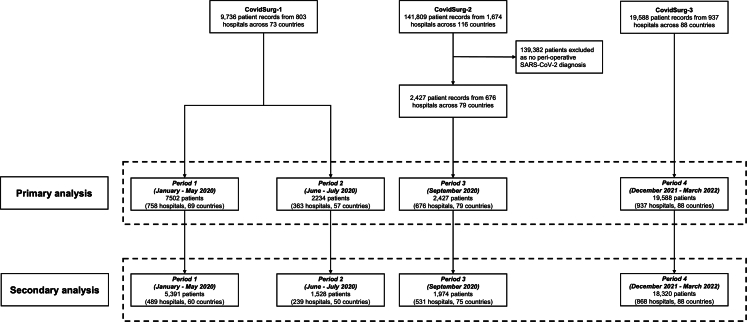
**Patient inclusion flow chart**.

This study included 31,751 patients with a SARS COV-2 diagnosis undergoing surgery across four pandemic periods from 1589 hospitals in 102 countries. A summary of the patient flow included is presented in Fig. 1. Overall, 23,770 (74.9%) patients were from high income countries, 8783 (27.7%) patients were ≥70 years, and 16,490 (51.9%) were female.

### Primary analysis

The overall 30-day mortality was 9.4% (2985/31,751). Mortality was highest in patients aged ≥70 years old (19.1% [1671/8760]), male sex (11.7% [1773/15,201]), ASA grades 3–5 (17.5% [2347/13,401]), and those undergoing emergency surgery (11.7% [2436/20,732]). Baseline patient, disease, and operation characteristics across the pandemic periods are presented in Table 1.

**Table 1:** Patient, disease, and operation characteristics, stratified by pandemic period.

	Pandemic period 1	Pandemic period 2	Pandemic period 3	Pandemic period 4	Total	p-value
Total	7502 (23.6)	2234 (7.0)	2427 (7.6)	19,588 (61.7)	31,751	
Age						
<40 years	1496 (19.9)	1020 (45.7)	792 (32.6)	7437 (38.0)	10,745 (33.8)	<0.001
40–49 years	662 (8.8)	273 (12.2)	286 (11.8)	2318 (11.8)	3539 (11.1)	
50–59 years	942 (12.6)	300 (13.4)	339 (14.0)	2504 (12.8)	4085 (12.9)	
60–69 years	1229 (16.4)	276 (12.4)	357 (14.7)	2730 (13.9)	4592 (14.5)	
≥70 years	3171 (42.3)	361 (16.2)	653 (26.9)	4598 (23.5)	8783 (27.7)	
(Missing)	2 (0.0)	4 (0.2)	0 (0.0)	1 (0.0)	7 (0.0)	
Sex						
Male	3852 (51.3)	1163 (52.1)	1150 (47.4)	9079 (46.3)	15,244 (48.0)	<0.001
Female	3640 (48.5)	1066 (47.7)	1277 (52.6)	10,507 (53.6)	16,490 (51.9)	
(Missing)	10 (0.1)	5 (0.2)	0 (0.0)	2 (0.0)	17 (0.1)	
ASA grades						
1	833 (11.1)	541 (24.2)	539 (22.2)	5048 (25.8)	6961 (21.9)	<0.001
2	2203 (29.4)	810 (36.3)	959 (39.5)	7321 (37.4)	11,293 (35.6)	
3–5	4416 (58.9)	876 (39.2)	929 (38.3)	7208 (36.8)	13,429 (42.3)	
(Missing)	50 (0.7)	7 (0.3)	0 (0.0)	11 (0.1)	68 (0.2)	
Revised cardiac risk index score						
0	2240 (29.9)	803 (35.9)	818 (33.7)	7836 (40.0)	11,697 (36.8)	<0.001
1	3324 (44.3)	1042 (46.6)	1102 (45.4)	7899 (40.3)	13,367 (42.1)	
2	1309 (17.4)	292 (13.1)	354 (14.6)	2553 (13.0)	4508 (14.2)	
≥3	622 (8.3)	93 (4.2)	153 (6.3)	1300 (6.6)	2168 (6.8)	
(Missing)	7 (0.1)	4 (0.2)	0 (0.0)	0 (0.0)	11 (0.0)	
Indication of surgery						
Benign	4189 (55.8)	1528 (68.4)	1548 (63.8)	12,710 (64.9)	19,975 (62.9)	<0.001
Cancer	1241 (16.5)	266 (11.9)	425 (17.5)	2910 (14.9)	4842 (15.2)	
Trauma	2059 (27.4)	429 (19.2)	454 (18.7)	3962 (20.2)	6904 (21.7)	
(Missing)	13 (0.2)	11 (0.5)	0 (0.0)	6 (0.0)	30 (0.1)	
Urgency of surgery						
Elective	1446 (19.3)	409 (18.3)	1009 (41.6)	8077 (41.2)	10,941 (34.5)	<0.001
Emergency	6053 (80.7)	1811 (81.1)	1418 (58.4)	11,506 (58.7)	20,788 (65.5)	
(Missing)	3 (0.0)	14 (0.6)	0 (0.0)	5 (0.0)	22 (0.1)	
Grade of surgery						
Minor	2029 (27.0)	722 (32.3)	769 (31.7)	6702 (34.2)	10,222 (32.2)	<0.001
Major	5408 (72.1)	1456 (65.2)	1658 (68.3)	12,881 (65.8)	21,403 (67.4)	
(Missing)	65 (0.9)	56 (2.5)	0 (0.0)	5 (0.0)	126 (0.4)	
Anaesthesia						
General	5506 (73.4)	1542 (69.0)	1705 (70.3)	13,991 (71.4)	22,744 (71.6)	<0.001
Locoregional	1965 (26.2)	647 (29.0)	721 (29.7)	5568 (28.4)	8901 (28.0)	
(Missing)	31 (0.4)	45 (2.0)	1 (0.0)	29 (0.1)	106 (0.3)	
Timing of diagnosis						
Preoperative	3789 (50.5)	1599 (71.6)	926 (38.2)	10,450 (53.3)	16,764 (52.8)	<0.001
Postoperative	3705 (49.4)	612 (27.4)	1501 (61.8)	9090 (46.4)	14,908 (47.0)	
(Missing)	8 (0.1)	23 (1.0)	0 (0.0)	48 (0.2)	79 (0.2)	
Country income group						
High	6654 (88.7)	1399 (62.6)	1419 (58.5)	14,298 (73.0)	23,770 (74.9)	<0.001
Upper middle	581 (7.7)	567 (25.4)	543 (22.4)	3263 (16.7)	4954 (15.6)	
Lower middle or low	267 (3.6)	268 (12.0)	465 (19.2)	2027 (10.3)	3027 (9.5)	

American Society of Anaesthesiologists (ASA) physical status classification grade.

The proportions of patients aged ≥70 years decreased over the course of the pandemic from 42.3% (3171/7502) in Period 1–23.5% (4598/19,588, Table 1) in Period 4. Over the same time periods the proportions of patients with ASA grades 3-5 decreased from 58.9% (4416/7502) to 36.8% (7208/19,588) and the proportions of patients having emergency surgery decreased from 80.7% (6053/7502) to 58.7% (11,506/19,588). Overall 30-day postoperative mortality decreased from Period 1 (18.4% [1378/7502]), Period 2 (9.9% [219/2234]), Period 3 (10.5% [246/2427]), through to Period 4 (5.8% [1132/19,588]), p < 0.001, Appendix p2, Table S1).

**Fig. 2:**
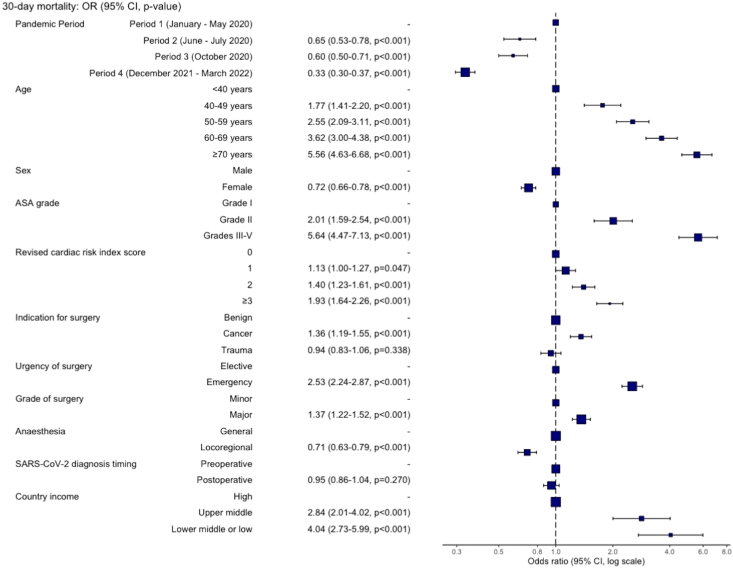
**Primary analysis of postoperative mortality across the four pandemic periods**. American Society of Anaesthesiologists (ASA) physical status classification grade. This multilevel logistic regression model included hospital and country as random effects. The full underlying data are presented in the Appendix (p3, Table S2).

In the multilevel model (Appendix p3, Table S2) accounting for hospital and country as random effects, compared with Pandemic Period 1, mortality was lower in Pandemic Period 2 (adjusted OR (aOR) 0.65, 95% confidence interval (CI) 0.53–0.78, p < 0.001), Pandemic Period 3 (aOR 0.60, 95% CI 0.50–0.71, p < 0.001), and Pandemic Period 4 (aOR 0.33, 95% CI 0.30–0.37, p < 0.001, Fig. 2).

### Secondary analyses

Secondary analyses explored the association between hospital preparedness and 30-day mortality. These analyses were based on data from hospitals for which SPI ratings were available; this included 27,213 patients across 1230 hospitals in 97 countries. The mean SPI rating was 90.9 (95% CI 90.8–91.1; standard deviation: 14.0).

**Fig. 3:**
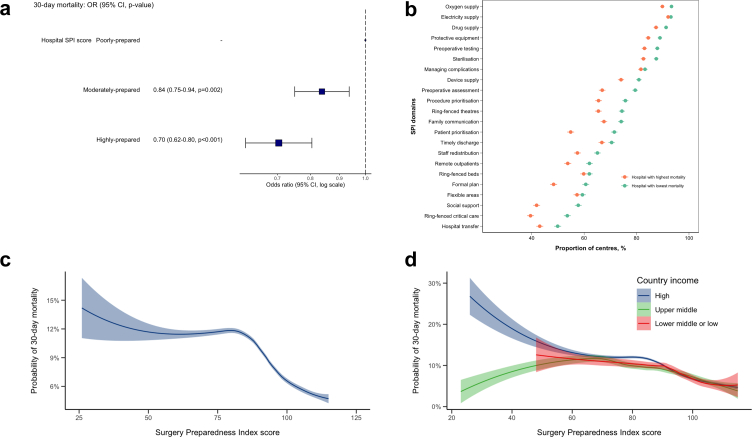
**Secondary analysis of association between Surgical Preparedness Index and 30-day postoperative mortality**. This figure shows the findings of multilevel models exploring association between the Surgical Preparedness Index (SPI) and 30-day postoperative mortality. In all models, country was incorporated as random effects and the following variables as fixed effects: pandemic periods, country income group, age, sex, ASA grade, revised cardiac risk index, indication for surgery, urgency of surgery, grade of surgery, anaesthesia and timing of diagnosis. (a–d) Relationship of SPI in patients across Pandemic Periods 1–4.

Firstly, hospitals were categorised by SPI tertile (cut-offs were ≤86 for poorly-prepared, 87–96 for moderately-prepared, and ≥97 for highly-prepared hospitals). Patients undergoing surgery in highly-prepared hospitals were younger, more likely to undergo elective surgery, and more likely to be in high income countries (Table 2). Across all pandemic periods, postoperative mortality was 5.8% (530/9071) in highly-prepared hospitals, 9.4% (857/9071) in moderately-prepared, and 11.2% (1019/9071) in poorly-prepared hospitals. In the multilevel model, compared to poorly-prepared hospitals, moderately-prepared (aOR 0.84, 95% CI 0.75–0.94, p = 0.002) and highly-prepared (OR 0.70, 95% CI 0.62–0.80, p < 0.001) hospitals had lower 30-day mortality (Fig. 3A, Appendix p4, Table S3).

**Table 2:** Baseline patient, disease, and operation characteristics for Pandemic Periods 1–4, stratified by hospital-level Surgical Preparedness Index.

	Poorly-prepared	Moderately-prepared	Highly-prepared	Total	p-value
Total	9071 (33.3)	9071 (33.3)	9071 (33.3)	27,213	
Pandemic period					
Period 1 (Jan–May 2020)	1981 (21.8)	2210 (24.4)	1200 (13.2)	5391 (19.8)	<0.001
Period 2 (Jun–Jul 2020)	502 (5.5)	431 (4.8)	595 (6.6)	1528 (5.6)	
Period 3 (Oct 2020)	883 (9.7)	738 (8.1)	353 (3.9)	1974 (7.3)	
Period 4 (Dec 2021–Mar 2022)	5705 (62.9)	5692 (62.7)	6923 (76.3)	18,320 (67.3)	
Age					
<40 years	3060 (33.7)	2865 (31.6)	3365 (37.1)	9290 (34.1)	<0.001
40–49 years	977 (10.8)	997 (11.0)	1081 (11.9)	3055 (11.2)	
50–59 years	1128 (12.4)	1106 (12.2)	1225 (13.5)	3459 (12.7)	
60–69 years	1280 (14.1)	1363 (15.0)	1296 (14.3)	3939 (14.5)	
≥70 years	2623 (28.9)	2738 (30.2)	2104 (23.2)	7465 (27.4)	
(Missing)	3 (0.0)	2 (0.0)	0 (0.0)	5 (0.0)	
Sex					
Male	4324 (47.7)	4369 (48.2)	4316 (47.6)	13,009 (47.8)	0.712
Female	4738 (52.2)	4700 (51.8)	4752 (52.4)	14,190 (52.1)	
(Missing)	9 (0.1)	2 (0.0)	3 (0.0)	14 (0.1)	
ASA grades					
1	2220 (24.5)	1844 (20.3)	2127 (23.4)	6191 (22.8)	<0.001
2	3204 (35.3)	3340 (36.8)	3212 (35.4)	9756 (35.9)	
3–5	3615 (39.9)	3877 (42.7)	3717 (41.0)	11,209 (41.2)	
(Missing)	32 (0.4)	10 (0.1)	15 (0.2)	57 (0.2)	
Revised cardiac risk index score					
0	3291 (36.3)	3176 (35.0)	3664 (40.4)	10,131 (37.2)	<0.001
1	3802 (41.9)	3955 (43.6)	3661 (40.4)	11,418 (42.0)	
2	1354 (14.9)	1354 (14.9)	1141 (12.6)	3849 (14.1)	
≥3	618 (6.8)	582 (6.4)	605 (6.7)	1805 (6.6)	
(Missing)	6 (0.1)	4 (0.0)	0 (0.0)	10 (0.0)	
Indication for surgery					
Benign	5683 (62.7)	5444 (60.0)	5870 (64.7)	16,997 (62.5)	<0.001
Cancer	1376 (15.2)	1466 (16.2)	1448 (16.0)	4290 (15.8)	
Trauma	2002 (22.1)	2154 (23.7)	1751 (19.3)	5907 (21.7)	
(Missing)	10 (0.1)	7 (0.1)	2 (0.0)	19 (0.1)	
Urgency of surgery					
Elective	3005 (33.1)	3224 (35.5)	3585 (39.5)	9814 (36.1)	<0.001
Emergency	6057 (66.8)	5843 (64.4)	5484 (60.5)	17,384 (63.9)	
(Missing)	9 (0.1)	4 (0.0)	2 (0.0)	15 (0.1)	
Grade of surgery					
Minor	2925 (32.2)	2817 (31.1)	2977 (32.8)	8719 (32.0)	0.037
Major	6108 (67.3)	6227 (68.6)	6078 (67.0)	18,413 (67.7)	
(Missing)	38 (0.4)	27 (0.3)	16 (0.2)	81 (0.3)	
Anaesthesia					
General	6320 (69.7)	6521 (71.9)	6568 (72.4)	19,409 (71.3)	<0.001
Locoregional	2720 (30.0)	2529 (27.9)	2491 (27.5)	7740 (28.4)	
(Missing)	31 (0.3)	21 (0.2)	12 (0.1)	64 (0.2)	
SARS-CoV-2 diagnosis timing					
Preoperative	4732 (52.2)	4403 (48.5)	5016 (55.3)	14,151 (52.0)	<0.001
Postoperative	4279 (47.2)	4662 (51.4)	4053 (44.7)	12,994 (47.7)	
(Missing)	60 (0.7)	6 (0.1)	2 (0.0)	68 (0.2)	
30-day mortality					
No	8028 (88.5)	8195 (90.3)	8524 (94.0)	24,747 (90.9)	<0.001
Yes	1019 (11.2)	857 (9.4)	530 (5.8)	2406 (8.8)	
(Missing)	24 (0.3)	19 (0.2)	17 (0.2)	60 (0.2)	
Country income group					
High	6030 (66.5)	7067 (77.9)	7297 (80.4)	20,394 (74.9)	<0.001
Upper middle	1726 (19.0)	1238 (13.6)	1078 (11.9)	4042 (14.9)	
Lower middle or low	1315 (14.5)	766 (8.4)	696 (7.7)	2777 (10.2)	

American Society of Anaesthesiologists (ASA) physical status classification grade.

Secondly, the association of each SPI domain with adjusted 30-day mortality was explored. Adjusted 30-day postoperative mortality ranged from 0.1% (8/5473) in quintile 1 (i.e., hospitals with lowest mortality) to 19.7% (1060/5371) in quintile 5 (i.e., hospitals with highest mortality) (Appendix p5, Table S4). Key domains that discriminated between quintile 1 and quintile 5 were patient and procedure prioritisation, ring-fenced theatres, preoperative assessment and formal plans (Fig. 3B).

Thirdly, an adjusted analysis was completed to explore risk factors for 30-day postoperative mortality within Pandemic Period 4 alone. Baseline patient, disease, and operation characteristics for Pandemic Period 4 are presented in the Appendix (p6, Table S5). In the analysis of Pandemic Period 4 data (Appendix p7-8, Table S6, Figure S1), vaccinated patients lower 30-day postoperative mortality than unvaccinated patients (4.9% [603/12,361] versus 7.4% [529/7178], aOR 0.49, CI 0.42–0.57, p < 0.001). Taking in to account SPI scores in the adjusted multilevel model for Period 4 only, compared to poorly-prepared hospitals, moderately-prepared (aOR 0.72, 95% CI 0.60–0.96, p = 0.002) and highly-prepared (aOR 0.66, 95% CI 0.55–0.79, p < 0.001) hospitals had lower 30-day postoperative mortality (Appendix p9-10, Tables S7–S8).

### Sensitivity analysis

An analysis of the relationship of SPI as a continuous variable with mortality found that adjusted 30-day postoperative mortality decreased with increasing hospital SPI ratings (Fig. 3C). When stratified by country income group, there was a consistent trend of lower mortality at SPI ≥60 as SPI rating increased (Fig. 3D); the small number of hospitals rated as having SPI <60 means that country-income sub-group results at that SPI rating should be interpretation with caution.

## Discussion

This study is unique in tracking global trends in surgical practice and outcomes throughout the COVID-19 pandemic. It has demonstrated a global decrease in postoperative mortality in patients with perioperative SARS-CoV-2 infections during the pandemic. From early-to mid-2020, a shift towards lower-risk patient selection was associated with reduced overall mortality. In 2021, the introduction of SARS-CoV-2 vaccination was associated with a further decrease in odds. However, these two factors do not fully explain the overall reduction in mortality across the pandemic, suggesting that the decreased SARS-CoV-2 virulence and increasing immunity from previous infections may have also contributed. These data support previous modelling of the benefits of prioritisation of SARS-CoV-2 vaccination for high-risk patients planned to undergo elective procedures that cannot be safely delayed, for example, cancer surgery.

The key secondary finding from this study was that postoperative mortality was lower in better prepared hospitals. Several previous studies have evaluated pandemic preparedness at country-level^24^^,^^25^ or within individual countries,^26^, ^27^, ^28^, ^29^, ^30^ but this is the first study to comprehensively evaluate the association between hospital-level preparedness and patient-level outcomes.^24^^,^^31^ This study indicates a patient benefit to measuring local preparation and strengthening preparedness at a local hospital-level. These findings are consistent across country-income settings indicating the importance of investment in surgical systems across all settings to strengthen future pandemic preparedness.

This study has identified clear actions for hospital leaders, surgical associations, and national policymakers. National-level pandemic-preparedness plans must integrate strategies that enable the continuation elective surgery, ensuring that all healthcare workers are prepared and resourced to maintain elective care. Regular SPI measurement and reporting should be embedded in these plans to guide efforted aimed at strengthening hospital preparedness. However, the implementation of SPI is not dependent on governments, since it is an open-access tool that frontline surgical teams can take ownership of and champion to guide local quality improvement initiatives. Future studies should track SPI globally and identify successful strategies that facilitate its successful implementation.

In future pandemics, hospitals must be prepared and agile so they can rapidly implement and de-implement pandemic protocols. During pandemic surges hospitals should implement measures to keep patients safe including patient prioritisation protocols, vaccination, and infection control measures such as transmission-free pathways. When infection rates fall, they should rapidly de-escalate these measures to reopen to minimise disruption to patient pathways and reduce treatment delays. Decisions on when to de-escalate infection control measures should be informed by hospital-level preparedness, as well as broader epidemiological considerations such as infection virulence and vaccine availability. These decisions must also be context-specific, taking into account local priorities, healthcare capacity, and resource availability.

Hospital-level preparedness should be regularly assessed using SPI to drive continuous improvement by identifying local context-specific priorities for whole hospital strengthening. It is unlikely that improvement in a single SPI domain is sufficient to substantially alter performance in a pandemic, rather there should be a focus on consistent, incremental improvements across all domains. Moreover, given surgical systems extensively supported the wider pandemic response, through medical gases, supply chains, and critical care facilities, investment in surgery is likely to be effective in both strengthening wider hospital and community resilience across multiple disease pathways, and boosting health system capacity and performance outside of crisis periods, for example by increasing access to medical gasses, diagnostics, and secure energy.

The major strength of this study is the pooled data from 1582 hospitals across three sequential cohort studies and highlighted the importance of rapid, prospective research to inform surgical care during crises such as pandemics. By harnessing data across 102 countries, the study identified many common issues across different hospitals by country income groups, demonstrating the need to invest in hospital preparedness and research infrastructure to support this. However, there are important limitations. Firstly, some patients with perioperative SARS-CoV-2 infection may have been missed if they were not tested as part of their routine clinical care or if their test returned a false negative result. In addition, it was not feasible in this pragmatic global study to collect data the SARS-CoV-2 variant for each patient. Secondly, there may be biases with the self-reported SPI ratings. However, SPI has been validated and compares well with other national-level indicators.^20^ Thirdly, all variables were captured as categorical, including age, limiting the opportunity to explore non-linear relationships of age. Despite this, use of these age categories was well-reported from previous studies, justifying its use in the present study. We used logistic regression to estimate odds ratios, which may overstate relative risks when outcomes are common, so the magnitude of effect should be interpreted with caution; alternative models such as Poisson regression may yield more accurate risk estimates. Finally, there may be residual confounding in the association between SPI and postoperative mortality, for example, as we have not fully accounted for all patients that might effect clinician decision making, nor hospital funding and diagnostic and acute care capacity.

Our findings have significant implications global pandemic preparedness policymaking,^32^^,^^33^ including by governments, the WHO, and major health funders such as the Gates Foundation. Firstly, given its critical role in life-saving care such as cancer treatment, children's health, and women's health, surgical planning must be embedded into broader health system pandemic planning at regional, national, and global levels. A particular focus should be on the interdependency between surgical care and primary health systems. Secondly, to avoid the extensive wider health impacts of lockdowns^11^^,^^20^ in the future, significant investment is needed to increase surgical and hospital system preparedness. It is estimated US$43 billion per year is needed to develop and sustain health emergency preparedness across 139 low- and middle-income countries,^32^ but hospitals are largely neglected in these calculations. It is essential to address the neglected needs of hospital systems in future funding rounds.

Finally, this study evaluated the importance of pandemic preparedness for maintaining safe surgical care, surgical care can be impacted by a broader range of health security risks, including extreme weather events and natural disasters, conflicts, terrorism, and other mass casualty incidents.^9^ Further validation of SPI is required to support its use when preparing for these broader risks. Nonetheless hospitals are critical national infrastructure^34^ with an integral role to crisis response; surgical services underpin this both by providing emergency and trauma care and supporting the wider hospital response. Embedding hospital-level preparedness into national emergency planning would create robust systems capable of responding to diverse health emergencies.

## Contributors

The writing group (DN, SKK, RA, WA, EA, RBC, ME, DG, JCG, AI, KJ, HK, BK, HL, SL, OAO, FP, MP, PP, MDS, JFFS, GT, AB) and the statistical analysis groups (AB, BK, SKK, DN, OAO) all contributed to data curation and interpretation. The writing group contributed to writing and critical revision of the manuscript. The steering group, national leads and dissemination committee contributed to study conception, protocol development, study delivery, and management. The collaborators contributed to data collection and study governance across included sites. The statistical analysis group had full access to the data in this study, accessed and verified the data. The writing committee had final responsibility for the decision to submit for publication. All collaborating authors are shown in the appendix.

## Data sharing statement

Data sharing requests will be considered by the writing group upon written request to the corresponding author. De-identified participant data and/or other pre-specified data will be available, subject to a written proposal and an agreed data sharing agreement.

## Declaration of interests

All authors declare no conflict of interest

## Appendix A Supplementary data

Supplementary Tables

Pubmed authorship list
