# Supplementary material for: 30-day postoperative mortality and the effects of hospital preparedness during the COVID-19 pandemic: a pooled analysis of prospective international cohort studies
Source: Lancet Reg Health Eur. 2026 Jan 29;62:101566. doi: 10.1016/j.lanepe.2025.101566 (PMC12870846; doi:10.1016/j.lanepe.2025.101566)
Supplement: Supplementary Tables [file mmc1.docx]

**30-day postoperative mortality and the effects of hospital preparedness during the COVID-19 pandemic: a pooled analysis of prospective international cohort studies**

*NIHR Global Health Research Unit on Global Surgery, COVIDSurg Collaborative*

**Collaborating authors are listed in Appendix E*

Table of Contents

[Primary analysis 2](#_Toc204210230)

[**Table S1:** Patient, disease, and operation characteristics across Pandemic Periods 1-4, stratified by 30-day postoperative mortality 2](#_Toc204210231)

[**Table S2:** Multilevel model for 30-day postoperative mortality across Pandemic Periods 1-4 3](#_Toc204210232)

[Secondary analyses 4](#_Toc204210233)

[**Table S3:** Multilevel model for 30-day postoperative mortality during Pandemic Periods 1-4, including Surgical Preparedness Index as a factor 4](#_Toc204210234)

[**Table S4:** Patient, disease, and operation characteristics across Pandemic Periods 1-4, stratified by adjusted 30-day postoperative mortality quintiles 5](#_Toc204210235)

[**Table S5:** Baseline patient, disease, and operation characteristics during Pandemic Period 4, stratified by 30-day postoperative mortality 6](#_Toc204210236)

[**Table S6:** Multilevel model for 30-day postoperative mortality during Pandemic Period 4 with binary SARS-CoV-2 vaccination status 7](#_Toc204210237)

[**Figure S1:** Multilevel model for 30-day postoperative mortality during Pandemic Period 4 with binary SARS-CoV-2 vaccination status 8](#_Toc204210238)

[**Table S7:** Baseline patient, disease, and operation characteristics in Pandemic Period 4, stratified by hospital-level Surgery Preparedness Index score 9](#_Toc204210239)

[**Table S8:** Multilevel model for 30-day postoperative mortality in Pandemic Period 4, including hospital-level Surgical Pandemic Preparedness Index score 10](#_Toc204210240)

[Appendix 11](#_Toc204210241)

[**Appendix A:** Description of the pandemic periods 11](#_Toc204210242)

[**Appendix B:** Data integrity processes in the cohort studies 12](#_Toc204210243)

[**Appendix C:** Summary of the Surgical Preparedness Index domains 13](#_Toc204210244)

[**Appendix D:** Statistical methodology 14](#_Toc204210245)

[**Appendix E:** Authorship 17](#_Toc204210246)

# Primary analysis

### Table S1: Patient, disease, and operation characteristics across Pandemic Periods 1-4, stratified by 30-day postoperative mortality

|  |  | **Alive** | **Died** | **p-value** |
| --- | --- | --- | --- | --- |
| Total |  | 28679 (90.6) | 2985 (9.4) |  |
| Pandemic Period | Period 1 | 6101 (81.6) | 1378 (18.4) | <0.001 |
|  | Period 2 | 2000 (90.1) | 219 (9.9) |  |
|  | Period 3 | 2171 (89.5) | 256 (10.5) |  |
|  | Period 4 | 18407 (94.2) | 1132 (5.8) |  |
| Age | <40 years | 10506 (98.0) | 214 (2.0) | <0.001 |
|  | 40-49 years | 3356 (95.2) | 168 (4.8) |  |
|  | 50-59 years | 3727 (91.5) | 348 (8.5) |  |
|  | 60-69 years | 3996 (87.2) | 584 (12.8) |  |
|  | ≥70 years | 7089 (80.9) | 1671 (19.1) |  |
|  | (Missing) | 5 (100.0) | 0 (0.0) |  |
| Sex | Male | 13428 (88.3) | 1773 (11.7) | <0.001 |
|  | Female | 15237 (92.6) | 1209 (7.4) |  |
|  | (Missing) | 14 (82.4) | 3 (17.6) |  |
| ASA grade | Grade 1 | 6831 (98.6) | 99 (1.4) | <0.001 |
|  | Grade 2 | 10741 (95.3) | 529 (4.7) |  |
|  | Grades 3-5 | 11054 (82.5) | 2347 (17.5) |  |
|  | (Missing) | 53 (84.1) | 10 (15.9) |  |
| Revised cardiac risk index | 0 | 11060 (94.9) | 594 (5.1) | <0.001 |
|  | 1 | 12258 (91.9) | 1078 (8.1) |  |
|  | 2 | 3718 (82.7) | 779 (17.3) |  |
|  | ≥3 | 1635 (75.4) | 532 (24.6) |  |
|  | (Missing) | 8 (80.0) | 2 (20.0) |  |
| Indication of surgery | Benign | 18324 (91.9) | 1609 (8.1) | <0.001 |
|  | Cancer | 4255 (88.1) | 575 (11.9) |  |
|  | Trauma | 6087 (88.4) | 798 (11.6) |  |
|  | (Missing) | 13 (81.2) | 3 (18.8) |  |
| Urgency of surgery | Elective | 10369 (95.0) | 548 (5.0) | <0.001 |
|  | Emergency | 18296 (88.3) | 2436 (11.7) |  |
|  | (Missing) | 14 (93.3) | 1 (6.7) |  |
| Grade of surgery | Minor | 9588 (94.0) | 611 (6.0) | <0.001 |
|  | Major | 19001 (89.0) | 2357 (11.0) |  |
|  | (Missing) | 90 (84.1) | 17 (15.9) |  |
| Anaesthesia | General | 20334 (89.6) | 2356 (10.4) | <0.001 |
|  | Locoregional | 8274 (93.1) | 615 (6.9) |  |
|  | (Missing) | 71 (83.5) | 14 (16.5) |  |
| Timing of diagnosis | Preoperative | 15280 (91.4) | 1443 (8.6) | <0.001 |
|  | Postoperative | 13328 (89.6) | 1541 (10.4) |  |
|  | (Missing) | 71 (98.6) | 1 (1.4) |  |
| Country income group | High | 21502 (90.7) | 2209 (9.3) | 0.483 |
|  | Upper middle | 4462 (90.3) | 478 (9.7) |  |
|  | Lower middle or low | 2715 (90.1) | 298 (9.9) |  |

### **Table S2:** Multilevel model for 30-day postoperative mortality across Pandemic Periods 1-4

|  |  | **No** | **Yes** | **OR (univariable)** | **OR (multivariable)** | **OR (multilevel)** |
| --- | --- | --- | --- | --- | --- | --- |
| Pandemic Period | Period 1 | 6101 (21.3) | 1378 (46.2) | - | - | - |
|  | Period 2 | 2000 (7.0) | 219 (7.3) | 0.48 (0.42-0.56, p<0.001) | 0.67 (0.56-0.79, p<0.001) | 0.65 (0.53-0.78, p<0.001) |
|  | Period 3 | 2171 (7.6) | 256 (8.6) | 0.52 (0.45-0.60, p<0.001) | 0.63 (0.53-0.73, p<0.001) | 0.60 (0.50-0.71, p<0.001) |
|  | Period 4 | 18407 (64.2) | 1132 (37.9) | 0.27 (0.25-0.30, p<0.001) | 0.38 (0.35-0.42, p<0.001) | 0.33 (0.30-0.37, p<0.001) |
| Age | <40 years | 10506 (36.6) | 214 (7.2) | - | - | - |
|  | 40-49 years | 3356 (11.7) | 168 (5.6) | 2.46 (2.00-3.02, p<0.001) | 1.84 (1.48-2.28, p<0.001) | 1.77 (1.41-2.20, p<0.001) |
|  | 50-59 years | 3727 (13.0) | 348 (11.7) | 4.58 (3.85-5.46, p<0.001) | 2.61 (2.16-3.17, p<0.001) | 2.55 (2.09-3.11, p<0.001) |
|  | 60-69 years | 3996 (13.9) | 584 (19.6) | 7.17 (6.12-8.44, p<0.001) | 3.64 (3.04-4.38, p<0.001) | 3.62 (3.00-4.38, p<0.001) |
|  | ≥70 years | 7089 (24.7) | 1671 (56.0) | 11.57 (10.03-13.42, p<0.001) | 5.39 (4.53-6.44, p<0.001) | 5.56 (4.63-6.68, p<0.001) |
| Sex | Male | 13428 (46.8) | 1773 (59.5) | - | - | - |
|  | Female | 15237 (53.2) | 1209 (40.5) | 0.60 (0.56-0.65, p<0.001) | 0.72 (0.66-0.78, p<0.001) | 0.72 (0.66-0.78, p<0.001) |
| ASA grade | Grade 1 | 6831 (23.9) | 99 (3.3) | - | - | - |
|  | Grade 2 | 10741 (37.5) | 529 (17.8) | 3.40 (2.75-4.24, p<0.001) | 1.88 (1.50-2.38, p<0.001) | 2.01 (1.59-2.54, p<0.001) |
|  | Grades 3-5 | 11054 (38.6) | 2347 (78.9) | 14.65 (12.02-18.07, p<0.001) | 4.78 (3.83-6.04, p<0.001) | 5.64 (4.47-7.13, p<0.001) |
| Revised cardiac risk index | 0 | 11060 (38.6) | 594 (19.9) | - | - | - |
|  | 1 | 12258 (42.8) | 1078 (36.1) | 1.64 (1.48-1.82, p<0.001) | 1.16 (1.03-1.30, p=0.012) | 1.13 (1.00-1.27, p=0.047) |
|  | 2 | 3718 (13.0) | 779 (26.1) | 3.90 (3.48-4.37, p<0.001) | 1.49 (1.31-1.70, p<0.001) | 1.40 (1.23-1.61, p<0.001) |
|  | ≥3 | 1635 (5.7) | 532 (17.8) | 6.06 (5.33-6.89, p<0.001) | 2.00 (1.72-2.32, p<0.001) | 1.93 (1.64-2.26, p<0.001) |
| Indication of surgery | Benign | 18324 (63.9) | 1609 (54.0) | - | - | - |
|  | Cancer | 4255 (14.8) | 575 (19.3) | 1.54 (1.39-1.70, p<0.001) | 1.31 (1.16-1.48, p<0.001) | 1.36 (1.19-1.55, p<0.001) |
|  | Trauma | 6087 (21.2) | 798 (26.8) | 1.49 (1.36-1.63, p<0.001) | 0.93 (0.83-1.04, p=0.184) | 0.94 (0.83-1.06, p=0.338) |
| Urgency of surgery | Elective | 10369 (36.2) | 548 (18.4) | - | - | - |
|  | Emergency | 18296 (63.8) | 2436 (81.6) | 2.52 (2.29-2.77, p<0.001) | 2.45 (2.18-2.76, p<0.001) | 2.53 (2.24-2.87, p<0.001) |
| Grade of surgery | Minor | 9588 (33.5) | 611 (20.6) | - | - | - |
|  | Major | 19001 (66.5) | 2357 (79.4) | 1.95 (1.78-2.14, p<0.001) | 1.41 (1.27-1.57, p<0.001) | 1.37 (1.22-1.52, p<0.001) |
| Anaesthesia | General | 20334 (71.1) | 2356 (79.3) | - | - | - |
|  | Locoregional | 8274 (28.9) | 615 (20.7) | 0.64 (0.58-0.70, p<0.001) | 0.77 (0.70-0.86, p<0.001) | 0.71 (0.63-0.79, p<0.001) |
| Timing of diagnosis | Preoperative | 15280 (53.4) | 1443 (48.4) | - | - | - |
|  | Postoperative | 13328 (46.6) | 1541 (51.6) | 1.22 (1.14-1.32, p<0.001) | 0.98 (0.90-1.07, p=0.725) | 0.95 (0.86-1.04, p=0.270) |
| Country income group | High | 21502 (75.0) | 2209 (74.0) | - | - | - |
|  | Upper middle | 4462 (15.6) | 478 (16.0) | 1.04 (0.94-1.16, p=0.430) | 2.47 (2.19-2.80, p<0.001) | 2.84 (2.01-4.02, p<0.001) |
|  | Lower middle or low | 2715 (9.5) | 298 (10.0) | 1.07 (0.94-1.21, p=0.309) | 3.89 (3.33-4.53, p<0.001) | 4.04 (2.73-5.99, p<0.001) |

*Multivariable model included patient and operative-level factors such as pandemic period, income group, age, gender, ASA grade, revised cardiac risk index, urgency of surgery, indication of surgery, grade of surgery, type of anaesthesia and timing of COVID-19 diagnosis as fixed effects. Multilevel included hospital and country as random effects.*

# Secondary analyses

### **Table S3:** Multilevel model for 30-day postoperative mortality during Pandemic Periods 1-4, including Surgical Preparedness Index as a factor

|  |  | **Alive** | **Dead** | **OR (univariable)** | **OR (multivariable)** | **OR (multilevel)** |
| --- | --- | --- | --- | --- | --- | --- |
| Hospital SPI rating | Poorly-prepared (≤86) | 8028 (32.4) | 1019 (42.4) | - | - | - |
|  | Moderately-prepared (87 - 96) | 8195 (33.1) | 857 (35.6) | 0.82 (0.75-0.91, p<0.001) | 0.83 (0.74-0.92, p<0.001) | 0.84 (0.75-0.94, p=0.002) |
|  | Highly-prepared (≥97) | 8524 (34.4) | 530 (22.0) | 0.49 (0.44-0.55, p<0.001) | 0.65 (0.58-0.73, p<0.001) | 0.70 (0.62-0.80, p<0.001) |
| Pandemic Period | Period 1 | 6101 (21.3) | 1378 (46.2) | - | - | - |
|  | Period 2 | 2000 (7.0) | 219 (7.3) | 0.48 (0.42-0.56, p<0.001) | 0.72 (0.58-0.88, p=0.001) | 0.64 (0.52-0.80, p<0.001) |
|  | Period 3 | 2171 (7.6) | 256 (8.6) | 0.52 (0.45-0.60, p<0.001) | 0.66 (0.55-0.78, p<0.001) | 0.64 (0.54-0.77, p<0.001) |
|  | Period 4 | 18407 (64.2) | 1132 (37.9) | 0.27 (0.25-0.30, p<0.001) | 0.39 (0.35-0.43, p<0.001) | 0.38 (0.33-0.42, p<0.001) |
| Age | <40 years | 10506 (36.6) | 214 (7.2) | - | - | - |
|  | 40-49 years | 3356 (11.7) | 168 (5.6) | 2.46 (2.00-3.02, p<0.001) | 1.75 (1.36-2.23, p<0.001) | 1.69 (1.32-2.17, p<0.001) |
|  | 50-59 years | 3727 (13.0) | 348 (11.7) | 4.58 (3.85-5.46, p<0.001) | 2.62 (2.11-3.25, p<0.001) | 2.60 (2.09-3.24, p<0.001) |
|  | 60-69 years | 3996 (13.9) | 584 (19.6) | 7.17 (6.12-8.44, p<0.001) | 3.70 (3.02-4.54, p<0.001) | 3.73 (3.03-4.59, p<0.001) |
|  | ≥70 years | 7089 (24.7) | 1671 (56.0) | 11.57 (10.03-13.42, p<0.001) | 5.32 (4.38-6.49, p<0.001) | 5.45 (4.45-6.67, p<0.001) |
| Sex | Male | 13428 (46.8) | 1773 (59.5) | - | - | - |
|  | Female | 15237 (53.2) | 1209 (40.5) | 0.60 (0.56-0.65, p<0.001) | 0.73 (0.66-0.80, p<0.001) | 0.73 (0.67-0.81, p<0.001) |
| ASA grade | Grade 1 | 6831 (23.9) | 99 (3.3) | - | - | - |
|  | Grade 2 | 10741 (37.5) | 529 (17.8) | 3.40 (2.75-4.24, p<0.001) | 1.96 (1.53-2.55, p<0.001) | 2.07 (1.60-2.67, p<0.001) |
|  | Grades 3-5 | 11054 (38.6) | 2347 (78.9) | 14.65 (12.02-18.07, p<0.001) | 4.95 (3.87-6.42, p<0.001) | 5.48 (4.23-7.08, p<0.001) |
| Revised cardiac risk index score | 0 | 11060 (38.6) | 594 (19.9) | - | - | - |
|  | 1 | 12258 (42.8) | 1078 (36.1) | 1.64 (1.48-1.82, p<0.001) | 1.15 (1.01-1.31, p=0.031) | 1.13 (0.99-1.29, p=0.062) |
|  | 2 | 3718 (13.0) | 779 (26.1) | 3.90 (3.48-4.37, p<0.001) | 1.48 (1.28-1.72, p<0.001) | 1.43 (1.23-1.66, p<0.001) |
|  | ≥3 | 1635 (5.7) | 532 (17.8) | 6.06 (5.33-6.89, p<0.001) | 1.96 (1.66-2.32, p<0.001) | 1.89 (1.60-2.25, p<0.001) |
| Indication for surgery | Benign | 18324 (63.9) | 1609 (54.0) | - | - | - |
|  | Cancer | 4255 (14.8) | 575 (19.3) | 1.54 (1.39-1.70, p<0.001) | 1.33 (1.16-1.52, p<0.001) | 1.37 (1.20-1.58, p<0.001) |
|  | Trauma | 6087 (21.2) | 798 (26.8) | 1.49 (1.36-1.63, p<0.001) | 0.96 (0.85-1.09, p=0.548) | 0.99 (0.87-1.13, p=0.875) |
| Urgency of surgery | Elective | 10369 (36.2) | 548 (18.4) | - | - | - |
|  | Emergency | 18296 (63.8) | 2436 (81.6) | 2.52 (2.29-2.77, p<0.001) | 2.46 (2.16-2.80, p<0.001) | 2.58 (2.26-2.94, p<0.001) |
| Grade of surgery | Minor | 9588 (33.5) | 611 (20.6) | - | - | - |
|  | Major | 19001 (66.5) | 2357 (79.4) | 1.95 (1.78-2.14, p<0.001) | 1.39 (1.24-1.57, p<0.001) | 1.36 (1.21-1.53, p<0.001) |
| Anaesthesia | General | 20334 (71.1) | 2356 (79.3) | - | - | - |
|  | Locoregional | 8274 (28.9) | 615 (20.7) | 0.64 (0.58-0.70, p<0.001) | 0.74 (0.66-0.83, p<0.001) | 0.72 (0.64-0.81, p<0.001) |
| SARS-CoV-2 diagnosis timing | Preoperative | 15280 (53.4) | 1443 (48.4) | - | - | - |
|  | Postoperative | 13328 (46.6) | 1541 (51.6) | 1.22 (1.14-1.32, p<0.001) | 0.96 (0.87-1.05, p=0.352) | 0.95 (0.86-1.05, p=0.294) |
| Country income group | High | 21502 (75.0) | 2209 (74.0) | - | - | - |
|  | Upper middle | 4462 (15.6) | 478 (16.0) | 1.04 (0.94-1.16, p=0.430) | 2.28 (1.98-2.61, p<0.001) | 2.62 (1.87-3.66, p<0.001) |
|  | Lower middle or low | 2715 (9.5) | 298 (10.0) | 1.07 (0.94-1.21, p=0.309) | 3.71 (3.14-4.38, p<0.001) | 3.77 (2.58-5.49, p<0.001) |

**The multivariable analysis included as fixed effects: pandemic periods, country income group, age, sex, ASA grade, revised cardiac risk index, urgency of surgery, indication of surgery, grade of surgery, anaesthesia and timing of SARS-CoV-2 diagnosis. For the multilevel analysis, hospital and country were random effects.*

### **Table S4:** Patient, disease, and operation characteristics across Pandemic Periods 1-4, stratified by adjusted 30-day postoperative mortality quintiles

|  |  | **Quintile 1** | **Quintile 2** | **Quintile 3** | **Quintile 4** | **Quintile 5** | **Total** | **p-value** |
| --- | --- | --- | --- | --- | --- | --- | --- | --- |
| Total |  | 5473 (20.1) | 5421 (19.9) | 5571 (20.5) | 5366 (19.7) | 5371 (19.7) | 27202 |  |
|  |  | 8 (0.1) | 234 (4.3) | 411 (7.4) | 692 (12.9) | 1060 (19.7) | 2405 (8.8) | <0.001 |
| Pandemic Period | Period 1 | 544 (9.9) | 787 (14.5) | 843 (15.1) | 1627 (30.3) | 1590 (29.6) | 5391 (19.8) | <0.001 |
|  | Period 2 | 298 (5.4) | 206 (3.8) | 230 (4.1) | 459 (8.6) | 333 (6.2) | 1526 (5.6) |  |
|  | Period 3 | 539 (9.8) | 244 (4.5) | 328 (5.9) | 368 (6.9) | 495 (9.2) | 1974 (7.3) |  |
|  | Period 4 | 4092 (74.8) | 4184 (77.2) | 4170 (74.9) | 2912 (54.3) | 2953 (55.0) | 18311 (67.3) |  |
| Age | <40 years | 2585 (47.2) | 1773 (32.7) | 1543 (27.7) | 1698 (31.6) | 1690 (31.5) | 9289 (34.1) | <0.001 |
|  | 40-49 years | 682 (12.5) | 550 (10.1) | 671 (12.0) | 560 (10.4) | 588 (10.9) | 3051 (11.2) |  |
|  | 50-59 years | 639 (11.7) | 695 (12.8) | 739 (13.3) | 712 (13.3) | 671 (12.5) | 3456 (12.7) |  |
|  | 60-69 years | 633 (11.6) | 820 (15.1) | 863 (15.5) | 794 (14.8) | 827 (15.4) | 3937 (14.5) |  |
|  | ≥70 years | 933 (17.0) | 1583 (29.2) | 1753 (31.5) | 1602 (29.9) | 1593 (29.7) | 7464 (27.4) |  |
|  | (Missing) | 1 (0.0) | 0 (0.0) | 2 (0.0) | 0 (0.0) | 2 (0.0) | 5 (0.0) |  |
| Sex | Male | 2458 (44.9) | 2470 (45.6) | 2748 (49.3) | 2698 (50.3) | 2630 (49.0) | 13004 (47.8) | <0.001 |
|  | Female | 3011 (55.0) | 2950 (54.4) | 2818 (50.6) | 2667 (49.7) | 2738 (51.0) | 14184 (52.1) |  |
|  | (Missing) | 4 (0.1) | 1 (0.0) | 5 (0.1) | 1 (0.0) | 3 (0.1) | 14 (0.1) |  |
| ASA grade | Grade 1 | 1634 (29.9) | 1039 (19.2) | 1222 (21.9) | 965 (18.0) | 1322 (24.6) | 6182 (22.7) | <0.001 |
|  | Grade 2 | 2274 (41.5) | 2004 (37.0) | 1855 (33.3) | 1859 (34.6) | 1763 (32.8) | 9755 (35.9) |  |
|  | Grades 3-5 | 1563 (28.6) | 2375 (43.8) | 2483 (44.6) | 2536 (47.3) | 2251 (41.9) | 11208 (41.2) |  |
|  | (Missing) | 2 (0.0) | 3 (0.1) | 11 (0.2) | 6 (0.1) | 35 (0.7) | 57 (0.2) |  |
| Revised cardiac risk index score | 0 | 2269 (41.5) | 2019 (37.2) | 2072 (37.2) | 1934 (36.0) | 1832 (34.1) | 10126 (37.2) | <0.001 |
|  | 1 | 2435 (44.5) | 2258 (41.7) | 2302 (41.3) | 2138 (39.8) | 2279 (42.4) | 11412 (42.0) |  |
|  | 2 | 553 (10.1) | 762 (14.1) | 769 (13.8) | 876 (16.3) | 889 (16.6) | 3849 (14.1) |  |
|  | ≥3 | 214 (3.9) | 381 (7.0) | 426 (7.6) | 417 (7.8) | 367 (6.8) | 1805 (6.6) |  |
|  | (Missing) | 2 (0.0) | 1 (0.0) | 2 (0.0) | 1 (0.0) | 4 (0.1) | 10 (0.0) |  |
| Indication for surgery | Benign | 3642 (66.5) | 3411 (62.9) | 3324 (59.7) | 3268 (60.9) | 3343 (62.2) | 16988 (62.5) | <0.001 |
|  | Cancer | 906 (16.6) | 843 (15.6) | 901 (16.2) | 800 (14.9) | 838 (15.6) | 4288 (15.8) |  |
|  | Trauma | 919 (16.8) | 1164 (21.5) | 1343 (24.1) | 1296 (24.2) | 1185 (22.1) | 5907 (21.7) |  |
|  | (Missing) | 6 (0.1) | 3 (0.1) | 3 (0.1) | 2 (0.0) | 5 (0.1) | 19 (0.1) |  |
| Urgency of surgery | Elective | 2363 (43.2) | 1932 (35.6) | 2225 (39.9) | 1530 (28.5) | 1754 (32.7) | 9804 (36.0) | <0.001 |
|  | Emergency | 3106 (56.8) | 3486 (64.3) | 3344 (60.0) | 3836 (71.5) | 3611 (67.2) | 17383 (63.9) |  |
|  | (Missing) | 4 (0.1) | 3 (0.1) | 2 (0.0) | 0 (0.0) | 6 (0.1) | 15 (0.1) |  |
| Grade of surgery | Minor | 1928 (35.2) | 1749 (32.3) | 1934 (34.7) | 1521 (28.3) | 1582 (29.5) | 8714 (32.0) | <0.001 |
|  | Major | 3525 (64.4) | 3658 (67.5) | 3630 (65.2) | 3828 (71.3) | 3768 (70.2) | 18409 (67.7) |  |
|  | (Missing) | 20 (0.4) | 14 (0.3) | 7 (0.1) | 17 (0.3) | 21 (0.4) | 79 (0.3) |  |
| Anaesthesia | General | 3754 (68.6) | 3950 (72.9) | 4014 (72.1) | 3900 (72.7) | 3782 (70.4) | 19400 (71.3) | <0.001 |
|  | Locoregional | 1706 (31.2) | 1461 (27.0) | 1547 (27.8) | 1457 (27.2) | 1569 (29.2) | 7740 (28.5) |  |
|  | (Missing) | 13 (0.2) | 10 (0.2) | 10 (0.2) | 9 (0.2) | 20 (0.4) | 62 (0.2) |  |
| SARS-CoV-2 diagnosis timing | Preoperative | 2950 (53.9) | 2937 (54.2) | 2634 (47.3) | 2883 (53.7) | 2746 (51.1) | 14150 (52.0) | <0.001 |
|  | Postoperative | 2507 (45.8) | 2445 (45.1) | 2933 (52.6) | 2481 (46.2) | 2618 (48.7) | 12984 (47.7) |  |
|  | (Missing) | 16 (0.3) | 39 (0.7) | 4 (0.1) | 2 (0.0) | 7 (0.1) | 68 (0.2) |  |
| Country income group | High | 3596 (65.7) | 4483 (82.7) | 4669 (83.8) | 3959 (73.8) | 3685 (68.6) | 20392 (75.0) | <0.001 |
|  | Upper middle | 1268 (23.2) | 549 (10.1) | 575 (10.3) | 740 (13.8) | 910 (16.9) | 4042 (14.9) |  |
|  | Lower middle or low | 609 (11.1) | 389 (7.2) | 327 (5.9) | 667 (12.4) | 776 (14.4) | 2768 (10.2) |  |

The analyses in Tables S5-S8 and Figure S1 relate to Pandemic Period 4 only

### **Table S5:** Baseline patient, disease, and operation characteristics during Pandemic Period 4, stratified by 30-day postoperative mortality

|  |  | **Alive** | **Died** | **p-value** |
| --- | --- | --- | --- | --- |
| Total |  | 18407 (94.2) | 1132 (5.8) |  |
| Age | <40 years | 7315 (98.6) | 105 (1.4) | <0.001 |
|  | 40-49 years | 2237 (97.0) | 69 (3.0) |  |
|  | 50-59 years | 2345 (93.9) | 153 (6.1) |  |
|  | 60-69 years | 2475 (90.8) | 251 (9.2) |  |
|  | ≥70 years | 4035 (87.9) | 554 (12.1) |  |
|  | (Missing) | 0 (NaN) | 0 (NaN) |  |
| Sex | Male | 8375 (92.4) | 684 (7.6) | <0.001 |
|  | Female | 10030 (95.7) | 448 (4.3) |  |
|  | (Missing) | 2 (100.0) | 0 (0.0) |  |
| ASA grade | Grade 1 | 4972 (99.0) | 51 (1.0) | <0.001 |
|  | Grade 2 | 7105 (97.2) | 203 (2.8) |  |
|  | Grades 3-5 | 6321 (87.8) | 878 (12.2) |  |
|  | (Missing) | 9 (100.0) | 0 (0.0) |  |
| Revised cardiac risk index score | 0 | 7610 (97.5) | 199 (2.5) | <0.001 |
|  | 1 | 7508 (95.3) | 373 (4.7) |  |
|  | 2 | 2245 (88.0) | 305 (12.0) |  |
|  | ≥3 | 1044 (80.4) | 255 (19.6) |  |
| Indication for surgery | Benign | 12036 (94.9) | 646 (5.1) | <0.001 |
|  | Cancer | 2654 (91.4) | 249 (8.6) |  |
|  | Trauma | 3716 (94.0) | 237 (6.0) |  |
|  | (Missing) | 1 (100.0) | 0 (0.0) |  |
| Urgency of surgery | Elective | 7800 (96.8) | 259 (3.2) | <0.001 |
|  | Emergency | 10607 (92.4) | 873 (7.6) |  |
|  | (Missing) | 0 (NaN) | 0 (NaN) |  |
| Grade of surgery | Minor | 6459 (96.6) | 226 (3.4) | <0.001 |
|  | Major | 11948 (93.0) | 906 (7.0) |  |
|  | (Missing) | 0 (NaN) | 0 (NaN) |  |
| Anaesthesia | General | 13002 (93.2) | 949 (6.8) | <0.001 |
|  | Locoregional | 5382 (96.7) | 183 (3.3) |  |
|  | (Missing) | 23 (100.0) | 0 (0.0) |  |
| SARS-CoV-2 diagnosis timing | Preoperative | 9763 (93.6) | 665 (6.4) | <0.001 |
|  | Postoperative | 8599 (94.8) | 467 (5.2) |  |
|  | (Missing) | 45 (100.0) | 0 (0.0) |  |
| SARS-CoV-2 vaccination | No | 6649 (92.6) | 529 (7.4) | <0.001 |
|  | Yes | 11758 (95.1) | 603 (4.9) |  |
| Country income group | High | 13565 (95.0) | 709 (5.0) | <0.001 |
|  | Upper middle | 2975 (91.5) | 277 (8.5) |  |
|  | Lower middle or low | 1867 (92.7) | 146 (7.3) |  |

### **Table S6:** Multilevel model for 30-day postoperative mortality during Pandemic Period 4 with binary SARS-CoV-2 vaccination status

|  |  | **Alive** | **Dead** | **OR (univariable)** | **OR (multivariable)** | **OR (multilevel)** |
| --- | --- | --- | --- | --- | --- | --- |
| Age | <40 years | 7315 (39.7) | 105 (9.3) | - | - | - |
|  | 40-49 years | 2237 (12.2) | 69 (6.1) | 2.46 (2.00-3.02, p<0.001) | 2.06 (1.65-2.55, p<0.001) | 1.93 (1.55-2.41, p<0.001) |
|  | 50-59 years | 2345 (12.7) | 153 (13.5) | 4.58 (3.85-5.46, p<0.001) | 2.96 (2.44-3.59, p<0.001) | 2.81 (2.31-3.42, p<0.001) |
|  | 60-69 years | 2475 (13.4) | 251 (22.2) | 7.17 (6.12-8.44, p<0.001) | 4.20 (3.50-5.05, p<0.001) | 4.08 (3.38-4.93, p<0.001) |
|  | ≥70 years | 4035 (21.9) | 554 (48.9) | 11.57 (10.03-13.42, p<0.001) | 6.54 (5.51-7.80, p<0.001) | 6.46 (5.38-7.76, p<0.001) |
| Sex | Male | 8375 (45.5) | 684 (60.4) | - | - | - |
|  | Female | 10030 (54.5) | 448 (39.6) | 0.60 (0.56-0.65, p<0.001) | 0.72 (0.66-0.78, p<0.001) | 0.72 (0.66-0.78, p<0.001) |
| ASA grade | Grade 1 | 4972 (27.0) | 51 (4.5) | - | - | - |
|  | Grade 2 | 7105 (38.6) | 203 (17.9) | 3.40 (2.75-4.24, p<0.001) | 1.89 (1.51-2.39, p<0.001) | 2.02 (1.60-2.55, p<0.001) |
|  | Grades 3-5 | 6321 (34.4) | 878 (77.6) | 14.65 (12.02-18.07, p<0.001) | 4.80 (3.84-6.06, p<0.001) | 5.65 (4.47-7.13, p<0.001) |
| Revised cardiac risk index score | 0 | 7610 (41.3) | 199 (17.6) | - | - | - |
|  | 1 | 7508 (40.8) | 373 (33.0) | 1.64 (1.48-1.82, p<0.001) | 1.19 (1.06-1.34, p=0.003) | 1.16 (1.03-1.31, p=0.012) |
|  | 2 | 2245 (12.2) | 305 (26.9) | 3.90 (3.48-4.37, p<0.001) | 1.52 (1.33-1.73, p<0.001) | 1.45 (1.26-1.66, p<0.001) |
|  | ≥3 | 1044 (5.7) | 255 (22.5) | 6.06 (5.33-6.89, p<0.001) | 1.99 (1.72-2.32, p<0.001) | 1.98 (1.69-2.32, p<0.001) |
| Indication for surgery | Benign | 12036 (65.4) | 646 (57.1) | - | - | - |
|  | Cancer | 2654 (14.4) | 249 (22.0) | 1.54 (1.39-1.70, p<0.001) | 1.35 (1.19-1.53, p<0.001) | 1.41 (1.24-1.60, p<0.001) |
|  | Trauma | 3716 (20.2) | 237 (20.9) | 1.49 (1.36-1.63, p<0.001) | 0.97 (0.87-1.08, p=0.571) | 0.96 (0.85-1.08, p=0.487) |
| Urgency of surgery | Elective | 7800 (42.4) | 259 (22.9) | - | - | - |
|  | Emergency | 10607 (57.6) | 873 (77.1) | 2.52 (2.29-2.77, p<0.001) | 2.50 (2.23-2.81, p<0.001) | 2.58 (2.28-2.92, p<0.001) |
| Grade of surgery | Minor | 6459 (35.1) | 226 (20.0) | - | - | - |
|  | Major | 11948 (64.9) | 906 (80.0) | 1.95 (1.78-2.14, p<0.001) | 1.39 (1.26-1.55, p<0.001) | 1.36 (1.22-1.51, p<0.001) |
| Anaesthesia | General | 13002 (70.7) | 949 (83.8) | - | - | - |
|  | Locoregional | 5382 (29.3) | 183 (16.2) | 0.64 (0.58-0.70, p<0.001) | 0.80 (0.72-0.89, p<0.001) | 0.72 (0.64-0.81, p<0.001) |
| SARS-CoV-2 diagnosis timing | Preoperative | 9763 (53.2) | 665 (58.7) | - | - | - |
|  | Postoperative | 8599 (46.8) | 467 (41.3) | 1.22 (1.14-1.32, p<0.001) | 1.01 (0.92-1.10, p=0.908) | 0.96 (0.88-1.06, p=0.457) |
| SARS-CoV-2 vaccination | No | 6649 (36.1) | 529 (46.7) | - | - | - |
|  | Yes | 11758 (63.9) | 603 (53.3) | 0.36 (0.33-0.40, p<0.001) | 0.36 (0.32-0.39, p<0.001) | 0.35 (0.31-0.39, p<0.001) |
| Country income group | High | 13565 (73.7) | 709 (62.6) | - | - | - |
|  | Upper middle | 2975 (16.2) | 277 (24.5) | 1.04 (0.94-1.16, p=0.430) | 2.33 (2.06-2.63, p<0.001) | 2.61 (1.89-3.60, p<0.001) |
|  | Lower middle or low | 1867 (10.1) | 146 (12.9) | 1.07 (0.94-1.21, p=0.309) | 3.27 (2.81-3.81, p<0.001) | 3.47 (2.41-5.02, p<0.001) |

** Hospital and country were random effects. Income group, age, gender, ASA grade, revised cardiac risk index, urgency of surgery, indication of surgery, grade of surgery, type of anaesthesia and timing of COVID-19 diagnosis.*

### **Figure S1:** Multilevel model for 30-day postoperative mortality during Pandemic Period 4 with binary SARS-CoV-2 vaccination status

**
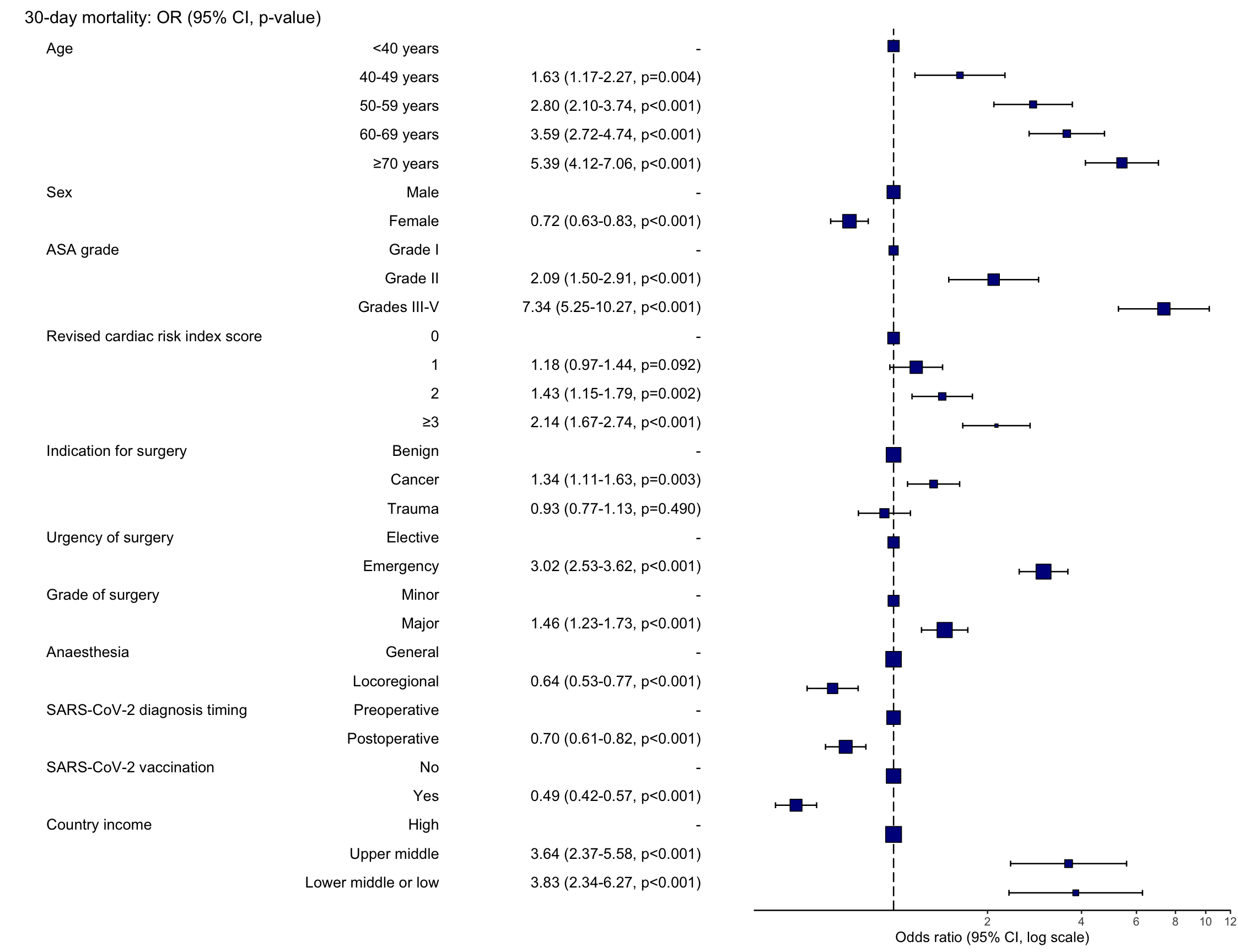
**

**In the multilevel model, hospitals and country were included as random effects and the remaining variables as fixed effects.*

### **Table S7:** Baseline patient, disease, and operation characteristics in Pandemic Period 4, stratified by hospital-level Surgery Preparedness Index score

|  |  | **Poorly-prepared** | **Moderately-prepared** | **Highly-prepared** | **Total** | **p-value** |
| --- | --- | --- | --- | --- | --- | --- |
| Total |  | 5705 (31.1) | 5692 (31.1) | 6923 (37.8) | 18320 |  |
| Age | <40 years | 2227 (39.0) | 2066 (36.3) | 2642 (38.2) | 6935 (37.9) | 0.012 |
|  | 40-49 years | 640 (11.2) | 683 (12.0) | 838 (12.1) | 2161 (11.8) |  |
|  | 50-59 years | 717 (12.6) | 700 (12.3) | 916 (13.2) | 2333 (12.7) |  |
|  | 60-69 years | 785 (13.8) | 817 (14.4) | 963 (13.9) | 2565 (14.0) |  |
|  | ≥70 years | 1335 (23.4) | 1426 (25.1) | 1564 (22.6) | 4325 (23.6) |  |
|  | (Missing) | 1 (0.0) | 0 (0.0) | 0 (0.0) | 1 (0.0) |  |
| Sex | Male | 2643 (46.3) | 2642 (46.4) | 3213 (46.4) | 8498 (46.4) | 0.995 |
|  | Female | 3061 (53.7) | 3050 (53.6) | 3709 (53.6) | 9820 (53.6) |  |
|  | (Missing) | 1 (0.0) | 0 (0.0) | 1 (0.0) | 2 (0.0) |  |
| ASA grade | Grade 1 | 1615 (28.3) | 1341 (23.6) | 1790 (25.9) | 4746 (25.9) | <0.001 |
|  | Grade 2 | 2158 (37.8) | 2198 (38.6) | 2524 (36.5) | 6880 (37.6) |  |
|  | Grades 3-5 | 1928 (33.8) | 2151 (37.8) | 2604 (37.6) | 6683 (36.5) |  |
|  | (Missing) | 4 (0.1) | 2 (0.0) | 5 (0.1) | 11 (0.1) |  |
| Revised cardiac risk index score | 0 | 2262 (39.6) | 2125 (37.3) | 2895 (41.8) | 7282 (39.7) | <0.001 |
|  | 1 | 2229 (39.1) | 2437 (42.8) | 2779 (40.1) | 7445 (40.6) |  |
|  | 2 | 810 (14.2) | 784 (13.8) | 805 (11.6) | 2399 (13.1) |  |
|  | ≥3 | 404 (7.1) | 346 (6.1) | 444 (6.4) | 1194 (6.5) |  |
| Indication for surgery | Benign | 3740 (65.6) | 3641 (64.0) | 4514 (65.2) | 11895 (64.9) | 0.218 |
|  | Cancer | 823 (14.4) | 865 (15.2) | 1059 (15.3) | 2747 (15.0) |  |
|  | Trauma | 1140 (20.0) | 1184 (20.8) | 1349 (19.5) | 3673 (20.0) |  |
|  | (Missing) | 2 (0.0) | 2 (0.0) | 1 (0.0) | 5 (0.0) |  |
| Urgency of surgery | Elective | 2167 (38.0) | 2415 (42.4) | 2958 (42.7) | 7540 (41.2) | <0.001 |
|  | Emergency | 3537 (62.0) | 3275 (57.5) | 3964 (57.3) | 10776 (58.8) |  |
|  | (Missing) | 1 (0.0) | 2 (0.0) | 1 (0.0) | 4 (0.0) |  |
| Grade of surgery | Minor | 1940 (34.0) | 1954 (34.3) | 2401 (34.7) | 6295 (34.4) | 0.728 |
|  | Major | 3764 (66.0) | 3736 (65.6) | 4521 (65.3) | 12021 (65.6) |  |
|  | (Missing) | 1 (0.0) | 2 (0.0) | 1 (0.0) | 4 (0.0) |  |
| Anaesthesia | General | 3927 (68.8) | 4123 (72.4) | 4987 (72.0) | 13037 (71.2) | <0.001 |
|  | Locoregional | 1771 (31.0) | 1565 (27.5) | 1935 (28.0) | 5271 (28.8) |  |
|  | (Missing) | 7 (0.1) | 4 (0.1) | 1 (0.0) | 12 (0.1) |  |
| SARS-CoV-2 diagnosis timing | Preoperative | 3091 (54.2) | 2833 (49.8) | 3809 (55.0) | 9733 (53.1) | <0.001 |
|  | Postoperative | 2570 (45.0) | 2857 (50.2) | 3114 (45.0) | 8541 (46.6) |  |
|  | (Missing) | 44 (0.8) | 2 (0.0) | 0 (0.0) | 46 (0.3) |  |
| SARS-CoV-2 vaccination | No | 2258 (39.6) | 2008 (35.3) | 2393 (34.6) | 6659 (36.3) | <0.001 |
|  | Yes | 3447 (60.4) | 3684 (64.7) | 4530 (65.4) | 11661 (63.7) |  |
| Country income group | High | 3556 (62.3) | 4390 (77.1) | 5473 (79.1) | 13419 (73.2) | <0.001 |
|  | Upper middle | 1232 (21.6) | 824 (14.5) | 846 (12.2) | 2902 (15.8) |  |
|  | Lower middle or low | 917 (16.1) | 478 (8.4) | 604 (8.7) | 1999 (10.9) |  |
| 30-day mortality | No | 5258 (92.2) | 5379 (94.5) | 6610 (95.5) | 17247 (94.1) | <0.001 |
|  | Yes | 436 (7.6) | 302 (5.3) | 299 (4.3) | 1037 (5.7) |  |
|  | (Missing) | 11 (0.2) | 11 (0.2) | 14 (0.2) | 36 (0.2) |  |

### **Table S8:** Multilevel model for 30-day postoperative mortality in Pandemic Period 4, including hospital-level Surgical Pandemic Preparedness Index score

|  |  | **No** | **Yes** | **OR (univariable)** | **OR (multivariable)** | **OR (multilevel)** |
| --- | --- | --- | --- | --- | --- | --- |
| Hospital SPI rating | Poorly-prepared | 5258 (30.5) | 436 (42.0) | - | - | - |
|  | Moderately-prepared | 5379 (31.2) | 302 (29.1) | 0.68 (0.58-0.79, p<0.001) | 0.75 (0.64-0.88, p=0.001) | 0.72 (0.60-0.86, p<0.001) |
|  | Highly-prepared | 6610 (38.3) | 299 (28.8) | 0.55 (0.47-0.63, p<0.001) | 0.64 (0.54-0.76, p<0.001) | 0.66 (0.55-0.79, p<0.001) |
| Age | <40 years | 7315 (39.7) | 105 (9.3) | - | - | - |
|  | 40-49 years | 2237 (12.2) | 69 (6.1) | 2.15 (1.57-2.91, p<0.001) | 1.88 (1.33-2.64, p<0.001) | 1.72 (1.21-2.44, p=0.002) |
|  | 50-59 years | 2345 (12.7) | 153 (13.5) | 4.55 (3.54-5.86, p<0.001) | 3.03 (2.25-4.09, p<0.001) | 2.95 (2.18-4.00, p<0.001) |
|  | 60-69 years | 2475 (13.4) | 251 (22.2) | 7.07 (5.62-8.95, p<0.001) | 4.02 (3.04-5.35, p<0.001) | 3.93 (2.95-5.24, p<0.001) |
|  | ≥70 years | 4035 (21.9) | 554 (48.9) | 9.57 (7.77-11.88, p<0.001) | 5.75 (4.39-7.59, p<0.001) | 5.58 (4.21-7.38, p<0.001) |
| Sex | Male | 8375 (45.5) | 684 (60.4) | - | - | - |
|  | Female | 10030 (54.5) | 448 (39.6) | 0.55 (0.48-0.62, p<0.001) | 0.72 (0.63-0.83, p<0.001) | 0.73 (0.63-0.84, p<0.001) |
| ASA grade | Grade 1 | 4972 (27.0) | 51 (4.5) | - | - | - |
|  | Grade 2 | 7105 (38.6) | 203 (17.9) | 2.79 (2.06-3.83, p<0.001) | 1.99 (1.41-2.86, p<0.001) | 2.19 (1.53-3.14, p<0.001) |
|  | Grades 3-5 | 6321 (34.4) | 878 (77.6) | 13.54 (10.30-18.22, p<0.001) | 5.78 (4.11-8.30, p<0.001) | 7.21 (5.04-10.32, p<0.001) |
| Revised cardiac risk index score | 0 | 7610 (41.3) | 199 (17.6) | - | - | - |
|  | 1 | 7508 (40.8) | 373 (33.0) | 1.90 (1.60-2.27, p<0.001) | 1.20 (0.98-1.46, p=0.074) | 1.17 (0.96-1.43, p=0.120) |
|  | 2 | 2245 (12.2) | 305 (26.9) | 5.20 (4.32-6.26, p<0.001) | 1.52 (1.22-1.90, p<0.001) | 1.42 (1.13-1.78, p=0.003) |
|  | ≥3 | 1044 (5.7) | 255 (22.5) | 9.34 (7.68-11.38, p<0.001) | 2.15 (1.69-2.75, p<0.001) | 2.04 (1.58-2.62, p<0.001) |
| Indication for surgery | Benign | 12036 (65.4) | 646 (57.1) | - | - | - |
|  | Cancer | 2654 (14.4) | 249 (22.0) | 1.75 (1.50-2.03, p<0.001) | 1.36 (1.13-1.64, p=0.001) | 1.35 (1.11-1.63, p=0.002) |
|  | Trauma | 3716 (20.2) | 237 (20.9) | 1.19 (1.02-1.38, p=0.027) | 0.89 (0.73-1.07, p=0.201) | 0.95 (0.78-1.15, p=0.610) |
| Urgency of surgery | Elective | 7800 (42.4) | 259 (22.9) | - | - | - |
|  | Emergency | 10607 (57.6) | 873 (77.1) | 2.48 (2.15-2.86, p<0.001) | 2.40 (2.02-2.87, p<0.001) | 2.75 (2.30-3.29, p<0.001) |
| Grade of surgery | Minor | 6459 (35.1) | 226 (20.0) | - | - | - |
|  | Major | 11948 (64.9) | 906 (80.0) | 2.17 (1.87-2.52, p<0.001) | 1.48 (1.25-1.76, p<0.001) | 1.40 (1.17-1.66, p<0.001) |
| Anaesthesia | General | 13002 (70.7) | 949 (83.8) | - | - | - |
|  | Locoregional | 5382 (29.3) | 183 (16.2) | 0.47 (0.40-0.55, p<0.001) | 0.65 (0.53-0.78, p<0.001) | 0.61 (0.50-0.74, p<0.001) |
| SARS-CoV-2 diagnosis timing | Preoperative | 9763 (53.2) | 665 (58.7) | - | - | - |
|  | Postoperative | 8599 (46.8) | 467 (41.3) | 0.80 (0.71-0.90, p<0.001) | 0.70 (0.61-0.81, p<0.001) | 0.71 (0.62-0.83, p<0.001) |
| SARS-CoV-2 vaccination | No | 6649 (36.1) | 529 (46.7) | - | - | - |
|  | Yes | 11758 (63.9) | 603 (53.3) | 0.64 (0.57-0.73, p<0.001) | 0.48 (0.41-0.55, p<0.001) | 0.50 (0.43-0.59, p<0.001) |
| Country income group | High | 13565 (73.7) | 709 (62.6) | - | - | - |
|  | Upper middle | 2975 (16.2) | 277 (24.5) | 1.78 (1.54-2.06, p<0.001) | 2.72 (2.27-3.25, p<0.001) | 3.29 (2.16-5.01, p<0.001) |
|  | Lower middle or low | 1867 (10.1) | 146 (12.9) | 1.50 (1.24-1.79, p<0.001) | 3.19 (2.55-3.98, p<0.001) | 3.11 (1.92-5.05, p<0.001) |

**For the multivariable analysis, the following variables were included as fixed effects: pandemic periods, income group, age, gender, ASA grade, revised cardiac risk index, urgency of surgery, indication of surgery, grade of surgery, type of anaesthesia and timing of SARS-CoV-2 diagnosis. For the multilevel analysis, hospital and country were included as random effects.*

# Appendix

### **Appendix A:** Description of the pandemic periods

1. Pandemic period 1: 1 January to 30 April 2020. Infections were caused by the wild-type strain, the original strain of SARS-CoV-2, leading to the first wave of infections and nationwide lockdowns.
2. Pandemic period 2: 1 May to 31 July 2020. Infections were caused by the wild-type strain.
3. Pandemic period 3: 5 October 2020 to 1 November 2020. Infections were caused by a combination of the Wild-Type Strain, Alpha variant (B.1.1.7; first identified in the UK in September 2020, which led to a surge of cases), and the Delta variant (B.1.617.2; first detected in India in October 2020, which also led to a surge of cases).
4. Pandemic period 4: 13 December 2021 to 28 February 2022. Infections were increasingly caused by the Omicron variant (B.1.1.529; first identified in South Africa in November 2021, which led to a surge of cases)

### **Appendix B:** Data integrity processes in the cohort studies

- Local principal investigators and team members underwent online training prior to the start of the study.
- Investigators in each hospitals were required to confirm eligibility of each patient on REDCap prior to data entry.
- If inconsistent data was entered for a patient, automated trigger warnings would appear to alert investigators of this error.
- Investigators were provided with an automated REDCap report to identify patient records with missing data. Prior to the database lock, investigators were emailed reminders to complete missing data, if possible.

### **Appendix C:** Summary of the Surgical Preparedness Index domains

| **Facilities and consumables** |
| --- |
| 1. Availability of reserved planned surgery theatres (ring-fenced theatres) |
| 1. Availability of reserved planned surgery beds (ring-fenced beds) |
| 1. Availability of reserved critical care beds for planned surgery (ring-fenced critical care) |
| 1. Flexibility to rearrange hospital areas to provide a segregated pathway for planned surgery (flexible areas) |
| 1. Access to diagnostics and interventions to identify and treat surgical complications (managing complications) |
| 1. Reliable supply of electricity (electricity supply) |
| 1. Reliable supply of supplementary oxygen (oxygen supply) |
| 1. Reliable supply and management of essential perioperative drugs (drug supply) |
| 1. Reliable supply and management of devices and implants (device supply) |
| 1. Sufficient surgical instrument and local sterilisation processes (sterilisation) |
| 1. Availability of protective measures for theatre teams (protective equipment) |
| **Staffing** |
| 1. Ability to redistribute staff within and between hospitals to maintain capacity (staff redistribution) |
| 1. Availability of reserved teams to provide planned surgical care (ring-fenced teams) |
| **Prioritisation** |
| 1. Cross-specialty patient prioritisation for surgery (patient prioritisation) |
| 1. Ability to identify and cancel procedures of limited clinical value (procedure prioritisation) |
| **Systems** |
| 1. Formal operational plan to continue planned surgery during external system shocks (formal plan) |
| 1. Ability to do preoperative assessment in the community (preoperative assessment) |
| 1. Access to routine preoperative testing for endemic and epidemic diseases (preoperative testing) |
| 1. Ability to transfer patients to another hospital with greater capacity (hospital transfer) |
| 1. Ability to facilitate timely discharges (timely discharge) |
| 1. Social support system to facilitate safe discharge (social support) |
| 1. Capacity to use telephone or video calls for outpatient appointments (remote outpatient appointments) |
| 1. Capacity and capability to communicate with family members (family communication) |

### **Appendix D:** Statistical methodology

*Statistical tests*

The χ2 test was used for categorical data. Non-parametric data summarised with medians and interquartile ranges and differences between the groups were tested using the Mann-Whitney U test. Parametric data were summarised with mean and standard deviation. Differences between groups were explored using a two-tailed Student’s t-test (two comparator groups) or one-way Analysis of Variance (ANOVA, three or more comparator groups).

*Multilevel logistic regression variable selection*

Multilevel logistic regression models were constructed to account for clinically plausible patient, disease, and operation factors. A total of ten variables were selected a priori. Variable selection was informed by the findings of previously published analyses of the CovidSurg-1^1^ and CovidSurg-2^2^ datasets. There were five variables that were found to be statistically significantly associated with postoperative mortality in patients with perioperative SARS-CoV-2 infection in both studies (see tabulation below). A further three variables were found to be statistically significantly associated with postoperative mortality in either CovidSurg-1 or CovidSurg-2. These eight variables were all selected to be included in the multilevel logistic regression models in this study. Two further variables were selected a priori by agreement of the statistics group. Anaesthesia modality had not been included in the models in the previous studies but was felt to be a clinically plausible risk factor. Timing of SARS-CoV-2 diagnosis was not statistically significant in CovidSurg-1, but this was based on SARS-CoV-2 testing availability and practices during early 2020, so it was felt that it was clinically plausible that the association of timing of diagnosis and mortality might have changed later in the pandemic and this variable was selected for this study.

**Mapping of multilevel logistic regression variable selection for this study compared to previous CovidSurg studies**

| **Variable** | **Identification as risk factor for postoperative mortality** | | **Included in this study** |
| --- | --- | --- | --- |
|  | **CovidSurg-1** | **CovidSurg-2** |  |
| Age | Significant | Significant | Yes |
| Sex | Significant | Not significant | Yes |
| ASA grade | Significant | Significant | Yes |
| Revised cardiac risk index | Not included in model | Significant | Yes |
| Number of comorbidities | Not significant | Not included | No |
| Respiratory complications | Not included in model | Not significant | No |
| White cell count | Not significant | Not included in model | No |
| Surgery indication | Significant | Significant | Yes |
| Surgery urgency | Significant | Significant | Yes |
| Grade of surgery | Significant | Significant | Yes |
| Anaesthesia modality | Not included in model | Not included in model | Yes |
| Preoperative vs. postoperative SARS-CoV-2 | Not significant | Not applicable | Yes |
| Country income group | Not included in model | Significant | Yes |

*The main adjusted model for postoperative mortality in patients with perioperative SARS-CoV-2 infection in CovidSurg-1 is presented in Figure 3 of the published paper^1^. The adjusted model for postoperative mortality in patients with SARS-CoV-2 infection in CovidSurg-2 is presented in Supplementary Table S7 of the published paper^2^; since this analysis only included patients with preoperative SARS-CoV-2, preoperative versus postoperative SARS-CoV-2 infection diagnosis timing is not applicable.*

*Multilevel logistic regression models*

Multilevel logistic regression was used to describe relationship between explanatory variable and 30-day mortality for both the primary and secondary analysis. Models were constructed using the following principles: (i) variables associated with outcome measures in previous studies were accounted for; (ii) population stratification by hospital or country of residence was incorporated as random effects with constrained gradients; (iii) all first-order interactions were checked and included in final models if found to be influential; and (iv) final model selection was done using a criterion-based approach by minimising the widely applicable information criterion (AIC) and discrimination determined using the c-statistic (area under the receiver operator curve ). Model coefficients are presented as odds ratio (OR) and 95% confidence intervals. A complete-case analysis was pre-planned if missing data were minimal (<5%) and missing at random. For missingness exceeding 5%, multiple imputation by chained equations was planned, assuming data were missing at random or completely at random, as outlined in the study protocols. For the SPI, tertiles were developed based on the following calculations (−∞, 𝑥[33] ], (𝑥[33], 𝑥[66] ] and (𝑥[66], +∞) where 𝑥[33], and 𝑥[66] are, are, respectively, the 33rd, and 66th percentiles of SPI.

^1^ COVIDSurg Collaborative. Mortality and pulmonary complications in patients undergoing surgery with perioperative SARS-CoV-2 infection: an international cohort study. Lancet. 2020 Jul 4;396(10243):27-38.

^2^ COVIDSurg Collaborative; GlobalSurg Collaborative. Timing of surgery following SARS-CoV-2 infection: an international prospective cohort study. Anaesthesia. 2021 Jun;76(6):748-758.

### **Appendix E:** Authorship

All individuals listed are PubMed-citable co-authors

**Writing group:** Dmitri Nepogodiev* (UK), Sivesh K Kamarajah* (UK), Radhika Acharya (UK), Waheed-Ul-Rahman Ahmed (UK), Ehab AlAmeer (Saudi Arabia), Ruth Blanco-Colino (Spain), Muhammed Elhadi (Libya), Dhruva Ghosh (India), James C Glasbey (UK), Arda Isik (Turkey), Kate Jolly (UK), Haytham Kaafarani (United States), Bryar Kadir (UK), Hans Lederhuber (Germany), Sezai Leventoğlu (Turkey), Omar M Omar (UK), Francesco Pata (Italy), Maria Picciochi (Portugal), Peter Pockney (Australia), Marie Dione Sacdalan (Philippines), Joana FF Simoes (Portugal), Georgios Tsoulfas (Greece), Aneel Bhangu^†^ (UK).

* joint first authors

^†^ senior author

**Statistics group:** Aneel Bhangu, Bryar Kadir, Sivesh K Kamarajah, Dmitri Nepogodiev, Omar M Omar. These statistics group had full access to the data in this study.

**CovidSurg-3 authors**

**Steering group:** Radhika Aacharya, Aneel Bhangu, Daoud Chaudhry, Ruth Blanco Colino, Irani Duran, Muhammed Elhadi, James Glasbey, Rohan Gujjuri, Sivesh Kamarajah, Santhosh Karri, Kayani Kayani, Stephen Knight, Samuel Lawday, Elizabeth Li, Harvi Mann, Fatima Mansour, Kenneth McLean, Dmitri Nepogodiev, Omar Omar, Maria Piccochi, Irene Santos, Joana Simoes, Chris Varghese.

**National leads/Dissemination Committee:**

Afghanistan: J Shah, Albania: I Dajti, A Gjata, Algeria: K Oussama, Argentina: L Boccalatte, MM  Modolo, Australia: P Pockney, K Raubenheimer, P Townend, Austria: F Aigner, Azerbaijan: E Samed, Bahrain: A Alderazi, Belgium: G VanRamshorst, Benin: K Fagnon, Bosnia and Herzegovina: C Anis, Brazil: G Baiocchi, I Buarque, G Mendonça Ataíde Gomes, Bulgaria: M Gohar, M Slavchev, Cameroon: G Ngock, C Nwegbu, Canada: A Brar, J Martin, Chile: MM  Modolo, M Olivos, China: W Yang, Colombia: J Calvache, G Mendiola Barrios, C Perez Rivera, Croatia: A Danic Hadzibegovic, T Kopjar, J Mihanovic, Cuba: O Olazábal, Czech Republic: R Novysedlak, Democratic Republic of Congo: D Masheka, Denmark: A El-Hussuna, Dominican Republic: S Batista, Ecuador: E Lincango-Naranjo, Egypt: S Emile, Ethiopia: M Gebreyohanes Mengesha, S Hailu, H Tamiru, M Worku, Finland: J Kauppila, France: A Arnaud, Georgia: Z Demetrashvili, Germany: M Albertsmeier, H Lederhuber, MW Löoffler, Ghana: B Ofori Appiah, S Tabiri, Greece: C Christou, G Tsoulfas, Guatemala: M Aguilera Lorena, G Grecinos, Hong Kong: K Futaba, Hungary: A Zarand, India: D Ghosh, T Goel, R Jain, Indonesia: G Kembuan, W Lie, Iran, Islamic Rep.: P Brouk, M Khosravi, M Mozafari, Iraq: HT Hashim, Ireland: C Cullinane, H Mohan, Israel: N Horesh, G Marom, Italy: G Gallo, F Pata, G Pelino, Japan: Y Fujimoto, N Kuroda, S Satoi, Jordan: F Ayasra, Kazakhstan: I R Fakhradiyev, Kenya: I Hisham, Kosovo: E Fekaj, Kuwait: M Jamal, Latvia: IA Apse, Lebanon: L Karout, Libya: M Elhadi, Lithuania: A Gulla, Madagascar: J Neny, L Samison, Malaysia: A Roslani, Mexico: I Duran Sanchez, DS Gonzalez Vazquez, MJ Martinez Lara, L Martinez Perez Maldonado, A Ramos De La Medina, Mongolia: S Erdene, J Nunez, Morocco: O Outani, Namibia: A Nashidengo, Nepal: R Shrestha, Netherlands: RND Hogenbirk, PKC Jonker, S Kruijff, P Steinkamp, WY van der Plas, New Zealand: S Farik, C Wells, W Xu, Nigeria: L Abdur-Rahman, A Ademuyiwa, AO Adisa, H Ekwunife, T Lawal, J Seyi-olajide, O Williams, North Macedonia: S Pejkova, Oman: Z Al Balushi, Pakistan: AU Qureshi, R Sayyed, Palestine: M Abu Mohsen Daraghmeh, S Abukhalaf, Panama: M Cukier, Paraguay: H Gomez Fernandez, Peru: X Vasquez, Philippines: MD Parreno-Sacdalan, Poland: P Major, M Waledziak, Portugal: G Nobre, Romania: E Bonci, I Negoi, Russia: V Kochetkov, S Efetov, A Litvin, Rwanda: A Ingabire, E Muntaneza, F Ntirenganya, Saudi Arabia: E AlAmeer, Serbia: D Radenkovic, Singapore: F Koh Hong Xiang, Slovak Republic: A Panyko, Slovenia: J Kosir, Somalia: H Ali, South Africa: B Biccard, Spain: R Blanco-Colino, A Minaya Bravo, Sri Lanka: U Jayarajah, D Wickramasinghe, Sudan: ME Adam Essa, A Mohammed, Sweden: M Rutegård, M Sund, Switzerland: M Adamina, E Gialamas, Syrian Arab Republic: M Alshaar, Tanzania: B Mbwele, Thailand: V Lohsiriwat, Trinidad and Tobago: S Charles, Tunisia: A Houssem , Turkey: A Isik, S Leventoğlu , Uganda: L Herve, H Lule, United Arab Emirates: H Alsaadi, S Alshryda, United Kingdom: C Desai, H Panesar, United States: D Argandykov, B Bankhead-Kendall, H Kaafarani, A Renne, Uruguay: F Bonilla, Yemen, Rep.: H Al Naggar.

| **Hospital leads** |
| --- |
| Afghanistan: M Delsoz (Kabul; NOOR Eye Hospital). |
| Albania: D Thereska (Tirana; University Hospital Center Nene Tereza); I Dajti (Tirana; University hospital Koco Gliozheni); L Zijaj (Vlora; Regional Hospital of Vlora). |
| Algeria: K Djebabria (Annaba; Centre Hospitalier Universitaire Ibn Rochd); Z Djama (Constantine; university hospital abdelhamid ben badis); A Laredj (Oran; EHS-Canastel); A Tidjane, J Mansouri (Oran; EHU-1st November 1954); M Abdoun (Setif; Hospitalo-Universitaire Saadna Abdennour, Ferhat Abbas University); O Riffi (Tlemcen; Infant-Mother Hospital of Tlemcen); SN Mesli (Tlemcen; University Hospital Center, Dr Tidjani Damerdji). |
| Argentina: SM Lucchini (Allende, Cordoba; Sanatorio Allende - Sede Cerro); L Boccalatte, RM Palacios Huatuco, F Padilla-Lichtenberger (Buenos Aires; Hospital Italiano de Buenos Aires); C Chwat (Buenos Aires; Hospital Universitario Austral); DA Pantoja Pachajoa (Cordoba; Clinica Universitaria Reina Fabiola); SM Lucchini (Cordoba; Sanatorio Allende - Sede Nueva Cordoba); A Duro (Provincia de Buenos Aires; Hospital Prof Dr Bernardo A Houssay); R Balmaceda, L Affronti (San juan; Sanatorio Argentino). |
| Aruba: M Gosselink (Oranjestad; Dr. Horacio E Oduber Hospital). |
| Australia: YH Lam (Adelaide; Flinders Medical Centre); SW Gan (Adelaide; Royal Adelaide Hospital); J Gundara (Brisbane; Logan Hospital); A Frankel (Brisbane; Princess Alexandra Hospital); S Bowman (Brisbane; Queen Elizabeth 2 Jubilee Hospital); M Roberts (Brisbane; Royal Brisbane and Women’s Hospital); MZ Zhu (Canberra; Canberra Hospital); S Salindera (Coffs Harbour NSW; Coffs Harbour Health Campus); T Asgill (Geelong; University Hospital Geelong); EWY Lun (Gosford; Gosford Hospital); AC Dawson (Gosford; Gosford Private Hospital); D Townend (Lismore; Lismore Base Hospital); KR Qin (Melbourne; Austin Hospital); M Pacilli (Melbourne; Monash Childrens Hospital); A Gray (Melbourne; Monash Health Casey Hospital); R Hodgson, A Khodarahmi (Melbourne; Northern Hospital); SS Apte (Melbourne; Peter MacCallum Cancer Centre); P Choong (Melbourne; St Vincent’s Hospital); J Lee (Melbourne; The Alfred Hospital); S Lidder (Melbourne; The Royal Melbourne Hospital); B Lauritz (Melbourne; Western Health - Footscray hospital and Sunshine hospital); M McLeod (Newcastle; Calvary Mater Newcastle); C O’Neill (Newcastle; John Hunter Hospital); S Bhat (Perth; Royal Perth Hospital); R Manley (Robina; Robina Hospital); M Cooper (Southport; Gold Coast University Hospital); N Merrett, C Berney (Sydney; Bankstown Hospital); C Apostolou (Sydney; Northern Beaches Hospital); C Nahm (Westmead; Westmead Hospital); U Pahalawatta (Wyong; Wyong Public Hospital). |
| Austria: A Leitner (Dornbirn; Krankenhaus der Stadt Dornbirn); I Königsrainer (Feldkirch; Landeskrankenhaus Feldkirch); H Hauser (Graz-West; Landeskrankenhaus); F Aigner (Graz; Barmherzige Brüder Krankenhaus, Graz); DB Lumenta, D Wagner (Graz; Medical University of Graz); TO Andraschofsky (Hall in Tirol; Landeskrankenhaus Hall); D Öfner (Innsbruck; Innsbruck Medical University); J Huber (Linz; Ordensklinikum Linz Elisabethinen); M Biebl (Linz; Ordensklinikum Linz GmbH Barmherzige Schwestern); J Presl (Salzburg; Paracelsus Medical University Salzburg); E Russe (Salzburg; Saint John of God Hospital Salzburg); A Binder (Tulln; Universitätsklinikum Tulln); M Zimmermann (Vienna; General Hospital of Vienna); CG Wiesinger (Wels; Klinikum Wels-Grieskirchen GmbH); F Trivik-Barrientos (Wiener Neustadt; Landesklinikum Wiener Neustadt). |
| Azerbaijan: E Samadov (Baku; Leyla Medical Center). |
| Bangladesh: M Shadrul Alam (Dhaka; Dhaka Medical College Hospital). |
| Belgium: N Komen (Antwerp; University Hospital Antwerp); B Dhondt (Bornem; AZ Rivierenland); J De Ceulaer (Brugge; AZ Sint-Jan Brugge-Oostende AV); G Van Ramshorst (Gent; University Hospital of Ghent). |
| Benin: F Kethy (Cotonou; Centre National Hospitalier et Universitaire Hubert Koutoukou Maga). |
| Bosnia and Herzegovina: M Soljic (Mostar; SKB University Clinical Hospital Mostar); S Bajramovic (Sarajevo; Clinical Center University of Sarajevo); A Cerovac (Zenica; Cantonal Hospital Zenica). |
| Brazil: R Esteves Pires (Belo Horizonte; Felicio Rocho); FAL Marson (Bragança Paulista; Hospital Universitário São Francisco de Assis na Providência de Deus); M Capuzzo Gonçalves (Goiânia; University Federal Hospital); A Vieira Barros (Maceio; Hospital Santa Casa de Misericordia de Maceio); R Pedrini Cruz (Porto Alegre; Hospital Nossa Senhora da Conceição); A Cunha Viana Júnior (Rio de Janeiro; Hospital Naval Marcílio Dias); FR Takeda (Sao Paulo; Hospital das Clinicas da Faculdade de Medicina da Universidade de São Paulo); P R Oliveira (Sao Paulo; Instituto de Ortopedia e Traumatologia do Hospital das Clinicas da Faculdade de Medicina da Universidade de Sao Paulo); U Ribeiro Junior (Sao Paulo; Instituto do Cancer do Estado de São Paulo); G Baiocchi, LP Kowalski, SDC Zequi (São Paulo; A.C. Camargo Cancer Center); R Flumignan (São Paulo; Hospital São Paulo); RL Nunes (São Paulo; Notre Dame Intermédica - Hospital Salvalus); A Gatti (Taboão da Serra; Hospital Geral de Pirajussara); PHDS Fernandes (Uberlandia; Federal University of Uberlandia). |
| Bulgaria: T Ivanov (Pleven; Heart and Brain - Pleven Hospital); M Karamanliev (Pleven; University Hospital Dr Georgi Stranski, Medical University - Pleven); M Slavchev (Plovdiv; University Hospital Eurohospital); T Yotsov (Ruse; University Hospital Medika); M Sokolov (Sofia; University Hospital Alexandrovska). |
| Cameroon: CG Nwegbu (Bamenda; Mbingo Baptist Hospital). |
| Canada: M Strickland (Edmonton; Royal Alexandra Hospital); K Verhoeff (Edmonton; University of Alberta Hospital); K Bailey (Hamilton; McMaster Children’s Hospital); H Shanthanna (Hamilton; St. Joseph’s Healthcare Hamilton); J Martin (London; London Health Sciences Centre and St Josephs Health Care London); EG Wong (Montreal; Jewish General Hospital); G Groot (Saskatoon SK; Saskatoon City Hospital/Royal University Hospital/St. Paul’s Hospital); S Lee (Vancouver; Royal Columbian Hospital); K Mayson (Vancouver; Vancouver General Hospital). |
| Chile: CA Mazuret Sepulveda (Santiago; Hospital Sótero de Río). |
| Colombia: NF Pedraza Alonso (Bogota; Colombiana de Trasplantes); PA Cabrera Rivera (Bogota; Fundacion Cardioinfantil-IC); J Rodriguez (Bogota; Fundacion Santa Fe de Bogota); DF Salcedo Miranda (Bogota; Hospital Simon Bolivar); LM Trujillo (Bogota; Instituto Nacional de Cancerologia); AJ Nieto Calvache (Cali; Fundación Valle del Lili); FJ Bonilla-Escobar (Cali; Hospital Universitario del Valle Evaristo García); CM Orozco-Chamorro (Popayan; Clinica La Estancia); EA Benavides Hernández (Popayan; Hospital Susana Lopez de Valencia); JA Calvache (Popayán; Hospital Universitario San José). |
| Croatia: G Šantak (Pozega; County General Hospital Pozega); J Mihanovic (Zadar; Zadar General Hospital); T Kopjar (Zagreb; University Hospital Centre Zagreb); I Luksic (Zagreb; University Hospital Dubrava). |
| Cuba: T Del Toro Simoni (Camagüey; Hospital Universitario Manuel Ascunce Domenech); O Suárez Batista (Santiago de Cuba; Hospital Dr. Ambrosio Grillo Portuondo). |
| Czech Republic: P Ihnát (Ostrava; University Hospital Ostrava). |
| Denmark: C Meyhoff (Copenhagen; Bispebjerg Hospital). |
| Dominican Republic: D Mejia De la Cruz (Santo Domingo; Hospital General Plaza de la Salud). |
| Ecuador: VD Alarcón Vela (Ambato; Hospital General Docente Ambato); E Ochoa Maldonado (Guayaquil; Hospital de Especialidades Teodoro Maldonado Carbo); SC Gómez López (Ibarra; Hospital San Vicente de Paul); N Campuzano (Quito; AXXIS Hospital Quito Ecuador); CA Mena García (Quito; Hospital General Docente de Calderón); MJ Paspuel Villacís (Quito; Hospital General Enrique Garcés); A Ayala Ochoa (Quito; Hospital General IESS Quito Sur); A Barreto Grimaldos (Quito; Hospital Metropolitano); NM Alegria Navarrete (Quito; Hospital Pablo Arturo Suarez); J Arboleda (Quito; Hospital Pediatrico Baca Ortiz); EJ Paredes Alvarez (Quito; Hospital San Francisco de Quito (IESS)); EP Lincango (Quito; Hospital Vozandes Quito); R Buenaño González (Quito; Hospital de Especialidades Eugenio Espejo); D Silva Segovia (Quito; Hospital militar (Hospital de Especialidades de FF.AA N°1)); L Fuenmayor-González (Quito; Novaclínica Santa Cecilia). |
| Egypt: MM Saad (Assiut; Assiut University Hospital); B Mostafa (Cairo; Ain Shams University Specialized Hospital); M Abdel-Maboud (Cairo; EL-Hussein University Hospital, Al-Azhar University, Faculty Of Medicine); AK Awad (Cairo; El Demerdash University Hospital); M El-Kassas (Cairo; Endemic Medicine Department, Helwan University); M ElFiky (Cairo; Kasr Al Ainy Faculty of Medicine, Cairo University); AY Azzam (Damietta; Damietta Specialized Hospital); A Sallam (Ismailia; Suez Canal University Hospital); A Shehta (Mansoura; Gastrointestinal surgery center); H Elfeki (Mansoura; Mansoura University Hospital); M Abdelkhalek (Mansoura; Oncology Center Mansoura University); M Omar (Qena; Qena University Hospital); M Elbahnasawy (Tanta; Tanta University Hospital); S Ghozy (giza; sheikh zayed specialized hospital). |
| Ethiopia: A Negussie (Addis Ababa; ALERT center); K Shumbash (Addis Ababa; Lancet Specialized Hospital); M Abebe (Addis Ababa; Saint Paul Hospital Millennium Medical College); S Hailu (Addis Ababa; Tikur Anbessa Specialized (Black Lion) Hospital); YB Akililu (Addis Ababa; Zewditu Memorial Hospital); B Atnafu (Bahir Dar; Bahir Dar University Tibebe Ghion Specialized Hospital); K Bekele (Goba; Maddawalabu University Goba Referral Hospital); MG Mengesha (Hawassa; Hawassa University Comprehensive Specialized Hospital); HT Derilo (Hossana; Wachemo University Nigist Elleni Mohammed Memorial Referral Hospital); G Mulugeta (Jimma; Jimma University Medical Center). |
| Finland: E Sarjanoja (Kemi; Länsi-Pohja Central Hospital); JH Kauppila (Oulu; Oulu University Hospital). |
| France: F Schmitt (Angers; CHU Angers); L Harper (Bordeaux; CHU Bordeaux); M Danguy des Déserts (Brest; Military Hospital Clermont Tonnerre (Hôpital des Armées)); A Police (Eaubonne; Hôpital Simone Veil); J Veziant (Lille; CHU Lille Hôpital Claude Huriez); E Duchalais (Nantes; CHU Nantes); E Kantor (Paris; AP-HP Hopital Bichat Claude Bernard); C Crétolle (Paris; Hôpital Necker Enfants Malades - APHP); S Gaujoux (Paris; Pitie Salpetriere); M Peycelon (Paris; Robert Debré Children University Hospital - APHP); AP Arnaud (Rennes; CHU Rennes - Hopital Sud); F Nappi (Saint Cenis; Centre Cardiologique du Nord); H Charbonneau (Toulouse; Clinique Pasteur). |
| Georgia: Z Demetrashvili (Tbilisi; N.Kipshidze Central University Clinic). |
| Germany: A Modabber, L Grüßer (Aachen; University Hospital Aachen); S Wolf (Augsburg; University Hospital Augsburg); D Kaemmerer (Bad Berka; Zentralklinik Bad Berka); C Kamphues (Berlin; Charité University Medicine - Campus Benjamin Franklin); P Höhn (Bochum; St. Josef-Hospital); M Velten, M Coburn, TO Vilz (Bonn; University Hospital Bonn); AC Rokohl, C Mallmann, MR Mallmann (Cologne; University Hospital of Cologne); U Bork (Dresden; University Hospital Carl Gustav Carus, Technical University Dresden); C Fung (Freiburg; University Medical Center Freiburg, Faculty of Medicine); C Koch (Giessen; University Hospital Giessen and Marburg); U Ronellenfitsch (Halle; University Hospital Halle); A Heuer (Hamburg; University Medical Center Hamburg-Eppendorf); S Welter (Hemer; Lung Clinic Hemer); S Michling (Kaufbeuren; Klinikum Kaufbeuren (Kliniken Ostallgäu-Kaufbeuren)); A Roth (Leipzig; University Hospital Leipzig); J Lindert (Lübeck; University Hosital Schleswig- Holstein); A Rissmann (Magdeburg; University Hospital Magdeburg); VC Linz (Mainz; University Hospital Mainz); S Seyfried (Mannheim; Mannheim University Medical Center (Universitätsmedizin Mannheim)); J Rolinger (Moenchengladbach; Kliniken Maria Hilf); MG Stoleriu (Munich Gauting; Asklepios Pulmonary Hospital); D Reim, J Gempt (Munich; Klinikum Rechts der Isar TUM School of Medicine); N Börner (Munich; Ludwig Maximilian University of Munich - Großhadern); AM Keppler (Munich; Ludwig Maximilian University of Munich - Innenstadt); L Schröder (Offenbach; Ketteler Krankenhaus); K Pfister (Regensburg; University Hospital Regensburg); J Herzberg (Reinbek; Krankenhaus Reinbek St. Adolf-Stift); F Gessler (Rostock; University Hospital Rostock); GA Stavrou (Saarbruecken; Klinikum Saarbruecken); M Quante (Tuebingen; University Hospital Tuebingen); C Konrads (Tübingen; BG Klinik); J Doerner (Wuppertal; Helios Universitätsklinikum Wuppertal (Universität Witten/Herdecke)). |
| Ghana: MT Morna (Cape-Coast; Cape Coast Teaching Hospital); EA Nachelleh (Ho; Ho Teaching Hospital); S Agyeiwaa Owusu (Tamale; Tamale Teaching Hospital). |
| Greece: G Micha (Athens; ‘Elena Venizelou’ General and Maternity hospital of Athens); E Fradelos (Athens; Agios Savvas Anticancer Hospital); D Haidopoulos (Athens; Alexandra General Hospital); N Memos (Athens; Aretaieion Hospital); A Ioannidis (Athens; Athens Medical Center); C Barkolias (Athens; Athens Naval and Veterans Hospital); N Michalopoulos (Athens; Attikon University General Hospital); KI Paraskevas (Athens; Central Clinic of Athens); M Sotiropoulou (Athens; Evaggelismos General Hospital); A Paspala (Athens; Evgenideio Hospital); K Pateas (Athens; G. Gennimatas Hospital); E Kyrodimos (Athens; Hippocratio General Hospital); EC Tampaki (Athens; KAT Athens General Hospital); K Roditis (Athens; Korgialenio-Benakio Hellenic Red Cross Hospital); D Schizas (Athens; Laiko University Hospital); K Apostolou (Athens; Mediterraneo Hospital); L Tzelves (Athens; Sismanoglio - Amalia Fleming General Hospital); M Spartalis (Athens; Sotiria General Hospital of Thoracic Diseases); S Xenaki (Heraklion Crete; University Hospital of Heraklion Crete); N Gougoulias (Katerini; General Hospital of Katerini); E Lostoridis (Kavala; Kavala General Hospital); G Koukoulis (Larissa; General Hospital of Larissa ‘Koutlimpaneio and Triantafylleio’); E Arnaoutoglou, MP Ntalouka (Larrisa; General University Hospital of Larissa); A Dimas (Lefkada; General Hospital of Lefkada); F Mulita (Patras; General University Hospital of Patras); V Mousafeiris (Patras; Karamandaneio Prefecture Children Hospital of Patras); A Papadopoulos (Piraeus; General Hospital of Nikaia); I Katsaros (Piraeus; Metaxa Cancer Hospital); N Zampitis (Piraeus; Tzaneio General Hospital); V Vrangalas (Thessaloniki; 424 General Military Hospital); I Valioulis (Thessaloniki; G. Gennimatas Thessaloniki General Hospital); O Ioannidis (Thessaloniki; George Papanikolaou General Hospital of Thessaloniki); T Dagklis (Thessaloniki; Hippocratio Hospital); C Anthoulakis, E Tsiridis, V Papadopoulos (Thessaloniki; Papageorgiou General Hospital); D Lytras (Volos; Achillopoyleio General Hospital of Volos). |
| Guatemala: G Recinos (Guatemala City; Hospital De Accidentes Ceibal); M Aguilera-Arevalo (Guatemala City; Hospital General San Juan De Dios); E Brolo (Guatemala; Hospital Universitario Esperanza). |
| Hong Kong SAR, China: K Futaba (Sha Tin; Prince of Wales Hospital). |
| Hungary: P Sotonyi (Budapest; Semmelweis University (please use for all units)). |
| India: P Behera (BHOPAL; All India Institute of Medical Sciences Bhopal); I Madabhavi (Bagalkot; Kerudi Cancer Hospital); PA Shah (Bangalore; Cyte Care Cancer Hospital); S Kumar Venkatappa (Bangalore; Victoria Hospital); S Chaudhary (Baroda; Government Medical College and SSG Hospital); N Mishra (Bhopal; Gandhi Medical College and Sultania Zanana Hospital); A Gupta (Chandigarh; Government medical college hospital); M Karthigeyan, S Mohindra (Chandigarh; Postgraduate Institute of Medical Education & Research, Chandigarh, India); A Rammohan (Chennai; Dr.Rela Institute & Medical Centre); A Rajanbabu (Cochin; Amrita Institute of Medical Sciences Hospital); P Kaul (Dehradun, Uttarakhand; Shri Guru Ram Rai Institute of Medical and Health Sciences); S Muthu (Dindigul, Tamil Nadu; Government Medical College and Hospital, Dindigul); DAS Rai (Gurugram, Haryana; Paras Hospital); M Raut (Gurugram; Artemis Health Institute); S Misra (Jodhpur; All India Institute of Medical Sciences (AIIMS), Jodhpur); A Sharma (Kolkata; Tata Medical Center); S Rajan (Lucknow; King George’s Medical University); P Pawar (Ludhiana; Christian Medical College & Hospital); S Lasrado (Mangalore, Karnataka; Father Muller Medical College); J Akbar (Mangalore; Yenepoya medical college hospital); B L, R P Shenoy (Manipal; Kasturba Medical College Hospital, Manipal); CS Pramesh (Mumbai; Tata Memorial Hospital); S Kale (NAVI MUMBAI; D Y Patil Hospital); S Sharma, SK Dube (New Delhi; All India Institute of Medical Sciences); HS Chhabra (New Delhi; Indian Spinal Injuries Center); L Bains (New Delhi; Maulana Azad Medical College); S Pankaj (Patna, Bihar; Indira Gandhi Institute of Medical Sciences); S Manglik (Pune, Maharashtra; Bharati Vidyapeeth); V Sodhai (Pune; KEM Hospital and Research Center); F Huda, S Basu (Rishikesh; All India Institute Of Medical Sciences); C Mahakalkar (Sawangi (Meghe), Wardha; Acharya Vinoba Bhave Rural Hospital); M Babu (Sullia; KVG Medical College & Hospital); A Phadnis (thane; jupiter hospital). |
| Indonesia: FA Damara (Bandung; Dr. Hasan Sadikin Central General Hospital); AK Harzif (Jakarta; Cipto Mangunkusumo National General Hospital & Universitas Indonesia); SL Anwar (Yogyakarta; Central General Hospital dr. Sardjito). |
| Iran: M Fadavipour (Abadan; Valiasr Educational Hospital). |
| Iran, Islamic Rep.: A Salimi asl (Bandar Abbas; Shahid Mohammadi Hospital); E Sabouri (Esfahan; Shariati Hospital); E Mohammadbeigi (Isfahan; Al Zahra Hospital); M Pourfridoni (Jiroft; Imam Khomeini Hospital); S Ahmadi (Kashmar; Hazrat-e Abolfazl Hospital); A Bahreyni (Kerman; Bahonar Hospital); SS Ghiasi (Mashhad; Ghaem Teaching Hospital); M Rahimi (Mashhad; Imam Reza hospital); H Yusefi (Qom; Shahid Beheshti Hospital); S Ramezani (Rasht; Poursina Hospital); A Soleymanitabar (Tehran; Baqiyatallah Hospital); M Mozafari (Tehran; Firoozabadi Hospital); MR Hosseini Siyanaki, R Tofighi (Tehran; Firoozgar General Hospital); F Esmaeili Tarki (Tehran; Imam Hossein Hospital); S Azadnajafabad (Tehran; Imam Khomeini Hospital Complex(IKHC)); S Ahmadi (Tehran; Rasool-e-Akram Hospital); F Sadat Rahimi (Tehran; Shahid Labbafinejad Hospital); S Afaghi (Tehran; Shahid Modarres Hospital); F Vosoughi (Tehran; Shariati Hospital); ZS Aghamir (Tehran; Sina Hospital); M Anjomrooz (Tehran; Ziaeian Hospital); M Safari (Urmia; Imam Khomeini Hospital); T Karami (Urmia; Mahzad Women’s Medical Center); K Kazemi Esfe (Yazd; Shahid Sadoughi Hospital); M Ghaemi (Zanjan; Ayatollah Mousavi Hospital). |
| Iraq: MA Ramadhan (Baghdad; Baghdad Medical City); AKA Karantenachy (Baghdad; Zafaraniyah General Hospital); H Aldawoody (Khanaqin; Khanaqin general hospital); MA Al-Juaifari (Najaf; Al-Najaf Al-Ashraf Teaching Hospital); AT Hashim (Nassiryah; Al-Hussein Teaching hospital); A Kareem Hama Ghareeb (Sulemaniyah; Shorsh teaching hospital). |
| Ireland: R Tummon (Cork; Cork University Hospital); S O’Brien (Cork; Mercy University Hospital); D James (Cork; South Infirmary Victoria University Hospital); A Dhannoon (Dublin; Beaumont Hospital); E Burke (Dublin; Connolly Hospital Blanchardstown); F Salameh (Dublin; Rotunda Hospital); M Kelly (Dublin; St James’s Hospital); IS Reynolds (Dublin; St Vincent’s University Hospital); É Ryan (Dublin; Tallaght Hospital); MG Davey (Galway; University Hospital Galway); A O’driscoll-collins (Kerry; University Hospital Kerry); J Pretorius (Letterkenny; LETTERKENNY UNIVERSITY HOSPITAL); B Creavin (Waterford; University Hospital Waterford/University College Cork). |
| Israel: E Quint (Beer-Sheva; Soroka University Medical Center); G Marom (Jerusalem; Hadassah Medical Center); O Nahtomi Shick (Jerusalem; Shaare Zedek Medical Center); E Bekhor (Petah Tikva; Hasharon Hospital); Y Zager (Ramat Gan; Sheba Medical Center). |
| Italy: G Consorti (Ancona; Ospedali Riuniti di Ancona); M Clementi (Aquila; San Salvatore Hospital, University of L’Aquila); GM Pirola (Arezzo; Ospedale San Donato USL Toscana Sud Est); A Lauretta (Aviano; Centro di Riferimento Oncologico di Aviano (CRO) IRCCS); A Picciariello (Bari; Azienda Ospedaliero Universitaria Consorziale Policlinico Di Bari); M Rottoli (Bologna; IRCCS Azienda Ospedaliero-Universitaria di Bologna); R Aspide (Bologna; IRCCS Istituto delle Scienze Neurologiche di Bologna); GM Prucher (Bologna; Ospedale Maggiore/Bellaria Carlo Alberto Pizzardi AUSL Bologna); D Raimondo (Bologna; Ospedale Villa Laura); G Armatura (Bolzano; Bolzano Central Hospital); J Andreuccetti (Brescia; ASST Spedali Civili, Ospedale di Brescia); M Podda (Cagliari; Cagliari University Hospital); N Cillara (Cagliari; Santissima Trinità - ATS Sardegna); A Rocca (Campobasso; Antonio Cardarelli); F Marino (Castellana Grotte (Ba);IRCCS ‘Saverio de Bellis’); M Veroux (Catania; Azienda Ospedaliero- Universitaria Policlinico San Marco); G Giannaccare (Catanzaro; University ‘Magna Graecia’ of Catanzaro); N Zanini (Cesena; Ospedale M. Bufalini); M Barone (Chieti; Policlinico Santissima Annunziata); A Balla (Civitavecchia; San Paolo Hospital); A Romanzi (Como; Valduce Hospital); B Nardo (Cosenza; Azienda Ospedaliera di Cosenza); D Sasia (Cuneo; Santa Croce e Carle Hospital, Cuneo); S Zonta (Domodossola; San Biagio Hospital, Domodossola - VB); P Carcoforo (Ferrara; Azienda Ospedaliero-Universitaria Di Ferrara); CV Feo (Ferrara; Azienda Unità Sanitaria Locale di Ferrara); G Maggiore, A Bottari (Firenze; Azienda Ospedaliera Universitaria Careggi); G Canonico (Firenze; Ospedale San Giovanni di Dio); N Tartaglia (Foggia; Ospedali Riuniti Azienda Ospedaliera Universitaria); F D’acapito (Forlì; Morgagni-Pierantoni); A Barberis (Genoa; E.O. Ospedali Galliera); S Scabini (Genoa; IRCCS Ospedale Policlinico San Martino); A Luzzi (Genoa; Ospedale Villa Scassi); N Depalma (Lecce; P.O.’Vito Fazzi’); M Spalluto (Legnano; Ospedale di Legnano); M Caffo (Messina; Policlinico Universitario G. Martino of Messina); SPB Cioffi (Milan; ASST Grande Ospedale Metropolitano Niguarda); NM Mariani (Milan; ASST Santi Paolo e Carlo); A Spinelli (Milan; Humanitas Research Hospital); A Aiolfi (Milan; Istituto Clinico Sant’Ambrogio); L Bertolaccini (Milan; Istituto Europeo di Oncologia - IRCCS -Milano); F Colombo (Milan; Ospedale Luigi Sacco Milano); F Ferrara (Milan; San Carlo Borromeo); P De Nardi (Milan; Università Vita-Salute San Raffaele); M Fiore (Milano; Fondazione IRCCS Istituto Nazionale dei Tumori, Milano); F Fusini (Mondovì; Regina Montis Regalis Hospital, Mondovì); LC Nespoli (Monza; Fondazione IRCCS San Gerardo dei Tintori Monza, Scuola di Medicina e Chirurgia, Università Milano Bicocca); M Manigrasso (Naples; Federico II University of Naples); A Belli (Naples; Istituto Nazionale Tumori Fondazione, Pascale IRCCS); S Gili (Naples; Ospedale S. Leonardo - ASL Napoli 3 sud, Castellammare di Stabia); A Tufo (Naples; ospedale del mare); P Campennì (Olbia; Mater Olbia Hospital); A Chessa (Orbetello; San Giovanni di Dio); F Toia (Palermo; Department of Surgical, Oncological and Oral Sciences. University of Palermo); L Licari (Palermo; FBF Buccheri La Ferla Palermo); L Cobianchi (Pavia; Policlinico San Matteo); L Conti (Piacenza; G. Da Saliceto); R Galleano (Pietra Ligure; Ospedale Santa Corona, Pietra Ligure (SV)); M Calabrò (Pinerolo; Edoardo Agnelli); L Andreani (Pisa; Azienda Ospedaliero Universitaria Pisana); E Pinotti (Ponte San Pietro; Policlinico San Pietro); L Petagna (Potenza; Azienda Ospedaliera Regionale ‘San Carlo’); VD Mandato (Reggio Emilia; Azienda Unità Sanitaria Locale - IRCCS di Reggio Emilia); C Marafante (Rivoli; Ospedale degli Infermi di Rivoli); V De Simone (Rome; Fondazione Policlinico Universitario Agostino Gemelli); F Mazzola (Rome; IRCCS ‘Regina Elena’ National Cancer Institute); R Angelico (Rome; Policlinico Tor Vergata Hospital, Rome); P Lapolla (Rome; Policlinico Umberto I Sapienza University of Rome); S Fiorelli (Rome; Sant’Andrea Hospital, Sapienza University of Rome); G Lisi (Rome; Sant’Eugenio Hospital); D Bernardi (San Donato; IRCCS Policlinico San Donato); A Porcu (Sassari; Cliniche San Pietro, A.O.U. Sassari); L Marano (Siena; Azienda Ospedaliero Universitaria Senese); V Tonini (Taranto; Santa Annunziata Hospital); A Brolese (Trento; Santa Chiara Hospital); U Grossi (Treviso; Ca’ Foncello); S Trungu (Tricase; Cardinale G Panico Hospital); R Tutino (Turin; Città della Salute e della Scienza); M Berselli (Varese Lombardy; University of Insubria, Ospedale di Circolo e Fondazione Macchi (Varese)); AS Laganà (Varese; Filippo Del Ponte Hospital, University of Insubria); F Lemma (Venezia; Dell’Angelo Hospital); S Zonta (Verbania; Castelli); T Campagnaro, A Antonelli (Verona; Azienda Ospedaliera Universitaria Integrata di Verona); M Inama (Verona; Ospedale Pederzoli); A Frontali (Vimercate; ASST Brianza - Ospedale di Vimercate); P Cianci (andria; Lorenzo Bonomo); M Abu Hilal (brescia; Fondazione Poliambulanza); R Papalia (rome; policlinico universitario campus bio medico of rome); G Vizzielli (udine; santa maria della misericordia). |
| Japan: H Yonekura (Aichi; Fujita Health University Bantane Hospital); T Chaki (Hokkaido; Sapporo Medical University Hospital); Y Okazawa (Hyogo; Hyogo Prefectural Amagasaki General Medical Center); T Namikawa (Kochi; Kochi Medical School Hospital); M Ida (Nara; Nara Medical University); A Kuriyama (Okayama; Kurashiki Central Hospital); D Hashimoto (Osaka; Kansai Medical University); Y Fujimoto (Saitama City; Saitama Children’s Medical Center); T Kato (Saitama; Saitama Medical Center, Saitama Medical University); T Kochiyama (Tokyo; Juntendo University Hospital); M Iwasaki (Tokyo; Nippon Medical School Hospital). |
| Jordan: S Alarood (Alkarak; Al Karak Hospital); A Qasem (Amman; Al-Basheer Hospital); M Alqedrh (Amman; Al-Essra Hospital); R Soudi (Amman; Eye Speciality Hospital); H Hussein (Amman; Ibn Al Haitham Hospital); B Alrayes (Amman; Islamic Hospital); L Sawalha (Amman; Jordan Hospital); A Abu salhiyeh (Amman; Jordan Red Cresent Hospital); Y Alawneh (Amman; Prince Hamza hospital); A Aladaileh (Aqaba; Islamic Hospital - Aqaba); T Sawadi (Ar Ramtha; King Abdullah University Hospital/ Jordan University of Science and Technology); Y Al Zu’bi (Irbid; Badea Governmental Hospital); A Khamees (Irbid; Irbid Specialty Hospital); AAM Fadhel (Irbid; Princess Basma Hospital); Y Al Zu’bi (Irbid; Yarmouk Governmental Hospital); M Alhawatmeh (Madaba; Al Nadeem Hospital);  M Ababneh (Zarqa;  New Zarqa Governmental Hospital). |
| Kuwait: R Zakaria (Kuwait City; Al Amiri Hospital). |
| Lebanon: N Bazzi (Beirut; Al Zahraa Hospital University Medical Center); F Fatouh (Beirut; Rafik Hariri University Hospital). |
| Libya: K Tamoos (Alabyar; Alabyar General Hospital); M Fathi Al Gharyani (Benghazi; Al Hawari General Hospital); G Alkadeeki (Benghazi; Al-jalaa Teaching/Trauma Hospital); W Aldressi (Benghazi; Benghazi Medical Center); A Alkaseek (Gharyan; Gharyan Central Hospital); G Yagoub (Misurata; Alhikma hospital); A Benamwor (Misurata; Misurata Cancer Center); AAY Almugaddami (Nalut; Nalut Central Hospital); M Bilfaqirah (Sebha; Al-Majd Clinic); M Abdelkabir (Sebha; Sabha Medical Center); N Abdelrahim (Sirte; Ibn Sina Teaching Hospital); R Berbash (Tajora-Tripoli; Yashfeen Clinic); RAI Ben jouira (Tajoura; National Heart Centre, Tajoura Heart Center Hospital); A Meelad (Tarhuna; Tarhuna General Hospital); H Alameen (Tripoli; Al-Abraj Hospital); K Ayad (Tripoli; Alkhalil hospital); A Elhadi (Tripoli; Karat Al-Ain Hospital); FAD Elhajdawe (Tripoli; Metiga Hospital); E Abdulwahed (Tripoli; Tripoli Central Hospital); MFK Abu hallalah (Tripoli; Tripoli Medical Center/ Tripoli University Hospital); IA Saleh (Zintan; Taqwa Clinic); N Ben Hasan (Zliten; Al Asmarya Islamic University, Zliten Medical Center). |
| Lithuania: D Venskutonis (Kaunas; LUHS Kaunas Hospital); A Dauksa (Kaunas; Lithuanian University of Health Sciences Kaunas Clinics); A Dulskas (Vilnius; National Cancer Institute); A Gulla (Vilnius; Vilnius University Hospital Santaros Klinikos). |
| Malaysia: AD Zakaria (Kelantan; Hospital Universiti Sains Malaysia); R Ramli (Kuala Lumpur; Universiti Kebangsaan Malaysia Medical Centre); AC Roslani (Kuala Lumpur; University Malaya Medical Centre); YJ Ng We Yong (Kuching, Sarawak; Sarawak General Hospital). |
| Mexico: EE Sosa Duran (Ciudad de México; Hospital Juárez de México); G Yanowsky-Reyes (Guadalajara; Antiguo Hospital Civil de Guadalajara); A Gonzalez Ojeda (Guadalajara; Hospital de Especialidades, CMNO-IMSS); J Beristain-Hernandez (Mexico City; Centro Médico Nacional La Raza); MA Mercado (Mexico City; Instituto Nacional de Ciencias Médicas y Nutrición ‘Salvador Zubirán’); F Bolanos-Morales (Mexico; Instituto Nacional de Enfermedades Respiratorias); A Navarrete-Peón (Pachuca; Sociedad Española de Beneficencia); M Noguez Castillo (Querétaro; Hospital de especialidades del niño y la mujer); CE Aguilar Alvarado (Tamaulipas; Beneficencia Española de Tampico); A Ramos-De la Medina (Veracruz; Hospital Español Veracruz). |
| Morocco: A Bourial (Casablanca; Mohammed VI International University Hospital); S Benamar (Rabat; Cheikh Zaid International University Hospital); A Elkoundi (Rabat; Hôpital Militaire Mohammed V); L Boualila (Rabat; Hôpital des Spécialités - ONO); A Souadka (Rabat; Institut National d’Oncologie). |
| Namibia: PR Nashidengo (Windhoek; Windhoek Central Academic Hospital). |
| Nepal: R Shrestha (Bhaktapur; Shahid Dharmabhakta National Transplant Centre); S Bhusal (Lalitpur; KIST Medical College and Teaching Hospital). |
| Netherlands: KR Wienholts, R Bakx (Amsterdam; Amsterdam UMC, University of Amsterdam); W Kelder (Groningen; Martini General Hospital Groningen); JF Lin (Groningen; University Medical Center Groningen); M Emous (Leeuwarden; Medisch Centrum Leeuwarden); E Boerma (Sittard/Heerlen; Zuyderland Medical Centre); J Konsten (Venlo; VieCuri Medisch Centrum). |
| New Zealand: A Lin (Wellington; Wellington Regional Hospital). |
| Nigeria: A Akinmade (Ado Ekiti; Afe Babalola University Multi-System Hospital); KJ Bwala (Bauchi; Abubakar Tafawa Balewa University Teaching Hospital Bauchi); P Agbonrofo (Benin City; University of Benin Teaching Hospital); S Olori (Gwagwalada; University of Abuja Teaching Hospital); TA Lawal (Ibadan; University College Hospital); II Aremu (Ilorin; General Hospital); E Morgan (Lagos; Awesome Grace Hospital); I Chukwu (Umuahia; Federal Medical Centre Umuahia). |
| North Macedonia: L Todorovic (Skopje; University Clinic for Pediatric Surgery); I Peev, S Pejkova (Skopje; University Clinic for Plastic and Reconstructive surgery, Faculty of Medicine, University St. Cyril and Mthodius). |
| Oman: J Massoud (Muscat; Khoula Hospital); S Sheik (Muscat; Royal Oman Police Hospital); S Kodange (Muscat; Sultan Qaboos Comprehensive Cancer Centre); F Ali (Muscat; Sultan Qaboos University Hospital). |
| Pakistan: T Khaliq (Islamabad; Kulsum International Hospital); U Saeed (Islamabad; South East Hospital); SH Waqar (Islamabad; The Pakistan Institute of Medical Sciences); MK Khan (Jamshoro; Liaquat University of Medical & Health Sciences); A Aziz (Karachi; Aga Khan University); S Saeed (Karachi; Dow University Hospital); L Rai (Karachi; Dr Ruth K.M. Pfau Civil Hospital); MTJ Khan (Karachi; Jinnah Medical College Hospital); A Ali (Karachi; PAF Faisal Hospital); F Ashraf (Karachi; Patel Hospital); AS Ammar (Lahore; Bahria International Hospital, Bahria Orchard); MZ Sarwar (Lahore; King Edward Medical University - Mayo Hospital); K Hayat (Lahore; Services Hospital Lahore); N Talat (Lahore; The Children’s Hospital & The Institute of Child Health Lahore); S Ahmad (Peshawar; Hayatabad Medical Complex); E Yaqoob (Rawalpindi; Benazir Bhutto Hospital); S Javed (Rawalpindi; Holy Family Hospital). |
| Paraguay: H Gomez-Fernandez (Asuncion; Centro Médico La Costa); HA Segovia Lohse (Lambaré; Hospital Distrital de Lambaré, Paraguay); R Sánchez (San Lorenzo; Hospital De Clínicas). |
| Peru: GC Manrique Sila (Arequipa; Honorio Delgado Espinoza Regional Hospital); GM Falcon Pacheco (Arequipa; Instituto Regional de Enfermedades Neoplásicas del Sur); R Mas Melendez (Ayacucho; Ayacucho Regional Hospital); JA Collantes Cubas (Cajamarca; Hospital Regional Docente de Cajamarca); J Rios Chiuyari (Lambayeque; Hospital regional de lambayeque); G Borda-Luque (Lima; Cayetano Heredia National Hospital); G Mendiola (Lima; Hospital Santa Rosa de Lima); F Cárdenas Escalante (Lima; Instituto Nacional de Enfermedades Neoplásicas); J Caballero-Alvarado (Trujillo; Hospital Regional Docente de Trujillo). |
| Philippines: EJ Castro (Bayombong; Region 2 Trauma and Medical Center); IM Lim (Manila; José R. Reyes Memorial Medical Center); MP Lopez (Manila; Ospital ng Makati); MC Lapitan (Manila; Philippine General Hospital, University Of The Philippines Manila); JAS Reyes (Pasig; The Medical City); KM Montejo (Quezon City; Victoriano Luna General Hospital). |
| Poland: PT Stogowski (Gdansk; Szpital im. M. Kopernika / Copernicus Hospital); T Stefaniak (Gdańsk; Uniwersyteckie Centrum Kliniczne); Ł Nawacki (Kielce; Wojewódzki Szpital Zespolony w Kielcach); K Kułak (Lublin; Independent Public Teaching Hospital No 1 in Lublin); K Szyluk (Piekary Śląskie; District Hospital of Orthopedics and Trauma Surgery); W Krawczyk (Sosnowiec; Wojewódzki Szpiital Specjalistyczny nr 5 im. Św Barbary); M Walędziak (Warsaw; Military Institute Of Medicine); D Borselle (Wrocław; Uniwersytecki Szpital Kliniczny we Wrocławiu). |
| Portugal: AL Preto Barreira (Almada; Hospital Garcia de Orta); D Chalo (Aveiro; Centro Hospitalar do Baixo Vouga); R Pedroso de Lima (Evora; Hospital do Espirito Santo); S Boligo (Lisbon; Hospital Sao Francisco Xavier); B Silva Mendes (Portimao; Centro Hospitalar Universitario do Algarve - Unidade de Portimão); C Granja (Porto; Centro Hospitalar e Universitário de São João); M Marques (Porto; IPO Porto); D Martins (Vila Real; Centro Hospitalar de Trás-os-Montes e Alto Douro, E.P.E.). |
| Romania: M Hogea (Brasov; Brasov Emergency Clinical County Hospital); I Balescu (Bucharest; ‘Dr. Ion Cantacuzino’ Clinical Hospital); F Grama (Bucharest; Coltea Clinical Hospital); EA Toma (Bucharest; Elias Emergency Hospital); I Negoi (Bucharest; Emergency Clinical Hospital Bucharest); C Predoi (Bucharest; Emergency Institute for Cardiovascular Diseases ‘Prof. Dr. C.C. Iliescu’); N Bacalbasa (Bucharest; Fundeni Clinical Institute); N Motas (Bucharest; Institute of Oncology Prof Dr Al Trestioreanu); NS Ionescu (Bucharest; Maria Sklodowska Curie Emergency Hospital); O Ginghina (Bucharest; Saint John Emergency Hospital); NO Zarnescu (Bucharest; University Emergency Hospital Bucharest); R Drasovean (Cluj-Napoca; Cluj-Napoca Emergency County Hospital); A Pasca, E Bonci (Cluj-Napoca; Prof Dr Ion Chiricuta Institute of Oncology); S Mogoanta (Craiova; Spitalul Judetean De Urgenta Din Craiova); M Pertea (Iasi; ‘Sf. Spiridon’ Emergency Clinical Hospital); M Dimofte (Iasi; Regional institute of Oncology Iasi). |
| Russian Federation: A Litvin (Kaliningrad; Immanuel Kant Baltic Federal University, Regional Clinical Hospital); S Efetov (Moscow; IM Sechenov First Moscow State Medical University); V Subbotin (Moscow; Moscow Clinical Scientific Center named after AS Loginov); A Bedzhanyan (Moscow; Petrovsky National Research Centre of Surgery); K Kadantseva (Moscow; V. Negovskiy Reanimatology Research Institute); A Yanishev (Nizhny Novgorod; Privolzhsky Research Medical University); S Efremov (Saint Petersburg; Saint Petersburg State University Hospital); A Butyrskii (Simferopol; Municipal Emegency Hospital No.6); E Drozdov (Tomsk; Tomsk regional oncology hospital); V Ten (Yuzhno-Sakhalinsk; Private healthcare institution ‘RZD-Medicine’). |
| Rwanda: A Costas-Chavarri (Kigali; Rwanda Military Hospital); M Ruhosha (Muhanga district; Kabgayi Hospital). |
| Saudi Arabia: N Alzerwi (Al-Majmaah; King Khalid General Hospital); M Basendowah (Jeddah; King Abdulaziz University Hospital); A Althumairi (Jeddah; King Khalid National Guard Hospital); M Alyami (Najran; King Khalid Hospital); A Althumairi (Riyadh; King Abdulaziz Medical City); A Alhefdhi (Riyadh; King Faisal Specialist Hospital); S Chowdhury (Riyadh; King Saud Medical City); T Nouh (Riyadh; King Saud University); R Khan (Tabuk; King Fahad Specialist Hospital); DY Alalawi (Tabuk; King Salman Armed Forces Hospital). |
| Serbia: V Zivaljevic (Belgrade; Centre for endocrine surgery, University Clinical Centre of Serbia); A Antic, D Knezevic (Belgrade; Clinic for Digestive surgery, University Clinical Center of Serbia, University of Belgrade, Faculty of Medicine); L Srbinovic (Belgrade; Clinic for Gynecology and Obstetrics Narodni Front); A Stefanovic (Belgrade; Clinic for Gynecology and Obstetrics, University Clinical Center of Serbia); D Jelovac (Belgrade; Clinic for Maxillofacial Surgery, School of Dental Medicine, University of Belgrade); A Jotic (Belgrade; Clinic for Otorhinolaryngology and Maxillofacial Surgery, University Clinical Center of Serbia); U Babic, U Bumbasirevic (Belgrade; Clinic of Urology, University Clinical Center of Serbia); D Stevanovic (Belgrade; Clnical hospital centre of Zemun); V Djukic (Belgrade; KBC Dr Dragisa Misovic-Dedinje); S Ducic (Belgrade; University Children’s Hospital); B Toskovic (Belgrade; University Hospital Medical Center Bezanijska Kosa); A Karamarkovic (Belgrade; Zvezdara University Medical Center); A Cvetkovic (Kragujevac; University Clinical Center of Kragujevac); D Zivkovic (Novi Sad; Institute for Child and youth Health Care of Vojvodina); M Protic (Sremska Kamenica, Novi Sad; Oncology Institute of Vojvodina, University of Novi Sad - Faculty of Medicine). |
| Singapore: JX Hing (Singapore; Changi General Hospital); DM Dimatatac (Singapore; KK Women’s and Children’s Hospital); KM Chue (Singapore; Sengkang General Hospital); W Ang (Singapore; Tan Tock Seng Hospital). |
| Slovak Republic: A Panyko, K Šimko (Bratislava; University Hospital Bratislava). |
| Slovenia: JA Košir (Ljubljana; University Medical Centre); D Bratus (Maribor; University Medical Centre). |
| South Africa: C Kloppers (Cape Town; Groote Schuur Hospital); C Makepeace (Cape Town; Victoria Hospital Wynberg). |
| Spain: C Barrena lópez (Albacete; Complejo hospitalario universitario de Albacete); H Aguado López (Albacete; Hellín Hospital); A Rodriguez Infante (Avilés; San Agustín University Hospital); L Ruiz-Villa (BARCELONA; Hospital Universitari Sagrat Cor); M Prieto (Barakaldo; Hospital Universitario Cruces); L Gomez Fernandez (Barcelona; Consorci Sanitari de Terrassa); JM Muñoz Vives (Barcelona; Fundació Althaia - Xarxa Assistencial Universitària de Manresa); A Carreras-Castañer, L Torres Íñiguez, R Pujol Muncunill (Barcelona; Hospital Clinic Barcelona); O Martin Sole (Barcelona; Hospital Sant Joan de Deu); J Nuñez (Barcelona; Hospital Universitario Mutua de Terrassa); M Jimenez Toscano (Barcelona; Hospital del Mar); I Vives (Barcelona; UCSI Pere Virgili); A Gil-Moreno, , J Nuñez (Barcelona; Vall d’Hebron University Hospital); U Garcia de cortazar (Bilbao; Hospital Universitario de Basurto); A Landaluce-Olavarria (Bizkaia; Hospital Urduliz); J De Haro (Brunete, Madrid; Hospital Los Mandronos); JA Gutiérrez Vásquez (Burgos; Hospital Santiago Apostol); V Jimenez (Getafe; Getafe University Hospital); MV Sosa (Gijón; Hospital de Cabueñes); AC Rahy-Martín (Las Palmas de Gran Canaria; Hospital Universitario de Gran Canaria Doctor Negrín); S Marcos Contreras (León; Complejo Asistencial Universitario de León); AM Castaño-Leon (Madrid; 12 de Octubre University Hospital); V Domínguez-Prieto (Madrid; Fundación Jimenez Diaz University Hospital); L Marquez (Madrid; Hospital Central de la Cruz Roja San Jose y Santa Adela); J Dziakova (Madrid; Hospital Clinico San Carlos); O Mateo-Sierra (Madrid; Hospital General Universitario Gregorio Marañón); M Diez Alonso (Madrid; Hospital Universitario Principe de Asturias); P Serrano Méndez (Madrid; Hospital Universitario la Paz); AM Minaya Bravo (Madrid; Hospital del Henares); A Abad-Motos (Madrid; Infanta Leonor University Hospital); JR Gómez López (Medina del Campo (Valladolid);Hospital Medina del Campo); M Carrasco Prats (Murcia; Hospital General Reina Sofía); B Gómez Pérez (Murcia; Hospital Universitario Virgen de la Arrixaca); E Cano-Trigueros (Murcia; Morales Meseguer University Hospital); L Varela Rodríguez (Oviedo; Hospital Universitario Central de Asturias (HUCA)); J Mata (Palma de Mallorca; Hospital Universitario Son Llàtzer); J Trebol (Salamanca; Complejo Asistencial Universitario de Salamanca); A Perez Ferrer (San Sebastian de Los Reyes; Infanta Sofía University Hospital); J Zabaleta (San Sebastian; Hospital Universitario Donostia); R Martín-Láez (Santander; Marqués de Valdecilla University Hospital); JR Oliver Guillen (Soria; Hospital Santa Bárbara); M Vallve-Bernal (Tarragona; Hospital Universitari De Tarragona Joan XXIII); JC Catalá Bauset (Valencia; Consorcio Hospital General Universitario); J Domenech Fernández (Valencia; Hospital Arnau de Vilanova); D Moro-Valdezate (Valencia; Hospital Clínico Universitario de Valencia); JC Bernal-Sprekelsen (Valencia; Hospital Universitario Doctor Peset); B De Andrés-Asenjo (Valladolid; Hospital Clínico Universitario de Valladolid); FJ Tejero-Pintor (Valladolid; Hospital Universitario Río Hortega); A Vazquez Melero (Vitoria-Gasteiz; Hospital Universitario Araba); MD Arribas Del Amo (Zaragoza; Hospital Clinico Universitario Zaragoza); L Sánchez Blasco (Zaragoza; Hospital General de la Defensa); V Duque Mallén (Zaragoza; Hospital Universitario Miguel Servet). |
| Sri Lanka: SPB Thalgaspitiya (Anuradhapura; Teaching Hospital Anuradhapura); T Gamage (Colombo; Lady Ridgeway Hospital for Children); D Wickramasinghe (Colombo; National Hospital of Sri Lanka); V Satchithanantham (Jaffna; Teaching Hospital, Jaffna); M Patabendige (Mahamodara, Galle; Teaching Hospital, Mahamodara, Galle); A Jayawardane (Sri Lanka; De Soysa Hospital for Women); W Wijenayake (Werahera; University Hospital, Kotelawala Defence University). |
| Sudan: S Galal Eldin (Khartoum; Al-Moalem Medical City); I Adel (Khartoum; Bashair Teaching Hospital); N Hilal (Khartoum; Ibn Sina Specialized Hospital); M Ahmed Elamin Elnour (Khartoum; Khartoum Teaching Hospital); AA Adam (Khartoum; Omdurman Teaching Hospital); T Fadalla (Khartoum; Ribat university hospital); A Mohammed alameen (Khartoum; Soba University Hospital); KAD Gasmalla (Wad Madani; University of Gezira Hospital). |
| Sweden: M Jawad (Kristianstad; Central Hospital in Kristianstad); M Chew (Linköping; Linköping University Hospital); A Älgå (Stockholm; South General Hospital); M Rutegård (Umea; Umea University Hospital). |
| Switzerland: T Liebs (BERN; Inselspital, Bern University Hospital, University of Bern); A Tampakis (Basel; University Hospital Basel); F Mongelli (Bellinzona; EOC Ospedale Regionale di Bellinzona e Valli); P Probst (Frauenfeld; Spital Thurgau AG); E Gialamas (Geneva; Geneva University Hospitals); R Galli (Liestal; Kantonsspital Baselland Liestal); M Di Giuseppe (Locarno; EOC Ospedale Regionale di Locarno); A Papadia (Lugano; EOC Ospedale Regionale di Lugano - Civico); J Gass (Luzern; Luzerner Kantonsspital); A Braga (Mendrisio; EOC Ospedale Regionale di Mendrisio); M Sauvain (Neuchatel; Hopital de Pourtales); T Steffen (St. Gallen; Kantonsspital St. Gallen); M Benoit (Visp; Spitalzentrum Oberwallis); M Adamina (Winterthur; Kantonsspital Winterthur); MA Schneider (Zürich; Universitätsspital). |
| Syrian Arab Republic: M Aloulou (Aleppo; Aleppo Private Hospital); AY Arnaout (Aleppo; St Louis Hospital); B Ahmad (Damascus; Al-Mouwasat University Hospital); O Alazki (Latakia; National Hospital); A Hammed (Latakia; Tishreen University Hospital); R Attoum (Tartus; Al Bassel Hospital). |
| Trinidad and Tobago: S Charles (San Fernando; San Fernando General Hospital). |
| Tunisia: B Gafsi (Monastir; University Hospital Fatouma Bourguiba); MA El Ghali (Sousse; Farhat Hatched Hospital); A Houssem (Sousse;  Sahloul Hospital). |
| Turkey: ÜC Köksoy (ANKARA; UFUK ÜNİVERSİTESİ TIP FAKÜLTESİ DR.RIDVAN EGE SAĞLIK ARAŞTIRMA UYGULAMA MERKEZİ HASTANESİ); M Sertkaya (Adıyaman; Özel Adıyaman Park Hospital); V Oter (Ankara; Ankara CITY Hospital); C Akyol (Ankara; Ankara University Medical School); CE Guldogan (Ankara; Liv Hospital); E Haberal (Ankara; SBÜ Ankara Onkoloji Eğitim ve Araştırma Hastanesi); S Erel (Ankara; Saglik Bilimleri University); NU Dogan (Antalya; Akdeniz University Hospital); O Yalkın (Bursa; Bursa City Hospital); U Sungurtekin (Denizli; Pamukkale University School of Medicine); A Ulkucu (Edirne; Trakya University Hospital); B Yigit (Elazig; Elazig Fethi Sekin City Hospital); AB Ulas (Erzurum; Ataturk University School of Medicine, Research and Training Hospital); S Zenger (Istanbul; American Hospital); Y Altinel (Istanbul; Bagcilar Research And Training Hospital); ÖP Zanbak Mutlu (Istanbul; Bahçelievler State Hospital); C Tatar (Istanbul; Istanbul Education and Research Hospital); C Sen (Istanbul; Istanbul University - İstanbul Faculty of Medicine); E Erginöz (Istanbul; Istanbul universty - Cerrahpaşa Medical faculty); A Özcan (Istanbul; Kanuni Sultan Suleyman Training and Research Hospital); K Saracoglu (Istanbul; Kartal Dr. Lutfi Kirdar Training and Research Hospital); C Taskiran (Istanbul; Koç University Medical School); B Canbay Torun (Istanbul; Liv Hospital Ulus); B Celik (Istanbul; Liv Hospital Vadistanbul); TK Uprak (Istanbul; Marmara University, School of Medicine); AN Sanli (Istanbul; Silivri State Hospital); E Baran (Istanbul; Sisli Hamidiye Etfal Training and Research Hospital); OF Ozkan (Istanbul; University of Health Science Umraniye Education and Research Hospital); B Citgez (Istanbul; Uskudar University Faculty of Medicine, Memorial Hospital); B Calik (Izmir; University of Health Sciences Izmir Bozyaka Training and Research Hospital); C Yüksel (Mersin; Mersin City Training and Research Hospital); R Şahin (Rize; Recep Tayyip Erdogan University); E Gonullu (Sakarya; Sakarya Faculty Of Medicine); E Colak, GO Kucuk (Samsun; Samsun University Samsun Training and Research Hospital); A Guner (Trabzon; Karadeniz Technical University Farabi Hospital); G Karadeniz Cakmak (Zonguldak; Zonguldak Bulent Ecevit University School of Medicine Research and Training Hospital); S Demirli Atici (İzmir; University of Health Sciences Tepecik Training and Research Hospital). |
| Uganda: H Lule (Kigumba; Kiryandongo Hospital). |
| Ukraine: M Paranyak (Lviv; Lviv Clinical Emergency Care Hospital). |
| United Arab Emirates: VS Basappanavar (Ajman; Sheikh Khalifa Medical City Ajman); K Abdel-Galil (Al-Ain, Abu Dhabi; Tawam Johns Hopkins Hospital); S Alshryda (Dubai; Al Jalila Children’s Speciality Hospital); FMA Abbas (Dubai; Dubai Hospital); D Mohammed (Dubai; Latifa Women and Children Hospital); R Kundra (Dubai; Mediclinic Parkview Hospital); ALR Michael (Dubai; Neurospinal Hospital); H Alsaadi (Dubai; Rashid Hospital). |
| United Kingdom: FC Anazor (Ashford; William Harvey Hospital); M Greenhalgh (Ashton-under-Lyne; Tameside and Glossop Integrated Care NHSFT); A Baldwin (Aylesbury; Stoke Mandeville, Wycombe General); P Ishak (Ayr; University Hospital Ayr); S Bandyopadhyay (Banbury; Horton Hospital); CS Ong (Bangor, North Wales; Ysbyty Gwynedd); M Lebe (Barnet; Barnet General Hospital); J Attwood (Barnsley; Barnsley Hospital NHS Foundation Trust); P Patel (Barrow in Furness; Furness General Hospital); N Ponugoti (Basingstoke; Basingstoke and North Hampshire Hospital); F Shekleton (Bath; Royal United Hospital Bath); F Georgiades (Bedford; Bedford Hospital); F Aljanadi (Belfast; Royal Victoria Hospital); N Chidumije (Birmingham; Heartlands Hospital); D Naumann (Birmingham; Queen Elizabeth Hospital Birmingham); C Nzekwue (Birmingham; Sandwell General Hospital); MH Siddique (Birmingham; Solihull Hospital); UA Halim (Blackburn; Royal Blackburn Hospital); T Chituku (Boston; Pilgrim Hospital); P Bobak (Bradford; Bradford Royal Infirmary); M Mohamed (Brighton; Royal Sussex County Hospital); CJ Bradshaw (Bristol; Bristol Royal Hospital for Children); L Rogers (Bristol; Bristol Royal Infirmary); H Claireaux (Camberley; Frimley Health NHS FT - Frimley Park); A Mansour, GD Stewart, J Ashcroft, L Wang, M Ghobrial, N Simon (Cambridge; Addenbrooke’s Hospital); A Coonar (Cambridge; Royal Papworth Hospital); T Combellack (Cardiff; University Hospital Llandough); R Luckwell (Cardiff; University Hospital of Wales); S Mannan (Carlisle; Cumberland Infirmary); R Ashour (Chester; Countess of Chester Hospital); S Federer (Chichester; St Richard’s Hospital); D Baskaran (Colchester; Colchester Hospital University); L Mcguigan (Coleraine; Causeway Hospital); A Surendran (Coventry; University Hospitals Coventry and Warwickshire NHS Trust); W Al-Khyatt, A Bateman (Derby; Royal Derby Hospital); M Zafar (Dumfries; Dumfries and Galloway Royal Infirmary); J Manickavasagam (Dundee; Ninewells Hospital); S Arumugam (Durham; University Hospital North Durham); P Cullis (Edinburgh; Royal Hospital for Children and Young People); S Davison (Edinburgh; Royal Infirmary of Edinburgh); A Laird (Edinburgh; Western General Hospital); J Phillips (Exeter; Royal Devon and Exeter Hospital); G Faulkner (Farnworth; Royal Bolton Hospital); C Grimes (Gillingham; Medway hospital); G Gradinariu (Glasgow; Golden Jubilee National Hospital); M Bogdan (Great Yarmouth; James Paget Univeristy NHS Foundation Trust Hospital); TK Madhuri (Guildford; Royal Surrey County Hospital); C Vosinakis, SP Hogston, S Ippoliti (Harrogate; Harrogate District Hospital); CA Leo (Harrow, London; St Mark’s Hospital); C Chiang (Hastings; East Sussex Healthcare (Conquest hospital and Eastbourne District General Hospital)); K Madhvani (Huddersfield; Huddersfield Royal Infirmary); M Mohamed (Inverness; Raigmore Hospital Inverness); G Karagiannidis (Ipswich; Ipswich Hospital); M Higgins (Kent; Darent Valley Hospital); J McKay (Kirkcaldy; Victoria Hospital Kirkcaldy); M Wilson (Larbert; Forth Valley Royal Hospital); A Peckham-Cooper, CT Karia, G Bourke (Leeds; St James’s University Hospital Leeds); K Brahmbhatt (Leicester; Leicester Royal Infirmary); D Worku (Lincoln; Lincoln County Hospital); R Lunevicius, S Pringle (Liverpool; Aintree University Hospital); B Kirmani (Liverpool; Liverpool Heart and Chest Hospital); M Jenkinson (Liverpool; The Walton Centre NHS Foundation Trust); F Brzeszczyński (Livingston; St Johns Hospital); T Szakmany (Llanyravon, Cwmbran; The Grange University Hospital); S Froghi (London; Ealing Hospital); M Fehervari (London; Hammersmith Hospital); E Bagouri (London; King’s College Hospital); I Gerogiannis (London; Kingston); M Bhatia (London; Princess Royal University Hospital); YC Tan (London; Queen Elizabeth Hospital, Woolwich); DA Raptis (London; Royal Free Hospital); MA Thaha, S Hammouche (London; Royal London Hospital); J Smelt (London; St George’s Hospital); A Singh, A Liddle (London; St Mary’s Hospital); HM Kocher (London; The London Clinic); H Layard Horsfall (London; The National Hospital for Neurology and Neurosurgery); C Parmar (London; The Whittington Hospital); A Nathan (London; University College London Hospital); J Sagar, R Talwar (Luton; Luton and Dunstable University Hospital); D Balasubramaniam (Maidstone; Tunbridge Wells Hospital); K Bhatia (Manchester; Manchester Royal Infirmary); S Bansal (Manchester; St Marys); E Myriokefalitaki (Manchester; The Christie Hospital); B Brown (Manchester; Wythenshawe Hospital); A Rangan (Middlesbrough; James Cook University Hospital); T Tay (Morecambe; Royal Lancaster Infirmary); JS Hammond (Newcastle upon Tyne; Newcastle Upon Tyne Hospitals NHS Foundation Trust); A Aujayeb (North Shields; Northumbria NHS Hospital Trust); K Shanthakunalan (Nottingham; Nottingham City Hospital); C Lewis-Lloyd (Nottingham; Queens Medical Centre); H Soleymani majd (Oxford; Churchill Hospital); J Hind (Oxford; John Radcliffe Hospital); H Gacaferi (Oxford; Nuffield Orthopaedic Centre); I Liew (Peterborough; Peterborough City Hospital); S Lakpriya (Plymouth; Derriford Hospital); Q Mustafa (Poole; Poole Hospital); M Noor (Portsmouth; Queen Alexandra Hospital); O Pathmanaban (Salford; Salford Royal Hospital); J Edwards (Sheffield; Sheffield Teaching Hospital NHS Foundation Trust); S Mastoridis (Slough; Frimley Health NHS FT - Wexham Park); M Rezacova (Southampton; Southampton General Hospital); E Laurent (Southend; Southend University Hospital); K Iyengar (Southport; Southport and Ormskirk NHS Trust); C Brown (St.Helier; Jersey General Hospital); R Baumber (Stanmore; Royal National Orthopaedic Hospital); S Karim (Stevenage; Lister Hospital); D Johnson (Stockport; Stepping Hill Hospital); B Sachdev (Stoke-on-Trent; Royal Stoke University Hospital); L Roberts (Sutton Coldfield; Good Hope Hospital); M Dube (Sutton-in-Ashfield; Kings Mill Hospital); M Hollyman (Taunton; Musgrove Park Hospital); N Ismail (Truro; Royal Cornwall Hospital); N Chandratreya (Weston-super-Mare; Weston General Hospital); J Barrow (Wigan; Wrightington, Wigan & Leigh NHS Foundation Trust); MFA Kamarizan (Wrexham; Wrexham Maelor Hospital). |
| United States: M Thomas (Aurora; University of Colorado Anschutz Medical Campus (CU Anschutz)); E Etchill (Baltimore, MD; Johns Hopkins Hospital); H Abdou (Baltimore; University of Maryland Medical Center Midtown); J Wang (Boston, MA; Boston Medical Center); H Kaafarani (Boston, MA; Massachusetts General Hospital); AC Pfaff (Boston; Beth Israel Deaconess Medical Center); SK Bridges (Boston; Boston Children’s Hospital); M Sulciner (Boston; Brigham and Women’s Hospital); M Naunheim (Boston; Massachusetts Eye and Ear); S Ross (Charlotte; Atrium Health Carolinas Medical Center); A Turan (Cleveland, Ohio; Cleveland Clinic Foundation); J Rodriquez (Colorado Springs; Memorial Hospital); HE Rice (Durham, NC; Duke University Medical Center); S Khan (Houston; Children’s Memorial Hermann Hospital); CL Roland (Houston; University of Texas MD Anderson Cancer Center); RJ Robitsek (Jamaica; Jamaica Hospital); B Daley (Knoxville; University of Tennessee Medical Center); E Giorgakis (Little Rock; University of Arkansas for Medical Sciences); RB Cameron (Los Angeles; Ronald Reagan UCLA Medical Center); P Benharash (Los Angeles; University of California Los Angeles); N Nudell (Loveland, Colorado; Medical Center of the Rockies); B Bankhead (Lubbock, Texas; University Medical Center Lubbock); T Diehl (Madison; University of Wisconsin); C O’Neil (Miami, FL; University of Miami Hospital); A Kothari (Milwaukee, WI; Medical College of Wisconsin); E Colonna (Minneapolis, Minnesota; Hennepin Healthcare); C Walters (Nashville; Vanderbilt University Medical Center); A Kalyanasundaram (New Haven, CT; Yale New Haven Hospital); P Petrone (New York, NY; NYU Langone Medical Center); A Liveris (New York; New York City Health and Hospitals - Jacobi Medical Center); N Glass (Newark, NJ; The University Hospital); C Paranjape (Newton, MA; Newton Wellesley Hospital); R Callcut (Sacramento; UC Davis Medical Center); A Choudhry (Syracuse; SUNY Upstate University Hospital); A Vincent (Washington; The George Washington University Hospital); SD Wexner (Weston; Cleveland Clinic Florida); K Dhaduk (Wilkes-Barre, Pennsylvania; Geisinger Wyoming Valley Medical Center); PN Martins (Worcester, MA; UMass Memorial Hospital). |
| Yemen, Rep.: M Al-Shehari (Sana’a; Al-Thawra Modern General Hospital). |

Collaborators

|  |
| --- |
| Afghanistan: S Qaderi, SH Mousavi, M Almahroush, M Delsoz (Kabul; NOOR Eye Hospital). |
| Albania: K Lilaj, E Shehi (Tirana; University Hospital Center Nene Tereza); I Dajti (Tirana; University hospital Koco Gliozheni); L Zijaj (Vlora; Regional Hospital of Vlora). |
| Algeria: A Chouakria, S Haddad, D Zineddine, ME Essalhi, K Djebabria (Annaba; Centre Hospitalier Universitaire Ibn Rochd); M Lahmar, B Lotfi, M Hichem, A Lakehal, Z Djama (Constantine; university hospital abdelhamid ben badis); A Bouchetara, A Tidjane, A Laredj (Oran; EHS-Canastel); B Tabeti, D Benali ammar, LA Bouziane, D Elkebir, A Tidjane, C Ouanezar, SA Zelmat, M Benmamar, AZ Boukli Hacene, J Mansouri (Oran; EHU-1st November 1954); , S Hind, ES Dif, SEH Seddik, A Djelloul , M Abdoun, S Bouaoud (Setif; Hospitalo-Universitaire Saadna Abdennour, Ferhat Abbas University); B Belarbi, A Bachiri, K Bensoltane, MA Mous, O Riffi (Tlemcen; Infant-Mother Hospital of Tlemcen); SEO Kacimi, SN Mesli (Tlemcen; University Hospital Center, Dr Tidjani Damerdji). |
| Argentina: L Vargas, D Deluca kobelanski, J Morales, JG Yaryura Montero, FR Pascual, SM Lucchini (Allende, Cordoba; Sanatorio Allende - Sede Cerro); R Bruballa, ML Petersen, R Boudou, P Violo Gonzalez, V Cano Busnelli, L Boccalatte, A Albani Forneris, F Holc, R Brandariz, M Buljubasich, L Llano, FB Casto, RS Croattini, C Poggi, C Uffelmann, MS Crespi Amor (Buenos Aires; Hospital Italiano de Buenos Aires); CM Vega, G Diaz Duarte, P Valdez, S Montal, C Chwat (Buenos Aires; Hospital Universitario Austral); FA Alvarez, J Liaño, MA Doniquian, F Llahi (Cordoba; Clinica Universitaria Reina Fabiola); H Borla, C Garcia astrada , M Furlan, ME Muriel, JS Della Fontana, SM Lucchini (Cordoba; Sanatorio Allende - Sede Nueva Cordoba); JC Pantoja Rodriguez, MM Caubet, S Matthiess, JL D’Addino, A Duro (Provincia de Buenos Aires; Hospital Prof Dr Bernardo A Houssay); J Kerman, SL Cumine, P Oliva, C Mas, MC Rey, R Balmaceda, M Galvarini, DV Saldivar Ozan, A Kerman (San juan; Sanatorio Argentino). |
| Aruba: M Gosselink (Oranjestad; Dr. Horacio E Oduber Hospital). |
| Australia: A Nicholson, V Kollias, B Beelders, D Watson, YH Lam (Adelaide; Flinders Medical Centre); T Sammour, K Kour, A Nagaratnam, MS Yee, SW Gan (Adelaide; Royal Adelaide Hospital); TG Mackay, J Gundara (Brisbane; Logan Hospital); R Shen, S Cooper, B King-Koi, S Oishi, D Highton, A Frankel (Brisbane; Princess Alexandra Hospital); S Li, T Millane, A Kataria, W Zore, S Bowman (Brisbane; Queen Elizabeth 2 Jubilee Hospital); C Bong, M Kwok, M Roberts (Brisbane; Royal Brisbane and Women’s Hospital); S Gananadha, G McKay, M El-Rashid, B Brew, K Thammasiraphop, MZ Zhu (Canberra; Canberra Hospital); V Machatsch, A Drahman, A Sutherland, S Salindera (Coffs Harbour NSW; Coffs Harbour Health Campus); S Sankpal, A Holmes, D Watters, S Nagra (Geelong; University Hospital Geelong); C Cornwell, T Le, S Laura, AC Dawson, EWY Lun (Gosford; Gosford Hospital); I Gunawardena, D Wong, P Fotheringham, G Francis, AC Dawson (Gosford; Gosford Private Hospital); S Shahrestani, JA Pasch, A Boccabella, J Reeves, D Townend (Lismore; Lismore Base Hospital); D Proud, A Grogan, P Le, V Peri, KR Qin (Melbourne; Austin Hospital); R Nataraja, S Leng , K Lim, L Tong, M Pacilli (Melbourne; Monash Childrens Hospital); T Hong, A Rajagopalan, J Jaya, S Peeroo, A Gray (Melbourne; Monash Health Casey Hospital); Y( Tsang, B Niknami, K Rowan, E Alpay, D Lowen, R Hodgson, , A Bui, S Condron, H Devan Nair, H Maung (Melbourne; Northern Hospital); A Heriot, T Yeung, T Poulton, SS Apte (Melbourne; Peter MacCallum Cancer Centre); L Chong, L Bradshaw, D Scott, S Banting (Melbourne; St Vincent’s Hospital); JCH Kong, Y Leang, K Shaw, M Alderuccio (Melbourne; The Alfred Hospital); J Bock, S Lidder, S Kanavathy (Melbourne; The Royal Melbourne Hospital); A Besson, R Suthakaran, J Qiao, C Choi, B Lauritz, J Yeung (Melbourne; Western Health - Footscray hospital and Sunshine hospital); S Stokes, M McLeod (Newcastle; Calvary Mater Newcastle); N Lott, J Gani, P Pockney, C O’Neill (Newcastle; John Hunter Hospital); S Bhat (Perth; Royal Perth Hospital); S Kahane, S Izwan, J Ng, J Kumar, R Manley (Robina; Robina Hospital); G Cox (Southport; Gold Coast University Hospital); J Daruwalla, S Badiani, S Mahmood, M Rosario, R Dalal, N Merrett, RH Moldovan (Sydney; Bankstown Hospital); N King, S Pincott, J Ip, A Pearson, C Apostolou,  (Sydney; Northern Beaches Hospital); Y Liang, A Chen, R De Silva , S Jacob, D Mwagiru, C Nahm (Westmead; Westmead Hospital); AC Dawson, R McGee, EWY Lun, U Pahalawatta (Wyong; Wyong Public Hospital). |
| Austria: A Leitner (Dornbirn; Krankenhaus der Stadt Dornbirn); G Djedovic, I Königsrainer (Feldkirch; Landeskrankenhaus Feldkirch); F Dreier (Graz-West; Landeskrankenhaus); P Schuh, S Uranitsch, C Schauer, G Moitzi, F Aigner, G Gemes (Graz; Barmherzige Brüder Krankenhaus, Graz); G Singer, J Lindenmann, P Puchwein, T Cohnert, B Michelitsch, DB Lumenta, A Hackl (Graz; Medical University of Graz); P Widschwendter, J Stephan, M Lengauer, TO Andraschofsky (Hall in Tirol; Landeskrankenhaus Hall); F Messner, F Naegele, M Ninkovic, L Gasteiger (Innsbruck; Innsbruck Medical University); P Kirchweger, D Rezaie, R Nadina, A Krause, C Rösch, J Huber (Linz; Ordensklinikum Linz Elisabethinen); A Fritz, T Saini, L Havranek, T Kratzer, I Fischer, M Biebl (Linz; Ordensklinikum Linz GmbH Barmherzige Schwestern); M Grechenig, M Lechner, R Gruber, I Muehlbacher , K Emmanuel, J Presl (Salzburg; Paracelsus Medical University Salzburg); J Schirnhofer, C Mittermair, G Wechselberger, F Koeninger, E Russe (Salzburg; Saint John of God Hospital Salzburg); P Riedl, E Haiden, A Binder (Tulln; Universitätsklinikum Tulln); D Traxler-Weidenauer, S Holawe (Vienna; General Hospital of Vienna); J Pfuner, P Köglberger, H Knotzer, CG Wiesinger (Wels; Klinikum Wels-Grieskirchen GmbH); J Burtscher, N Busse, F Trivik-Barrientos (Wiener Neustadt; Landesklinikum Wiener Neustadt). |
| Azerbaijan: A Ibrahimli, E Bayramov, E Samadov (Baku; Leyla Medical Center). |
| Bangladesh: S Islam, H Hakim, DM Hasanuzzaman, AK Basher, M Shadrul Alam (Dhaka; Dhaka Medical College Hospital). |
| Belgium: RM Bouttelgier, D Bulthé, J Bontinck, R Rasschaert, B Dhondt (Bornem; AZ Rivierenland); A Diez-Fraile, P Lamoral, J Abeloos, B Veys, J De Ceulaer (Brugge; AZ Sint-Jan Brugge-Oostende AV); A Van den Berg, D Van de putte, P Pattyn, H Vanommeslaeghe, G Van Ramshorst (Gent; University Hospital of Ghent). |
| Benin: F Kethy (Cotonou; Centre National Hospitalier et Universitaire Hubert Koutoukou Maga). |
| Bosnia and Herzegovina: J Miskovic, V Dragisic, M Kajic, M Boras, M Soljic (Mostar; SKB University Clinical Hospital Mostar); N Sabanovic Bajramovic, S Bajramovic (Sarajevo; Clinical Center University of Sarajevo); T Zulović, E Cehic, M Kovačević, A Cerovac (Zenica; Cantonal Hospital Zenica). |
| Brazil: I Reis, R Fernandes Rezende, OV Alvarenga Pereira, G Waldolato, R Esteves Pires (Belo Horizonte; Felicio Rocho); C Vantini Capasso Palamim, FAL Marson (Bragança Paulista; Hospital Universitário São Francisco de Assis na Providência de Deus); L Da Silveira Botacin, M Avelino, L Azevedo De Camargo (Goiânia; University Federal Hospital); C Záu Serpa de Araujo, A Lira dos Santos Leite, GMA Gomes, I Lima Buarque, A Vieira Barros (Maceio; Hospital Santa Casa de Misericordia de Maceio); EE Guerra, R Pedrini Cruz (Porto Alegre; Hospital Nossa Senhora da Conceição); C Pichler de Oliveira, L Moutinho, I Vieira Toledo , A Cunha Viana Júnior (Rio de Janeiro; Hospital Naval Marcílio Dias); FR Takeda (Sao Paulo; Hospital das Clinicas da Faculdade de Medicina da Universidade de São Paulo); ALM Lima, VC Carvalho, JDS Silva (Sao Paulo; Instituto de Ortopedia e Traumatologia do Hospital das Clinicas da Faculdade de Medicina da Universidade de Sao Paulo); FR Takeda, MFKP Ramos, A Dias, RM Terra, U Ribeiro Junior (Sao Paulo; Instituto do Cancer do Estado de São Paulo); B Tirapelli Gonçalves, S Aguiar Júnior, F Baroni Alves Makdissi, F José Fernandez Coimbra, G Baiocchi, LP Kowalski, T Da Silva, JG Vartanian, GB Carvalho, C Da Silva, R Ribeiro Meduna (São Paulo; A.C. Camargo Cancer Center); L Areias, FA Pereira Júnior, PFG Schippers, L Nakano, R Flumignan (São Paulo; Hospital São Paulo); ML Rodrigues Barbosa da Silva, PZ David, NJ Kim, TR Ijichi, RL Nunes (São Paulo; Notre Dame Intermédica - Hospital Salvalus); R Oliva, C Nardi, A Gatti (Taboão da Serra; Hospital Geral de Pirajussara); PHDS Fernandes (Uberlandia; Federal University of Uberlandia). |
| Bulgaria: V Neykov, D Georgiev, E Filipov, T Ivanov (Pleven; Heart and Brain - Pleven Hospital); A Mehta, E Merashka, M Abdullahi, D Dimitrov, D Nguen, M Karamanliev (Pleven; University Hospital Dr Georgi Stranski, Medical University - Pleven); N Belev, B Atanasov, M Slavchev (Plovdiv; University Hospital Eurohospital); P Kamenova, A Stavrov, A Vricheva, I Mihaylov, T Yotsov (Ruse; University Hospital Medika); D Dardanov, M Sokolov (Sofia; University Hospital Alexandrovska). |
| Cameroon: J Tanyi, CG Nwegbu, J Brown (Bamenda; Mbingo Baptist Hospital). |
| Canada: R Anantha, S McLennan, M Strickland (Edmonton; Royal Alexandra Hospital); D Bigam, K Dajani, A Dell, K Verhoeff (Edmonton; University of Alberta Hospital); D Briatico, A Luo, K Bailey (Hamilton; McMaster Children’s Hospital); L Luketic, S Ellis, M Anvari, D Hong, H Shanthanna (Hamilton; St. Joseph’s Healthcare Hamilton); AB Prempeh, E Schemitsch, J Martin (London; London Health Sciences Centre and St Josephs Health Care London); M Boutros, D Patel, EG Wong (Montreal; Jewish General Hospital); A Persad, G Groot (Saskatoon SK; Saskatoon City Hospital/Royal University Hospital/St. Paul’s Hospital); M Mozel, A Hasnat, S MacKenzie, S Lee (Vancouver; Royal Columbian Hospital); C Dandurand, J Suderman, M Diczbalis, L Lukoko, C Broe, K Mayson, J Street (Vancouver; Vancouver General Hospital). |
| Chile: FL De Cicco, J Saavedra, VJ Sepúlveda Zambrano, CA Mazuret Sepulveda (Santiago; Hospital Sótero de Río). |
| Colombia: F Giron Luque, A Garcia, NG Patino-Jaramillo, NF Pedraza Alonso (Bogota; Colombiana de Trasplantes); CA Polania Sandoval, N Caballero Otálora, SV Agudelo Mendoza, CJ Perez Rivera, PA Cabrera Rivera (Bogota; Fundacion Cardioinfantil-IC); JN Rodriguez Niño, C Rodriguez, D Sanabria, G Herrera-Almario (Bogota; Fundacion Santa Fe de Bogota); D Robayo, MA Triviño Cortes, M Vergel Gómez (Bogota; Hospital Simon Bolivar); RE Pinilla Morales, JL Velez Bernal, FR Ruiz Echeverría, R Pareja (Bogota; Instituto Nacional de Cancerologia); SE Sinisterra Díaz, I Cujiño, M Velasquez Galvis, AJ Nieto Calvache (Cali; Fundación Valle del Lili); J Orozco Mera, AM Martinez-Blanco, K Camargo-Parra, G Clemen, M Badiel (Cali; Hospital Universitario del Valle Evaristo García); MA Solís-Parra, LA Agredo Luna, JA Moreno Muñoz, CA Oliveros Ruiz, CM Orozco-Chamorro (Popayan; Clinica La Estancia); F Agredo Villaquiran, J Gomez, LF Leon Giron, M Palechor, EA Benavides Hernández (Popayan; Hospital Susana Lopez de Valencia); VD Olave Montaño, C Cortes-Mora, N Ortiz , A Sánchez-Gómez, JA Calvache (Popayán; Hospital Universitario San José). |
| Croatia: G Šantak (Pozega; County General Hospital Pozega); I Leto, D Smolic, S Baterl, M Jukić, J Mihanovic (Zadar; Zadar General Hospital); A Danic Hadzibegovic, M Maric, G Augustin, D Hiršl, T Kopjar, T Tokic (Zagreb; University Hospital Centre Zagreb); M Tarle, M Lorencin, M Mamic, I Luksic (Zagreb; University Hospital Dubrava). |
| Cuba: RDLC Rodríguez Ciria, O Suárez Batista (Santiago de Cuba; Hospital Dr. Ambrosio Grillo Portuondo). |
| Czech Republic: L Tulinsky, L Martinek (Ostrava; University Hospital Ostrava). |
| Denmark: N Songthawornpong, N Aagaard, CU Strømmen, RM Højsgaard, C Meyhoff (Copenhagen; Bispebjerg Hospital). |
| Dominican Republic: R Acra-Tolari, JA Echavarría Uceta, J Rivas, H Herrera, D Mejia De la Cruz (Santo Domingo; Hospital General Plaza de la Salud). |
| Ecuador: NA Cáceres Cárdenas, PV Bonilla Sanchez, RP Andrade Salinas, FE Yepez Yerovi, VD Alarcón Vela (Ambato; Hospital General Docente Ambato); AR Menendez Mite, E Arteaga Cedeño, DF Perez Correa, J Teran Jurado, E Ochoa Maldonado (Guayaquil; Hospital de Especialidades Teodoro Maldonado Carbo); DA Ronquillo Andrade, SC Gómez López (Ibarra; Hospital San Vicente de Paul); D Flores, E Otañez, J Crespo, L Ramírez, N Campuzano (Quito; AXXIS Hospital Quito Ecuador); MDC Reyes Puig, DS Peñaherrera Toapaxi, L Haro Supa, A Andrade, CA Mena García (Quito; Hospital General Docente de Calderón); AA Bernabé Esteban, A Zavala Segovia, MA Meza Fonseca, MJ Paspuel Villacís (Quito; Hospital General Enrique Garcés); E Proaño , GA Molina Proaño, G Jimenez (Quito; Hospital General IESS Quito Sur); SA Zárate León, J Arboleda, LE Cadena Castro, G Gavilanes Loor, F Ron, A Barreto Grimaldos, E Vásconez (Quito; Hospital Metropolitano); DM Noboa, P Melgar Muñoz, M Montero Vega, C Endara, WS Cartagena, NM Alegria Navarrete (Quito; Hospital Pablo Arturo Suarez); J García, E Zambrano, S Acosta , MD Mena Ramirez, J Arboleda,  (Quito; Hospital Pediatrico Baca Ortiz); RP Vargas Cordova, H Alexander-Leon, CE Muñoz Aguirre, MF Sotalin, S Valdivieso (Quito; Hospital San Francisco de Quito (IESS)); JR Negrete Ocampo , LF Huilca Logroño, CM Dominguez, AP Solis Pazmino, EP Lincango (Quito; Hospital Vozandes Quito); D Villegas Montalvo, CA Llerena Ojeda, MC Veintimilla Gonzalez, R Buenaño González (Quito; Hospital de Especialidades Eugenio Espejo); FD Rivadeneira Proano, BV Falconí Noriega, JA Mesias Logroño, F Endara, D Silva Segovia (Quito; Hospital militar (Hospital de Especialidades de FF.AA N°1)); FJ Guerra Brandt, GV Peña Saltos, N Parra Paredes, AP Celi-De La Torre, L Fuenmayor-González (Quito; Novaclínica Santa Cecilia). |
| Egypt: HAS Abd Elazeem Mohammed, P Gadelsyed, FA Monib, ME Ahmed Mohamed, MM Saad, O Farouk (Assiut; Assiut University Hospital); L El Fiky (Cairo; Ain Shams University Specialized Hospital); AES El kady, H Abozied, H Ghaith, A Bayomy, M Abdel-Maboud (Cairo; EL-Hussein University Hospital, Al-Azhar University, Faculty Of Medicine); A Elgazar, MA Elbadawy, A Elgenidy, AK Awad (Cairo; El Demerdash University Hospital); W Omar, A Tawheed, M Ibrahim , E Abdulkader, M El-Kassas (Cairo; Endemic Medicine Department, Helwan University); MMA Marei, A Nabil, M ElFiky (Cairo; Kasr Al Ainy Faculty of Medicine, Cairo University); S Ghozy, AY Azzam (Damietta; Damietta Specialized Hospital); M Sallam, EA Fayad, A Al-Touny, SA Al-Touny, A Sallam (Ismailia; Suez Canal University Hospital); MM Emara, AM Farouk, A Shehta (Mansoura; Gastrointestinal surgery center); A Sakr, M Shalaby, M Sadek, H Elghadban, H Elfeki (Mansoura; Mansoura University Hospital); IH Metwally, K Abdelwahab, M Shetiwy, K Gaballa, M Abdelkhalek (Mansoura; Oncology Center Mansoura University); M Alansary, M Omar (Qena; Qena University Hospital); S Abd-elsalam, A Mohamed Ads, M Elbahnasawy (Tanta; Tanta University Hospital); AS Abbas (giza; sheikh zayed specialized hospital). |
| Ethiopia: M Kidane, K Mohammed, A Tibelt, A Negussie (Addis Ababa; ALERT center); Y Abye Negatu, H Haile, K Shumbash (Addis Ababa; Lancet Specialized Hospital); H Hanna, F Gebreegziabher Gebrehiwot, Y Tedla, M Abebe (Addis Ababa; Saint Paul Hospital Millennium Medical College); YS Bezabih, S Hailu (Addis Ababa; Tikur Anbessa Specialized (Black Lion) Hospital); YB Akililu (Addis Ababa; Zewditu Memorial Hospital); BW Zewdie, D Bedane Hunde, D Degarege, , B Atnafu (Bahir Dar; Bahir Dar University Tibebe Ghion Specialized Hospital); G Debele, K Bekele (Goba; Maddawalabu University Goba Referral Hospital); MG Mengesha (Hawassa; Hawassa University Comprehensive Specialized Hospital); M Desalegn, M Seid, T Ejajo, D Mekango, HT Derilo, WF Besira (Hossana; Wachemo University Nigist Elleni Mohammed Memorial Referral Hospital); YY Metaferia, B Tesso, L Mude, G Mulugeta (Jimma; Jimma University Medical Center). |
| Finland: E Sarjanoja (Kemi; Länsi-Pohja Central Hospital); H Huhta, O Helminen, JH Kauppila (Oulu; Oulu University Hospital). |
| France: L Rony, F Bastard, F Schmitt (Angers; CHU Angers); L Harper (Bordeaux; CHU Bordeaux); C Andro, A Maffert, S Johan, P Aries, M Danguy des Déserts (Brest; Military Hospital Clermont Tonnerre (Hôpital des Armées)); M Andrea, C Lionel, V Villefranque, R Montero Macías, D Krief, A Police (Eaubonne; Hôpital Simone Veil); G Piessen, C Eveno (Lille; CHU Lille Hôpital Claude Huriez); N Periard, E Duchalais (Nantes; CHU Nantes); R Soussan, E Kantor (Paris; AP-HP Hopital Bichat Claude Bernard); C Crétolle (Paris; Hôpital Necker Enfants Malades - APHP); C De Ponthaud, O Scatton, C Goumard, L Genser, S Gaujoux (Paris; Pitie Salpetriere); L Ali, A Bonnard, A El Ghoneimi, P Mariani-Kurdjian, M Peycelon (Paris; Robert Debré Children University Hospital - APHP); M Juricic, S Laraqui Hossini, C Defert, AP Arnaud (Rennes; CHU Rennes - Hopital Sud); T Morichau-Beauchant, J Nahum, JM Maillet, G Mathieu (Saint Cenis; Centre Cardiologique du Nord); H Charbonneau (Toulouse; Clinique Pasteur). |
| Georgia: A Tvaladze, K Khutsishvili, Z Demetrashvili (Tbilisi; N.Kipshidze Central University Clinic). |
| Germany: P Winnand, F Hölzle, A Modabber, J Wallqvist, S Baumgarten, S Ziemann, B Schäfer (Aachen; University Hospital Aachen); F Sommer, B Sommer, E Shiban, H Kerndl, TD Warm, S Wolf, MC Schrempf (Augsburg; University Hospital Augsburg); T Schreiber, D Kaemmerer (Bad Berka; Zentralklinik Bad Berka); C Schineis, JC Lauscher, K Beyer, LD Lee, C Kamphues (Berlin; Charité University Medicine - Campus Benjamin Franklin); W Uhl, JJ Strotmann, J Knipschild , A Zamparas, P Höhn, T Fahlbusch (Bochum; St. Josef-Hospital); M Wittmann, TR Glowka, E Güresir, T Randau, F Bakhtiary, M Velten, M Silaschi, Z Kohistani, S Strieth, A Mustea, F Recker, JC Kalff, J Wach, E Schindler, M Wimmer (Bonn; University Hospital Bonn); LM Heindl, PA Wawer Matos, C Cursiefen, M Simon, AC Rokohl, B Babic, L Schiffmann, MR Mallmann, C Domröse, C Mallmann (Cologne; University Hospital of Cologne); J Weitz, O Radulova-Mauersberger, S Korn, C Praetorius, U Bork (Dresden; University Hospital Carl Gustav Carus, Technical University Dresden); J Straehle, N Neidert, J Beck, O Schnell, C Fung (Freiburg; University Medical Center Freiburg, Faculty of Medicine); M Sander, E Schneck, M Knitschke, M Hecker, C Koch (Giessen; University Hospital Giessen and Marburg); I Seiwerth, U Kisser, J Klose, J Kleeff, U Ronellenfitsch (Halle; University Hospital Halle); A Reiter, TO Klatte, K Frosch, A Korthaus , A Heuer (Hamburg; University Medical Center Hamburg-Eppendorf); I Gockel, D Branzan, AA Geisler, A Roth (Leipzig; University Hospital Leipzig); J Merkle, B Larsen, W Czerniak, T Straube, J Lindert (Lübeck; University Hosital Schleswig- Holstein); F Meyer, R Croner, H Krause, S Turial, A Rissmann (Magdeburg; University Hospital Magdeburg); MJ Battista, A Hasenburg, S Rohleder, TT König, VC Linz (Mainz; University Hospital Mainz); C Reissfelder, N Rahbari, E Birgin, M Rassweiler-Seyfried, S Seyfried (Mannheim; Mannheim University Medical Center (Universitätsmedizin Mannheim)); A Kirschniak, P Wilhelm, S Göller, L Van den Hil , J Rolinger (Moenchengladbach; Kliniken Maria Hilf); R Hatz, I Koch, D Steinhart, MG Stoleriu (Munich Gauting; Asklepios Pulmonary Hospital); N Dr. Seyfried, H Bronger, M Tariq, M Jahnen, D Reim, L Baumgart, B Meyer, A Wagner, J Gempt (Munich; Klinikum Rechts der Isar TUM School of Medicine); C Lampert, E Berg, A Zati Zehni, J Boschet, M Albertsmeier, N Börner (Munich; Ludwig Maximilian University of Munich - Großhadern); J Goedeke, G Cepele, M Lerchenberger, K Frank, AM Keppler (Munich; Ludwig Maximilian University of Munich - Innenstadt); I Pastau, E Schröder-Langfeld, R Alabo, W Bank, L Schröder (Offenbach; Ketteler Krankenhaus); K Oikonomou, K Pfister (Regensburg; University Hospital Regensburg); T Strate, H Honarpisheh, J Herzberg (Reinbek; Krankenhaus Reinbek St. Adolf-Stift); T Freiman, S Won, DD Dubinski, B Behmanesh, F Gessler (Rostock; University Hospital Rostock); R Widyaningsih, GA Stavrou (Saarbruecken; Klinikum Saarbruecken); A Königsrainer, C Steidle, C Yurttas, MW Löffler, M Quante (Tuebingen; University Hospital Tuebingen); P Ziegler, C Konrads (Tübingen; BG Klinik); S Degener, L Gallardo zamora, S Esmaeili Fathabadi, F Von Rundstedt, YM Lee, J Doerner, R Seiberth (Wuppertal; Helios Universitätsklinikum Wuppertal (Universität Witten/Herdecke)). |
| Ghana: EO Ofori, K Agyen Mensah, GA Rahman , P Koggoh (Cape-Coast; Cape Coast Teaching Hospital); DYD Agbley , N Naabo, N Jiagge , I Hagbevor, EA Nachelleh (Ho; Ho Teaching Hospital); TJK Adjeso, EMT Yenli, S Amoako Asirifi, M Sheriff, BD Nuertey, S Agyeiwaa Owusu, S Tabiri (Tamale; Tamale Teaching Hospital). |
| Greece: K Kalopita, K Stroumpoulis, I Grypiotis, V Kyvelos, G Micha (Athens; ‘Elena Venizelou’ General and Maternity hospital of Athens); D Korkolis, A Sarafi, D Balalis, DK Manatakis (Athens; Agios Savvas Anticancer Hospital); N Thomakos, A Rodolakis, A Prodromidou, K Angelou, E Stamatakis, D Haidopoulos (Athens; Alexandra General Hospital); L Chardalias, C Kontopoulou, D Politis, M Konstadoulakis, N Memos (Athens; Aretaieion Hospital); MK Konstantinidis, A Ioannidis (Athens; Athens Medical Center); DK Manatakis, N Tasis (Athens; Athens Naval and Veterans Hospital); T Sidiropoulos, M Papadoliopoulou, P Drakakis, C Koratzanis, P Kokoropoulos, N Michalopoulos (Athens; Attikon University General Hospital); KI Paraskevas (Athens; Central Clinic of Athens); S Kapiris, A Paraskeva , K Stamatis, M Vailas, M Sotiropoulou (Athens; Evaggelismos General Hospital); D Dellaportas, P Lykoudis, C Nastos, E Pikoulis, A Paspala (Athens; Evgenideio Hospital); C Aggeli, A Thanasa, C Parianos , K Pateas (Athens; G. Gennimatas Hospital); G Chrysovitsiotis, A Chrysovergis, E Giotakis, V Papanikolaou, E Kyrodimos (Athens; Hippocratio General Hospital); O Bellou, N Korres, E Fandridis, F Giannoulis, S Spyridonos, EC Tampaki (Athens; KAT Athens General Hospital); P Tsiantoula, A Antoniou, S Tzamtzidou, T Giannoulopoulos, K Roditis (Athens; Korgialenio-Benakio Hellenic Red Cross Hospital); N Machairas, S Kykalos, A Syllaios, L Karydakis, MD Keramida, D Schizas (Athens; Laiko University Hospital); K Apostolou (Athens; Mediterraneo Hospital); I Manolitsis, A Skolarikos, P Mourmouris, T Bellos, L Tzelves (Athens; Sismanoglio - Amalia Fleming General Hospital); E Spartalis, GE Zakynthinos, G Schismenou, M Spartalis (Athens; Sotiria General Hospital of Thoracic Diseases); E Xynos, E Chrysos, S Xenaki (Heraklion Crete; University Hospital of Heraklion Crete); P Christidis, N Gougoulias (Katerini; General Hospital of Katerini); E Lostoridis, I Triantafyllidis (Kavala; Kavala General Hospital); K Bouliaris, C Kolla, C Doudakmanis, A Koulouktsis, G Koukoulis (Larissa; General Hospital of Larissa ‘Koutlimpaneio and Triantafylleio’); A Daponte, J Hajiioannou, E Gkrinia, C Rarras, C Donoudis, E Arnaoutoglou, A Samara, MP Ntalouka (Larrisa; General University Hospital of Larissa); A Politi, A Dimas (Lefkada; General Hospital of Lefkada); G Verras, K Bouchagier, M Argentou, L Tchabashvili, F Mulita (Patras; General University Hospital of Patras); N Kalyva, V Mousafeiris (Patras; Karamandaneio Prefecture Children Hospital of Patras); EA Manioti, O Mouzakis, E Barkolias , G Zeringa, A Papadopoulos (Piraeus; General Hospital of Nikaia); E Kontis, L Katsiaras, E Kaouras, P Manikis, I Katsaros (Piraeus; Metaxa Cancer Hospital); A Tsiaka, M Merrakos, E Bourmpouteli, I Papazacharias, N Zampitis, D Georgiadou (Piraeus; Tzaneio General Hospital); A Tsolakidis, F Stavrou, A Mantevas, I Tilaveridis, V Vrangalas (Thessaloniki; 424 General Military Hospital); P Antonogloudis, KM Valiouli, M Anastasakis , I Valioulis (Thessaloniki; G. Gennimatas Thessaloniki General Hospital); S Simeonidis, S Bitsianis, E Anestiadou, L Loutzidou, O Ioannidis (Thessaloniki; George Papanikolaou General Hospital of Thessaloniki); I Tsakiridis, I Kalogiannidis, A Mamopoulos, G Kapetanios, T Dagklis (Thessaloniki; Hippocratio Hospital); C Zymperdikas, C Anthoulakis, C Kaselas, I Spyridakis, M Tsopozidi, K Papavasiliou, E Tsiridis, P Chatzikomnitsa, G Arampatzis, P Bangeas, P Loufopoulos, G Arampatzis (Thessaloniki; Papageorgiou General Hospital); SC Liapis, N Tsantikos, D Lytras (Volos; Achillopoyleio General Hospital of Volos). |
| Guatemala: A Vaquiro Valencia, AI Toledo Castejón, Z Kleiman, R Quevedo, J Montufar, G Recinos (Guatemala City; Hospital De Accidentes Ceibal); G Ajcip, JM Cojulun Barrera, J Mollinedo-Hun, JA Alvarez Nufio, M Aguilera-Arevalo (Guatemala City; Hospital General San Juan De Dios); E Brolo (Guatemala; Hospital Universitario Esperanza). |
| Hong Kong SAR, China: A Kwan, RW Lau, MF Ho, J Chan, GKC Wong, K Futaba (Sha Tin; Prince of Wales Hospital). |
| Hungary: L Hidi, P Legeza, G Gyurok, P Sotonyi (Budapest; Semmelweis University (please use for all units)). |
| India: JA Santoshi, M Nagar, V Verma, R Ul HAQ, P Behera (BHOPAL; All India Institute of Medical Sciences Bhopal); L Sasatti,  I Madabhavi (Bagalkot; Kerudi Cancer Hospital); RB Jayaram, P Chiriapanda uthappa, D Raju, A Kudpaje, PA Shah (Bangalore; Cyte Care Cancer Hospital); S Temkar, PK Tungala, MK Habeeb, S Kumar , S Kumar Venkatappa (Bangalore; Victoria Hospital); NR Desai, D Shah, S Chaudhary (Baroda; Government Medical College and SSG Hospital); A Kumar, A Rai, N Mishra (Bhopal; Gandhi Medical College and Sultania Zanana Hospital); S Singh, R Kansay, R Kaushik, A Gupta (Chandigarh; Government medical college hospital); L Kaman, P Salunke, Y Sakaray, K Singh, M Karthigeyan, V Kumar, M Tripathi, DP Jani, DRK Bhatta, S Mohindra (Chandigarh; Postgraduate Institute of Medical Education & Research, Chandigarh, India); JS Rajasekar, M Rela, I Kaliamoorthy, G Narasimhan, A Rammohan (Chennai; Dr.Rela Institute & Medical Centre); A Sidhu, M Moni, D T Sathyapalan, A Rajanbabu (Cochin; Amrita Institute of Medical Sciences Hospital); PK Garg, A Tiwari, P Kaul (Dehradun, Uttarakhand; Shri Guru Ram Rai Institute of Medical and Health Sciences); C E, S Muthu (Dindigul, Tamil Nadu; Government Medical College and Hospital, Dindigul); A Gupta, DK Sharma, DAS Rai (Gurugram, Haryana; Paras Hospital); VM Hanjoora, A Govil,  M Raut (Gurugram; Artemis Health Institute); JR Vishnoi, P Pareek, P Singh, N Sharma, S Misra (Jodhpur; All India Institute of Medical Sciences (AIIMS), Jodhpur); A Pipara, G Aggarwal, P Jain,  A Sharma (Kolkata; Tata Medical Center); V Kumar, S Rajan (Lucknow; King George’s Medical University); R Jain, K Mandrelle, T Goel, M Kumari, P Pawar (Ludhiana; Christian Medical College & Hospital); A Viola D Souza, BZ Hameed, S Shetty, D Kanagal, S Lasrado (Mangalore, Karnataka; Father Muller Medical College); V M, N Mohammed, R Shetty, A Ht rao, DKK K M, J Akbar (Mangalore; Yenepoya medical college hospital); S Mathew, S Ibrahim, G Menon, N Kamath, S D’Cruz, B L, A Suresh kumar, M Ubarale (Manipal; Kasturba Medical College Hospital, Manipal); S Patkar, P Poddar, M Pal, S Thiagarajan, S Joshi (Mumbai; Tata Memorial Hospital); R Ghodke, A Gunjotikar, S Singh, DP Bhor, DS Dhar, S Kale (NAVI MUMBAI; D Y Patil Hospital); PR Menon, AK Bisoi, K Sikka, A Mishra, S Sharma, A Chaturvedi, SS Kale, D Gp, S Mittal, RS Chouhan, SK Dube (New Delhi; All India Institute of Medical Sciences); HS Chhabra (New Delhi; Indian Spinal Injuries Center); K Garg, A Mishra, L Gupta, L Bains (New Delhi; Maulana Azad Medical College); J Rani, K Abhilashi (Patna, Bihar; Indira Gandhi Institute of Medical Sciences); P Anand, R Patil, MN Ketkar, R Martins, S Manglik (Pune, Maharashtra; Bharati Vidyapeeth); R Nerlikar, K Aher, A Faqih, V Sodhai (Pune; KEM Hospital and Research Center); V Singh, N Raj, V Mago, A Mondal, S Basu, F Huda, A Gaurav, R Kottayasamy Seenivasagam, VK Panwar, L Manoj Joshua (Rishikesh; All India Institute Of Medical Sciences); DS Kshirsagar, M Kaple, A Shukla, K Mahuli, S Deshpande, C Mahakalkar (Sawangi (Meghe), Wardha; Acharya Vinoba Bhave Rural Hospital); S Hegde, S Sreeram, S M Prasad, B Hu (Sullia; KVG Medical College & Hospital); D Dhanani, P Belani, P Lad, R Lohia, A Phadnis (thane; jupiter hospital). |
| Indonesia: A Faried, H Herman, FA Damara (Bandung; Dr. Hasan Sadikin Central General Hospital); A Shadrina, J Hidayat, AR Tantri, IU Surya, IS Febriana, AK Harzif, A Auerkari (Jakarta; Cipto Mangunkusumo National General Hospital & Universitas Indonesia); D Sari, F Indrarti, R Cahyono, SL Anwar (Yogyakarta; Central General Hospital dr. Sardjito). |
| Iran: M Asadi, Z Arizavi (Abadan; Valiasr Educational Hospital). |
| Iran, Islamic Rep.: M Sheybani-Arani, F Khajavi-Mayvan, S Defaee, SMS Ahmadi Rashti, M Shahi, A Salimi asl (Bandar Abbas; Shahid Mohammadi Hospital); I Mohammadbeigy, M Norouzi, K Maktabi, M Fakhrolmobasheri (Isfahan; Al Zahra Hospital); F Seyedi, H Mohammadi sardoo, A Kamali, A Hamidi (Jiroft; Imam Khomeini Hospital); S Ahmadi (Kashmar; Hazrat-e Abolfazl Hospital); H Nejad Biglari, A Alinaghi Langari, N Shahabinejad, A Payandeh, A Karbalaie (Kerman; Bahonar Hospital); M Etezadpour, A Babazadeh baghan, H Mottaghi Moghaddam Shahri, E Heidari (Mashhad; Ghaem Teaching Hospital); R Assadi, A Jangjoo, L Mashhadi, M Hosseinzadeh Maleki (Mashhad; Imam Reza hospital); H Fatemi manesh, N Najafian motahaver, Z Heydari, M Sotudeh, H Yusefi (Qom; Shahid Beheshti Hospital); C Emir Alavi, B Bakhshayesh Eghbali, A Sedighinejad, A Atefi (Rasht; Poursina Hospital); A Bolouriyan, T Chartab Mohammadi, M Bagheri (Tehran; Baqiyatallah Hospital); A Tizmaghz, P Brouki Milan (Tehran; Firoozabadi Hospital); A Karbalaie, E Shahbazi, Z Saberi, M Tofighi, N Hemmati, M Alemrajabi, B Shakiba (Tehran; Firoozgar General Hospital); SM Vahabi, MH Nasirpour, B Danaei, A Nasirpour (Tehran; Imam Hossein Hospital); MR Keramati, S Delazar, R Pourahmad, M Rashidbeygi (Tehran; Imam Khomeini Hospital Complex(IKHC)); M Mirsalehi, SMR Mohajeri (Tehran; Rasool-e-Akram Hospital); F Sadat Rahimi, F Esmaeili Tarki (Tehran; Shahid Modarres Hospital); MH Nabian, M Khajeh Alizadeh Attar, MN Tahmasebi, L Oryadi zanjani, F Vosoughi (Tehran; Shariati Hospital); SMK Aghamir, M Salehi Shadkami, Z Azimbeik (Tehran; Sina Hospital); S Pourhedayat, M Pashaei (Urmia; Imam Khomeini Hospital); S Vazifekhah, F Ghaffarizadeh, S Sane, H Houshyar (Urmia; Mahzad Women’s Medical Center); SA Mousavi, M Mansouri, A Meidany (Yazd; Shahid Sadoughi Hospital); MM Ansari, S Jalili, F Eskandari, M Ghaemi (Zanjan; Ayatollah Mousavi Hospital). |
| Iraq: MA Ramadhan (Baghdad; Baghdad Medical City); AKA Karantenachy (Baghdad; Zafaraniyah General Hospital); H Aldawoody (Khanaqin; Khanaqin general hospital); R Maala , M Alwash, Y Zwain , MA Al-Juaifari (Najaf; Al-Najaf Al-Ashraf Teaching Hospital); AT Hashim (Nassiryah; Al-Hussein Teaching hospital); Z Aras, H Ahmed kareem, C Ismael, S Sherzad, A Kareem Hama Ghareeb (Sulemaniyah; Shorsh teaching hospital). |
| Ireland: M Corrigan, A Mustafa, G Ismaili , R Tummon (Cork; Cork University Hospital); A Nic Giolla Bháin, D Hogan, H Agnus Moorthiraj, B Nolan, M O’Riordain, S O’Brien (Cork; Mercy University Hospital); M Javadpour, B Kewlani, S Murphy, S Ramjit, A Dhannoon (Dublin; Beaumont Hospital); M Aremu, SY Chee, Y Al-Mukhaizeem , E Burke (Dublin; Connolly Hospital Blanchardstown); C McCarthy, M Geary, C O connor, DB O’Connor, F Salameh (Dublin; Rotunda Hospital); J Larkin, P Mccormick, BJ Mehigan, C Keogh, C Donohue (Dublin; St James’s Hospital); H Heneghan, O Ryan, C Kennedy, P Cromwell, IS Reynolds (Dublin; St Vincent’s University Hospital); J Mahon, EC Roche, B Arthurs, J O’riordan, R Casey, É Ryan (Dublin; Tallaght Hospital); T Harding, IG Brown, C Flood, R Farnan, M Kerin, MG Davey (Galway; University Hospital Galway); A O’Neill, M O’Sullivan, B Yousaf, J O Connor, A O’driscoll-collins (Kerry; University Hospital Kerry); Y Mohamed, N Nemat, AB Alsayed , J Pretorius (Letterkenny; LETTERKENNY UNIVERSITY HOSPITAL); H Earley, P Neary, C Nic Gabhann , S Shet, B Creavin (Waterford; University Hospital Waterford/University College Cork). |
| Israel: S Abu Freih, I Hazan, S Davidesko, E Quint (Beer-Sheva; Soroka University Medical Center); JA Demma, Y Fishman, L Luques, G Marom (Jerusalem; Hadassah Medical Center); Y Gozal, N Avni, D Greenman, O Nahtomi Shick (Jerusalem; Shaare Zedek Medical Center); N Tibi, M Yassin, W Abu Rashed, N Issa, ר פודוקשיק, E Bekhor (Petah Tikva; Hasharon Hospital); R Anteby, M Kyzer , A Mansour, N Horesh, Y Zager, S Soffer (Ramat Gan; Sheba Medical Center). |
| Italy: L Catarzi, G Consorti (Ancona; Ospedali Riuniti di Ancona); A Grasso , M Clementi (Aquila; San Salvatore Hospital, University of L’Aquila); M Gubbiotti, GM Pirola (Arezzo; Ospedale San Donato USL Toscana Sud Est); S Pollesel, A Lauretta (Aviano; Centro di Riferimento Oncologico di Aviano (CRO) IRCCS); R Laforgia, G Martines, A Picciariello (Bari; Azienda Ospedaliero Universitaria Consorziale Policlinico Di Bari); A Gori, IS Russo, A Belvedere, T Violante, M Rottoli (Bologna; IRCCS Azienda Ospedaliero-Universitaria di Bologna); E La Corte,  E Migliorino, S Bolognesi , R Aspide (Bologna; IRCCS Istituto delle Scienze Neurologiche di Bologna); AM Baietti, S Guicciardi, R Mancini, MP Lauretta, G Fabbri, GM Prucher (Bologna; Ospedale Maggiore/Bellaria Carlo Alberto Pizzardi AUSL Bologna); A Arena, S Ferla, A Raffone, L Cocchi (Bologna; Ospedale Villa Laura); A Malpaga, G Scotton, F Notte, G Bertelli, G Armatura (Bolzano; Bolzano Central Hospital); L Zanin, F Parolini, E Biancardi, F Ferrari, S Cattaneo, J Andreuccetti, G Pignata, F Odicino, D Alberti, M Fontanella (Brescia; ASST Spedali Civili, Ospedale di Brescia); A Pisanu, F Boriani, A Figus, V Murzi, E Gessa, M Podda (Cagliari; Cagliari University Hospital); R Cabula, A Borzacchelli, A Cannavera, M Deplano, ML Robuschi (Cagliari; Santissima Trinità - ATS Sardegna); P Avella, C Luciani (Campobasso; Antonio Cardarelli); F Pezzolla, R Isernia, G Lantone (Castellana Grotte (Ba);IRCCS ‘Saverio de Bellis’); C Distefano, R Gioco, A Volpicelli, V Pavone, M Veroux, M Migliore, L Stella, G Riccioli, D Corona (Catania; Azienda Ospedaliero- Universitaria Policlinico San Marco); V Scorcia, A Carnevali, M Borselli, G Currò, MG Cristofaro, G Giannaccare (Catanzaro; University ‘Magna Graecia’ of Catanzaro); F Catena, C Vallicelli, D Perrina, N Zanini (Cesena; Ospedale M. Bufalini); F Mucilli, SM Maggiore, M Liberati, I Dell’Atti, M Barone, A Muraglia (Chieti; Policlinico Santissima Annunziata); P Lepiane, F Saraceno, A Balla (Civitavecchia; San Paolo Hospital); A Vannelli, A Farro, M Maspero, B Zamburlini, A Romanzi (Como; Valduce Hospital); D Paglione, P Roberto andrea, M Osso, A Pietramala, E Bonaiuto, B Nardo (Cosenza; Azienda Ospedaliera di Cosenza); MC Giuffrida, G Giraudo, S Armentano, E Beltrami, D Sasia (Cuneo; Santa Croce e Carle Hospital, Cuneo); M De Francesco, F Calabrese, G Bortolin (Domodossola; San Biagio Hospital, Domodossola - VB); M Koleva Radica, MG Sibilla, V Giordano, F Pepe, M Chiozza, P Carcoforo (Ferrara; Azienda Ospedaliero-Universitaria Di Ferrara); N Fabbri, A Pesce, M Bernabei, A Bianchera, CV Feo (Ferrara; Azienda Unità Sanitaria Locale di Ferrara); LG Locatello, O Gallo, M Tomaiuolo, S Scaringi, P Stefano, G Maggiore (Firenze; Azienda Ospedaliera Universitaria Careggi); T Nelli, R Borreca, E Adinolfi, A Anastasi, GIL Mottola (Firenze; Ospedale San Giovanni di Dio); G Pavone, M Pacilli, A Gerundo, A Ambrosi, V Lizzi, N Tartaglia (Foggia; Ospedali Riuniti Azienda Ospedaliera Universitaria); G Ercolani, L Solaini, CA Pacilio, F D’acapito (Forlì; Morgagni-Pierantoni); F Costanzo, A Razzore, A Barberis (Genoa; E.O. Ospedali Galliera); M Frascio, P Fregatti, B Sperotto, M Sparavigna, A Barbazza, S Scabini (Genoa; IRCCS Ospedale Policlinico San Martino); E Romairone, S Carrabetta, F Floris, F Ré, A Luzzi (Genoa; Ospedale Villa Scassi); MG Spampinato, S D’Ugo, M Filipponi, G Rollo, N Depalma (Lecce; P.O.’Vito Fazzi’); G Baldazzi, D Cassini, K Favilla, M Spalluto (Legnano; Ospedale di Legnano); F Fleres, M Aguennouz, F Benedetto, V Ficarra, M Caffo (Messina; Policlinico Universitario G. Martino of Messina); P Capitani, V Torrano , S Cimbanassi, R Giudici, SPB Cioffi (Milan; ASST Grande Ospedale Metropolitano Niguarda); B Giuliani, M Giovenzana, AM Saibene (Milan; ASST Santi Paolo e Carlo); G Mercante, F Gaino, F Carbone, A Luberto , A Spinelli (Milan; Humanitas Research Hospital); D Bona, A Sozzi, A Aiolfi (Milan; Istituto Clinico Sant’Ambrogio); G Sedda, L Spaggiari, L Bertolaccini (Milan; Istituto Europeo di Oncologia - IRCCS -Milano); F Cammarata, I Pezzoli, A Bondurri, F Albanesi, A Maffioli, F Colombo (Milan; Ospedale Luigi Sacco Milano); F Ferrara (Milan; San Carlo Borromeo); V Andreasi, F Gagliardi, M Piloni, L Bertoglio, P De Nardi (Milan; Università Vita-Salute San Raffaele); PL Proto, G Fallabrino, F Valenza, G Maltese, M Fiore (Milano; Fondazione IRCCS Istituto Nazionale dei Tumori, Milano); V Testa, R Rimonda, A Gattolin, A Trecci, F Fusini (Mondovì; Regina Montis Regalis Hospital, Mondovì); R Fruscio, N Tamini, S Ornaghi, P Masseria, LC Nespoli (Monza; Fondazione IRCCS San Gerardo dei Tintori Monza, Scuola di Medicina e Chirurgia, Università Milano Bicocca); M Milone, GD De Palma, S Vertaldi, A Marello, M Manigrasso (Naples; Federico II University of Naples); V Granata, R Patrone, F Izzo, C Cutolo (Naples; Istituto Nazionale Tumori Fondazione, Pascale IRCCS); S Gili (Naples; Ospedale S. Leonardo - ASL Napoli 3 sud, Castellammare di Stabia); G Marte, P Federico, A Petrillo, F Iazzetta, A Tufo (Naples; ospedale del mare); I Raimondo, D Di Giorgio, V Tondolo, G Garganese, P Campennì (Olbia; Mater Olbia Hospital); A Fiorini, A Chessa (Orbetello; San Giovanni di Dio); M Franza, A Cordova, E Cammarata, M Tripoli, F Toia (Palermo; Department of Surgical, Oncological and Oral Sciences. University of Palermo); S Viola, C Callari, D Di Miceli, L Licari (Palermo; FBF Buccheri La Ferla Palermo); F Calabretto, S Frassini, L Cobianchi (Pavia; Policlinico San Matteo); P Capelli, F Banchini, C Grassi, D Bonfili, L Conti (Piacenza; G. Da Saliceto); O Ghazouani, R Galleano (Pietra Ligure; Ospedale Santa Corona, Pietra Ligure (SV)); A Muratore, NS Pipitone Federico, M Calabrò (Pinerolo; Edoardo Agnelli); L Rossi, F Pecchia, G Ravenni, M Mastrangelo, S Korasidis, L Andreani (Pisa; Azienda Ospedaliero Universitaria Pisana); E Pinotti (Ponte San Pietro; Policlinico San Pietro); G Pascale, A Pascale, P Di Lascio, A Giuliani, L Petagna (Potenza; Azienda Ospedaliera Regionale ‘San Carlo’); V Mastrofilippo, L Aguzzoli, VD Mandato (Reggio Emilia; Azienda Unità Sanitaria Locale - IRCCS di Reggio Emilia); M Garino, R Barone, E Moggia, A Borello, C Marafante (Rivoli; Ospedale degli Infermi di Rivoli); V Cozza, E Bevilacqua, GF D’Onofrio, F Giovinazzo, C Parrilla, V De Simone, C Ratto, G Scambia, G Sganga, A Lanzone, F Ardito (Rome; Fondazione Policlinico Universitario Agostino Gemelli); R Pellini, G Petruzzi, G Simone, R Mastroianni, F Mazzola (Rome; IRCCS ‘Regina Elena’ National Cancer Institute); C Pathirannehalage Don, L Siragusa, G Tisone, G Sica, G Vanni (Rome; Policlinico Tor Vergata Hospital, Rome); A Mingoli, G Brachini, B Cirillo, PM Cicerchia, P Bruzzaniti, P Lapolla (Rome; Policlinico Umberto I Sapienza University of Rome); C Menna, C De Nunzio, G Melina, E Monaco, P Mercantini, S Fiorelli (Rome; Sant’Andrea Hospital, Sapienza University of Rome); D Spoletini, R Menditto, M Campanelli, G Lisi (Rome; Sant’Eugenio Hospital); L Bonavina, ELG Asti , S Siboni, A Lovece, P Milito, D Bernardi (San Donato; IRCCS Policlinico San Donato); T Perra, S Dessole, C Doria, F Pisanu, A Porcu (Sassari; Cliniche San Pietro, A.O.U. Sassari); F Roviello, L Verre, L Carbone, R Piagnerelli, L Marano (Siena; Azienda Ospedaliero Universitaria Senese); J Shahu, M Cervellera, L Sartarelli, V Tonini (Taranto; Santa Annunziata Hospital); FA Ciarleglio, G Viel, M Brolese, A Brolese (Trento; Santa Chiara Hospital); MS Mangano, P Midrio, M Romano, M Piccino, G Zanus, U Grossi (Treviso; Ca’ Foncello); S Trungu (Tricase; Cardinale G Panico Hospital); M Santarelli, C Piceni, B Parrella, L Brignone, M Marro, R Tutino (Turin; Città della Salute e della Scienza); M Desio, E Cocozza, G Borroni, S Megna, M Berselli (Varese Lombardy; University of Insubria, Ospedale di Circolo e Fondazione Macchi (Varese)); FM Di Flamminio, F Ghezzi, A Cromi, J Casarin, AS Laganà (Varese; Filippo Del Ponte Hospital, University of Insubria); M Franzinelli, F Lemma (Venezia; Dell’Angelo Hospital); G Bocca, G Querini, V Iori (Verbania; Castelli); M Franchi, G Molteni, GF Veraldi, A Guglielmi, R Salvia, T Campagnaro, G Turri (Verona; Azienda Ospedaliera Universitaria Integrata di Verona); M Piazza, A Vitali, H Impellizzeri, G Moretto, M Inama (Verona; Ospedale Pederzoli); F Dell’Aglio, A Frontali (Vimercate; ASST Brianza - Ospedale di Vimercate); R Tumolo, I Conversano, G Scialandrone, E Restini, P Cianci (andria; Lorenzo Bonomo); E Bannone, I Neri (brescia; Fondazione Poliambulanza); B Zampogna, GT Capolupo, F Carannante, G Pascarella, M Caricato (rome; policlinico universitario campus bio medico of rome); S Pregnolato, G Battello, G Calini, A Lechiancole, F Traunero, G Vizzielli, L Driul, C Tartari (udine; santa maria della misericordia). |
| Japan: H Kato, A Horiguchi, D Koike, H Tsunobuchi, H Yonekura (Aichi; Fujita Health University Bantane Hospital); S Tachibana, Y Yoshikawa, S Kazuma, S Satoshi, T Chaki (Hokkaido; Sapporo Medical University Hospital); T Okuno, Y Shida, F Heike, Y Okazawa (Hyogo; Hyogo Prefectural Amagasaki General Medical Center); K Hanazaki, T Namikawa (Kochi; Kochi Medical School Hospital); M Kawaguchi, K Kawamura, H Okada, M Ida (Nara; Nara Medical University); A Kuriyama (Okayama; Kurashiki Central Hospital); T Murakawa, H Kinoshita, N Miyashita, M Sekimoto, D Hashimoto (Osaka; Kansai Medical University); N Kuratani, S Ishida, M Takada, T Sakurai, Y Fujimoto (Saitama City; Saitama Children’s Medical Center); T Kato (Saitama; Saitama Medical Center, Saitama Medical University); I Kawagoe, M Hayashi, S Kaneko, M Fukuda, T Kochiyama (Tokyo; Juntendo University Hospital); M Ishikawa, Y Genda, H Mase, M Iwasaki (Tokyo; Nippon Medical School Hospital). |
| Jordan: M Abu-Jeyyab, N Hijazin , A Alja’afreh, S Alarood (Alkarak; Al Karak Hospital); S Hayajneh, F Al-kasaji, A Khaled, H Al-Fahel (Amman; Al-Basheer Hospital); A Alsuradi, A Eid, Z Al-sheikh ali, SJM Nofal, M Alqedrh , L Tarawneh (Amman; Al-Essra Hospital); R Soudi (Amman; Eye Speciality Hospital); R Alzu’bi, M Diab, MM Alkurdieh , BOF Alawneh, H Hussein (Amman; Ibn Al Haitham Hospital); S Al-Tahayneh , A Murad, S Husain, Z Ibrahim, B Alrayes (Amman; Islamic Hospital); S Khader, R Elmusa, R Saket , S Alameen , L Sawalha (Amman; Jordan Hospital); A Ali deeb, A Khamees (Amman; Jordan Red Cresent Hospital); H Qandeel, O Rousan, H Almoumani , L Hussein , Y Alawneh (Amman; Prince Hamza hospital); MEH Albanna, R Abu Salah, L Tarawneh, M Mraiyan, A Aladaileh, Z Al-sheikh ali (Aqaba; Islamic Hospital - Aqaba); R Falah, T Al-Dabaa, AR Al manasra, M Al-howthi, A Nasr, T Sawadi (Ar Ramtha; King Abdullah University Hospital/ Jordan University of Science and Technology); F Wafai, S Al Sharie, SA Al-Kailani, A Rababah (Irbid; Badea Governmental Hospital); A Alsheikh, H Al-Balas, S Awadi, W Almdallal, A Khamees (Irbid; Irbid Specialty Hospital); A Al-Darobi, R Ennab, A Al-Bourah, A Muashi , H Fadhel, AAM Fadhel (Irbid; Princess Basma Hospital); A Al Malkawi, A N. Abu Lil, A Al Sharie, D Al Zubi (Irbid; Yarmouk Governmental Hospital); A Alajalen, S Al-Falahat, A Zurgan, M Alfuqaha, M Alhawatmeh (Madaba; Al Nadeem Hospital); H Ababneh, B Al_sharash, M Ababneh (Zarqa;  New Zarqa Governmental Hospital). |
| Kuwait: DAH Alwadani , R Zakaria (Kuwait City; Al Amiri Hospital). |
| Lebanon: S Dbouk, M Bazzi, M Moussa, H Bazzi, R Awad, N Bazzi (Beirut; Al Zahraa Hospital University Medical Center); M Owiedat, R Itani (Beirut; Makassed General Hospital); S Karout, AR Achek, M B. Kassab, R Itani, F Fatouh (Beirut; Rafik Hariri University Hospital). |
| Libya: M Alzwei, K Tamoos (Alabyar; Alabyar General Hospital); M Fathi Al Gharyani (Benghazi; Al Hawari General Hospital); F Al maadany, M Alsharif (Benghazi; Al-jalaa Teaching/Trauma Hospital); IA Abuzeid, MN Albaraesi , W Aldressi (Benghazi; Benghazi Medical Center); H Bileid Bakeer, A Mohammed Abodina, ه سالم الحداد, A Alqaarh, A Alkaseek (Gharyan; Gharyan Central Hospital); G Yagoub , I. Larbah (Misurata; Alhikma hospital); A Egdeer, M Alazabi, G Birqeeq, A Msherghi, AAY Almugaddami (Nalut; Nalut Central Hospital); H Embarek, M Bilfaqirah (Sebha; Al-Majd Clinic); A Abdalhadi, F Abdulrahman, N Abdulla, S Abdeewi , M Abdelkabir (Sebha; Sabha Medical Center); N Abdelrahim (Sirte; Ibn Sina Teaching Hospital); D Abdoun, A Abudher , A Alamin, R Berbash (Tajora-Tripoli; Yashfeen Clinic); M Aliwa, I Zbida (Tajoura; National Heart Centre, Tajoura Heart Center Hospital); A Altobal, M Ly, A Mohamed alabany, A Meelad, A Khalifa (Tarhuna; Tarhuna General Hospital); S Alhudhairy, N Ali, R Alarabi, S Tamer, H Alameen (Tripoli; Al-Abraj Hospital); AE Elzoubi, K Ayad (Tripoli; Alkhalil hospital); A Jamal, MM Alwarfalli, AM Elosta, A Khaled, A Elhadi (Tripoli; Karat Al-Ain Hospital); SE Dwaga , FAD Elhajdawe (Tripoli; Metiga Hospital); E Alshareea, M Binnawara , E Abdulwahed (Tripoli; Tripoli Central Hospital); M Elghazal, M Said, A Abozid, A Bozakok, MFK Abu hallalah, O Altarhoni (Tripoli; Tripoli Medical Center/ Tripoli University Hospital);  IA Saleh (Zintan; Taqwa Clinic); H Ben Hasan , N Ben Hasan, M Alkchr (Zliten; Al Asmarya Islamic University, Zliten Medical Center). |
| Lithuania: E Dainius, A Parseliunas, E Kubiliute, A Kavaliauskaitė, D Venskutonis (Kaunas; LUHS Kaunas Hospital); K Urbonas, Z Dauksa, Z Dambrauskas, A Dauksa (Kaunas; Lithuanian University of Health Sciences Kaunas Clinics); E Stratilatovas, V Bernotaite, A Dulskas (Vilnius; National Cancer Institute); K Strupas, T Poskus, R Damaseviciute, A Grigonytė, JP Druta, A Gulla (Vilnius; Vilnius University Hospital Santaros Klinikos). |
| Malaysia: JY Soh, Z Zakaria, MP Wong, AFN Mohd Ghazi, R Ramely, AD Zakaria (Kelantan; Hospital Universiti Sains Malaysia); I Chik, DA Abdul Aziz, F Imran, N Md Din, R Ramli (Kuala Lumpur; Universiti Kebangsaan Malaysia Medical Centre); SA Nah, ARH Ramli, R Saaid, CY Tan, AC Roslani (Kuala Lumpur; University Malaya Medical Centre); K Voon, KL Tan, L Shien Loong , J Chung, J Choo Jun Hao, YJ Ng We Yong (Kuching, Sarawak; Sarawak General Hospital). |
| Mexico: GE Lugo Zamudio, Z Aboharp Hasan, A Rojas Aguilar, EE Sosa Duran (Ciudad de México; Hospital Juárez de México); LG Peña Balboa, R Santana Ortiz, J Orozco-Perez, SA Trujillo Ponce (Guadalajara; Antiguo Hospital Civil de Guadalajara); C Fuentes Orozco, FJ Barbosa Camacho, TA Cueto Valadez, AE Cueto Valadez, A Gonzalez Ojeda (Guadalajara; Hospital de Especialidades, CMNO-IMSS); O Vazquez-Romero, J Beristain-Hernandez (Mexico City; Centro Médico Nacional La Raza); N Salgado-Nesme, GA Buerba, FE Alvarez-Bautista, OE Posadas-Trujillo (Mexico City; Instituto Nacional de Ciencias Médicas y Nutrición ‘Salvador Zubirán’); AS Pimienta Ibarra, P Santillan-doherty, FJ Armas Zarate, JA Santibanez-Salgado, J Carranza Sarmina , F Bolanos-Morales (Mexico; Instituto Nacional de Enfermedades Respiratorias); A Maldonado Del Arenal, DA Macedo Falcon, E Nieto Ortega, L Escudero-Roque, A Navarrete-Peón, RD Licona-Meníndez (Pachuca; Sociedad Española de Beneficencia); LP Espinoza Padrón, D Herappe , V Servín, G Chavez, M Noguez Castillo (Querétaro; Hospital de especialidades del niño y la mujer); J Chávez Pacheco, A Izaguirre (Tamaulipas; Beneficencia Española de Tampico); L Martinez, MJ Martínez , DS Gonzalez , II Durán Sánchez , A Ramos-De la Medina (Veracruz; Hospital Español Veracruz). |
| Morocco: W Lahlou , S Driouich, O Zerzif El Miliani, C Elkettani, A Bourial (Casablanca; Mohammed VI International University Hospital); S Elghiati, S Touzani, S Toumi (Rabat; Cheikh Zaid International University Hospital); M Bensghir, H Balkhi, M Meziane, A Baite, A Elkoundi (Rabat; Hôpital Militaire Mohammed V); O Cherkaoui, I Skitioui, R Bouanane, A Amazouzi, N Boutimzine, L Boualila (Rabat; Hôpital des Spécialités - ONO); A Houmada, AM Majbar, A Benkabbou, R Mohsine, A Ghannam, A Souadka (Rabat; Institut National d’Oncologie). |
| Namibia: JT Abebrese, S Seibes, R Tjipetekera, PR Nashidengo (Windhoek; Windhoek Central Academic Hospital). |
| Nepal: S Koju, R Shrestha (Bhaktapur; Shahid Dharmabhakta National Transplant Centre); A Joshi, Y Dwa, S Bhusal (Lalitpur; KIST Medical College and Teaching Hospital). |
| Netherlands: EM Meima - van Praag, A Pronk, R Hompes, KR Wienholts, C Van Helsdingen, P Van Amstel, R Bakx (Amsterdam; Amsterdam UMC, University of Amsterdam); P Steinkamp, L Huisman (Groningen; Martini General Hospital Groningen); U Nakshbandi, CHC Houtsma, R Hogenbirk, P Steinkamp, S Kruijff, JF Lin (Groningen; University Medical Center Groningen); L Van der Laan, R Poelstra, PKC Jonker (Leeuwarden; Medisch Centrum Leeuwarden); K Van Dam, M Martens, E Boerma (Sittard/Heerlen; Zuyderland Medical Centre); F Aarts, T Schok, J Konsten (Venlo; VieCuri Medisch Centrum). |
| New Zealand: A Walter, A Bathgate, A Lin (Wellington; Wellington Regional Hospital). |
| Nigeria: A Adeyeye, AI Okunlola, A Akinmade (Ado Ekiti; Afe Babalola University Multi-System Hospital); A Musa Kirfi, A Adamu, KJ Bwala (Bauchi; Abubakar Tafawa Balewa University Teaching Hospital Bauchi); ED Odai, M Ezeanochie, O Odutola, AG Akpede, P Agbonrofo, E Egbor (Benin City; University of Benin Teaching Hospital); G Akaba, AM Umar, T Ibekwe, P Mshelbwala , S Olori (Gwagwalada; University of Abuja Teaching Hospital); R Abdus-salam, JA Balogun, A Omigbodun, A Malomo, TA Lawal (Ibadan; University College Hospital); II Aremu (Ilorin; General Hospital); EA Morgan, E Poluyi, J Daramola, E Morgan (Lagos; Awesome Grace Hospital); U Ezomike, S Ekpemo, C Uchendu, I Chukwu (Umuahia; Federal Medical Centre Umuahia). |
| North Macedonia: N Cokleska Shuntov, T Risteski, L Todorovic (Skopje; University Clinic for Pediatric Surgery); T Jovanoski, V Trenchev, K Jovanovska, S Tusheva, I Peev, G Georgieva, B Nikolovska, B Srbov, S Azmanova Mladenovska, S Pejkova (Skopje; University Clinic for Plastic and Reconstructive surgery, Faculty of Medicine, University St. Cyril and Mthodius). |
| Oman: M Al Naamani, J Massoud (Muscat; Khoula Hospital); P Shukla Misra, T Philip, P Umman, D Joshi, S Sheik (Muscat; Royal Oman Police Hospital); SM Ahmed, A Al Farai, K Galaal, J Mantri, S Kodange, P Andreoni (Muscat; Sultan Qaboos Comprehensive Cancer Centre); H Al Miskry, M Al Hinai, B Dawud, D Ghosh, Z Al Balushi, F Ali (Muscat; Sultan Qaboos University Hospital). |
| Pakistan: I Rashid, MS Malik, DS Azam, S Ali (Islamabad; Kulsum International Hospital); Z Zahid , DR Uppal, A Khan,  SR Uppal, U Saeed (Islamabad; South East Hospital); F Akhtar, DN Mahmood, N Aftab, DM Mehmood, SH Waqar (Islamabad; The Pakistan Institute of Medical Sciences); Z Zaheen, HH Khan, MA Qadri, MK Khan (Jamshoro; Liaquat University of Medical & Health Sciences); U Chishti, U Waqar, RD Ukrani, I Tahir, A Aziz (Karachi; Aga Khan University); UH Abdul Rauf, R Askari, O Saleem, AK Mahar, MR Malik, S Saeed (Karachi; Dow University Hospital); KA Samo, AS Memon, M Abbasi, H Khan, D Nanjiani, L Rai (Karachi; Dr Ruth K.M. Pfau Civil Hospital); RS Martins, UA Khawaja, A Saleem, MTA Shaikh, MTJ Khan (Karachi; Jinnah Medical College Hospital); M Ahmed, PDA Shaikh, K Rehman, A Azhar , A Ali (Karachi; PAF Faisal Hospital); MT Siddiqui, F Ashraf (Karachi; Patel Hospital); F Hanif, M Atiq, M Haroon, M Imran Khan, A Sarwar, AS Ammar (Lahore; Bahria International Hospital, Bahria Orchard); SA Naqi, S Manzoor, M Sohail, M Ayyub Anjum, M Farooq, MH Janjua, MZ Sarwar (Lahore; King Edward Medical University - Mayo Hospital); MN Rafique , H Amin, MA Saleem, K Khalid, H Ahmad, K Hayat (Lahore; Services Hospital Lahore); MB Mirza, HM Adnan , W Tahir, MU Aziz, GM Zafar, N Talat (Lahore; The Children’s Hospital & The Institute of Child Health Lahore); Z Ud din, S Akbar, S Parveen, S Fahad, H Ali (Peshawar; Hayatabad Medical Complex); AU Ashraf Butt, AR Malik, F Rauf, MS Khan, E Yaqoob (Rawalpindi; Benazir Bhutto Hospital); MA Ul Haq, E Yaqoob, S Javed (Rawalpindi; Holy Family Hospital). |
| Paraguay: DS Avila, C Chirico (Asuncion; Centro Médico La Costa); HR Segovia Lohse, HA Segovia Lohse (Lambaré; Hospital Distrital de Lambaré, Paraguay); C Chavez Rivaldi, MJ Martinez Velázquez , DM Añazco Mareco, A Arévalo Barreto, R Sánchez (San Lorenzo; Hospital De Clínicas). |
| Peru:  AF Vera Portilla, MM Manchego De La Cruz, GC Manrique Sila (Arequipa; Honorio Delgado Espinoza Regional Hospital); GM Falcon Pacheco (Arequipa; Instituto Regional de Enfermedades Neoplásicas del Sur); Y Auqui Medina, R Mas Melendez (Ayacucho; Ayacucho Regional Hospital); K Diaz, FE Campos Montoya, P Pozo Quispe, S Perez , JA Collantes Cubas (Cajamarca; Hospital Regional Docente de Cajamarca); J Rios Chiuyari (Lambayeque; Hospital regional de lambayeque); G Lizzetti, JF Quispe Mateo, ZS Leon Cabrera, L Bello, GS Yamamoto Seto, G Borda-Luque (Lima; Cayetano Heredia National Hospital); YE Villacorta Acosta, E Oré , W Siccha Dionicio , A Salazar, G Mendiola (Lima; Hospital Santa Rosa de Lima);  F Berrospi, E Payet, J Perez Villena (Lima; Instituto Nacional de Enfermedades Neoplásicas); S García Valenzuela, M Lopez, J Caballero-Alvarado (Trujillo; Hospital Regional Docente de Trujillo). |
| Philippines: J Flor, EJ Castro (Bayombong; Region 2 Trauma and Medical Center); CL Ramos, JMI Abellera, MB Balictar, H Amo, IM Lim (Manila; José R. Reyes Memorial Medical Center); KLE Violago, JN De Chavez, JB Baun, MT Tan, MP Lopez (Manila; Ospital ng Makati); E Estrella, A Chua, A Moreno, SL Bravo, R Li, MC Lapitan (Manila; Philippine General Hospital, University Of The Philippines Manila); JA Salud, RD Rivera, EG Tancinco, BK Chaplin, JAS Reyes (Pasig; The Medical City); MC Ampuan, A Sendad, KM Montejo (Quezon City; Victoriano Luna General Hospital). |
| Poland: W Kloc, S Adamski, M Węclewicz, O Liczbik, PT Stogowski (Gdansk; Szpital im. M. Kopernika / Copernicus Hospital); K Anuszkiewicz, D Łaski, Ł Kaska, J Jankau, T Stefaniak (Gdańsk; Uniwersyteckie Centrum Kliniczne); J Bicki, Ł Nawacki (Kielce; Wojewódzki Szpital Zespolony w Kielcach); K Żak, M Leziak, K Frankowska, A Ziółkiewicz, K Kułak, R Tarkowski (Lublin; Independent Public Teaching Hospital No 1 in Lublin); P Grabowski, R Domagalski, R Łanowy, P Januszyk, K Szyluk (Piekary Śląskie; District Hospital of Orthopedics and Trauma Surgery); Z Lorenc, M Nycz, W Kotyczka, D Szkudlapski, W Krawczyk, M Mietła (Sosnowiec; Wojewódzki Szpiital Specjalistyczny nr 5 im. Św Barbary); A Kwiatkowski, K Bartosiak, M Mawlichanów, T Syryło, M Walędziak, A Różańska-Walędziak (Warsaw; Military Institute Of Medicine); D Patkowski, S Gerus, D Borselle (Wrocław; Uniwersytecki Szpital Kliniczny we Wrocławiu). |
| Portugal: S Henriques, M Trindade, J Vaz, B Cismasiu, AL Preto Barreira (Almada; Hospital Garcia de Orta); C Amarante Dias, D Chalo (Aveiro; Centro Hospitalar do Baixo Vouga); M Rocha Melo, J Oliveira, C Velez, J Bolota, R Pedroso de Lima, A Machado (Evora; Hospital do Espirito Santo); MF Cunha, II Sampaio da Nóvoa Gomes Miguel, J Rachadell, E Amorim, B Silva Mendes (Portimao; Centro Hospitalar Universitario do Algarve - Unidade de Portimão); S Pinho, AR Teles, P Ramos, H Meleiro, C Granja (Porto; Centro Hospitalar e Universitário de São João); AP Ferreira Pinto, AM Correia, C Baía, JO Silva, M Marques (Porto; IPO Porto); B Vieira, J Ribeiro, A Dupont, C Leal, U Fernandes, D Martins (Vila Real; Centro Hospitalar de Trás-os-Montes e Alto Douro, E.P.E.). |
| Romania: C Cobelschi, A Hogea, M Hogea (Brasov; Brasov Emergency Clinical County Hospital); N Bacalbasa, I Balescu (Bucharest; ‘Dr. Ion Cantacuzino’ Clinical Hospital); A Chitul, C Bezede, E Ciofic, D Cristian, F Grama (Bucharest; Coltea Clinical Hospital); V Calu, O Enciu, A Miron, EA Toma (Bucharest; Elias Emergency Hospital); B Stoica, B Diaconescu, C Ciubotaru, V Negoiță, I Negoi (Bucharest; Emergency Clinical Hospital Bucharest); M Stefan, D Filipescu, T Bute, L Valeanu (Bucharest; Emergency Institute for Cardiovascular Diseases ‘Prof. Dr. C.C. Iliescu’); I Popescu, N Bacalbasa (Bucharest; Fundeni Clinical Institute); M Iliescu, M Davidescu, E Jianu, AO RUSU (married MOLDOVAN), N Motas (Bucharest; Institute of Oncology Prof Dr Al Trestioreanu); A Serban, TS Gavriliu, A Dobrescu, S Hamei, NS Ionescu (Bucharest; Maria Sklodowska Curie Emergency Hospital); RM Mirica, RV Iosifescu, M Zamfir-Chiru-Anton, AB Văcărașu, M Mardare, O Ginghina (Bucharest; Saint John Emergency Hospital); R Costea, E-C Zarnescu, R Pargaru (Bucharest; University Emergency Hospital Bucharest); RR Scurtu, A Raluca-Cristina, A Caziuc, S Tranca, R Drasovean (Cluj-Napoca; Cluj-Napoca Emergency County Hospital); P Achimas-Cadariu, IC Vlad, A Irimie, VA Gata, DM Herghea, C Lisencu, DT Eniu, V Schitcu, E Bonci, A Pasca (Cluj-Napoca; Prof Dr Ion Chiricuta Institute of Oncology); S Paitici, D Andreas, D Daniel, C Mogoanta, S Mogoanta (Craiova; Spitalul Judetean De Urgenta Din Craiova); M Pertea (Iasi; ‘Sf. Spiridon’ Emergency Clinical Hospital); S Lunca, A Musina, N Velenciuc, CE Roata, S Morarasu, M Dimofte (Iasi; Regional institute of Oncology Iasi). |
| Russian Federation: M Kurtenkov, A Frolova (Kaliningrad; Immanuel Kant Baltic Federal University, Regional Clinical Hospital); V Kochetkov, I Timchenko, L Sidorova, P Tsarkov, S Efetov (Moscow; IM Sechenov First Moscow State Medical University); I Dushin, Р Акчулпанов, V Subbotin, A Klimov (Moscow; Moscow Clinical Scientific Center named after AS Loginov); Y Frolova, N Korchazhkina, A Mikhailova, A Bedzhanyan (Moscow; Petrovsky National Research Centre of Surgery); V Likhvantsev, L Berikashvili, M Yadgarov, A Kuzovlev, K Kadantseva (Moscow; V. Negovskiy Reanimatology Research Institute); A Bazaev,  A Abelevich (Nizhny Novgorod; Privolzhsky Research Medical University); S Konev, M Alexeev, A Trofimov, V Skvortsov, S Efremov, O Kuleshov (Saint Petersburg; Saint Petersburg State University Hospital); Z Seytnebieva, A Dzhanaeva, A Aliev, M Rumyantseva, A Golomidov, A Butyrskii (Simferopol; Municipal Emegency Hospital No.6); M Grishenko, A Baranov, E Drozdov (Tomsk; Tomsk regional oncology hospital); Y Kudryavcev, T Mikhaylova, К Ким, V Ten (Yuzhno-Sakhalinsk; Private healthcare institution ‘RZD-Medicine’). |
| Rwanda: M Eugene, F Byiringiro, E Seruyange, A Costas-Chavarri (Kigali; Rwanda Military Hospital); M Ruhosha (Muhanga district; Kabgayi Hospital). |
| Saudi Arabia: M Rayzah, AK Alnemare, HM Albar, A Almutairi, N Alzerwi (Al-Majmaah; King Khalid General Hospital); M Alharthi, M Ghunaim, A Aljiffri , M Alkhatieb, N Trabulsi (Jeddah; King Abdulaziz University Hospital); A Althobaiti, A Alyami, M Algarni, A Alyami (Jeddah; King Khalid National Guard Hospital); M Alzamanan, M Hajlan, D Cortés-Guiral, M Alqannas, M Alyami (Najran; King Khalid Hospital); O AlAamer, S Alahmed, S Breakeit, A Alkabli, A Althumairi (Riyadh; King Abdulaziz Medical City); A Alhefdhi, O Alomar (Riyadh; King Faisal Specialist Hospital); SA Mir, M Eltayeb , FA Andrabi, S Alshahrani, S Chowdhury (Riyadh; King Saud Medical City); T Nouh (Riyadh; King Saud University); R Khan (Tabuk; King Fahad Specialist Hospital); A Alayed, H Adi, K Alsubaie , A Alqabasani , K AI Nwijy, DY Alalawi (Tabuk; King Salman Armed Forces Hospital). |
| Serbia: I Paunovic, N Slijepcevic, K Tausanovic, M Buzejic, V Zivaljevic (Belgrade; Centre for endocrine surgery, University Clinical Centre of Serbia); S Kmezić, I Pejovic, L Aleksić, M Veselinović, B Tadic, N Grubor, M Reljic, T Babic (Belgrade; Clinic for Digestive surgery, University Clinical Center of Serbia, University of Belgrade, Faculty of Medicine); M Perovic, L Srbinovic (Belgrade; Clinic for Gynecology and Obstetrics Narodni Front); S Kadija, K Jeremic Stefanovic, M Radojevic, I Pilic, A Stefanovic (Belgrade; Clinic for Gynecology and Obstetrics, University Clinical Center of Serbia); O Pavlovic, C Kuzmanovic, R Spasic, D Jelovac, S Hajdarević (Belgrade; Clinic for Maxillofacial Surgery, School of Dental Medicine, University of Belgrade); M Folic, J Milovanovic, A Trivic, S Krejovic Trivic, A Jotic (Belgrade; Clinic for Otorhinolaryngology and Maxillofacial Surgery, University Clinical Center of Serbia); V Santric, B Stankovic, I Vukovic, N Prijović, U Babic, Z Dzamic, M Zivkovic, B Milojevic, B Kajmaković, U Bumbasirevic (Belgrade; Clinic of Urology, University Clinical Center of Serbia); N Mitrovic, D Jasarovic, A Lazic, B Lukić, D Stevanovic (Belgrade; Clnical hospital centre of Zemun); P Savic, M Milanovic, N Pijanovic, M Radosavljevic (Belgrade; KBC Dr Dragisa Misovic-Dedinje); M Lazovic, S Ducic (Belgrade; University Children’s Hospital); V Milosavljevic, B Crnokrak, A Sekulic, I Nadj, H Karaca (Belgrade; University Hospital Medical Center Bezanijska Kosa); J Juloski, V Cuk, M Kenic, I Krdzic, A Karamarkovic (Belgrade; Zvezdara University Medical Center); B Stojanovic, B Milosevic, M Pavlovic (Kragujevac; University Clinical Center of Kragujevac); V Djan, V Trajkovic, M Milenkovic (Djermanov), R Goran, D Zivkovic (Novi Sad; Institute for Child and youth Health Care of Vojvodina); M Kresoja, M Ranisavljević, D Golijanin, M Đurić, M Protic (Sremska Kamenica, Novi Sad; Oncology Institute of Vojvodina, University of Novi Sad - Faculty of Medicine). |
| Singapore: YLM Seet, PS Lew, NZ Teo, A Chiow, JX Hing (Singapore; Changi General Hospital); Y Low, C Choo, DM Dimatatac (Singapore; KK Women’s and Children’s Hospital); F Leong, S Ngaserin, HW Chua , A Tan, KM Chue (Singapore; Sengkang General Hospital); MNA Tan, KY Wong, N Goh, EY Tan, W Ang (Singapore; Tan Tock Seng Hospital). |
| Slovak Republic: B Gális, K Šimko, L Czako, M Vavro, A Panyko, B Dvoranova (Bratislava; University Hospital Bratislava). |
| Slovenia: J Grosek, A Tomazic, T Košir Božič, JA Košir (Ljubljana; University Medical Centre); A Cokan, M Pakiž, N Kavčič, T Bratuš, D Bratus (Maribor; University Medical Centre). |
| South Africa: M Flint, S Gumede, N Almgla, SM Peters, M Hannington, C Kloppers (Cape Town; Groote Schuur Hospital); A Davies, C Pryce, D Van Eck, ZM Limalia, C Makepeace, G Dunbar (Cape Town; Victoria Hospital Wynberg). |
| Spain: A Sánchez Mozo (Albacete; Complejo hospitalario universitario de Albacete); H Aguado López (Albacete; Hellín Hospital); G Mínguez Ruiz, A Suárez Sánchez, C Magadán Álvarez, MV Sosa, A Rodriguez Infante (Avilés; San Agustín University Hospital); E González Marín, M Latorre Gómez, S De la Cruz Ahufinger, C Pijoan-Lara, L Ruiz-Villa (BARCELONA; Hospital Universitari Sagrat Cor); I Villalabeitia Ateca, T Pascual, B Villota Tamayo, S Mambrilla, A Perfecto, M Prieto (Barakaldo; Hospital Universitario Cruces); L Cayetano Paniagua, P Palma, S Pou Macayo, A Adroher, L Gomez Fernandez (Barcelona; Consorci Sanitari de Terrassa); J Querolt Coll, G Triana, M Xicola, M Font , M Rosines Cubells, JM Muñoz Vives (Barcelona; Fundació Althaia - Xarxa Assistencial Universitària de Manresa); M López-Baamonde, R Valero, I Gracia, N Fabregas, L Gomez Lopez, A Carreras-Castañer, A Castells, LJ Ramirez-Nuñez, M Ferrer Banús, R Sieira-Gil, A Ferrer Fuertes, FJ Cuesta-González, D Gutiérrez Medina, M Monfort Mira, M Renau-Cerrillo, C Vargas Reverón (Barcelona; Hospital Clinic Barcelona); X Tarrado, M Coronas Soucheiron, C Massaguer, B Capdevila Vilaró, O Martin Sole (Barcelona; Hospital Sant Joan de Deu); B Escudero, I Omiste, F Angles Crespo, D Bosch Garcia, J Nuñez (Barcelona; Hospital Universitario Mutua de Terrassa); SM Jaume Böttcher, M Jimenez Toscano (Barcelona; Hospital del Mar); A Pueyo Ferrer, JJ Puig Galy, S Boveda gonzalez, M Caicedo Toro , I Vives (Barcelona; UCSI Pere Virgili); V Bebia, M Espinosa-Bravo, L Porteiro Mariño, V Alonso Mendoza, A Gil-Moreno, R Blanco-Colino, R Vilallonga, S Gonzalez Suarez, M Maiza, E Espin-Basany, E Espin-Basany, L Pons Pellicé, F Moreira Borim, M Jurado Ruiz, E Guerra-Farfan, J Nuñez (Barcelona; Vall d’Hebron University Hospital); I Villamor , M Arrieta, A Lara, D Escobar (Bilbao; Hospital Universitario de Basurto); B Estraviz, MA Leon Valarezo, A Sainz Lete, JC Zevallos-Quiroz, A Landaluce-Olavarria (Bizkaia; Hospital Urduliz); S Bleda, J De Haro (Brunete, Madrid; Hospital Los Mandronos); ME Zarzosa Martin, S Montolío-Doñate, JA Gutiérrez Vásquez (Burgos; Hospital Santiago Apostol); A Valle Rubio, JL Ramos rodriguez, A García, M Yeh Ahumada, V Jimenez (Getafe; Getafe University Hospital); MV Sosa (Gijón; Hospital de Cabueñes); C Rosas bermudez, M Pelloni, O Benet Muñoz, AC Rahy-Martín (Las Palmas de Gran Canaria; Hospital Universitario de Gran Canaria Doctor Negrín); M Molina Bravo, C López Viloria, LA Suarez Gonzalez, G Gutiérrez Carrillo, S Marcos Contreras (León; Complejo Asistencial Universitario de León); J Delgado Fernandez, I Paredes, A Lagares, AM Castaño-Leon (Madrid; 12 de Octubre University Hospital); SM González Soares, MP Cidón Palacio, M Martín Sánchez, V Domínguez-Prieto (Madrid; Fundación Jimenez Diaz University Hospital); TW Jorgensen, L Marquez (Madrid; Hospital Central de la Cruz Roja San Jose y Santa Adela); D Rivera-Alonso, JL Garcia galocha, JM Muguerza, ME Ossola, J Dziakova (Madrid; Hospital Clinico San Carlos); C Rey Valcarcel, B Quintana-Villamandos, J Rio, M Tousidonis, O Mateo-Sierra (Madrid; Hospital General Universitario Gregorio Marañón); F Mendoza-Moreno, C Vera Mansilla, B Matías-García, N Morales Palacios, E Serrano Yébenes, M Diez Alonso (Madrid; Hospital Universitario Principe de Asturias); FJ Reinoso, S Valderrabano Gonzalez, H Perez-Chrzanowska, A Martínez de Aragón, R De la Oliva , P Serrano Méndez (Madrid; Hospital Universitario la Paz); A Sánchez Gollarte, A Galvan, C Guijarro Moreno, E González , AM Minaya Bravo (Madrid; Hospital del Henares); J Ripollés-Melchor , EM Sáez-Cerezal, M García-Nebreda, JM Martínez-Gómiz, A Abad-Motos (Madrid; Infanta Leonor University Hospital); JC Martín del Olmo, MDP Concejo Cutoli, J Trujillo Díaz, CM Martinez Moreno, J Atienza Herrero, JR Gómez López (Medina del Campo (Valladolid);Hospital Medina del Campo); D García Escudero, R Lax Perez, JA Martínez Alonso, AI Avellaneda Camarena, M Valero Soriano, M Carrasco Prats (Murcia; Hospital General Reina Sofía); J Gil Martínez, PJ Gil Vázquez, A Balaguer Román, A Delegido García, B Gómez Pérez (Murcia; Hospital Universitario Virgen de la Arrixaca); R Diaz Serrano, L Hidalgo Lariz, P Sanchis, DS García García, E Cano-Trigueros (Murcia; Morales Meseguer University Hospital); B Perez-Lozana, A Paz-Aparicio, VR Louçao Prada, M Fernández Mendez, L Varela Rodríguez, LJ García Flórez (Oviedo; Hospital Universitario Central de Asturias (HUCA)); E Colás-Ruiz, MI Valldeperas Hernández, J Fernández Manzano, M Castro Suárez, J Mata (Palma de Mallorca; Hospital Universitario Son Llàtzer); L Muñoz-Bellvis, J Trebol (Salamanca; Complejo Asistencial Universitario de Salamanca); C Cassinello, M Barrionuevo Ramos, C Sanz, J Cardenas-Gomez, A Perez Ferrer (San Sebastian de Los Reyes; Infanta Sofía University Hospital); JA Lizarbe, B Aguinagalde, I López, A Fernandez-Monge, A Beguiristain, J Zabaleta (San Sebastian; Hospital Universitario Donostia); R Da Silva Freitas, R Evangelista Zamora, CÁ Peláez Sánchez, V García Milán, J Jimeno Fraile, R Martín-Láez (Santander; Marqués de Valdecilla University Hospital); A López De Fernández ,  JR Oliver Guillen (Soria; Hospital Santa Bárbara); E Julià-Verdaguer, A Caro, A Martínez, J Ferreres Serafini, M Vallve-Bernal, R Jorba (Tarragona; Hospital Universitari De Tarragona Joan XXIII); I López Sánchez, K Aghababyan, O Gil-Albarova, P Renovell Ferrer, CS Romero Garcia (Valencia; Consorcio Hospital General Universitario); I Miranda, J Sanz Romera, C Chiappe, R Valverde-Vázquez, J Domenech Fernández (Valencia; Hospital Arnau de Vilanova); R Badenes, L Pérez Santiago, R Gadea Mateo, E Muñoz Sornosa, D Moro-Valdezate, J Ortega Serrano (Valencia; Hospital Clínico Universitario de Valencia); S Gomez-Abril, MT Torres Sánchez, JC Bernal-Sprekelsen (Valencia; Hospital Universitario Doctor Peset); V Garcia-Virto, M Pascual Samaniego, J Beltrán de Heredia, B De Andrés-Asenjo (Valladolid; Hospital Clínico Universitario de Valladolid); MT Fernández Martín , M Bailón, C Aldecoa, D Pacheco Sánchez, E Asensio Díaz, FJ Tejero-Pintor (Valladolid; Hospital Universitario Río Hortega); M Sánchez-Rubio, I Herrero, D Garcia López de Goicoechea, M Camuera, A Vazquez Melero (Vitoria-Gasteiz; Hospital Universitario Araba); VM Borrego Estella, R Carramiñana Nuño, L Medina Mora , MD Arribas Del Amo (Zaragoza; Hospital Clinico Universitario Zaragoza); G Pola Bandres, S Visiedo Sanchez, E Delgado Blanco, J Gil-Rodriguez, E Carnicer Escusol, L Sánchez Blasco (Zaragoza; Hospital General de la Defensa); C Gracia-Roche, T Gimenez Maurel, I Gascon Ferrer, V Duque Mallén (Zaragoza; Hospital Universitario Miguel Servet). |
| Sri Lanka: S Srishankar, D Wanigasekera, KJ Senanayake (Anuradhapura; Teaching Hospital Anuradhapura); ADTS Athukorala, T Gamage (Colombo; Lady Ridgeway Hospital for Children); DN Samarasekera, S Sivaganesh, S Seneviratne, D Subasinghe, S Garusinghe, D Wickramasinghe (Colombo; National Hospital of Sri Lanka); B Balagobi, S Gobishangar, V Satchithanantham (Jaffna; Teaching Hospital, Jaffna); L Dasanayake, M Riehan, C Baddegama , D Wickramasooriya (Mahamodara, Galle; Teaching Hospital, Mahamodara, Galle); DMCS Jayasundara, D Ilukpitiya, A Jayawardane (Sri Lanka; De Soysa Hospital for Women); S Jayasekara, R Perera, D Rodrigo, R Wickramarachchi , W Wijenayake (Werahera; University Hospital, Kotelawala Defence University). |
| Sudan: HKS Hamid, S Galal Eldin (Khartoum; Al-Moalem Medical City); E Adel Hamdoun Aziz, I Adel (Khartoum; Bashair Teaching Hospital); A Abdelbagi, MAA Adam, M Hassan, S Mohamed , N Hilal (Khartoum; Ibn Sina Specialized Hospital); M Ahmed Elamin Elnour (Khartoum; Khartoum Teaching Hospital); R Sabri, M Hajalamin, O Ahmed, E Hasan, AA Adam (Khartoum; Omdurman Teaching Hospital); T Fadalla (Khartoum; Ribat university hospital);  HA Fadlalmola (Khartoum; Soba University Hospital); W Abdelwahab, H Mustafa , K Ali, KAD Gasmalla (Wad Madani; University of Gezira Hospital). |
| Sweden: L Pieteris, Y Saeed, S Elmarimi, M Saeed, M Jawad (Kristianstad; Central Hospital in Kristianstad); A Oscarsson, L De Geer, H Didriksson, C Jonsson, H Andersson, M Chew (Linköping; Linköping University Hospital); J Malmstedt, A Älgå (Stockholm; South General Hospital). |
| Switzerland: GJ Kocher, R Saadeh, T Manogaran, T Erdil (BERN; Inselspital, Bern University Hospital, University of Bern); O Kollmar, M Bolli, A Lalos, A Tampakis (Basel; University Hospital Basel); A Tessitore, D La Regina, A Cianfarani, S Cafarotti , F Mongelli (Bellinzona; EOC Ospedale Regionale di Bellinzona e Valli); F Hauswirth, E Sebestyen, M Muller, D Salinovic, P Probst (Frauenfeld; Spital Thurgau AG); M Chevallay, F Ris, C Brasset, A Litchinko, E Gialamas (Geneva; Geneva University Hospitals); R Galli (Liestal; Kantonsspital Baselland Liestal); M Puglisi, M Di Giuseppe (Locarno; EOC Ospedale Regionale di Locarno); ML Gasparri, A Papadia (Lugano; EOC Ospedale Regionale di Lugano - Civico); V Kremo, J Metzger, A Scheiwiller, J Gass (Luzern; Luzerner Kantonsspital); F Castronovo, A Ottone, V Zacesta, A Braga (Mendrisio; EOC Ospedale Regionale di Mendrisio); M Racine, A Saadi, AX Wiseman, Y Sprunger, M Sauvain (Neuchatel; Hopital de Pourtales); M Oberlechner, P Kempter, L Walther, T Steffen (St. Gallen; Kantonsspital St. Gallen); S Happ, S Biber, M Benoit (Visp; Spitalzentrum Oberwallis); G Peros, TU Müller, P Müller, M Giardini, M Adamina (Winterthur; Kantonsspital Winterthur); A Müller, F Kalt, G Aeby, JMA Toti, MA Schneider (Zürich; Universitätsspital). |
| Syrian Arab Republic: A Ghazal, R Masri, H Zayat, AA Kayali (Aleppo; Aleppo Private Hospital); Y Nerabani, MN Sawas, A Khaled, I Arnaout, M Aldaher (Aleppo; St Louis Hospital); M Balouli, H Al Houri, A Alhouri, S Abbas, B Ahmad (Damascus; Al-Mouwasat University Hospital); H Othman, A Othman, Y As, O Alazki (Latakia; National Hospital); A Alloush, N Ali, N Hassan, D Sayed Ahmad, M Oukan, A Hammed (Latakia; Tishreen University Hospital); MG Attoum, R Hajjouz, R Attoum (Tartus; Al Bassel Hospital). |
| Trinidad and Tobago: S Chackan, S Charles (San Fernando; San Fernando General Hospital). |
| Tunisia: M Baccar, S Toumi, B Gafsi (Monastir; University Hospital Fatouma Bourguiba); I Gharbi, A Letaief, MA El Ghali (Sousse; Farhat Hatched Hospital); M Ben Othmen, A Houssem (Sousse;  Sahloul Hospital). |
| Turkey: Z Çetinkaya, DS Korkmaz, E Durmuş, D Yavuz, ÜC Köksoy (ANKARA; UFUK ÜNİVERSİTESİ TIP FAKÜLTESİ DR.RIDVAN EGE SAĞLIK ARAŞTIRMA UYGULAMA MERKEZİ HASTANESİ); M Sertkaya (Adıyaman; Özel Adıyaman Park Hospital); EB Bostanci, E Pişkin, MK Colakoglu, V Oter (Ankara; Ankara CITY Hospital); MA Koç, AS Çalış, Ş Ersöz, I Gecim, C Akyol (Ankara; Ankara University Medical School); MM Ozmen, E Gundogdu, M Moran, CE Guldogan (Ankara; Liv Hospital); L Doğan, S Sarıdemir, OC Güler (Ankara; SBÜ Ankara Onkoloji Eğitim ve Araştırma Hastanesi); M Süleyman, AM Barlas, MR Pekcici, A Sadioğlu, S Erel (Ankara; Saglik Bilimleri University); S Doğan, B Yağmur Aras, NU Dogan (Antalya; Akdeniz University Hospital); EI Turhan, M Nalbant , N Unal Odabas, D Yigit, O Yalkın (Bursa; Bursa City Hospital); U Özgen, H Sungurtekin (Denizli; Pamukkale University School of Medicine); M Ömür, Ç Bayram, E Şenödeyici, İ Yavuz, A Ulkucu (Edirne; Trakya University Hospital); RK Liman, M Uzun, A Kut, B Yigit (Elazig; Elazig Fethi Sekin City Hospital); Y Aydin, A Eroglu, A Dostbil, AB Ulas (Erzurum; Ataturk University School of Medicine, Research and Training Hospital); B Gurbuz, U Can, F Artukoglu, D Buğra, S Zenger (Istanbul; American Hospital); YE Aktimur, A Barcin, S Meriç, N Bugdayci , Aİ Sayar, Y Altinel (Istanbul; Bagcilar Research And Training Hospital); ÖP Zanbak Mutlu (Istanbul; Bahçelievler State Hospital); G Olgac, B Kaymak, E Aydın, YS Kömek, R Hasanov, C Tatar, UO Idiz (Istanbul; Istanbul Education and Research Hospital); L Aydemir, D Altun, C Sen (Istanbul; Istanbul University - İstanbul Faculty of Medicine); SS Uludağ, MF Ozcelik, AK Zengin, E Kose, A Turna, E Erginöz (Istanbul; Istanbul universty - Cerrahpaşa Medical faculty); MA Bozkurt, E Somuncu, E Bozdağ, S Yilmaz, Y Kara, A Özcan (Istanbul; Kanuni Sultan Suleyman Training and Research Hospital); R Sarı, E Memişoğlu, D Copur, R Demirhan (Istanbul; Kartal Dr. Lutfi Kirdar Training and Research Hospital); D Vatansever, B Giray, E Balik, İH Özata, E Bozkurt, C Taskiran (Istanbul; Koç University Medical School); B Canbay Torun (Istanbul; Liv Hospital Ulus); AC Dural, E Sivrikoz (Istanbul; Liv Hospital Vadistanbul); A Akmercan, A Saracoglu, M Ergenç, TK Uprak, MU Ugurlu (Istanbul; Marmara University, School of Medicine); S Sayır, S Tozdogan, AN Sanli (Istanbul; Silivri State Hospital); AE Tufan, S Ömeroğlu, E Baran (Istanbul; Sisli Hamidiye Etfal Training and Research Hospital); M Kalın, HS Ulgur, EF Kirkan , HK Karakullukcu (Istanbul; University of Health Science Umraniye Education and Research Hospital); H Ozsahin, B Citgez (Istanbul; Uskudar University Faculty of Medicine, Memorial Hospital); YK Şen, M Yildirim, İ Demir, Ş Çalık, B Calik (Izmir; University of Health Sciences Izmir Bozyaka Training and Research Hospital); ND Bulut Yüksel, H Pülat (Mersin; Mersin City Training and Research Hospital); R Şahin (Rize; Recep Tayyip Erdogan University); F Altintoprak, AT Harmantepe, M Coşkun, M Doğangün, E Gonullu (Sakarya; Sakarya Faculty Of Medicine); K Yemez, MS Uyanik, S Polat, M Candan, AB Ciftci, E Colak, GO Kucuk (Samsun; Samsun University Samsun Training and Research Hospital); ME Reis, K Eyuboglu, O Dumlu, AN Yuzgec, A Guner (Trabzon; Karadeniz Technical University Farabi Hospital); H Balbaloglu, I Tasdoven, G Karadeniz Cakmak (Zonguldak; Zonguldak Bulent Ecevit University School of Medicine Research and Training Hospital); T Kaya, G Kilinc Tuncer, K Tuncer, K Teker, S Demirli Atici (İzmir; University of Health Sciences Tepecik Training and Research Hospital). |
| Uganda: K Japheth, H Lule (Kigumba; Kiryandongo Hospital). |
| Ukraine: M Paranyak (Lviv; Lviv Clinical Emergency Care Hospital). |
| United Arab Emirates: VS Basappanavar (Ajman; Sheikh Khalifa Medical City Ajman); S Elnikety, H Mohamed, N Alkaabi, N Baniyas, K Abdel-Galil (Al-Ain, Abu Dhabi; Tawam Johns Hopkins Hospital); A Mohamed, S Kailasam sivamurthy, AAA Nugud Abd Alwahab Aljafary, A Jacob, A Al-Fraihat, S Alshryda, I Majid (Dubai; Al Jalila Children’s Speciality Hospital); FMA Abbas (Dubai; Dubai Hospital); MA Tahlak, SA George Varayannoor, D Mohammed (Dubai; Latifa Women and Children Hospital); N Abdulla , M Alshamsi, R Kundra (Dubai; Mediclinic Parkview Hospital); Z Mohamed, A Msaddi, ALR Michael (Dubai; Neurospinal Hospital); B Elyafawi, H Khansaheb, AA Mohammed, Y Wardeh, H Alsaadi (Dubai; Rashid Hospital). |
| United Kingdom: B Dhinsa, J Relwani, FC Anazor (Ashford; William Harvey Hospital); P Cook, M Perry, A Purohit, N Bechar, M Greenhalgh (Ashton-under-Lyne; Tameside and Glossop Integrated Care NHSFT); S Jordan, Z Elahi, A Baldwin (Aylesbury; Stoke Mandeville, Wycombe General); A Ng, S Bhattacharya, A Ali, P Ishak (Ayr; University Hospital Ayr); A Womersley (Banbury; Horton Hospital); SJ Ahmad, MW Saqib, R Morris, AK Lala, CS Ong (Bangor, North Wales; Ysbyty Gwynedd); C Chandrakumar, TD Stringfellow, M Lebe (Barnet; Barnet General Hospital); J Attwood (Barnsley; Barnsley Hospital NHS Foundation Trust); R Chhabra, P Patel (Barrow in Furness; Furness General Hospital); M Karbowiak, B Doughty, CHK Patel, D D’Souza, N Ponugoti (Basingstoke; Basingstoke and North Hampshire Hospital); NOT Rees, J Cheuk, S Thornton, F Shekleton (Bath; Royal United Hospital Bath); A Mian, S Khan, A Sheikh, K Rajaratnam, F Georgiades (Bedford; Bedford Hospital); M Jones, T Oyebanji, J Strickland, N Fundano, F Aljanadi (Belfast; Royal Victoria Hospital); S Raveendran, S Azam, N Mirtorabi, M Hoque, O Tucker, N Chidumije (Birmingham; Heartlands Hospital); K Kayani, A Jackowski, Y Al-Najjar, H Mann (Birmingham; Queen Elizabeth Hospital Birmingham); F Ghini, M Albendary, AYY Mohamedahmed, P Bhattacharya (Birmingham; Sandwell General Hospital); P Sequeiros, M Mohsin, A Mehmood, J Ali, M Bashir , MH Siddique, S Ikram (Birmingham; Solihull Hospital); A Qureshi, S Ahmad, S Hadi, R Jugdey, UA Halim (Blackburn; Royal Blackburn Hospital); S Kirmani, A Northey, M Abrar, S Adhya, T Chituku, A Tennakoon (Boston; Pilgrim Hospital); HL Morley, F Khaliq, R Saghir, N Thomas, D Waugh (Bradford; Bradford Royal Infirmary); D Peristeri, A Athanasiou (Brighton; Royal Sussex County Hospital); B Martin, M Baquedano, M Caputo, F Rapetto, CJ Bradshaw (Bristol; Bristol Royal Hospital for Children); J Ackah, N Blencowe (Bristol; Bristol Royal Infirmary); L Howse, N Grundy, B Smith, A Biggs, H Claireaux (Camberley; Frimley Health NHS FT - Frimley Park); P Hutchinson, E Irune, A Hardy, S Meghji, B Fish, A Mansour, J Armitage, A Colquhoun, I Chipurovski, AMAM Ahmed, GD Stewart, N Havers, M Abou-Abdallah, O Baker, A Marton, J Ashcroft, RJ Davies, B Zacharia, S Liau, CY Tan, L Wang, W Khan, KM Seah, A Durrani, J Roszpopa, M Ghobrial, C Currow, ANS Silva, C Patient, N Russell, N Simon (Cambridge; Addenbrooke’s Hospital); EC Pama, D Samaraweera, S Mushtaq, N Asemota, MI Tahmid, A Coonar (Cambridge; Royal Papworth Hospital); SZY Ooi, P Pemmasani, G Tahhan, S Chan, T Combellack (Cardiff; University Hospital Llandough); GS Davies, D Manson, K Lam, G Lee, R Luckwell (Cardiff; University Hospital of Wales); A Mannan, F Ayeni, S Mannan (Carlisle; Cumberland Infirmary); D Myatt, A Barclay, H Bakhit, S Dodd, R Ashour, S Sawalha (Chester; Countess of Chester Hospital); S Sexton, A Rayner, S Federer (Chichester; St Richard’s Hospital); S Rout, S Towell, M Ghoneim, M Elsabbagh, A Walid Mohamed, D Baskaran, H Elfeky (Colchester; Colchester Hospital University); W Campbell, R Coulson, M Carvalho, L Mcguigan (Coleraine; Causeway Hospital); A Boulton, A Stephens, V Kolaityte (Coventry; University Hospitals Coventry and Warwickshire NHS Trust); N Gokhare Viswanath, W Al-Khyatt, S Fendius, NA Kerr, F Hurasha, M Abdalla, A Ebrahim, A Bateman (Derby; Royal Derby Hospital); L Thompson, JR Apollos, M Zafar (Dumfries; Dumfries and Galloway Royal Infirmary); E How Hong, CH Leong, H Gray, F Ahmed, T Brow, K Ragupathy, SC Khaw, J Manickavasagam (Dundee; Ninewells Hospital); CE Ng, A Nada, S Supparamaniam, S Wallace-King, S Arumugam (Durham; University Hospital North Durham); O Impey, P Cullis (Edinburgh; Royal Hospital for Children and Young People); RJ Skipworth, E Harrison, V Zamvar, S Davison (Edinburgh; Royal Infirmary of Edinburgh); O El-Koubani, J Ayers, H Paterson, T Watcyn-Jones, A Laird (Edinburgh; Western General Hospital); M Waroński, Y Lim, A Kushairi, F McDermott, J Phillips (Exeter; Royal Devon and Exeter Hospital); E Spurring, CEH Fang, S Newman, E Waller, G Faulkner (Farnworth; Royal Bolton Hospital); O Bajomo, M Christodoulou, A Owusu-Addo, H Thomas (Gillingham; Medway hospital); W Elbakbak, E Peng, M Thomas, K Shaikhrezai, G Gradinariu (Glasgow; Golden Jubilee National Hospital); G Bhatta, W Stupalkowska, S Rai, A Hinton, M Bogdan (Great Yarmouth; James Paget Univeristy NHS Foundation Trust Hospital); H Assalaarachchi, TK Madhuri (Guildford; Royal Surrey County Hospital); J Appleyard, W Ding, A Potts, C Vosinakis, R Maguire, S Allen, C McNaught, SP Hogston, A Gyori, C Reynoldson, AR Royson, R Turner, S Ippoliti (Harrogate; Harrogate District Hospital); J Watfah, CA Leo, J Warusavitarne (Harrow, London; St Mark’s Hospital); S Kapoor, J Jeater, D Brown, L Brown Fumeau, C Chiang (Hastings; East Sussex Healthcare (Conquest hospital and Eastbourne District General Hospital)); B Hama, M Zaman, K Madhvani (Huddersfield; Huddersfield Royal Infirmary); AS Elhalawany, C Frew, F Sneddon, D Da Luz, M Mohamed (Inverness; Raigmore Hospital Inverness); G Karagiannidis (Ipswich; Ipswich Hospital); S Hill, C Walsh, S Warburton, M Sange, M Higgins (Kent; Darent Valley Hospital); M Allison, N Manimaran, J Lucocq, J McKay (Kirkcaldy; Victoria Hospital Kirkcaldy); E Gardner, V Sharma, Z Slevin, M Wilson (Larbert; Forth Valley Royal Hospital); C Moriarty, L White , G Toogood, B Huntly, R Wade, A Peckham-Cooper, P Gurung, T Griffiths, J Aldoori, AJ Cockbain, S Louette, L Scott, S Fraser (Leeds; St James’s University Hospital Leeds); ML Collins, J Shepherd, A Thakker, K Brahmbhatt (Leicester; Leicester Royal Infirmary); D Worku (Lincoln; Lincoln County Hospital); A Santini, SFM Gillani, A Sud, Z Shakoor, R Lunevicius, A Schache, C Loh (Liverpool; Aintree University Hospital); F Bassily, M Shaw, B Kirmani (Liverpool; Liverpool Heart and Chest Hospital); T Elmoslemany, R Zakaria, CP Millward, M Jenkinson (Liverpool; The Walton Centre NHS Foundation Trust); L Lancerotto, M Carvill, F Brzeszczyński (Livingston; St Johns Hospital); J Moreau, S Sheehan, V Evans, SN Yew, T Szakmany, C Spiers (Llanyravon, Cwmbran; The Grange University Hospital); F Soggiu, H Sheth, A Alamin, S Froghi (London; Ealing Hospital); J Ploski, D Spalding, M Pai, C Fotopoulou, M Fehervari (London; Hammersmith Hospital); A Vasireddy, EH Abd Wahab, C Gibson, L Nordin, E Bagouri (London; King’s College Hospital); M Bence, P Kapsampelis, C Obasi, F Gerges, S Gillani, I Gerogiannis (London; Kingston); T Singhal, TY Selvamani, M Bhatia (London; Princess Royal University Hospital); T Yeoh, R Dyke, D Leong, E Headon, YC Tan (London; Queen Elizabeth Hospital, Woolwich); JM Pollok, S Staubli, S Pericleous (London; Royal Free Hospital); JD Jayasinghe , D Prce, MT Berlanga Rojas, Y Benallal, MA Thaha, H Lu, S Dawson-Bowling, A Carlos, S Hanna, S Hammouche (London; Royal London Hospital); G Santhirakumaran, J Selvakumar, J Smelt (London; St George’s Hospital); P Seyed-Safi, T MacKinnon, A Beer, T Howard, A Singh, A Liddle, E Wilson, S Chadha, H Hodgson, J Cann (London; St Mary’s Hospital); HM Kocher (London; The London Clinic); H Marcus, C Hill, S Williams, H Layard Horsfall (London; The National Hospital for Neurology and Neurosurgery); K Sharma, N Efthymiou , C Parmar (London; The Whittington Hospital); A Ng, S Patel, K Patel, N Wang, A Nathan, J Rassam (London; University College London Hospital); S Ahmed, N Ul ain, R Talwar, L Jones, E Salau, S Ebrahim (Luton; Luton and Dunstable University Hospital); M Hassan, M Riad, D Balasubramaniam (Maidstone; Tunbridge Wells Hospital); H Greenlee, H Moxon, K Bhatia (Manchester; Manchester Royal Infirmary); R Norawat, S Sathyaprasad, S Desbruslais, S Bansal (Manchester; St Marys); M Smith, B Winter-Roach, B Decruze, AM Floreskou, E Myriokefalitaki (Manchester; The Christie Hospital); C Carey, N Akhavan Fomani, S Michael, S Lodhi (Manchester; Wythenshawe Hospital); L Kottam, H Elamin Ahmed, J Norman, K ElSanhoury, R Walker, A Rangan (Middlesbrough; James Cook University Hospital); O Ryska, E Davies, T Raymond, P Hawkin, T Tay (Morecambe; Royal Lancaster Infirmary); J Hawkyard, F Barbour, TR Jones, S Pandanaboyana, JS Hammond (Newcastle upon Tyne; Newcastle Upon Tyne Hospitals NHS Foundation Trust); Y Mahmoud, D Townshend, A Aujayeb (North Shields; Northumbria NHS Hospital Trust); O Cottle, SO Nwose (Nottingham; Nottingham City Hospital); D Humes, J Jones, J Couch, A Dias Samarawickrama Yapa (Nottingham; Queens Medical Centre); L Ismail, M Goricar, H Soleymani majd (Oxford; Churchill Hospital); K Shah, R Taberham, S Shrestha, H Chaudry (Oxford; John Radcliffe Hospital); C Bretherton, SR Small (Oxford; Nuffield Orthopaedic Centre); M Awadallah, S Yadu, B Davies, O Johnson, I Liew (Peterborough; Peterborough City Hospital); A Ahmed, SK Kallikere lakshmana , S Lakpriya (Plymouth; Derriford Hospital); O Nasim, A Durrani, O Ahmed, J Brockwell, Q Mustafa, I Pilkington (Poole; Poole Hospital); S Stefan, J Bapty, R Harvitkar, J Khan, SKC Toh, M Noor (Portsmouth; Queen Alexandra Hospital); F Colombo, H Maye , R Ravindranath Nambiar, J Crawford, O Pathmanaban (Salford; Salford Royal Hospital); AE Ward, O Rominiyi, A Bacon, R Nair, A Adamec, J Edwards (Sheffield; Sheffield Teaching Hospital NHS Foundation Trust); J El Kafsi, S Bhagat, S Mastoridis (Slough; Frimley Health NHS FT - Wexham Park);  M Rezacova (Southampton; Southampton General Hospital); H Raja, B Panamarenko, J Wong, E Laurent (Southend; Southend University Hospital); AW Thakur, C Sangani, W Lloyd, N Ahuja, K Iyengar (Southport; Southport and Ormskirk NHS Trust); T Walker, J Pearce, S Keogh-bootland, C Brown (St.Helier; Jersey General Hospital); S Niyas, S Tewari, Z Mahmood, S Adegbola, S Karim (Stevenage; Lister Hospital); T Havenhand, A Ramesh, S Bromage, Y Zhang, M Condon, D Johnson (Stockport; Stepping Hill Hospital); WC Soon, S Saifuddin, V Shah, K Kapur, B Sachdev (Stoke-on-Trent; Royal Stoke University Hospital); D Jochems, E Sultana, P George Pandeth, L Roberts (Sutton Coldfield; Good Hope Hospital); A Tolat, P Kaur , M Dube (Sutton-in-Ashfield; Kings Mill Hospital); M Hanson, O Beaumont, C Grieco, R Dru, M Hollyman (Taunton; Musgrove Park Hospital); N Ismail (Truro; Royal Cornwall Hospital); N Anyaugo, A Skaria, A White, L Barnard, N Chandratreya (Weston-super-Mare; Weston General Hospital); A Rajpura, T Board, N Jagadeesh, V Thakker (Wigan; Wrightington, Wigan & Leigh NHS Foundation Trust); CT Chuah, M Dyer, MFA Kamarizan (Wrexham; Wrexham Maelor Hospital). |
| United States: S Lai, J Lee, S Vemuru, L Leonard, A McCranie, M Thomas (Aurora; University of Colorado Anschutz Medical Campus (CU Anschutz)); I Mannoh, E Etchill (Baltimore, MD; Johns Hopkins Hospital); S Kavic, J Pearl, J Kang, F Fang, H Abdou (Baltimore; University of Maryland Medical Center Midtown); K Vogel, S Wason, J Wang (Boston, MA; Boston Medical Center); A Renne, JA Proaño-Zamudio, D Argandykov, H Kaafarani (Boston, MA; Massachusetts General Hospital); G Brat, B Hall, J Etra, AC Pfaff (Boston; Beth Israel Deaconess Medical Center); JB Finkelstein, A See, M Halpin, D Mathieu, JG Meara, SK Bridges (Boston; Boston Children’s Hospital); S Hirji, C Raut, Z Cooper (Boston; Brigham and Women’s Hospital); C Burks, A Zhou, M Naunheim (Boston; Massachusetts Eye and Ear); C Reinke, L McCarthy, D Manning, R Perkins, S Ross (Charlotte; Atrium Health Carolinas Medical Center); E Ekrami, E Kutlu Yalcin, T Bowman, D Elder, L Liu, A Turan (Cleveland, Ohio; Cleveland Clinic Foundation); T Schroeppel, C Decker, Z Stillman, EA Hennessy, J Rodriquez (Colorado Springs; Memorial Hospital); D Moris, S Kesseli, ES Hwang, D Schaps, HE Rice (Durham, NC; Duke University Medical Center); D Bavishi, S Tariq, M Matuszczak, A Upton, E Sorial, S Khan (Houston; Children’s Memorial Hermann Hospital); N Bhutiani, A Hassan, C Butler, C Scally, CL Roland (Houston; University of Texas MD Anderson Cancer Center); K McKenzie, S Konda, A Ganta, RJ Robitsek (Jamaica; Jamaica Hospital); H Many, A Cavalea, R Savoy , B Daley (Knoxville; University of Tennessee Medical Center); H Hardgrave, D Krinock, J Nigh, G Klutts, T Osborn, E Giorgakis, J Laryea (Little Rock; University of Arkansas for Medical Sciences); L Hua-Feng, RB Cameron (Los Angeles; Ronald Reagan UCLA Medical Center); J Hadaya, C Juillard, D Beswick, RM Mabeza, P Benharash (Los Angeles; University of California Los Angeles); J Dunn, L Britton - Zier, H Breda Pessetti, J Schmidt (Loveland, Colorado; Medical Center of the Rockies); E Kim , K Shrestha, A Valgarðsson (Lubbock, Texas; University Medical Center Lubbock); V Hsiao, J Murtha, M Argo, A Wiener, T Diehl, SN Zafar (Madison; University of Wisconsin); W Ramsey, N Goel, C O’Neil (Miami, FL; University of Miami Hospital); R Morris, S Sheriff, N Verhagen, M Flitcroft, A Kothari (Milwaukee, WI; Medical College of Wisconsin); RM Nygaard, F Endorf (Minneapolis, Minnesota; Hennepin Healthcare); A Hoang, C Adams, D Roberts, S Chotai, C Walters, A Lorinc (Nashville; Vanderbilt University Medical Center); R Tripathi, SMH Jeoffrey, A Nasir, H Ellauzi, A Kalyanasundaram (New Haven, CT; Yale New Haven Hospital); M Torres, H Hakmi, T Brasileiro Silva Pacheco, C Brathwaite, P Petrone (New York, NY; NYU Langone Medical Center); D Alqunaibit, C Graham, D Feingold, A Liveris (New York; New York City Health and Hospitals - Jacobi Medical Center); B Leoce, Y Abedin, F Elgammal, G Tsui, N Glass (Newark, NJ; The University Hospital); A Renne, A Dorken-Gallastegi, M Bokenkamp, A Gebran, C Paranjape (Newton, MA; Newton Wellesley Hospital); J Adams, L Weber Graeff, R Mcnulty (Sacramento; UC Davis Medical Center); R Zerna Encalada, A Choudhry (Syracuse; SUNY Upstate University Hospital); E Shaykhinurov, A Canonico, E Heinz, I Ibi, A Vincent (Washington; The George Washington University Hospital); Z Garoufalia, R Gefen, MR Freund, E Silva-Alvarenga, SD Wexner (Weston; Cleveland Clinic Florida); V Mangaroliya (Wilkes-Barre, Pennsylvania; Geisinger Wyoming Valley Medical Center); AS Munoz Abraham (Worcester, MA; UMass Memorial Hospital). |
| Yemen, Rep.: F Alhajami, B Alshaikh, M Al-Dhaheri, W Ahmed, M Al-Shehari, S Shream (Sana’a; Al-Thawra Modern General Hospital). |

**CovidSurg-2 authors**

#### Operations Committee (alphabetical):

| Waheed-Ul-Rahman Ahmed, Leah Argus, Alasdair Ball, Aneel Bhangu, Edward P Bywater, Ruth Blanco-Colino, Amanpreet Brar, Daoud Chaudhry, Brett E Dawson, Irani Duran, Muhammed Elhadi, James C Glasbey, Rohan R Gujjuri, Conor S Jones, Ewen M Harrison, Sivesh K Kamarajah, James M Keatley, Samuel Lawday, Elizabeth Li, Harvinder Mann, Ella J Marson, Kenneth A Mclean, Dmitri Nepogodiev, Lisa Norman, Riinu Ots, Oumaima Outani, Maria Picciochi, Irène Santos, Catherine Shaw, Joana FF Simoes, Elliott H Taylor, Isobel M Trout, Chris Varghese, Mary L Venn, William Xu. |
| --- |

#### Dissemination Committee (alphabetical by country):

| **Albania**: Irida Dajti, Arben Gjata; **Algeria**: Salah Eddine Oussama Kacimi; **Argentina**: Luis Boccalatte, Maria Marta Modolo; **Australia**: Daniel Cox, Peter Pockney, Philip Townend; **Austria**: Felix Aigner, Irmgard Elisabeth Kronberger; **Azerbaijan**: Elgun Samadov; **Bahrain**: Amer Alderazi; **Bangladesh**: Kamral Hossain; **Barbados**: Greg Padmore; **Belgium**: Gabrielle van Ramshorst; **Benin**: Ismaïl Lawani; **Bosnia and Herzegovina**: Anis Cerovac, Samir Delibegovic; **Brazil**: Glauco Baiocchi, Gustavo Mendonça Ataíde Gomes, Igor Lima Buarque; **Bulgaria**: Muhammad Gohar, Mihail Slavchev; **Cameroon**: Chukwuemeka Nwegbu; **Canada**: Arnav Agarwal, Amanpreet Brar, Janet Martin, Joshua Ng-Kamstra; **Chile**: Maria Marta Modolo, Maricarmen Olivos; **China**: Wenhui Lou, Dong-Lin Ren; **Colombia**: Jose Andres Calvache, Carlos J- Perez Rivera; **Croatia**: Ana Danic Hadzibegovic, Tomislav Kopjar, Jakov Mihanovic; **Cuba**: Pablo Mijahil Avilés Jiménez; **Cyprus**: Nikolaos Gouvas; **Czech Republic**: Jaroslav Klat, René Novysedlák; **Democratic Republic of the Congo**: Nicolas Amisi; **Denmark**: Peter Christensen, Alaa El-Hussuna; **Dominican Republic**: Sylvia Batista; **Ecuador**: Eddy Lincango-Naranjo; **Egypt**: Sameh Emile; **El Salvador**: Danilo Alfonso Arévalo Sandoval; **Ethiopia**: Hailu Dhufera, Samuel Hailu, Mengistu G Mengesha; **Finland**: Joonas H Kauppila; **France**: Alexis P Arnaud; **Georgia**: Zaza Demetrashvili; **Germany**: Markus Albertsmeier, Hans Lederhuber, Markus W. Löffler; **Ghana**: Daniel Kwesi Acquah, Bernard Ofori, Stephen Tabiri; **Greece**: Symeon Metallidis, Georgios Tsoulfas; **Guatemala**: Maria-Lorena Aguilera-Arevalo, Gustavo Recinos; **Hungary**: Tamás Mersich, Dániel Wettstein; **India**: Dhruva Ghosh; **Indonesia**: Gabriele Kembuan; **Iran**: Peiman Brouki Milan, Mohammad Hossein Khosravi, Masoud Mozafari; **Iraq**: Ahmed Hilmi; **Ireland**: Helen Mohan; **Israel**: Oded Zmora; **Italy**: Gaetano Gallo, Francesco Pata, Gianluca Pellino; **Japan**: Yuki Fujimoto, Naoto Kuroda, Sohei Satoi; **Jordan**: Mohamad K. Abou Chaar, Faris Ayasra; **Kazakhstan**: Ildar Fakhradiyev; **Kenya**: Intisar Hisham Said Hamdun; **Korea, Republic (South)**: Jang Jin-Young; **Kuwait**: Mohammad Jamal; **Lebanon**: Lina Karout; **Libya**: Muhammed Elhadi; **Lithuania**: Aiste Gulla; **Madagascar**: Fanjandrainy Rasoaherinomenjanahary, Luc Hervé Samison; **Malaysia**: April Camilla Roslani; **Mexico**: Iran Irani Durán Sánchez, Diana Samantha Gonzalez, Laura Martinez, María José Martínez, Alejandra Nayen, Antonio Ramos-De la Medina; **Mongolia**: Jade Nunez; **Morocco**: Oumaima Outani; **Namibia**: Pueya Rashid Nashidengo; **Nepal**: Rakesh Shah, Ashish Lal Shrestha; **Netherlands**: Pascal Jonker, Schelto Kruijff, Milou Noltes, Pieter Steinkamp; **New Zealand**: Chris Varghese, Deborah Wright; **Nigeria**: Lukman Abdur-Rahman, Adesoji Ademuyiwa, Adewale Adisa, Babatunde Osinaike, Justina Seyi-Olajide, Omolara Williams, Emmanuel Williams; **North Macedonia**: Sofija Pejkova; **Oman**: Zainab Al Balushi; **Pakistan**: Ahmad Uzair Qureshi, Raza Sayyed; **Palestine**: Mustafa Abo Mohsen, Sadi A. Abukhalaf; **Panama**: Moises Cukier; **Paraguay**: Hugo Gomez-Fernandez; **Peru**: Sebastian Shu Yip, Ximena Paola Vasquez Ojeda; **Philippines**: Marie Dione Sacdalan; **Poland**: Piotr Major; **Portugal**: José Azevedo, Miguel F. Cunha, Irène Santos; **Qatar**: Ahmad Zarour; **Romania**: Eduard-Alexandru Bonci, Ionut Negoi; **Russia**: Sergey Efetov, Viktor Kochetkov, Andrey Litvin; **Rwanda**: Jc Allen Ingabire, Georges Bucyibaruta, Ntirenganya Faustin, Sosthene Habumuremyi, Alphonsine Imanishimwe, Haragirimana Jean de Dieu, Emmanuel Munyaneza, Isaie Ncogoza; **Saudi Arabia**: Ehab Alameer; **Senegal**: Abdourahmane Ndong; **Serbia**: Dejan Radenkovic; **Singapore**: Min Hoe Chew, Frederick Koh, James Ngu; **Slovakia**: Arpád Panyko; **Slovenia**: Uros Bele, Jurij Aleš Košir; **Somalia**: Hassan Daoud; **Spain**: Ruth Blanco-Colino, Ana Maria Minaya Bravo; **Sri Lanka**: Umesh Jayarajah, Dakshitha Wickramasinghe; **Sudan**: Mohammed Elmujtba Adam Essa Adam; **Sweden**: Martin Rutegård, Malin Sund; **Switzerland**: Michel Adamina, Eleftherios Gialamas, Karoline Horisberger; **Syria**: Muhammad Alshaar; **Taiwan**: Abel Huang; **Thailand**: Varut Lohsiriwat; **Trinidad and Tobago**: Shane Charles; **Tunisia**: Haithem Jlassi; **Turkey**: Arda Isik, Sezai Leventoğlu; **Uganda**: Hervé Monka Lekuya, Hervé Monka Lekuya, Herman Lule; **Ukraine**: Slava Kopetskyi; **United Arab Emirates**: Hayder Alsaadi, Sattar Alshryda; **United States**: Osaid Alser, Brittany Bankhead-Kendall, Kerry Breen, Haytham Kaafarani, Hassan Mashbari; **Uruguay**: Fernando Bonilla Cal; **Yemen**: Hamza Al-Naggar; **Zambia**: Mayaba Maimbo; **Zimbabwe**: Dennis Mazingi. **Non-country specific:** Tom Abbott, Michel Adamina, Melika Akhbari, Ruth Benson, Shivam Bhanderi, Bruce Biccard, Edward Caruana, Sohini Chakrabortee, Reema Chapatwala, Ainhoa Costas-Chavarri, Andreas K Demetriades, Anant Desai, Salomone Di Saverio, Thomas Drake, John Edwards, Jonathan Evans, Marco Fiore, Samuel Ford, Christina Fotopoulou, Alexander Fowler, Kaori Futaba, Ian Ganly, Harelimana Grace James, Ewen Griffiths, Alessandro Gronchi, Peter Hutchinson, Gabriella Yael Hyman, Joseph Incorvia, Ritu Jain, Michael Jenkinson, Tabassum Khan, Stephen Richard Knight, Angelos Kolias, Søren Kudsk-Iversen, Tsun Yu Kwan, Elaine Leung, Julio Mayol, Siobhan McKay, John G. Meara, Emily Mills, Susan Moug, Akshay Patel, Roberto Perinotti, Henry E. Rice, Keith Roberts, Andrew Schache, Richard Shaw, Neil Smart, Matthew Stephens, Grant D. Stewart, Ella Teasdale, Peter Vaughan-Shaw, Raghavan Vidya, Naomi Wright, Funmilola Wuraola, Natalie Zimmelman. **Corporate members:** Association of Surgeons in Training (ASiT), European Society of Coloproctology (ESCP), EuroSurg, G4 Alliance, GlobalPaedSurg, Global Initiative for Children’s Surgery (GICS), ItSURG, Irish Surgical Research Collaborative (ISRC), Italian Society of Colorectal Surgery (SICCR), PTSurg, S-ECCO, South African Society of Anaesthesiologists, SpainSurg. |
| --- |

#### Hospital Leads:

| **Albania**: Ervis Agastra *(Regional Hospital of Durres, Durres)*; Dariel Thereska *(University Hospital Center Nene Tereza, Tirana)*; Irida Dajti *(University hospital Koco Gliozheni, Tirana)*. |
| --- |
| **Argentina**: Sergio Martin Lucchini *(Sanatorio Allende - Sede Cerro, Allende, Cordoba)*; Veronica Laudani *(Hospital General de Niños Pedro de Elizalde, Buenos Aires)*; Luis Boccalatte *(Hospital Italiano de Buenos Aires, Buenos Aires)*; Carina Chwat *(Hospital Universitario Austral, Buenos Aires)*; Ivana Ines Pedraza Salazar *(Instituto Oncológico Alexander Fleming, Ciudad Autónoma Buenos Aires)*; Diana Alejandra Pantoja Pachajoa *(Clinica Universitaria Reina Fabiola, Cordoba)*; Sergio Martin Lucchini *(Sanatorio Allende - Sede Nueva Cordoba, Cordoba)*; Agustin Duro *(Hospital Prof Dr Bernardo A Houssay, Provincia de Buenos Aires)*; José Alfredo Calderón Arancibia *(Hospital Público Materno Infantil de Salta, Salta)*. |
| **Australia**: Daniel Cox, Giuliana D’Aulerio, Nagendra Dudi-Venkata, Natasha Egoroff, Shebani Farik, Natalie Lott, Jana-Lee Moss, Sarah Rennie, Lorwai Tan, Chris Varghese, Uyen Giao Vo, David Watson, David Watters, Deborah Wright *(Oceania Coordinating Committee)*; Tim Bright, Paul Hollington, Xuanyu Zhou *(Flinders Medical Centre, Adelaide)*; Hidde M Kroon *(Royal Adelaide Hospital, Adelaide)*; Anthony Farfus *(The Queen Elizabeth Hospital, Adelaide)*; John Barker *(Armidale Rural Referral Hospital, Armidale)*; Eleanor Watson *(Ballarat Base Hospital, Ballarat)*; Sean Stevens *(Colac Area Health, Colac)*; Haider Latif *(University Hospital Geelong, Geelong)*; Amanda Caroline Dawson *(Gosford Hospital, Gosford)*; Alwin Chuan *(Liverpool Hospital, Liverpool)*; Vijayaragavan Muralidharan *(Austin Hospital, Melbourne)*; Enoch Wong *(Box Hill Hospital, Melbourne)*; Travis Ackermann *(Casey Hospital, Melbourne)*; Maurizio Pacilli *(Monash Childrens Hospital, Melbourne)*; Russell Hodgson *(Northern Hospital, Melbourne)*; Alexander Heriot *(Peter MacCallum Cancer Centre, Melbourne)*; Peter Choong *(St Vincent’s Hospital, Melbourne)*; Wendy Brown *(The Alfred Hospital, Melbourne)*; Surjit Lidder *(The Royal Melbourne Hospital, Melbourne)*; Justin Yeung *(Western Health - Footscray hospital and Sunshine hospital, Melbourne)*; Luke Traeger *(Mount Gambier and Districts Health Service, Mount Gambier)*; Guillermo Regalo *(Belmont District Hospital, Newcastle)*; Ralph Gourlay *(Calvary Mater Newcastle, Newcastle)*; Peter Pockney *(John Hunter Hospital, Newcastle)*; Peter Pockney *(Maitland Hospital, Newcastle)*; Sarit Badiani *(Bankstown Hospital, Sydney)*; Cherry Koh *(Lifehouse, Sydney)*; Soni Putnis *(Wollongong Public Hospital, Wollongong)*; Amanda Caroline Dawson *(Wyong Public Hospital, Wyong)*. |
| **Bahrain**: Fayza Haider *(Salmaniya Medical Complex, Manama)*. |
| **Bangladesh**: Ashrarur Rahman Mitul *(Dhaka Shishu (Children) Hospital, Dhaka)*. |
| **Belgium**: Niels Komen *(University Hospital Antwerp, Antwerp)*; Bert Dhondt *(AZ Rivierenland, Bornem)*; Serge Cappeliez, Manon Pigeolet *(CHU de Charleroi, Charleroi)*; Gabrielle van Ramshorst *(University Hospital of Ghent, Gent)*; Martijn Schoneveld, Jasper Stijns *(UZ Brussel, Jette)*; Wouter Oosterlinck *(UZ Leuven, Leuven)*; Nicolas Flamey *(AZ Delta, Roeselare)*. |
| **Benin**: Cyrille Kpangon *(Centre Hospitalier Universitaire de Zone de Suru-Lére, Cotonou)*; Mouhamed Agbadebo *(Zoumè - Hôpital de Zone de Dassa-Zoumè, Dassa)*; Sèmèvo Romaric Tobome *(Centre Hospitalier Départemental de l’Atacora, Natitingou)*; Ismaïl Lawani *(Centre Hospitalier Universitaire et Departemental Oueme Plateau, Porto Novo)*. |
| **Bosnia and Herzegovina**: Anis Cerovac *(University Clinical Center Tuzla, Tuzla)*. |
| **Brazil**: Aldo Vieira Barros *(Hospital Santa Casa de Misericordia de Maceio, Maceio)*; Samuel Aguiar Júnior, Glauco Baiocchi, Heloisa Galvão do Amaral Campos, Jefferson Gross, Felipe José Fernandez Coimbra, Luiz Paulo Kowalski, Fabiana Makdissi, Suely Nakagawa, Joao Pedreira Duprat Neto, Jose Guilherme Vartanian, Guilherme Yazbek, Stenio C Zequi *(A.C. Camargo Cancer Center, São Paulo)*; Ronald Flumignan *(Hospital São Paulo, São Paulo)*. |
| **Bulgaria**: Mihail Slavchev *(University Hospital Eurohospital, Plovdiv)*. |
| **Canada**: Natalia Jaworska *(Foothills Medical Centre - University of Calgary, Calgary)*; Angela Dell *(University of Alberta Hospital, Edmonton)*; Harsha Shanthanna *(St. Joseph’s Healthcare Hamilton, Hamilton)*; Janet Martin *(London Health Sciences Centre and St Josephs Health Care London, London)*; Abdollah Behzadi *(Trillium Health Partners, Mississauga)*; Carolyn Nessim *(The Ottawa Hospital, Ottawa)*; Michelle Mozel *(Eagle Ridge Hospital, Port Moody)*; Pascal St-germain *(Centre Hospitalier Universitaire de Québec, Québec)*; Crispin Russell *(Saint John Regional Hospital, Saint John)*; Gary Groot *(Saskatoon City Hospital/Royal University Hospital/St. Paul’s Hospital, Saskatoon SK)*; Najib Safieddine *(Michael Garron Hospital, Toronto)*; Duminda Wijeysundera *(St. Michael’s Hospital, Toronto)*; Antoine Eskander *(Sunnybrook Hospital, Toronto)*; Sami Chadi *(Toronto Western Hospital, Toronto)*; Shawn MacKenzie *(Royal Columbian Hospital, Vancouver)*; Alana Flexman *(Vancouver General Hospital, Vancouver)*. |
| **Chile**: Fernando Heredia *(Clínica Universitaria de Concepción, Concepción)*; Maria Marta Modolo *(Hospital Barros Luco Trudeau, Santiago)*; Julio Villanueva *(Hospital Clínico San Borja-Arriarán, Santiago)*; Sofia Waissbluth *(Hospital Clinico Universidad Católica, Santiago)*; Roberto Macchiavello *(Hospital de Urgencia Asistencia Pública Dr Alejandro del Río, Santiago)*; Mario I Escudero *(Hospital San Jose, Santiago)*; Tyare Fuentes *(Hospital Sótero de Río, Santiago)*; Ximena Mimica *(Instituto Oncologico Fundacion Arturo Lopez Perez, Santiago)*; Maricarmen Olivos *(Roberto del Río Children’s Hospital, Santiago)*. |
| **Colombia**: Dinimo Bolivar Saenz *(Clinica Colsubsidio Calle 94, Bogota)*; Lina Caicedo *(Clinica Reina Sofia, Bogota)*; Juan Pablo Alzate, Joaquin Luna *(Clínica Universitaria Colombia, Bogota)*; Nestor Fabian Pedraza Alonso *(Colombiana de Trasplantes, Bogota)*; Camilo Ortiz Silva *(El Tunal, Bogota)*; Carlos J- Perez Rivera *(Fundacion Cardioinfantil-IC, Bogota)*; Juliana Rodriguez *(Fundacion Santa Fe de Bogota, Bogota)*; Liliana Silva-Igua, Martha Luz Torres *(Hospital Universitario Mayor, Bogota)*; Lina María Trujillo *(Instituto Nacional de Cancerologia, Bogota)*; Albaro José Nieto Calvache *(Fundación Valle del Lili, Cali)*; Julián Balanta-Melo *(Hospital Universitario del Valle Evaristo García, Cali)*; Rafael Figueroa - Casanova *(Clinica Avidanti - Ibague, Ibague)*; Oscar-Julián García-Montoya *(Clínica Avidanti - Manizales, Manizales)*; Carlos Andres Marulanda Toro, Marcela Velez Botero *(SES Hospital de Caldas, Manizales)*; Maria Clara Mendoza Arango *(Hospital Universitario San Vicente Fundacion, Medellin)*; Eneida Diaz Martinez *(IMAT Oncomedica, Monteria)*; Valentina Gutiérrez Perdomo *(Hospital Universitario Hernando Moncaleano Perdomo, Neiva)*; Jose Andres Calvache *(Hospital Susana Lopez de Valencia, Popayan)*; Jose Andres Calvache *(Hospital Universitario San José, Popayán)*; Emileth Montenegro *(Clínica Avidanti Santa Marta, Santa Marta)*. |
| **Croatia**: Jakov Mihanovic *(Zadar General Hospital, Zadar)*. |
| **Cuba**: Pablo Mijahil Avilés Jiménez *(Hospital Pediátrico Juan Manuel Márquez, La Habana)*. |
| **Cyprus**: Nikolaos Gouvas *(Nicosia General Hospital, Nicosia)*. |
| **Czech Republic**: René Novysedlák *(Motol University Hospital, Prague)*. |
| **Dominican Republic**: Julia Rodriguez-Abreu *(CEDIMAT - Centro de Diagnóstico, Medicina Avanzada, Laboratorio y Telemedicina, Santo Domingo)*; Dolores Mejía *(Hospital General Plaza de la Salud, Santo Domingo)*. |
| **Ecuador**: Eddy Lincango-Naranjo *(AXXIS Hospital Quito Ecuador, Quito)*. |
| **Egypt**: Galal Abouelnagah, Sameh Shehata *(Alexandria Main University Hospital, Alexandria)*; Ahmed Hossam Eldin Fouad Rida *(El Hadara University Hospital Alexandria University, Alexandria)*; Ramy A. Hassan, Mahmoud M. Saad *(Assiut University Hospital, Assiut)*; Mohamed Reda Loaloa *(Benha University Hospital, Benha)*; Badr Mostafa, Mohamed Qassem *(Ain Shams University Specialized Hospital, Cairo)*; Mohamed Fahmy *(Al Zahraa University Hospital, Cairo)*; Hesham Abozied *(EL-Hussein University Hospital, Al-Azhar University, Faculty Of Medicine, Cairo)*; Ahmed Y Azzam *(Damietta Specialized Hospital, Damietta)*; Sherief Ghozy *(sheikh zayed specialized hospital, giza)*; Asser Sallam *(Suez Canal University Hospital, Ismailia)*; Ahmed Shehta *(Gastrointestinal surgery center, Mansoura)*; Sameh Emile *(Mansoura University Hospital, Mansoura)*; Mohamed Abdelkhalek *(Oncology Center Mansoura University, Mansoura)*; Rehab Samaka *(Menofia University Hospital, Menofia)*; Amr Morsy *(Minya University Hospital, Minya)*; Ahmed Elshawadfy Sherif *(National Liver Institute, Menoufia University, Shibin Elkom)*. |
| **El Salvador**: Danilo Alfonso Arévalo Sandoval *(Clínica de Gineco-Oncología Dr. Danilo Arévalo, San Salvador)*. |
| **Ethiopia**: Abraham Negussie *(ALERT center, Addis Ababa)*; Tigist Fisseha *(Eka kotebe General Hospital, Addis Ababa)*; Kibruyisfaw Shumbash *(Myungsung Christian Medical Centre, Addis Ababa)*; Metasebia Abebe *(Saint Paul Hospital Millennium Medical College, Addis Ababa)*; Samuel Hailu *(Tikur Anbessa Specialized (Black Lion) Hospital, Addis Ababa)*; Seid Mohammed Yasin *(Yekatit 12 hospital medical college, Addis Ababa)*; Yemisirach Bizuneh Akililu *(Zewditu Memorial Hospital, Addis Ababa)*; Abebe Megersa *(Ambo University Referral Hospital, Ambo)*; Teshome Tefera *(Arbaminch General Hospital, Arbaminch)*; Melatework Assefa *(Adisalem Primary Hospital, Bahir Dar)*; Bahru Atnafu *(Bahir Dar University Tibebe Ghion Specialized Hospital, Bahir Dar)*; Bereket Tsegaye *(Debre Berhan Comprehensive Specialized Hospital, Debre Birhan)*; Yoseph Solomon Bezabih *(Debre Markos Comprehensive specialized Hospital, Debre Markos)*; Silamlak Sisay *(Dessie Referral Hospital, Dessie)*; Kebebe Bekele *(Maddawalabu University Goba Referral Hospital, Goba)*; Moa Jira *(Hiwot Fana specialized University Hospital, Harar)*; Mengistu G Mengesha *(Hawassa University Comprehensive Specialized Hospital, Hawassa)*; Habtamu Derilo *(Wachemo University Nigist Elleni Mohammed Memorial Referral Hospital, Hossana)*; Eyueal Degefa *(Jimma University Medical Center, Jimma)*; Anteneh Tadesse *(Mekelle University Ayder Comprehensive Specialised Hospital, Mekelle)*; Melkamu Nidaw *(Pawi General Hospital, Pawi)*. |
| **Finland**: Elise Sarjanoja *(Länsi-Pohja Central Hospital, Kemi)*; Joonas H Kauppila *(Oulu University Hospital, Oulu)*. |
| **France**: Sylvie Testelin *(CHU Amiens, Amiens)*; Sophie Boucher *(CHU Angers, Angers)*; Lionel Jouffret *(Centre Hospitalier Avignon, Avignon)*; Zaher Lakkis *(CHU Besançon, Besancon)*; Alban Zarzavadjian Le Bian *(Avicenne Hospital, Bobigny)*; Luke Harper *(CHU Bordeaux, Bordeaux)*; Marc Danguy des Déserts *(Military Hospital Clermont Tonnerre (Hôpital des Armées), Brest)*; Benoît André *(Centre Hospitalier Intercommunal de Castres-Mazamet, Castres)*; Karem Slim *(Ferrand - CHU Clermont-Ferrand, Clermont)*; Romain Verhaeghe *(Clinique des 2 Caps, Coquelles)*; Andrea Police *(Hôpital Simone Veil, Eaubonne)*; Edouard Girard *(CHU Grenoble-Alpes, Grenoble)*; Alexandre Chebaro *(CHU Lille Hôpital Claude Huriez, Lille)*; Armande Subayi Nkembi *(CHU Lille hôpital Jeanne de Flandres, Lille)*; Laurent Arnalsteen *(Hôpital Privé La Louvière, Lille)*; Quentin Ballouhey *(CHU Limoges, Limoges)*; Diane Mege *(Timone, Marseille)*; Clement Jeandel *(CHU Montpellier (Lapeyronie), Montpellier)*; Emilie Duchalais *(CHU Nantes, Nantes)*; Pierre-Alban Bouche *(Hôpital Cochin - APHP, Paris)*; Gilles Manceau *(Hôpital européen Georges-Pompidou, Paris)*; Célia Crétolle *(Hôpital Necker Enfants Malades - APHP, Paris)*; Erik Hervieux *(Hôpital Trousseau - APHP, Paris)*; Noémie Girard *(Institut Curie, Paris)*; Agathe Seguin-Givelet *(institut mutualiste montsouris, Paris)*; Sebastien Gaujoux *(Pitie Salpetriere, Paris)*; Belinda De Simone *(Centre Hospitalier Intercommunal Poissy Saint Germain en Laye, Poissy)*; Matthieu Boisson *(CHU Poitiers, Poitiers)*; Damien Bergeat *(CHU Rennes - Hopital Pontchaillou, Rennes)*; Alexis P Arnaud *(CHU Rennes - Hopital Sud, Rennes)*; Fabien Fredon *(Junien - Centre Hospitalier Roland Mazoin, Saint)*; Francesco Nappi *(Centre Cardiologique du Nord, Saint Cenis)*; Radwan Kassir *(CHU Reunion, Saint Denis)*; Aurélien Scalabre *(CHU Saint Etienne, Saint Etienne)*; Federico Migliorelli *(Centre Hospitalier Intercommunal des Vallées de l’Ariège, Saint Jean de Verges)*; Romain Verhaeghe *(CMCO Côte d’Opale, Saint Martin Boulogne)*; Anne-Cecile Ezanno *(Hia Begin, St Mande)*; Barbara Seeliger *(HUS, Pole Hépato-Digestif / IHU-Strasbourg, Strasbourg)*; Charlotte Vaysse *(CHU Toulouse, Toulouse)*; Helene Charbonneau, Vincent Misrai *(Clinique Pasteur, Toulouse)*; Olivier Abbo *(Hôpital des Enfants -CHU Toulouse, Toulouse)*; Martina Aida Angeles *(Institut Claudius Regaud - Institut Universitaire du Cancer de Toulouse, Toulouse)*; Laurent Brunaud *(lès-Nancy - CHRU NANCY, Vandoeuvre)*. |
| **Georgia**: Zaza Demetrashvili *(N.Kipshidze Central University Clinic, Tbilisi)*. |
| **Germany**: Ali Modabber *(University Hospital Aachen, Aachen)*; Sebastian Wolf *(University Hospital Augsburg, Augsburg)*; Carsten Kamphues *(Charité University Medicine - Campus Benjamin Franklin, Berlin)*; Philipp Höhn *(St. Josef-Hospital, Bochum)*; Tim R. Glowka *(University Hospital Bonn, Bonn)*; Alexander Christopher Rokohl *(University Hospital of Cologne, Cologne)*; Ulrich Bork *(University Hospital Carl Gustav Carus, Technical University Dresden, Dresden)*; Georg Fluegen *(University Hospital Duesseldorf, Duesseldorf)*; Raymund E. Horch *(Universitätsklinikum Erlangen, Erlangen)*; Andrea Schmedding, Andreas Schnitzbauer *(Frankfurt University Hospital, Goethe University, Frankfurt)*; Helge Eberbach, Daniel Schlager *(University Medical Center Freiburg, Faculty of Medicine, Freiburg)*; Fritz Spelsberg *(Klinikum Fürstenfeldbruck, Fürstenfeldbruck)*; Lena Keppler *(Partenkirchen - Klinikum Garmisch-Partenkirchen, Garmisch)*; Andreas Hecker *(University Hospital Giessen and Marburg, Giessen)*; Susanne Wolfer *(University Hospital Goettingen / Universitätsmedizin Goettingen, Goettingen)*; Ulrich Ronellenfitsch *(University Hospital Halle, Halle)*; Christine Nitschke *(University Medical Center Hamburg-Eppendorf, Hamburg)*; Christian Peiper *(Evangelisches Krankenhaus Hamm, Hamm)*; Ibrahim Hakami *(KRH Nordstadt-Siloah Hospitals, Hannover)*; Stefan Welter *(Lung Clinic Hemer, Hemer)*; Karine Nikolaieva *(Sana Klinikum, Hof)*; Andreas Roth *(University Hospital Leipzig, Leipzig)*; Judith Lindert *(University Hosital Schleswig- Holstein, Lübeck)*; Konstantinos Gousias *(St Marien Hospital Lünen, Lünen)*; Anke Rissmann *(University Hospital Magdeburg, Magdeburg)*; Valerie Catherine Linz *(University Hospital Mainz, Mainz)*; Nuh Rahbari, Marie-Claire Rassweiler-Seyfried *(Mannheim University Medical Center (Universitätsmedizin Mannheim), Mannheim)*; Anna Eleonora Gut *(Isarklinikum, München)*; Jens Gempt, Daniel Reim, Arthur Wagner *(Klinikum Rechts der Isar TUM School of Medicine, Munich)*; Markus Albertsmeier, Alexander M. Keppler *(Ludwig Maximilian University of Munich - Großhadern, Munich)*; Mircea Gabriel Stoleriu *(Asklepios Pulmonary Hospital, Munich Gauting)*; Tim Saier *(Berufsgenossenschaftliche Unfallklinik Murnau, Murnau)*; Josef Stadler *(RoMed Klinik Prien am Chiemsee, Prien am Chiemsee)*; Julia Christina Kaiser *(Klinikum St. Hedwig, Barmherzige Brüder, Regensburg)*; Stefan M. Brunner, Karin Pfister *(University Hospital Regensburg, Regensburg)*; Jonas Herzberg *(Krankenhaus Reinbek St. Adolf-Stift, Reinbek)*; Kai Nowak, Tobias Reinhard *(RoMed Klinikum Rosenheim, Rosenheim)*; Gregor A. Stavrou *(Klinikum Saarbruecken, Saarbruecken)*; Alfred Königsrainer, Christian Konrads, Markus Quante *(University Hospital Tuebingen, Tuebingen)*; Simon Laban *(University Hospital Ulm, Ulm)*; Silke Pusch von *(Kreisklinik Wolfratshausen, Wolfratshausen)*; Markus Hirschburger *(Klinikum Worms, Worms)*; Johannes Doerner *(Helios Universitätsklinikum Wuppertal (Universität Witten/Herdecke), Wuppertal)*; Armin Wiegering *(University Hospital Würzburg, Würzburg)*. |
| **Greece**: Ekaterini Christina Tampaki *(KAT Athens General Hospital, Athens)*. |
| **Guatemala**: Alitza Gutiérrez Ruiz *(Hospital Universitario Esperanza, Guatemala)*; Alejandra Rodas *(Centro Clínico Cabeza y Cuello, Guatemala City)*; Ana Lucía Portilla, Gustavo Recinos *(Hospital General De Enfermedades, Guatemala City)*; Maria-Lorena Aguilera-Arevalo, Jacqueline Carrera *(Hospital General San Juan De Dios, Guatemala City)*; Amalia Barrios Duarte *(Hospital Herrera Llerandi, Guatemala City)*; Megan Lowey *(Sanatorio Las Majadas, Guatemala City)*; Sabrina Barillas *(Symmetria, Guatemala City)*. |
| **India**: Atul Suroy *(India Coordinating Committee)*; Dhaivat Vaishnav *(Zydus Hospital, Ahmedabad Gujarat)*; Raghunandan Gorantlu Chowdappa *(Shri Krishna Hospital and pramukhswami medical college, Anand , Gujarat)*; Irappa Madabhavi *(Kerudi Cancer Hospital, Bagalkot)*; Dhananjaya Bhat *(Aster RV Hospital, Bangalore)*; Sunil Kumar Venkatappa *(Victoria Hospital, Bangalore)*; Sumit Thakar *(Sri Sathya Sai Institute of Higher Medical Sciences, Bengaluru)*; Kavitha Jain *(Sri Shankara Cancer Hospital and Research Centre, Bengaluru)*; Aruna Kumar *(Gandhi Medical College and Sultania Zanana Hospital, Bhopal)*; Manoj Nagar *(ALL INDIA INSTITUTE OF MEDICAL SCIENCE BHOPAL, BHOPAL)*; Tushar Mishra, Arunkumar Sekar *(All India Institute Of Medical Sciences - Bhubaneswar, Bhubaneswar)*; Anand Gupta *(Government medical college hospital, Chandigarh)*; Lileswar Kaman, Madhivanan Karthigeyan, Manjul Tripathi *(Postgraduate Institute of Medical Education & Research, Chandigarh, India, Chandigarh)*; Ashwin Rammohan *(Dr.Rela Institute & Medical Centre, Chennai)*; Sudheer Othiyil vayoth, Anupama Rajanbabu *(Amrita Institute of Medical Sciences Hospital, Cochin)*; Anbukkani Subbian *(Kovai Medical Centre and Hospital, Coimbatore)*; Rahul Gupta *(Synergy Institute of Medical Sciences, Dehradun)*; Monish Raut *(Artemis Health Institute, Gurugram)*; Nissi Evelyn. R *(Government Dental College and Hospital, Hyderabad)*; Lavanya Kannaiyan *(Rainbow children’s hospital, Hyderabad)*; Dr. Anil Matai *(Santokh ba Durlabhji memorial hospital, Jaipur)*; Sanjeev Misra *(All India Institute of Medical Sciences (AIIMS), Jodhpur, Jodhpur)*; Vishal Bhende *(Shree Krishna Hospital, Karamsad)*; Sathish Muthu *(Government Hospital, Velayuthampalayam, Karur)*; Indranil Ghosh *(Institute of Neurosciences, Kolkata, Kolkata)*; Abhishek Sharma *(Tata Medical Center, Kolkata)*; Ankur Bajaj, Shiv Rajan *(King George’s Medical University, Lucknow)*; Gaurav Agarwal *(Sanjay Gandhi Post Graduate Institute Of Medical Sciences, Lucknow)*; Pranay Pawar *(Christian Medical College & Hospital, Ludhiana)*; Philip Alexander *(Lady Willingdon Hospital, Manali)*; M Vijayakumar Vijayakumar *(Yenepoya medical college hospital, Mangalore)*; BM Zeeshan Hameed, Badareesh L *(Kasturba Medical College Hospital, Manipal, Manipal)*; Navneet Kumar Chaudhry *(Maharishi Markandeshwar Institute of Medical Sciences & Research, Mullana, Ambala, Haryana)*; Lipika Baliarsing, Satish Dharap *(BYL Nair Hospital, mumbai)*; Amruta Kulkarni *(Fortis Hospital Mulund, Mumbai)*; Yuvaraja Thyavihally *(Kokilaben Dhirubhai Ambani Hospital, Mumbai)*; Rahul Deo Sharma *(Lilavati Hospital & Research Centre, Mumbai)*; C S Pramesh *(Tata Memorial Hospital, Mumbai)*; Rajesh Soni *(Soni Hospital, Nagpur)*; Surya Kumar Dube, Shilpa Sharma *(All India Institute of Medical Sciences, New Delhi)*; Harvinder Singh *(Indian Spinal Injuries Center, New Delhi)*; Lovenish Bains *(Maulana Azad Medical College, New Delhi)*; Rahul Ghodke *(Sanjay Clinic & Apollo Hospital, New Mumbai)*; Ashwani Kumar *(Government Medical College, Patiala)*; Vivek Sodhai *(Lokmanya Hospital for Special Surgery, Pune)*; Suvendu Maji *(Bengal cancer foundation(BIMS Hospital), Purba Bardhaman)*; Somprakas Basu *(All India Institute Of Medical Sciences, Rishikesh)*; Chandrashekhar Mahakalkar *(Acharya Vinoba Bhave Rural Hospital, Sawangi (Meghe), Wardha)*; Ravi Kannan *(Cachar Cancer Hospital and Research Centre, Silchar)*; Asif Mehraj *(Sher-i-Kashmir Institute of Medical Sciences, Srinagar)*; Ranganath N *(KVG Medical College & Hospital, Sullia)*; Ashish Phadnis *(jupiter hospital, thane)*; I Yadev *(Government Medical College Thiruvananthapuram, Thiruvananthapuram)*; Alfie Kavalakat *(Jubilee Mission Medical College & Research Institute, Thrissur)*; Rohin Mittal *(Christian Medical College & Hospital, Vellore)*; Karthik Chandra Vallam *(Mahatma Gandhi cancer hospital and research institute, Visakhapatnam)*. |
| **Iran, Islamic Rep.**: Hamed Akhavizadegan, Esmaeil Rezghi Maleki, Naser Yousefzadeh Kandevani *(Baharloo hospital, Tehran)*. |
| **Ireland**: Hilary Ikele, Catherine McNestry *(Mayo University Hospital, Castlebar)*; Christina Fleming, Stephen O’Brien *(Cork University Hospital, Cork)*; Sami Abd Elwahab, Niall Davis, Mohsen Javadpour *(Beaumont Hospital, Dublin)*; Brendan McDonnell *(Coombe Women and Infants University Hospital, Dublin)*; Clare O Connor *(Rotunda Hospital, Dublin)*; Jarlath Bolger, Cillian Clancy, Stefanie M Croghan, Noel Donlon *(St James’s Hospital, Dublin)*; Carolyn Cullinane *(St Vincent’s University Hospital, Dublin)*; Ben Creavin, Muheilan Muheilan *(Tallaght Hospital, Dublin)*; Helen Earley *(University Hospital Galway, Galway)*; Syed Mohammad Umar Kabir *(LETTERKENNY UNIVERSITY HOSPITAL, Letterkenny)*; Muhammad Fahadullah *(University Hospital Limerick, Limerick)*; Éanna Ryan *(Tullamore University Hospital, Tullamore)*; Tara Connelly *(University Hospital Waterford/University College Cork, Waterford)*. |
| **Israel**: Oded Zmora *(SHAMIR MEDICAL CENTER, BE’ER YA’AKOV)*. |
| **Japan**: Daisuke Hashimoto *(Kansai Medical University, Osaka)*. |
| **Jordan**: Majdi Ali Alqudah *(Al Iman Hospital, Ajloun)*; Amer Alajalen *(Al Karak Hospital, Alkarak)*; Rand Y. Omari *(Abdali Hospital, Amman)*; Faris Ayasra, Abdulrahman Qasem *(Al-Basheer Hospital, Amman)*; Yazan Alawneh *(Ibn Al Haitham Hospital, Amman)*; Amer Ahmad, Omar Aladawi, Bourhan Alrayes, Hanan Haidar, Shatha Husain, Faisal Qassem *(Islamic Hospital, Amman)*; Adnan Sumadi *(Istiklal Hospital, Amman)*; Ala’a Abu Salhiyeh, Balqees Mahmoud Al-Manaseer, Zaid Alsunna, Hazim Ra’ed, Faten Reyad Bani Hamad *(Jordan University Hospital, Amman)*; Amro Abuleil *(Jordanian royal medical services, Amman)*; Mohamad K. Abou Chaar *(King Hussein Cancer Center, Amman)*; Elmi Ahmed Mohamed Jimaale *(The Speciality Hospital, Amman)*; Marah Abu-Mehsen, Noor Olaywah, Omar Wafi *(King Abdullah University Hospital/ Jordan University of Science and Technology, Ar Ramtha)*; Hazim Ababneh *(Ar Ramtha Govermental Hospital, Irbid)*; Luai Abu-Ismail, Almu’atasim Khamees *(Irbid Specialty Hospital, Irbid)*; Ahmad Alkhatib *(Princess Basma Hospital, Irbid)*. |
| **Kazakhstan**: Raikhan Bolatbekova *(Almaty Oncology Centre, Almaty)*; Mukhtar Kulimbet *(City Cardiology Center, Almaty)*; Talgat Nurgozhin, Timur Saliev, Baurzhan Zhussupov *(City Clinical Hospital No. 4, Almaty)*; Ydyrys Almabayev, Ildar Fakhradiyev, Timur Saliev *(Karasay Central District Hospital, Almaty)*; Dilyara Kaidarova *(Kazakh Institute of Oncology and Radiology, Almaty)*. |
| **Libya**: Khalil Tamoos *(Alabyar General Hospital, Alabyar)*; Ahmed Aqeelah *(Althawra Hospital Albyda, Albayda)*; Alsnosy abdullah Khalefa mohammed *(Almarj Teaching Hospital, Almarj)*; Faraj Al maadany, Ghadah Alkadeeki *(Al-jalaa Teaching/Trauma Hospital, Benghazi)*; Milad Gahwagi *(Benghazi Medical Center, Benghazi)*; Wafa Aldressi *(N. B. Hospital, Benghazi)*; Mohamed Amnaina *(Al-Wahda Hospital, Darna)*; Arowa hassan abdulrahman Alansari *(Ghadames general hospital, Ghadames)*; Akram Alkaseek *(Gharyan Central Hospital, Gharyan)*; Ghozlan Yagoub *(Alhikma hospital, Misurata)*; Anass Ben Amer, Marwa Salem *(Misurata Central Hospital, Misurata)*; Ayman Almugaddami *(Nalut Central Hospital, Nalut)*; Dania Burgan *(National Cancer Institute, Sabratha - Libya, Sabratha)*; Mohammed Abdelkabir *(Sabha Medical Center, Sebha)*; Khayriyah Alshareef *(Tripoli - Yashfeen Clinic, Tajora)*; Rayet al islam Ben jouira *(National Heart Centre, Tajoura Heart Center Hospital, Tajoura)*; Ayman Meelad *(Tarhuna General Hospital, Tarhuna)*; Ahmad Bouhuwaish *(Tobruk Medical Center, Tobruk)*; Sumayya Essayah Dwaga, Houda Khalifa *(Aljala Maternity Hospital, Tripoli)*; Bushray Almiqlash *(Alkhadra Hospital, Tripoli)*; Taha Suliaman *(Crown of Health Clinic, Tripoli)*; Mohammed Alawami *(Medical Care Clinic, Tripoli)*; Fras Elhajdawe *(Metiga Hospital, Tripoli)*; Hajir Aboazamazem *(Tripoli Central Hospital, Tripoli)*; Ibrahim Ellojli, Ahmed Msherghi *(Tripoli Medical Center/ Tripoli University Hospital, Tripoli)*; Ismail Ali Saleh *(Zintan Teaching Hospital, Zintan)*; Mohammed Alayan *(Zliten Teaching Hospital, Zliten)*. |
| **Malaysia**: April Camilla Roslani *(University Malaya Medical Centre, Kuala Lumpur)*. |
| **Namibia**: Marcel Didier Ndayishyigikiye *(Ongwediva Medipark Teaching Hospital, Ongwediva)*; Akutu Munyika *(Onandjokwe Intermediate Hospital, Oniipa)*; Philipp Plarre *(Mediclinic Cottage Hospital, Swakopmund)*; David W Borowski *(Welwitschia Hospital, Walvis Bay)*; Pueya Rashid Nashidengo *(Windhoek Central Academic Hospital, Windhoek)*. |
| **Netherlands**: Milou Noltes, Pieter Steinkamp *(University Medical Center Groningen, Groningen)*. |
| **New Zealand**: Cameron Wells *(Middlemore Hospital, Auckland)*; Rebecca Teague *(North Shore Hospital, Auckland)*; Brodie Elliott *(Christchurch Hospital, Christchurch)*; David Kieser *(Southern Cross Hospital, Christchurch)*; Omar Mohyieldin *(Dunedin Public Hospital, Dunedin)*; Chris Varghese *(Waikato Hospital, Hamilton)*; Nick McIntosh *(Hawke’s Bay Hospital, Hastings)*; Cheyaanthan Haran, Sarah Rennie *(Wairarapa Hospital, Masterton)*; Jasmin King *(Taranaki Base Hospital, New Plymouth)*; Jeong Ha *(Wellington Regional Hospital, Wellington)*; Matthew James McGuinness *(Whangarei Hospital, Whangarei)*. |
| **Nigeria**: Opeoluwa Adesanya *(Federal Medical Centre, Abeokuta)*; Julius Olaogun *(Ekiti - Ekiti State University Teaching Hospital, Ado)*; Akinola Akinmade *(Afe Babalola University Multi-System Hospital, Ado Ekiti)*; Kefas Bwala *(Abubakar Tafawa Balewa University Teaching Hospital Bauchi, Bauchi)*; Peter Agbonrofo *(University of Benin Teaching Hospital, Benin City)*; Akinwale Afolabi *(Kebbi - Federal Medical Centre, Birnin)*; Usang Usang *(University of Calabar Teaching Hospital, Calabar)*; Sebastian Ekenze *(University of Nigeria Teaching Hospital, Enugu)*; Samson Olori *(University of Abuja Teaching Hospital, Gwagwalada)*; Taiwo Akeem Lawal *(University College Hospital, Ibadan)*; Justina Seyi-Olajide *(Lagos University Teaching Hospital, Idi Araba)*; Abiodun Okunlola *(Federal Teaching Hospital, Ido Ekiti, Ido Ekiti)*; Omolara Williams *(Lagos State University Teaching Hospital, Ikeja)*; Adewale Adisa *(Ife - Obafemi Awolowo University Teaching Hospitals Complex, Ile)*; Lukman Abdur-Rahman *(University of Ilorin Teaching Hospital, Ilorin)*; Stephen Kache *(Barau Dikko Teaching Hospital, Kaduna)*; Danjuma Sale *(Epsilon Specialist Hospital Barnawa, Kaduna)*; Lofty-John Anyanwu *(Aminu Kano Teaching Hospital, Kano)*; Chukwuma Okereke *(Federal Medical Centre, Owo, Ondo State)*; Musliu Adetola Tolani *(Ahmadu Bello University Teaching Hospital, Zaria)*. |
| **North Macedonia**: Venko Filipce *(University clinic for neurosurgery, Faculty of medicine, University St.Cyril and Methodius, Skopje)*; Lazar Todorovic *(University Clinic for Pediatric Surgery, Skopje)*; Sofija Pejkova *(University Clinic for Plastic and Reconstructive surgery, Faculty of Medicine, University St. Cyril and Mthodius, Skopje)*; Sotir Stavridis *(University Clinic of Urology, Skopje)*. |
| **Oman**: John George Massoud *(Khoula Hospital, Muscat)*; Sareyah Alsibai *(Sultan Qaboos University Hospital, Muscat)*. |
| **Pakistan**: Rizwan Sultan *(Islamabad medical complex, Islamabad)*; Humera Naz Altaf, Abu Bakar Hafeez Bhatti *(Shifa international hospital, Islamabad)*; Shahzad Hussain Waqar *(The Pakistan Institute of Medical Sciences, Islamabad)*; Aliya Aziz *(Aga Khan University, Karachi)*; Asad Ali Kerawala *(Cancer Foundation Hospital, Karachi)*; Lajpat Rai *(Dr Ruth K.M. Pfau Civil Hospital, Karachi)*; Mariyah Anwer *(Jinnah Post Graduate Medical Center, Karachi)*; Aiman Tariq *(National Institute of Cardiovascular Diseases, Karachi)*; Bushra Ayub *(Patel Hospital, Karachi)*; Sami ullah Niazi *(South City Hospital, Karachi)*; Muhammad Yasir Naseem *(Government Mian Meer Hospital Lahore, Lahore)*; Muhammad Zeeshan Sarwar *(King Edward Medical University - Mayo Hospital, Lahore)*; Muhammad Imran Khokhar *(Lahore General Hospital, PGMI, AMC, Lahore)*; Imdad Ahmad Zahid *(Services Hospital Lahore, Lahore)*; Haroon Javaid Majid *(Shaikh Zayed Hospital, Lahore)*; Nabila Talat *(The Children’s Hospital & The Institute of Child Health Lahore, Lahore)*; Muhammad Asif *(District Headquarter Hospital - Mandi Bahauddin, Mandi Bahauddin)*; Muhammad Hamid Chaudhary *(Chaudhary pervaiz elahi institute of Cardiology, Multan)*; Umer Farooq *(Tehsil Headquarters Hospital Pasrur, Pasrur district Sialkot)*; Siddique Ahmad *(Hayatabad Medical Complex, Peshawar)*; Waleed Mabood *(Khyber teaching hospital, Peshawar)*; Syed Imran Bukhari *(Lady Reading Hospital, Peshawar)*; Muhammad Tariq *(North West General Hospital and Research Centre, Peshawar)*; Eesha Yaqoob *(Benazir Bhutto Hospital, Rawalpindi)*; Saad Javed *(District Headquarter Hospital - Rawalpindi, Rawalpindi)*; Saad Javed *(Holy Family Hospital, Rawalpindi)*; Muhammad Usman Malik *(District Headquarter & Teaching Hospital - Sargodha, Sargodha)*; Hassan Nawaz Yaqoob *(Social Security Hospital, Sheikhupura, Sheikhupura)*. |
| **Panama**: Moises Cukier *(Pacifica Salud Hospital, Panama)*. |
| **Peru**: Glenda Marina Falcon Pacheco *(Instituto Regional de Enfermedades Neoplásicas del Sur, Arequipa)*; Robinson Mas Melendez, Arazzelly Del Pilar Paucar Urbina *(Ayacucho Regional Hospital, Ayacucho)*; Jose Rios Chiuyari *(Hospital regional de lambayeque, Lambayeque)*; Carlos Eduardo Otiniano Alvarado *(Arzopispo Loayza National Hospital, Lima)*; Lorena Fuentes Rivera Lau *(British American Hospital, Lima)*; Giuliano Borda-Luque, Milagros Niquen-Jimenez *(Cayetano Heredia National Hospital, Lima)*; Claudia Arias *(Edgardo Rebagliati Martins National Hospital, Lima)*; Sergio Zegarra *(Guillermo Almenara National Hospital, Lima)*; Jenner Betalleluz Pallardel *(Hospital de Emergencias Jose Casimiro Ulloa, Lima)*; Regina Amparo Ugarte Oscco *(Hospital Emergencia Ate Vitarte, Lima)*; Gian Mendiola *(Hospital Santa Rosa de Lima, Lima)*; Yahaira Tatiana Carpio Colmenares *(SANNA - Clínica El Golf, Lima)*; Carlos Shiraishi Zapata *(Hospital Il Talara, Talara)*; Maria Rosa Ortiz *(Hospital Belén de Trujillo, Trujillo, La Libertad)*. |
| **Philippines**: Marie Dione Sacdalan *(Philippine General Hospital, University Of The Philippines Manila, Manila)*. |
| **Poland**: Piotr Major *(Jagiellonian University Medical College, Krakow)*. |
| **Portugal**: Filipe Castro Borges *(Hospital Garcia de Orta, Almada)*; Octavio Viveiros *(Clínica de Santo António - Grupo Lusíadas, Amadora)*; Pedro Serralheiro *(Hospital Dr. Antonio José de Almeida, Cascais)*; Paulo Santos-Costa *(IPO Coimbra, Coimbra)*; Filipa Mendes *(Centro Hospitalar Cova da Beira, Covilha)*; Miguel Rocha Melo *(Hospital do Espirito Santo, Evora)*; Paulo Cardoso, Ana Soares *(Hospital Faro, Centro Hospitalar Universitario do Algarve, Faro)*; José Azevedo *(Hospital da Horta, E.P.E., Horta)*; Rita Gonçalves Pereira *(Centro Hospitalar de Leiria, E.P.E., Leiria)*; Nelson Silva *(Hospital CUF Infante Santo, Lisboa)*; André Caiado *(Instituto Português de Oncologia de Lisboa Francisco Gentil, Lisboa)*; Maria Luís Sacras *(Centro Hospitalar Universitário Lisboa Central, Lisbon)*; Pedro Azevedo *(Hospital Beatriz Angelo, Loures)*; Rui Almeida-Reis *(Centro Hospitalar do Tamega e Sousa, Penafiel)*; Miguel F. Cunha *(Centro Hospitalar Universitario do Algarve - Unidade de Portimão, Portimao)*; João Oliveira *(Centro Hospitalar do Porto, Porto)*; Jorge Nogueiro, Mafalda Sampaio-Alves *(Centro Hospitalar e Universitário de São João, Porto)*; Luciana Cidade Costa *(Hospital da Prelada, Porto)*; Catarina Baía *(IPO Porto, Porto)*; Ana Cláudia Deus *(Hospital do Litoral Alentejano, Santiago do Cacém)*; Rita Branquinho *(Centro Hospitalar Médio Tejo, Tomar)*; André Marçal *(Centro Hospitalar de Trás-os-Montes e Alto Douro, E.P.E., Vila Real)*; André Tojal *(Centro Hospitalar Tondela-Viseu, Viseu)*. |
| **Qatar**: Ahmad Zarour *(Hamad General Hospital, Doha)*; Ahmad Zarour *(Hazm Mebaireek General Hospital, Doha)*. |
| **Romania**: Silviu Tiberiu Makkai-Popa *(Sf. Constantin Hospital, Brasov)*; Aurel Mironescu *(Spitalul Clinic De Copii Brasov, Brasov)*; Florin Grama *(Coltea Clinical Hospital, Bucharest)*; Elena Adelina Toma *(Elias Emergency Hospital, Bucharest)*; Ionut Negoi *(Emergency Clinical Hospital Bucharest, Bucharest)*; Daniela Filipescu *(Emergency Institute for Cardiovascular Diseases ‘Prof. Dr. C.C. Iliescu’, Bucharest)*; Nicolae Bacalbasa *(Fundeni Clinical Institute, Bucharest)*; Natalia Motas *(Institute of Oncology Prof Dr Al Trestioreanu, Bucharest)*; Sebastian Ionescu *(Maria Sklodowska Curie Emergency Hospital, Bucharest)*; Octav Ginghina *(Saint John Emergency Hospital, Bucharest)*; Radu Costea, Narcis Octavian Zarnescu *(University Emergency Hospital Bucharest, Bucharest)*; Radu Drasovean *(Cluj-Napoca Emergency County Hospital, Cluj)*; Eduard-Alexandru Bonci *(Prof Dr Ion Chiricuta Institute of Oncology, Cluj)*; Mihail-Gabriel Dimofte *(Regional institute of Oncology Iasi, Iasi)*; Vlad Porumb *(Spitalul Clinic de Urgență Militar Dr Iacob Czihac, Iasi)*. |
| **Russian Federation**: Mikhail Kirov *(City Hospital #1, Arkhangelsk)*; Yegor Molitvin *(Engels City Clinical Hospital Number 1, Engels)*; Andrey Litvin *(Immanuel Kant Baltic Federal University, Regional Clinical Hospital, Kaliningrad)*; Vadim Pykhteev *(Territorial hospital #2, Krasnodar)*; Marianna Raevskaya *(AV Vishnevsky Center of Surgery, Moscow)*; Sergey Efetov *(IM Sechenov First Moscow State Medical University, Moscow)*; Aleksandr Butyrskii *(Municipal Emegency Hospital No.6, Simferopol)*. |
| **Saudi Arabia**: Mushabab Alshahrani *(aseer central hospital, abha)*; Azah Althumairi *(Ahsa - King Abdulaziz National Guard Hospital, Al)*; Nasser Alzerwi *(Majmaah - King Khalid General Hospital, Al)*; Ahmed Al Ameer *(King Abdullah Hospital, Bisha)*; Ahmed Al Ameer *(Maternity and Children’s Hospital, Bisha)*; Tariq Madkhali *(King Fahad Specialist Hospital, Dammam)*; Abddulrahman Saleh Almulhim *(King Fahad Hospital Hofuf, Hofuf)*; Salman Ghazwani *(King Fahd Central Hospital, Jazan University, jazan)*; Abdu Ayoub *(Prince Mohd Bin Nassir Hospital, Jazan)*; Othman Iskander *(Sabia General Hospital, Jazan)*; Mohammed Ghunaim *(International Medical Center, Jeddah)*; Mohammed Alharthi *(King Abdulaziz University Hospital, Jeddah)*; Turki M Alzaidi *(King Fahad Armed Forces Hospital, Jeddah)*; Azah Althumairi *(King Khalid National Guard Hospital, Jeddah)*; Mohammad Alyami *(King Khalid Hospital, Najran)*; Abdulrahman Al Amri *(Najran university hospital, Najran)*; Azah Althumairi *(King Abdulaziz Medical City, Riyadh)*; Abdullah AlFakhri *(King Fahad Medical City, Riyadh)*; Amal Alhefdhi *(King Faisal Specialist Hospital, Riyadh)*; Sharfuddin Chowdhury *(King Saud Medical City, Riyadh)*; Thamer Nouh *(King Saud University, Riyadh)*; Ameen Alshehri *(Prince Mohammed bin Abdulaziz Hospital, Riyadh)*; Abdulrahman Alzahrani *(Security Forces Hospital, Riyadh)*; Dr Yousef Alalawi *(King Salman Armed Forces Hospital, Tabuk)*; Selmy Awad *(King Faisal Medical Complex, Taif City)*. |
| **Senegal**: Ibrahima Konate, Abdourahmane Ndong, Jacques Tendeng *(Louis - Saint-Louis Regional Hospital, Saint)*. |
| **Singapore**: Nan Zun Teo *(Changi General Hospital, Singapore)*; Frederick Koh *(Sengkang General Hospital, Singapore)*. |
| **Slovenia**: Jurij Aleš Košir *(University Medical Centre, Ljubljana)*; Uros Bele *(University Medical Centre, Maribor)*. |
| **Somalia**: Sabra Aqil *(Hargeisa Group Hospital, Hargeisa)*. |
| **Spain**: Cristina Barrena López, Ana Sánchez Mozo *(Complejo hospitalario universitario de Albacete, Albacete)*; Antonio Rodriguez Infante *(San Agustín University Hospital, Avilés)*; Patricia Caja Vivancos, Mikel Prieto *(Hospital Universitario Cruces, Barakaldo)*; Igor Alberdi San Roman *(San Eloy Hospital, Barakaldo)*; Laura Gomez Fernandez *(Consorci Sanitari de Terrassa, Barcelona)*; Josep Maria Muñoz Vives *(Fundació Althaia - Xarxa Assistencial Universitària de Manresa, Barcelona)*; Anna Carreras-Castañer, Berta Díaz-Feijoo, Ramon Sieira-Gil, Victor Turrado-Rodriguez *(Hospital Clinic Barcelona, Barcelona)*; Anna Sánchez López, Santiago Sánchez-Cabús *(Hospital de la Santa Creu i Sant Pau, Barcelona)*; Marta Jimenez Toscano *(Hospital del Mar, Barcelona)*; MªPilar Canals Sin, Saura García Laura, Oriol Martin Sole, Pedro Palazon Bellver, Sonia Pérez-Bertólez, Jordi Prat-Ortells, Mireia Riba Martínez, Josep Rubio-Palau, Xavier Tarrado *(Hospital Sant Joan de Deu, Barcelona)*; Jorge Nuñez *(Hospital Universitario Mutua de Terrassa, Barcelona)*; Veronica Alonso Mendoza, Coro Bescós, Eloy Espin-Basany, Martin Espinosa-Bravo, Daniel Gil-Sala, Susana González-Suárez, Nuria Montferrer Estruch, Jorge Nuñez, Lucia Porteiro Mariño, Ana Rodríguez-Tesouro, Fabian Rojas Portilla, M Pilar Tormos Pérez, Inmaculada Vives *(Vall d’Hebron University Hospital, Barcelona)*; Unai Garcia De Cortazar, Kiara Tudela *(Hospital Universitario de Basurto, Bilbao)*; Aitor Landaluce-Olavarria *(Hospital Urduliz, Bizkaia)*; Mercedes Estaire Gómez *(Hospital General Universitario de Ciudad Real, Ciudad Real)*; Jorge Almoguera *(Hospital Universitario de Fuenlabrada, Fuenlabrada)*; Bakarne Ugarte-Sierra *(Usansolo - Hospital Universitario de Galdakao, Galdakao)*; Virginia Jimenez *(Getafe University Hospital, Getafe)*; Marta Bertrand, Laura Cárdenas Puiggrós, Olga Delisau-Puig, Jorge Garcia-Adamez, David Julià Bergkvist, Eloy Maldonado-Marcos *(Hospital Universitari de Girona Dr. Josep Trueta, Girona)*; Lucia Diego García *(Hospital Universitario de Guadalajara, Guadalajara)*; Marta Roldón Golet *(Hospital General San Jorge, Huesca)*; Iván Soto-Darias *(Complejo Hospitalario Universitario Insular-Materno Infantil, Las Palmas de Gran Canaria)*; Aida Cristina Rahy-Martín *(Hospital Universitario de Gran Canaria Doctor Negrín, Las Palmas de Gran Canaria)*; Diego Enjuto, Adolfo Ramos-Luengo *(Severo Ochoa University Hospital, Leganés)*; Juan Delgado Fernandez, Carolina Lugo Duarte, Cristina Ojeda Thies *(12 de Octubre University Hospital, Madrid)*; Lucila Marquez *(Hospital Central de la Cruz Roja San Jose y Santa Adela, Madrid)*; Diana Crego Vita *(Hospital Central De La Defensa Gomez Ulla, Madrid)*; Jana Dziakova *(Hospital Clinico San Carlos, Madrid)*; Ana Maria Minaya Bravo *(Hospital del Henares, Madrid)*; Jorge Caño Velasco, Olga Mateo-Sierra, Begoña Quintana-Villamandos, Cristina Rey Valcarcel, Javier Rio *(Hospital General Universitario Gregorio Marañón, Madrid)*; Laura Román García de León *(HOSPITAL PUERTA DE HIERRO MAJADAHONDA, Madrid)*; Marcello Di Martino, Jorge Prada *(Hospital Universitario de la Princesa, Madrid)*; Javier Serrano González *(Hospital Universitario de Torrejón de Ardoz, Madrid)*; Manuel Losada *(Hospital Universitario del Sureste, Madrid)*; Jose Tomas Castell Gomez, Ramon Corripio-Sanchez, Alexander Forero-Torres, José Manuel Morales-Puebla, Hanna Perez-Chrzanowska, Santiago Valderrabano Gonzalez, Alvaro Yebes, Ignacio Zapardiel *(Hospital Universitario la Paz, Madrid)*; Manuel Diez Alonso, Nelson Morales Palacios *(Hospital Universitario Principe de Asturias, Madrid)*; Alberto Cabañero Sánchez, Fátima Sánchez Fernández *(HOSPITAL UNIVERSITARIO RAMON Y CAJAL, Madrid)*; Alfredo Abad Gurumeta, Ane Abad-Motos, Fernando Corella, Javier Ripollés-Melchor, Rosa Sanz-Gonzalez *(Infanta Leonor University Hospital, Madrid)*; Marta Alcaraz Fuentes *(HOSPITAL SANITAS LA ZARZUELA, MADRID)*; Maria Teresa Fernández Martín *(Hospital Medina del Campo, Medina del Campo (Valladolid))*; Pablo Calvo Espino *(Hospital Universitario de Móstoles, Móstoles)*; Milagros Carrasco Prats, Antonio-José Fernández-López, Damián García Escudero, Vanesa Garcia Soria, Jesús Aarón Martínez Alonso, Miguel Ruiz-Marín *(Hospital General Reina Sofía, Murcia)*; Beatriz Gómez Pérez, Joaquin Moya-Angeler *(Hospital Universitario Virgen de la Arrixaca, Murcia)*; Daniel Fernández Martínez *(Hospital Universitario Central de Asturias (HUCA), Oviedo)*; Heura Llaquet Bayo *(Hospital de Palamós, Palamós)*; Enrique Colás-Ruiz *(Hospital Universitario Son Llàtzer, Palma de Mallorca)*; Susana Bella Romera, M.teresa Gavaldà Pellicé, Misericòrdia Jordà Solé, Enrique Jose Ruiz Velasquez *(Hospital Universitari Sant Joan, Reus)*; Bernardo Núñez *(Corporación Sanitaria Parc Taulí, Sabadell (Barcelona))*; Raul Jimenez, Jon Zabaleta *(Hospital Universitario Donostia, San Sebastian)*; Maria Jose González-Gimeno, Irene Ortega Vázquez, Antonio Perez Ferrer *(Infanta Sofía University Hospital, San Sebastian de Los Reyes)*; Rubén Martín-Láez, Marcelo Moreno Suarez *(Marqués de Valdecilla University Hospital, Santander)*; Miguel Angel Freiria Eiras *(Hospital HM Rosaleda, Santiago de Compostela)*; Irene Ramallo-Solís *(Hospital Universitario Virgen del Rocio, Sevilla)*; Juan-Carlos Gomez-Rosado *(Hospital Universitario Virgen Macarena, Seville)*; Jose Ramon Oliver Guillen *(Hospital Santa Bárbara, Soria)*; Mar Achalandabaso Boira *(Hospital Universitari De Tarragona Joan XXIII, Tarragona)*; Juan Carlos Catalá Bauset *(Consorcio Hospital General Universitario, Valencia)*; Julio Domenech *(Hospital Arnau de Vilanova, Valencia)*; Rafael Badenes *(Hospital Clínico Universitario de Valencia, Valencia)*; Juan Carlos Bernal-Sprekelsen *(Hospital Universitario Doctor Peset, Valencia)*; Jorge Sancho-Muriel *(Hospital Universitario y Politécnico La Fe, Valencia)*; Beatriz De Andrés-Asenjo *(Hospital Clínico Universitario de Valladolid, Valladolid)*; Francisco J Tejero-Pintor *(Hospital Universitario Río Hortega, Valladolid)*; Marc Vallve-Bernal *(Hospital Insular Nuestra Señora de Los Reyes, Valverde)*; Alba Vazquez Melero *(Gasteiz - Hospital Universitario Araba, Vitoria)*; Laura Sánchez Blasco *(Hospital General de la Defensa, Zaragoza)*; Jorge Escartin *(Hospital Royo Villanova, Zaragoza)*; Victoria Duque Mallén *(Hospital Universitario Miguel Servet, Zaragoza)*. |
| **Sri Lanka**: Selvaratnam Srishankar *(Teaching Hospital Anuradhapura, Anuradhapura)*; Umesh Jayarajah, Charitha Sooriyabandara *(District General Hospital Chilaw, Chilaw)*; Oshan Basnayake *(Cancer Institute Maharagama, Colombo)*; Nalaka Gunawansa, Dakshitha Wickramasinghe *(National Hospital of Sri Lanka, Colombo)*; Prabuth dulanjan Weeraddana *(Base Hospital Horana, Sri Lanka, Horana)*; Thanusan Vimalakanthan *(Teaching Hospital,Jaffna, Jaffna)*; Shanthamoorthy Gishanthan *(District General Hospital Polonnaruwa, Polonnaruwa)*; Pramodh Chandrasinghe *(North Colombo Teaching Hospital, Ragama)*. |
| **Switzerland**: Eleftherios Gialamas, Marc-Olivier Sauvain *(Hopital de Pourtales, Neuchatel)*. |
| **Syrian Arab Republic**: Ahmad Ghazal *(Aleppo University Hospital, Aleppo)*; Yusra Al-Sabbagh *(Al-Assad University Hospital, Damascus)*; Turki Alhassoun *(Al-Bairouni University Hospital, Damascus)*; Sara Maa Albared *(Al-Hilal Hospital, Damascus)*; Antoine Naem *(Al-Mouwasat University Hospital, Damascus)*; Hareth Alnahr *(Damascus Hospital, Damascus)*; Ghassan Jisry *(Obstetrics and gynaecology University hospital, Damascus)*; Ali Hammed *(Tishreen University Hospital, Latakia)*. |
| **Turkey**: Arda Isik *(Erzincan University Hospital, Erzincan)*. |
| **Uganda**: Okedi Francis Xaviour *(Adjumani General Hospital, Adjumani)*; Gaston Turinawe *(Hoima regional referral hospital, Hoima)*; Isaac Mubezi *(Iganga district hospital, Iganga)*; Franck K. Sikakulya *(Kampala International University Teaching Hospital, Ishaka)*; Andrew Kakeeto *(Jinja regional referral hospital, Jinja)*; Dr. Wilberforce M. Kabweru, Hervé Monka Lekuya *(Mulago Referral hospital, Kampala)*; Ronald Kiweewa *(St Francis Hospital Nsambya, Kampala)*; Herman Lule *(Kiryandongo Hospital, Kigumba)*; Paul Matovu *(Kisiizi Hospital, Rukungiri)*; Otolia Isaac *(Soroti Regional Referral Hospital, Soroti)*. |
| **United Arab Emirates**: Mohamed Mashhour *(Mediclinic Alnour Hospital, Abu Dhabi)*; Amin El Helw *(Sheikh Shakhbout Medical City, Abu Dhabi)*; Sattar Alshryda, Safeena Kherani, Awadelkarim Mohamed *(Al Jalila Children’s Speciality Hospital, Dubai)*; Ferial Mohamed Ali Abbas *(Dubai Hospital, Dubai)*; Diary Mohammed *(Latifa Women and Children Hospital, Dubai)*; Ehab Aldlyami *(Medcare Orthopaedic and Spine, Dubai)*; Rakesh Kundra, Mohamed Mashhour *(Mediclinic City Hospital Dubai, Dubai)*; Mohamed Mashhour *(Mediclinic Wellcare Hospital, Dubai)*; Antony Louis Rex Michael *(Neurospinal Hospital, Dubai)*; Hayder Alsaadi *(Rashid Hospital, Dubai)*; Kareem S. Khalil *(Al-Qassimi hospital, Sharjah)*. |
| **United Kingdom**: Rachel Dbeis, Shafaque Shaikh *(Aberdeen Royal Infirmary, Aberdeen)*; Jenny Ferry *(Nevill Hall Hospital, Abergavenny)*; Aiman Jamal, Haleema Siddique *(Stoke Mandeville, Wycombe General, Aylesbury)*; Rishi Das, Nikhil Ponugoti *(Basingstoke and North Hampshire Hospital, Basingstoke)*; Sivesh K Kamarajah *(Queen Elizabeth Hospital Birmingham, Birmingham)*; Pornjittra Rattanasirivilai, Asma Sultana *(East Lancashire Hospitals NHS Trust, Blackburn)*; Frances Mosley *(Bradford Royal Infirmary, Bradford)*; Matthew Chan *(Southmead Hospital, Bristol)*; Antony Bateman, Gareth Davies-Jones *(University Hospitals of Derby and Burton, Burton upon Trent)*; Fanourios Georgiades, Grant D. Stewart *(Addenbrooke’s Hospital, Cambridge)*; Navid Ahmadi, Aman Coonar *(Royal Papworth Hospital, Cambridge)*; Mariam Baig, Chetan Khatri, Arthika Surendran *(University Hospitals Coventry and Warwickshire NHS Trust, Coventry)*; Julian Sonksen *(Russell’s Hall Hospital, Dudley)*; Robert Sinnerton *(Ninewells Hospital, Dundee)*; Caitlin Brennan *(Royal Infirmary of Edinburgh, Edinburgh)*; Gemma Faulkner, Michael Greenhalgh *(Royal Bolton Hospital, Farnworth)*; Hannah Emerson, Kiran Singisetti *(Gateshead Health NHS Foundation Trust, Gateshead)*; Joshua Totty *(Hull University Teaching Hospitals NHS Trust, Hull)*; Michael Wilson *(Forth Valley Royal Hospital, Larbert)*; Terence Lo *(St James’s University Hospital Leeds, Leeds)*; Harriet Corbett, Ijeoma Okonkwo *(Alder Hey in the Park, Liverpool)*; Gill Arbane, Kariem El-Boghdadly *(Guy’s and St Thomas’ Hospitals, London)*; Cyrus Kerawala *(The Royal Marsden NHS Foundation Trust, London)*; Chetan Parmar *(The Whittington Hospital, London)*; Tom Abbott, Michael Bath, Funlayo Odejinmi *(Whipps Cross University Hospital, London)*; Jayesh Sagar, Rishi Talwar *(Luton and Dunstable University Hospital, Luton)*; Samuel Newman *(Royal Lancaster Infirmary, Morecambe)*; John Hammond, John Moir *(Newcastle Upon Tyne Hospitals NHS Foundation Trust, Newcastle upon Tyne)*; Natalie Duric, Tamas Szakmany *(Royal Gwent Hospital, Newport)*; Ahmar Iftikhar Talib, Mina Youssef *(Norfolk and Norwich University Hospital, Norwich)*; Christopher Lewis-Lloyd *(Nottingham City Hospital, Nottingham)*; Christopher Lewis-Lloyd *(Queens Medical Centre, Nottingham)*; Mariam Lami *(John Radcliffe Hospital, Oxford)*; Khurram Ayub, Benjamin Dean *(Nuffield Orthopaedic Centre, Oxford)*; Supriya Balasubramanya, Sathya Lakpriya, Luke Rogers *(Derriford Hospital, Plymouth)*; Paul Turner *(Royal Preston Hospital, Preston)*; Mark Maher *(Royal Berkshire Hospital, Reading)*; Kohila Sigamoney *(Salford Royal Hospital, Salford)*; John Edwards *(Sheffield Teaching Hospital NHS Foundation Trust, Sheffield)*; Jihène El Kafsi, John Hardie *(Frimley Health NHS FT - Wexham Park, Slough)*; David Johnson *(Stepping Hill Hospital, Stockport)*; Christin Henein *(Sunderland Eye Infirmary, Sunderland)*; Marianne Hollyman *(Musgrove Park Hospital, Taunton)*; Ketan Agarwal, Simon Powell *(Wirral University Teaching Hospital, Wirral)*; Govind Singh Chauhan *(Royal Wolverhampton NHS Trust, Wolverhampton)*. |
| **United States**: Rakesh Patel *(Johnston Memorial Hosptital, Abingdon)*; Joel Gagnier *(University Of Michigan Medical Center, Ann Arbor, MI)*; Heather Carmichael *(University of Colorado Anschutz Medical Campus (CU Anschutz), Aurora)*; Kristofor A. Olson *(Dell Seton Medical Center at the University of Texas, Austin)*; Eric Etchill *(Johns Hopkins Hospital, Baltimore, MD)*; Joseph Incorvia *(Boston Children’s Hospital, Boston)*; Sameer Hirji *(Brigham and Women’s Hospital, Boston)*; Matthew Naunheim *(Massachusetts Eye and Ear, Boston)*; Frederick Drake *(Boston Medical Center, Boston, MA)*; Haytham Kaafarani *(Massachusetts General Hospital, Boston, MA)*; Caroline Reinke *(Atrium Health Carolinas Medical Center, Charlotte)*; Anna Alecci *(Rush University Medical Centre, Chicago, IL)*; Dennis Vaysburg *(University of Cincinnati Medical Center, Cincinnati)*; Jennifer Rodriquez *(Memorial Hospital, Colorado Springs)*; Emily Shih *(Baylor University Medical Center, Dallas)*; Vin Shen Ban *(University of Texas Southwestern, Dallas)*; Julia Coleman *(Denver Health, Denver)*; Henry E. Rice *(Duke University Medical Center, Durham, NC)*; Krista Kaups *(University of California San Francisco (UCSF) - Fresno, Fresno)*; Emmanouil Giorgakis *(University of Arkansas for Medical Sciences, Little Rock)*; Maggie DiNome *(University of California Los Angeles, Los Angeles)*; Neal Bhutiani *(University of Louisville Hospital and Norton Hospital, Louisville)*; Omar Alnachoukati *(Medical Center of the Rockies, Loveland, Colorado)*; Brittany Bankhead-Kendall *(University Medical Center Lubbock, Lubbock, Texas)*; Taylor Aiken, Thomas Diehl *(University of Wisconsin, Madison)*; Ankush Gosain *(Le Bonheur Children’s Hospital, Memphis, MA)*; Rishi Rattan *(University of Miami Hospital, Miami, FL)*; Muhammad Owais Abdul Ghani *(Vanderbilt University Medical Center, Nashville)*; Nensi Melissa Ruzgar *(Yale New Haven Hospital, New Haven, CT)*; Anna Liveris *(New York City Health and Hospitals - Jacobi Medical Center, New York)*; Nina Glass *(The University Hospital, Newark, NJ)*; Charu Paranjape *(Newton Wellesley Hospital, Newton, MA)*; Theresa Chin *(University of California Irvine, Orange)*; Antonio Meola *(Stanford Health Care, Palo Alto, CA)*; Kristina Nicholson *(University of Pittsburgh Medical Center, Pittsburgh, PA)*; John Squiers *(Baylor Scott & White The Heart Hospital, Plano, Texas)*; Stephanie Lueckel *(The Rhode Island Hospital, Providence, RI)*; Janani Reisenauer *(Mayo Clinic, Rochester, MN)*; Rachael Callcut *(UC Davis Medical Center, Sacramento)*; Ahmed Mansour *(UT Health San Antonio (UTHSA), University Hospital (UHS), San Antonio)*; Allison Berndtson *(University of California San Diego, San Diego)*; Lucy Kornblith *(University of California San Francisco, UCSF (use separate entry for Fresno), San Fransisco)*; Sara Seegert *(ProMedica Toledo Hospital, Toledo)*; Paulo Martins *(UMass Memorial Hospital, Worcester, MA)*. |
| **Yemen, Rep.**: Hamza Al-Naggar, Mohammed Al-Shehari *(Al-Thawra Modern General Hospital, Sana’a)*; Ibrahim Al-Raimi *(University of Science & Technology Hospital, Sana’a)*. |
| **Zimbabwe**: Allan Ngulube *(Mpilo Central Hospital, Bulawayo)*; Maphios Siamuchembu *(United Bulawayo Hospitals, Bulawayo)*; Willard Mushiwokufa *(Gweru Provincial Hospital, Gweru)*; Busisiwe Mlambo *(Parirenyatwa hospital, Harare)*; Simbarashe Chinyowa *(Sally Mugabe Central Hospital, Harare)*. |

#### Local Collaborators:

| **Albania**: Ervis Agastra *(Regional Hospital of Durres, Durres)*; Enton Bollano, Kostandin Gjyli, Dariel Thereska *(University Hospital Center Nene Tereza, Tirana)*; Irida Dajti, Jola Kerpaci, Enxhi Vrapi, Lorena Zijaj *(University hospital Koco Gliozheni, Tirana)*. |
| --- |
| **Algeria**: Bahraoui Djahida, Belabbes Fatima zohra, Kouidri Khadidja *(Ibn sina, Adrar)*; Oussama Bali, Nassim Benallel, Ismahene Lalmi *(Public hospital establishment of Sig, Mascara)*; Meriem Abdoun, Nesrine Aouabed, Souad Bouaoud, Kamel Bouchenak, Assia Haif, Zineddine Soualili *(Saadna Mohamed Abdnour (Abdnoor) Teaching Hospital, Setif)*. |
| **Argentina**: Joaquin Bastet, Agustín Bianco, Daniel Capitaine, Jorge Centeno Lozada, Carlos Esquivel, Rodrigo Figueroa, Manuel Garcia, Pablo Martín García, José Ignacio Gerchunoff, Fernando Martinez lascano, Jose Mondino, María Emilia Muriel, Carmignani Pablo, Martin Passadore, Alejandra Tornini, Rogelio Traverso, Lara Vargas, Gerardo Zanoni *(Sanatorio Allende - Sede Cerro, Allende, Cordoba)*; Norberto Berber, Estefanía Cotta, Marcela Di Vincenzo, Esteban sebastian Gallino, Cecilia Gigena, Bianca Grassano, Veronica Laudani, Ignacio Lugones, Constanza Madrid, Maximiliano Maricic, Antonio Alberto Martinez, Andres Rosso, Pablo Scher, Sofia Tachella, Damaris Idara Anabel Zezular *(Hospital General de Niños Pedro de Elizalde, Buenos Aires)*; Carla Abuawad, Agustin Albani Forneris, Carola Allemand, Laura Gisela Alvarez Calzaretta, Luis Boccalatte, Jorge G. Boretto, Rocio Boudou, Rodrigo Brandariz, Martin Buljubasich, Arturo Burchakchi, Martin Buttaro, Juan Pablo Campana, Virginia Cano Busnelli, Tomas Carminatti, Agustina Florencia Castro Lalín, Julian Cereghini, María Sol Crespi Amor, Roberto Sebastián Croattini, Maria Sol Fernandez, Marcelo Figari, Uriel Fraidenraij, Diego Gallegos, Agustín Maria García-Mansilla, Marcos Gonzalez, Matias Ignacio Gonzalez, Esteban Gonzalez Salazar, Jeremias Goransky, Guillermo Hernandez Gauna, Felipe Higuera, Fernando Holc, Esteban Gabriel Jauregui, Juan Larrañaga, Juan Liyo, Lionel Llano, Pablo Lobos, Emilia Luzzi, Gustavo Mastroianni, Santiago Miguel Mata-Suarez, Horacio F. Mayer, Santiago Mc Loughlin, Efrain Mendoza, Ricardo Esteban Mentz, Pedro Mercado, Pablo Moyano, Florencia Noll, Diego Odetto, Agustina Rene Oliva, Rafael Perez Vidal, Catalina Poggi, Eduardo Jorge Premoli, María Lourdes Ramos, Patricio Rosas, Luciano Rossi, Gustavo Rossi, Jose Saadi, Jordán Scherñuk, Pablo Slullitel, María Verona Stang, María Victoria Taboada, Sebastian Tirapegui, Constanza Uffelmann, Carlos Vaccaro, Roberto Vagni, Ana Clara Valerio, Celeste Soledad Zarratea *(Hospital Italiano de Buenos Aires, Buenos Aires)*; Carina Chwat, Silvina Montal, Brian Morris, Pedro Valdez *(Hospital Universitario Austral, Buenos Aires)*; Fernando Diaz-Couselo, Ivana Ines Pedraza Salazar, Luciana Sabatini *(Instituto Oncológico Alexander Fleming, Ciudad Autónoma Buenos Aires)*; Fernando Andres Alvarez, Micaela Avila, Nicole Benitez Benitez, Nicolás Bruera, Marcelo Doniquian, Manuel Gielis, Julian Liaño, Florencia Llahi, Facundo Mandojana, Walter Páez, Diana Alejandra Pantoja Pachajoa, Matias Parodi, Héctor Picon molina, Agustin Pinsak, German Viscido *(Clinica Universitaria Reina Fabiola, Cordoba)*; Roberto Badra, Diego Belisle, Hernan Borla, Georgina Eberle, Agustin Esteban, Carlos Ignacio Ferrero, Micaela Furlan, José Sebastian García, Lucas Granero, Mariel Henzenn, Rodrigo Juaneda, Sergio Martin Lucchini, Esteban Politi Vidal, Guillermo Romero Reyna, José Gabriel Yaryura Montero *(Sanatorio Allende - Sede Nueva Cordoba, Cordoba)*; Maria Mercedes Caubet, José Luis D’Addino, Agustin Duro *(Hospital Prof Dr Bernardo A Houssay, Provincia de Buenos Aires)*; José Alfredo Calderón Arancibia *(Hospital Público Materno Infantil de Salta, Salta)*. |
| **Aruba**: Deepu Daryanani, Martijn Gosselink, Alex Ponson *(Dr. Horacio E Oduber Hospital, Oranjestad)*. |
| **Australia**: Mohamed Afzal, Mathew Amprayil, Mark Brooke-Smith, John Chen, James Grantham, Benjamin Gricks, Amanda Hii, Nikhil Kundu, David Liu, Charles Livingston, Matthew Marshall-Webb, Christina McVeay, Hamish Moore, Gavin Nair, Bee Shan Ong, Dominic Parker, Victoria Rudolph-Stringer, Malgorzata Szpytma, Ravi Vissapragada, Melissa Yun Wee, Geoffrey Yuet Mun Wong, Xuanyu Zhou *(Flinders Medical Centre, Adelaide)*; Dylan Richard Barnett, Melissa Bochner, John Bolt, Karel Buddingh, Brendon Coventry, Sean Davis, Joseph Dawson, Stuart Denham, Christopher Dobbins, Nagendra Dudi-Venkata, Robert Fitridge, Siang Wei Gan, Izhar-Ul Haque, Matheesha Herath, Shivangi Jog, Harsh Kanhere, Hidde M Kroon, Beatrice Kuang, Yick Ho Lam, Virginia Lambert, Alicia Lim, Grace Maina, Cea-Cea Moller, Eu Nice Neo, Eu Ling Neo, Alain Nguyen, Shalvin Prasad, Jennifer Roy, Christine Russell, Tarik Sammour, Nicholas Smith, Richard Smith, Conrad Stranz, Saam Tourani, Steven Tran, Lucinda Van de Ven, Leigh Warren, Robert Whitfield, Jamie Wormald, Yijie Yin *(Royal Adelaide Hospital, Adelaide)*; Christopher Bierton, Benjamin Cribb, Anthony Farfus, Katarina Foley, Daniel Ong, Matthew Watson *(The Queen Elizabeth Hospital, Adelaide)*; John Barker, Jacob Hampton, James Karam, Anya Rugendyke, Tyson Zhang *(Armidale Rural Referral Hospital, Armidale)*; Carolyn Vasey, Eleanor Watson *(Ballarat Base Hospital, Ballarat)*; Eduardo Apellaniz, Adam Frankel, Lara Gahan, Glen R Guerra, Amanda Liesegang, Nicholas Lutton, Andrew Maurice, Scott Miron, Aveechal Prasad, Ashleigh Sercombe, Thanusan Sivapalan, Syed Danial Syed Ahmad, Chin Li Tee, Lachlan Yaksich *(Princess Alexandra Hospital, Brisbane)*; Martin Batstone, Omar Breik, Thomas Young *(Royal Brisbane and Women’s Hospital, Brisbane)*; Michael Field, Benjamin Scott, Sean Stevens *(Colac Area Health, Colac)*; Gabriella Charlton, Si Chen, Henry Yan Chi Cheung, Neha Gauri, Ross Hayhurst, Sophie James, Seyoung Jang, Fangzhi Jia, Helen Zhang *(Concord Repatriation General Hospital, Concord West, Sydney)*; Randeep Aujla, Michael Finsterwald, Digby Percy, Alvin Tanaya, Anand Trivedi, Uyen Giao Vo *(Fremantle Hospital, Fremantle, Western Australia)*; Andrew Aylett, Richard Grills, Angela Holmes, Matthew Ming Kei Kwok, Anton Lambers, Leo Lambers, Haider Latif, Evania Lok, Sonal Nagra, Richard Page, Nicholas Paltoglou, Vishwakar Panuganti, Sophie Riddell, Gareth Rudock, Hannah Tan, Kirk Underwood, David Watters, Daniel Youssef *(University Hospital Geelong, Geelong)*; Ricci Amoils, Nazli Bahtigur, Savitha Bengeri, Lily Builth-Snoad, Hock Ping Cheah, Paul Chen, Kenneth Chew, Alyssa Chong, Sara Clark, Rhea Darbari Kaul, Amanda Caroline Dawson, Eliya Devan, Christina Ewington, Aleeza Fatima, Sophia Fitt, John Gaul, Paloma Ghosal, Amy Gojnich, Indu Gunawardena, Peter Hamer, Samuel Holmes, Zhen Hou, Danielle Jolly, James Dimitri Kane, Micayla Kaufman, Tanishq Khandelwal, Sukhwant Khanijaun, Kelvin Kwok, Edward Latif, Sharon Laura, Vivian Lee, Catherine Leung, Ina Liang, Peter Lin, Elizabeth Weng Yan Lun, Haili Luo, Victor Ly, Jolande Ma, Brooke Macnab, Richard McGee, Stephanie Miles, William Munro, Harry Narroway, Tin Yau Ngan, Anthony Noor, Upuli Pahalawatta, Melissa Park, Cameron Parkin, Rita Poon, Shanmugam S Somasundaram, Claudia Saab, Renwick Simpson, Dana Steinel, Emily Taylor, Adrian Tchen, Shu Thong, Kelvin Tran, Kevin Tree, Lucille Vance, Ken Wong, Victor Yu, Michael Zhang *(Gosford Hospital, Gosford)*; Aram Cox, David Chi Hau Tan, Nova Thani *(Royal Hobart Hospital, Hobart)*; Shefali Das, Taigh Macdonald, Paul Salama, Gagandeep Sandhu, Rachel Shadbolt *(Joondalup Health Campus, Joondalup, Western Australia)*; Du Phan, David Townend *(Lismore Base Hospital, Lismore)*; Alwin Chuan, Eunmaro Ju, Sang mi Lee *(Liverpool Hospital, Liverpool)*; Stephen Barnett, Daniel Cox, Hajar Hasan Kheslat, Andrew Higgins, Chloe Jamieson-Grigg, Victoria Jenkins, Brett Larner, Vijayaragavan Muralidharan, Marcos Perini, David Proud, Kirby Qin, Georgina Riddiough, Sivendran Seevanayagam, Damien M. Wu, Chris Zhao *(Austin Hospital, Melbourne)*; Sonali Aggarwal, Vinna An, Melanie Battershell, Elliot Chan, King Tung Cheung, Anna Drake, Janindu Goonawardena, Benjamin Hunt, Christopher Ip, Vikram Iyer, Anshini Jain, Joshua Kealey, Chen Lew, Christopher Seng Hong Lim, Tara Luck, Sean Mackay, Natalie Maher, Georgia Maroske, Nicholas Roubos, Shomik Sengupta, Christopher Steen, Zac Tsigaras, Zachary Tuttle, Salena Ward, Marli Williams, Enoch Wong *(Box Hill Hospital, Melbourne)*; Travis Ackermann, Evie Yeap, Jessie Zhou *(Casey Hospital, Melbourne)*; Amy Coates, Nora Mutalima, Ton Tran *(Dandenong Hospital, Melbourne)*; Ramesh Nataraja, Maurizio Pacilli, Claire Sharpin *(Monash Childrens Hospital, Melbourne)*; David Bird, Kay Tai Choy, Sarah Condron, Jurstine Daruwalla, Isabela dos Anjos, Hanna El-Khoury, Robert Fabian, Andrew Gillard, Rebecca Greenop, Russell Hodgson, Michael Issa, Sharon Lee, Krinal Mori, Nikki Petrakis, Maryum Qureshi, Danielle Sabella, Prassannah Satasivam, Niranjan Sathianathen, Sean Ezekiel Seow, Amanda Shen, Margaret Shi, Meher Tabassum, Rodrigo Teixeira, Josephine Vivian-Taylor, Jennifer Wheatley *(Northern Hospital, Melbourne)*; Alexander Heriot, Sally Shepherd, Mikael Soucisse *(Peter MacCallum Cancer Centre, Melbourne)*; Sebastian King, Brendan O’Connor, Warwick Teague *(Royal Children’s Hospital, Melbourne)*; Simon Banting, Lynn Chong, Peter Choong, Sharnel Clatworthy, Angela Cochrane, Adrian Fox, Michael Wei Hii, Anna Isaacs, Mary Ann Johnson, Brett Knowles, Andrew Newcomb, Veronique Price, Jaishankar Raman, Matthew Read, Alistair Rowcroft, Lillian Taylor, Salena Ward, Gavin Wright *(St Vincent’s Hospital, Melbourne)*; Wendy Brown, Prem Chana, Kalai Shaw *(The Alfred Hospital, Melbourne)*; Jacob Bock, Jordan Cory, Katharine Drummond, Jack Lahy, Surjit Lidder, Thomas McIntire, Benjamin Price, Claire Stark *(The Royal Melbourne Hospital, Melbourne)*; Alex Besson, Richard Gartrell, Brianne Lauritz, Ha My Ngoc Nguyen, Chui Foong Ong, Meron Pitcher, Lorna Scullion, Howard Tang, Danielle Taylor, Brigid Wolf, Justin Yeung *(Western Health - Footscray hospital and Sunshine hospital, Melbourne)*; Luke Traeger, Matthew Watson, Matthias Wichmann *(Mount Gambier and Districts Health Service, Mount Gambier)*; Amelia Davis, Amelie Maurel, Kyle Raubenheimer *(Armadale Health Service, Mount Nasura)*; Cassidy Campbell, Ashley Colaco, Maria Julia Corbetta Machado, Abbie Heffernan, Costa Karihaloo, Isabella Ludbrook, Sean SW Park, Bibi Nabeeha Peerally, Guillermo Regalo, Camila Singhai, Heidi Stevens, Sergey Vavilov *(Belmont District Hospital, Newcastle)*; Anne-Marie Aubin, Ralph Gourlay, Margaret Harris, Zhi Ying Lim, Rebecca Spring, Melissa Stieler *(Calvary Mater Newcastle, Newcastle)*; Nicholas Adamson Barnes, Ahmad Seraj Alam, Ali Alsoudani, Francesco Amico, Rebecca Anning, Zsolt J. Balogh, Alison Blatt, Scott Cairns, Jesse Carroll, Andrew Caterson, Adam Christie, Daron Cope, Tyson Dale, Grace Dennis, Anchal Duggal, Brendan Ennis, Mark Fenton, Yi Xin Joanna Fu, Ana Galevska-Dimitrovska, Jonathan Gani, Scott Gelzinnis, Madelyn Gramlick, Claudia Hadlow, Ruth Hardstaff, Munish Heer, Merran Holmes, Bridget Hone, Sophie Hu, Linna Huang, Ramiz Iqbal, Niall Jefferson, Dimithi Kasthurirathne, Stephen Kuo, Wayne Yan Lau, Hsin-Ping Liang, Daniel Lim, Rosalina Lin, Jack McDonogh, Elysse Mcilwain, Yan Joyce Ming, Christine O’Neill, Ryan James Ocsan, Benjamin Oosthuizen, Nicole Organ, James Otieno, Felicity Park, Amanda Paterson, Marisol Perez Cerdeira, Luke Peters, Josefin Petersson, Peter Pockney, Sonia Rubbo, Venesa Siribaddana, Kabilan Thurairajah, Caleb Ting, Antonia Watson, Teagan Way, Elvina Wiadji, Ellisha Willoughby *(John Hunter Hospital, Newcastle)*; Ashley Bailey, Samuel Broadbent, Viswanathan Narayanan, Samuel Stephens, Anna Wilkes, Jie Zhao *(Maitland Hospital, Newcastle)*; Mohammed Ballal, Carl D’Souza, Clara Forbes, Matthew Goss, Monique Haddleton, Jacinda Harty, Dickon Hayne, Nicole Hew, Wee Ling Koh, Shawn Lee, Shahbaz S Malik, Naseer Mohammed Abdul, Jana-Lee Moss, Toby Richards, Yi Th Ng Seow, Anand Trivedi, Uyen Giao Vo, James Wong *(Fiona Stanley Hospital, Perth)*; Leigh Archer, Nur Sabrina Binti Babe Azaman, Tina Dilevska, Jonathan Foo, Tasvinder Hans, Shirley Jansen, Mohit Kumar, Christopher Leeson, Terence Pham, Fernando Picazo Pineda, Saravanan Rajakumar, Supisara Suk-Udom, Jonathan Tan, Benjamin Thurston, Bichen Zhao *(Sir Charles Gairdner Hospital, Perth)*; Shivangi Gupta, Lachlan Hou, Nivedan Jeyamanoharan, James Leigh, Wei Shearn Poh, Michael Sala *(St John of God Midland Public and Private Hospital, Perth)*; Rebecca Crothers, Madhulika Dravid, Ben Harrison, Sumayya Islam, Conor McCartney, Henco Nel *(St John of God Subiaco Hospital, Perth)*; Natasha Pearson *(Goulburn Valley Health Hospital, Shepparton)*; Christos Apostolou, Sarit Badiani, Christophe Berney, Richard Chou, Sam Hanna, Andrew Lam, Vanessa Ma, Salonee Shubhen Phanse *(Bankstown Hospital, Sydney)*; Michael Elliott, Daniel Phung *(Lifehouse, Sydney)*; Jessica Barry, Eleanore Clark-Mackay, Jennifer Cope, Richard Halliwell *(Westmead Hospital, Westmead)*; Ji Chen, Marie Shella De Robles, Alyssa Llorando, Humaira Haider Mahin, Soni Putnis, Dominic Rao, Faisal Syed *(Wollongong Public Hospital, Wollongong)*; Daniel Abulafia, Benjamin Buckland, Timothy Cordingley, Ashe DeBiasio, Andrew Drane, Patrick Ireland, Danielle Jolly, Benji Julien, Lan-Hoa Le, Austin Yeon Suk Lee, Eu Jhin Loh, Andrew Middleton, Brienna Mortimer, Samuel Sebastian, Brooke Short, Peter Stewart, Stephanie van Ruyven, Daniel Wong, Charry Zhang, Bonnie Zhu, William Ziaziaris *(Wyong Public Hospital, Wyong)*. |
| **Austria**: Aran Leitner, Lukas Tolzman, Matthias Zitt *(Krankenhaus der Stadt Dornbirn, Dornbirn)*; Gergely Rakos, György Székely *(Krankenhaus der Barmherzigen Brüder Eisenstadt, Eisenstadt)*; Gabriel Djedovic, Ingmar Königsrainer *(Landeskrankenhaus Feldkirch, Feldkirch)*; Felix Aigner, Caterina Allmer, Barbara Herritsch, Martin Mitteregger, Christian Schauer, Gerald Seitinger, Carmen Siebenhofer, Stefan Uranitsch *(Barmherzige Brüder Krankenhaus, Graz, Graz)*; David Duller, Erwin Mathew, Christoph Skias *(Krankenhaus der Elisabethinen, Graz)*; Alexandros Andrianakis, Armin Belarmino, Petra Brinskelle, Christoph Castellani, Tina Cohnert, Melanie Fediuk, Andrea Fink, Clemens Holzmeister, Judith Kahn, Josip Kresic, Andreas Leithner, Joerg Lindenmann, David Lumenta, Birgit Michelitsch, Saulius Mikalauskas, Sebastian P. Nischwitz, Paul Puchwein, Andrej Roj, Peter Schemmer, Georg Singer, Freyja-Maria Smolle-Juettner, Holger Till, Axel Wolf *(Medical University of Graz, Graz)*; Tibor Oliver Andraschofsky, Rafael Angerer, Daniel Arco, Claudia Kaufmann, Nura Kilic, Peter Widschwendter *(Landeskrankenhaus Hall, Hall in Tirol)*; Reinhard Angermann, Marlies Bauer, Nicole Bergmann, Christian Freyschlag, Martin Gisinger, Can Gollmann-Tepeköylü, Michael Graber, Alexander Haim, Carina Harasser, Bettina Härter, Jakob Hirsch, Markus Hofer, Johannes Holfeld, Anna Lena Huber, Martina Kralinger, Dietmar Krappinger, Irmgard Elisabeth Kronberger, Marlene Kuen, Michael Liebensteiner, Franka Messner, Alex Messner, Felix Naegele, Marijana Ninkovic, Yvonne Nowosielski, Antonia Osl, Cornelia Ower, Leo Pölzl, Teresa Rauchegger, Daniel Reimer, Johannes Riecke, Anton H. Schwabegger, Filipp Sokolovski, Anna Strimmer, Markus Süss, Martin Thaler, Julian Umlauft, Claus Zehetner, Alain G. Zeimet *(Innsbruck Medical University, Innsbruck)*; Jakob Allerstorfer, David Haslhofer, Daniel Hofer, Matthias Luger, Lorenz Pisecky, Nikolaus Poier, Nina Rubicz, Christoph Schmolmüller, Paul Zwittag *(Kepler University Hospital, Johannes Kepler University, Linz)*; Tobias Rossmann, Francisco Ruiz-Navarro, Harald Stefanits *(Kepler University Hospital, Johannes Kepler University of Linz (Neuromed Campus), Linz)*; Hend Elsayed, Peter Habertheuer, Tereza Hajkova *(Konventhospital Barmherzige Brueder, Linz)*; Wolfgang Loidl, Ferdinand Luger, Amadeus Windischbauer *(Ordensklinikum Linz Elisabethinen, Linz)*; Ines Fischer, Reinhold Függer, Patrick Kirchweger, Thomas Saini *(Ordensklinikum Linz GmbH Barmherzige Schwestern, Linz)*; Julian Berger, Wolfgang Fraz, Arastoo Nia *(Landesklinikum Neunkirchen, Neunkirchen)*; Mihaly Kenez, Margit Nichita *(Krankenhaus Oberpullendorf, Oberpullendorf)*; Astrid Magele, Thomas Mayr, Chiara Noe *(Universitätsklinikum Sankt Pölten, Pölten)*; Reinhard Bittner, Kurosch Borhanian, Isabella Dornauer, Klaus Emmanuel, Ana Gabersek, Antonia Gantschnigg, Michael Grechenig, Ricarda Gruber, Jörg Hutter, Tarkan Jäger, Oliver Koch, Michael Lechner, Lisa Manzenreiter, Franz Mayer, Iris Muehlbacher, Jaroslav Presl, Daniel Rezaie, Philipp Schredl, Martin Varga, Michael Weitzendorfer, Angela Wimmer *(Paracelsus Medical University Salzburg, Salzburg)*; Eberhard Brunner, Michael de Cillia, Michaela Gruber, Martin Grünbart, Elmar Heinrich, Hannes Hoi, Vanessa Kemmetinger, Christof Mittermair, Victoria Mosshammer, Christian Obrist, Peter Paal, Judith Roesch, Elisabeth Russe, Tobias Schaetz, Jan Schirnhofer, Karl Schwaiger, Gottfried Wechselberger, Helmut Weiss *(Saint John of God Hospital Salzburg, Salzburg)*; Maximilian Lanner *(Kardinal Schwarzenberg Klinikum, Schwarzach im Pongau)*; Alf-Dorian Binder, Thomas Gürtler *(Universitätsklinikum Tulln, Tulln)*; Marcus Fink, Daniel Reichhold, Radoslava Stoyanova *(Barmherzige Schwestern Krankenhaus Wien, Vienna)*; Klara Beitl, Martin H. Bernardi, Philip Datler, Christopher Dawoud, Alex Farr, Philipp Foessleitner, Christoph Grimm, Felix Harpain, Mir Alireza Hoda, Simone Holawe, Marlene Kranawetter, Johannes Ott, Alexandra Perricos, Maximilian Pesta, Robert Pillerstorff, Christian Reiterer, Stefan Riss, Klara Rosta, Georg Scheriau, Edda Tschernko, Rene Wenzl, Dominik Wiedemann, Peter Wohlrab, Bernhard Zapletal, Matthias Zimmermann, Daniel Zimpfer *(General Hospital of Vienna, Vienna)*; Oliver Findl, Andreea Fisus, Manuel Ruiss *(Hanusch Hospital, Vienna)*; Melanie Komaz, Nikolaus Meindl, Florian Primavesi, Karl Heinz Stadlbauer, Stefan Stättner, Florian Steiner *(Salzkammergut Klinikum Vöcklabruck, Vöcklabruck)*; Ondrej Cerny, Eva Falkensammer, Hans Knotzer, Paul Köglberger, Bernhard Poidinger, Clemens Georg Wiesinger *(Klinikum Wels-Grieskirchen GmbH, Wels)*; Johannes Burtscher, Sebastian Rath, Felipe Trivik-Barrientos *(Landesklinikum Wiener Neustadt, Wiener Neustadt)*. |
| **Azerbaijan**: Gunay Aliyeva, Vuqar Behbudov, Gurbankhan Muslumov, Natiq Zeynalov *(Scientific Center of Surgery named M.Topchubashov, Baku)*. |
| **Bahrain**: Mahmood Alam, Fatema Alfayez, Fayza Haider, Batool Hasan, Maryam Mahdi, Husain Mulla, Kawthar Qader, Amr Saeed, Ahmed Shirazi *(Salmaniya Medical Complex, Manama)*; Aysha Albastaki, Nuha Birido, Hiba Hameed Chagla, Abdulla Dawaishan, Baheya Dawaishan, Abeer Farhan, Isam Juma, Asher Khan, Aqsa Mohammad eqbal Patel, Madhu Srinivasan, Fatema Waheed Akbar Mohamad Akbar Nawab Deen *(King Hamad University Hospital, Muharraq)*; Seemal AbdulQadir, Abdulla Jabr, Fauzia Maqsood *(Awali Hospital, Riffa)*; Noora Almoosa, Mai Naseer *(BDF hospital, West Riffa)*. |
| **Bangladesh**: Mohammed Shadrul Alam, AKM Khairul Basher, Shahnoor Islam *(Bangladesh Medical College Hospital, Dhaka)*; S.M. Nazmul Islam, Sabbir Karim, Ashrarur Rahman Mitul *(Dhaka Shishu (Children) Hospital, Dhaka)*; Muntasir Faisel, Sadia Khan, Iftekhar Ibne Mannan, Nawshin Nazia *(Popular Medical College Hospital, Dhaka)*. |
| **Barbados**: Malissa Bentham, Sasha Corbin, Alex Doyle, Asha Eastmond, Amelia Haynes, Margaret O’Shea, Greg Padmore, Emil Phillips, Keisha Walkes *(Queen Elizabeth Hospital, Bridgetown)*. |
| **Belarus**: Evgeniy L. Artyushkov, Mark L. Kaplan, Vladislav Straltsov *(Gomel City Clinical Hospital No 3, Gomel)*; Yauheniya Litvina, Alexei Lyzikov, Victor E. Tihmanovich *(Gomel Regional Clinical Cardiology Center, Gomel)*. |
| **Belgium**: Mafalda Borges, Tim Brits, Nicolas De Hous, Stefan De Wachter, Niels Komen, Tomas Menovsky, Xavier Mortiers, Dorien Vermeulen, Nils Vleminckx, Dirk Ysebaert *(University Hospital Antwerp, Antwerp)*; Julie Bontinck, Danny Bulthé, Bert Dhondt, Ricky Rasschaert, Anne-Sophie Van Haver *(AZ Rivierenland, Bornem)*; Koen Van Belle, Laurence Verstraeten, Manon Vounckx *(Europe Hospitals, Brussels)*; Serge Cappeliez, Sébastien D’ulisse, Yasmine De Bruyne, Badih El Nakadi, Tessely Heloise, Matteo Luisetto, Sotirios Marinakis, Maxime Maton, Manon Pigeolet, Claire Viste *(CHU de Charleroi, Charleroi)*; Elke Van Daele, Gabrielle van Ramshorst, Mathieu Vandeputte *(University Hospital of Ghent, Gent)*; Marc Duinslaeger, Daniel Jacobs-Tulleneers-Thevissen, Yanina Jansen, Ward Janssens, Rastislav Kunda, Nouredin Messaoudi, Michael Ruyssers, Martijn Schoneveld, Jasper Stijns, Ellen Van Eetvelde, Marian Vanhoeij *(UZ Brussel, Jette)*; Wouter Oosterlinck, Jef Van den Eynde, Raf Van den Eynde *(UZ Leuven, Leuven)*; Ahmed M. Chaoui, Nicolas Flamey *(AZ Delta, Roeselare)*. |
| **Benin**: Marcellin Akpla, Cyrille Kpangon, Souliath Lawani *(Centre Hospitalier Universitaire de Zone de Suru-Lére, Cotonou)*; Hermann Agossou, Hulrich Aouagbe Behanzin, Covalic Bokossa *(Hopital de Menontin, Cotonou)*; Mouhamed Agbadebo, Hubert Dewanon, Hugues Yome *(Hôpital de Zone de Dassa-Zoumè, Dassa-Zoumè)*; Alassan Boukari, Oswald Gbehade, Sèmèvo Romaric Tobome *(Centre Hospitalier Départemental de l’Atacora, Natitingou)*; Labissi Francois Amossou, Francis Dossou, Ismaïl Lawani *(Centre Hospitalier Universitaire et Departemental Oueme Plateau, Porto Novo)*. |
| **Bosnia and Herzegovina**: Tatjana Barišić, Miran Boras, Zdrinko Brekalo, Ivana Čuljak Blagojević, Ana Damjanović, Vedran Dragisic, Ana Dugandžić Šimić, Filip Gunaric, Martin Kajic, Darko Knežević, Tanja Krešić, Valentina Lasić, Ludvig Letica, Vlatka Martinovic, Iva Mikulic, Josip Miskovic, Hrvoje Pehar, Pejana Rastović, Irena Sesar, Violeta Šetka- Čuljak, Martina Soljic, Dejan Tiric, Vajdana Tomić, Anja Vasilj *(SKB University Clinical Hospital Mostar, Mostar)*; Anis Cerovac, Igor Hudic *(University Clinical Center Tuzla, Tuzla)*. |
| **Brazil**: Renato Aguera Oliver, Felipe Azenha Lamonica, Jorge De Medeiros, Henrique Donizetti Bianchi Florindo, Rodolfo Jose Favaretto Filho, Fernanda Costa Pereira, Rodrigo Rodrigues, Antonio Antunes Rodrigues Junior, Fernanda Ruiz de Andrade, Maisa Salvetti, Débora Schalge Campioto, Mayra Tuboi Lamonica, Gustavo Urbano, Ricardo Villela Prado *(Hospital Estadual Américo Brasiliense, Américo Brasiliense)*; Nivaldo Alonso, Carlos Ferreira dos Santos, Cristiano Tonello *(Hospital de Reabilitacao de Anomalias Craniofaciais de Bauru, Bauru)*; Robinson Esteves Pires, Ricardo Fernandes Rezende, Igor Reis *(Felicio Rocho, Belo Horizonte)*; Fernando Augusto Lima Marson, Erick Pires Ferreira, Camila Vantini Capasso Palamim *(Hospital Universitário São Francisco de Assis na Providência de Deus, Bragança Paulista)*; Marcelo Brandao, Tiago Henrique de Souza, Rebecca Maunsell *(Hospital De Clinicas da Unicamp, Campinas)*; Alfeu Accorsi Neto, Guilherme Accorsi, Murilo Francisco Fernandes, Camila Machareth, Barbara Viegas Moura *(Hospital Padre Albino, Catanduva)*; Vanessa Dias, Luciano Guarienti, Lia Regina de Sampaio, Cristiano Vendrame *(Supera Oncologia - Hospital Regional do Oeste, Chapeco)*; Tatiane Amorim Coelho, Karin Becker, Andre Dias, Camila Girardi Fachin, Monica Maria Gomes-da-Silva, Isabela Moraes, Amanda Pinto, Adriano Seikiti Stychnicki *(Complexo Hospital de Clínicas da UFPR, Curitiba)*; Nathalia Siqueira Julio, José Mauro dos Santos, Humberto Fenner Lyra Junior, João Carlos Costa de Oliveira, Tiago Rafael Onzi, Marlus Tavares Gerber *(Hospital Universitário Professor Polydoro Ernani de São Thiago- HU/UFSC/EBSERH, Florianopolis)*; Filipe Osni Coelho *(Maternidade Carmela Dutra, Florianópolis)*; Janaína Carla da Silva, Carolina Panis, Daniel Rech *(Ceonc - Hospital de Câncer de Francisco Beltrão, Francisco Beltrão-PR)*; Melissa Avelino, Lais Botacin, Mateus Capuzzo Gonçalves *(University Federal Hospital, Goiânia)*; Gustavo Mendonça Ataíde Gomes, Igor Lima Buarque, Amanda Lira dos Santos Leite, Laercio Pol-Fachin, Tainá Santos Bezerra, Aldo Vieira Barros *(Hospital Santa Casa de Misericordia de Maceio, Maceio)*; Alcimar lavareda dos Santos Junior Alcimar, Robson Amorim, Maria Eduarda Bellotti Leão, João José Corrêa Bergamasco, Cintia Cardoso Pinheiro, Rubem Alves Da Silva Neto, Elaine Francisca De Araújo, Lilian Guimaraes, Tatiana Liborio-Kimura, Isabelle Melo da Camara, Victor Ripardo Siqueira, Juan Rodriguez, Jeancarllo Silva, Rubem Alves Silva Junior *(Hospital Universitário Getúlio Vargas, Manaus)*; Cláudio Cardoso, Andre Guimaraes, Wislene Sarajane Moreira Alves, Agna Soares Da Silva Menezes *(Hospital Dilson Godinho, Montes Claros)*; Marcelo Araujo, Kattiucy Brito, Josie Marcelle Lira Albuquerque *(Hospital Universitário Antonio Pedro, Niteroi)*; Jairo Alberto Dussan-Sarria, Andressa Souza, Andre Ricardo Stüker *(Hospital Unimed, Novo Hamburgo)*; Tiago Bresciani, Luciana Cadore Stefani, Leandro Totti Cavazzola, Tainá Costa, Matheus Dasqueve, Gustavo De Bacco Marangon, Otávio Ritter Silveira Martins, Josy Rodrigues, Guilherme Roloff Cardoso, Brasil Silva Neto, Tilaê Soares, Aline Zanella *(Hospital de Clínicas de Porto Alegre, Porto Alegre)*; Lucas Torelly Filippi, Enilde Eloena Guerra, Ricardo Pedrini Cruz *(Hospital Nossa Senhora da Conceição, Porto Alegre)*; Antonio Nocchi Kalil, Gustavo Laporte, Moacyr Salem *(Irmandade da Santa Casa de Misericórdia de Porto Alegre, Porto Alegre)*; Murilo de Lima Brazan, Caio Antonio de Campos Prado, Elaine Christine Dantas Moises, Hilda Satie Suto, Alice Gadotti Yasuda, Ana Carolina Tagliatti Zani *(Centro de Referencia da Saude da Mulher de Ribeirao Preto - Mater, Ribeirao Preto)*; Roberto Cardoso Cardoso dos Santos, Geraldo Duarte, Edwaldo Edner Joviliano, Carolina Lourenço Gomes dos Santos, Silvana Maria Quintana, Edwin Tamashiro, Fabiana Cardoso Pereira Valera, Marília Veccechi Bijos Zaccaro *(Clinics Hospital, Ribeirao Preto Medical School, University of Sao Paulo, Ribeirao Preto)*; Isis Andreotti, Leonardo Lima, Wilson Salgado Jr. *(Hospital Estadual de Ribeirao Preto, Ribeirao Preto)*; Alonço da Cunha Viana Júnior, Lana Moutinho, Cora Pichler de Oliveira, Isabela Trindade Martins, Isabela Vieira Toledo *(Hospital Naval Marcílio Dias, Rio de Janeiro)*; Marco Antonio Correa Guimaraes-Filho *(Pedro Ernesto University Hospital, Rio de Janeiro)*; Rodrigo Arrivabeno, Claudio Bovolenta Murta, Danniel Frade Said, Jose Pontes Junior, Felipe Guimarães Pugliesi *(Hospital Brigadeiro, Sao Paulo)*; Paulo Gregorio, Alessandro Mariani, Fabio Minamoto *(Hospital das Clinicas da Faculdade de Medicina da Universidade de São Paulo, Sao Paulo)*; Juliana Lourenço da Silva, Bruno Muller, Caio Zanon *(Hospital Santa Paula, Sao Paulo)*; Vladimir C. Carvalho, Ana Lucia Munhoz Lima, Priscila R Oliveira, Jorge dos Santos Silva *(Instituto de Ortopedia e Traumatologia do Hospital das Clinicas da Faculdade de Medicina da Universidade de Sao Paulo, Sao Paulo)*; Mariana Faccini Teixeira, Luiz Paulo Kowalski, Marco Kulcsar, Leandro Matos, Kamilla Schmitz Nunes *(Instituto do Cancer do Estado de São Paulo, Sao Paulo)*; Joel Abdala Junior, Emne Abdallah, Samuel Aguiar Júnior, Glauco Baiocchi, Heloisa Galvão do Amaral Campos, Genival Barbosa Carvalho, Igor Correia de Farias, Cassia da Silva, Fabio Fernando Eloi Pinto, Luciana Facure, André Godoy, Jefferson Gross, Felipe José Fernandez Coimbra, Luiz Paulo Kowalski, Bruna Kupper, Fernanda Leite, Matheus de Melo Lôbo, Fabiana Makdissi, Henrique Mantoan, Narimã Marques, Tomas Marques, João Paulo Medici, Silvio Melo torres, Katheryne Merlos Garcia, Suely Nakagawa, Joao Pedreira Duprat Neto, Rafael Ribeiro Meduna, Ana Carolina Scintini Herbst, Silvana Soares Dos Santos, Bruna Tirapelli Gonçalves, Jose Guilherme Vartanian, Stenio C Zequi *(A.C. Camargo Cancer Center, São Paulo)*; Marcelo Antonini, Carolina Brienze, José Francisco Farah, Rodrigo Gonçalves, Vinicius Machado, Luis Roberto Nadal, Danilo Nadal Rodrigues, Adrieli Pansani, Maria Luiza Rocha, Marcelo Simonsen, Daniela Tsuchiya, Rafaela Vasques *(Hospital do Servidor Público Estadual - Francisco Morato de Oliveira, São Paulo)*; Jorge Amorim, Ana Alyra Carvalho, Rebeca Correia, Brena Costa dos Santos, Ronald Flumignan, Henrique Jorge Guedes Neto, Luis Nakano, Mariana Pereda, Vladimir Vasconcelos *(Hospital São Paulo, São Paulo)*; Jamile Barakat Awada, Guilherme Gava, Tiago Riuji Ijichi, Nam Jin Kim, Rafael Nunes, Fabio Pinto *(Notre Dame Intermédica - Hospital Salvalus, São Paulo)*; Thiago Henrique Sigoli Pereira, Gustavo Jardim Volpe *(Hospital Estadual Serrana, Serrana)*; Arthur Gatti, Caroline Nardi, Ramon Oliva *(Hospital Geral de Pirajussara, Taboão da Serra)*; Layze Braz de Oliveira, Herica Emilia Félix de Carvalho, Ivonizete Pires Ribeiro, Gabriel Renan Soares Rodrigues, Álvaro Francisco Lopes de Sousa *(Hospital Getúlio Vargas, Teresina)*; Michel Chebel, Paulo Henrique de Sousa Fernandes, Marcelo Augusto Faria Freitas, Juliano Rodrigues da Cunha *(Federal University of Uberlandia, Uberlandia)*; Michel Chebel, Paulo Henrique de Sousa Fernandes, Juliano Rodrigues da Cunha *(Uberlandia Medical Center, Uberlandia)*. |
| **Bulgaria**: Zeenia Ather, Dobromir Dimitrov, Emil Filipov, Muhammad Gohar, Elitsa Gyokova, Ivelina Ilieva, Tsvetomir Ivanov, Martin Karamanliev, Vasil Neykov *(University Hospital Dr Georgi Stranski, Medical University - Pleven, Pleven)*; Boyko Atanasov, Nikolay Belev, Mihail Slavchev *(University Hospital Eurohospital, Plovdiv)*; Paolina Kamenova, Kaloyan Tonev, Tsanko Yotsov *(University Hospital Medika, Ruse)*; Evguenia Hristova, Kolyo Spassov, Tsanko Tsankov *(Fifth City Hospital Sofia - 5th MBAL, Sofia)*; Dragomir Dardanov, Petar Gribnev, Manol Sokolov *(University Hospital Alexandrovska, Sofia)*. |
| **Cameroon**: James Brown, Chukwuemeka Nwegbu, John Tanyi *(Mbingo Baptist Hospital, Bamenda)*. |
| **Canada**: Heather Hurdle, Natalia Jaworska, Anthony MacLean, Joshua Ng-Kamstra, Michael Sander *(Foothills Medical Centre - University of Calgary, Calgary)*; Salim Al Riyami, Krittika Bali, David Bigam, Khaled Dajani, Angela Dell *(University of Alberta Hospital, Edmonton)*; Mehran Anvari, Rafik Bolis, David Choi, Susan Ellis, Michael Gupta, Wael Hanna, Dennis Hong, Cynthia Horner, Lea Luketic, Peter Moisiuk, Harsha Shanthanna, Yaron Shargall *(St. Joseph’s Healthcare Hamilton, Hamilton)*; Hilda Alfaro, Nawar Alkhamesi, Laura Allen, Muriel Brackstone, Eunice Chan, Davy Cheng, Jason Chui, Nelson Gonzalez, Brent Lanting, S. Danielle MacNeil, Janet Martin, Robert Mayer, Jacob McGee, Mahesh Nagappa, Nicholas Power, Agya Prempeh, Mehdi Qiabi, Hassan Razvi, Emil Schemitsch, Herman Sehmbi, Ushma Shah, Yamini Subramani, Edward Vasarhelyi, Kelly Vogt, Homer Yang *(London Health Sciences Centre and St Josephs Health Care London, London)*; Abdollah Behzadi, Amanpreet Brar, Ali Ghorbani Abdehgah *(Trillium Health Partners, Mississauga)*; Saba Balvardi, Liane Feldman, Julio Flavio Fiore Jr, Melissa Hanson, Brent Hopkins, Pepa Kaneva, Lawrence Lee, Julia Leonard *(McGill University Health Center, Montreal)*; Jad Abou-Khalil, Andre Martel, Carolyn Nessim, James Stevenson *(The Ottawa Hospital, Ottawa)*; Susan Lee, Richard Merchant, Michelle Mozel *(Eagle Ridge Hospital, Port Moody)*; Samuel Avoine, Laurence Belanger, Mathieu Belanger, Etienne Belzile, Anne-Sophie Blais, Sofia Boucher-Kovalik, Cindy Boulanger-Gobeil, Etienne Cardinal, Pierre-Olivier Champagne, Jonathan Cloutier, Simon Corriveau-Durand, Mathieu Cote, Maxime Cote, Mathilde Côté, Valérie Courval, Suzanne Demers, Christine Desbiens, Mehdi El Ouazzani, Karine Girard, Ève-Marie Girard, Karo Gosselin, Helene Khuong, Nathalie Labrecque, Eve-Lyne Langlais, Audrey Larouche, Andréane Lavallée, Madeleine Lemyre, Sarah Maheux-Lacroix, Melissa Marien, Patrick Marin, Julie Mauger, Genevieve Milot, Sylvie Nadeau, Sebastien Nguyen, Mélinda Paris, Stéphane Pelet, Marie Plante, Carole Plante, Frederic Pouliot, Marie Claude Renaud, Mathilde Sarlabous, Isabelle Schmit, Pascal St-germain, Raphaël St-germain *(Centre Hospitalier Universitaire de Québec, Québec)*; Brian Johnston, Crispin Russell *(Saint John Regional Hospital, Saint John)*; Gary Groot, Nicole Labine, Amit Persad, Hong Pham, Melissa Wood *(Saskatoon City Hospital/Royal University Hospital/St. Paul’s Hospital, Saskatoon SK)*; Riordan Azam, Najib Safieddine *(Michael Garron Hospital, Toronto)*; Amit Atrey, Andrew Beckett, Daniel Cohen, Sunit Das, Julian Daza, Cheryl Dunkerton, Yosef Ellenbogen, Ciara Hanley, Sorcha Kellett, Amir Khoshbin, Karim Ladha, Amanda McFarlan, Spencer Montgomery, Janneth Pazmino-Canizares, Hrishikesh Suresh, Duminda Wijeysundera *(St. Michael’s Hospital, Toronto)*; Antoine Eskander, Ravleen Vasdev *(Sunnybrook Hospital, Toronto)*; Maira Ahmed, Sami Chadi, Fred Gentili, Aristotelis Kalyvas, Matthias Millesi, Can Sarica, Taariq Shaikh, Leslie St. Jacques, Raha Tabasinejad, Gelareh Zadeh *(Toronto Western Hospital, Toronto)*; Marvin Hsiao, Nicole Jedrzejko, Shawn MacKenzie *(Royal Columbian Hospital, Vancouver)*; Peter Black, Claire Broe, Charlotte Dandurand, Rebecca Grey, Philemon Leung, Andrew Lindberg, Juan Mata Gutierrez, Kelly Mayson, Adam Meneghetti, Drew Phillips, John Street *(Vancouver General Hospital, Vancouver)*. |
| **Chile**: Fernando Heredia *(Clínica Universitaria de Concepción, Concepción)*; Carolina Carvajal Calderón, Daniel Alejandro Donoso Pizarro, Esteban Fernández, Maria Marta Modolo, Ángela Molero, Nastassja Mutarello, Jorge Núñez Lucic, Katherine Ochoa Gaete, Catalina Ruiz Lopez, Rina Sepúlveda, Camila Ulloa *(Hospital Barros Luco Trudeau, Santiago)*; Mauricio Barreda, Matias Dallaserra, Matías Günther Wood, Mauricio Sandoval Tobar, Julio Villanueva *(Hospital Clínico San Borja-Arriarán, Santiago)*; Francisco Garcia-Huidobro, Sofia Waissbluth *(Hospital Clinico Universidad Católica, Santiago)*; Jimena Dona, Roberto Macchiavello, Carolina Soto Diez *(Hospital de Urgencia Asistencia Pública Dr Alejandro del Río, Santiago)*; Mario I Escudero, Daniel Igor, Javier Mena, José Tomás Reyes *(Hospital San Jose, Santiago)*; Catalina Arredondo Soto, Jose Campos, Tyare Fuentes, Javier Gonzalez, Barbara Rivera *(Hospital Sótero de Río, Santiago)*; Jaime Altamirano-Villarroel, David Cohn, Luis Marin de Amesti, Ximena Mimica, Pedro Recabal, Camilo Sandoval *(Instituto Oncologico Fundacion Arturo Lopez Perez, Santiago)*; Bruno Catoia fonseca, Monica Contador, Andres Hodali, Camila Pincheira, Andrea Ramos Mantilla, Claudio Salas Garrido, Janina Torres, Marco Valenzuela *(Roberto del Río Children’s Hospital, Santiago)*. |
| **China**: Zehua Chen, Jiankun Hu, Jin Tao, Kun Yang, Yuexin Zhang *(West China Hospital, Sichuan University, Chengdu, Sichuan Province)*; Yuan Chen, Wang Cunchuan, Wah Yang *(The First Affiliated Hospital of Jinan University, Guangzhou)*; Xueli Bai, Tingbo Liang, Tao Ma *(The First Affiliated Hospital of Zhejiang University School of Medicine, Hangzhou)*; Wenhui Lou, Ning Pu, Hanlin Yin *(Zhongshan Hospital, Shanghai)*. |
| **Colombia**: Dinimo Bolivar Saenz, Camilo Caicedo, Diego Felipe Tellez Beltran *(Clinica Colsubsidio Calle 94, Bogota)*; Alejandro Escobar, Carolina Perez Granados, Lisbeth Alexandra Urueña Pinzon *(Clinica Palermo, Bogota)*; Carlos Bonilla, Joaquin Luna, Diana Santana *(Clinica Reina Sofia, Bogota)*; Carlos Bonilla, Diana Santana, Oscar Serrano *(Clínica Universitaria Colombia, Bogota)*; Andrea Garcia, Fernando Giron Luque, Nasly G. Patino-Jaramillo, Nestor Fabian Pedraza Alonso *(Colombiana de Trasplantes, Bogota)*; Laura Giselle Contreras Baquero, Nestor Augusto Muñoz Botero, Jhoana Andrea Murillo Castellanos, Camilo Ortiz Silva, Andrea Juliana Vega Calvera *(El Tunal, Bogota)*; Lina M. Acosta Buitrago, Maria Paz Bohórquez-Tarazona, Paulo Andrés Cabrera Rivera, Felipe Casas J, Valeria Cormane Alfaro, Julian Corso, Bayron Guerra, Albert Franz Guerrero-Becerra, Akram Kadamani Abiyomaa, Felix Ramon Montes, Manuel Santiago Mosquera Paz, Carlos J- Perez Rivera, Camilo Andrés Polanía Sandoval, Natalia Quintana, Carlos Fernando Roman Ortega, Carlos Santacruz, Manuela Téllez, Camilo Alejandro Velandia Sánchez *(Fundacion Cardioinfantil-IC, Bogota)*; Fernando Arias-Amézquita, Luis Felipe Cabrera Vargas, Natalia Cortes Murgueitio, Elkin Escorcia, Kemel Ahmed Ghotme Ghotme, Jorge Luis Gomez-Mayorga, Alvaro Felipe Guerrero Vergel, Gabriel Herrera-Almario, Eduardo Londono-Schimmer, Juan A. Mejia, Guillermo Monsalve, Juliana Rodriguez, Camilo Rodriguez, Juan Nicolas Rodriguez Niño, Luis Martín Rodríguez Ortegón, Daniel Sanabria *(Fundacion Santa Fe de Bogota, Bogota)*; Andres Alvarez, Arnulfo Andrade, Javier Ardila-Montealegre, David Baquero, Edgar Mauricio Barrios Vidales, María Alejandra Caicedo Giraldo, María Camila Carvajal, Maria Castillo, Maria Carolina Castillo Florez, María Angelica Cendales, Danny Conde Monroy, Henry Cortes, Mario Daniel, Jairo De la Peña, Elena Leonor Delgado-Nieto, Hernando Espitia, Carlos Figueroa Avendaño, Edgar Oswaldo Hernandez Burgos, Andres Isaza-Restrepo, German Londono, Margarita Maria Maldonado, Andrea Montenegro, Juan Carlos Navarro, Jorge Navarro-Alean, Katherine Parra Abaunza, Eliana Pulido, Erika M. Ramírez Amaya, Carlos Eduardo Rey Chaves, Natalia Andrea Rivera Rincón, William Mauricio Riveros Castillo, Lizeth Rodriguez Sanchez, Wilson Rubiano, Maria Russi, Juan Carlos Sabogal Olarte, Javier Mauricio Salgado Tovar, Maria Juliana Sanchez, Liliana Silva-Igua, David Mauricio Solano Varela, Martha Luz Torres, Paula Torres Gomez, Marcial Trillos, Ana María Vargas Patiño, Felipe Vargas-Barato, Juan pablo Villate leon, María Alejandra Wagner Useche *(Hospital Universitario Mayor, Bogota)*; Jesus Acosta, Adriana Almeciga, Luis Becerra Mendez, Rafael Jose Beltran, Ana Bonilla, Miguel Buitrago, Marino Cabrera, Lina Caicedo, Pedro Hernando Calderon Quiroz, Diego Camacho-Nieto, Carlos Andrés Carvajal Fierro, Sergio Cervera Bonilla, Sandra Diaz, Helena Facundo, Jorge Forero, Mauricio Garcia Mora, Karena Victoria Garcia Tirado, German Fabian Godoy Perez, Felipe Gonzalez, Oscar Guevara, Jairo Alonso Hernandez, David Ricardo Herrera Mora, Juan David Lalinde, Carlos Lehmann, Eduardo Leon Llanos, Byron Eduardo Lopez De Mesa Lopez, Ivan Mariño, Monica Medina, Raúl Eduardo Pinilla Morales, Angela Puerto, Ma. Andrea Quintero-Ortíz, Juliana Rendón Hernández, Juliana Rodriguez, Elio Fabio Sánchez Cortés, Alvaro Eduardo Sánchez Hernández, Diana Santana, Raul Suarez, Oscar Suescun, Lina María Trujillo, Marco Vanegas, Rodolfo Varela, Jorge Luis Velez Bernal, David Viveros-Carreño *(Instituto Nacional de Cancerologia, Bogota)*; Daniela Camargo Gómez, Angelica Fletcher, Abel Merchan *(Centro de Investigaciones Oncológicas Clínica San Diego - CIOSAD, Bogotá)*; Euler Javier Burbano Luna, Indira Cujiño, Albaro José Nieto Calvache, Mauricio Velasquez Galvis, Lina Maria Vergara Galliadi *(Fundación Valle del Lili, Cali)*; Orlando Abonia Gonzalez Abonia Gonzalez, Maria Fernanda Acuna Saravia, Roberto Arroyave, Jose Maria Barreto Angulo, Francisco Javier Bonilla-Escobar, Uriel Cardona, Ivan Castañeda Giacometto, José Luis Castillo, Diego Fernando Castillo-Cobaleda, Diego Jose Caycedo Garcia, Brenda Marcela Coll Tello, Juan Carlos Dueñas-Ramirez, Oscar Andres Escobar Vidarte, Luis Figueroa, Herney Garcia-Perdomo, Alden Gomez, Laura Gomez, Alejandro Gomez, Ana María Grande-Gil, David Guarin, Natalia Guzman, Enrique Herrera Castañeda, Marisol Hinaoui, Anuar Armando Idrobo Escobar, Christian kammerer Kammerer, Sergio Leon, Carlos Antonio Llanos Lucero, Angie López, Alexander Maximiliano Martinez-Blanco, Antonio José Montoya Casella, Ricardo Andres Niño Corredor, Sebastian Ordoñez, Diego Alfredo Palta Uribe, Andrea Carolina Perea Serna, July Ríos, Gabriel Ríos-Samper, Juan David Rivera Garcia, Juan Camilo Salcedo Moreno, John Sandoval, Guillermo Alberto Sarmiento Ramirez, Paola Andrea Tabares Romero, Ricardo Urzola, Iliana Maria Valdes-Duque, Miguel Velasquez, Lina Maria Villegas, James Zapata-Copete, José Omar Zorrilla Lara, Mauricio Zuluaga Zuluaga *(Hospital Universitario del Valle Evaristo García, Cali)*; Alejandra Echeverri Moreno, Juan Sebastian Figueroa, Rafael Figueroa - Casanova, Adolfo Enrique Gómez Ortiz, Maria Fernanda Gonzalez Mosos, Alejandro González-Orozco, Juan Jose Jaramillo Roncancio, María Camila Leyva Martínez, Monica Mosos, Juan Sebastian Ramirez, Henry Andrés Rodríguez, Juan David Saavedra Henao, María Alejandra Torrado Varón *(Clinica Avidanti - Ibague, Ibague)*; Oscar-Julián García-Montoya, Leidy Natalia Idarraga Ramírez, Lorena Ocampo *(Clínica Avidanti - Manizales, Manizales)*; Monica Cardona Marin, Ana Maria Marin Gonzalez, Carlos Andres Marulanda Toro *(SES Hospital de Caldas, Manizales)*; David Alejandro Mejia, Maria Clara Mendoza Arango, Luis Emiro Vanegas *(Hospital Universitario San Vicente Fundacion, Medellin)*; Yicel Alvarez Martinez, Sandra Aruachan Vesga, Dayana Vanessa Baron Fuentes, Alejandra Caballero salas, Eneida Diaz Martinez, Gustavo Antonio Martinez Estrada, Kelly Viloria Campo *(IMAT Oncomedica, Monteria)*; Juan Antonio Corralez Alvarez, Cesar David Galindo Regino, Angela Maria Giraldo Velasquez, Valentina Gutiérrez Perdomo, Carlos Enrique Melo Moreno, Jorman H. Tejada, Jesus Hernan Tovar, German Alirio Tovar *(Hospital Universitario Hernando Moncaleano Perdomo, Neiva)*; Edison Alexander Benavides Hernández, Jose Andres Calvache, Claudia Milena Orozco-Chamorro *(Hospital Susana Lopez de Valencia, Popayan)*; Gustavo Adolfo Angel, Christian Ali Buesaquillo, Jose Andres Calvache, Victor David Olave Montaño, Andrés Sánchez-Gómez *(Hospital Universitario San José, Popayán)*; Daniela Patricia Escalante Ureche, Jairo Martinez Garrido, Emileth Montenegro *(Clínica Avidanti Santa Marta, Santa Marta)*. |
| **Congo, Dem. Rep.**: Eben-ezer Genda *(Centre Hospitalier Saint-Vincent, Bukavu)*; Nicolas Amisi, Fabrice Eboma *(CENTRE HOSPITALIER IMEYA SECOURS, Kinshasa)*. |
| **Croatia**: Kristina Bitunjac, Karlo Grulović, Marijana Vučković *(General and Veterans Hospital ‘Hrvatski Ponos’ Knin, Knin)*; Goran Šantak *(County General Hospital Pozega, Pozega)*; Emanuel Borovic, Ana Bosak Versic, Damir Hasandić, Natasa Poldan Grabar, Suzana Srsen Medancic *(University Hospital Center Rijeka, Rijeka)*; Lucija Brkic, Sara Cokarić, Petra Pavic Palac, Zeljka Samac, Josipa Tomić, Marija Vrdoljak *(General Hospital Sibenik, Sibenik)*; Ivan Bacic, Bernarda Bakmaz, Nikolina Bratošević Vučičić, Domagoj Brzic, Samir Canovic, Emilio Dijan, Maja Grgec Dragicevic, Robert Karlo, Suzana Konjevoda, Petra Kovačević, Ivan Kovačić, Frane Markulić, Luka Matak, Jakov Mihanovic, Domagoj Morović, Gordan Perišić, Andrea Simic, Neven Skitarelić, Matea Veršić, Vanja Žufić, Matea Zuzul *(Zadar General Hospital, Zadar)*; Srdan Ante Anzic, Iva Carevic, Dubravka Heli Litvic, Duska Markov-Glavas, Tatjana Savic Jovanovic *(Children’s Hospital Srebrnjak, Zagreb)*; Goran Augustin, Jerko Biloš, Bojan Biočina, Vedrana Biosic, Dino Bobovec, Iva Botica, Boris Bumber, Petra Čerina, Ana Danic Hadzibegovic, Katarina Duric Vukovic, Hrvoje Gasparovic, Lucija Gatin, Kresimir Grsic, Ika Gugić Radojković, Ivan Jelčić, Zeljko Kastelan, Juraj Kolak, Tomislav Kopjar, Tomislav Kulis, Kristian Kunjko, Marjan Maric, Marcel Marjanović Kavanagh, Borna Milicic, Trpimir Morić, Miram Pasini, Luka Penezić, Drago Prgomet, Ratko Prstacic, Andreja Prtorić, Rudolf Radojković, Ivan Romić, Tomislav Sečan, Dora Škrljak Šoša, Juraj Slipac, Mislav Tomic, Jurica Zedelj, Toni Zekulić, Zoran Zimak *(University Hospital Centre Zagreb, Zagreb)*; Mia Lorencin, Ivica Luksic, Matija Mamic *(University Hospital Dubrava, Zagreb)*. |
| **Cuba**: Pablo Mijahil Avilés Jiménez, Norberto Miranda Espinsa, Ailén Sánchez Cruz *(Hospital Pediátrico Juan Manuel Márquez, La Habana)*. |
| **Cyprus**: Stavros A. Antoniou, Michael Kakas, Yiannis Panayiotou *(Mediterranean Hospital of Cyprus, Limassol)*; Heyam Almezghwi, Kalbim Arslan, Hasan Besim, Ali Özant, Necdet Özçay *(Near East University Hospital, Nicosia)*; Nikolaos Gouvas, Georgios Kokkinos, Panayiotis Papatheodorou, Ioanna Pozotou, Olga Stavrinidou, Anneza Yiallourou *(Nicosia General Hospital, Nicosia)*. |
| **Czech Republic**: Lukáš Burda, Michal Dosoudil, Lubomir Martinek *(Hospital & Oncological Centre Novy Jicin, Novy Jicin)*; Wladyslaw B. Gawel, Milan Lerch, Matúš Peteja, Jan Žatecký *(Slezská nemocnice v Opavě, p.o., Opava)*; Toman Daniel, Martin Formánek, Veronika Javurkova, Markéta Kepičová, Jaroslav Klat, Nicole Macečková, Petr Matousek, Ondrej Simetka, Petr Vavra, Karol Zelenik *(University Hospital Ostrava, Ostrava)*; Jan Černý, Sami Khan, Robert Lischke, René Novysedlák, Martin Přibyl, Temoore Younus *(Motol University Hospital, Prague)*; Yenuksha Amarasena, Karel Klíma, Setareh Pirmorad *(Všeobecná Fakultní Nemocnice, Prague)*; Zuzana Hotová, Aleš Mladěnka *(Nemocnice Třinec p.o., Třinec)*. |
| **Denmark**: Nulvin Bozo, Peter Christensen, Julie Lykke Harbjerg, Louise Hviid, Helle Ø Kristensen, Mira Mekhael, Mette Fugleberg Nielsen, Laerke Paulsen, Saija Sinimäki, Louise Zinck Mogensen *(Aarhus University Hospital, Aarhus)*; Peter Bonde, Anders Lyng Ebbehøj, Anne-Sophie Fenger, Aleksander Fjeld Haugstvedt, Christine Hangaard Hansen, Maria Lovisa Jönsson, Lars N Jorgensen, Peter-Martin Krarup, Anne-Louise Lihn, Christian Meyhoff, Helena Otte, Anas Ould Si Amar, Henrik Palm, Nis Schlesinger, Henry Smith, Ida Tryggedsson *(Bispebjerg Hospital, Copenhagen)*; Anne Reiss Axelsen, Jens Kristian Bælum, Mark Bremholm Ellebaek, Signe Bremholm Ellebæk, Anders Hogh *(Odense and Svendborg University hospital, Odense and Svendborg)*. |
| **Dominican Republic**: Maria Acosta, Yancy Acosta, Pedro Baez, Sylvia Batista, Luis Alfredo Betances, Luis Rodolfo Bonilla, Daniel Cabreja, Wilton Cabrera Cruz, María Mabel Collado Expósito, Aldo Crespo, Octavio Cruz-Pineda, Claudio Samuel D’óleo García, Pedro Díaz, Remberto Escoto, Jatnna Figueroa, Luis Garcia, U Garcia-Dubus Rodriguez, Humberto Gomez, Adrian Grullon, Fatherin Guerrero, Lillian Guzman, Thelma Rocío Jiménez Mosquea, Engels Lazala, Ariela Lopez, Leeany Maletta Francisco, Jhomayri Mercado, Herisardy Munoz, Jeffrey Paulino, Maricely Ambar Perez Fernandez, Rudeily Reyes, Lilia Rosa Reyes Guilamo, Ruben Rivas, Claudia Alejandra Rivas Torres, Ada Rodríguez, Julia Rodriguez-Abreu, Madelin Tamar Rosario Villa, Eleazar Santana, Joel Soto, Nassin Tactuk, Cristina Kristel Tonos Sardiñas, Raul Ubinas, Jose Victoria, Aaron Villegas *(CEDIMAT - Centro de Diagnóstico, Medicina Avanzada, Laboratorio y Telemedicina, Santo Domingo)*; Ricardo Acra-Tolari, Larissa Beltran, Luis Fernand Betances, Alejandro Blaubach, Denisse Idalia Campos Mejía, Nathalia Capellan, María Fernanda Cedeño Bruzual, Saray Cordero Spencer, Gabriella Cuevas Lantigua, Luis De jesus, Gabriela Díaz, Pedro Pablo Díaz Vásquez, Karla Disla, Jesús Antonio Echavarría Uceta, Paola Irina Eusebio Jimenez, Damaris Fernandez, Marlin Fernandez Camilo, Jiomar Manuel Figueroa Germosen, Maruel Fortunato, Leyla Gabriel Fernández, Alicia German Dihmes, Héctor Herrera, Elizabeth Leon Cuevas, Jose Alejandro Mata, Mirian Mateo De La Cruz, Dolores Mejia De la Cruz, Marcos Mirambeaux, Fabio Ortiz De La Cruz, Henry Paulino, Gabriela Pelletier, Merycarla Pichardo, Rodriguez Pumarol Próspero Enrique, Julio Rivas, Ann Stephany Sanchez Marmolejos, Roman Santana Santana, Irina Suero Almanzar, María Magdalena Vásquez Sánchez *(Hospital General Plaza de la Salud, Santo Domingo)*. |
| **Ecuador**: Nicolás Campuzano, Eddy Lincango-Naranjo, José Ricardo Negrete Ocampo *(AXXIS Hospital Quito Ecuador, Quito)*; Maria Armas, Alvaro Santiago LeMarie Guerra, Gustavo León Vizcaya *(Hospital Padre Carollo, Quito)*. |
| **Egypt**: Omar Ahmed Abdelwahab, Ahmed O. Elmehrath, Mohammed Ezzat Mostafa *(El Fayrouz Specialized Hospital, Abu Kabir, Al Sharqia)*; Gamal Amira, Ibrahim Sallam, Mohamed Sherief *(MISR Cancer Center, Al Jizah)*; Ahmed Abdelmajeed, Mostafa Abdou, Ahmed Abo Shanab, Aya Abodeeb, Roger Aboelkhel, Nour Eldin Abosamak, Amna Abou Bakr, Samar Aboubakr, Galal Abouelnagah, Yossof Abouelnagah, Omar AbouMadawy, Dina Adel, Nermeen Afifi, Sara Ahmed, Abedelrahman Ahmed, Mohamed AL Sayed, Abdelrahman Alberkamy, Mohamed Aldahma, Ahmed Ali, Mostafa Ali, Mohamed Amin Bakr, Alaa Anter, Doaa Asal, Fouad Ashoush, Olfat Ashraf, Abdel Rahman Ashraf, Rewan Atta, Salma Badr, Mohamed Bahaaeldin, Mostafa Bastawesy, Sara Darwish, Rasha El kharashy, Ziad Elassar, Seifeldin Elbadawy, Dina Elkhity, Alromisaa Elsaka, Manal Elsayed, Mostafa Elsayed Elsayed Hewalla, Mohamed Elshafey, Youssof Eshac, Fares Eshac, Amr Essameldin, Moataz Ewedah, Yara Ezz, Mohamed Farag, Inas Gadelkarim, Ziyad Gadelrab, Dina Gamal, Mira Ghaly, Nathalie Girgis, Omar Gouda, Nancy H. El Goweini, Nour Hafez, Youssef Hafez, Abdelkader Hamed Abdin, Rodina Hanno, Aliaa Hassanin, Mohamed Hassanin, Hanan M. Hemead, Abdelrahman Hemida, Maher Hosain, Ahmed Hossam, Hamza Hussein Aly Salama Aly, Mohamed Ibrahim, Abdelrahman Ibrahim, Nourhan Ibrahim, Noha Mohamed Salah Ibrahim Moussa Hamouda, Monica Iskander, Islam Khalifa, Mostafa Kotb, Nada Mahmoud, Alaa Mahmoud Abo shabana, Nora Mamdouh, Maram Metwalli, Khaled Metwally, Mahmoud Moghazy, Moustafa Mohamed, Mohamed Mourad, Esraa Moustafa, Kamilia Mubarek, Nour Eldin Nader, Ahmed Nasser, Samaa Omar, Mostafa Shehata Qatora, Mohamed Ragab, Marawan Ragal, Dina Ramadan, Rana Ramadan, Sara Ramadan, John Romany, Ahmed Sabry, Yasmeen Said, Sara Salamah, Ahmed Mostafa Saleh, Mona Saleh Mesbah Mohamed Elkaffas, Alaa Salem, Hashem Salim, Ahmed Samih, Ahmed Samir Abdelaal, Ahmed Shaheen, Yassmeen Sharafeldin Mohammed, Sameh Shehata, Abdelrahman Shehata, Mohamed Shemeis, Karim Shenit, Mennatallah Sheta, Asmaa Soffar, Yousef Tanas, Ahmed Tarek, Nermin Yehia *(Alexandria Main University Hospital, Alexandria)*; Omar Khaled Mohamed Eid, Ammar Yasser Abdulfattah, Ahmed ElSaghir, Mohamed Fouad Elganainy, Ahmed Hossam Eldin Fouad Rida, Omar Ibrahim Elsayed *(El Hadara University Hospital Alexandria University, Alexandria)*; Omar Elmandouh, Omar Hamam, Mostafa Ahmed Shehata *(Wingat Royal Hospital, Alexandria)*; Areej A. Abdelaziz, Ahmed M. Abbas, Wael Abd El-Ghani, Hossam Aldein S. Abd Elazeem, Mustafa Abd Elsayed, Ahmed Yassien Abd-Elkariem, Shimaa Abdalla, Mahmoud Abdel-Aleem, Khaled Abdelazeem, Mahmoud Abdelfattah, Mohammed Abdelhafez, Mohamed M. Abdelkarem, Ali Abdelraouf, Lamess Abdullaha, Hossam Abubeih, Moaiad Eldin Ahmed, Nagm Eldin Abu Elnga Ahmed, Ahmed Ahmed, Sarah Ahmed Saad, Sherif Alaa, Ali Alhussaini, Abdelrahman Ahmed Abdelrahman Ali, Ibrahim Ali, Wagdi Ali, Mohamed Ali Mohamed, Mohamed Ashraf, Muhammad Bassiouni, Mohamed G. El-adawy, Mohammad El-Sharkawi, Hussein Elkhayat, Khaled Elmaghraby, Shady Elsdfy, Mohamed Elsharkawy, Almoutaz Eltayeb, Mohamed Esmat Mohamed, Esraa Essam, Mahmoud Fahd, Osama Farouk, Rabea Gadelkareem, Ahmed Ghoneim, Mohammed Hamada Takrouney, Ahmed Hamdan, Abd El-Rahman Hamed, Ahmad Hasan, Ramy A. Hassan, Mohamed Abdelghafor Hassanin, Islam Hawal, Kerollos Henes, Mohamed Omar Herdan, Helal F. Hetta, Omar Ibrahem, Mostafa Ibrahim, Islam H. Ibrahim, Mahmoud Kamel, Mohamed Khallaf, Shrouk M.elghazaly, Abobakr Mahfouz, Osman Mahmoud, Wesam Mahran, Doha Mahrous, Abadeer Marsis, Ayman Mohamed, Aliae Mohamed Hussein, Abdelrahman Mohamed saad, Amr Mohamed Sayed, Mahmoud M Mohammed, Ahmed Mokhtar, Fatma A. Monib, Ahmed Nageeb, Mariam Albatoul Nageh, Mohammed Nageh, Nehal gamal Omar, Abdelrahman Ragab, Abdelrahman Ramadan, Abdallah Rashad Temerik, Mahmoud M. Saad, Aya Sabry, Hadeer Safwat, Omar Salah, Ahmed Elhussiny Salah Mahmoud, Ahmed Saleh, Mahmoud Sallam, Ahmed Samir, Reem Sayad, Esraa Sayed, Ahmed.s Sedik, Mohammed Shahine, Abdelrahman Shehata, Antonios Soliman, Wael Soliman, Mohamed Gamal Taher, Randa Wanees, Ebrahim Ahmed Yousof, Ahmed Youssef, Omar Zein Elabedeen *(Assiut University Hospital, Assiut)*; Ahmed Emad Sayed Hassan *(Al-Azhar University Hospital, Assuit)*; Shrouk Abdel Fattah, Ahmed Abostate, Samar Ali, Saad Ali Saad Salama, Mohamed Allam, Ahmed ALsadek, Ebrahim Arafa, Ahmed Barakat, Mohammed El Sherpiny, Gehad Elbehairy, Mahmoud Eleisawy, Abdelrahman Elgendy, Rewan Elhawary, Sherif Eltregy, Mahmoud Hamdy, Gehad Hassan, Aya Khalifa, Ahmed Mahmoud, Ahmed Mohamed Altukhy, Kareem Noah, Yasser Noureldin, Mostafa Nowar, Mohamed Reda Loaloa, Moustafa Saad, Ahmed Saad Elsaeidy, Khaled Saad Elsaeidy, Hesham Sabry, Mostafa Sameh, Ahmed Tarek Said-elnaby, Mohamed Zahed, Mohamed Zahran, Abdelrahman Zaid, Ahmed Zaki Zoghary *(Benha University Hospital, Benha)*; Mohamed Atef, Ahmed Mohamed, Mohamed Youssef *(Beni Suef University Hospital, Beni Suef)*; Mahmoud Hossameldin Saad Abdelhamid, Emad Alazab, Ahmed K. Awad, Amr Darwesh, Lobna El Fiky, Ibrahim ElGarhy, Badr Mostafa, Mohamed Adel Nassef, Mohamed Qassem, Mahmoud Shaban, Mahmoud Mohamed Mohamed Shalaby, Abdelrahman Wahba, Omar Youssif Omar Fouad *(Ain Shams University Specialized Hospital, Cairo)*; Menan Elsadek, Mohamed Fahmy, Dalia Gad *(Al Zahraa University Hospital, Cairo)*; Ahmed Abdelsamed, Hesham Abozied, AbdulHakeem Bayomy, Mohamed Elsalhy, Ahmed Fahim, Ahmed Seleim *(Al-Azhar University Hospitals, Cairo)*; Eman Afifi, Fatma Alzahraa Gamal, Mennatullah Gamal *(El Demerdash University Hospital, Cairo)*; Omnia Eldesouky, Mohamed hatem elmetwalli eldwini Eldwini, Mostafa Medhat Fahmy Fahmy, Ahmed Hussein, Abdallah Ouf, Omar Sami, Salem Shaat, Abdullah Wael Mostafa Khalil Bahi *(El Nozha Cairo Governorate, Heliopolis Hospital, Cairo)*; Abdurhman Atea, Ibrahim Gamal *(EL-Hussein University Hospital, Al-Azhar University, Faculty Of Medicine, Cairo)*; Mohamed El Kassas, Wael Omar, Ahmed Tawheed *(Endemic Medicine Department, Helwan University, Cairo)*; Saeid Al-oribi, Nuran Khaled Aly, Mahmoud ElFiky, Alaa Eldine Elmaghraby, Abdulrahman Elrahmany, Motaz Elsherbeeny, Youssef Helmy, Ahmed Nabil, Ahmed Samir Farahat, Mostafa Soliman, Amr Wassef *(Kasr Alainy Hospital, Cairo)*; Abdelrahman Abdelrahman, Seif ElSaban, Galal Ghaly, Rana Hamdy, Hamada Mondy *(National Cancer Institute, Cairo)*; Lubna Abdallah, Abdelrhman KZ Darwish, Mohamed Rabea *(The National Hepatology and Tropical Research Institute, Cairo)*; Mohammed A Azab, Ahmed Y Azzam *(Damietta Specialized Hospital, Damietta)*; Alzhraa Salah Abbas, Sherief Ghozy *(sheikh zayed specialized hospital, giza)*; Marwa El-Deeb, Mohamed Fawzy, Galal Ghaly, Maher Ibraheem *(Baheya Foundation for Treatment of Breast Cancer, Giza)*; Abdelrahman Bakry, Sarah Elnems, Randa Elsheikh, Mohamed Ibrahim Gbreel, Mahmoud Hafez, Mohamed Jammal, Khaled M Hamam, Abdelrahman M Makram, Omar Mohamed Makram, Salma Rabie, Sherine Yousery Askalany *(October 6 University Hospital, Giza)*; Mokhtar Mohamed Ibrahim Abushanab, Abdulrahman Eid, Mohammed Refaat Ibrahiem Amin El Ghalid *(Research institute of ophthalmology, Giza)*; Ahmed Adel abdelaty, Elsayed A. Fayad, Asmaa Radwan, Asser Sallam, Moataz Sallam, Ahmad Shokry *(Suez Canal University Hospital, Ismailia)*; Ahmed Mohamed Farouk, Ahmed Shehta *(Gastrointestinal surgery center, Mansoura)*; Ahmed Elfallal, Hossam Elfeki, Mohamed Elsaeed, Mahmoud Elsaid, Sameh Emile, Amany Makroum, Dina Mohamed elsaid, Mohamed Mostafa, Mohamed W Omar, Mohamed Rezk, Ahmed Sakr, Aly Sanad, Mostafa Shalaby, Mohammed Shawqy, Mohamed Shetiwy, Dr. Ashraf Shoma, Noura Tawakl, Asmaa Yunes *(Mansoura University Hospital, Mansoura)*; Mohamed Abdelkhalek, Amr Abouzid, Khalid Atallah, Rami Elmorsi, Ahmed Elsherbini, Khaled Gaballa, Omar Hamdy, Mohamed Hamdy, Islam Hany Metwally, Basel Refky, Mosab Shetiwy, Mohammed Zuhdy *(Oncology Center Mansoura University, Mansoura)*; Mohammed A Zahran, Fatima Abdellah, Ahmed Abdelmawla, Ahmed Abdrabou, Asmaa Abubakr, Rawda Al Gohary, Mohammed Hamdy Al-Shazly, Mohamed Ali, Abdallah R Allam, Aya Aposaeeda, Mohammed Asfour, Ammar Ayman, Abdelrahman Azzam Omran, ibrahim Tawfiq Daghash, Mahmoud Ahmed Ebada, Taher Eid, Ahmed El Kelany, Esraa El Shemy, Mohammed El-Hag-Aly, Omar ELgamal, Mahmoud Elghoury, Ahmed Farag ElKased, Takwa Hamed Ellakwa, Hamed Ellakwa, Amr Elmeanawy, Salma Elnoamany, Alaa Elsabagh, Abdelrahman Elsawey, Enas Elshabrawy, Seliman ELShakhs, Naira Elsoudy, Esraa Ezzat, Ahmed Fawzy, Abrar Gamal, Ahmed Gameel, Khaled Gharbia, Mohamed Ghonaim, Alaa Ghonaim, Ahmed Hafez, Abdelrahman Hafez, Zainab Ismail, Mohamed Khaled, Mohammed Meselhy, Pola Mikhail, Mervat Mohamed, Mohamed Mougahed, Abdulla Mustafa, Ahmed Nada, Janna Omran, Mohamed S. Ebiad, Ibrahiem Saleh, Nourhan Salem, Osama Salem, Rehab Samaka, Ahmed Samir, Salma Selim, Ghada Shalaby, Hoda Sherif, Hatem Soltan, Mahmoud Wahbah, Eman Zakaria Abdelbary, Ahmad Helmy Zayan *(Menofia University Hospital, Menofia)*; Abdelrahman Afify, Hossam Ali Hadiya, Dr Mohamed Jamal Elshref, Mostafa Mohamed Ahmed, Nourhan Nasser *(Minya University Hospital, Minya)*; Hesham Abdeldayem, Ibrahim Abdelkader Salama, Khaled Ammar, Islam Ayoub, Amr Aziz, mohammad Taha Badawy, Mohamed Balabel, Yahya Fayed, Emad Hamdy Gad, Maher Gomaha, Essam Hammad, Osama Hegazy, Elsayed Hegazy, Tarek Ibrahim, Mostafa Kallaf, Mahmoud Macshut, Ammar Magdy, Ahmed Oteem, Ahmed Sallam, Samy Samaan, Mohamed Sharshar, Ahmed Elshawadfy Sherif, Hany Shoreem, Elsayed Soliman, Hossam Eldeen Soliman, Taha Yassein, Hazem Zakaria *(National Liver Institute, Menoufia University, Shibin Elkom)*; Abdullah Eldaly, Sarah Mashaly *(El Menshawy General Hospital, Tanta)*; Sherief Abd-Elsalam, Wafaa Abdel-Elsalam, Ahmed Abdullah Shaalah, Mohamed Elbahnasawy, Ismael Elhalaby, Mohamed Hamada, Sarah Hamdy Soliman, Ahmed Hawila, Mohamed Sherif Morsy, Mohmed Naieem, Mohammed Nasreddin, Samar Salman, Sameh Sarsik, Ahmed Shabana, Engy Tolba, Mohamed Zagho *(Tanta University Hospital, Tanta)*. |
| **El Salvador**: Danilo Alfonso Arévalo Sandoval *(Clínica de Gineco-Oncología Dr. Danilo Arévalo, San Salvador)*. |
| **Estonia**: Liisa Kams, Tõnu Rätsep, Karolin Riips *(Tartu University Hospital, Tartu)*. |
| **Ethiopia**: Oumer Abdurehman, Azarias Admasu, Ataklitie Berhea, Yegeremu Eado, Nebiyou Hailu, Meklit Kidane, Abdurezak Mohammed, Khalid Mohammed, Abraham Negussie, Admasu Tibelt, Mersha Abebe Woldemariam, Lydya Yonael *(ALERT center, Addis Ababa)*; Megersa Alemu, Tigist Fisseha *(Eka kotebe General Hospital, Addis Ababa)*; Abenezer Tirsit Aklilu, Frehun Asele, Solomon Assefa, Dawit Azmach, Philimon Bekele, Dr.Mickyas Mamo, Nahom Maru, Haile Mekuria, Abel Menkir, Filagot Mikru, Kibruyisfaw Shumbash, Eyerusalem Siraw, Hilkiah Suga, Natnael Sumoro, Aklilu Teka, Efeson Thomas, Dereje Woldemariam, Estifanos Wubishet *(Myungsung Christian Medical Centre, Addis Ababa)*; Metasebia Abebe, Engida Abebe, Kirubel Abebe, Fitsum Asfaw, Eyerusalem Bergene, Mahder Eshete, Fitsum Gebreegziabher Gebrehiwot, Yetsedaw Gedefaw, Mulualem Wondafrash Mengesha, Netsanet Mengiste, Abeje Menjeta, Mahteme Bekele Muleta, Sena Sefera, Yonatan Tedla, Daniel Teklu, Henok Teshome, Leake Tirfe, Sahle Tsegabrhan, Milkias Tsehaye, Bereket Worku *(Saint Paul Hospital Millennium Medical College, Addis Ababa)*; Million Molla, Melka Supha, Tewodros Taye *(Saint Peter specialised hospital, Addis Ababa)*; Nebyou Abebe, Berhanu Alemu, Abera Chanie, Hailegebriel Degefu, Dawit Desalegn, Hiwot Gebre, Samuel Hailu, Husnia Hussen, Dawit Kassa, Tsegazeab Laeke, Tihitena Negussie Mammo, Samuel Negash, Abat Sahlu, Abraham Genetu Tiruneh, Amanuel Wolde, Hanna getachew Woldeselassie, Mnewer Y. Ahmed, Betelhem Zewdneh *(Tikur Anbessa Specialized (Black Lion) Hospital, Addis Ababa)*; Jibril Fentaw, Shemsedin Salia, Ketema Tabore, Seid Mohammed Yasin *(Yekatit 12 hospital medical college, Addis Ababa)*; Yemisirach Bizuneh Akililu, Addisalem Gurara *(Zewditu Memorial Hospital, Addis Ababa)*; Tassew Abreha, Teklebirhan Abrha, Gaym Beyene, Niguse Hailu, Mulu Atsbaha Weldu *(Adigrat General Hospital, Adigrat)*; Desalegn Abdissa, Abebe Megersa *(Ambo University Referral Hospital, Ambo)*; Teshome Tefera *(Arbaminch General Hospital, Arbaminch)*; Mequannet Tesfaw, Melatework Wolle *(Adisalem Primary Hospital, Bahir Dar)*; Dawit Asmamaw, Biniam Zemedu Assefa, Bahru Atnafu, Dereje Bedane, Ephrem Bekele, Aderaw Getie, Solomon Melkamu, Workineh Mengesha, Getachew Shumye, Gashaye Tagele, Abrham Amare Tesfa, Wubshet Workneh *(Bahir Dar University Tibebe Ghion Specialized Hospital, Bahir Dar)*; Sileshi Genetu, Eneyew Mebratu *(Gamby General Hospital, Bahir Dar)*; Lidya Chanyalew, Tilahun Deresse, Adissu Girma, Marta Seid, Dagim Shimelash, Abrham Shitaw, Bereket Tsegaye *(Debre Berhan Comprehensive Specialized Hospital, Debre Birhan)*; Tewabe Ayalew, Yoseph Solomon Bezabih, Tesfaye Diress, Berhanu Kassahun, Bersabeh Kassaye, Esubalew Mulugeta, Getasew Tesfaw, Beminet Yimenu *(Debre Markos Comprehensive specialized Hospital, Debre Markos)*; Semir Benecha, Silamlak Sisay *(Dessie Referral Hospital, Dessie)*; Mudesir Aman, Kebebe Bekele, Adem Ibrahim, Alem Mekete, Abdi Tesemma *(Maddawalabu University Goba Referral Hospital, Goba)*; Moa Jira, Adnan Abdulkadir Mohammed *(Hiwot Fana specialized University Hospital, Harar)*; Yasir Younis Abdullahi *(Jugal General Hospital, Harar)*; Ephrem Adem, Mahlet Ahmedin, Yared Assefa, Fitsum Ayde, Alazar Berhe, Zersenay Gebremeskel, Mengistu G Mengesha, Eneyew Getachew Siyoum, Sintayehu Teresa, Zinaye Wude, Fasika Yemer, Dagnachew Yohannes, Ewunetu Zeleke, Gulilat Zerihun *(Hawassa University Comprehensive Specialized Hospital, Hawassa)*; Derje Worku Degefe, Habtamu Derilo, Hankore Tamirat Derilo, Nebiyu Eliyas *(Wachemo University Nigist Elleni Mohammed Memorial Referral Hospital, Hossana)*; Firew Bayissa, Eyueal Degefa, Yadani Deressa, Lidya Gemechu, Tegenu Gurmu, Ashenafi Kasaye, Lemi Melese, Lemesa Muleta, Gersam Mulugeta, Seifu Taye, Abraham Teshome, Birhanu Tesso *(Jimma University Medical Center, Jimma)*; Dr.Gebreagziabher Gebrekirstos, Mhreteab Haile, Haftamu Kassa, Mohammed Saddik, Anteneh Tadesse *(Mekelle University Ayder Comprehensive Specialised Hospital, Mekelle)*; Alemneh Mengist, Melkamu Nidaw *(Pawi General Hospital, Pawi)*. |
| **Finland**: Elise Sarjanoja *(Länsi-Pohja Central Hospital, Kemi)*; Juuso Heikkinen, Olli Helminen, Heikki Huhta, Joonas H Kauppila, Tommi Kotkavaara, Matti-Aleksi Mosorin, Joel Pitkänen, Cheng Qian, Jaakko Sinikumpu, Henri Sova, Mikko Tastula, Ville P Virta *(Oulu University Hospital, Oulu)*. |
| **France**: Jérémie Bettoni, Stéphanie Dakpé, Bernard Devauchelle, Nolwenn Lavagen, Sylvie Testelin *(CHU Amiens, Amiens)*; François Bastard, Kim Bin, Sophie Boucher, Renaud Breheret, Olivier Fouquet, Alexandre Gueutier, Nicolas Henric, Alexis Kahn, Jean-Daniel Kün-Darbois, Didier Moukoko, Anna Pineau, Guillaume Podevin, Françoise Schmitt *(CHU Angers, Angers)*; Fadi Alshawared, Carlos Daniel Beyrne, Lionel Jouffret, Laurene Lugans, Lysa Marie-Macron *(Centre Hospitalier Avignon, Avignon)*; Alexandre Doussot, Zaher Lakkis *(CHU Besançon, Besancon)*; Omar Ahmed, Louy Alnajjar, Tommaso Cipolat Mis, Souha Fliss, Audrey Giocanti-Auregan, Thomas Gregory, Patrice Guiraudet, Adrien Le Fouler, Emmanuel Martinod, Ilaria Onorati, Marine Peretti, Julien Quilichini, Dana Radu, Tresallet Trésallet, Alban Zarzavadjian Le Bian *(Avicenne Hospital, Bobigny)*; Luke Harper *(CHU Bordeaux, Bordeaux)*; Christophe Andro, Marc Danguy des Déserts, Alexis Maffert *(Military Hospital Clermont Tonnerre (Hôpital des Armées), Brest)*; Benoît André, Tracy Chapman, Maxime Halden *(Centre Hospitalier Intercommunal de Castres-Mazamet, Castres)*; Julie Fayon, Catherine Mattevi, Karem Slim *(CHU Clermont-Ferrand, Clermont-Ferrand)*; Herjean Marion, Romain Verhaeghe *(Clinique des 2 Caps, Coquelles)*; Lynda Bendjemar, Charre Lionel, Elie Mikhael, Rosa Montero Macías, Andrea Police, Vincent Villefranque, Enrico Volpin *(Hôpital Simone Veil, Eaubonne)*; Edouard Girard, Bertrand Trilling *(CHU Grenoble-Alpes, Grenoble)*; Emmanuel Boleslawski, Alexandre Chebaro, Houlzé-Laroye Constance, Vincent Drubay, Mehdi El amrani, Clarisse Eveno, Katia Lecolle, Louis Martin, Barbara Noiret, Guillaume Piessen, Stephanie Truant, Philippe Zerbib *(CHU Lille Hôpital Claude Huriez, Lille)*; Estelle Aubry, Armande Subayi Nkembi *(CHU Lille hôpital Jeanne de Flandres, Lille)*; Laurent Arnalsteen, Franck Denimal, Antoine Lamblin *(Hôpital Privé La Louvière, Lille)*; Quentin Ballouhey, Benjamin Barrat, François Caire, Niki Christou, Laurent Fourcade, Jerome Laloze, Margaux Mekann Bouv-Hez, Henri Salle, Abdelkader Taibi, Jeremy Tricard, Julie Usseglio *(CHU Limoges, Limoges)*; Sophie Chopinet, Emilie Gregoire, Diane Mege *(Timone, Marseille)*; Marion Delpont, Clement Jeandel *(CHU Montpellier (Lapeyronie), Montpellier)*; Claire Blanchard, Vincent Crenn, Stéphane de Vergie, Waast Denis, Emilie Duchalais, Henri Fragnaud, Nicolas Regenet, Jerome Rigaud, Yoann Varenne *(CHU Nantes, Nantes)*; Philippe Anract, Maxime Barat, David Biau, Pierre-Alban Bouche, Raphaël Dautry, Anthony Dohan, Louis Idier, Elena Lang, Camille Thouny, Stylianos Tzedakis *(Hôpital Cochin - APHP, Paris)*; François Audenet, Antoine Cazelles, Alexandre Chamouni, Mehdi Karoui, Gilles Manceau, Arnaud Mejean *(Hôpital européen Georges-Pompidou, Paris)*; Célia Crétolle *(Hôpital Necker Enfants Malades - APHP, Paris)*; Pauline Clermidi, Erik Hervieux, Tristan Langlais, Lorenzo Leonelli, Emeline Maisonneuve, Beaud Nicolas, Ophelie Perrot, Doriane Prost, Anne Thomin, Thouement Thouement *(Hôpital Trousseau - APHP, Paris)*; Noémie Girard *(Institut Curie, Paris)*; Yoann Athiel, Richard Berry, Guillaume Boddaert, Stéphane Bonnet, Nathalie Cathala, Christel Conso, Christine Denet, Anaïs Laforest, Yael Levy-Zauberman, Petr Macek, Annick Mombet, Didier Ollat, Adriana Scamporlino, Agathe Seguin-Givelet, Frederic Zadegan *(institut mutualiste montsouris, Paris)*; Chamakhi Ahmed Amine, Baratte Baratte, Emmanuel Chartier-Kastler, Nathalie Chereau, Pierre-Antoine Colas, Igor Duquesne, Sebastien Gaujoux, Laurent Genser, Gaëlle Godiris Petit, Claire Goumard, Ariola Hasani, Chetana Lim, Sophie Martellotto, Charlotte Melot, Fabrice Menegaux, Ugo Pinar, Marc Pocard, Morgan Roupret, Olivier Scatton, Thomas Seisen, Noullet Séverine, Célia Turco *(Pitie Salpetriere, Paris)*; Elie Chouillard, Belinda De Simone *(Centre Hospitalier Intercommunal Poissy Saint Germain en Laye, Poissy)*; Paul Beganton, Matthieu Boisson, Denis Frasca, Thomas Kerforne *(CHU Poitiers, Poitiers)*; Damien Bergeat, Lisa Corbiere, Anis Gasmi, Marwane Ghemame, Sonia Guérin, Zine-Eddine Khene, Marie Livin, Fedy Mahmoud, Betty Maillot, Aude Merdrignac, Frederic Mouriaux, Fabien Robin, Laurent Sulpice, Charles Vazeux *(CHU Rennes - Hopital Pontchaillou, Rennes)*; Alexis P Arnaud, Nicolas Bertheuil, Soline Bonneau, Clément Thierry Cazemajou, Ianis Cousin, Coralie Defert, Elisa Fustec, Melodie Juricic, Vincent Lavoue, Gwenaël Mevel, Krystel Nyangoh Timoh, Annaëlle Renault, Philippe Violas *(CHU Rennes - Hopital Sud, Rennes)*; Lilian Schwarz, Jean Jacques Tuech *(CHU Rouen, Rouen)*; Tristan Morichau-Beauchant, Francesco Nappi *(Centre Cardiologique du Nord, Saint Cenis)*; Radwan Kassir, Frederique Sauvat *(CHU Reunion, Saint Denis)*; Elie Haddad, Aurélien Scalabre, Sophie Vermersch *(CHU Saint Etienne, Saint Etienne)*; Federico Migliorelli *(Centre Hospitalier Intercommunal des Vallées de l’Ariège, Saint Jean de Verges)*; Andrea Patrizi, Romain Verhaeghe *(CMCO Côte d’Opale, Saint Martin Boulogne)*; Fabien Fredon, Alexia Roux *(Centre Hospitalier Roland Mazoin, Saint-Junien)*; Adeline Aimé, Anne-Cecile Ezanno, Brice Malgras *(Hia Begin, St Mande)*; Zineb Cherkaoui, Antonio D’urso, Emanuele Felli, Cristians Alejandro Gonzalez, Mihaela Ignat, Didier Mutter, Patrick Pessaux, Barbara Seeliger, Michel Vix *(HUS, Pole Hépato-Digestif / IHU-Strasbourg, Strasbourg)*; Hugo Gornes, Charlotte Vaysse, Kélig Vergriete *(CHU Toulouse, Toulouse)*; Pierre Berthoumieu, Ludivine Genre, Hélène Le Gall, Vincent Misrai, Trocard Pierre *(Clinique Pasteur, Toulouse)*; Olivier Abbo, Marc Chalhoub *(Hôpital des Enfants -CHU Toulouse, Toulouse)*; Martina Aida Angeles, Mathilde Del, Alejandra Martinez *(Institut Claudius Regaud - Institut Universitaire du Cancer de Toulouse, Toulouse)*; Laurent Brunaud, Thomas Fuchs-Buder, Antoine Vancon *(CHRU NANCY, Vandoeuvre-lès-Nancy)*. |
| **Gabon**: Camara Abraham Faya, Elvam Asaph, Elisee Baruwa, Gwen Hofman, Solomon Machemedze, Michael Mayombo Idiata, Roger Muhemi, Olivier Ndizeye, Elysé Nkunzimana, Jennifer O’Connor, Zachary O’Connor, Simplice Tchoba *(Bongolo Hospital, Lebamba)*; Natacha Boumas *(Centre Hospitalier universitaire mère enfant Fondation Jeanne Ebori, Libreville)*. |
| **Georgia**: Zaza Demetrashvili, Grigol Devidze, Givi Pisarevi *(N.Kipshidze Central University Clinic, Tbilisi)*. |
| **Germany**: Sabine Baumgarten, Linda Grüßer, Frank Hölzle, Pascal Kowark, Ana Kowark, Ali Modabber, Rolf Rossaint, Benedikt Schäfer, Julia Wallqvist, Philipp Winnand, Sebastian Ziemann *(University Hospital Aachen, Aachen)*; Matthias Anthuber, Tobias Broecheler, Florian Edlinger, Yvonne Goßlau, Alexander Hyhlik-Duerr, Florian Maksymiw, Ehab Shiban, Florian Sommer, Björn Sommer, Sebastian Wolf, Sebastian Zerwes *(University Hospital Augsburg, Augsburg)*; Katharina Beyer, Carsten Kamphues, Johannes Christian Lauscher, Lucas D. Lee, Florian N Loch, Christian Schineis *(Charité University Medicine - Campus Benjamin Franklin, Berlin)*; Ilgar Aghalarov, Orlin Belyaev, Chris Braumann, Annika Enste, Tim Fahlbusch, Torsten Herzog, Philipp Höhn, Julian Horn, Julia Jedanowski, Julia Knipschild, Andreas Minh Luu, Prem Vignesh Mohan, Leonie Siemen, Illya Slobodkin, Johanna Josefine Strotmann, Waldemar Uhl, Katerina Wolf *(St. Josef-Hospital, Bochum)*; Mark Coburn, Eva Egger, Klaus Eichhorn, Jana Enderes, Frederick Far, Alina Franzen, Tim R. Glowka, Erdem Güresir, Alexis Hadjiathanasiou, Jörg C. Kalff, Zaki Kohistani, Steffen Manekeller, Alexander Mustea, Chris Probst, Thomas Randau, Florian Recker, Patrick Schuss, Nadine Straßberger-Nerschbach, Sebastian Strieth, Hendrik Treede, Cornelius J. van Beekum, Hartmut Vatter, Markus Velten, Tim O. Vilz, Dieter Wirtz, Maria Wittmann *(University Hospital Bonn, Bonn)*; Michael Behr, Dirk Rolf Bulian, Benedikt Marche, Tobias Moczko, Robin Otchwemah, Sissy-Amelie Schulz, Panagiotis Thomaidis *(Cologne-Merheim Medical Center (CMMC), Witten / Herdecke University, Cologne)*; Christiane Bruns, Claus Cursiefen, Christian Domröse, Hans Fuchs, Ludwig Maximilian Heindl, Michael R. Mallmann, Christoph Mallmann, Dominik Alexander Ratiu, Alexander Christopher Rokohl *(University Hospital of Cologne, Cologne)*; Ulrich Bork, Ulrich Canzler, Marius Distler, Sandra Korn, Cornelia Meisel, Marcus Meusel, Andrea Petzold, Christian Praetorius, Janusz von Renesse, Juergen Weitz, Pauline Wimberger *(University Hospital Carl Gustav Carus, Technical University Dresden, Dresden)*; Nour Alkhanji, Georg Fluegen, Stephen Fung, Kefah Jaber, Wolfram Trudo Knoefel, Christian Vay *(University Hospital Duesseldorf, Duesseldorf)*; Octavian Clonda, Tatiana Cottin, Evelina Juodiene, Domantas Juodis, Biljana Petrovic, Oleksandra Solodarenko *(Rottal-Inn-Kliniken, Eggenfelden)*; Johannes Binder, Robert Grützmann, Danilo Hackner, Raymund E. Horch, Stefanie Junker *(Universitätsklinikum Erlangen, Erlangen)*; Mani Arsalan, Severine Banek, Felix Chun, Sara Fatima Faqar-Uz-Zaman, Daniel Keese, Luis Kluth, Udo Rolle, Andrea Schmedding, Andreas Schnitzbauer, Arnaud Van Linden, Thomas Walther *(Frankfurt University Hospital, Goethe University, Frankfurt)*; Jörg Bayer, Jürgen Beck, Helge Eberbach, Christian Fung, Luisa Mona Kraus, Christian Leiber, Nicolas Neidert, Richard Sandkamp, Daniel Schlager, Oliver Schnell, Antonia Schulte, Jakob Strähle *(University Medical Center Freiburg, Faculty of Medicine, Freiburg)*; Stefanie Jarmusch, Helmut Franz Georg Novotny, Fritz Spelsberg *(Klinikum Fürstenfeldbruck, Fürstenfeldbruck)*; Johannes Becker, Christian Fulghum, Bernhard Gonschor, Marit Herbolzheimer, Lena Keppler, Sina Nicolaiciuc, Alexander Trulson, Holger Vogelsang, Benno Zimmermann *(Klinikum Garmisch-Partenkirchen, Garmisch-Partenkirchen)*; Amir Ali Akbari, Andreas Boening, Fabian Edinger, Andreas Hecker, Matthias Hecker, Michael Knitschke, Christian Koch, Martin Reichert, Michael Sander, Götz Schmidt, Emmanuel Schneck, Eberhard Uhl *(University Hospital Giessen and Marburg, Giessen)*; Silvia Flachs Nóbrega, Philipp Kauffmann, Clemens Miller, Marcus Nemeth, Denise Sievers, Susanne Wolfer *(University Hospital Goettingen / Universitätsmedizin Goettingen, Goettingen)*; Ulrich Kisser, Jorg Kleeff, Johannes Klose, Kerstin Lorenz, Nancy Papendick, Stefan Plontke, Ulrich Ronellenfitsch, Ingmar Seiwerth, Susanne Steer, Christoph Thomssen, Jörg Ukkat *(University Hospital Halle, Halle)*; Christian W. Dumpies, Isabel Fischer, Michael Gessner, John Hanke, Friederike Klauke, Sebastian Leuschner, Thomas Mendel, Katharina Müller, Birte Schmidt, Valentin Schreiter, Peter Stosberg, Franziska Vinz *(BG Klinikum Bergmannstrost, Halle (Saale))*; Beate Herbig, Johannes Sander, Thilo Maria Schulte *(Schön Klinik Hamburg Eilbek, Hamburg)*; Christian Stephan Betz, Julian Bewarder, Johannes Bier, Arne Böttcher, Simon Burg, Chia-Jung Busch, Lara Bußmann, Martin Gosau, Annika Heuer, Jakob Izbicki, Till Orla Klatte, Daniela König, Leon-Gordian Köpke, Nikolaus Moeckelmann, Christine Nitschke, Mark Praetorius, Matthias Priemel, Rupert Stadlhofer, Martin Stangenberg, Faik G. Uzunoglu, Lukas Wittig, Henrike Zech, Nina Zeller *(University Medical Center Hamburg-Eppendorf, Hamburg)*; Christian Peiper, Frederic Roux, Tsiona Spaeth *(Evangelisches Krankenhaus Hamm, Hamm)*; Roland Fricker, Thomas Müller, Lars Schröder *(Klinikum Hanau, Hanau)*; Mohammed Alasmari, Clara Boeker, Ibrahim Hakami, Ibrahim Abdullah Hakami, Julian W Mall *(KRH Nordstadt-Siloah Hospitals, Hannover)*; Ioannis Kyritsis, Stefan Welter *(Lung Clinic Hemer, Hemer)*; Christian Graeb, Kristin Huber-Strößner, Karine Nikolaieva *(Sana Klinikum, Hof)*; Daniela Branzan, Markus Doss, Ines Gockel, Carolin Jödicke, Georg Osterhoff, Christina Pempe, Andreas Roth, Robert Sucher *(University Hospital Leipzig, Leipzig)*; Tina Adler, Kathrin Kelly, Judith Lindert, Janica Merkle, Julia Siebert *(University Hosital Schleswig- Holstein, Lübeck)*; Christoph Hirche, Ulrich Kneser, Christian Tapking *(BG Trauma Center Ludwigshafen, Ludswigshafen am Rhein)*; Rachit Agrawal, Konstantinos Gousias, Homeira Qureischie *(St Marien Hospital Lünen, Lünen)*; Roland Croner, Lisa Koslowski, Hardy Krause, Frank Meyer, Anke Rissmann, Salmai Turial *(University Hospital Magdeburg, Magdeburg)*; Bilal Al-Nawas, Marco Johannes Battista, Jan Goedeke, Annette Hasenburg, Julia Heider, Valerie Catherine Linz, Lena Katharina Mueller, Simon Zeller *(University Hospital Mainz, Mainz)*; Sina- Louisa Patrizia Jentschura, Karl-Friedrich Kowalewski, Maximilian Kriegmair, David Männle, Nuh Rahbari, Marie-Claire Rassweiler-Seyfried, Christoph Reissfelder, Nicole Rotter, Claudia Scherl, Steffen Seyfried *(Mannheim University Medical Center (Universitätsmedizin Mannheim), Mannheim)*; Andreas Kirschniak, Jens Rolinger, Peter Wilhelm *(Kliniken Maria Hilf, Moenchengladbach)*; Franz G. Bader, Anna Eleonora Gut, Stephanie Ottl *(Isarklinikum, München)*; Alexandra Viktoria Behr, Alexandros Diamantis, Andreas Fichter, Jens Gempt, Florian Grill, Matthias Heck, Daniel Jira, Michael Kallmayer, Florestan Koll, Stefan Luhne, Bernhard Meyer, Robert Patachia, Ilaria Pergolini, Daniel Reim, Seyer Safi, Christoph Schäffer, Moritz Schirren, Arthur Wagner, Helmut Wegmann, Markus Wirth, Zhaojun Zhu *(Klinikum Rechts der Isar TUM School of Medicine, Munich)*; Ughur Aghamaliyev, Markus Albertsmeier, Mahmoud Almaghrabi, Wolfgang Böcker, Jan Bruder, Konstantin Frank, Viktoria Herterich, Verena Huber, Matthias Ilmer, Christian Kammerlander, Alexander M. Keppler, Carl Neuerburg, Viktor H. von Ehrlich-Treuenstätt, Jens Werner *(Ludwig Maximilian University of Munich - Großhadern, Munich)*; Nikolaus Börner, Florian Fegg, Daniela Hartmann, Roland Ladurner, Paris Liokatis, Kathrin Patzer, Justin Gabriel Schlager, Wenko Smolka, Petra Zimmermann *(Ludwig Maximilian University of Munich - Innenstadt, Munich)*; Rudolf Hatz, Diana Steinhart, Mircea Gabriel Stoleriu *(Asklepios Pulmonary Hospital, Munich Gauting)*; Claudio Glowalla, Tim Saier, Dorien Schneidmueller *(Berufsgenossenschaftliche Unfallklinik Murnau, Murnau)*; Karl Wilhelm Henkel, Josef Stadler, Martin Steiner *(RoMed Klinik Prien am Chiemsee, Prien am Chiemsee)*; Katharina Hölz, Julia Christina Kaiser, Christian Knorr *(Klinikum St. Hedwig, Barmherzige Brüder, Regensburg)*; Stefan M. Brunner, Britta Kuehlmann, Kyriakos Oikonomou, Karin Pfister, Lukas Prantl *(University Hospital Regensburg, Regensburg)*; Roland Flurschütz, Jonas Herzberg, Human Honarpisheh, Marie Kröger, Dominic Lepiorz, Charlotte Luths, Andreas Niemeier, Yara Sras, Tim Strate, Thore Winter *(Krankenhaus Reinbek St. Adolf-Stift, Reinbek)*; Kai Nowak, Tobias Reinhard *(RoMed Klinikum Rosenheim, Rosenheim)*; Thomas Freiman, Florian Gessler, Sae-Yeon Won *(University Hospital Rostock, Rostock)*; Gregor A. Stavrou, Rizky Widyaningsih *(Klinikum Saarbruecken, Saarbruecken)*; Sebastian Hoffmann, Ruth Christine Schäfer, Johannes Tobias Thiel *(BG Klinik, Tübingen)*; Elisa Bertolani, Alfred Königsrainer, Christian Konrads, Markus W. Löffler, Markus Quante, Christoph Steidle, Lisa Überrück, Can Yurttas, Patrick Ziegler, Alexander Zimmermann *(University Hospital Tuebingen, Tuebingen)*; Christian Bolenz, Davut Dayan, Jens Greve, Thomas K. Hoffmann, Wolfgang Janni, Simon Laban, Niklas Löbig, Fabienne Schochter, Julius Malte Vahl, Felix Wezel *(University Hospital Ulm, Ulm)*; Veronika Greif, Silke Pusch von, Stefan Schmidbauer *(Kreisklinik Wolfratshausen, Wolfratshausen)*; Markus Hirschburger, Imke Marsch, Rolf Schneider *(Klinikum Worms, Worms)*; Lars Boenicke, Stephan Degener, Johannes Doerner, Nici Markus Dreger, Franz Christian Horstmeier, Jakob Kruschwitz, Adrian Rombach, Nele Schmidt, Rose Seiberth, Jaswinder Singh, Marieke Smit, Friedrich-Carl von Rundstedt, Hubert Zirngibl *(Helios Universitätsklinikum Wuppertal (Universität Witten/Herdecke), Wuppertal)*; Joachim Diessner, Sabine Friedrich, Christoph-Thomas Germer, Philipp Helmer, Johannes Herrmann, Peter Kranke, Hubert Kübler, Johan Lock, Christopher Lotz, Rainer Meffert, Patrick Meybohm, Sophie Müller, Quirin Notz, Maria Popp, Nicolas Schlegel, Tobias Schlesinger, Benedikt Schmid, Magdalena Sitter, Andreas Steinisch, Agnes Treutlein, Anne van den Berg, Armin Wiegering, Thomas Erik Wurmb *(University Hospital Würzburg, Würzburg)*. |
| **Ghana**: Nana Kwaku Agyeman-Duah, Enoch Appiah, Ralph Armah, Christopher Asare, Lawrence Awere-Kyere, Dennis Daary, Delali Gakpetor, Stephen Minlah Allah, Ambe Obbeng, Dorcas Osei-Poku, Diana Puozaa, Enoch Tackie *(Greater Accra Regional Hospital, Accra)*; Nii Armah Adu-Aryee, Nelson Agboadoh, Offei Asare, Antoinette Bediako Bowan, George Darko Brown, Joe-Nat Clegg-Lamptey, Florence Dedey, Cedric Dery, Benjamin Sena Fenu, Marian Abedua Harrison, Philemon Kumassah, Ekins Kuuzie, Josephine Nsaful, David Olatayo Olayiwola, Cecilia Smith *(Korle-Bu Teaching Hospital, Accra)*; Charles Banka, Romeo Hussey *(Berekum Holy Family Hospital, Berekum)*; Diallo Abdoul Azize, Luke Adagrah Aniakwo, Yvonne Adofo-Asamoah, Evans Kofi Agbeno, Meshach Manu Agyapong, Thomas Agyen, Kwasi Agyen Mensah, Baba Alhaji Bin Alhassan, Mabel Amoako-Boateng, Peter Appiah-Thompson, Nita Asamoa-Manu Gyimah, Moses Asante- Bremang, Alvin Asante-Asamani, Henry Atawurah, Anthony Baffour Appiah, Richard Ogirma Baidoo, Ebikela Ivie Baidoo, Benedict Boakye, Abigail Boateng, Dora Dadoe, Makafui Seth Caleb-Joshua Kwasi Dayie, Samuel Debrah, Dr Kingsley Doku, Enti Enti, Sebastian Ken-Amoah, Patience Koggoh, Richard Kpangkpari, Patrick Maison, Samuel Mensah, Philip Mensah, Teresa Aba Mensah, Martin Tangnaa Morna, Jilac Nimako-Mensah, John Nkrumah, Michael Nortey, Emmaunel Owusu Ofori, Isabella Naa Morkor Opandoh, Ethel Osei-Tutu, Jefferson Owusu Adae, Kofi Quansah, Elizabeth Quartson, Ganiyu Adebisi Rahman, David Walawah, Makafui Yigah, Safia Yussif *(Cape Coast Teaching Hospital, Cape-Coast)*; Nuna Jiagge, Emmanuel Nachelleh *(Ho Teaching Hospital, Ho)*; Jane Acquaye, Kwabena Agbedinu, Fareeda Agyei, Akosua Agyemang-Prempeh, Kwabena Amo-Antwi, Michael Amoah, George Amoah, Yaw A Amoako, Daniel Gyawu Aning, Frank Ankobea-Kokroe, Dominic Annor Mintah, David Anyitey-Kokor, Adu Appiah-kubi, Joshua Arthur, Vincent Ativor, Isaac Barnor, Yasmine Braimah, Regina Darko- Asante, Anthony Davor, Mohammed Duah Issahalq, Mawutor Dzogbefia, Tano Emile, Papa Fiifi - Yankson, Valerie Gaveh, Senyo Gudugbe, Solomon Gyabaah, Frank Enoch Gyamfi, Adam Gyedu, Derrick Gyimah, Bernard Hammond, Boakye - Yiadom Jonathan, Yorke Joseph, Thomas Okpoti Konney, Anna Konney, Kwasi Kusi, Ishmael Kyei, Agbenya Lovi, Nuhu Naabo, Boateng Nimako, Beauty Nyadu, Obed Ofori Nyarko, Ben Blay Ofosu-Barko, Philip Peprah Oppong, Anita Osabutey, Martha Poku, Robert Sagoe, Abiboye Yifieyeh *(Komfo-Anokye Teaching Hospital, Kumasi)*; George Ansong *(Tamale Central hospital, Tamale)*; Adam Abass, Alhassan Abdul-Mumin, Theophilus Adjeso, John Abanga Alatiiga, Munira Amadu, Nathaniel Annan, German Azahares Leal, Mohammed Bukari, Alexis Buunaaim, Ernest Cheyuo, Latif Daboo Salifu, Michael Damah, Malcolm Dery, Edem Kojo Dzantor, Odoniel Guerra Garcia, Yabasin Iddrisu Baba, Adamu Issaka, Abdul-Jalilu Mohammed Muntaka, James Murphy, Yaa Nyarko Agyeman, Wisdom Opoku Amankwaa, Imoro Osman, Samuel Pie, Anwar Sadat Seidu, Mohammed Sheriff, Ana Maria Simono Charadan, Stephen Tabiri, Abraham Titigah, Mundashiru Yahaya, Musah Yakubu, Edwin Mwintiereh Ta-ang Yenli *(Tamale Teaching Hospital, Tamale)*. |
| **Greece**: Ioannis Grypiotis, Nikolaos Kiriakopoulos, Georgios Koliopoulos, Vasilis Kyvelos, Georgia Micha, Triada Papadopoulou *(‘Elena Venizelou’ General and Maternity hospital of Athens, Athens)*; Dimitrios Balalis, Evangelos Fradelos, Dimitrios Korkolis *(Agios Savvas Anticancer Hospital, Athens)*; Nicholas Alexakis, Kyveli Angelou, Dimitrios Haidopoulos, Anastasia Prodromidou, Alexandros Rodolakis, Nikolaos Thomakos *(Alexandra General Hospital, Athens)*; Dimitris Psychogios, Pantelis Antonakis, Konstantinos Bramis, Leonidas Chardalias, Ioannis Contis, Nikos Dafnios, Dionysios Dellaportas, Papalouka Dimitra, Georgios Fragkoulidis, Georgios Gkiokas, Antonios Gklavas, Theodoros Hadjizacharias, Dimitra Karageorgou, Manousos Konstadoulakis, Christina Kontopoulou, Dimitrios Massaras, Nikolaos Memos, Ioannis Papaconstantinou, Dimitrios Politis, Andreas Polydorou, Konstantinos Stamatis, Theodosios Theodosopoulos, Antonios Vezakis *(Aretaieion Hospital, Athens)*; Konstantinos Avgerinos, Jevgeni Katunin, Aristotelis Kechagias, Dionysia Kelgiorgi, Neoklis Kritikos, Pasi Pengermä *(Athens Bioclinic Hospital, Athens)*; Theodosios Bisdas, Argyrios Ioannidis, Michael Konstantinidis, Sofia Konstantinidou, Nikolaos Patelis *(Athens Medical Center, Athens)*; Maria Ioanna Antonopoulou, Eirini Deskou, Vasileios Kalles, Dimitrios K. Manatakis, Nikolaos Stamos, Nikolaos Tasis *(Athens Naval and Veterans Hospital, Athens)*; Nikolaos Arkadopoulos, Nikolaos Danias, Panagiota Economopoulou, Maximos Frountzas, Panagiotis Kokoropoulos, Nikolaos Michalopoulos, Jonida Selmani, Theodoros Sidiropoulos, Panteleimon Vassiliu *(Attikon University General Hospital, Athens)*; Kosmas I. Paraskevas *(Central Clinic of Athens, Athens)*; Dimitrios Bartziotas, Konstantinos Bouchagier, Ilias Galanis, Theodosis Kalamatianos, Stylianos Kapiris, Angeliki Kolinioti, Eleni Mavrodimitraki, Panagiotis Metaxas, Alexandrina Nikova, Michail Psarologos, Maria Sotiropoulou, George Stranjalis *(Evaggelismos General Hospital, Athens)*; Konstantinos Albanopoulos, Panagiotis Kondilis, Gavriella Zoi Vrakopoulou *(Evgenideio Hospital, Athens)*; Aristeidis Chrysovergis, Georgios Chrysovitsiotis, Evangelos Giotakis, Vasiliki Kanellopoulou, Efthymios Kyrodimos, Andreas Larentzakis, Pavlos Pantos, Vasileios Papanikolaou, Spyridon Potamianos, Charalampos Theodoropoulos, Alexandra Triantafyllou, Tania Triantafyllou *(Hippocratio General Hospital, Athens)*; Kleoniki Georgousi, Peter Panagiotou, Ekaterini Christina Tampaki *(KAT Athens General Hospital, Athens)*; Emmanouil Avramidis, George Babis, Evangelos Zafeiris *(Konstantopouleio General Hospital of Athens, Athens)*; Afroditi Antoniou, Nikolaos Bessias, Dimitris Maras, Theofanis Papas, Konstantinos Roditis, Ioannis Tsagkos, Paraskevi Tsiantoula *(Korgialenio-Benakio Hellenic Red Cross Hospital, Athens)*; Andreas Alexandrou, Efstratia Baili, Alexandros Charalabopoulos, Dimitrios Dimitroulis, Panagiotis Dorovinis, Zoe Garoufalia, Prodromos Kanavidis, Ioannis Karavokyros, Lysandros Karydakis, Stylianos Kykalos, Eleandros Kyros, Nikolaos Machairas, Aikaterini Mastoraki, Nikolaos Nikiteas, Alexandros Papalampros, Dimitrios Schizas, Antonia Skotsimara, Paraskevas Stamopoulos, Athanasios Syllaios, Alexis Terras, Nefeli Tomara, Gerasimos Tsourouflis, Ilias Vagios, Constantinos Zografos *(Laiko University Hospital, Athens)*; Konstantinos Apostolou, Nikolaos Georgopoulos *(Mediterraneo Hospital, Athens)*; Theofani Antoniou, Christina Antzaka, Areti Falara, Socrates Fragoulis, Panagiotis Ftikos, Fedra Matsouka, Konstantinos Perreas, Panagiota Rellia, Evangelia Samara, Anna Smirli, Androniki Tasouli, Apostolos Thanopoulos *(Onassis Cardiac Surgery Center, Athens)*; Petros Loukas Chalkias, Georgia Dedemadi, Panagiotis Mourmouris, Andreas Skolarikos, Nikoletta Theochari, Lazaros Tzelves *(Sismanoglio - Amalia Fleming General Hospital, Athens)*; Stylianos Gaitanakis, Theodoros Milas, Emmanuel Theodorakis *(Sotiria General Hospital of Thoracic Diseases, Athens)*; Paraskevi Karona, Pagona Kastanaki, Angelos Tzouganakis *(Chania General Hospital ‘St George’, Chania)*; Christos Agalianos, Ioannis Tsouknidas, Andreas Xenakis *(Naval and Veterans Hospital of Crete, Chania)*; Emmanuel Chrysos, Konstantinos Lasithiotakis, Taxiarchis Nikolouzakis, Sofia Xenaki, Evangelos Xynos *(University Hospital of Heraklion Crete, Heraklion Crete)*; Eftychios Lostoridis, Eleni-Aikaterini Nagorni, Antonio Pujante, Paraskevi Tourountzi *(Kavala General Hospital, Kavala)*; Aggeliki Al, Kyriaki Baxevanidou, Konstantinos Bouliaris, Matheos Efthimiou, Christos Kalfountzos, Georgios Koukoulis, Vasileios Lachanas, Konstantinos Petropoulos, Ioannis Tsitiridis *(General Hospital of Larissa ‘Koutlimpaneio and Triantafylleio’, Larissa)*; Fragkiskos Angelis, Eleni Arnaoutoglou, Ioannis Baloyiannis, Metaxia Bareka, Anna Bouronikou, Gregory Christodoulidis, Alexandros Daponte, Maria Fergadi, Nick Gkolias, Eleni Gkrinia, Jiannis Hajiioannou, Nikos Kalogritsas, Eleni Karoni, Christos Korais, Giorgos Krestinidis, Antigoni Ktisti, Dimitrios Magouliotis, Charikleia Maiou, Konstantinos Malizos, Maria Minasidou, Maria Ntalouka, Anna Maria Ntziovara, Effrosyni Palla, Konstantinos Perivoliotis, Fani Saini, Athina Samara, Athanasios Saratziotis, Charalampos Skoulakis, Efthymios Solomi, Konstantinos Stamoulis, Christos Dimitrios Terzoudis, Evangelia Tsironi, George Tzovaras, Kyriaki Vallianou, Dimitris Zacharoulis, Anna Ziogkou, Κωνσταντίνος Δακής *(General University Hospital of Larissa, Larrisa)*; Liolis Elias, Ioannis Maroulis, Francesk Mulita, Kerasia-Maria Plachouri, Michail Vailas *(General University Hospital of Patras, Patras)*; Charalampos Doitsidis, Eva Filo, Ioanna Gkalonaki, Eleni Kogia, Konstantina Kontopoulou, Magdalini Mitroudi, Christina Panteli, Ioannis Patoulias, Olga Ioulia Semkoglou, Dimitrios Sfoungaris, Ioannis Valioulis, Γιωργος Κουτσουμης *(G. Gennimatas Thessaloniki General Hospital, Thessaloniki)*; Ioannis Astreidis, Panagiotis Christidis, Orestis Ioannidis, Lydia Loutzidou, Antonis Mantevas, Konstantinos Paraskevopoulos, Dimitris Tatsis *(George Papanikolaou General Hospital of Thessaloniki, Thessaloniki)*; Apostolos Athanasiadis, Themistoklis Dagklis, Ioannis Kalogiannidis, Georgios Kapetanios, Apostolos Mamopoulos, Chrysoula Margioula-Siarkou, Stamatios Petousis, Ioannis Tsakiridis *(Hippocratio Hospital, Thessaloniki)*; Christos Anthoulakis, Chrysanthos Christou, Antonios Fantakis, Eirini Iordanidou, Christos Kaselas, Sousana Panagiotidou, Vasileios Papadopoulos, Kyriakos Papavasiliou, Athanasios Piachas, Ioannis Siasios, Ioannis Spyridakis, Theodoros Theodoridis, Andreas Tooulias, Eleftherios Tsiridis, Maria Tsopozidi, Georgios Tsoulfas *(Papageorgiou General Hospital, Thessaloniki)*. |
| **Guatemala**: María Alemán, Estuardo Brolo, Alitza Gutiérrez Ruiz, Ana Lucia Lemus *(Hospital Universitario Esperanza, Guatemala)*; Jennifer Greenberg, Krisna Mishel Morales Chew, José Rodrigo Oliva, Alejandra Rodas *(Centro Clínico Cabeza y Cuello, Guatemala City)*; María Alejandra De León Lima, Ismar Lopez Muralles, Ana Lucía Portilla, Gustavo Recinos, Felipe Solares *(Hospital General De Enfermedades, Guatemala City)*; Maria-Lorena Aguilera-Arevalo, Gaby Ajcip, Claudia Anton, Jacqueline Carrera, Jose Cojulun, Mario-Andrés Flores, Noriega José, Carlos Adolfo Marroquín Paiz, Steffanía Morales, Ramiro Najera, Eduardo Quiñónez Lorenzana, Pablo Rivera, Lesly Rodas, Victor Santos, Dianne Sosa, Natalia Ybarra *(Hospital General San Juan De Dios, Guatemala City)*; Amalia Barrios Duarte, José David Pérez Cajti, Carlos Régil *(Hospital Herrera Llerandi, Guatemala City)*; walter A Osorio, Rember Rosales Arriola, Luis-Fernando Talé-Rosales *(Hospital Juan Jose Arevalo Bermejo, Guatemala City)*; Danilo Herrera, Servio Tulio Torres Rodríguez, Sergio Alejandro Villeda *(Hospital San Vicente, Guatemala City)*; Joshua Anicetti, Megan Lowey, Andrea Michelle Lowey Medina *(Sanatorio Las Majadas, Guatemala City)*; Javier Ardebol, Kathia Barillas, Sabrina Barillas, Francis De Leon, Salvador Recinos *(Symmetria, Guatemala City)*; Miguel-Angel Marroquin-Alpirez, Yessica Yax *(Hospital Nacional de San Marcos, San Marcos)*. |
| **Hong Kong SAR, China**: Sophie Hon, Yuk Ho Liu, Alex Qinyang Liu, Shirley Liu *(Alice Ho Miu Ling Nethersole Hospital, Hong Kong)*; Hei Tung Natalie Chiu, Chi Man Tom Chow, Victor Hau, Ho Wai Ip, Brian Mak, Chung Ying Mok, Dennis Ng, Yin yu Eva Siu, Kiu Fung Wong *(North District Hospital, Hong Kong)*; Jingya Jane Pu, Yu-xiong Su *(Queen Mary Hospital, Pok Fu Lam)*; Kit Ying Au-Yeung, David Yuen Chung Chan, Albert Chan, Shannon Melissa Chan, Tsz Ching Chang, Tor Wo Chiu, Wang Kei Chiu, Kaori Futaba, Zhexi He, Man Fung Ho, Kevin Ki Wai Ho, Jacky Yan Kit Ho, Janet Wui Cheung Kung, Cheuk Ho Lam, Rainbow W.H. Lau, Samuel Ka Kin Ling, Hon Ting Lok, Tony Wing Chung Mak, Chi-Fai Ng, Simon Ng, Kelvin Kwok-Chai Ng, Calvin S.H. Ng, Michael Tim Yun Ong, Teresa Tan, Sui Fan Tang, Jeremy Yuen-Chun Teoh, Anthony Teoh, Bess Siu Yan Tsui, George Kwok Chu Wong, Randolph Wong, Kwok Chuen Wong, Chi Hang Yee *(Prince of Wales Hospital, Sha Tin)*. |
| **Hungary**: Zsuzsanna Antal, Attila Kalman, Peter Voros *(Ist Department of Pediatrics, Semmelweis University, Budapest)*; Kiarash Bahrehmand, Timea Echim, Tamás Mersich, Zoltan Novak, Tamás Sztipits, Dániel Wettstein *(National Institute of Oncology, Budapest)*; Laszló Ádám Bihari, László Hidi, Laszlo Piros, Balazs Rózsa, Peter Sotonyi, Lilla Szatai *(Semmelweis University (please use for all units), Budapest)*; György Herczeg, Bálint Pordány, Fanni Tornyi *(Szent Imre Egyetemi Oktatókórház, Budapest)*; Kristof Dede, Tamás Egyed, György Saftics *(Uzsoki Hospital, Budapest)*; Judit Kulcsicka-Gut, Zsófia Sipos, Dezso Toth *(Miskolc Academic County Hospital, Miskolc)*. |
| **India**: Bhavin Patel, Dhaivat Vaishnav *(Zydus Hospital, Ahmedabad Gujarat)*; Raghunandan Gorantlu Chowdappa, Hardil Majmudar, Saptak Mankad, Sohilkhan Pathan *(Shri Krishna Hospital and pramukhswami medical college, Anand , Gujarat)*; Sharathkumarkl L, Irappa Madabhavi, Lokesh Sasatti *(Kerudi Cancer Hospital, Bagalkot)*; Dhananjaya Bhat, Subramanyam Mahankali, Santhosh kumar Sampengere Annayappa *(Aster RV Hospital, Bangalore)*; Tulika Agrawal, Premkumar Anandan, Aditya Baindur, Manjunath Bd, Savitha C, Anitha G S, Sandeep Harigond, Tejeswini K K, Venkatesh Kesarla, Sunil Kumar Venkatappa, Mallikarjuna Manangi, Hareesh P B, Dr jyothi k r Ranjan, Athish Shetty, Santhosh Shivashankar Chikkanayakanahalli, Tanvi Sunil, Shreya Syamala, Kavya Tharanath *(Victoria Hospital, Bangalore)*; Aditya Atal, Niranjana Rajagopal, Sumit Thakar *(Sri Sathya Sai Institute of Higher Medical Sciences, Bengaluru)*; Srinath B S, Kavitha Jain, Vinod Nk, Thirumanikandan P L *(Sri Shankara Cancer Hospital and Research Centre, Bengaluru)*; Aruna Kumar, Nitu Mishra, Sushruta Shrivastava, Dr Rekha Wadhwani *(Gandhi Medical College and Sultania Zanana Hospital, Bhopal)*; Reyaz Ahmad, Zainab Ahmad Haq, Prateek Behera, Ritika Dhurwe, Rehan Haq, Vaibhav Jain, Anuj Jain, Sunaina Karna, Manoj Nagar, Kameshwarachari Pushpalatha, Sumit Raj, John Ashutosh Santoshi, Pooja Singh, Virendra Verma, Dr Vaishali Waindeskar, Dr Moorat Singh Yadav *(ALL INDIA INSTITUTE OF MEDICAL SCIENCE BHOPAL, BHOPAL)*; Zaheda Aziz, Koyel Chakraborty, Preetam Chappity, Gurudip Das, Debajyoti Datta, Saubhagya Kumar Jena, Madhabananda Kar, Susanta Khuntia, Pankaj Kumar, Ravi Kumar, Aswathi Kv, Abhijeet Mishra, Tushar Mishra, Swastik Mishra, Yash Mittal, Dillip Muduly, Ritesh Panda, Sibasish Panigrahi, Sucheta Parija, Saroj Patra, Bikram Rout, Rabi Sahu, Saurav Sarkar, Arunkumar Sekar, Sweta Singh, Sanjibani Sudha, Mahesh Sultania, Sujit Tripathy, Paulson Varghese *(All India Institute Of Medical Sciences - Bhubaneswar, Bhubaneswar)*; Anand Gupta, Rajeev Kansay, Robin Kaushik, Simrandeep Singh *(Government medical college hospital, Chandigarh)*; Sunil Kumar Gupta, Lileswar Kaman, Madhivanan Karthigeyan, Siddhant Khare, Vishal Kumar, Sandeep Mohindra, Ninad Patil, Pravin Salunke, Ajay Savlania, Kavindra Singh, Manjul Tripathi *(Postgraduate Institute of Medical Education & Research, Chandigarh, India, Chandigarh)*; Pradeep Krishna RV, Gomathy Narasimhan, Ashwin Rammohan, Mohamed Rela *(Dr.Rela Institute & Medical Centre, Chennai)*; Akhila Appukuttan, Swati Goudar, Shweta Mallick, Sudheer Othiyil vayoth, Anupama Rajanbabu, Christi Titus *(Amrita Institute of Medical Sciences Hospital, Cochin)*; Ezhir Selvan Chidambarasamy, Dr Devdas Madhavan, Anandan Murugesan, Kuppurajan Narayanasamy, Barani kumar P B Pb, Dr Firoz Rajan, Anbukkani Subbian, Paari Vijayaragavan *(Kovai Medical Centre and Hospital, Coimbatore)*; Rahul Gupta, Arvind Kumar *(Synergy Institute of Medical Sciences, Dehradun)*; Biplob Borthakur, Tapan singh Chauhan, Akhil Govil, Shubhra Gupta, Vijay Mohan Hanjoora, Monish Raut, Ashish Sharma, Aseem Srivastava, Saurabh Tiwari, Vartika Vishwani *(Artemis Health Institute, Gurugram)*; Nissi Evelyn. R, Karuna Sree Pendyala, Navakoti Prasad *(Government Dental College and Hospital, Hyderabad)*; Jyoti Bothra, Mainak Deb, Koushik Herle, Harish Jayaram, Lavanya Kannaiyan, Abirami Krithiga, Mukta W *(Rainbow children’s hospital, Hyderabad)*; Dr. Anil Matai, Pooja Nagpal, Prachi Pathak *(Santokh ba Durlabhji memorial hospital, Jaipur)*; Sumit Banerjee, Ramkaran Chaudhary, Gautam Ram Choudhary, Ankita Chugh, Pawan Dixit, Abhay Elhence, Nitesh Gahlot, Mayank Garg, Navdeep Kaur Ghuman, Deepak Jha, Prakash Kala, Amanjot Kaur, Vijay Madduri, Sanjeev Misra, Himanshu Pandey, Puneet Pareek, Manish Pathak, Kirtikumar J Rathod, Mahaveer Singh Rodha, Rahul Saxena, Naveen Sharma, Shashank Shekhar, Mahendra Singh, Pratibha Singh, Bhaskar Suryanarayanan, Jeewan Ram Vishnoi *(All India Institute of Medical Sciences (AIIMS), Jodhpur, Jodhpur)*; Shivang Amin, Vishal Bhende, Rohit Kumar, Tanishq Sharma *(Shree Krishna Hospital, Karamsad)*; Kesavan Murugesan, Sathish Muthu, Abhinav Balachandar Subbiah Ramasamy *(Government Hospital, Velayuthampalayam, Karur)*; Debarshi Chatterjee, C Gerber Gerber, Indranil Ghosh, Upasana Naskar *(Institute of Neurosciences, Kolkata, Kolkata)*; Gaurav Aggarwal, Sanjit Kumar Agrawal, Azaz Ahmed, Sujoy Gupta, Prateek Jain, Deepak Jain, Vishal Kewlani, Amrit Pipara, Noopur Priya, Roopak Raja, Sudip Shakya, Abhishek Sharma, Robin Thambudorai *(Tata Medical Center, Kolkata)*; Dimple Kharkongor, Anjoo Agarwal, Naseem Akhtar, Akshay Anand, Mona Asnani, Ankur Bajaj, Arun Chaturvedi, Akhilanand Chaurasia, Loreno E. Enny, Surabhi Garg, Sameer Gupta, Anoop kumar Jaiswal, Somil Jaiswal, Yashpal Jaware, Navneet Kala, Ruchi Karnatak, Apjit Kaur, Vijay Kumar, Manoj Kumar, Ambrish Kumar, Upander Kumar, Amit kumar Shrivastava, Seema Mehrotra, Brijesh Mishra, Anand Mishra, Dr Namrata, Bal Krishna Ojha, Ahmad Ozair, Uma Shanker Pal, Amita Pandey, Nancy Raja, Shiv Rajan, Pooja Ramakant, Ashutosh Roy, Rekha Sachan, Satyanarayan Sankhwar, Pushp Sankhwar, Divya Sarin, Ayushi Shukla, Sushil Singh, Mohit Singh, Kul Ranjan Singh, Renu Singh, Uma Singh, Urmila Singh, Vandana Solanki, Abhinav Arun Sonkar, Chhitij Srivastava, Parijat Suryawanshi, Vivek Tewarson, Rajat Verma, Manju Lata Verma, Awdhesh Yadav *(King George’s Medical University, Lucknow)*; Gaurav Agarwal, Gyan Chand, K.m.m.vishvak Chanthar, Dileep Hoysal, Anjali Mishra *(Sanjay Gandhi Post Graduate Institute Of Medical Sciences, Lucknow)*; Nitin Batra, Arun Bhatti, Rupali Chopra, Shakina David, Tapasya Dhar, Uma Kant Dutt, Rohini Dutta, Sumir Gandhi, Parvez David Haque, Ritu Jain, Paul Sudhakar John B, Sreejith Kannummal Veetil, Gurvinder Kaur, Navneet Kumar, Anil Luther, Anupam Mahajan, Amit Mahajan, Kavita Mandrelle, Shefin Mathews, Vishal Michael, Partho Mukherjee, Dr Pinki Pargal, Rajesh Paul, Pranay Pawar, Anupam Phillip, Rachel Phillips, Abhishek Samuel, Noel Singh, Inderjot Singh, Abhijit Singh, Sarvpreet Singh Grewal, Anusha Singhania, Selven Thirumalai, Ashish Varghese, Joshua Wesley *(Christian Medical College & Hospital, Ludhiana)*; Philip Alexander, Josy Thomas, Pradeep Zechariah *(Lady Willingdon Hospital, Manali)*; Mariam anjum Ifthikar, Rohan Thomas Mathew, Rohan Shetty, M Vijayakumar Vijaykumar *(Yenepoya medical college hospital, Mangalore)*; BM Zeeshan Hameed, Sufyan Ibrahim, Gayathri Jyothish, Sunil Krishna, Badareesh L, Stanley Mathew, Arjun Suresh kumar *(Kasturba Medical College Hospital, Manipal, Manipal)*; Navneet Kumar Chaudhry, Narinder Singh, Dr piyush kumar Sinha, Rachith Sridhar *(Maharishi Markandeshwar Institute of Medical Sciences & Research, Mullana, Ambala, Haryana)*; Sunny Agarwal, Srikant Balasubramaniam, Lipika Baliarsing, Swati Chhatrapati, Charulata Deshpande, Satish Dharap, Ashni Dharia, Sarita Fernandes, Suraj Gandhi, Mangesh Gore, Abhilash Jayakumar, Rameshwar Mhamane, Anand Nirgude, Sandesh Parab, Manish Patil, Amit Peswani, Anjana Sahu, Sarika Samel, Fagun Shah *(BYL Nair Hospital, mumbai)*; Dipti Haridas, Amruta Kulkarni, Vijay Shetty *(Fortis Hospital Mulund, Mumbai)*; Bejoy Abraham, Varun Agarwal, Quazi Ahmad, Ashishkumar Asari, Mohammad Ismail Attar, Nagaraja Sekhar Ayyalasomayajula, Sutej Bachawat, Dr shivani Bachhav, Vivek Badhe, Sanjiv Badhwar, Shubhabrata Banerjee, Dr VIKAS Basa, Vipul Bothara, Somnath Chattopadhyay, Dr Sohin Chaudhari, Rahul Chavan, Priyank Chawathe, Shailja Dadhich, Anuj Dalal, Avinash Date, Mandar Deshpande, Preetham Dev, Niren Dongre, Anirudha Doshi, Maya Gade, Shreyash Gajjar, Mohan Gawande, Amol Ghalme, Dr bhavisha Ghugare, Ishita Gupta, Manoj Jain, Divakar Jain, Saumya Sekhar Jenasamant, Vaishali Joshi, Vinay Joshi, Neha Kalwadia, Nandkishore Kapadia, Hari Bipin Radhakrishnan Kattana, Tirathram Kaushik, Akshat Kayal, Shama Kovale, Yogesh Kulkarni, Abhaya Kumar, Kranthi Kumar, Kashmira Kumawat, Vidyadhar Lad, Namrata Maskara, Rajesh Mistry, Smruti Ranjan Mohanty, Kanchan Motwani, Manoj Mulchandani, Mandar Nadkarni, Sanjay Pandey, Dr. Mrunal Parab, Dinshaw Pardiwala, Amrita Patkar, Neha Pawar, Abhijit Pawar, Abhinav Pednekar, Vishal Peshattiwar, Harshwardhan Pokharkar, Ojas Potdar, Amit Pothare, Faizan Rahmani, Sunil Rajput, Nalla Ramji Narendra, Anuradha Rao, Suresh Rao, Himanshu Rohela, Rajendra Sakhrekar, Dhanshree Salunkhe, Gursev Sandlas, Hrishikesh Sarkar, Dr Afroz Satpathy, Raghuram Sekhar, Yashwant Shelke, Sanket sadanand Shetty, Dr Shweta Shetye, Anshumala Shukla kulkarni, Umang Singal, Faisal Solanki, Rajendra Sonawane, Raghavendraswami Thete, Yuvaraja Thyavihally, Raj Vhatkar, Sameer Vora, Santosh Waigankar, Shruti Wasnik, Mona Yadav, Rammohan Yedave *(Kokilaben Dhirubhai Ambani Hospital, Mumbai)*; Hriday Acharya, Anant Bangar, Manoj Bharucha, Parag Dhumane, Santosh Karmarkar, Rajesh Nathani, Archana Nehe, Abhay Nene, Webster Jerry Noronha, Naresh Palapalle, Priyank Patel, Dilroop Poyyil, Rajeev Redkar, Munjal Shah, Rahul Deo Sharma, Shruti Tewari *(Lilavati Hospital & Research Centre, Mumbai)*; Ganesh Bakshi, Vikram Chaudhari, Anuja Deshmukh, Ashwin Desoouza, Stuti Gupta, Deepa Nair, Prakash Nayak, Shraddha Patkar, C S Pramesh, Ajay Puri, Sajid Qureshi, Prakash Shetty, Ts Shylasree, Purvi Thakkar, Shivakumar Thiagarajan, Virendra Kumar Tiwari, Saiesh Reddy Voppuru *(Tata Memorial Hospital, Mumbai)*; Rajesh Soni, Anushri Soni, Gira Soni *(Soni Hospital, Nagpur)*; Junaid Alam, Dinesh Bagaria, Minu Bajpai, Akshay Kumar Bisoi, Arvind Chaturvedi, Sandeep Chauhan, Narendra Choudhary, Rajendra Singh Chouhan, Sunil Chumber, Surya Kumar Dube, Kamran Farooque, Vasubabu Gudala, Amit Gupta, Mohit Joshi, Apoorva Kabra, Shashank Sharad Kale, Abhinav Kumar, Subodh Kumar, Dhruv Mahajan, Rajesh Malhotra, P Ramesh Menon, Biplap Misra, Samarth Mittal, Rajinder Parshad, Pratyusha Priyadarshini, Sushma Sagar, Pradeep Brijkishor Sharma, Shilpa Sharma, Vijay Sharma, Vivek Trikha, Mayank Tyagi *(All India Institute of Medical Sciences, New Delhi)*; Kuldeep Bansal, Harvinder Singh, Kalyan Varma *(Indian Spinal Injuries Center, New Delhi)*; Lovenish Bains, Anurag Mishra, Rajdeep Singh *(Maulana Azad Medical College, New Delhi)*; Rohit Bhardwaj, Abhishek Mittal, Sabarirajan Ponnusamy, Gyan Ranjan Singh, Isha Tuli *(Safdarjung Hospital, New Delhi)*; Dr Pramod Bhor, Dr Sanjay Dhar, Rahul Ghodke, Sachin Kale *(Sanjay Clinic & Apollo Hospital, New Mumbai)*; Manisha Aggarwal, Himani Gupta, Gurleen Kaur, Ashwani Kumar *(Government Medical College, Patiala)*; Ajinkya Deshpande, Anup Gadekar, Tanmay Jaysingani, Taufiq Panjwani, Rakesh Patil, Vivek Sodhai, Narendra Vaidya, Dr sunil kumar Vishwakarma, Utkarsha Wayal *(Lokmanya Hospital for Special Surgery, Pune)*; Debnarayan Dutta, Suvendu Maji *(Bengal cancer foundation(BIMS Hospital), Purba Bardhaman)*; Rajnish Arora, Somprakas Basu, Mohit Dhingra, Pankaj Kumar Garg, Amit Gupta, Farhanul Huda, Pankaj Kandwal, Ravi Kant, Rajkumar Kottayasamy Seenivasagam, Navin Kumar, Shashank Kumar, Lokavarapu Manoj joshua, Radheyshyam Mittal, Dharma Ram Poonia, Deepak Rajput, Saravanan Sadhasivam, Dr Sudhir Singh, Vivek Singh *(All India Institute Of Medical Sciences, Rishikesh)*; Kshitija Chandanwale, Chandrashekhar Mahakalkar, Melissa Philip, Vaibhav Thorat *(Acharya Vinoba Bhave Rural Hospital, Sawangi (Meghe), Wardha)*; Yousuf Choudhury, Devishmita Das, Mautushi Das, Subhadra Goala, Ravi Kannan, Farhana yasmin Laskar, Parbin Laskar, Kapil Malik, Poulome Mukherjee, Dr.ABHISHEK Sarkar, S Thoibisana Singha, M Nongalei Singha, Damayanti Singha, Gowtham Srungavarapu, Ritesh Tapkire, Siempui Tling *(Cachar Cancer Hospital and Research Centre, Silchar)*; Syed Muzamil Ishaq Andrabi, Gowhar Aziz Bhat, Nisar Chowdri, Robindera Kour, Asif Mehraj, Fazl Parray, Raahil Shah, Dr ZAMIR AHMAD Shah, Rauf Wani *(Sher-i-Kashmir Institute of Medical Sciences, Srinagar)*; Soujanya Adamala, Ravikanth Gowder, Siddhi Hegde, Mashitha M S, Ranganath N, Shreya Sreeram *(KVG Medical College & Hospital, Sullia)*; Dr.sushil Ankadavar, Darshan Bafna, Dr.Bharat Dhanani, Dr. Nikhil Ingle, Piyush Jadhao, Sinu Joseph, Swapnil Kapote, Shirin Karkada, Vijay Kumar, Parag Lad, Rajan Lohia, Suyog Madje, Venkateshwaran Narasiman, Ashish Phadnis *(jupiter hospital, thane)*; Meer Chisthi, Gejoe George, I Yadev *(Government Medical College Thiruvananthapuram, Thiruvananthapuram)*; Harihara Jothi, Alfie Kavalakat, Dr Dilber Pareed *(Jubilee Mission Medical College & Research Institute, Thrissur)*; Godwin David .C. Mathew, Livingston Abel, Mansi Agrawal, Rabindranath B, Manish Baldia, Ravi Kishore Barla, Manisha Beck, Santosh Benjamin, Jeremy Bliss, Lisa Cherian, Sreekar Devarakonda, Geley Ete, Arun Jacob Philip George, Amish Gohil, Deeptiman James, Mark Ranjan Jesudason, Lallu Joseph, Treasa Joseph, Kathir Joyson, Gomathi Karnan, Albert Kota, Pushplata Kumari, Anitha Loganathan, Vasanth Mark Samuel, John Mathew, Rohin Mittal, Senthil K Nathan, Joby Elizabeth Ninan, Ajay Philip, R.Priyadarshini Priyadarshini, Suganya S, Habie Samuel, Gilbert Samuel, Daniel Selvaraj, Srujan Sharma, Suraj Surendran, Hariharan T D, Santhosh Kumar Thangaraj, Vinotha Thomas, Varghese Thomas, John K Thomas, Harish Y S *(Christian Medical College & Hospital, Vellore)*; Manobhiram Boggavarapu, Karthik Chandra Vallam, Dr.murali Krishna Voonna *(Mahatma Gandhi cancer hospital and research institute, Visakhapatnam)*. |
| **Indonesia**: Marilaeta Cindryani Lolobali, Christopher Ryalino, Tjokorda Gde Agung Senapathi, Mahadewa Tjokorda, i Made Gede Widnyana *(RSUP Sanglah, Denpasar)*; Dita Aditianingsih, Aino Auerkari, Susilo Chandra, Fachreza Aryo Damara, Mohammad Adya Firmansha Dilmy, Achmad Kemal Harzif, Sidharta Kusuma Manggala, Dedy Pratama, Affan Priyambodo, Andi Ade Ramlan, Ratna Farida Soenarto, Adhrie Sugiarto, Ilham Utama Surya, Tamara Tango, Aida Rosita Tantri, Raihanita Zahra *(Cipto Mangunkusumo National General Hospital & Universitas Indonesia, Jakarta)*; Dedy Fachrian, Andi Hasyim, Ade Susanti *(Raden Mattaher General Hospital, Jambi)*; Teddy Saputra, Erwin Syarifuddin *(RS Ibnu Sina YW-UMI, Makassar)*; Andi Asadul Islam, Gabriele Kembuan, Hendra Pajan *(RSUP Dr. Wahidin Sudirohusodo, Makassar)*; Ahmad Hannan Amrullah, Khoirul Anam, Thirza Hadipranata, Julius Albert Sugianto *(Ngimbang General Hospital, Ngimbang)*; Aidyl Fitrisyah, Mayang Indah Lestari, Nur Rachmat Lubis, Rizal Zainal, Zulkifli Zulkifli *(Dr Mohammad Hoesin General Hospital, Palembang)*; Erick Gamaliel Amba, Warren Lie, Andika adiputra Thehumury *(RSUD SAWERIGADING PALOPO, PALOPO)*; Tedy Apriawan, Yunus Kuntawi Aji, Roidah Taqiyya Zahra Wathoni *(dr. Soetomo General Academic Hospital, Surabaya)*; Sumadi Lukman Anwar, Teguh Aryandono, Wirsma Arif Harahap, Juni Kurniawaty, Djayanti Sari, Artanto Wahyono, Yunita Widyastuti, Akhmad Yun Jufan *(Central General Hospital dr. Sardjito, Yogyakarta)*. |
| **Iran, Islamic Rep.**: Parisa Arjmand, Mohammad Etezadpour, Babak Ganjeifar, Masoumeh Hosseinpoor, Hassan Mehrad-Majd, Mohsen Rajati *(Ghaem Teaching Hospital, Mashhad)*; Reza Assadi, Ehsan Noori, Parisa Rajaei *(Imam Reza hospital, Mashhad)*; Samira Hajisadeghi, Mohammad Mehdizadeh, Mina Soltani *(Fatemiyeh Oral and Maxillofacial Surgery Center, Qom)*; Narges Alizadeh, Gholamreza Azarnia Azar Nia, Hamid Heidari, Seyedmohamad Hosseini Zavareh Hosseini Zavareh, Ali Moazami Pour Moazami Pour, Farokh Savaddar, Mostafa Vahedian *(Forqani Hospital, Qom)*; Hoora Amouzegar, Gholamreza Azarnia Azar Nia, Mojdeh Bahadorzadeh, Seyedeh Homa Hemmasi, Ahmad Kachoie, Ali Karimi Karimi, Sepideh Miraj, Monireh Mirzaie, Hossein Mokarami Mokarami, Amrollah Salimi *(Imam Reza Hospital, Qom)*; Roghayyeh Ahangari, Ali Ahmadvand, Ali Bashiri, Morteza Borhani, Mohammad Haidari, Mohammad Taghi Imani Khosroshahi, Shahrokh Jahan Bini, Mohsen Koosha, Mohammad Kazem Moslemi, Ali Naghibi, Shahrzad Tehrani, Zahra Yazdi *(Izadi Hospital, Qom)*; Seyyed Hassan Adeli, Majid Alborzi, Hamed Bagheri, Mohsen Eshraghi, Hassan Fatemi manesh, Mohammad Ghomeisi, Seyed fakhreddin Hejazi, Ahmad Kachoie, Saeed Madani, Nima Najafian motahaver, Samieh Norouzi, Mahdi Pezeshki Modarres, Ali Shafiee, Jamshid Vafaeimanesh, Hossein Yusefi *(Shahid Beheshti Hospital, Qom)*; Ahmad Kachoie, Saeed Madani *(Shohada Hospital, Qom)*; Elahe Hosseini, Pourya Medhati, Hamed Nikoupour *(Abu-Ali Sina Hospital Shiraz, Shiraz)*; Kayvan Aghazadeh, Shahin Bastaninejad, Payman Dabirmoghaddam, Reza Erfanian, Mohammadreza Firouzifar, Farrokh Heidari, Shirin Irani, Ebrahim Karimi, Ali Kouhi, Masoud Motasaddi zarandy, Mahtab Rabbani Anari, Saleh Sandoughdaran, Saeed Sohrabpour, Ardavan Tajdini, Nasrin Yazdani *(Amir Alam Hospital, Tehran)*; Hamed Akhavizadegan, Esmaeil Rezghi Maleki, Naser Yousefzadeh Kandevani *(Baharloo hospital, Tehran)*; Nima Bagheri, Seyed Amir Javadi, Seyed Hadi Kalantar, Farzaneh Keneshlou, Zahid Hussain Khan, SM Javad Mortazavi *(Imam Khomeini Hospital Complex(IKHC), Tehran)*; Sayedali Ahmadi, Jaber Hatam, Mohammad Hossein Khosravi *(Rasool-e-Akram Hospital, Tehran)*; Mohammad Hossein Nabian, Leila Oryadi zanjani, Fardis Vosoughi *(Shariati Hospital, Tehran)*; Mohammadreza Golbakhsh, Seyyed Hossein Shafiei, Babak Siavashi *(Sina Hospital, Tehran)*. |
| **Iraq**: Ali Al-Isawi, Mohammed Al-Masood *(Al-hillah Teaching Hospital, Babil)*; Dania Al-Najjar *(AlKhayal Medical Centre, Baghdad)*; Yarub Gahtan, Sara Nabil *(Alkindy teaching hospital, Baghdad)*; Najat Abdul Hameed, Jumana Abdul Hameed, Abdullah Ahmed *(Baghdad Medical City, Baghdad)*; Ahmed Hilmi *(Ibn Sina, Baghdad)*; Ali Akadh, Rand Hussein *(Zafaraniyah General Hospital, Baghdad)*; Abbas Aljebur, Sadik Hassan, Haithem Hussein Ali *(Basra children speciality hospital, Basra)*; Fahad Al-Hasani, Mustafa Wameedh Ibrahim, Mubder Mohammed Saeed *(Basra Teaching Hospital, Basra)*; Haidar Muhssein *(University of Kufa college of medicine, Najaf)*. |
| **Ireland**: Success Akindoyin, Hilary Ikele, Catherine McNestry *(Mayo University Hospital, Castlebar)*; Stevie Barry, Nikhil Dewan, Bosom Ekwere, Murtaza Essajee, Ream Langhe, Calista Marshall, Darya Musa *(Cavan General Hospital, Cavan)*; Zeeshan Ahmad, Emmet Andrews, Bruno Chan Chin, Mark Corrigan, Amy Edwards Murphy, Christina Fleming, Niamh Foley, Padraig Gardiner, Daniel Hechtl, Michelle Hsiao, Mohd yasser Kayyal, Shane Killeen, Maria Lyons, Stephen O’Brien *(Cork University Hospital, Cork)*; Cathy Burke, Matt Hewitt, Marwa Mohamed, Syeda Farah Nazir, Mei Yee Ng *(Cork University Maternity Hospital, Cork)*; Nor Azlia Abdul Wahab, Cathy Monteith, Oladayo Oduola *(Our Lady of Lourdes Hospital Drogheda, Drogheda, Co louth)*; Lylas Aljohmani, Timothy Nugent *(Beacon Hospital, Dublin)*; Sami Abd Elwahab, Paula Corr, Lauren Crone, Niall Davis, Johnathon Harris, Arnold Hill, Mohsen Javadpour, David Kearney, Robert Anthony Keenan, Deirdre Nolan, James Ryan *(Beaumont Hospital, Dublin)*; Mark Philip Hehir, Brendan McDonnell, Carmen Regan *(Coombe Women and Infants University Hospital, Dublin)*; Michael Geary, Fergal Malone, Claire McCarthy, Clare O Connor, Donal B O’Connor *(Rotunda Hospital, Dublin)*; Jarlath Bolger, Cillian Clancy, Shane Considine, Stefanie M Croghan, Noel Donlon, Emma Donohoe, Caroline Herron, John Larkin, Thomas Hugh Lynch, Barry Maguire, Rustom Manecksha, Andrea Mc Carthy, Katharina Nagassima, Erica O’Sullivan, Pat Rohan, Ryan Roopnarinesingh, Salloum Salloum *(St James’s Hospital, Dublin)*; Thomas Aherne, Mary Barry, Gareth John Bowen, Ellen Boyle, Carolyn Cullinane, Joseph Dowdall, Ann Hanly, Ahmed Hassanin, Helen Heneghan, Conor Hurson, Orlaith Kelly, Rory Kennelly, Aoife Kiernan, Sean T Martin, Nawar Masarani, Damian McCartan, Enda W McDermott, Ben Murphy, Kin Cheung Ng, Nwabundo Njeze, Aine O’Neill, Ruth S Prichard, Ned Quirke, Ian Sean Reynolds, Des Winter *(St Vincent’s University Hospital, Dublin)*; Rowan Casey, Ben Creavin, Mutaz Elamin, Amy Gillis, Dara Kavanagh, Michael Kelly, Áine McNamee, Muheilan Muheilan, Paul Neary, Patrick Owens, Akshaya Ravi, Paul Ridgway *(Tallaght Hospital, Dublin)*; Paul Carroll, Chris Collins, Amenah Dhannoon, Helen Earley, Amy Fowler, Aisling Hogan, Aoife Lowery, Peter McAnena, Charlie Timon, Stewart Walsh *(University Hospital Galway, Galway)*; Wisam Al-Ramli, Imran Azeem, Muhammad Usaama Bahadoor, Zsolt Bodnar, Hassan Elmusharaf, Faisal Saeed Hassan, Adam Hingum, Huilun Huan, Seamus Jennings, Syed Mohammad Umar Kabir, Mohamed Hamed Khalid, Mariya Kuteva, Syed Nadeem Mujtaba, Shanell Peeriyah, Hina Rehman, Muhammad assam Sarwar, Tony Shaju, Ian Stephens, Michael Sugrue, Joseph Thomas, Manvydas Varzgalis, Saqib Zeeshan *(LETTERKENNY UNIVERSITY HOSPITAL, Letterkenny)*; Alisha Jaffer, Larne Jones-Whiting, Colin Peirce *(University Hospital Limerick, Limerick)*; Sean Johnston, Seantee Lim, Éanna Ryan *(Tullamore University Hospital, Tullamore)*; Alwaleed Abdelgadir, Sara Ahmed, Youssef Al-Mukhaizeem, Tara Connelly, Fiachra Cooke, Clare Crowley, Ivor Cullen, Michael Flanagan, Amy Fogarty, Orna Glynn, Claudia Guerrero Martinez, Mohamed Alfatih Hamza, Mekki Hassan, Ibrahim Hegazy, Rhodri Hill, Amr Kazim, Azriny Shaziela Khalid, Muhammad Abdullah Khalid, Zubair Majeed, Aidan Manning, Peter McCullough, Seamus Murphy, Peter Neary, Anthony Noone, Gerrard O’Donoghue, John O’Kelly, Eddie Odonnell, Elaf Osman, Jessica Ryan, Rafeh Saeed *(University Hospital Waterford/University College Cork, Waterford)*. |
| **Israel**: Ahmad Abo Arar, Arsan Abu abed, Daniel Dykman, Ahmad Elnassasra, Inbar Gatot, Haim Gavriel, Ruthie Gold- Deutch, Nadav Haim, Jonathan Hammerschlag, Yehuda Hershkovitz, Ahmad Jaber, Adi Kenoshi, Ron Lavy, Omar Majadla, Limor Muallem-Kalmovich, Hilli Nativ, Igor Rabin, Yael Sandler, Oded Zmora, Osnat Zmora *(SHAMIR MEDICAL CENTER, BE’ER YA’AKOV)*; Miklosh Bala, Yonatan Avraham Demma, Yuri Fishman, Gad Marom *(Hadassah Medical Center, Jerusalem)*; Naor Avni, Ofra Carmel, Amicur Farkas, Michael Ron Freund, Lior Gonen, Yaacov Gozal, Dmitry Greenman, Stanislav Kocherov, Nevo Margalit, Orit Nahtomi Shick, Israel Alexander Ostrovsky, Rivka Pardes, Michal Perets, Tal Shahar, Henry Shapiro, James Tankel, David Teren, Reuven Yahud, Shlomo Yellinek *(Shaare Zedek Medical Center, Jerusalem)*. |
| **Italy**: Paolo Balercia, Lisa Catarzi, Giuseppe Consorti *(University Hospital Umberto, Ancona)*; Pasquale Cianci, Domenico Gattulli, Marina Minafra, Enrico Restini *(Lorenzo Bonomo, andria)*; Enrico Andolfi, Filippo Annino, Frezza Barbara, Alessia Biancafarina, Edoardo Bussolin, Marco De Prizio, Ulpjana Gjondedaj, Marilena Gubbiotti, Gianni Mura, Giuseppe Antonino Pellicano’, Giacomo Maria Pirola, Rezart Sulce *(Ospedale San Donato USL Toscana Sud Est, Arezzo)*; Gennaro Martines, Vincenzo Papagni, Arcangelo Picciariello *(Azienda Ospedaliero Universitaria Consorziale Policlinico Di Bari, Bari)*; Stefano Magnone, Michele Pisano, Elia Poiasina *(Papa Giovanni XXIII Hospital, Bergamo)*; Laura Alberici, Filippo Antonacci, Alessandro Arena, Angela Belvedere, Fabio Bernagozzi, Paolo Bernante, Pietro Bertoglio, Lorenzo Bianchi, Maria Bisulli, Barbara Bonfanti, Safia Boussedra, Jury Brandolini, Crescenzo Cacciapuoti, Stefano Cardelli, Riccardo Casadei, Matteo Cescon, Alessandro Cipolli, Riccardo Cipriani, Luca Contu, Francesco Costa, Niccolo’ Daddi, Eugenia De Crescenzo, Pierandrea De Iaco, Alessandra De Palma, Massimo Del Gaudio, Anna Nunzia Della Gatta, Giampiero Dolci, Giulia Dondi, Matteo Droghetti, Sergio Nicola Forti Parri, Elena Garelli, Chiara Gelati, Giuliana Germinario, Federico A. Giorgini, Carlo Ingaldi, Elio Jovine, Kenji Kawamukai, Antonio Lanci Lanci, Raffaele Lombardi, Maria Elisa Lozano Miralles, Claudio Marchetti, Michele Masetti, Francesco Minni, Daniele Morezzi, Daniele Parlanti, Alice Pellegrini, Anna Myriam Perrone, Anna paola Pezzuto, Marco Pignatti, Gianluigi Pilu, Valentina Pinto, Gilberto Poggioli, Silvana Bernadetta Puglisi, Diego Raimondo, Matteo Ravaioli, Claudio Ricci, Sara Ricciardi, Francesco Ricotta, Roberta Rizzo, Angela Romano, Matteo Rottoli, Riccardo Schiavina, Renato Seracchioli, Matteo Serenari, Margherita Serra, Piergiorgio Solli, Gioia Sorbi, Mario Taffurelli, Marta Tanzanu, Achille Tarsitano, Marco Tesei, Gabriele Vago, Tommaso Violante, Simone Zanotti *(IRCCS Azienda Ospedaliero-Universitaria di Bologna, Bologna)*; Raffaele Aspide, Giacomo Bertolini, Carlo Bortolotti, Alessandro Carretta, Ambra Caruso, Alfredo Conti, Carla De Vita, Filippo Friso, Emanuele La Corte, Diego Mazzatenta, Alessandro Pirina, Vittoria Rosetti, Carmelo Sturiale, Matteo Vincenzi, Matteo Zoli *(IRCCS Istituto delle Scienze Neurologiche di Bologna, Bologna)*; Francesco Castagnini, Davide Maria Donati, Tommaso Frisoni, Stefano Lucchini, Emanuela Palmerini, Francesco Traina *(IRCCS Istituto Ortopedico Rizzoli, Bologna)*; Anna Maria Baietti, Bruno Berselli, Silvia Bolognesi, Erich Fabbri, Francesco Farnia, Pietro Maremonti, Concetta Marganella, Ernesto Pasquini, Vito Antonio Piserchia, Gian Marco Prucher, Alessandra Razzaboni, Silvia Ricci, Giacomo Sollini, Caterina Testoni *(Ospedale Maggiore/Bellaria Carlo Alberto Pizzardi AUSL Bologna, Bologna)*; Mohammed Abu Hilal, Nine de Graaf, Roberta La Mendola *(Fondazione Poliambulanza, brescia)*; Giulia Arrigoni, Gian Luca Baiocchi, Elena Cagnazzi, Rossella D’Alessio, Francesco Doglietto, Federico Ferrari, Marco Fontanella, Batog Igor, Sarah Molfino, Franco Odicino, Pier Paolo Panciani, Giorgio Saraceno, Enrico Sartori, Luca Zanin *(ASST Spedali Civili, Ospedale di Brescia, Brescia)*; Giuseppe Esposito, Federica Frongia, Adolfo Pisanu, Mauro Podda *(Cagliari University Hospital, Cagliari)*; Nicola Cillara, Alessandro Cannavera Putzu, Raffaele Sechi *(Santissima Trinità - ATS Sardegna, Cagliari)*; Emmanuele Abate, Massimiliano Casati, Letizia Laface, Marcello Schiavo *(Ospedale Vittorio Emanuele III - Carate Brianza, Carate Brianza (MB))*; Fabio Marino, Fabrizio Perrone *(IRCCS ‘Saverio de Bellis’, Castellana Grotte (Ba))*; Paolo Annicchiarico, Alessandro Cappellani, Matteo Cavallo, Alessia Giaquinta, Rossella Gioco, Massimiliano Veroux, Pierfrancesco Veroux, Antonino Zanghì *(Azienda Ospedaliero- Universitaria Policlinico San Marco, Catania)*; Antonio Cianci, Arturo Lo Giudice, Maria Grazia Matarazzo, Giorgio Ivan Russo, Giuseppe Sarpietro, Carmen Emanuela Scandura *(Policlinic Hospital ‘G. Rodolico’, Catania)*; Ida Barca, Adriano Carnevali, Antonio Carpino, Maria Giulia Cristofaro, Gilda De Paola, Giuseppe Giannaccare, Daniela Novembre, Giuseppe Sammarco, Vincenzo Scorcia, Angeli Christy Yu *(University ‘Magna Graecia’ of Catanzaro, Catanzaro)*; Marcello D’Andrea, Lorenzo Mongardi, Luigino Tosatto *(Ospedale M. Bufalini, Cesena)*; Mirko Barone, Felice Mucilli, Angelo Muraglia *(Policlinico Santissima Annunziata, Chieti)*; Ottavia Caserini, Domenico Benvenuto Giuliani, Marco Monti, Alessia Morello, Edoardo Segalini *(ASST Crema, Crema)*; Felice Borghi, Desiree Cianflocca, Alberto Daniele, Danilo Donati, Enrico Gelarda, Giorgio Giraudo, Maria Carmela Giuffrida, Alessandra Marano, Elena Olearo, Vincenzo Pruiti Ciarello, Andrea Puppo, Valentina Testa *(Santa Croce e Carle Hospital, Cuneo, Cuneo)*; Marco Giacometti, Sandro Zonta *(San Biagio Hospital, Domodossola - VB, Domodossola)*; Eleonora Monti, Andrea Porta, Daniele Sambucci *(Ospedale Sacra Famiglia Fatebenefratelli, Erba)*; Arianna Birindelli, Barbara Carrara, Bruno Compagnoni, Roberto Del Giudice, Sara Elisabetta Dester, Daniele Lomiento, Silvia Ruggiero, Lucio Taglietti, Fabio Viotti *(ASST Valcamonica Ospedale di Esine, Esine)*; Domenico Lacavalla, Savino Occhionorelli, Michele Rubbini *(Azienda Ospedaliero Universitaria San’Anna, Ferrara)*; Massimiliano Bernabei, Nicolò Fabbri, Marta Fazzin, Carlo V. Feo, Marco Torchiaro *(Azienda Unità Sanitaria Locale di Ferrara, Ferrara)*; Renato Costi, Edoardo Virgilio *(di Vaio Hospital, Fidenza)*; Lorenzo Arlia, Giuseppe Barbato, Ilenia Bartolini, Andrea Bottari, Chiara Bruno, Alessandro Bruscino, Carlotta Checcucci, Fabio Cianchi, Francesco Coratti, Rosita De Vincenti, Annamaria Di Bella, Massimiliano Fambrini, Laura Fortuna, Oreste Gallo, Giacomo Gigliucci, Luca Giovanni Locatello, Gherardo Maltinti, Jacopo Martellucci, Paolo Prosperi, Maria Novella Ringressi, Flavia Sorbi, Fabio Staderini, Antonio Taddei *(Azienda Ospedaliera Universitaria Careggi, Firenze)*; Alessandro Anastasi, Giuseppe Canonico, Emiliano Chisci, Linda Gabellini, Fabrizio Masciello, Stefano Michelagnoli, Tommaso Nelli, Luca Tirloni *(Ospedale San Giovanni di Dio, Firenze)*; Giovanni Alemanno, Armando Arminio, Renata Beck, Carlo Bergamini, Antonella Cotoia, Vincenzo Lizzi, Giuseppe Maccagnano, Vito Pesce, Antonio Luciano Sarni, Nicola Tartaglia, Fernanda Vovola *(Ospedali Riuniti Azienda Ospedaliera Universitaria, Foggia)*; Andrea Avanzolini, Antonio Bocchino, Raffaele Bova, Davide Cavaliere, Fabrizio D’acapito, Giorgio Ercolani, Francesca Fappiano, Carlo Alberto Pacilio, Leonardo Solaini *(Morgagni-Pierantoni, Forlì)*; Giancarlo D’Andrea, Laura Lavalle, Veronica Picotti *(Fabrizio Spaziani, Frosinone 03100)*; Andrea Barberis, Marco Filauro, Matteo Santoliquido *(E.O. Ospedali Galliera, Genoa)*; Alessandra Aprile, Fabio Barra, Raffaele De Rosa, Raquel Diaz, Simone Ferrero, Piero Fregatti, Claudio Gustavino, Michele Iester, Chiara Kratochwila, Andrea Massobrio, Davide Pertile, Stefano Scabini, Umberto Scovazzi, Domenico Soriero, Marco Sparavigna, Carlo Traverso, Aldo Vagge *(IRCCS Ospedale Policlinico San Martino, Genoa)*; Denise Gambardella, Manfredo Tedesco *(Giovanni Paolo II Hospital, Lamezia Terme)*; Stefano D’Ugo, Norma Depalma, Marcello Giuseppe Spampinato *(P.O.’Vito Fazzi’, Lecce)*; Angelo Airoldi, Ariberto Brivio, Marco Chiarelli, Ludovica Gibelli, Bonfanti Giulia, Samuele Grandi, Giovanni Pesenti, Fulvio Tagliabue, Mauro Zago *(ASST di Lecco - P.O di Lecco, Lecco)*; Riccardo Lenzi, Jacopo Matteucci, Luca Muscatello *(Ospedale Apuane, Massa)*; Gaia Colletti, Marco Guido Confalonieri, Andrea Costanzi, Colomba Frattaruolo, Andrea Locatelli, Michela Monteleone *(San Leopoldo Mandic, Merate (LC))*; Giorgio Badessi, Maria Caffo, Gerardo Caruso, Eugenio Cucinotta, Antonino Francesco Germano’, Carmelo Mazzeo *(Policlinico Universitario G. Martino of Messina, Messina)*; Camillo Leonardo Bertoglio, Paolo De Martini, Giovanni Ferrari, Alessandro Giani, Pietro Maria Lombardi, Michele Mazzola *(ASST Grande Ospedale Metropolitano Niguarda, Milan)*; Ludovica Baldari, Daniele Bissacco, Luigi Boni, Elisa Cassinotti, Maurizio Domanin, Lorenzo Pignataro, Sara Torretta, Santi Trimarchi *(Fondazione IRCCS Ca’ Granda - ospedale Maggiore Policlinico, Milan)*; Daniele Armellin, Silvia Basato, Laura Bernardi, Francesca Bunino, Giovanni Capretti, Francesco Maria Carrano, Carlo Castoro, Giovanni Colombo, Andrea Costantino, Francesca De Lucia, Armando De Virgilio, Matteo Di Bari, Fabio Ferreli, Francesca Gaino, Marco Gramellini, Carlotta La Raja, Luca Malvezzi, Salvatore Marano, Giuseppe Mercante, Flavio Milana, Gennaro Nappo, Georgios Peros, Francesca Pirola, Vanessa Rossi, Elena Russo, Giuseppe Spriano, Sara Tamburello, Alessandro Zerbi *(Humanitas Research Hospital, Milan)*; Alberto Aiolfi, Davide Bona, Andrea Sozzi *(Istituto Clinico Sant’Ambrogio, Milan)*; Laura Adamoli, Mohssen Ansarin, Luca Bertolaccini, Sabine Cenciarelli, Francesco Chu, Rita De Berardinis, Uberto Fumagalli Romario, Giacomo Pietrobon, Giulia Sedda, Lorenzo Spaggiari, Marta Tagliabue *(Istituto Europeo di Oncologia - IRCCS -Milano, Milan)*; Antonella Ardito, Maria Caputo, Paola Cellerino, Valentina D’alessandro, Elisa Galfrascoli, Maria Paola Giusti, Marco Lotti, Roberto Santambrogio, Marco Antonio Zappa *(Ospedale Fatebenefratelli e Oftalmico, Milan)*; Andrea Bondurri, Alessandro Michele Bonomi, Francesco Colombo, Michele Achille Crespi, Piergiorgio Danelli, Angelo Gabriele Epifani, Luca Ferrario, Alice Frontali, Claudio Guerci, Anna Maffioli *(Ospedale Luigi Sacco Milano, Milan)*; Francesco Ferrara *(San Carlo Borromeo, Milan)*; Luca Antonio Aldrighetti, Domenico Baccellieri, Luca Bertoglio, Giulia Bonavina, Massimo Candiani, Giorgio Candotti, Arianna Casiraghi, Laura Mariangela Castellano, Paolo Ivo Cavoretto, Roberto Chiesa, Federica Cipriani, Paola De Nardi, Guido Fiorentini, Filippo Gagliardi, Alessandro Galdini, Alessandro Grandi, Elena Marotta, Simonetta Massaron, Andrea Melloni, Pietro Mortini, Gianluca Nocera, Martina Piloni, Mirko Pozzoni, Francesca Ratti, Riccardo Rosati, Alessandro Ferdinando Ruffolo, Pierpaolo Sileri, Alfio Spina, Andrea Vignali *(San Raffaele Scientific Institute, Milan, Milan)*; Cristina Barberio, Luigi Beretta, Francesca Cavenago, Nora Di Tomasso, Stefano Fresilli, Giovanni Landoni, Stefano Lazzari, Gaetano Lombardi, Marilena Marmiere, Fabrizio Monaco, Gabriele Todaro, Stefano Turi, Alberto Zangrillo *(Università Vita-Salute San Raffaele, Milan)*; Francesca Bertolina, Giorgio Bogani, Stefano Bonomi, Pierfrancesco Cadenelli, Valentina Chiappa, Stefano Piero Bernardo Cioffi, Davide Citterio, Lara Valentina Comini, Umberto Cortinovis, Maurizio Cosimelli, Antonino Ditto, Marco Fiore, Massimiliano Gennaro, Lorenzo Giannini, Alessandro Gronchi, Marcello Guaglio, Marco Guzzo, Andrea Leva, Alberto Macchi, Elena Manzo, Fabio Martinelli, Ilaria Mattavelli, Vincenzo Maria Mazzaferro, Francesco Raspagliesi, Luigi Rolli, Laura Sala, Roberto Salvioni, Mario Santinami, Silvia Segattini, Luca Sorrentino, Carlotta Zaborra *(Fondazione IRCCS Istituto Nazionale dei Tumori, Milano, Milano)*; Alexandre Anesi, Gianmaria Casoni Pattacini, Mattia Di Bartolomeo, Francesca Pecchini, Arrigo Pellacani, Micaela Piccoli *(Azienda Ospedaliero Universitaria di Modena, Modena)*; Federico Fusini, Andrea Gattolin, Marco Migliore, Roberto Rimonda, Diego Sasia, Elisabetta Travaglio *(Regina Montis Regalis Hospital, Mondovì, Mondovì)*; Federica Brunetti, Marco Cereda, Marco Ceresoli, Luca Cigagna, Cristina Dell’Oro, Alessandro Fogliati, Robert Fruscio, Tommaso Grassi, Maini Marzia Isabella, Luca Carlo Nespoli, Massimo Oldani, Sara Ornaghi, Nicolò Tamini *(Ospedale San Gerardo, Monza)*; Carmine Antropoli, Antonio Castaldi, Alessio Palumbo *(Azienda Ospedaliera di rilievo nazionale Antonio Cardarelli, Naples)*; Gaia Altieri, Umberto Bracale, Francesco Corcione, Marcello De Luca, Giovanni Domenico De Palma, Maria Michela Di NUZZO, Ruggero Lionetti, Dalila Loredana Lo Bue, Gaetano Luglio, Gianluca Pagano, Roberto Peltrini, Nello Pirozzi, Francesca Paola Tropeano *(Federico II University of Naples, Naples)*; Alessia Aversano, Andrea Belli, Maria D’amico, Paolo Delrio, Francesco Izzo, Renato Patrone, Daniela Rega *(Istituto Nazionale Tumori Fondazione, Pascale IRCCS, Naples)*; Pietro Maida, Ester Marra, Gianpaolo Marte, Andrea Tufo *(ospedale del mare, Naples)*; Francesco Bianco, Antonio Cappiello, Simona Gili, Paola Incollingo, Alessandra Novi *(Ospedale S. Leonardo - ASL Napoli 3 sud, Castellammare di Stabia, Naples)*; Giulia Bagaglini, Claudio Iovino, Francesco Menegon Tasselli, Maria Paola Menna, Francesco maria Romano, Settimio Rossi, Guido Sciaudone, Francesco Selvaggi, Lucio Selvaggi, Francesca Simonelli *(Universitá della Campania ‘Luigi Vanvitelli’, Naples, Naples)*; Guido Coretti, Mario Pannullo, Adolfo Renzi *(Ospedale Fatebenefratelli, Napoli)*; Francesca Ascari, Giuliano Barugola, Giacomo Ruffo *(IRCCS Ospedale Sacro Cuore Don Calabria, Negrar di Valpolicella (Verona))*; Paolo Baroffio, Paolo Bellora, Laura Enrica Benedetti, Cristina Cerri, Giordana D’Aloisio, Maurizio Ferrari, Elisa Francone, Sergio Gentilli, Herald Nikaj *(Azienda Ospedaliero Universitaria Maggiore della Carità, Novara)*; Antonella Chessa, Alessandro Fiorini *(San Giovanni di Dio, Orbetello)*; Luca Campagnaro, Franco Chioffi, Pietro Ciccarino, Roberto Colasanti, Francesco de Falco, Fotios Kalfas, Gioacchino Mattisi, Giulia Nezi, Matteo Palma, Angelica Rizzoli, Domenico Rossi, Davide Russo, Renato Salvador, Francesco Volpin *(Azienda Ospedaliera di Padova, Padova)*; Guido Bissolotti, Stefano Fusetti, Francesco Lemma *(University of Padova, Padova)*; Vito Chiantera, Mariano Catello Di Donna, Giulio Sozzi *(ARNAS Civico Hospital, Palermo)*; Emanuele Cammarata, Sofia Campanella, Daniela Canzonieri, Adriana Cordova, Federico De Michele, Ettore Dinoto, Mara Franza, Leo Licari, Daniele Matta, Domenico Mirabella, Felice Pecoraro, Roberto Pirrello, Pierfrancesco Pugliese, Fernando Rosatti, Giuseppe Salamone, Francesca Toia, Massimiliano Tripoli *(Department of Surgical, Oncological and Oral Sciences. University of Palermo, Palermo)*; Cosimo Callari, Dario Di Miceli, Leo Licari *(FBF Buccheri La Ferla Palermo, Palermo)*; Alfredo Annicchiarico, Luca Bellanti, Michela Bergonzani, Roberto Berretta, Elisa Cabrini, Vito Andrea Capozzi, Fausto Catena, Federico Cozzani, Paolo Del Rio, Marco Domenichini, Anna Fornasari, Antonio Freyrie, Tiziana Frusca, Mario Giuffrida, Gennaro Perrone, Giulia Rossi, Matteo Rossini, Andrea Varazzani *(Azienda Ospedaliero - Universitaria di Parma, Parma)*; Vittorio Arici, Antonio Bozzani, Lorenzo Cobianchi, Matteo Filardo, Marika Sharmayne Milani, Franco Ragni *(Policlinico San Matteo, Pavia)*; Andrea de Manzoni Garberini *(Ospedale Civile Spirito Santo, Pescara)*; Edoardo Baldini, Diana Carpaneto, Michele Cauteruccio, Corrado Ciatti, Luigi Conti, Serena Gattoni, Pietro Maniscalco, Gerardo Palmieri, Calogero Puma Pagliarello *(G. Da Saliceto, Piacenza)*; Giuseppe Caristo, Raffaele Galleano, Michele Malerba *(Ospedale Santa Corona, Pietra Ligure (SV), Pietra Ligure)*; Marcello Calabrò, Francesca Farnesi, Elia Giuseppe Lunghi, Andrea Muratore, Nicoletta Sveva Pipitone federico *(Edoardo Agnelli, Pinerolo)*; Joel Reuben Abel, Lorenzo Andreani, D’arienzo Antonio, Vittorio Aprile, Riccardo Balestri, Giacomo Benettini, Stefano Berrettini, Luca Bruschini, Massimo Chiarugi, Federico Coccolini, Simone Colangeli, Camilla Cremonini, Lodovica Cristofani Mencacci, Iacopo Dallan, Silvia De Santi, Gregorio Di Franco, Lorena Di Girolami, Giacomo Fiacchini, Niccolò Furbetta, Stylianos Korasidis, Marco Lucchi, Andrea Morandi, Luca Morelli, Serena Musetti, Carlo Maria Neri, Matteo Palmeri, Miriana Picariello, Francesco Porcelli, Marco Puccini, Nicolo’ Roffi, Erica Statuti, Dario Tartaglia, Alberto Tonelli, Matteo Vianini *(Azienda Ospedaliero Universitaria Pisana, Pisa)*; Gianluca Baronio, Mauro Montuori, Enrico Pinotti *(Policlinico San Pietro, Ponte San Pietro)*; Stefano Maria Massimiliano Basso, Federica Maffeis, Paolo Ubiali *(Azienda Per L’assistenza Sanitaria N. 5 Friuli Occidentale, Pordenone)*; Lorenzo Aguzzoli, Saverio Coiro, Giuseppe Falco, Vincenzo Dario Mandato, Valentina Mastrofilippo, Simone Mele *(Azienda Unità Sanitaria Locale - IRCCS di Reggio Emilia, Reggio Emilia)*; Caterina Baldi, Carlo Corbellini, Gianluca Matteo Sampietro *(Ospedale Di Rho - ASST Rhodense, Rho)*; Massimo Dugo, Mauro Garino, Chiara Marafante, Antonio Masciandaro, Elisabetta Moggia, Alessandra Murgese *(Ospedale degli Infermi di Rivoli, Rivoli)*; Felice Eugenio Agro’, Gabriella Teresa Capolupo, Filippo Carannante, Marco Caricato, Vincenzo Denaro, Erica Mazzotta, Rocco Papalia, Giuseppe Pascarella, Alessandro Strumia, Biagio Zampogna *(policlinico universitario campus bio medico of rome, rome)*; Matteo Cinquepalmi, Marco Colasanti, Celeste Del Basso, Federica Falaschi, Nicola Guglielmo, Roberto Luca Meniconi, Alessandra Pecoraro, Sofia Usai *(Azienda Ospedaliera San Camillo - Forlanini, Rome)*; Giacomo Crescentini, Antonella Larcinese, Emanuele Picone, Giovanni Sinibaldi *(Fatebenefratelli Isola Tiberina, Rome)*; Annamaria Agnes, Salvatore Agnes, Sergio Alfieri, Francesco Belia, Valentina Bianchi, Giuseppe Bianco, Alberto Biondi, Paola Campennì, Valerio Cozza, Sabatino D’Archi, Domenico D’Ugo, Veronica De Simone, Marta Di Grezia, Sofia Di Lorenzo, Federica Ferracci, Valeria Fico, Gianluca Franceschini, Pietro Fransvea, Giulio Gasparini, Luca Gordini, Antonio La Greca, Francesco Litta, Celestino Pio Lombardi, Angelo Alessandro Marra, Angelo Parello, Marco Maria Pascale, Romeo Patini, Gilda Pepe, Roberto Persiani, Caterina Puccioni, Carlo Ratto, Fausto Rosa, Gianmarco Saponaro, Lorenzo Scardina, Tedesco Silvia, Giuseppe Tropeano *(Fondazione Policlinico Universitario Agostino Gemelli, Rome)*; Maria Benevolo, Daniele Bugada, Flaminia Campo, Maria Gabriella Dona’, Valentina Manciocco, Paolo Marchesi, Riccardo Mastroianni, Francesco Mazzola, Silvia Moretto, Raul Pellini, Gerardo Petruzzi, Barbara Pichi, Giuseppe Simone, Gabriele Tuderti, Jacopo Zocchi *(IRCCS ‘Regina Elena’ National Cancer Institute, Rome)*; Luigi Marino Cosentino, Andrea Sagnotta *(Ospedale San Filippo Neri, Rome)*; Roberta Angelico, Vittoria Bellato, Michela Campanelli, Marzia Franceschilli, Michele Grande, Giorgio Lisi, Tommaso Maria Manzia, Lorenzo Petagna, Bruno Sensi, Giuseppe Sica, Leandro Siragusa, Giuseppe Tisone *(Policlinico Tor Vergata Hospital, Rome, Rome)*; Marco Assenza, Barbara Binda, Massimo Biondi, Gioia Brachini, Placido Bruzzaniti, Mauro Casagrande, Flavia Ciccarone, Pierfranco Maria Cicerchia, Bruno Cirillo, Daniele Crocetti, Giancarlo D’ambrosio, Vito D’andrea, Francesca De Felice, Giorgio De Toma, Carlo Della Rocca, Giulia Duranti, Pietro Familiari, Enrico Fiori, Giovanni Battista Fonsi, Alessandro Frati, Stefania La Rocca, Filippo La Torre, Pierfrancesco Lapolla, Giovanni Marruzzo, Simona Meneghini, Andrea Mingoli, Francesco Pata, Andrea Picchetto, Antonella Polimeni, Diego Ribuffo, Maurizio Salvati, Antonio Santoro, Paolo Sapienza, Luigi Simonelli, Valentino Valentini, Martina Zambon, Giuseppa Zancana, Emma Zuppi *(Policlinico Umberto I, Rome)*; Simone D’Annunzio, Cosimo De Nunzio, Silvia Fiorelli, Mohsen Ibrahim, Chiara Loffredo, Domenico Massullo, Cecilia Menna, Rocco Monica, Massimiliano Pelli, Erino Angelo Rendina, Leonardo Teodonio, Andrea Tubaro *(Sant’Andrea Hospital, Sapienza University of Rome, Rome)*; Giulio Argenio *(AOU Ruggi, Salerno)*; Giulio Accarino, Accarino Giancarlo, Antonio Nicola Giordano *(San Giovanni di Dio e Ruggi d’Aragona, Salerno)*; Luca Cardinali, Elisa Sebastiani, Grazia Travaglini *(Madonna del Soccorso Hospital, San Benedetto del Tronto)*; Erika Andreatta, Emanuele Luigi Giuseppe Asti, Daniele Bernardi, Luigi Bonavina, Caterina Froiio, Andrea Lovece *(IRCCS Policlinico San Donato, San Donato)*; Chiara Copelli, Alfonso Manfuso *(IRCCS Casa Sollievo della Sofferenza, San Giovanni Rotondo)*; Pasquale Di Maio, Marco Giudice, Oreste Iocca *(Civic Hospital of Sanremo, Sanremo)*; Rosario Cennamo, Tommaso Cornali, Francesco Di Marzo *(Valtiberina, Sansepolcro)*; Cristian Altana, Francesco Bussu, Giampiero Capobianco, Anna Giacomina Carta, Sandro Ciccarello, Maria Laura Cossu, Pietrina Cottu, Giacomo De Riu, Salvatore Dessole, Francesco Dessole, Salvatora Dettori, Carlo Doria, Alessandro Fancellu, Claudio F Feo, Giorgio Carlo Ginesu, Giuliana Giuliani, Marco Giuseppe Iannuccelli, Massimo Madonia, Roberto Mancino, Olindo Massarelli, Gianfranco Meloni, Fabio Milia, Andrea Mulliri, Teresa Perra, Marco Petrillo, Antonio Piras, Franco Piredda, Francesco Pisanu, Alberto Porcu, Davide Rizzo, Angelino Sanna, Antonio Mario Scanu, Fabrizio Scognamillo, Damiano Soma, Anna Rita Tanca, Alessandro Tedde, Matteo Tedde, Luigi Angelo Vaira *(Cliniche San Pietro, A.O.U. Sassari, Sassari)*; Andrea Bartalini Cinughi de Pazzi, Osvaldo Carpineto Samorani, Daniele Fusario, Luigi Marano, Fabio Marino, Gaia Oldrà, Anna Lisa Pesce, Stefania Angela Piccioni, Luca Resca, Vincenzo Ricchiuti, Franco Roviello, Vinno Savelli *(Azienda Ospedaliero Universitaria Senese, Siena)*; Alberto Abrate, Pierpaolo Bordoni, Lorenzo Bosio, Guglielmo Clarizia, Francesco Fleres, Marco Franzini, Antonio Fratto, Pierluigi Giumelli, Alessandro Grechi, Alessandro Longhini, Fabrizio Lorusso, Elisa Scarnecchia, Federica Scolari, Alessandro Spolini *(Ospedale di Sondrio (ASST Valtellina e Alto Lario), Sondrio)*; Vincenzo Maiuri, Matteo Papandrea, Arturo Roncone *(Soverato Civil Hospital, Soverato)*; Lorenzo Conti, Andrea Rizzi, Marco Rovagnati *(Galmarini Hospital, Tradate, Lombardy)*; Alberto Brolese, Tommaso Cai, Francesco Antonio Ciarleglio, Francesca Dalprà, Gianni Malossini, Liliana Mereu, Irene Tamanini, Saverio Tateo, Giovanni Viel *(Santa Chiara Hospital, Trento)*; Enrico Battistella, Paolo Boscolo Rizzo, Cristoforo Fabbris, Marco Massani, Giacomo Spinato, Roberta Tutino *(Ca’ Foncello, Treviso)*; Ugo Grossi, Alessandro Iacomino, Simone Novello, Maurizio Romano, Serena Rossi, Giulio Santoro, Giacomo Zanus *(Ospedale Ca’ Foncello - Università di Padova (DISCOG), Treviso)*; Sokol Trungu *(Cardinale G Panico Hospital, Tricase)*; Giada Aizza, Gabriele Bellio, Selene Bogoni, Marina Bortul, Biagio Casagranda, Sara Cortinovis, Nicolò de Manzini, Davide Drigo, Paola Germani, Manuela Mastronardi, Lucia Paiano, Silvia Palmisano *(Cattinara University Hospital, Trieste)*; Pier Luigi Filosso, Francesco Guerrera, Matteo Marro, Mauro Rinaldi, Enrico Ruffini, Stefano Salizzoni *(Città della Salute e della Scienza, Turin)*; Laura Bardelli, Mattia Berselli, Giacomo Borroni, Eugenio Cocozza, Matteo Desio, Salomone Di Saverio, Bottazzoli Elisa, Giuseppe Ietto, Valentina Iori, Domenico Iovino, Lorenzo Livraghi, Valentina Marchionini, Stefano Megna, Emma Amal Nahal, Mara Palumbo, Valeria Quintodei, Alessandra Zullo *(University of Insubria, Ospedale di Circolo e Fondazione Macchi (Varese), Varese Lombardy)*; Lucrezia D’Alimonte, Giovanni Pirozzolo, Chiara Vignotto *(Dell’Angelo Hospital, Venezia)*; Tommaso Campagnaro, Andrea Caravati, Simone Conci, Carlotta De Cristofaro, Gabriele Gecchele, Tommaso Giuliani, Jacopo Graziosi, Alfredo Guglielmi, Salvatore Paiella, Corrado Pedrazzani, Tommaso Pollini, Simone Rattizzato, Andrea Ruzzenente, Roberto Salvia, Giulia Turri *(Azienda Ospedaliera Universitaria Integrata di Verona, Verona)*; Matilde Bacchion, Giovanni Butturini, Andrea Casaril, Alessandro Giardino, Harmony Impellizzeri, Marco Inama, Frigerio Isabella, Gianluigi Moretto *(Ospedale Pederzoli, Verona)*; Marco De Zuanni, Enrica Deiana, Mario Guglielmo *(Istituto Oncologico del Mediterraneo, Viagrande Catania)*; Alessandro Broglia, Claudia Casarini, Caterina Costanza Zingaretti *(Ospedale Civile di Voghera, Voghera)*; Marta Bonaldi, Giovanni Cesana, Francesco Mastriale, Stefano Olmi, Matteo Uccelli *(San Marco Hospital GSD, Zingonia (BG))*. |
| **Japan**: Yasuyuki Fukami, Takuya Saito, Tsuyoshi Sano *(Aichi Medical University, Aichi)*; Naoki Hirai, Kazuyoshi Hirota, Tetsuya Kushikata, Tasuku Oyama, Junichi Saito *(Hirosaki University Hospital, Aomori)*; Katsuhiko Ishibashi, Mizue Kamiyama, Kyongsuk Son, Kentaroh Tarao, Takayuki Yamada *(Chiba University Hospital, Chiba)*; Toshiya Shiga *(International University of Health and Welfare Ichilawa Hospital, Chiba)*; Chisaki Aze, Yoshihiko Deguchi, Hirotoshi Hasegawa, Tatsuki Hoshino, Yasushi Innami, Hiroyuki Inoue, Shingo Ito, Takeshi Nomura, Tomomi Ogihara, Reina Okada, Takashi Ouchi, Yuri Sekiya, Keikoku Tachibana, Emi Takano, Masae Yamamoto *(Tokyo Dental College, Ichikawa General Hospital, Chiba)*; Shuko Matsuda, Yuka Matsuki, Kenji Shigemi *(University of Fukui, Fukui)*; Teruyuki Hiraki, Yui Inoue, Shosaburo Jotaki *(Kurume University Hospital, Fukuoka)*; Tatsuro Abe, Masatoshi Eto, Junichi Inokuchi, Eiji Kashiwagi, Fumio Kinoshita, Satoshi Kobayashi, Ken Lee, Takashi Matsumoto, Keisuke Monji, Hidekazu Naganuma, Masaki Shiota, Ario Takeuchi *(Kyushu University, Fukuoka)*; Shinju Obara, Saori Takatsuki, Saori Tanaka *(Fukushima Medical University Hospital, Fukushima)*; Koji Iida, Kota Kagawa, Shuichiro Neshige *(Hiroshima University Hospital, Hiroshima)*; Tomohiro Chaki, Naoyuki Hirata, Satoshi Kazuma, Motonobu Kimizuka, Sho Kumita, Noriaki Nishihara, Sato Satoshi, Atsushi Sawada, Shunsuke Tachibana, Michiaki Yamakage *(Sapporo Medical University Hospital, Hokkaido)*; Yuta Nakamura, Kozo Sato *(Shonan Kamakura General Hospital, Kanagawa)*; Tomoya Irie, Tomoko Irisawa, Eiki Kanemaru, Yuko Koga *(Yokohama city university hospital, Kanagawa)*; Yoshihiko Chiba, Jun Makino, Shinnosuke Ohama, Shinichiro Okada, Kano Teruaki *(Yokosuka General Hospital Uwamachi, Kanagawa)*; Tatsuya Kida, Tomohide Takei *(Yokosuka Kyosai Hospital, Kanagawa)*; Kazuhiro Hanazaki, Hiroyuki Kitagawa, Tsutomu Namikawa *(Kochi Medical School Hospital, Kochi)*; Toshiyuki Mizota, Chikashi Takeda, Shintaro Yagi *(Kyoto University Hospital, Kyoto)*; Hiroshi Imai, Makoto Ishitobi, Manabu Kato, Kouhei Nishikawa, Takeshi Sasaki, Hiroshi Yonekura *(Mie University Hospital, Mie)*; Akihiro Kanaya *(Sendai Medical Center, Miyagi)*; Daisuke Irimada, Haruka Ishikawa, Yu Kaiho *(Tohoku University Hospital, Miyagi)*; Mitsuru Ida, Masahiko Kawaguchi, Kenji Kawamura, Munehiro Ogawa, Hiroshi Okada *(Nara Medical University, Nara)*; Shunji Endo, Yoshinori Fujiwara, Masaharu Higashida, Hisako Kubota, Toshimasa Okada, Hironori Tanaka, Tomio Ueno, Kazuhiko Yoshimatsu *(Kawasaki Medical School Hospital, Okayama)*; Motohiro Kikukawa, Akira Kuriyama, Susumu Matsushime *(Kurashiki Central Hospital, Okayama)*; Daisuke Hashimoto, Hishikawa Hidehiko, Haruaki Hino, Yoji Hisamatsu, Akio Kamiya, Hidefumi Kinoshita, Masato Kita, Toshinori Kobayashi, Taku Michiura, Hirokazu Miki, Tomohiro Murakawa, Hidetaka Okada, Tomohito Saito, Ryoichi Saito, Motohiko Sugi, Genichiro Sumi, So Yamaki, Tomohisa Yamamoto, Aya Yoshida *(Kansai Medical University, Osaka)*; Tatsuya Kambara, Sayaka Kanematsu, Okazaki Satoshi *(Kansai Medical University Kori Hospital, Osaka)*; Takeshi Hijikawa, Hiroaki Kitade, Hidesuke Yanagida *(Kansai Medical University Medical Centre, Osaka)*; Tomoyuki Fujita, Satsuki Fukushima, Naoki Tadokoro *(National Cerebral and Cardiovascular Center, Osaka)*; Taku Furukawa, Yusuke Iizuka, Yuji Otsuka, Masamitsu Sanui, Ikumi Sawada *(Jichi Medical University, Saitama)*; Hideki Iwahashi, Morikazu Miyamoto, Masashi Takano *(National Defense Medical College, Saitama)*; Tsutomu Mieda *(Saitama Medical University Hospital, Saitama)*; Chihiro Ando, Tetsuro Isada, Taku Ishizaki *(Todachuo Medical Hospital, Saitama)*; Qaed Bani Amer, Yuki Fujimoto, Sachi Ishida, Yasuma Kobayashi, Norifumi Kuratani, Tomoe Sakurai, Misa Takada *(Saitama Children’s Medical Center, Saitama City)*; Yutaka Iba, Junji Tsukagoshi, Akira Yamada *(Teine Keijinkai Hospital, Sapporo)*; Yuki Amano, Kentaro Fumoto, Shusaku Noro *(Nakamura Memorial Hospital, Sapporo City, Hokkaido)*; Ayataka Fujimoto, Naoto Kuroda, Kyoichi Tomoto *(Seirei Hamamatsu General Hospital, Shizuoka)*; Yukiyasu Okamura, Teiichi Sugiura, Katshuhiko Uesaka *(Shizuoka Cancer Center, Shizuoka)*; Natsuki Takemura *(International University of Health and Welfare, Mita Hospital, Tokyo)*; Kento Kuroda *(Jikei University Daisan Hospital, Tokyo)*; Megumi Hayashi, Satoi Kaneko, Izumi Kawagoe, Tsukasa Kochiyama, Ai Yamaguchi *(Juntendo University Hospital, Tokyo)*; Nobutsugu Abe, Tadao Ando, Mieko Chinzei, Kouichi Hirano, Yoichi Kobayashi, Ryota Matsuki, Hironori Matsumoto, Akira Motoyasu, Harumasa Nakazawa, Hikari Noguchi, Kaio Okamura, Motoaki Ono, Hiroyuki Seki, Eiji Sunami, Atsushi Tajima, Shinji Tanigaki, Kohji Uzawa, Hidenobu Watanabe *(Kyorin University Hospital, Tokyo)*; Yukari Furuhata, Satoshi Toyama, Tokujiro Uchida *(Medical Hospital of Tokyo Medical and Dental University, Tokyo)*; Yutaka Enomoto, Yuri Furukawa, Yoko Hasumi, Jinso Hirota, Katsuyuki Iida, Shingo Ikeda, Tatsuhiko Ikeda, Natsuko Kawamata, Yosuke Kawasaki, Minako Koizumi, Yoshiharu Kono, Shigeki Kuzuhara, Junichi Maeda, Mai Moriyama, Shoichi Nagamoto, Rinako Nakanishi, Michio Noda, Atsushi Seichi, Shintaro Takahashi, Kobayashi Takashi, Misuzu Takeda, Kenta Tanakura, Katsuyuki Terajima, Munechika Tsuji, Chiharu Ueshima, Naoya Yamamoto, Mari Yamamoto, Toshiya Yokota, Yuki Yoshioka, Sakoh Yuri *(Mitsui Memorial Hospital, Tokyo)*; Shintaro Iwata, Akira Kawai, Shuhei Osaki *(National Cancer Center Hospital, Tokyo)*; Masashi Ishikawa, Masae Iwasaki, Tomonori Morita *(Nippon Medical School Hospital, Tokyo)*; Reina Hirooka, Yoshinori Nakata, Shigehito Sawamura *(Teikyo University Hospital, Tokyo)*; Yusuke Ishida, Aya Kawachi, Takayuki Kobayashi, Fumiaki Nagashima, Naoki Suzuki *(Tokyo Medical University, Tokyo)*; Kohei Ando, Noriko Miyazawa, Yukio Tanaka *(Kugawa Hospsital for Orthopedic Surgery, Yamanashi)*. |
[truncated: 311,760 more chars]
