# Supplementary material for: 30-day postoperative mortality and the effects of hospital preparedness during the COVID-19 pandemic: a pooled analysis of prospective international cohort studies
Source: Lancet Reg Health Eur. 2026 Jan 29;62:101566. doi: 10.1016/j.lanepe.2025.101566 (PMC12870846; doi:10.1016/j.lanepe.2025.101566)
Supplement: Pubmed authorship list [file mmc2.docx]

| Dmitri | Nepogodiev |
| --- | --- |
| Sivesh K | Kamarajah |
| Aneel | Bhangu |
| Radhika | Aacharya |
| Waheed-Ul-Rahman | Ahmed |
| Ehab | ElAmeer |
| Ruth | Blanco-Colino |
| Muhammed | Elhadi |
| Dhruva | Ghosh |
| James C | Glasbey |
| Arda | Isik |
| Kate | Jolly |
| Haytham | Kaafarani |
| Bryar | Kadir |
| Hans | Lederhuber |
| Sezai | Leventoğlu |
| Omar M | Omar |
| Francesco | Pata |
| Maria | Picciochi |
| Peter | Pockney |
| Marie Dione | Sacdalan |
| Joana FF | Simoes |
| Georgios | Tsoulfas |
| Daoud | Chaudhry |
| Ruth | Blanco Colino |
| Irani | Duran |
| James | Glasbey |
| Rohan | Gujjuri |
| Sivesh | Kamarajah |
| Santhosh | Karri |
| Kayani | Kayani |
| Stephen | Knight |
| Samuel | Lawday |
| Elizabeth | Li |
| Harvi | Mann |
| Fatima | Mansour |
| Kenneth | McLean |
| Omar | Omar |
| Maria | Piccochi |
| Irene | Santos |
| Joana | Simoes |
| Chris | Varghese |
| Maryam | A |
| Sultan | A Al Amri |
| Juan | A Mejia |
| Gregor | A Stavrou |
| N | Aagaard |
| Junaid | Aamir |
| Jesús | Aarón Martínez Alonso |
| F | Aarts |
| Y | Aawsaj |
| Norhafiza | Ab Rahman |
| Islah Munjih | Ab Rashid |
| Teresa | Aba Mensah |
| Muna | Aba Zaid |
| Muath | Abaalkhail |
| Adnan | Ababneh |
| H | Ababneh |
| Hazim | Ababneh |
| Laila | Ababneh |
| M | Ababneh |
| Roba | Ababneh |
| Rafael | Abad Alonso |
| Alfredo | Abad Gurumeta |
| A | Abad-Gurumeta |
| A | Abad-Motos |
| Ane | Abad-Motos |
| Mussab | Abaker |
| John | Abanga Alatiiga |
| S | Abas |
| Adam | Abass |
| J | Abassy |
| E | Abate |
| Emmanuele | Abate |
| C | Abatini |
| Sheraz | Abayazeed Ahmed |
| Olukayode | Abayomi |
| Alaa | Abazeed |
| J | Abba |
| Abdurrahman | Abba Sheshe |
| Bader | Abbad |
| F | Abbadessa |
| Francesco | Abbadessa |
| Osaid | Abbadi |
| Malaz | Abbakar |
| A | Abbas |
| Ahmed M | Abbas |
| AM | Abbas |
| AS | Abbas |
| Asad | Abbas |
| Aya M | Abbas |
| F | Abbas |
| FM A | Abbas |
| FMA | Abbas |
| Jihad | Abbas |
| Manzar | Abbas |
| Omer | Abbas |
| S | Abbas |
| M | Abbasi |
| A | Abbasov |
| Aykhan | Abbasov |
| Olivier | Abbo |
| Daniel | Abbott |
| S | Abbott |
| T | Abbott |
| Tom | Abbott |
| Tom EF | Abbott |
| Waleed | Abd |
| Tayma | Abd Alghafour |
| Wael | Abd El-Ghani |
| HA S | Abd Elazeem |
| HAS | Abd Elazeem Mohammed |
| Reda | Abd ElGhany |
| Mustafa | Abd Elsayed |
| Ahmed | Abd Elwahab |
| Sami | Abd Elwahab |
| NN | Abd Kahar |
| EH | Abd Wahab |
| MM | Abd-Elkarem |
| AY | Abd-Elkariem |
| S | Abd-elsalam |
| Sherief | Abd-Elsalam |
| Joel | Abdala Junior |
| Ahmad | Abdalah |
| H | Abdalaziz |
| Lana | Abdalgadir Ahmed Mohamed |
| A | Abdalhadi |
| Alya | Abdalhadi |
| Ahmed | Abdalla |
| M | Abdalla |
| Samir | Abdalla |
| Shimaa | Abdalla |
| Siddig | Abdalla |
| Hdaya | Abdalla S Benabdalla |
| EA | Abdallah |
| Emne | Abdallah |
| Ghaida | Abdallah |
| Lubna | Abdallah |
| M | Abdallah |
| Munir | Abdallah |
| Rasha | Abdallah |
| Hani | Abdalnour |
| Bashar | Abdeen |
| S | Abdeewi |
| Saedah | Abdeewi |
| L | Abdeh |
| Louai | Abdeh |
| S | Abdel Al |
| Shrouk | Abdel Fattah |
| R | Abdel Jalil |
| Ali H | Abdel Sater |
| Mahmoud | Abdel-Aleem |
| M | Abdel-bari |
| Wafaa | Abdel-Elsalam |
| Areej | Abdel-Fattah |
| Nour | Abdel-Fattah |
| K | Abdel-Galil |
| Ibrahim | Abdel-Hafez |
| M | Abdel-Maboud |
| Abdelrahman | Abdelaal |
| Khaled | Abdelazeem |
| S | Abdelaziem Mustafa |
| Areej A | Abdelaziz |
| Mohammed | Abdelaziz |
| A | Abdelbagi |
| Mohamad | Abdelbagi |
| Abouelnour | Abdelbaset |
| Hesham | Abdeldayem |
| Mahmoud | Abdelfattah |
| Alwaleed | Abdelgadir |
| Khaled | Abdelgalel |
| Moslem | Abdelghafar |
| Mohamed | Abdelghafor Hassanin |
| Mohammed | Abdelhafez |
| Abdelkarim | Abdeljalil |
| M | Abdelkabir |
| Mohammed | Abdelkabir |
| Ibrahim | Abdelkader Salama |
| M | Abdelkareem |
| Mohamed | Abdelkareem |
| Mohamed M | Abdelkarem |
| M | Abdelkarim |
| Mostafa | Abdelkarim |
| M | Abdelkhalek |
| Mohamed | Abdelkhalek |
| Fatima | Abdellah |
| A | Abdelmajeed |
| Ahmed | Abdelmajeed |
| Abubaker | Abdelmalik |
| Ahmed | Abdelmawla |
| ElTahir | Abdelrahim |
| N | Abdelrahim |
| A | Abdelrahman |
| Abdelrahman | Abdelrahman |
| Haneen | Abdelrahman |
| Ali | Abdelraouf |
| Karim | Abdelraouf Moawad |
| S | Abdelrhman |
| K | Abdelsaid |
| A | Abdelsamed |
| Ahmed | Abdelsamed |
| K | Abdelwahab |
| W | Abdelwahab |
| Hafni | Abderrazaq |
| Desalegn | Abdissa |
| H | Abdou |
| Hossam | Abdou |
| Mostafa | Abdou |
| Diallo | Abdoul Azize |
| D | Abdoun |
| M | Abdoun |
| Meriem | Abdoun |
| Mohammad | Abdow |
| Ahmed | Abdrabou |
| Mahmmoud | Abdualqader |
| Aya | Abdul Al |
| DA | Abdul Aziz |
| Jumana | Abdul Hameed |
| Najat | Abdul Hameed |
| N | Abdul Maei |
| Norazila | Abdul Rahim |
| Noorneza | Abdul Rahman |
| UH | Abdul Rauf |
| Omar | Abdul Salam |
| H | Abdul-Jabar |
| Hani B | Abdul-Jabar |
| Alhassan | Abdul-Mumin |
| Safaa | Abdulaal |
| Omar | Abdulateef |
| Abdulmajeed | Abdulaziz Saeedi |
| F | Abdulfattah |
| Muhammad | AbdulHakeem |
| Sakhr | Abdulhakeem Al-maswari |
| E | Abdulkader |
| Adnan | Abdulkadir Mohammed |
| Amal | Abdulkareem |
| Miriam | Abdulkarim Polo |
| Mohammed | Abdull |
| N | Abdulla |
| Abakar | Abdullaev |
| Abdullah | Abdullah |
| Bahiyah | Abdullah |
| Nabila | Abdullah |
| Saleha | Abdullah |
| Shahbaz | Abdullah |
| Nayrouz | abdullah Abulshuwashi |
| Zakarya | Abdullah Al-Zaazaai |
| Ibrahim | Abdullah Hakami |
| Alsnosy | abdullah Khalefa mohammed |
| Muhammad | Abdullah Khalid |
| Ahmed | Abdullah Shaalah |
| Lamess | Abdullaha |
| Alhassan | Abdullahi |
| Habiba | Abdullahi |
| IH | Abdullahi |
| Lawal | Abdullahi |
| M | Abdullahi |
| Sani | Abdullahi |
| Sani | Abdullahi Yunusa |
| Abdulmalek | Abdulmalek |
| Abdulmuez | Abdulmalik |
| Ahmed | Abdulmohsen |
| Seemal | AbdulQadir |
| Nabil | Abdulqawi |
| Abdullah | Abdulrahem |
| F | Abdulrahman |
| Mamuda | Abdulrahman |
| Mohamed | Abdulrahman |
| Taha | Abdulrahman |
| Muna | AbdulRazzaq Tahlak |
| Albaraa | Abdulsalam |
| Fareed | Abdulsalam |
| Khalifa | Abdulsalam |
| Moruf | Abdulsalam |
| Taiceer | Abdulwahab |
| E | Abdulwahed |
| Eman | Abdulwahed |
| Murad | Abdunabi |
| L | Abdur-Rahman |
| Lukman | Abdur-Rahman |
| Ebrahim | Abdurab |
| Oumer | Abdurehman |
| Abdussemee | Abdurrazzaaq |
| R | Abdus-salam |
| Rukiyat | Abdus-salam |
| Mehmet | Abdussamet Bozkurt |
| Anthonia | Abe |
| Nobutsugu | Abe |
| Tatsuro | Abe |
| Engida | Abebe |
| Kirubel | Abebe |
| M | Abebe |
| Metasebia | Abebe |
| Nebyou | Abebe |
| Mersha | Abebe Woldemariam |
| JT | Abebrese |
| Francisco | Abed |
| Haneen | Abed |
| Lina | Abedalqader |
| Y | Abedin |
| Yasmin | Abedin |
| Marian | Abedua Harrison |
| Livingston | Abel |
| MK | Abel |
| A | Abelevich |
| Alexander | Abelevich |
| M | Abellán |
| Miriam | Abellan Lucas |
| JMI | Abellera |
| J | Abeloos |
| K | Abhilashi |
| Wijden | Abichou |
| Rubaba | Abid |
| Adekunle | Abiodun |
| Olajide | Abiola |
| Paul | Abiola |
| Henry | Abiyere |
| OH | Abiyere |
| Ahmad | Abo Arar |
| Mustafa | Abo Mohsen |
| Ahmed | Abo Shanab |
| Ghaleb | Aboalsamh |
| Hajir | Aboazamazem |
| Aya | Abodeeb |
| A | Aboelkassem Ibrahim |
| Roger | Aboelkhel |
| Z | Aboharp Hasan |
| Ziad | Aboharp Hasan |
| Farah | Abojeila |
| Ibrahim | Abolaji Alabi |
| Orlando | Abonia Gonzalez Abonia Gonzalez |
| A | Abood |
| NE | Abosamak |
| Yahya | Abosnaina |
| Ahmed | Abostate |
| Amna | Abou Bakr |
| MK | Abou Chaar |
| Mohamad K | Abou Chaar |
| Hussein | Abou-Abbass |
| M | Abou-Abdallah |
| AK | Abou-Foul |
| J | Abou-Khalil |
| Jad | Abou-Khalil |
| A | Abouassi |
| Layth | Abouassi |
| Majd | Abouassi |
| Samar | Aboubakr |
| Mohammed | Aboubeirah |
| Mohamed | Abouelazayem |
| Galal | Abouelnagah |
| Yossof | Abouelnagah |
| H | Aboulkassem |
| Omar | AbouMadawy |
| Shereen | Aboutaleb |
| Ayham | Aboutrab |
| Amr | Abouzid |
| A | Abozid |
| H | Abozied |
| Hesham | Abozied |
| Bejoy | Abraham |
| Camara | Abraham Faya |
| Jenevive | Abrahams |
| S | Abramowicz |
| Shelly | Abramowicz |
| M | Abrar |
| A | Abrate |
| Alberto | Abrate |
| Tassew | Abreha |
| A | Abreu da Silva |
| Alberto | Abreu da Silva |
| Teklebirhan | Abrha |
| Arsan | Abu abed |
| Laith | Abu Abed |
| Najib | Abu Draz |
| S | Abu Freih |
| MFK | Abu hallalah |
| M | Abu Hamraa |
| M | Abu Hilal |
| Mohammed | Abu Hilal |
| Dima Y | Abu Ismail |
| Mustafa | Abu Jayyab |
| M | Abu Mohsen Daraghmeh |
| Mustafa | Abu Mohsen Daraghmeh |
| Dima | Abu muhfouz |
| Hamza | Abu Obead |
| W | Abu Rashed |
| R | Abu Salah |
| A | Abu salhiyeh |
| Alaa | Abu Salhiyeh |
| Md | Abu Sayed |
| Ammar | Abu Tarieh |
| FJ | Abu Zanouneh |
| Hamdoon | Abu-Arish |
| Basil | Abu-Eisheh |
| Nizar | Abu-Ishkerih |
| Luai | Abu-Ismail |
| M | Abu-Jeyyab |
| Tareq | Abu-libdeh |
| Marah | Abu-Mehsen |
| I | Abu-Nayla |
| Ahmed | Abu-Zaid |
| Z | Abual-Rub |
| M | Abualjadayel |
| Faisal | Abualteen |
| Carla | Abuawad |
| Abdullah | Abubakar |
| Ahmed | Abubakar |
| M | Abubakar |
| Abubakr | Abubakr |
| Asmaa | Abubakr |
| Hossam | Abubeih |
| Burçin | Abud |
| Hadeel | Abudari |
| A | Abudher |
| Abdulhafid | Abudher |
| Ehab | Abuhamour |
| Maaly | Abuhlaiga |
| Khatab | Abuissa |
| Mahmoud | Abukhadra |
| S | Abukhalaf |
| SA | Abukhalaf |
| Sadi | Abukhalaf |
| Sadi A | Abukhalaf |
| Muhammad | Abukhater |
| Daniel | Abulafia |
| Amro | Abuleil |
| Saleh | Abumahara |
| Huthifa | Abunawas |
| A | Abuown |
| Mohammad | Aburahmah |
| Fatima | Aburayyan |
| Sarah | Aburima |
| Abdelrahman | Abusabeib |
| Samer | Abusadah |
| Marwa | Abusalem |
| Malek | Abusannoga |
| A | Abutaka |
| Ahmad | Abutaka |
| Malak | Abutaleb |
| Khalil | Abuzaina |
| IA | Abuzeid |
| Y | Abye Negatu |
| Eduard | Acatrinei |
| Giulio | Accarino |
| Guilherme | Accorsi |
| Alfeu | Accorsi Neto |
| F | Acebes García |
| Fernando | Acebes García |
| M | Achalandabaso Boira |
| Mar | Achalandabaso Boira |
| Hriday | Acharya |
| M | Acharya |
| Metesh | Acharya |
| Shivanie | Acharya |
| AR | Achek |
| Michele | Achille Crespi |
| P | Achimas-Cadariu |
| Patriciu | Achimas-Cadariu |
| AS | Acikgoz |
| J | Ackah |
| Travis | Ackermann |
| Jesus | Acosta |
| Maria | Acosta |
| S | Acosta |
| Úrsula | Acosta |
| Yancy | Acosta |
| Lina M | Acosta Buitrago |
| MA | Acosta Mérida |
| J | ACourt |
| Jane | Acquaye |
| R | Acra-Tolari |
| Ricardo | Acra-Tolari |
| Gastón | Acuña |
| Luke | Adagrah Aniakwo |
| AA | Adam |
| John | Adam |
| MAA | Adam |
| Eman | Adam Abdalla |
| Laszló | Ádám Bihari |
| ME | Adam Essa |
| Soujanya | Adamala |
| A | Adamec |
| M | Adamina |
| Michel | Adamina |
| L | Adamoli |
| Laura | Adamoli |
| C | Adams |
| J | Adams |
| Katie | Adams |
| S | Adamski |
| Nicholas | Adamson Barnes |
| A | Adamu |
| Akshath | Adapa |
| E | Addae-Boateng |
| Bassam | Addas |
| A | Addissie |
| Andi | Ade Ramlan |
| Miguel | Adeba García |
| Emmanuel | Adebajo |
| Ademola | Adebanjo |
| Idowu | Adebara |
| IO | Adebara |
| Olaolu | Adebayo |
| Sikiru A | Adebayo |
| Ganiyu | Adebisi Rahman |
| Oluwaseun | Adeboyejo |
| Muhammad | Adeel Akhtar |
| S | Adegbola |
| Samuel | Adegbola |
| Samuel | Adegboyega Olatoke |
| Dina | Adel |
| I | Adel |
| Ahmed | Adel abdelaty |
| E | Adel Hamdoun Aziz |
| Mohamed | Adel Nassef |
| Aderinsola | Adelaja |
| N | Adeleke |
| Amos | Adeleye |
| Elena | Adelina Toma |
| Ephrem | Adem |
| Samuel | Ademola |
| A | Ademuyiwa |
| Adesoji | Ademuyiwa |
| AO | Ademuyiwa |
| Abimbola | Adeniran |
| Abiodun | Adeniran |
| AS | Adeniran |
| AA | Adeniyi |
| Adebayo | Adeniyi |
| Mehjabeen | Adenwalla |
| Adewale | Aderounmu |
| Opeoluwa | Adesanya |
| Oluwaseyi | Adesina |
| Muideen | Adesola |
| AbdulHafiz | Adesunkanmi |
| Musliu | Adetola Tolani |
| Masud | Adewusi |
| Olabisi | Adeyemo |
| OT | Adeyemo |
| A | Adeyeye |
| Ademola | Adeyeye |
| Ibrahim | Adham |
| Mohamed | Adhnan Thaha |
| S | Adhya |
| H | Adi |
| Hussam | Adi |
| A | Adiamah |
| Alfred | Adiamah |
| Valentine | Adikaibe |
| Ahmed | Adil |
| Md Tanveer | Adil |
| Ali | Adil Ali karar |
| E | Adinolfi |
| Andika | adiputra Thehumury |
| Adewale | Adisa |
| AO | Adisa |
| Dita | Aditianingsih |
| Theophilus | Adjeso |
| TJK | Adjeso |
| Alsafe | Adlan |
| Tina | Adler |
| Ainal | Adlin Naffi |
| A | Admasu |
| Azarias | Admasu |
| HM | Adnan |
| Nadir | Adnan Hacım |
| Yvonne | Adofo-Asamoah |
| Gustavo | Adolfo Angel |
| Carlos | Adolfo Marroquín Paiz |
| Mohammed | Adrees |
| Zulay | Adriana Calderon Barajas |
| Ludwigvan | Adriano Bustamante Silva |
| Marcos | Adrianzén |
| María | Adrien Lara |
| A | Adroher |
| Maame | Aduse-Poku |
| R | Advani |
| Rajeev | Advani |
| Mohammad | Adya Firmansha Dilmy |
| Joel | Adze |
| G | Aeby |
| Majed | Aeed |
| S | Afaghi |
| L | Affronti |
| Amgad | Afifi |
| Eman | Afifi |
| Nermeen | Afifi |
| Abdelrahman | Afify |
| Abdulrahman O | Afolabi |
| Akinwale | Afolabi |
| BB | Afolabi |
| Bosede | Afolabi |
| M | Afonso-Garcia |
| S | Afroze |
| Salim | Afshar |
| N | Aftab |
| R | Aftab |
| Raiyyan | Aftab |
| Oludolapo | Afuwape |
| Ameer | Afzal |
| Maimoona | Afzal |
| Mohamed | Afzal |
| Sadia | Afzal |
| Christos | Agalianos |
| M | Agapov |
| Mikhail | Agapov |
| Anjoo | Agarwal |
| Arnav | Agarwal |
| Gaurav | Agarwal |
| K | Agarwal |
| Ketan | Agarwal |
| Sunny | Agarwal |
| Varun | Agarwal |
| Ervis | Agastra |
| Mouhamed | Agbadebo |
| Ademola | Agbaje |
| Kwabena | Agbedinu |
| DYD | Agbley |
| Nelson | Agboadoh |
| P | Agbonrofo |
| Peter | Agbonrofo |
| Mohammed | Ageeli |
| A | Aggarwal |
| G | Aggarwal |
| Gaurav | Aggarwal |
| Manisha | Aggarwal |
| Sonali | Aggarwal |
| C | Aggeli |
| K | Aghababyan |
| Kristina | Aghababyan |
| Ifeanyi | Aghadi |
| Ilgar | Aghalarov |
| Ughur | Aghamaliyev |
| SMK | Aghamir |
| ZS | Aghamir |
| Afag | Aghayeva |
| Kayvan | Aghazadeh |
| ET | Agida |
| Eyaofun | Agida |
| J | Agilinko |
| I | Agledahl |
| A | Agnes |
| Annamaria | Agnes |
| Salvatore | Agnes |
| H | Agnus Moorthiraj |
| O | Agodirin |
| Hermann | Agossou |
| C | Agostini |
| P | Agoston |
| Amit | Agrawal |
| Mansi | Agrawal |
| Rachit | Agrawal |
| S | Agrawal |
| Tulika | Agrawal |
| LA | Agredo Luna |
| F | Agredo Villaquiran |
| Héctor J | Aguado |
| HJ | Aguado |
| H | Aguado Lopez |
| H | Aguado López |
| Omar | Aguayo |
| JL | Aguayo-Albasini |
| SV | Agudelo Mendoza |
| M | Aguennouz |
| Renato | Aguera Oliver |
| Mauricio | Agüero Mariño |
| S | Aguiar Jr |
| S | Aguiar Júnior |
| Samuel | Aguiar Júnior |
| A | Aguilar |
| Ana | Aguilar |
| Ruben | Aguilar |
| CE | Aguilar Alvarado |
| Jorge L | Aguilar Frasco |
| J | Aguilar-Jimenez |
| M | Aguilera Lorena |
| Maria | Aguilera Lorena |
| M | Aguilera-Arevalo |
| Maria-Lorena | Aguilera-Arevalo |
| ML | Aguilera-Arévalo |
| B | Aguinagalde |
| Borja | Aguinagalde |
| Asier | Aguirre |
| L | Aguzzoli |
| Lorenzo | Aguzzoli |
| Fareeda | Agyei |
| S | Agyeiwaa Owusu |
| Akosua | Agyemang-Prempeh |
| Thomas | Agyen |
| K | Agyen Mensah |
| Kwasi | Agyen Mensah |
| S | Ahad |
| Waseem | Ahamed |
| Roghayyeh | Ahangari |
| K | Aher |
| Thomas | Aherne |
| TM | Aherne |
| Hamza | Ahlaou |
| Saravpreet | Ahluwalia |
| Afaf | Ahmad |
| Aline | Ahmad |
| Amer | Ahmad |
| Ashfaq | Ahmad |
| Aya | Ahmad |
| B | Ahmad |
| Basel | Ahmad |
| Fateen | Ahmad |
| H | Ahmad |
| Maleeha | Ahmad |
| Manzoor | Ahmad |
| Misbahu | Ahmad |
| Mohamad | Ahmad |
| Niarah | Ahmad |
| Quazi | Ahmad |
| Reyaz | Ahmad |
| Rofida | Ahmad |
| S | Ahmad |
| Shabir | Ahmad |
| Shahrukh | Ahmad |
| Sheraz | Ahmad |
| Siddique | Ahmad |
| SJ | Ahmad |
| Tareq | Ahmad |
| Y | Ahmad |
| Zeeshan | Ahmad |
| Muzaffar | ahmad Ahmad |
| Ayah | Ahmad Al_shraideh |
| Haneen | Ahmad Alhami |
| Shafiq | Ahmad Chughtai |
| Zainab | Ahmad Haq |
| Faraz | ahmad Khan |
| Mumtaz | Ahmad Khan Khan |
| Fuad | Ahmad Khan Niazi |
| Zamir | AHMAD Shah |
| Imdad | Ahmad Zahid |
| N | Ahmadi |
| Navid | Ahmadi |
| S | Ahmadi |
| Sayedali | Ahmadi |
| SMS | Ahmadi Rashti |
| Ali | Ahmadvand |
| A | Ahmed |
| Abdelkareem | Ahmed |
| Abdullah | Ahmed |
| Abedelrahman | Ahmed |
| Ahmed | Ahmed |
| Aishah | Ahmed |
| AMAM | Ahmed |
| Arooj | Ahmed |
| Ayman | Ahmed |
| Azaz | Ahmed |
| Ehsan | Ahmed |
| F | Ahmed |
| Faazil | Ahmed |
| Faryal | Ahmed |
| Ghazia | Ahmed |
| Haseeb | Ahmed |
| Hassaan | Ahmed |
| I | Ahmed |
| Idrees | Ahmed |
| Iffat | Ahmed |
| Irshad | Ahmed |
| Islam | Ahmed |
| Jawad | Ahmed |
| K | Ahmed |
| Kaleem | Ahmed |
| Khalid | Ahmed |
| M | Ahmed |
| Maira | Ahmed |
| Manhal | Ahmed |
| Mariam | Ahmed |
| Mnewer Y | Ahmed |
| Mohammed | Ahmed |
| Muhammed | Ahmed |
| MY | Ahmed |
| Nasir | Ahmed |
| Nauman | Ahmed |
| O | Ahmed |
| Omar | Ahmed |
| S | Ahmed |
| Safia | Ahmed |
| Sara | Ahmed |
| Shahnoor | Ahmed |
| Shakil | Ahmed |
| SM | Ahmed |
| W | Ahmed |
| Waqas | Ahmed |
| Zubair | Ahmed |
| Abdelrahman | Ahmed Abdelrahman Ali |
| Omar | Ahmed Abdelwahab |
| Manal | Ahmed Altoumi |
| Chamakhi | Ahmed Amine |
| Mohammed | ahmed Babikir |
| Mahmoud | Ahmed Ebada |
| M | Ahmed Elamin Elnour |
| Kemel | Ahmed Ghotme Ghotme |
| Abdilaahi | Ahmed Hayir |
| H | Ahmed kareem |
| Abdallah | Ahmed Mezel Al-Azzam |
| ME | Ahmed Mohamed |
| Elmi | Ahmed Mohamed Jimaale |
| Muhammad | Ahmed Naseer |
| Talha | Ahmed Qureshi |
| Tilal | Ahmed Raza |
| Shujat | Ahmed Riaz |
| Sarah | Ahmed Saad |
| Khursheed | Ahmed Samo |
| Imtiaz | Ahmed Shakir |
| Mostafa | Ahmed Shehata |
| Ebrahim | Ahmed Yousof |
| Mahlet | Ahmedin |
| A | Ahmeidat |
| Ismail | Ahmet Bilgin |
| Muhammad | Ahsan Iqbal Siddiqui |
| Muhammad | ahsan Khan |
| Naeimah | Ahseen |
| Abdurahman | Ahtash |
| N | Ahuja |
| K | AI Nwijy |
| Khaled | AI Nwijy |
| Turki | AI Zahrani |
| Martina | Aida Angeles |
| AL S | Aidar |
| Askar | Aidarov |
| E | Aigbivbalu |
| F | Aigner |
| Felix | Aigner |
| Taylor | Aiken |
| Ramez | Ailabouni |
| A | Aime |
| Adeline | Aimé |
| Rakotonarivo | Aina Andrianina Vatosoa |
| A | Aiolfi |
| Alberto | Aiolfi |
| A | Airey |
| Angelo | Airoldi |
| Oseremen | Aisuodionoe-Shadrach |
| Rita | Ait benhamou |
| E | Aitken |
| Jake | Aitken |
| Javier | Aitor Zabala Lopez-Maturana |
| Giada | Aizza |
| Shereen | Ajab |
| Okeoghene | Ajagha |
| Olalekan | Ajai |
| Hatem | Ajaj |
| Dany | Ajami |
| Akinlabi | Ajao |
| Adekunle | Ajayi |
| Peter | Ajayi |
| G | Ajcip |
| Gaby | Ajcip |
| Samuel | Ajekwu |
| Temitope | Ajekwu |
| Narjiss | Aji |
| Hafees | Ajibola |
| Olalekan | Ajiboye |
| Abdulrazag | Ajlan |
| Nadia | Ajomah |
| G | Akaba |
| Godwin | Akaba |
| OG | Akaba |
| Ali | Akadh |
| M | Akalin |
| Murat | Akalin |
| Chris | Akani |
| Bolaji | Akanni |
| Ömer | Akay Ömer |
| E | Akaydin |
| A | Akbar |
| Ali | Akbar |
| Bilal | Akbar |
| J | Akbar |
| S | Akbar |
| Hazrat | Akbar Akbar |
| A | Akbas |
| Ahmet | Akbas |
| Oktay | Akça |
| Mertcan | Akçay |
| Yesim | Akdeniz |
| Nouf | Akeel |
| Adeleke | Akeem Aderogba |
| Taiwo | Akeem Lawal |
| Utku | Akgor |
| N | Akhavan Fomani |
| Hamed | Akhavizadegan |
| Melika | Akhbari |
| Amina | Akhtar |
| F | Akhtar |
| Fahad | Akhtar |
| MA | Akhtar |
| Munazza | Akhtar |
| Naseem | Akhtar |
| Tasleem | Akhtar |
| Ramsha | Akhund |
| Jehad | Akiely |
| YB | Akililu |
| E | Akin |
| Emrah | Akin |
| Opeyemi | Akinajo |
| Success | Akindoyin |
| Olufemi | Akinloa |
| A | Akinmade |
| Akinola | Akinmade |
| Sanusi | Akinsola |
| Tosin | Akinyemi |
| Khalid | Akkour |
| Michel | Akl |
| A | Akmercan |
| Ahmet | Akmercan |
| Elif | Akova Deniz |
| AG | Akpede |
| Marcellin | Akpla |
| Oghenevwegba | Akpoghor |
| Shahzad | Akram |
| Farah | Akthar |
| YE | Aktimur |
| Anton | Akulaev |
| C | Akyol |
| Cihangir | Akyol |
| Aggeliki | Al |
| Murad | Al Abdallah |
| Qurrat | Al Ain Atif |
| Ghadeer | Al Ajmi |
| Khalil | Al Ajmi |
| Nazeeh | Al Aktaa |
| Marwan | Al Aliwy |
| Ahmed | Al Ameer |
| Abdulrahman | Al Amri |
| Ayman | Al Amri |
| A | Al Ansari |
| Tala | Al Asadi |
| Samer | Al Athath |
| S | Al Awwad |
| A | Al Ayed |
| Rawan | Al Azhar |
| Abd | Al Aziz Lanagrán Torres |
| Ghalib | Al Badaai |
| Nawf | Al Balushi |
| Z | Al Balushi |
| Zainab | Al Balushi |
| Wameedh | Al Bassam |
| Zakaria | Al Bdour |
| Seif | Al Dahabrh |
| Mahmood | AL Dhaheri |
| Sajedah | Al DHOUN |
| Mohammed | Al Dosouky |
| Mohammed | Al Duhileb |
| Jubran | Al Faifi |
| A | Al Farai |
| M | Al Farsi |
| Maather | Al Farsi |
| Wadha | Al Ghafri |
| Alaa | Al Ghafry |
| Rawda | Al Gohary |
| Hessa | Al Habes |
| Hiba | Al Hage Diab |
| Abdulaziz | Al Harthi |
| I | Al Hasan |
| M | Al Hinai |
| AbdulAziz | Al Hindi |
| Mohammed | Al HOSNI |
| H | Al Houri |
| Lama | Al Humaid |
| Rayet | al islam Ben jouira |
| Mohammed | Al Jamahir |
| Ziad | Al jarad |
| Hussain | Al Jawad |
| Maha | Al kalbani |
| Moza | Al Kalbani |
| Lamya | Al Kharusi |
| Safiya | Al Kharusi |
| Ahmad | Al Khassawneh |
| Salim | Al Lahham |
| F | Al maadany |
| Faraj | Al maadany |
| Leyla | Al Mahdawi |
| Hossam | Al Mahdy |
| Reem | Al Makari |
| A | Al Malkawi |
| AR | Al manasra |
| H | Al Miskry |
| Rehab | Al Moagal |
| Tareg | Al Momani |
| Fadhl | Al muhtadi |
| Rahaf | Al Mulke |
| Mohammed | Al Mutani |
| M | Al Naamani |
| Aminah | Al Nafesa |
| Hamza | Al Naggar |
| H | Al Naggar. |
| Syed | Al Nahian |
| Majed | Al Najjar |
| Tasneem | Al najjar |
| Awaji | Al nami |
| Saleh | Al Nassar |
| Shehanah | Al Omair |
| Faisal | Al Otaibi |
| Hani | Al Qadhi |
| Rashed | Al Qudhaya |
| R | Al Raddadi |
| Amani | Al Raisi |
| Asmaa | Al Rashed |
| Nihal | Al Riyami |
| S | Al Riyami |
| Salim | Al Riyami |
| Hilal | Al Sabti |
| Ghiath | Al Saied |
| Ghadeer | Al sanany |
| Mohamed | AL Sayed |
| Maha | Al shaibi |
| A | Al Sharie |
| Ahmed | Al Sharie |
| S | Al Sharie |
| Asmaa | Al shukri |
| Hana | Al Shurman |
| Omar | Al Smadi |
| Saud | Al Subaie |
| Kais | Al Suyyagh |
| M | Al Tarakji |
| Hajr | Al Wadei |
| Zakaria | Al Yahya |
| Attiya | Al zahrani |
| Zeina | Al Zein |
| Y | Al Zu’bi |
| D | Al Zubi |
| Anwaar | Al_Dhafif |
| Buthina | Al_jarmozi |
| Siham | Al_maqtari |
| B | Al_sharash |
| Salma | Al- Houssami |
| Rashid | Al-Abri |
| Emad | Al-Absi |
| Sofian | Al-Adwan |
| S | Al-Ameri |
| Saba | Al-ameri |
| Hussam | Al-atiyah |
| M | Al-Azzawi |
| Marwa | Al-Azzawi |
| Nimer | Al-azzeh |
| Ismail A | Al-Badawi |
| H | Al-Balas |
| Hasan | Al-Balas |
| A | Al-Bourah |
| T | Al-Dabaa |
| Tawfik | Al-Dabaa |
| Ali | Al-Darabah |
| A | Al-Darobi |
| Awsan | AL-Dhaheri |
| M | Al-Dhaheri |
| Wedad | Al-dolat |
| S | Al-Embideen |
| Somya | Al-Embideen |
| Fatima | Al-Eryani |
| H | Al-Fahel |
| S | Al-Falahat |
| A | Al-Fraihat |
| Maha | Al-Gilani |
| Amro | Al-Habib |
| Y | Al-Harazi |
| A | Al-Harbawee |
| A | Al-Harbawi |
| Fahad | Al-Hasani |
| Amer | Al-hebbah |
| M | Al-howthi |
| Mohammed | Al-howthi |
| Ali | Al-Isawi |
| Mohammad | Al-Jadaan |
| Amar | Al-Jarrah |
| Hothaifa | Al-Jarrah |
| Salsabeel | Al-Jarrah |
| MA | Al-Juaifari |
| SA | Al-Kailani |
| Adil | Al-Karim Manji |
| F | Al-kasaji |
| Farah | Al-kasaji |
| Ziad | Al-Khaddar |
| E | Al-Kharashi |
| W | Al-Khyatt |
| Motasem | Al-latayfeh |
| Ahmed | Al-madhrahi |
| Sanabel | Al-Maghrabi |
| Shehab | Al-Mahdi |
| Husain | Al-Mahmeed |
| Hazem | Al-Mandeel |
| S | Al-Maqtari |
| Sahar | Al-maqtari |
| Hayder | Al-Masari |
| Mohammed | Al-Masood |
| M | Al-Masri |
| Mahmoud | Al-Masri |
| Abdulrahman | Al-Mohammad |
| Ahmad | Al-Mouakeh |
| Abdullah | Al-Mujaini |
| Y | Al-Mukhaizeem |
| Youssef | Al-Mukhaizeem |
| A | Al-mukhtar |
| H | Al-Naggar |
| Hamza | Al-Naggar |
| Dania | Al-Najjar |
| H | Al-Najjar |
| Hani | Al-Najjar |
| Y | Al-Najjar |
| Bilal | Al-Nawas |
| H | Al-Omishy |
| Ayman | Al-oqabi |
| Saeid | Al-oribi |
| Malk | Al-Osta |
| Hadeel | Al-Othman |
| Mohammad | Al-Qannas |
| Ali | Al-Qannass |
| M | Al-qattan |
| Mohammad | Al-Qattan |
| Qusay | Al-Qurashi |
| Ali | Al-Radhi |
| Osman | Al-Radi |
| Ibrahim | Al-Raimi |
| Wisam | Al-Ramli |
| Aya | Al-Rashdi |
| Nina | Al-Saadi |
| Rafat | Al-saban |
| Yusra | Al-Sabbagh |
| A | Al-Samaraee |
| Tariq | Al-Shaiji |
| Ghadeer | Al-Shaikh |
| Sonds | Al-Shammakh |
| Ahmad | Al-Shaye |
| M | Al-Shehari |
| Mohammed | Al-Shehari |
| Z | Al-sheikh ali |
| Zaid | Al-sheikh ali |
| Abdallah | Al-Shibi |
| Abdel-Ellah | Al-Shudifat |
| Mutlaq | Al-Sihan |
| Ibrahim | Al-Slaibi |
| A | Al-Sukaini |
| Ahmad | Al-Sukaini |
| S | Al-Tahayneh |
| Y | Al-Tamimi |
| Yahia | Al-Tamimi |
| Mohannad | Al-Tarakji |
| Abdullatif | Al-Terki |
| Mohammad | Al-thaher |
| A | Al-Touny |
| SA | Al-Touny |
| Mohammed | Al-Urfan |
| Muntadhir | Al-uzri |
| Omer | Al-Yahri |
| M | Al-Yaseen |
| Mustafa | Al-Yaseen |
| Karim | Al-Zazay |
| Reham | Al-Zyadat |
| S | Alaa |
| Sherif | Alaa |
| O | AlAamer |
| Ohood | AlAamer |
| J | Alabbad |
| Jasim | Alabbad |
| Amira | Alabbasi |
| Yousof | Alabdulkarim |
| Reem | AlAbdulwahed |
| R | Alabo |
| Mohamad | Alabras |
| A | Aladaileh |
| Ammar | Aladaileh |
| Omar | Aladawi |
| Samuel | Alade |
| Timothy | Aladelusi |
| A | Aladeojebi |
| Ali | Alafif |
| Abeer | Alaglan |
| Adel | Alahaidib |
| Hani | Alahdal |
| Feras | Alahmad |
| Ibrahim | Alahmadi |
| Ahmed | Alahmari |
| S | Alahmed |
| Salman | Alahmed |
| N | Alajaji |
| Nouf | Alajaji |
| A | Alajalen |
| Amer | Alajalen |
| Suha | Alajmi |
| Turki | Alajmi |
| Felix | Alakaloko |
| Deem | Alakeel |
| Amira | Alakhdury |
| Mohamed | Alaktaa |
| DY | Alalawi |
| Y | Alalawi |
| Yousef | Alalawi |
| Ahmad M | AlAli |
| Mohammed | Alali |
| Azhar | Alam |
| Junaid | Alam |
| Mahmood | Alam |
| Muhammad | Alam |
| R | Alam |
| Ruhina | Alam |
| Walid | Alame |
| H | Alameen |
| Hind | Alameen |
| S | Alameen |
| E | AlAmeer |
| Ehab | Alameer |
| A | Alamin |
| Samer | Alammari |
| Ahmed | Alamri |
| O | Alamri |
| Ossama | Alamri |
| Gary | Alan Bass |
| Zehra | Alan köylü |
| F | Alanazi |
| A | Alanbuki |
| Omar | Alannaz |
| M | Alansary |
| Khalid | Alaqeely |
| Sara | Alaqel |
| A | Alarabi |
| Alarabi | Alarabi |
| R | Alarabi |
| Rehab | Alarabi |
| Zuhair | Alaradi |
| Isaias | Alarcón |
| VD | Alarcón Vela |
| S | Alarood |
| Salameh | Alarood |
| Raquel | Alarza |
| Ala | Alasadi |
| Sabreen | ALashmali |
| Datonye | Alasia |
| A | Alasmari |
| Mohammed | Alasmari |
| Mohammad | Alassaf |
| Ali | Alassiri |
| Blanca | Alastrue Giner |
| Abdullah | Alatar |
| Mohammed | Alateeq |
| Zainab | Alattas |
| Abdulbari | Alawadhi |
| Khalid | Alawadi |
| Huda | Alawami |
| Mohammed | Alawami |
| Ahmed | Alawi |
| K | Alawi |
| Khalil | Alawi |
| BOF | Alawneh |
| F | Alawneh |
| Fade | Alawneh |
| Y | Alawneh |
| Yazan | Alawneh |
| Mohammed | Alayan |
| Barnabas | Alayande |
| A | Alayed |
| Ahmad | Alayed |
| Nada | Alayed |
| Hala | Alayyoubi |
| Emad | Alazab |
| Basma | Alazabi |
| M | Alazabi |
| D | Alazawi |
| O | Alazki |
| Ghaleb | Alazzeh |
| Jr | Alba |
| Basil | Albaba |
| Ismail | Albadawi |
| I | Albader |
| Ibtisam | Albader |
| MA S | Albader |
| Zamzam | Albadi |
| Obey | Albaini |
| Adel | Albaiti |
| Ibrahim | Albakry |
| Erminia | Albanese |
| F | Albanesi |
| A | Albani Forneris |
| Agustin | Albani Forneris |
| MEH | Albanna |
| Konstantinos | Albanopoulos |
| F | Albaqami |
| HM | Albar |
| MN | Albaraesi |
| Ali | Albargawi |
| AA | Albaroudi |
| Antonio | Albarracín Marín Blázquez |
| Abdullah | Albarrak |
| Majed | Albarrak |
| Mohammad | Albasheer |
| Aysha | Albastaki |
| Mariam | Albatoul Nageh |
| Nof | Albawardy |
| A | Albdah |
| Abdullah | Albdah |
| M | Albendary |
| Mohamed | Albendary |
| Igor | Alberdi San Roman |
| Laura | Alberici |
| Abdelrahman | Alberkamy |
| Kim | Albers |
| Mara | Albert Fort |
| Julius | Albert Sugianto |
| D | Alberti |
| José | Alberto Atristain Pesquera |
| Jairo | Alberto Dussan-Sarria |
| Julio | Alberto Gobernado Tejedor |
| Antonio | Alberto Martinez |
| Carlo | Alberto Pacilio |
| Ramon | Alberto Ramos |
| Luis | Alberto Reyes Figueroa |
| Jose | Alberto Rojo López |
| Guillermo | Alberto Sarmiento Ramirez |
| Cesar | Alberto Vergel Cabrera |
| M | Albertsmeier |
| Markus | Albertsmeier |
| Markus | Albertsmeiers |
| B | Albi Martin |
| V | Albino |
| Nouran | Albishty |
| Majid | Alborzi |
| A | Alburakan |
| Ahmed | Alburakan |
| Bader | Alburayh |
| Hanadi | AlBusaidi |
| Asem | Albzzaz |
| F | Alcaide Matas |
| Fernando | Alcaide Matas |
| Ignacio | Alcalá Rueda |
| Marta | Alcaraz Fuentes |
| Luis | Alcides García Barrionuevo |
| F | Alconchel |
| Felipe | Alconchel |
| Khalid | Aldaghiri |
| Homoud | AlDahash |
| M | Aldaher |
| Mohamed | Aldahma |
| M | Aldakheel |
| Ahmad | Aldakhil |
| Lateefa | Aldakhyel |
| Abdulrahman | Aldakkan |
| Shuaib | Aldalal |
| Joel | Aldana |
| M | Aldawbali |
| H | Aldawoody |
| Wassim | Aldebeyan |
| C | Aldecoa |
| Saif | Aldeen Al Dwairi |
| Ala | aldeen Hasan |
| Hossam | Aldein S |
| A | Alderazi |
| Amer | Alderazi |
| M | Alderuccio |
| Naif | Aldhaam |
| Amirah | Aldhurais |
| Ehab | Aldlyami |
| Jose | Aldo Guzman Barba |
| J | Aldoori |
| W | Aldressi |
| Wafa | Aldressi |
| K | Aldridge |
| Kerrie | Aldridge |
| Manar | Aldubaai |
| Fozan | Aldulaijan |
| Hamdan | Aldumaini |
| Omolabake | Ale |
| Anna | Alecci |
| Muhammad | Aleem |
| NM | Alegria Navarrete |
| Maryam | ALeissa |
| Gabriela | Alejandra Buerba |
| María | Alejandra Caicedo Giraldo |
| María | Alejandra De León Lima |
| Maria | Alejandra Giraldo |
| Diana | Alejandra Pantoja Pachajoa |
| Claudia | Alejandra Rivas Torres |
| María | Alejandra Torrado Varón |
| María | Alejandra Wagner Useche |
| Daniel | Alejandro Donoso Pizarro |
| Cristians | Alejandro Gonzalez |
| Jose | Alejandro Mata |
| David | Alejandro Mejia |
| Oscar | Alejandro Sánchez García |
| Camilo | Alejandro Velandia Sánchez |
| Sergio | Alejandro Villeda |
| Evgeniya | Aleksandrova |
| L | Aleksić |
| Lidija | Aleksić |
| Aisha | Alelwany |
| María | Alemán |
| G | Alemanno |
| Giovanni | Alemanno |
| M | Alemrajabi |
| Berhanu | Alemu |
| Megersa | Alemu |
| Verónica | Alen Villamayor |
| Jurij | Aleš Košir |
| Abdulkarim | Alesmail |
| Angelo | Alessandro Marra |
| Cosimo | Alex Leo |
| Nicholas | Alexakis |
| Dinesh | Alexander |
| Golomidov | Alexander |
| ME | Alexander |
| Philip | Alexander |
| Tamara | Alexander |
| Thomas | Alexander |
| Edison | Alexander Benavides Hernández |
| Jhon | Alexander Hoyos Castro |
| Israel | Alexander Ostrovsky |
| Dominik | Alexander Ratiu |
| H | Alexander-Leon |
| María | Alexandra Heras Garceau |
| Josephine | Alexandra Lim |
| María | Alexandra Pesántez Peralta |
| Lisbeth | Alexandra Urueña Pinzon |
| Maria | Alexandra Velicu |
| Andreas | Alexandrou |
| Vlad | Alexandru Gata |
| Vlad | Alexe |
| M | Alexeev |
| Jan | Alexeis Lacuata |
| V | Alexoudi |
| BahaUldin | Alezabi |
| J | Alfaifi |
| A | AlFakhri |
| Abdullah | AlFakhri |
| Mohammad | Alfarah |
| O | Alfarhan |
| Hilda | Alfaro |
| A | Alfaro-Goldaracena |
| Alejandro | Alfaro-Goldaracena |
| Dina | Alfarra |
| Mohamed | Alfatih Hamza |
| Fatema | Alfayez |
| Ahmed | Alfeqeeh |
| Abdallah | Alferdaus |
| Alex | Alfieri |
| S | Alfieri |
| Sergio | Alfieri |
| R | Alfkey |
| JP | Alfonso |
| Danilo | Alfonso Arévalo Sandoval |
| Carolina | Alfonso Carrillo |
| Diego | Alfonso Paiva Vera |
| Stephanie | Alford |
| Doaa | Alfraidy |
| Luis | Alfredo Betances |
| José | Alfredo Calderón Arancibia |
| Diego | Alfredo Palta Uribe |
| José | Alfredo Pérez Meave |
| Raymundo | Alfredo Pérez Uribe |
| Helmut | Alfredo Segovia Lohse |
| M | Alfuqaha |
| A | Älgå |
| Andreas | Älgå |
| Noran | Algadi |
| Barbara | Algar-Yañez |
| M | Algarni |
| Mohammed | Algarni |
| Saad | Algarni |
| A | Alghamdi |
| Abdulaziz | Alghamdi |
| Abdullah A | Alghamdi |
| Ahmad | Alghamdi |
| Ibtihal | Alghamdi |
| R | Alghamdi |
| Rami | Alghamdi |
| Saleh | ALghamdi |
| Susan | Alghamdi |
| Thabet | Alghazal |
| Abdallah | Alghazo |
| A | Alghuliga |
| Lolowah | Alghuson |
| Azuolas | Algimantas Kaminskas |
| Sultan | Alhabdan |
| Marwah | AlHADAD |
| Amani | Alhaddad |
| W | Alhaddad |
| Wafa | Alhaddad |
| Ahmed | Alhadeethi |
| Abdulmueti | Alhadi |
| Albrra | Alhag |
| Ammar | Alhaidari |
| F | Alhajami |
| Awatif | Alhaje |
| Baba | Alhaji Bin Alhassan |
| Zahrah | Alhajji |
| Naser | AlHajri |
| Nuraddin | Alhakami |
| Hani | Alhalal |
| A | Alhamed |
| Ahmad | Alhamid |
| Aos | Alhamid |
| Mohammad | Alhamid |
| Othman | Alhammad |
| Tariq | Alhammali |
| Abdelrahman | AlHarazi |
| A | Alharbi |
| Bandar | Alharthi |
| M | Alharthi |
| Mohammed | Alharthi |
| Nawaf | Alharthi |
| R | Alharthi |
| Sultan | Alharthi |
| Hasan | Alhasan |
| Aya | ALhassan |
| Basmah | Alhassan |
| Turki | Alhassoun |
| Naif | Alhathal |
| Majd | Alhattab |
| M | Alhawatmeh |
| Mohammad | Alhawatmeh |
| Alaa | Alhazmi |
| Barrag | Alhazmi |
| Norah | Alhazzaa |
| A | Alhefdhi |
| Amal | Alhefdhi |
| Boshra | Alhelal |
| Fahad | Alhelal |
| Nawal | Alhemyari |
| Ameen | Alherabi |
| Rahaf | Alhindi |
| Mofarej | Alhogbani |
| A | Alhojaili |
| R | Alhossaini |
| Rana | Alhossaini |
| Tarek | Alhouni |
| A | Alhouri |
| Ahmad | Alhouri |
| S | Alhudhairy |
| Adnan | ALhumaida |
| Omar | Alhunaidi |
| Dhyia | Alhuq Al-surimi |
| Ali | Alhussaini |
| Ahmad | Alhussein |
| Meshari | Alhuthayl |
| A | Ali |
| Ahmed | Ali |
| Ahsan | Ali |
| AK | Ali |
| Almigdad | Ali |
| Ammar | Ali |
| Amna | Ali |
| Aneeqa | Ali |
| Aoun | Ali |
| Danish | Ali |
| Douaa | Ali |
| EE | Ali |
| F | Ali |
| Faizah | Ali |
| Ghazanfar | Ali |
| H | Ali |
| HB | Ali |
| Ibrahim | Ali |
| Imran | Ali |
| Irfan | Ali |
| J | Ali |
| K | Ali |
| L | Ali |
| M | Ali |
| Marah | Ali |
| Maria | Ali |
| Mehboob | Ali |
| Mohamed | Ali |
| Mostafa | Ali |
| Muhammad | Ali |
| N | Ali |
| Noman | Ali |
| Roshneen | Ali |
| S | Ali |
| Sadaf | Ali |
| Salem | Ali |
| Samar | Ali |
| Sana | Ali |
| SM | Ali |
| Wagdi | Ali |
| Yakubu | Ali |
| Sani | Ali Aji |
| Amir | Ali Akbari |
| Imran | ali Ali |
| Majdi | Ali Alqudah |
| Luqman | Ali Bajwa |
| Christian | Ali Buesaquillo |
| A | Ali deeb |
| Muhammad | Ali Ghufran |
| Hossam | Ali Hadiya |
| AA | Ali Karar |
| Asad | Ali Kerawala |
| Mehmet | Ali Koç |
| Farman | Ali Laghari |
| Mohamed | Ali Mohamed |
| Abubakr | Ali Mohammed Alhassan Humidan |
| Mohamed | Ali Ossman |
| Saad | Ali Saad Salama |
| Ismail | Ali Saleh |
| Faaiz | Ali Shah |
| Shafqat | Ali Shaikh |
| M | Aliaga-Ramos |
| Alfonso | Aliaga-Sanchez |
| Alfonso | Alias |
| A | Aliev |
| Mehmet | Alim Turgut |
| A | Alinaghi Langari |
| Oleg | Aliosin |
| Badra | Aliou Kone |
| Mir | Alireza Hoda |
| German | Alirio Tovar |
| Halil | Alis |
| A | Alissa |
| M | Aliwa |
| Mohamed | Aliwa |
| Gunay | Aliyeva |
| Z | Aliyeva |
| Zumrud | Aliyeva |
| Ibrahim | Aliyu Mukhtar |
| Narges | Alizadeh |
| A | Aljaafreh |
| Alaa | Aljabali |
| F | Aljaber |
| Maniee | Aljabri |
| Noor | Aljabri |
| Abdulmalek | Aljafari |
| Abdulmoiz | Aljafari |
| Anas | Aljaiuossi |
| Mohammed | Aljanabi |
| F | Aljanadi |
| Firas | Aljanadi |
| Asma | Aljanfi |
| Ramez | Aljasem |
| Fawzi | Aljassir |
| Abbas | Aljebur |
| Ali | Aljewaied |
| A | Aljiffri |
| Murad | Aljiffry |
| Alia | Aljifri |
| R | Aljohani |
| Adil | Aljohari |
| Lylas | Aljohmani |
| Ayat A | Aljuba |
| Roaa | Aljunaidi |
| Abdullah | Aljunaydil |
| Abdulaziz | Aljurayyan |
| Omar | Aljuroushi |
| Ali | Aljuzair |
| N | Alkaabi |
| A | Alkabli |
| G | Alkadeeki |
| Ghadah | Alkadeeki |
| Maher | Alkahal |
| Mohammed | Alkahlan |
| AbdulAziz | AlKanhal |
| Samer | Alkarak |
| Safya | Alkarky |
| A | Alkaseek |
| Akram | Alkaseek |
| Soliman | Alkassem |
| Ola | Alkasser |
| Abdullah | Alkassim |
| Hani | Alkattan |
| Muhannad | Alkazrooni |
| M | Alkchr |
| Hana | Alkeelani |
| Ahmad | Alkhaledi |
| Wael | Alkhaleel |
| Ahmed | Alkhalifah |
| Nawar | Alkhamesi |
| Ahmed | ALKhamis |
| Jumaa | Alkhamis |
| Waleed H | Alkhamis |
| Omar | Alkhanbashi |
| Nour | Alkhanji |
| Nadiya | Alkharousi |
| Al-Salt | Alkharusi |
| Suhad | Alkhateb |
| Abdulrahman | Alkhatib |
| Ahmad | Alkhatib |
| M | Alkhatieb |
| Maram | Alkhatieb |
| Wafa | Alkhayal |
| Saud | Alkhayrat |
| Ali | Alkhdor |
| A | Alkhuzaie |
| Khadija | ALKiyumi |
| N | Alkreedees |
| MM | Alkurdieh |
| Rauoof | Alkuwafi |
| Hasbi | Allah Amin |
| Abdallah R | Allam |
| Mohamed | Allam |
| AY | Allan |
| Jennifer | Allan |
| A | Allana |
| Carola | Allemand |
| Calisha | Allen |
| Laura | Allen |
| M | Allen |
| S | Allen |
| Jc | Allen Ingabire |
| Jakob | Allerstorfer |
| J | Allison |
| M | Allison |
| Caterina | Allmer |
| A | Alloush |
| Mohammad | Allouzi |
| Marta | Allue |
| Abdulaziz | Almaawi |
| Ydyrys | Almabayev |
| H | Almabrouk |
| Hadeel | Almadani |
| Mahmoud | Almaghrabi |
| Asem | Almaghrebi |
| Noof | AlMaharbi |
| M | Almahroush |
| Ali | Almalaq |
| Osama | Almalik |
| Ahmad | Almalki |
| Saeed | Almalki |
| Nora | Almana |
| Raed | Almannie |
| Maha | Almansour |
| Khuloud | Almaqrahi |
| Hassan | Almarashi |
| Fayez | Almari |
| Felwa | AlMarshad |
| Amal | Almasri |
| M | Almasri |
| Murad | Almasri |
| Ashraf | Almatar |
| Bikheet | Almatar |
| Mohammed | Almatrafi |
| Sulaiman | Almazeedi |
| W | Almdallal |
| Adriana | Almeciga |
| A | Almeida |
| AC | Almeida |
| Alexandre | Almeida |
| JI | Almeida |
| Mafalda | Almeida |
| João | Almeida Pinto |
| Juliana | Almeida Rego |
| J | Almeida-Pinto |
| R | Almeida-Reis |
| Rui | Almeida-Reis |
| Meshal | Almeshal |
| Razan | Almesned |
| Heyam | Almezghwi |
| N | Almgla |
| Naser | Almgla |
| A | Almhmadi |
| Sari | Almiani |
| Bushray | Almiqlash |
| Teresa | Almiron |
| Abdulrahman | Almjersah |
| A | Almofarreh |
| Jorge | Almoguera |
| Khalid | Almohaimeed |
| Zuhoor | Almohanady |
| Hadi | Almohsen |
| M | Almond |
| C | Almondo |
| Abd | Almonem Shaikh Ahmad |
| Noora | Almoosa |
| Jaime Jr | Almora |
| Amira | Almosa |
| Eman | Almotairi |
| H | Almoumani |
| Abdullah | Almousa |
| Aya | Almoustafa |
| Abdulrahman | Almuawi |
| Saif | Almudares |
| Abdullah | Almufarrih |
| AAY | Almugaddami |
| Ayman | Almugaddami |
| Abddulrahman | Almulhim |
| AS | Almulhim |
| Ahmad | Almulla |
| Abdullah | Almunifi |
| Majed | Almuraee |
| Fatema | Almushawah |
| A | Almutairi |
| Hanan | Almutairi |
| Abdulrahman | Almutawa |
| Hisham | Almutawa |
| S | Almutrafi |
| Omar | Alnachoukati |
| Ahmed | Alnaeem |
| Hareth | Alnahr |
| Manal | Alnaimi |
| Zahra | Alnajem |
| Louy | Alnajjar |
| Tareq | Alnajjar |
| AQ | Alnami |
| Mohammed | Alnamshan |
| Mohamed | alnaser Alnehum |
| Mahmoud | Alnasser |
| Mohammed | Alnasser |
| AK | Alnemare |
| M | Alnemary |
| Omar | Alneser |
| Mohammad | Alnoaiji |
| K | Alnwijy |
| Suliman | Alobaid |
| S | Alobaysi |
| Saad | Alobaysi |
| Marwa | ALodine |
| Alanoud | Alomair |
| Abdulaziz | Alomar |
| Amar | Alomar |
| O | Alomar |
| Osama | Alomar |
| Soha | Alomar |
| Marta | Alomar Bofill |
| Faris | Alomran |
| Hadeel | AlOmran |
| Nivaldo | Alonso |
| Santiago | Alonso Bartolomé |
| N | Alonso de la Fuente |
| Marta | Alonso Fernández |
| Erick | Alonso González-García de Rojas |
| Jairo | Alonso Hernandez |
| Miguel | Alonso Juarranz |
| V | Alonso Mendoza |
| Veronica | Alonso Mendoza |
| P | Alonso Ortuño |
| A | Alonso Poza |
| Isabel | Alonso Sebastian |
| L | Alonso-Lamberti |
| Hadel | Alosta |
| Ahmed | AlOtaibi |
| Haifa | Alotaibi |
| M | Alotaibi |
| Naif H | Alotaibi |
| M | Aloulou |
| Mohammad | Aloulou |
| J | Alowais |
| Jalal | Alowais |
| Ous | Alozairi |
| Nuri | Alper Sahbaz |
| N | Alpert |
| Poppy | Alport |
| A | Alqaarh |
| A | Alqabasani |
| Abdulmajeed | AlQahtani |
| Awadh | Alqahtani |
| Bandar | Alqahtani |
| Fahad | Alqahtani |
| Loai | Alqahtani |
| Moraya | Alqahtani |
| S | Alqahtani |
| Saad M | Alqahtani |
| Lina | AlQalisi |
| A | Alqallaf |
| M | Alqannas |
| Mashhour | Alqannas |
| Abdulellah | Alqarni |
| Saad | Alqarni |
| S | Alqasem |
| Saad | Alqasem |
| Nooraldin | Alqasemi |
| Hussain | Alqaser |
| Mohammed | Alqassab |
| Abdullah | Alqattan |
| Shatha | Alqawasmi |
| M | Alqedrh |
| Mohannad | Alqedrh |
| Maitha | Alqemzi |
| Shahad | Alqreen |
| Sadeel | Alqudah |
| Basma | Alqudaimi |
| D | Alqunaibit |
| Dalia | Alqunaibit |
| Aya | Alqurpaa |
| Mera | Alrabadi |
| Murtagi | Alraboui |
| Qutaiba | Alradawneh |
| M | Alrahawy |
| Reem | AlRakaf |
| M | Alramadhan |
| M | Alrashed |
| Muath | Alrashed |
| B | Alrayes |
| Bourhan | Alrayes |
| Mohammed | AlRayih |
| F | Alresaini |
| Fay | Alresaini |
| Mohammed | Alreshidan |
| Mohammed | Alrezami |
| Omar | Alrifai |
| Lolwah | Alriyees |
| Meaad | Alromaihi |
| Sarah | Alrubaish |
| Noorsabah | Alrubays |
| Sara | Alsaad |
| H | Alsaadi |
| Hayder | Alsaadi |
| Sabaa | Alsaadi |
| Shatha | AlSAAFIN |
| Salman | AlSabah |
| T | Alsabahi |
| Tareq | Alsabahi |
| Mohammed | Alsabri |
| Norah | Alsabty |
| Ahmed | ALsadek |
| Mohammed | Alsadiq |
| Wael | Alsado |
| Sara | Alsaeiti |
| Maryam | Alsafi |
| Sameh S | Alsafty |
| Abdulrahman | Alsaggaf |
| Yasir | Alsagoor |
| Jawaher | Alsahabi |
| N | Alsahan |
| Abdulhakim | Alsaiad |
| Bayan | Alsaid |
| Abdulaziz | Alsaif |
| Laith | AlSaket |
| Musab | Alsakka |
| M | Alsakkaf |
| Mazen | Alsakkaf |
| Radfan | Alsalal |
| Ree M | Alsalamah |
| Khaled | Alsaleh |
| Mohammad | Alsaleh |
| Nuha | Alsaleh |
| AlHanouf | Alsaloom |
| Alanoud | Alsamari |
| Hisham | Alsanawi |
| O | Alsaraireh |
| M | Alsayadi |
| Musaed | Alsayadi |
| R | Alsayadi |
| Ramzi | Alsayadi |
| AB | Alsayed |
| Yazeed | Alsebayel |
| Nahar | Alselaim |
| Muhannad | Alsemari |
| O | Alser |
| Osaid | Alser |
| M | Alshaar |
| Muhammad | Alshaar |
| A | Alshahrani |
| M | Alshahrani |
| Mubarak | Alshahrani |
| Mushabab | Alshahrani |
| S | Alshahrani |
| Jaffar | Alshahri |
| B | Alshaikh |
| Khaled | Alshaikh |
| Reem | Alshaipani |
| Omar | AlShakhshir |
| M | Alshalhoub |
| Artefaa | Alshamari |
| Abdulaziz | Alshammari |
| Nouf | Alshammari |
| Sulaiman | Alshammari |
| Turki | Alshammari |
| M | Alshamsi |
| S | Alshanafey |
| Saud | Alshanafey |
| Qutaiba | Alshannaq |
| Rana | Alshara |
| Ebrahim | Alsharabi |
| Mohamed | Alsharedi |
| E | Alshareea |
| Entisar | Alshareea |
| Khayriyah | Alshareef |
| Haneen | Alshargabi |
| Mokhtar | Alshargabi |
| F | Alsharif |
| M | Alsharif |
| Nasser | Alsharif |
| Marwa | Alsharji |
| Fadi | Alshawared |
| Ibrahim | Alshaygy |
| M | Alshehari |
| Fatma | AlShehhi |
| A | Alshehri |
| Abdulmajeed | AlShehri |
| Ameen | Alshehri |
| Khalid | Alshehri |
| M | Alshehri |
| Mohammed | Alshehri |
| Yasir | AlShehri |
| A | Alsheikh |
| Sara | Alshekh |
| A | Alshitwi |
| Mahmoud | Alshourman |
| S | Alshryda |
| Sattar | Alshryda |
| Sareyah | Alsibai |
| Mohanad | Alsidig |
| Saif | Alsobhi |
| Zeyad | AlSolami |
| Ali | Alsoudani |
| A | Alsoufi |
| K | Alsowaina |
| Fahd | AlSubaie |
| K | Alsubaie |
| Norah | Alsubaie |
| A | Alsuhaibani |
| Youssuf | AlSuhaibani |
| Rima | Alsulaiman |
| Samir | Alsulaimani |
| Thuwaiba | Alsulaimani |
| Yazeed | Alsuliman |
| Afnan | Alsultan |
| Thuraya | AlSumai |
| Zaid | Alsunna |
| A | Alsuradi |
| Munir | Alsuwaimel |
| Saleh | Alsuwaydani |
| Suhir | Alsuwiyah |
| Abdulmalik | Altaf |
| Kiran | Altaf |
| Zahra | Altaf |
| T | Altahan |
| Talal | Altahan |
| Assma | Altaher |
| Bassam | Altalhi |
| Jaime | Altamirano-Villarroel |
| Cristian | Altana |
| O | Altarhoni |
| A | Althobaiti |
| Awwadh | Althobaiti |
| W | Althobaiti |
| Waleed | Althobaiti |
| Ibrahim | Althubaiti |
| A | Althumairi |
| Azah | Althumairi |
| Gaia | Altieri |
| Y | Altinel |
| Yuksel | Altinel |
| F | Altintoprak |
| Fatih | Altintoprak |
| A | Altobal |
| Ahmed | Altobal |
| Abdulmajeed | Altoijry |
| Roula | Altom |
| DF | Altomare |
| D | Altun |
| Abdulrahman | Alturki |
| Mohammad | Altuwaijri |
| Talal | Altuwaijri |
| Basmah | Altuwayjiri |
| Ikhlass | Altwejri |
| Antonio | Alvarado |
| Rosalinda | Alvarado |
| Vítor | Alvarenga |
| OV | Alvarenga Pereira |
| Andres | Alvarez |
| E | Alvarez |
| Estibaliz | Alvarez |
| FA | Alvarez |
| MR | Alvarez |
| Sofia | Álvarez |
| Javier | Alvarez Gama |
| Alfonso | Alvarez Manilla Orendain |
| Yicel | Alvarez Martinez |
| JA | Alvarez Nufio |
| Iago | Alvarez Saez |
| Eva | Alvarez Torres |
| FE | Alvarez-Bautista |
| Mario | Alvarez-Gallego |
| Fernanda | Alves |
| Paulo | Alves |
| R | Alves |
| Ricardo | Alves |
| Rubem | Alves Da Silva Neto |
| Rubem | Alves Silva Junior |
| A | Alvi |
| Kashif | Alvi |
| Abdullah | Alwabari |
| Muath | Alwabel |
| DAH | Alwadani |
| A | Alwadiya |
| Athari | Alwael |
| Saba | Alwahedy |
| Mustafa | Alward |
| MM | Alwarfalli |
| M | Alwash |
| Mohammed | Alwashahi |
| Ian | Alwayn |
| Nasser | Alwehaibi |
| Mohamed | Aly |
| Hussain | Alyafii |
| Reem | Alyahya |
| A | Alyami |
| Ali | Alyami |
| Alwaleed | Alyami |
| Hamad | Alyami |
| M | Alyami |
| Mohammad | Alyami |
| M | Alyazidi |
| Mohammed | Alyousef |
| Ana | Alyra Carvalho |
| Mohammad | AlZaatreh |
| Adil | Alzadjali |
| Maen | Alzaeem |
| Ali | Alzahir |
| Fatma | Alzahraa Gamal |
| A | Alzahrani |
| Abdullah | Alzahrani |
| Abdulrahman | Alzahrani |
| Ahmed | Alzahrani |
| Meshari | Alzahrani |
| Mohammed A | Alzahrani |
| Mosa | Alzahrani |
| Saud | Alzahrani |
| Mamdouh | Alzaibak |
| M | Alzamanan |
| Mahdi | Alzamanan |
| Mohammed | Alzamanan |
| Khaled | AlZamel |
| Motasem | Alzaqh |
| N | Alzarooni |
| Ahmad | Alzedam |
| Hussam I A | Alzeerelhouseini |
| N | Alzerwi |
| Nasser | Alzerwi |
| A | Alzetani |
| Yasmeen | Alzghoul |
| Gmaan | Alzhrani |
| B | Alzomaili |
| Mai | Alzoubi |
| Malak | Alzoubi |
| A | Alzouhir |
| Sayel H | Alzraikat |
| Mutaz | Alzubi |
| R | Alzubi |
| Yazan | Alzubi |
| Tareq | Alzughayyar |
| Ahmed | Alzughoul |
| Abduljabbar | Alzuhair |
| M | Alzwei |
| Faiyazudin | Amado Ibrahim |
| Munira | Amadu |
| Mohammad | Amaireh |
| Emma | Amal Nahal |
| Nur | Amalina Che Din |
| Mudesir | Aman |
| Yuki | Amano |
| MJ | Amaral |
| C | Amarante Dias |
| Yenuksha | Amarasena |
| Abrham | Amare Tesfa |
| Justin Z | Amarin |
| Ghandi | Amayreh |
| A | Amazouzi |
| Maricely | Ambar Perez Fernandez |
| GK | Ambler |
| Marijus | Ambrazevicius |
| Gabriela | Ambriz Gonzalez |
| A | Ambrosi |
| Pantelis | Amditis |
| Abdur-rafee | Ameen |
| P | Ameerally |
| Ibrahim | Amer |
| Mostafa | Amer |
| Francesco | Amico |
| D | Amin |
| Dina | Amin |
| H | Amin |
| Mohamed | Amin |
| Sana | Amin |
| Shehzadi | Amin |
| Shivang | Amin |
| Verda | Amin |
| Vishal | Amin |
| Mhd | Amin Alzabibi |
| Mohamed | Amin Bakr |
| N | Amin Sahid |
| Mohammed | Amir |
| Doha | Amir AtaAlmanan |
| Malaz | Amir Ataalmanan |
| Tebyan | Amir AtaAlmanan |
| Seyed | Amir Javadi |
| Ahmad | Amir Kayali |
| Mohamed | Amir Mrad |
| G | Amira |
| Gamal | Amira |
| Nicolas | Amisi |
| Zarafshan | Amjid |
| Ahmed | Ammar |
| AS | Ammar |
| Khaled | Ammar |
| Imane | Ammouze |
| Mohamed | Amnaina |
| H | Amo |
| Kwabena | Amo-Antwi |
| George | Amoah |
| Michael | Amoah |
| Joachim | Amoako |
| Yaw A | Amoako |
| S | Amoako Asirifi |
| Mabel | Amoako-Boateng |
| Ricci | Amoils |
| E | Amorim |
| Edgar | Amorim |
| JE | Amorim |
| Jorge | Amorim |
| Robson | Amorim |
| L | Amorim Braz |
| Tatiane | Amorim Coelho |
| Happy | Amos |
| Hoora | Amouzegar |
| Regina | Amparo Ugarte Oscco |
| L | Ampollini |
| Mathew | Amprayil |
| MC | Ampuan |
| Sultan | Amrayev |
| A | Amro |
| Adham | Amro |
| Sarah | Amro |
| E | Amzallag |
| Vinna | An |
| Khoirul | Anam |
| Akshay | Anand |
| P | Anand |
| Premkumar | Anandan |
| G | Anania |
| Sankar | Ananth |
| R | Anantha |
| Dulce | Añasco |
| M | Anastasakis |
| A | Anastasi |
| Alessandro | Anastasi |
| Jose | Anatolio Resendiz |
| Ernesto | Anaya |
| DM | Añazco Mareco |
| F | Anazor |
| FC | Anazor |
| Fitzgerald | Anazor |
| T | Andabaka |
| Carmela | Andal |
| Veronica | Andaya |
| Jon | Ander Lizarbe |
| Kajsa | Anderin |
| Megan | Anders |
| C | Anderson |
| Ikenna | Anderson Aneke |
| H | Andersson |
| Henrik | Andersson |
| Chihiro | Ando |
| Kohei | Ando |
| Tadao | Ando |
| Enrico | Andolfi |
| FA | Andrabi |
| A | Andrade |
| Arnulfo | Andrade |
| Marlene | Andrade |
| R | Andrade |
| Francisco | Andrade González |
| RP | Andrade Salinas |
| TO | Andraschofsky |
| Benoît | André |
| M | Andrea |
| Vito | Andrea Capozzi |
| Viviana | Andrea Hernández Angel |
| Jhoana | Andrea Murillo Castellanos |
| Natalia | Andrea Rivera Rincón |
| Paola | Andrea Tabares Romero |
| L | Andreani |
| Lorenzo | Andreani |
| SM | Andreani |
| Stefano | Andreani |
| D | Andreas |
| V | Andreasi |
| Erika | Andreatta |
| Predrag | Andrejevic |
| P | Andreoni |
| Isis | Andreotti |
| Fernando | Andres Alvarez |
| Paulo | Andrés Cabrera Rivera |
| Jose | Andres Calvache |
| Carlos | Andrés Carvajal Fierro |
| José | Andrés Cifuentes Rodenas |
| Carlos | Andres Colunga Tinajero |
| Oscar | Andres Escobar Vidarte |
| Patricio | Andrés Freile Pazmiño |
| Ainhoa | Andres Imaz |
| Carlos | Andres Marulanda Toro |
| Ricardo | Andres Niño Corredor |
| Camilo | Andrés Polanía Sandoval |
| Henry | Andrés Rodríguez |
| Conrado | Andrés Ros |
| Eva | Andreu Riobello |
| J | Andreuccetti |
| Thomas | Andrew Maccabe |
| Emmet | Andrews |
| Kiah | Andrews |
| Liantsoa | Andriamanana |
| Alexandros | Andrianakis |
| Herimampionona E | Andriantsoa |
| C | Andro |
| Christophe | Andro |
| A | Andronic |
| Farah | Androus |
| Iván | Andújar Lara |
| IA | Aneke |
| Alexandre | Anesi |
| Katerina | Anesti |
| E | Anestiadou |
| E | Ang |
| K | Ang |
| Keng-Leong | Ang |
| W | Ang |
| Wei-Wen | Ang |
| Z | Ang |
| N | Angamuthu |
| Carol | Angel |
| J | Ángel |
| Miguel | Angel Alonso Prieto |
| Miguel | Angel Calderon-Llamas |
| Jose | Ángel Diez Ares |
| Miguel | Angel Freiria Eiras |
| Miguel | Angel García García |
| Miguel | Ángel García Ureña |
| Miguel | Ángel Gordo Vega Gordo-Vega |
| Miguel | Angel Hernandez Bartolome |
| Miguel | Angel Jimenez Botello |
| Miguel | Angel Martin-Ferrero |
| Miguel | Ángel Mercado |
| Roberto | Ángel Núñez-González |
| Luis | Angel Suarez Gonzalez |
| Miguel | angel Zavala gonzalez |
| Maria | Angela Dealino |
| Elisa | Angela Diego-Alonso |
| Estefany | Angela Flores Anaya |
| Stefania | Angela Piccioni |
| María | Ángeles Gascón Domínguez |
| Maria | Angelica Arada |
| María | Angelica Cendales |
| R | Angelico |
| Roberta | Angelico |
| Sophia | Angelides |
| Fragkiskos | Angelis |
| Carlo | Angelo Cajucom |
| Erino | Angelo Rendina |
| Luigi | Angelo Vaira |
| Mario | Angelo Zamora |
| D | Angelou |
| Dimitrios | Angelou |
| K | Angelou |
| Kyveli | Angelou |
| Eva | Angenete |
| Rafael | Angerer |
| Reinhard | Angermann |
| F | Angles Crespo |
| Francesc | Angles Crespo |
| Raquel | Angulo Artal |
| Joshua | Anicetti |
| Hilmi | Anil Dincer |
| Olalekan | Anipole |
| C | Anis |
| Dayang | Anita Abdul Aziz |
| Dusabimana | Anitha |
| Laura | Aniukstyte |
| N | Anjarwalla |
| Naffis | Anjarwalla |
| M | Anjomrooz |
| Momina | Anjum |
| Waleed | Anjum |
| Mariam | anjum Ifthikar |
| sushil | Ankadavar |
| Jacob | Ankeny |
| Frank | Ankobea-Kokroe |
| Jo | Ann Chiu |
| Mary | Ann Johnson |
| Leigh | Ann O'Banion |
| Ju | Ann Tan |
| Patricia | Ann Uy |
| S | Annamalai |
| Nathaniel | Annan |
| Angeli | Anne Ang |
| Mary | Anne Carol Cueto |
| Daryl | Anne del Mundo |
| Sheela | Anne George Varayannoor |
| Shireen | Anne Nah |
| Jeryl | Anne Silvia Reyes |
| V | Annessi |
| J | Annett |
| A | Annicchiarico |
| Alfredo | Annicchiarico |
| Paolo | Annicchiarico |
| Rebecca | Anning |
| Filippo | Annino |
| Dominic | Annor Mintah |
| Willmar | Anoso |
| Philippe | Anract |
| Mehwish | Ansar |
| MM | Ansari |
| M | Ansarin |
| Mohssen | Ansarin |
| Muhammad | Ansary |
| N | Anscomb |
| George | Ansong |
| Zsuzsanna | Antal |
| M | Antar |
| Srdan | Ante Anzic |
| R | Anteby |
| Alaa | Anter |
| J | Anthoney |
| Dennis | Anthony Isah |
| Robert | Anthony Keenan |
| C | Anthoulakis |
| Christos | Anthoulakis |
| M | Anthuber |
| Matthias | Anthuber |
| A | Antic |
| Tina | Anto Menachery |
| Claudia | Anton |
| BT | Antón-Eguía |
| Filippo | Antonacci |
| Pantelis | Antonakis |
| A | Antonelli |
| B | Antonelli |
| K | Antoniadis |
| Morena | Antonilli |
| Marcelo | Antonini |
| Giuseppe | Antonino Pellicano |
| Codina | Antonio |
| Darienzo | Antonio |
| Luca | Antonio Aldrighetti |
| Jose | Antonio Carbonell Lopez |
| Francesco | Antonio Ciarleglio |
| Juan | Antonio Corralez Alvarez |
| Marco | Antonio Correa Guimaraes-Filho |
| Luis | Antonio Cuellar Martin |
| Caio | Antonio de Campos Prado |
| Marco | Antonio de la Rosa Abaroa |
| Jesús | Antonio Echavarría Uceta |
| José | Antonio Fernández-Dívar Sánchez |
| Jose | Antonio Gazo Martínez Gazo |
| Juan | Antonio González León |
| Carlos | Antonio Llanos Lucero |
| Gustavo | Antonio Martinez Estrada |
| José | Antonio Ortega-Jiménez |
| Pedro | Antonio Parra Baños |
| Luis | Antonio Pascua-Gómez |
| Vito | Antonio Piserchia |
| José | Antonio Posada |
| Jose | Antonio Salud |
| José | Antonio Sánchez Martínez |
| Marco | Antonio Zappa |
| Ingrid | Antonios |
| A | Antoniou |
| Afroditi | Antoniou |
| George A | Antoniou |
| Stavros A | Antoniou |
| Theofani | Antoniou |
| P | Antonogloudis |
| MI | Antonopoulou |
| Carmine | Antropoli |
| J | Antunes |
| Antonio | Antunes Rodrigues Junior |
| Christina | Antzaka |
| R | Anula |
| K | Anuszkiewicz |
| M | Anvari |
| Mehran | Anvari |
| S | Anwar |
| Sibtain | Anwar |
| SL | Anwar |
| Mariyah | Anwer |
| Rabia | Anwer |
| Lofty-John | Anyanwu |
| N | Anyaugo |
| Ngozi | Anyaugo |
| Solomon | Anyimba |
| David | Anyitey-Kokor |
| A | Anzak |
| Anam | Anzak |
| Alejandra | Anzures Mendoza |
| Nesrine | Aouabed |
| Hulrich | Aouagbe Behanzin |
| Salah | Aoun |
| SG | Aoun |
| AA | Apampa |
| Daniel | Aparicio Sánchez |
| Eduardo | Apellaniz |
| Olus | Api |
| Jeyakumar R | Apollos |
| JR | Apollos |
| Aya | Aposaeeda |
| C | Apostolou |
| Christos | Apostolou |
| K | Apostolou |
| Konstantinos | Apostolou |
| Enoch | Appiah |
| Adu | Appiah-kubi |
| Peter | Appiah-Thompson |
| J | Appleyard |
| Akhila | Appukuttan |
| Tedy | Apriawan |
| A | Aprile |
| Alessandra | Aprile |
| Vittorio | Aprile |
| IA | Apse |
| SS | Apte |
| Ahmed | Aqeelah |
| Muhammad | Aqib |
| Sabra | Aqil |
| F | Aquila |
| Laura | Aquino |
| Fatima | Arab |
| Khalid | Arab |
| N | Arab |
| Ebrahim | Arafa |
| EJ | Aragon Achig |
| J | Aragon-Chamizo |
| Juan | Aragón-Chamizo |
| Paula | Aragón-Ramos |
| R | Aram |
| G | Arampatzis |
| Iñigo | Arana |
| Francisca | Aranda Lozano |
| Roy | Arangoytia |
| Coro | Aranzabal Urrutia |
| Z | Aras |
| DH S | Araujo |
| Marcelo | Araujo |
| MS | Araujo |
| Asuvathan | Aravinthan |
| Hana | Arbab |
| Gill | Arbane |
| Jeric | Arbizo |
| J | Arboleda |
| Ricardo | Arceo-Olaiz |
| James | Archer |
| JE | Archer |
| Leigh | Archer |
| Daniel | Arco |
| Javier | Ardebol |
| Javier | Ardila-Montealegre |
| Antonella | Ardito |
| F | Ardito |
| Rohan | Ardley |
| Francisco | Ardura |
| L | Areias |
| LL | Areias |
| Alexander | Arekhandia |
| I | Aremu |
| II | Aremu |
| Isiaka | Aremu |
| M | Aremu |
| A | Arena |
| Alessandro | Arena |
| Octavio | Arencibia |
| G | Aresu |
| Mapuor | Areu |
| A | Arévalo Barreto |
| Alejandro | Arévalo Barreto |
| D | Argandykov |
| Giulio | Argenio |
| M | Argentou |
| M | Argo |
| Leah | Argus |
| Claudia | Arias |
| Fernando | Arias-Amézquita |
| Sarra | Aribi |
| Tomather | Aribi |
| C | Arican |
| Vittorio | Arici |
| Celso | Ariel Fernandez |
| P | Aries |
| Iqtaza | Arif |
| Numera | Arif |
| Salman | Arif |
| Wirsma | Arif Harahap |
| M | Arigoni |
| Hassan | Arishi |
| Abimbola | Ariyibi |
| Z | Arizavi |
| Parisa | Arjmand |
| Nikolaos | Arkadopoulos |
| S | Arkani |
| Y | Arkha |
| Yasser | Arkha |
| Lorenzo | Arlia |
| James | Arlidge |
| Edward | Arlu Dinoy |
| Ralph | Armah |
| Nii | Armah Adu-Aryee |
| Anuar | Armando Idrobo Escobar |
| T | Armao |
| Maria | Armas |
| FJ | Armas Zarate |
| Kunwar | Armash Ahsan |
| G | Armatura |
| Daniele | Armellin |
| Jan | Armelynn Santos |
| Mikel | Armendariz |
| S | Armentano |
| A | Arminio |
| Armando | Arminio |
| J | Armitage |
| Alison | Armstrong |
| Lara | Armstrong |
| Laurent | Arnalsteen |
| AY | Arnaout |
| I | Arnaout |
| Khaled | Arnaout |
| E | Arnaoutoglou |
| Eleni | Arnaoutoglou |
| A | Arnaud |
| Alexis | Arnaud |
| Alexis P | Arnaud |
| AP | Arnaud |
| Ingus | Arnolds Apse |
| Abayomi | Arogundade |
| Soliudeen | Arojuraye |
| Rajnish | Arora |
| Lorena | Arrabal |
| R | Arrangoiz |
| Rodrigo | Arrangoiz |
| Eduardo | Arrea Salto |
| Catalina | Arredondo Soto |
| MD | Arribas Del Amo |
| M | Arrieta |
| Mirentxu | Arrieta |
| Giulia | Arrigoni |
| Rodrigo | Arrivabeno |
| Roberto | Arroyave |
| A | Arroyo |
| Diego | Arroyo |
| Analia | Arrua |
| E | Arrue |
| Emmy | Arrue Del Cid |
| Mani | Arsalan |
| Daneyal | Arshad |
| Hajra | Arshad |
| M | Arshad |
| Muhammad | Arshad |
| Owais | Arshad |
| Kalbim | Arslan |
| Kemal | Arslan |
| Muhammad | Arslan |
| Nuhi | Arslani |
| E | Arteaga Cedeño |
| Miriam | Artés Artés |
| Mariano | Artés Caselles |
| Joshua | Arthur |
| B | Arthurs |
| Darwin | Artidoro Quispe-Cruz |
| Enrique | Artigues |
| Dmitri | Artioukh |
| F | Artukoglu |
| Evgeniy L | Artyushkov |
| Sandra | Aruachan Vesga |
| A | Arulanantham |
| Arulprashanth | Arulanantham |
| S | Arumugam |
| Sathyaseelan | Arumugam |
| M | Arumugasamy |
| Abhinav | Arun Sonkar |
| Kseniya | Arutyunyan |
| Shobhit | Arya |
| Teguh | Aryandono |
| Fachreza | Aryo Damara |
| Vasileios | Arzoglou |
| Y | As |
| Malke | Asaad |
| P | Asaad |
| Mohammed | Asaad Salem |
| M | Asadi |
| Andi | Asadul Islam |
| Muhammad | Asadullah Khawaja |
| Doaa | Asal |
| Nita | Asamoa-Manu Gyimah |
| Moses | Asante- Bremang |
| Alvin | Asante-Asamani |
| Elvam | Asaph |
| Christopher | Asare |
| Offei | Asare |
| Ashishkumar | Asari |
| Fernando | Ascanio Gosling |
| F | Ascari |
| Francesca | Ascari |
| J | Ascensão |
| Frehun | Asele |
| N | Asemota |
| E | Asensio Díaz |
| Enrique | Asensio Díaz |
| Luis | Asensio Gomez |
| Fitsum | Asfaw |
| Mohammed | Asfour |
| Ali | Asgar Hatim Ali |
| S | Asghar |
| Syed | Asghar Naqi |
| T | Asgill |
| Ruba | Asha |
| James | Ashbridge |
| J | Ashcroft |
| James | Ashcroft |
| S | Asher |
| Qamar | Ashfaq Ahmad |
| Susannah | Ashfield |
| Robert U | Ashford |
| Mohamed | Ashiq Mohamed Salim |
| Keyoumars | Ashkan |
| C | Ashmore |
| R | Ashour |
| Subhi | Ashour |
| F | Ashoush |
| Fouad | Ashoush |
| Adeela | Ashraf |
| Anam | Ashraf |
| F | Ashraf |
| M | Ashraf |
| Mohamed | Ashraf |
| Olfat | Ashraf |
| Sumaira | Ashraf |
| Abrar | Ashraf Ali |
| AU | Ashraf Butt |
| Mohammed | Ashrafi |
| John | Ashutosh Santoshi |
| Daniel | Ashworth |
| Yvonne | Asiedu |
| Muhammad | Asif |
| Suleman | asif Asif |
| Daniel | Asiimwe |
| Lois | Asiimwe |
| Ibrahim | Asiri |
| Mohammad | Asiri |
| Muhammad | Asjad |
| A | Askari |
| Alan | Askari |
| R | Askari |
| Aatif | Aslam |
| Imran | Aslam |
| Madiha | Aslam |
| Muhammad | Aslam |
| Sher | Aslam |
| Dawit | Asmamaw |
| Mona | Asnani |
| O | Asodisen |
| Sanjay | Asopa |
| R | Aspide |
| Raffaele | Aspide |
| A | Asqalan |
| R | Assadi |
| Reza | Assadi |
| M | Assaf |
| H | Assalaarachchi |
| Muhammad | assam Sarwar |
| Melatework | Assefa |
| Solomon | Assefa |
| Yared | Assefa |
| Marco | Assenza |
| Adel | Assiri |
| E | Asti |
| ELG | Asti |
| I | Astreidis |
| Ioannis | Astreidis |
| Aalap | Asurlekar |
| Manisha | Aswani |
| Sertaç | Ata Güler |
| Gustavo | Ataide |
| Aditya | Atal |
| Khalid | Atallah |
| B | Atanasov |
| Boyko | Atanasov |
| Hana | Atarabulsi |
| Henry | Atawurah |
| Abdurhman | Atea |
| Mohamed | Atef |
| A | Atefi |
| E | Athanasakis |
| Apostolos | Athanasiadis |
| A | Athanasiou |
| Antonios | Athanasiou |
| R | Athayde Nemésio |
| Zeenia | Ather |
| Muhammad | Ather Siddiqi |
| Yoann | Athiel |
| ADTS | Athukorala |
| Ruvinder | Athwal |
| Nora | Atiah |
| Clara | Atieno Odhiambo |
| J | Atienza Herrero |
| H | Atif |
| Terkaa | Atim |
| M | Atiq |
| Gokhan | Atis |
| Vincent | Ativor |
| A | Atiya |
| Bence | Atkari |
| Joseph | Atley |
| B | Atnafu |
| Bahru | Atnafu |
| Kazeem | Atobatele |
| KM | Atobatele |
| O | Atoyebi |
| Oluwole | Atoyebi |
| Amit | Atrey |
| Mulu | Atsbaha Weldu |
| R | Atta |
| Rewan | Atta |
| Joseph | Attard |
| Ahmad | Attia |
| Hajer | Attia |
| MG | Attoum |
| R | Attoum |
| Majed | Attoun Attoun |
| L | Attwell |
| J | Attwood |
| Safa | Atyah |
| Anne-Marie | Aubin |
| Angelica | Aubrey Morla |
| E | Aubry |
| Estelle | Aubry |
| Ziad | Audat |
| François | Audenet |
| A | Auerkari |
| Aino | Auerkari |
| G | Augustin |
| Goran | Augustin |
| Iñigo | Augusto |
| Jorge | Augusto Centurion |
| Gabriel | Augusto Cuevas Almando |
| Marcelo | Augusto Faria Freitas |
| Fernando | Augusto Lima Marson |
| Nestor | Augusto Muñoz Botero |
| A | Aujayeb |
| Avinash | Aujayeb |
| Pritpal | Aujla |
| Randeep | Aujla |
| Y | Auqui Medina |
| Nicoleta | Aurelia Sanda |
| Fahad | Aurif |
| Akawu | Auta |
| Andrea | Avanzolini |
| EK | Avci |
| M | Avelino |
| Melissa | Avelino |
| P | Avella |
| R | Avellana |
| AI | Avellaneda Camarena |
| S | Averbach |
| Sarah | Averbach |
| Alessia | Aversano |
| Konstantinos | Avgerinos |
| DS | Avila |
| Micaela | Avila |
| Pedro | Avila |
| N | Avni |
| Naor | Avni |
| Samuel | Avoine |
| Yonatan | Avraham Demma |
| Emmanouil | Avramidis |
| Andrej | Avsenak |
| Ahmed K | Awad |
| AK | Awad |
| Hadeel | Awad |
| R | Awad |
| Rabih | Awad |
| S | Awad |
| Selmy | Awad |
| Yasir | Awad |
| Alaa | Awad Hussein Ameri |
| M | Awadallah |
| M | Awadelkarim |
| S | Awadi |
| Mudi | Awaisu |
| Ahmed | Awaji |
| Kholoud | Awaji |
| Mirna | Awbakh |
| Lawrence | Awere-Kyere |
| Abimbola | Awopeju |
| Ahmed | Awrayit |
| Ziad | Awwad |
| K | Ayad |
| Kusay | Ayad |
| A | Ayala Ochoa |
| Tewabe | Ayalew |
| Omobolaji | Ayandipo |
| F | Ayasra |
| Faris | Ayasra |
| Y | Ayasra |
| Yazeed | Ayasra |
| A | Ayav |
| Engi̇n | Aybar |
| Eli̇f | Aybeni̇z Yildirim |
| Fatma | Ayca Gultekin |
| Fitsum | Ayde |
| L | Aydemir |
| C | Aydin |
| Cengiz | Aydin |
| Y | Aydin |
| Yener | Aydin |
| E | Aydın |
| Hüsnü | Aydın |
| Karim | Ayed |
| Brook | Ayele |
| Adewale | Ayeni |
| F | Ayeni |
| Funbi | Ayeni |
| J | Ayers |
| Iyehunwa | Ayinmode |
| Y | Aykanat |
| Andrew | Aylett |
| A | Ayman |
| Ammar | Ayman |
| Segun | Ayodeji Ogunkeyede |
| Olabamidele | Ayodele |
| Lateef | Ayodele Baiyewu |
| Malachy | Ayogu Emeka |
| J | Ayorinde |
| Abdu | Ayoub |
| Islam | Ayoub |
| Telce | Aysen Gurbuz |
| E | Aytac |
| Erman | Aytac |
| B | Ayub |
| Bakhtawar | Ayub |
| Bushra | Ayub |
| Khurram | Ayub |
| A | Ayubi |
| Azaz | Ayubi |
| E | Ayuso Herrera |
| M | Ayyaz |
| Mahmood | Ayyaz |
| M | Ayyub Anjum |
| Muhammad | Ayyub Anjum |
| MA | Azab |
| Mohammed A | Azab |
| Junaid | Azad |
| S | Azadnajafabad |
| German | Azahares Leal |
| Ayesha | Azam |
| DS | Azam |
| Mohammad | Azam |
| Riordan | Azam |
| S | Azam |
| Tayyab | Azam |
| Tariq | Azam Siddiqi |
| Elias | Azar |
| Faris | Azar |
| Gholamreza | Azarnia Azar Nia |
| Chisaki | Aze |
| Imran | Azeem |
| Mohd | Azem Fathi Mohammad Azmi |
| Felipe | Azenha Lamonica |
| Constança | Azevedo |
| José | Azevedo |
| P | Azevedo |
| Pedro | Azevedo |
| L | Azevedo De Camargo |
| A | Azhar |
| Amirah | Azhar |
| Faryal | Azhar |
| Saad | Azher |
| Nor | Azimah Abd Aziz |
| Z | Azimbeik |
| A | Aziz |
| Aliya | Aziz |
| Amr | Aziz |
| Humaira | Aziz |
| MU | Aziz |
| Tehmina | Aziz |
| Zaheda | Aziz |
| Gowhar | Aziz Bhat |
| Oula | Azizeh |
| MG | Azizeldine |
| Marah | Azkoul |
| Nor | Azlia Abdul Wahab |
| Dawit | Azmach |
| S | Azmanova Mladenovska |
| Angélica | Azucena Soto Carvajal |
| Ahmed Y | Azzam |
| AY | Azzam |
| Belal | Azzam |
| Abdelrahman | Azzam Omran |
| Rabindranath | B |
| Srinath | B S |
| J | Baaij |
| Mahdi | Baba |
| Abdulaziz | Babaier |
| OF | Babalola |
| Olakunle | Babalola |
| C | Baban |
| Maryam | Babar |
| MS | Babar |
| Mustapha | Babatunde |
| O | Babawale |
| Zeneb | Babay |
| Auwal | Babayo Kwankiyel |
| A | Babazadeh baghan |
| Maryam | Babba Danagundi |
| B | Babic |
| T | Babic |
| U | Babic |
| C | Babin |
| George | Babis |
| BH B | Babu |
| M | Babu |
| Narendra | Babu Siddaiah |
| Miruna | Babut |
| B | Baca |
| Bilgi | Baca |
| N | Bacalbasa |
| Nicolae | Bacalbasa |
| J | Bacarese-Hamilton |
| T | Bacarese-Hamilton |
| M | Baccar |
| Domenico | Baccellieri |
| Matilde | Bacchion |
| Roudi | Bachar |
| Sutej | Bachawat |
| Christopher | Bache |
| Shivani | Bachhav |
| A | Bachiri |
| S | Bachiri |
| T | Bächler |
| Thomas | Bächler |
| Ivan | Bacic |
| A | Bacon |
| Andrew | Bacon |
| F | Badahdah |
| Vanessa | Badas |
| C | Baddegama |
| M | Badedi |
| R | Badenes |
| Rafael | Badenes |
| Thomas | Badenoch |
| Giorgio | Badessi |
| Vivek | Badhe |
| Sanjiv | Badhwar |
| S | Badiani |
| Sarit | Badiani |
| M | Badiel |
| H | Badr |
| Salma | Badr |
| Roberto | Badra |
| A | Badran |
| Nour | Badran |
| Saif | Badran |
| Ssekitooleko | Badru |
| Albert | Baduell |
| Saleh | Baeesa |
| Santiago | Baena |
| Pedro | Baez |
| N | Baeza Pintado |
| M | Baeza-Murcia |
| Mohammed | Bafaquh |
| Anthony | Baffour Appiah |
| Darshan | Bafna |
| Giulia | Bagaglini |
| Jose | Bagan |
| Dinesh | Bagaria |
| Hamed | Bagheri |
| M | Bagheri |
| Nima | Bagheri |
| E | Bagouri |
| Mohamed | Bahaaeldin |
| Mojdeh | Bahadorzadeh |
| Ece | Bahçeci |
| Mohammad | Bahhour |
| Hans | Bahlmann |
| Kiarash | Bahrehmand |
| A | Bahreyni |
| Nazli | Bahtigur |
| Xueli | Bai |
| M | Baia |
| C | Baía |
| Catarina | Baía |
| Amal | Baicha |
| Mariam | Baidoun |
| AM | Baietti |
| M | Baig |
| Mariam | Baig |
| MM A S | Baig |
| Ashley | Bailey |
| Craig | Bailey |
| James | Bailey |
| K | Bailey |
| E | Baili |
| Efstratia | Baili |
| M | Bailón |
| Martín | Bailón |
| Aditya | Baindur |
| L | Bains |
| Lovenish | Bains |
| G | Baiocchi |
| Glauco | Baiocchi |
| A | Baite |
| Ankur | Bajaj |
| H | Bajjah |
| Hadeel | Bajjah |
| O | Bajomo |
| Minu | Bajpai |
| S | Bajramovic |
| Khalid | Bajunaid |
| Abu | Bakar Hafeez Bhatti |
| A | Bakare |
| Adewumi | Bakare |
| Jennifer | Baker |
| Joseph | Baker |
| M | Baker |
| Markus | Baker |
| O | Baker |
| OJ | Baker |
| Olivia | Baker |
| Thomas | Baker |
| I | Bakheit |
| Imad | Bakheit |
| H | Bakhit |
| Ahmed | Bakhsh |
| B | Bakhshayesh Eghbali |
| Akkasha | Bakhtiar |
| F | Bakhtiary |
| Khalid | Bakier Mohammed |
| Batoul | Bakkar |
| WJ | Bakker |
| B | Bakmaz |
| Bernarda | Bakmaz |
| G | Bakolas |
| Lubna | Bakr |
| Abdelrahman | Bakry |
| Ganesh | Bakshi |
| R | Bakx |
| Roel | Bakx |
| Pavin | Bal |
| Miklosh | Bala |
| Abubakar | Bala Muhammad |
| Vladimir | Balaban |
| Mohamed | Balabel |
| Abhinav | Balachandar Subbiah Ramasamy |
| B | Balagobi |
| Balasingam | Balagobi |
| C | Balague Ponz |
| A | Balaguer Román |
| Andrés | Balaguer Román |
| M | Balaguer-Castro |
| Mariano | Balaguer-Castro |
| Edward | Balai |
| A | Balakrishnan |
| Anita | Balakrishnan |
| D | Balalis |
| Dimitrios | Balalis |
| Cosmin | Balan |
| Julián | Balanta-Melo |
| Reyes | Balanzá |
| A | Balaphas |
| D | Balasubramaniam |
| Dinesh | Balasubramaniam |
| Srikant | Balasubramaniam |
| SP | Balasubramanian |
| Supriya | Balasubramanya |
| P | Balau |
| Egine | Balayan |
| H | Balbaloglu |
| Mohammed | Balbola |
| L | Baldari |
| Ludovica | Baldari |
| T | Baldasso |
| G | Baldazzi |
| C | Baldi |
| Caterina | Baldi |
| Manish | Baldia |
| E | Baldini |
| Edoardo | Baldini |
| A | Baldwin |
| AJ | Baldwin |
| Melissa | Baldwin |
| R | Baldwin-Smith |
| Paolo | Balercia |
| I | Balescu |
| Riccardo | Balestri |
| Ameera | Balhareth |
| Krittika | Bali |
| Oussama | Bali |
| Lipika | Baliarsing |
| MB | Balictar |
| E | Balik |
| Emre | Balik |
| H | Balkhi |
| Alasdair | Ball |
| Alice | Ball |
| L | Ball |
| A | Balla |
| Mohammed | balla Yousif balla |
| M | Ballabio |
| Mohammed | Ballal |
| K | Ballantyne |
| Eulalia | Ballester |
| E | Ballester Vazquez |
| Roberto | Ballestero |
| Marta | Ballesteros-Pomar |
| Monica | Ballon |
| Q | Ballouhey |
| Quentin | Ballouhey |
| M | Balluerca |
| Maria | Balluerca |
| Brenda | Balmaceda |
| R | Balmaceda |
| Zsolt J | Balogh |
| JA | Balogun |
| Mosimabale | Balogun |
| Simon | Balogun |
| M | Balouli |
| Maram | Balouli |
| I | Baloyiannis |
| Ioannis | Baloyiannis |
| G | Baltazar |
| Carlos | Baltazar Branco |
| Andrea | Balthazar |
| Saba | Balvardi |
| F | Bàmbina |
| VS | Ban |
| Tuba | Banaz |
| F | Banchini |
| GB K D | Bandara |
| A | Bandiera |
| S | Bandyopadhyay |
| Severine | Banek |
| Ayan | Banerjea |
| A | Banerjee |
| Abhirup | Banerjee |
| Shubhabrata | Banerjee |
| Sumit | Banerjee |
| Anant | Bangar |
| Mohammed | Bangash |
| P | Bangeas |
| Qaed | Bani Amer |
| Abdulsalam | Bani Hamad |
| Morad | Bani-hani |
| Eman | Baninasr |
| N | Baniyas |
| Oluseyi | Banjo |
| OO | Banjo |
| W | Bank |
| Charles | Banka |
| B | Bankhead |
| B | Bankhead-Kendall |
| Brittany | Bankhead-Kendall |
| Thomas | Banks |
| B | Banky |
| E | Bannone |
| Kuldeep | Bansal |
| S | Bansal |
| Sujesh | Bansal |
| S | Banting |
| Simon | Banting |
| Anas | Bany Issa |
| Hammam | Bany yasin |
| Carina | Banziger |
| Abubakar | Bappah Jaafar |
| John | Baptist Ssenyondwa |
| JC C | Baptista-Silva |
| J | Bapty |
| M | Baquedano |
| Mai | Baquedano |
| David | Baquero |
| Ahmed | Barakat |
| J | Barakat Awada |
| Jamile | Barakat Awada |
| Oussama | Baraket |
| Perel | Baral |
| E | Baran |
| Elif | Baran |
| A | Baranov |
| Maxime | Barat |
| Baratte | Baratte |
| Vidmantas | Barauskas |
| Frezza | Barbara |
| Andrew | Barbas |
| Giuseppe | Barbato |
| A | Barbazza |
| Y | Barbé |
| Rafael | Barberá |
| Cristina | Barberio |
| A | Barberis |
| Andrea | Barberis |
| L | Barbier |
| Luis | Barbier |
| O | Barbier |
| FJ | Barbosa Camacho |
| Genival | Barbosa Carvalho |
| Joao | Barbosa-Breda |
| F | Barbour |
| Ida | Barca |
| Indalecio | Barcelata Rodriguez |
| JC | Barcelon |
| Elizabeth | Bárcena |
| A | Barcin |
| A | Barclay |
| Laura | Bardelli |
| Metaxia | Bareka |
| Vanona | barijaona Razafindraibe |
| Kathia | Barillas |
| Sabrina | Barillas |
| Goran | Barisic |
| Tatjana | Barišić |
| John | Barker |
| Jonathan | Barker |
| Sergey | Barkhatov |
| C | Barkolias |
| E | Barkolias |
| AM | Barlas |
| Adam | Barlow |
| C | Barlow |
| R | Barmasse |
| B | Barmayehvar |
| C | Barmpagianni |
| L | Barnard |
| Stephen | Barnett |
| Isaac | Barnor |
| Paolo | Baroffio |
| R | Baron |
| Ryan | Baron |
| M | Barone |
| Mirko | Barone |
| R | Barone |
| F | Baroni Alves Makdissi |
| G | Baronio |
| Gianluca | Baronio |
| Salma | Baroudi |
| F | Barra |
| Fabio | Barra |
| A | Barrabe |
| AG | Barranquero |
| A | Barraquio |
| B | Barrat |
| Benjamin | Barrat |
| Mauricio | Barreda |
| Diogo | Barreiro |
| C | Barrena lópez |
| Cristina | Barrena López |
| E | Barret |
| Alexandra | Barreto |
| Analy | Barreto Galeano |
| A | Barreto Grimaldos |
| ZM | Barrett-Brown |
| Diana | Barretto |
| Belinda | Barrientos Nuñez |
| JM | Barrio |
| M | Barrionuevo Ramos |
| Maria | Barrionuevo Ramos |
| Maria | Barrios Carvajal |
| A | Barrios Duarte |
| Amalia | Barrios Duarte |
| AV | Barros |
| N | Barros Jr |
| Hannah | Barrow |
| J | Barrow |
| C | Barry |
| Jessica | Barry |
| Mary | Barry |
| Peter | Barry |
| Stevie | Barry |
| Ashish | Bartakke |
| Andrea | Bartalini Cinughi de Pazzi |
| B | Bartalucci |
| JL | Bartha Rasero |
| I | Bartolini |
| Ilenia | Bartolini |
| K | Bartosiak |
| A | Bartsch |
| Raquel | Bartz |
| Dimitrios | Bartziotas |
| Anupama | Barua |
| G | Barugola |
| Giuliano | Barugola |
| Elisee | Baruwa |
| Enes | Baş |
| Vikas | Basa |
| VS | Basappanavar |
| Silvia | Basato |
| Vladimir | Bascarevic |
| M | Basendowah |
| Mohammed | Basendowah |
| A | Basgaran |
| A | Basha |
| Beibit | Bashabayev |
| AK | Basher |
| Aladdin | Bashir |
| Alia | Bashir |
| M | Bashir |
| Osman | Bashir |
| Rasha | Bashir |
| Y | Bashir |
| Ali | Bashiri |
| V | Bashkirova |
| Tayseer | Basi |
| Francis | Basimbe |
| Muhammad | Basir |
| D | Baskaran |
| Dinnish | Baskaran |
| Oshan | Basnayake |
| PS | Basnyat |
| GA | Bass |
| Ali | Bassi |
| C | Bassi |
| F | Bassily |
| Muhammad | Bassiouni |
| Shahin | Bastaninejad |
| F | Bastard |
| François | Bastard |
| Mostafa | Bastawesy |
| Alberto | Basterra Rincon |
| Joaquin | Bastet |
| Mae-Lynn | Bastion |
| S | Basu |
| Somprakas | Basu |
| Utkarsha | Basu |
| Mediatrice | Batangana |
| A | Bateman |
| Antony | Bateman |
| S | Baterl |
| MF | Bath |
| Michael | Bath |
| A | Bathgate |
| RE | Baticulon |
| Ronnie | Baticulon |
| Oguzkagan | Batikan |
| S | Batista |
| Sylvia | Batista |
| P | Batistotti |
| HH | Batjer |
| Brian | Batko |
| Olivia | Batog |
| Fizza | Batool |
| Sehrish | Batool |
| Nitin | Batra |
| Martin | Batstone |
| G | Battello |
| Melanie | Battershell |
| MJ | Battista |
| Giovanni | Battista Fonsi |
| Enrico | Battistella |
| Marlies | Bauer |
| R | Baumber |
| Rachel | Baumber |
| L | Baumgart |
| S | Baumgarten |
| Sabine | Baumgarten |
| JB | Baun |
| Mireia | Bauzá |
| D | Bavishi |
| Dauda | Bawa |
| Kyriaki | Baxevanidou |
| Z | Baxter |
| Zachary | Baxter |
| Muhannad | Bayazid |
| J | Bayer |
| Jörg | Bayer |
| Z | Bayhan |
| Zulfu | Bayhan |
| Firew | Bayissa |
| Morgan | Bayley |
| J | Bayne |
| A | Bayomy |
| AbdulHakeem | Bayomy |
| Gabriel | Bayona-Alvarado |
| Ç | Bayram |
| E | Bayramov |
| A | Bazaev |
| Andrey | Bazaev |
| Borja | Bazán Inostroza |
| Alberto | Bazan Soto |
| Muhammad | Bazil Musharraf |
| H | Bazzi |
| M | Bazzi |
| N | Bazzi |
| Brian | Bbosa |
| Manjunath | Bd |
| Saja | Bdour |
| David | Beahm |
| P | Beak |
| AJ | Beamish |
| Carlos | Beas Ruiz-Velasco |
| Ana | Beatriz Calderon Alvarado |
| O | Beaumont |
| V | Bebia |
| R | Becerra |
| FC | Becerra García |
| Luis | Becerra Mendez |
| N | Bechar |
| H | Bechri |
| Hajar | Bechri |
| J | Beck |
| Jürgen | Beck |
| Manisha | Beck |
| Renata | Beck |
| Johannes | Becker |
| Karin | Becker |
| Andrew | Beckett |
| Dereje | Bedane |
| D | Bedane Hunde |
| María | Bedate Núnez |
| D | Beddy |
| Antoinette | Bediako Bowan |
| Alvaro | Bedoya-Ronga |
| A | Bedzhanyan |
| Arkady | Bedzhanyan |
| Charlotte | Bee |
| Helen | Beech |
| N | Beech |
| B | Beelders |
| A | Beer |
| Phillipa | Beesley |
| Paul | Beganton |
| E | Begoña |
| Alvarez-ramos | Begoña Aranzazu |
| María | Begoña Gregorio Crespo |
| María | Begoña Pastor Nieto |
| A | Beguiristain |
| Adolfo | Beguiristain |
| Vuqar | Behbudov |
| P | Behera |
| Prateek | Behera |
| Kevin | Behm |
| B | Behmanesh |
| Michael | Behr |
| Mona | Behravesh |
| Abdollah | Behzadi |
| Khalid | Beidas |
| Klara | Beitl |
| M | Bejarano Serrano |
| Miguel | Bejarano Serrano |
| Ephrem | Bekele |
| K | Bekele |
| Kebebe | Bekele |
| Philimon | Bekele |
| Mahteme | Bekele Muleta |
| M | Bekheit |
| E | Bekhor |
| Fanny | Belais |
| Laurence | Belanger |
| Mathieu | Belanger |
| P | Belani |
| Othman | Belarabi |
| B | Belarbi |
| Armin | Belarmino |
| E | Belcher |
| Elizabeth | Belcher |
| Raluca | Belchita |
| Rocío | Belda |
| Uros | Bele |
| María | Belén Alonso Bartolomé |
| Ana | Belén Casas Marcos |
| Sofia | Belen Diaz Pineda |
| Ana | Belén Gallardo |
| María | Belén Ramírez Senent |
| N | Belev |
| Nikolay | Belev |
| A | Belgaumkar |
| Ajay | Belgaumkar |
| AP | Belgaumkar |
| Orkia | Belhadri |
| Francesco | Belia |
| Orimisan | Belie |
| Diego | Belisle |
| ZH | Belkhadir |
| Susana | Bella Romera |
| A | Bellacci |
| L | Bellanti |
| Luca | Bellanti |
| V | Bellato |
| Vittoria | Bellato |
| D | Bellemare |
| Jack | Bellerby |
| A | Belli |
| Andrea | Belli |
| Gabriele | Bellio |
| C | Bellis |
| Sergi | Bellmunt-Montoya |
| Funmi | Bello |
| J | Bello |
| Jibril | Bello |
| L | Bello |
| Kabiru | Bello Abubakar |
| Shahir | Bello Umar |
| Nafisatu | Bello-Muhammad |
| F | Bellolio |
| Paolo | Bellora |
| T | Bellos |
| O | Bellou |
| E | Beltrami |
| GA | Beltramini |
| Larissa | Beltran |
| Miguel | Beltran |
| J | Beltrán de Heredia |
| Juan | Beltrán de Heredia |
| Pablo | Beltran Miranda |
| P | Beltrán-Miranda |
| A | Belvedere |
| Angela | Belvedere |
| Orlin | Belyaev |
| Etienne | Belzile |
| Anass | Ben Amer |
| Esraa | Ben esmael |
| Omar | Ben Forge Risk |
| Mahmoud | Ben ghrema |
| H | Ben Hasan |
| Hayat | Ben Hasan |
| N | Ben Hasan |
| RAI | Ben jouira |
| Emadeddin T M | Ben Khalifa |
| M | Ben Othmen |
| A | Ben-Sassi |
| D | Benali ammar |
| Y | Benallal |
| Nassim | Benallel |
| S | Benamar |
| A | Benamwor |
| Adeka | Benard |
| EA | Benavides Hernández |
| M | Bence |
| Meryem | Benchekroun Belabbes |
| L | Bendjemar |
| Lynda | Bendjemar |
| Semir | Benecha |
| E | Benedetti |
| F | Benedetto |
| Magdalena | Benegas |
| O | Benet Muñoz |
| Giacomo | Benettini |
| Maria | Benevolo |
| Savitha | Bengeri |
| Erika | Bengtson |
| P | Benharash |
| Peyman | Benharash |
| Rema | Benhariz |
| Kristina | Benirschke |
| Oscar | Benitez |
| Nicole | Benitez Benitez |
| Ana | Benítez Riesco |
| I | Benítez-Linero |
| Miles | Benjamin |
| MW | Benjamin |
| Santosh | Benjamin |
| A | Benkabbou |
| Amine | Benkabbou |
| M | Benmamar |
| Fatma | Benmasoud |
| S | Bennett |
| M | Benoit |
| M | Bensghir |
| Mustapha | Bensghir |
| Guy | Benshetrit |
| EA B | Bensi |
| Zaineb | Benslimane |
| K | Bensoltane |
| Charlotte | Benson |
| Ruth | Benson |
| Malissa | Bentham |
| R | Bento |
| Domenico | Benvenuto Giuliani |
| R | Berbash |
| German | Berbel |
| Norberto | Berber |
| Martin | Berden |
| Gutiérrez | Bérénice |
| Luigi | Beretta |
| E | Berg |
| C | Bergamini |
| Carlo | Bergamini |
| Andrej | Bergauer |
| D | Bergeat |
| Damien | Bergeat |
| Eyerusalem | Bergene |
| Julian | Berger |
| A | Bergeron |
| Nicole | Bergmann |
| M | Bergonzani |
| Michela | Bergonzani |
| Alazar | Berhe |
| Ataklitie | Berhea |
| L | Berikashvili |
| Müserref | Beril Dincer |
| J | Beristain-Hernandez |
| Jose-Luis | Beristain-Hernandez |
| Loreto | Berjon De La Vega |
| Muhammet | Berkay Sakaoglu |
| Eva | Berkeveld |
| MT | Berlanga Rojas |
| Hugo | Bermejo |
| Lorena | Bermell Marco |
| AA | Bernabé Esteban |
| M | Bernabei |
| Massimiliano | Bernabei |
| Silvana | Bernadetta Puglisi |
| Fabio | Bernagozzi |
| Aldo | Bernal Hernandez |
| JC | Bernal-Sprekelsen |
| P | Bernante |
| Paolo | Bernante |
| D | Bernardi |
| Daniele | Bernardi |
| Laura | Bernardi |
| Martin H | Bernardi |
| M | Bernasconi |
| AE | Berndtson |
| Allison | Berndtson |
| Emily | Berner |
| C | Berney |
| Christophe | Berney |
| M | Bernon |
| V | Bernotaite |
| Rania | Berrami |
| Roberto | Berretta |
| Stefano | Berrettini |
| Sara | Berrocal |
| Juan | Berrocal Cuadrado |
| Pedro | Berrones Moreno Berrones Moreno |
| F | Berrospi |
| Francisco | Berrospi |
| Brendan | Berry |
| G | Berry |
| Janet | Berry |
| Richard | Berry |
| Bruno | Berselli |
| M | Berselli |
| Mattia | Berselli |
| G | Bertelli |
| Nicolas | Bertheuil |
| Pierre | Berthoumieu |
| L | Bertoglio |
| Luca | Bertoglio |
| P | Bertoglio |
| Pietro | Bertoglio |
| L | Bertolaccini |
| Luca | Bertolaccini |
| Elisa | Bertolani |
| G | Bertoli |
| Francesca | Bertolina |
| Giacomo | Bertolini |
| Marta | Bertrand |
| Coro | Bescós |
| T | Bese |
| Tugan | Bese |
| Hasan | Besim |
| WF | Besira |
| Marc | Besselink |
| N | Besser |
| Nikolaos | Bessias |
| A | Besson |
| Alex | Besson |
| Lauren | Best |
| D | Beswick |
| Daniel | Beswick |
| J | Betalleluz Pallardel |
| Jenner | Betalleluz Pallardel |
| Alva | Bethurum |
| Nagat | Bettamer |
| Ricardo | Bettencourt Morais |
| J | Bettoni |
| Jérémie | Bettoni |
| CS | Betz |
| Alexandra | Beuca |
| K | Bevan |
| E | Bevilacqua |
| J | Bewarder |
| Julian | Bewarder |
| Gaym | Beyene |
| K | Beyer |
| Katharina | Beyer |
| YS | Bezabih |
| C | Bezede |
| Cosmin | Bezede |
| TS | Bezerra |
| Andriy | Beznosenko |
| Ashwin | Bhadresha |
| S | Bhagat |
| Bhuvanshyam | Bhaktavatsalam |
| Bhalchandra | Bhalerao |
| A | Bhalla |
| Ash | Bhalla |
| Rohan | Bhalla |
| Anuradha | Bhama |
| AR | Bhama |
| Shivam | Bhanderi |
| A | Bhangu |
| Balamurali | Bharathan |
| Rohit | Bhardwaj |
| Aman | Bhargava |
| Manoj | Bharucha |
| S | Bhasin |
| Dhananjaya | Bhat |
| S | Bhat |
| K | Bhatia |
| Kailash | Bhatia |
| M | Bhatia |
| Mohit | Bhatia |
| Anuj | Bhatnagar |
| DRK | Bhatta |
| G | Bhatta |
| Gakul | Bhatta |
| P | Bhattacharya |
| S | Bhattacharya |
| AB H | Bhatti |
| Arun | Bhatti |
| Hamza | Bhatti |
| Khalid | Bhatti |
| Samiullah | Bhatti |
| Waqar | Bhatti |
| A | Bhavaraju |
| Avi | Bhavaraju |
| Vishal | Bhende |
| D | Bhojwani |
| Deepika | Bhojwani |
| DP | Bhor |
| Pramod | Bhor |
| S | Bhudia |
| S | Bhusal |
| Subarna | Bhusal |
| N | Bhutiani |
| Neal | Bhutiani |
| Shameen | Bhutto |
| T | Bhuvanakrishna |
| M | Biala |
| Marwa | Biala |
| Alessia | Biancafarina |
| E | Biancardi |
| A | Bianchera |
| Lorenzo | Bianchi |
| Valentina | Bianchi |
| M | Bianchini |
| Agustín | Bianco |
| F | Bianco |
| Francesco | Bianco |
| Giuseppe | Bianco |
| M | Biasini |
| D | Biasoni |
| David | Biau |
| L | Bibby |
| S | Biber |
| B | Biccard |
| Bruce | Biccard |
| David | Bichell |
| Jordan | Bickerdyke |
| J | Bicki |
| Heena | Bidd |
| Lauren | Bidois |
| M | Biebl |
| Mumbere | Bienfait |
| Johannes | Bier |
| W | Bierman |
| Christopher | Bierton |
| W | Biffl |
| Walter | Biffl |
| D | Bigam |
| David | Bigam |
| Katherine | Bigay |
| Benjamin | Bigelow |
| A | Biggs |
| Michael | Biggs |
| Sarah | Biggs |
| P | Bigot |
| Okker | Bijlstra |
| Yemurai | Bikwa |
| Saad | Bilal Ahmad |
| Mustafa | Bilal Hamarat |
| Javeria | Bilal Qamar |
| Ali | Bilal Ulas |
| H | Bileid Bakeer |
| Roman | Bilenko |
| M | Bilfaqirah |
| I | Biliatis |
| Ioannis | Biliatis |
| A | Billè |
| Mestan | Bilmez |
| Jerko | Biloš |
| Kim | Bin |
| Manerh | Bin Mosa |
| A | Bin Nasser |
| Ahmad | Bin Nasser |
| Sari | Bin nour |
| Fayez | Bin Omran |
| Khalid | Bin Saad |
| Osama | Bin Sohail |
| Sinan | Binboga |
| Elif | Binboğa |
| Barbara | Binda |
| A | Binder |
| AD | Binder |
| Alf-Dorian | Binder |
| J | Binder |
| Johannes | Binder |
| Ahmed | Binjaloud |
| M | Binnawara |
| Faiqa | Binte Aamir |
| Bojan | Biočina |
| Alberto | Biondi |
| Massimo | Biondi |
| Vedrana | Biosic |
| Garance | Biosse-Duplan |
| Hari | Bipin Radhakrishnan Kattana |
| David | Bird |
| Sophie | Bird |
| E | Birgin |
| Nuha | Birido |
| Arianna | Birindelli |
| Elisa | Birnbaum |
| Erdal | Birol Bostanci |
| SL | Birolo |
| G | Birqeeq |
| P | Bisagni |
| Theodosios | Bisdas |
| Tayfun | Bisgin |
| AK | Bisoi |
| Daniele | Bissacco |
| Guido | Bissolotti |
| Maria | Bisulli |
| Samer | Bitar |
| S | Bitsianis |
| MN | Bittar |
| Reinhard | Bittner |
| Kristina | Bitunjac |
| Achille | Bizimana |
| Yemisirach | Bizuneh Akililu |
| Karin | Björnström Karlsson |
| Peter | Black |
| James | Blackwell |
| Michael | Blackwell |
| J | Blair |
| James | Blair |
| Anne-Sophie | Blais |
| I | Blake |
| Nikita | Blake |
| T | Blanc |
| Claire | Blanchard |
| David | Blanco |
| J | Blanco |
| Lara | Blanco Terés |
| R | Blanco-Colino |
| Nyinawabagesera | Blandine |
| JL | Blas Laina |
| J | Blasco-Moreu |
| L | Blasco-Torres |
| Alison | Blatt |
| Alejandro | Blaubach |
| Ben | Blay Ofosu-Barko |
| Aida | Blaya |
| Krešimir | Blažević |
| A | Blazquez Martin |
| Cait | Bleakley |
| S | Bleda |
| Silvia | Bleda |
| S | Bleibleh |
| Sabri | Bleibleh |
| N | Blencowe |
| Natalie | Blencowe |
| NS | Blencowe |
| C | Blier |
| Jeremy | Bliss |
| Frank | Bloemers |
| Nina | Blomme |
| O | Bloom |
| C | Blundell |
| Benedict | Boakye |
| M | Boal |
| T | Board |
| Timothy | Board |
| Rachael | Boardley |
| Abigail | Boateng |
| P | Bobak |
| Peter | Bobak |
| Bailea | Bobich |
| Marcin | Bobiński |
| Dino | Bobovec |
| G | Bocca |
| A | Boccabella |
| L | Boccalatte |
| LA | Boccalatte |
| Luis | Boccalatte |
| G | Bocchialini |
| Antonio | Bocchino |
| Melissa | Bochner |
| J | Bock |
| Jacob | Bock |
| Wolfgang | Böcker |
| Gabriela | Bocsa |
| Guillaume | Boddaert |
| A | Boddy |
| Alex | Boddy |
| Chris | Bode |
| CO | Bode |
| AS | Bodla |
| Zsolt | Bodnar |
| M | Boeck |
| Marissa | Boeck |
| A | Boeckxstaens |
| C | Boeker |
| Clara | Boeker |
| Lars | Boenicke |
| Andreas | Boening |
| Catherine | Boereboom |
| D | Boerma |
| E | Boerma |
| EG | Boerma |
| Evert-Jan | Boerma |
| MA | Boermeester |
| G | Bogani |
| Giorgio | Bogani |
| Amalia | Bogarin |
| M | Bogdan |
| Monica | Bogdan |
| Andrei | Bogdan Văcărașu |
| Aleksandar | Bogdanovic |
| Ivan | Bogdanovic |
| Manobhiram | Boggavarapu |
| Selene | Bogoni |
| Matthieu | Boisson |
| S | Bojic |
| Jovana | Bojičić |
| M | Bokenkamp |
| Mary | Bokenkamp |
| Areej | Bokhari |
| Covalic | Bokossa |
| M | Boland |
| F | Bolanos-Morales |
| Francina | Bolanos-Morales |
| Raikhan | Bolatbekova |
| A | Bolbarán |
| Christian | Bolenz |
| Emmanuel | Boleslawski |
| Jarlath | Bolger |
| S | Boligo |
| Rafik | Bolis |
| Dinimo | Bolivar Saenz |
| Enton | Bollano |
| M | Bolli |
| S | Bolognesi |
| Silvia | Bolognesi |
| J | Bolota |
| Joana | Bolota |
| A | Bolouriyan |
| M | Bolster-van Eenennaam |
| John | Bolt |
| Basak | Bolukbasi |
| D | Bona |
| Davide | Bona |
| E | Bonaiuto |
| Marta | Bonaldi |
| G | Bonavina |
| Giulia | Bonavina |
| L | Bonavina |
| Luigi | Bonavina |
| E | Bonci |
| Eduard-Alexandru | Bonci |
| G | Bond-Smith |
| Peter | Bonde |
| A | Bondurri |
| Andrea | Bondurri |
| Mireya | Bonet |
| Barbara | Bonfanti |
| Christopher | Bonfield |
| D | Bonfili |
| C | Bong |
| L | Boni |
| Luigi | Boni |
| A | Bonilla |
| Ana | Bonilla |
| Carlos | Bonilla |
| F | Bonilla |
| Fernando | Bonilla Cal |
| PV | Bonilla Sanchez |
| FJ | Bonilla-Escobar |
| A | Bonnard |
| Soline | Bonneau |
| Stéphane | Bonnet |
| Jorge | Bonnin |
| Stefano | Bonomi |
| J | Bontinck |
| Julie | Bontinck |
| Kian | Boon Wong |
| K | Booth |
| M | Boras |
| Miran | Boras |
| Sorour | Borayek |
| G | Borda-Luque |
| Giuliano | Borda-Luque |
| M | Bordenave |
| P | Bordoni |
| Pierpaolo | Bordoni |
| A | Borello |
| Elaine | Borg |
| Jeremy | Borg Myatt |
| F | Borges |
| Mafalda | Borges |
| N | Borges |
| F | Borghi |
| Felice | Borghi |
| AB J | Borgstein |
| Alexander | Borgstein |
| Morteza | Borhani |
| Kurosch | Borhanian |
| F | Boriani |
| U | Bork |
| Ulrich | Bork |
| H | Borla |
| Hernan | Borla |
| N | Börner |
| Nikolaus | Börner |
| Emanuel | Borovic |
| David W | Borowski |
| B | Borraccino |
| R | Borreca |
| VM | Borrego Estella |
| G | Borroni |
| Giacomo | Borroni |
| D | Borselle |
| M | Borselli |
| Kim | Borsky |
| Biplob | Borthakur |
| L | Bortolasi |
| G | Bortolin |
| Carlo | Bortolotti |
| Marina | Bortul |
| Maksym | Boruta |
| A | Borzacchelli |
| Ana | Bosak Versic |
| D | Bosanquet |
| David | Bosanquet |
| KD | Bosch |
| Marina | Bosch |
| D | Bosch Garcia |
| David | Bosch Garcia |
| M | Bosch-Ramírez |
| J | Boschet |
| Paolo | Boscolo Rizzo |
| Lorenzo | Bosio |
| Raul | Bosio |
| EB | Bostanci |
| Lais | Botacin |
| Pedro | Botelho |
| Vipul | Bothara |
| Jyoti | Bothra |
| Iva | Botica |
| Carlos | Boto |
| A | Bottari |
| Andrea | Bottari |
| A | Böttcher |
| Arne | Böttcher |
| L | Boualila |
| Lina | Boualila |
| R | Bouanane |
| Othmane | Bouanani |
| S | Bouaoud |
| Souad | Bouaoud |
| K | Bouchagier |
| Konstantinos | Bouchagier |
| M | Bouchard |
| PA | Bouche |
| Pierre-Alban | Bouche |
| Kamel | Bouchenak |
| S | Boucher |
| Sophie | Boucher |
| Sofia | Boucher-Kovalik |
| A | Bouchetara |
| N | Bouchiba |
| R | Boudou |
| Rocio | Boudou |
| Judy | Boughey |
| A | Bouhuwaish |
| Ahmad | Bouhuwaish |
| Alassan | Boukari |
| AZ | Boukli Hacene |
| Cindy | Boulanger-Gobeil |
| K | Bouliaris |
| Konstantinos | Bouliaris |
| A | Boulton |
| AJ | Boulton |
| Natacha | Boumas |
| A | Bourial |
| G | Bourke |
| Grainne | Bourke |
| E | Bourmpouteli |
| Anna | Bouronikou |
| S | Boussedra |
| Safia | Boussedra |
| Saber | Boutayeb |
| N | Boutimzine |
| M | Boutros |
| RM | Bouttelgier |
| LA | Bouziane |
| Raffaele | Bova |
| S | Boveda gonzalez |
| Claudio | Bovolenta Murta |
| Conor | Bowe |
| D | Bowen |
| J | Bowen |
| Joel | Bowen |
| Christopher | Bowler |
| S | Bowman |
| T | Bowman |
| Louis | Boyce |
| H | Boyd-Carson |
| Hannah | Boyd-Carson |
| Joshua | Boyes |
| C | Boyle |
| Connor | Boyle |
| E | Boyle |
| Ellen | Boyle |
| T | Boyle |
| Rebecca | Boyles |
| K | Bozada Gutierrez |
| Katya | Bozada-Gutiérrez |
| A | Bozakok |
| E | Bozdağ |
| Bahadır | Bozkırlı |
| E | Bozkurt |
| Emre | Bozkurt |
| MA | Bozkurt |
| Nulvin | Bozo |
| Adel | Bozorgzadeh |
| Antonio | Bozzani |
| Andries | Braat |
| Umberto | Bracale |
| G | Brachini |
| Gioia | Brachini |
| Muriel | Brackstone |
| Molly | Bradbury |
| Thomas | Bradley |
| Catherine | Bradshaw |
| CJ | Bradshaw |
| L | Bradshaw |
| Luke | Bradshaw |
| S | Bradulskis |
| A | Braga |
| Maria | Bragado González |
| H | Braham |
| D | Brahmbhatt |
| K | Brahmbhatt |
| Yasmine | Braimah |
| Konstantinos | Bramis |
| Irene | Brana |
| G | Branagan |
| Graham | Branagan |
| C | Branco |
| Mariana | Branco Lopes |
| Marcelo | Brandao |
| R | Brandariz |
| Rodrigo | Brandariz |
| Jury | Brandolini |
| A | Branquinho |
| R | Branquinho |
| Rita | Branquinho |
| D | Branzan |
| Daniela | Branzan |
| A | Brar |
| Amanpreet | Brar |
| T | Brasileiro Silva Pacheco |
| Benjamin | Braslow |
| C | Brasset |
| G | Brat |
| C | Brathwaite |
| CE M | Brathwaite |
| Nikolina | Bratošević Vučičić |
| D | Bratt |
| D | Bratus |
| Dejan | Bratus |
| T | Bratuš |
| C | Braumann |
| Chris | Braumann |
| Mauro | Bravo |
| SL | Bravo |
| SL R | Bravo |
| Layze | Braz de Oliveira |
| Esther | Brea Gómez |
| S | Breakeit |
| Sarah | Breakeit |
| D | Breda |
| H | Breda Pessetti |
| Mikhail | Bredikhin |
| K | Breen |
| Kerry | Breen |
| R | Breheret |
| Renaud | Breheret |
| O | Breik |
| Omar | Breik |
| Zdrinko | Brekalo |
| Mark | Bremholm Ellebaek |
| Signe | Bremholm Ellebæk |
| C | Brennan |
| Caitlin | Brennan |
| P | Brennan |
| Paul | Brennan |
| F | Brennfleck |
| Tiago | Bresciani |
| F | Bretagnol |
| C | Bretherton |
| Christopher | Bretherton |
| C | Brett-Miller |
| RG | Breuer |
| B | Brew |
| H | Brewer |
| Hilary | Brewer |
| E | Brian |
| D | Briatico |
| SK | Bridges |
| Elsie | Bridgman |
| Carolina | Brienze |
| Tim | Bright |
| Rhiannon | Brignall |
| L | Brignone |
| Pradeep | Brijkishor Sharma |
| Svetlana | Brincat |
| Lukas | Briner |
| Rebecca | Brinkler |
| Petra | Brinskelle |
| Edulfo | Britez Barrios |
| Kattiucy | Brito |
| Francisca | Brito da Silva |
| Analia | Britos |
| Tim | Brits |
| E | Britton |
| John | Britton |
| L | Britton - Zier |
| Linda | Britton-Zier |
| Ariberto | Brivio |
| G | Brixton |
| Genevieve | Brixton |
| Lucija | Brkic |
| Samuel | Broadbent |
| Phoebe | Brobbey |
| A | Broch |
| J | Brockwell |
| C | Broe |
| Claire | Broe |
| Tobias | Broecheler |
| Alessandro | Broglia |
| N | Brogly |
| A | Brolese |
| Alberto | Brolese |
| M | Brolese |
| E | Brolo |
| Estuardo | Brolo |
| S | Bromage |
| Stephen | Bromage |
| H | Bronger |
| Mark | Brooke-Smith |
| P | Brouk |
| Peiman | Brouk |
| P | Brouki Milan |
| Peiman | Brouki Milan |
| T | Brow |
| A | Brown |
| Allison | Brown |
| Andrew | Brown |
| B | Brown |
| BC | Brown |
| Benjamin | Brown |
| C | Brown |
| Christopher | Brown |
| D | Brown |
| IG | Brown |
| J | Brown |
| James | Brown |
| L | Brown |
| O | Brown |
| S | Brown |
| Sarah | Brown |
| SR | Brown |
| V | Brown |
| Victoria | Brown |
| Wendy | Brown |
| L | Brown Fumeau |
| R | Bruballa |
| Nolan | Bruce |
| Jan | Bruder |
| Elizabeth | Bruenderman |
| Nicolás | Bruera |
| Carlo | Brugiotti |
| Marcos | Bruna Esteban |
| Laurent | Brunaud |
| A | Brunelli |
| Alessandro | Brunelli |
| Aina | Brunet-Garcia |
| Federica | Brunetti |
| Eberhard | Brunner |
| SM | Brunner |
| Stefan M | Brunner |
| U | Brunner |
| Chiara | Bruno |
| E | Brunocilla |
| Christiane | Bruns |
| A | Brunt |
| Luca | Bruschini |
| A | Bruscino |
| Alessandro | Bruscino |
| P | Bruzzaniti |
| Placido | Bruzzaniti |
| F | Brzeszczyński |
| Filip | Brzeszczyński |
| Domagoj | Brzic |
| R | Bschorer |
| I | Buarque |
| Igor | Buarque |
| IL | Buarque |
| Amina | Buba |
| Olha | Bubliieva |
| Pamela | Buchwald |
| Benjamin | Buckland |
| Abbigayle | Buckton-Perkins |
| Georges | Bucyibaruta |
| Alina-Maria | Budacan |
| Karel | Buddingh |
| O | Budha Magar |
| Veronika | Budyakova |
| R | Buenaño González |
| Javier | Buendia Pérez |
| AD | Bueno Cañones |
| Luz | Bueno Rey |
| GA | Buerba |
| T | Bueser |
| Teofila | Bueser |
| Marco | Bueter |
| K | Buffenoir |
| Daniele | Bugada |
| N | Bugdayci |
| Mumtaz | Bughio |
| D | Buğra |
| Dursun | Buğra |
| Lily | Builth-Snoad |
| JJ P | Buitendag |
| Acosta | Buitrago |
| Miguel | Buitrago |
| Cristina | Bujoreanu |
| Mohammed | Bukari |
| SI | Bukhari |
| Walid | Bukhari |
| Ruth | Bulder |
| M | Buljubasich |
| Martin | Buljubasich |
| D | Bulthé |
| Danny | Bulthé |
| M | Bulugma |
| ND | Bulut Yüksel |
| U | Bumbasirevic |
| Uros | Bumbasirevic |
| Boris | Bumber |
| Francesca | Bunino |
| J | Bunni |
| Gisele | Bunogerane Juru |
| Andres | Bur |
| A | Burahee |
| Ahmet | Burak Ciftci |
| Muhammet | Burak Kamburoğlu |
| Cemil | Burak Kulle |
| Arturo | Burchakchi |
| Emine | Burcu Cigsar |
| Lukáš | Burda |
| Eleanor | Burden |
| Gemma | Burdge |
| L | Burdine |
| Lyle | Burdine |
| Zoe | Burdon |
| S | Burg |
| Simon | Burg |
| Dania | Burgan |
| Julio | Burgos |
| Barbara | Burgos-Blasco |
| Mohammad | Burhan Khan |
| M | Burhan Ul Haq |
| Cathy | Burke |
| E | Burke |
| JR | Burke |
| C | Burks |
| Ciersten | Burks |
| C | Burlew |
| Clay | Burlew |
| Nikita | Burlov |
| Alexandr | Burmistrov |
| S | Burnard |
| N | Burnside |
| Nathan | Burnside |
| J | Burtscher |
| Johannes | Burtscher |
| FE | Buruiana |
| Musa | Busarira |
| Opeyemi | Busayo Borokinni |
| Chia-Jung | Busch |
| CJ | Busch |
| Hassan | Bushaala |
| Raisa | Bushra |
| Ayşe | Büşra Önder |
| N | Busse |
| Edoardo | Bussolin |
| Lara | Bußmann |
| Francesco | Bussu |
| Juan | Bustamante-Munguira |
| Nasir | Bustangi |
| Laura | Busto |
| Sara | Busto Suarez |
| Ronald W | Busuttil |
| Marko | Buta |
| T | Bute |
| C | Butler |
| Charles | Butler |
| John | Butler |
| Roshan | Butt |
| U | Butt |
| Usman | Butt |
| W | Butt |
| Martin | Buttaro |
| W | Butterworth |
| Giovanni | Butturini |
| A | Butyrskii |
| Aleksandr | Butyrskii |
| Alexis | Buunaaim |
| Süleyman | Büyükaşık |
| Çağrı | Büyükkasap |
| Dmitrii | Buzanakov |
| M | Buzejic |
| Kefas | Bwala |
| KJ | Bwala |
| Matthew | Bye |
| F | Byiringiro |
| Fidele | Byiringiro |
| J | Byrne |
| Matthew | Byrne |
| MH V | Byrne |
| Edward P | Bywater |
| Savitha | C |
| JM | Cabada Lee |
| N | Caballero Otálora |
| Alejandra | Caballero salas |
| VD | Caballero Sarabia |
| J | Caballero-Alvarado |
| Alberto | Cabañero Sánchez |
| Pedro | Cabeça Santos |
| A | Cabeleira |
| Carmen | Cabeza Oliver |
| K | Cabillas |
| Ana | Cabral |
| Daniel | Cabreja |
| Marino | Cabrera |
| PA | Cabrera |
| Wilton | Cabrera Cruz |
| PA | Cabrera Rivera |
| E | Cabrini |
| Elisa | Cabrini |
| R | Cabula |
| M | Caccetta |
| Crescenzo | Cacciapuoti |
| NA | Cáceres Cárdenas |
| LE | Cadena Castro |
| P | Cadenelli |
| Pierfrancesco | Cadenelli |
| A | Cadersa |
| Luciana | Cadore Stefani |
| Sarah | Cadwell-Sneath |
| S | Cafarotti |
| M | Caffo |
| Maria | Caffo |
| EP | Cagigal Ortega |
| Deniz | Caglar |
| Mehmet | Çağlar Çakıcı |
| Elena | Cagnazzi |
| Hüseyin | Cahit Yalçın |
| R | Cahyono |
| Tommaso | Cai |
| A | Caiado |
| André | Caiado |
| Camilo | Caicedo |
| Lina | Caicedo |
| M | Caicedo Toro |
| Bartomeu | Caimari |
| François | Caire |
| Alison | Cairns |
| Scott | Cairns |
| P | Caja Vivancos |
| Patricia | Caja Vivancos |
| Ensar | Çakır |
| G | Cakmak |
| Guner | Cakmak |
| Gül | Çakmak |
| F | Calabrese |
| F | Calabretto |
| M | Calabrò |
| Marcello | Calabrò |
| E | Calcerrada Alises |
| Marta | Calderón |
| Aranzazu | Calero-Lillo |
| B | Calik |
| Bulent | Calik |
| F | Calikoglu |
| Fikret | Calikoglu |
| Ana M | Calinescu |
| G | Calini |
| Ş | Çalık |
| AS | Çalış |
| MP | Callahan |
| R | Callan |
| C | Callari |
| Cosimo | Callari |
| R | Callcut |
| Rachael | Callcut |
| Paola | Calleja Hermosa |
| Laura | Calles-Sastre |
| Alexandra | Calmels |
| V | Calu |
| Valentin | Calu |
| J | Calvache |
| JA | Calvache |
| Jose | Calvache |
| Jorge | Calvera |
| P | Calvo Espino |
| Pablo | Calvo Espino |
| Marta | Calvo Fernández |
| Raul | Calvo Gonzalez |
| A | Calvo Rey |
| Jitoko | Cama |
| C | Camacho |
| Aldo | Camacho Gomez |
| F | Camacho Zacarías |
| P | Camacho-Carrasco |
| Diego | Camacho-Nieto |
| Jaume | Cámara Cabrera |
| Marina | Cámara Vallejo |
| E | Camarero |
| Enrique | Camarero Rodríguez |
| Daniela | Camargo Gómez |
| K | Camargo-Parra |
| Marta | Camats Terré |
| William | Cambridge |
| Amisha | Cameron |
| Iain | Cameron |
| RB | Cameron |
| Robert B | Cameron |
| Joan | Camí |
| María | Camila Carvajal |
| María | Camila Leyva Martínez |
| April | Camilla Roslani |
| A | Camillo |
| Juan | Camilo Salcedo Moreno |
| NG | Caminsky |
| E | Cammarata |
| Emanuele | Cammarata |
| F | Cammarata |
| L | Camp |
| Lauren | Camp |
| Khaled | Campa |
| Luca | Campagnaro |
| T | Campagnaro |
| Tommaso | Campagnaro |
| N | Campain |
| Sofia | Campanella |
| M | Campanelli |
| Michela | Campanelli |
| Abigail | Campbell |
| Cassidy | Campbell |
| Katie | Campbell |
| R | Campbell |
| W | Campbell |
| William | Campbell |
| Paula | Campelos Fernández |
| P | Campennì |
| Paola | Campennì |
| Flaminia | Campo |
| Ana | Campos |
| J | Campos |
| Jose | Campos |
| Elena | Campos Carot |
| Juan | Campos Garcia |
| FE | Campos Montoya |
| N | Campuzano |
| Nicolás | Campuzano |
| Borja | Campuzano Bitterling |
| B | Campuzano-Bitterling |
| M | Camuera |
| Maite | Camuera |
| U | Can |
| Ugur | Can Dulger |
| Ahmet | Can Sarı |
| Ozan | Can Tatar |
| MªPilar | Canals Sin |
| A | Canas-Martinez |
| B | Canbay Torun |
| M | Candan |
| Mert | Candan |
| Susana | Candeias Rodrigues |
| M | Candiani |
| Massimo | Candiani |
| Giorgio | Candotti |
| E | Canelles Corell |
| Ruben | Canelo Professor |
| C | Canhoto |
| Dalibor | Cankoski |
| J | Cann |
| A | Cannavera |
| Alessandro | Cannavera Putzu |
| M | Cannoletta |
| O | Cano |
| V | Cano Busnelli |
| Virginia | Cano Busnelli |
| J | Caño Velasco |
| Jorge | Caño Velasco |
| E | Cano-Trigueros |
| Emiliano | Cano-Trigueros |
| A | Canonico |
| G | Canonico |
| Giuseppe | Canonico |
| Rita | Canotilho |
| Samir | Canovic |
| Elif | Cansu Gundogdu |
| M | Cantalejo-Diaz |
| Daniel | Cantero |
| Miriam | Cantos |
| Stephen | Canty |
| Ulrich | Canzler |
| Daniela | Canzonieri |
| H | Cao |
| R | Capanna |
| B | Capdevila Vilaro |
| B | Capdevila Vilaró |
| Blanca | Capdevila Vilaró |
| Nathalia | Capellan |
| P | Capelli |
| L | Capezzuoli |
| Daniel | Capitaine |
| L | Capitan-Morales |
| Luis-Cristobal | Capitan-Morales |
| P | Capitani |
| H | Capitelli-McMahon |
| V | Capizzi |
| Giampiero | Capobianco |
| R | Capoglu |
| Recayi | Çapoğlu |
| GT | Capolupo |
| Serge | Cappeliez |
| Alessandro | Cappellani |
| Antonio | Cappiello |
| Giovanni | Capretti |
| M | Caputo |
| Maria | Caputo |
| Massimo | Caputo |
| M | Capuzzo Gonçalves |
| Mateus | Capuzzo Gonçalves |
| A | Carabias |
| Petru | Caraja |
| F | Carannante |
| Filippo | Carannante |
| C | Carapinha |
| Andrea | Caravati |
| Inez | Carballo |
| MI | Carballo |
| F | Carbone |
| L | Carbone |
| E | Carbonneau |
| G | Carcano |
| P | Carcoforo |
| Stefano | Cardelli |
| Daniel | Cardenas |
| T | Cardenas |
| Jimmy | Cárdenas Coaquira |
| F | Cárdenas Escalante |
| Fernando | Cárdenas Escalante |
| Laura | Cárdenas Puiggrós |
| D | Cárdenas Ruiz de Castilla |
| J | Cardenas-Gomez |
| Kristin | Cardiel Nunez |
| Etienne | Cardinal |
| Luca | Cardinali |
| Uriel | Cardona |
| Monica | Cardona Marin |
| Cláudio | Cardoso |
| J | Cardoso |
| N | Cardoso |
| Nicole | Cardoso |
| P | Cardoso |
| Paulo | Cardoso |
| Andre | Cardoso Almeida |
| Roberto | Cardoso Cardoso dos Santos |
| Fabiana | Cardoso Pereira Valera |
| Cintia | Cardoso Pinheiro |
| M | Caretto |
| Iva | Carevic |
| C | Carey |
| Charles | Carey |
| M | Caricato |
| Marco | Caricato |
| Jochem | Caris |
| F | Carissimi |
| Giuseppe | Caristo |
| Janaína | Carla da Silva |
| Renan | Carlo Colombari |
| Giorgio | Carlo Ginesu |
| Luca | Carlo Nespoli |
| A | Carlos |
| W | Carlos |
| William | Carlos |
| Jose | Carlos Barcelon |
| Juan | Carlos Bernal-Sprekelsen |
| Juan | Carlos Catalá Bauset |
| João | Carlos Costa de Oliveira |
| Juan | Carlos Dueñas-Ramirez |
| Juan | Carlos Enciso |
| Juan | Carlos Ibarrola Peña |
| Juan | Carlos Martín del Olmo |
| Juan | Carlos Navarro |
| Juan | Carlos Rodríguez-Sanjuán |
| Juan | Carlos Sabogal Olarte |
| P | Carlos Santos |
| Laura | Carlson |
| M | Carlucci |
| Ofra | Carmel |
| Maria | Carmela Giuffrida |
| Marie | Carmela Lapitan |
| Ana | Carmen Carbajal |
| María | Carmen Cervera |
| Maria | Carmen Suescun López |
| H | Carmichael |
| Heather | Carmichael |
| Alyssa | Carmina Almelor |
| Tomas | Carminatti |
| Lizette | Carmona |
| A | Carnevali |
| Adriano | Carnevali |
| E | Carnicer Escusol |
| Esmeralda | Carnicer Escusol |
| A | Caro |
| Cristina | Caroça |
| Maria Carolina | Castillo Florez |
| Andrea Carolina | Perea Serna |
| Ana Carolina | Scintini Herbst |
| Ana Carolina | Tagliatti Zani |
| Amanda Caroline | Dawson |
| Diana | Carpaneto |
| Osvaldo | Carpineto Samorani |
| Antonio | Carpino |
| YT | Carpio Colmenares |
| S | Carrabetta |
| R | Carramiñana Nuño |
| FM | Carrano |
| J | Carranza Sarmina |
| Barbara | Carrara |
| Yolanda | Carrascal |
| M | Carrasco Prats |
| Milagros | Carrasco Prats |
| M | Carrasco-Prats |
| Carla | Carratalá Pérez |
| R | Carreira Garcia |
| Carolina | Carreiro |
| Guillermo | Carreño |
| Jacqueline | Carrera |
| A | Carreras-Castañer |
| Anna | Carreras-Castañer |
| Alessandro | Carretta |
| MM | Carrick |
| FM | Carrier |
| M | Carrillo-Rivas |
| Mariana | Carrillo-Rivas |
| E | Carrington |
| Diego | Carrion |
| Conor | Carroll |
| Jesse | Carroll |
| PA | Carroll |
| Paul | Carroll |
| Daniel | Carson |
| Samuel | Carson |
| WS | Cartagena |
| J | Carter |
| Clifford | Caruana |
| Edward | Caruana |
| Edward J | Caruana |
| EJ | Caruana |
| Ed | Caruna |
| Ambra | Caruso |
| Gerardo | Caruso |
| J | Caruso |
| James | Caruso |
| Carolina | Carvajal Calderón |
| AA | Carvalho |
| GB | Carvalho |
| Joanna | Carvalho |
| L | Carvalho |
| M | Carvalho |
| MF | Carvalho |
| VC | Carvalho |
| Vladimir C | Carvalho |
| MM | Carvello |
| Karen | Carver |
| Shea | Carver |
| M | Carvill |
| Riccardo | Casadei |
| Alfonso | Casado |
| Biagio | Casagranda |
| Mauro | Casagrande |
| Núria | Casanova Torrequebrada |
| Andrea | Casaril |
| J | Casarin |
| Claudia | Casarini |
| Estefania | Casas |
| Felipe | Casas J |
| Marcos | Casas Sánchez |
| M | Casati |
| Massimiliano | Casati |
| Ottavia | Caserini |
| L | Casetti |
| R | Casey |
| Rowan | Casey |
| Arianna | Casiraghi |
| T | Casiraghi |
| Florence | Caslake Holding |
| Gianmaria | Casoni Pattacini |
| C | Cassinello |
| D | Cassini |
| E | Cassinotti |
| Elisa | Cassinotti |
| Francesco | Castagnini |
| Antonio | Castaldi |
| Néstor | Castán Villanueva |
| W | Castañeda |
| Ivan | Castañeda Giacometto |
| António | Castanheira |
| S | Castanheira Rodrigues |
| Sara | Castanheira Rodrigues |
| Mario | Castaño |
| AM | Castaño-Leon |
| Ana M | Castaño-Leon |
| Fabiola | Castedo |
| Rute | Castelhano |
| Christoph | Castellani |
| J | Castellanos |
| A | Castells |
| K | Castillo |
| Maria | Castillo |
| Laura | Castillo Pardo |
| Cameron | Castle |
| FB | Casto |
| C | Castoro |
| Carlo | Castoro |
| EJ | Castro |
| Emma | Castro |
| Marinelle | Castro |
| S | Castro |
| Filipe | Castro Borges |
| Beatriz | Castro Catalan |
| R | Castro de la Mata |
| C | Castro Ruiz |
| M | Castro Suárez |
| Marta | Castro Suárez |
| F | Castronovo |
| JC | Catalá Bauset |
| V | Catalán |
| Sandra | Cataldi |
| Sergiu | Catalin Baraian |
| Ioan | Catalin Vlad |
| J | Catarino |
| L | Catarzi |
| Lisa | Catarzi |
| Mariano | Catello Di Donna |
| F | Catena |
| Fausto | Catena |
| Andrew | Caterson |
| N | Cathala |
| Nathalie | Cathala |
| Russell | Cathcart |
| X | Cathelineau |
| Valerie | Catherine Linz |
| LD | Cato |
| Bruno | Catoia fonseca |
| S | Cattaneo |
| J | Catto |
| James | Catto |
| A | Catton |
| MM | Caubet |
| M | Cauteruccio |
| Michele | Cauteruccio |
| A | Cavalea |
| Alexander | Cavalea |
| S | Cavaleiro |
| Davide | Cavaliere |
| P | Cavallé Busquets |
| Matteo | Cavallo |
| Francesca | Cavenago |
| L | Cayetano Paniagua |
| Ladislao | Cayetano Paniagua |
| Valentín | Cayuela |
| M | Cazador Labat |
| Antoine | Cazelles |
| A | Caziuc |
| R | Cazzaniga |
| S | Cebi |
| Sukru | Cebi |
| Laura | Cebolla Rojas |
| L | Cecchini |
| I | Cecconello |
| E | Cehic |
| Albertas | Cekauskas |
| F | Celebi |
| Fehmi | Çelebi |
| AP | Celi-De La Torre |
| B | Celik |
| P | Cellerino |
| Paola | Cellerino |
| Claudia | Celotti |
| Ahmet | Cem Dural |
| S | Cenciarelli |
| Sabine | Cenciarelli |
| Rosario | Cennamo |
| Omer | Cennet |
| Ana | Centeno Álvarez |
| Jorge | Centeno Lozada |
| Alvaro | Centeno Velasco |
| Jessica | Centurión |
| Carmen | Cepeda-Franco |
| G | Cepele |
| Miljan | Ceranic |
| Marco | Cereda |
| Julian | Cereghini |
| Ülkü | Ceren Köksoy |
| Marco | Ceresoli |
| Petra | Čerina |
| C | Cernei |
| Ondrej | Cerny |
| Jan | Černý |
| A | Cerovac |
| Anis | Cerovac |
| Cristina | Cerri |
| C | Cerro Zaballos |
| M | Cervellera |
| I | Cervera |
| Iria | Cervera |
| S | Cervera |
| Sergio | Cervera Bonilla |
| Andrea | Cerveró |
| Laura | Cervini |
| Giovanni | Cesana |
| Andrei | Cesar Abella |
| Pablo | Cesar Arteaga Asensio |
| Francisco | César Becerra García |
| Matteo | Cescon |
| Z | Çetinkaya |
| Mehmet | Ceyhan |
| Hilal | Chaaban |
| Abou | Chaar |
| Mohammad | Chaar |
| Reem | Chabaan |
| Carolyn | Chabuz |
| S | Chackan |
| Hannah | Chacon |
| R | Chadha |
| Radhika | Chadha |
| S | Chadha |
| Sami | Chadi |
| T | Chaki |
| Tomohiro | Chaki |
| Sohini | Chakrabortee |
| Koyel | Chakraborty |
| Marc | Chalhoub |
| B | Challacombe |
| D | Chalo |
| Alexandre | Chamouni |
| Pierre-Olivier | Champagne |
| Albert | Chan |
| Annie | Chan |
| Carlos | Chan |
| CD | Chan |
| CH | Chan |
| Corey | Chan |
| Elliot | Chan |
| Eunice | Chan |
| J | Chan |
| L | Chan |
| M | Chan |
| Matthew | Chan |
| S | Chan |
| Bruno | Chan Chin |
| Aye | Chan Thu |
| Prem | Chana |
| Gyan | Chand |
| M | Chand |
| Kshitija | Chandanwale |
| K | Chandarana |
| Karishma | Chandarana |
| Danny | Chandla |
| Amarbaj | Chandock |
| Susilo | Chandra |
| Karthik | Chandra Vallam |
| C | Chandrakumar |
| Bhargavi | Chandrasekar |
| Pramodh | Chandrasinghe |
| N | Chandratreya |
| Nitya | Chandratreya |
| G | Chang |
| Grace | Chang |
| Steven | Chang |
| Abera | Chanie |
| K M M vishvak | Chanthar |
| H | Chanty |
| Lidya | Chanyalew |
| Ghita | Chaoui |
| Reema | Chapatwala |
| BK | Chaplin |
| Brandon | Chapman |
| Tracy | Chapman |
| Preetam | Chappity |
| A | Charalabopoulos |
| Alexandros | Charalabopoulos |
| Vasileios | Charalampakis |
| H | Charbonneau |
| Helene | Charbonneau |
| Eduard | Charchyan |
| L | Chardalias |
| Leonidas | Chardalias |
| A | Chari |
| S | Charles |
| Shane | Charles |
| Gabriella | Charlton |
| L | Charre |
| T | Chartab Mohammadi |
| Emmanuel | Chartier-Kastler |
| T | Chase |
| Muhammad | Chatni |
| Debarshi | Chatterjee |
| S | Chatterji |
| Somashree | Chatterji |
| Somnath | Chattopadhyay |
| A | Chaturvedi |
| Arun | Chaturvedi |
| Arvind | Chaturvedi |
| P | Chatzikomnitsa |
| M | Chatzikonstantinou |
| Sohin | Chaudhari |
| Vikram | Chaudhari |
| Ramkaran | Chaudhary |
| S | Chaudhary |
| Umer | Chaudhry |
| Madhu | Chaudhury |
| H | Chaudry |
| G | Chauhan |
| Sandeep | Chauhan |
| Akhilanand | Chaurasia |
| Rahul | Chavan |
| N | Chavarrias |
| Nuria | Chavarrias |
| Vijay | Chavda |
| G | Chavez |
| J | Chávez Pacheco |
| C | Chavez Rivaldi |
| Cristhian | Chavez Rivaldi |
| Sergio | Chávez Valladares |
| Petr | Chavkin |
| Priyank | Chawathe |
| Shahidah | Che Alhadi |
| NA | Che Bakri |
| James | Chean Khun Ng |
| A | Chebaro |
| Alexandre | Chebaro |
| Michel | Chebel |
| C | Checcucci |
| Carlotta | Checcucci |
| SY | Chee |
| Nabeel | Cheema |
| R | Chelva |
| Ruth | Chelva |
| A | Chen |
| D | Chen |
| F | Chen |
| Ji | Chen |
| John | Chen |
| Lee-lynn | Chen |
| Lee-may | Chen |
| Paul | Chen |
| Pengchi | Chen |
| Si | Chen |
| Tony | Chen |
| Yuan | Chen |
| Zehua | Chen |
| Arthur | Chen Wun Tan |
| Jade | Chen Zhao |
| D | Cheng |
| Davy | Cheng |
| Antony | Chengahomwe |
| Martin | Chenu |
| YJ | Cheong |
| Wai | Cheong Soon |
| Nathalie | Chereau |
| Lisa | Cherian |
| O | Cherkaoui |
| Zineb | Cherkaoui |
| Huey-Lan | Chern |
| Viktor | Cherniienko |
| Roman | Chernikov |
| Maxim | Chernykh |
| Davies | Cheruiyot |
| Jayant | Cherukat |
| E | Cherullo |
| A | Chessa |
| Antonella | Chessa |
| G | Chetty |
| J | Cheuk |
| J | Cheung |
| Kin | Cheung Ng |
| M | Chevallay |
| Dylan | Chew |
| Kenneth | Chew |
| M | Chew |
| Michelle | Chew |
| Natalie | Cheyne |
| Ernest | Cheyuo |
| HS | Chhabra |
| R | Chhabra |
| Swati | Chhatrapati |
| David | Chi Hau Tan |
| Leslie | Chi Yan Cheung |
| Z | Chia |
| Zoe | Chia |
| C | Chiang |
| Chu-Hao | Chiang |
| Ryan | Chiang |
| Vito | Chiantera |
| Valentina | Chiappa |
| C | Chiappe |
| MF | Chiappetta |
| A | Chiappini |
| Marco | Chiarelli |
| R | Chiarpenello |
| M | Chiarugi |
| Massimo | Chiarugi |
| Yoshihiko | Chiba |
| Ihediwa | Chibuike George |
| S | Chidambaram |
| N | Chidumije |
| Nnaemeka | Chidumije |
| Christopher | Chien Liang Liao |
| Roberto | Chiesa |
| I | Chik |
| Maxwell | Chimhina |
| Theresa | Chin |
| Hiong | Chin Lim |
| John | Chinda |
| Rosanne | Ching |
| Tsz | Ching Chang |
| Simbarashe | Chinyowa |
| Mieko | Chinzei |
| Franco | Chioffi |
| A | Chiow |
| Adrian | Chiow |
| M | Chiozza |
| I | Chipurovski |
| Francesca | Chircop |
| P | Chiriapanda uthappa |
| C | Chirico |
| Carlos | Chirico |
| Radu | Chirvasuta |
| Emiliano | Chisci |
| U | Chishti |
| Uzma | Chishti |
| Meer | Chisthi |
| T | Chituku |
| Tsitsi | Chituku |
| A | Chitul |
| Andrei | Chitul |
| JA | Chiu |
| Shelton | Chivanga |
| C | Choi |
| D | Choi |
| David | Choi |
| Sarah | Choi |
| H | Cholewa |
| Hanna | Cholewa |
| Alyssa | Chong |
| C | Chong |
| L | Chong |
| Lynn | Chong |
| Yew-Lam | Chong |
| C | Choo |
| Candy | Choo |
| J | Choo Jun Hao |
| E | Choolani Bhojwani |
| Ekta | Choolani Bhojwani |
| P | Choong |
| Peter | Choong |
| Sophie | Chopinet |
| Rupali | Chopra |
| S | Chopra |
| R | Choron |
| S | Chotai |
| Silky | Chotai |
| Ravi | Chotalia |
| Richard | Chou |
| A | Chouakria |
| Narendra | Choudhary |
| A | Choudhry |
| Asad | Choudhry |
| Yousuf | Choudhury |
| RS | Chouhan |
| E | Chouillard |
| Elie | Chouillard |
| MF | Chowdhry |
| A | Chowdhury |
| Abeed | Chowdhury |
| M | Chowdhury |
| Mahbub | Chowdhury |
| S | Chowdhury |
| Sharfuddin | Chowdhury |
| Shihab | Chowdhury |
| Nisar | Chowdri |
| D | Chrastek |
| MA | Christensen |
| P | Christensen |
| Peter | Christensen |
| Bordianu | Christian |
| Nicole | Christian |
| Franz | Christian Horstmeier |
| Johannes | Christian Lauscher |
| Jean | Christian Urimubabo |
| A | Christiano |
| P | Christidis |
| Panagiotis | Christidis |
| Adam | Christie |
| Julia | Christina Kaiser |
| Ekaterini | Christina Tampaki |
| Elaine | Christine Dantas Moises |
| Ruth | Christine Schäfer |
| Gregory | Christodoulidis |
| M | Christodoulou |
| D | Christoforidis |
| Dimitrios | Christoforidis |
| Alexander | Christopher Rokohl |
| C | Christou |
| CD | Christou |
| Chrysanthos | Christou |
| N | Christou |
| Niki | Christou |
| Angeli | Christy Yu |
| Megan | Chrysikopoulou |
| E | Chrysos |
| Emmanuel | Chrysos |
| A | Chrysovergis |
| Aristeidis | Chrysovergis |
| G | Chrysovitsiotis |
| Georgios | Chrysovitsiotis |
| F | Chu |
| Francesco | Chu |
| H | Chu |
| K | Chu |
| Andre | Chu Qiao Lo |
| A | Chua |
| HW | Chua |
| Richelle | Chua |
| CT | Chuah |
| Alwin | Chuan |
| Rachel | Chubsey |
| KM | Chue |
| Kwok | Chuen Wong |
| Ankita | Chugh |
| Jason | Chui |
| K | Chui |
| I | Chukwu |
| Isaac | Chukwu |
| Sunil | Chumber |
| Felix | Chun |
| Mohsin | Chundrigar |
| Tariq | Chundrigar |
| C | Chung |
| E | Chung |
| H | Chung |
| J | Chung |
| Raymond | Chung Siang Lim |
| Yuliya | Churina |
| C | Chwat |
| Carina | Chwat |
| Fabio | Cianchi |
| Antonio | Cianci |
| P | Cianci |
| Pasquale | Cianci |
| A | Cianfarani |
| D | Cianflocca |
| Desiree | Cianflocca |
| Marco | Ciappara Paniagua |
| FA | Ciarleglio |
| C | Ciatti |
| Corrado | Ciatti |
| Sandro | Ciccarello |
| Pietro | Ciccarino |
| Flavia | Ciccarone |
| R | Cicco |
| Candan | Cicek |
| PM | Cicerchia |
| M | Ciciliot |
| Marta | Cicuendez López Ocaña |
| Luciana | Cidade Costa |
| MP | Cidón Palacio |
| AB | Ciftci |
| Luca | Cigagna |
| N | Cillara |
| Nicola | Cillara |
| Ángel | Cilleruelo Ramos |
| Bonifacio | Cimadevilla Calvo |
| S | Cimbanassi |
| B | Cimenoglu |
| Berk | Cimenoglu |
| Marilaeta | Cindryani Lolobali |
| Charles | Cini |
| Matteo | Cinquepalmi |
| Juan | Cintas Catena |
| A | Cioci |
| Alessia | Cioci |
| SP B | Cioffi |
| SPB | Cioffi |
| E | Ciofic |
| Tommaso | Cipolat Mis |
| Alessandro | Cipolli |
| Federica | Cipriani |
| R | Cipriani |
| Riccardo | Cipriani |
| V | Ciriello |
| B | Cirillo |
| Bruno | Cirillo |
| N | Cirocchi |
| B | Cismasiu |
| Brigitta | Cismasiu |
| G | Cisternino |
| B | Citgez |
| D | Citterio |
| Davide | Citterio |
| C | Ciubotaru |
| Cezar | Ciubotaru |
| Agne | Cizauskaite |
| H | Claireaux |
| Cillian | Clancy |
| H | Clancy |
| Maria | Clara Mendoza Arango |
| Ana | Clara Valerio |
| Olga | Claramonte Bellmunt |
| Guillem | Claret |
| G | Clarizia |
| Guglielmo | Clarizia |
| J | Clark |
| Julie | Clark |
| Mhairi | Clark |
| Sara | Clark |
| Simon | Clark |
| Eleanore | Clark-Mackay |
| Eleanor | Clarke |
| EM | Clarke |
| Gareth | Clarke |
| Theo | Clarke |
| M | Claro |
| Mariana | Claro |
| Sharnel | Clatworthy |
| Marie | Claude Renaud |
| Ana | Cláudia Deus |
| Muhawenimana | Claudine |
| Oliver | Claydon |
| Sean | Cleary |
| Joe-Nat | Clegg-Lamptey |
| G | Clemen |
| Quentin | Clemens |
| M | Clementi |
| Laura | Clementoni |
| P | Clermidi |
| Pauline | Clermidi |
| R | Clifford |
| Zoe | Clifford |
| Paballo | Clinton Khaeane |
| Octavian | Clonda |
| E | Clough |
| Ethan | Clough |
| Jonathan | Cloutier |
| AL | Clynch |
| Daniel | Coakley |
| Amy | Coates |
| C | Cobelschi |
| L | Cobianchi |
| Lorenzo | Cobianchi |
| M | Coburn |
| Mark | Coburn |
| L | Cocchi |
| F | Coccolini |
| Federico | Coccolini |
| Angela | Cochrane |
| Elliott | Cochrane |
| AJ | Cockbain |
| E | Cocozza |
| Eugenio | Cocozza |
| Clara | Codony |
| Peter | Coe |
| JC | Coffey |
| Daniel | Cohen |
| Oliver | Cohen |
| Olivia | Cohen |
| David | Cohn |
| T | Cohnert |
| Tina | Cohnert |
| TU | Cohnert |
| FJ F | Coimbra |
| Saverio | Coiro |
| JM | Cojulun |
| Jose | Cojulun |
| JM | Cojulun Barrera |
| A | Cokan |
| Andrej | Cokan |
| Sara | Cokarić |
| N | Cokleska Shuntov |
| Natalija | Cokleska Shuntov |
| Ashley | Colaco |
| E | Colak |
| Eli̇f | Çolak |
| MK | Colakoglu |
| Simone | Colangeli |
| L | Colao García |
| Laura | Colao García |
| Pierre-Antoine | Colas |
| E | Colás-Ruiz |
| Enrique | Colás-Ruiz |
| Marco | Colasanti |
| Roberto | Colasanti |
| J | Coleman |
| Julia | Coleman |
| NL | Coleman |
| Laura | Colet Oliver |
| Katerine | Colina |
| J | Colina Casas |
| M | Colino |
| JA | Collantes Cubas |
| B | Collard |
| M | Colledan |
| P | Collera |
| Pablo | Collera |
| G | Colletti |
| Gaia | Colletti |
| Tom | Collicott |
| Amber | Collier |
| T | Collier |
| KP | Colling |
| Kristin | Colling |
| CG | Collins |
| Chris | Collins |
| Emma | Collins |
| Michelle L | Collins |
| ML | Collins |
| Rachael | Collins |
| J | Collis |
| F | Colombo |
| Francesco | Colombo |
| Giovanni | Colombo |
| Anthony | Colon |
| E | Colonna |
| Emily | Colonna |
| ET | Colonna |
| A | Colquhoun |
| Pablo | Colsa |
| N | Colucci |
| CA | Colunga Tinajero |
| Gary | Colville |
| HV | Colvin |
| T | Combellack |
| Tom | Combellack |
| LV | Comini |
| Amanda | Compadre |
| B | Compagnoni |
| Bruno | Compagnoni |
| MDP | Concejo Cutoli |
| María | concepcion Alonso González |
| V | Concepción Martín |
| S | Conci |
| Simone | Conci |
| Odilia | Conde |
| Danny | Conde Monroy |
| M | Condon |
| Melissa | Condon |
| Sarah | Condron |
| Ana | Conesa |
| Anna | Conesa |
| M | Confalonieri |
| K | Conlon |
| Tara | Connelly |
| TM | Connelly |
| E | Connolly |
| H | Connolly |
| Michael | Connolly |
| Patricia | Conroy |
| Soraya | Conroy |
| Shane | Considine |
| C | Conso |
| Christel | Conso |
| E | Consorti |
| G | Consorti |
| Giuseppe | Consorti |
| J | Constable |
| Houlzé-Laroye | Constance |
| Raquel | Constantino-duarte |
| Monica | Contador |
| Alfredo | Conti |
| L | Conti |
| Lorenzo | Conti |
| Luigi | Conti |
| Ioannis | Contis |
| Elisa | Contreras Saiz |
| Luca | Contu |
| I | Conversano |
| P | Cook |
| Fiachra | Cooke |
| Paul | Cool |
| E | Coomber |
| A | Coonar |
| Aman | Coonar |
| M | Cooper |
| S | Cooper |
| Z | Cooper |
| Zara | Cooper |
| D | Cope |
| Daron | Cope |
| Jennifer | Cope |
| Chiara | Copelli |
| D | Copur |
| Francesco | Coratti |
| C | Corbellini |
| Carlo | Corbellini |
| Harriet | Corbett |
| HJ | Corbett |
| Lisa | Corbiere |
| Sasha | Corbin |
| Francesco | Corcione |
| Marta | Córcoles |
| F | Cordera |
| Fernando | Cordera |
| Saray | Cordero Spencer |
| Timothy | Cordingley |
| A | Cordonnier |
| A | Cordova |
| Adriana | Cordova |
| Diego | Córdova García |
| E | Cordova-Calle |
| Fernando | Corella |
| Guido | Coretti |
| Joel | Corkill |
| Valeria | Cormane Alfaro |
| Tommaso | Cornali |
| DK | Cornelio |
| Philip | Cornford |
| J | Cornish |
| C | Cornwell |
| D | Corona |
| Jose | Corona-Cruz |
| M | Coronas Soucheiron |
| Maria | Coronas Soucheiron |
| Alma-Andreea | Corpodean |
| Paula | Corr |
| Eva | Corral Rubio |
| A | Correa Bonito |
| Alba | Correa Bonito |
| AM | Correia |
| Bernardo | Correia |
| J | Correia |
| R | Correia |
| Rebeca | Correia |
| RM | Correia |
| S | Correia |
| Igor | Correia de Farias |
| Tiago | Correia de Sá |
| T | Correia-de-Sá |
| M | Corrigan |
| Mark | Corrigan |
| Patricia | Corriols Noval |
| Ramon | Corripio-Sanchez |
| Simon | Corriveau-Durand |
| Julian | Corso |
| Henry | Cortes |
| Ludivina | Cortes |
| Ruben | Cortes |
| Susana | Cortes |
| D | Cortés Guiral |
| Natalia | Cortes Murgueitio |
| D | Cortés-Guiral |
| Delia | Cortés-Guiral |
| C | Cortes-Mora |
| Edgar-Joaquin | Cortes-Torres |
| Sara | Cortinovis |
| Umberto | Cortinovis |
| Jordan | Cory |
| M | Cosimelli |
| Maurizio | Cosimelli |
| R | Cosker |
| M | Coşkun |
| Codrut | Cosmin Nistor-Ciurba |
| Francesco | Costa |
| Francisca | Costa |
| Laura | Costa |
| M | Costa |
| MJ M A | Costa |
| Tainá | Costa |
| Brena | Costa dos Santos |
| Fernanda | Costa Pereira |
| D | Costa Santos |
| Daniel | Costa Santos |
| Rui | Costa Soares |
| Andreea | Costache |
| Victor | Costache |
| S | Costantini |
| Andrea | Costantino |
| Caterina | Costanza Zingaretti |
| A | Costanzi |
| Andrea | Costanzi |
| F | Costanzo |
| A | Costas-Chavarri |
| Ainhoa | Costas-Chavarri |
| Simbad | Costas-Ochoa |
| P | Coste Mazeau |
| R | Costea |
| Radu | Costea |
| B | Costeira |
| Beatriz | Costeira |
| Cristina | Costeira |
| Rad | Costel Claudiu |
| O | Costerousse |
| Renato | Costi |
| Mathieu | Cote |
| Maxime | Cote |
| G | Côté |
| Mathilde | Côté |
| A | Cotoia |
| Antonella | Cotoia |
| C | Cotsoglou |
| Estefanía | Cotta |
| E | Cotte |
| Tatiana | Cottin |
| O | Cottle |
| Pietrina | Cottu |
| J | Couch |
| P | Coughlin |
| Patrick | Coughlin |
| R | Coulson |
| Valérie | Courval |
| Ianis | Cousin |
| Mariana | Couto Bártolo |
| E | Couture |
| Brendon | Coventry |
| Sarah | Cowan |
| Aram | Cox |
| Daniel | Cox |
| DR A | Cox |
| G | Cox |
| India | Cox |
| Shanice | Cox |
| Joanne | Cozens |
| V | Cozza |
| Valerio | Cozza |
| Federico | Cozzani |
| Emily | Crane |
| M | Crank |
| A | Craus-Miguel |
| Andrea | Craus-Miguel |
| Joanna | Craven |
| J | Crawford |
| Emily | Crawley |
| Simon | Craxford |
| M | Creanga |
| B | Creavin |
| Ben | Creavin |
| Diana | Crego Vita |
| D | Crego-Vita |
| Camilla | Cremonini |
| V | Crenn |
| Vincent | Crenn |
| Giacomo | Crescentini |
| R | Cresner |
| MS | Crespi Amor |
| Ant`Onia | Crespí Mir |
| A | Crespo |
| Aldo | Crespo |
| J | Crespo |
| Jesús | Crespo-Sanjuán |
| C | Crétolle |
| Célia | Crétolle |
| Benjamin | Cribb |
| Mark | Cribb |
| A | Crichton |
| R | Crichton |
| Peter | Cripps |
| Mambote | Crispin Olivier Ntoto |
| Alessandra | Cristaudi |
| D | Cristian |
| Joana | Cristina Domingues |
| Claudia | Cristina Lopes Moreira |
| María | Cristina Martínez Canto |
| Aida | Cristina Rahy-Martín |
| J | Cristini |
| Ignacio | Cristobal |
| Lodovica | Cristofani Mencacci |
| MG | Cristofaro |
| Rebecca | Critchley |
| Sergiu | Crivat |
| Bojana | Crnobrnja |
| B | Crnokrak |
| RS | Croattini |
| Daniele | Crocetti |
| SM | Croghan |
| Stefanie M | Croghan |
| Gheorghe | Croitor |
| Cristina | Croitoru |
| A | Cromi |
| Rachael | Crompton |
| P | Cromwell |
| Lauren | Crone |
| R | Croner |
| Roland | Croner |
| B | Cros |
| Beatriz | Cros |
| GW V | Cross |
| Katie | Cross |
| Rebecca | Crothers |
| Clare | Crowley |
| R | Crowley |
| AS | Crugnale |
| EM | Cruz |
| Joao | Cruz |
| L | Cruz |
| A | Cruz Cidoncha |
| Maria | Cruz Iglesias Moreno |
| Octavio | Cruz-Pineda |
| A | Cuadrado-Garcia |
| Angel | Cuadrado-García |
| María | Cuaresma |
| Aldrin | Cuasay |
| Daniela | Cubek |
| Eugenio | Cucinotta |
| Ciprian | Cucoreanu |
| Osmar | Cuenca |
| Miguel | Cuende Diez |
| M | Cuesta Argos |
| Mario | Cuesta Argos |
| FJ | Cuesta-González |
| Francisco J | Cuesta-González |
| MA C | Cueto |
| AE | Cueto Valadez |
| TA | Cueto Valadez |
| Esteban | Cueva-Martinez |
| Ainhoa | Cuevas |
| Gabriella | Cuevas Lantigua |
| I | Cujiño |
| Indira | Cujiño |
| V | Cuk |
| Vladica | Cuk |
| M | Cukier |
| Moises | Cukier |
| Serdar | Culcu |
| Ivana | Čuljak Blagojević |
| Ivor | Cullen |
| James | Cullen |
| C | Cullinane |
| Carolyn | Cullinane |
| P | Cullis |
| Paul | Cullis |
| O | Cullivan |
| SL | Cumine |
| T | Cuming |
| A | Cumpstey |
| Wang | Cunchuan |
| C | Cunha |
| MF | Cunha |
| Miguel | Cunha |
| Miguel F | Cunha |
| A | Cunha Viana Júnior |
| RM | Cunningham |
| Y | Cunningham |
| T | Curl-Roper |
| G | Currò |
| C | Currow |
| Chelise | Currow |
| T | Curry |
| Terry | Curry |
| C | Cursiefen |
| Claus | Cursiefen |
| Alexa | Curtis |
| M | Curtis |
| Nathan | Curtis |
| Carolina | Curtis Martínez |
| C | Curtis-Martínez |
| JM | Curvas |
| J | Cuschieri |
| R | Cuthbert |
| Rory | Cuthbert |
| C | Cutolo |
| V | Cvetanovska Naunova |
| A | Cvetkovic |
| Ana | Cvetkovic |
| W | Cymes |
| L | Czako |
| Ladislav | Czako |
| Louie | Czelline De Leon |
| W | Czerniak |
| Viktoria | Czok |
| Amanda | Czyz |
| F | D’acapito |
| Alonço | da Cunha Viana Júnior |
| D | Da Luz |
| AM R | da Silva |
| C | Da Silva |
| Cassia | da Silva |
| T | Da Silva |
| R | Da Silva Freitas |
| L | Da Silveira Botacin |
| A | Daadipour |
| Dennis | Daary |
| Ezeddin | Dabbagh |
| Payman | Dabirmoghaddam |
| John | Dabis |
| Latif | Daboo Salifu |
| F | Dacapito |
| Fabrizio | Dacapito |
| Niccolo | Daddi |
| JL | DAddino |
| Shailja | Dadhich |
| Dora | Dadoe |
| Nikos | Dafnios |
| Hattan | Dagestani |
| Yaquob | Daghriri |
| T | Dagklis |
| Themistoklis | Dagklis |
| M | DAgruma |
| Lara | Daham |
| Hiba | Dahhan |
| Abdifatah | Dahir Ali |
| Nick | Dai |
| E | Dainius |
| Edvinas | Dainius |
| K | Dajani |
| Khaled | Dajani |
| I | Dajti |
| Irida | Dajti |
| Elorm | Daketsey |
| S | Dakpé |
| Stéphanie | Dakpé |
| Anuj | Dalal |
| R | Dalal |
| Mohammed | Dalaleh |
| Tyson | Dale |
| Valentina | Dalessandro |
| Rossella | DAlessio |
| B | Daley |
| Brian | Daley |
| S | Dalgleish |
| Stephen | Dalgleish |
| Lucrezia | DAlimonte |
| P | Daliya |
| Iacopo | Dallan |
| Matias | Dallaserra |
| Khadija | Dalmar |
| E | Dalmasso |
| Giordana | DAloisio |
| Francesca | Dalprà |
| A | Daly |
| Catriona | Daly |
| Michael | Damah |
| FA | Damara |
| PD | Damasceno |
| R | Damaseviciute |
| Manuel | Damasio Cotovio |
| Z | Dambrauskas |
| Zilvinas | Dambrauskas |
| Giancarlo | Dambrosio |
| Nurullah | Damburacı |
| R | Damian |
| Maria | Damico |
| Ana | Damjanović |
| B | Danaei |
| Nyirasebura | Dancilla |
| G | DAndrea |
| Giancarlo | DAndrea |
| Marcello | DAndrea |
| Vito | Dandrea |
| C | Dandurand |
| Charlotte | Dandurand |
| Piergiorgio | Danelli |
| G | DAngelo |
| M | Danguy des Déserts |
| Marc | Danguy des Déserts |
| Aghyad | Danial |
| Syed | Danial Syed Ahmad |
| Nikolaos | Danias |
| A | Danic Hadzibegovic |
| Ana | Danic Hadzibegovic |
| D | Daniel |
| James | Daniel |
| Mario | Daniel |
| Toman | Daniel |
| Carlos | Daniel Beyrne |
| Esteban | Daniel Mendoza Galván |
| Ron | Daniel Rivera |
| karla | Daniela Pérez |
| A | Daniele |
| Alberto | Daniele |
| IR | Daniels |
| Emeka | Danielson Odai |
| Daniel | Danielsson |
| Muhammad | Daniyal Daniyal |
| Muhammad | Daniyan |
| Simone | DAnnunzio |
| Mark | Danton |
| Israel | Daodu |
| Muhammad | Daood Daood |
| Mohamed | Daoub |
| Hasan | Daoud |
| Hassan | Daoud |
| Maher | Daoud |
| Reda | Daoud |
| A | Daponte |
| Alexandros | Daponte |
| Jih | Dar Yau |
| F | DAragon |
| J | Daramola |
| Rhea | Darbari Kaul |
| Sabatino | DArchi |
| D | Dardanov |
| Dragomir | Dardanov |
| Vincenzo | Dario Mandato |
| George | Darko Brown |
| Regina | Darko- Asante |
| Lynn | Darragh |
| H | Darraj |
| J | Daruwalla |
| Jurstine | Daruwalla |
| Amr | Darwesh |
| Abdelrhman KZ | Darwish |
| Sara | Darwish |
| Deepu | Daryanani |
| Andre | Das |
| Devishmita | Das |
| Gurudip | Das |
| Mautushi | Das |
| Nivedita | Das |
| R | Das |
| Rishi | Das |
| Shefali | Das |
| Sunit | Das |
| M | Dasa |
| L | Dasanayake |
| Lanka | Dasanayake |
| Mustafa | Dashti |
| Matheus | Dasqueve |
| D | Dass |
| Debashis | Dass |
| Silvio | Däster |
| Avinash | Date |
| Philip | Datler |
| Debajyoti | Datta |
| Rupen | Dattani |
| Mashal | Daud |
| E | Dauer |
| A | Dauksa |
| Albertas | Dauksa |
| Z | Dauksa |
| Giuliana | DAulerio |
| Marta | DAuria |
| Raphaël | Dautry |
| D | Davenport |
| MG | Davey |
| Andras | David |
| Avril | David |
| Bryony | David |
| Martínez | David |
| PZ | David |
| Rotimi | David |
| Shakina | David |
| Alejandro | David Bueno Cañones |
| Godwin | David C Mathew |
| Mamun | David Dornseifer |
| Cesar | David Galindo Regino |
| Parvez | David Haque |
| Jose | David Jimenez Parra |
| Juan | David Lalinde |
| Edward | David Lumley |
| Victor | David Olave Montaño |
| Brayan | David Pedroso Alvarenga |
| José | David Pérez Cajti |
| Juan | David Rivera Garcia |
| Juan | David Saavedra Henao |
| Ever | David Sosa Ferreira |
| Jorge | David Vera Florentin |
| José | Davide |
| M | Davidescu |
| Mihnea | Davidescu |
| S | Davidesko |
| Lazar | Davidovic |
| B | Davidson |
| Brian | Davidson |
| GH | Davidson |
| A | Davies |
| Angharad | Davies |
| Anna | Davies |
| B | Davies |
| Camilla | Davies |
| E | Davies |
| Elinor | Davies |
| GS | Davies |
| James | Davies |
| M | Davies |
| Mark | Davies |
| Peter | Davies |
| RJ | Davies |
| G | Davies-Jones |
| Gareth | Davies-Jones |
| Amelia | Davis |
| NF | Davis |
| Niall | Davis |
| Sean | Davis |
| Timothy | Davis |
| S | Davison |
| Stephen | Davison |
| Anthony | Davor |
| Kaveh | Davoudi |
| Abdulla | Dawaishan |
| Baheya | Dawaishan |
| N | Dawe |
| Nicholas | Dawe |
| Rsheeda | Dawelbait |
| Lujain | Dawod |
| Omar | Dawod |
| Oseyi | Dawodu |
| Christopher | Dawoud |
| AC | Dawson |
| Hannah | Dawson |
| Jonathon | Dawson |
| Joseph | Dawson |
| Pamela | Dawson |
| S | Dawson-Bowling |
| B | Dawud |
| Bashar | Dawud |
| A | Day |
| Arthur | Day |
| Davut | Dayan |
| Julian | Daza |
| Rachel | Dbeis |
| S | Dbouk |
| Samer | Dbouk |
| S | DCruz |
| U | De Andres Olabarria |
| B | De Andrés-Asenjo |
| Beatriz | De Andrés-Asenjo |
| Itziar | De Ariño |
| Alvaro | de Arriba |
| Mario | De Arriba Alonso |
| Gustavo | De Bacco Marangon |
| J | De Barros |
| R | De Berardinis |
| Rita | De Berardinis |
| Henry | de Berker |
| Karina | De Bleecker |
| Yasmine | De Bruyne |
| J | De Ceulaer |
| JN | De Chavez |
| FL | De Cicco |
| Michael | de Cillia |
| J | De Coster |
| E | De Crescenzo |
| Eugenia | De Crescenzo |
| Carlotta | De Cristofaro |
| Francesco | de Falco |
| Francesca | De Felice |
| M | De Francesco |
| A | De Gea Rico |
| L | De Geer |
| Lina | De Geer |
| C | De Gheldere |
| Nine | de Graaf |
| MR | De Graaff |
| Pauleen | de Grano |
| J | De Haro |
| Joaquin | De Haro |
| I | De Haro Jorge |
| Irene | de Haro Jorge |
| T | De Hoop |
| Nicolas | De Hous |
| P | De Iaco |
| Pierandrea | De Iaco |
| Luis | De jesus |
| Milton | de Jesus |
| José | de Jesús Cárdenas Barón |
| José | De Jesus Casco Samudio |
| Gregorio | de Jesus Labrador Hernandez |
| M | De Kock |
| Marcel | de Kock |
| Michèle | de Kok |
| S | De la Cruz Ahufinger |
| MF I | De La Cruz Monroy |
| Paloma | de la Dehesa Cueto-Felgueroso |
| Angela | de la Hoz |
| A | De la Hoz Rodriguez |
| Anabel | De la Llave Serralvo |
| R | De la Oliva |
| Jairo | De la Peña |
| MA | De la Rosa Abaroa |
| M | De la Rosa-Estadella |
| Marta | de la Rosa-Estadella |
| Javier | de la Torre |
| Olga | De la Varga-Martínez |
| FB | de Lacy |
| Francis | De Leon |
| Murilo | de Lima Brazan |
| Marcello | De Luca |
| Francesca | De Lucia |
| Nicolò | de Manzini |
| A | De Manzoni Garberini |
| Andrea | de Manzoni Garberini |
| J | De Marchi |
| Paolo | De Martini |
| Jorge | De Medeiros |
| Matheus | de Melo Lôbo |
| Federico | De Michele |
| MD C | De Miguel-Ardevines |
| P | De Nardi |
| Paola | De Nardi |
| C | De Nunzio |
| Cosimo | De Nunzio |
| AL | De Oliveira Lopez |
| Alba | de Pablo García-Cuenca |
| Alessandra | De Palma |
| GD | De Palma |
| Jojiemar | De Pano |
| Gilda | De Paola |
| M | De Pastena |
| C | De Ponthaud |
| H | De Praetere |
| Marco | De Prizio |
| PR | De Reuver |
| Giacomo | De Riu |
| S | De Robles |
| Katherine | de Rome |
| Raffaele | De Rosa |
| Silvia | De Santi |
| Chathuranka | De Silva |
| Kanishka | De silva |
| R | De Silva |
| T | De Silva |
| B | De Simone |
| Belinda | De Simone |
| V | De Simone |
| Veronica | De Simone |
| G | De Smul |
| Anabela | de Sousa Salgueiro Oliveira |
| A | De Souza |
| G | De Toma |
| Giorgio | De Toma |
| S | De Vergie |
| Stéphane | de Vergie |
| R | De Vincenti |
| Rosita | De Vincenti |
| A | De Virgilio |
| Armando | De Virgilio |
| Carla | De Vita |
| Jean-Paul PM | De Vries |
| JP P M | De Vries |
| TS | de Vries Reilingh |
| Stefan | De Wachter |
| Marco | De Zuanni |
| Christopher | Deacon |
| Laura | Deacon |
| B | Dean |
| Benjamin | Dean |
| H | Dean |
| Mainak | Deb |
| G | Debele |
| Ashe | DeBiasio |
| Samuel | Debrah |
| Laila | Debri |
| C | Decker |
| Cassie | Decker |
| Georges | Decker |
| B | Decruze |
| Kristof | Dede |
| Georgia | Dedemadi |
| Florence | Dedey |
| Natalija | Dediulia |
| Haya | Deeb |
| S | Defaee |
| C | Defert |
| Coralie | Defert |
| D | Degarege |
| Eyueal | Degefa |
| Hailegebriel | Degefu |
| S | Degener |
| Stephan | Degener |
| I | Deglurkar |
| Yoshihiko | Deguchi |
| A | Dehal |
| D | Dehart |
| Dustin | Dehart |
| Enrica | Deiana |
| Krisztian | Deierl |
| A | Deirino |
| D | Deisher |
| DO | Dejana |
| T | Dejanovic |
| Tatjana | Dejanovic |
| Inga | Dekeryte |
| NA M | Dekker |
| Nicole | Dekker |
| Mathilde | Del |
| Celeste | Del Basso |
| María | Del Campo Lavilla |
| Marco | Del Chiaro |
| Massimo | Del Gaudio |
| R | Del Giudice |
| Roberto | Del Giudice |
| Maria | del Mar Martí-Ejarque |
| Maria | del Mar Soriano |
| Maria | Del Pilar Concejo Cutoli |
| Arazzelly | Del Pilar Paucar Urbina |
| Eneko | Del Pozo Andres |
| Paolo | Del Rio |
| MD | del Toro Lopez |
| T | Del Toro Simoni |
| Czarlo | Dela Victoria |
| Christopher | Delaney |
| KS | Delank |
| S | Delazar |
| A | Delegido García |
| Ana | Delegido García |
| Daniel | Delfau Lafuente |
| Roque | Delfino Licona-Meníndez |
| E | Delgado Blanco |
| Elena | Delgado Blanco |
| L | Delgado Búrdalo |
| J | Delgado Fernandez |
| Juan | Delgado Fernandez |
| Malik | Delhen |
| Samir | Delibegovic |
| D | Deligiannidis |
| C | Delimpalta |
| Demre | Delipinar |
| Olga | Delisau-Puig |
| A | Dell |
| Angela | Dell |
| JS | Della Fontana |
| AN | Della Gatta |
| Carlo | Della Rocca |
| F | DellAglio |
| D | Dellaportas |
| Dionysios | Dellaportas |
| I | DellAtti |
| J | Dellonder Frigolé |
| Cristina | DellOro |
| Mathilde | Delorme |
| Marion | Delpont |
| Paolo | Delrio |
| M | Delsoz |
| D | Deluca kobelanski |
| Suzanne | Demers |
| Z | Demetrashvili |
| Zaza | Demetrashvili |
| Andreas | Demetriades |
| Andreas K | Demetriades |
| C | Demetriou |
| Charis | Demetriou |
| Jevgenijs | Demicevs |
| H | Demir |
| Hakan | Demir |
| İ | Demir |
| Anil | Demi̇r |
| Bengi | Demirayak |
| Gokhan | Demirayak |
| Sibel | Demirel |
| Serdar | Demirgan |
| R | Demirhan |
| Recep | Demirhan |
| F | Demirkiran |
| Fuat | Demirkıran |
| S | Demirli Atici |
| Semra | Demirli Atıcı |
| JA | Demma |
| Daniel | Dempsey |
| Khaled | Demyati |
| S | Demyttenaere |
| FC | den Boer |
| Frank | den Boer |
| Vincenzo | Denaro |
| Christine | Denet |
| Stuart | Denham |
| Franck | Denimal |
| W | Denis |
| Waast | Denis |
| A | Denning |
| M | Denning |
| B | Dennis |
| Grace | Dennis |
| Jonathan | Dennis |
| Barry | Dent |
| H | Dent |
| Rahul | Deo Sharma |
| N | Depalma |
| Norma | Depalma |
| M | Deplano |
| Yadani | Deressa |
| Tilahun | Deresse |
| Habtamu | Derilo |
| HT | Derilo |
| Aleksandr | Derinov |
| Tyche | Derksen |
| Aleksandar | Đermanović |
| Cedric | Dery |
| Malcolm | Dery |
| A | Desai |
| Anant | Desai |
| C | Desai |
| Nimai | Desai |
| NR | Desai |
| Dawit | Desalegn |
| M | Desalegn |
| Christine | Desbiens |
| S | Desbruslais |
| L | Desender |
| Amit | Deshmukh |
| Anuja | Deshmukh |
| Ajinkya | Deshpande |
| Aniket | Deshpande |
| Anvay | Deshpande |
| Charulata | Deshpande |
| Mandar | Deshpande |
| S | Deshpande |
| M | Desio |
| Matteo | Desio |
| Eirini | Deskou |
| Ashwin | Desoouza |
| J | Desroches |
| Francesco | Dessole |
| S | Dessole |
| Salvatore | Dessole |
| S | Dester |
| Salvatora | Dettori |
| AC | Deus |
| Preetham | Dev |
| Eliya | Devan |
| Sreekar | Devarakonda |
| B | Devauchelle |
| Bernard | Devauchelle |
| V | Devezas |
| Vítor | Devezas |
| Grigol | Devidze |
| Michael | Dewan |
| Nikhil | Dewan |
| Tanushree | Dewan |
| V | Dewan |
| Varun | Dewan |
| Hubert | Dewanon |
| Jayan | Dewantha Jayasinghe |
| Ffion | Dewi |
| Madlen | Dewi |
| Swarnendu | Dey |
| Jamie | Deyell |
| Daniel | Deziel |
| K | Dhaduk |
| Ahmed | Dhaif |
| Fatema | Dhaif |
| Bharat | Dhanani |
| D | Dhanani |
| A | Dhannoon |
| Amenah | Dhannoon |
| DS | Dhar |
| Sanjay | Dhar |
| Tapasya | Dhar |
| Satish | Dharap |
| Ashni | Dharia |
| Dharminder | Dhillon |
| Govind | Dhillon |
| Haradeen | Dhillon |
| Mohit | Dhingra |
| B | Dhinsa |
| Rishi | Dhir |
| Harshadkumar | Dhirajlal Rajgor |
| B | Dhondt |
| Bert | Dhondt |
| Slsabela | Dhoon |
| Zainab | Dhorat |
| Hailu | Dhufera |
| Parag | Dhumane |
| Ritika | Dhurwe |
| Matteo | Di Bari |
| Mattia | Di Bartolomeo |
| A | Di Bella |
| Annamaria | Di Bella |
| F | Di Candido |
| F | Di Chiara |
| Nadine | Di Donato |
| FM | Di Flamminio |
| G | Di Franco |
| Gregorio | Di Franco |
| D | Di Giorgio |
| Lorena | Di Girolami |
| M | Di Giuseppe |
| Marta | Di Grezia |
| P | Di Lascio |
| F | Di Lella |
| Sofia | Di Lorenzo |
| Pasquale | Di Maio |
| S | Di Maria Grimaldi |
| C | Di Martino |
| M | Di Martino |
| Marcello | Di Martino |
| Francesco | Di Marzo |
| D | Di Miceli |
| Dario | Di Miceli |
| M | Di Muro |
| S | Di Saverio |
| Salomone | Di Saverio |
| Alberto | Di Somma |
| G | Di Taranto |
| Nora | Di Tomasso |
| Ettorino | Di Tommaso |
| Marcela | Di Vincenzo |
| M | Diab |
| Yasser | Diab |
| B | Diaconescu |
| Bogdan | Diaconescu |
| A | Diamantis |
| Alexandros | Diamantis |
| A | Dias |
| Andre | Dias |
| André | Dias |
| Beatriz | Dias |
| David | Dias |
| Nuno | Dias |
| Richard | Dias |
| Vanessa | Dias |
| A | Dias Samarawickrama Yapa |
| Consuelo | Diaz |
| J | Diaz |
| Jose | Diaz |
| K | Diaz |
| Raquel | Diaz |
| S | Diaz |
| Sandra | Diaz |
| C | Díaz |
| Gabriela | Díaz |
| Pedro | Díaz |
| R | Diaz Del Gobbo |
| G | Diaz Duarte |
| A | Díaz García |
| Alberto | Díaz García |
| Eneida | Diaz Martinez |
| Alba | Diaz Padillo |
| P | Díaz Peña |
| Patricia | Díaz Peña |
| D | Díaz Pérez |
| David | Díaz Pérez |
| R | Diaz Serrano |
| Gonzalo | Díaz Tapia |
| Tamara | Díaz Vico |
| Fernando | Diaz-Couselo |
| PJ | Diaz-Delgado |
| Berta | Díaz-Feijoo |
| R | Diaz-Ruiz |
| Aubrey | Dickason |
| Edward | Dickson |
| K | Dickson |
| Kathryn | Dickson |
| M | Diczbalis |
| Marcel | Didier Ndayishyigikiye |
| H | Didriksson |
| Helen | Didriksson |
| Victor | Diego Caballero Sarabia |
| Lucia | Diego García |
| B | Diéguez |
| Beatriz | Diéguez |
| Maria | Dieguez López |
| T | Diehl |
| Thomas | Diehl |
| Joachim | Diessner |
| MD | Diestro |
| E | Díez |
| M | Diez Alonso |
| Manuel | Diez Alonso |
| Fernando | Diez Burón |
| A | Diez-Fraile |
| ES | Dif |
| Holly | Digne-Malcolm |
| Emilio | Dijan |
| E | Dikicier |
| Enis | Dikicier |
| Merve | dilara Öney |
| Seda | Dilek Yetut |
| Tina | Dilevska |
| Michael | DiMaio |
| A | Dimas |
| DM | Dimatatac |
| Julian | Dimech |
| Papalouka | Dimitra |
| James | Dimitri Kane |
| D | Dimitrijevic |
| Ivan | Dimitrijevic |
| Christos | Dimitrios Terzoudis |
| Nikolaos | Dimitrokallis |
| Dimitrios | Dimitroulis |
| D | Dimitrov |
| Dobromir | Dimitrov |
| M | Dimofte |
| Mihail-Gabriel | Dimofte |
| Danielle | Dimsoy |
| HA | Dincer |
| D | Ding |
| W | Ding |
| Maggie | DiNome |
| Joseph | DiNorcia |
| Ettore | Dinoto |
| Marie | Dione Parreno-Sacdalan |
| Marie | Dione Sacdalan |
| Sandra | Dios-Barbeito |
| Matthew | Dipper |
| Tesfaye | Diress |
| R | Dirks |
| Tariq | Diryaq |
| S | Discepola |
| Karla | Disla |
| C | Distefano |
| M | Distler |
| Marius | Distler |
| Antonino | Ditto |
| Emre | Divarci |
| H | Divecha |
| Pawan | Dixit |
| J | Dixon |
| Lauren | Dixon |
| Toby | Dixson |
| Bahraoui | Djahida |
| Z | Djama |
| Kaveh | Djamali |
| V | Djan |
| Vladimir | Djan |
| K | Djebabria |
| G | Djedovic |
| Gabriel | Djedovic |
| A | Djelloul |
| A | Djouani |
| V | Djukic |
| Vladimir | Djukic |
| M | Djuric |
| Igor | Djurisic |
| Christopher | Dobbins |
| A | Dobrescu |
| S | Dodd |
| Sophie | Dodd |
| M | Doe |
| J | Doerner |
| Johannes | Doerner |
| NU | Dogan |
| Bayram | Doğan |
| Keziban | Doğan |
| L | Doğan |
| Lütfi | Doğan |
| S | Doğan |
| Selen | Doğan |
| Selim | Doğan |
| M | Doğangün |
| Francesco | Doglietto |
| Anthony | Dohan |
| Sasho | Dohcev |
| Ciaran | Doherty |
| Daniel | Doherty |
| Laura | Doherty |
| Charalampos | Doitsidis |
| K | Doklestic |
| Kingsley | Doku |
| Rachael | Dolan |
| Giampiero | Dolci |
| Brett | Doleman |
| María | Dolores Arribas Del Amo |
| Maria | Dolores Burgueño |
| Maria | Dolores Mateo Arzo |
| R | Domagalski |
| Maurizio | Domanin |
| J | Domenech |
| Julio | Domenech |
| J | Domenech Fernández |
| M | Domenichini |
| Marco | Domenichini |
| L | Domenici |
| Giovanni | Domenico De Palma |
| Efren | Domingo |
| EJ | Domingo |
| María | Domingo |
| Carlos | Domingo Del Pozo |
| JC | Domingues |
| Marisa | Domingues Santos |
| CM | Dominguez |
| V | Domínguez-Prieto |
| Ismael | Dominguez-Rosado |
| C | Domröse |
| Christian | Domröse |
| Jimena | Dona |
| Hans | Donald de Boer |
| Danilo | Donati |
| G | Dondi |
| Giulia | Dondi |
| Niren | Dongre |
| Urszula | Donigiewicz |
| MA | Doniquian |
| Marcelo | Doniquian |
| Francisco | Donis |
| Henrique | Donizetti Bianchi Florindo |
| N | Donlon |
| Noel | Donlon |
| Lauren | Donnelly |
| GF | DOnofrio |
| Christopher | Donoghue |
| Emma | Donohoe |
| C | Donohue |
| C | Donoudis |
| Richard | Donovan |
| Maebh | Doohan |
| A | Doorgakant |
| A | Dorafshar |
| Amir | Dorafshar |
| Amy | Doran |
| C | Doria |
| Carlo | Doria |
| Ander | Dorken Gallastegi |
| A | Dorken-Gallastegi |
| Isabella | Dornauer |
| Vitaly | Dorofeev |
| Panagiotis | Dorovinis |
| Emmet | Dorrian |
| Isabela | dos Anjos |
| L | Dos Santos Carregal |
| Jorge | dos Santos Silva |
| Anirudha | Doshi |
| Neel | Doshi |
| Alexios | Dosis |
| Michal | Dosoudil |
| Markus | Doss |
| Francis | Dossou |
| A | Dostbil |
| Ivan | Dot Pascuet |
| C | Dott |
| Cameron | Dott |
| Sarah K | Dotters-Katz |
| Zain | Douba |
| C | Doudakmanis |
| B | Doughty |
| T | Doulias |
| A | Doussot |
| Alexandre | Doussot |
| G | Dovell |
| Andraz | Dovnik |
| Joseph | Dowdall |
| Elizabeth | Doxford-Hook |
| Alex | Doyle |
| Joseph | Doyle |
| V | Dragisic |
| Vedran | Dragisic |
| Antonella | Dragotto |
| A | Drahman |
| P | Drakakis |
| Anna | Drake |
| Frederick | Drake |
| FT | Drake |
| T | Drake |
| Thomas | Drake |
| Thomas D | Drake |
| A | Drane |
| Andrew | Drane |
| R | Drasovean |
| Radu | Drasovean |
| Madhulika | Dravid |
| Walter | Dreak Erabu |
| F | Dreier |
| William | Drew |
| Davide | Drigo |
| S | Driouich |
| L | Driul |
| Matteo | Droghetti |
| E | Drozdov |
| Evgeniy | Drozdov |
| R | Dru |
| Vincent | Drubay |
| Isabella | Drummond |
| Katharine | Drummond |
| JP | Druta |
| Joe | Drybrough |
| Carl | DSouza |
| D | DSouza |
| J | DSouza |
| H | Du Preez |
| Therese | Du Preez |
| Mohammed | Duah Issahalq |
| Mireia | Duart |
| Dayhana | Duarte |
| Geraldo | Duarte |
| Handsome | Dube |
| M | Dube |
| Manas | Dube |
| Ngqabutho | Dube |
| SK | Dube |
| Vivek | Dubey |
| DD | Dubinski |
| E | Duchalais |
| Emilie | Duchalais |
| N | Duchateau |
| S | Ducic |
| Sinisa | Ducic |
| Stefan | Ducic |
| E | Duck |
| Abigail | Duckett |
| Thomas | Dudding |
| J | Dudek |
| Nagendra | Dudi-Venkata |
| NN | Dudi-Venkata |
| S | Duff |
| Caoimhe | Duffy |
| Ana | Dugandžić Šimić |
| Anchal | Duggal |
| L | Duggleby |
| Domenico | DUgo |
| Massimo | Dugo |
| S | DUgo |
| Stefano | DUgo |
| Marc | Duinslaeger |
| Paula | Dujovne Lindenbaum |
| Prabuth | dulanjan Weeraddana |
| Onur | Dülgeroğlu |
| Sébastien | Dulisse |
| David | Duller |
| A | Dulskas |
| Audrius | Dulskas |
| Mihai | Dumbrava |
| S | Dumitra |
| Cătălin | Dumitraș |
| O | Dumlu |
| Christian W | Dumpies |
| G | Dunbar |
| Mariya | Dunbobbin |
| R | Duncan |
| Sharon | Duniya |
| Cheryl | Dunkerton |
| J | Dunn |
| Julie | Dunn |
| D | Dunne |
| Declan | Dunne |
| Henry | Dunne |
| N | Dunne |
| J | Dunning |
| M | Dunstan |
| Leanne | Dupley |
| A | Dupont |
| JP | Duprat |
| M | Duque |
| V | Duque Mallén |
| Victoria | Duque Mallén |
| V | Duque-Mallen |
| Igor | Duquesne |
| AC | Dural |
| I | Duran |
| Oscar | Durán Anguiano |
| M | Durán Ballesteros |
| I | Duran Sanchez |
| II | Durán Sánchez |
| VM | Durán-Muñoz-Cruzado |
| Francesc | Duran-Valles |
| Giulia | Duranti |
| Norah | Durayb |
| Andrew | Durden |
| Natalie | Duric |
| M | Đurić |
| Katarina | Duric Vukovic |
| H | Durio Yates |
| Vladimir | Durleshter |
| E | Durmuş |
| A | Duro |
| Agustin | Duro |
| A | Durrani |
| Amer | Durrani |
| Antonio | Durso |
| AZED | Durst |
| Ayberk | Dursun |
| I | Dushin |
| Vincent | Dusingizimana |
| Lambert | Dusingizimana Rutayisire |
| K | Dusu |
| Debnarayan | Dutta |
| Rohini | Dutta |
| B | Dvoranova |
| LS | Dvorkin |
| Y | Dwa |
| Yam | Dwa |
| SE | Dwaga |
| Mays | Dweik |
| Simon | Dwerryhouse |
| O | Dwidar |
| Oliver | Dwidar |
| Adam | Dyas |
| Claudia | Dyball |
| M | Dyer |
| R | Dyke |
| Daniel | Dykman |
| Z | Dzamic |
| Zoran | Dzamic |
| A | Dzhanaeva |
| Khasan | Dzhumabaev |
| Berik | Dzhumabekov |
| Timur | Dzhumatov |
| J | Dziakova |
| Jana | Dziakova |
| Daniel | Dzinotyiwei |
| Mawutor | Dzogbefia |
| Andee | Dzulkarnaen Zakaria |
| Gul | e Raana Raana |
| Yegeremu | Eado |
| N | Eardley |
| Nicola | Eardley |
| Sophie | Earl |
| H | Earley |
| Helen | Earley |
| Hannah | Earnshaw |
| Asha | Eastmond |
| Samuel | Ebbs |
| John | Ebenezer |
| H | Eberbach |
| Helge | Eberbach |
| Georgina | Eberle |
| Mohamed S | Ebiad |
| Fabrice | Eboma |
| A | Ebrahim |
| Abdulla | Ebrahim |
| S | Ebrahim |
| Keramatollah | Ebrahimi |
| JA | Echavarría Uceta |
| Alejandra | Echeverri Moreno |
| G | Echeverría-Dávila |
| T | Echim |
| Timea | Echim |
| S | Eckhouse |
| Panagiota | Economopoulou |
| Salah | Eddine Oussama Kacimi |
| B | Eddy |
| Ekaniyere | Edetanlen |
| Fabian | Edinger |
| Stephen | Edino |
| Ulbar | Edinson |
| Dileepa | Ediriweera |
| Hans | Edison Yap |
| Florian | Edlinger |
| Jennifer | Edmondson |
| R | Edmondson |
| Edwaldo | Edner Joviliano |
| Kemebradikumo | Edonkumoh |
| Maria | Eduarda Bellotti Leão |
| Byron | Eduardo Lopez De Mesa Lopez |
| Ruben | Eduardo Morán Galaviz |
| Carlos | Eduardo Otiniano Alvarado |
| Luis | Eduardo Pérez-Sánchez |
| Raúl | Eduardo Pinilla Morales |
| Blas | Eduardo Quintero Sada |
| Carlos | Eduardo Rey Chaves |
| Alvaro | Eduardo Sánchez Hernández |
| Jorge | Eduardo Sisa Acosta |
| J | Edwards |
| John | Edwards |
| John G | Edwards |
| Tomos | Edwards |
| Amy | Edwards Murphy |
| Andrew | Edwards-Bailey |
| Isaac | Edyedu |
| Arthur | Ee |
| Cho | Ee Ng |
| S | Efetov |
| Sergey | Efetov |
| SK | Efetov |
| Chizoba | Efobi |
| Erik | Efrain Sosa Duran |
| S | Efremov |
| Edgard | Efren Lozada Hernandez |
| Evangelos | Efthimiou |
| Matheos | Efthimiou |
| N | Efthymiou |
| B | Egan |
| Richard | Egan |
| RJ | Egan |
| E | Egbor |
| Esezobor | Egbor |
| IK | Egbuchulem |
| John | Egbuji |
| A | Egdeer |
| Eva | Egger |
| A | Egglestone |
| Peter | Egharevba |
| N | Egoroff |
| Natasha | Egoroff |
| Tamás | Egyed |
| N | Eibinger |
| Klaus | Eichhorn |
| A | Eid |
| Abdulrahman | Eid |
| Ahmad | Eid |
| J | Eid |
| Raja | Eid |
| Taher | Eid |
| C | Eiriz Fernandez |
| Gustavo | Eisenberg |
| Vitalijus | Eismontas |
| Abdullah | Eissa |
| Eiji | Eiwa |
| T | Ejajo |
| Ikechukwu | Ejiofor |
| Aruna | Ekanayaka |
| NG | Eke |
| Onyeanunam | Ekeke |
| Sebastian | Ekenze |
| Murat | Ekin |
| Perihan | Ekmekçi |
| S | Ekpemo |
| Ekemini | Ekpo |
| E | Ekrami |
| Elyad | Ekrami |
| Bosom | Ekwere |
| H | Ekwunife |
| B | El Ahmadi |
| Brahim | El Ahmadi |
| Zein | el Amir |
| M | El Amrani |
| Mehdi | El amrani |
| N | El Arbi |
| Hadi | El Assaad |
| A | El Azhari |
| Abdessamad | El Azhari |
| Hind | El Azzazi |
| Yassine | El Bouazizi |
| S | El Drubi Vega |
| Sara | El Falaha |
| L | El Fiky |
| Lobna | El Fiky |
| Khaled | El Gazzar |
| MA | El Ghali |
| A | El Ghoneimi |
| Nancy H | El Goweini |
| M | El Hadi |
| Mohamed | EL Hag |
| Joe | El Hage |
| M | El Hechi |
| Amin | El Helw |
| Abd | El Jawad Al Gasi |
| AES | El kady |
| J | El Kafsi |
| Jihène | El Kafsi |
| M | El Kassas |
| Mohamed | El Kassas |
| Ahmed | El Kelany |
| Rasha | El kharashy |
| Saja | El Masaoudi |
| M | El Moheb |
| Mohamad | El Moheb |
| Sara | El Mustapha |
| Badih | El Nakadi |
| A | El Ouahabi |
| Abdessamad | El Ouahabi |
| Mehdi | El Ouazzani |
| Mustafa | El Sheikh |
| Esraa | El Shemy |
| Mohammed | El Sherpiny |
| Amr | El Yamany |
| H | El Youzouri |
| Mohamed G | El-adawy |
| Magdy S | El-Bahnasawy |
| K | El-Boghdadly |
| Kariem | El-Boghdadly |
| Marwa | El-Deeb |
| Abd | El-Fattah Mouhandes |
| Mohammed | El-Hag-Aly |
| S | El-Hasani |
| A | El-Hussuna |
| Alaa | El-Hussuna |
| M | El-Kassas |
| Hanna | El-Khoury |
| O | El-Koubani |
| Mustafa | El-lami |
| Y | El-Masry |
| Khaled | El-Qawaqzeh |
| Jazal | El-Qudah |
| Abd | El-Rahman Hamed |
| M | El-Rashid |
| Mohammad | El-Sharkawi |
| Ahmed M | El-Sharkawy |
| Yasin | El-Wajeh |
| Ahmed | Elaffandi |
| Faisal | Elagili |
| Minahil | Elahi |
| Z | Elahi |
| Zain | Elahi |
| Mohamed | Elakkad |
| Faisl | Elamin |
| Mutaz | Elamin |
| H | Elamin Ahmed |
| Ziad | Elassar |
| A | Elawad |
| Amar | Elawad |
| M | Elayyan |
| Abd | Elazeem |
| MA | Elbadawy |
| Seifeldin | Elbadawy |
| M | Elbahnasawy |
| Mohamed | Elbahnasawy |
| W | Elbakbak |
| Osama | Elbargathe |
| Sofian | Elbarouni |
| Ibrahim | Elbashir |
| P | Elbe |
| Peter | Elbe |
| Gehad | Elbehairy |
| A | Eldaly |
| Abdullah | Eldaly |
| Tarik | Eldarat |
| M | ElDeeb |
| Hossam | Eldeen Soliman |
| D | Elder |
| Omnia | Eldesouky |
| Nour | Eldin Abosamak |
| Nagm | Eldin Abu Elnga Ahmed |
| Moaiad | Eldin Ahmed |
| Nour | Eldin Nader |
| Alaa | Eldine Elmaghraby |
| Sami | Eldirdiri |
| Samwal | Eldirdiri |
| OA | Elebute |
| Olumide | Elebute |
| Mahmoud | Eleisawy |
| Peter | Elemile |
| Carmen | Elena Badillo Bercebal |
| Felicia | Elena Buruiana |
| Laura | Elena Fernandez Rios |
| María | Elena Muñoz Fernández |
| Anna | Eleonora Gut |
| Mustafa | Elfadli |
| A | Elfallal |
| Ahmed | Elfallal |
| H | Elfeki |
| Hossam | Elfeki |
| H | Elfeky |
| M | ElFiky |
| Mahmoud | ElFiky |
| Jamael | Elfitori |
| Jane | Elford |
| Alaa | Elgaili |
| Omar | ELgamal |
| F | Elgammal |
| Sarah | Elgarf |
| Ibrahim | ElGarhy |
| A | Elgazar |
| Abdelrahman | Elgendy |
| A | Elgenidy |
| H | Elghadban |
| Omar | Elghany |
| M | Elghazal |
| Shrouk M | elghazaly |
| SM | Elghazaly |
| S | Elghiati |
| Mahmoud | Elghoury |
| A | Elhadi |
| Ahmed | Elhadi |
| M | Elhadi |
| F | Elhajdawe |
| FAD | Elhajdawe |
| Fras | Elhajdawe |
| I | Elhalaby |
| Ismael | Elhalaby |
| AS | Elhalawany |
| A | Elhamshary |
| Rewan | Elhawary |
| Abhay | Elhence |
| Yassein | Elhussein |
| M | Elhusseini |
| Ahmed | Elhussiny Salah Mahmoud |
| Liolis | Elias |
| Bottazzoli | Elisa |
| Maria | Elisa Lozano Miralles |
| Irmgard | Elisabeth Kronberger |
| Sara | Elisabetta Dester |
| Carlo | Elises |
| Nebiyu | Eliyas |
| Sandra | Elizabeth Centurion Rolon |
| Joby | Elizabeth Ninan |
| Hisham | Eljack |
| Mohamed | Eljack |
| H | Elkadi |
| R | Elkady |
| D | Elkebir |
| C | Elkettani |
| Abdulmohimen A | Elkhadar |
| Fatimah | Elkhafeefi |
| H | Elkhayat |
| Hussein | Elkhayat |
| Dina | Elkhity |
| Esraa | Elkouba |
| A | Elkoundi |
| Abdelghafour | Elkoundi |
| Hamed | Ellakwa |
| H | Ellauzi |
| Yosef | Ellenbogen |
| Tressa | Ellett |
| Brodie | Elliott |
| JA | Elliott |
| L | Elliott |
| Michael | Elliott |
| Clayton | Ellis |
| S | Ellis |
| Susan | Ellis |
| Ibrahim | Ellojli |
| Amna | Elmabrouk |
| Khaled | Elmaghraby |
| Walid | Elmahdy |
| Asma | Elmahgoub |
| N | Elmaleh |
| Nabil | Elmaleh |
| Omar | Elmandouh |
| S | Elmarimi |
| Amr | Elmeanawy |
| Ahmed O | Elmehrath |
| Walid | Elmoghazy |
| Randa | Elmokhtar |
| U | Elmore |
| Rami | Elmorsi |
| T | Elmoslemany |
| Tarek | Elmoslemany |
| Nabiha | Elmsherghi |
| Mohammed | Elmujtaba |
| Mohammed | Elmujtba Adam Essa Adam |
| R | Elmusa |
| Reem | Elmusa |
| Hassan | Elmusharaf |
| AlaELDEIN | Elnaema |
| Hatem | Elnageh |
| Ahmad | Elnassasra |
| Sarah | Elnems |
| M | Elniel |
| Mohammed | Elniel |
| S | Elnikety |
| Salma | Elnoamany |
| Mohamed | Elobaid |
| Enilde | Eloena Guerra |
| AM | Elosta |
| Abdulrahman | Elrahmany |
| Mohamedyasin | Elrashid |
| Nasre | Elrefai |
| Alaa | Elsabagh |
| Seif | ElSaban |
| M | Elsabbagh |
| Menan | Elsadek |
| Marwa | Elsadig |
| Mohamed | Elsaeed |
| Said | Elsagheer |
| Ahmed | ElSaghir |
| Reem | Elsahti |
| Mahmoud | Elsaid |
| Alromisaa | Elsaka |
| Mohamed | Elsalhy |
| K | ElSanhoury |
| Kareem | ElSanhoury |
| Abdelrahman | Elsawey |
| Ahmed | Elsayed |
| Hend | Elsayed |
| Hisham M | Elsayed |
| Manal | Elsayed |
| Mostafa | Elsayed Elsayed Hewalla |
| ME | Elsayed Hewalla |
| Shady | Elsdfy |
| Ahmed | Elshabrawy |
| Enas | Elshabrawy |
| Mohamed | Elshafey |
| Ghazi | Elshafie |
| H | Elshafie |
| Mohamed | Elshaibi |
| Seliman | ELShakhs |
| Mohamed | Elsharkawy |
| Ahmed | Elshawadfy Sherif |
| H | Elsheikh |
| Hizabr | Elsheikh |
| Randa | Elsheikh |
| Motaz | Elsherbeeny |
| Ahmed | Elsherbini |
| Hashim | Elshibly |
| Khaled | ElSisy |
| Natasha | Elson |
| Naira | Elsoudy |
| Chase | Elswick |
| Mohammed | Eltahier Abdalla Omer |
| Aymad | Eltawab |
| Almoutaz | Eltayeb |
| M | Eltayeb |
| Momin | Eltayeb |
| Sherif | Eltregy |
| Alejandro | Elúa |
| Sherif | Elwatidy |
| B | Elyafawi |
| Bilal | Elyafawi |
| Mohammed | Elzain |
| M | Elzoghby |
| AE | Elzoubi |
| A | Emad Mashhour |
| Ahmed | Emad Sayed Hassan |
| Eurico | Emanuel do Vale Gonçalves de Castro Alves |
| Carmen | Emanuela Scandura |
| MM | Emara |
| Sherif | Emara |
| H | Embarek |
| Ysabelle | Embury-Young |
| Stanley | Emeka Nwabuoku |
| H | Emerson |
| Hannah | Emerson |
| S | Emile |
| Sameh | Emile |
| Sameh H | Emile |
| Tano | Emile |
| Herica | Emilia Félix de Carvalho |
| María | Emilia Muriel |
| Mahmut | emin Çiçek |
| C | Emir Alavi |
| Cem | Emir Guldogan |
| Luis | Emiro Vanegas |
| M | Emiroglu |
| Mustafa | Emiroglu |
| K | Emmanuel |
| Klaus | Emmanuel |
| Muhawenimana | Emmanuel |
| Oscar | Emmanuel Posadas-Trujillo |
| Benjamin | Emmerson |
| O | Emmerson |
| Basonga | Emmy |
| M | Emous |
| Ayyah | Emran |
| Yunus | Emre Aktimur |
| Murat | Emre Reis |
| K | Emslie |
| Nosakhare | Enaruna |
| Alejandro | Encinas Bascones |
| Alberto | Encinas Vicente |
| O | Enciu |
| Octavian | Enciu |
| C | Endara |
| F | Endara |
| Jana | Enderes |
| Shunji | Endo |
| F | Endorf |
| Frederick | Endorf |
| FW | Endorf |
| Cristian | Ene Roata |
| I | Enemosah |
| Akif | Enes Arikan |
| J | Engel |
| C | English |
| Sefiu | Eniola |
| DT | Eniu |
| D | Enjuto |
| Diego | Enjuto |
| R | Ennab |
| Raed | Ennab |
| Brendan | Ennis |
| Loreno E | Enny |
| E | Enoch |
| Elizabeth | Enoch |
| Frank | Enoch Gyamfi |
| Yutaka | Enomoto |
| Laura | Enrica Benedetti |
| Adolfo | Enrique Gómez Ortiz |
| Carlos | Enrique Melo Moreno |
| Guillermo | Enrique Reyes Gamonal |
| Christian | Enrique Soulé Martínez |
| Joaquim | Enseñat Nora |
| David | Ensor |
| Annika | Enste |
| Enti | Enti |
| Kenneth | Enwerem |
| Sarah | Epton |
| Aritz | Equisoain Azcona |
| Huseyin | Eraslan |
| Aydın | Eray Tufan |
| G | Ercan |
| Gulcin | Ercan |
| C | Ercetin |
| Candas | Ercetin |
| G | Ercolani |
| Giorgio | Ercolani |
| S | Erdene |
| Sarnai | Erdene |
| T | Erdil |
| Hüseyin | Erdi̇nç |
| B | Erdle |
| Emre | Erdoğan |
| S | Erel |
| Reza | Erfanian |
| M | Ergenç |
| E | Erginöz |
| Ergin | Erginöz |
| S | Ergun |
| Sefa | Ergün |
| F | Eriberto |
| Jan | Erik Detran |
| Thomas | Erik Wurmb |
| E | Eriksson |
| Clarissa | Ern Hui Fang |
| A | Eroglu |
| T | Erol |
| S | Erridge |
| O | Ersen |
| Cevper | Ersoz |
| Ş | Ersöz |
| Mar | Escales |
| J | Escartin |
| Jorge | Escartin |
| Alejandro | Escobar |
| D | Escobar |
| Daniel | Escobar |
| Pedro | Escobar |
| Elkin | Escorcia |
| Remberto | Escoto |
| B | Escudero |
| Berta | Escudero |
| Mario I | Escudero |
| MI | Escudero |
| L | Escudero-Roque |
| Anita | Eseenam Agbeko |
| Akaninyene | Eseme Ubom |
| Marlow | Esguerra |
| Fares | Eshac |
| Youssof | Eshac |
| Mahder | Eshete |
| Mabroka | Eshnaf |
| Mohsen | Eshraghi |
| F | Eskandari |
| Ammar | Eskander |
| Antoine | Eskander |
| P | Eskander |
| S | Esmaeili Fathabadi |
| F | Esmaeili Tarki |
| Muhib | Esmael |
| E | Esmail |
| Mohamed | Esmat Mohamed |
| Nereida | Esparza Arias |
| Isaac | Esparza Estrada |
| E | Espin-Basany |
| Eloy | Espin-Basany |
| M | Espino Segura-Illa |
| Carla | Espinola |
| CA | Espinosa |
| J | Espinosa |
| Alfonso | Espinosa Ruiz |
| M | Espinosa-Bravo |
| Martin | Espinosa-Bravo |
| LP | Espinoza Padrón |
| R | Espinoza-Llerena |
| F | Espitalier |
| Hernando | Espitia |
| A | Esposito |
| G | Esposito |
| Giuseppe | Esposito |
| Carlos | Esquivel |
| Mehmet | Eşref Ulutaş |
| M | Essa |
| Murtaza | Essajee |
| Leila | Essakalli Hossyni |
| ME | Essalhi |
| Esraa | Essam |
| Amr | Essameldin |
| Sumayya | Essayah Dwaga |
| Abdalrahem | Essied Alzubi Alzoubi |
| M | Estaire Gómez |
| Mercedes | Estaire Gómez |
| Mohamad | Estanbouli |
| Agustin | Esteban |
| E | Esteban Agustí |
| Enrique | Esteban Agustí |
| Gustavo | Esteban Lugo Zamudio |
| Ricardo | Esteban Mentz |
| O | Esteban Sinovas |
| Nuria | Estellés Vidagany |
| R | Esteves Pires |
| Robinson | Esteves Pires |
| José | Estevez Tesouro |
| Maria | Esther Ferreira Aguilera |
| Mª | Esther Valsero Herguedas |
| Lujan | Estigarribia |
| EE | Estrada |
| B | Estraviz |
| Begoña | Estraviz |
| E | Estrella |
| Emmanuel | Estrella |
| E | Etchill |
| Eric | Etchill |
| Geley | Ete |
| M | Etezadpour |
| Mohammad | Etezadpour |
| Masatoshi | Eto |
| J | Etra |
| Josune | Etxabe Gurrutxaga |
| M | Eugene |
| Muneza | Eugene |
| Ngoga | Eugene |
| Mª | Eugenia Marín Martínez |
| Fabiola | Eugenia Michel Campos |
| Maria | eugenia Torguet muñoz |
| Felice | Eugenio Agro |
| Corinne | Eulalie Solo |
| Narimantas | Evaldas Samalavicius |
| R | Evangelista Zamora |
| Betsy | Evans |
| Daisy | Evans |
| H | Evans |
| J | Evans |
| Jonathan | Evans |
| Jonathan P | Evans |
| R | Evans |
| Rhodri | Evans |
| V | Evans |
| Victoria | Evans |
| Annija | Evelīna Berga |
| Nissi | Evelyn |
| Nissi | Evelyn R |
| C | Eveno |
| Clarisse | Eveno |
| Anokhin | Evgeny |
| Moataz | Ewedah |
| Christina | Ewington |
| Emma | Ewins |
| R | Exley |
| K | Eyuboglu |
| Kayahan | Eyuboglu |
| AC | Ezanno |
| Anne-Cecile | Ezanno |
| M | Ezeanochie |
| Michael | Ezeanochie |
| Manal | Ezeddin kamel Sheta |
| Sean | Ezekiel Seow |
| Constantine | Ezeme |
| Ekene | Ezenwa |
| Francis | Ezenwankwo |
| Grace | Ezeoke |
| U | Ezomike |
| Uchechukwu | Ezomike |
| Yara | Ezz |
| Esraa | Ezzat |
| Mohammed | Ezzat Mostafa |
| Horacio | F Mayer |
| Erich | Fabbri |
| G | Fabbri |
| N | Fabbri |
| Nicolò | Fabbri |
| Cristoforo | Fabbris |
| Robin | Faber |
| Robert | Fabian |
| Carlos | Fabián Cárdenas Melgarejo |
| German | Fabian Godoy Perez |
| Nestor | Fabian Pedraza Alonso |
| Elio | Fabio Sánchez Cortés |
| OM | Faboya |
| Omolara | Faboya |
| N | Fabregas |
| Neus | Fabregas |
| Berta | Fabregó Capdevila |
| Mariana | Faccini Teixeira |
| Dedy | Fachrian |
| H | Facundo |
| Helena | Facundo |
| Ayrton | Facundo Valdovinos |
| Luciana | Facure |
| T | Fadalla |
| M | Fadavipour |
| MG | Fadel |
| AAM | Fadhel |
| H | Fadhel |
| HA | Fadlalmola |
| Saba | Fadli |
| Begoña | Fadrique |
| Alice | Fae Ferreira |
| J | Fagan |
| Paula | Fagan |
| Mohammed | Fagihi |
| K | Fagnon |
| S | Fahad |
| Shoaib | Fahad Hussain |
| Muhammad | Fahadullah |
| Mahmoud | Fahd |
| BA | Fahey |
| Mir | Fahiem-ul-Hassan |
| Ahmed | Fahim |
| M | Fahim |
| Muhammad | Fahim Ahsan Ahsan |
| T | Fahlbusch |
| Tim | Fahlbusch |
| T | Fahmawee |
| MW | Fahmi |
| Mohd | Fahmi Abd Aziz |
| Mohamed | Fahmy |
| Waleed | Fahmy |
| Aini | Fahriza Ibrahim |
| Ahmed | Faidh Ramzee |
| Ahmad | Faidzal Othman |
| Mehmet | Faik OZcelık |
| Sara | Faily |
| Martina | Faimali |
| K | Fairhurst |
| Mohd | Fairudz Mohd Miswan |
| Abdulrahman | Faisal Al-Garadi |
| Muhammad | faisal Khan |
| Muntasir | Faisel |
| Mohamed | Faizal Bin Sikkandar |
| Fouzia | Faizi |
| Ildar | Fakhradiyev |
| Ildar R | Fakhradiyev |
| Seyed | fakhreddin Hejazi |
| M | Fakhrolmobasheri |
| Mohammed | Fakhrul-Aldeen |
| Ghinwa | Fakih |
| R | Falah |
| Areti | Falara |
| Federica | Falaschi |
| M | Falcioni |
| G | Falco |
| Giuseppe | Falco |
| Mónica | Falcón Coronado |
| GM | Falcon Pacheco |
| BV | Falconí Noriega |
| Fabio | Falconieri |
| Sian | Falder |
| Eva | Falkensammer |
| G | Fallabrino |
| Jean-Michel | Fallah |
| Simon | Fallis |
| Deema | Fallouh |
| M | Fambrini |
| Massimiliano | Fambrini |
| P | Familiari |
| Pietro | Familiari |
| Kathleen FM | Fan |
| Sui | Fan Tang |
| Maheriandrianina | Fanambinana Voahary Rajaonarivony |
| A | Fancellu |
| Alessandro | Fancellu |
| E | Fandridis |
| CEH | Fang |
| F | Fang |
| M | Fanjul |
| Antonios | Fantakis |
| Francesca | Fappiano |
| A | Faqih |
| Frederick | Far |
| Ahmed | Farag |
| Mohamed | Farag |
| Ahmed | Farag ElKased |
| Syeda | Farah Nazir |
| Ahmad | Faraz |
| Hala | Fares |
| Imadeddine | Farfour |
| Anthony | Farfus |
| Abeer | Farhan |
| Siti | Farhan Moh Pauzi |
| Waad | Farhat |
| C S | Faria |
| Giles | Faria |
| Mohammed | Farid |
| Ratna | Farida Soenarto |
| Shehla | Faridoon |
| A | Faried |
| S | Farik |
| Shebani | Farik |
| Sofia | Farina |
| Ignacio | Fariña |
| Anna | Faris |
| Amicur | Farkas |
| Andras | Farkas |
| Tallat | Farkhanda |
| R | Farnan |
| F | Farnesi |
| Francesca | Farnesi |
| Francesco | Farnia |
| M | Farooq |
| MS | Farooq |
| Muhammad | Farooq |
| Umer | Farooq |
| Usman | Farooq |
| Muhammad | Farooq Afzal |
| Kamran | Farooque |
| Bakhtawar | Farooqui |
| AM | Farouk |
| Ayman | Farouk |
| O | Farouk |
| Osama | Farouk |
| Barnaby | Farquharson |
| Alex | Farr |
| Faisal | Farrash |
| R | Farre Font |
| R | Farrell |
| T | Farrell |
| Paul | Farrelly |
| Ramon | Farres Coll |
| Cristina | Farrés Pla |
| Anna | Farrés Rabanal |
| Joseph | Farrimond |
| A | Farro |
| Isabelle | Farrow |
| Melanie | Farrugia |
| A | Farsi |
| Ali | Farsi |
| Deema | Farsi |
| Nada | Farsi |
| S | Farsi |
| Sara | Farsi |
| Omer | Faruk Ozkan |
| Anas | Fatani |
| H | Fatemi manesh |
| Hassan | Fatemi manesh |
| MS A T | Fathelbab |
| M | Fathi Al Gharyani |
| Muad | fathi khalleefah Abu hallalah |
| Muhammed | Fatih Simsekoglu |
| Aleeza | Fatima |
| Mishal | Fatima |
| Zareen | Fatima |
| Sara | Fatima Faqar-Uz-Zaman |
| Martha | Fatima Irene De La Cruz Monroy |
| Dahiana | Fatima Velazquez |
| Belabbes | Fatima zohra |
| F | Fatouh |
| Fathallah | Fatouh |
| Adedeji | Fatuga |
| Alastair | Faulkner |
| G | Faulkner |
| Gemma | Faulkner |
| Jordan | Faulkner |
| Ntirenganya | Faustin |
| K | Favilla |
| H | Fawi |
| Ahmad | Fawzi |
| Ahmed | Fawzy |
| M | Fawzy |
| Mohamed | Fawzy |
| A | Fayad |
| EA | Fayad |
| Elsayed A | Fayad |
| MT | Fayed |
| Yahya | Fayed |
| Julie | Fayon |
| S | Fayose |
| Samuel | Fayose |
| Ana | Fazenda |
| M | Fazlur Rahman |
| Marta | Fazzin |
| N | Fearon |
| IS | Febriana |
| S | Federer |
| P | Federico |
| M | Fediuk |
| Melanie | Fediuk |
| Florian | Fegg |
| M | Fehervari |
| D | Feingold |
| E | Fekaj |
| Liane | Feldman |
| Luis | Felipe Ávila-Ramírez |
| Luis | Felipe Cabrera Vargas |
| Alvaro | Felipe Guerrero Vergel |
| Diego | Felipe Tellez Beltran |
| A | Fell |
| Adam | Fell |
| Lucy | Fell |
| Emanuele | Felli |
| S | Felmban |
| S | Fendius |
| Sarah | Fendius |
| Anne-Sophie | Fenger |
| Nathalie | Fennell |
| Joseph | Fennelly |
| Humberto | Fenner Lyra Junior |
| Jibril | Fentaw |
| Mark | Fenton |
| Carlo V | Feo |
| CF | Feo |
| Claudio F | Feo |
| CV | Feo |
| Ksenia | Feoktistova |
| Sina | Ferahman |
| Alessandro | Ferdinando Ruffolo |
| Michael | Feretis |
| Maria | Fergadi |
| D | Ferguson |
| David | Ferguson |
| Douglas | Ferguson |
| H | Ferguson |
| Liam | Ferguson |
| S | Ferla |
| M | Fernadez |
| Luis | Fernand Betances |
| Maria | Fernanda Acuna Saravia |
| María | Fernanda Cedeño Bruzual |
| Maria | Fernanda Gonzalez Mosos |
| Maria | Fernanda Mijares Olivo |
| Maria | Fernanda Pedrero Escalas |
| Djanira | Fernandes |
| PHDS | Fernandes |
| Sara | Fernandes |
| Sarita | Fernandes |
| U | Fernandes |
| V | Fernandes |
| Vânia | Fernandes |
| R | Fernandes Rezende |
| Ricardo | Fernandes Rezende |
| Damaris | Fernandez |
| Jesus | Fernandez |
| Leticia | Fernandez |
| ML | Fernandez |
| Aida | Fernández |
| Esteban | Fernández |
| P | Fernández Bernabé |
| Patricia | Fernández Bernabé |
| Marlin | Fernandez Camilo |
| À | Fernández Camuñas |
| Alba | Fernández Candela |
| Elisabeth | Fernandez Castro |
| L | Fernández Gómez Cruzado |
| Laura | Fernández Gómez Cruzado |
| Maria | Fernandez LLorente |
| J | Fernández Manzano |
| MT | Fernández Martín |
| D | Fernández Martínez |
| Daniel | Fernández Martínez |
| M | Fernández Mendez |
| FJ | Fernández Pablos |
| Laura | Fernández Vega |
| A | Fernández-Candela |
| A | Fernandez-Colorado |
| Anna | Fernandez-Colorado |
| PV | Fernández-Fernández |
| Maria | Fernandez-Hevia |
| AJ | Fernández-López |
| Antonio-José | Fernández-López |
| MR | Fernández-Marín |
| Elena | Fernandez-Martin |
| A | Fernandez-Monge |
| Arantza | Fernandez-Monge |
| María-Carmen | Fernández-Moreno |
| Diego | Fernández-Samos Fernández |
| Paula | Fernandez-Valdes-Bango |
| L | Fernandez-Vega |
| Chamal | Fernando |
| D | Fernando |
| Daniel I | Fernando |
| Diego | Fernando Castillo-Cobaleda |
| Fabio | Fernando Eloi Pinto |
| Carlos | Fernando Roman Ortega |
| Diego | Fernando Ruiz Chiriboga |
| Jorge | fernando Tone |
| John | Ferns |
| Federica | Ferracci |
| A | Ferraiolo |
| Antonella | Ferrara |
| F | Ferrara |
| Francesco | Ferrara |
| Mariantonia | Ferrara |
| Paula | Ferrara |
| F | Ferrari |
| Federico | Ferrari |
| G | Ferrari |
| Giovanni | Ferrari |
| Maurizio | Ferrari |
| L | Ferrario |
| Luca | Ferrario |
| I | Ferraz |
| Inês | Ferraz |
| C | Ferreira |
| Cassandra | Ferreira |
| Cátia | Ferreira |
| Filipa | Ferreira |
| Gonçalo | Ferreira |
| Joaquim | Ferreira |
| Rocio | Ferreira |
| Carlos | Ferreira dos Santos |
| AP | Ferreira Pinto |
| F | Ferreli |
| Fabio | Ferreli |
| M | Ferrer Banús |
| A | Ferrer Fuertes |
| Ada | Ferrer Fuertes |
| Carolina | Ferrer Gomez |
| E | Ferrer-Inaebnit |
| Carlos | Ferreras García |
| J | Ferreres Serafini |
| P | Ferrero |
| S | Ferrero |
| Simone | Ferrero |
| E | Ferrero Herrero |
| Abel | Ferrés |
| CC | Ferro |
| C | Ferron |
| Jenny | Ferry |
| İbrahim | Fethi Azamat |
| Giacomo | Fiacchini |
| V | Ficarra |
| Andreas | Fichter |
| Valeria | Fico |
| Lovely | Fidelis |
| Mark | Field |
| Michael | Field |
| Xavier | Field |
| Drew | Fielder |
| Roberto | Fierro-Rizo |
| Marcelo | Figari |
| Helen | Figgins |
| Blas | Figueredo |
| Jatnna | Figueroa |
| Juan | Figueroa |
| Luis | Figueroa |
| Rodrigo | Figueroa |
| Rafael | Figueroa - Casanova |
| Carlos | Figueroa Avendaño |
| A | Figus |
| Papa | Fiifi - Yankson |
| RF | Filarca |
| RL | Filarca |
| Matteo | Filardo |
| Marco | Filauro |
| Venko | Filipce |
| MD | Filipe |
| D | Filipescu |
| Daniela | Filipescu |
| E | Filipov |
| Emil | Filipov |
| M | Filipponi |
| Eva | Filo |
| JG | Finch |
| Oliver | Findl |
| Alasdair | Findlay |
| L | Findlay |
| Austin | Findley |
| N | Fine |
| Andrea | Fink |
| Marcus | Fink |
| JB | Finkelstein |
| L | Finnegan |
| Michael | Finsterwald |
| M | Fiore |
| Marco | Fiore |
| S | Fiorelli |
| Silvia | Fiorelli |
| Guido | Fiorentini |
| E | Fiori |
| Enrico | Fiori |
| A | Fiorini |
| Alessandro | Fiorini |
| N | Firat |
| Necattin | Firat |
| Mohd | Firdauss Osman |
| Kanwal | Firdos |
| Fatin | Firman |
| Mohammadreza | Firouzifar |
| Anne | Fischer |
| I | Fischer |
| Ines | Fischer |
| Isabel | Fischer |
| B | Fish |
| Brian | Fish |
| A | Fisher |
| Y | Fishman |
| Yuri | Fishman |
| T | Fisseha |
| Tigist | Fisseha |
| Andreea | Fisus |
| Robert | Fitridge |
| Aidyl | Fitrisyah |
| Sophia | Fitt |
| C | Fitzgerald |
| Laura | Fitzmaurice |
| Mohammed | Fiyaz Chowdhry |
| Aleksander | Fjeld Haugstvedt |
| Silvia | Flachs Nóbrega |
| Etienne | Flamant |
| N | Flamey |
| Nicolas | Flamey |
| Michael | Flanagan |
| O | Flannery |
| M | Flatman |
| M | Flavin |
| Julio | Flavio Fiore Jr |
| Jose | Flavio Videira |
| Daniel | Fleitas |
| C | Fleming |
| Christina | Fleming |
| J | Fleming |
| F | Fleres |
| Francesco | Fleres |
| Angelica | Fletcher |
| Alana | Flexman |
| I | Flindall |
| M | Flint |
| Neil | Flint |
| Vojko | Flis |
| Souha | Fliss |
| M | Flitcroft |
| C | Flood |
| J | Flor |
| Delia | Florean |
| Krisniel | Florence Solis |
| Agustina | Florencia Castro Lalín |
| D | Flores |
| Mario-Andrés | Flores |
| Natalia | Flores Amador |
| Paola | Flores Becerril |
| R | Flores Clotet |
| AM | Floreskou |
| Ioan-Alexandru | Florian |
| Ignat | Florin |
| F | Floris |
| G | Fluegen |
| Georg | Fluegen |
| CD Q | Flumignan |
| R | Flumignan |
| RL G | Flumignan |
| Ronald | Flumignan |
| Roland | Flurschütz |
| William | Flynn |
| Philipp | Foessleitner |
| Amy | Fogarty |
| Alessandro | Fogliati |
| Samo | Fokter |
| Eugene | Foley |
| Katarina | Foley |
| Niamh | Foley |
| M | Folic |
| Miljan | Folic |
| S | Folli |
| A | Folorunso |
| Filipa | Fonseca |
| GB | Fonsi |
| M | Font |
| M | Fontana |
| T | Fontana |
| M | Fontanella |
| Marco | Fontanella |
| Jonathan | Foo |
| Chui | Foong Ong |
| Clara | Forbes |
| D | Ford |
| S | Ford |
| Samuel | Ford |
| Jennifer | Foreman |
| Jorge | Forero |
| Alexander | Forero-Torres |
| S | Forlani |
| Martin | Formánek |
| A | Fornasari |
| Anna | Fornasari |
| Alice | Fort-Schaale |
| Beniamino | Forte |
| L | Fortuna |
| Laura | Fortuna |
| Maruel | Fortunato |
| J | Foster |
| L | Foster |
| Paul | Foster |
| P | Fotheringham |
| C | Fotopoulou |
| Christina | Fotopoulou |
| Mohammed | Fouad |
| Mohamed | Fouad Elganainy |
| Daniel | Fountain |
| DM | Fountain |
| Olivier | Fouquet |
| L | Fourcade |
| Laurent | Fourcade |
| M | Fourtounas |
| A | Fowler |
| Alexander | Fowler |
| Amy | Fowler |
| Carolyn | Fowler |
| GE | Fowler |
| Adrian | Fox |
| Wiliam | Foy |
| Sara | Fra Fernández |
| Danniel | Frade Said |
| E | Fradelos |
| Evangelos | Fradelos |
| Gema | Fraga |
| Georgios | Fragkoulidis |
| Henri | Fragnaud |
| Socrates | Fragoulis |
| Uriel | Fraidenraij |
| A | Franceschi |
| Marzia | Franceschilli |
| Gianluca | Franceschini |
| Antonino | Francesco Germano |
| M | Franchi |
| G | Francis |
| Okedi | Francis Xaviour |
| Elaine | Francisca De Araújo |
| Salvador | Francisco Campos Campos |
| José | Francisco Farah |
| Murilo | Francisco Fernandes |
| Antonio | Francisco Guisado Calderón |
| Álvaro | Francisco Lopes de Sousa |
| Luis | Francisco Martín Anoro |
| Juan | Francisco Pintado Mejia |
| Labissi | Francois Amossou |
| E | Francone |
| Elisa | Francone |
| K | Frank |
| Konstantin | Frank |
| A | Frankel |
| Adam | Frankel |
| J | Franken |
| Josephine | Franken |
| K | Frankowska |
| Pietro | Fransvea |
| Helmut | Franz Georg Novotny |
| Albert | Franz Guerrero-Becerra |
| M | Franza |
| Mara | Franza |
| Alina | Franzen |
| M | Franzinelli |
| M | Franzini |
| Marco | Franzini |
| Denis | Frasca |
| M | Frascio |
| S | Fraser |
| Sheila | Fraser |
| S | Frassini |
| M | Frasson |
| Matteo | Frasson |
| A | Frati |
| Alessandro | Frati |
| C | Frattaruolo |
| Colomba | Frattaruolo |
| Antonio | Fratto |
| Wolfgang | Fraz |
| Fabien | Fredon |
| Ebru | Freed |
| J | Freedman |
| Hawys | Freeman |
| P | Fregatti |
| Piero | Fregatti |
| Ali | Freihat |
| M | Freijeiro |
| T | Freiman |
| Thomas | Freiman |
| A | Frena |
| Stefano | Fresilli |
| Claudia | Fretes |
| Martin | Fretes |
| K | Fretwell |
| Matthew | Freudmann |
| MR | Freund |
| C | Frew |
| A | Freyrie |
| Antonio | Freyrie |
| Christian | Freyschlag |
| K | Freystaetter |
| L | Frias |
| Roland | Fricker |
| Danielle | Friedman |
| Sabine | Friedrich |
| Pamela | Frigerio |
| H | Frima |
| F | Frio |
| Filippo | Friso |
| Tommaso | Frisoni |
| A | Fritz |
| Frank | Frizelle |
| S | Froghi |
| Caterina | Froiio |
| A | Frolova |
| Y | Frolova |
| Yulia | Frolova |
| F | Frongia |
| Federica | Frongia |
| A | Frontali |
| Alice | Frontali |
| K | Frosch |
| J | Frost |
| Rhiannon | Frostick |
| Maximos | Frountzas |
| Brytt | Frunt |
| Tiziana | Frusca |
| R | Fruscio |
| Robert | Fruscio |
| Diego | Frutos |
| Fares | Ftaieh |
| Panagiotis | Ftikos |
| H | Fu |
| Ali | Fuat Kaan Gok |
| Hans | Fuchs |
| Thomas | Fuchs-Buder |
| DP | Fudulu |
| L | Fuenmayor-González |
| JR | Fuentes |
| Lucía | Fuentes |
| T | Fuentes |
| Tyare | Fuentes |
| C | Fuentes Orozco |
| Clotilde | Fuentes Orozco |
| Lorena | Fuentes Rivera Lau |
| Alvaro | Fuentes-Martín |
| Reinhold | Függer |
| Mette | Fugleberg Nielsen |
| Ayataka | Fujimoto |
| Y | Fujimoto |
| Yuki | Fujimoto |
| Tomoyuki | Fujita |
| Yoshinori | Fujiwara |
| Yasuyuki | Fukami |
| D | Fuks |
| M | Fukuda |
| Satsuki | Fukushima |
| Christian | Fulghum |
| S | Fulginiti |
| Alamea | Fulivai |
| Mairi | Fullarton |
| Lucy | Fuller |
| U | Fumagalli Romario |
| Uberto | Fumagalli Romario |
| Kentaro | Fumoto |
| N | Fundano |
| Tania | Funes |
| C | Fung |
| Christian | Fung |
| Stephen | Fung |
| Man | Fung Ho |
| Kiu | Fung Wong |
| N | Furbetta |
| Niccolò | Furbetta |
| Hasim | Furkan Gullu |
| Ibrahim | Furkan Küçük |
| Chris | Furkert |
| M | Furlan |
| Micaela | Furlan |
| Yukari | Furuhata |
| Taku | Furukawa |
| Yuri | Furukawa |
| G | Fusai |
| Giuseppe | Fusai |
| Daniele | Fusario |
| Stefano | Fusetti |
| F | Fusini |
| Federico | Fusini |
| A | Fuson |
| Elisa | Fustec |
| K | Futaba |
| Kaori | Futaba |
| Konrad | Futyma |
| O | Fuwa |
| Sotonye | Fyneface-Ogan |
| Franz | G Bader |
| Jorge | G Boretto |
| Nasly | G Patino-Jaramillo |
| Anitha | G S |
| K | Gaballa |
| Khaled | Gaballa |
| Linda | Gabellini |
| M | Gaber |
| Ana | Gabersek |
| A | Gabr |
| Abdullah | Gabr |
| Ayman | Gabr |
| Hesham | Gabr |
| A | Gabre-Kidan |
| Juan | Gabriel Castro Ríos |
| Juan | Gabriel De Leon |
| Leyla | Gabriel Fernández |
| Esteban | Gabriel Jauregui |
| Justin | Gabriel Schlager |
| Mircea | Gabriel Stoleriu |
| José | Gabriel Yaryura Montero |
| Cristina | Gabriela Alzate Arsuaga |
| Claudia | Gabriela Mitrofan |
| Laura | Gabriela Peña Balboa |
| Angelo | Gabriele Epifani |
| Maria | Gabriella Dona |
| Sofia | Gabrilovich |
| H | Gacaferi |
| Hamez | Gacaferi |
| Mahir | Gachabayov |
| Dalia | Gad |
| Corina | Gaddi |
| A | Gadducci |
| Maya | Gade |
| R | Gadea Mateo |
| Ricardo | Gadea Mateo |
| Anup | Gadekar |
| Rabea | Gadelkareem |
| Inas | Gadelkarim |
| Ziyad | Gadelrab |
| P | Gadelsyed |
| Alice | Gadotti Yasuda |
| Taha | Gadouali |
| B | Gafsi |
| A | Gagliano |
| F | Gagliardi |
| Filippo | Gagliardi |
| N | Gagné |
| Joel | Gagnier |
| Gunilla | Gagnö |
| Lara | Gahan |
| Nitesh | Gahlot |
| Yarub | Gahtan |
| S | Gahunia |
| Sukhpreet | Gahunia |
| Milad | Gahwagi |
| F | Gaino |
| Francesca | Gaino |
| A | Gainza |
| Stylianos | Gaitanakis |
| A | Gaitanidis |
| Apostolos | Gaitanidis |
| K | Gajjar |
| Ketankumar | Gajjar |
| Shreyash | Gajjar |
| Urska | Gajsek |
| Delali | Gakpetor |
| T | Gala |
| Tanzeela | Gala |
| K | Galaal |
| Jacopo | Galafassi |
| S | Galal Eldin |
| Ilias | Galanis |
| C | Galata |
| B | Galbreath |
| Zinaida | Galchikova |
| Alessandro | Galdini |
| Mark | Galea |
| Ana | Galevska-Dimitrovska |
| Elisa | Galfrascoli |
| M | Galhoum |
| Mohamed | Galhoum |
| Luis | Galindo |
| Lorena | Galindo Iñiguez |
| P | Galindo Jara |
| Pablo | Galindo Jara |
| Antonio | Galindo Nava |
| M | Galipienso Eri |
| B | Gális |
| Branislav | Gális |
| B | Gallagher |
| K | Gallagher |
| Nicola | Gallagher |
| P | Gallagher |
| T | Gallagher |
| L | Gallardo zamora |
| R | Galleano |
| Raffaele | Galleano |
| Lander | Gallego |
| Diego | Gallegos |
| R | Galli |
| E | Galliamov |
| Eduard | Galliamov |
| A | Gallinat |
| G | Gallo |
| Gaetano | Gallo |
| O | Gallo |
| Oreste | Gallo |
| Daniel | Galun |
| A | Galvan |
| Armando | Galvan |
| Yaiza | Galvañ Félix |
| A | Galvan Pérez |
| Heloisa | Galvão do Amaral Campos |
| M | Galvarini |
| T | Gamage |
| Abrar | Gamal |
| Dina | Gamal |
| Ibrahim | Gamal |
| Mennatullah | Gamal |
| S | Gamal Badr |
| Nehal | gamal Omar |
| Mohamed | Gamal Taher |
| Erick | Gamaliel Amba |
| C | Gambacciani |
| Denise | Gambardella |
| Ahmed | Gameel |
| Hammaad | Gamieldien |
| E | Gammeri |
| Abdulmohimen | Gammoudi |
| SW | Gan |
| Grace | Gana |
| S | Gananadha |
| Thanga | Ganapathy |
| M | Ganau |
| Mario | Ganau |
| Lundeg | Ganbold |
| Sumir | Gandhi |
| Suraj | Gandhi |
| Pablo | Gandia Gonzalez |
| Kugarajh | Ganeshapillai |
| Gagana | Ganga |
| Srinivasan | Gangadharan |
| J | Gani |
| Jonathan | Gani |
| Babak | Ganjeifar |
| I | Ganly |
| Ian | Ganly |
| S | Gans |
| Abel | Ganso |
| A | Ganta |
| Antonia | Gantschnigg |
| Jiali | Gao |
| George | Garas |
| Daniel | Garay Lechuga |
| Stephen | Garba |
| Saudat | Garba Habib |
| E | Garca Rico |
| R | Garcés García |
| Raúl | Garcés García |
| Sofía | Garcés Palacios |
| M | Garcés-Albir |
| A | Garcia |
| Alba | Garcia |
| Andrea | Garcia |
| Cesar | Garcia |
| D | Garcia |
| David | Garcia |
| Felipe | Garcia |
| Francisco | Garcia |
| Iset | Garcia |
| Luis | Garcia |
| Luz | Garcia |
| Manuel | Garcia |
| Sean | Garcia |
| A | García |
| Antonio | García |
| Federico | García |
| J | García |
| M | García |
| Lucia | Garcia Alcalde |
| M | Garcia Alonso |
| Cristina | García Amador |
| C | Garcia astrada |
| LA | García Barrionuevo |
| S | Garcia Botella |
| Juan | García Cardo |
| Carlos | Garcia Cardona |
| Miguel | García Castillo |
| Vázquez | García César |
| Elena | Garcia De Castro |
| E | García de Castro Rubio |
| U | Garcia de cortazar |
| Unai | Garcia De Cortazar |
| Amaia | Garcia Dominguez |
| Antonio | García Domínguez |
| Melody | García Domínguez |
| J | Garcia Egea |
| D | García Escudero |
| Damián | García Escudero |
| LJ | García Flórez |
| JL | Garcia galocha |
| DS | García García |
| Elena | García García |
| JJ | Garcia Gutierrez |
| Saura | García Laura |
| D | Garcia López de Goicoechea |
| V | García Milán |
| JD | Garcia Montesino |
| Mauricio | Garcia Mora |
| Tito | García Moreno |
| G | García Operé |
| Victoria | Garcia Peces |
| D | Garcia Perez |
| Cristina | García Pérez |
| JM | García Pérez |
| Pablo | Garcia Pimentel |
| EP | Garcia Santos |
| Miguel | García Sanz |
| Javier | Garcia Septiem |
| J | García Septiem |
| Vanesa | Garcia Soria |
| S | García Valenzuela |
| O | García Villar |
| JE | García Villayzán |
| Mariana | García Virosta |
| Jorge | Garcia-Adamez |
| David | Garcia-Aguilera |
| L | Garcia-Aparicio |
| Luis | García-Aparicio |
| J | Garcia-Borda |
| Hector | Garcia-Chavez |
| M | García-Conde |
| U | Garcia-Dubus Rodriguez |
| D | García-Escudero |
| Luis | Garcia-Florez |
| Francisco | Garcia-Huidobro |
| E | Garcia-Loarte Gomez |
| Daniel | García-López |
| Belen | Garcia-Montesinos Perea |
| Oscar-Julián | García-Montoya |
| Francisca | Garcia-Moreno Nisa |
| M | García-Nebreda |
| Guillermo | García-Operé |
| Herney | Garcia-Perdomo |
| V | Garcia-Pineda |
| V | García-Porcel |
| J | García-Quijada |
| L | Garcia-Sancho Tellez |
| Luis | Garcia-Sancho Tellez |
| V | García-Soria |
| MA | García-Ureña |
| V | Garcia-Virto |
| Virginia | Garcia-Virto |
| V | García-Virto |
| Jeronimo | Garcialopez De Llano |
| Laura | Garden |
| Padraig | Gardiner |
| A | Gardner |
| E | Gardner |
| Elena | Garelli |
| R | Garfinkle |
| K | Garg |
| Mayank | Garg |
| PK | Garg |
| Rajnish | Garg |
| Surabhi | Garg |
| Vipul | Garg |
| G | Garganese |
| S | Garibaldi |
| M | Garino |
| Mauro | Garino |
| T | Garmanova |
| Tatyana | Garmanova |
| Gregorio | Garmendia |
| M | Garner |
| Madeleine | Garner |
| A | Garnero |
| Z | Garoufalia |
| Zoe | Garoufalia |
| R | Garrido |
| S | Garrido |
| S | Garrido-Ondono |
| Gonzalo | Garrigos |
| Richard | Gartrell |
| Kendyll | Gartrelle |
| G | Garulli |
| S | Garusinghe |
| George | Gasana |
| I | Gascon Ferrer |
| Isabel | Gascon Ferrer |
| I | Gascon-Ferrer |
| Peter | Gaskell |
| Cameron | Gaskill |
| KAD | Gasmalla |
| Anis | Gasmi |
| Giulio | Gasparini |
| Hrvoje | Gasparovic |
| ML | Gasparri |
| J | Gass |
| M | Gass |
| Markus | Gass |
| A | Gasset-Teixidor |
| L | Gasteiger |
| Matt | Gaston |
| A | Gasulla-Rodriguez |
| Anna | Gasulla-Rodriguez |
| VA | Gata |
| Anna | Gateley |
| Lucija | Gatin |
| Juan | Gatón |
| Inbar | Gatot |
| Francesca | Gatta |
| A | Gatti |
| Arthur | Gatti |
| A | Gattolin |
| Andrea | Gattolin |
| S | Gattoni |
| Serena | Gattoni |
| Domenico | Gattulli |
| S | Gaujoux |
| Sebastien | Gaujoux |
| John | Gaul |
| A | Gaunt |
| A | Gaurav |
| Neha | Gauri |
| Claudia | Gauto |
| Julio | Gauto |
| Guilherme | Gava |
| MT | Gavaldà Pellicé |
| Valerie | Gaveh |
| G | Gavilanes Loor |
| Haim | Gavriel |
| TS | Gavriliu |
| Mothana | Gawad |
| Mohan | Gawande |
| Wladyslaw B | Gawel |
| Larsa | Gawria |
| I | Gawron |
| Oswald | Gbehade |
| Olalere | Gbolahan |
| Tjokorda | Gde Agung Senapathi |
| D | Gearon |
| E | Geary |
| M | Geary |
| Michael | Geary |
| A | Gebran |
| Anthony | Gebran |
| Hiwot | Gebre |
| F | Gebreegziabher Gebrehiwot |
| Fitsum | Gebreegziabher Gebrehiwot |
| Gebreagziabher | Gebrekirstos |
| Zersenay | Gebremeskel |
| M | Gebreyohanes Mengesha |
| Mengistu | Gebreyohanes Mengesha |
| Gabriele | Gecchele |
| I | Gecim |
| A | Geddes |
| Yetsedaw | Gedefaw |
| R | Gefen |
| A | Gegundez Simon |
| Alberto | Gegúndez Simón |
| AA | Geisler |
| E | Gelarda |
| Enrico | Gelarda |
| Chiara | Gelati |
| Ryan | Geleit |
| Jaume | Gelonch |
| Scott | Gelzinnis |
| Lidya | Gemechu |
| NA | Gemelli |
| G | Gemes |
| C | Gemmell |
| E | Gemmill |
| Elizabeth | Gemmill |
| J | Gempt |
| Jens | Gempt |
| Eben-ezer | Genda |
| Y | Genda |
| George | Gendrikson |
| Sileshi | Genetu |
| Abraham | Genetu Tiruneh |
| S | Gennari |
| M | Gennaro |
| Massimiliano | Gennaro |
| Ludivine | Genre |
| L | Genser |
| Laurent | Genser |
| Fred | Gentili |
| S | Gentilli |
| Sergio | Gentilli |
| Charles | Geoffrey Dermot Stewart |
| J | Geoghegan |
| Clemens | Georg Wiesinger |
| B | George |
| Gejoe | George |
| K | George |
| R | George |
| Smitha | George |
| Suku | George |
| John | George Massoud |
| P | George Pandeth |
| SA | George Varayannoor |
| Tom | Georgi |
| F | Georgiades |
| Fanourios | Georgiades |
| Gregory | Georgiadis |
| D | Georgiadou |
| D | Georgiev |
| G | Georgieva |
| Gordana | Georgieva |
| Irina | Georgieva |
| Sotirios | Georgios Popeskou |
| Nikolaos | Georgopoulos |
| N | Georgopoulou |
| Kleoniki | Georgousi |
| J | Geraghty |
| David | Gerardo Miranda Gómez |
| C Gerber | Gerber |
| Anna | Gergen |
| F | Gerges |
| A | Germain |
| Alicia | German Dihmes |
| Paola | Germani |
| Christoph-Thomas | Germer |
| Giuliana | Germinario |
| Daniel | Gero |
| I | Gerogiannis |
| SL | Gerritsen |
| A | Gerundo |
| S | Gerus |
| E | Gessa |
| M | Gessesse |
| F | Gessler |
| Florian | Gessler |
| Michael | Gessner |
| Eneyew | Getachew Siyoum |
| Hanna | getachew Woldeselassie |
| Aderaw | Getie |
| Anne | Getz |
| Khaled | Ghabban |
| Raghad | Ghadri |
| S | Ghaem-Maghami |
| M | Ghaemi |
| F | Ghaffarizadeh |
| H | Ghaith |
| Mohamed | Ghali |
| Asma | Ghallab |
| Basmah | Ghallab |
| Amol | Ghalme |
| G | Ghaly |
| Galal | Ghaly |
| Mira | Ghaly |
| Reem | Ghamgh |
| Naji | Ghamri |
| A | Ghanbari |
| K | Ghandour |
| Hossam | Ghanem |
| R | Ghanem |
| Anum | Ghani |
| A | Ghannam |
| Abdelilah | Ghannam |
| R | Ghannam |
| Rana | Ghannam |
| I | Gharbi |
| Khaled | Gharbia |
| Mulham | Gharib |
| Ruwaid | Gharib |
| ABDUlRAOUF | Ghariba |
| H | Ghattaura |
| Sakina | Ghauth |
| Anas | Ghawi |
| Ibrahim | Ghayada |
| Sumayyah | Ghayth Bahroun |
| A | Ghazal |
| Ahmad | Ghazal |
| Muhammad | Ghazali Hasheem |
| Ramlah | Ghazanfor Ghazanfor |
| Mojahid | Ghazi |
| O | Ghazouani |
| Salman | Ghazwani |
| Rahel | Ghebre |
| S | Ghedan |
| Marwane | Ghemame |
| F | Ghezzi |
| SS | Ghiasi |
| F | Ghini |
| Bennis | Ghita |
| Peter | Ghiya |
| M | Ghobrial |
| Marios | Ghobrial |
| R | Ghodke |
| Rahul | Ghodke |
| Mohammad | Ghomeisi |
| Alaa | Ghonaim |
| Mohamed | Ghonaim |
| A | Ghoneim |
| Ahmed | Ghoneim |
| M | Ghoneim |
| Ali | Ghorbani Abdehgah |
| Paloma | Ghosal |
| D | Ghosh |
| Dhruv | Ghosh |
| Indranil | Ghosh |
| K | Ghosh |
| Rukmini | Ghosh |
| A | Ghouri |
| Amna | Ghouri |
| Youssef | Ghoussoub |
| S | Ghozy |
| Sherief | Ghozy |
| K | Ghufoor |
| Bhavisha | Ghugare |
| Muhammad | Ghulam Qadir Qadir |
| Zohal | Ghulam-Jelani |
| Saqib | Ghumman |
| M | Ghunaim |
| Mohammed | Ghunaim |
| M | Giacometti |
| Marco | Giacometti |
| Anna | Giacomina Carta |
| E | Gialamas |
| Eleftherios | Gialamas |
| D | Gianardi |
| Accarino | Giancarlo |
| Maria | Giangreco |
| Alessandro | Giani |
| G | Giannaccare |
| Giuseppe | Giannaccare |
| A | Gianni |
| A | Giannini |
| L | Giannini |
| Lorenzo | Giannini |
| F | Giannoulis |
| T | Giannoulopoulos |
| Uyen | Giao Vo |
| Alessia | Giaquinta |
| M | Giardini |
| Matteo | Giardini |
| Alessandro | Giardino |
| Ludovica | Gibelli |
| A | Giblin |
| Anna-Victoria | Giblin |
| C | Gibson |
| Joanna | Gibson |
| Manuel | Gielis |
| Cecilia | Gigena |
| J | Gigliotti |
| Giacomo | Gigliucci |
| CG | Gil |
| Marta | Gil |
| Lucia | Gil Cidoncha |
| Catarina | Gil Gil |
| J | Gil Martínez |
| Inés | Gil Prados |
| Ismael | Gil Romea |
| PJ | Gil Vázquez |
| O | Gil-Albarova |
| Oscar | Gil-Albarova |
| A | Gil-Catalan |
| Oscar | Gil-de-Sagredo |
| A | Gil-Moreno |
| J | Gil-Rodriguez |
| Daniel | Gil-Sala |
| J | Gilabert Estellés |
| Juan | Gilabert-Estellés |
| C | Gilbert |
| Gasengayire | Gilbert |
| S | Gili |
| Simona | Gili |
| Mireia | Gili-Bueno |
| C | Gill |
| DF | Gill |
| H | Gill |
| Ian | Gill |
| Parmilan | Gill |
| S | Gillani |
| SFM | Gillani |
| Andrew | Gillard |
| Lars | Gillberg |
| C | Gillezeau |
| J | Gilliland |
| Jack | Gilliland |
| AE | Gillis |
| Amy | Gillis |
| Katie | Gilmore |
| G | Gilna |
| Gareth | Gilna |
| Clara | Giménez Francés |
| T | Gimenez Maurel |
| Teresa | Gimenez Maurel |
| C | Giménez-Francés |
| Antonio | Gimenez-Gaibar |
| Mar | Gimeno Gimeno Vicente |
| Jesus | Gimeno Hernandez |
| Cesar | Ginesta |
| Christian | Gingert |
| O | Ginghina |
| Octav | Ginghina |
| Audrey | Giocanti-Auregan |
| R | Gioco |
| Rossella | Gioco |
| V | Giordano |
| Andrea | Giorga |
| E | Giorgakis |
| Emmanouil | Giorgakis |
| Federico A | Giorgini |
| E | Giotakis |
| Evangelos | Giotakis |
| Konstantinos | Gioutsos |
| Jesús | Giovanni Inzunza Miranda |
| Luca | Giovanni Locatello |
| M | Giovenzana |
| D | Giovinazzo |
| F | Giovinazzo |
| Jordi | Giralt López de Sagredo |
| Ricardo | Girao |
| E | Girard |
| Edouard | Girard |
| Ève-Marie | Girard |
| Karine | Girard |
| Noémie | Girard |
| Camila | Girardi Fachin |
| G | Giraudo |
| Giorgio | Giraudo |
| B | Giray |
| Burak | Giray |
| Nathalie | Girgis |
| Adissu | Girma |
| F | Giron Luque |
| Fernando | Giron Luque |
| E | Girsowicz |
| SS | Gisbertz |
| Suzanne | Gisbertz |
| Laura | Gisela Alvarez Calzaretta |
| Laura | Giselle Contreras Baquero |
| Shanthamoorthy | Gishanthan |
| Martin | Gisinger |
| Francis | Githae Muriithi |
| Adam | Gittins |
| Marco | Giudice |
| R | Giudici |
| M | Giuffrida |
| Mario | Giuffrida |
| MC | Giuffrida |
| Bonfanti | Giulia |
| Vitiello | Giulia |
| Maria | Giulia Cristofaro |
| A | Giuliani |
| B | Giuliani |
| Giuliana | Giuliani |
| Tommaso | Giuliani |
| Cesar | Giuliano Sisa Segovia |
| P | Giulianotti |
| Pierluigi | Giumelli |
| Marco | Giuseppe Iannuccelli |
| Elia | Giuseppe Lunghi |
| Marcello | Giuseppe Spampinato |
| A | Gjata |
| Arben | Gjata |
| Ulpjana | Gjondedaj |
| Kostandin | Gjyli |
| Ioanna | Gkalonaki |
| Ioannis | Gkekas |
| Georgios | Gkiokas |
| Antonios | Gklavas |
| Nick | Gkolias |
| E | Gkrinia |
| Eleni | Gkrinia |
| Laurence | Glancz |
| N | Glass |
| Nina | Glass |
| O | Glehen |
| Ana | Gleisner |
| P | Glen |
| Alexa | Glencer |
| I | Glisovic Jovanovic |
| Claudio | Glowalla |
| Tim R | Glowka |
| TR | Glowka |
| Zhanna | Glushchenko |
| Tamara | Glyn |
| Orna | Glynn |
| C | Gnanachandran |
| Subhadra | Goala |
| S | Gobishangar |
| Huseyin | Gobut |
| I | Gockel |
| Ines | Gockel |
| C | Godbole |
| Jerry | Godfrey Makama |
| N | Godin |
| M | Godinho |
| Gaëlle | Godiris Petit |
| André | Godoy |
| Rosana | Godoy |
| J | Goedeke |
| Jan | Goedeke |
| N | Goel |
| Neha | Goel |
| T | Goel |
| MA | Goh |
| N | Goh |
| Farhan | Gohar |
| M | Gohar |
| Muhammad | Gohar |
| Amish | Gohil |
| K | Gohil |
| Raluca | Goicea |
| Amy | Gojnich |
| E | Gokcen |
| N | Gokhare Viswanath |
| B | Göksoy |
| Krishnan | Gokul |
| Mohammadreza | Golbakhsh |
| H | Golcher |
| Ikponmwosa | Gold |
| Ruthie | Gold- Deutch |
| A | Goldenberg-Sandau |
| Anna | Goldenberg-Sandau |
| Carma | Goldstein |
| D | Golijanin |
| Danica | Golijanin |
| S | Göller |
| Can | Gollmann-Tepeköylü |
| Grigoriy | Gololobov |
| A | Golomidov |
| Maher | Gomaha |
| Fabio | Gomes |
| GM A | Gomes |
| GMA | Gomes |
| H | Gomes |
| Alden | Gomez |
| Alejandro | Gomez |
| Dhanwant | Gomez |
| Hugo | Gomez |
| Humberto | Gomez |
| J | Gomez |
| Laura | Gomez |
| P | Gomez |
| Viviana | Gomez |
| Nadia | Gómez |
| Manuel | Gomez Cervantes |
| CJ | Gómez Díaz |
| H | Gomez Fernandez |
| L | Gomez Fernandez |
| Laura | Gomez Fernandez |
| L | Gomez Lopez |
| JR | Gómez López |
| SC | Gómez López |
| B | Gómez Pérez |
| Beatriz | Gómez Pérez |
| J | Gomez Rivas |
| Juan | Gómez Rivas |
| Carlos | Gomez Roig |
| Maria | Gómez Romero |
| Nuria | Gomez Romeu |
| Tania | Gómez Sanz |
| J | Gómez Suárez |
| Paula | Gómez Valles |
| Leticia | Gómez Viana |
| S | Gomez-Abril |
| Segundo | Gomez-Abril |
| Francisco | Gómez-Bosch |
| H | Gomez-Fernandez |
| Hugo | Gomez-Fernandez |
| A | Gómez-Pedraza |
| Antonio | Gómez-Pedraza |
| J | Gomez-Rosado |
| Juan-Carlos | Gomez-Rosado |
| T | Gómez-Sanz |
| Álvaro | Gonçalves |
| BT | Gonçalves |
| JP | Gonçalves |
| N | Gonçalves |
| Rodrigo | Gonçalves |
| Rita | Gonçalves Pereira |
| KM | Gondal |
| Lior | Gonen |
| Bernhard | Gonschor |
| E | Gonullu |
| Emre | Gönüllü |
| AD | Gonzales |
| DS | Gonzalez |
| Elena | Gonzalez |
| Felipe | Gonzalez |
| J | Gonzalez |
| Javier | Gonzalez |
| Judit | Gonzalez |
| Marcos | Gonzalez |
| Nelson | Gonzalez |
| Paloma | Gonzalez |
| R | Gonzalez |
| E | González |
| Enrique | González |
| Gloria | González |
| Natalia | Gonzalez Alcolea |
| FX | Gonzalez Argente |
| Miren | Gonzalez Benito |
| Rebeca | Gonzalez Celdran |
| M | Gonzalez De Miguel |
| Melania | González de Miguel |
| Carlos | Gonzalez De Pedro |
| Daniel | Gonzalez Garcia-Cano |
| JA | Gonzalez Lopez |
| E | González Marín |
| Adelina | Gonzalez Martinez |
| A | Gonzalez Ojeda |
| Alejandro | Gonzalez Ojeda |
| Marta | González Pérez |
| Elena | González Revilla |
| Esteban | Gonzalez Salazar |
| SM | González Soares |
| S | Gonzalez Suarez |
| DS | Gonzalez Vazquez |
| Rocio | Gonzalez-Aguado |
| Carolina | Gonzalez-Gomez |
| E | Gonzalez-Gonzalez |
| Rogelio | González-López |
| MT | Gonzalez-Nicolas-Trebol |
| Alejandro | González-Orozco |
| Susana | González-Suárez |
| FM | González-Valverde |
| Azucena | Gonzalo |
| Paula | Gonzálvez Guardiola |
| S | Goodrum |
| Peter | Goodwin |
| Ferhana | Gool |
| Janindu | Goonawardena |
| Gomathy | Gopal |
| S | Gopalswamy |
| R | Gopi Reddy |
| Praveen | Gopinath |
| Rajesh | Gopireddy |
| Merima | Goran |
| R | Goran |
| Jeremias | Goransky |
| Raghunandan | Gorantlu Chowdappa |
| Sergey | Gordeyev |
| L | Gordini |
| Luca | Gordini |
| Alasdair | Gordon |
| Mangesh | Gore |
| Stefanos | Gorgoraptis |
| A | Gori |
| M | Goricar |
| Matej | Goricar |
| David | Gorin |
| Hugo | Gornes |
| S | Gortazar |
| Sara | Gortázar de las Casas |
| Christina | Gory |
| A | Gosain |
| Ankush | Gosain |
| M | Gosau |
| Martin | Gosau |
| Matthew | Goss |
| Karo | Gosselin |
| M | Gosselink |
| Martijn | Gosselink |
| D | Gossot |
| Yvonne | Goßlau |
| Lucy | Gossling |
| Mark | Gotecha |
| Iltimass | Gouazar |
| A | Goubran |
| Alex | Goubran |
| Omar | Gouda |
| Swati | Goudar |
| Ben | Goudsmit |
| N | Gougoulias |
| C | Goumard |
| Claire | Goumard |
| Lysander | Gourbault |
| Ralph | Gourlay |
| Konstantinos | Gousias |
| Nikolaos | Gouvas |
| Henry | Govekar |
| Arantza | Govela Hinojosa |
| A | Govil |
| Akhil | Govil |
| Mithila | Govind |
| M | Gowda |
| Ravikanth | Gowder |
| Benjamin | Gowers |
| A | Goyal |
| Amit | Goyal |
| Y | Gozal |
| Yaacov | Gozal |
| K | Gözal |
| D | Gp |
| Michael | Graber |
| P | Grabowski |
| Rosalind | Grace Beckett |
| Heather | Grace Dulnuan |
| Harelimana | Grace James |
| Linda | Grace Puerto Tamayo |
| Eileen | Grace Tancinco |
| Charmaine | Grace Valeros |
| Cesar | Gracia |
| I | Gracia |
| Isabel | Gracia |
| M | Gracia |
| C | Gracia-Roche |
| Carlos | Gracia-Roche |
| G | Gradinariu |
| George | Gradinariu |
| Christian | Graeb |
| C | Graham |
| C | Grainger |
| T | Grainger |
| F | Grama |
| Florin | Grama |
| Marco | Gramellini |
| Madelyn | Gramlick |
| V | Granata |
| Michele | Grande |
| Alessandro | Grandi |
| Samuele | Grandi |
| Steven | Grandjean |
| Carmen | Grañén |
| Lucas | Granero |
| S | Granieri |
| C | Granja |
| Cristina | Granja |
| James | Grantham |
| Bianca | Grassano |
| E | Grasset |
| C | Grassi |
| T | Grassi |
| Tommaso | Grassi |
| A | Grasso |
| M | Grasso |
| João | Graveto |
| A | Gray |
| H | Gray |
| Matthew | Gray |
| Maria | Grazia Matarazzo |
| Jacopo | Graziosi |
| M | Grechenig |
| Michael | Grechenig |
| A | Grechi |
| Alessandro | Grechi |
| G | Grecinos |
| Gustavo | Grecinos |
| L | Green |
| S | Green |
| Sofia | Green |
| Jennifer | Greenberg |
| M | Greenhalgh |
| Michael | Greenhalgh |
| MS | Greenhalgh |
| H | Greenlee |
| D | Greenman |
| Dmitry | Greenman |
| Rebecca | Greenop |
| A | Gregg |
| Emilie | Gregoire |
| P | Gregoric |
| Pavle | Gregoric |
| Minja | Gregorič |
| Paulo | Gregorio |
| Gordon | Gregory |
| Kate | Gregory |
| Thomas | Gregory |
| Veronika | Greif |
| Jens | Greve |
| Dilraj | Grewal |
| Rebecca | Grey |
| Maja | Grgec Dragicevic |
| Petar | Gribnev |
| Benjamin | Gricks |
| C | Grieco |
| Christian | Grieco |
| Ryan | Griffin |
| XL | Griffin |
| E | Griffiths |
| Ewen | Griffiths |
| T | Griffiths |
| A | Grigonytė |
| M | Grigoroiu |
| Florian | Grill |
| Richard | Grills |
| JV | Grilo |
| Gabriella | Grima |
| G | Grimbizis |
| C | Grimes |
| E | Grimley |
| C | Grimm |
| Christoph | Grimm |
| A | Grimonprez |
| M | Grishenko |
| A | Grivas |
| T | Grivas |
| A | Groen |
| LC | Groen |
| A | Grogan |
| R | Grolman |
| A | Gronchi |
| Alessandro | Gronchi |
| G | Groot |
| Gary | Groot |
| J | Grosek |
| Jan | Grosek |
| Jefferson | Gross |
| JL | Gross |
| U | Grossi |
| Ugo | Grossi |
| Travis | Grotz |
| Ellen | Groundwater |
| T | Grove |
| Kresimir | Grsic |
| Željko | Grubač |
| Michaela | Gruber |
| R | Gruber |
| Ricarda | Gruber |
| N | Grubor |
| Nikola | Grubor |
| Danica | Grujičić |
| Adrian | Grullon |
| Karlo | Grulović |
| Martin | Grünbart |
| Catherine | Grundy |
| N | Grundy |
| L | Grüßer |
| Linda | Grüßer |
| R | Grützmann |
| Robert | Grützmann |
| I | Grypiotis |
| Ioannis | Grypiotis |
| S | Guadagni |
| M | Guaglio |
| Marcello | Guaglio |
| E | Guaitoli |
| Javier | Gualis |
| M | Guardia |
| N | Guàrdia |
| Luciano | Guarienti |
| CA | Guariglia |
| David | Guarin |
| E | Guasch |
| M | Gubbiotti |
| Marilena | Gubbiotti |
| Ali | Guboug |
| Rita | Gudaityte |
| A | Gudal |
| Vasubabu | Gudala |
| Senyo | Gudugbe |
| HJ | Guedes Neto |
| Noemi | Guemes-Villahoz |
| C | Guerci |
| Claudio | Guerci |
| Sonia | Guérin |
| Bayron | Guerra |
| EE | Guerra |
| Glen R | Guerra |
| Jeffy | Guerra |
| FJ | Guerra Brandt |
| Odoniel | Guerra Garcia |
| E | Guerra-Farfan |
| Ernesto | Guerra-Farfan |
| P | Guerreiro |
| Francesco | Guerrera |
| Fatherin | Guerrero |
| Claudia | Guerrero Martinez |
| A | Gueutier |
| Alexandre | Gueutier |
| J | Guevara |
| O | Guevara |
| Oscar | Guevara |
| R | Guevara |
| Ika | Gugić Radojković |
| L | Guglielmetti |
| Laura | Guglielmetti |
| A | Guglielmi |
| Alfredo | Guglielmi |
| Mario | Guglielmo |
| Nicola | Guglielmo |
| S | Guha |
| S | Guicciardi |
| Gustavo | Guida |
| B | Guidi |
| Gonçalo | Guidi |
| Afrika | Guido |
| Marco | Guido Confalonieri |
| Christopher A | Guidry |
| C | Guijarro Moreno |
| Jeremy | Guilford |
| José | Guilherme Gonçalves Nobre |
| Jose | Guilherme Vartanian |
| Laura | Guillamon Vivancos |
| P | Guillamot Ruano |
| Paloma | Guillamot Ruano |
| Hander | Guillermo Acosta Diaz |
| Andre | Guimaraes |
| Lilian | Guimaraes |
| A | Guimarães |
| André | Guimarães |
| Felipe | Guimarães Pugliesi |
| MA C | Guimaraes-Filho |
| Patrice | Guiraudet |
| John | Guirguis |
| María | Guisasola Rabés |
| Rohan R | Gujjuri |
| Ambrin | Gul |
| Ayaz | Gul |
| Hina | Gul |
| Sana | Gul |
| Brian | Gulack |
| Emre | Gülçek |
| CE | Guldogan |
| Mert | Güler |
| OC | Güler |
| SA | Güler |
| A | Gulla |
| Aiste | Gulla |
| Murat | Gultekin |
| A | Gumarao |
| S | Gumede |
| Yohesuwary | Gunarasa |
| Filip | Gunaric |
| Nalaka | Gunawansa |
| I | Gunawardena |
| Indu | Gunawardena |
| J | Gundara |
| E | Gundogdu |
| EC | Gundogdu |
| Emre | Gundogdu |
| A | Guner |
| Ali | Guner |
| A | Gunjotikar |
| Matías | Günther Wood |
| A | Gupta |
| Amit | Gupta |
| Anand | Gupta |
| Ashish | Gupta |
| Himani | Gupta |
| Ishita | Gupta |
| L | Gupta |
| Michael | Gupta |
| Rahul | Gupta |
| Sameer | Gupta |
| Shivangi | Gupta |
| Shubhra | Gupta |
| Stuti | Gupta |
| Sujoy | Gupta |
| Addisalem | Gurara |
| Ahmet | Guray Durmaz |
| B | Gurbuz |
| Alican | Güreşin |
| E | Güresir |
| Erdem | Güresir |
| Radu | Gurghiș |
| Muhammed | Gürlük |
| Tegenu | Gurmu |
| E | Gurrea-Almela |
| Elena | Gurrea-Almela |
| Thomas | Gürtler |
| P | Gurung |
| N | Gusani |
| Aleksandr | Gusev |
| C | Gustavino |
| Claudio | Gustavino |
| G | Gutiérrez Carrillo |
| Gonzalo | Gutiérrez Carrillo |
| D | Gutiérrez Medina |
| Valentina | Gutiérrez Perdomo |
| Alitza | Gutiérrez Ruiz |
| M | Gutierrez Samaniego |
| Maria | Gutierrez Samaniego |
| Bernardo | Gutiérrez Sougarret |
| JA | Gutiérrez Vásquez |
| Jose | Gutierrez-Banos |
| CA | Gutschow |
| Fatih | Guven |
| Lillian | Guzman |
| Marlin | Guzman |
| Natalia | Guzman |
| T | Guzman |
| L | Guzmán |
| Marco | Guzzo |
| Sadiya | Gwadabe |
| Usman | Gwaram |
| GP | Gwini |
| Grace | Gwini |
| Solomon | Gyabaah |
| Daniel | Gyawu Aning |
| Adam | Gyedu |
| Derrick | Gyimah |
| Elitsa | Gyokova |
| A | Gyori |
| Joseph | Gyuro |
| G | Gyurok |
| Anton | H Schwabegger |
| Jorman | H Tejada |
| Jeong | Ha |
| Patrick | Ha |
| Mohamed | Habad |
| Charlène | Habarugira Inyange |
| A | Habeeb |
| MK | Habeeb |
| O | Habeeb |
| A | Habeebullah |
| Alaa | Habeebullah |
| Awais | Habeebullah |
| E | Haberal |
| Peter | Habertheuer |
| N | Habib |
| Zakaria | Habib |
| Sosthene | Habumuremyi |
| NA | Hacim |
| A | Hackl |
| Danilo | Hackner |
| J | Hadaya |
| Joseph | Hadaya |
| E | Haddad |
| Elie | Haddad |
| S | Haddad |
| Alyazeed | Haddadin |
| Basel | Haddadin |
| Monique | Haddleton |
| James | Haddow |
| James | Hadfield |
| JN | Hadfield |
| S | Hadi |
| Seyed | Hadi Kalantar |
| Amatallah | Hadi Shamsan |
| Danny | Hadidi |
| Thirza | Hadipranata |
| Alexis | Hadjiathanasiou |
| Andreas V | Hadjinicolaou |
| M | Hadjipavlou |
| Theodoros | Hadjizacharias |
| Claudia | Hadlow |
| L | Haenen |
| Rehana | Hafeez |
| Abdelrahman | Hafez |
| Ahmed | Hafez |
| Mahmoud | Hafez |
| Nour | Hafez |
| Youssef | Hafez |
| Verity | Haffenden |
| Surgeon | Hafiz Riaz Hussain Awan |
| Jonathan | Hagan |
| L | Hagander |
| I | Hagbevor |
| Stephanie | Hage |
| R | Hagger |
| Lea | Haiby |
| Arwa | Haidar |
| Hanan | Haidar |
| Mohammad | Haidari |
| E | Haiden |
| Fayza | Haider |
| Sajjad L | Haider |
| Ali | Haider Bangash |
| Humaira | Haider Mahin |
| Nisar | Haider Zaidi |
| D | Haidopoulos |
| Dimitrios | Haidopoulos |
| Assia | Haif |
| Manal | Haij |
| H | Haile |
| Mhreteab | Haile |
| Nebiyou | Hailu |
| Niguse | Hailu |
| S | Hailu |
| Samuel | Hailu |
| Alexander | Haim |
| Nadav | Haim |
| Maira | Haimona |
| A | Hainsworth |
| Mohd | Hairul Nizam Harun |
| Bashar | Haj Hassan |
| Luma | Haj Kassem |
| Huzifa | Haj-Ibrahim |
| M | Hajalamin |
| S | Hajdarević |
| Mohammed | Hajhamad |
| Farnaz | Haji |
| J | Hajiioannou |
| Jiannis | Hajiioannou |
| Samira | Hajisadeghi |
| R | Hajjouz |
| Tereza | Hajkova |
| M | Hajlan |
| Mana | Hajlan |
| Awsam | Hakami |
| Hadi | Hakami |
| I | Hakami |
| Ibrahim | Hakami |
| Riyadh | Hakami |
| H | Hakim |
| H | Hakmi |
| S | Halaseh |
| Sattam | Halaseh |
| Maxime | Halden |
| Yusuf | Halidu Bako |
| UA | Halim |
| Usman | Halim |
| C | Halkias |
| B | Hall |
| Benjamin | Hall |
| Claire | Hall |
| J | Hall |
| Nicola | Hall |
| J | Hallet |
| E | Halliday |
| Richard | Halliwell |
| Safa | Halman |
| M | Halpin |
| yasser | Halwani |
| B | Hama |
| Ahmed | Hamad |
| Hisham | Hamad |
| O | Hamad |
| MK | Hamada |
| Mohamed | Hamada |
| Mohammed | Hamada Takrouney |
| Shahed | Hamadieh |
| Z | Hamady |
| Hesham | Hamaly |
| Khaled M | Hamam |
| Omar | Hamam |
| Mohanad | Hamandi |
| H | Hamayel |
| M | Hambraeus |
| Ahmed | Hamdan |
| Alaa | Hamdan |
| Haya | Hamdan |
| Sara | Hamdoni |
| Emad | Hamdy |
| Mahmoud | Hamdy |
| Mohamed | Hamdy |
| Omar | Hamdy |
| R | Hamdy |
| Rana | Hamdy |
| Mohammed | Hamdy Al-Shazly |
| Emad | Hamdy Gad |
| Sarah | Hamdy Soliman |
| H | Hamed |
| Mona | Hamed |
| Abdelkader | Hamed Abdin |
| ALi | Hamed ALSharqi |
| Takwa | Hamed Ellakwa |
| Mohamed | Hamed Khalid |
| BZ | Hameed |
| Hiba | Hameed Chagla |
| S | Hamei |
| Peter | Hamer |
| Adwaa | Hamid |
| HK S | Hamid |
| HKS | Hamid |
| Khalid ZMY | Hamid |
| Mohammed | Hamid |
| Muhammad | Hamid Chaudhary |
| Amani | hamid Lamari |
| A | Hamidi |
| Barbara CS | Hamilton |
| E | Hamilton |
| Agnes | Hamilton-Baillie |
| Ali | Hammad |
| Essam | Hammad |
| Farah | Hammad |
| M | Hammad |
| Mirza | Hammad Rauf |
| A | Hammed |
| Ali | Hammed |
| Salah | Hammed |
| Jonathan | Hammerschlag |
| Jaap | Hamming |
| Bernard | Hammond |
| Eric | Hammond |
| J | Hammond |
| John | Hammond |
| JS | Hammond |
| Rob | Hammond |
| Dalia | Hammouche |
| S | Hammouche |
| Salah | Hammouche |
| Mohammad | Hammouri |
| Jacob | Hampton |
| M | Hampton |
| Matthew | Hampton |
| S | Hamrang-Yousefi |
| Alexandra | Hamshere |
| Ahmed | Hamss |
| Ammar | Hamza |
| Amr | Hamza |
| HM | Hamza |
| Muhammad | Hamza Sadiq |
| Basem | Hamzah |
| Ismail | Hamzaoglu |
| Sook | Han Yee |
| K | Hanazaki |
| Kazuhiro | Hanazaki |
| Angela | Hancock |
| S | Handa |
| Siddhartha | Handa |
| Chin | Hang Sophia Sin |
| Chi | Hang Yee |
| Christine | Hangaard Hansen |
| Umma | Hani Jaafaru |
| F | Hanif |
| Mohammad | Hanino |
| VM | Hanjoora |
| John | Hanke |
| Ciara | Hanley |
| Margaret | Hanley |
| A | Hanly |
| Ann | Hanly |
| H | Hanna |
| Joseph | Hanna |
| M | Hanna |
| N | Hanna |
| S | Hanna |
| Sam | Hanna |
| Wael | Hanna |
| A | Hannah |
| Ahmad | Hannan Amrullah |
| Jonathan | Hannay |
| M | Hannington |
| Rodina | Hanno |
| Abdullah | Hanoun |
| Tasvinder | Hans |
| L | Hansen |
| A | Hanson |
| M | Hanson |
| Melissa | Hanson |
| Islam | Hany Metwally |
| Zi | Hao Reuel Heng |
| S | Happ |
| Bushra | Haq |
| I | Haq |
| Rehan | Haq |
| Maha | Haqqani |
| MH | Haqqani |
| Izhar-Ul | Haque |
| Saeef | Haque |
| Cheyaanthan | Haran |
| Carina | Harasser |
| Timothy | Hardcastle |
| H | Hardgrave |
| C | Hardie |
| Claire | Hardie |
| J | Hardie |
| John | Hardie |
| Max | Hardie Boys |
| T | Harding |
| Thomas | Harding |
| Ruth | Hardstaff |
| J | Hardt |
| Sarah | Hardwick |
| A | Hardy |
| Alistair | Hardy |
| NP | Hardy |
| Rawan | Harfoush |
| Dipti | Haridas |
| Sandeep | Harigond |
| Solonirina | Harinarindra Ranaivoson |
| Muhammad | Haris Chishti |
| Muhammad | Haris Janjua |
| Nagenthiram | Harivallavan |
| A | Harky |
| Amer | Harky |
| Niels | Harlaar |
| NJ | Harlaar |
| Natasha | Harley |
| AT | Harmantepe |
| Laura | Harmon |
| Christopher | Harmston |
| Camila | Haro |
| L | Haro Supa |
| Aaya | Haron |
| M | Haroon |
| Waqqas | Haroon |
| Felix | Harpain |
| L | Harper |
| Luke | Harper |
| Aenone | Harper Machin |
| Gemma | Harrell |
| R | Harries |
| Rhiannon | Harries |
| G | Harris |
| Johnathon | Harris |
| Margaret | Harris |
| Muhammad | Harris Siddique |
| Annabelle | Harrison |
| Ben | Harrison |
| E | Harrison |
| EM | Harrison |
| Ewen M | Harrison |
| Haidee | Harrison |
| Siew-Ling | Harrison |
| K | Harrison-Phipps |
| C | Hart |
| V | Hart |
| Bettina | Härter |
| Chris | Hartley |
| Daniela | Hartmann |
| Jacinda | Harty |
| Jessica | Harvey |
| R | Harvitkar |
| R | Harwood |
| Rachel | Harwood |
| AK | Harzif |
| Ahmad | Hasan |
| Asif | Hasan |
| Batool | Hasan |
| E | Hasan |
| MT | Hasan |
| R | Hasan |
| Raashad | Hasan |
| Rama | Hasan |
| Hajar | Hasan Kheslat |
| Damir | Hasandić |
| Ariola | Hasani |
| R | Hasanov |
| DM | Hasanuzzaman |
| Hirotoshi | Hasegawa |
| A | Hasenburg |
| Annette | Hasenburg |
| Fuad | Hashem |
| Lina | Hashem |
| M | Hashem |
| Mohamed | Hashem |
| Yaldasadat | Hashemipour |
| Adil | Hashim |
| AT | Hashim |
| HT | Hashim |
| D | Hashimoto |
| Daisuke | Hashimoto |
| Ali | Hashmi |
| Shiraz | Hashmi |
| Ismail | Hasirci |
| David | Haslhofer |
| A | Hasnat |
| A | Hassan |
| Abdulhakim | Hassan |
| Ahmed | Hassan |
| F | Hassan |
| Gehad | Hassan |
| Ismail | Hassan |
| Karim | Hassan |
| M | Hassan |
| Mekki | Hassan |
| Muha | Hassan |
| Muhammad | Hassan |
| Murtuza | Hassan |
| N | Hassan |
| Nabeel | Hassan |
| R | Hassan |
| Ramy A | Hassan |
| Roa | Hassan |
| Sadik | Hassan |
| Sadiq | Hassan |
| Shamira | Hassan |
| Sulaiman | Hassan |
| Usman | Hassan |
| Yosef | Hassan |
| Arowa | hassan abdulrahman Alansari |
| Seyyed | Hassan Adeli |
| Abdelmonem | hassan eid Abdelmonem |
| Mohamed | Hassan Fathy Hassan Abdallah |
| Mohsin | Hassan Khan Roshan |
| Hiba | Hassan Rehmtallah Ahmed |
| A | Hassanin |
| Ahmed | Hassanin |
| Aliaa | Hassanin |
| Mohamed | Hassanin |
| Karen | Hassell |
| Chiraz | Hassoun |
| Maher | Hassounah |
| Anas | Hassouneh |
| Esraa | Hassouneh |
| Yoko | Hasumi |
| Andi | Hasyim |
| Jaber | Hatam |
| Mohamed | hatem elmetwalli eldwini Eldwini |
| Isobel | Hatrick |
| R | Hatz |
| Rudolf | Hatz |
| C | Hatzantonis |
| Victor | Hau |
| AF | Haugstvedt |
| J | Hauptman |
| H | Hauser |
| F | Hauswirth |
| T | Havenhand |
| Tom | Havenhand |
| N | Havers |
| L | Havranek |
| Hanadi | Hawa |
| Nouran | Hawa |
| I | Hawal |
| Islam | Hawal |
| M | Hawari |
| Mohammad | Hawari |
| A | Hawila |
| Ahmed | Hawila |
| Lydia | Hawker |
| P | Hawkin |
| Alexander | Hawkins |
| Robyn | Hawkins |
| J | Hawkyard |
| S | Hayajneh |
| M | Hayashi |
| Megumi | Hayashi |
| K | Hayat |
| Khizar | Hayat |
| Zara | Hayat |
| Wasim | Hayat Khan |
| F | Hayati |
| Firdaus | Hayati |
| Hussein | Hayati |
| C | Hayden |
| Dana | Hayden |
| Max | Hayden |
| Andrew | Hayes |
| Ross | Hayhurst |
| Tony | Haykal |
| Dickon | Hayne |
| AB | Haynes |
| Alex | Haynes |
| Amelia | Haynes |
| Abi | Hayward |
| Marko | Hazabent |
| I | Hazan |
| J | Hazelton |
| Mohamed | Hazem Okail |
| Khairul | Hazim Hamdan |
| Luis | Hdez Miguelena |
| Zhexi | He |
| E | Headon |
| Tristan | Heath |
| Chelsea L | Heaven |
| Daniel | Hechtl |
| Matthias | Heck |
| R | Heckburn |
| Andreas | Hecker |
| M | Hecker |
| Matthias | Hecker |
| Mohamed | Hedi Ghalloussi |
| Alex | Hedley |
| Megan | Hedlund |
| So | Hee Kim |
| Chan | Hee Koh |
| Anna | Heeney |
| B | Heer |
| Munish | Heer |
| Abbie | Heffernan |
| Elsayed | Hegazy |
| Ibrahim | Hegazy |
| Osama | Hegazy |
| S | Hegde |
| Siddhi | Hegde |
| E | Heidari |
| Farrokh | Heidari |
| Hamid | Heidari |
| Julia | Heider |
| F | Heike |
| Juuso | Heikkinen |
| LM | Heindl |
| Elmar | Heinrich |
| E | Heinz |
| Karl | Heinz Stadlbauer |
| J | Heisterkamp |
| Hala | Helal |
| Dulce | Helena Ferreira de Carvalho Carvalho |
| Sabrina | Helena Rossi |
| Pablo | Helguera |
| Dubravka | Heli Litvic |
| Michael | Helley |
| Philipp | Helmer |
| Hadeel | Helmi |
| O | Helminen |
| Olli | Helminen |
| Youssef | Helmy |
| Ahmad | Helmy Zayan |
| Tessely | Heloise |
| Hanan M | Hemead |
| Abdelrahman | Hemida |
| N | Hemmati |
| M | Hemmila |
| Mark | Hemmila |
| Nigel | Henderson |
| ER | Hendriks |
| H | Heneghan |
| Helen | Heneghan |
| Christin | Henein |
| Kerollos | Henes |
| Marilyn | Heng |
| EA | Hennessy |
| Elizabeth A | Hennessy |
| Sarah | Henning |
| Nicolas | Henric |
| Jose | Henrique Albuquerque Messias |
| Paulo | Henrique de Sousa Fernandes |
| Tiago | Henrique de Souza |
| Thiago | Henrique Sigoli Pereira |
| J | Henriques |
| P | Henriques |
| Pedro | Henriques |
| S | Henriques |
| Susana | Henriques |
| A | Henry |
| Alastair | Henry |
| Jaymie | Henry |
| David | Henshall |
| DE | Henshall |
| Patrick | Hensley |
| Mariel | Henzenn |
| Hwan | Heo |
| D | Herappe |
| Dorihela | Herappe |
| J | Heras Aznar |
| Matheesha | Herath |
| Beate | Herbig |
| Marit | Herbolzheimer |
| György | Herczeg |
| Fernando | Heredia |
| Carles | Heredia Llinàs |
| DM | Herghea |
| Tojomamy | Herinjaka Ralaizafindraibe |
| A | Heriot |
| Alexander | Heriot |
| T | Herklots |
| Koushik | Herle |
| H | Herman |
| Alba | Hernáez Arzoz |
| Jesus | Hernan Tovar |
| E | Hernandez |
| María | Hernandez |
| P | Hernandez |
| Roberto | Hernandez |
| Araceli | Hernández |
| I | Hernández |
| Inés | Hernández |
| R | Hernández |
| MA | Hernandez Bartolome |
| Guillermo | Hernandez Gauna |
| A | Hernandez Gutierrez |
| J | Hernandez Gutierrez |
| Alicia | Hernández Gutierrez |
| L | Hernández Miguelena |
| Araceli | Hernández Ramos |
| Estefania | Hernández-García |
| M | Hernández-García |
| Miguel | Hernández-García |
| S | Hernandez-Kakauridze |
| J Mindy | Hernández-Nava |
| JM | Hernández-Nava |
| Pedro | Hernando Calderon Quiroz |
| C | Heron |
| Charlotte | Heron |
| J | Héroux |
| Ana | Herranz Arriero |
| Danilo | Herrera |
| H | Herrera |
| Héctor | Herrera |
| Miguel | Herrera |
| Enrique | Herrera Castañeda |
| Norberto | Herrera Merino |
| DR | Herrera Mora |
| G | Herrera-Almario |
| Gabriel | Herrera-Almario |
| J | Herrera-Esquivel |
| A | Herrera-Gomez |
| N | Herrera-Merino |
| Julio | Herrera-Zamora |
| I | Herrero |
| Imanol | Herrero |
| Sofía | Herrero Gámiz |
| M | Herrero-Lopez |
| Jose | Herreros |
| Rubén | Herreros Ruiz-Valdepeñas |
| Barbara | Herritsch |
| F | Herrle |
| Johannes | Herrmann |
| P | Herrod |
| Caroline | Herron |
| Jonathan | Herron |
| Yehuda | Hershkovitz |
| Viktoria | Herterich |
| L | Herve |
| Luc | Hervé Samison |
| E | Hervieux |
| Erik | Hervieux |
| J | Herzberg |
| Jonas | Herzberg |
| Torsten | Herzog |
| Helal F | Hetta |
| HF | Hetta |
| Thusitha | Hettiarachchi |
| R | Hettige |
| A | Heuer |
| Annika | Heuer |
| Mateo | Hevia |
| Pelayo | Hevia Rodríguez |
| Nicole | Hew |
| Matt | Hewitt |
| B | Heyd |
| Z | Heydari |
| Marie | Heyne-Pietschmann |
| Emily | Heywood |
| ML | Hibbard |
| M | Hichem |
| L | Hidalgo Lariz |
| C | Hidalgo Salinas |
| Camila | Hidalgo Salinas |
| J | Hidayat |
| Lydia | Hiddema |
| Hishikawa | Hidehiko |
| L | Hidi |
| László | Hidi |
| Masaharu | Higashida |
| George | Higginbotham |
| Andrew | Higgins |
| M | Higgins |
| Mark | Higgins |
| S | Higgs |
| D | Highton |
| Felipe | Higuera |
| Eva | Higuera Miguélez |
| Amanda | Hii |
| Raid | Hijazeen |
| Zaid | Hijazi |
| N | Hijazin |
| Takeshi | Hijikawa |
| Ahmed | Hijjawi |
| N | Hilal |
| Fabián | Hilario Mendoza Pedraza |
| A | Hill |
| Arnold | Hill |
| C | Hill |
| CE | Hill |
| Charles | Hill |
| Ciaran | Hill |
| G | Hill |
| MJ | Hill |
| Rhodri | Hill |
| S | Hill |
| A | Hilley |
| Roxane | Hillier |
| Ahmed | Hilmi |
| Thomas | Hilton |
| Alexander | Himstead |
| Marisol | Hinaoui |
| J | Hind |
| S | Hind |
| T | Hine |
| C | Hing |
| Caroline | Hing |
| JX | Hing |
| Janmejay | Hingu |
| Adam | Hingum |
| Haruaki | Hino |
| CA | Hinojosa |
| Carlos | Hinojosa |
| A | Hinton |
| Naoki | Hirai |
| Teruyuki | Hiraki |
| Chetan | Hirani |
| Kouichi | Hirano |
| Naoyuki | Hirata |
| C | Hirche |
| Christoph | Hirche |
| S | Hirji |
| SA | Hirji |
| Sameer | Hirji |
| Reina | Hirooka |
| Jinso | Hirota |
| Kazuyoshi | Hirota |
| F | Hirri |
| H | Hirsch |
| Jakob | Hirsch |
| Scott | Hirsch |
| Markus | Hirschburger |
| Mohammed | Hirsi |
| Omar | Hirsi |
| D | Hiršl |
| Yoji | Hisamatsu |
| I | Hisham |
| Intisar | Hisham Said Hamdun Korea |
| L | Hitchman |
| Daniel | HL Lemmers |
| Ahmad | Hmaideh |
| Majedah | Hmeidan |
| Wut | Hmone |
| B | Ho |
| M | Ho |
| MF | Ho |
| Michael | Ho |
| Cheuk | Ho Lam |
| Yick | Ho Lam |
| Yuk | Ho Liu |
| A | Hoang |
| Katie | Hoban |
| Maria | Hobrok |
| Andres | Hodali |
| Katherine | Hodge |
| Victoria | Hodgetts Morton |
| H | Hodgson |
| Harry | Hodgson |
| R | Hodgson |
| Russell | Hodgson |
| Ghazal | Hodhody |
| Min | Hoe Chew |
| Daniel | Hofer |
| Markus | Hofer |
| Mary | Hoffman |
| Sebastian | Hoffmann |
| Thomas K | Hoffmann |
| Gwen | Hofman |
| Aisling | Hogan |
| AM | Hogan |
| D | Hogan |
| J | Hogan |
| John | Hogan |
| Kathryn | Hogan |
| A | Hogea |
| M | Hogea |
| Mircea | Hogea |
| R | Hogenbirk |
| Rianne | Hogenbirk |
| RND | Hogenbirk |
| Anders | Hogh |
| SP | Hogston |
| P | Höhn |
| Philipp | Höhn |
| Hannes | Hoi |
| Chi | Hoi Lee |
| RM | Højsgaard |
| Christie | Hok Yung Shum |
| Cole | Holan |
| S | Holawe |
| Simone | Holawe |
| C | Holbrook |
| Charlotte | Holbrook |
| F | Holc |
| Fernando | Holc |
| Daniel | Holena |
| Johannes | Holfeld |
| Abin | Holla |
| E | Holler |
| Paul | Hollington |
| Alexander | Hollis |
| M | Hollyman |
| Marianne | Hollyman |
| T | Holme |
| Thomas | Holme |
| A | Holmes |
| Angela | Holmes |
| Merran | Holmes |
| Samuel | Holmes |
| F | Holmner |
| D | Holroyd |
| David | Holroyd |
| M | Holscher |
| Christopher | Holt |
| Phillip | Holt |
| Katharina | Hölz |
| F | Hölzle |
| Frank | Hölzle |
| Clemens | Holzmeister |
| Seyedeh | Homa Hemmasi |
| R | Hompes |
| Sophie | Hon |
| H | Honarpisheh |
| Human | Honarpisheh |
| Bridget | Hone |
| D | Hong |
| Dennis | Hong |
| T | Hong |
| Jen | Hong Ong |
| Bee | Hong Soon |
| Iain | Hood |
| Kheng | Hooi Chan |
| Mee | Hoong See |
| N | Hope |
| Brent | Hopkins |
| J | Hopkins |
| M | Hoque |
| Raymund E | Horch |
| Vladyslav | Hordoskyi |
| N | Horesh |
| A | Horiguchi |
| Karoline | Horisberger |
| AP | Hormis |
| Julian | Horn |
| Cynthia | Horner |
| Jonathan | Horsnell |
| Rahim | Horuz |
| S | Horvath |
| Maher | Hosain |
| Tatsuki | Hoshino |
| S | Hosny |
| F | Hossain |
| Fahad | Hossain |
| Kamral | Hossain |
| N | Hossain |
| Ruhella | Hossain |
| T | Hossain |
| Ahmed | Hossam |
| Ahmed | Hossam Eldin Fouad Rida |
| Mahmoud | Hossameldin Saad Abdelhamid |
| Mohammad | Hossein Khosravi |
| Mohammad | Hossein Nabian |
| Seyyed | Hossein Shafiei |
| Elahe | Hosseini |
| MR | Hosseini Siyanaki |
| Seyedmohamad | Hosseini Zavareh Hosseini Zavareh |
| Masoumeh | Hosseinpoor |
| M | Hosseinzadeh Maleki |
| Zuzana | Hotová |
| Lachlan | Hou |
| Zhen | Hou |
| Yara | Houdifa |
| C | Houlden |
| A | Houmada |
| Corey | Hounschell |
| H | Houshyar |
| Zakaria | Houssaïn Belkhadir |
| A | Houssem |
| Ammar | Houssem |
| Helen | Houston |
| R | Houston |
| CHC | Houtsma |
| T | Houwen |
| E | How Hong |
| T | Howard |
| D | Howden |
| Michael | Howells |
| Sean | Howells |
| Emma | Howie |
| L | Howse |
| Dileep | Hoysal |
| Tarteel | Hrerat |
| Evguenia | Hristova |
| Kalina | Hristova |
| Marvin | Hsiao |
| Michelle | Hsiao |
| V | Hsiao |
| Yu | Hsuen Yang |
| A | Ht rao |
| B | Hu |
| Jiankun | Hu |
| Sophie | Hu |
| Yu-Ning | Hu |
| L | Hua-Feng |
| Lien | Hua-Feng |
| Eduardo | Huaman |
| E | Huamán |
| E | Huamán Egoávil |
| Huilun | Huan |
| Abel | Huang |
| Lana | Huang |
| Linna | Huang |
| Wai | Huang Teng |
| Thomas | Hubbard |
| J | Huber |
| Verena | Huber |
| Kristin | Huber-Strößner |
| Lauren | Huckaby |
| F | Huda |
| Farhanul | Huda |
| Shahab | Huda |
| Igor | Hudic |
| VE | Hudson |
| Victoria | Hudson |
| Mónica | Huecas |
| M | Huecas-Martinez |
| MA | Huertas Fernandez |
| Benjamin | Huggon |
| Thomas | Hugh Lynch |
| Andrew | Hughes |
| Anne | Hughes |
| Dominique | Hughes |
| F | Hughes |
| I | Hughes |
| Isabel | Hughes |
| JL | Hughes |
| Víctor | Hugo Alcalá Torres |
| H | Huhta |
| Heikki | Huhta |
| Sze | Hui Wong |
| LF | Huilca Logroño |
| L | Huisman |
| R | Hultgren |
| Rebecka | Hultgren |
| Waseem | Humayoun |
| Luis | Humberto Govea-Camacho |
| D | Humes |
| David | Humes |
| Bailey | Humphreys |
| L | Humphreys |
| S | Humphries |
| Ellie | Humphry |
| Majd | Hunaiti |
| WeiPin | Hung |
| Adam | Hunt |
| Benjamin | Hunt |
| I | Hunt |
| J | Hunt |
| Janette | Hunt |
| Louise | Hunt |
| B | Huntly |
| Lucy | Huppler |
| F | Hurasha |
| Heather | Hurdle |
| Conor | Hurson |
| Najam | Husain |
| S | Husain |
| Shatha | Husain |
| Amy | Huseyin |
| Mohammed | Husien Yosif Elhafiz |
| Azar | Hussain |
| Fathi | Hussain |
| Mohammed | Hussain |
| Musarrat | Hussain |
| Zahra | Hussain |
| Zainab | Hussain |
| Shabbar | Hussain Changazi |
| Zahid | Hussain Khan |
| Musheer | Hussain Mohamed |
| Riaz | Hussain Siddiqui |
| Shahzad | Hussain Waqar |
| Dr | Hussaini |
| Ahmed | Hussein |
| Bili | Hussein |
| H | Hussein |
| Hamzeh | Hussein |
| Hasan | Hussein |
| KM A | Hussein |
| L | Hussein |
| R | Hussein |
| Rand | Hussein |
| Haithem | Hussein Ali |
| Hamza | Hussein Aly Salama Aly |
| Maab | Hussein Yousif Elhafiz |
| Husnia | Hussen |
| Romeo | Hussey |
| P | Hutchinson |
| Peter | Hutchinson |
| PJ | Hutchinson |
| R | Hutchison |
| Jörg | Hutter |
| Mohammed | Huwaysh |
| K | Huynh |
| Victoria | Huynh |
| Louise | Hviid |
| Suk | Hwan Lee |
| ES | Hwang |
| Joe | Hwong Pang |
| Ayah | Hyasat |
| Alexander | Hyhlik-Duerr |
| GY | Hyman |
| Boel | Hynning |
| Arianne | I Lupián-Angulo |
| Alessandro | Iacomino |
| Despoina | Iakovou |
| I | Iannone |
| Christopher | IAnson |
| F | Iazzetta |
| Yutaka | Iba |
| Marta | Ibáñez Nieto |
| FJ | Ibáñez-Aguirre |
| T | Ibekwe |
| I | Ibi |
| Treasure | Ibingira |
| Betul | Ibis |
| Iftekhar | Ibne Mannan |
| Gbadebo | Ibraheem |
| M | Ibraheem |
| Maher | Ibraheem |
| Omar | Ibrahem |
| A | Ibrahim |
| Abdelrahman | Ibrahim |
| Adem | Ibrahim |
| Ahmed | Ibrahim |
| Firas | Ibrahim |
| Hamza | Ibrahim |
| Isakwa | Ibrahim |
| Islam H | Ibrahim |
| M | Ibrahim |
| Mohamed | Ibrahim |
| Mohammad | Ibrahim |
| Mohsen | Ibrahim |
| Mostafa | Ibrahim |
| Nourhan | Ibrahim |
| S | Ibrahim |
| Sadiq | Ibrahim |
| Saidu | Ibrahim |
| Shaimaa | Ibrahim |
| Sufyan | Ibrahim |
| Tarek | Ibrahim |
| Z | Ibrahim |
| Zainab | Ibrahim |
| Omar | Ibrahim Elsayed |
| Mohamed | Ibrahim Gbreel |
| Mohammed | Ibrahim Mohammed Ali |
| Samah | Ibrahim Omer Mohamed Osman Mohamed Osman |
| Mustapha | Ibrahim Usman |
| A | Ibrahimli |
| GC | Icaza de Marín |
| M | Ida |
| Mitsuru | Ida |
| Denisse | Idalia Campos Mejía |
| Damaris | Idara Anabel Zezular |
| Yabasin | Iddrisu Baba |
| Ehanga | Idi Marcel |
| Louis | Idier |
| UO | Idiz |
| Jeuel | Idowu |
| Olufemi | Idowu |
| Muhammad | Idrees Anwar |
| M | Idrissi |
| Michele | Iester |
| G | Ietto |
| Giuseppe | Ietto |
| N | Iflazoglu |
| Fizza | Iftikhar |
| Muhammad | Iftikhar |
| Zainab | Iftikhar |
| Ahmar | Iftikhar Talib |
| Kueni | Igbagiri |
| Oluwasuyi | Ige |
| Eva | Iglesias Garcia |
| Jose | Ignacio Blanes |
| Carlos | Ignacio Ferrero |
| José | Ignacio Gerchunoff |
| Matias | Ignacio Gonzalez |
| Jose | Ignacio González Martín |
| Ricardo | Ignacio Olmedo Bareiro |
| José | Ignacio Sánchez Méndez |
| Juan | Ignacio Stenner |
| Mihaela | Ignat |
| Batog | Igor |
| Daniel | Igor |
| P | Ihnát |
| Grace | Ihsiu Todd |
| Katsuyuki | Iida |
| Koji | Iida |
| Patricio | III Dumlao |
| Cesar | III Jacinto |
| Yusuke | Iizuka |
| Munirdeen | Ijaiya |
| Adebimpe | Ijarotimi |
| Attiya | Ijaz |
| Ferdinand | Ijekeye |
| TR | Ijichi |
| Shingo | Ikeda |
| Tatsuhiko | Ikeda |
| Hilary | Ikele |
| Adeel | Ikram |
| H | Ikram |
| S | Ikram |
| Syed | Ikramullah Ikramullah |
| Ijezie | Ikwuezunma |
| Haifaa | Il hadad |
| N | Ilahi |
| R | Ilic |
| Rosanda | Ilic |
| Drochioi | Ilie Cristian |
| M | Iliescu |
| Madalina | Iliescu |
| Ivelina | Ilieva |
| Abdullah | Ilktac |
| Matthias | Ilmer |
| D | Ilukpitiya |
| Hiroshi | Imai |
| SM B | Imam |
| Alphonsine | Imanishimwe |
| Abbassi | Imed |
| H | Impellizzeri |
| Harmony | Impellizzeri |
| O | Impey |
| F | Imran |
| Farrah-Hani | Imran |
| Jonathan | Imran |
| Rizwana | Imran |
| Muhammad | Imran Anwar |
| Syed | Imran Bukhari |
| M | Imran Khan |
| Muhammad | Imran Khokhar |
| Joe | Imumoren |
| Hina | Inam |
| M | Inama |
| Marco | Inama |
| Ilker | Ince |
| P | Incollingo |
| Paola | Incollingo |
| Joseph | Incorvia |
| Mayang | Indah Lestari |
| F | Indrarti |
| Ivana | Ines Pedraza Salazar |
| Vera | Inês Ribeiro |
| César | Infante |
| A | Ingabire |
| Carlo | Ingaldi |
| Nikhil | Ingle |
| Laura | Inglis |
| Zorka | Inic |
| César | Íñiguez Martínez |
| Maria | Inmaculada Ruiz Montesinos |
| María | Inmaculada Valldeperas Hernández |
| Yasushi | Innami |
| Junichi | Inokuchi |
| Hiroyuki | Inoue |
| Yui | Inoue |
| Osvaldo | Insfran |
| Sylvie | Inyange |
| M | Inzunza |
| Maria | Ioanna Antonopoulou |
| Evangelia | Ioanna Tsiourva |
| A | Ioannidis |
| Argyrios | Ioannidis |
| O | Ioannidis |
| Orestis | Ioannidis |
| Oreste | Iocca |
| Cojocaru | Ion |
| Serban | Ion Bubenek Turconi |
| NS | Ionescu |
| Sebastian | Ionescu |
| Florin | Iordache |
| Eirini | Iordanidou |
| V | Iori |
| Valentina | Iori |
| RV | Iosifescu |
| Olga | Ioulia Semkoglou |
| Claudio | Iovino |
| D | Iovino |
| Domenico | Iovino |
| Christopher | Ip |
| J | Ip |
| M | Ip |
| Ayberk | İplikçi |
| S | Ippoliti |
| Simona | Ippoliti |
| P | Ipponi |
| Atif | Iqbal |
| Ayesha | Iqbal |
| Faizan | Iqbal |
| Javaid | Iqbal |
| Mohammad | Iqbal |
| Ramiz | Iqbal |
| Zafar | Iqbal |
| Omer | Iqbal Cheema |
| Magnifique | Irakoze |
| Shirin | Irani |
| Iran | Irani Durán Sánchez |
| Maria | Iraola |
| MJ | Irarrázaval |
| Patrick | Ireland |
| Beyza | Irem Yabaci |
| Rebecca | Ireson |
| Oseihie | Iribhogbe |
| Tomoya | Irie |
| Daisuke | Irimada |
| A | Irimie |
| Alexandru | Irimie |
| Gunko | Irina |
| Paola | Irina Eusebio Jimenez |
| Tomoko | Irisawa |
| Omorodion | Irowa |
| Abeer | Irshad |
| E | Irune |
| Ekpemi | Irune |
| E | Irvine |
| V | Irvine |
| Isabirye | Isa |
| Otolia | Isaac |
| Olusegun | Isaac Alatise |
| John | Isaac Merin |
| Anna | Isaacs |
| R | Isaacs Beron |
| Joana | Isabel Almeida |
| Ana | Isabel Avellaneda Camarena |
| Maria | Isabel Manso |
| Maria | Isabel Prieto-Nieto |
| Inês | Isabel Sampaio da Nóvoa Gomes Miguel |
| Frigerio | Isabella |
| Tetsuro | Isada |
| Aliyu | Isah |
| Andres | Isaza-Restrepo |
| Gloria | Isela Mendoza Frías |
| R | Isernia |
| Muhammad | Isfandyar Khan Malik |
| P | Ishak |
| Amna | Ishaq |
| Nazia | Ishaque |
| Katsuhiko | Ishibashi |
| S | Ishida |
| Sachi | Ishida |
| Yusuke | Ishida |
| H | Ishii |
| Haruka | Ishikawa |
| M | Ishikawa |
| Masashi | Ishikawa |
| Makoto | Ishitobi |
| Taku | Ishizaki |
| Daniyal | Ishtiaq |
| Christian | Isichei |
| Mercy | Isichei |
| Filipe | Isidro |
| A | Isik |
| Ozgen | Isik |
| Monica | Iskander |
| Othman | Iskander |
| D | Isla-Ortiz |
| David | Isla-Ortiz |
| AA | Islam |
| N | Islam |
| Rahela | Islam |
| S | Islam |
| Shahnoor | Islam |
| SM Nazmul | Islam |
| Sumayya | Islam |
| C | Ismael |
| Salam | Ismael |
| Fahad | Ismail |
| Hlma | Ismail |
| L | Ismail |
| Lamiese | Ismail |
| M | Ismail |
| N | Ismail |
| Nasiru | Ismail |
| O | Ismail |
| Samir | Ismail |
| Zainab | Ismail |
| Jameel | Ismail Ahmad |
| Mohammad | Ismail Attar |
| Hafsa | Ismail Ibrahim Naiya |
| Taha | ismail Sefrioui |
| G | Ismaili |
| Ghiath | Ismayl |
| SM | Isolani |
| Fadi | Issa |
| Michael | Issa |
| Mohannned | Issa |
| N | Issa |
| Adamu | Issaka |
| R | Itani |
| Rania | Itani |
| Shingo | Ito |
| Oda | Ituze |
| Stoian | Iudin |
| Muresan | Iulia Andrada |
| Kevin | Ivan P Chan |
| Giorgio | Ivan Russo |
| N | Ivancevic |
| T | Ivanov |
| Tsvetomir | Ivanov |
| Anna | Ivanova |
| Nenad | Ivanović |
| Igors | Ivanovs |
| Andjela | Ivezić |
| Ebikela | Ivie Baidoo |
| Paolo | Ivo Cavoretto |
| Hideki | Iwahashi |
| M | Iwasaki |
| Masae | Iwasaki |
| Shintaro | Iwata |
| Ifeanyi | Iwuagwu |
| K | Iyengar |
| Karthikeyan | Iyengar |
| Priyanka | Iyer |
| Vikram | Iyer |
| David | Izadi |
| A | Izaguirre |
| Aldo | Izaguirre |
| J | Izbicki |
| Jakob | Izbicki |
| Ana | Izquierdo |
| O | Izquierdo |
| S | Izwan |
| F | Izzo |
| Francesco | Izzo |
| E | Jabagat |
| Abd | Jabar Nazimi |
| Nicolette | Jabbour |
| Ahmad | Jaber |
| Kefah | Jaber |
| Abdulla | Jabr |
| Massa | Jabra |
| R | Jach |
| J | Jackman |
| Jamaall | Jackman |
| A | Jackowski |
| Claire | Jackson |
| H | Jackson |
| K | Jackson |
| Karl | Jackson |
| Richard | Jackson |
| A | Jacob |
| S | Jacob |
| Arun | Jacob Philip George |
| Adeline | Jacobs |
| Daniel | Jacobs-Tulleneers-Thevissen |
| F | Jácome |
| Filipa | Jácome |
| Jean | Jacques Tuech |
| Piyush | Jadhao |
| J | Jaekers |
| Jay | Jaemin Park |
| Ahmad | Jafar |
| Mehraneh | Jafari |
| Alisha | Jaffer |
| N | Jagadeesh |
| Tarkan | Jäger |
| Tomaz | Jagric |
| Asif | Jah |
| Abdussalam | Jahan |
| Alhadi | Jahan |
| Shahrokh | Jahan Bini |
| M | Jahnen |
| A | Jain |
| Amit | Jain |
| Anshini | Jain |
| Anuj | Jain |
| Deepak | Jain |
| Divakar | Jain |
| Kavitha | Jain |
| Manoj | Jain |
| P | Jain |
| Prateek | Jain |
| R | Jain |
| Ritu | Jain |
| Sunjay | Jain |
| Vaibhav | Jain |
| Anthony | Jaipersad |
| Somil | Jaiswal |
| Molly | Jakeman |
| Patricia | Jako |
| James | Jakub |
| Abdul | Jalil |
| S | Jalili |
| S | Jallad |
| Samer | Jallad |
| Heba | Jaloun |
| A | Jamal |
| Abid | Jamal |
| Aiman | Jamal |
| M | Jamal |
| Mohammad | Jamal |
| Faris | Jamal Abu Zanouneh |
| Mohamed | Jamal Elshref |
| Sarfraz | Jamali |
| Saja | Jamaliah |
| Suniza | Jamaris |
| PS | Jambulingam |
| W | Jamel |
| Rorisang | Jamela |
| A | James |
| D | James |
| Deeptiman | James |
| G | James |
| Sophie | James |
| Tobias | James |
| Tracy | James |
| Ayotunde | James Fasunla |
| Matthew | James McGuinness |
| Ryan | James Ocsan |
| Ifeanyi | James Orji |
| Salem | Jamhour |
| NB | Jamieson |
| Nigel | Jamieson |
| Chloe | Jamieson-Grigg |
| Manahil | Jamil |
| T | Jamil |
| Tahir | Jamil |
| AA B | Jamjoom |
| G | Jamjoum |
| Mohamed | Jammal |
| Kashif | Jan |
| Yousaf | Jan |
| Gregor | Jan Kocher |
| Josif | Janchulev |
| Aeris | Jane D Nacion |
| Jingya | Jane Pu |
| Seyoung | Jang |
| Akash | Jangan |
| A | Jangjoo |
| DP | Jani |
| A | Janjua |
| Atif A | Janjua |
| Azwa | Janjua |
| MH | Janjua |
| J | Jankau |
| Wolfgang | Janni |
| Shirley | Jansen |
| Yanina | Jansen |
| J | Janson |
| M | Janssen |
| Y | Janssen |
| Ward | Janssens |
| Gediminas | Januška |
| P | Januszyk |
| K | Japheth |
| Alejandra | Jara Maquilón |
| Enas | Jaradat |
| Gustavo | Jardim Volpe |
| Ruari | Jardine |
| Peter | Jarin |
| UM | Jariod-Ferrer |
| Natalie | Jarkas |
| Abdulaziz | Jarman |
| Stefanie | Jarmusch |
| C | Jarry |
| M | Jarvis |
| Kristijonas | Jasaitis |
| D | Jasarovic |
| Elmer | Jason Cruz |
| Miren | Jasone Diez Zapirain |
| Y | Jauhari |
| SM | Jaume Böttcher |
| S | Jaunoo |
| SM | Javad Mortazavi |
| M | Javadpour |
| Mohsen | Javadpour |
| Haroon | Javaid Majid |
| Hannah | Javanmard-Emamghissi |
| Anum | Javed |
| Ayesha | Javed |
| Aymen | Javed |
| Dania | Javed |
| Hina | Javed |
| S | Javed |
| Saad | Javed |
| Sundas | Javed |
| Umer | Javed Chughtai |
| Muhammad | Javed Iqbal |
| Manal | Javid |
| P | Javid |
| Domingo | Javier Aguilera Maidana |
| Francisco | Javier Bonilla-Escobar |
| Euler | Javier Burbano Luna |
| Francisco | Javier Fernández Pablos |
| Víctor | Javier García Porcel |
| Carlos | Javier Gómez Díaz |
| Antonio | Javier Gomez Poveda |
| Ernesto | Javier Guerrero Casillas |
| Francisco | Javier Ibáñez-Aguirre |
| Francisco | Javier León Frutos |
| Francisco | Javier Llamas- Macias |
| Francisco | Javier Ortiz de Solórzano Aurusa |
| Francisco | Javier Redondo Calvo |
| Veronika | Javurkova |
| M | Jawad |
| Monir | Jawad |
| Muhammad | Jawad |
| Z | Jawad |
| Muhammad | Jawad Zafar |
| Yashpal | Jaware |
| Haya | Jawish |
| Natalia | Jaworska |
| J | Jaya |
| Abhilash | Jayakumar |
| U | Jayarajah |
| Umesh | Jayarajah |
| Harish | Jayaram |
| RB | Jayaram |
| B | Jayasankar |
| Balaji | Jayasankar |
| S | Jayasekara |
| JD | Jayasinghe |
| Ravindri | Jayasinghe |
| Sumudu | Jayasinghe |
| DMCS | Jayasundara |
| A | Jayawardane |
| Asanka | Jayawardane |
| Tanmay | Jaysingani |
| Basel | Jazieh |
| Haragirimana | Jean de Dieu |
| Clement | Jeandel |
| J | Jeater |
| William | Jebril |
| Julia | Jedanowski |
| Nicole | Jedrzejko |
| Ana | Jeelani |
| D | Jeevan |
| David | Jeevan |
| Niall | Jefferson |
| Nikola | Jeftic |
| R | Jeganathan |
| Reubendra | Jeganathan |
| Shah | Jehan |
| Muhammad | Jehangir Malik |
| Emran | Jeitan |
| Clara | Jeketera |
| Ivan | Jelčić |
| Jelenko | Jelenkovic |
| C | Jelley |
| D | Jelovac |
| Drago | Jelovac |
| D | Jenkins |
| Victoria | Jenkins |
| M | Jenkinson |
| MD | Jenkinson |
| Michael | Jenkinson |
| Michael D | Jenkinson |
| E | Jenner |
| Seamus | Jennings |
| H | Jenny |
| Eric | Jensen |
| C | Jenvey |
| SMH | Jeoffrey |
| H | Jeong |
| K | Jeremic Stefanovic |
| S | Jeri-McFarlane |
| Webster | Jerry Noronha |
| Ora | Jesner |
| Hans | Jesper Del Mundo |
| Jose | Jesus Herrera |
| Antonia | Jesús López López |
| Rey | Jesus Romero |
| Joel | Jesús Sánchez Estupiñan |
| Marko | Jevric |
| Anupama | Jeyakumar |
| Nivedan | Jeyamanoharan |
| Rathan | Jeyapalan |
| D | Jeyaretna |
| Deva | Jeyaretna |
| C | Jezieniecki |
| Carlos | Jezieniecki |
| Deepak | Jha |
| Mark | Jheric Tesil |
| Eu | Jhin Loh |
| Fangzhi | Jia |
| Wei | Jia |
| Shawn | Jia Hwang Tan |
| N | Jiagge |
| Nuna | Jiagge |
| E | Jianu |
| L | Jiao |
| M | Jibreel |
| T | Jichi |
| Tarik | Jichi |
| Wen | Jie Chin |
| Cristian | Jimenez |
| G | Jimenez |
| Raul | Jimenez |
| V | Jimenez |
| Virginia | Jimenez |
| Laura | Jiménez |
| V | Jiménez Carneros |
| E | Jimenez Higuera |
| M | Jiménez Jiménez |
| J | Jimenez Miramón |
| Javier | Jimenez Miramón |
| LJ | Jimenez Ramirez |
| Carmen | Jimenez Sanchez |
| M | Jimenez Toscano |
| Marta | Jimenez Toscano |
| X | Jimenez Villanueva |
| Carlos | Jiménez Viñas |
| Marta | Jiménez-Jiménez |
| L | Jimenez-Roldan |
| Luis | Jimenez-Roldan |
| J | Jimeno Fraile |
| Jaime | Jimeno Fraile |
| Nam | Jin Kim |
| Hiang | Jin Tan |
| Jang | Jin-Young |
| Daniel | Jira |
| Moa | Jira |
| Ghassan | Jisry |
| Jin | Jiun Mah |
| Haithem | Jlassi |
| Manal | Jmaileh |
| Luis | Joaquín García Flórez |
| Fahmi | Jobran |
| D | Jochems |
| Carolin | Jödicke |
| Derlis | Joel Ojeda Villasboa |
| HK M | Joeng |
| Shivangi | Jog |
| S | Johan |
| Syamim | Johan |
| Anika | Johanna Agoncillo |
| Marco | Johannes Battista |
| Celestine | John |
| J | John |
| Gareth | John Bowen |
| Christopher | John Macapugay |
| C | Johnson |
| D | Johnson |
| David | Johnson |
| O | Johnson |
| Brian | Johnston |
| Sean | Johnston |
| Craig | Johnstone |
| J | Johnstone |
| Vladimir | Jokic |
| Miloš | Joković |
| Joshua | Jolissaint |
| JS | Jolissaint |
| Danielle | Jolly |
| Sami | Jomaa |
| Danny | Jon Nian Wong |
| E | Jonas |
| Boakye-Yiadom | Jonathan |
| A | Jones |
| C | Jones |
| D | Jones |
| Elizabeth | Jones |
| G | Jones |
| Gbenga | Jones |
| GP | Jones |
| J | Jones |
| L | Jones |
| M | Jones |
| Mark | Jones |
| R | Jones |
| Robin | Jones |
| Rosalind | Jones |
| RP | Jones |
| Terence | Jones |
| TR | Jones |
| Larne | Jones-Whiting |
| FH W | Jonker |
| Frederik | Jonker |
| Pascal | Jonker |
| PK C | Jonker |
| PKC | Jonker |
| C | Jonsson |
| Carina | Jonsson |
| ML | Jönsson |
| R | Jorba |
| Misericòrdia | Jordà Solé |
| Caitlin | Jordan |
| S | Jordan |
| Shannon | Jordan |
| Stevan | Jordan |
| Henrique | Jorge Guedes Neto |
| Eduardo | Jorge Premoli |
| Lars N | Jorgensen |
| T | Jorgensen |
| Thomas W | Jorgensen |
| TW | Jorgensen |
| M | Jornet-Gibert |
| Montsant | Jornet-Gibert |
| Noriega | José |
| Diego | José Almada Casañas |
| Antonio | Jose Alonso Villalba |
| Francisco | José Barbosa Camacho |
| Rafael | Jose Beltran |
| Diego | Jose Caycedo Garcia |
| João | José Corrêa Bergamasco |
| Rodolfo | Jose Favaretto Filho |
| F | José Fernandez Coimbra |
| Felipe | José Fernandez Coimbra |
| Pedro | José Gil Vázquez |
| Maria | Jose Gomez-Jurado |
| Maria | Jose González-Gimeno |
| Juan | Jose Jaramillo Roncancio |
| Christoph | José Klein Zampaña |
| Marcelo | José Maia Azevedo Costa |
| María | José Martínez |
| Maria | Jose Martinez Velázquez |
| Antonio | José Montoya Casella |
| Albaro | José Nieto Calvache |
| Carlos | Jose Perez Rivera |
| Maria | José Reche Padilla |
| Enrique | Jose Ruiz Velasquez |
| María | José Sangüesa |
| Juan | José Segura-Sampedro |
| Carlos | Jose Zuloaga Fernandez del Valle |
| María | Josefa Cuevas López |
| Johanna | Josefine Strotmann |
| Lallu | Joseph |
| Lule | Joseph |
| Reece | Joseph |
| Sinu | Joseph |
| Treasa | Joseph |
| Yorke | Joseph |
| A | Joshi |
| Anuja | Joshi |
| Ashoo | Joshi |
| D | Joshi |
| Mohit | Joshi |
| P | Joshi |
| Prabesh | Joshi |
| S | Joshi |
| Vaishali | Joshi |
| Vinay | Joshi |
| Y | Joshi |
| Zaman | Joshua |
| Shosaburo | Jotaki |
| Harihara | Jothi |
| A | Jotic |
| Ana | Jotic |
| Lionel | Jouffret |
| Patrick | Jovan Gagno |
| T | Jovanoski |
| Tomislav | Jovanoski |
| K | Jovanovska |
| Katerina | Jovanovska |
| L | Jovcheski |
| JM | Jover |
| E | Jovine |
| Elio | Jovine |
| DP | Joyce |
| Yan | Joyce Ming |
| Karen | Joyce Velasco |
| Kathir | Joyson |
| Jesus | Jr Dabalos |
| Eunmaro | Ju |
| Jose | Juan Gonzalez Sanchez |
| Rodrigo | Juaneda |
| Moises | Juarez |
| M | Juarez-Pomes |
| Yolanda | Jubete Castañeda |
| N | Judkins |
| Nicholas | Judkins |
| Mary | Jue Xu |
| R | Jugdey |
| C | Juillard |
| Catherine | Juillard |
| Matthew | Jukes |
| M | Jukić |
| David | Julià Bergkvist |
| Maria | Julia Corbetta Machado |
| E | Julià-Verdaguer |
| Maria | Juliana Sanchez |
| Andrea | Juliana Vega Calvera |
| Benji | Julien |
| Yuki | Julius Ng We Yong |
| Aaron | Julius Punnen |
| J | Juloski |
| Jovan | Juloski |
| Isam | Juma |
| Irfan | Jumabhoy |
| S | Junca-Marti |
| Josephine | Jung |
| M | Jung |
| Stefanie | Junker |
| Evelina | Juodiene |
| Domantas | Juodis |
| M | Jurado Ruiz |
| Maria | Jurado Ruiz |
| Jonas | Jurgaitis |
| M | Juricic |
| Melodie | Juricic |
| Steffanie | Jury |
| Richard | Justin Davies |
| B | Juthani |
| Gs | Jutley |
| Zeljka | Jutric |
| Nina | Jyne Minette Dela Cruz |
| Gayathri | Jyothish |
| Tejeswini | K K |
| DKK | K M |
| Samuel | Ka Kin Ling |
| H | Kaafarani |
| Claudia | Kabanyana |
| Rakan | Kabariti |
| Taha | Kabbaj |
| Saadullah | Kabbany |
| Obaida | Kabel |
| Tousif | Kabir |
| Mohammed | Kabir Abdullahi |
| Mohammed | Kabir Abubakar |
| Apoorva | Kabra |
| Navid | Kabuli |
| Wilberforce M | Kabweru |
| Stephen | Kache |
| Ahmad | Kachoie |
| SEO | Kacimi |
| Akram | Kadamani Abiyomaa |
| K | Kadantseva |
| Saidu | Kadas |
| Nardeen | Kader |
| Mustafa R | Kadhim |
| Mohamad | Kadi |
| M | Kadija |
| S | Kadija |
| Innih | Kadiri |
| Lama | Kadoura |
| D | Kaemmerer |
| Alper | Kafkasli |
| Abdullah | Kağan Zengin |
| Kota | Kagawa |
| S | Kahane |
| Hiba | Kahi |
| J | Kahiu |
| Josephine | Kahiu |
| Ehab | Kahka |
| A | Kahn |
| Alexis | Kahn |
| J | Kahn |
| Judith | Kahn |
| Li | Kai |
| Dilyara | Kaidarova |
| Yu | Kaiho |
| S | Kailasam sivamurthy |
| Suresh | Kailasam Sivamurthy |
| M | Kajic |
| Martin | Kajic |
| B | Kajmaković |
| Boris | Kajmaković |
| Michael | Kakas |
| Andrew | Kakeeto |
| V | Kakotkin |
| Victor | Kakotkin |
| Navneet | Kala |
| Prakash | Kala |
| Dana | Kalagi |
| I | Kalaitsidou |
| Theodosis | Kalamatianos |
| N | Kalavrezos |
| Nicholas | Kalavrezos |
| Ayrat | Kaldarov |
| A | Kale |
| Ahmet | Kale |
| S | Kale |
| Sachin | Kale |
| SS | Kale |
| R | Kalenderov |
| Anna | Kaleva |
| Fotios | Kalfas |
| JC | Kalff |
| Jörg C | Kalff |
| Christos | Kalfountzos |
| I | Kaliamoorthy |
| VK | Kalidindi |
| Muhammad | Kalim |
| M | Kalın |
| Murat | Kalın |
| Senad | Kalkan |
| M | Kalkat |
| K | Kalkwarf |
| Kyle | Kalkwarf |
| Mostafa | Kallaf |
| Mamdouh | Kallas |
| Vasileios | Kalles |
| SK | Kallikere lakshmana |
| Michael | Kallmayer |
| Katinka | Kallos |
| Attila | Kalman |
| I | Kalogiannidis |
| Ioannis | Kalogiannidis |
| Nikos | Kalogritsas |
| K | Kalopita |
| Neeraj | Kalra |
| NS | Kalson |
| F | Kalt |
| Athula | Kaluarachchi |
| Neha | Kalwadia |
| A | Kalyanasundaram |
| Asanish | Kalyanasundaram |
| N | Kalyva |
| Aristotelis | Kalyvas |
| A | Kam da Silva Andrade |
| M | Kamal |
| Wajahat | Kamal |
| Awad | Kamal Awad Osman |
| Bhavani | Kamalakannan |
| A | Kamali |
| Nelson | Kamali |
| A | Kamalov |
| L | Kaman |
| Lileswar | Kaman |
| MFA | Kamarizan |
| N | Kamath |
| Tatsuya | Kambara |
| B | Kamburoglu |
| F | Kamel |
| Mahmoud | Kamel |
| P | Kamenova |
| Paolina | Kamenova |
| M | Kamenskikh |
| BS | Kamera |
| Aleksejs | Kaminskis |
| Akio | Kamiya |
| Mizue | Kamiyama |
| Hatem | Kamkoum |
| Christian | kammerer Kammerer |
| Christian | Kammerlander |
| Elhusain | Kamoka |
| C | Kamphues |
| Carsten | Kamphues |
| Liisa | Kams |
| Rahul | Kanade |
| D | Kanagal |
| Trisha | Kanani |
| Anastasios | Kanatas |
| S | Kanavathy |
| Prodromos | Kanavidis |
| Akihiro | Kanaya |
| Pankaj | Kandwal |
| S | Kaneko |
| Satoi | Kaneko |
| Vasiliki | Kanellopoulou |
| Eiki | Kanemaru |
| Sayaka | Kanematsu |
| Pepa | Kaneva |
| Sara | Kanfar |
| C | Kang |
| J | Kang |
| Niel | Kang |
| Harsh | Kanhere |
| R | Kanitkar |
| Hadyn K N | Kankam |
| Burak | Kankaya |
| Lavanya | Kannaiyan |
| Ravi | Kannan |
| Sreejith | Kannummal Veetil |
| Kari | Kansal |
| R | Kansay |
| Rajeev | Kansay |
| Baturay | Kansu Kazbek |
| Ravi | Kant |
| Uma | Kant Dutt |
| S | Kanthasamy |
| E | Kantor |
| Astha | Kantroo |
| E | Kaouras |
| Nandkishore | Kapadia |
| G | Kapetanios |
| Georgios | Kapetanios |
| S | Kapiris |
| Stylianos | Kapiris |
| Mark L | Kaplan |
| Mehmet | Kaplan |
| Nathan | Kaplan |
| Tuğba | Kaplan |
| M | Kaple |
| C | Kapoen |
| K | Kapoor |
| S | Kapoor |
| Swapnil | Kapote |
| K | Kapriniotis |
| P | Kapsampelis |
| Lakith | Kapuge |
| K | Kapur |
| Anna | Kapustina |
| AR | Kar |
| Madhabananda | Kar |
| Halil | Kara |
| Y | Kara |
| Yasi̇n | Kara |
| O | Karaaslan |
| Kerim | Karabulut |
| Mehmet | Karabulut |
| Bugrahan | Karaca |
| H | Karaca |
| G | Karadeniz Cakmak |
| Dimitra | Karageorgou |
| G | Karagiannidis |
| Georgios | Karagiannidis |
| Omer | Karahan |
| Tayfun | Karahasanoğlu |
| Sema | Karakaş |
| HK | Karakullukcu |
| Basil | Karam |
| BS | Karam |
| E | Karam |
| James | Karam |
| Jose | Karam |
| Mohammad | Karam Chaaban |
| E | Karaman |
| M | Karamanliev |
| Martin | Karamanliev |
| A | Karamarkovic |
| Aleksandar | Karamarkovic |
| T | Karami |
| S | Karandikar |
| Sharad | Karandikar |
| P | Karanicolas |
| AKA | Karantenachy |
| Shoura | Karar |
| Irem | Karatas |
| Ioannis | Karavokyros |
| A | Karbalaie |
| M | Karbowiak |
| A | Kareem Hama Ghareeb |
| CT | Karia |
| Maahir | Kariem |
| Nazmie | Kariem |
| Costa | Karihaloo |
| A | Karim |
| S | Karim |
| Sabbir | Karim |
| Ebrahim | Karimi |
| Ali | Karimi Karimi |
| Liz | Karina |
| Rajeev | Kariyattil |
| Shirin | Karkada |
| B | Karki |
| Robert | Karlo |
| Rahi | Karmarkar |
| Santosh | Karmarkar |
| Sunaina | Karna |
| Gomathi | Karnan |
| Ruchi | Karnatak |
| Paraskevi | Karona |
| Eleni | Karoni |
| Mehdi | Karoui |
| L | Karout |
| Lina | Karout |
| S | Karout |
| Samar | Karout |
| Mariia | Karpenko |
| Ilya | Karpov |
| M | Karthigeyan |
| Madhivanan | Karthigeyan |
| Intan | Kartika Kamarudin |
| Eranda | Karunadasa |
| L | Karydakis |
| Lysandros | Karydakis |
| Ashenafi | Kasaye |
| C | Kaselas |
| Christos | Kaselas |
| M | Kashif |
| Muhammad | Kashif |
| Eiji | Kashiwagi |
| F | Kashora |
| V | Kasivisvanathan |
| Ł | Kaska |
| George | Kasotakis |
| D | Kassa |
| Dawit | Kassa |
| Haftamu | Kassa |
| MB | Kassab |
| Mohamad B | Kassab |
| Berhanu | Kassahun |
| Miklos | Kassai |
| Al-Faraaz | Kassam |
| K | Kassam |
| Nausheen | Kassam |
| Bersabeh | Kassaye |
| R | Kassir |
| Radwan | Kassir |
| Terhemen | Kasso |
| Pagona | Kastanaki |
| Zeljko | Kastelan |
| Dimithi | Kasthurirathne |
| A | Kataria |
| Lena | Katharina Mueller |
| Sheyla | Katherine Diaz Mora |
| Tharangani | Kathiravan |
| Anne | Kathleen Ganal-Antonio |
| Mary | Kathryn Abel |
| H | Kato |
| Manabu | Kato |
| T | Kato |
| Airi | Katoh |
| I | Katsaros |
| L | Katsiaras |
| Emmanuel | Katsogridakis |
| A | Kattakayam |
| Arjun | Kattakayam |
| Abdullah | Kattan |
| Jevgeni | Katunin |
| Matthew | Katz |
| Philipp | Kauffmann |
| Micayla | Kaufman |
| A | Kaufmann |
| Angelika | Kaufmann |
| Claudia | Kaufmann |
| P | Kaul |
| J | Kauppila |
| JH | Kauppila |
| Joonas | Kauppila |
| Joonas H | Kauppila |
| Krista | Kaups |
| Amanjot | Kaur |
| Apjit | Kaur |
| Gurleen | Kaur |
| Gurvinder | Kaur |
| Harmanpreet | Kaur |
| J | Kaur |
| Jasprit | Kaur |
| P | Kaur |
| R | Kaur |
| Navdeep | Kaur Ghuman |
| Jaspreet | Kaur Seehra |
| M | Kaushal |
| Manish | Kaushal |
| R | Kaushik |
| Robin | Kaushik |
| Tirathram | Kaushik |
| Vivek | Kaushik |
| Alfie | Kavalakat |
| A | Kavaliauskaitė |
| Dara | Kavanagh |
| DO | Kavanagh |
| N | Kavčič |
| Niko | Kavčič |
| S | Kavic |
| Thumuluru | Kavitha Madhuri |
| Aya | Kawachi |
| I | Kawagoe |
| Izumi | Kawagoe |
| M | Kawaguchi |
| Masahiko | Kawaguchi |
| Akira | Kawai |
| Natsuko | Kawamata |
| Kenji | Kawamukai |
| K | Kawamura |
| Kenji | Kawamura |
| Abdulmonem | Kawas |
| Yosuke | Kawasaki |
| Mahmoud | Kawu Magashi |
| Hannah | Kay |
| Rozan | Kaya |
| T | Kaya |
| Tayfun | Kaya |
| Akshat | Kayal |
| AA | Kayali |
| B | Kayan |
| B | Kayani |
| K | Kayani |
| Stephen | Kaye |
| B | Kaymak |
| Olushola | Kayode Fasiku |
| Victor | Kayode-Nissi |
| E | Kayombo |
| Emile | Kayombo |
| Silvia | Kayser Mata |
| MY | Kayyal |
| Ammar | Kayyali |
| E | Kazachenko |
| Ekaterina | Kazachenko |
| Ozgur | Kazan |
| Mohammad | Kazem Moslemi |
| K | Kazemi Esfe |
| Amr | Kazim |
| Muhammad | Kazim Rahim Najjad |
| Abbas | Kazmi |
| S | Kazuma |
| Satoshi | Kazuma |
| S | Kazzaz |
| Sarmad | Kazzaz |
| Joshua | Kealey |
| D | Kearney |
| David | Kearney |
| James M | Keatley |
| E | Kebapçı |
| Andrey | Kebkalo |
| Aristotelis | Kechagias |
| Nahla | Kechiche |
| B | Keeler |
| Daniel | Keese |
| Samer | Kefo |
| Farid | Kehdy |
| Wang | Kei Chiu |
| W | Kelder |
| Dionysia | Kelgiorgi |
| Sorcha | Kellett |
| Brian | Kelley |
| Andrew | Kelly |
| Ben | Kelly |
| John | Kelly |
| Kathrin | Kelly |
| Kevin | Kelly |
| M | Kelly |
| Mairéad | Kelly |
| Michael | Kelly |
| Orlaith | Kelly |
| Ronan | Kelly |
| Sarah | Kelly |
| CJ | Kelty |
| Ifeanyichukwu | Kelvin Egbuchulem |
| Uchenna | Kelvin Omeje |
| Aya | Kelzia |
| Ifeanyi | Kem Onubogu |
| Ben-Lawrence | Kemah |
| Achmad | Kemal Harzif |
| G | Kembuan |
| Gabriele | Kembuan |
| Vanessa | Kemmetinger |
| P | Kempter |
| Sebastian | Ken-Amoah |
| Brittany | Kendall |
| Farzaneh | Keneshlou |
| Mihaly | Kenez |
| M | Kenic |
| Marko | Kenic |
| C | Kennedy |
| L | Kennedy |
| N | Kennedy |
| R | Kennedy |
| Andrew | Kennedy-Dalby |
| R | Kennelly |
| Rory | Kennelly |
| Maria | Kenner |
| Yvann | Kenneth Benosa |
| Adi | Kenoshi |
| A | Kent |
| E | Kent |
| I | Kent |
| C | Keogh |
| John | Keogh |
| S | Keogh-bootland |
| Markéta | Kepičová |
| Alexander M | Keppler |
| AM | Keppler |
| Lena | Keppler |
| MR | Keramati |
| MD | Keramida |
| AA | Kerawala |
| Cyrus | Kerawala |
| Thomas | Kerforne |
| M | Kerin |
| MJ | Kerin |
| A | Kerman |
| J | Kerman |
| H | Kerndl |
| Jola | Kerpaci |
| David | Kerr |
| Megan | Kerr |
| NA | Kerr |
| Megan | Kershaw |
| Venkatesh | Kesarla |
| M | Keskin |
| Metin | Keskin |
| S | Kesseli |
| F | Kethy |
| MN | Ketkar |
| S | Ketting |
| MH F | Keulen |
| B | Kewlani |
| Vishal | Kewlani |
| Amana | Kezze |
| Nikhil | Khadabadi |
| Mamoona | Khadam |
| Salma | Khadem Alsrouji |
| S | Khader |
| Sereen | Khader |
| Kouidri | Khadidja |
| F | Khadwardi |
| Ali | Khafaja |
| AKM | Khairul Basher |
| F | Khajavi-Mayvan |
| M | Khajeh Alizadeh Attar |
| A | Khajuria |
| Abdullah | Khalaf |
| M | Khalaf |
| A | Khaled |
| Ahmed | Khaled |
| Mohamed | Khaled |
| Mhd | Khaled Alkasser |
| Nuran | Khaled Aly |
| Omar | Khaled Mohamed Eid |
| A | Khaleel |
| Tahir | Khaleeq |
| M | Khalefa |
| Ismaeel | Khalid |
| K | Khalid |
| Muhammad | Khalid |
| Shahril | Khalid |
| Tabinda | Khalid |
| Ziaullah | Khalid |
| Ahmed | Khalid alhadheeri |
| Shayan | Khalid Ghaloo |
| Raja | Khalid Shabbir |
| A | Khalifa |
| Aya | Khalifa |
| Eiman | Khalifa |
| Houda | Khalifa |
| Islam | Khalifa |
| M | Khalifa |
| A | Khalil |
| Kareem S | Khalil |
| Mariam | Khalil |
| Mohammed | Khalil |
| Rasha | khalil Alsayyad |
| F | Khaliq |
| Sameera | Khaliq |
| T | Khaliq |
| Tanwir | Khaliq |
| Mohamed | Khallaf |
| A | Khamees |
| Almuatasim | Khamees |
| A | Khan |
| Aimal | Khan |
| Aneesah | Khan |
| Anwar | Khan |
| Asher | Khan |
| Farheen | Khan |
| Fatima | Khan |
| Fatma | Khan |
| H | Khan |
| Hamad | Khan |
| Hamza | Khan |
| Hassan | Khan |
| HH | Khan |
| J | Khan |
| Jamal | Khan |
| Jim | Khan |
| K | Khan |
| Karishma | Khan |
| Khizar | Khan |
| Maaz | Khan |
| Maham | Khan |
| Majid | Khan |
| Maryam | Khan |
| MK | Khan |
| MS | Khan |
| MT | Khan |
| MTJ | Khan |
| Najeed | Khan |
| R | Khan |
| Rohma | Khan |
| Romaisa | Khan |
| S | Khan |
| Sabina | Khan |
| Sadia | Khan |
| Salman | Khan |
| Sami | Khan |
| Shane | Khan |
| Shifa | Khan |
| Tabassum | Khan |
| U | Khan |
| W | Khan |
| Wasim | Khan |
| WH | Khan |
| Tanishq | Khandelwal |
| Aseel | Khanfer |
| Sukhwant | Khanijaun |
| H | Khansaheb |
| Hamda | Khansaheb |
| Zubair | Khanzada |
| Siddhant | Khare |
| H | Kharkar |
| Dimple | Kharkongor |
| Barihan | Khasawneh |
| Saad | Khashogji |
| Hazem | Khatab |
| Roa | Khatatbeh |
| Chetan | Khatri |
| Mazin | Khattabi |
| F | Khatun |
| Rong | Khaw |
| SC | Khaw |
| I | Khawaja |
| UA | Khawaja |
| S | Khayat |
| Maymona | Khayata |
| Tareq | Kheirbek |
| Samer | Khel |
| Zine-Eddine | Khene |
| Osama | Kherallah |
| Safeena | Kherani |
| Talal | Khewater |
| Priyatma | Khincha |
| MJ N | Kho |
| A | Khodarahmi |
| Ahmad | Khoja |
| Christopher | Khoory |
| Victoria E | Khoronenko |
| Amir | Khoshbin |
| M | Khosravi |
| Mohammad | Khosravi |
| Gleb | Khrykov |
| KJ O | Khu |
| Wafa | Khudier |
| Susanta | Khuntia |
| Helene | Khuong |
| Taran | Khurana |
| Muhammad | Khurram Jameel |
| Zain | Khurshid |
| K | Khutsishvili |
| K | Khwaja |
| E | Khya |
| Kevin | Ki Wai Ho |
| Yong | Kiat Goh |
| Sey | Kiat Terence Lim |
| Muthoni | Kibunyi |
| H | Kiconco |
| Tatsuya | Kida |
| B | Kidane |
| M | Kidane |
| Meklit | Kidane |
| John | Kiely |
| P | Kienle |
| K | Kieran |
| Aoife | Kiernan |
| C | Kies |
| David | Kieser |
| SY | Kiessling |
| Omar | Kifayeh |
| Motohiro | Kikukawa |
| Nura | Kilic |
| G | Kilinc |
| Gizem | Kilinc |
| G | Kilinc Tuncer |
| S | Killeen |
| Shane | Killeen |
| Rhona | Kilpatrick |
| B | Kim |
| E | Kim |
| G | Kim |
| Gleb | Kim |
| NJ | Kim |
| M | Kimbrough |
| Mary | Kimbrough |
| Motonobu | Kimizuka |
| Angharad | King |
| J | King |
| Jasmin | King |
| M | King |
| Martin | King |
| N | King |
| SD | King |
| Sebastian | King |
| Stratton | King |
| B | King-Koi |
| Dale | Kingsley Sy |
| G | Kinnaman |
| Fumio | Kinoshita |
| H | Kinoshita |
| Hidefumi | Kinoshita |
| J | Kinross |
| J | Kinsella |
| Racheal | Kirabo |
| P | Kirchweger |
| Patrick | Kirchweger |
| Nikolaos | Kiriakopoulos |
| Lydia | Kirillova |
| A | Kirk |
| EF | Kirkan |
| B | Kirmani |
| BH | Kirmani |
| Bilal | Kirmani |
| S | Kirmani |
| Y | Kirmizi |
| Yasemin | Kirmizi |
| Mikhail | Kirov |
| A | Kirschniak |
| Andreas | Kirschniak |
| Joel | Kiryabwire |
| Ravi | Kishore Barla |
| A | Kisiel |
| U | Kisser |
| Ulrich | Kisser |
| Castro | Kisuule |
| Masato | Kita |
| Hiroaki | Kitade |
| Hiroyuki | Kitagawa |
| M | Kitchen |
| Yury | Kitsenko |
| Madhav | Kittur |
| Ronald | Kiweewa |
| Tevfik | Kıvılcım Uprak |
| Abdulqader | Klaho |
| D | Klaristenfeld |
| Jaroslav | Klat |
| T | Klatte |
| TO | Klatte |
| Tobias | Klatte |
| Friederike | Klauke |
| J | Kleeff |
| Jorg | Kleeff |
| Z | Kleiman |
| A | Kler |
| Fredrik | Klevebro |
| Karel | Klíma |
| S | Klimopoulos |
| A | Klimov |
| JH G | Klinkenbijl |
| W | Kloc |
| C | Kloppers |
| Christo | Kloppers |
| J | Klose |
| Johannes | Klose |
| Luis | Kluth |
| G | Klutts |
| Garrett | Klutts |
| S | Kmezić |
| Stefan | Kmezić |
| Elizabeth | Kmiotek |
| U | Kneser |
| Ulrich | Kneser |
| Jure | Knez |
| D | Knezevic |
| Djordje | Knezevic |
| Darko | Knežević |
| Dominic | Knight |
| J | Knipschild |
| Julia | Knipschild |
| M | Knitschke |
| Michael | Knitschke |
| WT | Knoefel |
| Christian | Knorr |
| H | Knotzer |
| Hans | Knotzer |
| Brett | Knowles |
| C | Knowles |
| J | Knowles |
| M | Ko |
| Satoshi | Kobayashi |
| Takayuki | Kobayashi |
| Toshinori | Kobayashi |
| Yasuma | Kobayashi |
| Yoichi | Kobayashi |
| Isaac | Kobe |
| Nina | Kobilica |
| T | Koc |
| MA | Koç |
| A | Kocatas |
| Ali | Kocataş |
| B | Kocer |
| Belma | Kocer |
| C | Koch |
| Christian | Koch |
| I | Koch |
| Oliver | Koch |
| GJ | Kocher |
| Hemant | Kocher |
| HM | Kocher |
| Stanislav | Kocherov |
| V | Kochetkov |
| Viktor | Kochetkov |
| VS | Kochetkov |
| T | Kochiyama |
| Tsukasa | Kochiyama |
| Koshy | Kochummen |
| Milan | Kocic |
| Bogdan | Koczy |
| S | Kodange |
| Almat | Kodasbaev |
| D | Koenig |
| F | Koeninger |
| Evans | Kofi Agbeno |
| Yuko | Koga |
| P | Koggoh |
| Patience | Koggoh |
| Eleni | Kogia |
| P | Köglberger |
| Paul | Köglberger |
| A | Koh |
| Amanda | Koh |
| Cherry | Koh |
| Frederick | Koh |
| F | Koh Hong Xiang |
| Frederick | Koh Hong Xiang |
| Z | Kohistani |
| Zaki | Kohistani |
| D | Koike |
| Minako | Koizumi |
| Edem | Kojo Dzantor |
| S | Koju |
| SY | Kok |
| H | Köken |
| Georgios | Kokkinos |
| A | Kokobelyan |
| P | Kokoropoulos |
| Panagiotis | Kokoropoulos |
| George | Kokosis |
| ÜC | Köksoy |
| V | Kolaityte |
| Valdone | Kolaityte |
| Juraj | Kolak |
| Arif | Kolethekkat |
| M | Koleva Radica |
| A | Kolias |
| Angelos | Kolias |
| Angeliki | Kolinioti |
| Georgios | Koliopoulos |
| Florestan | Koll |
| C | Kolla |
| V | Kollias |
| O | Kollmar |
| Otto | Kollmar |
| Maria | Kolokotroni |
| Kemalettin | Koltka |
| A | Kolusab |
| Snigdha | Komatineni |
| Melanie | Komaz |
| YS | Kömek |
| N | Komen |
| Niels | Komen |
| S | Kommu |
| A | Konarski |
| Ibrahima | Konate |
| Can | Konca |
| Igor | Koncar |
| S | Konda |
| Panagiotis | Kondilis |
| Anish | Koneru |
| S | Konev |
| JCH | Kong |
| Daniela | König |
| TT | König |
| A | Königsrainer |
| Alfred | Königsrainer |
| I | Königsrainer |
| Ingmar | Königsrainer |
| Suzana | Konjevoda |
| Anna | Konney |
| Yoshiharu | Kono |
| C | Konrads |
| Christian | Konrads |
| M | Konstadoulakis |
| Manousos | Konstadoulakis |
| K | Konstantinidi |
| Michael | Konstantinidis |
| MK | Konstantinidis |
| Sofia | Konstantinidou |
| C | Konstantinou |
| J | Konsten |
| Joop | Konsten |
| E | Kontis |
| C | Kontopoulou |
| Christina | Kontopoulou |
| Konstantina | Kontopoulou |
| C | Kontovounisios |
| Kenneth | Koo |
| Mohsen | Koosha |
| Slava | Kopetskyi |
| T | Kopjar |
| Tomislav | Kopjar |
| Leon-Gordian | Köpke |
| C | Korais |
| Christos | Korais |
| S | Korasidis |
| Stylianos | Korasidis |
| C | Koratzanis |
| Moniba | Korch |
| N | Korchazhkina |
| DS | Korkmaz |
| D | Korkolis |
| Dimitrios | Korkolis |
| S | Korn |
| Sandra | Korn |
| M | Kornaszewska |
| Lucy | Kornblith |
| LZ | Kornblith |
| Grigory | Korolev |
| Porfyrios | Korompelis |
| N | Korres |
| A | Korthaus |
| Inye | Korubo |
| E | Kose |
| Selçuk | Köse |
| Andrey | Koshel |
| Z | Koshnow |
| Rakesh | Koshy |
| RM | Koshy |
| J | Kosir |
| JA | Košir |
| T | Košir Božič |
| Lisa | Koslowski |
| M | Kost |
| Ioannis | Kostakis |
| M | Kostic |
| Yevhenii | Kostiuchenko |
| Albert | Kota |
| Tuncay | Kötan |
| Jan | Kotarski |
| Mostafa | Kotb |
| A | Kothari |
| Tommi | Kotkavaara |
| Denis | Kotov |
| L | Kottam |
| R | Kottayasamy Seenivasagam |
| Rajkumar | Kottayasamy Seenivasagam |
| W | Kotyczka |
| Ali | Kouhi |
| G | Koukoulis |
| Georgios | Koukoulis |
| O | Koukoura |
| A | Koulouktsis |
| Marinos | Koulouroudias |
| Mohammed | Koumu |
| Mandeep | Koundu |
| Alaa | Kour |
| K | Kour |
| Mohammad | Kour |
| Robindera | Kour |
| A | Kourdouli |
| Marinos S | Kouris |
| V | Kouritas |
| Vasileios | Kouritas |
| S | Koussayer |
| Samer | Koussayer |
| M | Koutentakis |
| A | Kouyoumdjian |
| Basel | Kouz |
| Bojan | Kovacevic |
| M | Kovačević |
| Petra | Kovačević |
| Ivan | Kovačić |
| Rok | Kovačič |
| Shama | Kovale |
| M | Kowal |
| Karl-Friedrich | Kowalewski |
| KF | Kowalewski |
| LP | Kowalski |
| Ana | Kowark |
| Pascal | Kowark |
| LP | Kowaski |
| Youssef | Kozah |
| R | Kozan |
| Ramazan | Kozan |
| Sarunas | Kozenevskis |
| Akvilė | Koženiauskaitė |
| Zoran | Kozomara |
| Richard | Kpangkpari |
| Cyrille | Kpangon |
| Martina | Kralinger |
| M | Kranawetter |
| Marlene | Kranawetter |
| Peter | Kranke |
| Dietmar | Krappinger |
| Peter-Martin | Krarup |
| Virgilijus | Krasauskas |
| C | Kratochwila |
| Chiara | Kratochwila |
| Johannes R | Kratz |
| T | Kratzer |
| A | Krause |
| H | Krause |
| Hardy | Krause |
| Joanna | Krawczyk |
| W | Krawczyk |
| I | Krdzic |
| Igor | Krdzic |
| S | Krejovic Trivic |
| Sanja | Krejovic Trivic |
| V | Kremo |
| Josip | Kresic |
| Tanja | Krešić |
| M | Kresoja |
| Giorgos | Krestinidis |
| D | Krief |
| Maximilian | Kriegmair |
| Eslam | Kriem |
| Andrey | Kriger |
| D | Krinock |
| Sunil | Krishna |
| Ashvin | Krishna Nair |
| Bal | Krishna Ojha |
| Mahesh | Krishna Pillai |
| Pradeep | Krishna RV |
| murali | Krishna Voonna |
| Vijay | Krishnamoorthy |
| Gargeshwari | Krishnamurthy Guru Raghavendra |
| Bala | Krishnan |
| E | Krishnan |
| Emily | Krishnan |
| Ratha | Krishnan Sriram |
| Sivakumar | Krishnasamy |
| Cristina | Kristel Tonos Sardiñas |
| HØ | Kristensen |
| Jens | Kristian Bælum |
| S | Kristinsson |
| Abirami | Krithiga |
| Neoklis | Kritikos |
| Zoran | Krivokapic |
| Marie | Kröger |
| Irmgard | Kronberger |
| Joshua | Kronenfeld |
| JP | Kronenfeld |
| Hidde M | Kroon |
| HM | Kroon |
| E | Kropf |
| I | Kruger |
| S | Kruijff |
| Schelto | Kruijff |
| SH | Kruijff |
| Jakob | Kruschwitz |
| SureshKannan | Ks |
| DS | Kshirsagar |
| Antigoni | Ktisti |
| Beatrice | Kuang |
| E | Kubiliute |
| Nikita | Kubin |
| Hubert | Kübler |
| Hisako | Kubota |
| V | Kubyshkin |
| Valery | Kubyshkin |
| A | Kuc |
| F | Kucuk |
| GO | Kucuk |
| S | Kudchadkar |
| Shantata | Kudchadkar |
| A | Kudpaje |
| Y | Kudryavcev |
| Søren | Kudsk-Iversen |
| B | Kuehlmann |
| Britta | Kuehlmann |
| Marlene | Kuen |
| D | Kufeji |
| N | Kugler |
| Magda | Kujawa |
| Yerlan | Kukubassov |
| K | Kułak |
| MA V | Kulcsar |
| Marco | Kulcsar |
| Judit | Kulcsicka-Gut |
| O | Kuleshov |
| Justas | Kuliavas |
| Mukhtar | Kulimbet |
| Tomislav | Kulis |
| Amol | Kulkarni |
| Amruta | Kulkarni |
| Avadhut | Kulkarni |
| G | Kulkarni |
| Gauri | Kulkarni |
| Nikhil | Kulkarni |
| R | Kulkarni |
| Rugved | Kulkarni |
| Yogesh | Kulkarni |
| A | Kumar |
| Abhaya | Kumar |
| Abhinav | Kumar |
| Ambrish | Kumar |
| Anil | Kumar |
| Aruna | Kumar |
| Arvind | Kumar |
| Ashwani | Kumar |
| J | Kumar |
| Kranthi | Kumar |
| L | Kumar |
| Manoj | Kumar |
| Mohit | Kumar |
| Naren | Kumar |
| Navin | Kumar |
| Navneet | Kumar |
| Neha | Kumar |
| Pankaj | Kumar |
| Ravi | Kumar |
| Rohit | Kumar |
| S | Kumar |
| Shashank | Kumar |
| Subodh | Kumar |
| Upander | Kumar |
| V | Kumar |
| Vijay | Kumar |
| Vishal | Kumar |
| Sanjit | Kumar Agrawal |
| Akshay | Kumar Bisoi |
| Navneet | Kumar Chaudhry |
| Pawan | Kumar Dhruva Rao |
| Surya | Kumar Dube |
| Pankaj | Kumar Garg |
| Sunil | Kumar Gupta |
| Anoop | kumar Jaiswal |
| Saubhagya | Kumar Jena |
| Vijay | Kumar Kumar |
| Barani | kumar P B Pb |
| Piravin | Kumar Ramakrishnan |
| Santhosh | kumar Sampengere Annayappa |
| Amit | kumar Shrivastava |
| Piyush | Kumar Sinha |
| Santhosh | Kumar Thangaraj |
| Virendra | Kumar Tiwari |
| S | Kumar Venkatappa |
| Sunil | Kumar Venkatappa |
| Sunil | kumar Vishwakarma |
| Sumudu | Kumarage |
| NK | Kumaran |
| Kanesh | Kumaran Seevalingam |
| M | Kumari |
| Pushplata | Kumari |
| Sameeta | Kumari |
| Sujatha | Kumari |
| Philemon | Kumassah |
| Kashmira | Kumawat |
| Sean | Kumer |
| Sho | Kumita |
| Felix | Kumolalo |
| JD | Kün-Darbois |
| Jean-Daniel | Kün-Darbois |
| Rastislav | Kunda |
| R | Kundra |
| Rakesh | Kundra |
| Nikhil | Kundu |
| Kristian | Kunjko |
| Joseph | Kunju Mathew |
| Yunus | Kuntawi Aji |
| Priyanka | Kunte |
| Isabella | Kuo |
| Louise | Kuo |
| Stephen | Kuo |
| Bruna | Kupper |
| Mohammed | Kura |
| DA B | Kuramoto |
| N | Kuratani |
| Norifumi | Kuratani |
| Musbahu | Kurawa |
| W | Kurdi |
| H | Kurihara |
| A | Kuriyama |
| Akira | Kuriyama |
| Andrii | Kurmanskyi |
| Juni | Kurniawaty |
| Kento | Kuroda |
| N | Kuroda |
| Naoto | Kuroda |
| Cameron | Kuronen-Stewart |
| M | Kurtenkov |
| Mikhail | Kurtenkov |
| Michael | Kurtz |
| A | Kushairi |
| Tetsuya | Kushikata |
| Kwasi | Kusi |
| Sidharta | Kusuma Manggala |
| A | Kut |
| Mariya | Kuteva |
| J | Kutkevicius |
| E | Kutlu Yalcin |
| Kudzayi | Kutywayo |
| Ekins | Kuuzie |
| Vsevolod | Kuzkov |
| C | Kuzmanovic |
| A | Kuzovlev |
| Shigeki | Kuzuhara |
| Aswathi | Kv |
| Nana | Kwaku Agyeman-Duah |
| A | Kwan |
| R | Kwan-Feinberg |
| Daniel | Kwesi Acquah |
| A | Kwiatkowski |
| Patrick | Kwizera |
| AM F | Kwok |
| Kelvin | Kwok |
| M | Kwok |
| Stephanie | Kwok |
| George | Kwok Chu Wong |
| Kelvin | Kwok-Chai Ng |
| David | Kwon |
| Audrey | Kwong |
| Jin | Kwun |
| Ishmael | Kyei |
| S | Kykalos |
| Stylianos | Kykalos |
| Matthew | Kynes |
| Harry | Kyriacou |
| Ioannis | Kyritsis |
| E | Kyrodimos |
| Efthymios | Kyrodimos |
| Eleandros | Kyros |
| Ioanna | Kyrou |
| V | Kyvelos |
| Vasilis | Kyvelos |
| M | Kyzer |
| Badareesh | L |
| Sharathkumarkl | L |
| D | L. Cruz Condori |
| A | L. Minussi |
| E | La Corte |
| Emanuele | La Corte |
| Antonio | La Greca |
| Roberta | La Mendola |
| Carlotta | La Raja |
| D | La Regina |
| Stefania | La Rocca |
| F | La Torre |
| Filippo | La Torre |
| M | La Torre |
| M | Labalde Martinez |
| Simon | Laban |
| Fernando | Labarga Rodríguez |
| Ivan | Labetov |
| A | Labib |
| PL | Labib |
| Nicole | Labine |
| Nathalie | Labrecque |
| Domenico | Lacavalla |
| Vasileios | Lachanas |
| Tunc | Lacin |
| AM | Lacy |
| Kaylan | Lad |
| P | Lad |
| Parag | Lad |
| Vidyadhar | Lad |
| MR | Ladd |
| Karim | Ladha |
| Oluwaseun | Ladipo-Ajayi |
| Roland | Ladurner |
| T | Laeke |
| Tsegazeab | Laeke |
| L | Laface |
| Letizia | Laface |
| George | Lafford |
| Yasser | Lafi |
| Anaïs | Laforest |
| R | Laforgia |
| AS | Laganà |
| A | Lagares |
| Daniella | Laguado |
| Gregorio | Laguna |
| Emilio | Lagunas Lostao |
| K | Lah |
| Raghad | Lahlooh |
| W | Lahlou |
| M | Lahmar |
| Zoe | Lahood |
| A | Lahoud-Velaochaga |
| Jack | Lahy |
| S | Lai |
| Alexandros | Laios |
| A | Laird |
| Alexander | Laird |
| A | Lakehal |
| Aarti | Lakhiani |
| K | Lakhoo |
| Z | Lakkis |
| Zaher | Lakkis |
| S | Lakpriya |
| Sathya | Lakpriya |
| Prashant | Lakshman |
| Antoine | Laktine |
| Nikhil | Lal |
| Ashish | Lal Shrestha |
| AK | Lala |
| M | Lallemand |
| Lara | Lallitsch |
| Ismahene | Lalmi |
| A | Lalos |
| J | Laloze |
| Jerome | Laloze |
| Andrew | Lam |
| Dominic | Lam |
| K | Lam |
| Susanna | Lam |
| YH | Lam |
| B | Lamb |
| Anton | Lambers |
| Leo | Lambers |
| J | Lambert |
| Virginia | Lambert |
| Antoine | Lamblin |
| M | Lami |
| Mariam | Lami |
| R | Lamm |
| S | Lammy |
| Simon | Lammy |
| P | Lamoral |
| C | Lampert |
| Valerie | Lan-Pak-Kee |
| L | Lancerotto |
| Luca | Lancerotto |
| Antonio | Lanci Lanci |
| A | Landaluce-Olavarria |
| Aitor | Landaluce-Olavarria |
| SA | Landeo Agüero |
| FJ | Landete Molina |
| Giovanni | Landoni |
| L | Landoni |
| Giulia I | Lane |
| J | Lane |
| Oliver | Lane |
| Rondall | Lane |
| Allison T | Lanfear |
| D | Lanfranco |
| Elena | Lang |
| K | Langeveld |
| Ream | Langhe |
| Eve-Lyne | Langlais |
| T | Langlais |
| Tristan | Langlais |
| F | Langlands |
| Fiona | Langlands |
| A | Langone |
| Maximilian | Lanner |
| R | Łanowy |
| Brent | Lanting |
| G | Lantone |
| L | Lanuza |
| A | Lanzone |
| Maria | Lapeña Rodríguez |
| MC | Lapitan |
| P | Lapolla |
| Pierfrancesco | Lapolla |
| G | Laporte |
| Gustavo | Laporte |
| A | Lara |
| Antonio | Lara |
| Arkaitz | Lara |
| Sabino | Lara |
| S | Laraqui Hossini |
| I | Larbah |
| Antonella | Larcinese |
| A | Laredj |
| Andreas | Larentzakis |
| S | Lario |
| Sandra | Lario Pérez |
| Meghan | Lark |
| J | Larkin |
| JO | Larkin |
| John | Larkin |
| Brett | Larner |
| Christopher J | LaRocca |
| Audrey | Larouche |
| Juan | Larrañaga |
| B | Larsen |
| David | Larson |
| Kelsey E | Larson |
| J | Laryea |
| Inmaculada | Lasa |
| Maria | Laseca |
| Mehran | Lashari |
| Valentina | Lasić |
| Kingsley | Lasing |
| Konstantinos | Lasithiotakis |
| Parbin | Laskar |
| D | Łaski |
| S | Lasrado |
| Luis | Lassaletta |
| Bibiana | Lasses Martínez |
| Manju | Lata Verma |
| I | Lataifeh |
| Edward | Latif |
| Ejaz | Latif |
| Haider | Latif |
| Sehrish | Latif |
| Usman | Latif |
| Stojan | Latinčić |
| Raquel | Latorre Fragua |
| M | Latorre Gómez |
| Raúl | Latorre Tomey |
| Abigail | Lau |
| Godfrey | Lau |
| Jo | Lau |
| Joshua | Lau |
| K | Lau |
| Maisie | Lau |
| Rainbow W H | Lau |
| RW | Lau |
| Annamart | Laubscher |
| Maritz | Laubscher |
| Veronica | Laudani |
| Johanna | Laukkarinen |
| Jheff | Laura |
| S | Laura |
| Sharon | Laura |
| Maria | Laura Cossu |
| Maria | Laura Pelegrina-Lopez |
| Vladimir | Laureano Velasquez Huarcaya |
| E | Laurent |
| R | Laurente |
| A | Lauretta |
| MP | Lauretta |
| Oliver | Lauridsen Siaw |
| B | Lauritz |
| Brianne | Lauritz |
| JC | Lauscher |
| N | Lavagen |
| Nolwenn | Lavagen |
| Laura | Lavalle |
| Andréane | Lavallée |
| Alcimar | lavareda dos Santos Junior Alcimar |
| Vincent | Lavoue |
| R | Lavy |
| Ron | Lavy |
| J | Law |
| Adedayo | Lawal |
| B | Lawal |
| Ishak | Lawal |
| Jamila | Lawal |
| T | Lawal |
| TA | Lawal |
| Ismail | Lawani |
| Ismaïl | Lawani |
| Souliath | Lawani |
| S | Lawday |
| R | Lax Perez |
| Raquel | Lax Perez |
| R | Lax-Pérez |
| H | Layard Horsfall |
| Hugo | Layard Horsfall |
| GR | Layton |
| Engels | Lazala |
| Gabriel | Lazar |
| Kirill | Lazarev |
| Alexander | Lazarides |
| A | Lázaro |
| A | Lazic |
| Aleksandar | Lazic |
| E | Lazova |
| M | Lazovic |
| Mikan | Lazovic |
| Stefano | Lazzari |
| Lan-Hoa | Le |
| P | Le |
| T | Le |
| Louise | Le Blevec |
| Adrien | Le Fouler |
| Hélène | Le Gall |
| S | Le Grange |
| B | Le Roy |
| Khrissa | Lea Elisa Violago |
| C | Leal |
| Clara | Leal |
| Rebeca | Leal |
| I | Leal Silva |
| Inês | Leal Silva |
| Sofia | Leandro |
| Y | Leang |
| Jessica | Leary |
| M | Lebe |
| A | Lechiancole |
| M | Lechner |
| Michael | Lechner |
| WK G | Leclercq |
| Wouter | Leclercq |
| V | Lecluyse |
| K | Lecolle |
| Katia | Lecolle |
| D | Lecumberri |
| David | Lecumberri |
| H | Lederhuber |
| FS | Ledesma |
| Adelaide | Lee |
| Alexandra | Lee |
| Dominic | Lee |
| G | Lee |
| J | Lee |
| Jonathan | Lee |
| Ken | Lee |
| KJ | Lee |
| Lawrence | Lee |
| LD | Lee |
| Lucas D | Lee |
| M | Lee |
| Minna | Lee |
| Rex H | Lee |
| S | Lee |
| Sharon | Lee |
| Shawn | Lee |
| SM | Lee |
| Susan | Lee |
| Vivian | Lee |
| YM | Lee |
| Kurt | Lee Chircop |
| Jeremy | Lee Jun Shern |
| Mary | Leech |
| E | Leede |
| Renee | Leen Laudato |
| Christopher | Leeson |
| S | Leeson |
| R | Lefroy |
| Christian | Legal |
| Adrian | Legaspi |
| Gerardo | Legaspi |
| P | Legeza |
| C | Lehmann |
| Carlos | Lehmann |
| Christian | Leiber |
| James | Leigh |
| AL S | Leite |
| Fernanda | Leite |
| AM | Leite-Moreira |
| Andreas | Leithner |
| A | Leitner |
| Aran | Leitner |
| JM | Lemée |
| F | Lemma |
| Francesco | Lemma |
| Chris | Lemos |
| Madeleine | Lemyre |
| Anna | Lena Huber |
| Catherine | Leng |
| S | Leng |
| M | Lengauer |
| Rebecca | Lenihan |
| P | Lennon |
| Hannah | Lennox-Warburton |
| Cumhur B | Lent Urman |
| Neil | Lenus |
| E | Lenzi |
| Elisa | Lenzi |
| Riccardo | Lenzi |
| CA | Leo |
| B | Leoce |
| Brian | Leoce |
| R | Leon |
| Sergio | Leon |
| ZS | Leon Cabrera |
| Elizabeth | Leon Cuevas |
| Ricardo | León Fernández |
| LF | Leon Giron |
| Eduardo | Leon Llanos |
| Muyenzi | Leon Ngeruka |
| JL | León Palacios |
| MA | Leon Valarezo |
| Gustavo | León Vizcaya |
| Alejandro | Leon-Andrino |
| Carlos | León-Espinoza |
| Vicente J | León-Muñoz |
| Julia | Leonard |
| L | Leonard |
| Laura | Leonard |
| Camillo | Leonardo Bertoglio |
| Lorenzo | Leonelli |
| CH | Leong |
| D | Leong |
| F | Leong |
| M | Leongito |
| Elena | Leonor Delgado-Nieto |
| L | Leotta |
| Jeffrey J | Leow |
| P | Lepiane |
| Dominic | Lepiorz |
| Milan | Lerch |
| M | Lerchenberger |
| Ricardo | Lerma |
| P | Lerut |
| A | Letaief |
| Noelia | Lete Aguirre |
| Ludvig | Letica |
| I | Leto |
| Ahmed | Letrache |
| Andraay | Leung |
| Catherine | Leung |
| Elaine | Leung |
| EY L | Leung |
| Philemon | Leung |
| S | Leung |
| Sebastian | Leuschner |
| Andrea | Leva |
| S | Leventoğlu |
| JH | Levin |
| Meta | Levstek |
| Yael | Levy-Zauberman |
| Chen | Lew |
| PS | Lew |
| Francesca | Lewis |
| Owen | Lewis |
| SE | Lewis |
| C | Lewis-Lloyd |
| Christopher | Lewis-Lloyd |
| R | Lewit |
| Ruth A | Lewit |
| A | Leyte |
| Antonio | Leyte Golpe |
| Francisco | Leyva Rodríguez |
| M | Leziak |
| Sara | Lhassani |
| Demin | Li |
| Lucy | Li |
| R | Li |
| Ryle | Li |
| S | Li |
| Z | Li |
| Zoe | Li |
| Chin | Li Tee |
| MR | Li Valencia |
| T | Liakakos |
| Hsin-Ping | Liang |
| Ina | Liang |
| Kaifeng | Liang |
| Tingbo | Liang |
| Y | Liang |
| J | Liaño |
| Julian | Liaño |
| CC L | Liao |
| SC | Liapis |
| S | Liau |
| Siong-Seng | Liau |
| M | Liberati |
| AS | Liberman |
| Tatiana | Liborio-Kimura |
| L | Licari |
| Leo | Licari |
| Anthony | Lichaa |
| RD | Licona-Meníndez |
| O | Liczbik |
| S | Lidder |
| Surjit | Lidder |
| A | Liddle |
| Samuel | Lie |
| W | Lie |
| Warren | Lie |
| Michael | Liebensteiner |
| T | Liebs |
| Amanda | Liesegang |
| B | Lieske |
| I | Liew |
| Ignatius | Liew |
| JC | Lifante |
| Nicholas | Lightfoot |
| Anne-Louise | Lihn |
| V | Likhvantsev |
| Abu | Lil |
| K | Lilaj |
| Nzabamwita | Liliane |
| J | Lilienstein |
| C | Lillo |
| Cristina | Lillo García |
| Alicia | Lim |
| Chetana | Lim |
| D | Lim |
| Daniel | Lim |
| Eric | Lim |
| IM | Lim |
| Ivy | Lim |
| JA | Lim |
| Jeffery ZK | Lim |
| K | Lim |
| Matthew | Lim |
| Seantee | Lim |
| Y | Lim |
| ALM | Lima |
| Leonardo | Lima |
| I | Lima Buarque |
| Igor | Lima Buarque |
| Leticia | Lima da Cruz |
| Catarina | Lima da Silva |
| Susana | Lima Oliveira |
| C | Lima-da-Silva |
| ZM | Limalia |
| Haruna | Liman |
| RK | Liman |
| A | Lin |
| DJ | Lin |
| JF | Lin |
| Peter | Lin |
| Rosalina | Lin |
| C | Linari |
| Eddy | Lincango |
| EP | Lincango |
| E | Lincango-Naranjo |
| Eddy | Lincango-Naranjo |
| Karin | Lind |
| Andrew | Lindberg |
| J | Lindenmann |
| Joerg | Lindenmann |
| Cortland | Linder |
| G | Linder |
| Gustav | Linder |
| J | Lindert |
| Judith | Lindert |
| Ebba K | Lindqvist |
| EK | Lindqvist |
| Elizabeth | Lindsay |
| Wee | Ling Koh |
| Yii | Ling Lau |
| Eu | Ling Neo |
| Erika | Linmey Tay Lasso |
| Rui | Lino |
| Kenneth | Linton |
| VC | Linz |
| Paris | Liokatis |
| C | Lionel |
| Charre | Lionel |
| Ruggero | Lionetti |
| C | Lipede |
| Christina | Lipede |
| L | Lippa |
| A | Lira dos Santos Leite |
| Amanda | Lira dos Santos Leite |
| Anna | Lisa Pesce |
| Joana | Lisboa |
| Robert | Lischke |
| C | Lisencu |
| Cosmin | Lisencu |
| G | Lisi |
| Giorgio | Lisi |
| A | Litchinko |
| F | Litta |
| Francesco | Litta |
| Max | Little |
| Joe | Littlechild |
| A | Litvin |
| Andrey | Litvin |
| Yauheniya | Litvina |
| Biquan | Liu |
| David | Liu |
| H | Liu |
| L | Liu |
| Shirley | Liu |
| T | Liu |
| A | Liveris |
| Anna | Liveris |
| Marie | Livin |
| Charles | Livingston |
| Lorenzo | Livraghi |
| João | Lixa |
| AS D | Liyanage |
| Pituwala | Liyanage Adithya Sirisena |
| Juan | Liyo |
| María | Liz Sánchez |
| Aintzane | Lizarazu |
| JA | Lizarbe |
| Diana | Lizet Cruz Condori |
| G | Lizzetti |
| Grecia | Lizzetti |
| G | Lizzetti-Mendoza |
| V | Lizzi |
| Vincenzo | Lizzi |
| F | Llahi |
| Florencia | Llahi |
| Eduardo | Llamazares Cobo |
| Alice | Llambias-Maw |
| Tamara | Llamero |
| Oscar | Llanes |
| L | Llano |
| Lionel | Llano |
| H | Llaquet Bayo |
| Heura | Llaquet Bayo |
| CA | Llerena Ojeda |
| Juan | Lliteras Jorge |
| Alyssa | Llorando |
| Almudena | Llorente |
| IC | Lloyd |
| W | Lloyd |
| William | Lloyd |
| T | Lo |
| Terence | Lo |
| Arturo | Lo Giudice |
| Yuri | Loaiza |
| A | Loayza |
| Niklas | Löbig |
| Pablo | Lobos |
| Dmitrijs | Lobovs |
| Andrea | Locatelli |
| LG | Locatello |
| Florian N | Loch |
| FN | Loch |
| Johan | Lock |
| Jessica | Lockhart |
| S | Lodhi |
| S | Lodhia |
| Andrew | Loehrer |
| Markus | Loffler |
| Markus W | Löffler |
| MW | Löffler |
| Chiara | Loffredo |
| N | Löfgren |
| Niklas | Löfgren |
| Anitha | Loganathan |
| K | Logishetty |
| C | Loh |
| Christopher | Loh |
| R | Lohia |
| Rajan | Lohia |
| V | Lohsiriwat |
| Varut | Lohsiriwat |
| Wolfgang | Loidl |
| Evania | Lok |
| Siddharth | Lokanathan |
| Lokesh | Lokesh |
| U | Lokman |
| CP | Lombardi |
| Gaetano | Lombardi |
| Raffaele | Lombardi |
| Cristina | Lombardia Gonzalez de Lera |
| D | Lomiento |
| Daniele | Lomiento |
| Z | Loncar |
| Zlatibor | Loncar |
| German | Londono |
| Eduardo | Londono-Schimmer |
| Rubina | Lone |
| C | Long |
| Emma | Long |
| Samuel | Long |
| M | Longhi |
| A | Longhini |
| Alessandro | Longhini |
| L | Longstaff |
| MW | Löoffler |
| C | Lopes |
| L | Lopes |
| LM | Lopes |
| Luciana | Lopes |
| Ariela | Lopez |
| F | Lopez |
| Fernando | Lopez |
| M | Lopez |
| Manuel | Lopez |
| MP | Lopez |
| MP J | Lopez |
| Raquel | Lopez |
| Angie | López |
| I | López |
| Iker | López |
| María | López |
| Emilio | Lopez Alcina |
| L | Lopez Antoñanzas |
| Leyre | López Antoñanzas |
| Aldo | Lopez Blanco |
| A | López Campillo |
| Adrián | López Campillo |
| A | López De Fernández |
| Alina | López De Fernández |
| CA | López de la Manzanara Cano |
| Clara | López de Lerma Martínez de Carneros |
| Sonia | Lopez Flores |
| Isabel | López García |
| Patricia | Lopez Gomez |
| Ruth | Lopez Gonzalez |
| Pablo | López Martínez |
| I | Lopez Muralles |
| Ismar | Lopez Muralles |
| Maria | Lopez pais |
| Francisco | López Rodríguez -Arias |
| I | López Sánchez |
| Isabel | López Sánchez |
| C | López Viloria |
| M | López-Baamonde |
| Santiago | Lopez-Ben |
| Javier | López-Martin |
| G | Lopez-Pena |
| Gabriel | Lopez-Pena |
| Enrique | López-Ruiz |
| Jaime | López-Sánchez |
| Pilar | López-Toribio López |
| P | Lora-Cumplido |
| J | Lord |
| Julia | Lord |
| Dalila | Loredana Lo Bue |
| Charmagne | Loren Ramos |
| Z | Lorenc |
| M | Lorencin |
| Mia | Lorencin |
| Kerstin | Lorenz |
| Cara | Lorenzi |
| A | Lorinc |
| Fabrizio | Lorusso |
| M | Losada |
| Manuel | Losada |
| E | Lostis |
| E | Lostoridis |
| Eftychios | Lostoridis |
| B | Lotfi |
| N | Lott |
| Natalie | Lott |
| Lawrence | Lottenberg |
| Marco | Lotti |
| Christopher | Lotz |
| Wenhui | Lou |
| Amina | Louari |
| M | Loubani |
| Mahmoud | Loubani |
| VR | Louçao Prada |
| Mauricio | Loucel Bellino |
| S | Louette |
| Stefan | Louette |
| P | Loufopoulos |
| Antony | Louis Rex Michael |
| Sina- | Louisa Patrizia Jentschura |
| Hannah | Louise Morley |
| Petros | Loukas Chalkias |
| SM | Louraoui |
| María | Lourdes Ramos |
| I | Lourenço |
| S | Lourenço |
| Juliana | Lourenço da Silva |
| Carolina | Lourenço Gomes dos Santos |
| L | Loutzidou |
| Lydia | Loutzidou |
| A | Louvrier |
| A | Lovece |
| Andrea | Lovece |
| Amy | Lovett |
| Agbenya | Lovi |
| Maria | Lovisa Jönsson |
| Dana | Low |
| Y | Low |
| Yee | Low |
| AJ | Lowery |
| Aoife | Lowery |
| Megan | Lowey |
| MJ | Lowey |
| A | Lowy |
| Christian | Lozano |
| Santiago | Lozano Calderon |
| P | Lozano Lominchar |
| Pablo | Lozano Lominchar |
| H | Lu |
| Htoo | Lu |
| A | Luberto |
| Gian | Luca Baiocchi |
| Roberto | Luca Meniconi |
| Marie | Lucas |
| Marco | Lucchi |
| SM | Lucchini |
| Stefano | Lucchini |
| Carmen | Lucero León Gámez |
| Ana | Lucia Lemus |
| Ana | Lucia Munhoz Lima |
| Ana | Lucía Portilla |
| Ana | Lúcia Preto Barreira |
| A | Lucianetti |
| C | Luciani |
| Antonio | Luciano Sarni |
| J | Luck |
| Tara | Luck |
| R | Luckwell |
| Rhys | Luckwell |
| J | Lucocq |
| Isabella | Ludbrook |
| Stephanie | Lueckel |
| Laurene | Lugans |
| Ferdinand | Luger |
| Matthias | Luger |
| Gaetano | Luglio |
| Carolina | Lugo Duarte |
| GE | Lugo Zamudio |
| Ignacio | Lugones |
| Sumant | Luhana |
| Stefan | Luhne |
| Pier | Luigi Filosso |
| Emanuele | Luigi Giuseppe Asti |
| M | Luis |
| Angel | Luis Agüero Delgado |
| Juan | Luis Blas Laina |
| Jorge | Luis Bustamante Polo |
| José | Luis Castillo |
| José | Luis DAddino |
| Rene | Luis Filarca |
| Victor | luis Gomez corujo |
| Jorge | Luis Gomez-Mayorga |
| Jose | Luis Lucena De La Poza |
| Jose | Luis Muñoz de Nova |
| Jose | Luis Rabago |
| Jose | Luis Ramos Rodriguez |
| Maria | Luís Sacras |
| Ana | Luis Siles |
| Jose | Luis Uquillas |
| Jorge | Luis Velez Bernal |
| Maris | Luisa García-Pérez |
| Maria | Luisa Gasparri |
| Maria | Luisa Reyes Diaz |
| Matteo | Luisetto |
| Florentina | Luiza Popescu |
| Maria | Luiza Rocha |
| AC O | Luk |
| M | Lukaszewski |
| Claudine | Lukban |
| L | Luke |
| L | Luketic |
| Lea | Luketic |
| Alexandr | Lukianov |
| Dejan | Lukic |
| B | Lukić |
| Lara | Lukman |
| Sumadi | Lukman Anwar |
| L | Lukoko |
| Ausra | Lukosiute-Urboniene |
| I | Luksic |
| Ivica | Luksic |
| Pwaluke | Luku |
| H | Lule |
| Herman | Lule |
| Joann | Lum |
| David | Lumenta |
| DB | Lumenta |
| Olivia | Lumiap Serevina |
| EWY | Lun |
| Joaquin | Luna |
| S | Lunca |
| Sorinel | Lunca |
| R | Lunevicius |
| Raimundas | Lunevicius |
| C | Luney |
| Catriona | Luney |
| EG | Lunghi |
| A | Luo |
| Haili | Luo |
| L | Luo |
| Weisang | Luo |
| X | Luo |
| V | Luoma |
| AI | Lupián-Angulo |
| L | Luques |
| Victor | Luraschi |
| O | Lusawana |
| WG | Lustre |
| Anil | Luther |
| Charlotte | Luths |
| Nicholas | Lutton |
| Martha | Luz Torres |
| A | Luzzi |
| Emilia | Luzzi |
| Jasen | Ly |
| M | Ly |
| Victor | Ly |
| Marie-Louise | Lydrup |
| G | Lye |
| Julie | Lykke Harbjerg |
| P | Lykoudis |
| Holly | Lyle |
| Michelle | Lynch |
| Anders | Lyng Ebbehøj |
| Grace | Lynn Estanislao |
| Louie | Lynn Sajorda |
| Maria | Lyons |
| O | Lyons |
| Oliver | Lyons |
| D | Lytras |
| Alexei | Lyzikov |
| Turki | M Alzaidi |
| Jose | M Barrio |
| Ahmed | M Chaoui |
| Rosa | M Jimenez-Rodriguez |
| Jose | M Jover |
| Mahmoud | M Mohammed |
| S | M Prasad |
| Mashitha | M S |
| Josep | M Sole-Sedeno |
| Damien | M Wu |
| Jolande | Ma |
| Justin | Ma |
| Tao | Ma |
| Vanessa | Ma |
| Sara | Maa Albared |
| R | Maala |
| Ghina | Maarawi |
| A | Maashi |
| Marah | Maayah |
| María | Mabel Collado Expósito |
| RM | Mabeza |
| Waleed | Mabood |
| Findlay | MacAskill |
| T | Maccabe |
| Giuseppe | Maccagnano |
| A | Macchi |
| Alberto | Macchi |
| Roberto | Macchiavello |
| Andrew | MacCormick |
| Hamish | Macdonald |
| L | MacDonald |
| Luisa | MacDonald |
| Taigh | Macdonald |
| Nicole | Macečková |
| Alexandre | Macedo |
| DA | Macedo Falcon |
| P | Macek |
| Petr | Macek |
| A | Machado |
| Daniela | Machado |
| Francisco | Machado |
| Luís | Machado |
| N | Machado |
| Nuno | Machado |
| Vinicius | Machado |
| N | Machairas |
| Nikolaos | Machairas |
| Camila | Machareth |
| V | Machatsch |
| Solomon | Machemedze |
| Marvellous | Machiri |
| J | Maciel |
| Alasdair | MacInnes |
| Emma | MacInnes |
| Dillon | MacIntyre |
| Nicola | Mackay |
| Sean | Mackay |
| TG | Mackay |
| K | MacKenzie |
| S | MacKenzie |
| Shawn | MacKenzie |
| T | MacKinnon |
| Anthony | MacLean |
| Catherine | Macleod-Hall |
| Brooke | Macnab |
| S Danielle | MacNeil |
| Mahmoud | Macshut |
| I | Madabhavi |
| Irappa | Madabhavi |
| S | Madan |
| Omar | Madani |
| R | Madani |
| Saeed | Madani |
| A | Madden |
| Vijay | Madduri |
| I | Made Gede Widnyana |
| Galia | Maderi |
| Devdas | Madhavan |
| TK | Madhuri |
| K | Madhvani |
| Kiran | Madhvani |
| Suyog | Madje |
| Azza | Madkhali |
| T | Madkhali |
| Tariq | Madkhali |
| Abdul | Madni |
| Massimo | Madonia |
| M | Madonini |
| Constanza | Madrid |
| Andrea | Madrigrano |
| Helen | Madsen |
| Doris | Mae Dimatatac |
| Liana | Mae Lobo |
| Junichi | Maeda |
| Y | Maeda |
| Yasuko | Maeda |
| Mohammad | Maen Ghannam |
| Federica | Maffeis |
| A | Maffert |
| Alexis | Maffert |
| A | Maffioli |
| Anna | Maffioli |
| A | Maffuz-Aziz |
| Antonio | Maffuz-Aziz |
| C | Magadán Álvarez |
| Ana | Magalhães |
| M | Magalhães Maia |
| Mariana | Magalhães Maia |
| Catalin | Magan |
| Nasir | Magboul |
| María | Magdalena Vásquez Sánchez |
| Ammar | Magdy |
| C | Magee |
| Astrid | Magele |
| Moses | Magezi |
| G | Maggiore |
| SM | Maggiore |
| Tamara | Maghathe |
| Ashraf | Maghrabi |
| Knut | Magne Augestad |
| Stefano | Magnone |
| V | Mago |
| Abdula | Magomedaliev |
| Dimitrios | Magouliotis |
| D | Magowan |
| Nikko | Magsanoc |
| Barry | Maguire |
| D | Maguire |
| PJ | Maguire |
| R | Maguire |
| N | Mahabbat |
| Nehal | Mahabbat |
| Amit | Mahajan |
| Anupam | Mahajan |
| Dhruv | Mahajan |
| C | Mahakalkar |
| Chandrashekhar | Mahakalkar |
| Praveena | Mahalingam |
| Talanayar | Mahalingam |
| Jehangir | Mahaluxmivala |
| Ishtiak | Mahamud |
| Subramanyam | Mahankali |
| AK | Mahar |
| H | Mahdi |
| Maryam | Mahdi |
| Shareef | Mahdi |
| Ameerah | Mahdi Abraheem Hasan |
| B | Mahendran |
| V | Mahendran |
| M | Maher |
| Mark | Maher |
| Natalie | Maher |
| M | Maher Al arje |
| Sarah | Maheux-Lacroix |
| Moufid | Mahfoud |
| Abobakr | Mahfouz |
| Arwa | Mahfouz |
| Mehmet | Mahir Ozmen |
| Ansar | Mahmood |
| Arif | Mahmood |
| Ashraf | Mahmood |
| DN | Mahmood |
| Farrukh | Mahmood |
| Namrah | Mahmood |
| S | Mahmood |
| U | Mahmood |
| Usama | Mahmood |
| Z | Mahmood |
| A | Mahmoud |
| Ahmed | Mahmoud |
| Fedy | Mahmoud |
| Nada | Mahmoud |
| Osman | Mahmoud |
| Safa | Mahmoud |
| Y | Mahmoud |
| Alaa | Mahmoud Abo shabana |
| Balqees | Mahmoud Al-Manaseer |
| F | Mahmoud Ali |
| Ayesha | Mahmud |
| Zayne | Mahmud-Ahmad |
| Taher | Mahnashi |
| Ebrahim | Mahomed |
| Wasim | Mahomed |
| J | Mahon |
| Freyia | Mahon-Daly |
| R | Mahoney |
| Wesam | Mahran |
| Doha | Mahrous |
| Mert | Mahsuni Sevinc |
| K | Mahuli |
| DA | Mahvi |
| David | Mahvi |
| P | Maida |
| Pietro | Maida |
| F | Maiello |
| A | Maiga |
| JM | Maillet |
| Betty | Maillot |
| Claire | Mailu |
| Mayaba | Maimbo |
| Miriam | Maimbo |
| B | Main |
| Grace | Maina |
| J | Maines |
| F | Maione |
| Pasquale | Maiorano |
| Charikleia | Maiou |
| M | Mair |
| Montserrat | Mairal Fraile |
| Siobhan | Mairead Rooney |
| Patrick | Maison |
| E | Maisonneuve |
| Emeline | Maisonneuve |
| A | Maity |
| Vincenzo | Maiuri |
| M | Maiza |
| Omar | Majadla |
| AM | Majbar |
| MA | Majbar |
| Faizan | Majeed |
| Zubair | Majeed |
| Suvendu | Maji |
| I | Majid |
| Ibrar | Majid |
| Sabeen | Majid |
| A | Majkowska |
| Agata | Majkowska |
| L | Majkowski |
| Hardil | Majmudar |
| P | Major |
| Piotr | Major |
| S | Majrashi |
| Brian | Mak |
| JK C | Mak |
| Josephine | Mak |
| Baje | Makama |
| Ronald | Makanda |
| Ayomide | Makanjuola |
| Fabiana | Makdissi |
| FB | Makdissi |
| C | Makepeace |
| R | Makin-Taylor |
| Jun | Makino |
| Raghad | Makki |
| Abdelrahman M | Makram |
| Amany | Makroum |
| Bogdan | Maksymenko |
| Florian | Maksymiw |
| K | Maktabi |
| CC | Makwe |
| Christian | Makwe |
| A | Malan |
| Asha | Malan |
| H | Malapati |
| Harsha | Malapati |
| O | Malard |
| M | Malavolta |
| FL | Malcolm |
| FH R | Maldonado |
| A | Maldonado Del Arenal |
| Meredid | Maldonado Santiago |
| Yeray | Maldonado Sotoca |
| Eloy | Maldonado-Marcos |
| Y | Maldonado-Sotoca |
| Almantas | Maleckas |
| Ans | Malek |
| M | Malerba |
| Michele | Malerba |
| Albert | Malet Contreras |
| Leeany | Maletta Francisco |
| Igor | Maleyko |
| Maulen | Malgazhdarov |
| B | Malgras |
| Brice | Malgras |
| Rajesh | Malhotra |
| N | Malibary |
| Nadim | Malibary |
| Ziad | Malibary |
| Aamer | Malik |
| AR | Malik |
| Boulaadas | Malik |
| Kapil | Malik |
| Kiren | Malik |
| Komail | Malik |
| Mariam | Malik |
| MR | Malik |
| MS | Malik |
| Shahbaz S | Malik |
| Sobia | Malik |
| Tahir | Malik |
| Lucy | Maling |
| Konstantinos | Malizos |
| J | Mall |
| G | Mallabiabarrena Ormaechea |
| G | Malleo |
| Shweta | Mallick |
| Evangelos | Mallidis |
| C | Mallmann |
| Christoph | Mallmann |
| Michael R | Mallmann |
| MR | Mallmann |
| J | Malmstedt |
| A | Malomo |
| F | Malone |
| Fergal | Malone |
| Gianni | Malossini |
| A | Malpaga |
| Julius | Malte Vahl |
| G | Maltese |
| G | Maltinti |
| Gherardo | Maltinti |
| Luca | Malvezzi |
| S | Mambrilla |
| Sara | Mambrilla |
| Nora | Mamdouh |
| Zaman | Mamedli |
| M | Mamic |
| Matija | Mamic |
| Rakotonaivo | Mamisoa Judicaël |
| Mickyas | Mamo |
| A | Mamopoulos |
| Apostolos | Mamopoulos |
| Sidi | Mamoun Louraoui |
| Chi | Man Tom Chow |
| Enrique | Manalang |
| Mallikarjuna | Manangi |
| J | Manara |
| Dimitrios K | Manatakis |
| DK | Manatakis |
| G | Manca |
| Gilles | Manceau |
| G | Mancebo |
| Gemma | Mancebo |
| MM | Manchego De La Cruz |
| Marta | Mancheño |
| R | Mancini |
| S | Mancini |
| Roberto | Mancino |
| Valentina | Manciocco |
| VD | Mandato |
| V | Mandic Markovic |
| Facundo | Mandojana |
| K | Mandrelle |
| Kavita | Mandrelle |
| RP | Manecksha |
| Rustom | Manecksha |
| Steffen | Manekeller |
| S | Manfredelli |
| Alfonso | Manfuso |
| Ahmed | Mangahy |
| MS | Mangano |
| Aneish | Mangarai |
| V | Mangaroliya |
| Moses | Mangena |
| Ali | Mangi |
| S | Manglik |
| E | Mangos |
| J | Mangwani |
| Jitendra | Mangwani |
| J | Manickavasagam |
| Jaiganesh | Manickavasagam |
| M | Manigrasso |
| P | Manikis |
| N | Manimaran |
| EA | Manioti |
| P | Maniscalco |
| Pietro | Maniscalco |
| Saptak | Mankad |
| R | Manley |
| H | Mann |
| Harvinder | Mann |
| A | Mannan |
| S | Mannan |
| Syed | Mannan |
| Aidan | Manning |
| D | Manning |
| Debra | Manning |
| R | Mannion |
| David | Männle |
| I | Mannoh |
| Ivy | Mannoh |
| T | Manogaran |
| L | Manoj Joshua |
| Lokavarapu | Manoj joshua |
| Veronica | Manolache |
| I | Manolitsis |
| F | Manresa-Manresa |
| Francisco | Manresa-Manresa |
| E | Manrique |
| Susana | Manrique |
| GC | Manrique Sila |
| P | Mansilla Doria |
| Percy | Mansilla Doria |
| MI | Manso |
| D | Manson |
| J | Manson |
| Joanna | Manson |
| Maheen | Mansoor |
| Aisha | Mansoor Ali |
| A | Mansour |
| Ahmed | Mansour |
| Atef | Mansour |
| F | Mansour |
| Omar | Mansour |
| Nayef | Mansour Alshammari |
| J | Mansouri |
| M | Mansouri |
| A | Mansuri |
| Ahmed | Mansy |
| A | Mantevas |
| Antonis | Mantevas |
| Henrique | Mantoan |
| B | Mantoglu |
| Baris | Mantoglu |
| J | Mantri |
| N | Manu |
| Nichola | Manu |
| Meshach | Manu Agyapong |
| Juan | Manuel Baez Melgarejo |
| Vicente | Manuel Borrego Estella |
| Jiomar | Manuel Figueroa Germosen |
| Jose | Manuel Luna Vazquez |
| Florencio | Manuel Marin Martinez |
| Juan | Manuel Martos Martinez |
| José | Manuel Morales-Puebla |
| José | Manuel Muñoz Camarena |
| Joaquin | Manuel Muñoz Rodríguez |
| Jose | Manuel Rabanal |
| H | Many |
| Lisa | Manzenreiter |
| Elena | Manzo |
| S | Manzoor |
| Shahneela | Manzoor |
| Sobia | Manzoor |
| Partson | Maphosa |
| Natalie | Maple |
| Sabreen | Maqbol |
| F | Maqboul |
| A | Maqsood |
| Fauzia | Maqsood |
| Rocio | Maqueda |
| R | Maqueda González |
| A | Maqus |
| C | Marafante |
| Chiara | Marafante |
| A | Marano |
| Alessandra | Marano |
| L | Marano |
| Luigi | Marano |
| Salvatore | Marano |
| Dimitris | Maras |
| André | Marçal |
| Brenda | Marcela Coll Tello |
| Josie | Marcelle Lira Albuquerque |
| Juan | Marcelo Delgado Godoy |
| Juan | Marcelo Portillo |
| A | Marchbank |
| Benedikt | Marche |
| G | Marchegiani |
| Paolo | Marchesi |
| Claudio | Marchetti |
| V | Marchionini |
| Valentina | Marchionini |
| Arnold | Marchis |
| Alfonso | Marco Garrido |
| Gian | Marco Prucher |
| A | Marco-Garrido |
| S | Marcos Contreras |
| Sergio | Marcos Contreras |
| P | Marcos-Santos |
| Pablo | Marcos-Santos |
| H | Marcus |
| Hani | Marcus |
| HJ | Marcus |
| M | Mardare |
| Mara | Mardare |
| Hiba | Mardini |
| MMA | Marei |
| A | Marello |
| P | Maremonti |
| Pietro | Maremonti |
| Gianluca | Maresca |
| A | Margalit |
| Adam | Margalit |
| Nevo | Margalit |
| Concetta | Marganella |
| Ana | Margarida Cinza |
| Ana | Margarida Correia |
| Lavinia | Margarit |
| Chrysoula | Margioula-Siarkou |
| Joseph | Margolick |
| G | Mari |
| Syeda | Maria Ahmad Zaidi |
| Marta | María Arroyo Domingo |
| Anna | Maria Baietti |
| Jose | Maria Barreto Angulo |
| Monica | Maria Bejasa |
| Francesco | Maria Carrano |
| Luz | Maria Cespedes Ramirex |
| Pierfranco | Maria Cicerchia |
| Davide | Maria Donati |
| Virginia | María Durán Muñoz-Cruzado |
| Sandra | Maria Gadin-Lopez |
| José | María Gallego Sánchez |
| José | María García Pérez |
| Agustín | Maria García-Mansilla |
| Angela | Maria Giraldo Velasquez |
| Monica | Maria Gomes-da-Silva |
| Ana | María Grande-Gil |
| Ursula | María Jariod Ferrer |
| Pietro | Maria Lombardi |
| Jose | Maria Lopesino |
| Carmen | maria Lopez Lopez |
| Margarita | Maria Maldonado |
| Tommaso | Maria Manzia |
| Ana | Maria Marin Gonzalez |
| Jose | Maria Martinez-de-la-Casa |
| José | María Martínez-Gómiz |
| Stefano | Maria Massimiliano Basso |
| José | María Matilla |
| Vincenzo | Maria Mazzaferro |
| Luis | María Merino Peñacoba |
| Ana | Maria Minaya Bravo |
| Luz | María Moratalla Charcos |
| Jose | Maria Muguerza |
| Josep | Maria Muñoz Vives |
| Carlo | Maria Neri |
| Jose | Maria Nieto Rodriguez |
| Anna | Maria Ntziovara |
| Alma | Maria Ojeda Rojas |
| Ana | Maria Parada Rodriguez |
| Marco | Maria Pascale |
| Giacomo | Maria Pirola |
| Silvana | Maria Quintana |
| Francesco | maria Romano |
| Thilo | Maria Schulte |
| Jose | Maria Silva Barandiaran |
| Ana | Maria Simono Charadan |
| Lina | María Trujillo |
| Iliana | Maria Valdes-Duque |
| Ana | María Vargas Patiño |
| Lina | Maria Vergara Galliadi |
| Lina | Maria Villegas |
| J | Marialva |
| Joana | Marialva |
| Laura | Mariangela Castellano |
| Alessandro | Mariani |
| NM | Mariani |
| P | Mariani-Kurdjian |
| Kalyani | Mariapan |
| Leo | Maric |
| M | Maric |
| Marjan | Maric |
| Maximiliano | Maricic |
| Hatem | Marie |
| Joan | Marie Flor |
| Alphonse | Marie Sibomana |
| Lysa | Marie-Macron |
| Anna | Mariel Torio |
| Melissa | Marien |
| Gabriel | Marin |
| H | Marin |
| Patrick | Marin |
| C | Marín |
| H | Marín |
| Héctor | Marín |
| Severiano | Marin Bertolin |
| Luis | Marin de Amesti |
| Glenda | Marina Falcon Pacheco |
| Hajamihamina | Marina Parfaite Randriantsoa |
| Sotirios | Marinakis |
| Srdjan | Marinković |
| F | Marino |
| Fabio | Marino |
| MV | Marino |
| Ivan | Mariño |
| L | Marino Cosentino |
| Luigi | Marino Cosentino |
| Daniel | Mario Chircop |
| Antonio | Mario Scanu |
| Herjean | Marion |
| Giovanni | Mariscalco |
| Elva | Marita Sarte |
| Puscas | Marius-Emil |
| Marcel | Marjanović Kavanagh |
| Fares | Marji |
| Vasanth | Mark Samuel |
| Ormond | Mark Taylor |
| D | Markaryan |
| Daniil | Markaryan |
| Naveed | Markhand |
| G | Markose |
| Pavel | Markov |
| Duska | Markov-Glavas |
| Ivan | Markovic |
| Velimir | Markovic |
| Frane | Markulić |
| Nici | Markus Dreger |
| Cynthia | Marlene Rodríguez Sosa |
| Derek | Marlor |
| Neale | Marlow |
| W | Marlow |
| Marilena | Marmiere |
| Elizabeth | Marmol |
| Urska | Marolt |
| G | Marom |
| Gad | Marom |
| Georgia | Maroske |
| Elena | Marotta |
| Ioannis | Maroulis |
| M | Marques |
| N | Marques |
| Narimã | Marques |
| Prescillia | Marques |
| Rita | Marques |
| TM D M | Marques |
| Tomas | Marques |
| M | Marqueta De Salas |
| Maria | Marqueta De Salas |
| L | Marquez |
| Lucila | Marquez |
| Ruth | Marquina González |
| AA | Marra |
| E | Marra |
| Ester | Marra |
| E | Marrano |
| A | Marreiro |
| M | Marro |
| Matteo | Marro |
| Miguel-Angel | Marroquin-Alpirez |
| G | Marruzzo |
| Giovanni | Marruzzo |
| Imke | Marsch |
| C | Marsh |
| Calista | Marshall |
| Rachel | Marshall-Roberts |
| Matthew | Marshall-Webb |
| Abadeer | Marsis |
| Ben | Marson |
| Ella J | Marson |
| FAL | Marson |
| Maria | Marta Modolo |
| G | Marte |
| Gianpaolo | Marte |
| Andre | Martel |
| Sophie | Martellotto |
| Jacopo | Martellucci |
| M | Martens |
| T | Martens |
| C | Marti |
| Rosa | Martí Fernández |
| A | Martin |
| Alexander | Martin |
| B | Martin |
| Benjamin | Martin |
| E | Martin |
| Emmeline | Martin |
| G | Martin |
| J | Martin |
| Janet | Martin |
| L | Martin |
| Louis | Martin |
| M | Martin |
| Mercedes | Martin |
| Niels | Martin |
| RC G | Martin |
| Rhona | Martin |
| S | Martin |
| Sean T | Martin |
| Sergio | Martin |
| Silvia | Martin |
| Fernández | Martín |
| EJ | Martin Antona |
| Belén | Martin Arnau |
| JC | Martín del Olmo |
| Pablo | Martín García |
| Elio | Martín Gutiérrez |
| Sergio | Martin Lucchini |
| Javier | Martín Monterrubio |
| P | Martin Playa |
| Pilar | Martín Rodrigo |
| Luis | Martín Rodríguez Ortegón |
| MB | Martín Salamanca |
| M | Martín Sánchez |
| O | Martin Sole |
| Oriol | Martin Sole |
| L | Martin-Albo Caballero |
| Cristobalina | Martin-Garcia |
| R | Martín-Láez |
| Rubén | Martín-Láez |
| E | Martin-Perez |
| Elena | Martin-Perez |
| O | Martin-Sole |
| L | Martinek |
| Lubomir | Martinek |
| F | Martinelli |
| Fabio | Martinelli |
| G | Martines |
| Gennaro | Martines |
| Alejandra | Martinez |
| L | Martinez |
| Laura | Martinez |
| MJ | Martinez |
| A | Martínez |
| Francisco | Martínez |
| MJ | Martínez |
| Walter | Martínez |
| JA | Martínez Alonso |
| Sara | Martínez Castro |
| A | Martínez de Aragón |
| Charo | Martínez García |
| Jairo | Martinez Garrido |
| Yaiza | Martinez Lahoz |
| MJ | Martinez Lara |
| Fernando | Martinez lascano |
| Teresa | Martínez Marivela |
| CM | Martinez Moreno |
| Gonzalo | Martinez Municio |
| Victoria | Martínez Muñoz |
| P | Martinez Pascual |
| Paula | Martinez Pascual |
| C | Martínez Pérez |
| L | Martinez Perez Maldonado |
| MJ | Martinez Velázquez |
| J J | Martínez Zarate |
| JA | Martínez-Alonso |
| AM | Martinez-Blanco |
| Lucía | Martínez-Costa |
| A | Martinez-German |
| JM | Martínez-Gómiz |
| E | Martínez-Hurtado |
| Sara | Martínez-Núñez |
| Irene | Martínez-Padilla |
| Carolina | Martinez-Perez |
| C | Martínez-Pinedo |
| Carlos | Martínez-Pinedo |
| Hector | Martinez-Said |
| Emmanuel | Martinod |
| Vlatka | Martinovic |
| Bardi | Martins |
| D | Martins |
| Daniela | Martins |
| Paulo | Martins |
| PN | Martins |
| R | Martins |
| Rita | Martins |
| RS | Martins |
| Ruben | Martins |
| G | Martins dos Santos |
| Gildasio | Martins dos Santos |
| Daniel | Martins Jordão |
| Mafalda | Martins Sousa |
| A | Marton |
| P | Martorell |
| Edgar | Martos |
| Nahom | Maru |
| Diana | Marujo |
| D | Marujo Henriques |
| Jeremy | Marume |
| H | Marwan |
| Hisham | Marwan |
| W | Marx |
| William | Marx |
| G | Maryan |
| F | Marzi |
| Maini | Marzia Isabella |
| Natalie | Marzouqa |
| C | Mas |
| R | Mas Melendez |
| Robinson | Mas Melendez |
| Mohammad | Masaarane |
| Shah | Masabat Saleem |
| Nawar | Masarani |
| Mona | masaud Amro Alazabi |
| A | Masciandaro |
| Antonio | Masciandaro |
| Fabrizio | Masciello |
| S | Masdoos |
| H | Mase |
| Michele | Masetti |
| Javeria | Mashal |
| Sarah | Mashaly |
| A | Mashat |
| H | Mashbari |
| Hassan | Mashbari |
| D | Masheka |
| Yehia | Mashhadany |
| L | Mashhadi |
| Mohamed | Mashhour |
| Sonia | Masih |
| Namrata | Maskara |
| Sushil | Maslekar |
| MS | Masood |
| Rumaisa | Masood |
| Khalid | Masood Gondal |
| M | Maspero |
| Joseph | Masri |
| Noor | Masri |
| R | Masri |
| Ruqaya | Masri |
| M | Masrur |
| Anas | Massad |
| C | Massaguer |
| Clara | Massaguer |
| Marco | Massani |
| Dimitrios | Massaras |
| Olindo | Massarelli |
| Simonetta | Massaron |
| M | Masse |
| P | Masseria |
| Benjamin | Massey |
| L | Massey |
| Lisa | Massey |
| E | Massie |
| A | Massobrio |
| Andrea | Massobrio |
| J | Massoud |
| Erfan | Massri |
| Domenico | Massullo |
| L | Masterson |
| Liam | Masterson |
| Aikaterini | Mastoraki |
| S | Mastoridis |
| Sotiris | Mastoridis |
| M | Mastrangelo |
| Francesco | Mastriale |
| F | Mastrilli |
| V | Mastrofilippo |
| Valentina | Mastrofilippo |
| Gustavo | Mastroianni |
| R | Mastroianni |
| Riccardo | Mastroianni |
| Manuela | Mastronardi |
| A | Mastrosimone |
| Alexandra | Mata |
| J | Mata |
| Javier | Mata |
| Juan | Mata Gutierrez |
| Anil | Matai |
| Luka | Matak |
| Oksana | Matanova |
| Maisoon | Matareed |
| Paloma | Maté Mate |
| Sergiu | Matei |
| Mirian | Mateo De La Cruz |
| O | Mateo-Sierra |
| Olga | Mateo-Sierra |
| Elvira | Mateos Alvarez |
| Blanca | Mateos-Serrano |
| Nikhil | Math |
| Erwin | Mathew |
| John | Mathew |
| Ryan | Mathew |
| S | Mathew |
| Stanley | Mathew |
| Shefin | Mathews |
| Neil | Mathias |
| D | Mathieu |
| G | Mathieu |
| P | Mathieu |
| Mariana | Matias |
| B | Matías-García |
| Belen | Matías-García |
| Slavko | Matić |
| Christie | Mato |
| P | Mato |
| David | Mato Mañas |
| Maxime | Maton |
| Leandro | Matos |
| LL | Matos |
| P | Matos Costa |
| Paulo | Matos Costa |
| Faisel | Matoug |
| Petr | Matousek |
| Paul | Matovu |
| Derek | Matsika |
| Fedra | Matsouka |
| Shuko | Matsuda |
| Ryota | Matsuki |
| Yuka | Matsuki |
| Hironori | Matsumoto |
| Takashi | Matsumoto |
| Kiichiro | Matsumura |
| Susumu | Matsushime |
| Daniele | Matta |
| Kavitha | Mattam |
| Ahmed | Mattar |
| Rafif | Mattar |
| Ilaria | Mattavelli |
| L | Mattei |
| Gianluca | Matteo Sampietro |
| Jacopo | Matteucci |
| Catherine | Mattevi |
| KT | Matthew Seah |
| W | Matthews |
| S | Matthiess |
| Gioacchino | Mattisi |
| Jordan | Mattson |
| M | Matuszczak |
| M | Matute-Najarro |
| Julie | Mauger |
| Rebecca | Maunsell |
| Amelie | Maurel |
| LR | Maurer |
| Andrew | Maurice |
| Sandra | Maurício |
| Edgar | Mauricio Barrios Vidales |
| William | Mauricio Riveros Castillo |
| Javier | Mauricio Salgado Tovar |
| David | Mauricio Solano Varela |
| José | Mauro dos Santos |
| Eleni | Mavrodimitraki |
| A | Maw |
| Proud | Mawere |
| M | Mawlichanów |
| Ludwig | Maximilian Heindl |
| Alexander | Maximiliano Martinez-Blanco |
| Fraser | Maxwell |
| R | May |
| Amanda | May Ong Vaño |
| H | Maye |
| Erik | Mayer |
| Franz | Mayer |
| Robert | Mayer |
| J | Mayes |
| Julio | Mayol |
| M | Mayombo Idiata |
| Michael | Mayombo Idiata |
| Thomas | Mayr |
| K | Mayson |
| Kelly | Mayson |
| Tagleb | Mazahreh |
| D | Mazingi |
| Dennis | Mazingi |
| Muhammad | Mazketly |
| Eshan | Mazumdar |
| CA | Mazuret Sepulveda |
| V | Mazzaferro |
| Diego | Mazzatenta |
| Michael | Mazzeffi |
| Carmelo | Mazzeo |
| F | Mazzola |
| Francesco | Mazzola |
| Michele | Mazzola |
| Erica | Mazzotta |
| F | Mazzotti |
| Ndubuisi | Mbajiekwe |
| Ronald | Mbiine |
| B | Mbwele |
| Andrea | Mc Carthy |
| Santiago | Mc Loughlin |
| Stephen | McAleer |
| Ian | McAllister |
| Peter | McAnena |
| B | McAree |
| G | McCabe |
| Olivia | McCabe-Robinson |
| C | McCaffer |
| A | McCanny |
| Damian | McCartan |
| C | McCarthy |
| Claire | McCarthy |
| L | McCarthy |
| Conor | McCartney |
| Katie | McCaughey |
| J | McCaul |
| Craig D | McClain |
| Adam | McClean |
| Anissa | McClelland |
| S | McCluney |
| P | Mccormick |
| W | McCormick |
| Wendy | McCormick |
| A | McCranie |
| S | McCrindle |
| Sarah | McCrindle |
| Peter | McCullough |
| Rebekah | McCullough |
| E | McDermott |
| Enda W | McDermott |
| F | McDermott |
| FD | McDermott |
| Frank | McDermott |
| C | McDonald |
| Sophie | McDonald |
| Brendan | McDonnell |
| Jack | McDonogh |
| K | McElhinney |
| Kathryn | McElhinney |
| Kevin | McElvanna |
| K | McEvoy |
| R | McEwen |
| Amanda | McFarlan |
| Jacob | McGee |
| R | McGee |
| Richard | McGee |
| K | McGivern |
| Connor | McGladdery |
| John | McGrath |
| JS | McGrath |
| N | McGrath |
| Michael | McGreevy |
| R | McGregor |
| Richard | McGregor |
| Cieran | McGrory |
| L | Mcguigan |
| Merrill | McHoney |
| Elysse | Mcilwain |
| Thomas | McIntire |
| Nick | McIntosh |
| Stuart | McIntosh |
| J | McIntyre |
| Joshua | McIntyre |
| RC | McIntyre Jr |
| Robert | McIntyre Jr |
| G | McKay |
| J | McKay |
| Joanne | McKay |
| Joseph | McKay |
| Siobhan | McKay |
| Mark | McKeever |
| James | McKelvie |
| K | McKenzie |
| KL | McKevitt |
| R | McKinney |
| Rachel | McKinney |
| Chris | McKinnon |
| C | Mclaren |
| N | McLarty |
| Jared | Mclauchlan |
| Nicole | McLaughlin |
| Kenneth A | Mclean |
| Morag | McLellan |
| Thomas | McLelland |
| S | McLennan |
| Lucy | McLeod |
| M | McLeod |
| Robert | Mcleod |
| J | McMahon |
| Sam | McNally |
| Anna | McNamara |
| John | McNamara |
| Áine | McNamee |
| C | McNaught |
| John | McNelis |
| P | McNelis |
| Catherine | McNestry |
| F | McNicol |
| Rebecca | McNicol |
| R | Mcnulty |
| D | McPartland |
| I | McPherson |
| Michael | McTague |
| Christina | McVeay |
| J | McVeigh |
| N | Md Din |
| Norshamsiah | Md Din |
| Azmi | Md Nor |
| A | Meagher |
| JG | Meara |
| John G | Meara |
| Eneyew | Mebratu |
| Mostafa | Medhat Fahmy Fahmy |
| Mark | Medhat Mikhail |
| Pourya | Medhati |
| Esther | Medina |
| Monica | Medina |
| L | Medina Mora |
| Manuel | Medina Pedrique |
| Heriberto | Medina-Franco |
| E | Medina-Manuel |
| S | Mediratta |
| Yuliya | Medkova |
| M | Medone |
| Paloma | Medrano |
| R | Medrano Caviedes |
| A | Meelad |
| Ayman | Meelad |
| Rainer | Meffert |
| Diane | Mege |
| Abebe | Megersa |
| S | Meghji |
| S | Megna |
| Stefano | Megna |
| Robert | Meguid |
| M | Mehdi |
| Mohammad | Mehdi |
| Mohammad | Mehdizadeh |
| BJ | Mehigan |
| A | Mehmood |
| DM | Mehmood |
| Manzer | Mehmood |
| Maria | Mehmood |
| G | Mehra |
| Hassan | Mehrad-Majd |
| Asif | Mehraj |
| Seema | Mehrotra |
| A | Mehta |
| D | Mehta |
| Sachin | Mehta |
| A | Meidany |
| EM | Meima - van Praag |
| EM | Meima-van Praag |
| Nikolaus | Meindl |
| Cornelia | Meisel |
| P | Meister |
| Vivek | Meiyappan |
| Arnaud | Mejean |
| Dolores | Mejía |
| Ana | Mejía Casado |
| D | Mejia De la Cruz |
| Dolores | Mejia De la Cruz |
| D | Mekango |
| Margaux | Mekann Bouv-Hez |
| Alem | Mekete |
| M | Mekhael |
| Mira | Mekhael |
| Haile | Mekuria |
| Polina | Melashenko |
| I | Melchor Corcóstegui |
| J | Melchor-Ruan |
| Javier | Melchor-Ruan |
| S | Mele |
| Simone | Mele |
| H | Meleiro |
| Hugo | Meleiro |
| M | Melendez |
| Antonio | Melero Abellán |
| L | Melero-Cortés |
| Lemi | Melese |
| P | Melgar Muñoz |
| Sara | Melgarejo |
| Sara | Melhem |
| G | Melina |
| Shannon | Melissa Chan |
| Ana | Melissa Hilvano-Cabungcal |
| Karla | Melissa Marchan Palma |
| Nensi | Melissa Ruzgar |
| Solomon | Melkamu |
| Andrea | Melloni |
| C | Mellor |
| K | Mellor |
| Christopher | Melnic |
| Ana | Melo |
| Isabelle | Melo da Camara |
| Silvio | Melo torres |
| Gianfranco | Meloni |
| Charlotte | Melot |
| R | Memba Ikuga |
| E | Memişoğlu |
| AS | Memon |
| K | Memon |
| N | Memos |
| Nikolaos | Memos |
| Javier | Mena |
| Jimmy | Mena |
| CA | Mena García |
| MD | Mena Ramirez |
| C | Menakaya |
| Thomas | Mendel |
| Cláudia | Mendes |
| Filipa | Mendes |
| JM | Mendes |
| Margarida | Mendes |
| Manuel | Mendez |
| G | Mendinhos |
| G | Mendiola |
| GC | Mendiola |
| Gian | Mendiola |
| G | Mendiola Barrios |
| Nishantha | Mendis |
| R | Menditto |
| G | Mendonça Ataíde Gomes |
| Gustavo | Mendonça Ataíde Gomes |
| Efrain | Mendoza |
| J | Mendoza Quevedo |
| F | Mendoza-Moreno |
| Fernando | Mendoza-Moreno |
| Fabrice | Menegaux |
| Adam | Meneghetti |
| S | Meneghini |
| Simona | Meneghini |
| Francesco | Menegon Tasselli |
| AR | Menendez Mite |
| Alejandro | Menéndez Moreno |
| A | Meneses-Garcia |
| Hao | Meng Yip |
| M | Mengesha |
| Mengistu G | Mengesha |
| MG | Mengesha |
| Workineh | Mengesha |
| Lucas | Mengíbar |
| Alemneh | Mengist |
| Netsanet | Mengiste |
| Abeje | Menjeta |
| Abel | Menkir |
| C | Menna |
| Cecilia | Menna |
| A | Menon |
| G | Menon |
| PR | Menon |
| Tomas | Menovsky |
| Philip | Mensah |
| Samuel | Mensah |
| Antonio | Meola |
| E | Merashka |
| Eunice | Mercado |
| Jhomayri | Mercado |
| MA | Mercado |
| Pedro | Mercado |
| G | Mercante |
| Giuseppe | Mercante |
| P | Mercantini |
| Maria | Mercedes Caubet |
| Abel | Merchan |
| Irfan | Merchant |
| Richard | Merchant |
| A | Merdrignac |
| Aude | Merdrignac |
| Leila | Mereles Noguera |
| Liliana | Mereu |
| R | Merh |
| S | Meric |
| S | Meriç |
| Serhat | Meriç |
| J | Merkle |
| Janica | Merkle |
| Katheryne | Merlos Garcia |
| S | Merola |
| M | Merrakos |
| N | Merrett |
| Tamas | Mersich |
| Tamás | Mersich |
| O | Merzlikin |
| Mohammed | Meselhy |
| JA | Mesias Logroño |
| SN | Mesli |
| Nouredin | Messaoudi |
| Gianfranco | Messina |
| Alex | Messner |
| F | Messner |
| Franka | Messner |
| YY | Metaferia |
| Symeon | Metallidis |
| Panagiotis | Metaxas |
| Gabriel | Metcalf-Cuenca |
| M | Metro |
| M | Metwalli |
| Maram | Metwalli |
| IH | Metwally |
| Khaled | Metwally |
| J | Metzger |
| Jürg | Metzger |
| Marcus | Meusel |
| Gwenaël | Mevel |
| Patrick | Meybohm |
| B | Meyer |
| Bernhard | Meyer |
| F | Meyer |
| Frank | Meyer |
| Inna | Meyer |
| Lucy | Meyer |
| C | Meyhoff |
| Christian | Meyhoff |
| MA | Meza Fonseca |
| M | Meziane |
| Rameshwar | Mhamane |
| Sang | mi Lee |
| A | Mian |
| Iftikhar | Mian |
| G | Micha |
| Georgia | Micha |
| Afieharo | Michael |
| ALR | Michael |
| S | Michael |
| Vishal | Michael |
| John | Michael DiBianco |
| John | Michael Ranson |
| D | Michailidou |
| N | Michalopoulos |
| Nikolaos | Michalopoulos |
| CW | Michalski |
| Eden | Micheal Ssettabi |
| Christodoulou | Michel |
| M | Michel |
| Maria | Michela Di NUZZO |
| Stefano | Michelagnoli |
| Alessandro | Michele Bonomi |
| B | Michelitsch |
| Birgit | Michelitsch |
| Andrea | Michelle Lowey Medina |
| Taku | Michiura |
| S | Michling |
| D | Micic |
| Dusan | Micic |
| Andrew | Middleton |
| SB | Middleton |
| R | Midha |
| Rajesh | Midha |
| Oleg | Midlenko |
| P | Midrio |
| Tsutomu | Mieda |
| J | Mielke |
| Sven | Mieog |
| M | Mietła |
| Christopher | Mifsud |
| Mohammed | Miftah |
| M | Migliore |
| Marco | Migliore |
| Federico | Migliorelli |
| E | Migliorino |
| G | Mignot |
| Toledano | Miguel |
| Francisco | Miguel González Valverde |
| José | Miguel Izquierdo |
| Gustavo | Miguel Machain V |
| Luis | Miguel Martinez Parra |
| Santiago | Miguel Mata-Suarez |
| Juan | Miguel Roberto Delgado |
| Jose | Miguel Villacampa Auba |
| Jose | Miguel Zaragozá García |
| L | Miguelena |
| Radu | Mihail Mirica |
| M | Mihalik |
| J | Mihanovic |
| Jakov | Mihanovic |
| I | Mihaylov |
| M | Mihmanli |
| Borz | Mihnea Bogdan |
| Pablo | Mijahil Avilés Jiménez |
| Saulius | Mikalauskas |
| Sarah | Mikdad |
| Elie | Mikhael |
| Pola | Mikhail |
| A | Mikhailova |
| T | Mikhaylova |
| Hirokazu | Miki |
| Z | Mikovic |
| Filagot | Mikru |
| Iva | Mikulic |
| Vytenis | Mikutaitis |
| Duha | milad Abdullah |
| Marko | Miladinov |
| Rita | Milan Moussa |
| Flavio | Milana |
| M | Milanovic |
| Miljan | Milanovic |
| Theodoros | Milas |
| Ruben | Milciades Varela Cano |
| Claudia | Milena Orozco-Chamorro |
| M | Milenkovic |
| BA | Miles |
| Stephanie | Miles |
| Fabio | Milia |
| Mihailo | Milićević |
| Borna | Milicic |
| Filip | Milisavljević |
| Gabriela-Mariana | Militaru |
| P | Milito |
| Aleksandar | Miljković |
| T | Millane |
| Kate | Millar |
| A | Miller |
| Clemens | Miller |
| D | Miller |
| Douglas | Miller |
| Sarah | Miller |
| Matthias | Millesi |
| Keith | Millikan |
| Emily | Mills |
| L | Mills |
| CP | Millward |
| K | Milne |
| Stephanie | Milne |
| Sean | Milner |
| B | Milojevic |
| Bogomir | Milojevic |
| M | Milone |
| V | Milosavljevic |
| B | Milosevic |
| Genevieve | Milot |
| Ioan | Milotoiu |
| J | Milovanovic |
| Jovica | Milovanovic |
| Amelia | Milton |
| Vladan | Milutinović |
| M | Mimbela |
| Ximena | Mimica |
| Chew | Min Hoe |
| Marina | Minafra |
| Fabio | Minamoto |
| Maria | Minasidou |
| A | Minaya Bravo |
| AM | Minaya Bravo |
| Ana | Minaya Bravo |
| AM | Minaya-Bravo |
| Ana | Minaya-Bravo |
| F | Mineo Bianchi |
| Matthew | Ming Kei Kwok |
| A | Mingoli |
| Andrea | Mingoli |
| Joan | Minguell-Monyart |
| G | Mínguez Ruiz |
| German | Mínguez Ruiz |
| Andreas | Minh Luu |
| Alina | Minich |
| A | Minicozzi |
| Annamaria | Minicozzi |
| Stephen | Minlah Allah |
| Francesco | Minni |
| G | Minto |
| T | Minto |
| Pawel | Miotla |
| Anum | Mir |
| SA | Mir |
| A | Mirabella |
| Domenico | Mirabella |
| Sepideh | Miraj |
| Ester | Miralpeix |
| Marcos | Mirambeaux |
| BH | Miranda |
| I | Miranda |
| Ignacio | Miranda |
| P | Miranda |
| Norberto | Miranda Espinsa |
| Enric | Miret Alomar |
| RM | Mirica |
| R | Mirnezami |
| Reza | Mirnezami |
| A | Miron |
| Adrian | Miron |
| Scott | Miron |
| Aurel | Mironescu |
| Taha | Mirsal |
| M | Mirsalehi |
| N | Mirtorabi |
| M | Mirza |
| MB | Mirza |
| Monireh | Mirzaie |
| M | Miserez |
| Krisna | Mishel Morales Chew |
| A | Mishra |
| Abhijeet | Mishra |
| Anand | Mishra |
| Anjali | Mishra |
| Anurag | Mishra |
| Brijesh | Mishra |
| N | Mishra |
| Nitu | Mishra |
| Swastik | Mishra |
| Tushar | Mishra |
| J | Miskovic |
| Josip | Miskovic |
| Biplap | Misra |
| Gourab | Misra |
| N | Misra |
| S | Misra |
| Sanjeev | Misra |
| Vincent | Misrai |
| Rajesh | Mistry |
| Riyam | Mistry |
| S | Mistry |
| Courtney | Mitchell |
| D | Mitchell |
| S | Mitchell |
| R | Mithany |
| S | Mitrasinovic |
| Magdalini | Mitroudi |
| N | Mitrovic |
| Nebojsa | Mitrovic |
| Abhishek | Mittal |
| Radheyshyam | Mittal |
| Rohin | Mittal |
| S | Mittal |
| Samarth | Mittal |
| Yash | Mittal |
| M | Mitteregger |
| Martin | Mitteregger |
| C | Mittermair |
| Christof | Mittermair |
| Tajnin | Mitu |
| V | Miu |
| Morikazu | Miyamoto |
| N | Miyashita |
| Noriko | Miyazawa |
| Toshiyuki | Mizota |
| Fatimah | MJ Hassan Almukhariq |
| Catherine | Mkandawire |
| Aleš | Mladěnka |
| Jan | Mlakar |
| Busisiwe | Mlambo |
| MM H | Moahmmed |
| N | Moawad |
| Nader | Moawad |
| Ali | Moazami Pour Moazami Pour |
| Maher | Moazin |
| Shahd | Mobarak |
| S | Mochet |
| N | Möckelmann |
| Paulina | Moctezuma Velázquez |
| Tobias | Moczko |
| A | Modabber |
| Ali | Modabber |
| Nawaf | Modahi |
| Gautam | Modak |
| N | Modi |
| MM | Modolo |
| Nikolaus | Moeckelmann |
| Aiman | Moeen |
| Bolaji | Mofikoya |
| Chiedozie | Mogbo |
| E | Moggia |
| Elisabetta | Moggia |
| Mahmoud | Moghazy |
| C | Mogoanta |
| Carmen-Aurelia | Mogoanta |
| S | Mogoanta |
| Stelian | Mogoanta |
| Roberto | Moguel |
| SMR | Mohajeri |
| Balqees | Mohamad |
| Raied | Mohamad |
| A | Mohamed |
| Ahmed | Mohamed |
| Ali | Mohamed |
| Awadelkarim | Mohamed |
| Ayman | Mohamed |
| Elsagad | Mohamed |
| Eyas | Mohamed |
| Guleed | Mohamed |
| H | Mohamed |
| Haider | Mohamed |
| Hozifa | Mohamed |
| I | Mohamed |
| Ishak | Mohamed |
| Liena | Mohamed |
| M | Mohamed |
| Maria | Mohamed |
| Marwa | Mohamed |
| Mazin | Mohamed |
| Mervat | Mohamed |
| Moustafa | Mohamed |
| Muyed | Mohamed |
| Omer | Mohamed |
| S | Mohamed |
| Sami | Mohamed |
| Sugad | Mohamed |
| Y | Mohamed |
| Z | Mohamed |
| A | Mohamed Ads |
| Mostafa | Mohamed Ahmed |
| A | Mohamed alabany |
| Ferial | Mohamed Ali Abbas |
| Omar | Mohamed Alsamahy |
| Ahmed | Mohamed Altukhy |
| Dina | Mohamed elsaid |
| Ahmed | Mohamed Farouk |
| Aliae | Mohamed Hussein |
| Mokhtar | Mohamed Ibrahim Abushanab |
| Omar | Mohamed Makram |
| Mahmoud | Mohamed Mohamed Shalaby |
| Abdelrahman | Mohamed saad |
| Noha | Mohamed Salah Ibrahim Moussa Hamouda |
| Amr | Mohamed Sayed |
| Ali Yasen Y | Mohamedahmed |
| AYY | Mohamedahmed |
| A | Mohammad |
| Abdulkader | Mohammad |
| Adam | Mohammad |
| Ahmad N | Mohammad |
| Alaa | Mohammad |
| Ammar | Mohammad |
| Mazen | Mohammad |
| Aqsa | Mohammad eqbal Patel |
| Aminu | Mohammad Mohammad |
| Syed | Mohammad Umar Kabir |
| E | Mohammadbeigi |
| I | Mohammadbeigy |
| S | Mohammadi |
| H | Mohammadi sardoo |
| A | Mohammed |
| AA | Mohammed |
| Abdurezak | Mohammed |
| Altayeb | Mohammed |
| Ayman | Mohammed |
| Burooj | Mohammed |
| D | Mohammed |
| Diary | Mohammed |
| Garba | Mohammed |
| K | Mohammed |
| Khalid | Mohammed |
| M | Mohammed |
| Marwa | Mohammed |
| MM | Mohammed |
| N | Mohammed |
| R | Mohammed |
| Rawabi | Mohammed |
| Rayhaan | Mohammed |
| Tajudeen | Mohammed |
| Naseer | Mohammed Abdul |
| A | Mohammed Abodina |
| A | Mohammed alameen |
| Usman | Mohammed Bello |
| Ghena | Mohammed fawaz Ashour |
| Ansam | Mohammed Ghaleb Alrobaiee |
| Anass | Mohammed Majbar |
| Abdul-Jalilu | Mohammed Muntaka |
| Mubder | Mohammed Saeed |
| Seid | Mohammed Yasin |
| Ricardo | Mohammed-Ali |
| Amina | Mohammed-Durosinlorun |
| Shamshuddin | Mohammedali |
| H | Mohan |
| Helen | Mohan |
| Helen M | Mohan |
| HM | Mohan |
| M | Mohan |
| Vijay | Mohan Hanjoora |
| K | Mohankumar |
| Khaled | Mohd Ahmed Hasanein |
| AFN | Mohd Ghazi |
| Nik | Mohd Hazleigh |
| Ayesha | Mohd Zain |
| S | Mohindra |
| Sandeep | Mohindra |
| Obaid | Mohmand |
| Tamara | Mohorko |
| Shahin | Mohseni |
| M | Mohsin |
| Muhammad | Mohsin |
| R | Mohsine |
| Raouf | Mohsine |
| Omar | Mohyieldin |
| Inshrah | Moin |
| J | Moir |
| John | Moir |
| Peter | Moisiuk |
| G | Moitzi |
| Hossein | Mokarami Mokarami |
| Ahmed | Mokhtar |
| A | Mokhtari |
| Mayad | Moktash |
| Bogdan | Moldovan |
| RH | Moldovan |
| A | Molero |
| Ángela | Molero |
| Sarah | Molfino |
| Andrej | Moličnik |
| Marta | Molina |
| Olimpia | Molina |
| M | Molina Bravo |
| GA | Molina Proaño |
| V | Molina Santos |
| M | Molina-Corbacho |
| Matilde | Molina-Corbacho |
| Joel | Molinas |
| C | Moliner Sanchez |
| Yegor | Molitvin |
| Million | Molla |
| Cea-Cea | Moller |
| J | Mollinedo-Hun |
| G | Molteni |
| Samuel | Molyneux |
| A | Mombet |
| Annick | Mombet |
| Ahmed | Momen |
| Moses | Momoh |
| Luisa | Mona Kraus |
| E | Monaco |
| Fabrizio | Monaco |
| A | Mondal |
| Jose | Mondino |
| M | Mondragon |
| Hamada | Mondy |
| M | Monfort Mira |
| Montserrat | Monfort Mira |
| Lorenzo | Mongardi |
| F | Mongelli |
| Francesco | Mongelli |
| M | Moni |
| FA | Monib |
| Fatma A | Monib |
| Rocco | Monica |
| Elsayed | Monier |
| Mohammad | Monir Abbas |
| Ana | Monís |
| Keisuke | Monji |
| Hervé | Monka Lekuya |
| Berta | Monleón López |
| Chloé | Monnier |
| Sonia | Monreal Clua |
| Guillermo | Monsalve |
| JR T | Monson |
| S | Montal |
| Silvina | Montal |
| G | Montalvo Dominguez |
| C | Montan |
| Carl | Montan |
| Blanca | Montcusí |
| JM | Monteiro |
| Cathy | Monteith |
| KM | Montejo |
| M | Montelatici |
| M | Monteleone |
| Michela | Monteleone |
| MT | Montella |
| Jon-Alexis | Montemayor |
| Andrea | Montenegro |
| Emileth | Montenegro |
| Lourdes | Montero Cruces |
| R | Montero Macías |
| Rosa | Montero Macías |
| M | Montero Vega |
| Lourdes | Montes-Jovellar |
| Mario | Montes-Manrique |
| Nuria | Montferrer Estruch |
| Spencer | Montgomery |
| Eleonora | Monti |
| Marco | Monti |
| Guillermo | Montiel |
| S | Montolío-Doñate |
| Margarita | Montrimaite |
| J | Montufar |
| M | Montuori |
| Mauro | Montuori |
| J | Moon |
| RD C | Moon |
| Alex | Moore |
| Emily | Moore |
| Hamish | Moore |
| R | Moore |
| Rachel | Moore |
| T | Moores |
| Thomas | Moores |
| N | Moorjani |
| Sergio | Mora |
| Isabel | Mora Oliver |
| I | Mora-Guzmán |
| Isabela | Moraes |
| H | Morais |
| Henrique | Morais |
| Mariana | Morais |
| A | Moral Duarte |
| I | Moraleda Gudayol |
| Ines | Moraleda Gudayol |
| E | Morales |
| J | Morales |
| Steffanía | Morales |
| X | Morales |
| Xavier | Morales |
| Víctor | Morales Ariza |
| Clara | Morales Comas |
| N | Morales Palacios |
| Nelson | Morales Palacios |
| Eduardo | Morales valencia |
| Carmelo | Morales-Angulo |
| JE | Morales-Castelan |
| D | Morales-Garcia |
| Dieter | Morales-Garcia |
| Olaya | Moramay Romero-Limón |
| M | Moran |
| Andrea | Morandi |
| S | Morarasu |
| J | Moreau |
| Joshua | Moreau |
| Cátia | Moreira |
| João | Moreira |
| F | Moreira Borim |
| Andreu | Morell |
| L | Morelli |
| Luca | Morelli |
| Alessia | Morello |
| A | Moreno |
| Amabelle | Moreno |
| DH | Moreno |
| Emmanuel | Moreno |
| Jaime | Moreno |
| Nicolas | Moreno Mata |
| JA | Moreno Muñoz |
| A | Moreno Pérez |
| Mucio | Moreno Portillo |
| Jesús | Moreno Sierra |
| Marcelo | Moreno Suarez |
| Teresa | Moreno y Suárez |
| L | Moreno-Gomez |
| Angela | Moreno-Gutierrez |
| E | Moreno-Palacios |
| M | Moreno-Portillo |
| FJ | Morera Ocón |
| Charlie | Moret |
| G | Moretto |
| Gianluigi | Moretto |
| Silvia | Moretto |
| Daniele | Morezzi |
| Catrin | Morgan |
| E | Morgan |
| EA | Morgan |
| Ellie | Morgan |
| Katrina | Morgan |
| R | Morgan |
| Rebecca | Morgan |
| Steffan | Morgan |
| M | Morgom |
| Jemiludeen | Morhason-Bello |
| Krinal | Mori |
| C | Moriarty |
| Catherine | Moriarty |
| Trpimir | Morić |
| T | Morichau-Beauchant |
| Tristan | Morichau-Beauchant |
| Manuel | Moriche Carretero |
| D | Moris |
| Demetrios | Moris |
| Tomonori | Morita |
| Mai | Moriyama |
| HL | Morley |
| MT | Morna |
| D | Moro-Valdezate |
| David | Moro-Valdezate |
| Radoslav | Morochovič |
| Melanie | Morote |
| Domagoj | Morović |
| Brian | Morris |
| C | Morris |
| Daniel | Morris |
| R | Morris |
| Rachel | Morris |
| Richard | Morris |
| Jo | Morrison |
| V | Morrison-Jones |
| Victoria | Morrison-Jones |
| A | Morsi |
| Amr | Morsy |
| MS | Morsy |
| Hatan | Mortada |
| Xavier | Mortiers |
| Brienna | Mortimer |
| P | Mortini |
| Pietro | Mortini |
| A | Morton |
| Alastair | Morton |
| Mostafa | Mosabha |
| A | Mosca |
| A | Moscalu |
| F | Mosley |
| Frances | Mosley |
| Roman | Mosneaga |
| Matti-Aleksi | Mosorin |
| Monica | Mosos |
| Jana-Lee | Moss |
| Mafdi | Mossaad |
| Victoria | Mosshammer |
| Ahmed MHAM | Mostafa |
| B | Mostafa |
| Badr | Mostafa |
| Mohamed | Mostafa |
| El | mostafa El Yaqine Er Raoudi |
| Ahmed | Mostafa Saleh |
| Alejandra | Mosteiro-Cadaval |
| D | Moszkowicz |
| Clara | Mota |
| Reza | Motallebzadeh |
| N | Motas |
| Natalia | Motas |
| Masoud | Motasaddi zarandy |
| Akira | Motoyasu |
| H | Mottaghi Moghaddam Shahri |
| D | Motter |
| Dema | Motter |
| GIL | Mottola |
| Kanchan | Motwani |
| Mohammed | Mouaz Shebani |
| Susan | Moug |
| Mohamed | Mougahed |
| Hajar | Moujtahid |
| Didier | Moukoko |
| F | Moura |
| Mário | Moura |
| Mohamed | Mourad |
| Mohamed | Mourad Gargouri |
| Frederic | Mouriaux |
| P | Mourmouris |
| Panagiotis | Mourmouris |
| MA | Mous |
| Mohammad | Mousa |
| V | Mousafeiris |
| SA | Mousavi |
| SH | Mousavi |
| Ahmed | Moussa |
| M | Moussa |
| Esraa | Moustafa |
| L | Moutinho |
| Lana | Moutinho |
| Tyler | Mouw |
| O | Mouzakis |
| H | Moxon |
| Joaquin | Moya-Angeler |
| Vimbai | Moyana Muguto |
| Pablo | Moyano |
| Assel | Moyo |
| Gilbert | Moyo |
| Nkosikhona | Moyo |
| M | Mozafari |
| Masoud | Mozafari |
| M | Mozel |
| Michelle | Mozel |
| Lynette | Mpagi Katassi |
| Christophe | Mpirimbanyi |
| Bushr | Mrad |
| M | Mraiyan |
| Mohamed | Mraiyan |
| A | Msaddi |
| P | Mshelbwala |
| Philip | Mshelbwala |
| A | Msherghi |
| Ahmed | Msherghi |
| Wanga | Mtimkulu |
| L | Muallem-Kalmovich |
| Limor | Muallem-Kalmovich |
| A | Muashi |
| Mulaya | Mubambe |
| Kamilia | Mubarek |
| Areeba | Mubarik |
| Teeba | Mubaydeen |
| Isaac | Mubezi |
| F | Mucilli |
| Felice | Mucilli |
| L | Mude |
| Dillip | Muduly |
| I | Muehlbacher |
| Iris | Muehlbacher |
| Hosam | Muftah |
| Mohammed | Mufth |
| Dionizi | Muganga |
| Aamer | Mughal |
| Samiullah | Mughal |
| Didace | Mugisa |
| W | Mugla |
| Walid | Mugla |
| JM | Muguerza |
| Liviu | Mugurel Bosinceanu |
| Arnold | Muguwu |
| Rafat | Muhammad |
| Saminu | Muhammad |
| Shamsuddeen | Muhammad |
| Shoaib | Muhammad |
| Nasser | Muhammad Amjad |
| Hafiz | Muhammad Arif Arif |
| Sulaiman | Muhammad Daneji |
| Usama | Muhammad Kathia |
| Muheilan | Muheilan |
| Roger | Muhemi |
| Daniel | MUHIRE Runanira |
| Khadija | Muhmmed |
| Haidar | Muhssein |
| Ali̇ | Muhtaroğlu |
| Mahmoud | Muhtaseb |
| P | Muiesan |
| William | Muirhead |
| Carolyne | Muiru |
| Mohd | Mujahed Alkurdieh |
| Sonia | Mukase |
| Rene | Mukezamfura |
| Partho | Mukherjee |
| Poulome | Mukherjee |
| S | Mukherjee |
| Samrat | Mukherjee |
| Soumitra | Mukherjee |
| K | Mukhtar |
| Venkat | mukund reddy Galiveeti |
| Claudia | Mulas Fernández |
| Manoj | Mulchandani |
| W | Mulder |
| Lemesa | Muleta |
| F | Mulita |
| Francesk | Mulita |
| Husain | Mulla |
| Omar | Mulla |
| B | Muller |
| Bruno | Muller |
| M | Muller |
| A | Müller |
| Amelie | Müller |
| Katharina | Müller |
| P | Müller |
| Sophie | Müller |
| Thomas | Müller |
| Tobias | Müller |
| TU | Müller |
| Andrea | Mulliri |
| Esubalew | Mulugeta |
| G | Mulugeta |
| Gersam | Mulugeta |
| Khizra | Mumtaz |
| Kudzayi | Munanzvi |
| M | Munarriz |
| Marina | Munarriz |
| Fasiha | Munawwar |
| N | Mundkur |
| G | Mundy |
| Severien | Muneza |
| Simbarashe | Mungazi |
| S | Mungo |
| Maathichsudhaar | Muniandy |
| JA | Municio Martín |
| Martino | Munini |
| S | Munot |
| Herisardy | Munoz |
| R | Muñoz |
| AS | Munoz Abraham |
| CE | Muñoz Aguirre |
| Iciar | Muñoz Lindez |
| E | Muñoz Sornosa |
| Ernesto | Muñoz Sornosa |
| JM | Muñoz Vives |
| V | Muñoz-Atienza |
| L | Munoz-Bellvis |
| L | Muñoz-Bellvis |
| Luis | Muñoz-Bellvis |
| William | Munro |
| Fatema | Munshi |
| E | Muntaneza |
| George-Ovidiu | Muntean |
| Maximilian | Muntean |
| Alexandra | Munteanu |
| Hal | Munton |
| Emmanuel | Munyaneza |
| Hussain | Munyif |
| Akutu | Munyika |
| Gianni | Mura |
| A | Murad |
| Musab | Murad |
| A | Muraglia |
| Angelo | Muraglia |
| T | Murakawa |
| Tomohiro | Murakawa |
| Sreedutt | Murali |
| V | Muralidharan |
| Vijayaragavan | Muralidharan |
| Musa | Murat Caliskan |
| A | Muratore |
| Andrea | Muratore |
| Alfred | Mureko |
| A | Murgese |
| Alessandra | Murgese |
| Uxue | Murgoitio |
| ME | Muriel |
| Judith | Murillo |
| Bhaven | Murji |
| B | Murphy |
| Ben | Murphy |
| C | Murphy |
| James | Murphy |
| Matthew | Murphy |
| Niamh | Murphy |
| S | Murphy |
| Seamus | Murphy |
| Suzanne | Murphy |
| Daniel | Murray |
| Emma | Murray |
| Isabella | Murray |
| James | Murray |
| Ghulam | Murtaza |
| J | Murtha |
| Anandan | Murugesan |
| Kesavan | Murugesan |
| V | Murzi |
| Darya | Musa |
| Eltahir | Musa |
| Reem | Musa |
| Salam | Musa |
| Kabir | Musa Adamu |
| A | Musa Kirfi |
| Abdullahi | Musa Kirfi |
| Muhammad | Musaab |
| Muhammad | Musaab Munir |
| Norah | Musallam |
| Othiniel | Musana |
| Nizar | Musawa |
| Esther | Muscat |
| Luca | Muscatello |
| Serena | Musetti |
| Trust | Mushawarima |
| Willard | Mushiwokufa |
| Asma | Mushtaq |
| Hassan | Mushtaq |
| S | Mushtaq |
| A | Musina |
| Ana-Maria | Musina |
| L | Musini |
| Muhammad | Muslim |
| Gurbankhan | Muslumov |
| Fawzie | Musrati |
| A | Mustafa |
| Abdulla | Mustafa |
| Fatima | Mustafa |
| H | Mustafa |
| Ismail | Mustafa |
| Norasyikin | Mustafa |
| Q | Mustafa |
| Shiar | Mustafa |
| Ahmad | Mustafa Ahmad |
| Muntaqa | Mustapha |
| Mushawiahti | Mustapha |
| A | Mustea |
| Alexander | Mustea |
| Muhammad | Mustehsan Bashir |
| Shameel | Musthafa |
| Emmanuel | Mutabazi |
| H | Mutair |
| Nora | Mutalima |
| Precious G T | Mutambanengwe |
| Nastassja | Mutarello |
| Guhan | Muthkumaran |
| S | Muthu |
| Sathish | Muthu |
| Immaculee | Mutimamwiza |
| D | Mutlu |
| Nyasha | Mutsonziwa |
| D | Mutter |
| Didier | Mutter |
| Fredderick | Mutyaba |
| Twaha | Muwanga |
| Peter | Muwanguzi |
| Nasir | Muzaffar |
| Rahil | Muzaffar |
| Syed | Muzamil Ishaq Andrabi |
| Michael | Mwachiro |
| D | Mwagiru |
| Garikai | Mwale |
| Victor | Mwangi |
| Claude | Mwaria |
| Edwin | Mwintiereh Ta-ang Yenli |
| Ha | My Ngoc Nguyen |
| Khine | Myat Win |
| D | Myatt |
| R | Myatt |
| Richard | Myatt |
| J | Myers |
| Jonathan | Myers |
| Yulanda | Myint |
| Aye | Myintmo |
| P | Myrelid |
| Anna | Myriam Perrone |
| E | Myriokefalitaki |
| Stephanie | Myszkowski |
| Ranganath | N |
| Isabella | Naa Morkor Opandoh |
| N | Naabo |
| Nuhu | Naabo |
| L | Naar |
| Leon | Naar |
| Farhat | Naaz Amir |
| Assumpta | Nabawanuka |
| Bibi | Nabeeha Peerally |
| Syed | Nabeel Zafar |
| MH | Nabian |
| A | Nabil |
| Ahmed | Nabil |
| Sara | Nabil |
| Syed | Nabil |
| EA | Nachelleh |
| Emmanuel | Nachelleh |
| L | Nacif |
| A | Nada |
| Ahmed | Nada |
| Danilo | Nadal Rodrigues |
| Sylvie | Nadeau |
| Areeba | Nadeem |
| Syed | Nadeem Mujtaba |
| Anuja | Nadeeshan Kumarasinghe |
| Narita | Nadia Maria |
| R | Nadina |
| I | Nadj |
| Mandar | Nadkarni |
| A | Nadler |
| Marah | Nadreen |
| Muhammad | Naeem |
| Shiza | Naeem |
| F | Naegele |
| Felix | Naegele |
| Abdullah | Nael |
| Antoine | Naem |
| Joelle | Naem |
| Mohamed | nafea Shaar |
| R | Nafees Ahmed |
| Geerthan | Nagachandra |
| Shoichi | Nagamoto |
| Sriveena | Naganathar |
| Hidekazu | Naganuma |
| Mahesh | Nagappa |
| M | Nagar |
| Manoj | Nagar |
| Pirashanthan | Nagarasa |
| A | Nagaratnam |
| Fumiaki | Nagashima |
| Katharina | Nagassima |
| Ahmed | Nageeb |
| MA | Nageh |
| Mohammed | Nageh |
| Ali | Naghibi |
| Marina | Nagiub |
| Eleni-Aikaterini | Nagorni |
| Pooja | Nagpal |
| S | Nagra |
| Sonal | Nagra |
| SA | Nah |
| MA K | Nahid |
| C | Nahm |
| Christopher | Nahm |
| O | Nahtomi Shick |
| Orit | Nahtomi Shick |
| J | Nahum |
| S | Naidoo |
| Mohmed | Naieem |
| Prashant | Naik |
| Dina | Nail |
| Gabriel | Naimy |
| Asha | Nair |
| Deepa | Nair |
| Dilip | Nair |
| Gavin | Nair |
| Manojkumar S | Nair |
| R | Nair |
| Sreedevi | Nair |
| N | Najafian motahaver |
| Nima | Najafian motahaver |
| M | Najdy |
| Ramiro | Najera |
| Norhan | Naji |
| Osama | Naji |
| Suely | Nakagawa |
| Yuta | Nakamura |
| Rinako | Nakanishi |
| L | Nakano |
| LC U | Nakano |
| Luis | Nakano |
| Cephas | Nakanwagi |
| A | Nakas |
| Apostolos | Nakas |
| Yoshinori | Nakata |
| Harumasa | Nakazawa |
| Teddy | Nakirijja |
| U | Nakshbandi |
| M | Nalbant |
| D | Nally |
| Syeda | Namayah Fatima Hussain |
| G | Nambi |
| T | Nambirajan |
| T | Namikawa |
| Tsutomu | Namikawa |
| Dr | Namrata |
| Christine | Namugenyi |
| Esther | Namutosi |
| Gael R | Nana |
| Asanga | Nanayakkara |
| S | Nandhra |
| P | Nanjaiah |
| D | Nanjiani |
| P | Nankivell |
| Paul | Nankivell |
| Naya | Naoum |
| L | Napolitano |
| Lena | Napolitano |
| F | Nappi |
| Francesco | Nappi |
| Gennaro | Nappo |
| SA | Naqi |
| Z | Naqui |
| Zaf | Naqui |
| Wajih | Naqvi |
| Akarshan | Naraen |
| Sara | Naranjo |
| Venkateshwaran | Narasiman |
| G | Narasimhan |
| Gomathy | Narasimhan |
| P | Narayan |
| Surya | Narayan |
| Viswanathan | Narayanan |
| Kuppurajan | Narayanasamy |
| C | Nardi |
| Caroline | Nardi |
| B | Nardo |
| P | Naredla |
| Pradyumna | Naredla |
| Dhanyata | Narendra |
| B | Narice |
| Harry | Narroway |
| H | Naseem |
| Haris | Naseem |
| J | Naseem |
| Mai | Naseer |
| Ramisha | Naseer Nagra |
| Ezzeldin | Nashed |
| A | Nashidengo |
| PR | Nashidengo |
| O | Nasim |
| A | Nasir |
| Abdulrasheed | Nasir |
| Ahmad | Nasir |
| Areeba | Nasir |
| Manal | Nasir |
| A | Nasirpour |
| MH | Nasirpour |
| Upasana | Naskar |
| A | Nasr |
| Mohammed | Nasreddin |
| H | Nassa |
| Goretti | Nassali |
| Rosemary | Nassanga |
| Ahmed | Nassar |
| Ahmed | Nasser |
| M | Nasser |
| Nourhan | Nasser |
| S | Nasser |
| Shaimaa | Nasser |
| Y | Nasser |
| Mohammed | Nassif |
| Yasar | Nassif |
| Moses | Nassimu |
| C | Nastos |
| Fernando | Natal Álvarez |
| J | Natale |
| Leidy | Natalia Idarraga Ramírez |
| Marisa | Natalia Martinez |
| R | Nataraja |
| Ramesh | Nataraja |
| RM | Nataraja |
| R | Nath |
| A | Nathan |
| Meena | Nathan |
| Senthil K | Nathan |
| Rajesh | Nathani |
| Paulino | Nathaniel III Zamesa |
| A | Nathens |
| Hilli | Nativ |
| D | Naumann |
| DN | Naumann |
| Fatima | Naumeri |
| M | Naunheim |
| Matthew | Naunheim |
| Shazia | Naureen |
| Sara | Nausheen |
| Devaraj M | Navaratnam |
| L | Navaratne |
| Lalin | Navaratne |
| A | Navarrete-Peón |
| Alberto | Navarrete-Peón |
| A | Navarro |
| Alex | Navarro |
| Daniel | Navarro |
| Rosalia | Navarro Casado |
| Sergio | Navarro Martínez |
| Jorge | Navarro-Alean |
| Alvaro | Navarro-Barrios |
| A | Navarro-Sánchez |
| Antonio | Navarro-Sánchez |
| Ł | Nawacki |
| Hossam | Nawara |
| Anfal | Nawawi |
| Hassan | Nawaz Yaqoob |
| Prakash | Nayak |
| Jvalant | Nayan Parekh |
| Alejandra | Nayen |
| A | Nayen Sainz de la Fuente |
| Syed | Nayyar Afaque |
| Fatima | Nayyer |
| Falak | Naz |
| Humera | Naz Altaf |
| Mariles | Nazal |
| Zuhail | Nazar |
| S | Nazarian |
| Nawshin | Nazia |
| Shahani | Nazir |
| Umer | Nazir |
| Mohd | Nazli Kamarulzaman |
| Ahmad | nazran Fadzli |
| Ahmad | nbrass Kabawi |
| Isaie | Ncogoza |
| Gamuchirai | Ndabvonga |
| Fillipus | Ndatewapo |
| Chinedu | Ndegbu |
| Olivier | Ndizeye |
| Sibusiso | Ndlovu |
| Abdourahmane | Ndong |
| Alexandru | Neagu |
| Annelise | Neal |
| N | Neal |
| Naomi | Neal |
| Gregory | Neal-Smith |
| P | Neary |
| Paul | Neary |
| PC | Neary |
| Peter | Neary |
| PM | Neary |
| Ahmet | Necati Sanli |
| Yen | Nee Jenny Bo |
| Wan | Nee Shue |
| D | Neely |
| Samuel | Negash |
| I | Negoi |
| Ionut | Negoi |
| V | Negoiță |
| Lucian | Negreanu |
| JR | Negrete Ocampo |
| G | Negri |
| A | Negussie |
| Abraham | Negussie |
| Tihitena | Negussie Mammo |
| Archana | Nehe |
| D | Nehra |
| N | Neidert |
| Nicolas | Neidert |
| J | Neil-Dwyer |
| H | Nejad Biglari |
| Valery | Nekoval |
| Corne | Nel |
| D | Nel |
| Daniel | Nel |
| Henco | Nel |
| R | Nel |
| Prathibha | Nelihela |
| Ellen | Nelissen |
| D | Nellensteijn |
| T | Nelli |
| Tommaso | Nelli |
| Caleb | Nelson |
| T | Nelson |
| N | Nemat |
| Marcus | Nemeth |
| Abhay | Nene |
| J | Neny |
| Y | Nerabani |
| F | Neri |
| I | Neri |
| J | Neri |
| R | Nerlikar |
| Shuichiro | Neshige |
| LC | Nespoli |
| Ashrafun | Nessa |
| Carolyn | Nessim |
| Carl | Neuerburg |
| Filipe | Neves |
| Miguel | Neves |
| Arnaldo | Neves Santos Silva |
| Andrew | Newcomb |
| Laura | Newitt |
| Jeremy | Newman |
| Matthew | Newman |
| S | Newman |
| Samuel | Newman |
| T | Newman |
| C | Newton |
| K | Newton |
| Lydia | Newton |
| V | Neykov |
| Vasil | Neykov |
| Yves | Nezerwa |
| Giulia | Nezi |
| A | Ng |
| Benjamin | Ng |
| Calvin S | Ng |
| CE | Ng |
| Chi-Fai | Ng |
| Dennis | Ng |
| J | Ng |
| JC K | Ng |
| Jia Y | Ng |
| Jimmy | Ng |
| KC | Ng |
| M | Ng |
| Michael | Ng |
| Sherwin | Ng |
| Simon | Ng |
| YJ | Ng We Yong |
| Joshua | Ng-Kamstra |
| Ming | Ngan Aloysius Tan |
| S | Ngaserin |
| Sabrina | Ngaserin |
| CW | Ngo |
| G | Ngock |
| AW T | Ngu |
| James | Ngu |
| James | Ngu Chi Yong |
| D | Nguen |
| Allan | Ngulube |
| Peter | Ngungi Njuki |
| Alain | Nguyen |
| Anh | Nguyen |
| Huan | Nguyen |
| Sebastien | Nguyen |
| TA | Nguyen |
| Ncamsile | Nhlabathi |
| Orna | Ni Bhroin |
| Arastoo | Nia |
| Mourad | Niazi |
| C | Nic Gabhann |
| A | Nic Giolla Bháin |
| V | Nicastro |
| Eu | Nice Neo |
| Jaya | Nichani |
| Margit | Nichita |
| Ella | Nicholas |
| A | Nicholson |
| K | Nicholson |
| Kristina | Nicholson |
| S | Nicholson |
| Mutekanga | Nicholus |
| Sergio | Nicola Forti Parri |
| Antonio | Nicola Giordano |
| Sina | Nicolaiciuc |
| Beaud | Nicolas |
| Humberto | Nicolas Galleano Ruiz |
| Juan | Nicolas Rodriguez Niño |
| T | Nicolás-López |
| Tatiana | Nicolás-López |
| Cristina | Niculae |
| S | Nida |
| Melkamu | Nidaw |
| Andreas | Niemeier |
| AJ | Nieto Calvache |
| E | Nieto Ortega |
| Ana | Nieto-Moreno |
| Elena | Nieto-Moreno |
| Ninad | Nigalye |
| J | Nigh |
| H | Nikaj |
| Herald | Nikaj |
| Nikolaos | Nikiteas |
| Kiselev | Nikolai |
| Karine | Nikolaieva |
| S | Nikolaou |
| B | Nikolic |
| Srdjan | Nikolic |
| Zoi | Nikoloudaki |
| Taxiarchis | Nikolouzakis |
| B | Nikolovska |
| Bisera | Nikolovska |
| Hamed | Nikoupour |
| Alexandrina | Nikova |
| Boateng | Nimako |
| Jilac | Nimako-Mensah |
| Suad | Nimale |
| Bahaa | Nimer |
| Oti | Nimi Aria |
| Avegail | Niña Uy |
| Aleksandar | Ninic |
| M | Ninkovic |
| Marijana | Ninkovic |
| M | Niquen-Jimenez |
| Milagros | Niquen-Jimenez |
| Anand | Nirgude |
| Sudhara | Niriella |
| Kanwal | Nisa |
| P | Nisar |
| Sebastian P | Nischwitz |
| SP | Nischwitz |
| Noriaki | Nishihara |
| Kouhei | Nishikawa |
| Karolina | Niska |
| GE | Nita |
| C | Nitschke |
| Christine | Nitschke |
| Amy | Nixon |
| S | Niyas |
| Irénée | Niyongombwa |
| Maria | Nizami |
| Rafal | Niziol |
| Narindra | Njarasoa Mihaja Razafimanjato |
| Nwabundo | Njeze |
| Tsi | Njim |
| Vinod | Nk |
| E | Nkenke |
| Theresa | Nkole |
| John | Nkrumah |
| J M Solange | Nkubito |
| Elysé | Nkunzimana |
| Ikenna | Nnabugwu |
| Henry | Nnajiuba |
| Kareem | Noah |
| S | Nobile |
| DM | Noboa |
| G | Nobre |
| A | Nobre Pinto |
| Antonio | Nocchi Kalil |
| Gianluca | Nocera |
| Michio | Noda |
| Chiara | Noe |
| C | Noel |
| Colin | Noel |
| J | Noel |
| Audrey | Noël |
| Sandra | Nofal |
| SJM | Nofal |
| Hikari | Noguchi |
| O | Nogueira |
| J | Nogueiro |
| Jorge | Nogueiro |
| Lidia | Noguera Roman |
| A | Nogués |
| Ana | Nogués |
| M | Noguez Castillo |
| Monica | Noguez Castillo |
| Barbara | Noiret |
| B | Nolan |
| Deirdre | Nolan |
| Florencia | Noll |
| Peter | Nolte |
| Milou | Noltes |
| Hamza | Noman |
| Takeshi | Nomura |
| Anthony | Noone |
| Anthony | Noor |
| Fazal | Noor |
| M | Noor |
| Mohammad | noor Sultan |
| Shahryar | Noordin |
| Ehsan | Noori |
| Farshad | Noori |
| R | Norawat |
| Rahul | Norawat |
| C | Norcini |
| M | Nordberg |
| Martin | Nordberg |
| L | Nordin |
| Zahida | Noreen |
| Mohd | Norhisham Azmi Abdul Rahman |
| David C | Noriega |
| D | Noriego Muñoz |
| J | Norman |
| Lisa | Norman |
| Shusaku | Noro |
| M | Norouzi |
| Samieh | Norouzi |
| Jonathan | Norris |
| Alan | Norrish |
| Michael | Nortey |
| A | Northey |
| Joel | Norton |
| Sam | Norton |
| William | Norton |
| Jaysonnel | Notario |
| TM | Noton |
| Toby | Noton |
| F | Notte |
| Quirin | Notz |
| T | Nouh |
| Thamer | Nouh |
| Mohammad | Nour Kitaz |
| Mohammad | Nour Shashaa |
| Sadaf | Noureen |
| Yasser | Noureldin |
| Z | Novak |
| Zoltan | Novak |
| Maria | Novella Ringressi |
| S | Novello |
| Simone | Novello |
| Daniela | Novembre |
| Alessandra | Novi |
| David | Novikov |
| A | Novikova |
| Anastasia | Novikova |
| Beatriz | Novoa |
| R | Novysedlak |
| René | Novysedlak |
| René | Novysedlák |
| K | Nowak |
| Kai | Nowak |
| Mostafa | Nowar |
| Yvonne | Nowosielski |
| Josephine | Nsaful |
| Steven | Nshuti |
| Edmond | Ntaganda |
| Maria | Ntalouka |
| MP | Ntalouka |
| Arnold | Ntege |
| Joseph | Ntege |
| Japhet | Ntezamizero |
| F | Ntirenganya |
| Faustin | Ntirenganya |
| Sarwar | Nubair |
| N | Nudell |
| BD | Nuertey |
| T | Nugent |
| Timothy | Nugent |
| W | Nugent III |
| AAA | Nugud Abd Alwahab Aljafary |
| Ashwani | Nugur |
| Afnan | Nuh |
| Yasir | Nuhu Jibril |
| KS | Nunes |
| QM | Nunes |
| Rafael | Nunes |
| RL | Nunes |
| Sara | Nunes |
| Margarida | Nunes Coelho |
| M | Nunes-Coelho |
| J | Nunez |
| Jade | Nunez |
| HM | Nuñez |
| J | Nuñez |
| Jorge | Nuñez |
| Ruth | Nuñez |
| Analia | Núñez |
| Bernardo | Núñez |
| J | Núñez |
| Henar | Nuñez Del Barrio |
| Jorge | Núñez Lucic |
| Sara | Núñez OSullivan |
| H | Núñez-Del Barrio |
| B | Nunez-Garcia |
| Brenda | Nunez-Garcia |
| R | Nunn |
| Duarte | Nuno Amaro |
| Cesar | Nuño-Escobar |
| JW | Nunoo-Mensah |
| Anna | Nunzia Della Gatta |
| Syeda | Nureena Syed Jafer Hussain Zaidi |
| Talgat | Nurgozhin |
| Amjad | Nuseir |
| SE | Nwabuoku |
| Callistus | Nwachukwu |
| Ijeoma | Nwachukwu |
| Ikechukwu | Nwafor |
| Chimaobi | Nwagboso |
| OG | Nwaorgu |
| Onyekwere | Nwaorgu |
| Nnamdi | Nwashilli |
| C | Nwegbu |
| CG | Nwegbu |
| Chukwuemeka | Nwegbu |
| Hope | Nwinee |
| SO | Nwose |
| David | Nwosu |
| Beauty | Nyadu |
| Krystel | Nyangoh Timoh |
| Yaa | Nyarko Agyeman |
| M | Nycz |
| Miriam | Nyeko-Lacek |
| Rachel M | Nygaard |
| RM | Nygaard |
| Domitille | Nyirahabakurama |
| Jeannette | Nyirahabimana |
| Alexandre | Nyirimodoka |
| C | Nzekwue |
| IC | Nzenwa |
| C | O connor |
| Clare | O Connor |
| J | O Connor |
| Helle | Ø Kristensen |
| Augustine | O Takure |
| S | O’Brien |
| A | O’driscoll-collins |
| Kristin | O'Mara-Gardner |
| C | O’Neil |
| C | O’Neill |
| Izegaegbe | Obadan |
| Osarobo | Obahiagbon |
| Kadhim | Obaid |
| Munzir | Obaid |
| LA | OBanion |
| Shinju | Obara |
| C | Obasi |
| John | Obateru |
| Ambe | Obbeng |
| K | Obeidat |
| Riyad | Obeidat |
| M | Oberlechner |
| Hamoud | Obied |
| D | Obrand |
| S | OBrien |
| Stephen | OBrien |
| Christian | Obrist |
| Ozoemene | Obuekwe |
| Ralph | Obure |
| Lorena | Ocampo |
| Lorena | Ocampos |
| Montserrat | Ocampos Hernandez |
| J | Ocaña |
| Savino | Occhionorelli |
| Francisco J | Ochoa Carrillo |
| Katherine | Ochoa Gaete |
| E | Ochoa Maldonado |
| Begoña | Ochoa Villalabeitia |
| C | OConnell |
| Eimear | OConnell |
| Rachel | Oconnell |
| Brendan | OConnor |
| C | OConnor |
| DB | OConnor |
| Donal B | OConnor |
| J | OConnor |
| Jennifer | OConnor |
| Z | OConnor |
| Zachary | OConnor |
| Narcis | Octavian Zarnescu |
| Sandhya | Od |
| ED | Odai |
| Amar | Odedra |
| A | Odeh |
| Abdulrahman | Odeh |
| Funlayo | Odejinmi |
| Diego | Odetto |
| Olubunmi | Odeyemi |
| F | Odicino |
| Franco | Odicino |
| Guillaume | Odilon Tsiambanizafy |
| Eddie | Odonnell |
| Richard | ODonnell |
| Cristina | ODonoghue |
| Gerrard | ODonoghue |
| A | Odriscoll-collins |
| Stella | Oduah |
| Tunde | Odunafolabi |
| Oladayo | Oduola |
| O | Odutola |
| Oluwatomi | Odutola |
| Eoin | OFarrell |
| Richard | Offiong |
| D | Öfner |
| Bernard | Ofori |
| EO | Ofori |
| B | Ofori Appiah |
| Obed | Ofori Nyarko |
| Munehiro | Ogawa |
| E | Ogden |
| Emma | Ogden |
| Roy | Ogenya |
| Tomomi | Ogihara |
| Richard | Ogirma Baidoo |
| Michael | OGrady |
| Rosemary | Ogu |
| H | Öğücü |
| Olukayode | Ogunade |
| Ibukunolu | Ogundele |
| O | Ogundoyin |
| Olakayode | Ogundoyin |
| Omowonuola | Ogundoyin |
| Olumuyiwa | Ogunlaja |
| Tolulope | Ogunrewo |
| Oluseyi | Ogunsua |
| AA | Ogunyemi |
| Benson | Oguttu |
| Cebrail | Oğuz |
| Ufuk | Oguz Idiz |
| JS | Oh |
| Patrick | OHagan |
| Shinnosuke | Ohama |
| Rozana | Ohara |
| Ephraim | Ohazurike |
| K | Oikonomou |
| Kyriakos | Oikonomou |
| S | Oishi |
| Cristina | Ojeda Thies |
| C | Ojeda-Thies |
| Olugbenga | Ojo |
| Owolabi | Ojo |
| H | Okada |
| Hidetaka | Okada |
| Hiroshi | Okada |
| Reina | Okada |
| Shinichiro | Okada |
| Toshimasa | Okada |
| Barbara | Okafor |
| BU | Okafor |
| Kaio | Okamura |
| Yukiyasu | Okamura |
| R | OKane |
| Y | Okazawa |
| V | Okechukwu |
| Aloy | Okechukwu Ugwu |
| John | OKelly |
| Oghenekevwe | Okere |
| Chukwuma | Okereke |
| Amina | Okhakhu |
| L | Okiror |
| W | Okoba |
| Louis | Okolie |
| Ijeoma | Okonkwo |
| Kelechukwu | Okoro |
| Eloka | Okoye |
| Onyedika | Okoye |
| Anthony | Okpani |
| Charles | Okpani |
| Thomas | Okpoti Konney |
| V | Oktseloglou |
| Stanley | Okugbo |
| Kehinde | Okunade |
| Abiodun | Okunlola |
| AI | Okunlola |
| T | Okuno |
| AA | Okunowo |
| Adeyemi | Okunowo |
| Blasius | Okwara |
| Afusat | Olabinjo |
| Titilola | Oladejo |
| Ajibola | Oladiran |
| Thomas | Olagboyega Olajide |
| Naomi | Olagunju |
| Hadijat | Olaide Raji |
| Adewale | Olajide |
| Peter | Olalekan Odeyemi |
| Julius | Olaogun |
| Oluwole | Olaomi |
| Rasaq | Olaosebikan |
| Ifedolapo | Olaoye |
| O | Olasehinde |
| Olalekan | Olasehinde |
| David | Olatayo Olayiwola |
| VD | Olave Montaño |
| Olayinka | Olawoye |
| Aminat | Olayinka Ahmed |
| Tolulope | Olayinka Sayomi |
| Dina | Olaywah |
| Noor | Olaywah |
| O | Olazábal |
| Massimo | Oldani |
| Megan | Oldbury |
| Gaia | Oldrà |
| Claudia | Olea Vielba |
| E | Olearo |
| Elena | Olearo |
| G | Olgac |
| Annette | Olieman |
| P | Oliva |
| R | Oliva |
| Ramon | Oliva |
| F | Oliva Mompean |
| Rachel | Olive |
| A | Oliveira |
| Antonio | Oliveira |
| CM | Oliveira |
| J | Oliveira |
| JM | Oliveira |
| Joana | Oliveira |
| João | Oliveira |
| P | Oliveira |
| Priscila R | Oliveira |
| Thomas | Oliver |
| Tibor | Oliver Andraschofsky |
| Darwin | Oliver Desposorio Armestar |
| Luis | Oliver García |
| JR | Oliver Guillen |
| M | Olivera Villanueva |
| CA | Oliveros Ruiz |
| Ana | Olivia Cortes-Flores |
| J | Olivier |
| M | Olivos |
| Maricarmen | Olivos |
| Didier | Ollat |
| I | Oller |
| Inmaculada | Oller |
| Sue | Olliff |
| Benjamin | Ollivere |
| Stefano | Olmi |
| C | Olona |
| S | Olori |
| Samson | Olori |
| Tope | Olowogbayi |
| KA | Olson |
| Kristofor A | Olson |
| SA | Olson |
| Adebayo | Olugbami |
| Denis | Oluka |
| D | Olulana |
| Dare | Olulana |
| Adeola | Olusanya |
| Anne | Olute |
| Fatudimu | Oluwafemi |
| Adeoye | Oluwakanyinsola Debo-Aina |
| Oscar-Everardo | Olvera-Flores |
| I | Omar |
| Kadra | Omar |
| M | Omar |
| Mabruka | Omar |
| Mohamed W | Omar |
| MS M | Omar |
| Samaa | Omar |
| Sana | Omar |
| W | Omar |
| Wael | Omar |
| Hayat | Omar Abunaaja |
| Ahmed | omar Abushahma |
| David | Omar Arriaga Zavala |
| Bashar | Omar Falah Alawneh |
| Mohamed | Omar Herdan |
| José | Omar Zorrilla Lara |
| Usra | Omara |
| Rand Y | Omari |
| Saranda | Ombashi |
| L | OMeara |
| Ahmed | Omer Kenawy |
| S | Ömeroğlu |
| Sinan | Ömeroğlu |
| A | Omigbodun |
| Akinyinka | Omigbodun |
| Paul | Omiragi |
| OA | Omisanjo |
| Olufunmilade | Omisanjo |
| I | Omiste |
| E | Omling |
| A | Omnia |
| Justina | Omoikhefe Alegbeleye |
| Philip | Omotosho |
| Janna | Omran |
| M | Ömür |
| Paul | Onakoya |
| MA | Onan |
| Julian | Onate |
| Mikel | Oñate |
| M | Oñate Aguirre |
| C | ONeil |
| A | ONeill |
| Aine | ONeill |
| C | ONeill |
| Christine | ONeill |
| JR | ONeill |
| Robert | ONeill |
| CS | Ong |
| CT | Ong |
| Daniel | Ong |
| Lester | Ong |
| Oluwafemi | Oni |
| Samson | Oni |
| T | Oni |
| S | Onida |
| Stephen | Onjefu |
| Motoaki | Ono |
| Elliot | Onochie |
| Ilaria | Onorati |
| L | Onos |
| Güralp | Onur Ceyhan |
| Mustafa | Onur Oztan |
| Ijeoma | Onwuagha |
| Ngozi | Onyeagwara |
| John | Onyeji |
| Ndubuisi | Onyemaechi |
| A | Oo |
| Aung | Oo |
| Chun | Ooi |
| Rucira | Ooi |
| SZY | Ooi |
| W | Oosterlinck |
| Wouter | Oosterlinck |
| S | Oosterling |
| Steven | Oosterling |
| Benjamin | Oosthuizen |
| Rivero | Opano |
| Hameedat | Opeyemi Abdussalam |
| Janis | Opincans |
| Wisdom | Opoku Amankwaa |
| Ijeoma | Oppah |
| Victory | Oputa |
| Alex | Orădan |
| Emmanuel | Oranu |
| Ngozi | Orazulike |
| Jorge | Ordemar |
| Sebastian | Ordoñez |
| E | Ore |
| E | Oré |
| Nicole | Organ |
| Ahmed | Organjee |
| Sergelen | Orgoi |
| Nazlı | Orhan |
| Mukadder | Orhan Sungur |
| M | ORiordain |
| J | Oriordan |
| JM | ORiordan |
| Mathias | Orji |
| Till | Orla Klatte |
| Martin | Ormeño |
| S | Ornaghi |
| Sara | Ornaghi |
| Mariano | Oropeza |
| J | Orozco Mera |
| J | Orozco Perez |
| CM | Orozco-Chamorro |
| J | Orozco-Perez |
| Jaime | Orozco-Perez |
| Soyombo | Orsoo |
| Ma Dolores | Orta Díaz |
| Natalia | Ortega |
| Shannat | Ortega |
| Manuel | Ortega Oria de Rueda |
| J | Ortega Serrano |
| I | Ortega Vazquez |
| Irene | Ortega Vázquez |
| Liz | Ortiz |
| N | Ortiz |
| Fabio | Ortiz De La Cruz |
| FJ | Ortiz de Solorzano-Aurusa |
| David | Ortiz López |
| Camilo | Ortiz Silva |
| Almudena | Ortiz Simón |
| MR | Ortiz-Argomedo |
| Mustafa | Oruç |
| L | Oryadi zanjani |
| Leila | Oryadi zanjani |
| Anita | Osabutey |
| L | Osagie |
| Olabisi | Osagie |
| Shuhei | Osaki |
| Muhammad | Osama Khan |
| Syed | Osama Zohaib Ullah |
| T | Osborn |
| Tamara | Osborn |
| Laura | Osborne |
| A | Oscarsson |
| Anna | Oscarsson |
| Lydia | Osea |
| Eustace | Oseghale |
| Peter | Osei-Bonsu |
| Dorcas | Osei-Poku |
| Ethel | Osei-Tutu |
| A | Oseira-Reigosa |
| Osarenkhoe | Osemwegie |
| Margaret | OShea |
| Philip | Osho |
| OA | Oshodi |
| YA | Oshodi |
| Babatunde | Osinaike |
| Antonia | Osl |
| Alkhansa | Osman |
| Amna | Osman |
| Elaf | Osman |
| Halim | Osman |
| I | Osman |
| Imoro | Osman |
| Khalid | Osman |
| Leyla | Osman |
| Mohamed | Osman |
| Rafael | Osmar Adorno Garayo |
| Filipe | Osni Coelho |
| A | Osorio |
| Derlis | Osorio |
| walter A | Osorio |
| C | Osório |
| M | Osso |
| ME | Ossola |
| Charles | Osterberg |
| Georg | Osterhoff |
| Erica | OSullivan |
| H | OSullivan |
| M | OSullivan |
| Laura | Osuna |
| Jaqueline | Osuna-Rubio |
| Roland | Osuoji |
| Edgar | Oswaldo Hernandez Burgos |
| Wessal | Otaif |
| E | Otañez |
| Sameer | Otayfah |
| Robin | Otchwemah |
| Ahmed | Oteem |
| V | Oter |
| Volkan | Oter |
| Henry | Othieno misanga |
| Sudheer | Othiyil vayoth |
| A | Othman |
| Abdullmujeeb | Othman |
| Ahmad | Othman |
| Eyas | Othman |
| H | Othman |
| Mohammed | Othman |
| Salasiah | Othman |
| James | Otieno |
| M | Otify |
| Mohamed | Otify |
| CE | Otiniano Alvarado |
| Job | Otokwala |
| Riinu | Ots |
| Yuji | Otsuka |
| Johannes | Ott |
| Helena | Otte |
| Stephanie | Ottl |
| J | Ottolina |
| A | Ottone |
| C | Ouanezar |
| Takashi | Ouchi |
| MY | Oudrhiri |
| Abdallah | Ouf |
| M | Oukan |
| Anas | Ould Si Amar |
| J | Ourieff |
| Jared | Ourieff |
| Susana | Ourô |
| Samia | Ousouss |
| K | Oussama |
| O | Outani |
| Oumaima | Outani |
| A | Ovaitt |
| E | Ovejero Merino |
| Rawan | Owaimer |
| Qais | Owais |
| Muhammad | Owais Abdul Ghani |
| R | Owen |
| David | Owens |
| P | Owens |
| Patrick | Owens |
| Cornelia | Ower |
| M | Owiedat |
| Mustafa | Owiedat |
| Jefferson | Owusu Adae |
| Emmaunel | Owusu Ofori |
| A | Owusu-Addo |
| Tasuku | Oyama |
| T | Oyebanji |
| Funmilayo | Oyediji |
| OA | Oyelakin |
| Oyeleye | Oyelakin |
| Oluwole | Oyeleye |
| Nasir | Oyelowo |
| O | Oyende |
| Olamide | Oyende |
| Oluniyi | Oyetunde Olubayo |
| B | Oyewole |
| Olugboyega | Oyewole |
| Esther | Oyewusi |
| Olumide | Oyinloye |
| Ahmad | Ozair |
| Muhammad | Ozair Awan |
| Farrukh | Ozair Shah |
| Gultekin | Ozan Kucuk |
| Ali | Özant |
| İH | Özata |
| V | Ozben |
| Volkan | Ozben |
| A | Özcan |
| Adem | Özcan |
| Necdet | Özçay |
| MF | Ozcelik |
| Egemen | Ozdemir |
| Kayhan | Ozdemir |
| Kamil | Özdoğan |
| Elif | Özeller |
| D | Ozgediz |
| Doruk | Ozgediz |
| U | Özgen |
| Utku | Özgen |
| I | Ozgur |
| Ilker | Ozgur |
| OF | Ozkan |
| Güneş | özlem Yıldız |
| BB | Ozmen |
| MM | Ozmen |
| Christine | Ozone |
| H | Ozsahin |
| Joshua | Ozua |
| Hareesh | P B |
| Thirumanikandan | P L |
| R | P Shenoy |
| Peter | Paal |
| Anishka | Pabari |
| Carmignani | Pablo |
| Juan | Pablo Alzate |
| Juan | Pablo Campana |
| Pedro | Pablo Díaz Vásquez |
| Juan | Pablo Idrovo |
| Juan | pablo Villate leon |
| D | Pacheco Sánchez |
| David | Pacheco Sánchez |
| M | Pachl |
| Max | Pachl |
| CA | Pacilio |
| M | Pacilli |
| Maurizio | Pacilli |
| F | Padilla-Lichtenberger |
| D | Padilla-Valverde |
| David | Padilla-Valverde |
| Greg | Padmore |
| Walter | Páez |
| Gianluca | Pagano |
| Luca | Pagano |
| Richard | Page |
| S | Page |
| Naila | Pagès |
| D | Paglione |
| U | Pahalawatta |
| Upuli | Pahalawatta |
| M | Pai |
| Lucia | Paiano |
| S | Paiella |
| Salvatore | Paiella |
| William | Paine |
| F | Pais |
| José | Pais |
| S | Paitici |
| Stefan | Paitici |
| Joana | Paiva |
| Filipe | Paiva-Santos |
| H | Pajan |
| Hendra | Pajan |
| Srbislav | Pajić |
| M | Pakiž |
| M | Pal |
| RR | Pala Bhaskar |
| Carlos | Palacios |
| Juan | Palacios |
| RM | Palacios Huatuco |
| S | Palagi |
| R | Palaia |
| attibele | Palaksha Manjunatha |
| Naresh | Palapalle |
| Kandasami | Palayan |
| P | Palazon Bellver |
| Pedro | Palazon Bellver |
| F | Palazzo |
| M | Palechor |
| A | Palepa |
| Alejandro | Palines |
| GM | Palini |
| Effrosyni | Palla |
| Henrik | Palm |
| Matteo | Palma |
| P | Palma |
| Nikolas | Palma Caucig |
| M | Palmeri |
| Matteo | Palmeri |
| Emanuela | Palmerini |
| Gerardo | Palmieri |
| Silvia | Palmisano |
| Sara | Palomares Casasús |
| Liliam | Palomino |
| Fiorella | Palomino Escalante |
| Nicholas | Paltoglou |
| Alessio | Palumbo |
| Mara | Palumbo |
| EC | Pama |
| Jessica | Pamela Portillo Sosa |
| Jorge | Pamias |
| Sousana | Panagiotidou |
| Peter | Panagiotou |
| P | Panahi |
| B | Panamarenko |
| Yiannis | Panayiotou |
| Guergana | Panayotova |
| Suresh | Panchakshariah |
| Abinash | Panda |
| Ritesh | Panda |
| S | Pandanaboyana |
| Amita | Pandey |
| Himanshu | Pandey |
| Sanjay | Pandey |
| V | Panduro-Correa |
| Lucía | Pañeda |
| Divyansh | Panesar |
| H | Panesar |
| M | Paniagua Garcia Senorans |
| Sibasish | Panigrahi |
| Carolina | Panis |
| Taufiq | Panjwani |
| S | Pankaj |
| S | Pankhania |
| Mario | Pannullo |
| Igor | Panov |
| Adrieli | Pansani |
| Rajeev | Pant |
| Christina | Panteli |
| Maria | Pantelidou |
| DA | Pantoja Pachajoa |
| JC | Pantoja Rodriguez |
| Pavlos | Pantos |
| Vishwakar | Panuganti |
| VK | Panwar |
| A | Panyko |
| Arpád | Panyko |
| Luisa | Paola Garzon |
| Maria | Paola Giusti |
| Maria | Paola Menna |
| Anna | paola Pezzuto |
| Francesca | Paola Tropeano |
| Ximena | Paola Vasquez Ojeda |
| Benjamin | Paolini |
| Pier | Paolo Panciani |
| Adrian | Papa |
| Ioannis | Papaconstantinou |
| A | Papadia |
| Andrea | Papadia |
| M | Papadoliopoulou |
| A | Papadopoulos |
| V | Papadopoulos |
| Vasileios | Papadopoulos |
| Triada | Papadopoulou |
| V | Papagni |
| Vincenzo | Papagni |
| Alexandros | Papalampros |
| Stylianos | Papalexandris |
| R | Papalia |
| Rocco | Papalia |
| Matteo | Papandrea |
| V | Papanikolaou |
| Vasileios | Papanikolaou |
| Theofanis | Papas |
| Panayiotis | Papatheodorou |
| K | Papavasiliou |
| Kyriakos | Papavasiliou |
| I | Papazacharias |
| Nancy | Papendick |
| Ketevan | Papidze |
| A | Papinutti |
| Sam | Pappas |
| B | Paquette |
| Mrunal | Parab |
| Sandesh | Parab |
| J | Parakh |
| Thammawat | Parakonthun |
| Arjun | Paramasivan |
| Javier | Páramo Zunzunegui |
| C | Paranjape |
| Charu | Paranjape |
| M | Paranyak |
| A | Paraskeva |
| KI | Paraskevas |
| Kosmas I | Paraskevas |
| K | Paraskevopoulos |
| Konstantinos | Paraskevopoulos |
| Rivka | Pardes |
| Ana | Pardilho |
| Dinshaw | Pardiwala |
| JM | Pardo Garcia |
| I | Paredes |
| Igor | Paredes |
| Susana | Paredes |
| EJ | Paredes Alvarez |
| Angie | Paredes Caturiny |
| Dilber | Pareed |
| P | Pareek |
| Puneet | Pareek |
| R | Pareja |
| Felipe | Pareja Ciuro |
| F | Pareja-Ciuró |
| A | Parello |
| Angelo | Parello |
| A | Parente |
| Alessandro | Parente |
| Charlotte | Parfitt |
| Pinki | Pargal |
| R | Pargaru |
| C | Parianos |
| Sucheta | Parija |
| Mélinda | Paris |
| P | Parise |
| Andy | Park |
| C | Park |
| Chang | Park |
| Felicity | Park |
| Jennifer | Park |
| Melissa | Park |
| P | Park |
| Paul | Park |
| Dominic | Parker |
| Katie-Louise | Parker |
| Robert | Parker |
| Benjamin | Parkin |
| Cameron | Parkin |
| Edward | Parkin |
| K | Parkins |
| D | Parlanti |
| Daniele | Parlanti |
| C | Parmar |
| Chetan | Parmar |
| Matias | Parodi |
| F | Parolini |
| Katherine | Parra Abaunza |
| N | Parra Paredes |
| Fazl | Parray |
| Pedro | Parreira |
| B | Parrella |
| MD | Parreno-Sacdalan |
| P | Parri |
| Paco | Parri |
| C | Parrilla |
| Andrew | Parrish |
| J | Parry |
| James | Parry |
| Laura | Parry |
| William | Parry-Smith |
| A | Parseliunas |
| Rajinder | Parshad |
| Simon | Parsons |
| Thomas | Parsons |
| Ramaa | Parulekar |
| S | Parveen |
| Sajitha | Parveen |
| Hammad | Parwaiz |
| A | Pasca |
| A | Pascale |
| G | Pascale |
| G | Pascarella |
| Giuseppe | Pascarella |
| JA | Pasch |
| J | Pascoe |
| LA | Pascua Gómez |
| À | Pascual |
| Ángela | Pascual |
| FR | Pascual |
| T | Pascual |
| Victoria | Pascual Escudero |
| Isabel | Pascual Miguelañez |
| Montserrat | Pascual Pascual Arellano |
| M | Pascual Samaniego |
| Miguel | Pascual Samaniego |
| Patricia | Pascual-Cambero |
| M | Pashaei |
| F | Pasini |
| Miram | Pasini |
| J | Paskas |
| A | Paspala |
| D | Paspaliari |
| MJ | Paspuel Villacís |
| S | Pasquali |
| Ernesto | Pasquini |
| R | Pasricha |
| Martin | Passadore |
| G | Passot |
| I | Pastau |
| Oscar | Pastor |
| Tania | Pastor |
| Ana | Pastor Zapata |
| F | Pata |
| M | Patabendige |
| Malitha | Patabendige |
| Robert | Patachia |
| S | Patauner |
| K | Pateas |
| A | Patel |
| Akshay | Patel |
| Arjun | Patel |
| Bhavin | Patel |
| CHK | Patel |
| D | Patel |
| Jamie | Patel |
| K | Patel |
| Kapila | Patel |
| Krishnakumure | Patel |
| Lopa | Patel |
| M | Patel |
| Minil | Patel |
| Mohammed | Patel |
| Nikhita | Patel |
| P | Patel |
| Panna | Patel |
| Preemal | Patel |
| Priyank | Patel |
| Rakesh | Patel |
| Reece | Patel |
| Riana | Patel |
| S | Patel |
| Shriyam | Patel |
| Sujan | Patel |
| Nikolaos | Patelis |
| Muna | Patell |
| J | Patena Forte |
| Joana | Patena Forte |
| Amanda | Paterson |
| H | Paterson |
| Hugh | Paterson |
| Manish | Pathak |
| Prachi | Pathak |
| Samir | Pathak |
| Sohilkhan | Pathan |
| AM | Pathanki |
| C | Pathirannehalage Don |
| O | Pathmanaban |
| Omar | Pathmanaban |
| Keren | Pathmanathan |
| A | Patience |
| C | Patient |
| Manish | Patil |
| Ninad | Patil |
| R | Patil |
| Rakesh | Patil |
| S | Patil |
| Romeo | Patini |
| NG | Patino-Jaramillo |
| Amrita | Patkar |
| Pradnya | Patkar |
| S | Patkar |
| Shraddha | Patkar |
| P | Patki |
| D | Patkowski |
| Ioannis | Patoulias |
| Saroj | Patra |
| Daniela | Patricia Escalante Ureche |
| Ana | Patricia Legido Morán |
| Martha | Patricia Pérez de León Vázquez |
| B | Patrício |
| Andrea | Patrizi |
| R | Patrone |
| Renato | Patrone |
| P | Pattyn |
| Kathrin | Patzer |
| M | Pau |
| A | Paul |
| Claudia | Paul |
| Emila | Paul |
| Michaela | Paul |
| Rajesh | Paul |
| Sharmila | Paul |
| Marc | Paul Lopez |
| Christopher | Paul Millward |
| Jean | Paul Rugambwa |
| Jean | Paul Shumbusho |
| Ana | Paula Ferreira Pinto |
| Ana | Paula Riverola Aso |
| Henry | Paulino |
| Jeffrey | Paulino |
| Martin | Paulo |
| Luiz | Paulo Kowalski |
| João | Paulo Medici |
| Nipseey | Pauloe Candelario |
| Laerke | Paulsen |
| P | Paunero Vazquez |
| Patricia | Paunero Vazquez |
| Aleksandra | Paunovic |
| I | Paunovic |
| Ivan | Paunovic |
| Petra | Pavic Palac |
| Aswathy | Pavithran |
| W | Pavlis |
| Maja | Pavlov |
| Pavel | Pavlov |
| Ivana | Pavlovic |
| M | Pavlovic |
| O | Pavlovic |
| G | Pavone |
| V | Pavone |
| Jānis | Pāvulāns |
| Abhijit | Pawar |
| Neha | Pawar |
| P | Pawar |
| Pranay | Pawar |
| Shweta | Pawar |
| Carmen | Payá-Llorente |
| A | Payandeh |
| E | Payet |
| Eduardo | Payet |
| CJ | Payne |
| Maria | Paz Bohórquez-Tarazona |
| A | Paz-Aparicio |
| V | Pazin |
| T | Pazionis |
| Janneth | Pazmino-Canizares |
| Andrejs | Pcolkins |
| A | Pearce |
| Adrian | Pearce |
| J | Pearce |
| L | Pearce |
| J | Pearl |
| A | Pearson |
| Natasha | Pearson |
| F | Pecchia |
| Francesca | Pecchini |
| G | Peck |
| A | Peckham-Cooper |
| Adam | Peckham-Cooper |
| Alessandra | Pecoraro |
| Felice | Pecoraro |
| Abhinav | Pednekar |
| NF | Pedraza Alonso |
| C | Pedrazzani |
| Corrado | Pedrazzani |
| Joao | Pedreira Duprat Neto |
| R | Pedrini Cruz |
| Ricardo | Pedrini Cruz |
| José | Pedro |
| José | Pedro Fernandes dos Santos |
| João | Pedro Melo Neves |
| João | Pedro Reis |
| Leire | Pedrosa |
| R | Pedroso de Lima |
| Rita | Pedroso de Lima |
| Michał | Pędziwiatr |
| Shanell | Peeriyah |
| S | Peeroo |
| I | Peev |
| Igor | Peev |
| R | Peevor |
| Hrvoje | Pehar |
| A | Peig-Font |
| Christian | Peiper |
| C | Peirce |
| Colin | Peirce |
| Bryony | Peiris |
| S | Pejkova |
| Sofija | Pejkova |
| I | Pejovic |
| Ilija | Pejovic |
| MR | Pekcici |
| CÁ | Peláez Sánchez |
| PM | Pelaéz Torres |
| D | Pelaggi |
| Stéphane | Pelet |
| Aylin | Pelin Cil |
| G | Pelino |
| Arrigo | Pellacani |
| Alice | Pellegrini |
| L | Pellegrino |
| Gabriela | Pelletier |
| J | Pelletier |
| Massimiliano | Pelli |
| R | Pellini |
| Raul | Pellini |
| G | Pellino |
| Gianluca | Pellino |
| M | Pelloni |
| Maria | Pelloni |
| Roberto | Peltrini |
| C | Peluso |
| P | Pemmasani |
| Christina | Pempe |
| LG | Peña Balboa |
| Emmanuel | Peña Gómez Portugal |
| E | Pena Gomez-Portugal |
| Emilio | Peña Ros |
| GV | Peña Saltos |
| DS | Peñaherrera Toapaxi |
| Julio | Peñarrocha |
| Luka | Penezić |
| E | Peng |
| Ed | Peng |
| Pasi | Pengermä |
| Rachel | Pennington |
| Edgardo | Penserga |
| F | Pepe |
| Gilda | Pepe |
| Philip | Peprah Oppong |
| M | Peralta Ferreira |
| Marisa | Peralta Ferreira |
| Rajeev | Peravali |
| Digby | Percy |
| Eduardo | Perea del Pozo |
| J | Pereca |
| Jelizaveta | Pereca |
| Adela | Pereda |
| Mariana | Pereda |
| MR | Pereda |
| A | Pereira |
| André | Pereira |
| C | Pereira |
| Cristiana | Pereira |
| Mário | Pereira |
| R | Pereira |
| Rute | Pereira |
| FA | Pereira Júnior |
| A | Pereira Rodrigues |
| A | Pereira-Neves |
| António | Pereira-Neves |
| Diego | PereiraNuñez |
| Oleg | Perepelitsa |
| Eranga | Perera |
| R | Perera |
| Remei | Perera Sarri |
| Michal | Perets |
| Marine | Peretti |
| Irene | Perez |
| S | Perez |
| Y | Perez |
| Yolanda | Perez |
| Clara | Pérez |
| Marisol | Perez Cerdeira |
| DF | Perez Correa |
| Cristina | Pérez Costoya |
| MD | Perez Diaz |
| Marta | Pérez Febles |
| A | Perez Ferrer |
| Antonio | Perez Ferrer |
| M | Perez Gonzalez |
| Marta | Perez Gonzalez |
| Marina | Pérez González |
| Carolina | Perez Granados |
| Francisco | Pérez López |
| A | Pérez Núñez |
| Sara | Pérez Palao |
| C | Perez Rivera |
| Carlos J | Perez Rivera |
| CJ | Perez Rivera |
| Natalia | Pérez Romero |
| Álvaro | Pérez Rubio |
| L | Pérez Santiago |
| Leticia | Pérez Santiago |
| V | Pérez Simón |
| Rafael | Perez Vidal |
| J | Perez Villena |
| Joan | Perez Villena |
| S | Perez-Bertolez |
| Sonia | Pérez-Bertólez |
| H | Perez-Chrzanowska |
| Hanna | Perez-Chrzanowska |
| B | Perez-Lozana |
| B | Pérez-Saborido |
| Baltasar | Pérez-Saborido |
| LE | Pérez-Sánchez |
| A | Perfecto |
| Ilaria | Pergolini |
| V | Peri |
| N | Periard |
| Ivan | Perić |
| S | Pericleous |
| Marcos | Perini |
| R | Perinotti |
| Roberto | Perinotti |
| Nuria | Peris |
| Gordan | Perišić |
| D | Peristeri |
| K | Perivoliotis |
| Konstantinos | Perivoliotis |
| C | Perkins |
| R | Perkins |
| Romeeka | Perkins |
| G | Peros |
| Georgios | Peros |
| LO | Perotto |
| M | Perovic |
| T | Perra |
| Teresa | Perra |
| Konstantinos | Perreas |
| Alexandra | Perricos |
| D | Perrina |
| AM | Perrone |
| Fabrizio | Perrone |
| G | Perrone |
| Gennaro | Perrone |
| Ophelie | Perrot |
| M | Perry |
| A | Persad |
| Amit | Persad |
| Roberto | Persiani |
| M | Pertea |
| D | Pertile |
| Davide | Pertile |
| Abirami | Perumal Kanniappan |
| A | Perutelli |
| Arsalan | Pervaiz |
| A | Peryt |
| F | Pesant |
| MA | Pesántez Peralta |
| A | Pesce |
| Vito | Pesce |
| T | Peschel |
| Giovanni | Pesenti |
| Vishal | Peshattiwar |
| Laura | Pesquera |
| P | Pessaux |
| Patrick | Pessaux |
| Maximilian | Pesta |
| Amit | Peswani |
| L | Petagna |
| Lorenzo | Petagna |
| M | Peteja |
| Matúš | Peteja |
| Chidiebere | Peter Echieh |
| Osborne | Peter Vaz |
| Luke | Peters |
| Paul | Peters |
| SM | Peters |
| ML | Petersen |
| Josefin | Petersson |
| Stamatios | Petousis |
| F | Petraglia |
| Nikki | Petrakis |
| Donatas | Petrauskas |
| Konstantin | Petrenko |
| A | Petrillo |
| Marco | Petrillo |
| P | Petrone |
| Konstantinos | Petropoulos |
| R | Petrov |
| Biljana | Petrovic |
| Milan | Petrovic |
| Alexandru | Petrusan |
| Bogdan | Petruț |
| G | Petruzzi |
| Gerardo | Petruzzi |
| Andrea | Petzold |
| M | Peycelon |
| Inês | Peyroteo |
| Mahdi | Pezeshki Modarres |
| I | Pezzoli |
| F | Pezzolla |
| AP | Pezzuto |
| AC | Pfaff |
| Carlos | Pfingst Rojas |
| K | Pfister |
| Karin | Pfister |
| J | Pfuner |
| A | Phadnis |
| Ashish | Phadnis |
| H | Pham |
| Hong | Pham |
| Terence | Pham |
| Du | Phan |
| YC | Phan |
| Isaac | Phang |
| Ajay | Philip |
| Ken | Philip |
| Melissa | Philip |
| T | Philip |
| Mark | Philip Hehir |
| Anupam | Phillip |
| A | Phillips |
| AW | Phillips |
| Drew | Phillips |
| Edward | Phillips |
| Emil | Phillips |
| J | Phillips |
| Jonathan | Phillips |
| JR A | Phillips |
| Rachel | Phillips |
| G | Philouze |
| M | Philp |
| J | Phull |
| Daniel | Phung |
| Athanasios | Piachas |
| R | Piagnerelli |
| C | Piazza |
| M | Piazza |
| AL | Picardo |
| Antonio L | Picardo |
| Miriana | Picariello |
| S | Picazo |
| Eduardo | Picazo Pineda |
| Fernando | Picazo Pineda |
| Andrea | Picchetto |
| A | Picciariello |
| Arcangelo | Picciariello |
| M | Piccino |
| M | Piccirillo |
| Micaela | Piccoli |
| C | Piceni |
| Merycarla | Pichardo |
| Barbara | Pichi |
| C | Pichler de Oliveira |
| Cora | Pichler de Oliveira |
| H | Pickard |
| L | Pickering |
| L | Pickett |
| Héctor | Picon molina |
| R | Picón Rodríguez |
| Rafael | Picón Rodríguez |
| Emanuele | Picone |
| V | Picotti |
| Veronica | Picotti |
| Samuel | Pie |
| Emil | Pieniowski |
| Alberto | Pieretti |
| F | Pieri |
| Stefano | Piero Bernardo Cioffi |
| Trocard | Pierre |
| G | Piessen |
| Guillaume | Piessen |
| L | Pieteris |
| Linas | Pieteris |
| A | Pietramala |
| G | Pietrobon |
| Giacomo | Pietrobon |
| N | Pigadas |
| Manon | Pigeolet |
| G | Pignata |
| L | Pignataro |
| Lorenzo | Pignataro |
| M | Pignatti |
| Marco | Pignatti |
| Lucinda | Pigott |
| N | Pijanovic |
| Nemanja | Pijanovic |
| C | Pijoan-Lara |
| E | Pikoulis |
| Elima | Pilar Cagigal Ortega |
| María | Pilar Camacho Carrasco |
| Claudia | Pilar Clemente Tomas |
| Adam | Pilarski |
| I | Pilic |
| I | Pilkington |
| Silja | Pillai |
| Srikumar | Pillai |
| Nivashen | Pillay |
| Robert | Pillerstorff |
| J | Pilling |
| M | Piloni |
| Martina | Piloni |
| G | Pilu |
| Gianluigi | Pilu |
| AS | Pimienta Ibarra |
| T | Pina-Vaz |
| Teresa | Pina-Vaz |
| Ugo | Pinar |
| Camila | Pincheira |
| S | Pincott |
| Anna | Pineau |
| Esther | Pinfold |
| Hock | Ping Cheah |
| L | Pingarrón-Martin |
| Gabriela | Pinheiro |
| J | Pinheiro Santos |
| S | Pinho |
| Sílvia | Pinho |
| RE | Pinilla |
| RE | Pinilla Morales |
| Muni | Pinjala |
| Veronica | Pino Diaz |
| E | Pinotti |
| Enrico | Pinotti |
| Agustin | Pinsak |
| Amanda | Pinto |
| Fabio | Pinto |
| J | Pinto |
| José | Pinto |
| Pilar | Pinto |
| V | Pinto |
| Valentina | Pinto |
| Victor | Pinto Angulo |
| VM | Pinto-Angulo |
| José | Pintor-Tortolero |
| Celestino | Pio Lombardi |
| Clarence | Pio Rey Yacapin |
| A | Pipara |
| Amrit | Pipara |
| RJ | Piper |
| NS | Pipitone Federico |
| Mirzemagomed | Pirakhmedov |
| Antonio | Piras |
| Kanapathipillai | Piratheep |
| Franco | Piredda |
| Erick | Pires Ferreira |
| Ivonizete | Pires Ribeiro |
| Alessandro | Pirina |
| Setareh | Pirmorad |
| Francesca | Pirola |
| GM | Pirola |
| Laszlo | Piros |
| Nello | Pirozzi |
| G | Pirozzolo |
| Giovanni | Pirozzolo |
| Roberto | Pirrello |
| A | Pirzada |
| Darryl | Pisani |
| Michele | Pisano |
| A | Pisanu |
| Adolfo | Pisanu |
| F | Pisanu |
| Francesco | Pisanu |
| Givi | Pisarevi |
| Lorenz | Pisecky |
| E | Pişkin |
| Erol | Pişkin |
| T | Pissanou |
| Meron | Pitcher |
| Joel | Pitkänen |
| L | Pitoni |
| Bradley | Pittam |
| Idoko | Pius Ogolekwu |
| MJ | Pizarro |
| P | Pizzini |
| Kerasia-Maria | Plachouri |
| Puneet | Plaha |
| Jordi | Planelles Gómez |
| Carole | Plante |
| Marie | Plante |
| Philipp | Plarre |
| Maria | Plata |
| Julio | Plata-Bello |
| Rebecca | Platoff |
| Guillermo | Plaza |
| Cristian | Plaza Valiente |
| Stefan | Plontke |
| J | Ploski |
| Jennifer | Ploski |
| Katherine | Plua |
| Artem | Pobelenko |
| José | Poblete Carrizo |
| Marc | Pocard |
| Adriana | Poch |
| P | Pockney |
| Madalina-Claudia | Pocol |
| M | Podda |
| Mauro | Podda |
| P | Poddar |
| Guillaume | Podevin |
| D | Podolsky |
| R | Poelstra |
| C | Poggi |
| Catalina | Poggi |
| J | Poggio |
| G | Poggioli |
| Gilberto | Poggioli |
| Elia | Poiasina |
| Bernhard | Poidinger |
| Nikolaus | Poier |
| Harshwardhan | Pokharkar |
| Martha | Poku |
| L | Pol-Fachin |
| Laercio | Pol-Fachin |
| G | Pola Bandres |
| Guillermo | Pola Bandres |
| Verónica | Polaino |
| Lucía | Polanco Pujol |
| CA | Polania Sandoval |
| S | Polat |
| Süleyman | Polat |
| Natasa | Poldan Grabar |
| E | Poletto |
| T | Poli |
| A | Police |
| Andrea | Police |
| Antonella | Polimeni |
| A | Politi |
| Esteban | Politi Vidal |
| C | Politis |
| D | Politis |
| Dimitrios | Politis |
| S | Politis |
| S | Pollesel |
| Tommaso | Pollini |
| JM | Pollok |
| E | Poluyi |
| Andreas | Polydorou |
| Leo | Pölzl |
| C | Pompili |
| Cecilia | Pompili |
| Luca | Ponchietti |
| Sabarirajan | Ponnusamy |
| Aleix | Pons Bartroli |
| L | Pons Pellicé |
| Alex | Ponson |
| Jose | Pontes Junior |
| N | Ponugoti |
| Nikhil | Ponugoti |
| Rita | Poon |
| I | Pop |
| I | Popescu |
| Irinel | Popescu |
| SG | Popeskou |
| A | Popoola |
| Ademola | Popoola |
| D | Popova |
| Maria | Popp |
| Francesco | Porcelli |
| A | Porcu |
| Alberto | Porcu |
| Bálint | Pordány |
| Andrea | Porta |
| L | Porteiro Mariño |
| Lucia | Porteiro Mariño |
| Carmen | Portenkirchner |
| Anna | Porter |
| AL | Portilla |
| M | Portinari |
| Vlad | Porumb |
| JA | Posada |
| OE | Posadas-Trujillo |
| T | Poskus |
| Tomas | Poskus |
| L | Posma-Bouman |
| Lisanne | Posma-Bouman |
| N | Post |
| Spyridon | Potamianos |
| Ojas | Potdar |
| Amit | Pothare |
| Stojan | Potrč |
| R | Potter |
| Ryan | Potter |
| A | Potts |
| S | Pou Macayo |
| Frederic | Pouliot |
| T | Poulton |
| R | Pourahmad |
| M | Pourfridoni |
| S | Pourhedayat |
| Arvid | Pourlotfi |
| D | Pournaras |
| Dimitri | Pournaras |
| Andrés | Pouy |
| Andrej | Povalij |
| S | Powell |
| Simon | Powell |
| E | Powell-Smith |
| Nicholas | Power |
| Dilroop | Poyyil |
| Carolina | Pozo |
| P | Pozo Quispe |
| Ioanna | Pozotou |
| Mirko | Pozzoni |
| Jorge | Prada |
| GM | Prada Hervella |
| IS | Pradeep |
| C | Praetorius |
| Christian | Praetorius |
| Mark | Praetorius |
| Prabhat | Prakash Narayan |
| Sankhya | Prakash Vel |
| C S | Pramesh |
| CS | Pramesh |
| B | Pramodana |
| L | Prantl |
| Lukas | Prantl |
| Aveechal | Prasad |
| Navakoti | Prasad |
| Shalvin | Prasad |
| Sathyamoorthy | Prasanna |
| Nil | Prat |
| J | Prat Ortells |
| Jordi | Prat-Ortells |
| Dedy | Pratama |
| D | Prce |
| C | Predoi |
| R | Preece |
| Ryan | Preece |
| S | Pregnolato |
| Y | Premakumar |
| AB | Prempeh |
| Agya | Prempeh |
| J | Presl |
| Jaroslav | Presl |
| Michael | Preston |
| AL | Preto Barreira |
| J | Pretorius |
| Drago | Prgomet |
| Martin | Přibyl |
| Benjamin | Price |
| C | Price |
| Cheri | Price |
| T | Price |
| Thea | Price |
| Veronique | Price |
| Ruth S | Prichard |
| M | Priemel |
| Matthias | Priemel |
| M | Prieto |
| Mikel | Prieto |
| M | Prieto Calvo |
| MI | Prieto Nieto |
| Rodrigo | Prieto-Aldape |
| N | Prijović |
| Nebojša | Prijović |
| Florian | Primavesi |
| Heather | Pringle |
| S | Pringle |
| Shirley | Pringle |
| R | Pritchard |
| Anna | Privratsky |
| Noopur | Priya |
| Vijayam | Priya Nair |
| Pratyusha | Priyadarshini |
| Priyadarshini | Priyadarshini |
| Affan | Priyambodo |
| Sujeewa | Priyantha Bandara Thalgaspitiya |
| E | Proaño |
| JA | Proaño-Zamudio |
| Chris | Probst |
| P | Probst |
| A | Prodromidou |
| Anastasia | Prodromidou |
| A | Pronk |
| Alejandro | Prosperi |
| P | Prosperi |
| Paolo | Prosperi |
| Doriane | Prost |
| M | Protic |
| PL | Proto |
| D | Proud |
| David | Proud |
| Arina | Provozina |
| Ratko | Prstacic |
| Andreja | Prtorić |
| GM | Prucher |
| V | Pruiti Ciarello |
| Vincenzo | Pruiti Ciarello |
| Lydia | Prusty |
| FR | Pruvot |
| C | Pryce |
| Michail | Psarologos |
| Dimitris | Psychogios |
| Ning | Pu |
| Marco | Puccini |
| Caterina | Puccioni |
| P | Puchwein |
| Paul | Puchwein |
| Emma | Puertas Ruiz |
| Angela | Puerto |
| A | Pueyo Ferrer |
| Alfredo | Pueyo Ferrer |
| Alberto | Pueyo Rabanal |
| Jonathan | Pugh |
| Pierfrancesco | Pugliese |
| M | Puglisi |
| JJ | Puig Galy |
| Marcel | Pujadas |
| Antonio | Pujante |
| Natalia | Pujol Cano |
| R | Pujol Muncunill |
| Rosa | Pujol Pina |
| N | Pujol-Cano |
| R | Pujol-Muncunill |
| H | Pülat |
| Eliana | Pulido |
| C | Puma Pagliarello |
| Calogero | Puma Pagliarello |
| Rodriguez | Pumarol Próspero Enrique |
| Oliver | Pumphrey |
| Diana | Puozaa |
| A | Puppo |
| Andrea | Puppo |
| Ajay | Puri |
| A | Purohit |
| Irina-Maria | Puscas |
| Silke | Pusch von |
| J | Pushpa-rajah |
| Kameshwarachari | Pushpalatha |
| S | Putnis |
| Soni | Putnis |
| Vadim | Pykhteev |
| Amjad | Qabbani |
| Kawthar | Qader |
| S | Qaderi |
| MA | Qadri |
| Shahin | Qadri |
| ST | Qadri |
| Qutaiba | Qafisha |
| Mubasher A | Qamar |
| H | Qandeel |
| Haitham | Qandeel |
| Mahmoud | Qandeel |
| Layth | Qaraqe |
| A | Qasem |
| Abdulrahman | Qasem |
| Ahmad | Qasim |
| Dalia | Qasrawi |
| Faisal | Qassem |
| Mohamed | Qassem |
| MS | Qatora |
| Abid | Qazi |
| Mehdi | Qiabi |
| Cheng | Qian |
| J | Qiao |
| Kirby | Qin |
| KR | Qin |
| Joo | Qing Cheng |
| Alex | Qinyang Liu |
| Yaman | Qoudra Danial |
| Helen | Quah |
| Kofi | Quansah |
| M | Quante |
| Markus | Quante |
| Elizabeth | Quartson |
| Mohamad | Qudah |
| B | Quddus |
| G | Querini |
| J | Querolt Coll |
| Jordi | Querolt Coll |
| R | Quevedo |
| Julien | Quilichini |
| Ralph | Quillin |
| Andrew | Quin |
| Eduardo | Quiñónez Lorenzana |
| E | Quint |
| M | Quintana |
| Natalia | Quintana |
| B | Quintana-Villamandos |
| Begoña | Quintana-Villamandos |
| J | Quintens |
| Ma Andrea | Quintero-Ortíz |
| Claudia | Quintero-Pérez |
| Saray | Quinto |
| Valeria | Quintodei |
| Ned | Quirke |
| Sinead | Quirke |
| Raquel | Quiroga |
| Manuel | Quiroz |
| Kewin | Quispe de la Roca |
| JF | Quispe Mateo |
| Zaki | Qulaghassi |
| Yazen | Qumsiyeh |
| Meysoon | Qurashi |
| Homeira | Qureischie |
| A | Qureishi |
| A | Qureshi |
| Ahmad | Qureshi |
| AU | Qureshi |
| Maryum | Qureshi |
| Sajid | Qureshi |
| I | R Fakhradiyev |
| P | R Oliveira |
| A | Rababah |
| Asmaa | Rababah |
| Lorenzo | Rabadan |
| Mahtab | Rabbani Anari |
| Mohamed | Rabea |
| Mohamed | Rabie |
| Salma | Rabie |
| Igor | Rabin |
| Will | Raby-Smith |
| Louie | Racelis |
| J | Rachadell |
| Juan | Rachadell |
| Nur | Rachmat Lubis |
| M | Racine |
| D | Radenkovic |
| Dejan | Radenkovic |
| A | Radhakrishnan |
| Ajay | Radhakrishnan |
| Mohamed | Radhi |
| M | Radojevic |
| Zeljko | Radojkovic |
| Rudolf | Radojković |
| M | Radosavljevic |
| Milena | Radosavljevic |
| Ishan | Radotra |
| Dragana | Radovanovic |
| Zoran | Radovanovic |
| Dana | Radu |
| Pisica | Radu-Mihai |
| O | Radulova-Mauersberger |
| Asmaa | Radwan |
| Emily | Rady |
| M | Rady |
| Fraser | Rae |
| L | Rae |
| Hazim | Raed |
| Marianna | Raevskaya |
| Henry | Rafael Acosta Castro |
| Mario | Rafael Medina Hernández |
| Tiago | Rafael Onzi |
| Tanvir | Rafe |
| A | Raffaele |
| A | Raffone |
| Yaseen | Rafi |
| A | Rafique |
| Atif | Rafique |
| MN | Rafique |
| Abdelrahman | Ragab |
| Mohamed | Ragab |
| Marawan | Ragal |
| Mahmoud | Raggad |
| Vidya | Raghavan |
| Franco | Ragni |
| K | Ragupathy |
| Kalpana | Ragupathy |
| Casimir FP | Rahantasoa Finaritra |
| N | Rahbari |
| Nuh | Rahbari |
| F | Rahim |
| Ferdous | Rahim |
| Rehana | Rahim |
| Sybghat | Rahim |
| M | Rahimi |
| Mana | Rahimzadeh |
| Atiqur | Rahman |
| Ayesha | Rahman |
| GA | Rahman |
| Khalid | Rahman |
| M | Rahman |
| Mohammad | Rahman |
| R | Rahman |
| S | Rahman |
| Abdel | Rahman Ashraf |
| A | Rahman Mitul |
| Ashrarur | Rahman Mitul |
| Abdel | Rahman Mohannad Ahmad Alwardat |
| Faizan | Rahmani |
| Yazan | Rahmeh |
| AC | Rahy-Martín |
| A | Rai |
| B | Rai |
| DAS | Rai |
| L | Rai |
| Lajpat | Rai |
| S | Rai |
| D | Raimondo |
| Diego | Raimondo |
| I | Raimondo |
| Christopher | Raine |
| Ryan | rainiel Abary |
| Amit | Raithatha |
| N | Raj |
| Sumit | Raj |
| Mohana | Raj Thanapal |
| H | Raja |
| Nancy | Raja |
| Roopak | Raja |
| K | Raja Shabbir |
| Parisa | Rajaei |
| Niranjana | Rajagopal |
| P | Rajagopal |
| A | Rajagopalan |
| Saravanan | Rajakumar |
| Firoz | Rajan |
| S | Rajan |
| Shiv | Rajan |
| A | Rajanbabu |
| Anupama | Rajanbabu |
| Tsirimalala | Rajaobelison |
| K | Rajaratnam |
| N | Rajaretnam |
| Niroshini | Rajaretnam |
| JS | Rajasekar |
| Mohsen | Rajati |
| Kishan | Rajdev |
| Srujan | Rajesh |
| AD | Rajgor |
| Amarkumar D | Rajgor |
| HO | Raji |
| A | Rajpura |
| Asim | Rajpura |
| Deepak | Rajput |
| K | Rajput |
| Sunil | Rajput |
| D | Raju |
| Mohamad | Rakka |
| K | Rakoczy |
| Gergely | Rakos |
| Haritiana | Rakotoarisoa |
| I | Rakvin |
| A | Raluca-Cristina |
| Apostu | Raluca-Cristina |
| Ruchi | Ram |
| Gautam | Ram Choudhary |
| Dharma | Ram Poonia |
| Jeewan | Ram Vishnoi |
| Nuno | Rama |
| Abdelrahman | Ramadan |
| Dina | Ramadan |
| Rana | Ramadan |
| Sara | Ramadan |
| MA | Ramadhan |
| Pooja | Ramakant |
| Aravindh | Ramalingam |
| I | Ramallo-Solís |
| Irene | Ramallo-Solís |
| R | Ramamoorthy |
| Jaishankar | Raman |
| Archana | Ramaswamy |
| Kamarajan | Ramayah |
| Makhmud | Ramazanov |
| Sean | Ramcharan |
| R | Ramely |
| A | Ramesh |
| Ashwanth | Ramesh |
| P | Ramesh Menon |
| S | Ramezani |
| Maharo | Ramifehiarivo |
| L | Ramírez |
| Rossana | Ramírez |
| Erika M | Ramírez Amaya |
| P | Ramirez Nieto |
| Pablo | Ramirez Romero |
| LJ | Ramirez-Nuñez |
| Nalla | Ramji Narendra |
| S | Ramjit |
| ARH | Ramli |
| R | Ramli |
| Roszalina | Ramli |
| A | Rammohan |
| Ashwin | Rammohan |
| Arvind | Rammohun |
| Catalina | Ramon Barcelo |
| Juan | Ramón Gómez López |
| Felix | Ramon Montes |
| Jose | Ramon Oliver Guillen |
| Juan | Ramón Sanz |
| Carina | Ramos |
| CL | Ramos |
| D | Ramos |
| German | Ramos |
| Kirsten | Ramos |
| MFKP | Ramos |
| Nuno | Ramos |
| P | Ramos |
| Patricia | Ramos |
| Pedro | Ramos |
| R A | Ramos |
| Rodrigo | Ramos |
| A | Ramos Bonilla |
| Antonio | Ramos Bonilla |
| A | Ramos De La Medina |
| Antonio | Ramos De La Medina |
| Raul | Ramos mange |
| Andrea | Ramos Mantilla |
| JL | Ramos rodriguez |
| A | Ramos-De la Medina |
| Antonio | Ramos-De la Medina |
| Adolfo | Ramos-Luengo |
| P | Ramos-Martin |
| Ritika | Rampal |
| Ganeshan | Ramsamy |
| Akash | Ramsaroop |
| L | Ramsay |
| W | Ramsey |
| Shivanand | Ramsubhagh |
| J | Ramzi |
| Ahmad | Ramzi Yusoff |
| Eyal | Ran Nachum |
| Reesha | Ranat |
| Probhodana | Ranaweera |
| Alin | Rancea |
| Jonathan | Randall |
| T | Randau |
| Thomas | Randau |
| A | Rangan |
| Amar | Rangan |
| Hardlife | Ranganai |
| J | Rani |
| Pallavi | Rani |
| M | Ranisavljević |
| Jyothi KR | Ranjan |
| Mark | Ranjan Jesudason |
| Smruti | Ranjan Mohanty |
| Gyan | Ranjan Singh |
| Kul | Ranjan Singh |
| S | Ranjit |
| Srinath | Ranjit |
| Anuradha | Rao |
| Dominic | Rao |
| Milind | Rao |
| Rohith | Rao |
| Suresh | Rao |
| Nayzak | Raoof |
| Sarah | Rapaport |
| F | Rapetto |
| Filippo | Rapetto |
| J | Rapp |
| DA | Raptis |
| C | Rarras |
| Mamisoa B | Rasamoelina |
| Hanta | Rasataharifetra |
| Abdallah | Rashad Temerik |
| Yasir | Rasheed |
| I | Rashid |
| Isbah | Rashid |
| Madeeha | Rashid |
| MM | Rashid |
| Pueya | Rashid Nashidengo |
| M | Rashidbeygi |
| Mohammed | Rashwan |
| Slobodan | Rašić |
| Hasan | Raslan |
| Fanjandrainy | Rasoaherinomenjanahary |
| Iqbal | rasool Wani |
| Karolina | Rasoul-Pelińska |
| F | Raspagliesi |
| Francesco | Raspagliesi |
| Melroy | Rasquinha |
| J | Rassam |
| R | Rasschaert |
| Ricky | Rasschaert |
| M | Rassweiler-Seyfried |
| Marie-Claire | Rassweiler-Seyfried |
| Pejana | Rastović |
| Sebastian | Rath |
| S | Rathinam |
| Sridhar | Rathinam |
| Kirtikumar J | Rathod |
| Tõnu | Rätsep |
| R | Rattan |
| Rishi | Rattan |
| Pornjittra | Rattanasirivilai |
| Francesca | Ratti |
| Deviney | Rattigan |
| S | Rattizzato |
| Simone | Rattizzato |
| C | Ratto |
| Carlo | Ratto |
| Varkha | Rattu |
| K | Raubenheimer |
| Kyle | Raubenheimer |
| Teresa | Rauchegger |
| F | Rauf |
| Yaseen | Rauf |
| Abdul | Rauf bin Ahmad |
| Diego | Raul Abente Arriola |
| Jordi | Raurich-Leandro |
| E | Rausa |
| C | Raut |
| Chandrajit | Raut |
| CP | Raut |
| M | Raut |
| Monish | Raut |
| M | Ravaioli |
| Matteo | Ravaioli |
| S | Raveendran |
| G | Ravenni |
| Selina | Ravenscroft |
| Akshaya | Ravi |
| N | Ravi |
| S | Ravindrakumar |
| S | Ravindran |
| R | Ravindranath Nambiar |
| David | Rawaf |
| Faisal | Rawagah |
| Arab | Rawashdeh |
| Shireen | Rawashdeh |
| J | Ray |
| Jaydip | Ray |
| S | Rayamajhi |
| T | Raymond |
| A | Rayner |
| Anthony | Rayner |
| Tom | Rayner |
| Fadi | Rayya |
| M | Rayzah |
| Musaed | Rayzah |
| Ahsan | Raza |
| Ali | Raza |
| M | Raza |
| N | Raza |
| Syed | Raza |
| Waqas | Raza |
| Ali | Raza Malik |
| Jeannie BA | Razafindrahita |
| Mohd | Razali Ibrahim |
| A | Razik |
| Naila | Raziq |
| Syed | Raziuddin Biyabani |
| Ahmad | Razouk |
| Radu | Razvan Scurtu |
| Hassan | Razvi |
| Alessandra | Razzaboni |
| A | Razzore |
| Fatima | Razzouk |
| F | Ré |
| Matthew | Read |
| Pedro | Recabal |
| Daniel | Rech |
| Ewa | Rechberger |
| G | Recinos |
| Gustavo | Recinos |
| Salvador | Recinos |
| Adrián | Recio Ayesa |
| F | Recker |
| Florian | Recker |
| A | Recordare |
| Souheil | Reda |
| Mohamed | Reda Loaloa |
| Madhuri | Reddy |
| Saiesh | Reddy Voppuru |
| Roberta | Redfern |
| Rajeev | Redkar |
| NOT | Rees |
| J | Reeves |
| Motasem | Refaat |
| Mohammed | Refaat Ibrahiem Amin El Ghalid |
| Basel | Refky |
| Daniela | Rega |
| Trina | Regalado |
| Guillermo | Regalo |
| Carmen | Regan |
| N | Regenet |
| Nicolas | Regenet |
| V | Reghuram |
| Carlos | Régil |
| Maria | Regina Alvarez |
| Lia | Regina de Sampaio |
| J | Reguera-Rosal |
| Abdul | Rehman |
| Hina | Rehman |
| K | Rehman |
| Riaz | RehmAn |
| S | Rehman |
| Abdur | Rehman Malik |
| Martin | Reichert |
| Daniel | Reichhold |
| J | Reid |
| Jennifer | Reid |
| Jeremy | Reid |
| Matthew | Reid |
| Rebecca | Reid |
| S | Reid |
| J | Reilly |
| John-Joe | Reilly |
| D | Reim |
| Daniel | Reim |
| Daniel | Reimer |
| Dietmar | Reinaldo |
| T | Reinhard |
| Tobias | Reinhard |
| C | Reinke |
| Caroline | Reinke |
| F | Reinkemeier |
| FJ | Reinoso |
| I | Reis |
| Igor | Reis |
| ME | Reis |
| Janani | Reisenauer |
| Anne | Reiss Axelsen |
| C | Reissfelder |
| Christoph | Reissfelder |
| A | Reiter |
| Christian | Reiterer |
| M | Rela |
| Mohamed | Rela |
| M | Reljic |
| Milorad | Reljic |
| Panagiota | Rellia |
| J | Relwani |
| Jai | Relwani |
| Xabier | Remirez Arriaga |
| Dong-Lin | Ren |
| Gabriel | Renan Soares Rodrigues |
| M | Renau-Cerrillo |
| Marina | Renau-Cerrillo |
| Elizabeth | Renaud |
| Annaëlle | Renault |
| Luke | Render |
| Vladimir | Rendevski |
| Juliana | Rendón Hernández |
| Walter | Rene Fretes Gonzalez |
| Mauricio | Rene Hernandez |
| Agustina | Rene Oliva |
| Herald | Rene Segovia Lohse |
| Teresa | Renedo Villar |
| A | Renne |
| Sarah | Rennie |
| M | Rennis |
| P | Renovell Ferrer |
| Pablo | Renovell Ferrer |
| E | Renza-Stingone |
| Adolfo | Renzi |
| Islamic | Rep |
| Luca | Resca |
| E | Restini |
| Enrico | Restini |
| Joel | Reuben Abel |
| T | Revez |
| Tatiana | Revez |
| Remedios | Revilla Amores |
| Julia | Revuelta Ramírez |
| Rocio | Revuelta Zorrilla |
| MC | Rey |
| S | Rey |
| C | Rey Valcarcel |
| Cristina | Rey Valcarcel |
| J | Rey-Biel |
| C | Rey-Valcarcel |
| Faten | Reyad Bani Hamad |
| Carmen | Reyero Fernández |
| Emilio | Reyes |
| GP | Reyes |
| JA S | Reyes |
| JAS | Reyes |
| JT | Reyes |
| Rudeily | Reyes |
| Albert | Reyes Claret |
| Adriana | Reyes Echeverría |
| MDC | Reyes Puig |
| E | Reyes Rodriguez |
| Carlos | Reyes Utrera |
| IS | Reynolds |
| J | Reynolds |
| JV | Reynolds |
| C | Reynoldson |
| Charmian | Reynoldson |
| M | Rezacova |
| M | Rezaei Tavirani |
| D | Rezaie |
| Daniel | Rezaie |
| Esmaeil | Rezghi Maleki |
| Mohamed | Rezk |
| M | Rghioui |
| Mounir | Rghioui |
| Jae | Rhee |
| HL | Rhodes |
| M | Riad |
| Mahmud | Riad |
| Xiana | Rial |
| Romualdas | Riauka |
| Kazim | Riaz |
| Sidra | Riaz |
| Luisana | Riba Combatti |
| M | Riba Martinez |
| Mireia | Riba Martínez |
| A | Ribeiro |
| Ana | Ribeiro |
| Barbara | Ribeiro |
| J | Ribeiro |
| Margarida | Ribeiro |
| R | Ribeiro |
| Ricardo | Ribeiro |
| Rui | Ribeiro |
| T | Ribeiro |
| VI | Ribeiro |
| U | Ribeiro Jr |
| U | Ribeiro Junior |
| R | Ribeiro Meduna |
| Rafael | Ribeiro Meduna |
| M | Ribolla |
| D | Ribuffo |
| Diego | Ribuffo |
| Guijarro-Jorge | Ricardo |
| David | Ricardo Herrera Mora |
| José | Ricardo Negrete Ocampo |
| Luis | Ricardo Ramirez Gonzalez |
| Andre | Ricardo Stüker |
| Vincenzo | Ricchiuti |
| Claudio | Ricci |
| S | Ricci |
| Silvia | Ricci |
| Pietro | Ricciardi |
| Sara | Ricciardi |
| G | Riccioli |
| Alexandra | Rice |
| D | Rice |
| HE | Rice |
| Henry E | Rice |
| SE | Rice-Townsend |
| Harvey | Rich |
| Dylan | Richard Barnett |
| Stephen | Richard Knight |
| J | Richards |
| S | Richards |
| T | Richards |
| Toby | Richards |
| P | Richebé |
| J | Riches |
| MC | Richir |
| Lysia | Richmond |
| M | Richmond |
| E | Richtig |
| Francesco | Ricotta |
| Mohammed | Rida |
| Sophie | Riddell |
| GE | Riddiough |
| Georgina | Riddiough |
| A | Ridgway |
| Paul | Ridgway |
| PF | Ridgway |
| Johannes | Riecke |
| P | Riedl |
| M | Riehan |
| Caroline | Rieser |
| John | Rietveld |
| Ebaa | Rifai |
| O | Riffi |
| J | Rigaud |
| Jerome | Rigaud |
| E | Righini |
| Caitlin | Rigler |
| L | Rigueros Springford |
| Karolin | Riips |
| Sushil | Rijal |
| C | Riley |
| Christopher | Riley |
| Lara | Rimmer |
| R | Rimonda |
| Roberto | Rimonda |
| Mauro | Rinaldi |
| MN | Ringressi |
| J | Rio |
| Javier | Rio |
| L | Rio Rodrigues |
| July | Ríos |
| J | Rios Chiuyari |
| Jose | Rios Chiuyari |
| AJ | Rios-Diaz |
| Gabriel | Ríos-Samper |
| Victor | Ripardo Siqueira |
| Brianda | Ripoll |
| Francisco | Ripoll Vidal |
| J | Ripollés-Melchor |
| Javier | Ripollés-Melchor |
| F | Ris |
| M | Risaliti |
| Mohamed | Rishard |
| OB F | Risk |
| Razan | Rislan |
| Stefan | Riss |
| A | Rissmann |
| Anke | Rissmann |
| T | Risteski |
| Anna | Rita Tanca |
| Ana | Rita Teles |
| Otávio | Ritter Silveira Martins |
| Tiago | Riuji Ijichi |
| FD | Rivadeneira Proano |
| D | Rivas |
| F | Rivas |
| J | Rivas |
| Julio | Rivas |
| R | Rivas |
| Ruben | Rivas |
| Barbara | Rivera |
| Pablo | Rivera |
| RD | Rivera |
| Teresa | Rivera Schmitz |
| D | Rivera-Alonso |
| Joaquín | Rivero Déniz |
| Anas | Riyahi |
| H | Riyat |
| M | Rizk |
| S | Rizvi |
| Andrea | Rizzi |
| Davide | Rizzo |
| Roberta | Rizzo |
| V | Rizzo |
| Angelica | Rizzoli |
| Ahmad | Rmman |
| Justin | Roake |
| Jun-Neng | Roan |
| CE | Roata |
| D | Robayo |
| Lydia | Robb |
| C | Robba |
| Matthew | Robert Marples |
| P | Roberto andrea |
| Miguel | Roberto Li Valencia |
| Luis | Roberto Nadal |
| Juan | Roberto Torres Cisneros |
| Carwyn | Roberts |
| D | Roberts |
| Jayson | Roberts |
| Keith | Roberts |
| L | Roberts |
| Laura | Roberts |
| M | Roberts |
| Matthew | Roberts |
| Tobias | Roberts |
| Phoebe | Robertson |
| R | Robertson |
| S | Robertson |
| Vaila | Robertson |
| B | Robertson-Smith |
| Fabien | Robin |
| Nicole | Robin |
| A | Robin Valle de Lersundi |
| Alvaro | Robin Valle de Lersundi |
| B | Robinson |
| RJ | Robitsek |
| Carlos | Robles Vidal |
| A | Robson |
| ML | Robuschi |
| Nicole | Robyn Bangayan |
| Andrea | Roca |
| A | Rocca |
| M | Rocha Melo |
| Miguel | Rocha Melo |
| João | Rocha-Neves |
| EC | Roche |
| Emny | Rochell Bobadilla Romero |
| M | Rochon |
| Thelma | Rocío Jiménez Mosquea |
| Peter | Rock |
| Alejandra | Rodas |
| Lesly | Rodas |
| S | Rodimov |
| Sergey | Rodimov |
| K | Roditis |
| Konstantinos | Roditis |
| K | Rodkey |
| A | Rodolakis |
| Alexandros | Rodolakis |
| Luis | Rodolfo Bonilla |
| Josep | Rodoreda |
| Laura | Rodrigáñez |
| D | Rodrigo |
| VS D | Rodrigo |
| Walter | Rodrigo Martínez Torres |
| José | Rodrigo Oliva |
| Catarina S | Rodrigues |
| Josy | Rodrigues |
| M | Rodrigues |
| Rodrigo | Rodrigues |
| SC | Rodrigues |
| Sónia | Rodrigues |
| ML | Rodrigues Barbosa da Silva |
| Juliano | Rodrigues da Cunha |
| C | Rodriguez |
| Camilo | Rodriguez |
| E | Rodriguez |
| Eduardo | Rodriguez |
| J | Rodriguez |
| JL | Rodriguez |
| Juan | Rodriguez |
| Juliana | Rodriguez |
| Kenny | Rodriguez |
| Natalia | Rodriguez |
| Ada | Rodríguez |
| Carballo | Rodríguez |
| Araceli | Rodrìguez |
| RDLC | Rodríguez Ciria |
| Jaime | Rodríguez de Alarcón |
| MI | Rodriguez Fernandez |
| A | Rodriguez Fraga |
| L | Rodríguez Gómez |
| Lorena | Rodríguez Gómez |
| Agustin | Rodriguez Gonzalez |
| A | Rodríguez Gonzalez |
| Carmen | Rodríguez Haro |
| A | Rodriguez Infante |
| Antonio | Rodriguez Infante |
| Cedillo | Rodrìguez Jonathan Rubén |
| JN | Rodriguez Niño |
| Lizeth | Rodriguez Sanchez |
| Ana | Rodríguez Sánchez |
| Leticia | Rodriguez Vaquero |
| J | Rodriguez-Abreu |
| Julia | Rodriguez-Abreu |
| Mario | Rodriguez-Lopez |
| Ana | Rodríguez-Tesouro |
| Raquel | Rodríguez-Uria |
| H | Rodriguez-Zentner |
| Homero | Rodriguez-Zentner |
| J | Rodriquez |
| Jennifer | Rodriquez |
| Anne-Jasmin | Roelofs |
| Judith | Roesch |
| Nicolo | Roffi |
| A | Rogers |
| L | Rogers |
| LJ | Rogers |
| Luke | Rogers |
| M | Rogers |
| S | Rogers |
| Pat | Rohan |
| Himanshu | Rohela |
| S | Rohleder |
| Andrej | Roj |
| A | Rojas Aguilar |
| Antonio | Rojas Aguilar |
| Fabian | Rojas Portilla |
| Yesenia | Rojas-Khalil |
| DA | Rojas-Tejada |
| J | Rojas-Ticona |
| Javier | Rojas-Ticona |
| Gheorghe | Rojnoveanu |
| Alicia | Rojo |
| JA | Rojo Lopez |
| AC | Rokohl |
| Christina L | Roland |
| CL | Roland |
| Pedro | Roldan Ramos |
| Marta | Roldón Golet |
| Dirk | Rolf Bulian |
| J | Rolinger |
| Jens | Rolinger |
| P | Rolland |
| Udo | Rolle |
| RA | Rollett |
| Rebecca | Rollett |
| L | Rolli |
| Luigi | Rolli |
| G | Rollo |
| A | Rolls |
| Catarina | Rolo Santos |
| Guilherme | Roloff Cardoso |
| Aristide | Romain Raherison |
| E | Romairone |
| David | Roman |
| Laura | Román García de León |
| Angela | Romano |
| M | Romano |
| Maurizio | Romano |
| Vito | Romano |
| John | Romany |
| A | Romanzi |
| Sèmèvo | Romaric Tobome |
| Adrian | Rombach |
| Esmeralda | Romero Bañuelos |
| Alejandro | Romero de Diego |
| CS | Romero Garcia |
| Fernanda | Romero Lechuga |
| CA | Romero Manqui |
| Guillermo | Romero Reyna |
| E | Romero-Bañuelos |
| A | Romero-De Diego |
| Ivan | Romić |
| O | Rominiyi |
| Ola | Rominiyi |
| Saleh | Romman |
| F | Ron |
| Michael | Ron Freund |
| Arturo | Roncone |
| U | Ronellenfitsch |
| Ulrich | Ronellenfitsch |
| DA | Ronquillo Andrade |
| L | Rony |
| J | Rooney |
| Joanna | Rooney |
| S | Rooney |
| Ryan | Roopnarinesingh |
| Oscar | Roque |
| F | Rosa |
| Fausto | Rosa |
| Isabel | Rosa Fernández Burgos |
| Maria | Rosa Ortiz |
| Lilia | Rosa Reyes Guilamo |
| Rember | Rosales Arriola |
| M | Rosario |
| Patricio | Rosas |
| C | Rosas bermudez |
| R | Rosati |
| Riccardo | Rosati |
| F | Rosato |
| Fernando | Rosatti |
| C | Rösch |
| J | Rose |
| Matthew | Rose |
| Nantambi | Rose |
| Emma | Rose Michelle Woolcock |
| A | Roselló Añón |
| Alejandro | Roselló Añón |
| Lauren | Rosenblum |
| M | Rosengart |
| Kari | Rosenkranz |
| SM | Roser |
| Steven | Roser |
| Vittoria | Rosetti |
| dolors | Rosines Cubells |
| M | Rosines Cubells |
| Aida | Rosita Tantri |
| A | Roslani |
| AC | Roslani |
| April | Roslani |
| Carlo | Ross |
| E | Ross |
| Fiona | Ross |
| H | Ross |
| L | Ross |
| Lauren | Ross |
| S | Ross |
| Samuel | Ross |
| Rolf | Rossaint |
| Ayane | Rossano |
| C | Rossborough |
| Domenico | Rossi |
| G | Rossi |
| Giulia | Rossi |
| Gustavo | Rossi |
| L | Rossi |
| Luciano | Rossi |
| S | Rossi |
| Serena | Rossi |
| Settimio | Rossi |
| V | Rossi |
| Vanessa | Rossi |
| Kayla | Rossini |
| Matteo | Rossini |
| Tobias | Rossmann |
| Andres | Rosso |
| Klara | Rosta |
| T | Rostkowski |
| J | Roszpopa |
| A | Roth |
| Andreas | Roth |
| Nicole | Rotter |
| M | Rottoli |
| Matteo | Rottoli |
| Nicholas | Roubos |
| Amy | Round |
| Morgan | Roupret |
| O | Rousan |
| Bikram | Rout |
| S | Rout |
| T | Routledge |
| Alexia | Roux |
| Frederic | Roux |
| Marco | Rovagnati |
| F | Roviello |
| Franco | Roviello |
| S | Roward |
| Alistair | Rowcroft |
| Alberto | Roxas |
| V | Roxo |
| Vanessa | Roxo |
| Ashutosh | Roy |
| C | Roy |
| H | Roy |
| Jennifer | Roy |
| Nathalie | Roy |
| S | Roy Mahapatra |
| Sunanda | Roy Mahapatra |
| T | Royle |
| AR | Royson |
| A | Różańska-Walędziak |
| F | Rozet |
| Balazs | Rózsa |
| Sophie | Rozwadowski |
| Ana | Ruano |
| Adriana | Ruano Campos |
| Michele | Rubbini |
| Sonia | Rubbo |
| Wilson | Rubiano |
| Nina | Rubicz |
| K | Rubin |
| E | Rubio |
| Enrique | Rubio |
| Mercedes | Rubio Manzanares Dorado |
| J | Rubio-Palau |
| Josep | Rubio-Palau |
| I | Rubio-Perez |
| Ines | Rubio-Perez |
| Irina | Rudenko |
| M | Rudic |
| Milan | Rudic |
| Gareth | Rudock |
| Victoria | Rudolph-Stringer |
| Agris | Rudzāts |
| Mario | Rueda |
| M | Ruel |
| SR | Rufai |
| Enrico | Ruffini |
| G | Ruffo |
| Giacomo | Ruffo |
| Anya | Rugendyke |
| F | Ruggiero |
| Silvia | Ruggiero |
| M | Ruhosha |
| Manuel | Ruiss |
| A | Ruivo |
| G | Ruiz |
| I | Ruiz |
| Fernanda | Ruiz de Andrade |
| FR | Ruiz Echeverría |
| Alicia | Ruiz Escobar |
| M | Ruiz Esquide |
| F | Ruiz Grande |
| Catalina | Ruiz Lopez |
| I | Ruiz Martin |
| Rebeca | Ruiz Roman |
| María | Ruiz Soriano |
| A | Ruiz-Escobar |
| M | Ruiz-Marín |
| Miguel | Ruiz-Marín |
| Francisco | Ruiz-Navarro |
| M | Ruiz-Soriano |
| L | Ruiz-Villa |
| Laura | Ruiz-Villa |
| Jordi | Rumià Arboix |
| Cristian | Ruminot |
| M | Rumyantseva |
| Stuart | Rundle |
| Mohd | Rusdi Draman |
| Loren | Rushton |
| J | Russ |
| E | Russe |
| Elisabeth | Russe |
| Christine | Russell |
| Crispin | Russell |
| N | Russell |
| Neil | Russell |
| Victoria | Russell |
| Maria | Russi |
| Davide | Russo |
| Elena | Russo |
| IS | Russo |
| AO | RUSU |
| M | Rutegård |
| Martin | Rutegård |
| D | Rutherford |
| Daylen | Rutledge |
| Michael | Ruyssers |
| NM | Ruzgar |
| A | Ruzzenente |
| Andrea | Ruzzenente |
| Oscar | Rwego |
| Christopher | Ryalino |
| É | Ryan |
| Éanna | Ryan |
| ÉJ | Ryan |
| James | Ryan |
| Jessica | Ryan |
| Neil | Ryan |
| O | Ryan |
| William | Ryan |
| Justyna | Rymarowicz |
| O | Ryska |
| Suganya | S |
| Inês | Sá |
| Claudia | Saab |
| A | Saad |
| Ebtesam | Saad |
| Haisam | Saad |
| Mahmoud M | Saad |
| MM | Saad |
| Moustafa | Saad |
| Nader | Saad |
| S | Saad |
| Safaa | Saad |
| Sanad | Saad |
| Ahmed | Saad Elsaeidy |
| Khaled | Saad Elsaeidy |
| R | Saadeh |
| A | Saadi |
| Jose | Saadi |
| A | Saadya |
| Sarra | Saaf |
| T | Saafan |
| R | Saaid |
| Rahmah | Saaid |
| J | Saavedra |
| N | Sabanovic Bajramovic |
| Marta | Sabater-Martos |
| Luciana | Sabatini |
| Abdulrahman J | Sabbagh |
| Danielle | Sabella |
| A | Saber |
| R | Saberi |
| Rebecca | Saberi |
| Z | Saberi |
| Predrag | Sabljak |
| Angelin | Sablon Herinirina |
| Arfa | Saboor |
| E | Sabouri |
| Yasser | Sabr |
| Joseph | Sabra |
| R | Sabri |
| Nur | Sabrina Binti Babe Azaman |
| A | Sabry |
| Ahmed | Sabry |
| Aya | Sabry |
| Hesham | Sabry |
| MD P | Sacdalan |
| Rekha | Sachan |
| B | Sachdev |
| Bobby | Sachdev |
| A | Sachdeva |
| Sanket | sadanand Shetty |
| F | Sadat Rahimi |
| Anwar | Sadat Seidu |
| Mohammed | Saddik |
| M | Sadek |
| Saravanan | Sadhasivam |
| Haleema | Sadia |
| Uzma | Sadia |
| H | Sadian |
| P | Sadigh |
| A | Sadioğlu |
| Amr | Saeed |
| B | Saeed |
| Bashayer | Saeed |
| Kareem | Saeed |
| Komal | Saeed |
| M | Saeed |
| Marwah | Saeed |
| Rafeh | Saeed |
| Ridwan | Saeed |
| S | Saeed |
| Summaya | Saeed |
| U | Saeed |
| Waqar | Saeed |
| Y | Saeed |
| Yousif | Saeed |
| Umer | Saeed Haroon |
| Faisal | Saeed Hassan |
| Sawsan | Saeid |
| Somcharoen | Saeteng |
| Manuel | Saez barba |
| P | Saez Carlin |
| Patricia | Saez Carlin |
| Maialen | Saez de Vicuña Salinas |
| EM | Sáez-Cerezal |
| Elena | Sáez-Ruiz |
| Mustafa | Safa Uyanik |
| Bassem | Safadi |
| H | Safari |
| M | Safari |
| Seyer | Safi |
| Najib | Safieddine |
| Fanonjomahasoa | Safiry Andofenohasina |
| Sergej | Safonov |
| Mohammad | Safri |
| György | Saftics |
| Hadeer | Safwat |
| J | Sagar |
| Jayesh | Sagar |
| Sushma | Sagar |
| R | Saghir |
| A | Sagnotta |
| Andrea | Sagnotta |
| Robert | Sagoe |
| Sunita | Saha |
| Arun | Sahai |
| Egbal | Sahal Abdelmajed |
| Zeynep | Şahan Çeti̇nkaya |
| Inci | Sahin |
| Can | Şahin |
| R | Şahin |
| Abat | Sahlu |
| K | Sahnan |
| Kapil | Sahnan |
| A | Sahni |
| Anjana | Sahu |
| Arnav | Sahu |
| Banchhita | Sahu |
| Rabi | Sahu |
| AM | Saibene |
| M | Said |
| Yasmeen | Said |
| A | Said Bayazeed |
| Skender | Saidi |
| Tim | Saier |
| Sara | Saif |
| S | Saifuddin |
| Altanchimeg | Sainbayar |
| Fani | Saini |
| T | Saini |
| Thomas | Saini |
| A | Sainz Lete |
| M | Sait |
| Salma | Sait |
| Junichi | Saito |
| Ryoichi | Saito |
| Takuya | Saito |
| Tomohito | Saito |
| Miguel | Saiz Sánchez-Buitrago |
| Hannan | Sajid |
| Zaina | Sajid |
| Y | Sakaray |
| R | Saket |
| Rawand | Saket |
| Orazbek | Sakhov |
| Rajendra | Sakhrekar |
| Mehmet | Sakinci |
| A | Sakr |
| Ahmed | Sakr |
| T | Sakurai |
| Tomoe | Sakurai |
| Laura | Sala |
| Michael | Sala |
| A | Salah |
| Omar | Salah |
| Alzhraa | Salah Abbas |
| Amna | Salam Al-Wandi |
| H | Salama |
| Paul | Salama |
| A | Salamah |
| Abdulrauf | Salamah |
| Sara | Salamah |
| F | Salameh |
| Michel | Salameh |
| Mohammed | Salameh |
| Babatunde | Salami |
| Giuseppe | Salamone |
| R | Salas |
| Claudio | Salas Garrido |
| E | Salau |
| Eniola | Salau |
| Adedayo | Salawu |
| Hafeez | Salawu |
| Nasiru | Salawu |
| A | Salazar |
| Adolfo | Salazar |
| A | Salazar-Tantaleán |
| DF | Salcedo Miranda |
| RA | Salcedo-Hernández |
| Rosa | Salcedo-Hernández |
| DV | Saldivar Ozan |
| Cesareo | Saldivar Patiño |
| Danjuma | Sale |
| A | Saleem |
| Abdulaziz | Saleem |
| Bushra | Saleem |
| Humaira | Saleem |
| Irfan | Saleem |
| MA | Saleem |
| Maleeha | Saleem |
| Mohammad | Saleem |
| O | Saleem |
| Tayyaba | Saleem |
| Samina | Saleem Dojki |
| Mohamed | Saleem Noor Mohamed |
| Aasim | Saleemi |
| Ahmed | Saleh |
| C | Saleh |
| IA | Saleh |
| Ibrahiem | Saleh |
| M | Saleh |
| Mahmoud | Saleh |
| Mamoun | Saleh |
| Mohannad | Saleh |
| Abddulrahman | Saleh Almulhim |
| Alhosen | Saleh M Aldelensi Alzubi |
| Mona | Saleh Mesbah Mohamed Elkaffas |
| M | Salehi Shadkami |
| Mohammed | Salele Aliyu |
| Alaa | Salem |
| H | Salem |
| Hani | Salem |
| Marwa | Salem |
| MC | Salem |
| Moacyr | Salem |
| Nourhan | Salem |
| Osama | Salem |
| Rima | Salem |
| Amin | Salem Ahmed Egdeer |
| Roaa | Salem Jwaid Alneimat |
| G | Salerno |
| R | Sales |
| Inês | Salgado |
| Wilson | Salgado Jr |
| N | Salgado-Nesme |
| Noel | Salgado-Nesme |
| Shemsedin | Salia |
| Timur | Saliev |
| Mohammed | Salihu |
| A | Salim |
| Azra | Salim |
| Hashem | Salim |
| Radhwan | Salim |
| Ruqyyah | Salim |
| Shaharyar | Salim |
| Armando | Salim Munoz Abraham |
| Amrollah | Salimi |
| A | Salimi asl |
| S | Salimoğlu |
| Semra | Salimoğlu |
| JR | Salinas Peña |
| S | Salindera |
| D | Salinovic |
| Thomas | Salisbury |
| Kabiru | Salisu |
| Stefano | Salizzoni |
| A | Sallam |
| Ahmed | Sallam |
| Ali | Sallam |
| Asser | Sallam |
| I | Sallam |
| Ibrahim | Sallam |
| M | Sallam |
| Mahmoud | Sallam |
| Moataz | Sallam |
| H | Salle |
| Henri | Salle |
| Mat | Salleh Sarif |
| Salloum | Salloum |
| Reem | Salman |
| S | Salman |
| Samar | Salman |
| Muhammad | Salman Farsi |
| Mohammad | Salman Siddiqi |
| Enrique | Salmerón-González |
| Bethan | Salmon |
| M | Salö |
| Ana | Salomé Cavaleiro Leitão de Carvalho |
| Emily | Salt |
| JA | Salud |
| P | Salunke |
| Pravin | Salunke |
| Dhanshree | Salunkhe |
| Ariadna | Salvadó |
| Renato | Salvador |
| Abegail | Salvana |
| Maurizio | Salvati |
| Maisa | Salvetti |
| R | Salvia |
| Roberto | Salvia |
| R | Salvioni |
| Roberto | Salvioni |
| Garrett | Salzman |
| Musore | Sam |
| Samy | Samaan |
| Zeljka | Samac |
| Nilofar | Samadi |
| Mustafa | Samadony |
| E | Samadov |
| Elgun | Samadov |
| Rehab | Samaka |
| Diana | Samantha Gonzalez |
| A | Samara |
| Athina | Samara |
| Evangelia | Samara |
| Mohammed | Samara |
| Dharmabandhu N | Samarasekera |
| DN | Samarasekera |
| D | Samaraweera |
| Dulan | Samaraweera |
| E | Samarut |
| S | Sambhwani |
| Sharan | Sambhwani |
| Daniele | Sambucci |
| E | Sambugaro |
| E | Samed |
| Mohammad | Sameer |
| Mostafa | Sameh |
| Sarika | Samel |
| Ashraf | Samer |
| Mhamed | Samer Alkhatib |
| Omar | Sami |
| Ahmed | Samih |
| Ahmed | Samir |
| A | Samir Abdelaal |
| Ahmed | Samir Abdelaal |
| Ahmed | Samir Farahat |
| Fuad | Samir Lopez Fernández |
| L | Samison |
| Ali | Samkari |
| Suzette | Samlalsingh |
| Sari | Samman |
| G | Sammarco |
| Giuseppe | Sammarco |
| T | Sammour |
| Tarik | Sammour |
| KA | Samo |
| II | Sampaio da Nóvoa Gomes Miguel |
| M | Sampaio-Alves |
| Mafalda | Sampaio-Alves |
| GM | Sampietro |
| Jack | Sample |
| Inderpaul | Samra |
| Mujuni | Samson |
| Chathurika | Samudani Dhanasekara |
| Diego | Samudio |
| Abhishek | Samuel |
| Gilbert | Samuel |
| Habie | Samuel |
| Odongo | Samuel |
| Claudio | Samuel Dóleo García |
| Thomas | Samuel William Greensmith |
| M | San Andrés |
| Joana | San Anton |
| Carlos | San Miguel |
| C | San Miguel Méndez |
| D | Sanabria |
| Daniel | Sanabria |
| Aly | Sanad |
| Aaron | Sanchez |
| R | Sánchez |
| Rosa | Sánchez |
| G | Sánchez Aniceto |
| Alejandro | Sanchez Arteaga |
| Alvaro | Sanchez Barrueco |
| L | Sánchez Blasco |
| Laura | Sánchez Blasco |
| F | Sanchez Cabezudo Noguera |
| Fatima | Sanchez Cabezudo Noguera |
| S | Sanchez Cabús |
| Ernesto | Sánchez Castillo |
| Ailén | Sánchez Cruz |
| Isabel | Sanchez Cuadrado |
| C | Sánchez del Pueblo |
| Cristina | Sánchez del Pueblo |
| Fátima | Sánchez Fernández |
| A | Sánchez Gollarte |
| Ana | Sánchez Gollarte |
| J | Sanchez Gonzalez |
| Javier | Sanchez Gonzalez |
| R | Sanchez Jimenez |
| A | Sanchez Lopez |
| Anna | Sánchez López |
| A | Sánchez Mozo |
| Ana | Sánchez Mozo |
| Alejandro | Sanchez Pellejero |
| Barriga | Sánchez Raquel |
| Marina | Sánchez Robles |
| Carlos | Sanchez Rodriguez |
| R | Sanchez Salas |
| Miguel | Sánchez Suárez |
| Cristina | Sánchez Torralvo |
| Vanessa | Sanchez Torrents |
| A | Sánchez-Arteaga |
| Santiago | Sánchez-Cabús |
| AB | Sánchez-Casado |
| N | Sanchez-Fuentes |
| S | Sánchez-García |
| A | Sánchez-Gómez |
| Andrés | Sánchez-Gómez |
| TA | Sánchez-Gómez |
| L | Sánchez-Guillén |
| Luis | Sánchez-Guillén |
| JI | Sanchez-Mendez |
| D | Sanchez-Pelaez |
| Daniel | Sanchez-Pelaez |
| C | Sanchez-Perez |
| M | Sánchez-Robles |
| C | Sánchez-Rodríguez |
| M | Sanchez-Rubio |
| M | Sánchez-Rubio |
| María | Sánchez-Rubio |
| R | Sanchez-Santos |
| Guillermo | Sanchez-Villaseñor |
| P | Sanchis |
| Antonio | Sanchís López |
| J | Sancho-Muriel |
| Jorge | Sancho-Muriel |
| L | Sanchon |
| Erdene | Sandag |
| G | Sandblom |
| Johannes | Sander |
| M | Sander |
| Michael | Sander |
| J | Sanders |
| Julie | Sanders |
| L | Sanderson |
| Gagandeep | Sandhu |
| A | Sandhya |
| Anu | Sandhya |
| R | Sandkamp |
| Richard | Sandkamp |
| Laura | Sandland-Taylor |
| Gursev | Sandlas |
| Yael | Sandler |
| Saleh | Sandoughdaran |
| Camilo | Sandoval |
| Hernan | Sandoval |
| John | Sandoval |
| M | Sandoval |
| Mauricio | Sandoval Tobar |
| Marco | Sandoval vaez |
| Cristian | Sandu |
| S | Sane |
| Miguel | Sanfeliu Giner |
| Vinita | Sangai |
| C | Sangani |
| M | Sange |
| Andrea | Sangheli |
| MJ | Sangüesa |
| Burmaa | Sanjaa |
| Shaikh | Sanjid Seraj |
| Ghaidaa | Sanjuq |
| Mohamad | Sankari |
| Pushp | Sankhwar |
| Satyanarayan | Sankhwar |
| S | Sankpal |
| AN | Sanli |
| Anand | Sanmugam |
| Angelino | Sanna |
| Tsuyoshi | Sano |
| K | Sanserino |
| Carlos | Santacruz |
| G | Šantak |
| Goran | Šantak |
| Roberto | Santambrogio |
| Diana | Santana |
| Eleazar | Santana |
| R | Santana Ortiz |
| Roman | Santana Santana |
| M | Santarelli |
| M | Santas |
| MS | Santero-Ramirez |
| Oscar | Santes Jasso |
| G | Santhirakumaran |
| Gowthanan | Santhirakumaran |
| Alvaro | Santiago LeMarie Guerra |
| Silvia | Santiago Maniega |
| Manuel | Santiago Mosquera Paz |
| JA | Santibanez-Salgado |
| P | Santillan-doherty |
| Patricio | Santillan-Doherty |
| Mario | Santinami |
| A | Santini |
| AJ A | Santini |
| Alasdair | Santini |
| Matteo | Santoliquido |
| OS | Santonocito |
| A | Santoro |
| Antonio | Santoro |
| Giulio | Santoro |
| BC | Santos |
| Blanca | Santos |
| E | Santos |
| Ema | Santos |
| Irène | Santos |
| Jorge | Santos |
| Jos´é | Santos |
| L | Santos |
| Marco | Santos |
| P | Santos |
| Patrícia | Santos |
| PM D D | Santos |
| R | Santos |
| Rui | Santos |
| SS | Santos |
| Victor | Santos |
| Tainá | Santos Bezerra |
| Pilar | Santos Cidon |
| Marta | Santos Espí |
| Paulo | Santos-Costa |
| H | Santos-Sousa |
| Hugo | Santos-Sousa |
| JA | Santoshi |
| Nada | Santrac |
| V | Santric |
| Masamitsu | Sanui |
| Mohammad | Sanwar |
| Sudip | Sanyal |
| C | Sanz |
| Cristina | Sanz |
| Edgar | Sanz |
| Mercedes | Sanz |
| Sandra | Sanz |
| A | Sanz Larrainzar |
| Amaia | Sanz Larrainzar |
| Andrea | Sanz Llorente |
| R | Sanz Lopez |
| G | Sanz Ortega |
| J | Sanz Romera |
| Jorge | Sanz Romera |
| Rosa | Sanz-Gonzalez |
| Ricardo | São Pedro |
| Codin | Saon |
| Rita | Sapage |
| P | Sapienza |
| Paolo | Sapienza |
| Gianmarco | Saponaro |
| D | Sapre |
| Dimple | Sapre |
| Teddy | Saputra |
| Zain | Saqfalhait |
| Madiha | Saqib |
| MW | Saqib |
| Hafiz | Saqib Sikandar |
| F | Saraceno |
| Giorgio | Saraceno |
| A | Saracoglu |
| Ayten | Saracoglu |
| K | Saracoglu |
| Kemal | Saracoglu |
| KT | Saracoglu |
| A | Sarafi |
| P | Saraiva |
| Pedro | Saraiva |
| Wislene | Sarajane Moreira Alves |
| Mumtaz | Sarang |
| I | Sarantitis |
| Ioannis | Sarantitis |
| Sarbpreet | Sarao |
| A | Saratziotis |
| Athanasios | Saratziotis |
| Athanasios | Saratzis |
| R | Saravanan |
| Husam | Sarayrah |
| Imraan | Sardiwalla |
| Kiran | Sarfraz |
| Muhammad | Sarfraz Khan |
| Matthew | Sargent |
| AbdulRahman | Sari |
| D | Sari |
| Djayanti | Sari |
| Can | Sarica |
| Monira | Sarih |
| Ankit | Sarin |
| Divya | Sarin |
| R | Sarı |
| Ramazan | Sarı |
| S | Sarıdemir |
| E | Sarjanoja |
| Elise | Sarjanoja |
| Abhishek | Sarkar |
| Hrishikesh | Sarkar |
| Saurav | Sarkar |
| Riad | Sarkis |
| Mathilde | Sarlabous |
| DR | Sarma |
| Muhammad | Sarmad Tamimy |
| Aisulu | Sarmenova |
| A | Sarmiento |
| Abigail | Sarmiento |
| JA | Sarmiento-Bobadilla |
| S | Sarnacki |
| AL | Sarni |
| V | Sarodaya |
| Varun | Sarodaya |
| G | Sarpietro |
| Giuseppe | Sarpietro |
| Khaled | Sarraf |
| KM | Sarraf |
| Claudia | Sarrais Polo |
| C | Sarre |
| Catherine | Sarre |
| Sera | Sarsam |
| S | Sarsik |
| Sameh | Sarsik |
| L | Sartarelli |
| Enrico | Sartori |
| J | Sarveswaran |
| Janahan | Sarveswaran |
| A | Sarwar |
| MZ | Sarwar |
| Safdar | Sarwar |
| Jahangir | Sarwar Khan Khan |
| Takeshi | Sasaki |
| L | Sasatti |
| Lokesh | Sasatti |
| D | Sasia |
| Diego | Sasia |
| S | Saso |
| Srdjan | Saso |
| Amit | Sastry |
[truncated: 82,917 more chars]
